# The Competition of Homophily and Popularity in Growing and Evolving Social Networks

**DOI:** 10.1038/s41598-018-33409-8

**Published:** 2018-10-18

**Authors:** Yezheng Liu, Lingfei Li, Hai Wang, Chunhua Sun, Xiayu Chen, Jianmin He, Yuanchun Jiang

**Affiliations:** 1 School of Management, Hefei University of Technology, Hefei, 230009 China; 2 Key Laboratory of Process Optimization and Intelligent Decision-making, Ministry of Education, Hefei, Anhui 230009 China; 3 Sobey School of Business, Saint Mary’s University, Halifax, Canada

## Abstract

Previous studies have used several models to investigate the mechanisms for growing and evolving real social networks. These models have been widely used to simulate large networks in many applications. In this paper, based on the evolutionary mechanisms of homophily and popularity, we propose a new generation model for growing and evolving social networks, namely, the Homophily-Popularity model. In this new model, new links are added, and old links are deleted based on the link probabilities between every node pair. The results of our simulation-based experimental studies provide evidence that the proposed model is capable of modelling a variety of real social networks.

## Introduction

One of the fundamental problems in the research of social networks is the evolutionary mechanisms of large social networks^1–4^. One of the best-known evolutionary mechanisms of social networks is *preferential attachment*^2^, which suggests that a new node will have a higher probability of linking to existing nodes that already have a large number of connections in the network^2^—that is, those nodes that are more visible than others^5^. We refer to this popularity-based attachment as the ‘*popularity*’ mechanism in this paper. Drawing upon this mechanism and network growth, the Barabasi-Albert (BA) model was proposed for generating and simulating social networks^2^. The BA model can reproduce the power-law degree distribution observed in many real social networks; however, it does not address several important characteristics of social networks, including clustering and community structures^3,6–9^. Therefore, a number of variations of the BA model, such as the multistage random growing network model and the local-world evolving network model, have been proposed to generate networks that more accurately resemble real social networks^10–12^.

Meanwhile, prior studies have suggested that an individual in a social network tends to connect not only to popular individuals such as superstars but also to less popular individuals who share that individual’s special interests^13,14^. Hence, *homophily*, which suggests that individuals in a social network tend to form relationships with others who share similar attributes^14–16^, should be considered as another important evolutionary mechanism. Homophily can be subdivided into *observed homophily* and *latent homophily*. Observed homophily explains the similarity in individuals’ preferences that are due to observable attributes such as age, location, and religious affiliation^15^. Latent homophily explains the similarity in individuals’ preferences that are due to unobservable attributes such as individuals’ interests^15^. Focusing on the homophily mechanism, Boguna *et al*.^17^ and Wong *et al*.^18^ parameterized the tendency to establish acquaintances by the spatial distances in a representative social space and then developed spatial random graph models in which the homophily between each pair of nodes is determined by the spatial distance between those nodes. The spatial random graph model can capture many generic properties of social networks, including the “small-world” properties, power-law degree distribution, and high level of clustering. However, these models cannot be used to study the evolutionary process of social networks because the network size is a model parameter and must be a fixed number.

Prior studies proposed a number of generation models for growing and scaling social networks by focusing on both the popularity and homophily mechanisms^1,4,19,20^. For example, Li *et al*.^19^ proposed the homophyly/kinship model, in which each node was associated with a distinct colour and that same colour was used to represent the homophily or kinship between nodes. In the homophyly/kinship model, when a new node is added into the network, it is assigned a new colour according to a given probability and then linked to existing nodes based on their degrees. Otherwise, it is assigned an existing colour and linked to existing nodes of the same colour based on their degrees. Papadopoulos *et al*.^4^ proposed the popularity*similarity (PS) model, which assumes that all nodes exist on a plane. PS model treats the time of a node’s creation as a proxy for the node’s popularity, and maps the birth time of the node to its radial coordinate. The angular distance between two nodes is treated as the similarity between them. New nodes are then connected to the closest *m* nodes based on the hyperbolic distances of their polar coordinates. The PS model is capable of providing results with strong clustering and a power law degree distribution. Zuev *et al*.^1^ proposed the geometric preferential attachment (GPA) model based on the PS model. In the GPA model, the probability that a new node is placed in a particular section is proportional to the density in that section. Ferretti *et al*.^20^ demonstrated that there was actually a duality among a class of growing spatial networks based on preferential attachment on the sphere and a class of static random networks on the hyperbolic plane. In fact, the BA model is equivalent to a static random network on a hyperbolic space with infinite curvature^20^.

The above models can capture many properties of real social networks. In this paper, we attempt to study generation models from the following perspectives:

1. How the homophily mechanism affects a node’s connectivity. We propose to use a multi-dimensional vector of nodes’ attribute preferences to compute the similarity between two nodes.
2. How networks are generated based on both the homophily and popularity mechanisms. We propose to use a binomial distribution to model the interplay of these two mechanisms during the network growth process.
3. How links change during the network growth process. In previously proposed models, the links between existing nodes are static and remain unchanged after they have been inserted into the network. We propose that links between existing nodes can be deleted at a certain probability during network evolution.
4. How the homophily and popularity mechanisms affect the structural properties of social networks.

In this paper, we combine the homophily and popularity mechanisms and propose the Homophily-Popularity (HP) model for growing and evolving social networks. In the proposed HP model, we provide a new framework to determine the number of new links, calculate the connectivity probability, and insert and delete links. Synthetic networks generated by the HP model can reflect many properties of real social networks. Using the HP model, when the dynamics of the homophily and popularity mechanisms change during the network generation process, the final generated networks would show diversity in many properties, such as degree distribution, degree correlation and community size. As the homophily mechanism becomes more dominant in the network generation process, the generated network gradually transforms from disassortative to assortative.

## Results

### The Homophily-Popularity model

In practice, popularity and homophily are known to be two dominant mechanisms of network evolution^21,22^. For example, the homophily effect is considerably more significant than the popularity effect on Facebook and Flickr but far less significant on YouTube, ScienceNet, and Epinions^23^. In this paper, we propose a novel social network generation model named the Homophily-Popularity (HP) model. The HP model attempts to fit different types of real networks through a variety of characteristics. The primary motivations for this study are as follows:

- Many previously proposed models assume that the number of new links in each step always remains constant. However, Leskovec *et al*.^24^ examined a wide range of real social networks and discovered that many of these networks densify over time, with the number of links growing superlinearly with regard to the number of nodes. In particular, the number of nodes *versus* the number of links fits a line on the logarithmic scale. This pattern can be formalized as ${M}_{t}={{N}_{t}}^{k}$ where *M*_*t*_ is the number of links at time *t*, *N*_*t*_ is the number of nodes at time *t* and the slope *k* ranges from 1.1 to 1.7^24^.
- There are two main reasons why an individual links to another in a social network. First, an individual is more likely to connect to others who share similar interests. Second, an individual is likely to connect to other individuals who are already well connected in the network. Wang *et al*.^22^ empirically studied the evolution of collaboration networks and determined that specialty homophily contributes 38% to the formation of co-author links, followed by preferential attachment (36%) and institution homophily (27%). This implies that link probability is determined by both homophily and popularity.
- In real social networks, nodes make their own decisions when balancing homophily and popularity. For example, some individuals may prefer to follow their own preferences to connect to friends, while others may prefer to connect to influential persons to obtain the information. Hence, the decisions to balance homophily and popularity are personalized and will vary from person to person^25^.
- Because attributes are often not of equal importance, some attributes may be popular, and other attributes may be niche. In general, individuals who are similar in niche attributes are more likely to connect with each other through homophily. For example, individuals who enjoy playing chess often have a greater probability of being friends than individuals who enjoy watching TV, because many people like watching TV and they cannot easily be distinguished.
- In the real world, online social networks are dynamic. New links may be formed, and old links may be broken. Specifically, the largest contribution to network evolution is the appearance of new links between old nodes^26,27^.

According to these motivations, the network generation algorithm of the PH model is summarized as follows:

#### Initialization

Similar to many generation models^2,10^, our PH model begins with a network of *m*_0_ nodes at the initial time *t*_0_. For simplicity, we set *t*_0_ = *m*_0_. These *m*_0_ nodes are fully connected. Each node *n*_*i*_ is associated with a vector of attribute preferences *I*_*i*_ = (*a*_*i*1_, *a*_*i*2_, …, *a*_*iX*_), where *X* is the total number of attributes and *a*_*ij*_ is a real number between 0 and 1 that represents the preference of node *n*_*i*_ for attribute *j*. The sum of all the elements in this vector is 1. This vector of attribute preferences *I*_*i*_ is used to determine the connectivity between nodes. The construction method for *I*_*i*_ will be described in the next subsection. At each time step, *t* > *t*_0_, a new node *n*_*t*_ with a new vector *I*_*t*_ is inserted into the network.

#### Determining the number of new links

In contrast to previous proposed models, our PH model assumes that the number of new links Δ*m*_*t*_ at time *t* is $\Delta {m}_{t}={{N}_{t}}^{k}-{({N}_{t}-1)}^{k}$. We round the real numbers to the nearest integers.

#### Calculating the connectivity probability

Our PH model assumes the probability that node *n*_*i*_ will link to node *n*_*j*_ at time *t* (denoted as ${p}_{ij}^{t}$) is a linear function of homophily and popularity:

1 $${p}_{ij}^{t}=(1-{\beta }_{i}){F}_{j}^{t}+{\beta }_{i}sim({n}_{i},{n}_{j}),$$

where ${F}_{j}^{t}$ is the popularity of node *n*_*j*_ at time *t*, and *sim*(*n*_*i*_, *n*_*j*_) is the homophily between nodes *n*_*i*_ and *n*_*j*_ at time *t*. We use *β*_*i*_(0 ≤ *β*_*i*_ ≤ 1) to model the preference of homophily for the node *n*_*i*_. Each *β*_*i*_, for all *i* = 1, 2, …, *N*, is a sample of *β*, where *β* is the preference distribution for homophily and *N* is the number of nodes in the network.

#### Determining ${F}_{j}^{t}$ and *sim*(*n*_*i*_,*n*_*j*_)

In many models, the popularity of node *n*_*i*_ is determined by its degree^2,10–12^. To make the popularity value ranges from 0 to 1, we define the popularity of node *n*_*i*_ at time *t* as

2 $${F}_{i}^{t}=\frac{{d}_{i}^{t}+1}{\mathop{\max }\limits_{j}({{d}_{j}}^{t}+1)},$$

where ${d}_{i}^{t}$ is the degree of node *n*_*i*_ at time *t*. Note that ${F}_{i}^{t}$ is always proportional to ${d}_{i}^{t}+1$, and the popularity of an isolated node (*d*_*i*_ = 0) is always positive.

The homophily can be modelled by the similarity between nodes^4,19^. Our PH model uses the aforementioned attribute preference vectors (*I*_*i*_, *i* = 1, …, *N*) of nodes to measure node similarity. The construction of vector *I*_*i*_, described in the next subsection, reflects the fact that popular attributes generally have small indices and niche attributes generally have large indices in the vector. Our PH model models the importance of attributes using a weight vector *W*, where the weight for the *i*^th^ attribute is proportional to *i*^2^ as follows:

3 $$W=({w}_{1},\cdots ,{w}_{X}),\,{w}_{i}=\frac{{i}^{2}}{{\sum }_{i=1}^{X}{i}^{2}}.$$

The attribute preference vectors for nodes *n*_*i*_ and *n*_*j*_ are denoted as *I*_*i*_ = (*a*_*i*1_, *a*_*i*2_,…, *a*_*iX*_) and *I*_*j*_ = (*a*_*j*1_, *a*_*j*2_,…, *a*_*jX*_), respectively. The similarity between the nodes *n*_*i*_ and *n*_*j*_ with respect to homophily is defined as

4 $${S}_{ij}=W({a}_{i1}{a}_{j1},\cdots ,{a}_{iX}{a}_{jX})^{\prime} =({w}_{1},\cdots ,{w}_{X})({a}_{i1}{a}_{j1},\cdots ,{a}_{iX}{a}_{jX})^{\prime} ={\sum }_{k=1}^{X}{w}_{k}{a}_{ik}{a}_{jk}.$$

The purpose of Equations (3) and (4) is to assign larger weights to niche attributes and smaller weights to popular attributes so that the similarity between two nodes reflects niche attributes more than popular attributes.

The homophily between the nodes *n*_*i*_ and *n*_*j*_ is defined as

5 $$sim({n}_{i},{n}_{j})=\frac{{S}_{ij}}{\mathop{\max }\limits_{k=1,\cdots ,t}{S}_{ik}}.$$

Because the homophily *sim*(*n*_*i*_, *n*_*j*_) is on the same scale as the popularity ${F}_{i}^{t}$, Equation (1) computes the connectivity probability between the two nodes.

#### Determine the parameter *β*

**Figure 1:**
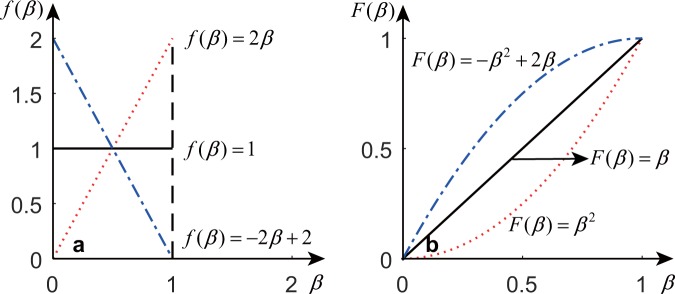
The probability density functions (**a**) and the cumulative distribution functions (**b**) for *β*.

In Equation (1), every node *n*_*i*_ can have a different preference value *β*_*i*_ that describes the preference of homophily for that node. Each *β*_*i*_ can be viewed as a random sample from a distribution of *β*. To illustrate how different distributions of *β* affect the final generated networks, we study three different types of probability density functions: uniform, monotonically decreasing within [0, 1] and monotonically increasing within [0, 1]. The probability density functions and the cumulative distribution functions are shown in Fig. 1.

When the probability density function is monotonically increasing, the mean of *β* is larger than 0.5, and homophily is stronger than popularity. We use ‘high’ to denote the networks generated in this case. When the probability density function is uniform, the mean of *β* equals 0.5; thus, homophily and popularity are approximately the same. We use ‘uniform’ to denote the networks generated in this case. When the probability density function is monotonically decreasing, the mean of *β* is less than 0.5; thus, homophily is weaker than popularity. We use ‘low’ to denote the networks generated in this case.

After all *β*_*i*_ values are acquired from the distribution *β*, the link probability ${p}_{ij}^{t}$ between any two nodes *n*_*i*_, *n*_*j*_ can be computed using Equations (1) through (5), resulting in a *n*_*t*_-dimension square matrix, *P*^*t*^, in which ${p}_{ij}^{t}$ is an element.

#### Inserting and deleting links

The link insertion and deletion process of the PH model is as follows:

At time *t*, we first compute the median of all elements in the link probability matrix *P*^*t*^ and use this value as the threshold ${T}_{{\rm{1}}}^{t}$. If the link probability of an existing link ${p}_{ij}^{t}$ is smaller than ${T}_{{\rm{1}}}^{t}$, we delete that link from the network. Let *m*_*t*_′ denote the number of links deleted at time *t*. The total number of new links to be inserted at time *t* should be Δ*m*_*t*_ + *m*_*t*_′ where $\Delta {m}_{t}={{N}_{t}}^{k}-{({N}_{t}-1)}^{k}$ and *N*_*t*_ is the number of nodes at time *t*. Second, to ensure that the new node *n*_*t*_ will not be an isolated node, we connect the new node to an existing node *n*_*i*_ with the probability ${\rm{\Pi }}({n}_{t},{n}_{i})=\frac{{p}_{ti}^{t}}{{\sum }_{j=1}^{t-1}{p}_{tj}^{t}}$. Third, we select the top ${T}_{2}^{t}$ links with the highest connection probabilities from the unconnected edges according to the matrix *P*^*t*^. Suppose the connect probabilities of the corresponding links ${l}_{(1)},{l}_{(2)},\cdots {l}_{({T}_{2}^{t})}$ are ${p}_{(1)},{p}_{(2)},\cdots {p}_{({T}_{2}^{t})}$. Then, link *l*_(*i*)_ will be chosen with a probability of ${\rm{\Pi }}^{\prime} ({l}_{(i)})=\frac{{p}_{(i)}}{{\sum }_{j=1}^{{T}_{2}^{t}}{p}_{(j)}}$. Here, ${T}_{{\rm{2}}}^{t}=\,\max \,\{200,l\cdot {t}^{2}\}$. Without loss of generality, we set *l* = 0.02. When the corresponding two endpoints of link *l*_(*i*)_ are not connected, insert the undirected link *l*_(*i*)_ into the network. This process repeats until the total Δ*m*_*t*_ + *m*_*t*_′ new links have been inserted into the network.

### Constructing and estimating the vector of attribute preferences

One key component of the HP model is the vector of attribute preferences *I*_*i*_ for node *i* in the network. Individuals/nodes may have information recorded for a different subset of attributes. In this subsection, we illustrate through a real-world example how to construct and estimate all the vectors *I*_*i*_ for nodes *i, i* = 1, *…*, *N*. The idea is to treat a vector of attribute preferences *I*_*i*_ as a random sample from the underlying distribution of attributes that can be estimated based on the social network data.

**Figure 2:**
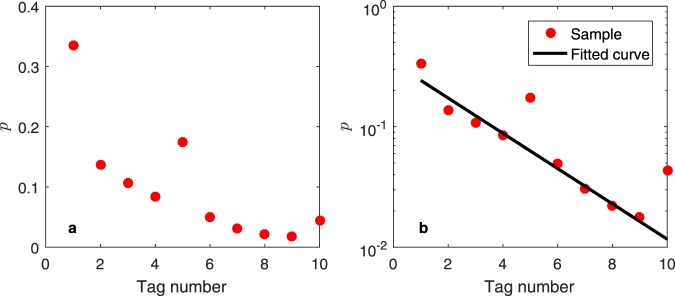
Tag number distribution for Weibo users: (**a**) normal scale, (**b**) logarithmic scale on the *y*-axis.

In some real online social networks such as Weibo, nodes’ attributes are reflected in users’ personal information: Weibo users can tag themselves; thus, users with the same tags can find each other faster. However, the tag settings are not mandatory. After a user has added tags, we regard those tags as the user’s real attributes. The total number of tags set by a user corresponds to the number of attributes associated with this user, and the set of attributes associated with a user is a subset of the set of all attributes of the social network. In this paper, we randomly select 150,000 Weibo users’ personal information and obtain 82,578 pieces of user tags information. The tag number distribution for Weibo users is shown in Fig. 2 in both normal and logarithmic scales on the *y*-axis.

The probability that a user has *i* tags approximately decreases as *i* increases, but the probabilities for *i* = 5 and *i* = 10 are abnormally high. This discrepancy may be caused by the tag-setting rules of Weibo: a new user registering a Weibo account can select at most five tags, and the system recommends other users for this new user account according to their tags; thus, the number of users with five tags is artificially huge. Moreover, users can add or delete tags on Weibo, but the upper limit is ten tags. Ignoring the exception points for *i* = 5 and *i* = 10, the scatter plot is approximately linear on the logarithmic scale. As shown in Fig. 2(b), the black solid line is the fitting function and the goodness of fit is *R*^2^ = 0.9712. Taking the logarithm of the exponential distribution function *p*(y) = *λe*^−*λy*^, we obtain

6 $$\mathrm{ln}(p(y))=\,\mathrm{ln}(\lambda )-\lambda y.$$

Moreover, researchers have indicated that human cognitive ability allows social network users to have stable interpersonal relationships with up to 150 friends^28^. A larger number of user tags indicates a stronger human cognitive ability for that user. Hence, the probability that a user has *i* tags is a decreasing function with respect to *i*. Because the decreasing trend is stronger than the linear relationship and the number of tags has a more stable mean and variance than the power distribution, we can conclude that the number of tags for each user will follow the exponential distribution.

**Figure 3:**
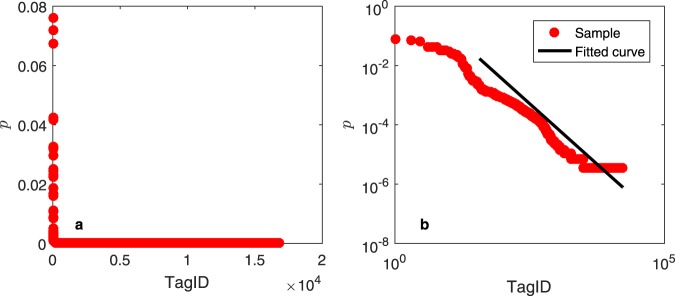
Tag distribution of Weibo users: (**a**) normal scale, (**b**) double logarithmic scale.

Furthermore, the total number of tags on Weibo is huge. Different users choose different tag subsets. Some tags are relatively popular, such as listening to music or watching movies, while others are relatively niche, such as studying social network analysis. The extreme inhomogeneity between tags causes the tag distribution to exhibit a significantly long tail. Figure 3(a) shows the tag distribution of Weibo users. Most tags are set by users with a low probability, but several tags are set by users with a high probability. The power law distribution has been widely used for fitting long-tail distributions in the social and economic fields^29^. However, distributions such as exponential or log-normal distributions may have similar effects^30^.

To identify the proper distribution for the tag distribution of Weibo users, we use the approach proposed in^30^ to fit the power law, exponential and log-normal distributions. Unfortunately, the three corresponding *p*-values are all 0, indicating that the tag distribution of Weibo users does not follow any of those distributions. To show how the model can be set up without using overly complicated probability models, we choose an alternative method. A power-law distribution is approximately linear in the double logarithmic scale; an exponential distribution is approximately linear in the logarithmic scale; and a log-normal distribution is a quadratic function in the double logarithmic scale. We then use the least squares method to fit the tag distribution of Weibo users. The goodness of fit are 0.9495 (power-law), 0.1473 (exponential), and 0.673 (log-normal) respectively. The power-law distribution fits the data best. Hence, we assume that the tag distribution of Weibo users approximately follows a power law distribution.

Figure 3(b) shows the scatter plot in the double logarithmic scale. We use the maximum likelihood method (MLE)^30^ to estimate the power exponent and the lower bound of the power-law behaviour. The black solid line in Fig. 3(b) is the resulting fitting curve.

Consequently, the vector of attribute preferences *I*_*i*_ for node *i* can be constructed as follows:

Step 1. Suppose that there are total *X* attributes in the network and that the number of attributes associated with each user is no more than *Y*, where *Y* ≤ *X*. For each node *n*_*i*_, we randomly select a sample from the exponential distribution *f*(*y*) = *λe*^−*λy*^ using the parameter *λ* and then round it up to the nearest integer *y*. If *y* ≤ *Y*, we let the number of attributes for *n*_*i*_ be *y*; otherwise, we resample *y* until *y* ≤ *Y*.

Step 2. Randomly select *y* samples *x*_1_, …, *x*_*y*_ from the power law distribution *g*(*x*) = *Cx*^−*γ*^ using the parameter *γ*, *x*_*i*_ ∈ [1, *X*], *i* = 1, 2, …, *y*. Here, we round the real numbers down to the nearest integers.

Step 3. For each *x*_*i*_, we randomly select a real value *z*_*i*_ from the interval [0,1] as the user’s preference for attribute *x*_*i*_ and assign *z*_*i*_ as the *x*_*i*_^th^ element of vector *I*_*i*_.

Step 4. We normalize all the elements in vector *I*_*i*_ = (*a*_*i*1_, *a*_*i*2_, …, *a*_*iX*_) by setting ${a}_{ij}={z}_{ij}/{\sum }_{k=1}^{X}{z}_{ik}$ so that ${\sum }_{j=1}^{X}{a}_{ij}=1$.

In the above algorithm, we assume that the attribute distribution approximately follows a power law distribution and that *x*_1_, …, *x*_*y*_ are sampled from this power law distribution. Here, *x*_1_, …, *x*_*y*_ are the indices of the attributes in vector *I*_*i*_. Hence, popular attributes generally have small indices, and niche attributes generally have large indices in vector *I*_*i*_.

### Experiments

These experiments use three probability density functions to examine how different preferences regarding the popularity and homophily mechanisms change the final generated networks. We compare our PH model with the previously proposed BA and PS models. All the experiments are executed with the total nodes *N* = 2,000. Other parameters in the experiment are as follows.

1. The total number of attributes in the networks *X* = 10.
2. The power exponent *γ* = 2. Because *X* = 10, setting *γ* = 2 causes the probabilities that the 11^th^ and higher attributes will be selected to be extremely small.
3. The upper bound of the number of attributes used by each user *Y* = 10.
4. The parameter for the exponential distribution *λ* = 1/3. In this case, the mean of the exponential distribution is 1/*λ* = 3.
5. The number of initial nodes *m*_0_ = 3.
6. The relationship between the number of nodes and the number of links is ${M}_{t}={{N}_{t}}^{k}$. To determine the power exponent *k*, we calculate the power exponent of 9 real social networks in the Stanford Large Network Dataset Collection. The results are shown in Table 1. Based on these results, we set the power exponent *k* to 1.2; thus, ${\rm{\Delta }}{m}_{t}={{N}_{t}}^{1.2}-{({N}_{t}-1)}^{1.2}$.

**Table 1:** The power exponent of 9 real social networks.

	Facebook	Gplus	Twitter	Epinions	LiveJournal	Pokec	Slashdot1	Slashdot2	Wiki-Vote
nodes	4039	107614	81306	75879	4847571	1632803	77360	82168	7115
edges	88234	13673453	1768149	508837	68993773	30622564	905468	948464	103689
*k*	1.37	1.42	1.27	1.17	1.17	1.20	1.22	1.22	1.30
*C*	0.6055	0.4901	0.5653	0.1378	0.2742	0.1094	0.0555	0.0603	0.1409

In the BA and PS models, the number of initial nodes *m*_0_ = 3, and the number of new links in each step *m* = 3. For the PS model, the parameter *β*′ controls the relative contributions of popularity and similarity, and the power-law exponent is *η* = 1 + 1/*β*′. The contribution of homophily increases as *η* increases. As previous research^4^ studied the cases for *η* = 2.1, 2.5, 3.0, we also adopt these values in our experiments.

To investigate whether the proposed model is capable of accurately modelling real social networks with different characteristics, in our experiments, we choose three representative social network datasets with distinct characteristics (i.e., YouTube, Twitter and DBLP) from the Stanford Large Network Dataset Collection. Previous empirical studies have indicated that the popularity effect is stronger than the homophily effect on YouTube^23^ but that the homophily effect is stronger than the popularity effect on DBLP^22^. Table 2 shows some basic information for these three types of networks. Because Twitter is a directed network, Twitter’s properties are calculated under a directed graph model. For example, the in-degree of a node in a directed network is used to compute how many nodes are connected to the node in the network. Therefore, in this paper, when the properties of Twitter are related to node degree, we always use the in-degree instead of the degree.

**Table 2:** The basic information of real social networks and the corresponding synthetic networks.

	nodes	Edges	Power exponent	*S*-Metric	PS		HP	
					*m*	*η*	*k*	case
YouTube	1134890	2987624	2.14	0.0159	2.63 ≈ 3	2.14	1.069	low
Twitter	81306	1768149	2.46	0.3889	21.74 ≈ 22	2.46	1.2724	uniform
DBLP	317080	1049866	3.26	0.6689	3.31 ≈ 3	3.26	1.0945	high
Facebook	4309	88234	2.25	0.4882	20.48 ≈ 20	2.25	1.3608	uniform

#### Network properties

The main properties of social networks can be summarized as follows:

- Clustering. Clustering is a typical property of social networks, where two individuals with a common friend are more likely to know each other. The clustering coefficient *c*_*i*_ of node *i* is defined as the fraction of the possible edges that could exist between the neighbours of node *i* that actually exist^3^. The average clustering coefficient of a network is the average of *c*_*i*_ over all the nodes in the network. Most complex networks show a high value for the average clustering coefficient *C*.
- Average path length. The average path length *L* of a social network is small. Because the average path length *L* is susceptible to outliers (i.e., long chains) for many social networks, we follow the Stanford Large Network Dataset Collection and use the 90-percentile effective diameter *D* to measure this property. Given a network, the 90-percentile effective diameter is the minimum number of hops required for 90% of all connected pairs of nodes to reach each other^31,32^.
- Community structure. The communities are dense subgraphs that tend to be well separated from each other. We follow the literature^33^ and use modularity *M* to measure whether the network has a community structure. A modularity no less than 0.3 provides clear evidence of the existence of community structures in the network. Furthermore, the community size distribution can be different in different networks.
- Degree distribution. Many social networks approximatively exhibit a power-law degree distribution where the power-law exponent often ranges from 2 to 3.
- Degree correlation. The degree correlation, that is, the probability that a node of degree *k* is connected to another node of degree *k*’ depends on *k*, always exists in real social networks^34^. Most social networks show “assortative mixing” on their degrees; that is, a high-degree node tends to be connected to other high-degree nodes. In contrast, networks such as the Internet show “disassortative mixing”, in which nodes with a low degree are more likely to be connected with nodes with a high degree^35^.

Then, we study these properties separately.

##### C, D, and M

**Figure 4:**
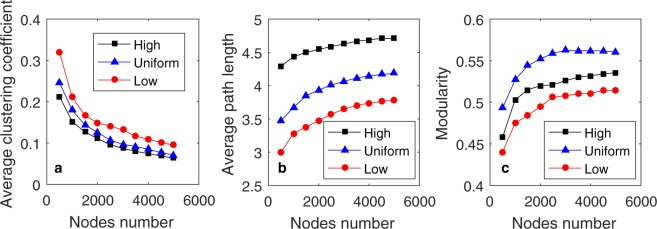
The dynamic evolutions of average clustering, average path length, and modularity.

Table 3 shows the *C*, *D*, and *M* values for different networks. As shown in Table 3, the networks generated by our PH model capture many generic properties of social networks, including a higher average clustering coefficient than networks generated by the BA model, a small average path length, and clear community structure. Moreover, for the networks generated by the PH model with the “high”, “uniform” and “low” distributions, as the popularity effect increases, the average clustering coefficient increases as well, but the average path length decreases. The dynamic evolutions of these properties from *N* = 500 to *N* = 5,000 are illustrated in Fig. 4. The average clustering coefficients of networks generated by the HP model using the three different distributions all decrease sub-linearly, and the average path length and modularity of these networks both increase as the networks grow and evolve, while their increasing and decreasing tendencies gradually diminish.

**Table 3:** The properties of each network.

	*C*	*D*	*M*	*SK*	*S-Metric*	*R*	*SL*
HP, low	0.149	4.3	0.495	4.3129	0.1832	0.8065	−0.268
HP, uniform	0.126	4.9	0.552	2.8017	0.4774	0.7857	0.0966
HP, high	0.111	5.9	0.520	0.9015	0.7080	0.86	0.3794
BA	0.02	4.3	0.371	1.1539	0.2690	0	−0.1300
PS, *η* = 2.1	0.814	3.8	0.767	3.6808	0.0336	0	−0.9636
PS, *η* = 2.5	0.767	4.7	0.871	3.2478	0.1135	0	−0.9468
PS, *η* = 3	0.716	5.7	0.903	2.2001	0.2967	0	−0.9387
YouTube	0.0808	6.5	0.687	12.8101	0.0159	0.9973	−0.8805
Twitter	0.383	4.5	0.793	4.3877	0.3889	0.6970	−0.7461
DBLP	0.6324	8	0.813	1.8607	0.6689	0.8850	−0.5242

##### Degree Distribution

**Figure 5:**
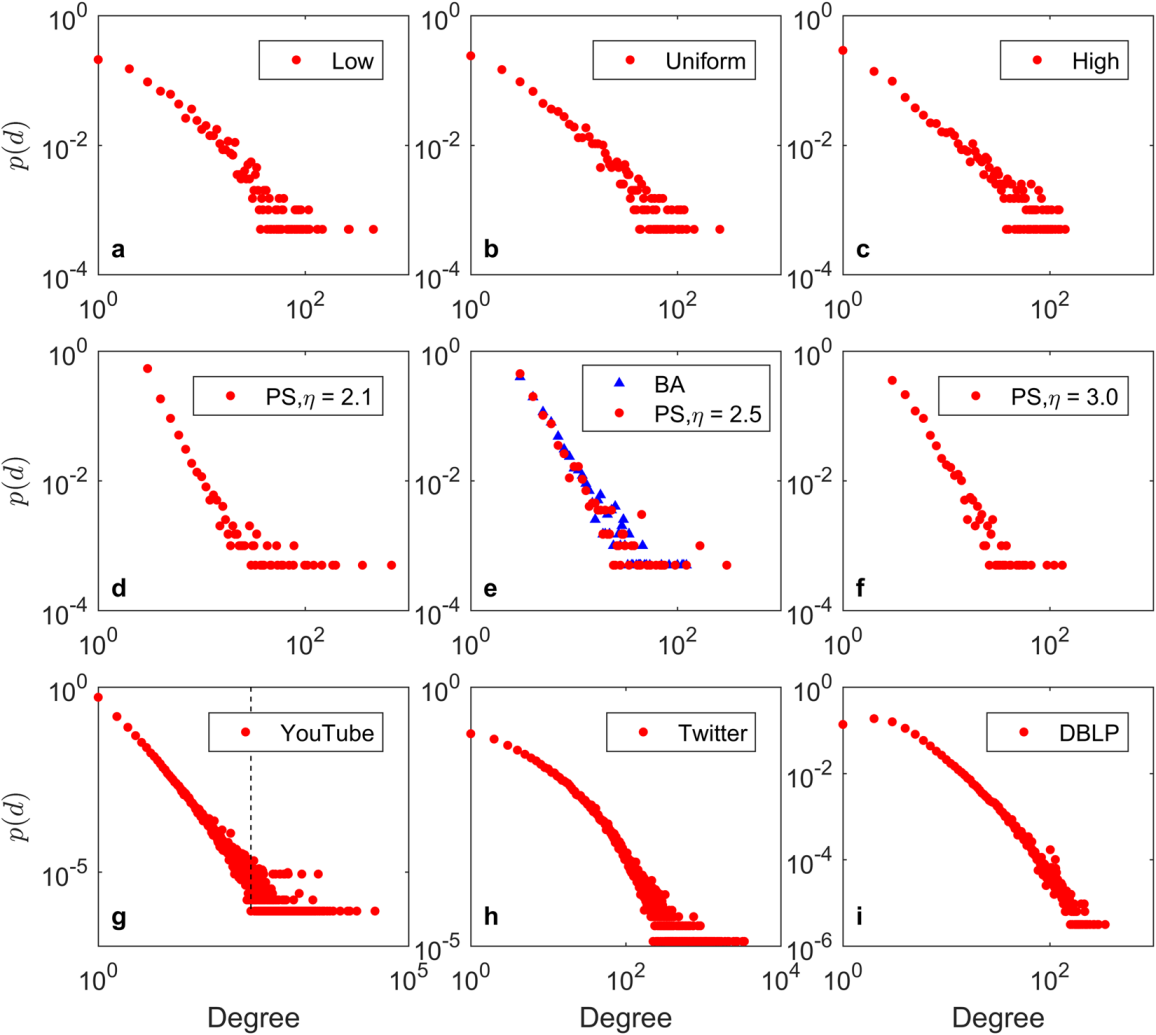
Degree distributions for (**a–c**) HP, (**d–f**) PS and BA, (**g**) YouTube, (**h**) Twitter and (**i**) DBLP.

Figure 5(a–c) shows a double logarithmic plot of the empirical degree distributions for three networks generated by the PH model with the ‘low’, ‘uniform’ and ‘high’ distributions. The power-law exponents are 2.41, 2.48, and 2.63, respectively. Figure 5(d–f) shows the degree distributions of the PS and BA models. Both models follow the power-law distribution; the power-law exponents are 2.49, 2.77, 2.96 for the PS model and 2.76 for the BA model. Figure 5(g–i) shows the degree distributions for YouTube, Twitter and DBLP. YouTube follows the power-law degree distribution with a power-law exponent of 2.14. The degree distributions for Twitter and DBLP exhibit steep downward trends in the tails.

When the popularity mechanism is dominant in the networks, as in Fig. 5(a,d,g), the tails (i.e., the high-degree parts) of the degree distributions are relatively longer because some nodes have very high degrees. In contrast, as the homophily mechanism becomes more dominant in the networks, as in Fig. 5(c,f,i), the tails (i.e., the high-degree parts) of the degree distributions are gradually shorter, and the relative degree of the maximum degree node decreases. This result is consistent with previous research^36^. Both the HP and PS models are able to generate networks that are similar to real social networks.

The plots of the degree distributions show little difference when the degrees of the nodes are small; however, the tails of the degree distributions have different patterns. To quantitatively measure the differences between the tails of two different degree distributions, we focus on the nodes with large degrees. Given a degree distribution, we compute a degree *q* that is the minimum value in which $p(q)=\,\min \,\{p(d)\}$ in the degree distribution. We then compute the skewness^37^ of the nodes with the degrees no fewer than *q* as a proxy of the relative length of the tail of the degree distribution. For example, in Fig. 5(g), we select all the nodes whose degrees are no smaller than the degree of the black dotted line to compute the skewness of the tail of the degree distribution of YouTube. Intuitively, the larger this skewness value is, the longer the tail of the degree distribution is. Table 3 shows the skewness (*SK*) of the tail of the degree distribution for each network.

##### Degree Correlation

**Figure 6:**
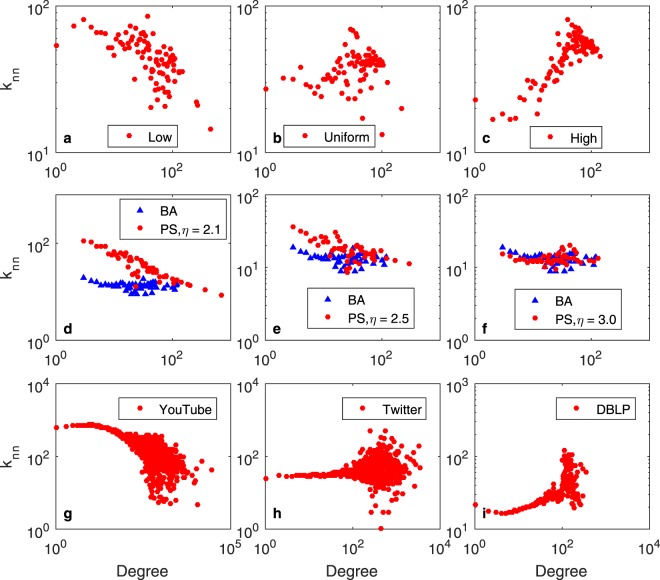
*k*_*nm*_ for (**a–c**) HP, (**d–f**) PS and BA, (**g**) YouTube, (**h**) Twitter and (**i**) DBLP.

Since the size of a real social network is finite, the direct evaluation of the degree correlation will lead to extremely noisy results^34^. Thus, this correlation is usually measured by *k*_*nn*_, the average degree of the nearest neighbours of nodes with degree *k*. We plot the distribution of *k*_*nn*_ in Fig. 6.

As shown in Fig. 6(a), the network generated by the HP model with the “low” distribution is disassortative. In the real world, many online social networks such as Myspace^38^ and YouTube^39^ exhibit disassortative mixing patterns. In Fig. 6(b), the network generated by the PH model with the “uniform” distribution seems to be mixed, similar to the real social networks Cyworld^38^ and Twitter shown in Fig. 6(h). In Fig. 6(b), the network generated by the HP model with the “high” distribution is assortative, similar to the real social networks Flickr^39^ and DBLP shown in Fig. 6(i).

Figure 6(d–f) shows that neither the BA nor the PS model were able to generate networks with the assortative mixing patterns that many real social networks exhibit.

To quantitatively measure the connection tendency, Li *et al*.^40^ proposed the *S*-Metric. They proved that there is an inherent relationship between the structural metric *S* and the degree correlation. The values of *S* range between 0 and 1. A large *S* value means that high-degree nodes tend to connect to other high-degree nodes. A small *S* value means that high-degree nodes tend to connect to low-degree nodes. The *S-*Metric also functions as an index to measure the extent to which the graph has a hub-like core. For graph *G* = (*V*, *E*), |*V*| = *n*, they define the metric

7 $$\begin{array}{cc}S=\frac{s}{{s}_{{\rm{\max }}}}; & s=\sum _{(i,j)\in E}{d}_{i}{d}_{j},\end{array}$$

where *d*_*i*_ denotes the degree of node *i* and *D* = {*d*_1_, *d*_2_,…, *d*_*n*_} is the degree sequence for *G*. Here, ${S}_{\max }$ is the maximum possible *S*-metric of the graph with degree sequence *D*. The *S*-metric for each network is listed in Table 3.

##### Community Sizes

**Figure 7:**
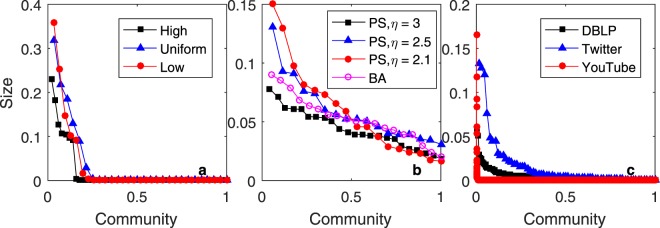
Community sizes for (**a**) HP, (**b**) PS and BA, (**c**) YouTube, Twitter and DBLP.

The community sizes are shown in Fig. 7. The abscissa (horizontal) axis represents the communities as percentages with respect to the whole network, and the ordinate (vertical) axis represents the size of each community as a percentage with respect to the whole network size. In Fig. 7(a), the community size distribution has a long tail, which means that most communities are small and only a few are large. This phenomenon can also be observed in real social networks as shown in Fig. 7(c).

**Figure 8:**
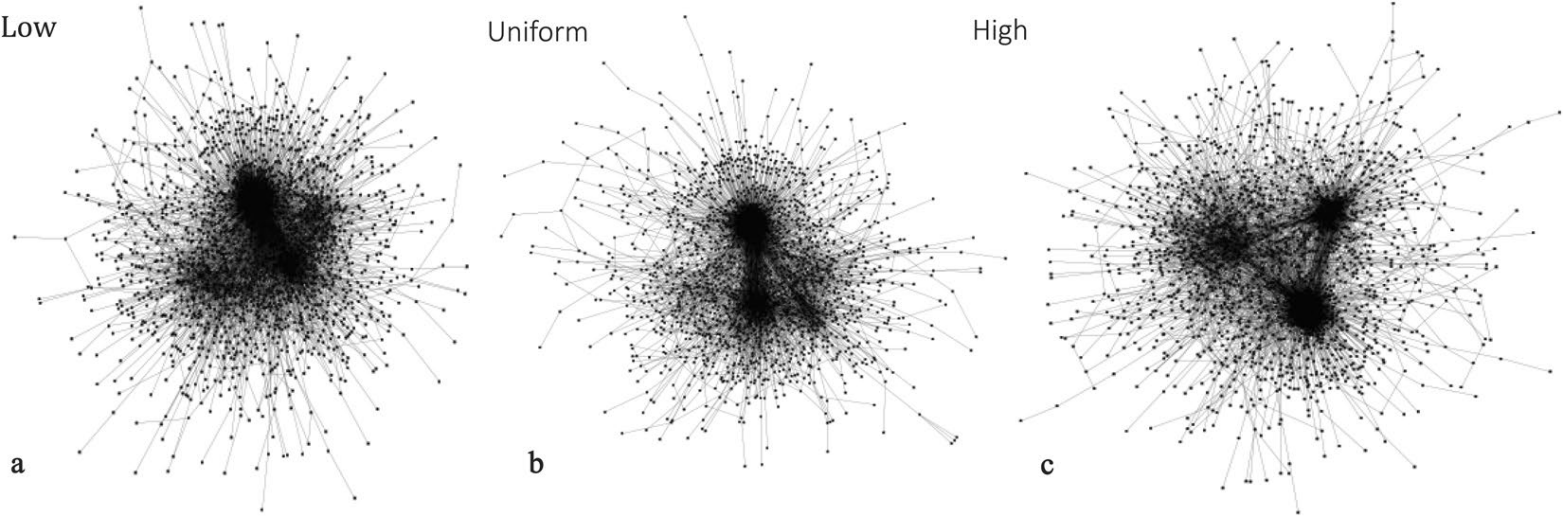
The graphs plotted for three synthetic networks generated by the HP model.

In addition, Fig. 7(c) shows that the size of the largest community on YouTube is larger than that on Twitter and that the size of the largest community on DBLP is the smallest. The popularity mechanism dominates YouTube, while the homophily mechanism dominates DBLP. Generally, the stronger the popularity effect is, the larger the size of the largest community is. We use the HP model with the ‘high’, ‘uniform’, and ‘low’ distributions to generate three synthetic networks. The results of t-tests indicate that the difference in the largest community size is always significant with respect to the ‘high’, ‘uniform’ and ‘low’ distributions used by the HP model. Figure 8 shows the graphs plotted for the three synthetic networks generated by the HP model.

##### Clustering Coefficient

**Figure 9:**
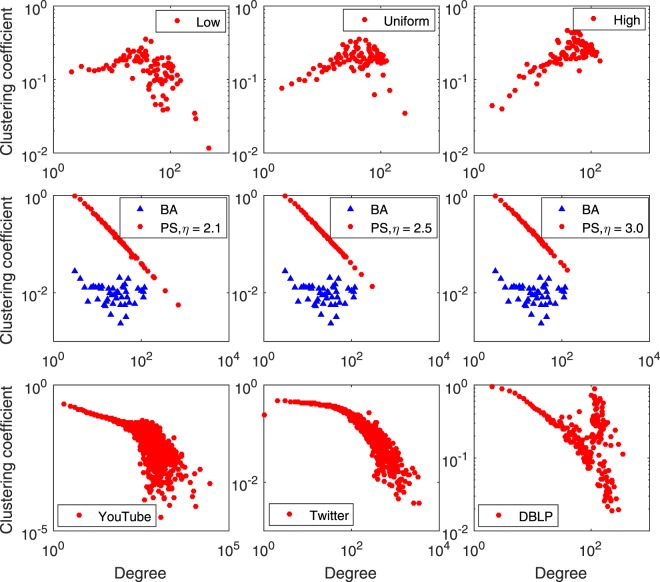
Clustering coefficients for (**a–c**) HP, (**d–f**) PS and BA, (**g**) YouTube, (**h**). Twitter and (**i**) DBLP.

Figure 9 shows the average value of the clustering coefficient for degree-*k* nodes as a function of *k* for the 10 networks.

Figure 9(g–i) shows the clustering coefficients of the three real social networks. The distributions of the clustering coefficients vary on different networks. As shown in Fig. 9(d–f), for the PS model, no correlation exists between the clustering coefficient and the model parameter *η*. The clustering coefficients fit to a straight line in the double logarithmic scale. As shown in Fig. 9(a–c), the HP model can yield different distributions, but these may be significantly different from real social network distributions. One possible reason is that the evolutionary mechanisms in real social networks are much more complicated than those in either the PS or HP models.

#### Quantitative Comparison of Models

We measure the average clustering coefficient (*C*), 90% effective diameter (*D*), and modularity (*M*) for each network. The qualitative results of the degree distribution, community size, and clustering coefficient are shown in Figs 5–9.

To quantitatively compare the generated synthetic networks and the real networks, we collect several additional quantitative metrics. For the degree distribution, we choose skewness(*SK*)^37^ as a quantitative metric. For the degree correlation, we choose the *S*-metric^40^. Regarding community structure, Fig. 7 shows that many small communities exist in the real networks. Therefore, we calculate the number of communities whose size is less than 1% of the network’s size (i.e., the number of nodes in the community is less than 0.01 *N*) and then use the ratio of these small communities to the total number of communities *R* as a quantitative metric for community structure.

As shown in Fig. 9, no known distribution function precisely fits the distribution of the clustering coefficients for real networks. We choose a linear function to fit the data points in double logarithmic coordinates and use the slope (*SL*) of the fitted function as another quantitative indicator of the clustering coefficient. Generally, *SL* measures the trend of the average clustering coefficient as the degree increases.

The results of the aforementioned metrics are listed in Table 3.

We describe each synthetic network and real network with a vector *I* = (*C*, *D*, *M*, *SK*, *S*, *R*, *SL*). This vector enables us to quantitatively measure the similarity between a synthetic network and a real network. It is reasonable to assume that a higher similarity between *I*_*synthetic*_ and *I*_real_ represents the increased accuracy with which the synthetic network models the real network. We employ four popular similarity measures: cosine similarity, correlation coefficient, normalized Euclidean distance, and Mahalanobis distance. The results are shown in Table 4, where many of the cosine similarity and correlation coefficient values are larger than 0.9. These results indicate that the synthetic networks model real networks relatively well. However, it also indicates that the cosine similarity and correlation coefficient metrics are not sufficient to significantly differentiate different models. The reason is that the scales of all the metrics are not the same. The normalized Euclidean distance and Mahalanobis distance are able to overcome this problem and provide a better means of differentiating between different models. The results are shown in Table 4.

**Table 4:** The similarity between different synthetic networks and real networks.

methods	networks	HP, low	HP, uniform	HP, high	BA	PS, *η* = 2.1	PS, *η* = 2.5	PS, *η* = 3
Cosine Similarity	YouTube	**0.9487**	0.8292	0.5760	0.6666	0.9239	0.8633	0.7341
	Twitter	**0.9950**	0.9589	0.8023	0.8641	0.9867	0.9762	0.9154
	DBLP	0.8497	0.9544	0.9864	**0.9904**	0.8466	0.9186	0.9790
Correlation Coefficient	YouTube	**0.9311**	0.7525	0.3892	0.5453	0.8928	0.8065	0.6297
	Twitter	**0.9920**	0.9335	0.6945	0.8130	0.9828	0.9667	0.8804
	DBLP	0.7747	0.9370	0.9805	**0.9892**	0.7772	0.8817	0.9707
Standardized Euclidean Distance	YouTube	**3.5469**	4.3079	5.2944	5.1290	4.7167	4.5589	4.6780
	Twitter	**2.2340**	2.4275	3.5235	3.4609	2.6436	2.3882	2.3571
	DBLP	4.3033	3.5833	3.3946	5.0420	4.8061	4.1326	**3.2649**
Mahalanobis Distance	YouTube	4.1783	**3.5138**	3.9157	4.1972	4.1911	3.7273	3.8089
	Twitter	4.1129	**3.3717**	3.9403	4.2148	4.0978	3.9284	3.5707
	DBLP	3.9560	3.8325	3.5273	4.2298	4.0650	3.8620	**3.4794**

The PS model with *η* = 3 fits DBLP the best for both the normalized Euclidean distance and Mahalanobis distance, but the BA model fits DBLP the best for both cosine similarity and the correlation coefficient.

Because this phenomenon may be the result of failing to calibrate the models for real networks, we conduct additional experiments in which we first calibrate the PS and HP models to real networks and then compare all the models.

##### Model calibration

The process for calibrating the model parameters are as follows:

The PS model has three parameters: *m*, *η* and *N*, where *N* is the number of nodes, and *m* is the number of new links added in each time step. The PS model assumes that when a new node enters the network, it will connect to *m* old nodes, where *m* is a constant. Thus, for the PS model, the relationship between the number of nodes *N*_*t*_ and the number of links *M*_*t*_ is *M*_*t*_ = *mN*_*t*_. In real social networks, the number of nodes and the number of links are known. Hence, we compute the corresponding *m* and use that as the parameter value in the PS model. The parameter *η* works with respect to the parameter *β*′, which controls the relative contributions of popularity and similarity, where *η* = 1 + 1/*β*′. Papadopoulos *et al*.^4^ showed that the power-law exponent of the degree distribution of the PS simulation network is *η*. We estimate the power exponent for each real network using the MLE method and use the value as the value of *η* in the PS model.

There are three important parameters in the HP model correspond to those in the PS model: the parameter *k* controls the relation between the number of nodes and the number of links; the parameter *β* controls the relative contributions of popularity and similarity; and the parameter *N* controls the number of nodes in the network. Without loss of generality, we assume that the other parameters of the HP model are fixed and are identical to the initial settings described at the beginning of this section.

The relationship between the number of nodes and the number of links in the HP model is ${M}_{t}={{N}_{t}}^{k}$. Hence, we are able to calculate the parameter *k* by substituting the number of nodes and links in the real network datasets for *N*_*t*_ and *M*_*t*_, respectively. The three different distributions of *β* correspond to the three cases (‘high’, ‘uniform’ and ‘low’) of the HP model. The previous experiments show that the degree correlation is closely related to *β*: the synthetic networks generated by the HP model with the ‘low’ distribution are disassortative, and the synthetic networks generated by the HP model with the ‘high’ distribution are assortative. Hence, we calculate the *S*-Metric for each real network. When the value is significantly smaller than 0.5 (i.e., from 0–0.35), the HP model selects the ‘low’ distribution to model the corresponding real network, and when the value is significantly larger than 0.5 (i.e., from 0.65–1), the HP model selects the ‘high’ distribution to model the network. Otherwise, the HP model selects the ‘uniform’ distribution to model the network.

We use four real social networks to calibrate the parameters of the PS and HP models. The basic information of the real networks and the corresponding parameters of the PS and HP models are shown in Table 2.

The number of nodes of the Facebook dataset is 4,309. We set the corresponding number of nodes for both the PS and HP models to be 4,309. For other datasets, we set *N* = 5,000.

##### Quantitative comparison after calibrating

To further verify whether this parameter adjustment process is appropriate, we use the ‘high’, ‘uniform’ and ‘low’ distributions to fit the four real networks. The properties of the real networks and generated synthetic networks are shown in Table 5. In Table 5, PS_YouTube refers to the PS synthetic network for YouTube, HP_YouTube_H refers to the HP synthetic network for YouTube with the ‘high’ distribution, and so on.

**Table 5:** The properties of each network (after calibrating).

	*C*	*D*	*M*	*SK*	*S-Metric*	*R*	*SL*
YouTube	0.0808	6.5	0.687	12.8101	0.0159	0.9973	−0.8805
PS_YouTube	0.817	3.91	0.844	5.6875	0.0196	0.0400	−0.9677
HP_YouTube_H	0.013	7.84	0.639	0.8795	0.6070	0.7549	0.6438
HP _YouTube_U	0.012	7.34	0.623	1.5134	0.5192	0.8370	0.4020
HP _YouTube_L	0.015	6.84	0.609	3.2060	0.2197	0.7303	−0.1101
Twitter	0.383	4.5	0.793	4.3877	0.3889	0.6970	−0.7461
PS_Twitter	0.824	2.04	0.737	6.5272	0.0459	0	−0.8860
HP _Twitter_H	0.104	5.02	0.499	0.8316	0.6866	0.8889	0.2423
HP _Twitter_U	0.121	4.64	0.5	2.3294	0.5377	0.8571	0.0905
HP _Twitter_L	0.154	3.90	0.551	5.8476	0.1760	0.75	−0.2019
DBLP	0.6324	8	0.813	1.8607	0.6689	0.8850	−0.5242
PS_DBLP	0.704	6.7	0.939	2.2737	0.3244	0	−0.9415
HP _DBLP_H	0.014	7.51	0.641	1.7318	0.5858	0.8345	0.6693
HP _DBLP_U	0.012	7.22	0.585	2.7413	0.4868	0.34	0.4783
HP _DBLP_L	0.016	6.88	0.588	5.3917	0.2075	0.5424	0.1431
Facebook	0.6055	4.7	0.834	7.7495	0.4882	0.1875	−0.144
PS_Facebook	0.852	1.9	0.669	5.9330	0.0318	0	−0.8974
HP _Facebook_H	0.211	4.4	0.514	1.0271	0.7595	0.8438	0.2439
HP _Facebook_U	0.261	3.77	0.544	4.4871	0.4287	0.5455	−0.0035
HP _Facebook_L	0.353	2.97	0.48	4.0048	0.1858	0.6154	−0.2660

Table 6 shows the normalized Euclidean and Mahalanobis distances between the real networks and the synthetic networks generated by the calibrated models.

**Table 6:** The similarity between different synthetic networks and real networks (after calibrating).

methods	networks	PS	HP _H	HP_U	HP_L
Standardized Euclidean Distance	YouTube	4.3395	4.0624	3.5815	**2.6202**
	Twitter	3.5877	3.6358	3.0428	**2.4703**
	DBLP	3.6503	**2.8582**	4.0894	4.1343
	Facebook	3.8478	4.4572	**3.0492**	3.7747
Mahalanobis Distance	YouTube	4.4552	4.9408	4.4197	**4.0599**
	Twitter	4.0477	3.7353	3.6808	**3.2023**
	DBLP	5.0214	**4.4695**	5.9735	5.3657
	Facebook	4.1591	4.1077	**3.4225**	4.6469

Under the two distance metrics, the optimal fittings for YouTube, DBLP and Facebook are HP_YouTube_L, HP_DBLP_H and HP_Facebook_U, respectively, which are consistent with the results of the parameter-calibrating process shown in Table 2. For Twitter, in Table 2, we select the ‘uniform’ case. However, the optimal fitting is the case ‘low’ under the two distance metrics. The primary reason is that the *S*-metric of Twitter is 0.3889, which is close to the threshold of 0.35 used to differentiate between the ‘low’ and ‘uniform’ cases.

In summary, the HP model is capable of modelling a variety of real social networks.

#### Sensitivity analysis

**Figure 10:**
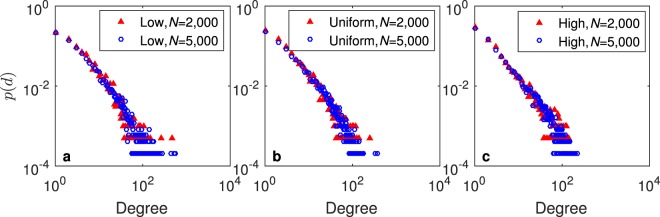
Degree distributions for *N* = 2,000 and *N* = 5,000.

To verify the sensitivity of the networks generated by the HP model to the network size *N* and the threshold ${T}_{2}^{t}$, we conduct the following sensitivity analysis experiments. We use the degree distribution to verify the sensitivity of the network generated by the HP model with respect to the network size *N*. Figure 10 shows the degree distribution for the networks generated by the HP model with the ‘high’, ‘uniform’, and ‘low’ distributions for *N* = 2,000 and *N* = 5,000. As the network grows, the shape of the degree distribution does not change significantly. Hence, the degree distribution of the HP model is not sensitive to the network size.

**Figure 11:**
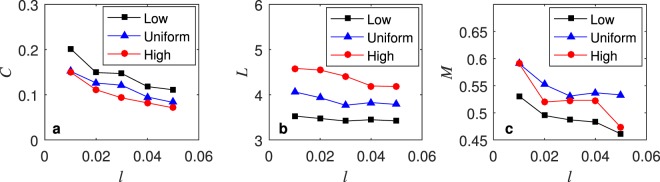
*C*, *L*, *M* for the networks under different *l*.

The threshold ${T}_{2}^{t}$ affects the size of the candidate set during the link-connecting process at time *t*. The larger the candidate set is, the greater the uncertainty is that a given candidate edge will be selected, ${T}_{2}^{t}=\,\max (200,l\,\cdot \,{t}^{2})$. Figure 11 shows the changes in network properties such as the average clustering coefficient (*C*), average path length (*L*), and modularity (*M*) as ${T}_{2}^{t}$ changes when *l* is between 0.01 and 0.05. As shown in Fig. 11, when *l* increases, the randomness of the edge connection increases, which causes *C*, *L*, and *M* to decrease slowly.

## Discussion

Because the homophily and popularity effects are known to be two important evolutionary mechanisms in many real social networks, we develop the HP model for generating social networks. The HP model is capable of reproducing many of the important structural properties found in real social networks.

Our experiments show that although the networks generated by different evolutionary mechanisms are similar along aspects such as high average clustering coefficients, small average path lengths, and significant community structures, they can vary in other aspects. Particularly, for both artificial networks generated by HP models and real-world social networks such as YouTube and DBLP, when the homophily mechanism is dominant during the network growth process, the resulting network will be assortative, and when the popularity mechanism is dominant during the network growth process, the resulting network will be disassortative.

In addition to generating synthetic networks, the HP model provides a new framework for studying social network evolution. For example, in this paper, the probability density function for each node denoting the favour of homophily is simply assumed to be monotonically increasing, uniform, or monotonically decreasing. With further enrichment from related studies of popularity and homophily, the HP model may incorporate other probability density functions that may more accurately model the real social network evolution.

This paper also provides several managerial implications for social marketing. Our study suggests that the degree correlation can distinguish whether the homophily or the popularity effect is dominant during network generation. Both the homophily and popularity effects can explain the phenomenon that consumers tend to make similar purchase decisions as their friends but can also result in different marketing and promotion strategies^15^. When the homophily effect is the main reason for network generation, people who are connected will be more likely to have similar product tastes. In this case, retailing companies should target an existing consumer’s friends directly. When popularity is the main effect, then a consumer’s purchase decision may be altered by that customer’s friends through their communications. In this case, retailing companies should target existing consumers and incite them to persuade their friends to make purchases. Hence, when a retailing company wishes to promote their products on a social network, they can use the degree correlation of the network to determine which promotion strategy to use.

Our study has some limitations that provide avenues for future research. First, the HP model has several input parameters. In future work, we will investigate the effectiveness of each of these parameters to develop new robust models with fewer parameters. Second, similar to other models, the HP model does not perform particularly well with respect to the clustering coefficient. We plan to conduct more research to address this issue.

## Methods

### Data Availability

To determine the power exponent *k*, we calculated the power exponent of 9 real social networks acquired from http://snap.stanford.edu/data/index.html. We selected four real-world representative social networks with distinct characteristics for our experiments: YouTube, DBLP, Twitter and Facebook (all acquired from http://snap.stanford.edu/data/index.html). YouTube is a popular video-sharing social network to which users can upload original videos, follow interesting users, and watch videos posted by other users. DBLP is a comprehensive database of research papers in computer science. Two authors are connected on DBLP if they have co-authored at least one paper. Twitter is a microblogging social network where users post and interact with others through messages. Facebook is an online social media where users can share news and pictures with others. YouTube, DBLP, and Facebook are undirected networks, while Twitter is a directed network. Table 2 shows the basic information for these four types of networks.

## Electronic supplementary material

Dataset 1
