# Supplementary material for: The Competition of Homophily and Popularity in Growing and Evolving Social Networks
Source: Sci Rep. 2018 Oct 18;8:15431. doi: 10.1038/s41598-018-33409-8 (PMC6194079; doi:10.1038/s41598-018-33409-8)
Supplement: Supplementary file 1 — Dataset 1 [file 41598_2018_33409_MOESM1_ESM.pdf]

Title: Homophily-Popularity：A Generation Model for growing and Evolving Social Networks
Authors: Yezheng Liu, Lingfei Li, Hai Wang, Chunhua Sun, Xiayu Chen, Jianmin He, Yuanchun Jiang

This file contains the tags for Weibo users. The data is formatted one user per line.The users’ names and other personal information are not provided in the dataset.
新闻趣事,搞笑幽默,天津生活
IT 数码
名人明星
健康,投资理财,旅游,美食,汽车
旅游
文艺,大学生,历史,法律,旅游,自由,读书,网络,VOA
文艺,美食
时尚,教育就业
军事
其他生活
时尚,娱乐,新闻资讯,旅游,文艺,投资理财
体育,MVP,第一控卫,篮球 NBA,双鱼座,冷酷,帅,麦迪,篮球,NBA
时尚,汽车,军事,IT 数码,文艺,体育
旅游
福建生活
搞笑幽默,新闻趣事,名人明星,穿衣美容
美食,游戏动漫,娱乐
IT 数码
美食,大叔控,美剧,留学,瘦身,狮子座,乐观,吃货
美女
投资理财,汽车,军事,IT 数码,美女
体育,美女,汽车,军事,IT 数码
时尚
美食
时尚,文艺
体育
茶文化,紫砂壶
手机控,宅男,旅游美食,股票,投资理财,健康
健康,娱乐,旅游,新闻资讯,投资理财
娱乐
旅游,福州生活,娱乐,IT 数码
动漫
娱乐,军事
名人明星,娱乐
美食,旅游,健康,投资理财,时尚
钓鱼岛

娱乐,新闻资讯,旅游,星座命理,美食,IT 数码,文艺
军事,旅游,IT 数码,美女,汽车
美食,手机,羽毛球
新闻资讯,娱乐,汽车,美食,旅游
名人明星
广西生活
投资理财,IT 数码,美女
健康,养生,时尚
旅游
健康,美女,投资理财,IT 数码,军事
健康,投资理财
旅游,娱乐
美容服饰,美食,云浮生活
新闻趣事,校园生活,视频音乐,体育资讯,搞笑幽默
汽车
美女,健康,美食
美食
微博奇葩
新闻资讯,体育
电影
健康,旅游,新闻资讯,时尚,娱乐
文艺
湖北生活
美食,汽车,旅游
军事,IT 数码,旅游
美食
搞笑幽默,视频音乐
投资理财,汽车,体育,IT 数码,军事
新闻资讯
搞笑幽默
军事,历史,呵呵
旅游,湖南生活,穿衣美容
旅游
读书分享
新闻资讯,娱乐,健康,教育就业,美食,文艺,旅游
新闻资讯,健康,娱乐,美女,时尚,政治,创意,爬山,旅游,美食
美女
投资理财,娱乐,旅游,时尚,文艺
其他生活
娱乐,偶喜欢玩
体育,健康,新闻资讯,旅游
健康,旅游,美女,军事
时尚,新闻资讯,健康,美食,文艺
娱乐

美食,投资理财,旅游,娱乐
旅游,时尚
美女
北京生活,健康,投资理财,旅游,美食,新闻资讯,汽车
新闻资讯,时尚,文艺,旅游,健康
健康,投资理财,旅游,美食,文艺
旅游,美食
旅游
美食
看电视剧，上网,爱睡觉
星座控,勋鹿党,喜欢唱歌,行星饭
汽车
美女
星座命理,文艺
军事,美女,健康,时尚,旅游
新闻趣事,校园生活,搞笑幽默,无锡生活,美食
登山,看电影,红酒,新闻资讯,健康,旅游,投资理财,文艺,流浪,玩
汽车,美女
旅游
汽车,游戏动漫,美女,军事
美食,投资理财,旅游,IT 数码
美食
体育,爱女儿,吃海鲜,乾隆控
美食
宠物图集,读书分享,星座命理,娱乐,文艺
体育
旅游
军事
旅游
新闻资讯
IT 数码
美食,旅游,搞笑幽默,娱乐
嘉兴生活,美女
教育就业,投资理财,美女,美食,娱乐
汽车
汽车,文艺,娱乐
娱乐
游戏动漫,IT 数码
音乐控,活泼,90 後,暮光之城,爱旅行,猫咪,书籍,水瓶女
军事,旅游,美女
听歌
美食,星座命理,健康
自由职业
新闻资讯,汽车,军事

游戏动漫
投资理财
汽车,美食,投资理财,旅游,健康
旅游
平面设计,插画,网球,学生一枚,旅游美食爱好者,萌宠,游戏动漫
健康,旅游,文艺
红酒,单纯,双鱼女,睡觉,自由,时尚,美食,旅游,80 后,外贸
平常心,吃货,偶喜欢玩
重庆生活
美女
投资理财,美食,体育,汽车,军事,文艺
汽车,游戏动漫,军事,娱乐
视频音乐,美食
90,美食,旅游,动漫,看书,知青愤青文青,学生
搞笑幽默,娱乐
军事
微博奇葩,搞笑幽默,星座命理,新闻趣事,穿衣美容,文艺,投资理财,健康
美食,IT 数码,旅游,随性
旅游,军事,文艺,娱乐
微博奇葩,穿衣美容,美女,新闻趣事,搞笑幽默,广东生活
娱乐,健康,星座命理,投资理财
健康,新闻资讯,军事
健康,时尚,美食,教育就业,旅游
名人明星,育儿百科,南昌生活,美食,美容服饰
穿衣美容
天津生活,美女,新闻趣事,搞笑幽默,微博奇葩
微博奇葩
读书,咖啡,奋斗,真诚
IT 数码,体育
美女,美食
美食
IT 数码,娱乐
健康,旅游
新闻资讯
新闻资讯
投资理财
新闻资讯,美女,娱乐,文艺,体育
新闻资讯,旅游,健康,美食,娱乐
娱乐,军事,IT 数码,文艺,体育
投资理财,旅游,汽车,IT 数码
韩国生活
美食
汽车
新闻趣事,穿衣美容,美食,旅游,微博奇葩

上海生活
文艺,美女
微博奇葩,疯疯癫癫
美食,旅游,汽车,体育
美食,80 后,Music,健康
新闻趣事
旅游,健康,美女,文艺,军事
健康,投资理财
宅,新闻趣事,搞笑幽默
健康,时尚,星座命理,美食,娱乐
医疗卫生保健,网购,公益,教育就业,赚钱,创业,健康,美容护肤,时尚潮流,服装
微博奇葩,搞笑幽默,旅游,美食
新闻资讯,健康,美食,投资理财,游戏动漫
正宗吃货一枚,旅游,学生
美食
大数据,教育就业
单身,Supreme 脑残粉,处女座,美食
辽宁生活
钟汉良,逛街,睡觉睡到自然醒,外貌协会,财经,旅游美食
旅游
IT 数码,美食,旅游,汽车,娱乐
旅游,星座命理,美食,文艺,娱乐
旅游
萌宠,美食
美食
天枰座,穿衣美容,星座命理,旅游,美食
新闻资讯,星座命理,美食,投资理财,旅游
教育就业,新闻资讯,星座命理,游戏动漫,娱乐
名人明星
文艺,星座命理
商业管理
体育资讯
军事
军事,旅游,娱乐,体育,文艺,IT 数码,时尚,汽车
搞笑幽默
健康,时尚,旅游,星座命理,美食,娱乐
旅游,懒人,水瓶女,爱旅行
娱乐
旅游
游戏动漫,散仙闲客
美食
摄影爱好者,幽默,音乐,好性格,偶喜欢玩,双子座,网购,吃,唱歌,上网
北京生活,旅游,文艺,新闻资讯,美食
汽车,新闻资讯,美女,娱乐

旅游
水瓶座
教育就业,健康,美食,旅游,时尚
投资理财,教育就业,汽车
健康,游戏动漫,旅游,美食,投资理财,活得精彩。。。
美食,IT 数码
星座命理
游戏动漫
时尚,星座命理,美食,IT 数码
健康
瞿诗溢,射手座
自由,美食,旅游,娱乐
投资理财,教育就业,汽车,旅游,IT 数码,体育
星座命理
美女,美食
美食
娱乐
时尚
旅游摄影,无聊,巨蟹,拍照,娱乐,美食,汽车
体育,娱乐
健康,美食,美女,游戏动漫,娱乐
美食,新闻趣事,穿衣美容,搞笑幽默
视频音乐
IT 数码,美女
投资理财,旅游,美食,娱乐,健康
美食,旅游,上海生活
旅游,娱乐,新闻资讯,文艺,体育
游戏动漫
美女,健康,军事,美食,体育
体育,动漫,运动
旅游,销售,摄影师,摄影控,计算机,旅游摄影,旅游爱好者,狮子座,看电影
教育就业,IT 数码,体育,娱乐
搞笑幽默
新闻资讯
星座命理,搞笑幽默
美食
星座命理,投资理财,IT 数码,美食,娱乐
新闻资讯,美食,娱乐,投资理财,星座命理
搞笑幽默,新闻趣事,微博奇葩,湖南生活,新闻资讯,美女,游戏动漫,IT 数码,军事
美食
exo,名人明星
军事
搞笑幽默,河南生活
投资理财,时尚,旅游,娱乐,美食

投资理财,旅游
新闻资讯,娱乐,体育,美女
旅游
健康,教育就业,投资理财,新闻资讯,军事
房地产,法律,书籍,低调,北京,处女座,成都,努力,自信,北京生活
旅游
美女
完美主义,足球,健康,投资理财
旅游,汽车,娱乐
教育就业,新闻资讯,军事,美食,健康
娱乐,星座命理,美女,游戏动漫,体育,篮球!!!
手机看小说,汪苏泷歌迷,许嵩歌迷,90后,闷骚,旱鸭子,各种宅,学生一枚,双鱼座,睡懒觉
旅游
健康养生,财经资讯,爱生活,家居,玉,翡翠,银饰品,菩提珠,佛珠,水晶饰品
时尚
其他生活
美女
文艺,学习沉默,永不妥协,梦想更独立
星座命理
美食
IT数码
健康
汽车,旅游,健康
写作,紫色,艺术,创业,广告,上网,小说
时尚,网购,美女,听歌
投资理财,时尚,健康,旅游,美食
军事,投资理财
娱乐,美食,旅游
娱乐,投资理财,IT数码,美食,旅游
美女,美食,IT数码,军事,新闻资讯
投资理财,星座命理,文艺,健康,体育,电影,咖啡
军事,新闻资讯
时尚,旅游
美食,军事,IT数码,体育,娱乐
美食,旅游,娱乐
星座命理,穿衣美容,美食,微博奇葩,健康,旅游
旅游摄影,巨蟹,喜欢唱歌,笑话,90後,淘宝控,活泼,阳光,广东生活
成都生活
美食,完美主义
校园,紫色,白羊座,学生,营销,运动,幽默,艺术,自由职业,生活
浙江生活
时尚,美食,健康
美女
旅游

汽车,旅游,美食,体育,娱乐
旅游,美食
美食,旅游,唱歌,时尚,睡觉
美食,旅游,新闻资讯,美女,IT 数码
汽车,游戏动漫,体育,娱乐,旅游
时尚,美食,娱乐
美食
文艺,星座命理,旅游
美食,旅游
健康,新闻资讯,投资理财,教育就业,军事,IT 数码
旅游
投资理财,汽车,旅游,美食,娱乐
旅游,健康,汽车,投资理财,美食
娱乐
时尚,旅游,汽车
视频音乐
教育就业,体育,IT 数码,投资理财,健康
旅游,IT 数码,美食,时尚,娱乐
美食
疯狂,无厘头,努力,旅游,娱乐,投资理财,新闻资讯,军事,体育,美女
健康
文艺,时尚,旅游
美食,时尚,健康,旅游,娱乐
视频音乐
美食,风景,泰剧
幽默
体育
信阳生活
新闻趣事,新闻资讯
浪漫,睡觉,平常心,双鱼座,自由职业,旅行,时尚,80 后
投资理财
教育就业,健康,旅游,美食,娱乐,盗版吃货，臭美
其他生活
新闻资讯,美女,IT 数码,军事,旅游
娱乐,投资理财,新闻资讯,健康
搞笑幽默,美食,萌宠
娱乐
体育
大学生,宅
娱乐
文艺
新闻趣事,浙江生活,搞笑幽默,军事,IT 数码,健康,新闻资讯
彩妆控,彩妆护肤,淘宝店主,巨蟹座女生,时尚潮流,美食,穿衣美容
美食,旅游

江苏生活
娱乐
美女,时尚,健康,教育就业,投资理财,体育
健康,旅游,教育就业,新闻资讯,娱乐
名人明星
搞笑幽默
娱乐
吃,上网,80 后,手机,时尚,美食,写作,交友,天秤座,旅行
娱乐,汽车,游戏动漫
投资理财,美女,汽车,军事,IT 数码
军事
IT 数码,历史,摄影,广告,时尚,体育,搞笑,旅游,123
亲情,校园,新闻,网购,艺术,交友,唱歌,上网,自由,音乐
体育资讯
欧美音乐,K 歌,拍照,IT 数码,娱乐
从前有个鬼,娱乐
旅游
美食,健康,旅游
美女,新闻资讯,军事,文艺
旅游
湛江生活,美图摄影
健康,星座命理,文艺,教育就业,经典教育,传统文化,私塾,四书五经,国学,经典文化
教育就业,旅游,体育
摩羯座,乐观,创业
旅游,教育就业,军事,新闻资讯
北京生活,教育就业
游戏动漫,美食,健康
旅游,美图摄影
旅游,健康,时尚,美食
军事,汽车,美食,旅游,IT 数码,体育
交友,平常心,睡觉
爱生活,喜欢旅游,旅游美食爱好者,美食,小说,看书
投资理财
自信,IT,世界杯,乐天派,创业,旅游
美女,汽车,时尚,投资理财,文艺
时尚,美食,星座命理,娱乐
军事,体育,娱乐,游戏动漫
IT 数码
旅游
旅游
时尚,旅游
小说,爱旅游,书籍,google,看电视,娱乐,美食,文艺,新闻资讯,旅游
新闻资讯,旅游,军事,IT 数码,美女
娱乐,游戏动漫,美女,体育,文艺

上海生活
娱乐,健康,旅游,美食
网络营销,环保,内向,IT 互联网,shopping,IT 数码
花样美男,双鱼座
IT 数码
新闻资讯,天秤座
投资理财,文艺,美食
新闻资讯,旅游,教育就业
IT 数码
广东生活
四川生活,游戏动漫,旅游,美食,体育
汽车,时尚,旅游,新闻资讯,美女
旅游,星座命理
美食,文艺,时尚,旅游,美食,IT 数码,娱乐
旅游,健康,时尚,体育,娱乐
美食,北京生活
浪漫,娱乐,深圳,完美主义,音乐爱好者,自恋,旅游美食爱好者,睡觉睡到自然醒,自然,笑
美食
IT 数码,宅男,摄影爱好者,自由,水瓶座,设计,数码,上网,80 后
汽车
文艺
教育就业,水瓶座,教师,热情,看书,摄影,自由,旅行,无厘头,活泼开朗
旅游
新闻资讯,娱乐,体育,IT 数码,汽车
旅游
娱乐,文艺,美食,90 後,善良,射手座
旅游
校园生活,视频音乐,美图摄影,名人明星,旅游,八卦,NBA,艺术,时尚
广东生活
健康
IT 数码
健康,旅游,美食,娱乐
IT 数码,旅游,汽车,体育,娱乐
新闻资讯,旅游,IT 数码,投资理财,体育
游戏动漫,军事
娱乐
睡觉,设计,娱乐,旅游,音乐,写作,新闻,时尚
娱乐
旅游,教育就业,汽车,IT 数码,文艺
名人明星
紫色,吃货
火影忍者,爱逗闷子,自以为是,思考以及想象力,找个贤妻良母型,运动,旅行
CalvinKlein,Armani,Burberry,LouisVuitton,Luxury
k 歌,喜欢听歌,收藏,90 后,游戏,网购,善良,双鱼座,吃,视频音乐

新闻资讯
旅游
旅游,中华龙城,景区,服务业
教育就业,娱乐
星座命理,美食,文艺,健康,IT 数码
娱乐
名人明星,视频音乐,新闻趣事,搞笑幽默,湖北生活,体育
美女,新闻趣事,北京生活,美食,旅游
汽车
游戏动漫,军事,新闻资讯,娱乐,投资理财,汽车,IT 数码
职场招聘,体育资讯,搞笑幽默,深圳生活
摄影爱好者,射手女,黄渤,唱歌,吉他
80 后,败家女王,爱电影爱运动,狮子座,扛不起单反,大白目
美女,文艺
IT 数码
旅游
美食
军事
美食,投资理财,文艺,体育,娱乐
文艺
听音乐,喜欢听歌,贝贝宠物,宠物美容,武汉宠物,宠物
旅游,时尚,文艺
美女,汽车,旅游,美食,军事,娱乐
时尚,旅游,新闻资讯,美食,健康,投资理财
旅游,汽车,新闻资讯,健康,IT 数码
美食
汽车,体育
美食,美女,体育,娱乐
星座命理,旅游,健康,时尚,美食
教育就业,新闻资讯,美女,健康,军事,IT 数码
旅游,文艺
星座命理
健康,时尚,投资理财,旅游
乐观,历史,读书写作,交友,徐福研究
旅游,IT 数码,娱乐,健康,新闻资讯
时尚
IT 数码
汽车,新闻资讯,健康,旅游,军事,性格开朗,爱好军事
体育
美食,汽车,娱乐,旅游,新闻资讯
摄影旅游骑行,义工,行善积德,做好事,自由职业,摄影爱好者,乐观,旅游,网购,自由
投资理财,美食,教育就业
旅游,健康,摄影
旅游爱好者,电影控,音乐爱好者,活泼开朗,孩子气,IT 数码,时尚,美食,文艺,娱乐

视频音乐,穿衣美容,英语,电影,八卦,时尚,水瓶座,宅,上网,娱乐
投资理财,时尚,军事,体育
旅游
IT 数码,汽车,旅游,美食
IT 数码,娱乐,时尚,美食,游戏动漫
宅,80 后
游戏动漫,网购,美食,平常心,狮子座,宅女
投资理财,娱乐,时尚,游戏动漫,IT 数码
美女,健康,文艺,体育,娱乐
名人明星
汽车,军事,IT 数码
时尚,美女,文艺,娱乐
edc,Eason,easo,90 後
游戏动漫,旅游,军事,美女
IT 数码
美食
时尚
电影迷,吉他,音樂
旅游
IT 数码
星座命理,汽车,美女
健康,美食,文艺,旅游
旅游爱好者,健康,看书,射手座,旅行,羽毛球,平常心,听歌,偶喜欢玩,运动
美女
时尚,IT 数码,旅游,文艺
娱乐,IT 数码,时尚,美女
娱乐
汽车,美女,IT 数码
旅游,游戏动漫
娱乐
娱乐
教育就业,文艺,IT 数码
IT 数码,汽车
体育
汽车,美女,旅游,美食,体育
娱乐,军事,IT 数码,新闻资讯,美女
体育
旅游,文艺,美食,教育就业,健康
健康,文艺
娱乐
星座命理,七堇年华。,是梦终空、
星座命理,文艺,健康,投资理财,时尚
IT 数码,娱乐,汽车,美女,写字
宁波生活

体育
山东生活
投资理财,时尚,游戏动漫,旅游,军事
健康,旅游,娱乐,音乐,美食,旅行,时尚
美女,文艺,游戏动漫
健康,爱美,爱生活
新闻资讯
游戏动漫
旅游
娱乐
历史,猫,动漫控,狗狗,吃,天秤座,宠物图集,美女,星座命理,美食
IT 数码
健康,投资理财,文艺,体育,娱乐
山顶来的金山人,奢牌,摄影,美食,休闲,汽车
新闻趣事,穿衣美容,旅游,美食,北京生活
星座命理
投资理财
投资理财,旅游,美女,娱乐,军事,美食,汽车,文艺
赣州生活
美食,星座命理,旅游,娱乐,文艺
时尚,文艺,娱乐
娱乐
娱乐
音乐,80 后
IT 数码
网络小说写手,足球,网游,旅行
美食
旅游
萌宠
智慧酒店
娱乐
新闻资讯,旅游,军事
美食,星座命理,新闻资讯,游戏动漫,投资理财
游戏动漫,IT 数码,军事
娱乐,教育就业,文艺,汽车,时尚,健康,旅游,军事
北京生活,娱乐
愛旅遊,成人笑话,丝袜美腿
投资理财,美女,汽车,IT 数码
文艺
旅游,IT 数码,美女,游戏动漫,美食
新闻资讯,游戏动漫,时尚,IT 数码,娱乐
星座命理,美食,体育,文艺,旅游,音乐,电影
蓝色,旅游
美女

旅游,汽车,美食,IT 数码
汽车,娱乐,新闻资讯,美食
美食,时尚,IT 数码,汽车,投资理财
体育
娱乐
游戏动漫
美女
IT 数码
新闻资讯
IT 数码,文艺,游戏动漫
娱乐
美食
健康,投资理财,军事
搞笑幽默
体育,游戏动漫,美女,军事,汽车
IT 数码
IT 数码,体育,魔兽世界
旅游,汽车,善良,美女,听歌
新闻趣事,投资理财
IT 数码
80 后,财经,英语,摩羯座,微博,网购,看书,旅游
体育,dota,足球,好性格,游戏,爱唱歌,双子
军事,新闻资讯,娱乐,美女
音乐
旅游
美食
美食
创业,财经,营销,80 后,自由
军事,旅游,新闻资讯,教育就业,投资理财
地产
汽车,新闻资讯,军事,体育,娱乐
IT 数码,健康,旅游,体育,军事
娱乐,汽车,IT 数码
娱乐,健康,美食,IT 数码,游戏动漫
美食
拍照,旅游,旅游摄影
娱乐
娱乐
教育就业,娱乐,汽车,投资理财,IT 数码
体育,娱乐,美女
热情,新闻资讯,投资理财,教育就业,旅游,美女,体育
美女,游戏动漫,旅游,星座命理,娱乐
汽车
文艺

潜水控
宅,旅游,美食,听歌,网络,新闻,美女,微博奇葩
旅游,新闻资讯,美女,IT 数码
旅游
旅游,新闻资讯,投资理财,文艺,IT 数码,羽毛球,交友,新闻,旅行
IT 数码
文艺,游戏动漫,旅游,美食,娱乐
美食,娱乐,bigbang
搞笑幽默,旅游,健康,体育,军事,教育就业
搞笑幽默
体育
文艺
旅游
娱乐,游戏动漫
新闻资讯,汽车,投资理财,军事,体育
美女,旅游,体育
文艺
娱乐
时尚,旅游,美食
名人明星
汽车,军事,IT 数码
旅游,文艺,美食
星座命理,文艺,美食,娱乐
文艺
时尚
美食,美食,旅游,游戏动漫
军事
江苏生活
感性,阿森纳,实况足球,摩羯座,有理想,潜力股
新闻资讯,美食,教育就业,健康,娱乐
美女
星座命理,健康,旅游,文艺,娱乐
体育
80 后
健康,美女,时尚
旅游,美食,80 后的尾巴
健康
搞笑幽默,IT 数码
IT 数码
美食,旅游,游戏动漫
新闻趣事
听歌,音乐
水瓶座加 A 型血,80 后
投资理财,旅游,娱乐,星座命理,美食

娱乐
体育
新闻资讯,娱乐,IT 数码,美食,健康,汽车,时尚,美女
健康,新闻资讯,娱乐,文艺,军事,体育
军事,游戏动漫,汽车,IT 数码,娱乐
星座命理
旅游
美食,IT 数码,文艺,体育,娱乐
健康,美女,汽车,时尚,IT 数码
好书
体育
旅游,健康,新闻资讯,美食,体育
体育,NBA,天枰座,校园,美食,旅游
文艺,时尚
名人明星,IT 数码
体育
旅游
校园生活,视频音乐,新闻资讯,IT 数码,投资理财,美女
时尚
旅游
健康,投资理财,旅游
汽车,悦翔 V3,奔奔,CX20,CX30,悦翔,逸动,长安汽车
新闻资讯,美食,军事,合肥人,忧国忧民,木工 DIY,摄影爱好者,人到中年,有感而发
军事,新闻资讯,汽车,IT 数码
投资理财,新闻资讯,旅游,美食,IT 数码
健康
听歌,NBA
体育
视频音乐,游戏动漫,娱乐,星座命理,IT 数码,出国党,上外附中,O 型血,法语,摩羯
游戏动漫,美女,体育
体育
汽车
健康,美食,文艺,娱乐
美食,健康,旅游,投资理财,时尚,星座命理,娱乐
动漫迷
旅游,娱乐,投资理财,体育,IT 数码
新闻资讯
体育
汽车,美食,美女,投资理财,娱乐
诚信做人
新闻趣事
美女
社会,新闻趣事
美食

汽车
旅游,体育
冷
视频音乐
旅游,军事,新闻资讯,时尚,娱乐,健康
游戏动漫
文艺,健康,时尚,娱乐,星座命理
美食
健康,新闻资讯,汽车,旅游,体育
舞蹈,教育,美容,真诚,娱乐,健康,星座命理,投资理财,旅游
星座命理
游戏动漫
微博奇葩,汽车
军事
旅游,美食,体育,文艺,娱乐
旅游,美女,健康,时尚,娱乐
VBA,汽车,足球,看书,旅游,体育
广东生活
新闻资讯,美食,游戏动漫,旅游,体育
其他生活,旅游
美女
汽车
星座命理,新闻趣事,搞笑幽默,广东生活,名人明星
烹饪,美国,狗狗
名人明星
IT 数码
健康,文艺,娱乐,时尚
娱乐,军事,投资理财,新闻资讯
旅游
星座运势
美女
视频音乐,JYJ,沈昌珉,金俊秀,朴有天,郑允浩,金在中,东方神起
音乐控,时尚,星座命理,娱乐
体育
健康养生
新闻资讯,投资理财,健康
美女
健康,时尚,旅游,美食,娱乐
新闻趣事,穿衣美容,微博奇葩
文艺,时尚,娱乐
文艺,娱乐
美食
安徽生活
游戏动漫,军事,美食,体育

旅游,健康,教育就业,美食,新闻资讯
体育,汽车,旅游,军事,投资理财
旅游,美女,美食,娱乐,汽车
A 股,股票
旅游
爬山
新闻趣事,旅游,萌宠,名人明星,广州生活
穿衣美容,IT 数码
微博奇葩,旅游,视频音乐,美食,肇庆生活
搞笑幽默,微博奇葩,新闻趣事,娱乐
娱乐
IT 数码,汽车,美食,娱乐,体育
红楼梦
投资理财,健康
旅游
军事
娱乐,摄影,音乐,生活,学生,天秤座
旅游,娱乐,美食,文艺
IT 数码,还有很多很多,处女座,自行车,游戏动漫,喜欢唱歌,唱 K,闷骚,影视,球迷
美食,星座命理,文艺,偶稀饭睡觉觉
旅游
健康
读书分享,美食,搞笑幽默,文艺
名人明星,美食,博爱,西班牙语,双子座,唱歌,艺术,旅游,看书,大学生
IT 数码
教育就业,军事,新闻资讯,汽车,投资理财
文艺,美女,夜猫子,旅游,美食,健康,时尚,娱乐
娱乐
旅游
美女,旅游,军事,文艺,娱乐
新闻趣事
美女
美剧,梦的解析
视频音乐,微博奇葩,美女,搞笑幽默,新闻趣事
时尚,美女,娱乐,教育就业,文艺
旅游
体育
娱乐,旅游,新闻资讯,军事,体育
旅游,文艺,娱乐
旅游
娱乐
健康,电影爱好者,乐观,天秤座,爱玩,篮球,运动,宅女,摄影
名人明星
时尚,旅行,唱歌,自由,河南生活

旅游,娱乐
微博奇葩,娱乐
语录,写作,媒体,传媒,校园
娱乐
军事
体育,IT 数码
美食,健康,投资理财,新闻资讯,旅游
美食,文艺,看书,白羊座,B 型血,旅游
读书分享,旅游,美容服饰,美食,大连生活
汽车,健康,投资理财,星座命理,新闻资讯,旅游
体育
美女
美女,汽车,美食,IT 数码,体育
搞笑幽默
萌宠
游戏动漫
教育就业
投资理财
IT 数码
游戏动漫,体育
娱乐
军事,体育,游戏动漫
投资理财
文艺,旅游,时尚,美食,娱乐
EXO,美食,时尚
时尚,IT 数码,星座命理,美女,投资理财
体育
IT 数码,娱乐,文艺,教育就业,星座命理,直率，画画，学生
旅游
美食
时尚,娱乐,看电影,医生,音乐发烧友,IT 数码,美食,汽车,投资理财,健康
美食,新闻趣事,萌宠,美女,视频音乐
旅游,美食,体育,娱乐,汽车
娱乐
旅游
投资理财,旅游,美食,健康
健康,时尚
旅游,美女,穿衣美容,星座命理,搞笑幽默
IT 数码
投资理财,旅游,星座命理,美食,娱乐
体育
旅游
旅游,新闻资讯,时尚,汽车,娱乐,IT 数码
美食,汽车,旅游

军事,体育
娱乐,时尚,美食,健康,旅游,舞蹈,画画
健康,军事,新闻资讯,投资理财,IT 数码
旅游,美食
娱乐
搞笑幽默
旅游,美食
旅游
汽车,旅游,娱乐,教育就业
美女,娱乐,时尚,游戏动漫,汽车,IT 数码
爱美食,时尚,汽车,娱乐,旅游
美食
视频音乐,新闻趣事,成都生活,搞笑幽默,微博奇葩,摩羯,偶喜欢玩,好性格,阳光
美食,新闻趣事,视频音乐,美女,旅游
文艺
旅游
旅游,旅行
新闻资讯
旅游,体育
投资理财,健康,旅游,美食,娱乐
健康,旅游,IT 数码,娱乐,体育
娱乐
时尚,旅游,健康,汽车,美食,娱乐
旅游
健康,汽车,投资理财,娱乐,IT 数码
娱乐
健康,旅游,美食,军事,汽车
游戏动漫,动漫控,动漫迷
听歌,动漫,魔兽世界,美食,游戏动漫
投资理财
美女
教育就业,投资理财,时尚,新闻资讯,健康
汽车
投资理财,汽车,健康,旅游,美食
投资理财
旅游,美食,穿衣美容,视频音乐,广东生活,时尚
美女,北京生活,体育
囧,爱玩,开朗,孩子气,双子座,做梦,韩剧,交朋友,娱乐,美女
搞笑幽默,广东生活,广告
时尚
教育就业,娱乐,时尚,新闻资讯,健康
睡觉控,吃货,大爱
星座命理
时尚,美女,新闻资讯,文艺,IT 数码

新闻趣事,视频音乐
旅游,文艺,游戏动漫,IT 数码,娱乐
搞笑幽默,体育
喜欢音乐,唱 K,O 型血,梅州生活
美食,文艺,娱乐
搞笑幽默,美食
平常心,旅行
旅游
旅游,教育就业,投资理财,健康,星座命理
新闻资讯
时尚,旅游,健康,汽车,体育
健康,美食,文艺,旅游
娱乐
校园生活,小说,上网,宅,电影,听歌
投资理财,军事,旅游,娱乐,体育
名人明星,萌宠,旅游,新闻趣事
文艺,旅游,健康,新闻资讯,时尚
星座命理
新闻资讯,美食
矛盾体,70 后,搞笑,喜欢唱歌,看书,上网
美食
星座命理,美食
健康,时尚,新闻资讯,娱乐,投资理财
时尚,旅游,健康,星座命理,娱乐
时尚,文艺,娱乐,星座命理,美食,军事
体育
名人明星
穿衣美容,美食,名人明星,美女,视频音乐
美食,健康,旅游,文艺,军事
健康,新闻资讯,IT 数码,汽车
娱乐,游戏动漫,体育
美食
娱乐,体育,文艺
健康,时尚,娱乐,汽车,旅游,美食
教育就业,健康,旅游,星座命理,美食
北京生活,旅游,美食,职场招聘
健康
新闻资讯
美食
美食
新闻资讯
健康,美食,IT 数码,体育,文艺
星座命理
旅游,文艺

军事,体育,健康
美食
娱乐,旅游,星座命理,美食,时尚,老板娘,优质,潮,童装
美女,星座命理,健康,旅游,美食
汽车,体育
八卦杂谈,名人明星,南京生活,新闻趣事,旅游,时尚,新闻资讯
搞笑幽默,新闻趣事,微博奇葩
餐饮管理,必胜客,团购,听歌,时尚,旅游,80 后,美食,音乐,电影
健康,時尚,英语,旅行,学生,射手座
旅游,星座命理,时尚,投资理财,娱乐
旅游,星座命理,新闻趣事,搞笑幽默,浙江生活
新闻资讯
娱乐,音乐
广东生活
美食
八卦杂谈
投资理财,汽车,旅游,美食,文艺
睡觉
时尚,旅游,投资理财,美食
游戏动漫,羽毛球,读书,艺术,看书
星座命理
体育
星座命理,IT 数码,美食,文艺,时尚
体育
IT 数码
投资理财
美食
新闻资讯,健康,旅游,美食,文艺
搞笑幽默,新闻趣事,微博奇葩,萌宠,旅游
财经,交友,旅游,微博,网络,天蝎座,新闻,好书,创业,股票
搞笑幽默,视频音乐,IT 数码
游戏动漫,美女,旅游,体育,IT 数码,双子座,邪恶的善良人
美食,旅游,投资理财,新闻资讯,IT 数码
游戏动漫,新闻资讯,健康,IT 数码
星座命理,美食
娱乐,游戏动漫
娱乐,军事,IT 数码,文艺,体育
搞笑幽默
美食
重庆生活
时尚
健康,娱乐,汽车,美女,旅游
南宁,美食控,健康,养生,美食
星座命理,旅游,美食

南昌生活
孤独的看客
投资理财,教育就业,汽车,军事,IT 数码
军事,投资理财,健康,美女,汽车
娱乐,时尚
DB2,Oracle,汽车,IT 数码
校园生活,搞笑幽默,军事,体育,教育就业,泡图书馆,篮球,幽默,大叔,盗墓笔记
旅游
文艺,财经,80 后,历史,旅游,读书,努力保持小资
健康,星座命理,美食,投资理财,旅游
IT 数码,体育,娱乐,机会永远都有,我喜欢创造机会,旅游,摩羯座,平常心,80 后,足球
军事,美食,旅游,教育就业
汽车,投资理财,美食,旅游,时尚
旅游
游戏动漫,旅游,历史,爱睡觉,哲学,手机看小说,计算机,旅游爱好者
美食
娱乐,时尚,汽车,星座命理,IT 数码
育儿,健康,美食
搞笑幽默
旅游,汽车,美食,军事,体育
健康,投资理财,旅游
汽车,文艺,军事,健康,时尚,投资理财
80 后,旅行,篮球,自由职业,创业,音乐
新闻资讯,汽车,美食
上网
游戏动漫,旅游,娱乐
新闻资讯,美女,汽车,IT 数码,体育
体育,IT 数码
喜欢猫猫狗狗
汽车
撒欢儿,美食
汽车,健康,军事,旅游,体育
视频音乐,旅游,名人明星
视频音乐,投资理财
时尚,军事,娱乐,体育,美女
美女
视频音乐
美女
投资理财,教育就业,新闻资讯,健康,旅游,营销
IT 数码
IT 数码
旅游
娱乐
生活,鼓浪屿,哈哈哈哈哈哈,维尼,淘宝控,习惯爱

听歌,美食,时尚,旅行,旅游,自由,宅,80 后,电影,音乐
娱乐,新闻资讯,投资理财,IT 数码,军事
文艺
健康
娱乐
旅游,美食,娱乐
时尚,美食,军事,文艺,健康
旅游
微博奇葩,文艺,新闻资讯,投资理财,IT 数码
美食
娱乐
旅游,军事,体育
电影,健康,汽车,投资理财,新闻资讯,欧美摇滚乐,旅游
管理咨询顾问,股票,羽毛球,投资理财,旅游
游戏动漫
娱乐
美女
道教画,道人,道教神仙,宗教,道士,道教,法师,高功,画家,风水师
娱乐,美食,时尚
体育,喜欢旅游,闷且浪,电影,篮球
星座运势
星座运势,情感生活,宠物图集,美食,校园生活
IT 数码,上网,看电影,发呆,听音乐,火影忍者,双子,爱睡觉
体育
法律人,苏州律师,律师
宅女一枚,美食,宅,游戏动漫,文艺,娱乐
健康,新闻资讯,娱乐,投资理财,IT 数码
美容服饰,八卦杂谈,爱至上励合,模特,95 后小女生,学生
美图摄影,美容服饰,科学探索,读书分享,旅游,文艺
游戏动漫
IT 数码,时尚,新闻资讯,娱乐,shopping,旅游,宅,听歌,80 后,电影
IT 数码
旅游
体育
IT 数码
娱乐
投资理财,新闻资讯,健康,旅游,娱乐
摄影,旅游,NBA,80 后,美剧,娱乐,生活,文学,英语,美食
娱乐,IT 数码
旅游,时尚,投资理财,汽车,IT 数码
商演,MIDI 音乐制作,天秤座,捕鱼达人,大提琴
新闻资讯
美食,教育就业,娱乐,投资理财,新闻资讯
旅游

娱乐
财经资讯,影视,志愿者,学习,互联网,育儿百科,新闻
健康,美女,新闻资讯,文艺,体育
旅游
音樂,喜欢旅游,爱吃,懒人,神经大条,搞笑幽默
内蒙古生活,看电视
健康,文艺,美食,时尚,吃货,旅游,双子座,80 后
娱乐
人力资源管理,足球,四国军旗,齐秦,NBA
汽车,IT 数码
茶艺茶道,经络,营养,运动,排毒,养心,健康产业
星座命理,娱乐,军事
教育就业,文艺,投资理财,新闻资讯,旅游
健康,军事,汽车,投资理财,体育
IT 数码,星座命理
美食,新闻趣事,搞笑幽默,汽车,军事,健康,投资理财
旅游
搞笑幽默,美食,视频音乐,山东生活
汕头生活
汽车
军事
IT 数码,新闻资讯,美食,旅游
娱乐
育儿百科
娱乐
穿衣美容,美食,星座命理,旅游,视频音乐
沈阳生活,微博奇葩,新闻趣事,搞笑幽默
文艺,投资理财,军事,体育
搞笑幽默
法律,咖啡,射手座,五月天,80 后
旅游
娱乐
文艺,旅游,新闻资讯,军事,IT 数码
搞笑幽默,新闻趣事,美食,旅游,内蒙古生活
教育就业,健康,文艺,娱乐
健康
美食,时尚,旅游,文艺,娱乐
新闻资讯,教育就业,美食,军事,文艺
星座命理
日本生活,微博奇葩
美食,股票,淘宝,dota,咖啡,美剧迷,爱旅行,手工,古灵精怪,龟梨和也
新闻资讯
紫色,网购
文艺,体育,天秤座,财经,历史,电影,旅行,吃货,美食

美女,娱乐
乐观,80后,处女座,听歌,善良,平常心,旅游
星座命理,文艺
美食
美食,健康
娱乐,旅游,美食,文艺,时尚
彩妆爱好者,爱幻想的双鱼座,言情小说爱好者,宅
校园生活
IT数码
时尚,美食,娱乐
军事,IT数码
投资理财,汽车,健康,美食,文艺
健康,新闻资讯,IT数码,听音乐,处女座,韩剧
电影电视迷,狮子座,健康养生,美容护肤,情感,爱幻想,听歌,80后宅女,美食,娱乐
新闻资讯
美女,游戏动漫,IT数码,体育
旅游
军事
健康,投资理财,旅游
旅游,健康,美食,投资理财,时尚
读书分享,美图摄影,美容服饰,大理生活,旅游
名人明星,旅游,视频音乐,新闻趣事,搞笑幽默
体育
军事,体育,IT数码
娱乐
迷糊,喜欢音乐,历史,喜欢旅游,敏感,睡懒觉,小女人,茶,逛街,看电影
视频音乐,旅游
军事,IT数码
投资理财,文艺,IT数码
文艺
教育就业,美女,新闻资讯,健康,IT数码
游戏动漫
体育
娱乐,超级无敌大黑熊,唱歌,篮球,新闻资讯,美女,健康,时尚,体育
旅游,旅行,80后,射手座,吃货,宅
电影控,动漫迷,心理学,宝宝,娱乐
搞笑幽默
运动,汽车,娱乐,语录,人力资源
教育就业,军事
文艺,旅游,星座命理
星座命理,穿衣美容
视频音乐,搞笑幽默,旅游,美食,新闻趣事
穿衣美容
名人明星,星座运势,新闻趣事,健康养生,历史讲坛

时尚
旅游
萌宠
旅游,穿衣美容,视频音乐,搞笑幽默,美食
旅游
娱乐,时尚,健康,旅游,美食
汽车,美食,军事,娱乐,体育
游戏动漫,时尚,娱乐,美食
旅游,游戏动漫,美女,体育,美食
旅游
美女,IT 数码,娱乐,健康,汽车,旅游,文艺
娱乐
VERSA,维尔萨,液压拉床,阀门控制系统,井口控制盘,天然气压缩机,电磁阀,气动阀门,炼油设备,气动元件
文艺,包子,林宥嘉,学生一族,涂鸦
名人明星,美食
搞笑幽默,美女,体育,时尚
美食,新闻资讯,旅游
军事,80 后,科技
汽车,美女,军事,娱乐
深圳生活,文艺,投资理财,新闻资讯
美女,游戏动漫
军事,IT 数码,体育
教育就业,军事,体育,文艺,新闻资讯
美女,健康,汽车,旅游,体育
新闻资讯,时尚,健康
美食,娱乐,旅游,时尚,星座命理
A 股
旅游,新闻资讯
新闻资讯,IT 数码,汽车,体育,肥胖
视频音乐
美女
旅游
时尚,星座命理,美食,娱乐,旅游
娱乐,旅游,美食
健康,旅游,汽车,体育,美食
娱乐
健康,星座命理,美食
旅游
体育
新闻资讯,投资理财,旅游,IT 数码,文艺
宠物图集,名人明星
美女,游戏动漫,旅游,美食,体育
新闻资讯,旅游,美食,文艺,娱乐

旅游,星座命理,军事,美食,娱乐
军事
旅游
旅游,美食
旅游,星座命理,教育就业,文艺
星座命理,美食,娱乐
美图摄影,八卦杂谈,搞笑幽默,美容服饰,视频音乐
搞笑幽默
微博奇葩,穿衣美容,搞笑幽默,上海生活,旅游
投资理财,美女,新闻资讯,军事,体育
多愁善感
文艺,美食,健康
云南生活
搞笑幽默,游戏
名人明星
汽车,旅游,美食,军事,体育
星座命理,新闻资讯,教育就业,健康,投资理财,旅游,IT 数码,军事
教育就业,旅游,时尚,文艺,娱乐
游戏动漫,军事,旅游,汽车,娱乐
新闻趣事,搞笑幽默,体育资讯,旅游,南京生活
体育
搞笑幽默
星座命理
教育就业,健康,投资理财,娱乐,美食
新闻资讯,文艺
教育就业,汽车,军事,体育
汽车
小说,体育,交友
游戏动漫
娱乐
新闻趣事
旅游
搞笑幽默
星座命理,文艺,旅游
美食,教育就业,文艺,投资理财,旅游
IT 数码
星座命理,加拿大生活
投资理财,教育就业,时尚
旅游,IT 数码,健康,美食
汽车,IT 数码,人不可有傲气,但不可无傲骨,音乐
娱乐,游戏动漫,文艺,午饭,人生,沉默
美食,90 没有后,双鱼座,睡觉,宅男,叔控
时尚,星座命理,美食,健康,旅游
教育就业,体育

汽车, 文艺, IT 数码, 旅游
搞笑幽默
萌宠, 微博奇葩, 搞笑幽默, 新闻趣事, 巴音郭楞生活
投资理财, 军事, 汽车, 体育
美食
汽车
健康
体育, 娱乐
文艺, 美食, 美食
娱乐
成都生活
饮食
旅游, 美女, 汽车, 军事, 体育
文艺, 教育就业, 美女, 军事
体育资讯, 旅游, IT 数码, 美食, 新闻趣事
军事, 美女, 健康
健康, 旅游, 文艺
体育
军事
微博奇葩, 旅游, 江苏生活, 新闻趣事
汽车, 旅游, IT 数码, 军事, 体育
美食控, 电子商务, 运动, 宅女, 爱吃, 家居, 双重性格, 美食, 创业, 旅游爱好者
美食
美食, 旅游
投资理财
娱乐
游戏动漫, IT 数码, 足球控, 耳机低烧患者, 医学生一只, 相声控, 手机达人, 电玩达人, 火影忍者骨灰级, 单身
IT 数码
幽默
旅游, 文艺, 娱乐, 健康
旅游, 美食, IT 数码
時尚, 健身, 教育就业, 健康, 时尚, 娱乐, 投资理财
IT 数码
搞笑幽默
健康, 文艺, 时尚
娱乐
汽车, 体育
投资理财, 健康
新闻趣事, 搞笑幽默, 星座运势, 美食, IT 数码, 文艺, 娱乐, 星座命理
美女
搞笑幽默
旅游, 美食, 游戏动漫, 汽车
文艺

旅游,健康,时尚,美食,新闻资讯
体育,动漫,囧,篮球,善良,好性格,投资理财,白羊座,NBA,80 后
双子座,小女人,向日葵,宠物,星座命理
菏泽生活,语过添情,新闻资讯
星座命理,旅游,投资理财,汽车,时尚
汽车,美食
热情,快乐大本营,音乐达人,网络,听歌,学生一族,完美主义,舞蹈,吃喝玩乐,旅游爱好者
时尚
旅游,星座命理,美食,娱乐
美食,娱乐,旅游,美女,军事,IT 数码
游戏动漫,川崎玫瑰,精分,篭城之将,般若,尊王倒幕,军国主义
旅游,美食
旅游
时尚,健康,美食,旅游,文艺
云南生活,IT 数码
旅游
文艺,健康,旅游,星座命理,军事
IT 数码,外汇,现货黄金
游戏动漫,汽车
绘画,诗词,书法,美术,公益
美食,名人明星,穿衣美容
美食,时尚
健康,投资理财,旅游,美食,娱乐
投资理财,健康
新闻资讯
一些事一些情,双鱼座,生活,上网,睡觉,电影,美食,90 后,旅行
体育
娱乐
星座命理,文艺,体育,健康
中国好男人
教育就业
新闻资讯,IT 数码,体育
娱乐,新闻资讯,教育就业,时尚,旅游
时尚,娱乐,教育就业,健康,美食
娱乐
摄影
时尚,健康,体育,教育就业,投资理财
时尚,美食,文艺,投资理财
名人明星,娱乐
美食,临汾生活,搞笑幽默,穿衣美容,旅游
旅游,美食,穿衣美容
汽车,军事,旅游,健康,新闻资讯,雨水河水可用水
陈彦舟
美食

教育就业
文艺,投资理财,80后,学习,足球迷,o型血,书,music,努力,真诚
美女,汽车,旅游,美食,军事,文艺
文艺,美食,美女
IT数码
星座命理,文艺,体育,娱乐,IT数码,宅男,网络,手机,语录,文字
那段屌丝岁月
投资理财,文艺,体育,健康,新闻资讯,娱乐,美女,游戏动漫,时尚
新闻资讯,军事,体育
其他生活
旅游,美食,福建生活
娱乐,体育
会计,70后,新闻趣事
投资理财,旅游,军事,娱乐,文艺
新闻资讯,投资理财,军事
时尚,旅游,军事,体育,娱乐
美食,健康,旅游,军事,体育
萌宠,游戏动漫
体育
美女,娱乐,体育
游戏动漫,健康,旅游,文艺,体育
新闻趣事,旅游,北京生活
新闻资讯,健康,旅游,文艺,体育
时尚,旅游,健康,教育就业,美食
娱乐
旅游
体育
旅游
高尔夫,电影,历史,天秤座,电子商务
天蝎,跑步,三国杀,电影爱好者
旅游
教育就业,健康
旅游,诗词,美剧,浪漫,温柔,DIY,爱幻想
婚姻
旅游
名人明星,旅游,美容服饰,搞笑幽默,美食
IT数码,军事,美女,健康,游戏动漫
投资理财
游戏动漫,美食,娱乐,IT数码,体育
旅游,健康,时尚,文艺,教育就业
美食
时尚
汽车,旅游,健康,投资理财,新闻资讯
美女,新闻资讯

新闻资讯,体育,IT 数码,汽车
旅游,美食,文艺
汽车,投资理财
广东生活,名人明星,美食,旅游
新闻资讯,汽车,旅游,军事
唐山生活,穿衣美容
汽车
体育,遊戲動漫,投資理財,IT 數碼,體育,娛樂
时尚
旅游,美食,IT 数码,体育,娱乐
文艺
我就是我,音乐达人,双鱼
IT 数码
时尚,汽车,文艺
体育
旅游,文艺,随性,喜欢旅游,我就是我,旅游美食爱好者,努力,快乐,爱自由
教育就业,文艺
金牛座,网络,生活,旅行
游戏动漫,汽车,旅游,美食,体育
读书分享,美图摄影,美食,新闻趣事
美女,体育
健康
文艺
健康,星座命理,新闻资讯,投资理财,IT 数码
体育,娱乐,投资理财
体育
旅游,文艺,游泳,电影爱好者,美剧迷
生活,爱睡觉,宅,软件,家居,网购,金牛座,环保,健康,美食
新闻资讯,美女,军事,体育,教育就业
IT 数码
美食,投资理财
汽车,娱乐,文艺,新闻资讯,IT 数码
教育就业,美食
投资理财,教育就业,军事,游戏动漫,时尚,汽车,美女
奋斗,银饰,网购,美食,80 后,自由,学生,时尚,宅,旅行
美食,旅游,星座命理,搞笑幽默
音乐达人,淘宝,购物,85 后,天蝎座,喜欢听歌,IT 互联网,设计师,娱乐,游戏动漫
偶喜欢玩,旅行,搞笑,80 后,电影,音乐,宅
投资理财,健康,文艺
文艺,新闻资讯,旅游,军事,IT 数码
美食,旅游,汽车
西安生活
娱乐
旅游,健康,星座命理,美食

文艺
上海生活
星座命理
汽车,投资理财,游戏动漫,旅游,美食,厨二,死胖宅
设计,听歌
Dota,篮球,UI 设计
新闻资讯,IT 数码,娱乐
赚钱,公益,爱自由,美食,媒体,吃货,吃,微博控,死神,80 后宅女
好书,健康
政治,旅游,美食,IT 数码,文艺
美食,新闻资讯,投资理财,旅游,娱乐
娱乐
美女
体育
时尚,新闻资讯,文艺
电影,音乐
美女,时尚
教育就业,军事,新闻资讯
健康,文艺,美食,娱乐,K 歌,时尚达人,拍照
教育就业
萌宠
旅游
IT 数码
搞笑幽默,新闻资讯,教育就业,投资理财,美食,军事
瓷砖,陶瓷,卫浴五金,建材,装饰,建筑,新材料,填词,作词,音乐
娱乐,IT 数码,新闻资讯,美女,汽车
星座命理,军事
机械
品牌,教育,时尚达人,热爱生活,人生,设计,时尚,艺术
穿衣美容
教育就业,新闻资讯,美女,旅游,文艺,IT 数码
娱乐,IT 数码
新闻资讯
军事,旅游,汽车,文艺,爱旅游,好书,摩羯座,宅,80 后
游戏动漫
美女,娱乐,美食,投资理财,教育就业,健康,IT 数码
肥料,肥,营养土,花肥,有机肥
搞笑幽默,双子女
健康,星座命理,旅游,投资理财,娱乐
美食,IT 数码,体育,文艺,娱乐
何胖子很矫情,何胖子很无趣
娱乐,游戏动漫
搞笑幽默
旅游,健康,美女,美食

旅游
汽车
汽车,健康,美女,军事,娱乐
投资理财
娱乐
娱乐,文艺,美食,星座命理,旅游
娱乐
政治改革,国际贸易,宏观经济,货币金融,商业,新闻资讯
IT 数码,投资理财,时尚,旅游,健康
旅游,美食,星座命理,美女,视频音乐
浪漫,喜欢音乐,单纯,羽毛球,乒乓球,天蝎,安静,快乐,旅游,美食
体育
星座命理,美食,娱乐
旅游,娱乐,投资理财,美女,健康
汽车,精神分裂
美食,美女,视频音乐,搞笑幽默
创业,动漫,睡觉,浪漫,网络,爱情,写作,互联网,美剧,交友
星座命理
旅游
投资理财,汽车,军事,IT 数码,体育
娱乐
新闻资讯,美女,体育,IT 数码
星座命理,文艺,娱乐,健康,教育就业,投资理财,新闻资讯
体育
健康
军事
美食
体育
汽车,旅游,IT 互联网,读书
游戏动漫
名人明星,微博奇葩,体育
音乐,艺术,文字,80 后,生活,好书,电影,自由,媒体,天蝎座
IT 数码
旅游
穿衣美容
旅行,文字,幽默
變態,小說迷,时尚,游戏动漫,星座命理,文艺
健康
健康,新闻资讯,旅游,文艺,体育
美食,小说,看书,电影
游戏动漫,军事,文艺
美食,旅游,健康,新闻资讯,教育就业
旅游
时尚,美女

天秤
娱乐
健康,投资理财,汽车,IT 数码,军事,新闻资讯,美女
旅游
乐观,小清新,健康,美食,娱乐
游戏动漫,美食,IT 数码,文艺,娱乐
美女,时尚,旅游,文艺,健康,美食
游戏动漫,唱歌,魔术,摄影,跳舞
游戏动漫
投资理财,旅游,IT 数码,体育,娱乐
投资理财,汽车,军事,IT 数码,体育
美食,游戏动漫,文艺,IT 数码,娱乐
健康,巨蟹座
美女
IT 数码
旅游,汽车,体育
汽车,时尚
萌宠,微博奇葩,名人明星,美女,IT 数码
美女,新闻资讯,游戏动漫,文艺,娱乐
旅游
星座命理
体育
健康,美女,星座命理,体育,娱乐
美食
汽车,军事,健康,旅游
爱睡觉,巨蟹座,电影迷,爱美食,爱电影,吃货
宠物图集,名人明星,美图摄影
时尚
游戏动漫,IT 数码,旅游,汽车
车世界,科学探索,美图摄影,读书分享
名人明星,安康生活,美女,旅游
投资理财
美女,军事,IT 数码,文艺,体育
星座命理,微博奇葩,搞笑幽默,美食,旅游
搞笑幽默,旅游,美食,萌宠
旅游,投资理财,健康
时尚,健康,教育就业,旅游,文艺
美食,健康,旅游,文艺,体育
旅游
旅游,美食,娱乐
美食,娱乐
新闻资讯,文艺,时尚,美食,娱乐
美食,时尚,投资理财,体育,财经,乐观,运动,旅游,音乐
美食,体育

电影,摩羯座,美食,游戏动漫,旅游
体育
IT 数码,文艺,体育
视频音乐
IT 数码,旅游,健康,新闻资讯,投资理财
时尚,新闻资讯,军事,投资理财,汽车
体育
游泳,日语,读书,爱旅行,书法,绘画,猫咪控,旅游
旅游
体育,英语,时尚,写作,汽车,听歌,吃货
美食
吐槽
IT 数码
娱乐
星座命理
搞笑幽默
吃喝玩乐,双鱼,时装,重口味,电影迷,咖啡,爱美食,设计,时尚,文艺
体育
旅游
娱乐,健康,时尚
体育
体育,听歌,旅游,80 后,音乐
偶稀饭睡觉觉,摄影爱好者
体育,文艺
教育就业
美食,娱乐
手工活,读书,古琴
新闻趣事
时尚
选矿,职业规划,新闻,自行车,创业,读书,好性格,自由,旅行,教育就业
美食
攝影,宅男,听音乐,IT 数码
梁静茹,游戏动漫
投资理财
旅游,听歌,80 后,音乐
投资理财
军事,游戏动漫
文艺
娱乐,汽车
文艺
善良,听歌,美女
IT 数码
游戏动漫
旅游,文艺

健康,IT 数码,美食,美女
IT 数码,教育就业,新闻资讯
新闻资讯,旅游,美女,投资理财,体育
纪实婚礼摄影,深圳婚礼摄影,人像摄影,婚礼摄影,摄影,时尚,旅行,音乐
美食,投资理财,旅游,健康,新闻资讯,时尚,摩羯
旅游,听歌,电影,音乐
娱乐
穿衣美容
军事
教育就业,新闻资讯,IT 数码,军事,汽车,体育
IT 数码,游戏动漫
投资理财,汽车,健康,旅游,体育
旅游
文艺,美女,美食,投资理财,娱乐,英文,钢琴
动漫控,星座控,日语,90 后但我不脑残,游戏动漫
娱乐,金牛座,幽默,乐观,小说,90 后,旅游,美食,旅行
教育就业
IT 数码
军事
旅游,IT 数码,汽车
美食控
新闻趣事
星座命理,时尚,旅游,新闻资讯,健康,文艺,娱乐
教育就业
健康,旅游,星座命理,美食,军事,时尚,投资理财,娱乐
娱乐,美女,健康,旅游,体育
旅游
汽车
健康,旅游,美食
古典音乐,房产,金牛座,NBA,交朋友,投资理财,健康,军事,IT 数码,体育
旅游,手机,囧,互联网,军事
娱乐,上网,善良,好书,小说,80 后,自由,宅
时尚,汽车,娱乐
投资理财,军事
北京生活
健康,美女
文艺,投资理财,教育就业,健康,星座命理
军事
IT 数码
娱乐
汽车,时尚,投资理财,美女,游戏动漫
新闻资讯,军事
新闻资讯,体育
汽车,IT 数码

文艺,美食,娱乐
时尚,健康,旅游,美食,文艺,娱乐
爱生活,85后,电影控
五月天,音乐
魔兽真三国无双,Dota,美剧
健康,新闻资讯,旅游,美食,教育就业
军事,体育
健康,投资理财,旅游,星座命理,美食
投资理财
大连生活
成都生活
健康,汽车,投资理财,旅游,军事
美女
自由,流浪,文艺
甘肃生活,我就是我,投资理财
我只爱吴庚霖,陈奕迅的弟弟,听歌,吃货,宅,天文爱好者
新闻资讯,投资理财,美食,健康
有机农业,生态农业,有机肥,鸡粪肥,发酵鸡粪,干鸡粪,鸡粪有机肥
体育资讯
娱乐
宅,吃,交友,宅女,上网,自由,旅游,自由职业,网络,女人40
体育
星座命理,新闻资讯,军事,汽车,娱乐
美女,汽车,投资理财,体育,文艺
时尚
海口生活
游戏动漫,IT数码,军事,投资理财
文艺,健康,时尚,孩子他妈
萌宠,旅游,美女,视频音乐,美食
娱乐
开朗，挑战性强
游戏,插画
军事
数码,90后,BNK,天蝎座,旅游,喜欢看电影
娱乐
旅游
投资理财,新闻资讯,娱乐,汽车
娱乐
旅游
文艺,创业,善良,乐观,射手座,唱歌,交友,自由,音乐
旅行,美食,90后,听歌
旅游
美女,娱乐
生活,娱乐,宅女,音乐,电影,美食,时尚,旅游

旅游,名人明星
苏州生活
IT 数码,海贼王,动漫,电影,80 后的尾巴,数码,吃,音乐,大学,听歌
旅游,健康,投资理财,体育,娱乐
教育就业,健康,娱乐
美女
体育,唱 K,旅游
旅游
娱乐
时尚,游戏动漫,美女,星座命理,体育
搞笑幽默
星座命理
IT 数码,汽车
江苏生活
旅游
IT 数码,旅游,健康,体育
美食
娱乐
时尚,美食,IT 数码,娱乐
旅游,微博奇葩,视频音乐,新闻趣事,重庆生活
文艺
星座命理,默默围观别人,寂寞人,天蝎座的
艺术,天蝎座,爱自由,80 後,睡懒觉,旅游美食爱好者,户外
体育,健康,时尚,汽车,旅游
IT 数码
军事,IT 数码,汽车,游戏动漫,美女
军事,晃悠,旅行,美食
文艺
书籍,睡懒觉,IT 数码,80 后
新闻资讯,旅游,美食,文艺,娱乐
旅游,健康,投资理财
美食,汽车,旅游
文艺
射手,发呆,shopping,听音乐,爱笑,钢琴,睡觉,旅行,美食
投资理财,健康,美食,军事
时尚,军事,文艺
游戏动漫,教育就业,军事
美女,旅游,美食,娱乐,投资理财
四川生活
汽车,IT 数码,体育,军事,投资理财
美食,投资理财,游戏动漫,美女,军事
美食
投资理财
旅游,教育就业,文艺,IT 数码,新闻资讯

娱乐
旅游,视频音乐,江苏生活,搞笑幽默
文艺,旅游,美食,娱乐,IT 数码
健康,新闻资讯,汽车
美食,美图摄影,读书分享,视频音乐,天津生活
旅游,美食,穿衣美容,视频音乐,浙江生活
新闻资讯
汽车,旅游,美食,体育
搞怪,女人,新闻趣事,搞笑幽默,微博奇葩
文艺
重口味,小女人,宅,北京,淘宝,购物,臭美
旅游,星座命理,北京生活,投资理财,汽车,军事
设计,读书
爱情,阅读,创业,美图摄影,旅游,搞笑幽默,其他生活
八卦,平常心,幽默,处女座,一些事一些情,宅,80 后,音乐,旅游,育儿
娱乐,投资理财,IT 数码,旅游,教育就业,文艺,时尚
重庆生活
旅游
摄影爱好者,文艺,娱乐,疗伤系,治愈系,励志,语录,心理,情感
美女
美图摄影,娱乐
时尚,美食,旅游,K 歌,90 后,安静
时尚,投资理财,汽车,健康,旅游
郑州生活,河南生活,股票
旅游,美食,宅,80 后,听歌,音乐
投资理财,旅游,体育,文艺,娱乐
娱乐
疯子,跑步,90 后非脑残,潮流,狗狗,音樂,真诚,陈奕迅,无厘头
数码,电影,娱乐,设计,时尚,英语,美剧,偶喜欢玩,唱歌,IT
个性,大学,亲情,数码,自由职业,校园,IT,财经,电子商务,好书
娱乐
娱乐,IT 数码
php,java,hadoop,软件开放,架构师,WEB,IT,程序员
游戏动漫,旅游
旅游
旅游,星座命理,美食,文艺,娱乐
新闻趣事,视频音乐,美女,校园生活
辽宁生活
投资理财,体育,时尚潮流,休闲,可爱,开朗
曼联,宅,LinkinPark,比基尼控,90 后,音乐
体育,军事
健康,时尚,投资理财,星座命理,旅游
旅游
旅游,萌宠,新闻趣事,搞笑幽默,美女

旅游,美食
教育,双子女,80後
巨蟹座,音乐,宅,下象棋,读书
投资理财
投资理财,体育,游戏动漫
80後,自贡生活,善良,射手座,K歌,欧美音乐,健康
娱乐
旅游
旅游
体育
投资理财
微博奇葩,穿衣美容,新闻趣事,其他生活,搞笑幽默
美食,穿衣美容,美女,视频音乐,旅游
时尚,汽车,美食,IT数码,体育,搞笑,玩儿
IT数码
微博奇葩
旅游
美食
美食
健康
美食,蝴蝶结控,90后
体育
汽车,旅游,美食,军事,IT数码
时尚,体育
旅游,美食,军事,IT数码,文艺
武汉生活,IT数码
美女,学习,心理学,交友,吃货
时尚,旅游,星座命理,美食,文艺
IT数码,视频音乐,美食,美女
汽车,美食
娱乐
旅游
时尚,体育,美食,文艺
投资理财,游戏动漫,汽车,IT数码
娱乐,健康,旅游,美食,教育就业
美女,游戏动漫,美食,IT数码,体育
美女,体育,娱乐
视频音乐,娱乐,游戏动漫
自由,吃,睡觉
美食,健康,投资理财,汽车,IT数码
新闻资讯,美女,军事,IT数码,文艺
旅游,军事
英伦入门,斋佬,信命,悲观主义,热爱过旅行,帆布鞋,独立,Punk,Britpop,Oasis
台球,斯诺克,运动型,羽毛球,射手男,汕尾男孩,黑黑的,阳光葱头

旅游,北京生活,投资理财
游戏动漫,投资理财,汽车,美食,旅游
投资理财,体育
美女,游戏动漫,美食
汽车,IT 数码
搞笑幽默,新闻资讯
美食,游戏动漫,汽车,IT 数码
旅游,投资理财,教育就业,新闻资讯,军事
体育,IT 数码,汽车,新闻资讯,军事
娱乐
旅游,美女,北京生活
投资理财
娱乐
旅游,体育,文艺,美食,军事
文艺,时尚,新闻资讯,旅游,军事
名人明星
娱乐
幽默,水瓶座,宅,80 后,美食,听歌
娱乐
哈尔滨生活
旅游
萌宠,美食,旅游,星座命理,美女
八卦杂谈,校园生活
IT 数码
旅游
偏执狂,宅女,美食爱好者,吃货一枚,神经大条,爱睡觉,星座命理
美食,旅游,时尚,星座命理,文艺
游戏,交友,天蝎座,上网,旅游
健康,教育就业,投资理财,IT 数码,美食
文艺
名人明星
文艺
美女,穿衣美容,旅游,微博奇葩,北京生活
美食,军事,IT 数码
体育,健康
律师,技术宅,法学
体育
新闻学院,新媒体,传播学系,媒介,媒体,传播学,传播
IT 数码
娱乐
旅游
旅游
健康,新闻资讯,投资理财,旅游,IT 数码
名人明星,微博奇葩,河南生活,搞笑幽默,美女

IT 数码
文艺,新闻资讯,旅游,军事,时尚
新闻资讯,游戏动漫,美女,军事,IT 数码
美食
娱乐
时尚,健康,星座命理,旅游,我就是我,旅行,80 后
美食,游戏动漫,新闻资讯,时尚,文艺,娱乐,星座命理
水瓶座,看书,睡觉,80 后,宅,时尚,旅游,美食
投资理财
新闻趣事,旅行,80 后,听歌,美食,体育,文艺
旅行,传媒,吃货,摄影
体育,教育就业,旅游,健康,IT 数码
IT 数码
天秤座,美食,健康
IT 数码
木星摩羯,囧,天秤座 O 型女,包衣鞋 BRA 控,狮子座,爱冬天
IT 数码
美食,一些事一些情,文字,平常心
super 慢热,DoctorWho,Marvel,思想上的抖 M,王 x 波书友会,中村老师❤,梦露我爱你,中村佑介脑残粉,冷门
教育就业,健康,体育,IT 数码,篮球,健身,唱歌,终身学习
教育就业,汽车,旅游,娱乐,体育
新闻,乐观,体育,吃货,环保,生活感悟,网络小说,IT,搞笑
旅游,星座命理,IT 数码,教育就业,新闻资讯
北京生活
四川生活
上海生活,体育
快乐,知足,奋斗,运动,电影,书法,电脑,素食,网络,素食者
IT 数码
汽车,旅游,时尚,文艺,娱乐
名人明星,广东生活
美女,汽车,投资理财,军事
投资理财,军事,体育,游戏动漫,旅游
军事
民主宪政,投资理财,教育就业,体育,娱乐
IT 数码
视频音乐
旅游
健康
文艺
旅游
新闻资讯,教育就业,投资理财,旅游,美食
IT 数码
旅游

汽车,体育
美女,文艺,汽车
80 後,新闻资讯,投资理财,汽车,军事
北京生活
健康,体育,投资理财,星座命理,美食
汽车,娱乐,旅游,体育
健康,教育就业,时尚,美食
独立制片,摄像,文艺,视听语言,导演,剪辑师,电影,音乐
帕西诺,SSAT,sat,留学,Milan,macbook,马自达 6,老师,雅思,托福
北京生活,视频音乐,体育资讯,医学,双鱼,地产,推理,时尚,音乐
新闻资讯,汽车,投资理财,健康,军事,IT 数码,文艺,体育,娱乐
文艺,投资理财,美女,旅游,IT 数码
健康,美食,旅游,汽车,IT 数码
搞笑幽默
教育就业,投资理财,新闻资讯,健康,体育
体育,财经,办公室,人力资源,狂人,炒股,时政
时尚,娱乐,旅游,汽车
投资理财
美女,IT 数码
健康,美食
IT 数码,汽车
其他生活
旅游,文艺,体育
旅游
军事,旅游
健康,旅游,美食,网购,吃货,平常心,80 后,生活,摄影
IT 数码
美女
健康
娱乐,健康,IT 数码,教育就业,美食,爱孩子,热爱生活,教育工作者
旅游,美食,IT 数码,娱乐
新闻资讯,旅游,军事,IT 数码
IT 数码
投资理财
旅游
美食
美食,IT 数码,体育,娱乐
天津生活
新闻资讯,教育就业
美女,体育,旅游
娱乐
汽车,新闻资讯,IT 数码,文艺,军事,美食
游戏动漫
新闻资讯

美女
娱乐
旅游,投资理财
投资理财,旅游,IT 数码
娱乐
美女,美食
军事
汽车,美女,文艺
搞笑,宅,金牛座,吃,娱乐,旅行,听歌,读书,处女座,电影
时尚,美食,教育就业,投资理财,娱乐
旅游,文艺,娱乐
健康,汽车,游戏动漫,军事,娱乐
旅游,文艺,美食,投资理财,娱乐
旅游,美女,汽车,美食,军事
美食
旅游,娱乐,健康,美食
教育就业,美女,健康,星座命理,IT 数码
娱乐,文艺,旅游,健康,美食,星座命理
健康,投资理财,旅游,星座命理
美女
旅游
旅游
射箭,宅,旅游,摄影,奶爸
健康,新闻资讯,时尚,投资理财,旅游
军事,汽车,时尚,IT 数码
旅游
文艺
健康,美食
健康
美食
星座命理,体育
美女
情感,西安生活,服饰,时尚,娱乐
视频音乐
健康,文艺,星座命理,旅游
娱乐,美食,旅游
娱乐,汽车,IT 数码
美食
旅游,投资理财,美食,文艺,体育
投资理财
汽车,体育,美女,文艺
旅游
新闻资讯
游戏动漫

健康
美食,体育,汽车,IT 数码
军事,IT 数码,体育
IT 数码,游戏动漫,旅游
美食,旅游,IT 数码,娱乐,体育
IT 数码
旅游
休闲游
体育
投资理财,汽车,旅游
娱乐
健康,美食
美女
福州生活,福建生活
投资理财,教育就业,娱乐,体育,文艺
美女,旅游,体育,投资理财
视频音乐
美女
穿衣美容
美女,新闻趣事,搞笑幽默,旅游,其他生活
驴友,张阳阳的串串,李宇春的玉米,旅游,军事,美食,文艺,娱乐
健康
文艺,投资理财,健康,新闻资讯,教育就业
偶稀饭睡觉觉,吃货,孩子气,射手座,咖啡
旅游
投资理财,快,戒贪,心平
IT 数码
淘宝控,美女
汽车
经济学,音乐,武道,历史,美食,面条控,面痴,网络营销,股票,投资
旅游
投资理财
健康
美女,旅游,军事,娱乐
健康,美女
美女
游戏动漫,教育就业,军事,IT 数码,体育
美女
汽车
上海生活,搞笑幽默,美食
美食,军事,IT 数码,时尚,游戏动漫
投资理财
个人成长,时间管理
文艺

旅游
体育
新闻资讯,教育就业,美食,旅游,时尚,健康,投资理财
搞笑幽默
健康,旅游,美食,IT 数码,军事
搞笑幽默,旅游,美食,娱乐,IT 数码
新闻资讯
IT 数码
时事热点,星座命理,汽车,美女,体育,军事
美食
江苏生活,工作,睡懒觉
写作,看书,拼字码字
游戏动漫
IT 数码,文艺
投资理财,军事,IT 数码,体育
汽车,旅游,体育
教育就业,体育
旅游
体育
IT 数码
IT 数码,投资理财,教育就业,娱乐,星座命理,汽车,游戏动漫,健康
浙江生活,新闻趣事,穿衣美容,健康,新闻资讯,汽车,执着,淡定,营销
军事
美食,美女,汽车,体育
搞笑幽默,星座命理,旅游,美女,IT 数码
娱乐
旅游,文艺,健康
教育就业
娱乐,时尚,健康,旅游
娱乐,游戏动漫
美女,新闻资讯,旅游,军事,体育
体育
IT 数码
美食,射手女,安静,B 型血,看小说,射手,宅,80 后,美食
投资理财,文艺,健康,军事
新闻资讯,教育就业,体育
教育就业,旅游,美食
将最复杂变简单
汽车
汽车,体育
文艺
体育
玛雅人,金港城,出租,两房,电信,宽带,全新,家私,电器,家电
军事

汽车,体育,IT 数码
时尚,游戏动漫
旅游
嘿嘿嘿和嘿嘿嘿
互联网
名人明星
时尚,新闻资讯,娱乐,星座命理,健康
美食
新闻资讯,旅游,文艺,体育
美食,健康
文艺,游戏动漫,旅游,美女,体育
青岛生活
军事
旅游,星座命理,美食,吃货,摄影爱好者,懒人,金牛座,淘宝控
健康,娱乐
健康,巧克力,户外
汽车,健康,旅游
投资理财,健康,美女,囧
军事,气动噪声,看书,美食
军事
看书,听歌,双子座,文字,电影,时尚,生活,音乐
游戏动漫,美食,IT 数码,旅游,娱乐
健康,旅游,美食
时尚
健康,旅游
搞笑幽默
美女
新闻资讯,汽车,健康,旅游,体育
汽车,军事,IT 数码,娱乐
IT 数码
美女
旅游,娱乐,文艺,美食,投资理财,偶尔安静,偶尔疯狂
大连生活
旅游
旅游,无聊就聊
生活,购物狂,时尚达人,内向
旅游
投资理财
说明书,彩盒等纸类印刷
美食,IT 数码,搞笑幽默,或许冒牌的型男,摄影,80 后
娱乐
健康,策划,IT 互联网,80 後,翻译,上网,摩羯女,健身,喜欢听歌
旅游
美女,汽车,体育

健康,旅游,汽车
体育
教育就业,旅游,新闻资讯,IT 数码,军事
一次远足
投资理财,新闻资讯
音樂,时尚,娱乐,美食,文艺
IT 数码,美女
美女,娱乐
时尚,娱乐,美食,健康,文艺
旅游,时尚,星座命理,美食,娱乐
IT 数码,体育
美食
美丽,整形医师
处女座
汽车,投资理财,IT 数码
美女
健康
时尚
思想,文化,阅读,文艺
投资理财,教育就业,健康,旅游
体育
新闻趣事,星座运势,旅游
汽车,投资理财,旅游,体育,健康
旅游,文艺,时尚,汽车,IT 数码
上海生活,交友
星座命理
健康,军事,旅游,教育就业,体育
健康,新闻资讯
美食,IT 数码
汽车,美女,美食,旅游,体育
IT 数码
汽车,美女,军事,娱乐
汽车,旅游,文艺,IT 数码,体育
军事,IT 数码
体育,IT 数码,美食
看书
体育
美食,健康
IT 数码
汽车,篮球~
美食,军事,投资理财,IT 数码,摩羯座
新闻资讯
文艺,时尚
投资理财,体育

新闻资讯
教育就业
视频音乐,郭德纲,台球
搞笑幽默,IT 数码,军事
教育就业,时尚,汽车,美食,体育
健康
IT 数码,投资理财,旅游,星座命理
湖南生活
军事
IT 数码,美女
新闻资讯,IT 数码,汽车,旅游
健康,体育
投资理财
就爱小兜兜,天秤座,吃,篮球,自由,年轻人
IT 数码
美食,旅游
旅游,军事,文艺,健康,投资理财,IT 数码
IT 数码,投资理财,美女,游戏动漫,星座命理
睡,玩,吃,宅
美食
军事
旅游
美食,娱乐,体育,IT 数码,汽车
新闻趣事
教育就业,健康,美食
时尚,美食,旅游,教育就业,健康,美食
娱乐
旅游,新闻资讯,美食,IT 数码,投资理财
电影,足球,汽车,听歌,旅游,新闻,娱乐,唱歌
投资理财
美食
文艺,美食,旅游,投资理财,游戏动漫
时尚,汽车,IT 数码,体育
投资理财,美食
电影爱好者,资讯,开朗,军事
70 后,深圳,热爱生活,旅游,投资理财,IT 数码,娱乐,体育
宅,旅游,电影,听歌,80 后
美女
投资理财,体育,军事,美女,自由,电影,80 后
美食
梅州生活
时尚,美食,体育
娱乐
IT 数码

娱乐
星座命理,时尚,健康,旅游,美食,娱乐
新闻资讯,宅
健康,旅游,美食,投资理财,娱乐
IT 数码,时尚
投资理财,健康,军事,体育,文艺,IT 数码
文艺
时尚,投资理财,健康,美食,旅游
美食
A 型血,爱去美容院,游泳,减肥,杭州,家有小孩,旅游,美食,80 后
IT 数码
搞笑幽默,萌宠,旅游,星座命理,美食
文艺,美女,IT 数码
美食,美女
搞笑幽默
汽车
旅游
文艺
美食
美女
单身,阳光,旅游,健康,星座命理,美女,时尚,娱乐,美食
搞笑幽默
星座命理,美食,军事,娱乐,新闻资讯
搞笑幽默
IT 数码,健康,旅游,美食,汽车
旅游
文学,经济,旅游,摄影,电力自动化,淘宝
IT 数码
旅游,动漫迷
星座命理
音乐,电影
美女
善良,感性,自信,自尊
旅游,美食,健康
北京生活,搞笑幽默
美容服饰,南京生活
北京生活,健康,体育
体育,文艺,投资理财,旅游,时尚
时尚,投资理财,文艺
新闻资讯,美食,旅游,IT 数码,体育
职场招聘,体育资讯,微博奇葩,搞笑幽默,视频音乐
书籍
历史,闷骚,沉默,情绪化,新闻资讯,互联网,深圳,独立,自由,软件
健康,新闻资讯,星座命理,美食,IT 数码

教育就业
星座命理,旅游,娱乐
旅游,娱乐,体育
娱乐
文艺,美食
体育
时尚,IT 数码
美女
新闻趣事,搞笑幽默
健康
唐山生活
娱乐
IT 数码,军事
文艺,健康,旅游
健康,旅游,星座命理,时尚,文艺
山东生活,搞笑幽默,新闻趣事,旅游,美女
汽车,旅游,美女,军事,健康
健康,旅游,娱乐,游戏动漫,美食
美女
旅游,美食,爱狗狗,爱吃的小馋妞,80 后
娱乐,健康,IT 数码,旅游,新闻资讯
江苏生活
新闻资讯,健康
良友,导师,精神修炼,文化
健康
旅游,娱乐,新闻资讯,军事
电影,天秤座,80 后
教育就业
新闻趣事
教育就业,星座命理,美食
IT 数码,娱乐,旅游,星座命理
汽车
汽车,旅游,美食,体育
健康,游戏动漫,美食,旅游,IT 数码
美女
汽车
娱乐
健康,美食,旅游,投资理财
微博奇葩,新闻趣事,搞笑幽默,辽宁生活,视频音乐
时尚,汽车,美女
旅游,娱乐,美食
旅游美食,IT 数码
我就是我
北京生活,足球,80 后,音乐,天蝎座

旅游,美女,汽车,娱乐
美食
娱乐
美女,军事,投资理财,汽车,体育
军事
娱乐
健康,教育就业,旅游,美食,娱乐
美食,健康,旅游,体育
生活,阅读,营销,唱歌,足球,NBA,歷史,旅遊
旅游
IT 数码,汽车
IT 数码,美图摄影,东营生活
新闻资讯
娱乐
旅游,美食,教育就业,投资理财,文艺
新闻资讯
汽车
旅游,军事,IT 数码,文艺,体育
体育
美食,关注孩子成长
IT 数码
娱乐
健康,汽车,IT 数码,文艺,娱乐
美食
投资理财
汽车
美食
旅游
美食,星座命理,旅游,IT 数码
教育就业
汽车,美食,旅游
新闻资讯
体育
IT 数码,体育
体育,文艺
旅游
旅游
b 型血
旅游,家有宝贝
新闻资讯,时尚,健康,旅游,美食
旅游,娱乐,军事,汽车,美女
旅游
投资理财
健康

体育
阳光,汽車,上海,80 後,怀旧,工作狂,旅游,白骨精,80 后
旅游
美女,北京生活,搞笑幽默
旅游,娱乐,新闻资讯,时尚,文艺
IT 数码
健康,旅游,美食,星座命理,娱乐,唱歌,生活,八卦,天蝎座
浙江生活
体育,美食,IT 数码,军事,文艺
美女,游戏动漫,体育
星座命理,爱旅游,看书,自由,文艺,摩羯,90 後
时尚,新闻资讯,娱乐,体育,旅游
投资理财
娱乐,平安,瓶盖
IT 数码
美女,文学
美食
教育就业,文艺,体育,投资理财,美食,娱乐
旅游
旅游
新闻资讯,旅游,美女,汽车,军事
旅游
星座命理,搞笑幽默,新闻趣事
健康
健康,星座命理,旅游,IT 数码,文艺
钢琴,中医,搞笑,天秤座,科幻,奋斗,健康
北京生活
微博奇葩
文艺
旅游
汽车,旅游摄影
视频音乐,宠物
娱乐,投资理财,健康,时尚,旅游,文艺
名人明星,沉默
IT 数码,体育,美女,军事,时尚,投资理财,美食,自由,宅,音乐
IT 数码
旅游,美食,娱乐,美女,游戏动漫
汽车,旅游,健康,军事,新闻资讯,娱乐
娱乐
军事
读书分享,旅游,美图摄影,八卦杂谈,美女
新闻资讯,投资理财,教育就业,健康,军事
搞笑幽默,娱乐
军事,IT 数码,时尚,娱乐

纠结的小双子,统计学,自由,电影,音乐,美食,学生,宅,90 后
美食
IT 数码
旅游,水瓶座
体育,游戏动漫,IT 数码,80 后
体育,健康,旅游,美食
教育就业,时尚,美食,健康,军事,汽车,娱乐,投资理财,体育
电子商务,代理加盟点,购物充值店,社交礼仪,职业规划,心理咨询,90,网络营销,演讲口才,教育就业
疼老婆的老男人
旅游,射手座,囧,数码,听歌,自由职业,偶喜欢玩,IT,设计,it
星座命理,文艺
激情,责任,使命,跑步,热爱生活,环保
投资理财,健康,美食
爱笑,爱旅游
时尚
投资理财
爱睡觉,90 後,旅遊,浙江生活,吃,搞笑,微博奇葩,新闻趣事,福建生活,文艺
it 互联网,娱乐,游戏动漫,新闻资讯,IT 数码,体育
星座命理,教育就业,文艺,娱乐,体育,奋斗,高三
美女,游戏动漫,旅游,军事,文艺
娱乐
帆布鞋,休闲鞋,校园小清新,英伦风,大牌香水迷,处女座,85 后
郭敬明,小时代,柯南,听歌,90 后,美食,宅,旅行,旅游,学生
军事,旅游
搞笑,唱歌,看书,射手座
投资理财,新闻资讯,美女,旅游,汽车
健康,时尚,美女,美食,体育
动漫,WOW,80 后宅女,夜猫子,爱玩,咖啡,爱美食,睡懒觉
美女,文艺,娱乐,时尚
娱乐
美女,投资理财,文艺,IT 数码,星座命理,美食
投资理财,美女,体育
长春生活,汽车,新闻资讯,投资理财
健康,新闻资讯,文艺,旅游,美食
宅男,摄影爱好者
旅游,美食,新闻趣事,广西生活
旅游
IT 数码
娱乐
美食,旅游
汽车,IT 数码,军事,投资理财,游戏动漫,体育,健康,旅游
旅游,美食,星座命理,文艺,娱乐
美女

新闻资讯,旅游,星座命理,美食
聚焦三农,有机粮食,有机大米,有机苹果,有机水果,有机蔬菜,有机生活,有机食品
美食,旅游,美容服饰
娱乐
新闻资讯,旅游,美食,体育,娱乐
唐山生活
投资理财,体育
娱乐,宅女一枚,律师
旅游,穿衣美容,视频音乐,星座命理,搞笑幽默
新闻趣事,北京生活,体育
星座命理
游戏动漫,时尚,军事
水瓶座加 A 型血,80 后,靠谱青年,唱歌,游戏,旅行
健康,旅游,军事,体育
时尚,娱乐,健康,星座命理,IT 数码,旅游,美食,体育
美女,体育
驴友
投资理财,新闻资讯
美女,萌宠,微博奇葩
星座命理,健康,新闻资讯,文艺,旅游
娱乐
烟台生活,80 后
汽车,投资理财,旅游,美食,娱乐
体育,娱乐,旅游,新闻资讯,健康
美女,游戏动漫,IT 数码
体育,娱乐,文艺,美食,旅游
旅游,军事,IT 数码,娱乐,星座命理
旅游
汽车,体育
新闻资讯
娱乐
文艺,美食,投资理财,健康,旅游
投资理财,IT 数码,体育,健康,时尚
美食
投资理财,军事,旅游,IT 数码
新闻资讯,IT 数码
时尚,健康,星座命理,美食,娱乐
旅游
美食,北京生活,视频音乐,新闻趣事,搞笑幽默
浙江生活
娱乐
游戏动漫,体育
旅游,IT 数码,投资理财,教育就业,新闻资讯
健康

星座命理,旅游,文艺,体育,娱乐
新闻资讯,教育就业,投资理财,汽车,健康,吃货,上网,听歌
IT 数码,军事,互联网,幽默,电影,好书,听歌,体育,大学生,美食
文艺
星座命理
美女
旅游,IT 数码,游戏动漫
体育,娱乐
汽车,体育
教育就业,时尚,旅游,IT 数码,娱乐
娱乐
娱乐
美食,星座命理,美女,江西生活,旅游
娱乐
星座命理
新疆生活,IT 数码,投资理财,娱乐
美女,漫画,健身,85 后,psp,喜欢音乐
军事,美食,健康,旅游,汽车,投资理财
视频音乐
健康,投资理财,游戏动漫,美女
时尚,健康,旅游,文艺,娱乐
广东生活
同性戀,美食
汽车,IT 数码,健康,时尚,投资理财
汽车,军事,IT 数码
娱乐
健康
娱乐
投资理财,文艺,IT 数码,教育就业
娱乐,文艺,IT 数码,饮食,旅行,双子座,好性格,宅男,听歌
娱乐,开朗,唱歌,上网,睡觉,学生,宅,k 歌,男人,90
新闻趣事
旅游,体育
旅游
新闻资讯,投资理财,汽车,旅游,体育
无所谓的乐天派,宅女
美食,旅游,IT 数码,体育,娱乐
旅游,美食,汽车,军事,体育
新闻趣事,旅游,浙江生活,穿衣美容
娱乐,时尚
文艺,军事,汽车,美女,新闻资讯
旅游
时尚,旅游,美食,文艺,娱乐
自由,平常心,乐观,睡觉,吃货,水瓶座,摄影,美食,电影

体育
体育
星座命理
健康,美食,时尚,旅游,娱乐
苏州生活
健康
美女,体育
时尚,旅游,IT 数码
娱乐,美食,美女,游戏动漫
音乐,偶喜欢玩,天秤座,听歌
旅游
娱乐,时尚,旅游,星座命理,美食
星座命理,美食,时尚,健康
娱乐
旅游
湖北生活,旅游,名人明星,美女,新闻趣事
星座命理
文艺,体育,旅游
游戏动漫
旅游,文艺,健康,投资理财,新闻资讯
美食
股票,营销,柯南,80 后宅女,歌手,恐怖片,茶
汽车
白羊女,投资理财
搞笑幽默
投资理财
旅游
IT 数码,美食,双子座
健康
摄影,美女,美食,IT 数码,球类运动达人,听歌,IT 民工
娱乐
军事,健康,娱乐,新闻资讯,投资理财
星座命理
IT 数码
Cler,营销策划,日蝎月蟹,豆瓣 er,网络,澄海 3C,宅,80 后,听歌
汽车,体育
自由人
娱乐
美食,游戏动漫,娱乐
nba,体育,浪漫,摄影爱好者,胡思乱想,自信,视频音乐,旅游,美女,广东生活
旅游,教育就业
星座命理
美女
健康,文艺,时尚,娱乐

美食,艺术,紫色,羽毛球
教育就业,投资理财,健康,旅游,时尚
健康,天蝎座
旅游,美食,月光族,镐控,韩剧控,时尚
汽车,IT 数码
网购,80 后宅女,睡懒觉,全职妈妈,护肤,育儿,时尚,健康,美食
时尚,旅游
美食,星座命理,时尚
娱乐
游戏动漫,时尚,IT 数码,体育,美女
股市,思考,管理,学习,汽车,投资理财
IT 数码,自由,听歌,旅游,80 后,设计师,音乐爱好者,户外,运动,交友
美食,穿衣美容,星座命理,搞笑幽默
健康,旅游,文艺,娱乐,体育
星座命理
军事
旅游,微博奇葩,新闻趣事,搞笑幽默,河南生活
IT 数码
中文系学生,军事,旅游
健康,新闻资讯,军事,美食,娱乐
游戏动漫,美女,IT 数码,体育
健康,美食,文艺,娱乐,投资理财
教育就业,汽车,IT 数码,文艺,体育
体育,美女
军事,新闻资讯
健康,新闻资讯,时尚,星座命理
动漫
旅游
IT 数码,无聊,热情,K 歌,拍照,篮球爱好者
美女,新闻趣事,旅游,我就是我,旅游美食,自由,看书,上网,健康,投资理财
微博奇葩
新闻资讯,体育,文艺,娱乐,投资理财
星座命理,旅游,视频音乐,穿衣美容,搞笑幽默
娱乐
美女,美食,军事
美女,IT 数码
体育
娱乐,IT 数码
美女,星座命理,时尚,娱乐,旅游,军事,新闻资讯,体育
游戏动漫,80 后,电子竞技,CS
搞笑幽默,三国杀,吃货,处女座,宅男,睡觉,90 后
八卦杂谈,爱自由
旅游,美女,体育
娱乐,美食,军事,游戏动漫,旅游

天枰座,狗狗,欧美控,护肤,美剧控,80 后的尾巴,书籍,爱电影,爱旅游,宠物
时尚
游戏动漫,文艺,偶喜欢玩,自由,电影,动漫,读书,时尚,双子座,90 后
美剧,宅
樱井翔,大野智,二宫和也,松本润,相叶雅纪
时尚
星座命理
投资理财,美女
旅游,四川生活
IT 数码,汽车,游戏动漫,娱乐
投资理财,教育就业,健康,旅游
江苏生活,教育就业
努力挣扎,交友,美女,营销,生活,互联网,创业,搞笑,美食,电影
时尚,健康,旅游,星座命理,美食,文艺,小说迷
搞笑幽默
A 股,股票
美食,健康
娱乐
推理,动漫,电影,音乐控
娱乐,美女,新闻资讯,军事,IT 数码,百变人生,由我掌控
90 后但我不脑残,高三,美术生一只,银他妈是种态度,海贼王,声优控恋声族,动漫迷,巨蟹座,神经质,宠物
军事
旅游
旅行,摄影,阅读,明信片,写信,交友,收藏,postcard,postcrossing
投资理财,健康,旅游,军事,美食
娱乐,体育
娱乐,美食
体育,威固,贴膜,汽车
体育,文艺
新闻资讯,时尚,教育就业,阳光宅男,85 后狮子男,吃货,生活,美女,看书,羽毛球
美女
IT 数码,星座命理,体育,美女
旅游,文艺
美女,旅游,IT 数码,汽车,投资理财,军事
教育就业,旅游,美女,美食,文艺
娱乐
旅游,星座命理,教育就业,文艺,娱乐
健康,文艺
美食,新闻资讯,汽车,旅游,星座命理
旅游
英国生活
娱乐
滨州生活

旅游,体育
IT 数码
WhatTheFuck
时尚,星座命理,娱乐
旅游,爱吃,迷糊
读书分享,重口味,动漫,校园生活,搞笑幽默
健康,美女,教育就业,汽车,旅游,IT 数码
旅游
投资理财,美食,游戏动漫,体育,IT 数码
90,哈利波特,电视剧,留学,吃喝玩乐,A 型血
摩羯座,好性格,偶稀饭睡觉觉,80 后
美女,军事,汽车,美食,IT 数码
动漫,瓶邪一生推,板车青黄紫赤,尊礼伏八静临,银土可逆不可拆
健康,时尚
美女
摄影,睡觉,搞笑,音乐,设计,玩儿,宅,美食,自由,白羊座
名人明星,娱乐,体育
美女
美女,教育就业,新闻资讯,军事,体育
爱旅行,咖啡控,文艺
军事,旅游,新闻资讯,美女,汽车,IT 数码
搞笑幽默,穿衣美容,旅游,美食,萌宠
汽车,健康,教育就业
原创,汽车,时尚,旅游,文艺,娱乐
搞笑幽默
萌宠,美食
新闻资讯,文艺,星座命理
旅游爱好者,美食,广州生活,穿衣美容
文艺
80 后,刘恺威,化妆品,购物,娱乐
旅游
投资理财,旅游,美食
技术宅
投资理财,健康
旅游
射手座,梦想
军事,IT 数码,文艺,新闻资讯,体育
IT 数码,教育就业,投资理财
健康,美食,体育,教育就业,旅游
偶喜欢玩
美食
美女,新闻趣事,上海生活,旅游,萌宠
美女
美食,新闻资讯,投资理财,健康,旅游

教育就业
星座命理,美食,旅游,穿衣美容
新闻资讯,旅游,IT 数码,美女,星座命理,文艺
体育
小说,喜欢旅游,投资理财,美食,健康
娱乐,美女,游戏动漫,军事,军事发烧友
娱乐
娱乐
美女
娱乐
文艺
娱乐,时尚,投资理财,体育,文艺,IT 数码
旅游,美食
美食
摩羯座,好书,小说
美食,穿衣美容
体育
新闻资讯,星座命理,投资理财,娱乐,旅游
娱乐
互联网,历史,科技,古典艺术,IT 数码
任性,做梦,思考,星座命理,美食,文艺,娱乐
投资理财
体育
娱乐
娱乐,体育
星座命理,美食,健康,时尚,娱乐
体育,星座命理,IT 数码,文艺,娱乐
搞笑幽默
DIY,搞笑幽默,北京生活,星座命理,旅游,化妆造型师,美剧控,白羊座 AB 型,网购,宅
旅游,健康,时尚,教育就业,娱乐
星座命理,广东生活,时尚,军事,美女,旅游,IT 数码,听歌
娱乐,投资理财,健康,时尚,汽车
旅游,美食,健康,娱乐,时尚
娱乐
美女,娱乐
时尚,IT 数码,游戏动漫,美食,旅游
健康,文艺,时尚,美食
娱乐,游戏动漫,时尚,体育
星座命理,美食,IT 数码,浪漫,80 后,营销,平常心,手机,偶稀饭睡觉觉,交友
娱乐,投资理财,美食
美女,IT 数码,体育,娱乐
旅游
美女
美食,无限的未来,endlessmirai,假发,cosplay

美食
健康,美女,游戏动漫,汽车
时尚,娱乐,文艺,星座命理,IT 数码
新闻资讯,健康,旅游
视频音乐,军事
四川生活,美食,旅游,穿衣美容
娱乐,旅游,星座命理
游戏动漫,投资理财,旅游,文艺,睡觉睡到自然醒
教育就业,投资理财,IT 数码,文艺,体育
IT 数码
视频音乐,娱乐,汽车
美容护肤,女包,女装,奢侈品,网购,购物,女性,淘宝,LV,旅游
汽车,投资理财,时尚,健康,旅游
投资理财,IT 数码
IT 数码
时尚,健康,星座命理,美食,娱乐
健康,美女,娱乐
亲情,传媒,语录,大学,IT,新闻趣事,萌宠,微博奇葩,搞笑幽默,穿衣美容
美食
娱乐
星座命理,文艺,游戏动漫
旅游,星座命理,美食,娱乐
搞笑幽默,时尚,文艺,吃货一枚
搞笑幽默,四川生活
美食,游戏,游泳,汽车,腕表,背心,运动,大海,海贼王,篮球
IT 数码,军事,投资理财,汽车,体育
时尚
健康,IT 数码,美食,娱乐,投资理财,美女
健康
上海生活,新闻趣事,穿衣美容,旅游,美食
军事,旅游,新闻资讯,健康,美女
美女,视频音乐
文艺
名人明星,旅游,美食
军事
IT 数码
投资理财,美食,星座命理,旅游,美女
体育,娱乐
投资理财
体育,教育就业,旅游,IT 数码,文艺
健康,投资理财,汽车,娱乐,IT 数码
偶稀饭睡觉觉,慢性子,学生一枚,90 后宅女,天蝎女,淡定,美食,旅游,游戏动漫,娱乐
投资理财,游戏动漫,星座命理,军事,文艺
美女

娱乐
健康,旅游,美食,汽车,时尚
时尚,美女,美食,军事,IT 数码
文艺,游戏动漫
星座命理,美食
娱乐
健康,美女,汽车,投资理财,体育,IT 数码
娱乐,IT 数码,游戏动漫,美食,体育
文艺
时尚,美女,囧,生活,善良,宅男,手机,天秤座
旅游
IT 数码
美女,游戏动漫,美食,星座命理,娱乐
投资理财,美食,星座命理,游戏动漫,汽车,美食
健康,文艺,美食,旅游
军事
穿衣美容
穿衣美容
IT 数码
汽车,投资理财,文艺,旅游,自由职业,亲情,吃货,交友
旅行,美食,天蝎座
游戏动漫
美女,汽车,IT 数码
微博奇葩,搞笑幽默
旅游,健康
新闻资讯
美女,文艺
体育
狮子座,DIY,数字油画,宅,音乐,听歌,美食
IT 数码,孙燕姿,时尚,宅,旅游,80 后,音乐,电影
娱乐
游戏动漫,IT 数码
娱乐
美食,视频音乐,广东生活
处女座,自由
美食
旅游,爱旅行,爱自由,青春,90,强迫症,文艺
美食,体育,旅游,汽车,教育就业,健康,美女,军事
IT 数码
旅游
健康,投资理财
IT 数码,电影发烧友,旅游摄影,摄影师,数码,音乐
新闻趣事,新闻资讯,教育就业
投资理财

时尚,新闻资讯,娱乐,旅游,健康
读书分享,旅游,美图摄影,视频音乐,90 后,学生,射手座,篮球,新闻,自由
投资理财,时政,电影,高尔夫,文学,音乐,旅游,营销,理财,摄影
新闻资讯,美女,游戏动漫,娱乐,IT 数码
娱乐,时尚,美女,美食,旅游,无敌金僧,我的美爸,那口才一级棒,机灵
游戏动漫,旅游,IT 数码
游戏动漫
娱乐
旅游,文艺,美食
IT 数码,星座命理,教育就业,新闻资讯,健康,时尚
教育就业
文艺,IT 数码,美女
新闻资讯,健康,体育
娱乐
娱乐
美女,90 后,狮子座,看书,唱歌,学生
旅游,时尚,投资理财,娱乐
美食
健康,投资理财
美女,娱乐,星座命理,军事,投资理财
沒瞭朋友。,身為 GAY 有瞭 BF,煩鈈想說
职场招聘,幸福,书,80 后的尾巴,爱玩,热爱生活
Mo,汽车,天蝎座,睡觉,摄影爱好者,听歌
IT 数码
体育
旅游,娱乐,摄影,交友,创业,浪漫,手机
汽车
军事
TVB,学生,媒体,狗狗,历史,金牛座,新闻资讯
随性,旅游摄影
80 後,美剧控,文艺
旅游,军事,教育就业,新闻资讯,投资理财
美食,视频音乐,体育资讯,西安生活,游戏动漫,娱乐,美女,体育,军事
教育就业,星座命理,文艺
投资理财,新闻资讯
IT 数码
汽车
教育就业,娱乐,美食,星座命理,时尚,投资理财,旅游
新闻资讯,军事,IT 数码,娱乐,体育,80 后,电影
射手座,美女,营销,娱乐,文字,自由,好书,读书,音乐,动漫
旅游
财经
星座命理,娱乐
微博奇葩,美食,搞笑幽默,浙江生活

美女,搞笑幽默
时尚,新闻资讯,健康,军事,文艺
IT 数码
时尚,旅游,美食
旅游
文艺
2015 年完成梦想,喜欢贝克汉姆,喜欢 angelababy,网购,言情小说爱好者,狮子座,创业,网络营销,服装网店,时尚
任性,固执,霸道,冷漠,顽皮,懒惰,偏激,叛逆,星座命理,旅游
郑州生活,时尚,健康,文艺
娱乐,汽车,羽毛球,乒乓球,双子
南京生活,IT 数码,健康,旅游,星座命理,美食
美女,时尚,投资理财,汽车
美食,旅游
爱睡觉,篮球爱好者,o 型血,动漫控,爱幻想,游戏动漫,旅游,美食,美女,体育
小说
语 C,动漫,K
娱乐
八卦杂谈,IT 数码,体育资讯,新闻趣事,郑州生活
新闻趣事,IT 数码,新闻资讯
幽默,财经,篮球,新闻,电子商务,电影,生活,自由,音乐,营销
广东生活,美女
健康,时尚,娱乐,旅游,美食,IT 数码,文艺
健康,旅游,美食,新闻资讯,文艺
体育,DOTA
教育就业,星座命理,文艺,健康
美食,美女,IT 数码
时尚
旅游,美女,时尚,投资理财,美食
旅游,娱乐,健康,新闻资讯,美食
LOL,旅游
文艺
文艺,娱乐,星座命理,美食,时尚,美女,汽车,旅游,军事,体育
文艺,体育
文艺,健康,游戏动漫,旅游,美食
游戏动漫
游戏动漫,军事,平常心,上网,善良,游戏
新闻趣事
军事
吃,电影
投资理财,军事,IT 数码,体育,娱乐
IT 数码,投资理财,娱乐,健康,旅游
文艺
赣州生活,财经资讯,教育,新闻趣事,健康养生

穿衣美容
娱乐
健康
新闻资讯,健康,时尚
搞笑幽默
美食
美食
文艺
情感生活,职场招聘,IT 数码,读书分享,旅游
努力做自己
军事,新闻资讯
IT 数码
旅游,星座命理,美食,娱乐,健康
新闻资讯,汽车,IT 数码
A 股,股票
娱乐
旅游,星座命理,美食,军事,IT 数码
游戏动漫,教育就业,文艺,美容美发,时尚女装,电影,营销,搞笑,英语,学生
新闻资讯,军事,美女
投资理财,新闻资讯,IT 数码,旅游
玩,安静,快乐,美食控,健身,投资理财,体育,游戏动漫,汽车,IT 数码
化妆品
美女,旅游,军事,娱乐
IT 数码,新闻资讯,魔術師,魔术,宅,摄影,射手座,阅读,幽默,小说
文艺,星座命理
旅行
娱乐,玩,时尚,艺术,上网,设计,文字,乐观,玩儿
游戏动漫,体育,娱乐
IT 数码
健康,新闻资讯,旅游,文艺
旅游,新闻资讯,IT 数码,投资理财,游戏动漫
新闻资讯
娱乐
娱乐,体育
旅游,美食,IT 数码,娱乐,时尚
打台球,看电影,上网,开朗
娱乐
教育就业
星座命理,新闻资讯,娱乐,游戏动漫,旅游,IT 数码
娱乐
爱旅行,IT 数码
美食,健康,时尚,旅游,娱乐
娱乐,文艺,健康
娱乐

文艺,IT 数码,汽车,游戏动漫
美食,旅游,健康,时尚,体育,吃货,网购
时尚,新闻资讯,旅游,文艺
美食,军事,娱乐,IT 数码,体育
游戏动漫,旅游,体育
美女,星座命理
时尚
体育
视频音乐,美食,美女,搞笑幽默,微博奇葩
传媒,美女,乐观,运动,设计,摄影爱好者,80 后,电影,创业,音乐
体育,军事,汽车
旅游,汽车,体育
新闻资讯,IT 数码,美食,旅游,投资理财
穿衣美容,夜猫子
教师,书,读书,文字控,高中生,文艺,大学生,艺术,IT,校园
美女,IT 数码,星座命理,体育,娱乐
新闻资讯,旅游,美食,文艺,娱乐,摩羯女,吃货,shopping,强迫症
旅游,新闻资讯,军事,IT 数码
娱乐
旅游
娱乐,旅游,体育,文艺,美食
娱乐
美食,娱乐,吃货,旅游
汽车
旅游,健康,新闻资讯,美女,时尚
美女
体育
美食,体育,娱乐
说爱毕竟太奢侈
电影,我爱 UC 浏览器
美食,旅游,教育就业
市场营销,心理学,日本,睡觉,医学,日剧控,IT 数码
娱乐,文艺
游戏动漫
时尚
上网,篮球,看书,学生,美食,时尚,电影,音乐
武汉生活
校园生活
美食,名人明星,视频音乐,美容服饰,旅游
情感生活,美食,搞笑幽默,郑州生活
自然,拍照,心理,旅游爱好者,律师
90 后,篮球,学生,宅,搞笑,唱歌,动漫
搞笑幽默
游戏动漫

旅游,美食,江苏生活,穿衣美容,85后
娱乐
80后的尾巴,摄影师,潮流,Music,天平座,LOL,wow,体育,时尚,美女
游戏动漫
娱乐
周杰伦,狗狗,射手座,娱乐,美食,旅游,健康,90后,音乐
健康,时尚,旅游,美食
江苏生活
旅游
新闻趣事,旅游
教育就业,IT数码,娱乐,投资理财,新闻资讯
搞笑幽默
上网,90后,旅游,手机,网购,媒体,美食,时尚,紫色,金牛座
美女,文艺,听歌,射手座,吃,羽毛球,宅男
旅游
江苏老乡,12级临床,淑女!!!,美食,旅游,文艺
O型血,善良,阅读,好性格,自由,宅女,处女座,乐观,吃货,艺术
体育
美女,游戏动漫,娱乐,投资理财,旅游,美食,文艺
微博奇葩,文艺,傻逼
时尚,小提琴,文艺,购物,会计,宅女一枚,欧美控,狗狗,情绪化
美食,军事,投资理财,新闻资讯,体育
宅文化,动漫,时尚潮流,电影,音乐,漫画,街拍,减肥,旅游,美食控
游戏动漫,IT数码
常德生活
教育就业
IT数码
旅游,游戏动漫,娱乐,文艺
健康,旅游,美食
游戏动漫,美食
IT数码,投资理财,汽车,旅游
文艺
娱乐,旅游,90后,听歌,巨蟹座
时尚,旅游
美女,播主,四班
娱乐
美女
SONE,旅游,游戏动漫,体育,娱乐
文艺,娱乐,内蒙古师范大学,内师大董伟,董伟,小益,歌手,翻译
体育,娱乐
汽车,美女,IT数码,体育
搞笑幽默,美女,IT数码,逻辑哲学,美食,历史,科学,建築,电影
体育
投资理财,现货黄金

海贼王我的最爱,海贼王,无聊,计算机,手机看小说,大学生,睡觉睡到自然醒,NBA,开朗,游泳
军事
创意,曹方,情绪化,小众音乐,说怪话
美食,投资理财,旅游,IT 数码,体育
新闻资讯,健康
旅游
名人明星
军事,IT 数码,汽车
旅游,美食,军事,文艺,体育
新闻资讯,星座命理,美食
旅游
太原生活,搞笑幽默
旅游,投资理财,健康,时尚
娱乐
娱乐
营销策划,市场分析,物流管理,企业管理
文艺,星座命理,娱乐,旅游,美食
投资理财,汽车,新闻资讯,美食,健康
旅游
上网,小说
星座命理,穿衣美容,旅游
娱乐
上海生活
旅游,汽车,体育
投资理财
美女
情感生活,北京生活,话剧,浪漫,爱旅游,爱电影,爱生活,双鱼座,美食,善良
军事天地
游戏动漫,星座命理,娱乐,文艺
娱乐
爱音乐,淘宝,跑步
时尚,旅游,星座命理,美食
军事,体育
减肥瘦身,屌丝男士,淘宝达人,健康养生,博客达人,网络营销,市场推广,淘宝客管理,市场营销,电子商务
美容,减肥,美容护肤,中医养生,中医养生培训,刮痧,按摩,火疗,经络护理
新闻趣事,穿衣美容,合肥生活,美食,时尚,健康,旅游,文艺
健康,娱乐,文艺,美食,旅游
双子座
北京生活,旅游,IT 数码,美女,校园生活
美食
游戏动漫
旅游,健康,文艺,时尚,投资理财,美食
娱乐

新闻趣事,微博奇葩,搞笑幽默,旅游,时尚,星座命理,文艺,娱乐
旅游
80后,工程师,音乐,GAME,游泳,电影
教育就业,娱乐
萌宠
搞笑幽默
投资理财
星座命理
健康,美食
体育
健康,花卉知识
新闻资讯,汽车,投资理财,军事
健康
重庆生活,娱乐
时尚,投资理财,旅游,美食,娱乐
上海生活,天蝎座,生活
汽车,旅游,新闻资讯,健康,时尚
体育
时尚,娱乐,文艺,IT数码,旅游,80后
娱乐
教育就业,IT数码,投资理财
新闻趣事,微博奇葩,搞笑幽默,DotA,体育
文艺
旅游,萌宠,微博奇葩,搞笑幽默,美食,IT数码,体育,娱乐
化工,喜欢交友,开朗,喜欢交流
微博奇葩,娱乐
娱乐
美女,体育,健康,旅游,军事
小清新,好性格,向日葵,热爱生活,娱乐,文艺,旅游
旅游
时尚,汽车,旅游
汽车,娱乐,游戏动漫,军事,IT数码
汽车
新闻资讯,汽车,旅游
文艺
游戏动漫
IT数码
微博奇葩
文艺,美女,旅游,健康,军事
教育就业,时尚,游戏动漫,美女,文艺,娱乐,军事
军事,投资理财,汽车,体育,娱乐
游戏动漫
IT数码
文艺,娱乐,教育就业,IT数码,旅游,美女

娱乐,教育就业,一身清风江湖上
旅游
娱乐
美食,星座命理,旅游,军事,IT 数码
教育就业,投资理财,汽车,IT 数码,安全行车夜视仪,红酒,咖啡,茶,汽车用品总代理
旅游,微博奇葩,新闻趣事,搞笑幽默,穿衣美容
旅游,美食,文艺,娱乐,时尚
IT 数码,旅游,美食,军事,娱乐
健康,娛樂
插画
体育,80 后,交友
旅游,美食,星座命理,娱乐
娱乐
一些事一些情,双鱼座,看书,旅行,电影
娱乐
新闻资讯
娱乐
体育
新闻资讯,汽车,投资理财,健康,美女,IT 数码
娱乐
美食
唯有美食与爱,不能辜负
旅游,时尚,文艺,娱乐,IT 数码,新闻资讯
动漫,电视剧,宅男,时尚,恐怖片,上海人,吃货
美食,文艺,娱乐,听歌
微博奇葩,穿衣美容,名人明星,搞笑幽默
IT 数码
体育
娱乐
新闻资讯,美食,体育
美图摄影,宅腐基,瓶邪,十年,陈奕迅,米米米字旗控,狗狗,吃货,天神花容,耽美
新闻资讯,教育就业,美女,文艺,体育
上海生活
军事
投资理财,IT 数码,美女
军事,IT 数码,文艺,体育,娱乐
文学
文艺
文艺,体育,娱乐
美食,星座命理,教育就业,游戏动漫,文艺
文艺范儿,饰品店,DIY 手工艺品
美食,娱乐,体育,教育就业
教育就业,投资理财,健康,旅游,美食,美食
85 后,巧克力,世界杯,睡懒觉,IT 数码,汽车

温柔,傻,情绪化,小女人,80后宅女,感性,公务员,单纯,暮光之城,天秤
娱乐
微博奇葩
旅游,美食,娱乐
广东生活
投资理财,新闻资讯,西安二手房,WWE
旅游,文艺,星座命理,时尚,教育就业
投资理财
汽车,投资理财,美女
旅游
投资,创业,赚钱,官场形形色色
搞笑幽默,新闻趣事,旅游,江苏生活,名人明星
汽车,铝合金门窗,阳光房
聊天,执着,感性,简单
娱乐,美食,新闻资讯,健康,旅游
娱乐
投资理财,游戏动漫,健康,美食,文艺
旅游
投资理财,旅游,美女,娱乐,体育
搞笑幽默,新闻趣事,旅游,美食,青岛生活
寻找自我,让心灵去旅行,80后,乐观
投资理财
A股,股票
IT数码
旅游,新闻资讯,投资理财,娱乐,体育,美女
投资理财,娱乐,时尚,教育就业,星座命理
时尚
学习,时事,90后,学生一枚,旅游爱好者,爱吃,影视,娱乐
IT数码,娱乐
美图摄影,美女,体育资讯,搞笑幽默,呼伦贝尔生活
军事,自由,旅行,听歌,电影
新闻资讯,旅游,汽车
健康,娱乐,体育
美食,游戏动漫,IT数码
美女,健康,教育就业,军事,体育
娱乐,投资理财,美食,汽车,IT数码
努力,活泼开朗,简单,医生,浪漫,好书,时尚,教育就业,美食,健康
新闻资讯,文艺,汽车,军事,IT数码
济宁生活,健康,新闻资讯,时尚,汽车,旅游
娱乐
其他生活
育儿,奋斗,经济,真实力,80後
投资理财
新闻资讯,旅游,IT数码,体育,美食

健康,旅游,投资理财,时尚,狗狗,电视剧,教育,女人,宝宝,shopping
旅游,美女,文艺,IT 数码
新闻资讯,旅游,投资理财
旅游
IT 数码,体育,汽车,教育就业,投资理财,娱乐,文艺
好书,文艺,时尚,旅游
星座命理
娱乐
美女
投资理财,文艺,养生,笑话
汽车
娱乐
娱乐,文艺,游戏动漫
旅游,电影
淘美食,冷笑话,宅,白羊座
其他生活
豆瓣,紫色,狮子座,小说,交友,宅女,音乐,听歌,80 后,电影
情感生活,天秤座
视频音乐
军事,投资理财
汽车,美食,旅游,文艺,娱乐
时尚
星座命理,旅游,美食
美食,旅行,软件,男人
游戏动漫
娱乐
娱乐
健康,水瓶座,宅女,好性格,时尚
投资理财,旅游,军事,汽车,IT 数码
电子竞技,双鱼座,冷笑话
萌宠
白羊座,睡觉,美食,听歌,电影
IT 数码
IT 数码,旅游,美食,娱乐
健康
旅游,视频音乐,美女,搞笑幽默,南京生活
美食,旅游,健康,文艺,娱乐
娱乐,投资理财,教育就业,文艺,体育
健康,星座命理,美食,娱乐,IT 数码
汽车,投资理财,军事,IT 数码
健康,美女,教育就业,体育,军事
娱乐
完美主义强迫症,爱猫,复古,化妆师,文艺
安妮海瑟薇,张东健,美剧,单身,文学,娱乐,英语,爱笑,双鱼

娱乐
教育就业,健康,新闻资讯,文艺,娱乐
娱乐
宠物图集,文艺
汽车,IT 数码,娱乐
健康
星座命理,健康,娱乐
天秤座
微笑,新妈妈,星座命理,文艺,时尚,娱乐
名人明星,萌宠,美食,视频音乐
美食
文艺,IT 数码,时尚,汽车,游戏动漫
娱乐
美女
文艺,汽车,投资理财,体育,IT 数码
IT 数码
美女,旅游
艺术,唱歌,摄影,时尚,旅游,美食,自由,音乐,电影,90 后
美女,旅游,汽车,体育
新闻趣事,星座运势,读书分享,情感生活,旅游,穿衣美容,星座命理
懒人,喜欢音乐,愤青,IT 数码
搞笑幽默
时尚,军事,体育,健康,游戏动漫,投资理财
娱乐
育儿百科
IT 数码
体育资讯
时尚,旅游,娱乐,美女,IT 数码
投资理财,教育就业,时尚,汽车,美女
体育,美食
教育就业,体育
新闻趣事,美食,名人明星,视频音乐,搞笑幽默
游戏动漫
旅游,美食,纠结,80 后,金牛座
游戏动漫
文艺,体育,看书,户外运动,篮球,唱歌,听歌
旅游,娱乐,IT 数码
美食,旅游,投资理财,美女,健康
游戏动漫
娱乐,美女,旅游,美食,IT 数码,汽车
校园,音乐,善良,90 后,吃货,上网,乐观,好书,摩羯座,动漫
旅游
娱乐,投资理财,时尚,汽车,IT 数码
汽车,IT 数码

娱乐,旅游,星座命理,美食
搞笑幽默,旅游,星座命理,美女
娱乐
美食
投资理财,健康,旅游,文艺,体育
健康,时尚,体育
旅游,教育就业,娱乐,体育,文艺
旅游,听歌,电影,音乐,90 后
娱乐
新闻资讯,健康,旅游,美食,文艺
投资理财
娱乐,体育,IT 数码
健康,时尚,汽车,旅游,美食
教育就业,美食
旅游,萌宠,微博奇葩,新闻趣事
军事,新闻资讯,游戏动漫,娱乐,IT 数码,音乐,电影,游戏
会计,80 后
娱乐
听音乐
投资理财
军事,体育
美女
IT 数码
搞笑幽默
教育就业
娱乐
新闻资讯
摩羯座,刘德华
女汉子,略腐略屌,exo 死忠,小透明,人格分裂
古典音乐,量子力学,古琴,物理,科幻
旅游
汽车
美食
时尚
新闻资讯
娱乐
辽宁生活
生活百科,幽默搞笑,音乐之声,风景旅行,星座物语,经典语录
投资理财,IT 数码,健康,美女
汽车,旅游,军事
体育资讯,体育
娱乐
邵阳生活
娱乐

游戏动漫,文艺,IT 数码
美食,视频音乐,名人明星,星座运势,天津生活
IT 数码,娱乐,健康,旅游,美食
萌宠,健康
娱乐
新闻资讯,旅游,军事
教育就业,美女,健康,旅游,体育
娱乐
娱乐八卦,美术,淡漠
美女
美女
娱乐
房产,美食,旅游,上海生活
游戏动漫,冷血,吃货,音乐,自由,看书,摩羯座
娱乐
美女
天津生活
教育就业,美女,健康,星座命理,IT 数码
新闻资讯
双鱼座,游戏,旅游,80 后,听歌
搞笑幽默,娱乐
穿衣美容
搞笑幽默
汽车
激情成就梦想,偶喜欢玩,电子商务
游戏动漫,wower,80 后,射手座,看书,羽毛球,动漫
微博奇葩,新闻趣事
文艺,游戏动漫,时尚,体育,IT 数码
游戏动漫,星座命理,体育
YOGA,林宥嘉
图片控,體貼,半熟女,性感
高三,发呆,听音乐,美食控,咖啡,宅,巨蟹座,睡觉,上网,自由
娱乐
IT 数码,时尚,汽车,游戏动漫,娱乐
时尚潮流,保养彩妆,EXO,BIGBANG,时尚,旅游,星座命理,娱乐
娱乐,IT 数码
娱乐
党建,媒体,司法,金融,阅读,读书,汽车
体育,娱乐,时尚,文艺,IT 数码,旅游
自由职业,电影
新闻资讯,健康,美食,投资理财
美食,旅游,健康,游戏动漫,娱乐
美女,军事,投资理财
旅游

旅游,IT 数码,双子
娱乐
美食,旅游
爱生活,爱旅行,双鱼座,80 後,营销,互联网,美食,seo,汽车,IT 数码
娱乐
微博奇葩,成都生活
游戏动漫,娱乐,体育,美女,IT 数码
新闻资讯
肾病答疑,肾病饮食,肾病,糖尿病饮食,糖尿病治疗,糖尿病常识,糖尿病
旅游,穿衣美容
汽车,美食,体育,IT 数码
旅游美食,爱大牌,淘宝达人,淘宝店主,逛街,杭州佬,时尚买手,二手奢侈品,双鱼座,网购
美食
娱乐
手指控,既宅又腐,声音控颜控
投资理财,军事,文艺,美女,教育就业,美食
星座命理,娱乐,旅游,美食,摄影,90 後,爱做梦,音乐,舞蹈,看书
中山汽车,汽车
冷笑话
旅游美食,市场营销,军事,体育
军事,游戏动漫
星座命理,健康,军事,美食,体育
游戏动漫,美食,娱乐,IT 数码,旅游
视频音乐
美食
汽车,电影,游戏,上网,娱乐,宅,音乐
娱乐
江苏生活,新闻趣事,视频音乐,教育就业,汽车,娱乐,投资理财
美女,游戏动漫,娱乐
江苏生活,苏州人
美女
时尚,投资理财,娱乐,旅游,美食
杭州市专业减肥,吾纤吾瘦,睡觉睡到自然醒,喜欢唱歌,旅游,美食,80 后,乐天派,爱自由,减肥
游戏动漫,体育,星座命理
北京生活
教育就业,投资理财,旅游,IT 数码,健康,体育,新闻资讯,军事,汽车
美食,军事,体育,文艺,IT 数码
新闻资讯,教育就业,时尚,健康,旅游
美女,新闻资讯,时尚,IT 数码,体育,吃喝玩乐
时尚,健康,娱乐,文艺,美食
体育,美女,投资理财,教育就业,新闻资讯
EXO 团饭,CP 勋鹿,坚持,努力,梦想
我的爱心,你的平安

文艺
教育就业,新闻资讯,投资理财,星座命理,军事
江苏生活,视频音乐,美女,历史,读书,旅游
娱乐
旅游,美图摄影,搞笑幽默,IT 数码
体育
文艺
体育
文艺,美食,星座命理,时尚,娱乐
时尚,军事
旅游
旅游
投资理财,美食,汽车,旅游,IT 数码
美女,体育
新闻资讯,游戏动漫,星座命理,体育,娱乐
体育
视频音乐
搞笑幽默
教育就业
旅游,文艺
金牛座,运动,b 型血,紫色,80 后,成长,亲情,爱情,文学,听歌
IT 数码
美女
娱乐
游戏动漫
娱乐
爱情,传媒,笑话,投资理财,新闻资讯
娱乐,投资理财,教育就业,健康,美食
星座命理,美食
美食
娱乐
娱乐
娱乐,IT 数码
文艺
帅哥,爱吃,快乐,美食,新闻资讯,美女,IT 数码,体育
文艺,新闻资讯,教育就业
健力宝,口琴,巨蟹座
娱乐
深圳生活,旅游,体育资讯
思考,生活大爆炸,驴友,美食,工作狂,80 后的尾巴,军事,教育就业,新闻资讯,文艺
娱乐
旅游,美女,IT 数码,投资理财,健康,时尚
新闻资讯
80 后,狮子座,上网,养狗卖狗,美容,女人,辣妈,运动,时尚

美食
投资理财
军事,乐观,电子商务,旅行
游戏动漫,美食,军事,IT 数码,娱乐
IT 数码,视频音乐,美图摄影,美女
游戏动漫
化妆造型师,旅游,美食,时尚,新闻资讯
娱乐
娱乐
视频音乐,萌宠,微博奇葩,搞笑幽默,游戏动漫,美女,军事,娱乐
汽车,星座命理,军事
摩羯女,双重性格,教师,投资理财,旅游,IT 数码,文艺,娱乐
军事,汽车,旅游,IT 数码,体育
健康,美食,娱乐,IT 数码
娱乐
美女
军事,投资理财,娱乐,IT 数码,文艺
游戏动漫,投资理财,教育就业,汽车,体育
微博奇葩,视频音乐,新闻趣事,搞笑幽默,上海生活
美食
体育
辽宁生活
视频音乐,新闻趣事,搞笑幽默,大连生活,娱乐,投资理财,时尚,美女
汽车,娱乐
星座命理,美食,北京生活,搞笑幽默,美女
汽车
IT 数码
萌宠
娱乐,美食,IT 数码,文艺,体育
美食,旅游,娱乐
星座
健康,文艺,时尚
IT 数码
新闻资讯,旅游,石油天然气管道,旅行
文艺,军事,美食,汽车,旅游
教育就业,健康,旅游,娱乐,投资理财
星座命理,健康,新闻资讯,IT 数码,军事
心情,旅行,旅游,听歌,电影,音乐
健康,文艺,体育,汽车,教育就业,投资理财,游戏动漫,美食,星座命理,娱乐
时尚,美食,星座命理,体育,娱乐
投资理财,军事
旅行,美食
科学探索,车世界,天津生活,体育资讯,IT 数码
教育就业,汽车,体育,娱乐,IT 数码

娱乐
游戏动漫,娱乐
美食
文艺,财会一族,财经潜质,为孤独逃亡,并极力想要摆脱,本质萝莉,御姐未遂,文青路上
美食,健康,育儿亲子,育儿,家庭保健,环保,乐观,婴幼儿健康养护,自由职业
文艺,美女,美食,娱乐,体育
文艺
健康,文艺,旅游,美食,新闻资讯
文艺,美食,旅游,健康,娱乐
旅游,时尚,美食,IT 数码,文艺
美食,游戏动漫
五月天
摄影爱好者,双重性格,80 後,b 型血,情绪化,文艺,美食,星座命理,旅游,健康
体育,运动,健康,美女,美食,旅游
新闻资讯,旅游,体育,投资理财,美食
健康,汽车,IT 数码
娱乐
IT 数码
上海生活
体育,篮球
文艺,军事,游戏动漫
体育,文艺
动漫,美女
旅游,体育
娱乐
猫奴,腐女
教育就业,健康,旅游,文艺,娱乐,电影,八卦,听歌
文艺,IT 数码,游戏动漫
旅游,投资理财,新闻资讯
美女,旅游,游戏动漫
IT 数码,美食,汽车,娱乐,体育
星座命理,美女,IT 数码,文艺,娱乐
文艺
时尚,美食,旅游,游戏动漫,娱乐
时尚,旅游,新闻资讯,美食
汽车,游戏动漫,美食,IT 数码,90 后
娱乐,星座命理
汽车,文艺,IT 数码,游戏动漫
体育,b 型血
美女,教育就业,汽车,IT 数码,文艺
旅游,美食,穿衣美容,新闻趣事,搞笑幽默
微博奇葩,美女,搞笑幽默,新闻趣事
数码,营销,幽默,大学生,旅行,囧,读书,时尚,偶喜欢玩,听歌
爱幻想,奋斗,时评,体育

投资理财,健康,旅游
IT 数码,新闻资讯,军事
体育
体育
CHANEL,PRADA,miumiu
温州生活,美容服饰,情感生活,旅游,新闻趣事
体育
游戏动漫,娱乐,文艺,90 后,音乐,听歌
搞笑幽默,车世界,体育资讯,旅游,美食
娱乐
娱乐
旅游,健康,军事,美女,IT 数码
乐观,时尚
文艺
投资理财,汽车
体育
文艺,新闻资讯,游戏动漫,教育就业
美食
搞笑幽默
美食,教育就业
旅游,新闻资讯,美食
美女,体育
健康
美女
教育就业,美食,旅游,时尚,美女
汽车,教育就业,IT 数码
娱乐
文化发展,平面广告,广告发布,活动策划
娱乐
游戏动漫,事业,爱情,个性,听歌
文艺,IT 数码,汽车,美女,投资理财
娱乐
美食,IT 数码
美食,旅游,萌宠
教育就业
健康,美食,投资理财,IT 数码,新闻资讯,体育,军事
德州生活,旅游,微博奇葩
技术宅,奋斗 ing,IT 菜鸟,北京青年
手绘,板绘,好茶组,冷战组,伏八,尊礼
喜欢旅游,笑,美食
乒乓球,感性
娱乐
游戏动漫
美食,新闻资讯,时尚,投资理财,娱乐

歌曲下载
正义流氓,不入党,不吸毒
穿衣美容,视频音乐,搞笑幽默,辽宁生活,美女
娱乐
IT 数码,美食,新闻资讯,娱乐
美食,军事,星座命理,健康,体育
90 后,电影
旅游,运动,美食,手机达人,Gundam,游戏动漫,游泳,Music,唱 K
游戏动漫
旅游,健康,汽车,体育,IT 数码
娱乐
新闻资讯,旅游,美食,文艺,健康
设计,听歌,旅行,时尚,双子座
学生,矮油,射手座
美女,娱乐
旅游
厦门生活,汽车,爱拼搏,爱生命,交友,90 后,80 后
IT 数码
IT 数码,游戏动漫,时尚,文艺,游戏,上网,吃货,大学,动漫,天秤座
娱乐
时尚,汽车,旅游
中公教师资格,中公教师招聘,搞笑幽默,旅游,美食,微博奇葩,北京生活
娱乐
旅游
旅游,美女
新闻资讯,时尚
NBA,双鱼座,乐观,交友,小说,美食,90 后,电影
美女,IT 数码,娱乐
云南青年,云南旅游,云南生活
娱乐,时尚,文艺
安徽生活
美食
游戏动漫,汽车,美女
新闻资讯
军事,旅游,投资理财,美女,IT 数码
娱乐
美食
教育就业,健康,西安学府,考研数学
福州生活,射手座 O 型血
文艺,游戏动漫,IT 数码,娱乐,体育
美食,旅游
文艺
汽车,军事,文艺
文艺,上网,处女座,80 后,睡觉,校园

娱乐,健康,旅游,体育,感性,古典,历史,足球,简单,美女
美女,星座命理,娱乐
健康,旅游,美食,体育
音乐
旅游,游戏动漫,美食,自由,90后
娱乐
娱乐,游戏动漫
汽车,军事,IT数码,文艺,娱乐
投资理财,时尚,旅游,星座命理,美食
微博奇葩,搞笑幽默
微博奇葩
投资理财
上海生活,旅游,微博奇葩,穿衣美容,美食,时尚
搞笑幽默
北京生活
健康养生,搞笑幽默,旅游,美女
历史,网络,唱歌,营销,白羊座,电影,足球,小说,80后
新闻资讯,军事,文艺,体育,娱乐
游戏动漫,娱乐
军事,IT数码
娱乐
美食
美食,文艺,旅游,时尚,星座命理
热爱生活,旅游
美女
娱乐,汽车,星座命理,IT数码
茶,烹饪,意大利,银魂,历史,游戏动漫,腐女,DIY,猫,美食控
旅游,时尚,服装设计,水瓶女,阅读,化妆,死宅,外貌协会,喜欢旅游
健康,旅游,文艺,美食
娱乐
娱乐
健康,教育就业,投资理财,旅游
Hero,花粉,仙后
表情控,吃货,幽默,搞基,电影,狮子座
运动,80后,听歌,偶稀饭睡觉觉,唱歌,音乐
健康
听歌,美食,爱古典音乐,90后,拖延症晚期,粉红控
舞蹈,旅游,还算典型金牛座,音乐,篮球
搞笑幽默
教育就业,投资理财,新闻资讯,健康,文艺
爱美食,k歌,生活,双鱼座,减肥
90后但我不脑残,大吃货,翻译,创业,小说,80后小疯子
娱乐
娱乐

城画热衷分子,爱狗,宅,电影,我爱常 4 小石头,旅行
爱异想,草泥马党党员,摄影爱好者,希澈澈,娱乐,自由
教师,职场培训,一切美好的事物,听歌,篮球,伪宅
体育资讯,美食
电影,猫痴,大学生,广告人,汉堡,自由
新闻资讯,旅游,健康,美食
天蝎座,megasoul,历史人文,胃口小的好咪咪,美食,上网
IT 数码,军事,旅游,美食
HSJ,乐观,电影,李起光,听歌,懒人一枚
小说,上海,中意睇书,80 后,篮球
甜食控,体育,广告,水果狂,点评网,newcastle
旅游,IT 数码
文艺
善良,射手座,90 后,电影,旅游,Java
穿衣美容
投资理财,缠中说禅,高手中的高手
时尚,游戏动漫,娱乐,IT 数码,美女
蘇打綠,Taro,选择恐惧症,电影,90 后,听歌
美女,旅游,体育,游戏动漫,汽车
电影,策划,体育,肥猫控,漯河小米俱乐部,旅行
文艺
娱乐
固执的金牛座,金牛男,80 后,生活
自由,射手,音乐,营销,购物狂,詩
美食,娱乐
旅游
篮球,电影,唱歌
健康,投资理财,IT 数码,文艺,娱乐
旅游,美食,汽车,美女,体育,爱玩,爱旅游,爱逛,爱吃,好性格
体育资讯
广东生活
美图摄影
校园生活
教育就业,健康,旅游,星座命理,投资理财
投资理财
时尚
投资理财,游戏动漫,时尚,体育,文艺
搞笑幽默,美食,旅游,贵州生活,新闻趣事
时尚,我会拥有快乐,无论现在或以后
新闻资讯,汽车,体育
投资理财,教育就业,时尚,旅游,文艺,健康
活泼,微博控,科技,网购,搞笑,乐观,吃货,美食,时尚,汽车
双子座,广告,大学,宅女,睡觉睡到自然醒,懒,漫画,星座命理
美食

新闻资讯,热爱
娱乐
IT 数码,旅游,搞笑幽默
旅游,健康,教育就业,投资理财,美食
白羊座,小说,电影
体育资讯
军事,新闻资讯,投资理财,汽车,体育
IT 数码
爱情,自由,校园,博客,大学生
体育
娱乐
亲情
爱睡觉,减肥,爱笑,懒,安静,腐,随性,迷糊,夜猫子,时尚
美食
投资理财,新闻资讯,旅游,美食
娱乐
搞笑幽默,安徽生活
美女
IT 数码,宁波生活
外汇
体育
IT 数码
军事,美食,文艺,健康,旅游
旅游
美女,旅游,投资理财
旅游,健康,新闻资讯,投资理财,军事
美剧迷,孩子气,80 后的尾巴,白羊女,Music,B 型血,安静,美食控,胡思乱想,淡定
新闻资讯,投资理财,军事
名人明星
健康,教育就业,星座命理,娱乐,新闻资讯
投资理财
体育,旅游
鳖女
美女
新闻资讯
萌宠
教育就业,健康,旅游,星座命理,文艺
体育,美食,健康,投资理财,娱乐
旅游,汽车,军事,IT 数码
健康,投资理财
投资理财,股票
汽车
时尚
旅游,体育,美食

投资理财,新闻资讯,汽车,IT 数码
旅游,美女,投资理财,新闻资讯,体育
怠惰,不安,悲观,软弱,伪善,懦弱,虚伪,自负
IT 数码,娱乐,教育就业,交友
时尚,健康,美食,IT 数码,旅游,美食,投资理财,娱乐
网购,生活,艺术,搞笑,天蝎座,时尚,自由,听歌,电影,音乐
旅游,星座命理,美食,文艺,娱乐
旅游,美食,娱乐
IT 数码,游戏动漫
美女
努力
娱乐
视频音乐,美女,名人明星,美食,旅游
旅游,投资理财,文艺
娱乐
听歌,股票
旅游
美女
娱乐
穿衣美容,文艺
汽车,投资理财,旅游,游戏动漫,体育
体育
投资理财,时尚,旅游,美食,娱乐
时尚
世博志愿者
娱乐
Dota
美食,旅游,羽毛球,80 后,汽车,唱歌
移动互联网,设计师,军事,IT 数码
吃货,K 歌,90 后但我不脑残,大学生,双鱼座,游戏动漫
健康
时尚,体育,IT 数码,美食,美女,游戏动漫
美图摄影
棒棒堂,敖犬
文艺
旅游,zakka,爱做饭不爱洗碗
美女
IT 数码,游戏动漫,旅游,投资理财,星座命理,汽车
旅行,学生
文藝,娛樂,健康,時尚,星座命理
旅游,IT 数码,文艺,娱乐
投资理财,新闻资讯,娱乐,时尚,教育就业
投资理财
旅游

健康
懒人,淡定,单身,双子,工作狂,相声,红楼梦,自然,宅,新闻资讯
娱乐,游戏动漫,健康
体育,汽车,美食
美容服饰,90后,佳能550d,美妆护肤,佳能eos,狗狗,GF1,悉尼,泰勒taylors,uts
娱乐
宅
萌宠,视频音乐,一颜
吃,小说,动漫,上网,自由,学生,90后,听歌,电影,音乐
白羊座,历史,北京,自行车,摄影爱好者,k歌,电影爱好者,旅游美食爱好者,新闻资讯,文艺
文艺
美食,文艺,娱乐,体育,军事
美食
八卦杂谈,陳經綸,活泼好动,附中,90后
娱乐
娱乐
体育
汽车,新闻资讯,美食,旅游,IT数码
耽美,腐,宅,浪漫,睡觉,文字,音乐,双子座,动漫,电影
听歌,旅行,90后,广告,巨蟹座,紫色,艺术,网络,睡觉,电影
搞笑幽默
娱乐
烹饪,管理学爱好者,经济学,旅游,美食,文艺
投资理财,IT数码
搞笑
新闻资讯,军事,体育
潮,时尚
90后但我不脑残,多愁善感,电影爱好者,上网,听音乐,爱电影,简单,星座
娱乐
美食,投资理财,教育就业
娱乐
娱乐
星座命理
体育,投资理财,新闻资讯
文艺,旅游,星座命理,美食,时尚
娱乐,军事,手机,旅游,IT数码,游戏。
旅游,新闻资讯,星座命理,美食,娱乐,世博志愿者
新闻趣事
健康,文艺,美食,旅游
美女,游戏动漫,IT数码
旅游
职场招聘,旅游,IT数码,太原生活,时尚,新闻资讯,星座命理,文艺
军事
游戏动漫,时尚,军事,娱乐,IT数码,体育,文艺,教育就业

微博奇葩
健康,体育,娱乐,投资理财,新闻资讯
摩羯,执着,运动,足球,真诚,文艺,体育,低调,阳光,法律
美食
游戏动漫
新闻资讯,旅游,文艺,体育
人人,八卦爱好者,影视,感情收藏家,大学生,理财,吃喝玩乐睡,双子座,囧,新闻
游戏动漫
学生,摩羯女,睡觉睡到自然醒,爱笑,90後,创意,爱生活,幸福,乐天派,怀旧
新闻资讯
萌物控,吐槽调戏两不误,囧,宅宅
新闻资讯,IT 数码,美食,旅游,汽车
时尚,文艺,娱乐,上网,小说,网购,动漫,听歌,好性格,美食
体育
美食,健康,文艺
汽车
旅游
李民浩,唱歌,蜡笔小新,减肥控,抹茶控,运动员,吸血鬼日记控,帆船,音乐
时尚,健康
旅游,新闻资讯,娱乐,教育就业,体育
体育,文艺,娱乐
美女
星座命理,游戏动漫,文艺,娱乐,这就是我
视频音乐,珠海生活,呆子,水瓶座,财经,囧,吃货,爱跳舞却跳不好,唱歌
娱乐
美食
投资理财,IT 数码,美女
美女,搞笑幽默,新闻趣事,视频音乐,山东生活
娱乐
唱歌,娱乐
旅游
军事
微博奇葩
名人明星,旅游,新闻趣事,视频音乐
美食,听歌,时尚,自由
旅游,IT 数码,汽车,娱乐,体育
文艺
世博志愿者
新闻资讯,旅游,美食,娱乐
新闻资讯,健康,旅游,美女,文艺
八卦杂谈
吸血鬼,法国,幽默,动漫,笑话,神经质,情绪化,美食,星座命理,文艺
学生一族,K 歌,旅游摄影,拍照,音乐迷,文字控,唱 K
旅游

懒,美食,上网,简单,发呆,文艺,睡觉,旅行,自由
新闻资讯
旅游,星座命理,文艺
美食
艺术,唱歌
旅游
南京,插画,炎亚纶,胡思乱想,娱乐,IT 数码
时尚,IT 数码,美女,新闻资讯,娱乐
娱乐,游戏动漫,美食,体育
美女
教育就业,体育,IT 数码,军事,旅游
汽车,教育就业,IT 数码
军事,男要实女要虚
IT 数码
IT 数码,汽车,游戏动漫,体育,美女
体育
健康,美食,旅游
体育
旅游
新闻趣事
娱乐
投资理财,时尚,旅游,文艺,娱乐
cosplay,music,摄影爱好者,古典艺术,爱唱歌,简单,摇滚,诗歌,音乐爱好者,真实力
体育
游戏动漫
美食,投资理财,文艺
娱乐
旅游
娱乐,睡觉,好性格,天蝎座,宅女
游戏动漫,体育
健康,投资理财,美食,IT 数码,娱乐
美食
死胖子,电影控,网购狂,收集控,深度二次元宅,双面人,腹黑,制服控,双马尾控
教育就业,投资理财,美食,健康,旅游
美女,游戏动漫
自由
旅游,玩儿,营销,新闻,动漫,90 后
IT 数码
旅游,汽车,游戏动漫,IT 数码,体育
旅游
星座命理
健康
健康,新闻资讯,美食,文艺
娱乐,汽车,新闻资讯,军事,IT 数码

街舞,运动,喜欢旅游,IT 数码,考研,健身,阳光,潮流,90 后但我不脑残,音乐达人
背包客,狮子座,随性,旅行爱好者,摄影爱好者,电影,大学,大学生,90 后,动漫
校园生活
健康,小说,好书,宅,旅游,金牛座
北京生活,萌宠,美食,旅游,穿衣美容
个性
IT 数码,美图摄影,视频音乐
笨笨,天平座,旅游,星座命理
娱乐
广东生活,星座命理,旅游,吃货
娱乐
美食
IT 数码,娱乐
幽默,浪漫,玩儿,游戏,旅游,运动,微博,美女,网络,音乐
文艺,时尚,美女,星座命理,美食
新闻资讯
新闻资讯,游戏,宅,网络
搞笑幽默
天津生活,IT 数码,80 后,吃海鲜,历史
尼坤 Nichkhun,SJherry,李弘基 FTisland,韩系控,idol 狂,張根碩,韓飯
娱乐
新闻资讯,文艺,美食
娱乐
IT 数码
娱乐,美食
投资理财,众里寻千千百度
科技
体育,文艺,美女
名人明星
微博奇葩,视频音乐,新闻趣事,搞笑幽默
体育
名人明星
娱乐
时尚
时尚,健康,旅游
娱乐,IT 数码
星座命理,健康,美食,娱乐,旅游,时尚
反家庭暴力法
IT 数码
娱乐
体育
乒乓球,夜猫子,手机看小说,宅男,购物,淘宝
吃货,吃,网络,足球,旅行,宅
时尚,投资理财,美食,旅游,健康

音樂,狮子女,时尚
美女,美食,时尚,教育就业,文艺
娱乐
IT 数码
健康,旅游,文艺
健康
微博奇葩,健康
旅游,睡觉,看书,美女,90 后
新闻资讯
动漫,新闻资讯,军事
搞笑幽默,上海生活,倒塔,美剧控,游戏动漫,文艺
投资理财,新闻资讯,美食
旅游,机械设计,幽默,开朗活泼,汽车,娱乐
小女人,向日葵,美食,臭美,外向,热情,听歌,运动
投资理财,旅游
美女
健康
投资理财
新闻趣事
发而皆中节
健康
微博奇葩
搞笑幽默,文艺,娱乐,IT 数码,游戏动漫,体育
美食,服饰,衣服,湛师,文艺,教育就业,健康,娱乐,时尚
深圳生活,深圳知名品牌,企业管理培训,企业管理咨询,政府质量奖,深圳市长质量奖,卓越绩效模式
IT 数码,教育就业,健康,新闻资讯,投资理财
旅游
娱乐
旅遊,星座命理,汽車,遊戲動漫,時尚
美食,军事,文艺,汽车,旅游
新闻趣事,视频音乐,梅州生活,搞笑幽默,校园生活
宅,旅行,学生,电影
体育
长春生活
体育
投资理财,IT 数码,旅游,美食,娱乐
时尚
美食,旅游,囧,吃货,读书,上网,自由,80 后
读书分享,美图摄影,新闻趣事,美食,搞笑幽默
旅游,美食,萌宠,北京生活,穿衣美容
雅安中学
新闻资讯,旅游,文艺,时尚,美食,娱乐
健康,旅游,星座命理,美食,娱乐

娱乐
IT 数码,美食,旅游,时尚,汽车
体育
WILLIAMS,F1
摩羯座,Music,美食
时尚,健康,娱乐
美女,文学,射手座,自由,美食,90 后,学生
星座命理,教育就业
汽车,体育
美女,健康,游戏动漫,教育就业,IT 数码
旅游,音乐爱好者,IT 数码,时尚,汽车
萌宠,美食,旅游,星座命理,浙江生活
健康,旅游,投资理财
投资理财,美食,新闻资讯,健康,旅游
军事,投资理财,教育就业,体育,娱乐
旅游
旅游
投资理财,旅游,新闻资讯,星座命理,IT 数码
文艺,星座命理,旅游,美食,娱乐
美食
星座命理,美食,军事,娱乐
搞笑幽默,旅游,美食,穿衣美容,健康
美食,投资理财,健康,时尚,娱乐
美女,IT 数码
我爱 UC 浏览器
健康,娱乐,投资理财,游戏动漫,IT 数码
旅游,IT 数码
佐鸣,剑网三,壮哉我大唐家堡,腐女
汽车
娱乐
娱乐
娛樂
游戏动漫
文艺
旅游
星座命理,娱乐,时尚
投资理财,教育就业,美食
IT 数码
投资理财,新闻资讯,美女,旅游,IT 数码
IT 数码
房地产销售,气质美女,没有安全感,怎么吃都不胖,孔雀女,自娱自乐女青年,相亲族
美食,星座命理,娱乐,时尚
IT 数码,美女,搞笑幽默
网络,IT 数码

游戏动漫
时尚,投资理财,旅游
视频音乐,宅男
IT 数码,星座命理,体育,摩羯座,生化危机
旅游,IT 数码,汽车
娱乐
IT 数码
健康,IT 数码,教育就业,新闻资讯,汽车
爱音乐,重口味,美剧,教育,時尚,幻想,健身,烹饪,理财
军事,星座命理,娱乐,美女,健康
健康,新闻资讯,美女,美食,军事
微博奇葩
体育
美食
娱乐
军事,美女,文艺,汽车,搞笑,摄影,帅哥,篮球,愤青,爱情
娱乐,教育就业
爱旅行,原创,文化,休闲,b 型血,水瓶女,教育就业,美食,健康,旅游
星座命理,美食
视频音乐,美女,新闻趣事,搞笑幽默,陕西生活
吃货一枚,随性,enactus,美食
美食
旅游,美食,教育就业,娱乐,美女
IT 数码
游戏动漫
IT 数码,美食,旅游,健康,美女
健康,摄影爱好者
游戏动漫
健康,新闻资讯,体育,娱乐,文艺
娱乐,IT 数码
科技前沿,设计,技巧,IT 互联网
体育,娱乐,游戏动漫,IT 数码
时尚,文艺,新闻资讯,旅游,娱乐,温柔
健康,旅游,星座命理,美食,娱乐
旅游,新闻资讯,投资理财,美食,娱乐
旅游,星座命理,美食,文艺,娱乐
本兮,极韵文化,gogomusic
旅游,星座命理
美女
设计,北京,生活,交友,时尚,微博,90 后
娱乐
美食
教育就业,新闻资讯,健康,旅游,娱乐
IT 数码,职场招聘,搞笑幽默,美食,美女

娱乐,叶阐,安东尼,Ango,颜东,郭顶,海贼王死粉,双鱼座,学生
星座命理,时尚,娱乐,健康,美食
星座命理,健康,教育就业,文艺
配那句地老天荒,有谁
健康,旅游,美食,宅女
投资理财
游戏动漫
娱乐
旅游,美女,微博奇葩,新闻趣事,搞笑幽默
汽车改装,篮球,摄影爱好者,摄影,文字,汽车
新闻趣事
搞笑幽默
游戏动漫
爱,努力
美女,投资理财,军事
新闻资讯,投资理财,军事
体育,IT 数码,新闻资讯
美食,新闻资讯,旅游,旅行,自行车
苏州生活
军事
美女,投资理财,星座命理
新闻资讯
娱乐,时尚
车世界,投资理财
娱乐
星座命理
游戏动漫,投资理财,IT 数码
音乐
健康,时尚,美食,旅游,娱乐
新聞資訊
时尚
美女,教育就业,军事
文艺
名人明星
娱乐,新闻资讯,旅游
教育就业,美食,IT 数码
爱咳嗽,牛鹿,吴亦凡,鹿晗
汽车,娱乐,美食,游戏动漫,体育
体育
文艺
IT 互联网,旅游摄影
搞笑幽默
新闻趣事,视频音乐,美食,旅游,搞笑幽默,体育,游戏动漫,IT 数码
体育

汽车
游戏动漫
游戏动漫,文艺,体育
游戏动漫
娱乐
星座命理,军事,旅游,IT数码,体育,汽车,教育就业,文艺
旅行
旅游,文艺
时尚,游戏动漫,娱乐,旅游,不曾拥有太阳,却享受了阳光
旅游
新闻资讯,投资理财
懒宅女,舞蹈狂,平面模特,音乐狂,追星族,购物狂,英皇控,TVB控,哈韩控,自拍
NBA,学生一枚,摩羯,深圳,强迫症,90後
双子座,自由
广州生活
喜欢听歌,90後,天枰座,音乐控,游戏动漫,IT数码
美食,星座命理
汽车
IT数码
幽默,IT,好性格,网络,摄影爱好者,小说,游戏,80后,旅行,睡觉
90后,音乐,水瓶座,动漫,小说
健康
宅,学生
文艺,美食,健康,娱乐
文艺,美食
美食
娱乐
旅游
旅游
游戏动漫,美食,文艺,娱乐
90后,天秤座,动漫,大学生
美食
旅游,游戏动漫,美食,IT数码
娱乐
美食,体育,文艺,娱乐
水瓶座,搞笑,营销,偶喜欢玩,行情,小清新,时尚,美食,军事,娱乐
美女,体育,娱乐
文艺
美女
视频音乐,美食,旅游,搞笑幽默,教育就业,美女,文艺
娱乐
冷笑话,随性,巨蟹座,散步,视频音乐,旅游
娱乐
我爱UC浏览器

宅男,互联网,电影,美剧,搞笑,偶喜欢玩,吃货,体育,NBA,美食
我爱UC浏览器
健康
星座命理
美食
美剧,上网,羽毛球,自由,听歌,90后,囧,双子座,大学生,手机
游戏动漫,IT数码,体育
军事,IT数码,文艺,体育,娱乐
星座命理,海外生活,穿衣美容,旅游,美食
小说封面,作者,小说,封面制作,封面,封面设计,处女座,沙宝亮,穿衣美容,视频音乐
互粉,时尚,文艺,阅读,读书,新闻,90后
美食
美食
旅游,军事,IT数码,娱乐,体育
游戏动漫
旅游,新闻趣事,江苏生活,汽车
猫咪控,睡觉睡到自然醒,宅女一枚,吃货一枚,盗墓笔记,神经质,花痴,90后但我不脑残,纠结,微博奇葩
IT数码
娱乐
IT数码,文艺,军事,美食,旅游
娱乐
娱乐
IT数码
新闻趣事,搞笑幽默,星座命理
名人明星
穿衣美容
新闻资讯,健康,美食,旅游,时尚
游戏动漫,动画狂
乒乓球,电影控,麦霸,玩儿,八卦,互联网,娱乐,心太软,胖胖,可爱
金融,新闻资讯,教育就业,投资理财
宅,90后,奋斗,IT数码,游戏动漫
美女
旅游
哗啦啦啦啦,music,K歌,心理,娱乐
时尚,丝袜,袜子
精神洁癖,男神林峯,吃货一枚,美女帅哥控
投资理财
时尚,旅游,文艺
娱乐
IT数码
时尚,美女
娱乐
娱乐

娱乐
乐天派,笨搭搭,没心没肺,游戏,偏执狂,射手座,射手,腹黑
教育就业,旅游,新闻资讯,时尚
娱乐
电商,健康,时尚,旅游,美食
文艺,篮球,大学生,阅读,学生,听歌
搞笑幽默
星座命理
时尚,健康,旅游,美食
IT 数码
娱乐
文艺
搞笑幽默,萌宠,旅游,美食,穿衣美容
视频音乐,搞笑幽默,娱乐
Hipa,爱家人,旅行,剩女前传,旅游,大学
投资理财,美食,时尚,汽车,IT 数码
IT 数码,体育,游戏动漫
搞笑幽默
旅游
娱乐
学生,网游达人,80 后,叛逆的鲁鲁修,自由,绿茶抹茶花茶
旅游,唱歌,小女人,小清新
新闻资讯,旅游,美食,娱乐
娱乐
旅游
旅游
娱乐
娱乐
娱乐,健康,旅游,星座命理,美食
动漫
天蝎座,音乐,90 后,眼袋始祖,李晟敏死忠饭,哈韩但爱国
星座命理
文艺
星座命理,美食,文艺,投资理财,娱乐
天生工作狂,宅女
军事,汽车,体育,健康,新闻资讯,篮球爱好者,金牛座,听歌
星座命理,汽车,IT 数码
新闻资讯
感性,摩羯女,艺术,怀旧,A 型血,美剧控,疯子,美食,偏执狂,美图摄影
旅行,射凯,听歌,东方众,宅,艺术
顾家,太慵懒,吃吃吃玩玩玩睡,学生,80 后,运动
吃,7 天画室,I,旅行,Jogging 運動,承德人
flickr,经济金融,处女座,唱 K,简简单单就好。,80 后
BiliBili,莲塘,游戏,博客,听歌,肖邦

大懒人,学生,凌晨六点,没喝过酒,唱歌,听歌
搞笑幽默
90后,美剧,设计,pizza爱好者,80后,学生
语录,好书,85后,听歌,罗志祥的fans,澄海中学
90后,瘋瘋癲癲,自由,旅行,李胜贤,动漫
睡觉,美食,自由,写作,叔控,烤猪
吃,音乐,御姐心,80后,处女座
吃,世博志愿者,阅读,巨蟹座,基督徒,听歌
旅行,boring女一枚,Damon控,萝御双修,爱湘菜,搞笑
旅行,唐禹哲,动漫,大霹雳,手机控,自由
睡觉,互联网,留学,吃货,美食
白羊,画画,爱旅游,简单,看电影,服装设计,文艺,90后,文学,音乐
笑话幽默精选,自诩为制作人,平常心,電影愛好者,宅,音乐
微博奇葩,上海生活
美食
新闻趣事,搞笑幽默,柳州生活,美女,职场招聘
旅行,网络,文字,紫色,财经,金牛座,股票,小说,看书
娱乐,文艺,时尚,美女
娱乐,美发设计师
美女
B2C,B2B,工业电器,资讯,MRO,工业电气,电子商务,工业品
美食,旅游,IT数码
新闻趣事,美女,视频音乐,旅游,美食
文艺,新闻资讯,健康,旅游,投资理财
娱乐
诗词,微笑,搞怪,新闻资讯,健康,星座命理,文艺,互粉,交友
娱乐,胡思乱想,囧,爱玩,手机,处女座,偶稀饭睡觉觉,情绪化
美食,文艺,睡觉,写作,偶喜欢玩,90后,读书,音乐,艺术,唱歌
旅游
美女,星座命理
摩羯女,动漫控,宅
娱乐
美女
美食
游戏动漫,IT数码
娱乐
游戏动漫,汽车,旅游,美食,IT数码
娱乐,时尚,健康,教育就业,星座命理,双子座,动漫
宠物图集
娱乐,时尚,投资理财,美食
美食,K歌,双重性格,喜欢旅游,网购,美食控,天秤座,上网,80后,生活
新闻资讯,时尚,军事,健康,汽车,IT数码
时尚,娱乐
视频音乐,善良,乐观,上网,90后,旅游,自由,听歌,音乐

新闻资讯,星座命理,娱乐,体育,IT 数码
健康,80 后,搞笑,爱情,星座,水瓶座
IT 数码
IT 数码
旅游,林宥嘉,科比,吉他
美食
一切美的事物,音乐,美术,创意
時尚
搞笑幽默
美食,宅,音乐,80 后,时尚
投资理财,文艺,娱乐,健康,时尚,旅游,美食,体育
娱乐
健康,美女,时尚,IT 数码
时尚
娱乐
汽车
娱乐,亲情友情爱情,不可缺一
汽车,新闻资讯,军事,IT 数码
情感生活
旅游,宅,时尚,90 后
时尚,健康,旅游,娱乐,文艺
IT 数码,娱乐,健康,美食
八卦杂谈,校园生活,搞笑幽默,新闻趣事,上网
体育,德国,麦霸,怀旧,nba,神经大条
游戏动漫,军事,IT 数码
娱乐
文艺
美食
娱乐
美女
动漫,奋斗,狮子座,善良,体育
汽车,旅游,体育,星座命理,游戏动漫
新闻资讯
逛街,电视剧,双鱼,吃喝玩乐,韩庚,健康,新闻资讯,旅游,美食,娱乐
新闻资讯,时尚,投资理财,美女,IT 数码
文艺,娱乐
双子座,疯狂,奋斗,李宇春的玉米,玉米一枚
娱乐,时尚,美食
娱乐
文艺,体育,娱乐
教育就业,投资理财,IT 数码
汽车
美食
娱乐,旅游,时尚,健康,美食

旅游,穿衣美容,搞笑幽默,留学,德语,电影,旅行,双子座,小说
学生
体育资讯
投资理财,旅游,健康,美女
游戏动漫,汽车,美女,娱乐,体育
美食,美女
娱乐
热血,正能量,旅游,时尚,文艺
新闻资讯,时尚,旅游,美食,娱乐,特别
SoundHorizon,全职高手,东方 Project,战勇
文艺
新闻资讯
名人明星,徐州生活,新闻趣事,搞笑幽默,更爱我爱的！,爱我所爱的
汽车,旅游,娱乐,美女,IT 数码
健康,美女,汽车,娱乐
星座命理
教育就业
美女,电影,股票,阅读,好书,亲情,手机,汽车,大学,体育
美食,娱乐
新闻资讯,游戏动漫,娱乐
IT 数码
护肤,电影迷,美女,自信,睡觉睡到自然醒,学生一族,矛盾体,感性,浪漫,美容
娱乐
平常心,睡觉,吃货,天蝎座,旅游,90 后,偶喜欢玩,看书,设计
美女
睡懒觉,玩,吃货一枚,美食控,逛街,80 后的尾巴,紫色,无厘头,吸血鬼日记,笑
80 后
旅游,美食,娱乐,时尚,汽车,音乐
广告,平常心
旅游,军事,体育
投资理财,旅游,体育
旅游,娱乐
投资理财,体育,娱乐
旅游
新闻资讯,美女,星座命理
视频音乐,搞笑幽默,星座,羽毛球,英语专业,创业,时尚,旅游,小说,90 后
美女,投资理财,文艺
娛樂
游戏动漫,教育就业
新闻资讯
游戏动漫,投资理财,娱乐
汽车,健康,体育
美食,军事,体育
新闻资讯,旅游,美食

健康,旅游,时尚,文艺,美食,娱乐
美女
旅游,美食,IT 数码,写作
车世界,重口味,大连生活,美女,IT 数码,娱乐
处女座
游戏动漫
90 後,矛盾体,宅腐双修,天秤座,自然神论者,听音乐,动漫,小说,吃货
时尚,娱乐
微博奇葩,星座命理
美女,投资理财,文艺
新闻资讯,美女,IT 数码
美食,军事,体育
游戏动漫,时尚,体育
旅游
静无止静, 易伤
新闻资讯,美女,游戏动漫,教育就业
美食,旅游,酷酷
旅游
美食,淘宝,爱音乐,单身,护肤,爱吃
旅游
NBA、追梦者,哈哈,微博,生活大爆炸,美女
娱乐
汽车,军事,IT 数码,体育,娱乐
江苏生活
搞笑幽默
Candy,天蝎座,小猪,Pink,菁
投资理财,旅游,IT 数码
军事
娱乐
文艺,美食
时尚,健康
星座命理,美食,金牛座,90 后
看电影,爱音乐,健康,美食
80 后
星座命理
娱乐,旅游,星座命理
娱乐
体育,娱乐,星座命理,IT 数码
萌宠,爱狗,腐女,Hmy,张根硕,白羊,Eason
美食
旅游
美女,军事,娱乐,体育,电影
汽车,IT 数码,体育,时尚,投资理财,美食
名人明星,微博奇葩,视频音乐,新闻趣事,搞笑幽默

时尚
娱乐,教育就业,星座命理,旅游,健康
新闻资讯,投资理财,汽车,游戏动漫,IT 数码
文艺,娱乐
旅游,和善,勤奋,坚持
体育
旅游,懒人,低调,神经大条,旅行,睡觉睡到自然醒
IT 数码,体育
新闻资讯,美女
美食
潮缺,江天佑,谢娜,张杰
时尚,游戏动漫
游戏动漫,旅游,美食,IT 数码,娱乐
自由
旅游
美食,时尚,双重人格,抽风,听歌,多愁善感
健康,美女,投资理财,旅游
80 末,室内手绘,室内设计,画画,唱歌,篮球,大学生
体育
星座命理
道教,美女
娱乐,IT 数码
新闻资讯,汽车,旅游,军事,美食
萌宠
校园生活
美食,娱乐,旅游,健康,教育就业
娱乐
时尚
萌宠
健康,星座命理,美食,娱乐
娱乐
美女,游戏动漫
旅游,汽车,投资理财,体育
美女
文艺,娱乐,星座命理,旅游,美食
旅游,美女,视频音乐,美食,搞笑幽默
美女,IT 数码,汽车,游戏动漫,旅游
穿衣美容
摄影渣,专业伪金属党,健身,耳机小烧,篮球,佛洛依德,克苏鲁,旅游,电影
文艺,IT 数码,时尚
穿衣美容,平常心
搞笑幽默
大连生活,穿衣美容
微博奇葩,时尚,文艺,娱乐

青春,時尚,Music,iphone,生活,艺术,旅游,IT 数码,UI,80 后
唱歌,学生,旅行,90 后,宅,旅游,电影,听歌,音乐,美食
新闻资讯,美女,健康,美食,军事,IT 数码
新闻趣事,微博奇葩,旅游,穿衣美容,搞笑幽默
娱乐
汽车,军事,IT 数码,娱乐,体育
教育就业,时尚,旅游,星座命理,娱乐
云南生活
随性,善良,书籍,电视剧迷,处女座
视频音乐
幸福
美食,旅游,星座命理,文艺,娱乐,拍照,心理,music
乐观,it,摇滚,服装,书,法国,粉红控
健康,旅游,时尚,美食,娱乐
上网,自由,网络,平常心,网购,美食,手机,狮子座,80 后,做自己喜欢的
时尚,投资理财,军事
旅游,美食,文艺
娱乐
时尚
名人明星,旅游,视频音乐
健康,美女,投资理财,娱乐,军事
美食
时尚,军事,文艺,美食,旅游,星座命理,新闻资讯
游戏动漫,美食,娱乐,IT 数码
旅游,美食,穿衣美容,搞笑幽默
学生,写作,明星,八卦,政治
文艺,时尚,新闻资讯,旅游,体育,音乐
文化,爱音乐,金牛座
IT 数码,交友,娱乐,生活
健康
美食,新闻趣事,搞笑幽默,投资理财,汽车
军事,教育就业,汽车,投资理财
新闻资讯
体育
娱乐,劲迷黄子华,双反,剪接制作狂,拉面控,孩子气,直辉,风云控,高达痴
旅游,健康,文艺,时尚
美食
投资理财,新闻资讯,汽车,军事,IT 数码
活泼,90,射手座,孩子气,追星,fashion,游戏动漫,娱乐
时尚,IT 数码
美女,健康,投资理财
新闻资讯,美食,体育,美女,投资理财
发呆,听音乐,新闻资讯,游戏动漫,旅游
陕西生活,名人明星,旅游,美女,搞笑幽默

新闻资讯,旅游,时尚,健康,美食,娱乐
旅游,体育资讯,美女,校园生活,武汉生活,睡懒觉,B 型血,玩儿
读书,旅行,玩儿,好书,阅读,双鱼座,语录,网购,小说,上网
视频音乐
汽车
科学探索,视频音乐,美图摄影,电影,历史,音乐,游戏,吃货
台湾生活
搞笑幽默
健康,游戏动漫,美食
文艺,美食,星座命理,时尚,娱乐
娱乐,IT 数码
IT 数码,投资理财,体育,汽车
旅游
音乐控,唱 k
體育
汽车,旅游,军事,体育,娱乐
旅游
游戏动漫,体育
健康
旅游,时尚,健康,教育就业,投资理财
节省用钱,将来成个好家庭,铃铛剑儿,动漫,生活,交友
旅游
美女
微博控,单身,电视剧,真诚,球迷,同志,体育,时尚
视频音乐,LetGo
健康,美食,星座命理,文艺,娱乐
新闻资讯
IT 数码
体育
娱乐,投资理财,汽车,美女,军事
处女座
旅游
投资理财,星座命理,IT 数码,时尚,健康
健康,偶喜欢玩,吃货,旅行,白羊座,唱歌,90 后,听歌
视频音乐,美女,美食,搞笑幽默,旅游
旅游
爱玩,睡觉,自由,交朋友,哈哈,美食,星座命理,时尚,文艺,娱乐
美食,旅游,健康,美女,汽车
旅游,新闻资讯,娱乐,投资理财,好性格,幽默,自由职业
娱乐,新闻资讯,汽车,健康,旅游,IT 数码,美食
文艺,美食,投资理财,旅游
旅游,时尚,IT 数码,汽车,娱乐
健康,体育,娱乐
体育,游戏动漫,美食

健康,新闻资讯,美食,星座命理,IT 数码,好书,电影,IT,吃,小说
美女
摩羯,宅女
投资理财,新闻资讯,教育就业,军事,文艺
IT 数码,娱乐,健康,旅游,星座命理
K 歌,热爱生活,自行车,神经质,宅男,时尚,美食,文艺,娱乐
购物狂,时尚,服装设计,互联网,电子商务,营销,市场营销,潮流,网络营销,服装
娱乐,军事,IT 数码
板绘,唱见厨,平行存在少女,社恐,同人音乐,东方 Project
健康,时尚,美食,旅游,星座命理
汽车,美食,娱乐
新闻资讯,投资理财,健康,旅游,娱乐
新闻资讯,娱乐,文艺,IT 数码
随性
星座命理,美食,IT 数码,体育,娱乐
电脑,工作狂,文字控,自行车,IT 数码
美食
游戏动漫,美食,体育
80 后
NBA,体育
美女,汽车,新闻资讯,军事,IT 数码
车世界,重口味,IT 数码,视频音乐,南京生活
时尚,健康,娱乐,美食,旅游
文艺,娱乐,美食,时尚,旅游
养生,金融,微博控,漫画,投资理财,旅游,军事,IT 数码,体育,娱乐
美食
娱乐,文艺
名人明星
名人明星
巨蟹,动漫,宅女加腐女,浪漫,美剧
文艺,健康,娱乐,教育就业,旅游,喜欢热闹
星座运势,体育资讯,旅游,IT 数码,美女
时尚,健康,美食,IT 数码,娱乐
IT 数码
上海生活
健康
时尚
新闻资讯,投资理财,旅游,美食,娱乐
健康,美食,旅游
美食
游戏动漫,时尚
娱乐,健康,美食,时尚
军事,学生
萌宠

健康,文艺,军事,汽车,IT 数码
旅游,美食,军事,90 后,学生,天蝎座,网购,校园,生活,乐观
娱乐
白羊座,网络,互联网
汽车,新闻资讯,体育,投资理财,旅游
音樂
新闻资讯,游戏动漫
巨蟹座,自由,唱歌,听歌,音乐,时尚,旅行,90 后,美食
新闻资讯,互联网,电子商务,宅
美食
新闻资讯
文艺,体育,娱乐
投资理财,教育就业,健康,旅游
文艺
时尚
搞笑幽默,浪漫,汽车,听歌
星座命理,微博奇葩,搞笑幽默,旅游
手机看小说,小说,新浪读书
上饶生活,新闻资讯,娱乐,教育就业
我爱 UC 浏览器
旅游,美食,穿衣美容,微博奇葩,搞笑幽默,狮子座,学生,校园,吃货,手机
娱乐
旅游,文艺,时尚,娱乐
汽车
娱乐,体育,文艺,时尚,旅游
美女
视频音乐
娱乐,文艺,健康,美食,投资理财
美食,时尚,旅游,汽车,投资理财
文艺
教育就业
旅游,新闻趣事,微博奇葩,星座命理,包头生活
娱乐
八卦掌,传统文化,国学,形意拳,自得园拳社,孙氏太极拳,孙禄堂
美女,体育,IT 数码,汽车,娱乐
娱乐
游戏动漫
汽车,体育
游戏动漫,文艺,体育,健康,美女
游戏动漫,健康,旅游,星座命理,美食
旅游
教育就业
腐女,宅,【佐鸣】火影,瓶邪,动漫
娱乐,文艺,IT 数码,美女,游戏动漫

游戏动漫
爱电影,科技创新,阳台种菜,海洋生物,动物控,环保
新闻资讯,美女,时尚
文艺,体育,娱乐
健康,旅游,星座命理,文艺
音乐,时尚,浪漫
新闻资讯
新闻资讯,时尚,军事,文艺,娱乐
旅游,IT 数码,娱乐,时尚,游戏动漫
IT 数码
娱乐
美女,新闻资讯,军事
汽车
教育就业,新闻资讯,旅游,IT 数码,汽车,投资理财
娱乐,吃
体育
游戏动漫
娱乐
美女,穿衣美容,贵州生活
摄影,博客,唱歌,浪漫,旅游,时尚,网络,听歌,90 后,射手座
游戏动漫
IT 数码,投资理财,体育,时尚,文艺
教育就业,旅游,健康,时尚,娱乐,学生一族
通辽生活
旅游,健康,投资理财
怀旧,情绪化,星座命理
美女
敢爱不敢恨
狗狗,听音乐,创意,运动,狮子座
IT 数码,文艺,体育
新闻资讯,IT 数码
星座命理
唱歌,爱看英语电影
旅游,健康,时尚,娱乐,美食
80 后
旅游
旅游
IT 数码,健康,时尚,汽车,教育就业
时尚
旅游,新闻趣事,重庆生活,搞笑幽默,穿衣美容
旅游,穿衣美容
穿衣美容
健康,星座命理,娱乐,IT 数码
IT 数码

旅游,娱乐
教育就业,文艺,投资理财,新闻资讯,健康
投资理财,美食,健康,娱乐,旅游
音乐控,情感,时尚潮流,韩剧,音乐爱好者,幸福,巨蟹,巨蟹女
美女,汽车,体育,投资理财,旅游
教育就业,健康,旅游
体育,文艺,旅游,美食,军事
旅游,军事,文艺
科学探索,搞笑幽默,旅游
美食,旅游
军事,旅游,健康,投资理财,时尚,美食
美女
韩国代购,化妆品,母婴用品,奶粉,釜山,旅游,健康,时尚,娱乐,星座命理
搞笑幽默
坶绱音乐,写作
视频音乐,微博奇葩,穿衣美容,新闻趣事,星座命理
娱乐
视频音乐,宅男
IT 数码,娱乐
旅游,微博奇葩,新闻趣事,搞笑幽默,河南生活
时尚
体育,教育就业,新闻资讯,健康,旅游,文艺,投资理财
IT 数码,时尚,新闻资讯,文艺
天秤座,星座命理
文艺
投资理财
健康,旅游,美食,IT 数码,文艺
体育
美食,旅游
电影,爱情,90 后
娱乐
汽车
星座命理
音乐发烧友,移动互联网,小米,紫色,网络、玩、
娱乐
微博奇葩,名人明星
科学探索,读书分享,旅游,新闻趣事
投资理财,星座命理,健康,新闻资讯,美食
准基的忠实粉丝
汽车,军事,时尚,体育,娱乐
处女座强迫症,中医控,星座命理,美食,旅游
萌宠
投资理财,星座命理,军事,IT 数码,体育
性爱是性更是爱,性包厢,性爱,日本,女优,AV,美女

娱乐
新闻资讯,军事,娱乐,哲学,情感,休闲,时尚潮流
双子座,亲情,睡觉觉,美食,听歌,小说,宅女,90 后,宅
新闻资讯,游戏动漫,教育就业,文艺,美女,娱乐,体育
星座命理,新闻资讯,时尚,汽车,投资理财
旅游,星座命理,美食,IT 数码,娱乐
名人明星
新闻资讯,健康
娱乐
体育,娱乐
健康,体育,娱乐
文艺,体育,娱乐
体育,美女,时尚
健康,体育,娱乐
健康,文艺,娱乐
健康,文艺,娱乐
文艺,体育,娱乐
安徽生活,旅游,星座命理,穿衣美容,新闻趣事
吃货,宅
文艺,健康,美食,娱乐,时尚
旅游
游戏动漫,IT 数码,美女
教育就业,投资理财,IT 数码
旅游,美食
健康,新闻资讯,娱乐
美食
文艺,体育,娱乐
文艺,体育,娱乐
文艺,游戏动漫,星座命理
美食
L115A3
健康,体育,娱乐
健康,新闻资讯,娱乐
文艺,体育,娱乐
文艺,体育,娱乐
健康,体育,娱乐
情感生活,视频音乐,搞笑幽默
健康,新闻资讯,娱乐
文艺,体育,娱乐
星座命理,美食,军事
体育,健康,文艺,娱乐
文艺,体育,娱乐
微博奇葩
旅游,微博奇葩,视频音乐,新闻趣事,搞笑幽默

娱乐
IT 数码
美食
搞笑幽默,芜湖生活,新闻趣事,微博奇葩,穿衣美容
星座命理,健康,时尚,教育就业
文艺,美女,体育,娱乐,游戏动漫
时尚
时尚,星座命理,美食,文艺,娱乐
星座命理
美食,军事,星座命理,游戏动漫
美女,新闻资讯,体育,教育就业
吃货,果粉,最强王者,网游,睡觉睡到自然醒
汽车,体育,游戏动漫,军事
教育就业
IT 数码
旅游,美女,投资理财,教育就业,军事,读书
美食
文艺,美食,旅游,健康
军事
巴彦淖尔盟生活
健康
时尚,星座命理,文艺,娱乐,旅游
娱乐,文艺,投资理财,美食,健康
天蝎座
健康,新闻资讯
搞笑幽默,传媒,阅读,乐观
车世界
旅游,健康,美食,时尚,文艺,体育
IT 数码
教育就业,投资理财
娱乐
旅游,新闻资讯,美食,健康
旅游,娱乐
娱乐,时尚
旅游,健康,时尚,美食,娱乐
汽车,体育
美女
搞笑幽默,新闻趣事,江苏生活
美食,旅游,娱乐
旅游,健康,游戏动漫,美女,时尚
投资理财,美女
娱乐
天不怕地不怕,善良,扮成熟,好孩子,好男人,90 后,懂得爱,爱
时尚,健康,旅游,体育

穿衣美容,宅女
设计,摄影,篮球,汽车,文艺,体育
游戏动漫
体育,娱乐
健康
体育
体育,教育就业,文艺,汽车
穿衣美容
美食
小说
军事,IT 数码,文艺,体育
快乐,新闻资讯,教育就业
军事天地
娱樂
新闻资讯,军事,娱乐
美女
历史,传统文化,汉服,游戏动漫
教育就业,旅游,健康,文艺
校园生活,八卦杂谈,美女
时尚,美食
美食,娱乐
健康,美食,体育,糟蹋新闻,某些音乐,篮球,旅游,电影
微博奇葩
萌宠,权志龙,迷糊,麦霸,简单,音乐控,music,浪漫,电视剧,音乐迷
教育就业,美食,IT 数码
射手座,北京,青岛,航运物流,电影,英语,吃,唱歌,魔兽世界,旅行
體育,美食,旅遊,時尚,娛樂
游戏动漫
娱乐
视频音乐,搞笑幽默,体育,娱乐
体育
文艺,体育,娱乐
娱乐,旅游,IT 数码,美女
健康,投资理财,娱乐,IT 数码
北京生活
游戏动漫
星座命理,美食,文艺,浪漫,90 后,双鱼座,吃,偶喜欢玩,语录,生活
律师
时尚
健康,美女,旅游,IT 数码,娱乐
萌宠
新闻资讯,教育就业,星座命理,美食,美女,文艺
美食
新闻资讯,美女,IT 数码,时尚

健康,投资理财,IT 数码
幻想,我就是我,music,90 后但我不脑残,电视剧,健身,投资理财
游戏动漫
游戏动漫,美女,军事
旅游
投资理财,汽车,新闻资讯,旅游,美食
文艺,健康,娱乐
宅女,好书,亲情,温柔,乐观,文字,网络,胡思乱想,原创,美食
爱生活,爱幻想,爱做梦,旅行,骑行爱好者,骑行,狮子座
娱乐,美食
游戏动漫,汽车,娱乐,IT 数码
娱乐
猫狗控,跑步,SAT,宅,看书,吃货,处女座
旅游
大连生活
IT 数码
淘宝四钻卖家 QQ,1847278598,时尚,美食,听歌,电影,音乐,90 后,我爱 UC 浏览器
IT 数码,信息无敌
新闻资讯,投资理财,汽车,文艺,娱乐
娱乐,美食,汽车,旅游,体育
宅男一族,IT 男一枚,教育就业
美女,旅游,健康,汽车,IT 数码
美食
旅游,微博奇葩,新闻趣事,视频音乐,美女
美食,星座命理
旅游
IT 数码
文艺
文艺
教育就业,旅游,IT 数码,体育,文艺
美女
教育就业,娱乐,时尚,星座命理,美食
跑步,娱乐,投资理财
汽车,旅游,IT 数码,健康,体育
读书分享,动漫,名人明星,美图摄影,IT 数码,广州
爱美食,睡懒觉,爱音乐,看电影,发呆,淘宝
娱乐
编辑,户外,美容护肤,完美主义,画画,心理
壁画,壁纸,墙纸销售,IT 数码,财经,IT
文艺,IT 数码,娱乐,投资理财,新闻资讯
西安生活,新闻趣事,搞笑幽默
新闻资讯
健康,旅游,文艺,星座命理,娱乐
健康,体育

娱乐
游戏动漫
旅游,游戏动漫,美女,汽车
娱乐
旅游,星座命理,健康,新闻资讯,时尚
星座命理
美食
美食
美食
美食,娱乐
美女,文艺,体育,娱乐,健康
IT 数码,游戏动漫
旅游
娱乐,游戏动漫
文艺,时尚,健康
体育,旅游,听歌,90 后
广告,设计,美术
韩国生活,娱乐
时尚,美女,星座命理,军事,IT 数码
游戏动漫,汽车,新闻资讯,军事,娱乐
旅游,美食,娱乐,文艺
美食控,魔羯座,美术,动漫迷,摄影
体育
文艺,游戏动漫,旅游,看书
新闻资讯,美食,IT 数码,文艺,娱乐
旅游,娱乐,时尚,美女
一些事一些情,狮子座,小说,上网,自由,电影,听歌,90 后,音乐
汽车
打球,随性,武术,爱旅行,网络,手机,狮子座,运动,夜猫子,忧郁
新闻资讯
美女,美食
娱乐
旅游
杨晓明
名人明星,穿衣美容,搞笑幽默,绍兴生活,娱乐,时尚,美女,文艺
吃吃吃吃货,蜂蜜,K 歌,游泳,羽毛球,旅游,自行车,吃货,松鼠
美食
体育
互听
旅游
爱情,读书,听歌,偶喜欢玩,偶稀饭睡觉觉,八卦,游戏,巨蟹座,学生,90 后
美女
新闻趣事
HITFM887,时尚

投资理财,新闻资讯,健康,旅游,娱乐
旅游,美食,文艺,时尚,娱乐
高中生,2 货青年欢乐多,三国杀,火影,学生一族
美女,军事,旅游
淘宝控,怀旧,团购,上网,睡觉,排球,臭美,孩子气,健康,美食
美食,美女
视频音乐,动漫,旅游,美食,美女,游戏动漫
新闻趣事
新闻资讯
旅游,时尚
体育
娱乐
吃,自由,乐观,狮子座,搞笑,看书,学生,平常心,娱乐,旅行
文艺
健康,美食,IT 数码,驴友,美食爱好者
时尚,音乐,旅行,宅
IT 数码,旅游,新闻资讯,汽车
旅游,美食,微博奇葩
欧美,心理,生活
娱乐,美女
美女
旅游,时尚,美食,娱乐
星座命理,搞笑幽默,名人明星,微博奇葩,穿衣美容
旅遊,视频音乐,萌宠
旅游,美食,娱乐
搞笑幽默,八卦杂谈,新闻趣事,名人明星,美女,小霸气
娱乐,教育就业
汽车,美食,文艺,教育就业,健康
时尚,军事,旅游,美食
星座运势
星座命理
搞笑幽默,八卦杂谈,美女,旅游,少女时代,90 后但我不脑残
名人明星,时尚,健康,文艺
美食,汽车
汽车,时尚,IT 数码
汽车
IT 数码,军事,旅游,美食,美女
旅游
军事
新闻趣事,旅游,美食,搞笑幽默,穿衣美容
旅游,美女,美食,文艺,娱乐
手工,吃货一枚
时尚,美食,旅游
欧美控,文化,自信,健康,投资理财,善良,幽默,理想主义

旅游
娱乐,时尚,游戏动漫,体育
汽车,IT 数码
体育
旅游,微博奇葩,美女,搞笑幽默
健康
美食
旅游,美食,穿衣美容,搞笑幽默,新闻趣事
新泽西,处女座,游戏,NYC
旅游,新闻趣事,广东生活,美食,美女
娱乐
投资理财
育儿百科
新闻资讯,美女
星座命理,美食
健康
时尚,文艺
美食,旅游,星座命理,体育,娱乐
时尚,美食,文艺,游戏动漫,娱乐
旅游,文艺
美女
体育
美女
健康,文艺,娱乐
时尚,旅游,美食,星座命理,娱乐
双鱼座,幽默,微博,电影,搞笑
宠物图集
美女,篮球爱好者,打球,日语,旅游摄影,完美主义,可爱,玩,创意,爱吃
新闻资讯,投资理财,美女,体育
shawn,Eason,柯尔摩,无所适从,不过怪咖
娱乐
搞笑幽默
福建生活
美食,旅游,娱乐
旅游
汽车,体育
旅游,美食,时尚
小说,强迫症,敏感,安徽生活
爱自由,思考,管理,情感,真诚,读书,汽车,创业,朋友,A 型血
汽车,投资理财,IT 数码,美女,游戏动漫
游戏动漫,投资理财,军事
健康,投资理财,旅游,文艺,娱乐
营销,大学,校园,电子商务,游戏,生活,艺术,美女,娱乐
美女

穿衣美容
IT 数码,军事,美食,游戏动漫,投资理财
娱乐
文艺
时尚,旅游,美食,健康,汽车,教育就业,IT 数码,星座命理
休闲,淡定,IT 数码,表演,安静,快乐,天平座,打球,寂寞,天秤
搞笑幽默,媒体,80 后,旅游
旅游
旅游
柯南,愤青,创意,阳光,单身,爱生活
镜音双子,GUMI,傲娇,病娇,腹黑,洛天依,结月缘,V 家,SEEU,东方 project
投资理财
强迫症,完美主义者
健康
时尚,投资理财,旅游,美食,娱乐
时尚,投资理财,美食,星座命理,旅游,健康
投资理财
新闻资讯
海绵宝宝,摄影,飞模控,电脑游戏爱好者,文艺,投资理财
娱乐,文艺,旅游,新闻资讯,健康
旅游
网购,幽默,艺术,天秤座,萝莉,90 后
娱乐
娱乐
新闻资讯,游戏动漫,美女,教育就业,旅游,美食,娱乐,体育,文艺
旅游
北京生活,美食,旅游
IT 数码,户外,麦霸,团购
IT 数码,军事,汽车,体育
汽车,军事,IT 数码,体育,娱乐
美食,娱乐
美食,IT 数码,文艺,体育,娱乐
娱乐
健康,美食,娱乐,大学生
旅游,文艺,体育
游戏动漫,娱乐,健康,投资理财,旅游
电影
文艺
娱乐
美女
旅游
投资理财
娱乐
新闻趣事,搞笑幽默

仙后,旅游,东方神起,美食,名人明星
八卦杂谈
美女
星座命理
IT 数码
体育
游戏动漫,娱乐
IT 数码
旅游,美女,美食,娱乐,体育
汽车,游戏动漫,文艺,IT 数码,娱乐
投资理财
健康,健身
教育就业,汽车,时尚,投资理财,体育
善良,摩羯座
情感生活
美女,汽车,旅游,娱乐,体育,时尚,听歌,双子座
新闻资讯,时尚,教育就业,投资理财,娱乐
健康
星座命理,旅游,文艺,Dream,Freedom,freedom
IT 数码
育儿百科
美食,游戏动漫,体育,娱乐
穿衣美容,时尚
生活,时尚,听歌,旅游
摄影,IT,好性格,美女,双鱼座,旅游
新闻趣事,搞笑幽默
外貌协会,懒人,射手女,淘宝,发呆,爱睡觉,重口味,我就是我,帅哥
四川生活
军事,IT 数码
生
体育
星座命理,游戏动漫,美食
旅游
旅游,星座命理,美食
健康,军事,汽车,娱乐,体育
程序员
星座命理,穿衣美容,名人明星,视频音乐,旅游
IT 数码,搞笑幽默
时尚,娱乐,美食,旅游,新闻资讯
O 型血,80 后,发呆,游戏动漫,美食,玩游戏,近视,双鱼座
荼蘼,同袍,汉服,天汉
星座命理
文艺
时尚

视频音乐,摄影爱好者,NBA,爵士鼓,everything,灰色,天蓝色,街舞,Music,Basketball
美女,幽默,搞笑,摄影,听歌,唱歌,上网,互联网,篮球,IT
娱乐,IT 数码,美食,健康,文艺
美女
健康,美女,军事,娱乐,体育
时尚,新闻资讯,体育,旅游,军事
娱乐
美食,时尚,投资理财,健康
旅游,美食
新闻趣事,雍正的妹妹
文艺,天蝎座,宅男,睡觉,吃货,摄影,双鱼座,摄影爱好者
搞笑幽默
娱乐
90 后,游戏,学生,吃
娱乐
搞笑幽默
文艺
娱乐
美女,时尚,军事,文艺,娱乐
投资理财,娱乐
搞笑幽默
娱乐
新闻资讯,投资理财,教育就业,IT 数码,军事
美女
军事
美食,投资理财
美女,其他生活,视频音乐,新闻趣事
视频音乐,艺术,玩儿,版画,水瓶座
健康,教育就业,星座命理,新闻资讯,美食
體育
星座命理,新闻趣事,湖南生活
奋斗,广州,粤语,天秤座
美女
娱乐
体育
健康,旅游,美食,娱乐
时尚,健康,旅游,文艺,美食
体育
星座命理
汽车,体育,IT 数码,美食,旅游
娱乐,星座命理,美食,健康,射手座,搞笑,听歌,时尚,音乐
旅游,汽车,游戏动漫,美食,娱乐
美食,娱乐,投资理财,健康,旅游
美食,时尚,新闻资讯

体育
美女,旅游,美食,娱乐,体育
星座命理,文艺,游戏动漫
日剧控,写作,娱乐,美食,游戏动漫,文字爱好者,日本文化爱好者,日漫追求者
投资理财
睡觉,乐观,平常心,吃,娱乐
新闻资讯,健康,军事
名人明星
美女,健康,美食,娱乐,文艺
购物达人,电子商务,吃,美食,时尚,自由,偶稀饭睡觉觉,80后,营销,微博
IT数码,美食,健康,时尚,旅游
星座命理
旅游
搞笑幽默,玩,阳光,爱生活
体育
军事,文艺
旅游,萌宠,视频音乐,穿衣美容
IT数码
美食
美食,旅游,星座命理,搞笑幽默,穿衣美容
娱乐,睡觉,欣赏的姿态
投资理财,旅游,军事,文艺,体育
旅游
SONY粉,WOWer,音乐改变世界,24格
健康,教育就业
健康,时尚,旅游,星座命理,美食
体育
微博奇葩
美食,汽车,时尚,游戏动漫,文艺
娱乐,旅游
娱乐
美女,文艺,娱乐,旅游,体育
传媒,篮球,听歌,电影,音乐
文艺
运动,美食
健康,文艺,投资理财,专注UI
爱自由,包包,爱幻想,看书,动漫,爱生活,爱睡觉,可爱,80後,天蝎女
军事,IT数码,旅游
娱乐
时尚,旅游
美女
穿衣美容
娱乐
旅游,星座命理,文艺

星座命理
体育
新闻资讯,健康,旅游
美食,旅游,广东生活
娱乐
新闻资讯
汽车,时尚,IT 数码,军事,美食,旅游,新闻资讯,娱乐
娱乐,美食
美女
游戏动漫,文艺,汽车,旅游,美食,IT 数码
文艺
文艺,IT 数码
体育
娱乐
google,诗歌,亲情,大学,交朋友,苹果,爱生活,影视,打篮球,IT 数码
旅游
时尚,汽车,教育就业,星座命理,健康,投资理财
游戏动漫,美食
穿衣美容,旅游,美食,广东生活,看书,80 后,淡淡的忧伤
写字,科幻,武术
美食
新闻资讯,教育就业,健康,投资理财,汽车,军事
娱乐
健康,时尚,投资理财,新闻资讯,文艺
星座命理,旅游,文艺,美食,娱乐
星座命理
新闻趣事,搞笑幽默,辽宁生活,视频音乐,旅游,学生
教育就业,投资理财,IT 数码
健康,体育,娱乐,游戏动漫
旅游,IT 数码,文艺,足球,电影控,写作,摄影爱好者,体育
星座命理
IT 数码,体育,汽车,旅游,军事
看书,爱旅游,爱电影
娱乐,星座命理,文艺
投资理财,美女,体育,旅游,IT 数码
浙江生活
旅游,健康,汽车,IT 数码
新闻资讯,投资理财
旅游
美食,教育就业,文艺,投资理财,健康
视频音乐,星座命理,名人明星,搞笑幽默,微博奇葩
活泼开朗,律师,旅游,星座命理
美食
时尚,旅游,文艺,新闻资讯,健康

健康,美女,娱乐,旅游
旅游,美食,娱乐,时尚
美女,IT 数码,汽车,旅游
游戏动漫
平面设计
资讯,天秤座
电影,旅行,时尚,旅游,自由,美食,宅,80 后,听歌,音乐
大学生,动漫,体育,音乐,交友,IT,新闻,电影,读书,听歌
穿衣美容,旅游
娱乐
听歌,游戏,网络,宅,IT,媒体,上网,搞笑,动漫,睡觉
视频音乐,娱乐
星座命理,美食,军事,文艺,娱乐
美食
美食,健康,旅游,文艺
娱乐
教育就业
娱乐,新闻资讯
健康,宅女,电影,善良,80 后,摄影爱好者,白羊座,自由,亲情,上网
体育
军事
体育资讯,成都生活
娱乐,IT 数码,星座命理
萌宠,名人明星,旅游,美食
读书分享,搞笑幽默,名人明星,美食,新闻趣事
健康,文艺,美食
娱乐,文艺,旅游,健康,教育就业
宠物图集
我爱 UC 浏览器
汽车,旅游,新闻资讯,美女,娱乐
天理,人欲,时尚,美食,宅,听歌,80 后,电影,音乐
萌宠
游戏动漫,旅游,美食
新闻趣事,美食,旅游
穿衣美容
娱乐,时尚,美食,旅游,星座命理
旅游,视频音乐
健康,星座命理,投资理财,娱乐,文艺
美食,健康,新闻资讯,教育就业,双子座,宅女,一些事一些情
娱乐
北京生活
汽车,健康,时尚,旅游
投资理财,时尚,美食,文艺,健康
登山

IT 数码
美食,旅游,美食,摄影
娱乐
学生一族,体育
搞笑幽默
LOL,宅,昆 12 中,DNF
汽车,时尚,游戏动漫,旅游,娱乐
娱乐
爱自由
美女,搞笑幽默
财经资讯,房产,车世界,旅游
体育,陈奕迅,林宥嘉,唱歌,科比,湖人,皇马,大学生,单身,吃喝玩乐
搞笑幽默
体育
旅游,微博奇葩,星座命理,新闻趣事
美女,微博奇葩,武汉生活,搞笑幽默,新闻趣事
新闻资讯
活泼开朗
新闻趣事,微博奇葩,搞笑幽默,旅游,厦门生活
游戏动漫,美食,军事,IT 数码,文艺
美食,娱乐,旅游,游戏动漫,文艺,投资理财
IT 数码
美女
宅男,爱幻想,奋斗,真诚,青春,Music,唱 K,坚强
摩羯,胡思乱想,教育就业,汽车,文艺,军事,体育,沉默,热爱生活,水瓶
边伯贤是小牛肉,李赫海是底线,SUPERJUNIOR,粉色重症者,萌物控正太控,山田凉介
健康,旅游,娱乐,军事,IT 数码
美食,娱乐,游戏动漫
星座命理
军事
新闻资讯,投资理财
搞笑幽默,音乐
军事,体育,旅游,旅行
工作,沉默,古典音乐,旅游
新聞資訊,美食,IT 數碼,娛樂,體育
美食,娱乐
奋斗,服饰,巨蟹,淘宝,服装,安静
IT 数码
文艺,健康,旅游,大学生,宅
看书,上网,摄影,旅行,电影,美食,听歌
体育
自由职业
文艺,爱毛毛,爱生活
娱乐

旅游
娱乐
驴行,登山,自行车,旅游,美食
投资理财,旅游
体育,美女
情感生活
美食
汽车,IT 数码,游戏动漫,体育,娱乐
旅游,美食,热爱生活,宠物,低调,爱旅游
美食,体育,娱乐,IT 数码,新闻资讯
娱乐,新闻资讯,美食,健康,游戏动漫
投资理财,旅游,美食,IT 数码,文艺
美食
读书分享
美女,浙江生活,冷暖自知,仅此而已,安静
娱乐,投资理财,美女,IT 数码
游戏动漫,美女,星座命理,体育,娱乐
美食,善良,好性格,白羊座,时尚,看书,旅行,平常心
健康,教育就业
美食,旅游,IT 数码,文艺,健康,摩羯,做作业,素食,生物钟
投资理财,汽车
搞笑幽默
萌宠
投资理财,军事,IT 数码
汽车,美女,体育
美食
旅游,投资理财,时尚
健康
娱乐
健康,时尚,美食,文艺,新闻资讯
福建生活,新闻趣事
旅游
汽车
旅游,穿衣美容,美女
新闻资讯
搞笑幽默,微博奇葩
IT 数码
健康,美女
娱乐,爱睡觉,爱旅游
美女,文艺,IT 数码,体育,旅游,军事
nano 唱见,二点五次元,唱见厨,DECO27,MAMO,nero,nico 厨,恋声癖
新闻趣事
健康,新闻资讯,娱乐
星座命理,美女,新闻资讯

IT 数码,文艺
爱生活,巨蟹座,学生,游戏动漫,星座命理
康复医学,电影,时尚
军事,IT 数码,文艺,体育,娱乐
IT 数码,运动
游戏动漫,体育,NBA
文艺,健康,旅游,星座命理,美食
娱乐,体育,美女,军事,音乐爱好者,书法,好书,IT 民工
美食
美女,游戏动漫
克亚营销,短信营销,整合营销,营销策划
天然呆
时尚
健康,旅游,美食,体育,文艺
时尚,游戏动漫
投资理财,新闻资讯,军事,IT 数码,娱乐
读书分享
旅游,美食,星座命理
军事
IT 数码,视频音乐,搞笑幽默
娱乐
旅游,宅,80 后
娱乐
娱乐
神经质,美食爱好者,电影控,旅游摄影
娱乐
体育,自由,旅行,交友,电影,音乐
健康,时尚,汽车,娱乐,美女
投资理财,汽车
六爻,周易
视频音乐
IT 数码
我爱 UC 浏览器
听歌
搞笑幽默
美食,新闻资讯,文艺,健康,旅游
时尚
广州生活
吃货一枚,时尚潮流,爱玩,爱美食,幻想,电脑,猫,化妆,情感,手机看小说
江苏生活
娱乐
旅游,美食,视频音乐,投资理财,健康
宅男,文艺,健康,新闻资讯,投资理财,军事
健康,教育就业,新闻资讯,美食,娱乐

旅遊,名人明星,搞笑幽默,台湾生活,视频音乐
微博奇葩,搞笑幽默
教育就业,娱乐,新闻资讯,投资理财,IT 数码
新闻
时尚,旅游
旅游,美食,IT 数码,体育,娱乐
旅游,深圳生活,体育资讯,喜欢旅游,双子
穿衣美容
旅游,时尚,美女
路西法,尋找自己,愛世間萬物,涅槃
游戏动漫
旅游,IT 数码,文艺,美食,娱乐
健康,旅游
体育
微博奇葩,旅游,搞笑幽默,视频音乐,90 后
宠物图集,搞笑幽默
成都生活
娱乐,文艺,旅游,星座命理,美食
满街逛,不够宅,射手座,生活,上网,旅游,自由,时尚,80 后
汽车,美食,新闻资讯,健康,旅游
IT 数码,体育,汽车,美女,游戏动漫
视频音乐,搞笑幽默,游戏动漫
投资理财,军事,文艺,旅游
看小说
娱乐,健康,旅游,星座命理,美食,愤青,爱幻想
娱乐,游戏动漫
音乐,时尚,旅游,打篮球
美食,娱乐
美食
讲信用的
摩羯座,摄影爱好者,NBA,音乐,创业,80 后,阅读,唱歌
体育
美食
投资理财,教育就业,星座命理,IT 数码,体育
健康,时尚,教育就业,旅游,文艺
娱乐
汽车,体育
汽车,旅游,新闻资讯,文艺,娱乐
军事,文艺,娱乐,汽车
网络,八卦爱好者,处女座,娱乐,睡觉
体育
搞笑幽默
喜欢运动
时尚,健康,新闻资讯,美食,娱乐

娱乐,旅游,篮球,运动,手机,电影,狮子座,NBA,幽默,听歌
时尚,文艺,游戏动漫,旅游,宅
体育
缘妈,抹茶控,号码百事通,美食,宅,80 后
旅游,广东生活
体育
美女,不知道啊
美女,新闻资讯,文艺,星座命理,军事
星座命理,娱乐,好玩
美食,旅游,美女,体育,娱乐
娱乐
IT 数码
萌宠
游戏动漫,广东对外贸易,多重性格,动漫爱好者,蘑菇头
星座命理,汽车,旅游,美食,娱乐
娱乐,投资理财,健康
穿衣美容,萌宠,旅游,美食,搞笑幽默
星座命理,时尚,教育就业,美食,旅游
珠海生活
文艺
旅游,时尚,文艺,娱乐,星座命理
强迫症,购物狂,动漫迷,看小说,时尚,文艺,娱乐
健康,时尚,娱乐
IT 数码
穿衣美容,lol
健康,文艺
汽车,星座命理
萌宠,美食,简单,神经质,法国,迷糊,学生一枚,安静,懒,美食控
美女
旅游,吃货,微白痴,爱旅行,90 后
投资理财
文艺
文艺
美女
搞笑幽默
教学,电影,天蝎座,生活
汽车,A 股,幽默,偶稀饭睡觉觉,80 后,好性格,看书,平常心,音乐,网购
娱乐
文艺,投资理财,娱乐,教育就业
投资理财,军事,娱乐
美食
新闻资讯,军事,IT 数码
新闻资讯,武汉招聘会
旅游,星座命理,美食,娱乐

娱乐
创意中国摄影,个性写真,厦门穿越摄影,古韵风,古装婚纱照,中国式婚照,厦门中国风摄影,厦门古装写真,厦门古装摄影
娱乐
体育,军事,游戏动漫,美女
微博奇葩,搞笑幽默,旅游,时尚
IT 数码
健康,IT 数码
好男人就是我,张国荣,似水流年
美食,IT 数码,文艺
文艺
动画,自恋,投资理财,网购,游戏,运动,美食,时尚,音乐,电影
听歌,读书,美食,喜欢静,却又喜爱热闹
玩儿,白羊座,篮球,唱歌,自由,80 后,听歌,音乐
做自己就好!
標準順德人,睡神一枚,不是美女,愛唱 K,90 后,郑中,张杰,靓仔
美食,萌宠,微博奇葩,搞笑幽默,视频音乐
紫色控,爱幻想爱期待,喜欢回忆过去,很慢热的,爱狗狗,超爱刺激,小身体有大力量,是睡魔,爱逛街拍照,相当倔强
矛盾体,放空
娱乐,宅,善良,艺术,90 后,学生
投资理财,教育就业,新闻资讯,英语单词,背单词,敏特,敏特英语,敏特记忆引擎,英语,英语学习
娱乐,汽车,时尚,健康,旅游,投资理财,文艺
IT 数码,游戏动漫
旅游
美食爱好者,爬山,射手座,美食,军事,IT 数码,体育,娱乐
旅游
投资理财
passion,24Drums,Architect
星座命理,新闻趣事,辽宁生活,IT 数码,美食,健康,游戏动漫
娱乐
体育资讯
旅游
汽车,体育
美女
绍兴生活,名人明星,美女,八卦杂谈
爱情,听歌,90 后
旅游
汽车,健康,旅游,星座命理,美食
健康,IT 数码
八卦,读书,小说
IT 数码
深圳生活

旅游,健康,时尚,星座命理,娱乐
汽车,游戏动漫,星座命理,美食,娱乐
时尚,美食,星座命理,文艺,娱乐
文艺,我,涵涵,70 后,睡觉
娱乐
旅游
旅游,健康,投资理财,美食,娱乐
分裂症,篮球,金融,商业地产,建筑信息 BIM,房地产
汽车,体育,旅游
体育
军事
美食,旅游,时尚,投资理财,教育就业
娱乐
美食
文艺
旅游,美食
美女,星座命理,文艺
投资理财,美食,健康,旅游,星座命理,游戏动漫
新闻资讯,时尚,美食,旅游,娱乐
娱乐
娛樂,健康,旅遊,文藝
投资理财,教育就业,80 后
视频音乐
汽车,星座命理,文艺
体育
微博奇葩,美食,萌宠,旅游,新闻趣事
彩妆
搞笑幽默,旅游
时尚,旅游,美食,文艺,90 后,学生,水瓶座,紫色
旅游,学生,宅女
投资理财
旅游
娱乐,美食
旅游,美女,美食,体育,IT 数码
文艺
美食
美食,健康,娱乐
美食
星座命理
旅游,星座命理,美女,时尚,娱乐
旅行,旅游,体育
IT 数码
旅游
美食,健康

音乐,偶喜欢玩,美食,睡觉
军事
军事
教师兼家庭主妇
健康,旅游,星座命理,美食,文艺
旅游,娱乐,文艺,汽车,IT 数码
巨蟹座,艺术,书籍,画画,好书,文学
游戏动漫,动漫,音乐,90 后,吃,游戏,上网,宅女,双鱼座
微博奇葩,新闻趣事,旅游,美食,萌宠
健康
美女
投资理财,美女,IT 数码,体育,娱乐
逛街,美容,穿衣美容,搞笑幽默,美食,广东生活
汽车,军事,IT 数码
视频音乐,听歌
教育就业,体育
IT 数码,教育就业,前台,美女,平常心,学生,街拍
游戏动漫,投资理财
美食,IT 数码
IT 数码
星座命理
狮子座,搞笑,交友,自由,唱歌
80 后,网购,自由职业,处女座,平常心,摄影,创业,时尚
投资理财
游戏动漫,娱乐,汽车
语录,平常心,交友,乐观,自由,游泳,美食,经济,唱歌,旅行
美女
IT 数码
萌宠,水瓶座,宅女,摄影,动漫,旅行,电影,自由,宅,80 后
iphone,iPhone,iphone4,三星手机,三星,手机控,手机党,手机达人,手机游戏,手机
护肤,美女,印度代购,芙若森修,精油,美容
左撇子,宅女一枚,喜欢音乐
星座命理
音乐,羽毛球,盘后票,盘中票,个人股,金融,内线,股票,A 股,上网
搞笑幽默
上海生活,育儿百科,搞笑幽默,新闻趣事
上海生活,星座命理
时尚,旅游
美女,美食,军事,体育,娱乐
天津生活
旅游,搞笑幽默,视频音乐,新闻资讯,教育就业,美女,娱乐,IT 数码
美女,娱乐,IT 数码
IT 数码,体育
体育

娱乐,健康,旅游,美食,体育,做饭,读书,听音乐,运动,美剧
美食
淘宝控,团购,淘宝,朋友,网络营销,健康,赚钱,网购,育儿,微博
旅游,美食,汽车,IT 数码
美女,军事,IT 数码,体育
体育
投资理财,教育就业,汽车,美女,旅游
摩羯座
娱乐
文艺,游戏动漫,新闻资讯,体育,娱乐,发呆 ing,阿岳
旅游
游戏动漫,美女,美食,娱乐,IT 数码
体育,运动,平常心,创业,80 后
汽车
汽车
文艺,美食,游戏动漫
文艺,体育,娱乐
名人明星
娱乐
美图摄影
体育
新闻资讯,投资理财,娱乐,IT 数码,旅游
健康
军事,IT 数码,体育,投资理财,汽车
微博奇葩
美食
健康,旅游,教育就业,IT 数码
电影,魔兽世界,80 后
酒类营销,斯洛克,旅行,80 后,足球,自由,快乐,音乐,阳光
古風中毒,瓶邪,腐宅双修,人不猥琐枉少年,三次元交流障碍
旅游,文艺
金融小头目
IT 数码
时尚,典型双子女,80 后宅妈,80 后少妇,80 后熟女,80 后女生
娱乐
体育,军事,汽车
投资理财,IT 数码,游戏动漫,军事,体育
投资理财,健康,新闻资讯,时尚,教育就业,读书,英语,音乐,电影,旅游
SailorMoon
游戏动漫,体育
geeky,游戏宅,远离热门话题,邪恶青年,双层吉士汉堡,怀旧,流行朋克,远离政治,熬夜冠军,自由至上
美女
文艺,电影,娱乐,美食,音乐

旅游
星座命理
文艺,健康,IT 数码,教育就业,新闻资讯
视频音乐,体育
旅游
星座命理
旅游
健康
旅游
文艺,旅游,投资理财
郑州,时尚,娱乐,IT 数码,周杰伦,汽车,摄影
校园生活
健康,军事,IT 数码,投资理财,体育
娱乐
财经资讯,孤独,时而疯癫,时而沉默,漫画控
IT 数码
细路女
旅游,司琴,时尚,音乐,80 后,旅行
体育
日语学习中,玩儿,吃货,80 后,射手座,帅哥美女控
旅游,军事,IT 数码,体育
投资理财
军事,IT 数码
时尚,健康,美食,旅游,文艺
时尚,星座命理,美食,IT 数码,娱乐
美女
星座命理,旅游
娱乐,美食,听歌
上网,我爱 UC 浏览器
健康
新闻资讯,IT 数码,汽车
旅行,90 后
铜佛,珐琅彩,日本铜器,康熙五彩,粉彩瓷器,青花瓷,黄花梨圆背交椅,沈阳古玩城,瓷器,古玩
游戏
体育
时尚
ACG,cosplay,猫,仓鼠,galgame,游戏,动画,漫画
新闻资讯,IT 数码,文艺
文艺,体育,娱乐
美食
体育
旅游
健康

美女,娱乐
我爱 UC 浏览器
我爱 UC 浏览器,电影,音乐,听歌,80 后
听歌,天蝎座,大学生,旅游
西门子,工控设备,PLC,天津,变频,一级代理
游戏动漫
娱乐,美食,宅女,善良,生活,交友,自由
新闻资讯
NBA,射手座,自由,动漫,学生,音乐
汽车,我爱 UC 浏览器,电影,听歌,时尚,90 后,音乐
互联网,营销,网购,电子商务,创业,囧,小说,运动,艺术,音乐
搞笑幽默
旅游,教師,基督徒,80 后宅女,A 型血,金牛,热爱生活
汉中生活
旅游,搞笑幽默,美女
美女
娱樂
星座命理,IT 数码,娱乐
投资理财,文艺,旅游
游戏动漫,旅游,IT 数码
可爱,真城,敏感
微博奇葩
娱乐
美食
健康
娱乐
山东生活,新闻趣事,善良,音乐,读书,语录,平常心,旅行,80 后,股票
军事
星座命理,微博奇葩
音乐控,狮子座
穿衣美容,微博奇葩,搞笑幽默,新闻趣事,视频音乐
西安生活
湖南生活,爱杨宵,爱乔振宇,爱张杰
文艺,美食,旅游
美食
创业,电影,网购,80 后,上网,白羊座,玩儿,八卦,leicester
IT 数码
减肥,逛街
交友,娱乐,美食,旅行,电影,音乐
教育就业,书法,艺术,文字,宅,读书,天秤座,大学生,80 后,文学
自由,学生,教育就业,IT 数码
爱玩的可怜男孩
新闻资讯
美食,更加积极向上,生活,英语,NBA,足球,自由,看书,听歌,80 后

健康,投资理财,旅游,星座命理,美食
娱乐,时尚,体育
美女,佛山生活,旅游
美食
时尚,听歌,偶喜欢玩,娱乐,美食,旅游
老广,￥钱钱钱￥,爱运动但不想动,讨厌听你讲大话,球星,爱广州,轻浮,小气鬼,旅行,电影
游戏动漫
星座命理
体育
星座命理
健康,旅游,时尚,教育就业,汽车
视频音乐
小说,喜欢交际,白色黑色紫色,玩儿,数学爱好,电影爱好者,动漫,开朗乐观,校园,双子座
娱乐
新闻资讯,IT 数码,汽车,美女,体育
游戏动漫,美女,健康,美食,IT 数码
体育
美食
名人明星
二货走起
旅游
时尚,汽车,美食,旅游
健康,美食,IT 数码,文艺,教育就业,边散步边爬山
白羊座,自由,90 后
汽车,旅游,IT 数码,体育,娱乐
芝加哥,巴塞罗那,尤文图斯,煎饼果子,爱 KG
健康,美女,娱乐
旅游
美食
美女,IT 数码,数码,看书,交友,汽车,IT,摩羯座,90 后,加油思索者
美女
纪中摄影协会用,纪中街拍
动漫,奋斗,游戏动漫,娱乐
体育资讯
星座命理
微博奇葩,美女,旅游,美食,新闻趣事
美女
教育就業,电影,学生,音乐
旅游,美男,设计,远走他方,狗猫控,神经质
上海生活
新闻资讯,娱乐,文艺,游戏动漫,星座命理
体育资讯,微博评论,做自己,马斯洛,中国梦
新闻资讯,游戏动漫,汽车,IT 数码
文艺

时尚,投资理财,旅游,军事,美食
新闻趣事
看书,动漫,迟钝,科幻,想象,懒惰,无聊,迷茫
美容服饰
星座运势
穿衣美容
anime 的一切,玖兰枢 sama,星座命理,ACG
SUJUonly13,BIGBANG
文艺
旅游
时尚
学生一枚,执着,电影迷,冷笑话,幻想,爱玩,文学,爱情,微博,美剧
时尚,健康,旅游,美食,投资理财,娱乐,微博,紫色,旅行
文艺,时尚,健康,星座命理,美食
自由,羽毛球,新闻,善良,生活,唱歌,音乐
广州,猫控,方大同,吉他,我就是我,music,摄影爱好者,文学,阅读,街拍
名人明星,爱自由
打鱼种菜。。。
IT 数码
搞笑幽默,新闻趣事
娱乐,游戏动漫
美食
八卦杂谈,新闻趣事,名人明星,西安生活,校园生活,娱乐
美食
旅游
旅游
IT 数码,军事
喜欢听歌,IT 數碼,旅游,微博奇葩,搞笑幽默
旅游,怀着学习的心态,窥览世界精彩
教育就业,娱乐,美食,IT 数码,投资理财
音乐迷,摄影爱好者,NBA
健康,旅游
IT 数码
体育
教育就业
美女
娱乐,美食,旅游,时尚,文艺
旅游,文艺
美食,视频音乐,新闻趣事,旅游
健康,文艺,娱乐
美食
旅游,新闻趣事
IT 数码
心理,K 歌,美食控,美食,听歌,天蝎女,傻,矛盾的存在！,人后抑郁,人前开朗

IT 数码,小说,80 后,电影,时评,IT 互联网,科技,媒体,财经,新闻
蓝花,夏陆,瓶邪,李赫宰,银赫,SpencerLee,非典型狮子女
汽车,游戏动漫,旅游,美食,文艺
娱乐,80 后
开朗豁达,80 后,金融
二维码
娱乐
游戏动漫
娱乐,教育就业
体育,美食,旅游,娱乐,时尚,军事
旅游
娱乐
旅游,美食,微博奇葩,搞笑幽默,杭州生活
微博奇葩
视频音乐
艺术,历史,互联网,游戏,旅游
投资理财
汽车,宅男,旅游,美食,文艺,投资理财
校园生活,爱,看海,登山,思考各种哲学,笑哈哈,运动更快乐,羽毛球,瑜伽,唱歌
名人明星
新闻资讯,星座命理,军事,IT 数码,娱乐
名人明星,娱乐
健康,旅游,美食,IT 数码
音乐,原创,教师,吉他,贝斯,电声乐队,萨克斯,声乐
旅游,运动,车,音乐,电影,红酒,香水
军事,IT 数码,连接服务器失败
新闻资讯,美女,投资理财,旅游,IT 数码
书法,服装设计,南京,喜欢听歌,天秤座,看电视,明星,旅游美食,历史,小说
汽车,健康,旅游,IT 数码,美女,军事
90 后
文艺,游戏动漫
一些事一些情,生活,娱乐,时尚,旅游,音乐
投资理财,美食,新闻资讯,旅游,文艺
汽车,投资理财,美食,IT 数码,体育,天蝎,爱睡觉,孩子气,寂寞
时尚,汽车,教育就业
教育就业,游戏动漫
美食
文艺,旅游,来看朋友,咖啡,数学,旅遊,乒乓球
网购
搞笑幽默
旅游
四川生活
文艺,追忆逝水流年
旅游,星座命理

美食,旅行,电影
搞笑幽默
健康,旅游,美食
听歌
听歌,吃,电影,大学生
上海生活,旅游爱好者,音乐爱好者,幸福,完美主义
穿衣美容
深圳生活
美女
新闻资讯,投资理财,健康,旅游,美食
剑网三,动漫
体育,旅游,汽车
健康,时尚,星座命理,美食,文艺,文学,90后,好书,白羊座,唱歌
英语,看书,创业……,旅游,电影,美食,旅行,时尚,80后,音乐
开朗乐观,爱交友,90后,巨蟹座,看书,自由,美食,旅游,学生,最喜欢极限运动
批发,水瓶座,网购,代购
健康
爱拔酱,关八,烤鸟,AKI,二狗子,小66,渣MAO,DOES,SID
美女,教育就业,投资理财,旅游,文艺
新闻趣事,星座命理,搞笑幽默,四川生活
电影,吃货,码字的,天秤座,天蝎座,宅女,旅行,80后,美食
音乐,美剧,社会学
英语,白羊座,紫色,看书,摄影,时尚,电影,音乐,旅行,听歌
文艺,娱乐,美女
美食
游戏动漫,娱乐
汽车,创业,我爱UC浏览器
娱乐
旅游
新闻趣事,八卦杂谈,美容服饰,温州生活,旅游,90后
旅游
娱乐
星座命理,时尚
射手座,幽默,上网,自由,学生,旅行,时尚,90后,电影,听歌
新闻趣事,名人明星,美国生活,旅遊,萌宠
nba,唱k,o型血,自由,听歌,80后,球迷,体育
新闻资讯,听歌,电影,音乐
IT数码,汽车,双截棍
微博奇葩
仙剑奇侠传,菱纱,仙剑迷
旅游
美女,穿衣美容,时尚,游戏动漫
娱乐,汽车,时尚,游戏动漫,美女
微博奇葩

美食
娱乐
IT 数码,娱乐
电影,90 后,画儿画,浮躁,天马行空
健康,时尚,星座命理,星座族,星座配,创业,偶稀饭睡觉觉,动漫,美食,电影
美女,时尚,旅游,健康,娱乐
旅游,新闻资讯,投资理财,老男孩儿,游走的鞋子
搞笑幽默
文艺,体育
文艺,旅游,独自行走,古琴,纳木错,散步,吃,考研,涂鸦,医路漫漫
IT 数码
美食
吃货,范儿,style,韩流,自拍,萌,旅行,网购,时尚,90 后
美食,健康
健康,星座命理,美食,IT 数码,娱乐
懒,爱笑,美食
时尚,旅游,星座命理,美食,文艺
健康,投资理财,旅游,星座命理,美食
美女,时尚,投资理财
宅,慵懒,小女人
娱乐,睡懒觉,柯南,爱吃,90 后但我不脑残,动漫迷
广东生活
投资理财,健康,旅游,游戏动漫,文艺,各种宅,美声,90 后但太淡定,为情所困
微博奇葩
星座运势,做好自己的唯一
健康,星座命理,娱乐,电影爱好者
娱乐
美食,汽车
时尚
微博奇葩
自由,怀旧
巨蟹女,幸福,美食,穿衣美容,宅女,网购,旅游,时尚
旅游,美食,星座命理,上海生活,新闻趣事
军事,新闻资讯,教育就业,投资理财,健康
爱猫,学生一枚,高考,bl,萝莉控,宅女加腐女,游戏动漫
旅游
IT 数码,游戏动漫,体育
体育,文艺,音乐,电影,跆拳道
新闻资讯,健康,时尚,投资理财,军事
新闻趣事
娱乐,体育,游戏动漫
美食,旅游
文艺,投资理财,旅游,娱乐,美食
美食,旅游,健康,文艺

校园生活,新闻趣事,欧美电影,欧美流行音乐,欧美街拍,美剧,时尚
美食
佛山生活
旅游,娱乐
新闻趣事,美食,穿衣美容,搞笑幽默,青岛生活
军事,房产
时尚,旅游
游戏动漫
新闻资讯,文艺,投资理财,IT 数码,健康,旅游
娱乐
IT 数码,购物,上网,时尚,音乐
处女座,唱歌,听歌
爱生活,简单,爱旅游,运动健身控,减肥 ing,ACG 爱好者
娱乐
体育
旅游,娱乐,汽车,时尚
美瞳控
星座命理,美食,娱乐,文艺,旅游,时尚
江苏生活,娱乐,体育
美食,娱乐
健康,文艺,旅游,美食,娱乐
体育,文艺
广州生活
旅游
我爱 UC 浏览器
游戏动漫,娱乐
新闻资讯
汽车,美女,星座命理,美食,IT 数码
时尚,美食,旅游,IT 数码,娱乐
萌宠
搞笑幽默
娛樂
新闻资讯,汽车,旅游,美食
摄影爱好者,设计,旅行,创业,生活,学生,吃货,电影,自由,美食
汽车
美女,技术宅
Rhythm,n,Blues,音乐,音乐教育舞蹈,舞蹈演员
娱乐,网购,天蝎座,唱歌,睡觉,电影,听歌,旅游,时尚,80 后
IT 数码,汽车,美女,旅游,军事
美女,微博奇葩,旅游,名人明星,视频音乐
娱乐,体育
我爱 UC 浏览器
体育
投资理财,文艺,IT 数码,健康,旅游

运动,天蝎座,唱歌,上网,自由,音乐,电影,听歌
新闻资讯
美食,娱乐
文艺,娱乐,美食,游戏动漫
美食,双子座,宅
K6K6
星座命理,美食,IT 数码,教育就业,汽车
汽车,娱乐,旅游
教育就业,娱乐,美食,体育,沛鸿民族中学
美食
新闻资讯,体育
娱乐
创意,自恋,旅游,娱乐
宅,听歌
投资理财,汽车,时尚,娱乐
时尚,教育就业,娱乐,文艺
汽车,阅读,搞笑,水瓶座,摄影,旅行,时尚
汽车,美女,旅游,军事,IT 数码
旅行,巨蟹座,网购,游戏,摄影,唱歌,电影,音乐
处女座,唱歌,动漫,睡觉,自由,时尚,旅行,美食,80 后,电影
体育,娱乐
娱乐,美食,星座命理
名人明星,娱乐
教育就业,旅游,美食,娱乐,投资理财
教育就业,投资理财,美女,健康,星座命理
美女
杭州生活,美食,旅游
娱乐,奋斗青年,天蝎女,微宅
新闻资讯,教育就业,旅游,星座命理,娱乐,爱旅游
投资理财
视频音乐,新闻趣事,微博奇葩,湖北生活,搞笑幽默
历史,自由
音乐,电影,80 后,旅游
时尚
健康,美女,汽车,娱乐,星座命理
电影,摄影爱好者,汽车,英语,唱歌,上海宁爱上海,狮子座,篮球,旅行,美食
HipHop,Breaking,旅游,天秤
美食,星座命理,新闻趣事,搞笑幽默
时尚,星座命理,旅游,新闻资讯,美食
视频音乐
美女,美食,军事,IT 数码
健康,新闻资讯,旅游,星座命理,教育就业
娱乐,星座命理,文艺
美食

娱乐
文艺,旅游,健康,时尚,娱乐
文艺,体育,娱乐
星座命理,不坚强
时尚,宅,旅行,旅游,电影,音乐
旅游
娱乐
穿衣美容,Nichkhun,维尼夫妇,Vbaby,宋茜,GD,Bigbang,TOP,2NE1,希望优雅的活着
kaji 本命 w,声控,唱见,声优,乙女游戏
投资理财
投资理财,美食,星座命理,健康
娱乐,星座命理,文艺,时尚
游戏动漫,星座命理,吃货,动漫,善良,游戏,音乐,偶稀饭睡觉觉,宅,摩羯座
NBA,电影,交朋友,音乐
唱歌,天秤座,美女,足球,体育,旅行,电影
体育,娱乐
旅游,娱乐
游戏动漫
游戏动漫
摩羯座
文艺
旅游
宅女加腐女,平面设计,睡觉睡到自然醒,美食,宅
星座命理
上网,自由,旅行,80 后,音乐,电影,听歌,美食,时尚
娱乐
美食,旅游,名人明星
幸福,音乐控,A 型血,狮子座,旅游
健康,旅游,汽车,星座命理,体育,慎重选择,选择了就坚持
音乐,发呆幻想,随意但不随便,80 后
赚钱,夜猫子,爱旅游,爱电影,男人,北京,玩,美食控,重口味,Music
旅行爱好者,宅女加腐女,拍照,篮球爱好者,时尚,设计师,音乐爱好者,咖啡
游戏动漫,IT 数码
新闻资讯,健康,美食,旅游
美女
视频音乐
coser,瓶邪,腐女
文艺
幽默,喜欢音乐,广播,感性,唱歌,看书,上网,自由,学生,军事
90 后
视频音乐
搞笑,旅游,电影,学生,幽默
体育
时尚,音乐,旅行,宅

健康,美食,新闻资讯,IT 数码,游戏动漫
美食,音乐
健康,美食,旅游
新闻趣事
时尚,旅游,文艺,体育,娱乐
代理,咨询,法律,中小企业,新法信息,诉讼代理,法律顾问,法律咨询
贝贝,处女座,看书,自由,旅游
美女,新闻资讯,汽车,军事,娱乐
游戏动漫,美女,文艺,体育,娱乐
米粉,大米,Apple,乐观,娱乐,时尚,囧,偶喜欢玩,偶稀饭睡觉觉,善良
时尚,乐观,自由,偶稀饭睡觉觉,文学,搞笑,天秤座,吃,旅游,运动
IT 数码,游戏动漫
潮流,汽车,电子,动漫,篮球
微博奇葩
上海生活
美食,文艺,时尚,旅游,娱乐
交朋友,90 后,怀旧,音樂,乐观,生活,狮子座,NBA,文艺,体育
投资理财
健康,新闻资讯,教育就业,投资理财,党员,文字,美食
时尚,旅游,星座命理
汽车,教育就业,投资理财
NY
美食
原石,翡翠,翡翠原石
翻译,电影,旅行,英语,文学,网购,读书,天蝎座,美食
搞笑幽默
旅游,时尚,健康,星座命理,投资理财,自由自在的感觉,美食,80 后,电影,旅行
美食,文艺,健康,旅游,90 后,学生
投资理财,教育就业,IT 数码,军事,新闻资讯
宁波生活
IT 数码
新闻资讯,时尚,娱乐,文艺,IT 数码
美食,教育就业,娱乐
我爱 UC 浏览器
娱乐
新闻资讯
搞笑幽默,萌宠,微博奇葩,电影迷,咖啡,广州
军事,电影,T 边缘人
游戏动漫,星座命理,美女,汽车,体育
孩子气,爱生活,大学生,双鱼座,星座命理,旅游,美食
名人明星,微博奇葩,美女,邓丽君,摄影爱好者,Eric,Clapton,吉他,鼓手,历史
美食
时尚
IT 数码

游戏动漫,健康,星座命理,IT 数码,娱乐
美食,时尚,旅游,星座命理,文艺
汽车,旅游,IT 数码,美食,娱乐
穿衣美容
体育
视频音乐
娱乐
微博奇葩,穿衣美容,视频音乐,新闻趣事,江苏生活
文艺,时尚,汽车,新闻资讯,旅游
教育就业
IT 数码
娱乐
IT 数码,新闻资讯,投资理财
美食,搞笑幽默,穿衣美容,微博奇葩,视频音乐
投资理财,旅游,IT 数码,文艺,体育
旅游,教育就业,投资理财,健康
文艺,新闻资讯,投资理财,教育就业,军事
旅游,爱旅游
天平座,篮球
美食,旅游
微博,文学,健康,旅游,新闻资讯
新闻趣事
美女,萌宠,新闻趣事,旅游,北京生活
军事
数码
无聊之人,军事,IT 数码,体育,汽车,美食
围巾系法,高端围巾,羊毛围巾,凯米尔酷创始人,羊绒围巾,时尚
健康
射手座,学生,美食,80 后,旅行,宅
美食,名人明星,穿衣美容,视频音乐,微博奇葩
搞笑幽默
旅游
旅游,文艺,游戏动漫,美食
内蒙古生活
火锅帝
读书分享,星座运势,狮子座,看书,上网,睡觉,电影,音乐,旅行,80 后
新闻资讯,健康,娱乐,旅游,美食
北京生活,视频音乐,新闻趣事,旅游,名人明星
美女
新闻趣事
美食,时尚,听歌,旅游,80 后,电影
旅行,自由,音乐,听歌
体育资讯
美女,游戏动漫,时尚,体育,娱乐

视频音乐
汽车,时尚,旅游,美食,80 后,电影
古灵精怪,英语,旅游,美食,星座命理,文艺,娱乐
娱乐
美食,军事,IT 数码,体育,娱乐
文艺
名人明星
娱乐,军事,星座命理,旅游,游戏动漫,IT 数码
军事
吉林生活,真实自我,90 后
黄麻岭
汽车,体育
岁月静好,现世安稳,星座命理
娱乐
IT 数码,健康,投资理财,体育
名人明星
旅游摄影
黑白灰,网球,白开水,茶,超爱太阳花,摄影控,围巾控,酒,自由,旅行
美食
酱油王,op,游泳,健身,羽毛球,死飛
健康
美女,发傻,即自我思考
旅游,桌球,篮球,80 后,音乐
搞笑幽默
美食
搞笑
旅游,娱乐
BIGBANG,强迫症,敏感细腻,吃货,五月天
音乐迷,90 后非脑残,时尚,新闻资讯,旅游,美食,IT 数码
17 的努娜,WonWoo,seventeen,全圆佑
起床困难户,学生一族,美食
时尚,旅行,我爱 UC 浏览器
汽车,美女,体育,IT 数码,娱乐
旅游摄影,西式烹饪,美食美酒,书法篆刻,五星级酒店,行政总厨
体育,足球,80 後,上班族
汽车
搞笑幽默,视频音乐,萌宠,星座命理
网络教育招生,成人高考招生,西安交通大学,吉林大学,山东中医药大学,潍坊医学院,潍坊学院,青岛理工大学,天津商业大学
旅行,美食
文艺,娱乐,美女
宜昌,杭州,海口,廉耻心,医疗,装饰装潢
旅游,IT 数码,时尚,健康,美食
80 后,米雪,活泼,吃货一枚

娱乐
星座命理
体育,囧,动漫,音乐发烧友
汽车,美女,健康,娱乐,体育,睡觉,赚钱
电影,美食,音乐
娱乐
旅游
时尚,旅游,美食,IT 数码,娱乐
IT 数码
文艺
新闻资讯
视频音乐,微博奇葩,搞笑幽默
文艺,时尚,游戏动漫,娱乐,仅仅就是萌呦
游戏动漫,美食,娱乐,汽车,旅游
好想做妈妈,超级喜欢小朋友,购物狂,王菲,水瓶座
旅游美食,投资理财
时尚,吃,摄影,自由,旅游,电影,音乐
我爱 UC 浏览器
健康,文艺
娱乐,星座命理,美女,投资理财
新闻资讯,场面
80 后,囧,射手座,听歌,电影,宅女,音乐,吃货,平常心,睡觉
文艺,时尚,旅行,听歌,旅游,音乐,电影
IT 数码,文艺
旅遊
格子控,天蝎女,90 后但我不脑残,维他命,旅游,教育就业,美食,星座命理
投资理财,星座命理,文艺
游戏动漫,美女,IT 数码,汽车,体育
搞笑幽默,广东生活
体育
IT 数码
健康,新闻资讯,娱乐
IT 数码,体育,娱乐,军事
三国杀,许褚日记,桌游
投资理财,旅游,娱乐
文艺
娛樂
电脑,游戏动漫
懒,财经,电子商务,外貌协会,迷糊,旅游美食爱好者,投资理财,娱乐
文艺,美女,旅游
动漫
体育
旅游
旅游美食爱好者,爱生活,快乐,驴友,旅游爱好者,努力

星座命理,健康
美食,健康,游戏动漫,投资理财
新闻资讯
文艺,旅游,健康,时尚,美食
健康,旅游,美食,文艺,娱乐
时尚,星座命理,文艺
健康,旅游,星座命理,美食,文艺
娱乐
娱乐
汽车,文艺,旅游,美食,时尚,娱乐
美女
服饰潮流,时尚,服装
韩寒,张国荣,尼采,80 后,哲学,光棍
文字,电影,小说
星座命理,新闻资讯,时尚,教育就业
旅游
教育就业,艺术,电影
时尚,旅游,娱乐,游戏动漫
旅游,美食,新闻资讯,投资理财,IT 数码
搞笑幽默
听歌,学生
视频音乐
投资理财,美食,军事,汽车,旅游
旅游,游戏动漫,汽车,美食,IT 数码
健康,美女,文艺,IT 数码
娱乐,健康,旅游
美女
美食
音樂,新闻资讯,投资理财,汽车,军事,娱乐
美女
宅,灵异,文字,摄影,美食,旅游
文艺
新闻资讯
浪客
汽车
军事
时尚,汽车,体育,IT 数码,教育就业,健康,旅游
旅游,健康,美女,汽车,娱乐
美女,教育就业,体育
健康,美食,娱乐,IT 数码
数码,重口味,汽车,旅游
IT 数码,投资理财
时尚
IT 数码,文艺,新闻资讯,投资理财,时尚

汽车
游戏动漫
旅游,美女,IT 数码,娱乐,体育
听歌
教育就业,投资理财,军事
星座命理
IT 数码,美食,星座命理,时尚,娱乐
美食
散得像一盘沙
旅游
教育就业,时尚,星座命理,旅游,健康
美女
娱乐,新闻资讯,健康,旅游,美食
健康
shori,佐藤胜利,胡夏
文艺,旅游,军事,健康,时尚,娱乐,美食
投资理财
旅游
IT 数码,游戏动漫
娱乐,健康,投资理财,旅游,美食
健康,旅游
健康,投资理财
体育,新闻资讯,娱乐,健康
美食,微博奇葩,搞笑幽默
新闻趣事,睡觉
海南生活
新闻资讯,健康,旅游,星座命理,娱乐
体育
投资理财,旅游,星座命理,美食,文艺
娱乐
文艺,游戏动漫,旅游,星座命理,美食
喜欢音乐,团购,70 后,餐饮,连锁,培训,采购,销售,渠道
游戏,自由,娱乐,上网,电影,90 后,听歌,时尚,我爱 UC 浏览器,音乐
时尚,娱乐,体育,文艺
旅游
娱乐
游戏动漫,娱乐
汽车,新闻资讯,投资理财,军事,体育
健康,娱乐
IT 数码
娱乐,教育就业,星座命理,旅游,投资理财
体育资讯
新闻资讯
投资理财,军事,健康,新闻资讯,汽车

文艺,健康,旅游,美食,时尚
旅游,睡觉睡到自然醒,小说,摄影
宅
新闻资讯
旅游,游戏动漫,美食
旅游
文艺,体育,旅游,宅,音乐,电影,双鱼座,动漫,摄影,偶稀饭睡觉觉
娱乐
郭敬明,bigbang,双子,美剧控,林峯,文字控,爱猫,欧美,插画
旅游
搞笑幽默
福建生活
音响设备,原色牛仔,美食,旅游
旅游,美食
巨蟹女,半斤八两,旅游
投资理财,新闻资讯
体育
木手机,无偶像,吃货,住宿生,反苏党,腐女,摩羯座,学生,成长 ING
旅游
健康,时尚
健康,美食,IT 数码,新闻资讯
融入舞蹈的精华
美食
福建生活
抑郁症,偏执狂,孤独症
教育就业,新闻资讯,汽车,体育
游戏动漫,唱歌,睡觉,上网,屌丝,动漫,宅
娱乐,芯片,听音乐,唱歌
美女,美食,军事,游戏动漫
军事
美食,游戏动漫,时尚,旅游,新闻资讯
体育,文艺,美食,娱乐
教育就业,健康,星座命理,文艺,体育
新闻趣事
体育
教育就业,军事
体育资讯,旅游
娱乐
投资理财,美食,旅游,健康,娱乐
游戏动漫,美食,爱 SNSD,爱各类游戏
时尚,教育就业,娱乐
旅游
搞笑幽默
视频音乐,旅游,美食,星座命理

美食,时尚,旅游,文艺,娱乐
法律,教育就业,人力资源,社会,新闻资讯
体育
美食,湖北生活
健康,新闻资讯
车世界,军事天地,名人明星,星海中学,11,4 班
美女,军事
健康
IT 数码
美女,娱乐,军人
新闻趣事,武汉生活
美食
汽车,旅游,健康,美食,娱乐
IT,射手座
想我所梦,爱我所爱,电影,钢琴,小清新,在路上,巨蟹座,90 后,音乐控
哈哈,咖啡控,医学,星星,名人明星
时尚,健康,旅游,美食,娱乐,love,khun
投资理财
NBA,羽毛球,微博,偶喜欢玩,爱情,文艺,娱乐,教育就业,时尚,美女
名人明星,美女,穿衣美容
学生,摩羯座
健康,新闻资讯,IT 数码,时尚,教育就业
体育
娱乐,旅游,美食
搞笑幽默,萌宠,微博奇葩,旅游,视频音乐
爱美食,sj,迷糊,追星
旅游
渣渣,码字的,死宅
旅游
新闻资讯,旅游,体育
爱情,旅游
海贼王死粉,名人明星,微博奇葩,视频音乐,新闻趣事,搞笑幽默
睡觉,浪漫,狮子座,传媒,唱歌,旅行,乐观,吃货,听歌,运动
时尚
健康,旅游,娱乐,军事,IT 数码
健康,投资理财,汽车,旅游,美食
旅游,美食
健康,美食
IT 数码,体育
健康,音乐,电影,时尚,听歌,90 后
投资理财
军事
美女,娱乐
游戏动漫

投资理财
音乐,电影,听歌,80 后,90 后,美食,旅游,旅行,时尚,宅
体育
美食
文艺
美食
娱乐,教育就业,体育
军事,IT 数码,文艺,体育,娱乐
心理学,巨蟹座,爱情
名人明星,视频音乐,微博奇葩,美食,萌宠
文艺,教育就业,美食,旅游
娱乐
微博奇葩,守望者,睡觉哥,互联网,手机,读书
健康,旅游,美食,时尚
文艺,旅游,星座命理,美食,体育
星座命理,美食,旅游,健康,投资理财
旅游,新闻趣事
IT 数码
美食
视频音乐,美食,旅游,星座命理,广东生活
教育就业,星座命理,IT 数码
汽车,IT 数码,旅游
娱乐,旅游,健康,美食
时尚
体育,时尚,健康,美女
双鱼座,时尚,巨蟹座,微博,网购
开朗,90 后,运动,大学
娱乐
美女,休闲,闷骚,网络,单身,随性,蓝色,学生一族,喜欢音乐,O 型血
投资理财,教育就业,体育,汽车,军事
健康,旅游,星座命理,美食,宅人,搞笑,小说,逛淘宝,大学,工作狂
星座命理,娱乐
美女,体育,汽车
汽车,美女,美食,新闻资讯
情感生活
新闻资讯,健康,娱乐,时尚,文艺
健康,游戏动漫,美女,文艺,娱乐
娱乐,旅游,投资理财,汽车,IT 数码
好性格
文字,网购,摩羯座,巨蟹座
健康
美女,名人明星,新闻趣事,搞笑幽默,微博奇葩
搞笑幽默
教育就业,IT 数码,体育,汽车,NBA 迷

名人明星,穿衣美容,星座命理,美食,搞笑幽默
时尚,美食,旅游,星座命理,健康
娱乐
时尚,美食,旅游,听歌,音乐
文艺,旅游,IT 数码
美食
娱乐
网购,微博,浪漫,学生
星座命理,游戏动漫,水瓶座,小说,学生
文艺,美食,IT 数码,娱乐
电子商务,美食,睡觉,唱歌,90 后,电影,好性格,玩儿,设计,自由
摩羯座,音乐,网购,微博,浪漫
射手座,艺术,睡觉,上网,看书,宅,旅游,音乐
美食
美食,投资理财,旅游,娱乐,军事
汽车,IT 数码
新闻资讯,旅游,美食,军事,娱乐,因为喜欢而牵挂
文艺,旅游,娱乐,星座命理
美女
IT 数码
美食
军事
健康,娱乐,新闻资讯,教育就业,美食
星座命理,美食,娱乐
旅游,娱乐
旅游,娱乐,美食
IT 数码,娱乐,文艺,双子座,汽车,睡觉,听歌,大学,小说,90 后
情绪化,偏执狂
文艺
星座命理
健康,看书,海贼王
triphop
交友,我爱 UC 浏览器
时尚,健康,美食
美女
新闻趣事
体育,娱乐
和动听的歌曲,喜欢伤心的文字
健康,时尚,美女
科学探索,旅游,读书分享,校园生活,新闻趣事
搞笑幽默
河南生活,娱乐
健康,新闻资讯,星座命理
新闻资讯,健康,美食,娱乐,体育

IT 数码
时尚,旅游,健康,星座命理,娱乐
时尚,投资理财,健康,汽车,体育
军事
流星雨,雨天,峰幂,八爷党,微博,双子座,偶稀饭睡觉觉,上网,宅女,90 后
旅游
时尚,旅游,星座命理,美食,娱乐
搞笑幽默,美食,女人,美食控
文艺
新闻趣事,星座命理,旅游,美食,我是 90 后…,我爱生活
时尚
娱乐,IT 数码,健康,新闻资讯
旅游,美食,文艺,娱乐
新闻资讯
旅游,美食,新闻资讯,IT 数码,投资理财,教育就业,文艺
自然敬畏者,理想主义者,二次元脑残粉,爱游戏,爱画画但不专业
投资理财
珠海一中,广外,篮球
睡懒觉,娱乐,游戏动漫,军事,IT 数码
旅游,健康,星座命理,新闻资讯,文艺
旅游,化妆品
美食,睡觉睡到自然醒,双重性格,爱音乐,爱旅游
IT 数码
搞笑幽默,美女,穿衣美容,美食
汽车
Darkwave,80s,Christian
娱樂,军事,娱乐,美女,汽车,志愿者
IT 数码,娱乐
时尚
投资理财
时尚
美食,湖南生活
时尚,新闻资讯,美食,星座命理,娱乐
新闻资讯
美女,好动，喜欢自由!
IT 数码,骚年
文艺,美女,游戏动漫,体育,娱乐
文艺,旅游,星座命理,美食
80 后的尾巴,玩儿,文字,欧美音乐,美食爱好者,时尚达人,潮人,无聊,怀旧,睡觉
紫色,广州,幽默,动漫,学生,双子,歌手,喜欢唱歌,90 后但我不脑残,喜欢听歌
健康,美食,文艺,娱乐,时尚,阅读,艺术
橘子
视频音乐
常州生活

教育就业
体育
汽车
旅行,纸质书,陶瓷,玻璃,设计,懒人一枚,该死的水瓶双鱼,猫科动物
旅行
娱乐
旅游,感性,低调,幸福,完美主义
摩羯座,音乐,学生
新闻趣事,陈翔,娜米,快乐大本营,谢娜,星星,张杰,巨蟹女
投资理财,汽车,IT 数码,旅游,美食
旅游
健康,投资理财,时尚,娱乐
娱乐,投资理财
美食,文艺,体育,娱乐
教育就业,美食,文艺,语录,手机,浪漫,好性格,善良,乐观,睡觉
体育
旅游
娱乐
体育
名人明星
新闻资讯,健康,美食,旅游,军事
美食,北京生活,微博奇葩,搞笑幽默,旅游,体育,娱乐,军事,新闻资讯
健康,时尚,文艺
海外生活,细节的地方,萌,東方 Project,OTAKU,11 区,Nico,JPOP,御宅,二次元
投资理财,娱乐,美食,时尚,文艺
苦逼二货,汽车,旅游,文艺,体育
善良,生活,汽车
旅游,军事,体育
厦门生活,80 后
新闻资讯,健康
名人明星
画画,化妆
時尚,娛樂,健康,旅遊,美食,期待完美爱情,双鱼座,瘦腿控,爱美丽也爱美食,好运气
美食,宠物图集,情感生活,名人明星,搞笑幽默
旅游,美食
娱乐
情人节,央视,柯南,机票,梧桐树,肯德基,台湾馆,减肥,点球大战,世界杯
健康,旅游
娱乐
健康,旅游,体育
乐观,睡觉,电影
时尚,娱乐
听歌,美食,旅游,电影
名人明星

k 歌,娱乐,90 后,听歌,天蝎座,唱歌,音乐,时尚,好性格
美女,旅游,美食,90 后,听歌
旅游,文艺,小说迷,音樂,有点懒懒的,爱睡觉,美食旅行,白羊座
星座命理,文艺,体育,娱乐,投资理财,美女
旅行,美食,电影,听歌,80 后,音乐
美食爱好者,淡定,热爱生活,品牌,偶像剧,宝宝,新闻趣事
时尚,美女,娱乐,游戏动漫
旅游,微博奇葩,美食,新闻趣事
美食爱好者,梅西,淘宝,感性
文艺,汽车,教育就业,体育
美国生活,投资理财
娱乐
腐,80 后的尾巴,神经大条,爱吃,睡觉睡到自然醒,睡懒觉,读书分享,视频音乐,美食,搞笑幽默
文艺
健康,军事,体育,娱乐
刘亦菲,SHINee,少女时代,维尼夫妇,尼坤,红薯夫妇,Cnblue,王力宏,郑容和,贾斯丁比伯
娱乐
我爱柯南,青春就是想象
旅游,IT 数码,文艺,体育
IT 数码,旅游
文艺,疯
体育
耽美,设计,双子座,淘宝控,愤青,懒人
娱乐
美食
体育,美食,刺身,寿司
商业,快乐,爱吃,玩
旅游,星座命理,美食
旅游,IT 数码,游戏动漫
调律,穆里尼奥,倾城之约,搞笑
IT 数码,游戏动漫
体育
游戏动漫
美女
娱乐,IT 数码,旅游
汽车,文艺,美食,旅游,娱乐
美食,娱乐
娱乐,投资理财,旅游,喜欢旅游,爬山,潮流,时尚,旅行,西安,登山
游戏动漫
包子控,恒星,张杰的粉丝,追星
旅游
新闻资讯,游戏动漫,体育,IT 数码,文艺
游戏动漫,美女,文艺,IT 数码,娱乐,双鱼座,宫崎骏

旅游
旅游
娱乐
旅游,体育,娱乐,投资理财
美食,广西生活,微博奇葩,搞笑幽默,萌宠
美食,游戏动漫,美女,汽车,军事
教育就业,娱乐
旅游,双子座,梦想,猫,英语,文字,随性,夜猫子,星座命理,文艺
搞笑幽默
吃喝玩乐,旅游美食爱好者,学生一族,健康
IT 数码
我爱学习,哈哈,旅游
腐女,星座,看电影,发呆,健康,IT 数码
美食,星座命理,旅游,体育,不会唱歌的歌神,宅
新闻趣事
逛街,爱睡觉,星座命理
娱乐
娱乐
娱乐,美食,旅游
汽车,美食,IT 数码,体育,娱乐
爱玩,旅游美食爱好者,疯子,双子女,完美主义,爱音乐,努力,90 后,文字,小说
时尚,IT 数码,旅游,美食,文艺
美食,星座命理,游戏动漫
音乐爱好者,历史,旅遊,篮球爱好者,80 後
投资理财,教育就业,汽车,IT 数码,文艺
新闻资讯,时尚,汽车,投资理财,旅游
搞笑幽默
美女,旅游,IT 数码,娱乐,80 后,听伤心情歌
时尚
时尚,美女,体育
文艺
美食,军事,游戏动漫,旅游,电影,听歌,音乐,旅行
教育就业,美食,游戏动漫,新闻资讯,娱乐
美女
音乐,电影,听歌,80 后,90 后,美食,旅游,旅行,时尚,宅
娱乐
美女,微博奇葩
汽车,旅游,美食,体育,娱乐
美女,文艺
时尚,美食,星座命理,投资理财,军事,体育
教育就业
IT 民工
IT 数码,教育就业,文艺,娱乐,健康
星座命理

公益,帅哥,shopping,我就是我,曼联
投资理财,时尚,旅游,健康,娱乐
萌宠
美食
旅游
音乐,巨蟹座,文字,微博,时尚,浪漫
旅游,星座命理,美食,军事,IT 数码
健康,时尚,投资理财,旅游,星座命理
文艺,体育,娱乐
星座命理
娱乐
我爱 UC 浏览器
旅游,文艺,娱乐,投资理财
美食,健康,旅游,新闻资讯
搞笑幽默,欺负老公,月光族,爱睡,爱偷懒
旅游,美食,IT 数码,娱乐
娱乐
娱乐
玩
汽车,健康,旅游,80 后,时尚,旅行,美食,音乐
文艺,体育,娱乐,汽车,投资理财
游戏动漫,旅游,星座命理,军事
美食
时尚,健康,旅游,旅行,80 后,天蝎座,睡觉,美食,羽毛球,吃货
健康,娱乐,旅游,美食,文艺
娱乐
IT 数码,投资理财,新闻资讯,文艺,娱乐
低调,90 後,体育
文艺
健康,旅游
美食
旅游,健康,美食
娱乐,旅游
微博奇葩
旅游
汽车,文艺,投资理财,美食,旅游
汽车,IT 数码
80 后,电影,听歌,旅行,美食
健康,IT 数码,文艺,新闻资讯,时尚
搞笑幽默,视频音乐,美女
旅游,微博奇葩,美食,新闻趣事
娱乐,旅游
星座命理,娱乐,IT 数码,文艺,游戏动漫
旅游,汽车,IT 数码,军事,娱乐

投资理财,教育就业,时尚,健康,小麦基因组,运动,学生,旅游,80 后,听歌
美食
名人明星,旅游,IT 数码,萝莉控
娱乐,星座命理,游戏动漫,时尚,IT 数码
旅游
娱乐
娱乐
体育,娱乐
美食
时尚,90 后,电影,音乐
体育资讯,校园生活
体育,健康,娱乐,新闻资讯,游戏动漫,文艺
时尚
美女,IT 数码
游戏动漫,新闻资讯,时尚,旅游,美食,文艺
娱乐,IT 数码
健康,新闻资讯,旅游,军事,学生,90 后
IT 数码
IT 数码,教育就业
时尚,旅游,美食,IT 数码
投资理财
健康,投资理财,文艺,娱乐,时尚
娱乐
旅游,可攻可受,二货,缺爱 ing,90 后,瓶邪控,努力 ing
体育
IT 数码
汽车,投资理财,IT 数码,旅游,健康,军事
游戏动漫,旅游,军事,娱乐
IT 数码
娱乐
体育,低调
旅游
美食,健康,旅游
穿衣美容,搞笑幽默
文艺
单身,阳光,爱睡觉
娱乐
娱乐,体育
IT 数码
蛋散
时尚,健康,美食,娱乐
搞笑,唱歌,媒体,数码,双鱼座,无聊就听歌
娱乐
美食,旅游,搞笑幽默

双重
美容服饰,美食,莆田生活,动漫
投资理财
娱乐,IT 数码
反政府,手机看小说,宅男,游戏,小说,睡觉,生活,听歌,看书,互联网
旅游,健康,新闻资讯,时尚,文艺
搞笑幽默,娱乐
哈哈哈哈
美食,星座命理,旅游
搞笑幽默
看电影,看小说,英语,阅读
新闻资讯,美食,教育就业,娱乐,文艺
旅游
动漫,幽默,看书,听歌,美食,自由,宅,90 后,电影,音乐
旅游
新闻资讯,旅游,美食,IT 数码,体育
旅游摄影,90 后但我不脑残
娱乐
星座命理,健康
IT 数码
星座命理,健康
娱乐
我爱 UC 浏览器
猫咪,旅游,宅,电影,音乐
体育,文艺,健康,汽车,IT 数码
新闻资讯
新闻资讯,美女,IT 数码
笑,拍照,怀旧
娱乐,文艺,美食,IT 数码
旅游
文艺,吃,校园,大学,金牛座
腐,双重性格,盗墓笔记,平常心,天平座,娱乐,投资理财,IT 数码,美食,游戏动漫
技术宅,单片机,体育,创业,90 后,篮球
时尚,文艺,星座命理,旅游,健康
体育,读书,建筑,交友,吉他
健康,视频,娱乐,电视剧,电影,风行
投资理财,健康,旅游,星座命理,美食
军事,文艺,健康,星座命理,旅游
IT 数码
汽车
星座命理,旅游,美食,娱乐,美女
我爱 UC 浏览器
美女
新闻资讯,汽车,军事

健康,旅游,文艺,娱乐
美食
健康,旅游,时尚,美食
游戏动漫,投资理财,娱乐
娱乐,80后,听歌
健康,游戏动漫,教育就业,投资理财,IT数码
健康,旅游
体育
音樂,麦霸,wow,b型血,篮球,80後,水瓶
健康,90后
新闻资讯,旅游,IT数码
投资理财,健康,旅游,文艺,娱乐
音乐,美食,文艺,体育
美食,旅游
新闻资讯
美女,体育,娱乐
汽车,娱乐,健康,文艺,IT数码
投资理财
娱乐
汽车,文艺,健康,旅游,游戏动漫,自我放逐
军事,汽车,IT数码,体育
旅游,星座命理,健康,游戏动漫,教育就业
鲁能泰山球迷,红酒达人,运动,营销,时尚,足球
健康,教育就业,时尚,新闻资讯,美食
时尚,军事,体育
健康,时尚
IT数码,美食,美女
投资理财
美女
旅游,美食
星座命理,会计
美女
健康,旅游,星座命理,美食,娱乐,hellokitty
中二,bilibili,ACFUN,东方
星座命理,美食,穿衣美容,新闻趣事,搞笑幽默
旅游,星座命理
搞笑幽默,睡觉睡到自然醒,喜欢旅游,低调
娱乐,文艺
教育就业,新闻资讯,文艺,旅游,怀疑论,理想主义,总想重新出发
穿衣美容
上海生活,新闻资讯
海贼王,鱼迷,大爱欧美风,看书,动漫,时尚,旅游,美食,电影,音乐
驴友,吃货,大学生,摄影爱好者,在路上,看书,旅行,写作,偶稀饭睡觉觉,西藏
时尚,旅游,健康,美食,文艺

我爱 UC 浏览器
娱乐,电影,囧,美食,旅行,听歌,水瓶座,80 后,爱情,吃
笑,暮光之城
旅游,偶像剧,开朗,乐观,搞笑,吃货,天秤座,美食
宅女加腐女,文化
名人明星,穿衣美容,视频音乐,萌宠,美女
游戏动漫,银魂控,历史宅,巨蟹座
善良,脾气暴,爱幻想,超敏感,小清新,无厘头,爱旅行爱生活
旅游,IT 数码
新闻资讯,娱乐
疯狂,运动,小说,音乐
美食
娱乐,美食,时尚
美国生活,星座命理,旅游,娱乐,文艺,时尚
美女
时尚,旅行,90 后,美食,旅游,电影,听歌,音乐
汽车,旅游
旅游
美女,汽车,投资理财,IT 数码,娱乐,体育
微博奇葩
iPhone,美国,留学,学生,穷人,海外,挣钱,机会,iPod,英语
旅游,IT 数码,军事
文艺,时尚,美食,娱乐,阳光男孩,幸福
IT 数码
IT 數碼,新聞資訊,旅遊,文藝
时尚,旅游,健康,新闻资讯,娱乐,文艺
喜欢旅游,开朗,星座,看电影,爱音乐,独立,火影,娱乐
美食,汽车,文艺,体育,娱乐
星座命理
旅游
体育,IT 数码,汽车
穿衣美容,娱乐
游戏动漫,学生一枚,耽美
IT 数码
美食,搞笑幽默
文艺
搞笑幽默,健康
要学术,要减肥,求雨,求实习
娱乐
时尚,美食,IT 数码,文艺,娱乐
星座命理
健康,美食,星座命理,文艺,娱乐
美食,旅游,星座命理
微博奇葩

文艺
旅游,90 后
微博奇葩,名人明星,新闻趣事,搞笑幽默,陕西生活,幽默
投资理财,汽车,新闻资讯,旅游,体育
陕西生活
旅游
娱乐
美图摄影
健康,旅游,新闻资讯,美食,投资理财
向前看
医学统计,疾病预防,新闻趣事,旅游,美食,搞笑幽默,星座命理
听歌,微博,时尚,音乐
微博奇葩
新闻,工薪阶层
新闻趣事
吃货,偶喜欢玩,80 后,网购
军事
追求自由和快乐
旅游
美食
文艺,健康,旅游,星座命理,时尚
娱乐
健康,游戏动漫,IT 数码,娱乐,美女
健康,美食
动动,足球,羽毛球,篮球,跑步,幻稚对成熟,还有…
IT 数码,美女
军事,旅游,读书,善良,财经,NBA,英语
美食,视频音乐
新闻资讯,美食,军事,娱乐
旅游,新闻资讯,娱乐
投资理财,文艺,军事,健康,游戏动漫
IT 数码
贵阳生活
时尚,美食
游戏动漫,健康,旅游,娱乐,体育
伸张正义,善解人意,做事认真,待人真诚,积极乐观向上,善良心软,文字控,水瓶,跳舞,听音乐
美女,诗词,文化,教育,学习,奋斗,善良,艺术,生活,大学生
IT 数码,新闻资讯
军事,投资理财,新闻资讯,健康
美女
汽车,体育
游戏动漫
游戏动漫

自由,交友,乐观,学佛
nba 爱好者
投资理财,健康,旅游,美食,娱乐
星座命理,娱乐,美女
文艺,美女
美食,娱乐,IT 数码
健康,汽车,教育就业,文艺,旅游
美食,IT 数码,文艺,体育,娱乐
游戏动漫,军事,娱乐,体育
双子座,外柔内刚
教育就业,美食,健康,旅游,时尚
人力资源,单纯,新闻资讯
旅游
旅游
娱乐
游戏动漫,娱乐,星座命理
微笑,投资理财,旅游,IT 数码,健康,新闻资讯,星座命理,体育
穿衣美容,名人明星,搞笑幽默
羽毛球,校园,学生,90 后,处女座,微博,交友,动漫,听歌,小说
旅游美食,电影控,吃货,我就是我,旅游美食爱好者,电影爱好者,随性,写字
健康,旅游,美食,IT 数码,文艺,爱好？吃苹果！
美女,听歌,90 后,龍崗中專
时尚
军事
文艺,音乐,文学
新闻趣事,星座运势,旅游,穿衣美容,星座命理,视频音乐,搞笑幽默
体育
美食
淄博生活,杂志,思考,阅读
时尚
健康
体育,游戏动漫
时尚
懒人,热爱生活,旅游,水瓶座,90
健康,旅游
旅游,军事,IT 数码,体育,娱乐
怀旧,睡懒觉,猫咪控,服饰
陕西生活
美食,文艺
娱乐
娱乐,文艺,健康,旅游,美食
健康,旅游,美食,时尚
幸运的射手座,偶稀饭看书,偶稀饭睡觉觉,自由,90 后
星座命理

笑,看小说,家居,盗墓笔记,神经质,爱旅游,爱电影,迷糊,天秤
低调低调
投资理财
体育
旅游,微博
时尚,健康,美食,旅游
看书,新闻,旅行,微博,交友,天秤座,语录,财经,广告
美食,听歌,电影,宅,音乐
月光,房奴,80 后
美图摄影,名人明星,美食,星座运势
穿衣美容,健康
文藝
回报,奋斗,努力,创业,为目标奋斗,不甘人后,自由
军事,旅游
IT 数码
美女
星座命理,自由,80 后,玩儿,网络,囧,足球
星座控,爱吃,偶像剧
体育资讯
汽车,投资理财,时尚,新闻资讯,IT 数码,军事,美剧,电影
IT 数码,旅游,星座命理,体育
五月天,温柔,娱乐
新闻资讯,时尚,IT 数码,娱乐,旅游
旅游,IT 数码,动漫,星座运势,美食,星座命理
时尚,健康,美食,文艺,娱乐
新闻资讯,美食,文艺,娱乐,美女
视频音乐
美食,时尚,星座命理,美女,娱乐
美女,IT 数码
旅游,游戏动漫,IT 数码,体育
娱乐,投资理财
新闻趣事
美食,健康,时尚,汽车,美食,娱乐
旅游,美食,梁静茹
新闻资讯,汽车,文艺
淘宝,听音乐,爱音乐,时尚,旅游,美食,娱乐
时尚,汽车,文艺,旅游,娱乐
时尚
健康,汽车,IT 数码,体育,时尚,旅游,美食
美食
微博奇葩,视频音乐,新闻趣事,旅游,美女,新闻资讯,时尚,军事,文艺,体育
体育
体育
美图摄影

体育
逛街,90,爱玩,爱美食,music
娱乐,文艺,健康,旅游,摩羯座,囧,听歌,大学生
娱乐
搞笑幽默,宅
猫咪控,游戏,吃,动漫,睡觉,音乐,90 后,自由职业,宅男
游戏动漫
投资理财,健康,新闻资讯,教育就业,文艺
娱乐,旅游,体育
宅,旅行,音乐,听歌,旅游,80 后,电影
新闻资讯,投资理财,旅游
旅游,健康,美女,投资理财,美食
游戏动漫,投资理财,旅游,娱乐
穿衣美容
IT 数码
好性格,偶稀饭睡觉觉,吃,小说,90 后
德国生活
投资理财
美食,旅游,星座命理,娱乐
汽车,投资理财,旅游,娱乐,体育
体育
玩游戏,旅游
美女
包包,挂包,手袋,钱包,奢侈品,名人明星,美女,穿衣美容
娱乐
星座命理,旅游,美食,视频音乐,搞笑幽默,陕西生活
军事,游戏动漫
娛樂
漫画,安静,美食,健康,文艺
IT 数码,美食控,古典艺术,粤语
广东生活
旅游,汽车,星座命理,美食,时尚
恐怖片,羽毛球,看书,爱吃,幻想,爱电影,动漫,O 型血,旅游美食
车世界,动漫,美图摄影,IT 数码
新闻趣事,视频音乐,微博奇葩,美女,搞笑幽默
美女
健康,美女,旅游,美食,IT 数码
双重性格,完美主义,自由,学生,校园生活,萝莉控
娱乐
文艺,娱乐
微博控
旅游,娱乐,体育
90 后,电影
健康,旅游,时尚,娱乐,IT 数码

看电影,旅游,沉默
美食
星座命理,新闻资讯,美食
娱乐
美食,旅游,足球,水瓶座
旅遊
IT 数码,梅州生活,美女,校园生活,搞笑幽默,瞌睡达人,热情开朗幽默,军事爱好,好强
穿衣美容,浙江生活,星座命理,美食
爱电影,黄宗泽
新闻资讯,旅游,美食,体育,汽车
新闻资讯,健康,旅游,美食
萌宠,搞笑幽默,淮南生活,微博奇葩,三国杀,墨尔本曳步舞,吃货,双子座,自由,动漫
美女
美女
美食,文艺,旅游,健康,汽车
体育,双鱼座,篮球,90 后,吃货
网络,巨蟹座,汽车,平常心,运动,艺术,唱歌,时尚,旅行,音乐
星座命理
新闻资讯,美食,教育就业,体育,健康
娱乐,音乐迷,金在中,唱歌,听音乐,看书,上网
名人明星
体育
汽车
美女
教育就业,旅游,文艺,IT 数码,体育
新闻资讯,美女
好性格,体育
名人明星
旅游,美食,星座命理,名人明星,美女
汽车
投资理财,美食,Anime,渣画手,中二,比泉秋名,蓝色平方,成田病,黑沼青叶,DRRR
视频音乐,名人明星,微博奇葩,萌宠,搞笑幽默
旅游,韩国
自由,设计,交友,语录,阅读,财经,爱情,平常心,娱乐,运动
双子女,看小说,腐腐
巨蟹座,浪漫,时尚
IT 数码,星座命理,娱乐,军事
翼神
游戏动漫
搞笑幽默,游戏动漫
新闻资讯
宅,交友,90 后,自由,新闻,上网,创业,娱乐,听歌,音乐
投资理财,健康,旅游,IT 数码,文艺
娱乐,新闻资讯,健康,时尚

游戏动漫
体育
娱乐,投资理财,汽车,时尚,体育
投资理财,汽车,文艺,新闻资讯
萌宠,旅游,搞笑幽默,星座命理,穿衣美容
篮球,文学
新闻资讯
时尚,新闻资讯,旅游,娱乐
美女
新闻趣事,美女,北京生活,搞笑幽默
90 後,双子女,睡觉,宅,时尚,健康,星座命理,娱乐
巨蟹女,向日葵
美食,太原生活,新闻资讯
文艺
视频音乐
新闻趣事
名人明星
古典音乐,深圳,旅行,音乐,咖啡,足球,自然,创业,汽车,心情
新闻资讯,汽车,IT 数码
IT 数码,囧,天蝎,music,数码,大学生
视频音乐
体育,娱乐
听歌,运动,旅行,会做饭,低调,狮子座,NBA,篮球,90 后
体育,投资理财
星座命理,新闻趣事,海南生活,搞笑幽默,爱阳光,爱生活,爱热闹,爱美食,爱幻想,爱睡觉
时尚,美食,娱乐
时尚,美食
90 后
游戏动漫
健康,旅游,美食,娱乐,文艺
娱乐,旅游,时尚,汽车,IT 数码,网店卖家,网店掌柜,高中生,喜欢听歌
时尚,文艺
搞笑幽默,时尚,文艺,美术
美女
旅游,文艺,健康,走走停停,不时回头看看,记录心情,双子座
体育,投资理财
旅游
genevieve,懒人
自由,时尚,旅游,90 后,电影,音乐
名人明星,视频音乐,美女
搞笑幽默
时尚,狮子座,游戏,摄影,平常心,宅,旅行,电影,动漫,音乐
美女,美食,文艺,旅游,健康
娱乐

教育就业
美图摄影,美食,情感生活,云南生活,美容服饰
内向
汽车,娱乐,宅女,游戏,动漫
美食
旅游,健康,时尚,IT 数码,文艺
新闻资讯,娱乐,健康,美食,旅游
搞笑幽默,足球,乐观,幽默,自由,80 后
健康,时尚,文艺,美食,新闻资讯
视频音乐,天秤座,微小说,淘宝男装,打台球,吃货,睡懒觉,看电影,潮流时尚,90 后
健康,财经金融,财经,金融投资
看电影,k 歌,娱乐,汽车
旅游
文艺,体育
吃货,搞笑,孙燕姿,巨蟹,情绪化
IT 数码
美食
娱乐
健康,正能量
时尚
时尚,文艺,星座命理,健康,投资理财
旅游
博客,亲情,白羊座,乐观,看书,睡觉,宅,听歌
美食
IT 数码,旅游,体育资讯,天蝎座
旅游,穿衣美容
美容服饰,宠物图集,美图摄影,星座运势,美食
安静,快乐,活泼,健身,购物狂,建筑,娱乐,汽车,旅游,投资理财
汽车
体育,娱乐
美食,娱乐,时尚
时尚,汽车,美女,游戏动漫,IT 数码
美食
吃
文艺,娱乐,睡觉睡到自然醒,世界杯,青春,自恋,睡觉,宅,哲学,手机看小说
搞笑幽默,江苏生活,大学,校园,财经,好书
文艺
美食,投资理财,娱乐,时尚
娱乐
新闻资讯,文艺,投资理财,旅游,娱乐
直销,热爱生活
佛,轮滑,IT 数码
娱乐
运动,文学,美剧,白羊座,电影控,高中生,爱音乐,淡定,打篮球,军事

星座命理,时尚,健康,旅游,美食
新闻趣事,搞笑幽默,视频音乐,名人明星,美女
娱乐,教育就业,新闻资讯,健康,旅游,星座命理,时尚
游戏动漫
体育,美食
军事天地,车世界,旅游,美食,台州生活
新闻资讯
游戏动漫
新闻资讯
巧克力,搞笑,运动,美食,听音乐,娱乐
旅游
军事,爬山,吃,网络,偶稀饭睡觉觉,乐观
游戏动漫,美食
妖精,学生一族,天蝎女,爱睡觉,胡思乱想,臭美,经济,懒,成都,正太控
汽车
星座命理,旅游
美女,兼容
美食,游戏动漫
教育就业
善良,看书,手机,小说,美食,自由,上网,交友,听歌,旅游
娱乐,旅游,星座命理,美食
游戏动漫,娱乐,IT 数码,健康
萌宠
IT 数码,投资理财,教育就业,娱乐,美食
旅行,美食,时尚,旅游,电影
时尚
ipad,乔布斯,苹果 4,iphone,苹果,苹果控,苹果迷,苹果 5,iphone4,iphone5
游戏动漫
时尚,娱乐
爱电影,80 后的尾巴,巨蟹女,文艺
不打扰,是我的温柔,娱乐
游戏动漫,汽车,文艺
教育就业,美食,娱乐,体育,时尚,IT 数码,游戏动漫
简单却不平凡
美女,游戏动漫
各种睡不着,美食控又减肥控,豪杰中的女人,水瓶座加 O 型血
文艺
美食,新闻资讯,健康,投资理财,星座命理
文艺,美食,投资理财,健康,新闻资讯
美食
Asenal
健康,美食,80 后,水瓶座
时尚,星座命理,旅游,文艺,娱乐
军事,文艺,汽车,健康,旅游,体育

体育
旅游,健康
体育,星座命理,新闻资讯,娱乐,教育就业
视频音乐
美食,IT 数码,体育
健康,80 后
游戏动漫,健康,旅游,星座命理,美食
萌宠,炮灰,相府门生,腐丫,漫迷,声控,古风
美食
新闻资讯,军事,音乐
旅游,健康,新闻资讯,投资理财,娱乐,时尚
汽车,IT 数码,体育
名人明星,视频音乐,新闻资讯,时尚,旅游,美食,娱乐
我爱 UC 浏览器
我就是我,高中生
投资理财
健康,体育,娱乐
星座命理,名人明星,娱乐
美食
时尚,IT 数码,娱乐
美食,娱乐,军事,旅游,星座命理,IT 数码,体育,文艺
旅游,低调,睡觉
娱乐
搞笑幽默
宅,美剧,旅行
视频音乐
娱乐
新闻趣事,旅行,好性格,篮球,看书
美食,健康,新闻资讯,娱乐,体育
懒人,游戏动漫,宅,小说,偶稀饭睡觉觉,白羊座,阅读,上网,自由
名人明星
旅游,美食,文艺,体育
穿衣美容
新闻资讯,星座命理,教育就业,文艺,娱乐,体育
美女,时尚,星座命理,旅游,文艺
健康,美女,星座命理,教育就业,IT 数码
美女,搞笑幽默
健康
学生一族
星座命理
时尚,汽车,投资理财,娱乐
执着,巨蟹,快乐,音乐爱好者,爱生活,90 後,听音乐,美食,穿衣美容,旅游
新闻趣事
美食

美食
人生,搞笑,交友,运动
投资理财,文艺,游戏动漫,旅游,美食
旅游
搞笑幽默,体育资讯,八卦杂谈
体育
美女,八卦杂谈,大连生活,搞笑幽默
北京生活
健康,旅游,时尚,汽车
睡觉听歌宅家里,上网,睡觉,自由,旅行,时尚,听歌,音乐
娱乐
娱乐,星座命理
财经资讯,创业,A股,低调,经济,金牛座,读书,足球,微笑,金融
美食,健康,旅游,星座命理,娱乐
浪漫,搞笑,90後,笑话,感性,媒体人,听音乐,自由,90后非脑残,冷笑话
星座命理,健康,娱乐,教育就业,体育
健康
旅游
教育就业
花草,园艺,园林,盆景,养护,花卉
美女,电影,90后,学生,宅,音乐
旅游
旅游,健康,教育就业,娱乐,美食
汽车
跑步,汽车,IT数码,娱乐
太原生活,自由职业,八卦,微博,网购,生活,上网
时尚
公平,正直,亲情,医疗,善良
美食
汽车
文艺
搞笑幽默
IT数码,投资理财,汽车,游戏动漫,旅游,哲学,手机看小说
美食,新闻资讯,健康,旅游,文艺
娱乐,健康,文艺,旅游,美食
健康,军事,汽车,美女,美食
搞笑幽默,星座命理,视频音乐,江苏生活,旅游
娱乐,投资理财,美女,旅游,美食,文艺
美女,美食,IT数码
文艺
我爱UC浏览器
投资理财
新闻趣事
名人明星,旅游,美食,80后,音乐

偶稀饭睡觉觉
手机,摄影爱好者,宠物,美食,汽车,旅游,IT 数码,文艺
名人明星,北京生活,旅游
旅游
文艺,美食,旅游,娱乐
听歌,音乐,旅行,时尚,宅,90 后
娱乐,汽车,游戏动漫,美女,健康
教育就业,健康,星座命理,娱乐
服装,工作狂,旅游美食,乐橙,爱吃。
体育,学生一族
健康,教育就业,文艺
足球,汽车
娱乐,健康,旅游,美食,文艺
平面设计,星座命理
星座命理,旅游,健康,汽车,体育
旅游
八卦,搞笑,汽车,吃,旅游
新闻资讯,文艺,IT 数码,娱乐
星座命理
旅游,教育就业,文艺,军事,体育
四川生活,偶稀饭睡觉觉
美食,健康,新闻资讯,旅游,文艺
体育,旅游,军事
视频音乐,游戏动漫
旅游
娱乐
教育就业,健康
教育就业
军事,美食,旅游,娱乐,IT 数码
文艺,健康,教育就业
星座命理
新闻资讯,IT 数码,文艺,游戏动漫,时尚
微博奇葩,广东生活,搞笑幽默,旅游
水瓶女,旅游
体育,游戏动漫,旅游
新闻趣事,旅游
文艺,IT 数码,游戏动漫,投资理财,幽默,运动,体育,文学,电影
文艺
IT 数码,文艺,体育
美食,文艺
游戏动漫,投资理财,美食,娱乐
美食,旅游,萌宠,名人明星
健康,投资理财,时尚
新闻资讯,汽车,游戏动漫,美女,IT 数码

旅游,美食,娱乐,教育就业,时尚
美女,军事,游戏动漫,文艺
健康,旅游,美食,文艺,娱乐
上网
游戏动漫
投资理财
美食,军事,IT 数码,体育
交友,听歌,上网
教育就业,投资理财,旅游
挣钱才是硬道理,动漫控,快乐大本营
娱乐
爱薄荷,B 型血,爱自由,耽美,游戏动漫
旅游,汽车,IT 数码,文艺,娱乐
时尚,投资理财,美食,星座命理,娱乐
新闻资讯
时尚
投资理财,美食,旅游,健康,娱乐
搞笑幽默,美食,旅游,美女
游戏动漫,美食,文艺,娱乐,美女
汽车
时尚
军事,旅游
娱乐
文艺,军事,IT 数码,体育,娱乐
旅游
美女,娱乐,体育,健康
投资理财,新闻资讯,汽车
娱乐,旅游
美女,文艺,娱乐,军事,时尚
跑步,电影爱好者,诗词,中医,music,文字控,游戏动漫,新闻资讯,娱乐,文艺
名人明星,玩儿,八卦,囧,音乐,手机,平常心,90 后
健康
娱乐
汽车,旅游,军事
健康
時尚,投資理財,文藝,旅遊,健康
文艺,军事,体育,娱乐,IT 数码,偶喜欢玩,好性格,宅女,音乐
孤单星球,冥王天蝎,星座命理,疯狂与安静,旅行,宅,电影,80 后,音乐
视频音乐,旅游,自由,电影,90 后,听歌,音乐
互联网,文字,创业,网购,电影
健康,星座命理,投资理财,时尚,美食
O 型血,德国,登山,设计师,韩剧,星座命理,旅游,娱乐,美食,时尚
钓鱼以静思,羽毛球控,绝世好爸
文艺

健康,旅游,IT 数码,文艺,时尚,可爱,水瓶女,冬瓜,吃货,90 后非脑残
时尚,文艺,旅游,美食,娱乐
娱乐,星座命理,时尚,文艺
体育
旅游,星座命理,时尚
码农,旅游,IT 数码
时尚,健康,旅游,美食
淘宝,教育,90 后,淡定,摇滚,音乐,睡觉,唱 K,视频音乐,汕头生活
旅游
名人明星,旅游,美女,穿衣美容
健康,文艺,娱乐,教育就业,时尚
娱乐,营销,手机,吐槽用的
军事
游戏动漫
沉默,汽车
文艺
IT 数码,星座命理,旅游,健康,教育就业,文艺
IT 数码
美女,好性格
文艺,娱乐,游戏动漫
旅游
体育,金牛座,篮球,80 后,电影,足球,时尚,音乐
娱乐
文艺,美食,时尚,旅游
娱乐
星座命理
外貌协会,射手座,上网,投资理财,旅游,美食,听歌,音乐
教育就业,健康,投资理财
星座命理
八卦杂谈,新闻资讯
美食,旅游,音乐
美食
娱乐,投资理财,旅游,星座命理,美食
設計,乐呵乐呵
美食,IT 数码,游戏动漫
IT,娱乐,健康,文艺,美食,旅游,奋斗
IT 数码,美食,时尚,美女,游戏动漫,美食
健康,游戏动漫,美食,星座命理
旅游,星座命理,IT 数码
娱乐,爱游戏,忧郁,射手座,宅女,80 后
美女
美食,微博奇葩,旅游,搞笑幽默,美女
金牛座
计算机,90 後,朋友,设计,创意,街拍,阳光,爱美文,美食,爱自由

旅游,新闻资讯,文艺,时尚
电影,lol,Jeep 牧马人,体育,文艺,汽车,美食,旅游
摩羯座,90 后但我不脑残,音乐爱好者,自由职业,数码,浪漫,时装,恐怖片努力 ing
旅游,美女
名人明星,其他生活,搞笑幽默,视频音乐,萌宠
吃货,读书,小說,經濟,音乐,旅行,慎独,随喜
IT 数码,娱乐,旅游
快乐,玩,健身,汽车,美女,投资理财,时尚,娱乐
教育就业,星座命理,新闻资讯,健康,娱乐
时尚,音乐
健康
星座命理
旅游
健康,旅游,美食,投资理财,娱乐
星座命理,旅游,美食,体育,娱乐
健康
文艺
健康,新闻资讯,文艺,娱乐,体育
创业,爱情,摩羯座,旅行,电影,自由,时尚
旅游
文艺
健康
美食,旅行,听歌,音乐,电影
移动互联网,微博,教师,交友,健康,IT 数码,旅游
新闻趣事,旅游
星座命理
投资理财,军事,健康,教育就业,体育
健康,投资理财,教育就业,星座命理
军事,IT 数码,游戏动漫
投资理财,娱乐
体育
大学生,外貌协会,幽默,吃,坚强,胡思乱想,电子商务,学习,购物狂,爱美食
军事,IT 数码,体育,投资理财,旅游,汽车
香港生活
看美女,投资理财,教育就业,IT 数码,体育
旅游
汽车
萌宠
娱乐,美食,汽车,投资理财,体育
美食
美食
星座控,水瓶座
IT 数码
汽车

美食
娱乐
体育
宣城生活,旅游,美食
旅游,美食,萌宠,微博奇葩,其他生活
时尚,娱乐,旅游,美食
视频音乐
健康
执着,美食
体育
喜欢听歌,新闻资讯,健康,投资理财,美食,军事
美女,健康,旅游,IT 数码,文艺
娱乐,游戏动漫,旅游
美食,旅游,投资理财,军事,IT 数码
文艺,体育,健康,美女,IT 数码
健康,教育就业,娱乐
穿衣美容
校园,IT 数码
美食
旅游
体育,游戏动漫
搞笑幽默
娱乐
搞笑幽默,名人明星,美女
健康
娱乐,时尚
时尚
健康,投资理财,汽车,军事,IT 数码
美国生活
IT 数码
游戏动漫,投资理财,文艺,旅游,IT 数码
自由,简单,吃货,爱玩,购物狂,睡懒觉,自由职业,双重性格,交朋友,双鱼座
教育就业
IT 数码
文艺
美食,新闻资讯,旅游,体育,娱乐
美食,健康,游戏动漫
校园生活,天津生活,IT 数码,美食,星座命理,旅游,教育就业
游戏动漫
糾結體,善良,删除癖,雙魚座
足球,我爱 UC 浏览器
游戏动漫,军事,体育
微博奇葩,视频音乐,新闻趣事,法国生活,搞笑幽默,时尚,娱乐
汽车

文艺
游戏爱好者,BBOY
时尚
游戏动漫
我不是扯线娃娃
周杰伦,时尚达人,驴友,潮人,魔羯座,文艺
投资理财,教育就业,汽车,美女,军事
时尚,教育就业
听歌,射手座,亲情,运动,旅行,90后,唱歌,阅读,交友,动漫
IT数码
美食,体育,投资理财,旅游,新闻资讯
伤感,购物狂,上班族,月光族
视频音乐,微博奇葩,搞笑幽默
我爱UC浏览器
美食,旅游
新闻资讯
军事
美女,体育
美食,汽车,体育,文艺,IT数码
健康
体育
娱乐
娱乐,K歌
IT数码
旅游,健康,星座命理
IT数码,学生一枚,动漫控,猫咪控,动画
视频音乐
健康,娱乐,旅游,美食
好好品歌词,射手座,唱歌,善良
新闻资讯
原创,摇滚,民谣,篮球,吉他,旅行,李宇春,ukulele
IT数码
娱乐
旅游
IT数码,投资理财,时尚,美女,健康
娱乐
旅游,90后,梦想,双子座,登山,交友,国旗班
旅游,美食
新闻资讯
美女,投资理财,汽车
体育,学生
文艺,时尚
军事,娱乐,体育,旅游,文艺
军事,新闻资讯

旅游
旅游,文艺,健康,时尚,体育
紫砂,紫砂爱好者,紫砂壶,爱美食,music,喜欢旅游,看电影,文艺,淘宝,美食
好性格,美女,羽毛球,睡觉,音乐
摄影控,科幻,街拍,淘宝控,美剧,自由职业,仙剑,古典音乐,文艺,时尚
美女
体育,美食,教育就业
投资理财
旅游,文艺
微博奇葩,新闻趣事,云南生活,美食,新闻资讯,投资理财,旅游,IT 数码
健康,旅游,游戏动漫,军事,IT 数码
美食
旅游,大学生
重庆生活,体育
娱乐
八卦杂谈,美女,中山生活,视频音乐,搞笑幽默
美食,时尚
IT 数码
体育
美女,健康,军事,娱乐
星座命理,IT 数码,文艺
时尚
美食,石家庄生活,微博奇葩,星座命理,穿衣美容
星座命理
投资理财,时尚,健康,旅游,IT 数码
星座命理
旅游,投资理财,IT 数码,游戏动漫,动漫,体育,娱乐
健康,新闻资讯,娱乐
娱乐
时尚,汽车,健康,旅游,教育就业,爱心,护士
体育
军事,新闻资讯,汽车,投资理财,职业患者,心灵旅行家,爱家达人,顿悟,光明的理想家
交友
文艺,美女,健康,旅游,美食
美食
投资理财
娱乐
美女,美食,时尚,汽车,体育
前方是绝路,希望在转角
IT 数码
健康,星座命理,教育就业,时尚,爱唱歌,娱乐,旅游
美女,文艺,游戏动漫,美食,体育
汽车,文艺,娱乐,体育,IT 数码
美食,文艺,健康,投资理财,旅游

娱乐,时尚
时尚,娱乐
新闻资讯,美女,汽车,IT 数码,体育
游戏动漫,健康,体育
基督徒,旅游,文艺
时尚,娱乐,投资理财,旅游
投资理财,汽车,游戏动漫,军事,体育
旅游,星座命理,军事
旅行,80 后
搞笑幽默
美女,军事,健康,旅游,娱乐,体育,偶喜欢玩,阅读,爱情
汽车,文艺
旅游
娱乐,新闻资讯,美食,投资理财,汽车
小资,厦门,安静,购物狂,看小说
游戏动漫,IT 数码,军事
娱乐
宠物图集,读书分享,美图摄影,美食,福州生活
美食
看小说,B 型血,o 型血,懒,宅女,随性,娱乐
美食,星座命理,健康,游戏动漫,投资理财
娱乐
秦时明月,古风,旅游
娱乐,旅游,时尚,旅行,学生,美食,90 后,电影,音乐
阅读,爱情,天秤座,运动,自由,看书,动漫,听歌,90 后,学生
新闻趣事
旅游,IT 数码,美女,游戏动漫,美食
搞笑幽默
汽车,美女,旅游,IT 数码
视频音乐,旅游
新闻资讯
维他命,银魂,电视剧,开心,水瓶,热情
福建生活
gelatopique,snidel,专柜代购,时尚,旅游
曾轶可脑残粉
娱乐
娱乐,体育
IT 数码,旅游,美女,娱乐,体育,汽车
执着,旅游
新闻资讯,军事,时尚,健康,旅游
旅游,爱情,娱乐,处女座,吃货,听歌
新闻资讯
健康,时尚
科学探索,美食,电影,自由,宅,旅行,时尚,旅游,听歌,音乐

汽车
IT 数码
IT 数码,鑫仔,撸啊撸,阿童木,阿木木,皇马,运营
手机,浪漫,美食,娱乐,旅游,教育就业,体育
旅游
搞笑幽默,时尚,90 后,听歌,音乐
旅游,天蝎座
汽车
旅游,健康,美食,投资理财,体育
旅行
文艺,体育,娱乐
IT 数码
娱乐
旅游,投资理财,教育就业,美女,文艺
健康
微博奇葩
处女座,思考,80 后的尾巴,做梦,美女,足球,传媒,自由
军事,IT 数码,文艺,体育,娱乐
运动,汽车,音乐,吃
IT 数码
新闻资讯,IT 数码,娱乐,旅游
80 後,平常心,美食
IT 数码,军事,教育就业,文艺,旅游
新闻资讯,美女,娱乐,军事,体育
娱乐
军事,娱乐
苹果控,购物狂,摄影,家居,喜欢旅游,手机,喜欢听歌,热爱生活,环保,独立
情绪化,聊天
游戏动漫,新闻资讯,健康,旅游,文艺
健康,美食,教育就业,时尚,投资理财
思考,爱自由,独立,流浪,男人
动漫你爱我吗?
旅游,时尚,美女,美食
旅游
军事,新闻资讯
狮子座,双鱼座
文艺,体育,娱乐
健康,旅游,星座命理
音乐
娱乐
IT 数码
文艺,投资理财,游戏动漫,IT 数码,偶喜欢玩,狮子座,游戏,原创,幽默,互联网
游戏动漫,文艺
娱乐

投资理财,美食
宠物图集,名人明星,台湾生活,视频音乐,美容服饰
福宝宝
旅游,星座命理,IT 数码,文艺,娱乐
旅遊,搞笑幽默,美食,穿衣美容,星座命理
美食
美女
军事,体育,投资理财,汽车,美女
娱乐,美女
文艺
心理学,巨蟹座,做梦,时尚,军事,文艺,娱乐
新闻趣事
娱乐,汽车,美女
乐天派,宅,团饭,90 后
11 锥,KrystalJung,Kimjongin,Parkchanyeol
军事,旅游
文艺,minoz,旅游,学生,宅,90 后,听歌
视频音乐
美女,投资理财,汽车,旅游,文艺
星座命理
旅游,娱乐
体育,汽车,篮球
投资理财,娱乐,美食
IT 数码,娱乐,旅游,美食,文艺
旅游,时尚,娱乐
教育就业
美女,美食,游戏动漫
新闻资讯,健康,星座命理
健康,旅游,体育
娱乐
新闻资讯,时尚,射手座,音乐,手机,偶喜欢玩
健康,投资理财,汽车,军事,IT 数码
文艺,吉他,时尚潮流,Music,文字控,唱 K,疯狂,爱美食,街舞
娱乐,台球,相声,时尚,网络,摄影,电影
旅游
美女,游戏动漫,军事
大学,体育
IT 数码
教育就业,旅游,美女
时尚,美食,星座命理,健康
投资理财
娱乐,新闻资讯,IT 数码,美女,游戏动漫
动漫
健康,美食,娱乐,时尚,文艺

什么的最讨厌了,小说
游戏动漫
个性,沉默,双鱼女,完美主义,时尚,健康
星座命理,美女,体育
健康,投资理财,IT 数码,新闻资讯,汽车
汽车,新闻资讯,文艺,美食,军事
军事,美食,旅游,体育,IT 数码
娱乐,摩羯座,时尚,80 后
游戏动漫
游戏动漫,旅游,美食,文艺
军事天地,开心,娱乐,IT 数码,军事,旅游,新闻资讯
文艺
体育
游戏动漫,美女,教育就业,军事,IT 数码
连接服务器失败
游戏动漫,娱乐,体育
娱乐
旅游,视频音乐,福建生活
军事,IT 数码,文艺,体育,娱乐,游戏,动漫,电影,篮球,数码控
青岛生活
旅游
娱乐
IT 数码,游戏动漫
汽车,旅游,音乐,电影,旅行,自由,听歌,80 后
美食
体育
健康
旅游,时尚,娱乐,欧美控,粤语,90,BIGBANG,闷骚
健康,文艺,教育就业
星座命理,文艺
投资理财,健康,教育就业,美食,旅游,旅行,电影,音乐
新闻资讯
美女
时尚,美女
游戏动漫
娱乐
美女,投资理财,时尚
IT 数码
网络营销,淘宝,购物,计算机,互联网,互粉,科技,爬山,上海,娱乐
新闻资讯,IT 数码,投资理财
时尚,教育就业,文艺,体育,娱乐
美女,美食,吃喝玩乐,狗狗,烹饪,游泳,养生,善良,生活,吃
健康,体育,娱乐
青春,内向,考研,看小说,睡觉睡到自然醒,政治,书籍,资讯,IT 数码,文艺

时尚, 新闻资讯, 健康, 星座命理, 美食
日本, 护肤品, 海外, 代购, 奢侈品, 复刻
美食, 时尚, 军事
娱乐
美食
健康, 健康快乐, 天然养生, 四季红糖姜茶, 健康快乐多, 红糖姜茶, 降尿酸痛风茶, 番石榴降糖茶, 小苦瓜降糖茶
视频音乐
玩, 爱笑, 美食控, 陈奕迅, 懒人, 外貌协会, 音乐达人, 睡懒觉, 文艺, 看电影
体育, 爱
美食, 星座命理, 文艺
微博奇葩
旅游
投资理财, 美女, 军事, 汽车, IT 数码, 90 后
八卦杂谈
汽车
文艺, 时尚, 旅游, 美食, 健康
水瓶座, 摇滚, 胡思乱想, 80 后的尾巴, 听音乐, 旅游, 汽车, 文艺, 体育
文艺, 时尚, 美食
IT 数码
搞笑幽默, 新闻趣事, 星座命理, 穿衣美容
投资理财
微博奇葩, 星座命理, 旅游, 美食, 新闻趣事
名人明星, 星座命理
诗经, 考古, 国学, 昆曲, 佛教, 汉服, 西班牙语
旅游, 时尚, 健康, 娱乐, 美食
IT 数码
我就是我, 原创, 人生, 70 后, 傻, 汽车
新闻资讯, 教育就业, 健康, 体育, 军事
健康
时尚, 微博, 浪漫, 巨蟹座, 文字
美食, 宅, 时尚, 旅行, 旅游, 美食, 80 后, 听歌, 音乐
时尚, 旅游, 星座命理, 娱乐
健康
星座运势, 成都生活, 搞笑幽默
美女, 新闻资讯, IT 数码, 美食
娱乐, 旅游
星座命理, 美食
时尚, 旅游, 星座命理, 文艺, 娱乐
健康, 旅游, 投资理财, 娱乐, 体育
军事, 游戏动漫
娱乐
穿衣美容, 宅, 美食
时尚, 娱乐, 美食

汽车,骑马爱好者,驴友,骑游,摄影爱好者,自驾游爱好者,北京,爬山,旅游
新闻资讯,汽车,旅游,时尚,美食
时尚
星座命理,美食,健康
新闻资讯,文艺,体育,NBA,善良,文字,看书,宅男,篮球
穿衣美容,视频音乐
娱乐
娱乐
美食
搞笑幽默
汽车,时尚,投资理财,教育就业,美女,体育,娱乐
娱乐
天蝎座
汽车,时尚,IT 数码,美食
旅游
爱自己多一点,芳香爱情,不该有的情绪,90 后,漂亮生活,美丽女人
健康,IT 数码,看书,新闻,科技
教育就业,健康,时尚,IT 数码
宅女,爱幻想,看小说,90 后非脑残,天秤,小清新,喜欢音乐,吃喝玩乐,好性格,娱乐
娱乐
汽车,体育
旅游,军事
旅游,娱乐,文艺
游戏动漫
动漫,学生,水瓶座,篮球
时尚,健康,文艺,投资理财,娱乐
旅游,娱乐,美食,八卦,亲情,交友
美食,旅游,时尚
星座命理,健康,文艺,娱乐,美食
旅游,军事,IT 数码,投资理财,汽车
三亚生活
旅游,游戏动漫,美食
O 型血,环保,白羊座,80 後,羽毛球,NBA,烹饪,美食,军事
广东生活
娱乐
旅游,美食
星座命理
教育就业,军事,健康,投资理财,汽车
健康,娱乐,美食,巨蟹女,爱生活,单身,阳光,宅,A 型血,90 后非脑残
新闻资讯,IT 数码,军事
睡觉,90 后,水瓶座,吃货,学生,音乐,小说,宅女,游戏,上网
健康,旅游,文艺,娱乐,美食
美食,美女,IT 数码,投资理财,娱乐
文艺,IT 数码

健康,旅游,星座命理,美食,文艺
电影
穿衣美容,美女
汽车,娱乐,旅游,美食,音乐
美食,健康
摄影爱好者,美女,时尚,音乐
新闻趣事
股票
90 后,御姐未满
游戏动漫,汽车,新闻资讯,IT 数码
汽车
萝莉是本命,平时很娇羞,有时会黑化,离别产物,腹黑,90 后,动漫,文学,宅,偶稀饭睡觉觉
健康,旅游,娱乐,唱歌,睡觉,时尚,美食,旅行,听歌,音乐
文艺
旅游,新闻资讯,汽车,IT 数码
微博奇葩,搞笑幽默
新闻趣事
吉他,文学,电影
时尚,IT 数码,美食,健康,教育就业
美食,文艺,娱乐,双子座,幽默,90 后,听歌,音乐
文艺
旅游,IT 数码,文艺,时尚,游戏动漫
新闻趣事,搞笑幽默,微博奇葩
海贼王,交友,喜欢音乐,动漫控,漫画
白羊座,爱情,美剧,乐观,娱乐,幽默,音乐,学生,时尚,90 后
汽车,游戏动漫,军事
动漫迷,W,TVXQ,JYJ,东方神起,金俊秀的死忠饭,仙后一枚,90 后
视频音乐,校园生活,搞笑幽默,台球,足球,玩,自由,旅行,电影,音乐
微博奇葩,美食,新闻趣事,搞笑幽默
娱乐,文艺
80 后
书画艺术,旅游美食爱好者,新闻资讯,星座命理,文艺,美食
穿衣美容,摄影,上网,睡觉
美食
娱乐,文艺
投资理财,健康,旅游,美食
摄影,小说,交友,幽默,新闻资讯,美女,汽车,旅游,美食,文艺
搞笑幽默
游戏动漫
美食,时尚,IT 数码,汽车,旅游
搞笑幽默,时尚,旅行,金牛座,汽车,创业,80 后,营销
时尚,健康,美女,旅游,军事
美女,汽车,体育
体育,军事,IT 数码

打篮球,旅游,体育
听歌,时尚,旅游,电影,音乐
名人明星
生活,财经
文艺,天蝎座 80 后
美女,旅游,投资理财,IT 数码,体育
WSM,生活,黄耀明,阅读
旅游,新闻资讯,汽车,IT 数码,投资理财
吃货,电影,手机,宅,音乐,90 后,白羊座,时尚,乐观,80 后
肖飞,汕头籍,美食,DIY
汽车,美食,娱乐
搞笑幽默
文艺
旅游
新闻资讯,美女
旅游,体育
美女
娱乐,体育
新闻资讯,投资理财,美食,健康,旅游
药理学,体育篮球,政治时事,药物制剂,药剂师,教育就业,体育,文艺,军事,旅游
娱乐
旅行,美食,音乐
新闻资讯,娱乐,文艺,时尚,汽车
娱乐,体育
旅游,IT 数码,文艺,动漫,看书,美食
火影忍者,篮球爱好者,美食,游戏动漫
名人明星,视频音乐,情感生活,读书分享,新闻趣事,文艺
美容服饰
IT 数码
文艺
我爱 UC 浏览器
旅游
体育
旅游,微博奇葩,其他生活,搞笑幽默,新闻趣事
新闻趣事,搞笑幽默,IT 数码,八卦杂谈
健康,旅游,文艺,美食,新闻资讯
音乐控,爱美食,喜欢音乐,喜欢听歌,旅游
文艺,看书,旅游,电影,80 后,宅
投资理财,美食,文艺
美食,娱乐
时尚,新闻资讯,健康,旅游,文艺
娱乐
美食
感恩人生,学会做人后做事,人生道理

健康,旅游,投资理财,美食,娱乐
旅游,八卦杂谈,篮球,乐观,射手座,宅
健康,投资理财,旅游,IT 数码
时尚,电影,音乐,枪械,汽车,摩托车,海贼王,我爱 UC 浏览器
投资理财,旅游,文艺,健康,时尚
新闻资讯,汽车,军事,IT 数码
微博奇葩,浙江生活,搞笑幽默
美女
文艺
美食,绘画,爱幻想,天蝎女,文艺
名人明星
健康,娱乐,美食,旅游,文艺
看电影,文艺
IT 数码,军事,投资理财,汽车,美女
旅游
娱乐,美女,投资理财,时尚,健康
游戏动漫,美女
IT 数码,娱乐,健康,星座命理
旅游
搞笑幽默
投资理财,健康,美女,军事
MG,80 后,体育健将,汽车广告策划,K 歌之王,双子座,O 型血,小眼迷人,孝顺,大哥
美女,美图摄影
健康,体育,游戏动漫
游戏动漫,娱乐,幽默,上网,网络,听歌,90 后
旅游,美食
新闻资讯,教育就业,旅游,美食,文艺
视频音乐,搞笑幽默
新闻趣事
游戏动漫
时尚,娱乐,文艺,旅游,美食
美食,IT 数码,吃,足球,看书,听歌
时尚
体育
体育
时尚,健康,文艺,旅游,美食
美食
时尚,美食,IT 数码
投资理财
文艺,90 后,宅女,白色,天蝎座,学生,偶稀饭睡觉觉
搞笑幽默
娱乐,健康,时尚,美食,体育
娱乐,美食,健康
旅游,投资理财,美食

文艺
体育
娱乐,军事,文艺,汽车,体育
美女,娱乐,游戏动漫
IT 数码,旅游,军事
读书分享,房产,IT 数码,新闻趣事,浙江生活
美女
汽车
IT 数码
美女,健康,投资理财,旅游,美食
我爱 UC 浏览器
IT 数码
新闻资讯,旅游,军事,娱乐,星座命理
电子商务,微博,自由,摄影,网购,90 后,听歌,营销,汽车,艺术
教育就业,新闻资讯,健康,旅游,时尚
娱乐
旅游,汽车
同城人
美女
美食
IT 数码,文字,文学,平常心,互联网,创业,音乐
视频音乐,自拍,cosplay
健康,投资理财,旅游,文艺,娱乐
游戏动漫
投资理财
星座命理,文艺
美女
时尚
魔兽,宅男,美食,电影,时尚
文艺,美食,军事,娱乐,旅游
时尚,旅游,美食,星座命理,IT 数码
汽车
教育就业,游戏动漫,新闻资讯,旅游,美食,阅读
汽车,娱乐
连云港生活,玩儿,宅,大学生,上网,微博,吃,新闻,读书,学生
新闻趣事,搞笑幽默
娱乐,旅游,健康
平常心,大学生,google,独立,学生一族,军事,美食,星座命理,文艺,IT 数码
体育
旅游,美食,星座命理,穿衣美容
IT 数码
投资理财
IT 数码,汽车,时尚,文艺,娱乐
神经质,芯片,偶喜欢玩,双子座,游戏,旅游,音乐,90 后,自由

汽车,文艺
娱乐,健康
娱乐
处在狂风暴雨中
搞笑幽默
娱乐,体育
运动,乐观,电视剧,爱睡觉,爱生活,舞蹈,奋斗,美食爱好者,喜欢听歌,热爱生活
爱电影,旅游,IT 数码,美女
军事,旅游,游戏动漫,美女,IT 数码
穿衣美容
双子女,吸血鬼
美食,健康,娱乐,游戏动漫,星座命理
星座命理
旅游,新闻资讯,体育,健康,IT 数码
游戏动漫,时尚,汽车,美女,IT 数码
新闻,媒体,电影
健康
体育
搞笑幽默,90 后
体育,美食,时尚,汽车,美女
游戏动漫,体育,篮球,爱猫,摄影,PSP,HipHop
淘宝,旅游
体育
健康
文艺,听音乐
娱乐,文艺,旅游,90 后
星座命理
汽车
时尚,健康,旅游,美食,娱乐
游戏动漫
时尚
美女
旅游,90 后
懒人,湖北生活,逛街,喜欢听歌,美食
文艺,美食,娱乐
80 后宅女,天蝎,教师,简单,南京
新闻趣事
新闻资讯
娱乐
美女,军事,美食,文艺,体育,热情,爱旅行,篮球爱好者
武汉生活
IT 数码
娱乐
游戏动漫

文艺,喜欢写东西,喜欢偷懒,哈哈
旅游,新闻资讯,美女,IT 数码,军事
新闻资讯,汽车,美女,军事,体育
旅游
财经资讯
美食
旅游,文艺,IT 数码,汽车,健康
美食,赣州生活,IT 数码,职场招聘,体育资讯
文艺
旅游
娱乐,美食,体育
小女人
文艺,体育,娱乐
美女,时尚,军事,娱乐
教育就业
体育
IT 数码
爱生活,读书,巨蟹座,紫色,学生一枚
旅游
美容服饰,工作,唱 K,散步,喜欢音乐,可爱
我爱 UC 浏览器
金华生活
游戏动漫
娱乐
汽车,美女,新闻资讯,旅游,军事
新闻资讯,旅游,美食,军事,IT 数码
新闻资讯
娱乐
娱乐,最爱热血少年漫,花开院龙二,节操君去了天国,BLGLBG 通吃,腐宅双修
星座命理
麻将机销售,13786152186,维修电话,13874844287,娱乐,投资理财,体育
文艺
美食
固执死脑筋,偏执,疯子
安徽生活,水瓶座,90 后,爱太阳,爱音乐,爱吉他,爱唱歌,爱文学,吃货,懒货
娱乐
IT 数码,投资理财,体育,听歌,上网,自由,旅游,80 后,电影
游戏动漫,IT 数码,体育,文艺
汽车,体育,IT 数码
旅游,Positive,HxC,BoomBap,冇銀用
代购 yanhee,附诊疗卡和发票,时尚,旅游,星座命理
美女
时尚,旅游,美食,文艺,娱乐
服装配搭,处事之道,育儿专家,婚后生活,性感妈妈,情感分享

听音乐,自然,文艺
音乐控,强迫症,学习,青春,淡定,随性,胡思乱想,幻想,神经质,娱乐
娱乐,旅游
音乐,旅游,旅行,电影,80 后
体育
景德镇美食,景德镇同城会,景德镇娱乐,景德镇资讯,景德镇
娱乐
名人明星
旅游
穿衣美容
军事,美女,汽车,时尚,星座命理
汽车
旅游,新闻资讯,美食,体育
文艺,军事,IT 数码,游戏动漫
IT 数码,健康,军事,星座命理
旅游
视频音乐
文艺,旅游,时尚,星座命理,娱乐,设计,艺术,大学生,摄影
星座命理,旅游,游戏动漫,汽车,美女,军事
美食,文艺
微博奇葩,Diorsman
旅游
求勾搭求抱走,苦逼日语系学生,二次元伪少女,辣椒才是本命,文具控,手控声控制服控,宅腐双修干物女
星座命理
娱乐,体育,汽车,健康,旅游
旅游,健康,娱乐,美食
淘宝,IT 数码,娱乐
体育
美食,新闻趣事,广东生活
体育
旅游
旅游,美食
美剧,大学生,90 后,旅游,电子商务,美食,小说,设计,网购,宅
书籍,睡懒觉,瘦身,记者,搞怪,服饰,喜欢听歌,天秤,时尚,娱乐
旅游,美食
健康,星座命理,听歌,狮子座
美食,旅游,视频音乐,穿衣美容,重庆生活
美食
IT 数码,体育资讯,搞笑幽默
娱乐,IT 数码,吃,跳舞,睡觉
美食
旅游,汽车
历史,哲学,反战,诗歌,佛学,读书

娱乐
汽车,时尚,旅游,美食,体育
我爱 UC 浏览器
美女
娱乐
美食,汽车,游戏动漫,体育
美食
新闻趣事
电影,音乐,旅行,怪咖,吃货睡货
幸福,健康,娱乐
时尚
音乐,传媒,时尚
教育就业,文艺,旅游,健康,美食,游戏动漫,爱动漫和小说
游戏动漫
美女,旅游,娱乐,体育
闷骚,時尚,梦想,重口味,90 後,胡思乱想,漫画,创业,敏感,猫咪控
矛盾纠结体,小哥本命,法律,杂物收藏,Sakura 一生堆,强迫症,英格兰,射手女,HP 一生堆
IT 数码
健康
美食
娱乐
娱乐
汽车,星座命理,文艺
军事
美女
教育就业,投资理财,星座命理,美食,文艺,80 后
军事,羽毛球,新闻,大学生,阅读,历史
八卦,旅游,美食,宅女
娱乐,时尚
心理学,营销,交友,幽默,电子商务,移动互联网,领导力,团队管理,旅行,看书
军事,IT 数码,文艺,汽车,时尚
体育,文艺
健康,汽车,IT 数码,军事,音乐,旅游,电影,美食,80 后,手机通讯从业者
没品无节操,我爱苏打绿,双射手,指甲控,大爱青峰,骨灰级钢丝,闹腾,哆啦 A 梦,旅游,美食
新闻资讯,投资理财,汽车,IT 数码
健康,投资理财,军事,汽车,新闻资讯,星座命理,娱乐,美女
时尚,汽车,美食,旅游,健康
体育
时尚,娱乐
美食
文艺
娱乐,军事,美女
乐观,投资理财,财经,电子商务
真三,LOL,听音乐

健康,旅游,美食
跳舞,动漫迷,美食爱好者,90后非脑残,cosplay,爱睡觉,校园,娯樂
名人明星
创业,娱乐,睡觉,旅行
媒体,设计,IT,90后,电影,网络,好书,互联网,SEO,网络营销
娱乐,游戏动漫
星座命理,青岛生活
新闻资讯,汽车,军事
计算机
语录,财经,交友,时尚,羽毛球,射手座,乐观,搞笑,上网,旅游
美食,旅游,健康,新闻资讯,投资理财
汽车,新闻资讯,教育就业,时尚,美女,旅游
旅游
旅游
娱乐
偶喜欢玩
美女
美食
星座命理,旅游,健康,美女,娱乐
平常心,搞笑,幽默,旅游,互联网,生活
投资理财,美食,娱乐
搞笑幽默
读书分享,科学探索,星座运势,视频音乐,校园生活
文艺
二,文艺,屌丝
宅,美食
电影
时尚,汽车,旅游,文艺,体育
天枰座,喜欢听歌,摄影爱好者,强迫症,音乐控,宅,萝莉控,游戏动漫
文艺,体育,娱乐
旅游,美食,投资理财,娱乐
娱乐,旅游,星座命理,美食
台湾生活
旅游
教育就业
投资理财
时尚,星座命理,娱乐
美食
时尚,旅游,星座命理,美食,娱乐
健康,旅游,教育就业
芬兰生活
娱乐,IT数码
游戏动漫,IT数码,娱乐,文艺,体育
微博奇葩

美食,星座命理,旅游,娱乐
游戏动漫,美女,旅游,美食,体育
IT 数码,投资理财,旅游,星座命理,娱乐,吃货,搞笑,生活,乐观,狮子座
新闻趣事
欧美音乐,电影爱好者,小说,生活大爆炸,美食,新闻资讯,游戏动漫,体育,娱乐
体育
学生,开朗,低调,90 後,吃货,乐观,交友,星座命理,美食,IT 数码
旅游,星座命理,美食
投资理财,汽车,健康,新闻资讯,军事
旅游
新闻资讯,文艺,IT 数码,游戏动漫,娱乐
心理学,wow,美食
文艺,游戏动漫,星座命理,娱乐
喜欢睡觉,运动,爱唱歌,听音乐
新闻资讯,教育就业,军事,体育
文字,囧,娱乐,完美玩赏!
星座命理,搞笑幽默,新闻趣事,视频音乐,微博奇葩
健康
宅星人
体育
时尚,汽车,文艺,旅游,美食
文艺,娱乐,体育
文艺,体育
时尚,美食,娱乐,星座命理
汽车,旅游
旅游,教育就业,文艺
美食,社工,射手座,自由,写作,自由职业,生活
娱乐
投资理财,汽车,时尚,文艺,娱乐
公益,吃喝玩乐,梦想,阳光,五月天,爱睡觉,爱生活,音乐达人,DIY,Android
IT 数码
福州生活,新闻趣事,体育资讯,IT 数码,美女
视频音乐
淘宝,购物,Music,心理,粉红控,健康,语录,美食,星座命理,娱乐
我爱 UC 浏览器
搞笑幽默
北京生活
文艺
游戏动漫,美女,文艺,娱乐
小清新,喜欢温柔与漂亮,讨厌作,幻想自由,幻想主宰地球,腼腆中一丝奔放,有点帅,美女
游戏动漫,随性,乐天派,看书,娱乐,天蝎座,旅游
银行,个人金融,投资理财
美食,宅女,旅行,睡觉,音乐,动漫,电影
娱乐,文艺,旅游,教育就业,健康

艺术
名人明星
游戏动漫
体育
时尚,教育就业,旅游
美食,旅游
旅游
投资理财,时尚,文艺,美食,旅游,星座命理
娱乐
我爱 UC 浏览器
我爱 UC 浏览器
射手,小说迷,懒人,游戏动漫
美食
赶潮
IT 数码
美女,娱乐
娱乐,旅游,体育
时尚,美食,体育
娱乐
文艺,汽车,跑步,养生,台球,喜欢音乐,休闲,书画,文学创作,诗词歌赋
动漫,狮子座,游戏,偶稀饭睡觉觉,好性格,乐观,网购
游戏动漫,美食
时尚,军事,健康
恐怖片,时尚,游戏动漫,星座命理,娱乐,IT 数码
IT 数码
旅游,汽车
旅游,美食
投资理财,体育
娱乐
美女,游戏动漫,文艺,娱乐,健康
娱乐
新闻资讯,健康,旅游,美食
汽车,新闻资讯,投资理财,健康
军事
IT 数码,美女
IT 数码
旅行
投資理財,IT 數碼,軍事,美女,娛樂
新闻资讯,吃,吃货,旅行,羽毛球,摄影,80 后,好书
安静,睡觉睡到自然醒,摩羯,动漫迷,90 后非脑残,大学生
新闻趣事
时尚
美食,星座命理,时尚,娱乐
体育

投资理财,新闻资讯,时尚
细田义彦,神木隆之介,KARNEVAL,RSP,yui,三浦春马,长泽雅美,8059,DMMD,DGM
美女,体育,旅游,新闻资讯,军事
河源生活
邱彬虹
军事,汽车
军事
体育,美女
旅游
美女
娱乐
八卦杂谈
90後,睡觉,上网,旅行,学生,陈奕迅,摩羯女
广西生活
时尚
IT数码,美食
美食,新闻趣事,搞笑幽默,微博奇葩,穿衣美容
美女,网络,营销,自由,摄影
美女
写作,好书,美女,投资理财,旅游
健康,文艺,旅游
汽车
汽车,文艺
时尚,健康
发呆,电脑,淡定,爱唱歌,学生,上网,体育,教育就业
星座命理
投资理财,军事,汽车
美食,星座命理,投资理财,娱乐
旅游,投资理财,文艺,新闻资讯
星座命理,美食,娱乐,汽车,体育
美女,旅行
90后,吵闹,安静
日漫,声优,神经病好勾搭,SPYAIR,天枰座
文艺
喜欢听歌,汽车,旅游,投资理财,IT数码,体育
IT数码
新闻资讯,投资理财,美女,汽车,体育
搞笑幽默,80后
IT数码
军事,投资理财,汽车,健康,美女
美食
新闻趣事
文艺,网络,摄影爱好者,英语,摄影
美女,汽车,体育

教练,机动车,培驾
旅游
美容服饰,读书分享,美图摄影,美食,健康,教育就业,旅游,星座命理
美食
旅游,体育,IT 数码,汽车,美女
文艺
双鱼
IT 数码,旅游,健康,娱乐,星座命理
游戏动漫
娱乐
体育
文艺,旅游,汽车,美女,娱乐
美食,投资理财,汽车,时尚,旅游
旅游,多愁善感,神经大条
湖北生活
搞笑幽默
文艺
IT 数码,宅,90 后,学生
教育就业,军事,美食,80 后,摄影,宅,时尚,旅游,电影,音乐
旅游,勇敢,执迷不悔,倔强,行走,热爱生活,西藏,天蝎座,狮子座,睡觉
旅游
美术,幽默,教育就业,游戏动漫,汽车
美女,时尚,娱乐,平常心,学生
视频音乐
美女,90 后
极简,白羊,80 后,恐怖片控,宅,不能见光,重口味
名人明星
体育,游戏动漫,娱乐
偶喜欢玩,设计,双子座女生,游戏,上网,美食,听歌,80 后
游戏动漫,寻找一颗心
健康
娱乐
新闻趣事
旅游
旅游,教育就业
娱乐,游戏动漫
娱乐,自由
搞笑幽默
旅游,美女,星座命理,IT 数码,时尚
美食
双重性格,科技,健身,学生一枚,古灵精怪,自信,梦想,爱自由,思考,文艺
意大利,欧美音乐,佛教,纠结,书籍,星座,吃货,活泼,爱睡觉,钢琴
IT 数码,投资理财
处女座,90 后,旅游

爱美食,superjunior
博客,医生,聊天,天秤座,发呆,股市
体育
90,懒人,坚强,睡觉
时尚,教育就业,美食,IT 数码
旅行
文艺,汽车,体育,娱乐
美食,唱歌,游戏,NBA,宅,玩儿,网购,足球,篮球,听歌
善良,交友,宅女,旅游
健康
法律,F1,O 型血,金牛,懒,看电影,汽车,美食
旅游,投资理财,教育就业,汽车,体育
时尚,娱乐,文艺,投资理财,体育
IT 数码
美食,123,自由,设计,旅行,時尚,电影,射手座
逛街
宠物,吃货,文艺,旅游,星座命理
美少女的谎言,轻度强迫症,整理控,小清新,吸血鬼日记,RunningMan,Virgo
体育
阳光,爱睡觉,爱生活,单身,娱乐
投资理财,美女,汽车,体育,娱乐
广东生活
80 後
教育就业,投资理财,军事
上网,睡觉,自由,工作,科幻,男人,爱笑,摩羯座 A 型血
游戏动漫
娱乐
星座命理
健康,游戏动漫,时尚,IT 数码,文艺
IT 数码
体育
时尚
IT 数码,文艺,体育
学生,善良笨笨的女孩,悲观主义,金牛座,溢声军,吃,音乐,宅,幽默,90 后
IT 数码,娱乐
文艺,投资理财,旅游,美食,体育
旅游,美食,游戏动漫,娱乐
新闻趣事,新闻资讯
IT 数码
夕阳西下,葬爱
体育,汽车,90 后
星座命理,娱乐
IT 数码
汽车,军事,IT 数码,体育,娱乐

教育就业,旅游,IT 数码,体育,娱乐
美食,健康,旅游,军事,文艺
游戏动漫
美容服饰
教育就业,水瓶座,听歌,90 后
IT 数码
美女,旅游,IT 数码
夜猫子,摩羯座,JayPark
美食
汽车,旅游,IT 数码,美食
星座命理,美食,旅游,健康,文艺
文艺
搞笑幽默,美食,穿衣美容,新闻趣事,微博奇葩
美食,旅游,搞笑幽默,美女
一些事一些情,搞笑,幽默
天蝎座,播音主持
穿衣美容
IT 数码,上网
新闻趣事
新闻资讯,健康,时尚,投资理财,美食
娱乐
美女,娱乐,IT 数码
美食
我爱 UC 浏览器
篮球,休闲
IT 数码,游戏动漫
时尚,汽车,旅游,军事,体育,学习,爱玩
八卦杂谈
新闻资讯,汽车,旅游,体育
萌宠,美食,福医基础医学院,大学,大学生,三下乡社会实践
文艺,投资理财,美食,旅游,娱乐
娱乐,微博,天秤座,乐观,吃,小说,睡觉,善良,90 后
美食
健康,文艺,娱乐,自由,旅行,旅游,80 后,听歌,电影,音乐
时尚
旅游
美食
123
旅游
视频音乐
投资理财,美食,游戏动漫,健康,旅游
IT 数码
旅游
军事

育儿百科
军事
旅游
美食,旅游,穿衣美容,微博奇葩
健康
美食,投资理财,教育就业
那些兄弟,亲情,好书,文学,自由
汽车
娱乐,美女,游戏动漫,新闻资讯,军事
文艺,时尚,教育就业,旅游,美食
美食,体育
动漫,卖腐,萌小受寻觅小倌,爱仙剑古剑轩辕,萌小梦行云,小蛮天明瑾轩控,爱幻三基三,稀饭唯美风,古风控,爱插画
美女,美食,旅游,健康,文字,电影,偶稀饭睡觉觉,双鱼座,宅男,历史
旅游
仙剑,游戏动漫,上网,阅读,小说
娱乐
王道文初涉,勋鹿灿白,二金土民,李泰民,鹿晗,SHINee,EXO
娱乐,星座命理,旅游,美食
新闻资讯,IT 数码,投资理财
体育,星座命理
IT 数码,微博,双鱼座,游戏,睡觉,看书,动漫,上网,音乐,80 后
时尚,健康,旅游,IT 数码,体育
视频音乐,旅游,新闻趣事
文艺,星座命理,健康,IT 数码,游戏动漫
美食,时尚,娱乐,旅游,健康
时尚,美食,文艺
新闻资讯,教育就业,军事
喜欢唱歌,90 后但我不脑残,水瓶,古典音乐,音乐控,o 型血,唱 K,原创,时尚,音乐
健康
旅游,星座命理,军事,娱乐,体育
美食,美女
美食
IT 数码
新闻趣事
星座命理,美食,健康,娱乐,摩羯座,网购,善良,宅女,旅行,80 后
游戏动漫
娱乐,文艺,吃货,90 后,射手座
星座命理
美食,文艺,游戏动漫
娱乐,学生
汽车,时尚,军事
游戏动漫
文艺,美食,教育就业,投资理财,美女

足球,新闻,手机,学生
视频音乐,美女
JOMO,插画,彩铅,橡皮章
健康,美食,娱乐
体育
体育
旅行,水瓶座,听歌
体育
模特,房地产,欧美,摇滚,歌剧,白羊座
美食
青春,時尚,90後,穿衣美容,自然,小清新,天蝎座
IT数码,美食,娱乐,体育,健康
美女,旅游
双子座
娱乐
健康
网购,搞笑,看小说,服饰,吃喝玩乐,新闻资讯,健康,时尚,文艺,娱乐
读书
星座运势,美容服饰,八卦杂谈,新闻趣事,搞笑幽默
时尚,教育就业,IT数码
时尚,IT数码,文艺
健康,教育就业,美食
硬件,好脾气,勇敢,旅游,西安
文艺,娱乐
行政,双子座,平常心,大学生,80后
旅游,美食,军事,IT数码,体育
投资理财
心理学,恐怖片,摇滚,喜欢音乐,爱睡觉,爱生活,善良,游戏,文艺
摄影爱好者,阅读,旅游,电影,吃货,好书,偶喜欢玩,自由
视频音乐
IT数码
健康,IT数码,星座命理,旅游,酒店建筑控,吃货,90后,双子座,好奇,有好玩的
投资理财,旅游,星座命理,美食,IT数码
教育就业,健康,旅游,军事,IT数码
投资理财
时尚,旅游
娱乐,美食,懒人,猫,偏执狂,电视剧
IT数码,军事,游戏动漫
旅游
旅游
体育
名人明星
名人明星
IT数码

校园, 美女, 足球, 爱玩, 学习
旅游
美女, 视频音乐, 搞笑幽默, 影视迷, 运动狂, 80 后, 不懂锅
军事
投资理财, 美食, 娱乐, 文艺, 时尚
搞笑幽默
游戏动漫
汽车, 旅游, 娱乐
旅游, 健康, 美食
新闻资讯, 健康, 教育就业, 文艺, 投资理财
微博, 手机, 旅游, 新闻资讯, 军事, 汽车, 游戏动漫, 美女, 美食, 娱乐
美食, 健康
萌宠
江苏生活, 搞笑幽默
随性, 梦想, 愤青
文艺, 娱乐
旅游, 文艺
动漫
善良, 偶稀饭睡觉觉, 金牛座, 自由, 90 后但我不脑残, 向日葵, 好书, 大叔控, 乐天派, 美食
文艺, 旅游, 娱乐, 美食, 星座命理
新闻趣事, 微博奇葩, 搞笑幽默
游戏动漫, 天蝎座, 游戏, 音乐, 动漫
娱乐, 金融, 活泼, 快乐, 美食控
军事
美图摄影, 八卦杂谈, 搞笑幽默, 情感生活, 星座运势
新闻资讯
看书, 唱歌, 处女座, 新闻资讯, 汽车
美食, 文艺, 娱乐
文艺, 星座命理
汽车, 旅游, 健康, 新闻资讯, 美食
投资理财, 教育就业, 健康, 旅游, 美食
品牌, 体育
IT 数码, 碼畜, 宅男
旅游
IT 数码
投资理财, 军事
吉他, 旅游, 90 後, 美食, 娱乐
旅游摄影, 音乐控, 电影迷, 幻想, 巧克力
IT 数码
美女, 投资理财, 体育
教育就业, 娱乐, 文艺, 游戏动漫
旅行, 宅, 电影, 90 后, 学生, 旅游, 听歌, 音乐
娱乐
星座命理, 80 后

新闻资讯,文艺,娱乐,时尚
娱乐
海贼王,陈奕迅,旅游,体育
体育
星座命理,美食,军事,IT 数码,体育
美食,穿衣美容
美女,娱乐,旅游,IT 数码,体育
游戏动漫,娱乐,美食
水瓶,美剧控,好书,巴萨,互联网,文字,美女,NBA,宅男,水瓶座
搞笑幽默
星座命理,文艺
旅游,听歌
健康,文艺,IT 数码,娱乐
体育,汽车
旅游
星座命理,美食
似水往昔浮流年,音乐
90 後,麦霸,北京,喜欢唱歌,唱 K,网球,欧美音乐,时尚,娱乐
投资理财,新闻资讯,文艺,吃,偶喜欢玩,唱歌,旅游
美食
娱乐,IT 数码,健康,新闻资讯,美女
体育,娱乐,游戏动漫
投资理财,新闻资讯,军事,文艺,体育
文艺,娱乐,美女,IT 数码,游戏动漫
新闻资讯,游戏动漫,IT 数码
投资理财,星座命理,文艺,美女
搞笑幽默
美食,新闻资讯,投资理财,汽车,娱乐
美女
娱乐
宅
IT 数码
游戏,girl
文艺
美女
IT 数码,旅游,美食,搞笑幽默
体育
体育
文化,明朝,思想,历史,新闻,文字,创意,沉默,人生
新闻资讯,时尚,投资理财,健康,美食
旅游,文艺
文艺,星座命理,健康,娱乐,双鱼座
我爱 UC 浏览器,听歌,电影
旅游,时尚,娱乐,美食,健康

娱乐
旅游,时尚,健康,美食,星座命理,娱乐
旅游
星座命理,美食,娱乐
新闻资讯,投资理财,80后
美食,娱乐
新闻趣事,神经大条,nba,强迫症,爱笑,历史,白羊座,宅女
IT数码
教育就业,享受生活,缘分
娱乐
美食,宅女
投资理财,美食,文艺,游戏动漫,健康
娱乐
体育
旅游美食爱好者,神经大条,美食
体育,教育就业,新闻资讯,IT数码,微博,阅读,善良,巨蟹座,动漫,旅游
体育,汽车,军事
汽车,军事
听歌,宅男,奋斗ing,学生
IT数码
星座命理,娱乐
旅游
健康,新闻资讯,体育
体育,文艺,军事,狮子座,看书,睡觉,宅,音乐,学生
搞笑幽默,微博奇葩,视频音乐,旅游
旅游,美食,汽车,美女,体育
汽车
名人明星,搞笑幽默,视频音乐,SOUL 自由,MOIVE,Fashion,MUSIC,双重人格,冷与热,慈悲猿猴
文艺,健康,娱乐
视频音乐,新闻趣事,搞笑幽默,美女
双重性格,完美主义,诗词,葡萄牙,小清新,处女座,赛车,皇马,写作,历史
老鸨,老妖,古风,cosplay,BIGBANG,学生,睡觉,自由,游戏动漫
不知道说什么…
死猪不怕开水烫,双子座
上海生活
新闻资讯,军事,教育就业,汽车,投资理财
旅游,SeanCody
旅游
偏执狂,les,旅游,健康,美食,星座命理
娱乐
旅游
汽车,IT数码,文艺,体育,娱乐
旅游,汽车,体育,娱乐

名人明星
投资理财,娱乐,美食,文艺
搞笑幽默
网络,手机,android,喜欢音乐,玩,时尚,娱乐,汽车,体育,IT 数码
冒险心细加自信
美食,星座命理
游戏动漫
健康
美女
天秤座,天天向上
新闻资讯,健康,投资理财,IT 数码,娱乐
旅游
美女,美图摄影,旅游,中山生活
狮子座,喜欢听歌,夜猫子,旅遊
唱 K,玩,美食控,吃货,幽默,b 型血
旅游
微博奇葩,视频音乐,新闻趣事,搞笑幽默,美女
星座命理,减肥,吃,大学,学生,宅,动漫,电影,偶稀饭睡觉觉,旅行
爱睡觉,乐天派,k 歌,爱自由,善良,美食
星座命理
娱乐,美食
娱乐,文艺
新闻资讯,健康,文艺,体育,娱乐
旅游,星座命理,投资理财,新闻资讯,健康
美食
体育,IT 数码,文艺,投资理财,新闻资讯,娱乐
星座命理,旅游
穿衣美容
时尚,娱乐,美食,体育,旅游
Hannah,Rachel,Aaron,Gwin,Sam,Hill,Gee,Atherton,Chris,Akrigg
美食
汽车,旅游,星座命理,美食,时尚
文艺,体育,汽车,旅游,美食
好性格,吃货,数码,学生,美食,吃,语录,互联网,新闻,电影
媒体,射手座,生活,篮球,交友,睡觉,旅游,90 后
旅游
汽车
天秤女,随性,多愁善感,逛街,热爱生活
IT 数码,杭州生活,美女,健康养生,旅游
搞笑幽默
文艺,美食,旅游,时尚,投资理财
江苏生活
体育
新闻资讯,健康,美食,星座命理,娱乐

投资理财
时尚,星座命理,游戏动漫,娱乐,文艺
体育,娱乐,大声笑,音乐
美女,动画,时尚潮流,手机控
新闻资讯,美食,旅游,汽车,体育
汽车,游戏动漫,美女,美食,体育
布袋戏,欧美 CP,EC,Spirk
旅游,健康,时尚,投资理财
游戏动漫
汽车,星座命理
新闻资讯
A 股,股票
游戏动漫
美食
文艺,体育,娱乐
娱乐,剑网三,九阴,galgame,TVGAME
军事
表演,媒体,旅游,90 后
星座命理,健康,投资理财,军事,美食,新闻资讯,旅游
涂鸦鞋,双子座,艺术,交友,动漫,篮球,听歌,音乐,学生,90 后
美食,星座命理
新闻资讯,美食,体育,投资理财,军事,娱乐
娱乐
旅游,广东生活
IT 数码,游戏动漫,美女,旅游,星座命理,美剧,电影,文学,听歌,射手座
IT 数码
爱情,游戏,手机,偶稀饭睡觉觉,金牛座,音乐,美食,交友
游戏动漫
星座命理,研究生,射手座
健康,时尚,星座命理,美食,娱乐
旅游,时尚,星座命理,美食,娱乐
汽车,娱乐,美食,体育,游戏动漫
我爱 UC 浏览器
体育
美食
美女
IT,主持人,电影爱好者,交朋友,80 後,军事,美食,教育就业,新闻资讯,娱乐
穿衣美容,美食,旅游
穿衣美容,美食,山东生活,名人明星,新闻趣事
爱吃,打篮球,旅游爱好者,音乐发烧友,天秤,科幻,90
美食,美女,IT,学生,艺术,网购
健康
汽车,美女,美食
美食,IT 数码,时尚,旅游,娱乐

健康,时尚,旅游,IT 数码,文艺
阳光,语录,武汉,平常心,桌球,双鱼座,生活,萌宠
紫色,体育,美女,篮球,c 罗,美菱格
IT 数码,健康,旅游,娱乐,新闻资讯
宅,旅游,文艺,时尚,美食
娱乐
男人,90,活泼开朗,时装,浪漫,游戏动漫,旅游,星座命理,美食,娱乐
旅游,文艺,IT 数码,体育,娱乐
搞笑幽默
体育,学生
投资理财,美食,军事,IT 数码,旅游
屎蛋,80 后
娱乐
娱乐
游戏动漫,娱乐,旅游,美食
健康,汽车,时尚,美女,娱乐,90 后但我不脑残,喜欢音乐,淘宝控
健康
美食
汽车,投资理财,新闻资讯,旅游,军事,美食
星座命理,IT 数码,投资理财,美女,游戏动漫
名人明星
搞笑幽默
懒人,小清新,金牛座
美女,娱乐
微博奇葩,穿衣美容
健康,旅游
新闻资讯,时尚,旅游,美食,娱乐
娱乐,学生,音乐,电影
健康,教育就业,旅游,数码,上网,阅读,大学生,亲情,旅行,90 后
文艺
体育,IT 数码
创业,股票,读书,军事,看电影,经济,时尚,教育就业,投资理财,新闻资讯
文艺,体育,娱乐
体育
海蓝
健康,文艺,IT 数码,体育,娱乐
娱乐,文艺,健康,美食,旅游
娱乐
体育,美女,旅游,IT 数码,娱乐
搞笑幽默,新闻趣事,美女,睡觉,90 后,美食
矛盾,淡定,纠结,策划,自由,旅行,运动,北京,设计
建筑工程技术,旅游,电影,上网,唱歌,90 后
旅游
健康,投资理财,美食,军事,IT 数码

军事
教育就业,旅游,IT 数码,娱乐,投资理财
美食
娱乐,健康,时尚,旅游,美食
游戏动漫
文艺,健康
游戏动漫
兴兴,低调,双子,EXO,爱音乐,唱 k
篮球，健身,漫画
IT 数码,体育
旅游
旅游
自由,踩车,足球,温柔的女人…
娱乐,文艺,IT 数码,体育
美女
旅游
体育,IT 数码,游戏动漫
新闻趣事
名人明星,美女,浙江生活
新闻资讯,天蝎座
文艺青年,帅哥,大叔,白领,双鱼,时尚,星座命理,文艺,娱乐,IT 数码
娱乐
学生,90 后,音乐控,天蝎女,爱魏晨,美食控
健康,投资理财,汽车,娱乐,IT 数码
汽车,娱乐,健康,旅游,新闻资讯
教育就业,健康,旅游,文艺
美女
微博奇葩
旅游,穿衣美容,新闻趣事,广东生活
美女,英国生活
旅游,娱乐
体育,娱乐,IT 数码,喜欢交朋友,乐于助人,有责任心,稳重,校园,学生,80 后
快乐中文网
名人明星
美食,時尚,娛樂,星座命理
微博奇葩,美食,穿衣美容,新闻趣事,东莞生活
淘宝控,服装,流浪,赛车,爱旅游,微博,无聊,收藏,O 型血,登山
汽车,投资理财,娱乐,旅游,IT 数码
游戏动漫
搞笑幽默
时尚达人,美食爱好者,热爱生活,旅游,文艺
新闻,英语,好性格,羽毛球,乐观,交友,幽默,看书,上网,自由
IT 数码
美食,军事,IT 数码,汽车

汽车
游戏动漫,美食,娱乐,IT 数码,体育
旅游
时尚
篮球
行政主管
躁郁症
张杰是唯一,星星爱 JASON,星星,张杰,90 后,狮子座
微博奇葩
IT 数码,娱乐,旅游,星座命理,美食
健康,发呆,阅读,女人,育儿
好吃的。,看电视,逛街
娱乐,IT 数码
美女,时尚,文艺,90 后,芯片
星座命理
星座命理,旅游,美食,娱乐
视频音乐
美食
旅游,新闻资讯,健康,投资理财,体育
游戏动漫
辽宁生活
ChilamCheung,TaylorSwift,AvrilLavigne,KatyPerry,ImagineDragons,FallOutBoy,Bonnie McKee,LeonaLewis
新闻资讯
体育,汽车,美女
美女,体育,娱乐
旅游,体育,篮球,巨蟹座
游戏动漫
娱乐
游戏动漫,娱乐
美食,娱乐,旅游,吃货,善良,上网,旅行,睡觉,唱歌
厦门生活
遊戲動漫,旅遊,新聞資訊,汽車,娛樂
游戏动漫
搞笑幽默,四川生活,视频音乐
旅游,90 后
80 后,听歌,自由职业,读书,善良
文艺
水瓶,奋斗,80 後,潮流,热爱生活,时尚达人,淡定,乐天派,浪漫,健康
萌宠,微博奇葩,佛山生活
文艺,美食,旅游
投资理财,旅游,星座命理
微博奇葩,天津生活
随性,娱乐,健康,星座命理,美食

游戏动漫,美食,娱乐,旅游
星座运势
游戏动漫
体育
旅游
咖啡控,阅读,美容服饰,独立,旅游爱好者,经济,读书,金融,投资理财,文艺
大学,80 後,完美主义,手机,摩羯座,好性格,学生,交友,体育,音乐
娱乐
视频音乐,名人明星,微博奇葩,星座命理,旅游
寂寞 ing,单身,我就是我,水瓶座,幽默,睡觉,交友,90 后
文艺,时尚,健康,旅游,美食
美食,文艺,时尚,旅游,娱乐
娱乐,喜欢炎亚伦,我要考大学!
健康,投资理财,军事,美食,旅游
美图摄影
魔兽世界,动画,学生,妹控,萝莉控,90,cosplay,cosplayer,游戏动漫
旅游
旅遊摄影
IT 数码
感性,浪漫,搞笑,好性格,学生,听歌,90 后但我不脑残,白羊座
音乐,粤语,怀旧,漫画,画画、,高中生,傻,幼稚,90 后
旅游美食爱好者,舞蹈,生活,个性,情感,猫,男人,旅游,娱乐,美食
娱乐
运动，篮球
汽车,体育,微博,运动,篮球,交友,音乐,水瓶座
星座命理,视频音乐,连云港生活
时尚
听歌,双子座,足球,阅读,80 后宅女,时尚,文艺,体育,娱乐
体育,文艺,新闻资讯,美女,游戏动漫
体育
动漫,小说,吃,听歌,手机,音乐,旅行,电影,90 后
美食
听歌,个性,美术,赛车,90 後,寂寞,爱幻想,紫色,文字控,柯南
健康,时尚,汽车,投资理财,美食
教育就业,新闻资讯,投资理财,文艺,体育
美食
穿衣美容
travel,HR,English,spanish,city,owl
IT 数码
IT 数码
时尚,娱乐,军事
旅游,健康,时尚,星座命理,美食
美食
投资理财,文艺

娱乐,美食,旅游
美女,文艺,美食,娱乐,旅游
健康
文艺
时尚,美食,怀旧,80 后,音乐,亲子,游戏,数码,儿童,宠物
星座命理
娱乐
星座命理,娱乐,放假,玩儿
文艺,教育就业,旅游,新闻资讯,体育
旅游,投资理财,娱乐,汽车,体育
自由,90 后,抑郁,宅女,文学,摩羯座,吃货
白羊座,偶稀饭睡觉觉,发呆,文艺,爱音乐,看电影,时尚,旅游,娱乐
视频音乐
穿衣美容,微博奇葩,星座命理
娱乐,IT 数码
视频音乐
军事,游戏动漫
新闻资讯,游戏动漫
文艺,健康,时尚,美食
手机,听音乐,IT 数码,单身 ing,奋斗 ing,宁静
求增肥方法,mentaiko,blackmonkey,灼眼的夏娜,日猫组,日语,游戏动漫
自由职业,睡觉,搞笑,体育,宅男,乐观,自由,宅,音乐,新闻
星座命理,娱乐,投资理财
新闻资讯,美女
超跑控
搞笑幽默
投资理财
星座命理,微博奇葩,搞笑幽默
汽车,娱乐,投资理财,IT 数码,时尚
其他生活
娱乐
旅游
旅游,军事,IT 数码,文艺,体育
星座命理
设计,淘宝,看电影,购物,妖精,文字控,射手,星座控,时尚
养生,宠物,爱生活,居家,時尚,旅游
时尚,娱乐
搞笑幽默
美食,美女,健康,新闻资讯,军事
时尚,投资理财,娱乐,旅游,健康
旅游,新闻资讯,美食,IT 数码,文艺
高中生,留学生,视频,法国,搞笑,网购,双子女,画画,旅游,游戏
懒
宠物图集

重口味
大学生,曼联,午夜游民,摩羯座,足球,摄影
健康,娱乐,时尚,爱美食,爱旅行
搞笑幽默
体育,游戏动漫
游戏动漫,体育,娱乐
游戏动漫,星座命理
游戏动漫
新闻资讯
投资理财,汽车,时尚,美女,娱乐
心情
旅游,美食,穿衣美容,新闻趣事,肇庆生活
美食,星座命理,旅游,健康
投资理财,IT 数码
喝酒唱歌
星座命理,美食,军事
旅游,军事,文艺,健康,娱乐,看书
体育
平常心,唱歌,听歌,电影,玩儿,天蝎座,睡觉,旅行,美食
奋斗,学习,A 型血,温柔,健康,美食,娱乐
汽车,游戏动漫,教育就业
军事,时尚,新闻资讯,健康,娱乐
文艺,星座命理,旅游,美食
健康,新闻资讯,汽车,旅游,娱乐
Eason,乐天派
娱乐
健康,美食,教育就业,时尚,旅游
军事,网络,微博,90 后
IT 数码
美食
美食
健康,文艺
教育就业
名人明星,交友,唱歌,宅,自由,学生
时尚,IT 数码
日本,新闻资讯,旅游,星座命理,美食,文艺
娱乐
穿衣美容,视频音乐,美食,萌宠,旅游
健康
投资理财,旅游,星座命理
新闻资讯,美食,投资理财,IT 数码,体育,80 后
萌宠,河南生活
美食,时尚,游戏动漫
新闻趣事,旅游

美食
旅游
旅游,读书分享,新闻趣事,星座运势
汽车
文艺,体育,新闻资讯,美女,娱乐
体育
淘宝,购物,爱睡觉,健康
K 歌,爱美食,电影爱好者
娱乐,游戏动漫,IT 数码,军事,旅游,星座命理
旅游,IT 数码,美食,健康,汽车
美食,旅游,健康,新闻资讯,汽车
旅游
健康,旅游,美食
购物狂,摄影,吃货一枚,喜欢旅游,时尚潮流,宅女一枚,户外,苹果,交朋友,手机
教育就业,新闻资讯,美女,投资理财,体育
文艺,时尚,健康,美食,娱乐
文艺
健康
摇滚,户外,天枰座,吸血鬼,摄影爱好者,赛车,IT 数码
美食,健康,时尚,服装,化妆师
玩儿,动漫,吃,大学生
学霸,台球,音乐,漫画,旅游,吃货,睡觉,处女座,篮球
萌宠
文艺,打包带
游戏动漫
星座命理
娱乐,投资理财
文艺,星座命理,旅游,游戏动漫,美食
搞笑幽默
美食
健康,汽车,军事
教育就业,投资理财,旅游,文艺
传媒,军事,爱生活,爱自然,汽车媒体人,偶喜欢玩,吃,足球,广告,80 后
旅游
投资理财,旅游,星座命理,美食
星座命理,时尚,投资理财,汽车,娱乐
宅,旅游,电影,音乐
旅游,健康,娱乐,星座命理
搞笑幽默
娱乐
情感生活,搞笑幽默,名人明星,校园生活,美图摄影
看电影,手机控,平常心
渣渣 coser 小透,COSPLAY,伪娘脸,抠脚大汉,萝莉控,不混圈
娱乐,星座命理,美食

军事,IT 数码,美女,美食,汽车
娱乐
投资理财,美食,星座命理,旅游,时尚,处女座 AB 型,乐天
健康,美食
健康
教育就业,健康,旅游,投资理财
美食
娱乐,IT 数码
萌宠,视频音乐,美食,喜欢听歌,90 后但我不脑残,心情,陈奕迅,幻想,吃货一枚,处女座
时常纠结,天蝎座,蓝色控
教育就业,旅游,美食,文艺,IT 数码
星座命理,IT 数码
睡觉睡到自然醒,懒,科比,o 型血,宅,睡觉,爱吃,疯狂,无厘头,努力
时尚,文艺
星座命理
文艺,娱乐
美食,美女,微博奇葩,投资理财,新闻资讯,健康,旅游,文艺
名人明星,金钟大，CHEN,EXO,益达，学生仔
旅游,时尚
旅游,JustinBieber,善良,巨蟹座女生,学生
旅游
美食,萌宠,新闻趣事,穿衣美容,上海生活,懒,爱笑,玩,美食控
娱乐
健康,时尚,汽车,美食
娱乐
视频音乐,体育
文艺,健康,时尚,星座命理,娱乐
军事,电影,娱乐,搞笑,音乐,篮球,旅游
投资理财,游戏动漫,体育
时尚,旅游,健康
美食,健康,汽车,旅游,体育
DC 迷,90 后,摄影,电影,漫威迷,动漫游戏,篮球控,弗老大球迷,火箭迷,Rocketman
汽车
娱乐
旅游
教育就业
旅游
金宝盈,广东实验技工,翠绿首饰
娱乐
八卦杂谈,新闻趣事,校园生活,搞笑幽默
旅游
微博奇葩,搞笑幽默
美食
星座命理

体育
海南生活,穿衣美容,旅游,娱乐,美食,文艺,时尚,健康
穿衣美容,旅游,萌宠,微博奇葩,搞笑幽默
星座命理,文艺
美食,教育就业,投资理财,新闻资讯,体育
旅游,美食,健康,时尚,文艺
游戏动漫,美食,文艺,旅游
新闻趣事,搞笑幽默,广东生活,微博奇葩
生活大爆炸,逛街,志愿者,梦想,学习,文藝,青春,旅游摄影,旅游,美女
军事,游戏动漫,体育,投资理财,汽车,IT 数码,文艺,旅游
镇江生活
娱乐
时尚,妞妞妈
文艺,投资理财,美食
名人明星
cosplay,淡定,敏感,暮光之城,杂志,茶,哈利波特,投资理财,时尚,IT 数码
美食,搞笑幽默,其他生活,旅游
星座命理
美女,IT 数码,文艺,体育,娱乐,听歌,睡觉,看书
IT 数码
旅游
美食,时尚,旅游,游戏动漫
旅游
游戏动漫
搞笑幽默,90 后
娱乐
les
军事
游戏动漫,文艺,娱乐,找找
巨蟹座,平常心,90 后,奋斗
旅游,文学,星座,心情,网络,手机,发呆,思考,单身,看书
穿衣美容,吃
IT 数码
娱乐
游戏动漫,IT 数码,军事,美食,我爱 UC 浏览器
90 后但我不脑残,K 歌,美食,IT 数码,时尚,投资理财
搞笑幽默
搞笑幽默,浙江生活,微博奇葩,美食,旅游
娱乐
娱乐,教育就业,文艺,投资理财,健康,美食
运动,神经质,射手座,睡觉,紫色,语录,发呆,听音乐
IT 数码,美食,军事,汽车,旅游
娱乐
杭州,长河高级中学,校友,校园生活,音乐,美食,看书

玩儿,自由,设计,电子商务,双子座,营销,NBA,宅,80 后,好性格
爱宠,健康,90 后,运动,旅行
动漫,旅游,美食,狮子座,美剧,睡觉,学生
朋迷，德涩社,雪,80 后
喜欢听歌,忧郁,乐观,建筑,方大同,好性格,重口味,怀旧,DOTA,旅游
星座命理,娱乐,体育,文艺,游戏动漫,火影,自然,动漫,上网,学生
文艺,IT 数码,娱乐,水瓶座,考试恐惧症,吃货,宅,数码,美剧,小说
娱乐,自由主义者
美食,搞笑,娱乐,幽默,电影,天蝎座,音乐,美食,旅游,乐观
美女
旅游,K 歌,TVB,粤语,设计师,美术,90 后,学生
微博奇葩,萌宠,搞笑幽默
射手座,神经质
健康,新闻资讯,游戏动漫,星座命理,IT 数码
新闻资讯
时尚,美女,娱乐,IT 数码
美女
单身,计算机,游泳,双子,摄影,学生,新闻资讯,旅游,健康
爱花儿,环游世界,海贼王,旅游美食爱好者,爱幻想,小清新,射手座,吃货一枚,梦想,时尚
音乐爱好者,游戏动漫
旅游
文艺,IT 数码,娱乐
旅游,健康,时尚,娱乐,星座命理
名人明星
视频音乐,旅游,美食,萌宠,星座命理,90 后,吃货,动漫
骑行,历史,文学
工藤新一,李宗翰,美食,旅游
娱乐,体育
旅游,美食,音乐
美女,游戏动漫
新闻资讯,美女,美食,军事,IT 数码
IT 数码,美食,游戏动漫,美女
时尚
旅游,时尚,健康,美食,投资理财
处女座,娱乐,自由,时尚,宅,听歌,90 后,电影,音乐
美食
学生,宅,90 后
投资理财,水瓶座,看书
汽车
80 后,自由,音乐
新闻资讯,旅游,娱乐,双鱼座,旅行,时尚,80 后,听歌,电影,音乐
情绪化,新闻,天蝎女,o 型血,偏执狂,爱吃,海绵宝宝
星座命理
新闻资讯,投资理财,旅游

旅游
时尚,旅游,爱幻想,自由,80 后,汽车,IT 数码,体育,娱乐
美女
旅游
新闻资讯,旅游,娱乐
IT 数码
电影迷,英国生活
投资理财,文艺,健康,IT 数码,体育
萌物控,双鱼座,文艺,娱乐,健康,美食
社会,美女,文艺,体育,娱乐
新闻趣事
投资理财,军事,体育
爱好书法,职业工程师
心理学,低调,房地产,跳舞,健康,时尚,旅游,美食,文艺
电影
IT 数码
搞笑幽默,微博奇葩,湖北生活,萌宠,美食
美女,健康,文艺,娱乐,学生,时尚,宅,90 后,听歌,音乐
娱乐,天蝎座,阅读,紫色,美食,旅行
美食,健康,娱乐,时尚,IT 数码
福建生活
爱许巍,美女,军事,IT 数码,娱乐,喜欢弹吉他,旅游,80 后,听歌,音乐
教育就业,汽车,IT 数码,娱乐,体育
IT 数码
健康,美食,星座命理,游戏动漫,文艺,宅女,双子座,小说,网购,偶稀饭睡觉觉
处女座,完美主义,高三,感性,胡思乱想,文字控,强迫症,淡定,懒人,宅
星座命理,投资理财,娱乐
自行车,梦想,懒人,游戏动漫
文艺,新闻资讯,时尚,健康,娱乐
IT 数码
美食
美食,旅游,狮子座,看书,学生,宅,90 后
游戏动漫,娱乐,体育,幽默,篮球,小说,听歌,宅,学生
鞋子批发,鞋子,淘宝货源,网店货源,代理商,网络分销,批发商,金融,市场营销,shopping
教育就业,美女,体育,我就是我
娱乐
IT 数码
打篮球,玩麻将,股票,干好工作
旅游,美食,娱乐,数码,动漫,听歌
新闻资讯
体育,游戏动漫,汽车
情感生活,八卦杂谈,新闻趣事,搞笑幽默
娱乐
快乐,美术,会计,室内设计,网络营销,新闻资讯,教育就业,投资理财,文艺,美食

美食
美食,时尚,小说,90 后非脑残,内向
美女
健康,旅游
美图摄影,新闻趣事
IT 数码
搞笑幽默
汽车
穿衣美容,搞笑幽默
美女,搞笑幽默,微博奇葩
旅游,广州生活,比萨意粉,牛扒,桌游吧,美食甜品休闲,经典语录,精美小吃,美食甜品,休闲
IT 数码
新闻趣事,微博奇葩,搞笑幽默
美食,健康,时尚,旅游
elf
旅游,美食,健康
爱 K 歌,爱自己,爱街舞,90 后,狮子座
娱乐
旅游,投资理财
娱乐,游戏动漫
新闻趣事
书,电影爱好者,跳舞,萌宠,四川生活
平常心,幽默,旅行,听歌,电影,音乐
江西生活,穿衣美容,名人明星,美食,星座命理
旅游
汽车,IT 数码
投资理财
穿衣美容
生驹里奈,渡边麻友,暗黑破坏神,美食,大学生
微博奇葩
旅游,军事,体育
湖北生活
偶稀饭睡觉觉,善良,奋斗,文艺,时尚
投资理财
健康
女装,淘宝,美女
天蝎座,电影,旅游
美食,旅游,汽车,文艺,体育
穿衣美容
美食
新闻趣事,文艺,体育
汽车,教育就业,IT 数码
新闻资讯,汽车,教育就业,军事,IT 数码
旅游

旅游,健康,时尚,娱乐,美食
福建生活
美食
娱乐
旅游
时尚,旅游,文艺,娱乐,星座命理
军事,美食
游戏动漫,投资理财,星座命理
新闻资讯,美食,文艺,娱乐,健康,爱情,90 后， 白羊座，,自信,阳光,微笑
新闻资讯
新闻资讯
视频音乐
文艺
娱乐
新闻趣事
游戏动漫
新闻趣事,美食,上海生活,星座运势,金牛座,乐观
学生,电影,90 后,80 后
美女,娱乐,教育就业,新闻资讯
娱乐,体育,购物,爱幻想,花痴,执着,服饰,多愁善感,美食狂热爱好者,狮子座女生
动漫,生活,动漫迷
入门级渣 coser,露中,米英,眉毛痴汉
文艺,旅游,娱乐,健康,投资理财
爱旅游,爱电影,跳舞,90 後,音樂,美女
名人明星,视频音乐,音乐控,各种乐,大同迷,直率,邪恶,美食控,怪才
萌宠,新闻趣事,上海生活,旅游,美食
疯子,做梦,宅,腐,爱自由,好性格,旅游,吃货,摄影,双鱼座
音乐
微博奇葩,萌宠,美食,旅游,吃货,旅行,摄影爱好者,足球,90 后
军事
丽水生活
善良,天秤座,阳光,爱生活,music,喜欢旅游,双重性格
搞笑幽默,爷们,開朗
美容服饰,武汉生活
娱乐,靠谱女青年,林宥嘉,曾轶可,爱少年,95 后
懒堕,猫猫。
外貌协会,怀旧,90 后但我不脑残,电脑,交朋友,喜欢音乐,ACG,游戏动漫
IT 数码
娱乐,旅游,健康,美食,体育
娱乐
美食,健康,旅游,文艺,星座命理
美食,体育
IT 数码
双鱼女,执着,感性,宅女,坚强,情绪化,发呆,听音乐

星座命理
游戏动漫
美食,音乐,双子座,宅女,80后
健康,时尚,旅游,星座命理,美食,文艺,娱乐
体育,文艺,IT数码,旅游,娱乐
娱乐
星座命理
江苏生活
娱乐
房产,景德镇生活,八卦杂谈,旅游,搞笑幽默
基督徒,武术爱好者,健美爱好者,音乐爱好者,吉他手,自由主义者,理想主义者
健康,旅游,美食,军事,体育
健康,新闻资讯,教育就业,投资理财,IT数码
时尚
时尚,健康,旅游,美食,娱乐,双子座,乐观,90后
娱乐,音乐
微博奇葩,星座命理,穿衣美容,新闻趣事,搞笑幽默
游戏动漫
旅游
体育,娱乐,游戏动漫
健康,旅游,星座命理,美食,娱乐
美女,游戏动漫,美食,IT数码,星座命理
穿衣美容,旅游
双鱼,游戏,吃,音乐控,游戏动漫
娱乐,文艺,星座命理
旅游,投资理财
不超3月美男控,爱幻想白痴少女,游泳拉丁OK,L阅读与写作,爱听歌唱K,爱旅游,吃货一枚,爱画画,爱exo,偏爱吸血鬼
时尚
投资理财
游戏动漫,体育,二逼青年,起床困难户,hazard,兰帕德,QQ仙侠传,切尔西死忠
文艺,美食,娱乐
旅游,星座命理,美食,娱乐,健康
旅游,星座命理,美食,文艺,体育
旅游,军事,汽车,体育
听歌,宅,时尚,旅游,电影,音乐,旅行,美食,80后,自由
新闻趣事
创意,爱吃,音乐,天蝎女,阅读,旅游,美食,时尚,游戏动漫,娱乐
星座命理,旅游
汽车,IT数码,军事,娱乐
投资理财,健康,汽车,体育
娱乐
美食
健康,旅游,美食,投资理财,时尚

淘宝返利,搞笑幽默,星座命理,美食,新闻趣事,穿衣美容
文艺,汽车,健康,娱乐,美食
美女,时尚,游戏动漫
健康
投资理财
旅游,美食
新闻资讯,军事,美食,游戏动漫,娱乐
时尚,健康,旅游,美食,娱乐
偶喜欢玩,90 后非脑残,发呆,睡觉睡到自然醒,看电视,梦想,爱自由,笑话,游戏动漫
健康,美女,汽车,文艺,娱乐
科幻,青春,低调,安静,聊天,连接服务器失败,唱 k,多愁善感,真诚,交朋友
文艺,投资理财,健康,时尚,旅游
时尚
新闻资讯
投资理财
健康
音乐,巨蟹座,汽车,运动,爱情,大学生
名人明星,八卦杂谈,搞笑幽默
健康,投资理财,美食,体育,汽车
旅游,新闻资讯,IT 数码,美食
茶粉
游戏动漫,军事
娱乐
汽车,旅游,军事,IT 数码
健康,美女,投资理财
时尚,IT 数码,新闻资讯,文艺,体育
新闻资讯,汽车,军事
游戏动漫
视频音乐,搞笑幽默,新闻趣事,青春
搞笑幽默,重庆生活,微博奇葩,美食,旅游,90 后
旅游
搞笑幽默
旅游,游戏动漫,娱乐
IT 数码,时尚,星座命理,美食,娱乐
南京生活
投资理财,时尚,健康,美食,旅游
新闻资讯,经济
文艺,美食,娱乐
美女,美食,文艺,旅游
星座命理,新闻资讯,投资理财
娱乐,星座命理,旅游,时尚,美食
体育,购物
美女,文艺,娱乐,焦宇,大庆 DJ 小宇,大庆金莎 DJ 小宇,DJ 小宇
体育

旅游
娱乐
美女,听歌,80 后,电影,音乐
澄空学园百合组,中文填词,翻唱歌姬,宅女,动漫,听歌,大吃货,美食,音乐
文艺,美食
游戏动漫
旅游,微博奇葩,新闻趣事,视频音乐,搞笑幽默
时尚,文艺
旅游
投资理财,美食,健康,旅游
体育
投资理财
摄影,小说迷,旅游,军事,IT 数码
旅游
新闻资讯,星座命理,美食,IT 数码,教育就业
90 後,多愁善感,k 歌,娱乐
新闻资讯,投资理财,健康,旅游,美食
孩子气,努力,健康
其实喜欢安静,喜欢听歌唱歌,喜欢看书
新闻资讯,游戏动漫,健康,偶稀饭睡觉觉,动漫,小说,宅女,自由,学生,90 后
游戏动漫
健康,方舟子,时尚,旅行
校园生活,00 后,粗枝大叶,旅游控,吃货,爱听歌,爱猫,爱狗,幽默,双子座
旅游,没有特别喜欢的,轻微强迫症,完美主义,天蝎座,90 后
微博奇葩
IT 数码
呃呃,关注我,90 后
美容服饰,爱情,蓝色,感性,星座命理,偶喜欢玩,天蝎座,狗狗,外貌协会
IT 数码,军事,美食,星座命理,旅游
投资理财
听歌,美女
游戏动漫
微博奇葩,视频音乐,搞笑幽默
美女,美食,娱乐,IT 数码
时尚,新闻资讯,文艺,游戏动漫
娱乐
娱乐,文艺,爱电影,单身,旅游爱好者,音乐爱好者,懒
没有自制力,高热量食物,猫系痴汉,独占欲,被爱妄想,空想主义,cos,低产,我会努力
美女
xbox360,halo,游戏动漫
名人明星,新闻趣事,视频音乐,美食,旅游
娱乐,旅游,囧,紫色,文学,生活,搞笑
星座命理,美食,娱乐,至天使米粥,刘洲成,学生,90 后
军事

时尚,健康,投资理财,旅游
美图摄影
旅游
投资理财,时尚,健康,IT 数码,娱乐
军事
平常心,吃,交友,摩羯座,自由,音乐,80 后
游戏动漫
星座命理,娱乐,IT 数码,投资理财,汽车
文艺
星座命理,娱乐
新闻资讯
旅游
美食
健康
80 后,音乐,舞蹈,购物,家居,旅游
健康,旅游,美食,时尚
文艺,新闻资讯,美食,时尚,旅游,健康
新闻资讯
美容护肤,生活,睡觉睡到自然醒,女人,妖精,健康,diy,旅游
军事,IT 数码,美女,游戏动漫,体育
茶叶,装饰设计,广州,野生茶,铁观音,百度,设计,普洱,IFI,番禺
美食,良家少女一枚,旅行,音乐,宅女
新闻资讯
健康
健康,教育就业,美食,投资理财,旅游
广东生活,旅游
游戏动漫,文艺,娱乐,新闻资讯,美食,旅游
美女
紫色,偶稀饭睡觉觉,善良,一些事一些情,天蝎座,80 后
新闻趣事
旅游爱好者,瑜伽,旅游,星座命理,美食,时尚,娱乐
新闻资讯,旅游,IT 数码
新闻资讯,IT 数码,旅游
美食,健康,游戏动漫,旅游,娱乐
旅游,美食,娱乐
美女
手工,佛珠,设计,原创
旅游,美食
旅游,时尚,健康,星座命理,娱乐
执着,旅游摄影,日语,矛盾体,星座命理,文艺,双子座,学生一族,90 后,动漫控
美女,视频音乐,金莎控,赵彤控,球迷,Mr 斗牛士
飙歌,骑行,电影,游戏动漫
数码,汽车,爱情,80 后,美剧,音乐,美食,听歌,电影,旅行
体育

IT 数码,文艺,教育就业,汽车,美食
游戏动漫,健康,娱乐,体育,日系,AKB48,学生
新闻资讯
娱乐,旅游,美食,文艺
学生。
娱乐,汽车,旅游,文艺,投资理财,体育
时尚,打球,双重性格,旅游
爱旅行,淘宝,艺术,游戏,网络,音乐,数码
福建生活
美食,娱乐,电影
体育,军事,IT 数码,文艺,娱乐
旅游,时尚,90 后,美食,电影
投资理财
旅游
美食
美甲
为未来而奋斗,为梦想而努力,雅思,大学生,记录克服历程,奋斗,学习,英语
90 后但我不脑残,superjunior,美食,名人明星,90 后
IT 数码
旅游
视频音乐
美食
娱乐,汽车,健康,旅游,军事,挖掘机,工程兵,工程机械修理,工程机械与维修,工程机械
文艺
美女,军事,IT 数码,体育,投资理财,徐州
健康,旅游,星座命理,美食,文艺
旅游,文艺,星座命理,美食,娱乐
IT 数码,教育就业,娱乐,星座命理,汽车,我爱 UC 浏览器
体育
旅行,听歌,80 后
美食,文艺,娱乐,旅游,体育
00,听歌
军事,美食,体育
Vintage 古着,复古宫廷黑,怪人一枚,美女,时尚,音乐,美食,电影
美女
体育,nba
新闻资讯,教育就业,IT 数码
星座命理,美食,军事,射手座,小说,上网,学生,宅,90 后
投资理财,文艺,汽车,时尚,娱乐,军事,IT 数码
文艺,90 后
新闻资讯,旅游,军事,IT 数码,美女,科技,音乐,电影,美食,旅行
水瓶座,幽默,吃货,海绵宝宝,大学生,健康,时尚,文艺
时尚,90 后,听歌,音乐
穿衣美容

吉他,音乐,沉默,大学生,90 后
传媒,教育,创业,阅读,旅游,新闻资讯
我爱 UC 浏览器
旅游
美女,旅行
体育
宠物图集
Ars,极简崇拜,食色者,奔小白,结构粪青,矛盾体
健康,体育,娱乐
娱乐
拍照摄影,笑點低,摄影爱好者,乐观,平常心,羽毛球,生活,90 后,慢跑,喜欢日语
Vans 控,不穿秋裤,利物浦死忠,mchotdog,张震岳,足球
投资理财
西安生活
睡觉
名人明星
旅游,健康,美食,文艺,好性格,双子座,小说,宅,摄影,音乐
开朗,乐观,有责任心,爱心的 80 后
搞笑幽默,文艺
新闻资讯,军事,IT 数码,体育,80 后
爱睡觉觉,爱购物,爱打桌球
美食,娱乐
学生一族,jason 迷,听音乐,旅游
投资理财,教育就业,汽车,游戏动漫,美女
IT 数码,旅游,甜心妹
美女,星座命理,美食,文艺,体育
星座命理
汽车,IT 数码,新闻资讯,体育,美食,旅游,音乐,80 后
听歌
旅游
萌宠
时尚
健康
时尚
游戏动漫,娱乐
旅游,美女,微博奇葩,视频音乐,萌宠
健康,美食
看电视…
文艺,体育
性情中人
美女
娱乐
粤语,娱乐,汽车,美食,IT 数码,体育
旅游,娱乐

重庆生活,独立,完美主义,活泼开朗,古灵精怪,懒,摩羯,爱自由,喜欢音乐,敏感
文艺,军事
美女
娯樂
美食,时尚,90后,音乐,电影,听歌
美食
美女,美食
健康,旅游,文艺
文艺,90后
投资理财,旅游,健康,新闻资讯,星座命理
夜猫子,矛盾体,傻,美剧迷
体育
微博奇葩
美女,萌宠,搞笑幽默
宅,90后,美食,听歌,音乐
军事,娱乐,时尚,美女
游戏动漫,美食
90后
旅行
我爱UC浏览器
星座命理,时尚
咖啡,小清新,盗墓笔记,好书,校园,阳光,单身,爱旅游,时尚,体育
汽车
宅女
军事,听歌,音乐
时尚,娱乐,旅游,听歌,美食
美女
美食,吃,偶喜欢玩,囧,90后
汽车,游戏动漫,军事,IT数码,体育
旅游,汽车,文艺,听歌,电影,80后,美食,时尚
搞笑幽默
找博哥,15382282776
文艺,android,书籍
美食
星座命理,美食,文艺,娱乐,旅游,音乐,电影,80后
文字控,90,学生,发呆,摩羯座,时尚
投资理财,旅游,美食,文艺,疯啦啦啦,阅读,唱歌,自由,旅行,80后
幽默
单纯,听歌,打篮球,绘画,单身
沟下女仔,唱下K仔,打下波仔
教育就业,IT数码,文艺,娱乐,体育
文艺,美女,教育就业,娱乐,IT数码
健康,时尚,旅游,美食,文艺,寻找酷色王琎
微博奇葩,搞笑幽默,旅游,美食,穿衣美容,快乐,热爱生活,爱电影,自由,巨蟹女

美食,旅游
动漫,搞笑,电影爱好者,高中生,90 后非脑残,写作,爱情公寓,火影忍者,NBA,音乐
名人明星
美女,美食,听歌,90 后
投资理财,新闻资讯,汽车,教育就业,军事
美食,汽车,军事,美女,IT 数码
团魂都被狗吃了,牛灿本命勿拆,取关请双向,其他西皮无节操,牛桃灿白请绕道,文艺得脚踏实地,偏执狂
电影,旅游,80 后,听歌,音乐
投资理财
旅游,美女,新闻资讯
体育
偶稀饭睡觉觉,低调,手机看小说,茶,吃货一枚,盗墓笔记,古灵精怪,宅女加腐女,活泼开朗,游戏动漫
旅游,星座命理,美食,听歌,音乐
八卦杂谈
手机看小说,纠结,手机控
体育
娱乐
旅游,美女,健康
旅游,微博奇葩,穿衣美容,山东生活,90 后,摄影爱好者,旅行,眼镜控,帽子控,女人味的运动风
时尚,健康,文艺
美食
游戏,睡觉,电影,90 后,旅游,美食,音乐
旅游,美女,体育
90 后
八卦,偶稀饭睡觉觉,网购,吃,大学生,交友,宅女,学生,时尚,90 后
时尚,教育就业,80 貓兒
美食
唱歌
汽车
视频音乐,旅游,萌宠,星座命理
新闻资讯
新闻资讯,教育就业,IT 数码,体育,文艺
时尚
音乐,电影
天枰座
体育,娱乐
汽车,星座命理
旅行,旅行控,旅行爱好者,摄影爱好者,摄影控,摄影
文艺,美食,新闻资讯,旅游,体育
娱乐,文艺,健康,星座命理
投资理财,旅游,健康,美食,娱乐

育儿,孩子,妈妈,淘宝
娱乐,投资理财,教育就业,汽车,体育
音乐控,小说控,游戏控,旅游,娱乐,偶稀饭睡觉觉,90 后
名人明星,视频音乐,微博奇葩,宅,90 后,电影,美食,听歌,音乐
健康,时尚,汽车,IT 数码,旅游
娱乐,医学生一只
星座命理
旅游,美食,健康,星座命理,时尚
体育
星座命理,天蝎座,宅女,90 后
80 后
投资理财
夜半歌声
体育
投资理财,时尚,军事,文艺,娱乐
文艺,美食,旅游
文艺,电影,90 后,听歌,音乐
教育就业,健康,旅游
文艺,健康,期待爱,却又怕失去,很纠结的人
投资理财
拍照 k 歌,娱乐圈,文学艺术,完美主义,张杰苏泷控,影视剧,时尚范儿,双鱼女,浪漫主义,音乐达人
游戏动漫
搞笑幽默,美女
视频音乐
文艺
文艺,娱乐,艺术,宅女,白羊女,创意,旅游爱好者,狗狗,格子控,电影
怀旧,生活,乐观,爱情,会计,休闲,健康,旅游,星座命理,美食
旅游,美食
星座命理
娱乐
传媒,听歌,网购,上网
性格开朗,爱好打篮球,。
名人明星,体育,双子座,玩,阿森纳,足球迷,淡定
星座运势
坦克世界,游戏
美女,汽车,文艺,体育,旅游
游戏动漫
努力,自然,偏执狂,IT 数码,听歌
娱乐
游戏动漫,腹黑,请叫我小纯洁
文艺,游戏动漫,娱乐,IT 数码,美食
星座命理
80 后,时尚

时尚,新闻资讯,健康,旅游,娱乐
投资理财,美食,美女,旅游,汽车
美食,娱乐,游戏动漫,星座命理,旅游
喜欢听歌,K 歌,搞怪,BIGBANG,热爱生活,NBA,动漫控
听歌,看电影,宅,玩游戏,音乐
旅游
旅游,音乐,电影,时尚,90 后,听歌
kitty 控,时尚,听歌,旅行,自拍狂,音乐
穿衣美容
我爱刘忻
搞笑幽默
美食,文艺,旅游,星座命理,娱乐
健康,投资理财,娱乐,旅游,美食,文艺
娱乐,游戏动漫
音乐,电影,听歌,80 后,美食,旅游,70 后,旅行,时尚,宅
新闻趣事
90 后
汽车,新闻资讯,美食,时尚,健康
睡觉,夜猫子,美食控,互粉,80 后的尾巴,懒人,爱笑,安静,健康
90 后
新闻资讯,健康,美食,文艺
投资理财,新闻资讯
体育,文艺,旅游
体育
读书,平常心,旅游,90 后,玩儿,娱乐,交友,白羊座,运动,大学生
美女,时尚,宅,电影,听歌,音乐,90 后
萌宠,搞笑幽默,爱美女，哈哈
旅游
体育
游戏动漫,文艺,IT 数码,旅游,娱乐
育儿,美食
投资理财
80 后
美食
内向,纠结,睡觉睡到自然醒,胡思乱想,旅游美食,寂寞,迷糊,80 后宅女,夜猫子,助理
星座命理,体育
汽车,天蝎座,摄影,上网,旅行,90 后,自由,学生,电影,音乐
美女,穿衣美容
吃货,天蝎座,自拍,文艺,听音乐,玩手机,宅女,长不胖
拖鞋,凉鞋,包邮,高跟鞋,单鞋,寻梦女鞋,女鞋
爱旅行,双鱼座,旅游摄影,电影,音乐,宅,旅行,80 后
新闻资讯,旅游,投资理财,美女,娱乐
80 后
旅游

互联网, 紫色, 历史, 浪漫, 音乐, 时尚, 读书
旅游, 视频音乐
摄影爱好者, 偶稀饭睡觉觉, 90 后但我不脑残, 凤凰传奇, 摩羯女, 懒人, 电影爱好者, 林俊杰, 谢霆锋
B 型血, 动画, 90 後, 摩羯座
深圳生活, 搞笑幽默, 美女
赣南医学院, 高安, 于都, 医生
美食, 旅游, 宅
运动, 美食, 90 后, 电影, 音乐
新闻资讯
小说迷, 欧美, 烹饪, 网游, 懒人, 拍照, 厦门, 偏执狂, 喜欢旅游, 古典艺术
游戏动漫, 为了部落, 暴雪亲爹, 联盟收割机, 游荡的食尸鬼, 美剧重症患者
穿衣美容, 名人明星
旅游, 美女, 新闻趣事, 搞笑幽默, 名人明星
音乐
时尚, 汽车, 投资理财, IT 数码, 文艺
旅游
微博奇葩
美女, 羽毛球, 听歌, 自由, 艺术, 营销, 生财有道, 财富之路, 雨燕车主, 丁方宁
服装, @@, 生活
美食, 游戏动漫, 文艺
美食, 旅游, 时尚, 娱乐, 文艺
80 后
教育就业, 时尚, 文艺, 娱乐, 星座命理
美女, 健康
文艺, 美食, 投资理财, 健康, 星座命理
爱逛街
汽車, 遊戲動漫, 美女, 軍事, IT 數碼, 90 后
美食, 旅游, 我不喜欢束缚, 但我喜欢简单
娱乐, 教育就业, 投资理财, 时尚, 文艺, 听歌, 宅, 90 后
北京生活, 穿衣美容, 星座命理, 旅游, 萌宠
星座命理, 大学生, 网购, 宅
健康, 旅游, 星座命理, 美食, 娱乐
星座命理
体育
美容服饰, 美图摄影, 情感生活
80 後, 唱 k, 喜欢听歌, 狮子座, 瘦身, 真诚, 愤青, 囧, 狗狗, 娱乐
娱乐, 旅行, 电子商务, 阅读, 新闻
健康
娱乐, 音乐
上海生活
电影, 90 后, 美食, 听歌, 音乐
电影　小说
新闻趣事

体育
琴棋书画
美食
阳泉生活
旅游
美女,时尚
星座命理,汽车
钓鱼,睡觉,唱歌,动漫,旅行,军事,文艺,IT 数码,投资理财,体育
伪宅真腐,黑长直的男人控,二次元症候群,美食控,大叔控,伪文青,小说
游戏动漫
军事,汽车,美女
娱乐
减肥,旅游,时尚,美食,90 后
新闻资讯,健康,旅游,美食
视频音乐,林宥嘉,音乐
BJD
萌宠
投资理财
减肥中,爱养生,会生活
美食,游戏动漫,美女,IT 数码
游戏动漫,淘宝控,外貌协会,吃货一枚,美食控,漫画,90 后但我不脑残,自恋,胡思乱想,矛盾体
IT 数码,90 后
宅,时尚,旅行,旅游,美食,电影,听歌,音乐
娱乐
新闻趣事,宠物图集,美食,北京生活,搞笑幽默
穿衣美容,旅游,美食
娱乐,文艺,投资理财,新闻资讯,星座命理
不要在哥面前 ZB,哥是 gay 啊!
时尚,汽车,旅游,IT 数码,体育
通辽生活,美食,视频音乐,新闻趣事,穿衣美容,内蒙古生活
新闻趣事
名人明星,娱乐
娱乐
电影,音乐,游戏
懒人,军迷,摩羯座,90 后,温和
娱乐
IT 数码
时尚,文艺,宅
搞笑幽默,微博,云川台球,水瓶座
游戏动漫
美女
读书分享,IT 数码,旅游,美容服饰,名人明星
80 后

旅游
我爱 UC 浏览器
时尚,教育就业,娱乐,文艺,旅游,宅,90 后,听歌,音乐
娛樂,复古,时光机
教育就业,新闻资讯,旅游,健康
体育,动漫
体育,IT 数码
文艺,美食
自由自在,自由
IT 数码
旅游,微博奇葩,新闻趣事,搞笑幽默
娱乐
星座命理
娱乐
新闻趣事,旅游,广东生活,名人明星,热爱生活
美食
VIP,双子座,少女时代,bigbang
娱乐,美食,游戏动漫,健康,星座命理
白羊女,cosplay,hiphop,动漫,90 後,爵士,韩国
汽车,娱乐
娱乐,八卦
偶稀饭睡觉觉,吸血鬼,宅男,金牛,夜猫子,听音乐,猫控,手机,90 后非脑残,游戏动漫
娱乐,汽车
体育
美食,河北生活,穿衣美容
IT 数码,美食,90 后
娱乐
旅游,美食,穿衣美容,星座命理
追星,90 后,微博
娱乐,美女,健康,军事,IT 数码
体育,旅游,电影,宅,听歌,音乐,90 后
投资理财
游戏动漫
美食
喜欢音乐,音乐控,爱美食,奋斗
福建生活,视频音乐,微博奇葩,搞笑幽默,旅游
星座命理,电影,听歌,90 后
星座命理,美食
音乐
美食,文艺,体育,投资理财
时尚,投资理财,旅游,体育,美女
美食,健康,星座命理,体育,娱乐
闽台交流,烘焙,校园生活,福建生活,美食
美食

旅游,健康,美食,文艺,投资理财
文艺,写作,阅读,文学,读书,艺术,听歌
体育
新闻资讯,军事,IT 数码,互联网,文字,新闻,体育
旅游,时尚,音乐,开朗的我
新闻资讯,健康,旅游,文艺,娱乐
游戏动漫,IT 数码
健康,旅游
教育就业,军事
健康,投资理财,星座命理
美食
新闻趣事,星座命理,搞笑幽默
四川生活,视频音乐,旅游,美食,萌宠
校园生活,美食,厦门生活
火影忍者,游戏动漫
美食
天平座,听歌,90 后,偶稀饭睡觉觉,看电视
凌晨看美剧,AJ,喜欢交朋友,NickiMinaj,太他妈聪明了,sneaker,子弹头男爱好者,NBA,自助游狂热份子
娱乐
游戏动漫
美女
搞笑幽默
游戏动漫
游戏动漫,军事,IT 数码
音乐,处女座
旅游,健康,娱乐,汽车,美女
处女座,亲情,宅女,学生,90 后
体育,时尚,旅游,美食,IT 数码
做个全职妈妈
宅,美食,旅游
旅游,投资理财
微博奇葩,星座命理,视频音乐,新闻趣事,湖北生活
时尚,娱乐
娱乐
时尚,体育,娱乐,美食
娱乐,广州市,广州
投资理财,文艺
体育
旅游
新闻资讯,教育就业,美女,娱乐,IT 数码
美食,星座命理,健康,旅游,娱乐
文艺,教育就业,健康,新闻资讯,美女
健康

微博奇葩,新闻趣事,美食,旅游,福建生活
时尚
新闻资讯,健康,时尚,美食,文艺
游戏动漫,娱乐
燃气改装
看电视,旅游,健康,时尚,美食,娱乐
时尚,旅游,体育,娱乐
看电影,听音乐
新闻趣事
美食,文艺,投资理财,旅行,音乐,电影,90 后
旅游,IT 数码,娱乐,东方神起,爬山,音乐控,养生,摇滚,潮流,笑话
广东生活,视频音乐,旅游,美食,搞笑幽默
文艺,游戏动漫,美食,星座命理,娱乐
健康,汽车,旅游,新闻资讯,IT 数码
郴州生活网,郴州,天蝎座,微博,网络,旅游,听歌,音乐
志哥黑得真抱歉,脱宅两年真抱歉,非黑暗系真抱歉,1 级的海很深啊,活在二次元少年,组队攻略次元门,嗯，求月厨勾搭
美女
射手座,紫色,80 后,自由,电影,平常心,乐观,看书,旅行,听歌
汽车,游戏动漫,IT 数码
健康,时尚,娱乐,星座命理,美食
创业,生活,足球,艺术,股票,搞笑,运动,幽默,天蝎座,电影
健康,时尚,美食,文艺,IT 数码,美食,旅游,娱乐
美剧,kazakh
语录,娱乐,旅游,美食
学习,简单,经济,教育,休闲,吃货一枚
时尚,文艺,美食,娱乐,旅游
时尚,教育就业,投资理财,健康,旅行,自由,旅游,音乐
广东生活
文艺,时尚,星座命理,80 后,双鱼座,美食,听歌,旅游,音乐
健康,汽车,体育
白色,乐观,傻,游戏动漫
美食,投资理财,健康,时尚,旅游
游戏动漫
自拍,购物,看电影,听歌,狮子座
打球,体育
汽车
体育,孤独,寂寞,冷
教育就业
胡小颖
股票,好性格,平常心
星座命理,文艺,美食,游戏动漫,时尚,娱乐
文艺
投资理财,旅游,IT 数码,文艺,体育

IT 数码
巨蟹座,唱歌,听歌
教育就业,旅游,买房,卖房
体育,听歌,画画,设计师
动漫控,乐天派,瓶邪,游戏动漫,旅游,美食,娱乐
健康
新闻趣事,生活,财经,羽毛球
IT 数码,汽车,美食,体育,网购,足球
军事
搞笑幽默
广东生活,搞笑幽默,视频音乐,美女
旅游,美食,名人明星,新闻趣事,广东生活
娛樂
情感生活
LeslieCheung,纠结体,有梦
游戏动漫,娱乐,新闻资讯,教育就业,投资理财
90 后非脑残,宅男
美食,汽车,体育
乐观,自由,羽毛球,处女座,大学生,运动,90 后
游戏动漫,gj
玩儿,幽默,摄影,唱歌,看书,宅,旅游,旅行,美食
穿衣美容,爱电影,青春,淡定,阳光,爱睡觉,五月天,交友,吃货
IT 数码,娱乐,芯片,网购,吃货,90 后,睡觉,双子座,宅,读书
Ace
娱乐,旅游,美食,时尚
旅游,健康,美女,时尚,军事
体育
旅游
音乐
电影
体育,球痴
新闻资讯
漂亮,健身,健康,时尚,化妆,护肤,美容美发,OL
爱情,平常心,巨蟹座,80 后
文艺
吃,电影,天蝎座,大学生,旅游
我爱 UC 浏览器
美食,美食
旅游,美食,听歌,音乐,宅
累
IT 数码,娱乐,旅游
企业管理,陕西云计算,云应用
水瓶座
校园,浪漫,艺术,大学生,双鱼座,宅女,自由,90 后,旅游,听歌

新闻资讯,教育就业,美女,军事
名人明星
视频音乐,上海生活,旅游,穿衣美容,搞笑幽默
体育
旅游
音乐,时尚,电影
上网
新闻资讯,90 后
自然科学,新闻资讯,教育就业,旅游,军事,体育
90 後,欧美风走偏掉,吃货,美剧控,NBA
投资理财,健康,星座命理,军事,体育
美食,music,电影,旅游,天秤座
健康,教育就业,旅游,星座命理,新闻资讯,投资理财,体育
比利时生活
旅游
美食,游戏动漫,游戏,动漫,睡觉,学生,宅,90 后,音乐
旅游,汽车,音乐,旅行,电影,80 后
文艺
搞笑幽默
旅游,娱乐,美食
视频音乐,河北生活,搞笑幽默,汽车,游戏动漫,动漫,媒体,重感情,喜欢交朋友,堕落已久
旅游
娱乐,90 后
八卦,音樂,狮子女,偶稀饭睡觉觉,K 歌,暮光之城,时尚,娱乐,美食,文艺
军事,IT 数码
天秤座,美食,幽默,好性格,音乐,生活,平常心,搞笑,乐观,电影
爱 LOLI,爱正太,美食,电影,听歌,音乐,宅,旅行,时尚,90 后
体育
旅游,美食,军事,IT 数码,文艺
美女,学生,小说,睡觉,自由,娱乐
CG,广播剧,魔兽,懒人,猫咪,耽美,美食,旅游
健康,汽车,投资理财,旅游,美食
旅游,IT 数码,游戏动漫,学生,90 后
射手女,咖啡控,拍照,K 歌,吃货,交友,微博,浪漫,文字控,娱乐
新闻趣事,视频音乐
健康,旅游,娱乐,IT 数码,时尚,新闻资讯,文艺
旅行,电影,时尚,听歌,旅游,音乐
新闻资讯,90 后
旅游
汽车,投资理财,IT 数码,文艺,新闻资讯
游戏动漫,投资理财,IT 数码
80 后
新闻趣事
美食

电影,听歌,90 后,学生,音乐
美食
娱乐,时尚,文艺
吸血鬼日记,福尔摩斯,时尚,美食,娱乐
健康,旅游
体育
旅游
汽车,军事,体育,宅,90 后
IT 数码
旅游
搞笑幽默
汽车,旅游,美食,军事,体育
IT 数码
美食,听歌
体育,健康,时尚,美食,娱乐
星座命理,美食,健康,教育就业
80 后
八卦,上网,吃货,旅行,宅女,水瓶座
旅游,美食,星座命理
求交友
时尚,美女,健康,星座命理,娱乐
娱乐
名人明星
美女,游戏动漫
百吃不胖,吃货,双鱼女
旅行,摩羯座,宅女,时尚,网购,音乐,羽毛球,听歌,媒体,动漫
小黑子,夏目,TAO
爱旅行,淘宝,媒体,购物,自拍
娱乐
名人明星,视频音乐,萌宠,旅游,星座命理
旅游
时尚,健康,旅游,文艺,娱乐
90 后
娱乐
游戏动漫,音乐,宅,80 后
搞笑,学生,90 后
汽车,美女,IT 数码,军事,电影,听歌,宅,旅行,音乐,80 后
旅游,健康,我就是个球,白痴,90 后
新闻资讯,游戏动漫,军事,IT 数码
娱乐
音乐,宅女,听歌,网购,偶稀饭睡觉觉,旅游,英语,摩羯座,唱歌
钕人,的爱
教育就业
娱乐,画画

娱乐
旅游,羽毛球,123,旅行,狮子座
传媒,营销,写作
教育就业,汽车,新闻资讯,美女,游戏动漫
理科,马来西亚,摄影,美女,时尚,80 后,旅游,音乐
美食,宅,90 后
星座命理,旅游,美食,文艺,娱乐
娱乐,体育,游戏动漫,美女
旅游,星座命理,投资理财,时尚,健康
美食
视频音乐,穿衣美容,星座命理,微博奇葩,名人明星
旅游
娱乐
双鱼座男生,手机,睡觉狂,篮球迷,90 后
旅游,星座命理,时尚,健康,美食,笑,活泼开朗,好性格,90
电影,80 后
游戏动漫,健康,文艺,娱乐
时尚,新闻资讯,IT 数码,汽车,体育
体育,投资理财,新闻资讯,健康,IT 数码
新闻资讯,IT 数码,娱乐,旅游
茶,旅游,营销,电子商务,咨询顾问
文艺
电影,旅游,听歌,音乐
体育
体育,娱乐,IT 数码,广告
90 后
体育
90 后,宅男,游戏
旅游,教育就业,时尚,美食,娱乐
新闻资讯,投资理财,IT 数码,军事,旅游
文艺
时尚,电影,自由,旅行
游戏动漫,90 後,听音乐
视频音乐,广东生活,搞笑幽默,美女
星座命理,旅游,新闻资讯,时尚,汽车
IT 数码,旅行,宅,音乐,90 后
网购,狮子座,小说,自由,旅游,美食,听歌,90 后,音乐,电影
投资理财,教育就业,新闻资讯,美食,健康
时尚,旅游,美食,文艺,娱乐
美食
浪漫,羽毛球,志愿者,美食爱好者,搞笑,80 后的尾巴,喜欢音乐,热情,K 歌,体育
平常心,音乐,篮球,幽默,游泳…
时尚
文艺,淘宝,微博控,喜欢唱歌,穿衣美容,宅,80 后

娱乐
教育就业,军事
新闻资讯,时尚,健康,星座命理,游戏动漫
教育就业,美女,文艺,新闻资讯,投资理财,法律人
汽车
爱自由,梦想,娱乐,健康,旅游,文艺
交友,80 后
美女
美女,体育,军事
睡觉,双鱼,腐,懒,游戏动漫
旅游摄影,好书,校园,新闻资讯,美食,军事,文艺,娱乐
名人明星,视频音乐,穿衣美容,搞笑幽默,微博奇葩
陕西生活
娱乐,体育,文艺,游戏动漫
搞笑,上网,学生一族,幽默,宅女,听歌,宅,90 后,音乐
文艺,旅游
游戏动漫,时尚,IT 数码,体育,音樂
游戏动漫
IT,教育,文学,美女,大学生,游戏,交友,自由,90 后
汽车
军事,体育
陕西生活
旅游
幽默,爱幻想,神经质,五月天,强迫症,读书,双子,考研,学生一族,健康
体育
旅游
孩子气,矛盾体,旅游,音乐,自由,紫色,睡觉,偶喜欢玩,宅,90 后
玩,安静,看电影,音乐达人,科幻,星座命理,旅游,萌宠,美食,搞笑幽默
美食,80 后,音乐,宅,时尚,90 后,电影
旅游
美食,文艺
新闻趣事,搞笑幽默,陕西生活
文艺,娱乐,教育就业
IT 数码,80 后,家,爱情,性爱
搞笑幽默,旅游,美食
美食爱好者,狮子座,烹饪,新闻资讯,文艺,美食,旅游
时尚,投资理财,星座命理,娱乐
俊介迷,玩儿,射手座,摄影
搞笑幽默,萌宠,旅游,视频音乐,美女
教育就业,IT 数码
搞笑幽默,新闻趣事,美女
娱乐,90 后
游戏动漫,体育
旅游

军事,时尚,投资理财,教育就业,美女
80 后
宅,时尚,旅行,听歌
体育
娱乐,IT 数码,汽车
搞笑幽默
北京生活,健康,时尚
教育就业
90 後非脑残,格子控,麦霸,非主流,幸福小女生,苏打绿,天秤座,90 后,嵩鼠
娱乐,时尚,健康
新闻趣事
青春,真诚,音乐爱好者,神经大条,孩子气
书是我命根子,裙子,植物控,文字控,西藏,音乐,恋婚纱,爱毛衣,绿色控,燕姿
旅游
科学探索,美图摄影,新闻趣事,视频音乐,郑州生活,电子商务
时尚,汽车,IT 数码,健康,娱乐
美女,IT 数码,投资理财
文艺,投资理财,音乐,美食,宅,时尚,旅游,90 后,听歌,电影
旅游,
重庆生活
教育就业,军事,旅游,汽车,文艺,体育
搞笑幽默
时尚,投资理财,健康,旅游,90 后非脑残,爱旅行,时尚潮流,淘宝,睡懒觉,巧克力
文艺,投资理财,时尚,娱乐
旅游
宅,时尚,旅行,旅游,90 后,80 后,听歌,电影,音乐
星座命理
IT 数码
星座命理,娱乐
星座命理,健康,投资理财,时尚,娱乐
布袋戏一生爱
汽车,电影
体育
娱乐
旅游
游戏动漫
娱乐
90 后
美食,旅行,音乐,听歌,90 后
旅游,旅行,上网,宅,低调,粤语,爱旅游,80 後,游戏动漫
新闻趣事,体育资讯,旅游,美食,美女,足球,网购,幽默,搞笑
时尚,教育就业,体育
美女,军事
搞笑幽默,旅游,美女,穿衣美容

时尚
美女,教育就业,IT 数码,时尚,旅游,听歌,音乐
美食
艺术
晋城生活
娱乐
美女,健康,旅游,体育,美食
健康,游戏动漫,投资理财
时尚
平常心,电子商务,好性格,自由,听歌,旅游,金牛座,电影,看书,音乐
80 后,喜欢旅游,体育
旅游
投资理财,健康,时尚,旅游,美食,看书,90 后
自嗨屌丝女
投资理财,娱乐,IT 数码
健康,旅游,星座命理,教育就业,美食
娱乐
听歌,音乐
Y2,Nino,嵐
汽车,新闻资讯,军事,80 后,美食,听歌,旅游,电影
汽车,美女
文艺
健康,文艺,体育
游戏动漫,星座命理,文艺,娱乐,love,LIVE
健康,教育就业,投资理财,旅行,听歌,90 后,旅游
写作,IT 数码
游戏动漫
旅游,自由,电影,音乐,美食
视频音乐
教育就业,健康,旅游,美食,文艺
穿衣美容,北京生活,美女
健康
听歌,旅游,宅,80 后,电影,音乐
旅游
娱乐
星座命理,美食,IT 数码,文艺,娱乐
音乐,听歌,美食
旅游,溜冰
音乐,听歌,80 后
投资理财,时尚,旅游,美食,体育
体育
搞笑幽默
投资理财
投资理财,时尚,汽车,体育,娱乐

名人明星,音乐
电视剧,疯狂,无厘头,新聞資訊,娛樂
新闻趣事
体育,旅游,90 后,旅行
旅游,文艺,娱乐,摩羯女,听歌,美食,90 后
IT 数码
游戏动漫
黄石生活
电影,旅游,运动,电影旅游运动
健康,时尚,IT 数码,美食,娱乐
视频音乐
搞笑幽默
娱乐
广东生活
娱乐,时尚,汽车,旅游
体育,IT 数码,娱乐,军事
娱乐,旅游,IT 数码,美女
健康
星座命理,军事,文艺,娱乐,投资理财,时尚,健康,旅游,美食
体育
健康,旅游,星座命理,美食,娱乐
美食
娱乐
电子商务,自由职业,宅
美食,IT 数码,时尚,健康,美女
文艺,旅游
军事
娱乐,IT 数码,文艺
汽车
北京,太极柔力球,武汉人,柔力球
时尚,新闻资讯,教育就业,投资理财,军事
汽车,时尚,美女,投资理财,美食
时尚
八卦杂谈,美女,搞笑幽默
郑允浩,东方神起
健康,汽车,美食,文艺,体育
文艺,美食,音乐
宅,听歌,90 后
美食
新闻资讯,游戏动漫,汽车,IT 数码,体育
美女,新闻资讯,投资理财,汽车,旅游,旅游股票
旅游,美女,文艺,体育,时尚,听歌
新闻趣事,北京生活
旅游摄影,美食,投资理财,健康,美食,文艺,百毒不侵,爱生活,双鱼女

投资理财,卧薪尝胆,三千越甲可吞吴
体育
美女
娱乐,美食,文艺,星座命理
处女座
广东生活
上海生活
投资理财
IT 数码
旅游
娱乐,美食
我爱 UC 浏览器
IT 数码,汽车,旅游,星座命理,美食
体育
军事
o 型血,双鱼,咖啡控,穿衣美容,江西生活
娱乐,游戏动漫,美女,文艺
电影,宅,美食,旅游,听歌,音乐
旅游,文艺
如果爱,请深爱
时尚
健康,旅游,美女,游戏动漫,文艺
视频音乐
游戏动漫,新闻资讯,文艺
美食
投资理财,美食,星座命理,教育就业,文艺,陶陶宝
IT 数码
美食
时尚,旅游,美食
文艺,旅游,投资理财,健康,美食
情感生活
美食,射手座,幽默,紫色,听歌,乐观,音乐
巨蟹座,爱幻想,热爱生活,人生,梦想,神经质,美食爱好者,睡不醒,迪拜,宅
90 后
旅游,星座命理,文艺,爱唯美
汽车
健康,汽车,旅游,星座命理,美食
微博奇葩
美食,微博奇葩,搞笑幽默,新闻趣事,美女
八卦杂谈
娱乐,汽车,文艺
新闻趣事,视频音乐,搞笑幽默
爱生活,李宇春,爱音乐,文字控,O 型血,狮子女,文艺
健康,教育就业,文艺,旅游,美食

睡觉睡到自然醒,nba,五月天,真诚,搞笑幽默
时尚
健康,旅游,IT 数码
时尚
投资理财
娱乐
美女
美食
游戏动漫,美食,IT 数码,乐观,80 后
很想去玩,法学,大龄 90 后,三国杀控,单身一族,金牛座,动漫控
Deutsch,deutsch,魔碣,星座
视频音乐,in 乐,小伤感,爱哭鬼,执迷不悟
策划,爬山,投资,汽车,文艺
名人明星
娱乐,美食
娱乐,体育,游戏动漫
游戏动漫,军事,美食,80 后
医生
时尚,旅游,美食,文艺,娱乐
健康,游戏动漫,星座命理,文艺,吃喝玩乐,小说,电影
音乐
美食
驻马店生活
旅游,美食
时尚,旅游,文艺,投资理财
爱张阳阳
星座命理,投资理财,汽车,文艺,体育
娱乐,文艺,IT 数码,美女,游戏动漫
娱乐
美食,IT 数码,娱乐
投资理财,美食,游戏动漫,文艺
摩托车,篮球,狮子座,吃货,运动,汽车
文艺,娱乐,体育,上网,大学生,电影,美食,90 后,宅
运动,孩子气,爱电影,旅游,三毛,NBA,书法,笑,古灵精怪,欧美音乐
教育就业,健康,新闻资讯,文艺,娱乐
时尚,娱乐,美女,旅游,健康,90 后
杂志,仙剑,旅游,IT 数码,文艺
健康
健康,娱乐,投资理财,旅游,体育
时尚,美食,文艺,新闻资讯,健康
手工,Music,大学,电视,健康,旅游,美食,文艺,娱乐
时尚,旅游,美食,美剧,吃货,自由,囧,射手座,音乐
新闻趣事
微博奇葩,搞笑幽默,天秤座,上网,旅行

文艺,体育,娱乐
电影
教育就业
娱乐,学生,听歌,旅游,旅行,音乐
微博奇葩,搞笑幽默,星座命理
南京生活,新闻资讯,传媒,贴钻,手机壳,戳戳乐
浙江生活
名人明星
性爱论,黄色,内涵,床上那些事,成人,黄段子,处女,夫妻那些事,做爱指南,性爱技巧
娱乐,生活,巨蟹座,幽默,偶稀饭睡觉觉,小说,运动,好性格,美食,看书
温柔善良的 80 后
80 后
娱乐
我爱 UC 浏览器
星座命理,时尚,教育就业,旅游,健康
曼联,天枰座,足球爱好者,IT 数码,体育
90 后
安徽生活,微博奇葩,搞笑幽默,steam,使命召唤 8,使命召唤,狮子座,运动,游戏
美食
新闻资讯,教育就业,旅游,娱乐,文艺
全品,高中,图书,出版,服务
美食
快乐大本营,90 後,市场营销,吸血鬼,白羊女,排球,星座命理,娱乐,教育就业,新闻资讯
时尚,游戏动漫,新闻资讯,星座命理
创意,政治,书籍,人生,文艺,旅游,投资理财
娱乐,IT 数码,汽车,健康,旅游,数码,游戏
精力充沛,英格兰,篮球,melo7,IT,旅游
美食,美食,体育,游戏动漫
时尚潮流,美剧控,妖精,电影爱好者,欧美,恐怖片
旅游
it,linux,HTML5,HTML,html,web,php,购物
娱乐
文艺,旅游
旅游
萌宠,旅游
囧,音乐爱好者,室内设计,海贼王,娱乐
崔雪莉饭,Niki 粉,夹心迷,立迷,大爱 TVB
偶喜欢玩,双鱼座,动漫,美食
娱乐
微博奇葩,美食,穿衣美容,搞笑幽默,旅游,娱乐
星座命理,旅游,美食
投资理财
星座命理
美食

教育就业
旅游,美食,体育
旅游,娱乐
健康
星座命理,游戏动漫,IT 数码,文学,篮球,动漫,睡觉,自由,宅,听歌
狮子座,交友
搞笑幽默
旅游
IT 数码,苹果,研发,销售总监
时尚
搞笑幽默
云南生活,美食,视频音乐
体育
旅游,娱乐,文艺,IT 数码,体育
时尚,健康,娱乐,偶稀饭睡觉觉,射手座,美食,旅游,音乐,90 后
萌宠
IT 数码,军事,新闻资讯,文艺,娱乐,音乐,旅游,电影
美食
微博奇葩,萌宠,穿衣美容,旅游,美食
犀利,社会,真相,现实,金融
投资理财,旅游,健康,时尚,文艺
健康,旅游,美食,IT 数码,娱乐,爱读书的好孩子
体育,健康,美女,星座命理,投资理财
汽车,时尚,娱乐,健康,新闻资讯
新闻资讯
美食,平常心,宅女,育儿,水瓶座,生活
时尚,旅游,星座命理,美食
美食,娱乐
星座运势,潍坊生活,80 后,电影,美食,工作,财经,时尚,读书,杂志
娱乐
IT 数码
新闻资讯
旅游,美食,穿衣美容,合肥生活
FC,Bayern,Munchen,Football,Fighting,travel
校园生活
名人明星,微博奇葩,搞笑幽默
电影控,传媒,音乐,旅行
游戏动漫
旅游,美食,时尚
美女
美食,萌宠
双子座,睡觉,学生,90 后,俞佳乐
美食,星座命理,旅游,文艺,娱乐
投资理财

摄影爱好者,德州扑克,搞怪,随性,吃喝玩乐,音乐发烧友,喜欢唱歌
健康,美食,文艺,娱乐,IT 数码
文艺,体育,娱乐
娱乐,文艺,体育
书法
旅游
美食
投资理财,旅游,健康,美食,汽车
80 后
体育,美女,旅游,美食,军事
教育就业,体育
健康,时尚,娱乐,文艺
游戏动漫,文艺,时尚,投资理财,新闻资讯
设计,电影迷,爱吃,旅游爱好者,喜欢唱歌
健康,娱乐,星座命理
美女,搞笑幽默,大连生活,视频音乐
健康,新闻资讯,教育就业,星座命理,文艺,听歌
旅游
娱乐
美食,教育就业,健康,时尚,投资理财
教育就业,体育,娱乐,时尚,新闻资讯
美女
射手,爱睡觉,单身,宅男,新闻资讯
美食,旅游,游戏动漫,军事
90 后
美食,广东生活,新闻趣事,微博奇葩,新闻资讯,游戏动漫
NBA
音乐,美食,80 后
美食,健康,投资理财,时尚,星座命理,新闻资讯,娱乐
美食
睡觉,学生,看书,宅,旅行,旅游,美食,电影,听歌,音乐
平模,模特,MichaelJackson,舞蹈,文字欲,唱歌,容祖儿,古巨基,卫兰,电影
萌妹控,cos,动漫,心理学,漫画,ACG,bl,les,耽美,音乐爱好者
美女,游戏动漫,教育就业,文艺,体育
体育
周杰伦,欧式复古风,洋装,剑网三,旅游,游戏动漫,天秤座,宅女,恶魔,天下 3
新闻趣事,视频音乐,旅游,美女,南京生活
萌宠
李云迪周杰伦
汽车,体育
旅游,健康,投资理财,文艺
军事,IT 数码
美剧,80 后宅女,青春,浪漫,摄影控,DIY,购物,淘宝控,奋斗,旅游
音乐,时尚,旅行,美食

旅游,IT 数码
娱乐
美食
新闻资讯,游戏动漫,汽车,美食,体育
美食
IT 数码
还没想好
仙剑,音乐发烧友,手机,NBA,博客,美食,哈利波特,游戏动漫,周杰伦,爱电影
军事,美女,教育就业,娱乐,体育
动漫,网络,游戏,交友,看书,宅,时尚,美食,电影,音乐
动漫
西班牙,汽车,时尚,投资理财,文艺,旅游
旅游,80 后,天枰座
穿衣美容
美食,微博奇葩,搞笑幽默,旅游,视频音乐
汽车
文艺
IT 数码,娱乐,旅游,体育,美食
文艺,星座命理,娱乐,游戏动漫,IT 数码
汽车
投资理财,健康,文艺,旅游
美食,旅游,新闻趣事,搞笑幽默,成都生活,音乐,80 后,听歌
旅游
双重性格,新闻资讯,IT 数码,游戏动漫,投资理财
阿米,游戏动漫,渣渣,APH
健康,娱乐,星座命理,旅游,美食
新闻资讯,健康,美食,美女,时尚,化妆师
爱旅行
90 后
教育就业,投资理财,新闻资讯,文艺,笨蛋一枚
教育就业,健康,旅游,星座命理,美食
新闻资讯,星座命理,健康,IT 数码,娱乐
汽车,时尚,娱乐,美女,投资理财
星座命理,音乐
搞笑幽默
旅游,新闻资讯,健康,投资理财,教育就业
文艺,乐观开朗,白羊座,时尚,电影,音乐
军事
电影,80 后
文艺,新闻资讯,健康,美食
视频音乐
新闻趣事
旅游,美女,军事,文艺,娱乐
星座命理

旅游,文艺,时尚
星座命理
林晨钰,爆乳门
美女,文艺
吃货一枚,小说迷,快乐大本营,射手座,爱美食,淘宝,热爱生活,看电影,夜猫子,shopping
IT 数码
创业,生活,旅游,平常心,天蝎座,音乐,时尚,汽车
魔兽世界,剑网三,90 后,日剧
穿衣美容,美食,名人明星,星座命理,视频音乐
美食
旅游,喜欢文字,钓鱼,登山,羽毛球,文藝青年
文艺,美食,游戏动漫,IT 数码,美女
娱乐
娱乐
美食,星座命理,旅游,健康,投资理财
旅游,军事,IT 数码,文艺,娱乐
新闻资讯,教育就业,军事,IT 数码,体育
健康,文艺,IT 数码,军事,体育
90 后
b 型血,爱自由,爱旅游,建筑,大叔控
名人明星
幽默,自由,时尚,80 后
萌宠,搞笑幽默,学生,音乐,80 后
旅游
游戏动漫,美食,文艺,娱乐
视频音乐
时尚,健康,旅游,星座命理,美食
娱乐,美食,星座命理,文艺,体育
娱乐
旅游,美食,文艺,体育,娱乐
新闻趣事,广州生活,房产,生活,网购,上网
文艺
美女,南昌生活,搞笑幽默,校园生活
美女,游戏动漫,体育,娱乐
文艺,游戏动漫,新闻资讯,教育就业
音乐
时尚
穿衣美容
足球,佛教,军事
发呆,爱笑,简单,爱生活,旅游
旅游,文艺,健康,IT 数码,设计,网络,交友,大学生
视频音乐
星座命理,时尚,美食
强迫症,宅,幻想,IT 数码

旅游,星座命理,美食,娱乐,时尚,冷笑话,天平座
NBA,足球,古典吉他,大金毛,豆瓣,ARCGIS,夭夭,民谣,天蝎,偏执狂
喜欢音乐,阳光,军事,美食,旅游,美女,娱乐
整容
美女,军事,旅游,IT 数码,文艺
视频音乐,80 后
Music,唱 K,宅,时尚,90 后,电影,听歌,音乐
萌宠
开腔赏的瓜西流,表情丰富有点 2,水瓶座懂的起噶,反正 H 不住,反正 H 不住就是,自嗨型够白痴
体育,旅游
美食,南京生活,微博奇葩,美女,穿衣美容,时尚
时尚,娱乐,90 后
搞笑幽默
教育就业
时尚,教育就业,娱乐,IT 数码
投资理财,教育就业,健康
时尚,美女,新闻资讯
美食,生活,处女座,电影,旅行
健康
旅游
做梦旅行
旅游
时尚,新闻资讯,IT 数码,体育,娱乐
美食,娱乐,健康,文艺,旅游
游戏,动漫,校园,天蝎座,唱歌,偶稀饭睡觉觉,宅男,平常心,交友,学生
旅游,游戏动漫,谁会爱我
睡觉睡到自然醒
旅行,创业,手机,大学生
感性,喜欢唱歌,射手,服饰,女人,星座命理,时尚,教育就业,娱乐,文艺
IT 数码,大学生
美女,时尚,娱乐
游戏动漫
90 后
咖啡,军事,足球迷,驴友,美食爱好者,时尚达人,吃喝玩乐
善良,90 后,听歌,白羊座
IT 数码
旅游,走走看看,唱唱跳跳,电影
健康,美食,旅游
时尚,投资理财
游戏动漫,星座命理,军事,好书,美食,音乐,电影,90 后,宅
西安舞蹈培训,西安舞蹈,西安 DDS 街舞,西安 DDS,街舞培训,HIPHOP,JAZZ,西安爵士舞,西安街舞培训,西安街舞
体育

健康
自由,美食,旅游,学生
唱歌,幽默,宅,游戏,睡觉,写作,学生,90 后,上网,听歌
IT 数码,学生,听歌,90 后,交友,游戏,电影,音乐
剑网 3
平常心,幽默,旅行,电影,80 后,旅游
搞笑幽默
美食,名人明星,旅游,新闻趣事,萌宠
军事,美食,美女,旅游,健康
名人明星
健康,新聞資訊,時尚,旅遊,美食
IT 数码,话说缘分
时尚,健康,旅游
漫画,摩羯女,内向,宅男,重庆,驴友,美食爱好者,单纯,金牛
旅游,休息视线,毒蝎子,我就是我
美女
军事
健康,旅游,星座命理,美食,文艺
IT 数码,体育资讯,新闻趣事
旅游
时尚,文艺,娱乐
我爱 UC 浏览器
游戏动漫
新闻资讯,健康,旅游,时尚,美食,电影,音乐
学生一族,爱自由,梦想,公益,创意,科技,科幻
IT 数码
美女,投资理财,汽车,新闻资讯,IT 数码,深圳,襄阳人
IT 数码
宅,运动,篮球,听歌
听歌
文艺
汽车,梦想能实现自己,自己的设计者
车饰,导航仪,射手座,爱好,性格,习惯,饮食,购物
美食,健康,新闻资讯,文艺,娱乐
平面设计,看电影,电影控,音樂,创意设计,星星,广告人,汽车,艺术,单身
双子肖马者,乐观,爱说,爱笑!
健康,美食,摄影,电影,宅,旅游,80 后,音乐
汽车,体育
时尚,文艺,美食,星座命理,旅游
时尚
双子座
新闻趣事,旅游,IT 数码
自由,手机,运动,娱乐,听歌
美食

信任
名人明星
单身求解放,娱乐,音乐,古筝
80 后,宅
旅游,水瓶座
投资理财
教育就业,健康,文艺,体育
时尚
旅游
星座命理,美食,娱乐,文艺
宠物图集,健康
教育就业,时尚,旅游,文艺
搞笑幽默,瑜伽,旅游爱好者,爱情,微博,文字,青春
搞笑幽默
体育,大学,囧,篮球
娱乐
IT 数码,汽车
美食
讨厌虚伪,坦率,说话直接
星座命理,健康,旅游,美食,IT 数码
新闻资讯
投资理财
旅游,IT 数码,时尚,美女,汽车
视频音乐,文艺
IT 数码,索尼笔记本电脑
时尚
游戏动漫,美食,娱乐,听歌
军事,游戏动漫,IT 数码
80 后,音乐,电影
旅游,娱乐,健康
网络营销专家,合肥网站优化,合肥 seo,合肥网站建设,合肥网络营销
体育资讯
时尚,文艺,教育就业,美食
美女,时尚,汽车,娱乐,新闻资讯
星座命理,双鱼座,小说,动漫,学生,90 后
美图摄影
经济,打篮球,自由,宅,我就是我,天平座
美食,搞笑幽默,旅游,90 后,电影
美食,痛苦 ing
艺术,文学,宅
CF
IT 数码
游戏动漫
喜欢爬山，跑步,游泳

旅游,美食,游戏动漫,投资理财,娱乐
娱乐,蜉蝣
文艺
IT 数码,军事,体育
娱乐,星座命理
投资理财,文艺
摄影,睡觉,学生,时尚,旅游,90 后,电影,美食,听歌,音乐
宅
娱乐
文艺,健康,90 后一追求淡定,迷茫,沉默,阅读,双鱼座,学生,旅行
投资理财
金牛,美食
电影控,夜猫子,文字控
旅游,穿衣美容
旅游,美食,投资理财,娱乐
娱乐
健康,新闻资讯
旅游
健康,美食
旅行,80 后
娱乐,明星
美食,80 后,旅游,音乐
亲情,音乐,生活,乐观,电影,自由,读书,羽毛球,好性格,旅游
汽车,投资理财,娱乐,游戏动漫
体育,娱乐,文艺,90 后
名人明星
偶稀饭睡觉觉,学生
游戏动漫,美食,娱乐
b 型血,奋斗,影视,喜欢音乐,大学生,善良,学生一枚,新闻资讯,教育就业,IT 数码
名人明星,旅游,视频音乐,新闻趣事,上海生活
投资理财,IT 数码,美食
游戏动漫,时尚,音乐,旅行,宅
双子座 O 型血,体育
搞笑幽默,星座命理,美食,名人明星,视频音乐
美食
新闻资讯,星座命理,美女
健康,旅游,汽车,看书,浪漫,新闻,摄影爱好者
睡觉,学生,旅游,80 后,游戏动漫
星座命理,娱乐
娱乐
旅游,军事
旅游,健康,时尚,美食
教育就业,旅游,娱乐,体育,汽车,电影
车世界,IT 数码,廊坊生活

美女,新闻资讯,军事,IT 数码,娱乐
旅行,90 后,听歌,音乐
游戏动漫,娱乐
体育
萌宠,摄影,动漫,篮球,敏感,热爱生活,打篮球
娱乐
星座命理,美食,文艺
新闻趣事,美食,萌宠,视频音乐,旅游
职场招聘
投资理财,旅游,军事
美食
退休教师,书法爱好者
体育
搞笑幽默,视频音乐,新闻趣事,旅游,美食
90 后
文艺,旅游
汽车
宅,听歌,90 后
音乐
IT 数码
新闻趣事
健康,文艺,游戏动漫,教育就业,投资理财
娱乐
汽车
旅游,健康,文艺
穿衣美容
娱乐
微笑,宅,看电影,网络小说,天秤座
外貌协会,强迫症,A 型血,K 歌,天蝎女,暮光之城,快乐,爱笑,自拍,心情
新闻资讯,美食,体育
读书分享
游戏动漫
名人明星
美食爱好者,法律,单身,美食控,音乐控,强迫症,吃货一枚,幸福,完美主义,偏执狂
星座运势
美食
宅女,射手座,善良,学生,宅
穿衣美容
娱乐
服装,80 后,90 后
福建生活
666888,123456789
校园生活,90 后
看电影,猫控,双重性格,爱笑,旅游美食爱好者,电影控,摩羯座

文艺,星座命理,健康,时尚,娱乐
投资理财,军事
健康,睡觉,80 后
新闻资讯,文艺,娱乐,旅游,成长 ing,K 歌,宅,90 后
偶稀饭睡觉觉,浪漫,好性格,好书,A 股,文学,旅行,电影,90 后,美食
听歌,音乐,电影
旅游,新闻资讯,健康,时尚,文艺
视频音乐,新闻趣事,微博奇葩,美食,看书,旅游,时尚,80 后,听歌,音乐
时尚,新闻资讯
投资理财,健康,时尚,汽车,旅游
汽车,美食,军事,体育,游戏动漫
投资理财,健康,新闻资讯,教育就业,IT 数码,英超
音乐
美食,新闻趣事,搞笑幽默
投资理财,IT 数码,文艺,体育,娱乐,梦想
体育资讯,美图摄影,佛山生活,学生
IT 数码
星座命理
旅游,汽车,军事,美女,健康,睡觉,90 后,小叁
宠物图集
健康,开朗,心理学,公益,社会
时尚,IT 数码
思考,爱自由,梦想,科比,旅游摄影,健康,旅游,娱乐,文艺,体育
星座命理,美食
旅游,星座命理,美食,文艺
娱乐,健康,文艺,美食,旅游
旅游,美食,文艺,教育就业,投资理财
中二病晚期,马猴烧酒,圆神,bili 君,acfun,Yooo 党,电影控
教育就业,真诚,青春,网球,玩,90 后,旅游,电影,听歌,音乐
Country
旅游,新闻资讯,军事,IT 数码,吃
80 后
娱乐
健康
美女,文艺,时尚,汽车,军事
星座命理,游戏动漫,文艺
90 后,睡觉,美食,宅
健康,美食,IT 数码,文艺,投资理财
教育就业,时尚,美食,旅游,健康
美食
时尚,新闻资讯,美食,教育就业,IT 数码
体育
旅游,美食,IT 数码,娱乐
游戏动漫

旅游,美食,文艺,娱乐,kown,自由
教育就业,新闻资讯,IT 数码,汽车,投资理财
旅游
体育
科学探索
娱乐
军事
新闻资讯,美食,投资理财
吃货,喜欢唱歌,B 型血,逛街,星座,宅,穿衣美容,视频音乐,旅行,80 后
狗狗,设计师,幽默,篮球,旅游,时尚,旅行,美食,听歌,音乐
看书,时尚,旅行,听歌
新闻资讯,茶
文艺,宅,美食,90 后,电影
狮子座
汽车,旅游,美食
美女,美食
星座命理
军事
健康,旅游,娱乐,美食,游戏动漫
微博奇葩,旅游,美食,萌宠
自由,音乐,听歌,90 后,80 后
时尚,美食,90 后
旅游
90 后非脑残,交友
不知名画手,Homosexual,百合,GL,BL,不知名写手,腐
教育就业
IT 数码,娱乐,旅游
90 后,幽默,旅行
游戏动漫
美食,时尚,教育就业,健康,旅游
投资理财,旅游,星座命理
娱乐,游戏动漫,星座命理,IT 数码,文艺
睡睡觉,拍拍照
投资理财,旅游,美女,娱乐
时尚,星座命理,美食,娱乐
体育,摩羯,爱笑,爱音乐,游泳,o 型血,乐观,独立
娱乐
美食,星座命理,IT 数码,时尚,文艺
古风,小说,动漫
体育
宅,时尚,90 后,旅行,美食
娱乐
体育
美剧,摩羯座

帅
娱乐
旅游,娱乐,体育,汽车,时尚
旅游,80 后,电影,宅,90 后
新闻资讯,IT 数码,娱乐
星座命理
旅游
时尚,旅游,娱乐,逛街,看书,音乐,艺术
美女,游戏动漫,军事
时尚
革命,家在北京,秤砣座,喜欢睡觉,手机客户端,移民新西兰,产品经理,移动互联网,爱谷歌不爱百度,80 后
屌丝,小邪恶,重口味,脏心眼,毒嘴
旅游,美食,游戏动漫
美食
汽车,游戏动漫,美女,军事,美食,游戏，音乐,宠物
旅游,时尚,美食,星座命理,文艺,电影,宅,听歌,90 后
旅游,时尚,文艺,美食,娱乐
星座命理,游戏动漫,美女,坚强,睡觉,怀旧,看电视,理财,美食爱好者,热爱生活
惠州生活,生活,互联网,123,玩儿,听歌
旅游,文艺,教育就业,时尚,新闻资讯,健康
时尚,娱乐,健康,旅游,星座命理
文艺
文艺,教育就业,新闻资讯,汽车,投资理财
美女,游戏动漫,时尚
名人明星,新闻趣事,视频音乐,搞笑幽默,情感生活
美食
搞笑幽默
体育
搞笑,体育,电影,飞行,自由
旅游,听歌,90 后,美食
IT 数码,游戏动漫,美女,体育,时尚,听歌,音乐,90 后
健康,体育,美食,星座命理,娱乐
穿衣美容,微博奇葩
娱乐
音乐
星座命理,音乐,狮子座,听歌
旅游
80 后
IT 数码
视频音乐
微博奇葩,星座命理,新闻趣事,旅游,穿衣美容
艺术,动漫,音乐,电影,交友,摄影爱好者,乐观,狮子座,爱情,英语
时尚,汽车,娱乐,体育,军事,动漫

美女
娱乐
娱乐
旅游,佛、书法,写字音乐,蓝色
搞笑,大学生,娱乐,上网,旅行,时尚,旅游,美食,电影,音乐
汽车,投资理财,IT 数码,娱乐
教育就业
新闻趣事,自由,小说,学生,上网,90 后,时尚,听歌,电影,音乐
IT 数码,美食
美女,游戏动漫,体育,健康
文学
平面设计,唱歌,学生,上网,处女座,疯子
游戏动漫,体育
情绪化,街拍,爱吃,双鱼座,爱幻想,学生一族,偶稀饭睡觉觉,美食控,孩子气
淘福啦众福,淘福啦商城,旅游,电子商务,居家创业,淘福啦网商若萱,淘福啦桂冠体系,淘福啦网购,80 后
美食
投资理财,旅游,美食,美女
星座命理
IT 数码,美食,美女,汽车
美女
旅游,时尚,文艺,睡觉,自由,学生,听歌,电影,美食,旅行
看书,唱歌,感性,懒人,睡懒觉,爱音乐,90 後,古灵精怪,傻
娱乐,美女
时尚,旅游,文艺,星座命理
娱乐
军事,娱乐,IT 数码
游戏动漫,文艺
新闻资讯,投资理财,军事
时尚,娱乐,美食控,活泼,喜欢音乐,笑,吃货一枚
旅游,美食
投资理财,汽车,美女,游戏动漫,美食
美食,文艺,娱乐
投资理财,健康,体育,娱乐
旅游
搞笑幽默,娱乐,旅游,体育,时尚
学习,美食
IT 数码
IT 数码
时尚,文艺
新闻资讯
旅游,美食,IT 数码
穿衣美容,体育
。。。,偶喜欢玩

网络,好漫画书,白羊座,听歌,90 后,学生,上网,篮球,音乐,90 后非脑残
视频音乐,音乐,电影
昆明生活
星座运势
时尚,教育就业,娱乐,美食
美图摄影,重口味,旅游,读书分享,动漫,吃货,音乐,开朗,天秤女
金牛座,看书,学生
自然律动
游戏动漫
旅游,投资理财
军事
新闻趣事,视频音乐,美女,星座命理,搞笑幽默
美食
搞笑幽默
教育就业
星座命理,娱乐,体育,IT 数码,汽车,健康,军事,文艺,旅游
游戏动漫
江苏生活,沉默,乒乓球,摩羯座
85 后,发呆,淘宝,美食,爱幻想,购物狂,文艺
韩叶喻黄高乔,全职高手,根正苗红好少年,爱的战士,fz 一生推
旅游,文艺,美食
金牛座,学生,吃,90 后,篮球,睡觉,交友,玩儿,宅
新闻资讯,健康,星座命理,旅游,美食
apple 达人,apple
80 后,音乐,睡觉
IT 数码,大学
小说,美食控,睡觉睡到自然醒,宅女,吃货,动漫控,音乐控,军事,90 后
汽车模型,偏执狂,教育就业,军事,IT 数码,体育
游戏动漫
上网,宅,时尚,80 后,听歌,音乐
旅游
娱乐
新闻趣事
微博奇葩,武汉生活,游戏动漫,美女,美食,IT 数码,体育
时尚,双重性格,汽车,好性格,快乐,安静,无厘头
美女,游戏动漫,娱乐,美食,90 后,宅男
时尚
文艺
美食
旅游,星座命理,文艺,IT 数码,娱乐
教育就业,美女,旅游,美食,体育
旅游,文艺
名人明星,时尚,娱乐
投资理财,时尚,健康,我爱信子和颓废

体育资讯,财经资讯,军事天地,旅游,视频音乐,听歌,宋词篮球
游戏动漫
健康,文艺,时尚,旅游,星座命理
新闻资讯,自由,旅行
教育就业,美食,健康,星座命理,旅游
阅读,看书,电影,新闻,平常心,写作,体育,睡觉,亲情,旅游
教育就业,文艺,娱乐,健康,旅游
军事,IT 数码,体育
厚街竹溪中学,旅行,美食,90 后
美食,星座命理,旅游,健康,娱乐
我爱 UC 浏览器
IT 数码,美女
健康,美女,时尚,美食
教育就业,投资理财,健康,旅游,美食
新闻资讯,健康,教育就业,美食,娱乐
听歌，看电影
旅游,美食,听歌,电影,音乐
美食,美女,健康,新闻资讯,军事,IT 数码
幽默,时尚,旅行,运动,手机,乐观,80 后
美食,时尚,星座命理,游戏动漫,娱乐,90 后
萌宠
80 后,怎样教育好孩子
投资理财,体育
穿衣美容,时尚,旅游,美食,90 后,电影,听歌
健康,旅游,美食,教育就业,娱乐
娱乐,80 后
搞笑幽默,听歌
健康,娱乐,文艺,投资理财,军事
美女
吃货一枚
耽美就是命
星座命理,娱乐
旅游
宠物图集,读书分享,视频音乐
投资理财,旅游,大学,校园,金牛座,大学生,90 后
IT 数码,文艺,时尚,健康,娱乐
旅游,美食,IT 数码,体育
音乐,自由,时尚,90 后
烦了,想你,真的,拾贰,美食,80 后,旅游,电影,交友,洛阳
平常心,旅游,巨蟹座,听歌,吃,网络,动漫
星座命理,娱乐
美食
传媒,爱音乐,听音乐,新闻资讯,美食,时尚,娱乐,文艺
旅游,健康,时尚,教育就业,投资理财

文艺
旅游
旅游,汽车,美女,IT 数码,娱乐
穿衣美容
旅游
新闻趣事,广东生活,旅游,微博奇葩,搞笑幽默
文艺,教育就业,旅游,美食,游戏动漫
穷游背包客,90 后的宅女,睡觉是头等大事,旅行爱好者,爱音乐爱唱歌,听青檬,跑步爱好者,五月天
投资理财,旅游,IT 数码,汽车,体育
爱睡觉
科技,投资理财,新闻资讯,旅游,军事,文艺
新闻趣事,美食,旅游,浙江生活,美女
娱乐
体育,天秤座,喜欢听音乐,看书,看电视剧,电影,喜欢军旅剧,有情节的,喜欢美食
IT 数码
90 后脑残
天蝎座,睡觉,音乐
看电影,美剧,笑话,养生,摄影控,健康,新闻资讯,IT 数码,体育,音乐
爱魏阿晨的铁橙
军事,IT 数码
小说,足球迷,游戏动漫,旅游,体育
美食,IT 数码
体育,娱乐,吉他菜鸟,BEYOND,架子鼓手,摇滚青年
时尚,娱乐,美食
游戏
旅游,教育就业,投资理财,美食,娱乐
汽车,投资理财,军事,IT 数码,旅游,爱情,交友,80 后,手机,双子座
星座运势,美图摄影,校园生活,搞笑幽默
新闻趣事
投资理财,健康,旅游,美食,IT 数码
文艺,IT 数码,生活,听歌,电影
美女
星座命理
军事
时尚,美食
自拍,化妆,吃喝玩乐,旅行,恐怖片,动画,听音乐,白羊座,街拍,爱美食
娱乐,教育就业,文艺,体育
IT 数码
电影,唱歌,好性格,天秤座,阅读,交友,美食
白羊座,读书,幽默,旅游,上网,生活,历史,英语,新闻,篮球
美食
星座命理
体育

娱乐,新闻资讯,时尚,游戏动漫,IT 数码
健康
无厘头,偶喜欢玩,时尚,狮子座,唱歌,吃,幽默
娱乐,健康
外貌协会,欧美控
游戏动漫
娱乐,汽车,健康,体育,旅游
健康
青春,同志,文艺,小怪兽的化身。
盗墓笔记,好书,IT,美女,体育
穿衣美容,娱乐
HaiDes
媒体,宅,奋斗,历史,阅读,演员,爱幻想,单身,90 後,文学
娱乐
浙江生活
90 后
娱乐
军事,IT 数码,文艺,体育,娱乐
教育就业,新闻资讯,投资理财,健康,会计,电子商务,网购,天蝎座的男生,看书,下棋
美食,时尚,教育就业,投资理财,娱乐,星座命理
投资理财
投资理财
美女
健康,飞雁滩,feiyantan,粗布,老粗布
美食,健康,时尚,IT 数码,娱乐
校园生活,体育资讯
时尚
广东生活,90 后,听歌,篮球,偶喜欢玩
旅游美食爱好者,工作狂,咖啡控,麦霸,团购,70 后,旅游达人,K 歌达人,美食达人,浙江生活
摄影控,摄影爱好者,旅游,美食,文艺
旅游
搞笑幽默
体育
体育
美食
健康,旅游,美食
星座运势
体育,美女,IT 数码,军事,汽车,商标,专利,著作权,律师
搞笑幽默,学生,听歌,90 后,音乐
信阳生活
新闻趣事
自信,公关,设计师,社会,奋斗,商业,IT 互联网,教育就业,IT 数码,投资理财
80 后
名人明星

美容,文艺,化妆品,朋友,IT 互联网,沉默,古典音乐,投资理财
shopping
吃货,爱张根硕,爱看韩剧,喜欢听歌,喜欢笑,喜欢睡觉
娱乐
军事,娱乐,时尚
听歌,音乐,时尚,宅
时尚,健康,旅游,投资理财,文艺
汽车
校园生活
游戏动漫
娱乐,女大生,文字,设计,美食,旅行,电影,音乐,90 后
小说
IT 数码
投资理财,美食,旅游,娱乐,游戏动漫
微博奇葩
健康,美食
美食,旅游,汽车
娱乐
文艺
文艺,汽车,美女,星座命理,军事,IT 数码,好书,交友,大学生
搞笑幽默,视频音乐,80 后,听歌,宅,音乐
教育就业,美女,军事,文艺,体育
搞笑幽默
IT 数码
IT 数码,体育
旅游
美女,微博奇葩,萌宠,搞笑幽默,奋斗中的 80 后,旅游,游戏,汽车
搞笑幽默
美女,游戏动漫
星座命理,时尚,健康,IT 数码,文艺
教育就业,健康,旅游,星座命理,娱乐
健康,旅游,星座命理
军事,新闻资讯,IT 数码,文艺,娱乐
体育
美女,汽车,娱乐,文艺,美食,80 后,音乐,旅行
宅女一枚,随性,青春,時尚,电影迷,梦想,健康
旅游,美食,文艺,体育,娱乐
娱乐,美食,旅游,文艺,健康
文艺
时尚,文艺
娱乐,健康,旅游,文艺,教育就业
书,music,娱乐
时尚,游戏动漫,旅游,星座命理,美食
旅游,娱乐

旅游,游戏动漫,美食,发呆者,漫画,小丑,大蛇丸,动漫,电影,90 后
娱乐,旅游,美食
娱乐
音乐
新闻趣事
美食,娱乐
新闻资讯,音乐
健康,新闻资讯,游戏动漫,IT 数码,娱乐
浙江生活,星座命理,旅游,萌宠,美食
IT 数码,军事
新闻资讯,健康,教育就业,星座命理,90 后
旅游
体育
时尚,美食,文艺,学生
网购,生活,潮流,摇滚,音乐控,笑话,台球,曼联,古典艺术,明星
旅游,美图摄影,新闻趣事,美食,名人明星
星座命理,文艺,投资理财,娱乐
文艺,娱乐,游戏动漫,美食,旅行,宅,时尚,电影,音乐
校园生活,搞笑幽默,八卦杂谈,互粉,英格兰,90,帅哥
美食,恋爱,大叔控
旅游,游戏动漫,善良,摄影,听歌,电影,80 后
星座命理
文艺
跳舞,IT 数码,缺爱 ing
体育,孝顺,抱负,细腻,毅力,白羊座,90 后,幽默,大学,创业
汽车,美女,游戏动漫,体育,娱乐
美图摄影
紫色
投资理财,美食,汽车,旅游,娱乐,潜力股,风趣,稳重豁达和善,笑天忆寒
IT 数码,健康,游戏动漫,投资理财,旅游,文艺
搞笑幽默,美食
旅游,游戏动漫,汽车,体育
美食,乐山生活
军事,健康,新闻资讯,投资理财
DOTA 爱好者,WoW,手机,宅男,动画,爱自由,吃喝玩乐,娱乐,游戏动漫,美食
体育,音乐,电影,听歌
时尚,体育,教育就业,军事,娱乐
娱乐
体育
美女
a 型血,英语,文字,吃喝玩乐,爱自由,梦想
音乐,90 后,听歌
美女
旅游,健康

文艺,教育就业,美女
汽车,时尚,投资理财,星座命理,健康
娱乐
星座命理
时尚
搞笑幽默
美食
IT 数码
娱乐
美女,IT 数码,视频音乐,蚌埠生活,搞笑幽默,朋友,宅
文艺,新闻资讯,时尚,投资理财,娱乐
军事
投资理财,旅游,教育就业,体育
汽车,IT 数码,美食,体育
娱乐
设计师
体育,文艺
投资理财,健康,旅游,IT 数码,体育
美女,投资理财,IT 数码,汽车,手表,旅游,电影
美食,健康,IT 数码,旅游
时尚,游戏动漫,旅游,娱乐,电影,听歌
新闻资讯,健康,旅游,美食,娱乐
新闻资讯
美食
河南生活
体育
80 后的尾巴,偶稀饭睡觉觉,音乐,游戏动漫,创意,感性,睡懒觉,爱吃,懒人,宅
搞笑幽默,美食,视频音乐,扬州生活,曼城,阿奎罗,旅游
美食,旅游,福州生活
五月天,听歌,电影,90 后
旅游
时尚,美食,娱乐,文艺,星座命理,吃货,diy,猫咪,水瓶座,狮子座
汽车
汽车,娱乐
游戏动漫,新闻资讯,文艺,娱乐,星座命理
紫色
时尚,投资理财,旅游,汽车
旅游,健康,游戏动漫,美食
80 后
吃货,星座控,处女座
看书,十字绣,吃零食,逛街等……
搞笑幽默
IT 数码,时尚,旅游,军事,体育
上网,偶稀饭睡觉觉,音乐,吃货,小说,睡觉,读书,手机,平常心,宅

投资理财,汽车,时尚,旅游,文艺
IT 数码
旅游
宅
美食
军事,IT 数码
呼呼的我,旅行,音乐,听歌,80 后
IT 数码,健康,星座命理,旅游,宅女,80 后
新闻趣事,新闻资讯,健康,军事,IT 数码
旅游
耽美腐宅女,游戏动漫,美食
汽车
美女,游戏动漫,军事,IT 数码,体育
美食,健康,体育
投资理财,文艺,星座命理,美食,娱乐
微博奇葩,美女,美食,旅游,星座命理
IT 数码,旅游,美女,投资理财,汽车
90 后
美女
旅游
旅游
SuperJunior,娱乐,微博控,励志的小伙
80 后
游戏动漫
想去旅行,自然卷,天然呆笑点很低,电影控,小清新
江西生活,体育
旅游,游戏动漫,IT 数码
创业
体育资讯,视频音乐,新闻趣事,校园生活,美女,摄影爱好者,哈哈
美女,游戏动漫,娱乐
游戏动漫
汽车
游泳,军事
旅游
健康,文艺,时尚,美容养生,处女座,美食,感性,90 后,文字控,爱幻想的人儿
美女,教育就业,投资理财,IT 数码
娱乐
旅游,美食,娱乐,唱歌,狮子座,音乐,宅
随和,籃球,徒步,一個人
平常心,读书,新闻,生活,美食,旅游
教育就业,文艺
偶喜欢玩,法律,狮子座,游戏动漫
天蝎座,90 后
健康

面瘫党,银他妈是种态度,cosplay,游戏动漫,动漫控,COS
美食,90 后
健康,新闻资讯,汽车,旅游,美女
星座命理,美食
星座命理,小说,美食,旅游,韩剧
旅游,美女,体育
美容,腐女,宅女一枚,天蝎,绘画,书,服装,胡歌,新闻资讯,美食
美食
A 股,自由职业,大学
游戏动漫,旅游
IT 数码
视频音乐,职场招聘,美食,体育资讯,长沙生活
名人明星
留学,电影控,加拿大,环保,电子,旅行
体育,教育就业,游戏动漫,旅游
娱乐
湖北生活
微博奇葩,90 后非脑残,愤青,睡觉睡到自然醒
星座命理,新闻趣事,搞笑幽默,微博奇葩,萌宠
健康,娱乐,星座命理,80 后,时尚,平常心,唱歌,金牛座
星座命理
健康
新闻趣事
文艺
美食
最爱普洱不加糖,神经质的天枰女,随遇而安平凡心,爱幻想
新闻资讯,教育就业,旅游
IT 数码
水瓶座,爱旅行,美食
投资理财,军事,美食,体育,IT 数码
教育就业,美女,健康,旅游,娱乐
IT 数码
旅游
旅游,90 后
正太控,田晨,外貌协会,腐女,美男空,多情,花粉,芒果粉,95 后,V 迷
投资理财,体育,宅
时尚,旅游,美食,文艺,娱乐
投资理财,游戏动漫,新闻资讯,IT 数码,时尚,旅行,听歌,80 后
逛街,巨蟹座,睡觉
旅游
投资理财,娱乐,好男人,痴情,健身,健美,伤感,野兽,猛男,型男
时尚,美食,80 后,90 后,听歌
娱乐
唱歌,看小说,bl,腐,懒,盗墓笔记,宅女加腐女,美食

80 后
汽车
星座命理,健康
爱哭爱笑爱发呆,小极端,处女座,爱吃糖,动漫
娱乐,文艺
游戏
娱乐
体育
吃货一枚,摩羯座
投资理财
旅游,汽车
旅游,美食,爱睡觉,发呆
旅行,美食,狮子男,画画,开朗,闷骚,努力
搞笑幽默,美食,美女
IT 数码,旅游,教育就业,美食,娱乐
星座命理
喜欢美的东西
美食
原宿,earthandmusic,olive,singel,日本,原单,潮流
自由,上网,学生,动漫,学习,电脑
爱睡懒觉,爱看电视,爱上网
投资理财,汽车,娱乐,IT 数码,旅游,美食
体育
汽车
教育就业,新闻资讯,健康,时尚,文艺
美食,游戏动漫
旅游
IT 数码,美女,娱乐,体育,游戏动漫
微博奇葩
游戏动漫
文艺,投资理财,时尚,旅游,美食
旅游
搞笑幽默
新闻资讯,健康,旅游,美食,文艺
汽车,时尚,美食,旅游,娱乐
旅游,美食,时尚,新闻资讯
风行,学生,男人,搞笑
名人明星,新闻趣事,萌宠,微博奇葩,搞笑幽默
IT 数码
健康,美女
文艺,美食
星座命理,娱乐
美食,互联网,手机,宅女
娱乐

时尚,健康,新闻资讯,教育就业,文艺
90 后但我不脑残,沉默,巨蟹座
河北生活
搞笑幽默
穿衣美容
小狮子,薇薇控
体育
娱乐
新闻资讯
旅游
文艺
时尚,游戏动漫,美女,新闻资讯,IT 数码
娱乐
时尚,娱乐
江苏生活
学英语练口语,尼采,健康,美食,文艺,阳光男孩,看书,运动健身,韩寒
健康,旅游,星座命理,教育就业,体育,美女,文艺,娱乐
自拍,旅游美食爱好者,简单,养生,宅女一枚,影视,奋斗
夜猫子,天秤,绘画,动漫控,艺术,爱自由,学生,睡觉,宅,Android
健康,新闻资讯,时尚,投资理财,星座命理
军事
体育
健康,教育就业,美食,文艺,娱乐
教育就业,投资理财,体育
而立之年,形婚,已出柜,纯音乐,魔兽世界,爱帅哥,白羊座,西藏
文艺,美食,游戏动漫,健康,旅游
新闻资讯,美食,游戏动漫,体育
娱乐
江苏生活
娱乐,文艺,体育
名人明星
娱乐
学生一枚,宏迷
投资理财
旅游
星座命理
名人明星
娱乐
文学,看书,天蝎座,好书,小说,动漫,篮球,自由,IT
时尚,健康,星座命理
搞笑幽默
体育
旅游
意大利生活

投资理财,美食,旅游,健康,文艺
美女
健康,教育就业,体育
水瓶,拍照,星座命理,健康,时尚,旅游
情感生活,财经资讯
健康,旅游,投资理财,美食
娱乐
游戏动漫,自由,新闻,小说,宅,上网
美食
视频音乐
名人明星
古典音乐,旅游
星座命理,健康,文艺
猫咪控,小说迷,无厘头,腹黑,学生一枚,幻想,爱睡觉,吃货一枚,cosplay,游戏动漫
美食
美女
动漫,美食,双鱼座,高中生,台球,b 型血
单身,美食,投资理财,游戏动漫,美女,汽车
娱乐
星座命理,娱乐,文艺,旅游,美食
IT 数码,搞笑幽默,八卦杂谈,CSOLer,魔羯座,宅
长腿控,减肥 ing,电影,美剧
文艺
搞笑幽默
旅游
河北生活,微博奇葩,新闻趣事
军事,新闻资讯
游戏动漫,美食,文艺,IT 数码,娱乐
IT 数码,娱乐
美食
搞笑幽默
俺是 70 后
时尚,我就是我,哈哈,平常心,双鱼座,善良,星座命理,美食,文艺,娱乐
单纯,旅游,星座命理,喜欢旅游,心情,活泼,幽默,交友
韩国,bigbang,韩饭,权志龙,林俊杰
90 后
北川,药王谷,蜜蜂,蜂蜜
大参林药店
IT 数码,娱乐
视频音乐
美食,娱乐,教育就业,健康,文艺
时尚,旅游,健康,美食
美食,旅游,90 后
新闻资讯,投资理财

EXO,音樂,时尚,90 後,自然
宠物图集
体育,军事,喜欢运动,听音乐,交朋友…
IT 数码,旅游,游戏动漫
汽车,投资理财,时尚,娱乐,星座命理
教育就业
时尚
教育就业,时尚,旅游,投资理财
新闻趣事
古典舞,小众文艺,旅游,文学,语言
旅行
文艺,旅游,时尚,健康
健康,游戏动漫,美女,星座命理,美食
美食,90 后
美女
潮阳职教
旅游
90 后
军事,投资理财,文艺
时尚,旅游,星座命理,美食
旅游
电影,听歌,音乐
音乐,聊天,做学霸
情感,律师,IT 互联网,80 后宅女,美女,新闻资讯,健康,旅游,体育
汽车
2B 青年,灰常喜欢沙滩,吃货
旅游,美食,文艺,体育,娱乐,游戏,上网
教育就业,文艺,新闻资讯,旅游
美食,娱乐,时尚,IT 数码,体育
娱乐,美食
教育就业,投资理财,时尚,旅游,文艺
白羊座,手机,设计,大学生,娱乐,游戏,动漫,学生,小说,音乐
娱乐
新闻资讯
美食,时尚,娱乐
教育就业
唐山生活
美女,微博奇葩,四川生活,搞笑幽默,视频音乐
美女,汽车,健康
投资理财,汽车,游戏动漫,星座命理,IT 数码
美食
名人明星,时尚,旅行,90 后,音乐,电影
微博奇葩,河南生活,新闻趣事,搞笑幽默,教育就业,投资理财,旅游,新闻资讯,文艺
体育,80 后

偶稀饭睡觉觉,善良,巨蟹座,上网,美食,旅游,自由,80 后
美食,校园生活
小清新,少女时代,自由,天蝎,打球
星座命理
看书,电影,美食,旅行,自由,听歌,旅游,音乐
游戏动漫,教育就业,汽车,时尚,娱乐
健康
文艺
新闻趣事
星座命理
简单,浪漫,music,微笑,自然,韩剧,时尚,健康,星座命理,双鱼座
旅游,名人明星,八卦杂谈,美容服饰,美食
美食
游戏动漫,吃货,画手,NEET
IT 数码
音乐,听歌,设计师
搞笑,淘宝,情感,上网,新闻,手机,网络,HR,移动互联网,娱乐
Android,社会,桌球,娱乐,设计师
时尚,健康,娱乐,星座命理,美食
旅游
游戏动漫
摩羯,强迫症,绘画
美女
汽车,游戏动漫,娱乐,美食
体育
葡萄牙,NBA,投资理财,体育,娱乐,策划,网络营销
娱乐,简单存在
军事,投资理财,汽车,教育就业,文艺,体育,娱乐,游戏动漫,旅游,IT 数码
文艺,美食
IT 数码,投资理财
八卦杂谈,摄影,唱歌,学生
游戏动漫,IT 数码,90 后
90 后
90 后
旅游,电影
喜欢听歌,计算机,美女,手机,拍照
本人性格不明,学生一枚
宅女,看书,动漫,睡觉,时尚,旅游,美食,80 后,电影,音乐
IT 数码,美食,新闻资讯,游戏动漫,投资理财
文艺,健康,旅游,美食
娱乐,美女
打篮球
体育
投资理财,健康,娱乐,美女

我爱UC浏览器
旅游,时尚,娱乐,文艺,健康
热爱生活,美食爱好者,策划
时尚
美食
新闻资讯,投资理财,健康,旅游
美食
情感生活
美女
新闻资讯,教育就业,文艺,体育
娱乐
亲情,幽默,好性格
时尚,文艺,游戏动漫,美食,小说,动漫,上网,旅游,宅
旅游,游戏动漫,投资理财,教育就业,军事,体育
海口生活
弹吉他,唱歌…
美食
IT数码,时尚,军事,旅游,娱乐
偶像剧,孩子气,看电视,喜欢旅游,自然,笑,迷糊,玩儿,旅游美食爱好者,乐天派
时尚,旅游
同性
视频音乐,美女
视频音乐
IT数码
新闻资讯
旅游
就是爱陈翔
美图摄影
旅游
投资理财,IT数码,美食,旅游,健康
文艺
体育
旅游,美食,微博,偶稀饭睡觉觉,旅行
营销
美女
90后
旅游
时尚,美食
美食
旅游,健康,美女,投资理财,美食
看書,美食控,動漫宅人,射手座,吉他,好书,美食,旅游,听歌,音乐
教育就业,娱乐
学习ing,处女座,旅游,时尚,艺术,音乐,google粉
射手座,搞笑,运动,娱乐,电影,音乐,80后

90
旅游
搞笑幽默,旅游
汪峰的粉丝
汽车,交友,唱歌,吃
淘宝购物,陈奕迅,宅生活,IT 数码,投资理财,娱乐,CDR,标志,logo,文学
运动,大学,浪漫,听歌,自由,数码,宅女,爱情,娱乐,时尚
名人明星,搞笑幽默,IT 数码,校园生活,视频音乐
90 后,喜欢听歌,交友,大学生,白羊座,爱唱歌,舞蹈,娱乐
教育就业,美食,时尚,健康,文艺
美食,星座命理,游戏动漫,文艺,娱乐
游戏动漫,汽车,文艺,IT 数码,娱乐
投资理财,旅游
花样美男,校园生活
宅,动漫控,书,music,篮球爱好者,学生一枚,书法,文艺,游戏动漫
汽车
有种幸福叫守候,电影,听歌
水瓶座,90 后,学生
娱乐,星座命理,金牛座,吃,电影,学生,听歌,90 后,睡觉,音乐
IT 数码,旅游,游戏动漫,电影
孝,音乐
娱乐
旅行,90 后
穿衣美容
旅游
IT 数码,军事,文艺,娱乐,体育
文藝,思子座,绝对的字恋
汽车
投资理财,汽车,时尚,娱乐,体育
汽车
萌宠
感性
旅游,新闻资讯,投资理财
教育就业
美女,游戏动漫,文艺,娱乐,音乐,睡觉
美食,旅游,游戏动漫
娱乐
懒,爱笑,游戏动漫,星座命理,美食
萌宠,文艺,IT 数码
健康,美食
微博,英语,A 股,处女座,大学生,乐观,睡觉,篮球,90 后,电影
旅游
新闻资讯,健康,美食,星座命理,投资理财
旅游

汽车,美食,时尚
时尚
美食,浙江生活,旅游,名人明星,视频音乐,音乐,电影,听歌,90后
电影,旅游,音乐
健康,体育,娱乐
美食
时尚,文艺,体育,娱乐,军事
旅游,健康,新闻资讯,投资理财,娱乐
微博奇葩
浙江生活
视频音乐,八卦杂谈,网络营销推广,网络策划,简约而不简单,外贸,旅行,生活,新闻,传媒
娱乐
美女,美食
文艺,体育,娱乐
健康
星座命理,美食,军事,教育就业
时尚,健康,投资理财,新闻资讯,娱乐,睡觉～发呆
旅游
萌宠,文艺,文学,90后
星座命理,美食,IT数码,文艺,娱乐,微博,历史,偶稀饭睡觉觉,艺术,销售
搞笑幽默,新闻趣事
爱自由,购物,淘宝,看电影,爱生活,爱睡觉,爱音乐,无厘头,海绵宝宝,娱乐
创新,网购,麦客庄园,创业
旅游,宅,时尚,电影,听歌,音乐,90后
旅游,游戏动漫,美食,娱乐,体育
美食,旅游
名侦探柯南
旅游,健康,新闻资讯,美食,IT数码
汽车,娱乐,投资理财,健康,旅游
时尚,旅游,星座命理,美食,娱乐
IT数码
资讯,音乐，看书，,海贼王,音乐,
名人明星,萌宠,美女,视频音乐,搞笑幽默
美女,娱乐,健康,投资理财,文艺
动漫,90后
投资理财
时尚,健康,旅游,美食,娱乐
旅游
摩羯女,EXO,Tiara,星座命理,时尚,娱乐
襄阳生活
娱乐
IT数码,娱乐,时尚,音乐,80后,宅
云南生活
时尚,教育就业,军事,IT数码,文艺

抚州生活,旅游,新闻趣事
文艺
纠结
简单快乐,向往自由与自然,淡然真诚,学无止境
美食
美食,娱乐,时尚,星座命理,文艺
海贼王,动漫,90 后,橱柜设计师
南京生活,新闻趣事,美图摄影,美食
爱吃泡面的家伙
视频音乐,星座命理
汽车,摄影爱好者,电气工程自动化,喜欢无线电,hifi
文艺
美食,午饭,狮子猫
美食,健康
IT 数码,文艺,美食,rap,system,biology
游戏动漫
旅游爱好者,IT,数码,自由职业,新闻趣事,宅,偶喜欢玩,狮子座,互联网
NANA,性手枪,朋克,英伦,RocknRoll,美食,文艺,游戏动漫
美食
美女,时尚,美食,娱乐
军事
汽车,旅游,健康,美女,IT 数码
娱乐,美女
美女
军事
搞笑幽默
电影,宅,90 后,时尚,听歌,音乐
军事,文艺,美女,星座命理,健康
健康
投资理财
美图摄影,新闻趣事,重庆生活,宅,射手座,读书
星座命理,文艺,教育就业,旅游
体育,汽车,旅游
天秤座,宅女,看小说,听歌,打游戏,喜欢天蓝,梦幻紫,黑,白,灰
旅游
时尚,旅行,自由,美食,80 后,听歌,旅游
娱乐,美女,汽车,投资理财
睡觉,健康,文藝,旅遊
美女,星座命理,搞笑幽默,新闻趣事,视频音乐
爱猫,桌球,宅,广告,90 后,吃货,睡觉
旅游
美食,文艺,时尚,健康,旅游
旅游,健康,星座命理,游戏动漫,文艺
IT 数码

旅游
我爱 UC 浏览器
美食
旅游
投资理财,美食,星座命理,健康
军事,旅游,IT 数码,新闻资讯
美女,新闻资讯
自然,军事,旅游,健康,汽车,体育
90,娱乐,IT 数码,体育,政治,科技,军事
文艺
美女
新闻资讯
新闻资讯,旅游,文艺,时尚,星座命理
旅游,八卦杂谈,视频音乐,校园生活,搞笑幽默,80 后
旅游,时尚,汽车,IT 数码,文艺,偏执狂,囧,涂鸦
时尚,星座命理,健康,旅游
90 后非脑残,多愁善感,喜欢唱歌,臭美,文艺
SuperJunior
投资理财,美女
星座命理
湖北生活
巨蟹座,英语,写作,微博控,小泷包,蓝色,宅,听歌,美食,旅游
美女
校园生活,体育资讯
体育
游戏动漫,旅游
体育,單車
IT 数码
时尚,旅游,微笑,看雪,听雨,旅行,听歌,低调,忧伤,随性
it,漫画,相声,爱吃,汽车,美食,IT 数码,体育,旅游
爱生活,爱音乐
游戏动漫
军事
美女
美食,健康,时尚,美食,旅游,娱乐
星座运势,美图摄影,网络,看书,偶稀饭睡觉觉,吃货,小说,篮球
时尚,旅游
穿衣美容,视频音乐,新闻趣事,哈尔滨生活,美女
听音乐,阳光,运动,细腻,丰富
旅游,星座命理,美食,文艺,娱乐
汽车
喜欢旅游,文艺,美食
游戏动漫,健康,美食,娱乐,IT 数码
搞笑幽默

星座命理,美食,新疆生活
朋友,投资理财,教育,逛街,军事
睡觉睡到自然醒,视频音乐,美容服饰,情感生活
星座命理,视频音乐,新闻趣事,美女,搞笑幽默
投资理财,汽车,美食,IT 数码,美女
爱情,创业,美女,听歌,天秤座,自由
旅游
旅游
IT 数码
健康,教育就业,星座命理,美食
美食,生活在别处
投资理财,时尚,音乐,旅行,宅
视频音乐
浴霸行业,商机,商友,互粉
旅游,美食,体育
体育
教育就业,美食,健康,星座命理,娱乐
苦力,建筑
健康,美食
新闻资讯,文艺,IT 数码
健康
汽车,音乐,游戏,射手座,电影
美食,搞笑幽默,微博奇葩,萌宠
自恋 ing,台球控,纠结控,蓝色,体育,娱乐
美食
文艺
军事,旅游,文艺,投资理财,教育就业
投资理财,美女,军事,IT 数码
旅游
健康,教育就业,星座命理,美食,娱乐
游戏动漫
中国人民大学,唐嫣,陈奕迅,动漫,梅尔吉布森,美食,英语,吃货爱美食,饿得快,帅哥爱好者
婚纱工作者
外貌协会
微博奇葩,美食,穿衣美容,星座命理
热爱生活,笑话,个性,书,80 後
旅游
旅游
文艺,美食,星座命理,健康,旅游
听歌,自由,旅行,时尚,旅游,美食,90 后,音乐,电影
美食
时尚,健康,教育就业,投资理财,美食
健康,新闻资讯,娱乐
时尚

新闻趣事,手机,时尚,媒体,听歌
时尚,IT 数码,汽车,旅游,体育
军事
外向,爱打篮球
量力而行
旅游,美食,IT 数码,体育,娱乐
健康,时尚,军事,旅游,娱乐
美女
旅游
汽车
娱乐
美术,投资理财,新闻资讯,旅游,健康,我乐于助人哦,CoolBoy
金光布袋戏,域界之初,霹雳布袋戲,游戏动漫
星座命理,搞笑幽默
财大 515,515
旅游
动漫,唱歌
爱电影,爱旅游,运动,努力,重庆,热爱生活,淡定,B 型血,TVB,健康
旅游,时尚,娱乐
游戏动漫
美女,文艺,工业设计,设计,设计师,汽车,体育
IT 数码
文艺,旅游
汽车
设计师,建筑,土木
体育,美食,IT 数码,军事,旅游,汽车
美女
爱音乐,爱和朋友喝酒,影视,爱吃,美食,体育
文艺,旅游,健康,什么都是浮云
文艺,80 后的尾巴,时尚,电影,旅行
新闻资讯,游戏动漫,体育,美食,旅游,80 后
英语,90 后,大学生
想睡觉,摩羯男
时尚,健康,旅游,美食,娱乐
唱歌,学生,手机,朋友,努力,美女,新闻资讯,星座命理,旅游,军事
80 后
游戏动漫,投资理财,旅游
新闻资讯,投资理财,健康,美食,体育
宅,听歌
汽车,投资理财,娱乐,体育
旅游
美女,星座命理
微博奇葩,穿衣美容
旅游

美食,旅游,时尚,娱乐,IT 数码
天蝎,人力资源,语录,体育
美女,军事
美女
美食,旅游,健康,美女,汽车,IT 数码
新闻资讯,美女,健康,星座命理,文艺
健康
文艺,旅游
旅游,时尚,娱乐,美食
学生一族,爱吃,喜欢旅游,懒人,双子,手机
星座命理,美食,游戏动漫
交朋友,奋斗,帅哥,单纯,篮球爱好者,朋友,热情,旅游,体育
烟台生活
我是一个温柔男,幽默男,还是一个孝男。
时尚,汽车,旅游,文艺,体育
娱乐
水瓶,学生一族,看小说,散步,影视,爱音乐,喜欢听歌
红酒知识学习,红酒达人,红酒控,红酒爱好者,红酒,旅行,艺术,网购,设计,创业
美容服饰,美图摄影,美食,心随意动,处女座洒脱,彩妆护肤,旅游美食,抠脚大汉,梦想
旅游,新闻资讯,汽车,军事,IT 数码
加拿大生活,休闲运动,休闲生活,音乐,钢琴曲,闲人,全职妈妈
美食
星座命理,文艺,IT 数码,军事,美食
搞笑幽默
健康,IT 数码,投资理财,教育就业,军事
娱乐,时尚
kanusoul
微博奇葩
美食,文艺
教育就业,投资理财,星座命理,美食,健康
时尚,旅游
名人明星
旅行,80 后,音乐,学生,自由,旅游,听歌
娱乐
猪扒包,美食
视频音乐
李晨,余文乐,陈奕迅,陈冠希,潮人,阿迪达斯三叶草,阿迪控,耐克控,品牌运动鞋,锐鞋网
游戏动漫,美食,旅游,星座命理,娱乐
宅
旅游,新闻趣事,视频音乐,美女
星座命理
汽车,旅行,音乐
游戏动漫
我就是我,夜猫子,神经大条,艾薇儿,娱乐,草莓酱,万能机器,90 后,爱情公寓,SHE

美女,教育就业,旅游,娱乐
长得没有存在感,哎……,娱乐
IT 数码
无与伦比的美丽
IT 数码,文艺,体育,教育就业
时尚,旅游,美食,文艺,娱乐
汽车,旅游,新闻资讯,文艺,时尚
名人明星,旅游,新闻趣事,穿衣美容
IT 数码
搞笑幽默,手机控,起床困难户
美女,星座命理,旅行,运动,善良,好书
IT 数码
娱乐
新闻资讯,健康,星座命理,时尚,娱乐
旅游
美图摄影,新闻趣事,宠物图集,情感生活,美容服饰
游戏动漫
美女,视频音乐,旅游,搞笑幽默
90 后,巨蟹座,宅女
校园生活
星座命理,搞笑幽默,睡觉,看书
游戏动漫,幽默,搞笑,游戏,上网,听歌,电影,80 后
古代人,历史,二货,伪文青但不装 B,80 后,摄影,HOCC,电影,旅游
旅游
IT 数码
宅
时尚
大爱 popping,Jamboom,壽司控,单细胞生物
投资理财
投资理财
美食
军事,汽车,体育
文艺,军事,投资理财
喜欢听歌,独自旅行,到处走走
上网,爱生活,购物狂,化妆,团购,潮,护肤,娱乐,时尚
旅游
新闻资讯
旅游
娱乐
喜欢旅游,快乐,爱旅行,搞笑幽默
IT 数码
星座命理
体育
搞笑幽默,星座命理,萌宠,名人明星

娱乐,教育就业,文艺,美食
少主本命,APH 深度中毒,银妈死忠党,我待瓶邪如初恋,瓶邪虐我千百遍
享受生活,享受人生
天秤座,网球,wow,书籍,登山,相声,公务员
时尚,文艺,健康,美食,娱乐
宅,电影,90 后,80 后
旅游,八卦杂谈,视频音乐,渭南生活,新闻趣事,陕西生活
健康,新闻资讯,游戏动漫,美食,星座命理
艺术,吃,摄影
星座命理
ACG,新闻资讯,体育,娱乐,游戏动漫
新闻资讯,军事,教育就业
投资理财,汽车,体育,旅行,旅游,美食,音乐,电影,80 后
美食
80 后的尾巴,b 型血,魔兽世界,爱玩,美女
佛山生活
新闻资讯,星座命理,文艺,宅,美食,旅游,90 后,听歌,音乐
文藝,电影
体育资讯,新闻趣事,美女,搞笑幽默,汽车,IT 数码,文艺,体育
股票
穿衣美容,吃,天蝎座,宅女,音乐,自由
投资理财,教育就业,旅游,军事,IT 数码
军事
IT 数码,星座命理
时尚,美食,旅游
网络,宅男,手机,摄影爱好者,动漫,电影,汽车,美女,英雄联盟
乐观,開朗
旅游,名人明星,视频音乐
娱乐
音乐,巨蟹座,文字,时尚,微博,网购,学生
投资理财,汽车,时尚,IT 数码,体育
娱乐
美食
游戏动漫,旅游,IT 数码
美女,时尚,星座命理,娱乐
吃货一枚,胡思乱想,自行车,书,喜欢旅游,休闲
旅游,星座命理,美食,娱乐,文艺
游戏动漫,健康,娱乐,旅游
伪文艺汉子,白羊座,星座命理,星座
散步,古典音乐,汽车,健康
军事
文艺,体育,娱乐
我就是我,旅游美食爱好者,军事,IT 数码
单身,自行车,忧郁,奋斗,平常心,心情,新闻资讯,音樂,体育,网络

健康,学生
旅游,摄影
军事,旅游,投资理财,文艺,体育
常德生活,旅游,星座命理,美食
新闻资讯
投资理财,教育就业,新闻资讯
健康
美食,听歌
搞笑幽默
时尚,汽车
爱自由
穿衣美容
微博奇葩
新闻资讯
健康,娱乐,旅游,文艺,时尚
90 后但我不脑残,音乐控,时尚,互粉,IT 数码,天蝎座,上网
Acan,Angelababy,YoonSoHee,bingbingbang,Kris,LUHAN,Joinsung,KimHyunA,Sehun,Krysta
l
健康,旅游,投资理财,美食,娱乐
美女,星座命理,娱乐
体育
娱乐
IT 数码,旅游
吃货一枚,腐女宅女耽美狼,御姐范儿,二货,肉末球儿
娱乐
娱乐
旅游,健康,汽车
风云再起
美食,娱乐
IT 数码,投资理财,时尚,星座命理
美食,旅游,新闻资讯,娱乐,汽车
喜欢旅游,90 后但我不脑残,音乐爱好者
电影,旅游,美食
搞笑幽默
娱乐
睡觉,美食控,懒,玩,爱唱歌,水瓶,独立,驴友,听歌,80 后
旅游,健康,娱乐,美食,文艺
狮子座,水瓶座,搞笑,双鱼座,写作,平常心,偶喜欢玩,囧,时尚,文学
星座命理
文艺,时尚,健康,美食,娱乐
健康,游戏动漫,星座命理,娱乐
健康
体育,工程,读书,营销,交友,旅游,80 后
文艺

旅行,电影,美食,90后,旅游
娱乐
星座命理
军事,新闻资讯
美食
星座命理,旅游,娱乐,美食,宅,时尚,90后
宠物图集,美容服饰,搞笑幽默,美食,重庆生活
吸血鬼日记,音乐发烧友,外贸,fashion,独立,狮子女,85后,武汉,文字,旅游
娱乐
文艺,美食,时尚,娱乐,旅游
美食,搞笑幽默,聪哥
游戏动漫
投资理财,美女,IT数码,娱乐,体育
游戏动漫,IT数码
搞笑幽默
穿衣美容
投资理财,文艺,时尚,美食,健康
游戏动漫,娱乐
music,逛街,台球,足球迷,天枰座
旅游,汽车,游戏动漫,体育
体育,AC米兰,足球,自由,吃,NBA
朴有天,孙燕姿,广播,名人明星,微博奇葩,搞笑幽默
穿衣美容
星座命理
星座命理
健康,美食,教育就业,自由,宅女,听歌,双鱼座
旅游美食爱好者,IT数码,娱乐
健康,音乐
美食,奇怪,大学生,自由,动漫,吃,巨蟹座,睡觉,看书,玩儿
投资理财,汽车,时尚,IT数码,美女
新闻趣事,视频音乐,名人明星,微博奇葩,美女,游戏动漫
游戏动漫
旅游
视频音乐,八卦杂谈,太原生活,美图摄影
旅游,星座命理,美食,文艺,娱乐
驴友,羽毛球,白羊座,90后,摄影狂
美食,健康,文艺,教育就业,旅游
体育,90后,篮球,天秤座,学生,囧
新闻资讯,投资理财,美女,美食,文艺,健康,IT数码,体育
文艺,体育,娱乐
看小说,爱幻想,英语,浪漫,紫色,服装,幸福,影视,时装
游戏动漫,体育
旅游,乐观,网络,旅行
新闻趣事

游戏动漫,文艺,星座命理,宅女,看书,小说,学生,动漫,上网,90 后
金牛座,宅女,小说,看书,动漫,学生,90 后,听歌,音乐
手机,乐观,动漫,上网,睡觉,自由,听歌,电影,美食
娱乐,游戏动漫,旅游,美女,IT 数码
投资理财,星座命理,军事,IT 数码,体育
穿衣美容
90 后
我爱 UC 浏览器
紫色,动漫控,完美主义,90 后但我不脑残,夜猫子,美剧迷,小说迷,美食
乐观,宅女,小说,交友,搞笑,幽默
天秤,音乐控,强迫症,神经大条,美食爱好者,摄影,宠物
星座命理
旅游
天秤,文艺,汽车,健康,IT 数码
加拿大生活
汽车,健康,旅游,美食,IT 数码
宅女加腐女,动漫迷,广播剧,爱玩,爱猫,双子座
90 后,听歌
美女
投资理财,旅游,娱乐,汽车
娱乐
娱乐
赛车,IT 数码,健康,文艺,体育
游戏动漫,投资理财,IT 数码
体育,旅行,玩儿,动漫
文艺
美女,汽车,旅游,美食,体育
健康
文艺,旅游,美食,90 后,学生,水瓶座,颜控,爱睡觉,爱舞蹈,爱音乐
文艺
游戏动漫,文艺,军事
健康,娱乐
IT 数码,时尚,星座命理,旅游,美食,处女座,仇玉党,懒人,欧美范,吃货
汽车,文艺
娱乐
健康,汽车,美女,文艺,体育
陈奕迅,活泼开朗,80 后的尾巴,偶喜欢玩,NBA,真诚,夜猫子,旅行,投资理财,体育
IT 数码,教育就业,投资理财,健康
IT 数码,文艺,娱乐,汽车,体育
投资理财,打球,喜欢旅游,热爱生活,纠结,内向,80 后
读书分享
娱乐,美女
体育
新闻资讯,星座命理,美食,娱乐,文艺

美食,偽 90 後,偽文青,蘇總後宮一員,愛做白日夢,小獅子
娱乐
创意,活泼,单身
体育
边伯贤,都暻秀,朴灿烈,行星饭,EXO,爱笑,双子座,阳光
法语教师
星座命理,健康,游戏动漫,游戏,美食,摄影,旅游,80 后
旅游
旅游
文艺,娱乐
时尚
狮子座,旅游,历史,电影
浙江生活
搞笑幽默,新闻趣事,穿衣美容,旅游,美食
教育就业
美食
旅游,美女,娱乐,IT 数码
军事,汽车
汽车,旅游
健康
游戏动漫,旅游,体育,双子,白色控,朴有天,公务员,运动,果粉,80 后
星座命理
新闻资讯,游戏动漫,娱乐
美食,搞笑幽默,穿衣美容
旅游,军事
旅游,新闻资讯,偶喜欢玩,生活,历史,娱乐
美食,旅游,时尚,娱乐
股票,财经,读书,旅行,生活,历史,足球,吃货,营销,文字
90 后,音乐,动漫,学生
军事
天秤座,游戏,运动,睡觉,宅,旅行,80 后
旅游
游戏动漫,汽车,体育,电影爱好者,巧克力,射手座,wower,80 后
摄影爱好者,IT 数码
穿衣美容,天秤座
新闻趣事,平常心,巨蟹座,音乐,阅读,乐观,睡觉,吃货,90 后
80 后
微博奇葩
美食,旅游,美女,穿衣美容
80 后,时尚
新闻资讯
旅游
文艺,IT 数码,旅游,星座命理,娱乐
旅游,文学,读书

粉红色暴力熊,宇宙大爆炸,崔胜贤TOP,TOP,GDragon权志龙,Bigbang的VIP,BigBang
娱乐,时尚,汽车,旅游,体育
娱乐
教育就业
汽车,用心交流
旅游
运动,篮球,体育
学生一族,shopping,蓝色,瘦身,旅游,星座命理,娱乐,汽车,IT数码
娱乐
视频音乐
时尚,健康,美食
90后
视频音乐
文艺,娱乐
娱乐
笑点低哭点高,初三党,Mk狂,左溢哥哥,小小溢声军
时尚,听歌,90后,学生,自由,宅
游戏动漫
体育,汽车,宅,听歌,时尚,电影,音乐,90后
IT数码,CF,金鱼,小狮子,摩羯座,在校学生
金华生活,Music,看书,动漫,80後
教育就业
宅,旅游
时尚
美食
娱乐
星座命理,旅游,汽车,美食,娱乐
旅游
汽车
文艺
健康,体育,IT数码
娱乐
娱乐
IT数码
游戏动漫
美食
健康,旅游,军事,娱乐,体育
90,80,刘忻,服饰,时尚
睡觉,宅,旅行
白湖
囧,吃,小说,看书,上网,宅,电影,80后
美女,汽车,游戏动漫
设计,建筑
健康,旅游,汽车,娱乐

文艺
音乐,美食,旅游
投资理财,IT 数码,汽车,时尚,美女
萌宠,美食
镇江生活,时尚
IT 数码,军事,美女
美女
游戏动漫
健康,旅游
文艺
健康,旅游,文艺,体育
美食
学车,租车,卖车,买车,汽车网,汽车
文艺,旅游,美食,娱乐
时尚,健康,旅游,美食,文艺,偶喜欢玩,阅读,乐观,设计,处女座
旅游
文艺,时尚,旅游
IT 数码
90 后,大学生,摩羯座
军事,汽车
游戏动漫,旅游,汽车,IT 数码,美女
军事,IT 数码
文艺
娱乐,慢热,双子座
娱乐
军事,美女,IT 数码
热爱生活,香港,笑话,营销,文字,历史,互联网,IT 数码,投资理财,旅游
体育资讯,职场招聘,IT 数码,搞笑幽默,乌鲁木齐生活
运动,体育模特,健康,时尚
青岛生活
娱乐,教育就业,美食,旅游,时尚
健康,美食,游戏动漫,文艺,旅游
体育
文艺,体育
时尚,文艺
游戏动漫,汽车,IT 数码,游戏,小说,金牛座,上网,宅,好性格,幽默
娱乐,美食,星座命理,旅游
时尚,旅游,星座命理,美食,娱乐
电影
旅游
爱自由,爱生活
生活,les,咖啡,狗狗,神经大条,人生,医学,我就是我,90 后非脑残
美女
美食

体育
电影
娱乐,健康,时尚,美食
娱乐
水瓶座,游戏动漫
投资理财,电影,八卦
健康,文艺
美食,游戏动漫
军事
旅行,自由,睡觉,摄影爱好者,时尚,数码,创业,美食,动漫,80后
星座命理,健康,时尚,投资理财,教育就业
健康,汽车,教育就业,投资理财,娱乐
娱乐
娱乐
美食,军事
1对1,中考
美女,游戏动漫
体育
娱乐
投资理财
新闻资讯,美食,时尚,汽车,娱乐
体育
文艺,广告,艺术,旅游
旅游
淘宝直通车,客服托管,直通车托管
旅游
文艺,教育就业,健康,旅游,星座命理
健康
我爱UC浏览器
鬼畜没有,纯手绘,郁闷,双生子,音频怪物,鼠标使用困难户,中二久治不愈,静候灵归
旅游
惠州生活
健康
娱乐,体育
旅游,时尚,美食,教育就业,新闻资讯,健康,文艺
时尚,旅游,星座命理,健康,投资理财
学生
旅游
旅游,健康,美女,体育,时尚
新闻资讯
娱乐,体育,美食
品牌营运,商业空间设计,品牌终端,广告传媒
教育就业,时尚,旅游,健康,文艺
娱乐

美食,IT 数码,军事,教育就业,美女,游戏动漫,文艺,投资理财,新闻资讯,娱乐
娱乐
星座命理,汽车,文艺
美女,游戏动漫,时尚,娱乐,文艺
性感,单身,萝莉控,盗墓笔记,感性,登山,旅游爱好者,经济,电影控
搞笑幽默
游戏动漫
文艺,好样的
星座命理,美食,文艺,时尚,游戏动漫,天平哦,不抗拒萌物
娱乐
健康,90 后,自由,旅游,运动
美食,宅女一枚,懒人
教育就业,投资理财,军事
美食,萌宠,星座命理,旅游
美食
暴躁
投资理财,新闻资讯,健康,旅游,美食
游戏动漫
教育就业
时尚,健康,美食,旅游,娱乐
美食,旅游,游戏动漫,娱乐,文艺
新闻资讯
汽车,投资理财
服饰,会计,时尚,旅游
文艺
孩子起名,农民工,农村人,处女座
新闻趣事
时尚
美食
乐观,体育,水瓶座,90 后
投资理财
体育资讯
广东生活,萌宠
健康
美食
星座运势,动漫迷,宅女一枚,摩羯女,小清新,微笑
旅游,体育
美女,娱乐
娱乐
体育,娱乐
星座运势,美图摄影,视频音乐,校园生活,美食,宅,音乐,90 后,听歌
广西生活,娱乐,禁忌,未来,是否可解释为,不会来
视频音乐,美食,名人明星,旅游,鸡涌,TOP,VIP,BIGBANG
胡思乱想,怀旧,随性,偏执狂,桌球,服饰,Music,温柔,可爱,爱睡觉

旅游,搞笑幽默,微博奇葩,美食
健身爱好者,穿衣美容,法語爱好
汽车,投资理财,军事,IT 数码,文艺
IT 数码
美女,娱乐,体育,IT 数码
广东生活,体育课,蓝球
新闻资讯,时尚,美食,旅游,教育就业,娱乐,摄影
娱乐
投资理财,文艺,旅游,美食
游戏动漫,宅,耽美,吃和睡
唱歌,浪漫,快乐大本营,商业,平常心,香港,善良,音乐迷,努力,梦想
美食
IT 数码,健康,旅游,美食,娱乐
健康,体育,娱乐
娱乐
IT 数码
C 语言,linux,p1000,android
不宅会死,购物狂,剁手党,面瘫控,古风爱好者
摄影爱好者,双子座,爱烹饪,周杰伦
美食,健康,投资理财,旅游,娱乐
电影,时尚,旅游,白羊座,摄影,唱歌,音乐
旅游,IT 数码,美女,汽车,投资理财
IT 数码,娱乐
视频音乐,新闻趣事,军事天地,烟台生活
新闻趣事
时尚,健康
旅游,苏州生活
吃货一枚,Music,沉默,娱乐,文艺,健康
游戏动漫
汽车,IT 数码,旅游,星座命理
体育
美女,汽车,美食
星座命理,教育就业,新闻资讯,投资理财,军事
八卦杂谈,平常心,运动,看书,金牛座,羽毛球,吃,文学,宅男,动漫
八卦杂谈,新闻,美剧,吃,电影
时尚,教育就业,美食,文艺,娱乐
羽毛球,唱歌
上海生活,新闻资讯
宠物图集
健康,文艺
爱情,浪漫,唱歌,音乐,巨蟹座,时尚,街拍,美食,旅遊,運動
美食,广东生活,80 后
体育,娱乐,旅游,军事,美食,80 后,音乐,电影
汽车,NBA

盗墓笔记,旅游爱好者,努力,活泼开朗,射手座,爱音乐,听音乐,星座命理,90后,上海
旅游,疯纸
IT数码,体育,健康
健康,新闻资讯,教育就业,投资理财
播音高考培训,播音主持培训,艺考培训,艺考集训,艺考,艺术高考,新锐艺术培训,成都新锐艺术,艺术高考培训,成都艺术培训
娱乐,文艺,IT数码,时尚,旅游
IT数码,体育,文艺,娱乐
美女,游戏动漫,娱乐,IT数码
娱乐,健康,美食
星座命理
文艺
穿衣美容,美食,萌宠,娱乐
投资理财,星座命理,文艺
健康
娱乐,健康,旅游,唱歌,乐观,听歌
时尚
文艺,时尚,投资理财,旅游,教育就业
体育,旅游,美女,汽车,IT数码,篮球
IT数码,时尚,健康,新闻资讯,旅游
娱乐
旅游,名人明星,美女,新闻趣事,搞笑幽默
投资理财
美食
新闻趣事
健康
文艺,体育,poppin,伍恒,时尚,街舞
旅游
时尚
美食
新闻资讯,军事
教育就业,新闻资讯,军事,时尚,娱乐
娱乐
体育资讯
新闻资讯,旅游,游戏动漫,IT数码,军事
投资理财,新闻资讯,旅游,体育
科技,投资理财,IT数码,军事,体育,娱乐
篮球控,90后,逍遥,一个努力的孩子,工作狂人,汽车人,电脑控
投资理财,体育
体育
娱乐,健康,美食,旅游
娱乐
旅游,娱乐
工业设计,冷笑话,自然醒,跑步,拖延症,设计,画画,music,五月天

魔蝎座,k 歌,k,古灵精怪,宅女一枚,无厘头
搞笑幽默
美食,时尚,健康,旅游,娱乐
游戏动漫,美女,新闻资讯,旅游,美食
娱乐
时尚,健康,旅游,美食,投资理财
娱乐
教育就业
美女,教育就业,投资理财,汽车,体育
游戏动漫
IT 数码
军事,美女
投资理财,新闻资讯,旅游,汽车,IT 数码,宅,听歌,音乐,90 后
新闻资讯,美食
旅游,美食
澳大利亚生活,美女
创业,天秤座,自由职业,文学
旅行,旅游,音乐
娱乐,星座命理,军事,体育
美食
健康
淡定,音樂,投资理财,军事,健康,新闻资讯
旅游,健康,时尚,新闻资讯,文艺,娱乐
游戏动漫
旅游,投资理财,健康
旅游
时尚,美女
娱乐
文艺
游戏动漫
SHINee,金钟云
时尚,娱乐,旅游,美女,健康
户外,寂寞,学生一枚,张杰,K 歌,创业,阅读
医生
星座命理,新闻资讯
溜冰
美食,教育就业,星座命理,IT 数码,旅游,美食
投资理财,新闻资讯,教育就业,游戏动漫,文艺
新闻资讯
美女,美食,娱乐,文艺,体育
美女,玩儿
搞笑幽默,美食,娱乐
新闻资讯,投资理财,教育就业
美食

娱乐
杰娜永恒,爱晨晨
旅游
娱乐,时尚,汽车,IT 数码
驴友,体育,天蝎座,去野
文艺,体育,娱乐
游戏动漫,旅游,星座命理,美食,文艺
旅游
娱乐
星座命理
旅游,热爱自由,摄影控,柯迷,宅
吃货,自由
旅游
旅游,健康,文艺,时尚
时尚,娱乐,美食,旅游,星座命理
娱乐
美食,娱乐,文艺,健康,旅游
美食,星座命理,旅游,健康,时尚
视频音乐,萌宠,天津生活,微博奇葩,搞笑幽默,狮子座,宅女,上网
Eason,旅游,双子座,幽默,足球,音乐
美食,汽车,时尚,旅游,美女
健康,星座命理,游戏动漫,美食,文艺,娱乐
健康,旅游,娱乐,IT 数码,美食
星座命理,金牛座,90 后,偶稀饭睡觉觉,善良,大学生,自由,唱歌,吃,动漫
旅游,搞笑幽默,淄博生活,军事,体育
教育就业,健康,旅游,美食,娱乐
名人明星
文艺
时尚,香港藝人,手机微博,自由,狮子座
80 后,香港藝人,感动
篮球
新闻资讯
爱电影,单身,上网,唱歌,幽默,桌球,视频音乐,新闻趣事,搞笑幽默
新闻资讯,体育,教育就业
娱乐
汽车,游戏动漫
教育就业,文艺,美食,旅游,娱乐
新闻趣事,吃喝,电玩,羽毛球,甜美,忧伤,爱音乐,爱逛街,掏宝控,狮子座
旅行,素颜控,宅女
旅游,美食
娱乐
文艺,时尚
IT 数码
体育

搞笑幽默
双鱼座,梁咏琪,美剧控
微博奇葩
服饰,周杰伦,足球,忧郁,劲舞,古典音乐,爱新鲜。爱冒险。,文字控,小清新,时尚
健康,时尚,文艺,娱乐
单身,爱旅游,漫画,感性,创意,音乐爱好者,销售,执着,旅游美食爱好者,摄影控
汽车
旅游,文艺
腐,中二,囧,美剧,偶稀饭睡觉觉,搞笑,宅女,动漫,80后
小说,歌手,彩信
视频音乐,IT数码
娱乐
娱乐
文艺,体育,美女,汽车,旅游
旅游
时尚,娱乐
娱乐,汽车,体育,时尚
美食
旅游
投资理财,旅游,健康,军事,IT数码
NBA,星座控,IT互联网
旅游
穿衣美容,90后但我不脑残,水瓶女,神经大条,爱自由,懒人,淡定,宅,上网
旅游,娱乐
IT数码
时尚
美食,文艺,娱乐
体育
唱歌,乒乓球,NBA,音乐
教育就业,旅游,星座命理,军事
美女
健康
美女,游戏动漫
教育就业,星座命理,IT数码
搞笑幽默
IT数码,看小说,喜欢旅游,电影控,任性,做梦,孩子气,神经大条
游戏动漫,IT数码,文艺
搞笑幽默
文艺
健康
星座命理
美图摄影
名人明星,新闻趣事,星座命理,山东生活,文艺
IT数码

穿衣美容
音乐
健康,旅游,美食,星座命理,娱乐,时尚
体育
投资理财,汽车,教育就业,体育
坚强,思考,奋斗,努力,哈哈,我就是我,游泳,逛街,开朗,画画
教育就业,投资理财,IT 数码
互联网
游戏动漫,时尚,投资理财,IT 数码,娱乐
健康,教育就业,投资理财,军事,IT 数码
文艺,潮妞,牛 X
新闻趣事
健康,新闻资讯,美女,游戏动漫,旅游
爱音乐不解释,水瓶座不解释,森系不解释,微博控不解释,小清新不解释,互粉
喜欢张杰谢娜,张杰控,张杰滴学生星,张杰我的偶像,张杰的星星,张杰
文艺
唱歌,网购,艺术,偶稀饭睡觉觉,浪漫,自由职业,旅行,时尚,搞笑,听歌
美女,军事,文艺,平常心,善良,天秤座
体育
文艺,旅游,星座命理,体育,娱乐
娱乐,游戏动漫
多愁善感,简单,古灵精怪,快乐大本营,敏感,古典音乐,低调,双重性格,服饰,爱睡觉
微博奇葩
汽车
旅游,时尚,读书,音乐
文艺
游戏动漫,娱乐,游戏,宅男,美食,历史,动漫
美女
美女,新闻资讯,IT 数码,军事,游戏动漫
游戏动漫
搞笑幽默,视频音乐,起床困难户,娱乐,上网,90 后,音乐
新闻资讯,旅游,时尚,文艺,娱乐
健康,星座命理,旅游,时尚,娱乐
体育
旅游,80 后,美食,睡觉
娱乐
美食,文艺,体育,投资理财,娱乐
文艺
新闻趣事,白百何迷,范范迷
美女
游戏动漫
囧,双子座,游戏,搞笑,幽默,上网,小说,动漫,宅女
汽车,娱乐,美女,旅游,新闻资讯,体育,游戏动漫
娱乐

時尚,娛樂
穿衣美容,旅游,微博奇葩,健康
黄金俏女郎,木村沙耶香,向上帝调头,站台,风语战士,范怡文,三眼神童,变种女狼
微博奇葩,旅游
娱乐
宅女,吃货,幽默,天蝎座,90,听音乐,语录,博客,双重性格,爱美食
旅游,IT 数码,娱乐,游戏动漫,美食
逛街,旅游,美食,街拍
游戏动漫,旅游,体育
文字,时尚,浪漫,学生,双鱼座
Single,单身,星座命理,IT 数码,娱乐,文艺,体育
旅游,军事,文艺,体育,娱乐
健康,体育,娱乐
娱乐,体育,游戏动漫
山东生活
新闻资讯
开心,乐天派
水瓶座,旅行
游戏动漫,投资理财,旅游,体育,喜欢睡觉旅游
体育,娱乐,投资理财,时尚,新闻资讯
新闻资讯,教育就业,美食
美食
体育,军事,美女,汽车,IT 数码,交友,天蝎座,玩儿,睡觉,宅
IT 数码
搞笑幽默
新闻资讯
健康,旅游,美食,文艺,教育工作者,美食爱好者,摄影旅游,钢琴黑白键,艺术,国画
视频音乐,yuchun,健身,金融,阅读写作,音乐,摩羯座,森林
穿衣美容,美食,搞笑幽默,微博奇葩,浙江生活
旅游
美食
旅游
郑州生活
视频音乐,90 后,天蝎座,上网
戚薇
旅游
美食,旅游
设计,小资,猫控,室内设计,学生一枚,跳舞
IT 数码,文艺,健康,娱乐,星座命理
健康,时尚,旅游,IT 数码,体育
教育就业,军事,文艺,体育,投资理财
旅游,发呆,网购,睡觉
学生
足球,羽毛球,旅游,电影

亲情,微博,平常心,内向,文静,上网,天蝎座,旅游,听音乐,90
宅女,金牛座,90 后,平常心,网购
英国,愤青,散步,影视,手机看小说,旅游,音乐
旅游
吃货,阅读
健康,汽车,体育,旅游,军事
完美主义,旅游,健康,美食,羽毛球
吃货
娱乐
娱乐
宠物图集
汽车
教育就业,投资理财
美食,旅游
时尚,旅游
军事,投资理财,汽车,体育
健康,旅游,星座命理,美食,娱乐,懒
旅游,星座命理,美食,IT 数码,娱乐
游戏动漫,旅游,美食,星座命理,娱乐
美食
旅游,搞笑幽默,新闻趣事,美女
文艺,IT 数码,星座命理,游戏动漫
微博奇葩,西安生活,娱乐,文艺,旅游,美食,星座命理,游戏动漫
河北生活
名人明星,星座运势
双重性格,继承者们,F（x）,完美主义,韩国,幽默,无厘头,娱乐,明星
80 后,射手座,好书,网购,运动,摄影
90 后
搞笑幽默,服装设计,爱生活,爱睡觉,绘画,我就是我,神经质,吃喝玩乐
娱乐
健康,文艺
健康,90 后,旅游,小说
娱乐
健康
体育,健康,星座命理,军事,IT 数码
睡觉,听音乐,加班 ING,旅游,减肥 ING,看电视
文艺,旅游
内裤文化,男士内裤,内裤控,穿衣美容,名人明星
时尚,文艺,游戏动漫,娱乐,体育
文艺
IT 数码,军事,新闻资讯,旅游
旅游,健康,星座命理,娱乐,军事
娱乐,星座命理
旅游,文艺,IT 数码,军事,双子座

星座命理,军事,健康
汽车,美食,新闻资讯,IT 数码
微博,动漫,小说
新闻资讯,文艺,军事,教育就业,投资理财,星座命理,体育,汽车
襄阳生活,新闻趣事,校园生活,美女
IT 数码,旅游,游戏动漫,时尚,体育,足球
视频音乐,旅游,美女,搞笑幽默,青海生活
投资理财,娱乐
投资理财,美女,美食,时尚,军事
旅游,新闻资讯,时尚,美食,IT 数码
游戏动漫,时尚
时尚,娱乐
微博,读书,动漫,游戏,天秤座,90 后
汽车,教育就业,体育
搞笑幽默,IT 数码,时尚
旅游,美食,娱乐,新闻资讯,生活,善良,大学生,偶稀饭睡觉觉
娱乐
汽车,摄影爱好者,天蝎座,股票,旅游,娱乐,搞笑
IT 数码,健康,娱乐
健康,旅游,IT 数码,美食,时尚
美女,美食,体育
娱乐,时尚,旅游
娱乐,小说,偶喜欢玩,听歌,音乐,上网,90 后
时尚
v 家,大小姐,东方 Project,动漫,IT 数码
娱乐,健康,星座命理
游戏动漫,娱乐,军事
健康,教育就业,体育,投资理财,新闻资讯
魏晨,双鱼座
体育
健康,旅游,文艺,美食,游戏动漫,时尚,汽车
娱乐
旅游,新闻资讯,文艺,美食
美食,视频音乐,天蝎座
美女,重口味,护肤,欧美控,新浪读书,健身,投资理财,娱乐,体育,军事
IT 数码,健康,星座命理,体育,娱乐
汽车
IT 数码,娱乐,投资理财,汽车,游戏动漫
旅游
美食
时尚
微博奇葩,新闻趣事,视频音乐,美女,搞笑幽默
美食,旅游,浙江生活
娱乐

美女,微博奇葩,视频音乐,新闻趣事,搞笑幽默
爱笑,快乐,大学,爱吃,周杰伦,90 後,爱电影,教育,逛街,娱乐
IT 数码,街舞,羽毛球
体育
天津生活
游戏动漫,美女,文艺
搞笑幽默,星座命理,穿衣美容,微博奇葩,美女
美食
IT 数码,娱乐,体育,汽车,时尚
汽车,军事
投资理财,IT 数码
美食
健康,时尚,汽车,美女,军事
娱乐
新闻资讯,娱乐
娱乐
新闻趣事,搞笑幽默,美女,美食,微博奇葩
IT 数码,时尚,旅游,旅行
投资理财
旅游,星座命理,文艺,美食
娱乐,汽车
动漫
美女,健康,军事,娱乐,体育,文艺
睡觉睡到自然醒,猫控,懒,吃货一枚,重口味,银魂,宅女加腐女
投资理财,重口味心理学,药罐子,彪悍女屌丝,仇子明滴脑残粉,猥琐大婶,耽美文
游戏动漫,娱乐,动漫,电影
投资理财,新闻资讯,时尚,健康
旅游,美女,时尚,娱乐
游戏动漫
历史,时尚,军事,美女,体育,IT 数码
星座命理
美食,新闻资讯,IT 数码,时尚,旅游
美食,娱乐,游戏动漫,健康,旅游,星座命理
情绪化,考研,旅游,美女,健康,时尚,娱乐,文艺,教育就业
娱乐,旅游,美食,星座命理
游戏动漫,军事
娱乐,健康,时尚,旅游,紫色
穿衣美容
时尚
新闻趣事,星座命理,旅游,美食,浙江生活,汽车
健康,旅游
游戏动漫,娱乐,干物女,爱自由,爱睡觉,驴行,动漫迷,85 后,腐妞,天蝎座
星座命理,文艺
美女

音乐爱好者,睡懒觉,单纯,善良,吃
汽车
体育
体育
善良,活泼,开朗,善良!
销售,80 后的尾巴,爱生活,粤语,热情,创业,翻译,英语,音乐
IT 数码
体育
旅游,美食,娱乐,体育
汽车,美女,游戏动漫,体育,IT 数码
旅游,时尚,教育就业,投资理财,健康
时尚,美食,旅游,娱乐
IT 数码,旅游,美食
新闻趣事,搞笑幽默,重庆生活
旅游,新闻资讯,教育就业,星座命理,美食
美食,旅游,星座命理
旅游,时尚,健康,汽车,投资理财
旅游,时尚,娱乐,美食
山东生活,乐天派
搞笑幽默,美女
设计师,金在中,superjunior,爱猫,郭敬明,怀旧,高三,文艺,旅游
巨蟹,星座命理
体育
旅游,健康,教育就业,投资理财,军事
体育
射手座,睡觉,美食,90 后,网购,旅游,宅,吃货,偶喜欢玩,听歌
健康
IT 数码
美容服饰,环保,音乐发烧友,时尚达人,社会
投资理财,健康
文艺,美食,军事,IT 数码,体育,娱乐
汽车,一吃货,绿源人,环保,爱旅游,户外,玩儿,听歌,历史,90 后
LOL
投资理财,星座命理,旅游,汽车,体育
体育
美食,健康,旅游,教育就业,娱乐
教育就业,美食,军事
健康,旅游,IT 数码
投资理财,军事,文艺
新闻资讯,投资理财,旅游
旅游,爱摩羯,天蝎女,迷糊虫,音乐控,电影,陈绮贞,前田敦子
美食,旅游,美国,汽车
美图摄影
娱乐,星座命理,体育,摩羯,学生一族

认真生活,娱乐
射手座,学生
新闻资讯
时尚,旅游,健康,美食,文艺
音乐
美食控,阳光,开朗,校园生活,视频音乐,美图摄影
新闻资讯,体育,健康,时尚,IT 数码,美女
星座命理,游戏动漫,体育
旅游
美女,军事,IT 数码
旅游,汽车,新闻资讯,军事,IT 数码
旅游
旅游
外星人,山内瑞玲,霹雳女狼星,我要你忘了,阴间来客,东亿,梦飞船,二亮浪漫曲
外籍,雇佣兵
平凡
娱乐
美食
雅思炉中的烤鸭,考研准备中,旅游,自由,美食
投资理财,时尚,旅游
穿衣美容,旅游,微博奇葩,天津生活
搞笑幽默,军事
美女
美食,文艺,汽车,游戏动漫,体育
旅游,体育
游戏动漫,投资理财,IT 数码
时尚,汽车,娱乐
娱乐
文艺,娱乐,健康,新闻资讯,体育
吃,宅
游戏动漫
时尚
娱乐,体育
娱乐
时尚,旅游,美食,健康
健康,教育就业,投资理财
文艺
旅游
星座命理,美食
IT 数码
星座命理,娱乐
民主自由,王菲持续迷恋者,旅行,美女文青,开放但不放荡
汽车,旅游,IT 数码,健康,教育就业,军事
新闻趣事

旅游,星座命理,美女,IT 数码,体育,55555
健康,文艺,体育
天津生活
健康,文艺
汽车
汽车,健康,星座命理,旅游
新闻趣事,健康
时尚,美食,星座命理,文艺,旅游
IT 数码
健康,IT 数码,美食,时尚,娱乐
搞笑幽默,90 后
娱樂
新闻资讯
IT 数码
青春,校园,学生,摩羯座,设计,网络
旅游,星座命理
教育就业,新闻资讯
新闻资讯
单身,天秤座,宅男,80 后,美女,汽车,旅游,IT 数码
军事
美食,新闻趣事,星座命理,穿衣美容,搞笑幽默,教育就业,新闻资讯,文艺,娱乐,健康
天秤座,美剧迷,it 互联网,爱睡觉,爱生活,爱吃,旅游爱好者,生活大爆炸,个性,美食
自恋狂,蘑菇头,小金鱼,眼镜控,阿狸控
娱乐,美食,星座命理,时尚,旅游,游戏动漫,文艺
旅游,游戏动漫
水瓶座,吃,唱歌,90 后
慢热后得瑟,叔控,努力奋斗中
美女,旅游,军事,文艺,体育
旅游
游戏动漫
美女,娱乐
娱乐
时尚,听歌,旅游,美食,电影,80 后,音乐
健康
美食
体育
文艺
巨蟹座
微博奇葩,搞笑幽默,旅游,美食,辽阳生活
文艺,游戏动漫,90 后
娱乐,星座命理,美食,教育就业
军事,游戏动漫,娱乐,投资理财,健康
互粉,钟意交友,90 后一追求淡定,90 后非脑残,姊妹之上
IT 数码,90 后,爱难过

萌宠,穿衣美容,星座命理,新闻趣事,上海生活
学生一族,低调,90 後
娱乐
广东生活
星座运势,美图摄影,八卦杂谈,新闻趣事,名人明星
新闻资讯,健康,美食,文艺,体育
投资理财,教育就业,健康,新闻资讯,IT 数码
美女,旅游,文艺,体育
文艺,游戏动漫,IT 数码
我爱 UC 浏览器
美食,星座命理,旅游,时尚,娱乐
时尚,IT 数码
文艺,娱乐,小说,听歌,音乐,亲情,时尚,上网,电影,美食
剑网三,星座命理,旅游
美女
星座命理,投资理财,时尚,健康,旅游
新闻趣事
文艺,体育,娱乐
教育就业
新闻资讯,美女,游戏动漫
画画,巧克力,书,music,发呆,看电影,旅游,文艺
美女,巨蟹座,80 后,吃货,旅行,看书,平常心,动漫,历史,网络
动漫,美女,游戏动漫,体育
时尚,健康,旅游,军事,新闻资讯
穿衣美容,搞笑幽默,福建生活,新闻资讯,健康,旅游,美食,文艺
游戏动漫
IT 数码,星座命理,娱乐,文艺,时尚
旅游
文艺,时尚,美食,健康,体育
军事,IT 数码,时尚,体育,汽车
美食
新闻资讯,教育就业,健康,旅游,美食
IT 数码
新闻趣事,教育就业,娱乐,旅游
名人明星,IT 数码,体育资讯,新闻趣事,搞笑幽默
健康,IT 数码
美食,星座命理,IT 数码,体育,娱乐
新闻资讯,美食,星座命理,游戏动漫,健康
美女
减肥,逛街,拍照,吃喝玩乐,喜欢音乐,懒人
投资理财,时尚,健康,美食,娱乐
军事,星座命理,新闻资讯,健康,IT 数码
音乐,电影,90 后
美食,音乐,听歌

美女,投资理财,IT 数码,美食,旅游,游戏动漫,体育,娱乐
娱乐
IT 数码
体育
健康,新闻资讯,娱乐
投资理财,时尚
文艺
怀旧,粤语,执着,电脑,电影迷,自由职业,体育
星座命理,游戏动漫,IT 数码,蛋疼
旅游
快乐,睡觉,宅,旅游
笑,健康,旅游,星座命理,美食
健康,旅游
游戏动漫,美食,IT 数码
美食
欧美音乐,爱设计,安静,女王范儿,潮,绘画,小清新,爱逛街,文艺
新闻资讯,时尚,文艺,娱乐,天蝎座,80 后,旅游,搞笑,上网,音乐
Taylorswift,读书,爱自由,旅游美食,新闻资讯,游戏动漫,文艺,IT 数码
末班车,威乐,童话演唱团,那么骄傲,爱是怎样炼,胡畔,爱过不伤心,火烧碧云宫
美女
娱乐
旅游,教育就业,星座命理,健康,娱乐
我爱 UC 浏览器
投资理财,汽车,时尚
宅
健康,旅游,美食,时尚,网购,音乐,射手座,好性格
健康,美食
体育,文艺
美食,旅游,汽车,娱乐,IT 数码,好性格,摄影,运动,偶喜欢玩
美女,汽车,旅游,IT 数码,体育
健康,娱乐,教育就业,新闻资讯
夜猫子,时事,欧美音乐,新闻资讯
美食
健康
IT 数码,星座命理,美食,美女,游戏动漫
健康,旅游,美食
游戏动漫
旅游,娱乐,文艺
IT 数码,文艺,娱乐,时尚,旅游
Eminem
娱乐
体育
美女,军事,健康,旅游,IT 数码
娱乐

做梦,宠物,音乐爱好者,动漫控,文艺,游戏动漫
养生,运动,收藏
旅游
健康,时尚,旅游,美食,文艺
LES,文艺,时尚
汽车,旅游,教育就业,体育
旅游
新闻趣事,星座命理,美食,旅游
i,SuperJunior,O 型血,Music,文艺,娱乐,健康,游戏动漫,星座命理
时尚,游戏动漫,娱乐,IT 数码,美女
喜欢听歌,90 后,纠结,单身,阳光,善良,狮子座,偶稀饭睡觉觉,大学生,爱幻想
郑秀妍
旅游,娱乐,教育就业,IT 数码,时尚
教育就业,文艺,军事,旅游,娱乐
新闻资讯,健康,时尚,文艺,娱乐
美女,IT 数码
80 后,电影,学生
美女
健康
星座命理,IT 数码,文艺,娱乐,健康
宅女,娱乐,音乐,时尚,80 后
冷笑话不冷么,减肥只是说说啦,睡睡睡睡睡不醒,疯疯疯疯人愿,小小泰迪熊,宅宅宅,爱旅游,电音,爱生活爱乐乐,O 型血
美食,旅游,搞笑幽默,穿衣美容,星座命理,军事
文艺,崇崇
娱乐
微博奇葩,搞笑幽默,教育就业,游戏动漫
教育就业
复合管技术,铝塑复合管,钢塑复合管,RTP 复合管,复合管
名人明星
星座命理
旅游,娱乐
学生
旅游,星座命理,健康,军事,文艺
单身,好性格,学生一族,爱吃,品牌,旅行,交朋友,看书,品位,双鱼女
旅游,时尚,水瓶座
健康,购物,返利,淘宝,高返利
健康,新闻资讯,娱乐,教育就业
热爱生活,爱自由,爱美食,爱音乐,爱旅行
自由,时尚,音乐,文职人员
无锡生活,穿衣美容,搞笑幽默,旅游
时尚
重口味,旅游,美容服饰
美食

汽车
投资理财,IT 数码,美食,健康
旅游,幽默,时尚,读书,运动
时尚
新闻资讯,健康,旅游,投资理财
心理学,体育,天蝎座
旅游,健康,时尚,音乐,跑步,K 歌,看书
旅游
娱乐,唱歌,艺术,运动,好性格,幽默
汽车
旅游
体育
减肥,帅哥,美容,旅游,美食,游戏动漫,娱乐
游戏动漫
新闻资讯
游戏动漫
文艺,娱乐,教育就业,辛勤耕耘的老 HR
新闻资讯
文艺,体育,娱乐
娱乐
星座命理
旅游,宅女,娱乐,时尚
开心的工作,快乐的生活!
善良,传媒,it 互联网,A 型血,健康,时尚,文艺,美女,新闻资讯
又是年底,又被做特了,懂的入
美女,游戏动漫,时尚
IT 数码
旅行,音乐,电影
游戏动漫
旅游美食,Hepburn,宅,90 后,动漫,巨蟹座,听歌
美食
健康
娱乐
IT 数码,娱乐,时尚,旅游,汽车,健康,美食
自由派,毛毛
美食
健康,体育,娱乐
喜欢音乐,投资理财,文学,美食,健康,旅游
搞笑幽默,文学,读书,运动,唱歌,游戏,摄影,旅行,大学生,90 后
IT 数码,娱乐,汽车,军事,体育,小清新
兼职,电子商务,80,旅游,在家创业,网上购物,创业,汽车
发誓好好爱自己
广东生活
新闻资讯,时尚,美食,旅游,健康,体育,娱乐

搞笑幽默
理财,水瓶座
房地产
小女人,小说
偶稀饭睡觉觉
IT 数码
健康,汽车,军事,IT 数码
投资理财,旅游
健康,文艺,旅游,时尚,美食,80 后
名人明星
IT 数码
体育,时尚,美食,健康,娱乐,旅游
游戏动漫
游戏动漫,军事,新闻资讯,美女
旅游,娱乐
娱乐,汽车,星座命理
时尚,文艺,美食,娱乐
宠物图集
文艺,体育,娱乐
时尚,教育就业,健康,娱乐,美食
投资理财,文艺,美女,旅游,娱乐
喜欢听歌,新闻,听歌
吸血鬼,IT 数码
汽车,新闻资讯,文艺,学生,electric,game,音乐,book,电影,computer
视频音乐,美女
健康,新闻资讯,体育
健康,美食,新闻资讯,投资理财,教育就业
摄影
娱乐
旅游
微博奇葩,搞笑幽默
电影迷,射手,美剧迷,汽车
汽车,旅游
美女,游戏动漫
宅,loveChris,欧美小清新音乐,艺考生,碉堡的创意,懒
文艺,旅游
体育
体育
IT 互联网,计算机,盗墓笔记,跑跑卡丁车,健康
IT 数码,投资理财,娱乐,时尚,美食
睡觉觉,网球,听歌发呆,爬山,简单,娱乐,美食
娱乐,美女,IT 数码
健康,体育,娱乐
投资理财,旅游,美食,IT 数码,文艺

旅游,军事,IT 数码
娱乐,IT 数码,美女
听音乐
军事,新闻资讯
美女,美食,星座命理
健康,体育,娱乐
搞笑幽默
美食,动漫
穿衣美容
娱乐
股票,英伦风,墨镜控,zippo 控,果粉,天蝎座,吃,交友
美女,军事,娱乐,文艺,体育
写字,潮人,傻,天蝎座,运动,漫画,偶稀饭睡觉觉,喜欢听歌,星座
乐观,吃货,美食,偶稀饭睡觉觉,巨蟹座,善良,时尚,浪漫,90 后非脑残
汽车,文艺
我爱画画
无语
星座命理
教育就业,投资理财,旅游,新闻资讯,汽车,IT 数码
健康
美女
时尚,幽默
新闻资讯,军事,文艺
爱音乐,听音乐,媒体,博客,淘宝,购物,美食,健康
旅游
情感生活,美食,大学生,恋上双子座,自由,90 後,美小妞,巨蟹座,90 后,吃货
美食
美食
时尚
周杰伦,80 後,闷骚,天枰座,猫咪控,爱猫,美图摄影,视频音乐,美女
宅女,听歌,好性格,唱歌,小说,上网,自由,音乐,80 后
北京生活
体育
新闻资讯,美女,IT 数码
北京生活
新闻资讯,游戏动漫,旅游,文艺,娱乐
娱乐,体育,汽车
视频音乐,美图摄影,美食,南京生活,读书分享
美女,微博奇葩,新闻趣事,搞笑幽默,嘉兴生活
新闻趣事,游戏动漫
游戏动漫,体育
旅行,音乐
体育
星座命理

安静,忧伤,幸福
新闻资讯,IT 数码,美女,时尚,投资理财
江苏生活,数码
时尚,新闻资讯,中医,青春,动画
偶喜欢玩,偶稀饭睡觉觉
军事
美国,美食,文艺,体育
健康,时尚,旅游,文艺,星座命理
娱乐
军事,IT 数码,汽车
旅游,时尚,读书,运动
旅游,时尚,读书,运动
旅游,健康,新闻资讯,投资理财
旅游
吸血鬼,足球迷,香港生活,教师,死神,欧美,古典艺术,视频音乐,新闻趣事
名人明星
新闻资讯,娱乐,体育,k 歌,街舞,大学生
名人明星,新闻趣事,视频音乐,八卦杂谈,搞笑幽默
游戏动漫,美女,娱乐
投资理财,时尚,美食,教育就业,健康,文艺
80 后
动漫
美女
广东生活,体育
美食,娱乐
时尚,投资理财,IT 数码,星座命理,娱乐
时尚,旅游,美女,汽车
教育就业
视频音乐,美食,萌宠,穿衣美容
娱乐,听歌,旅游,美食,90 后
旅游,游戏动漫
体育,旅游,健康,文艺,IT 数码
美女
星座命理,IT 数码,美食,娱乐,健康,K 歌
萌宠,娱乐
旅游
篮球,听歌,电影,大学生,摄影,摄影爱好者,娱乐,羽毛球,时尚,旅行
文艺
摩羯座,艺术,旅游,80 后,音乐
娱乐,时尚,健康,旅游,美食
宠物,美食,穿衣美容,萌宠
旅游,健康,IT 数码,娱乐,体育
教育就业,游戏动漫
教育就业,汽车,时尚,游戏动漫

健康,旅游,游戏动漫,IT 数码
校园生活,搞笑幽默,美女,新闻资讯,娱乐,美食,旅游,军事
搜索引擎营销,网络营销推广,SEO 研究
娱乐,90 后宅女,李宇春,安静但有点疯狂,新闻,心细,执着
娱乐
搞笑幽默
体育
娱乐
健康,新闻资讯,体育
魏晨,学生一族,紫色
美女,穿衣美容,星座命理,搞笑幽默,新疆生活
美女,游戏动漫
体育
美女,美食,微博奇葩,旅游,新闻趣事
IT,篮球爱好者
娱乐
广东生活
体育
IT 数码
教育就业,文艺,投资理财,汽车
时尚,娱乐,美食,星座命理
旅行,钓鱼爱好者,金牛座,动漫
美食,IT 数码,娱乐
葡家二弟控,葡萄控,波点控,条纹控,格子控,蝴蝶结控,蕾丝控,碎花控,苹果控,微博控
时尚,旅游,娱乐,处女座,新闻,传媒,紫色,乐观
娱乐
时尚,娱乐,星座命理,文艺
健康
80 后
视频音乐
美女,美食,娱乐,体育
双子座
游戏动漫,IT 数码
江门生活
美女,投资理财,旅游,军事
体育,娱乐
美女,汽车,IT 数码,美食,体育,科幻,旅游爱好者,影视,打篮球,爱吃
娱乐
时尚
游戏动漫
健康
健康,娱乐
星座命理,好书,亲情,北漂,70 后,天秤座
美女,教育就业,军事,IT 数码,体育

娱乐
美女,IT 数码,文艺
新闻资讯
古风控,瓶邪,盗墓笔记,剑网三,懒,名人明星
A 型血,工作,房产,自然,坚强,怀旧,奋斗
美食
星座命理,时尚,文艺,娱乐
娱乐,任性,坚强,手机,善良,处女座,看书,音乐,听歌
健康
新闻资讯,旅游,军事,文艺,教育就业
男人,自信,睡觉睡到自然醒,安静,爱幻想,笑,IT 数码
台球
时尚,星座命理,旅游,文艺
美女,汽车,旅游,美食,体育
娱乐,王乃恩,希澈,韩庚,圭贤,厉旭,SJ,腐女,bl
投资理财,文艺,健康,教育就业,军事
健康,娱乐
恐怖片,美食
文艺,星座命理,娱乐,体育
娱乐,体育,木有标签
文艺,旅游,美女,娱乐,教育就业,美食,体育
睡懒觉,足球,音乐
旅游,北京生活,搞笑幽默,新闻趣事,微博奇葩,穿衣美容,视频音乐
自由,旅游,音乐,运动,搞笑,体育,天秤座,90 后,游戏,NBA
汽车,IT 数码,时尚,星座命理,健康
健康,时尚,教育就业,投资理财
体育
体育
电子商务,精油,芳疗,养生,护肤,时尚,上海生活,星座命理,美食,穿衣美容
娱乐,健康,魔羯座,乒乓球,外貌协会,苹果控,宅女加腐女,90 後,爱电影,周杰伦
时尚,汽车,体育,娱乐,IT 数码
娱乐
神经大条,囧,吃货
矛盾体
时尚,双鱼女,我就是我,北京,爱旅游,欧美
游戏动漫
名人明星,体育资讯
体育,游戏动漫
投资理财,健康,旅游,教育就业,娱乐
旅游
娱乐
体育
美女,教育就业,美食
旅游,时尚,美食,文艺,娱乐,健康

旅游,娱乐,文艺,IT 数码,体育
美女,投资理财,旅游,IT 数码,星座命理,美食,军事,文艺,体育,娱乐
上海生活
娱乐,美食,健康,美女,文艺
穿衣美容
活泼,旅游美食,爱生活,紫色,自由职业,看电视,阅读
星座命理
旅游
娱乐
美女
宠物图集,美食,星座运势,动漫
DIVINE 乐队,磨宾林,漫画,吉他,特鲁姆普,音乐,斯诺克,体育,文艺
文艺
80 后,旅游,听歌,音乐
体育,军事,投资理财,IT 数码
新闻趣事
健康,汽车,新闻,旅游,狮子座,摄影,搞笑,爱情,电影,80 后
山东生活,美女,投资理财,军事,汽车
旅游,星座命理
娱乐
健康,旅游,美食
美食,投资理财
健康,星座命理,IT 数码
花样美男,美女,时尚,文艺
美食,新闻资讯,体育,IT 数码
汽车,美食,娱乐,星座命理,游戏动漫,高达迷,动漫迷,水瓶座,音乐控,笑话
搞笑幽默
健康,旅游
北京生活
旅游,娱乐
文艺
汽车,IT 数码,体育
旅游,健康
健康,时尚,汽车,美女,娱乐
旅游,时尚,娱乐,文艺
旅游,IT 数码,文艺
时尚,旅游
旅游,汽车,美食,星座命理,娱乐,偶稀饭睡觉觉,水瓶座
娱乐,游戏动漫
旅游,宅,听歌,90 后
汽车,星座命理,游戏动漫
娱乐
娱乐
美女,健康

娱乐
文艺,金牛座
邦明诚一
美食,旅游,美女,汽车,娱乐
时尚
新闻趣事
搞笑幽默
汽车,旅游
美食,时尚,旅游,IT 数码,星座命理
军事
健康,游戏动漫,IT 数码,军事
旅游
汽车,体育
娱乐
星座命理,健康,时尚,文艺
文艺,时尚,娱乐,健康,旅游
军事,IT 数码,文艺,体育,娱乐,时尚
汽车
娱乐,时尚,文艺,新闻资讯,投资理财
美女
娱乐,文艺,教育就业
美女,游戏动漫,汽车
文艺
教育就业,健康,旅游,星座命理,娱乐,汽车,美女
新闻资讯,投资理财,体育
文艺,旅游,时尚,教育就业,投资理财
微博奇葩,搞笑幽默
幻想,聊天,时尚,星座命理,美食,文艺,娱乐
搞笑幽默,微博奇葩
娱乐,旅游,美食,军事,文艺
新闻资讯,美女,教育就业
旅游,健康,美女,时尚,娱乐
游戏动漫
搞笑幽默
娱乐
美食,娱乐,游戏动漫
宅女
旅游
睡觉,完美主义,陈奕迅,唱 k,爱旅游,爱电影,低调,90 後
星座命理
旅游,搞笑幽默,名人明星,视频音乐,微博奇葩
涂鸦,爱美食,插画,巨蟹座,文艺
汽车,时尚,旅游,美食,娱乐
体育,文艺,星座命理,时尚,健康

娱乐
时尚,IT 数码,新闻资讯,旅游,文艺,游戏动漫,星座命理,军事
娱乐
娱乐,时尚,旅游
美女,视频音乐,美食,旅游,穿衣美容
娱乐
娱乐,游戏动漫,美女
我爱 UC 浏览器
游戏动漫,旅游,美食,军事,IT 数码
娱乐
游戏动漫,娱乐,旅游,IT 数码,美食
文艺,旅游,星座命理,美食,娱乐
游戏动漫
娱乐
汽车
爱健康,娱乐
旅游
找,Emily 的小世界
时尚,旅游,星座命理,美食,娱乐
美女
坐等数快乐
游戏动漫,新闻资讯,投资理财,军事,文艺,IT 数码,娱乐
旅游
新闻资讯,美女,游戏动漫,文艺
美女,汽车,时尚,体育,娱乐
星座命理,健康,时尚,美食,文艺
天蝎座,唱歌,80 后
旅游,健康
IT 数码,处女座,网络,旅游,睡觉
视频音乐
体育,市场营销
电影,网购
娱乐,文艺,美食
军事
教育就业
时尚,新闻资讯,旅游,美食,文艺,体育
爱旅行,爱拼搏,无语强迫症,致命刀疤淇,爱自由,爱生活
美女
茧镇奇缘,爱情闯进门,宋茜
健康,时尚
IT 数码
微博奇葩
美食,文艺,游戏动漫,时尚,娱乐
紫色,阅读,双子座,动漫,听歌,旅游,旅行,80 后,电影,音乐

娱乐
教育就业,健康
健康
美食
穿衣美容
美食,旅游,时尚,健康,娱乐,新闻资讯
广东生活,星座命理,美女,旅游
帽子控,帆布控,柯迷
娱乐,文艺,军事,旅游,星座命理
娱乐,星座命理,健康,投资理财,时尚
汽车,投资理财,健康,旅游,美食
旅游,新闻资讯,时尚,教育就业,投资理财
IT 数码
军事,时尚,美女,教育就业,投资理财,旅游,健康
时尚
娱乐
林峰,汽车,教育就业,新闻资讯,健康,听歌,90 后
時尚
星座命理,体育
独立坚强,简单,养生,美食,文艺,投资理财
星座命理,健康,美食,娱乐
游戏动漫
娱乐,IT 数码,新闻资讯,文艺,教育就业,时尚
时尚,娱乐,旅游,IT 数码,汽车
汽车,时尚,游戏动漫,军事,娱乐
美食
娱乐
健康,美食,听歌,知录妈咪,孕产妇营养,营养与健康,营养师,营养医学,养生保健,电子商务
时尚
旅游,健康,娱乐,投资理财
军事,新闻资讯,健康,旅游,体育,时尚,星座命理
健康,游戏动漫
美食
互粉,爆料,有奖转发,活动,美食,嗅探,新闻,揭露,曝光
游戏动漫,动漫,电影,游戏
军事,文艺,体育,娱乐,IT 数码
电影,音乐,旅游
文艺,体育,娱乐
娱乐,IT 数码
旅游
健康,旅游,投资理财
新闻资讯,时尚,投资理财,汽车,IT 数码,体育,80 後
时尚,文艺
体育,IT 数码

文艺,90 后,学生
IT 数码
财经,汽车,90 后,吃,巨蟹座,搞笑,八卦,偶喜欢玩,小说,英语
心理,热情,张杰
文字,英语,爱笑,玩,潮流,搞笑,吃货,fashion,猫控,娱乐
娱乐,旅游,旅行,善良
90 后
游戏动漫
汽车,美女,旅游,体育
美女,健康,娱乐,旅游,时尚
亲情,偶喜欢玩
时尚,教育就业,健康,星座命理,文艺
美女,旅游,IT 数码,时尚
美女,旅游,军事,文艺,体育
八卦杂谈
天涯歌女,古典艺术,容中尔甲,暮光之城,迷恋爱剧场,娼妇凯蒂
游戏动漫
娱乐,文艺
星座命理,美女,游戏动漫,娱乐,时尚
视频音乐,名人明星
宠物图集,情感生活,星座运势,美图摄影,读书分享
美食,旅游,新闻资讯,投资理财,文艺
军事,体育
自由
视频音乐
体育
教育就业,健康,时尚
美食,投资理财,美食,健康,旅游,IT 数码,军事,新闻资讯
时尚,美食,新闻资讯,旅游,游戏动漫
新闻资讯
上网
游戏动漫,文艺,娱乐,时尚,IT 数码
山东生活
汽车,投资理财,旅游,军事,体育,自由,旅行,80 后
汽车,星座命理,IT 数码
美食,新闻资讯,星座命理,文艺,投资理财
音乐,艺术,摇滚乐,娱乐,美女,军事,时尚,文艺
创业,篮球
文艺
時尚,星座命理,美食
娱乐,体育,美食
文艺,时尚,旅游,音乐,电影
时尚,投资理财,旅游,星座命理,美食
穿衣美容

娱乐,体育
美食
星座命理,旅游
亲情,猫咪控,桌球,IT 数码
基督徒,圣经,爱耶稣,摄影
视频音乐
旅游,美食
健康,娱乐
娱乐,旅游,体育
美食,穿衣美容
体育,娱乐,投资理财,美食,文艺
IT 数码
星座命理,美食,娱乐
旅游,体育,汽车
名人明星,穿衣美容,美女,游戏动漫,文艺,娱乐
交朋友,爱笑,爱旅游,80 后的尾巴,文艺
天蝎座,小说
潮州生活
时尚
广东生活,美女
娱乐,旅游,美食,汽车,体育
安静
亨利,小贝,佩德罗,哈维,小罗,米兰,巴萨,IT 数码,Juniper,CISCO
萌宠,腐,宅到死,動漫,天蝎座
巴萨,考研,营销,英语,留学生,教育就业,旅游,IT 数码
江苏生活,穿衣美容
视频音乐,坚强独立,五月天,苏打绿,安静,音樂,爱旅行,流浪,笨蛋,健康
穿衣美容
游戏动漫,美食
新闻资讯,游戏动漫,汽车,体育,娱乐
苏州生活,文艺
美女
美食,大学生,微笑,上网
美食,軍事,遊戲動漫,時尚,IT 數碼
健康,文艺,星座命理
美食
汽车,娱乐,军事,IT 数码,体育
摄影控,旅游
娱乐
娱乐
娱乐
IT 数码
娱乐
乐观向上

视频音乐,汽车,新闻趣事,游戏动漫,搞笑幽默,娱乐
情绪化,咖啡,努力,布丁,养生,90 後,音乐迷,厦门,外贸,语录
IT,新闻资讯
娱乐
宅男,善良,巨蟹座,羽毛球,吃
游戏动漫,体育,文艺,时尚,美食,健康,星座命理
电影爱好者,宠物,管理,吃喝玩乐,美食,书,喜欢音乐,看电视,钓鱼,美剧控
娱乐
IT 数码
汽车
美女,汽车,娱乐,IT 数码,体育
文艺,游戏动漫
足球迷,时尚,汽车,军事,美食
美女,NBA,自由
娱乐
军事,美女,健康,投资理财,娱乐
游戏动漫,美女,健康,新闻资讯,IT 数码
汽车
美女,体育
娱乐,文艺,旅游
爱旅游,宅女
逛街,懒
投资理财
一些事一些情,财经,低调,无聊,时装
娱乐
游戏动漫
旅游,文艺,游戏动漫,体育
萌宠,微博奇葩,星座命理,穿衣美容
80 后,电影,旅游,自由,旅行,听歌,美食,音乐
旅游,健康,美女,新闻资讯,教育就业
偶稀饭睡觉觉
汽车,美食,军事,IT 数码,文艺
搞笑,三分钟热度,傻得可爱,八卦女,爱睡觉,性格开朗,大大咧咧
80 後,重口味,旅游美食爱好者,执着,IT 数码,汽车
时尚,投资理财,IT 数码,娱乐,汽车,旅游,军事,体育
汽车,新闻资讯,时尚,文艺,自由
美女,军事,文艺,体育,娱乐
娱乐
娱乐
娱乐
IT 数码,美女,娱乐,体育,单身,爱生活
天蝎,或疯狂,浅文艺
健康,时尚,教育就业,星座命理
娱乐

娱乐
娱乐
美食,阅读,网购,艺术,天秤座,上网,旅游
旅游,军棋,美食,健康,投资理财
娱乐
娱乐
娱乐
娱乐
娱乐
旅游,好书,音乐,摄影,电影,自由,运动,学生
IT 数码,娱乐
娱乐,文艺
游戏动漫,星座命理,健康,新闻资讯,投资理财,美食,时尚
投资理财
军事,体育,娱乐
摄影渣蜕变中,黑眼圈 Queen,二次元症候群
教育就业,投资理财,健康,旅游,美食
IT 数码,小说,上网,睡觉,自由,80 后,数码,足球,乐观,游戏
苹果,电影爱好者
阿 Q
旅游,体育
IT 数码,时尚,文字控,唱 K
体育
美食
文艺,新闻资讯,军事,IT 数码,汽车
美食,美女,视频音乐,新闻趣事,微博奇葩
旅游,星座命理,健康,美女,投资理财
IT 数码
美食,娱乐
文艺
旅游爱好者,娱乐
搞笑幽默
看书,电影,TVB,数码,旅游
娱乐
时尚,健康,文艺,娱乐
90 后非脑残,吃货,健身,快乐,看电影,狗狗,爱旅行,Music,娱乐,文艺
美女,萌宠
随性
体育
文艺,时尚,旅游
遊戲動漫
七种武器,土炮,日本洋刀,老鳖叉,狼牙棒,砍刀,弓箭,打狗棍
穿衣美容,白羊座,敏感,小清新,执着,军旅迷,乐观,电影控,喜欢音乐,科幻
古色古风古香,无方少年游,腐腐腐腐腐性向,莱昂纳多,射手座,海贼王 onepiece,火影

naruto,盗墓笔记瓶邪党,日漫痴,林弯弯 snaki
设计,Nichkhun
美食,娱乐,健康
体育,娱乐
健康,新闻资讯,投资理财,娱乐,美食
美食,娱乐,旅游
时尚,旅游,游戏动漫,美食,美女
音乐达人
时尚,娱乐
星座命理
娱乐,美食
幽默,搞笑,睡觉,唱歌,网购,80 后,巨蟹座,亲情,爱情
穿衣美容,时尚,投资理财,健康
IT 数码
微博奇葩,萌宠,旅游,美女
厨师
旅游
微博奇葩,美食,旅游
时尚
健康,星座命理,旅游
旅游,吃货
教育就业
旅游,游戏动漫,文艺,娱乐
自由,宅,旅行,电影,听歌,旅游,美食,80 后
文艺,美女
军事,投资理财,汽车,IT 数码,星座命理
化妆,旅游爱好者,电子商务,财经,IT
游戏动漫
游戏动漫,文艺
海南生活
街拍,单身,军事,摄影爱好者,汽车,平常心,天秤座,生活,足球,摄影
汽车
美女
射手座,学生,体育,动漫,宅
旅游
时尚,美女,教育就业,娱乐,IT 数码
视频音乐,陕西生活
美女,健康,美食,军事
时尚,旅游,美食,文艺,娱乐
时尚,旅游,星座命理,娱乐,美食
名人明星
星座命理
游戏动漫
gay,同志电影,帅哥,同志

上海生活
美女,IT 数码,健康
旅游
体育
文艺,旅游,时尚,新闻资讯,娱乐
文艺,教育就业,新闻资讯
星座命理,微博奇葩,新闻趣事,搞笑幽默
八卦,射手座,艺术,大学生,听歌,网购,校园,娱乐,美食
健康
gay,文艺,钟汉良,快乐大本营,南非
娱乐
娱乐,微博,宅
chemistry
旅游
娱乐,教育就业,投资理财,文艺,IT 数码
汽车,旅游,美食,娱乐,星座命理
美食,旅游,时尚,汽车,娱乐
健康,旅游,星座命理,军事,IT 数码,体育
新闻资讯
新闻资讯,IT 数码,娱乐,健康
美食,时尚
小说,听歌,上网,90 后,睡觉,学生,美食,狮子座,音乐,动漫
娱乐,投资理财,教育就业,文艺,旅游
文艺
名人明星
星座命理,IT 数码
游戏动漫,时尚,星座命理,美女,乐观,微博,幽默
名人明星,美食,旅游,微博奇葩
美食,新闻趣事,IT 数码,长春生活,搞笑幽默,金牛座,数码
文艺,体育,娱乐
旅游,娱乐,军事
时尚,旅游,美食,娱乐
娱乐
体育
教育就业
旅游,汽车
星座命理,美食
美食,旅游,星座命理,美女,时尚,文艺,娱乐
健康,体育,娱乐
IT 数码,美女
军事,体育
IT 数码,体育,美食,健康
IT 数码,时尚,星座命理,文艺,娱乐
时尚,投资理财

教育就业,军事,美女,新闻资讯,投资理财
健康
时尚,星座命理,娱乐,旅游,教育就业,投资理财
美女,游戏动漫,美食,军事,体育
汽车,旅游,体育
美食,旅游,时尚
健康,旅游,体育,IT 数码
减肥,美容,彩妆,睡觉,游乐场,旅游
篮球
美食,星座运势,IT 数码,北京生活,是诸佛教,自净其意,众善奉行,诸恶莫作
娱乐
游戏动漫,汽车,旅游,美食,美女,体育,爱拜仁！爱足球！
星座命理,时尚,旅游,文艺,娱乐
陕西生活
穿衣美容
投资理财
文艺,微博,文学,打篮球,钢琴
时尚,游戏动漫,美女
天津夜场网,南京夜场网,太原夜场网,深圳夜场网,上海夜场网,北京夜场网,宁波夜场网,杭州夜场网,温州夜场网,福州夜场网
军事发烧友,科技创新,数码控,汽车爱好者,旅游美食
文艺,美食,美女
上海生活,宅女加腐女,睡觉,穿衣美容,爱,喜欢旅游,美容
IT 数码
星座命理,旅游,时尚,文艺,美食
美图摄影
环保,足球,美食,军事
微博奇葩
美食,娱乐,健康,旅游
萌宠,摩羯座,80 后
教育就业,娱乐
美食,军事,健康,旅游,投资理财,钓鱼爱好者,拉布拉多控,巨蟹座
萌宠,90 后但我不脑残,射手女,柯南
汽车,文艺,美女,IT 数码,娱乐
美食,文艺
名人明星,萌宠,微博奇葩,搞笑幽默
娱乐,体育
旅遊,汽車,時尚,文藝,娛樂
文艺
汽车,时尚,旅游,投资理财,体育
穿衣美容
手机软件开发,学科竞赛,软件代理销售,教育信息化,纺织服装设计
搞笑幽默,大地,蓝天,篮球
美图摄影

IT 数码,游戏动漫,汽车
睡懒觉,K 歌,搞怪,微笑,吃,跳舞,周杰伦,爱电影,低调,夜猫子
新闻资讯,投资理财,教育就业,旅游,美女,文艺,IT 数码
购物狂
深海恐惧症,密集恐惧症
吃货,手机党,懒虫一大条,外冷内热,狮子座,好性格
看电视,音乐控,旅游,文艺,美食,娱乐
娱乐
美食
美食,新闻资讯,健康,时尚,IT 数码
美女,名人明星,穿衣美容,微博奇葩,新闻趣事
美食,萌宠,微博奇葩
时尚
健康,教育就业,体育,文艺,军事,书法,喜欢音乐,好书,旅游美食
旅游摄影,读书,旅游美食爱好者,欧美,天蝎
健康,教育就业,文艺,美女
娱乐
体育资讯,八卦杂谈,视频音乐,美女,搞笑幽默
美食,游戏动漫,娱乐
健康,体育,娱乐
爱紫色,85 后,矛盾体,爱生活,神经大条,宅
旅游,投资理财,文艺
双子女,偶稀饭睡觉觉,无厘头,任性,偶喜欢玩,唱歌,时尚,爱情,美食,音乐
睡觉睡到自然醒,购物狂,吸血鬼日记,购物,花痴,水瓶女,书,乐天派,玩儿,梦想
玩,微笑,喜欢听歌,潮人,夜猫子,跳舞,爱电影,红酒,培训,DJ
旅行,睡觉,宅,学生,体育
健康,娱乐
旅游,投资理财,娱乐,美食,时尚
臭美,神经大条,强迫症
旅游
个性,IT 数码
自由职业,喜欢听歌,爱玩,新闻资讯,娱乐,美女,美食,星座命理
IT 数码
星座命理
军事,新闻资讯,汽车
美女,苏州生活,旅游
健康,美食,娱乐
新闻资讯,旅游,IT 数码,时尚,投资理财
游戏动漫,旅游,美食,文艺,娱乐
美食,游戏动漫
娱乐
旅游
微博奇葩,美女,视频音乐,旅游,搞笑幽默,懒人
娱乐

游戏动漫,IT 数码
假斯文
体育资讯,听歌,电影,音乐
游戏动漫
旅游
汽车,旅游,军事,文艺,体育,大学生
教育就业
IT 数码,星座命理,美食,军事,健康,鏇呮父,文艺,体育,娱乐
健康,教育就业,文艺,美食
星座命理,娱乐
教育就业
娱乐
旅游,娱乐,美食
旅游
娱乐,美女,开朗,活泼,热情,运动
旅游
娱乐,健康,旅游,文艺
旅游,星座命理,美食,娱乐,文艺
游戏动漫,时尚,旅游,美食,文艺
军事
我爱 UC 浏览器
乐观,偶喜欢玩,玩儿,小说,爱情
美食
网店,宅,金贤重,旅游,美食,听歌,电影,音乐
娱乐
健康,娱乐,美食
时尚
河南大学生,80 后,大学,文化传媒,豫文化,河南文化,郑州,河南
汽车
游戏动漫
美食,体育,娱乐
健康,美女,教育就业,投资理财,新闻资讯
旅游
时尚,游戏动漫,旅游,文艺,美食
健康,娱乐,美食,旅游,星座命理,时尚
随性,学生一族,娱乐,旅游,新闻资讯,时尚
三次元滚蛋,星座控,青黄一生推,TVB,吃货,巨蟹 A
健康,美食,教育就业,投资理财,旅游,新闻资讯
美女
娱乐,美女,IT 数码,文艺
星座命理
IT 数码,美食
新闻资讯,健康,汽车,投资理财
文艺

旅游,投资理财,教育就业,汽车,美食
娱乐,军事,美食,时尚,汽车,IT 数码
K 歌,摄影控,旅游摄影,美食爱好者
石家庄,武警,暗黑 3,游戏动漫,汽车,军事,IT 数码
汽车
新闻资讯,汽车,文艺,旅游,星座命理
新闻资讯
娱乐
健康,美食
新闻资讯
投资理财,健康,美女,旅游,美食,射手座,乐观,搞笑
娱乐,体育,汽车,IT 数码,文艺,美食
投资理财,旅游
旅游,星座命理,军事,体育,文艺
90 后,学生
文艺,游戏动漫,美食
微博奇葩
美女,體育,IT 數碼
教育就业,美食,旅游,体育,娱乐
游戏动漫,新闻资讯,文艺,汽车,星座命理,IT 数码,娱乐
新闻资讯
爱美食,养生,热爱生活,发呆,好书,星座命理,媒体
星座命理,教育就业,投资理财,时尚,游戏动漫,IT 数码,文艺,娱乐
搞笑幽默,微博奇葩,美食,穿衣美容,名人明星,娱乐
旅游
军事
美女
时尚,汽车,文艺,IT 数码,美食
健康,汽车,投资理财
旅游,星座命理
要高考,娛樂,可爱,娱乐,水瓶座,小说,美食,自由,学生,90 后
美食,投资理财,汽车,健康
美女,赚钱才是硬道理,时尚,爱自由,常州,钓鱼,玩儿,设计,美食,摄影
游戏动漫,投资理财,时尚,旅游,IT 数码,体育
音乐
娱乐
旅游,美食,文艺,娱乐,健康
旅游,IT 数码
热爱生活,美食爱好者,敏感,开心,爱,诗歌,喜欢音乐,销售,财经,善良
杨幂
唱歌,听歌,美女,游戏,好性格,大学,90 后,玩儿,校园,交友
时尚
新闻趣事
娱乐,游戏动漫,军事,IT 数码,文艺

文艺,体育,军事
拍照,爬山,影视,K 歌,欧美,时装,音乐达人,篮球爱好者,时尚达人,旅游
健康,旅游
文艺,时尚,IT 数码,汽车,娱乐,体育
音乐
汽车,IT 数码,体育,娱乐,游戏动漫
听歌,唱歌
投资理财,星座命理,时尚,美食
教育就业,美食,汽车,旅游,娱乐
军事
美食
文艺
旅游,汽车,美食,娱乐
新闻资讯,健康,旅游,美食,娱乐
健康,冰山雪农,西域干果折扣店,皇冠店铺,淘宝店铺
搞笑幽默
军事,IT 数码
80 后,营销,医药,保健
美食,健康,体育,篮球
旅游美食爱好者,摄影,睡觉,篮球,体育
新闻资讯
文艺,IT 数码,k 歌
时尚,星座命理,文艺,电影,天蝎座,睡觉,宅女,旅游,读书,听歌
美食
IT 数码
美食,文艺,娱乐
爱自由,天秤座,听歌,宅男,爱幻想,奋斗,时评
娱乐
名人明星,微博奇葩,旅游,美女,娱乐
徐浩,好性格,射手座
汽车
美食
娱乐
游戏动漫
美食,投资理财,时尚
娱乐,游戏动漫
健康,时尚,旅游,美食,IT 数码,娱乐
浪漫,金牛座,亲情,电影,网购,善良,乐观,动漫,生活,时尚
投资理财,新闻资讯,军事,旅游,体育
江苏生活
名人明星,搞笑幽默,视频音乐,曲靖生活,新闻趣事
旅游,星座命理
娱乐,汽车,投资理财,军事,IT 数码
健康,星座命理,美食,文艺,娱乐

喜欢听歌,爱笑,阳光,旅游美食爱好者,快乐
体育
Super,Junior
文艺,娱乐
旅游,投资理财,生活
IT 数码
游戏动漫
健康,星座命理,旅游,美食,娱乐
文艺,尚道,汽车,改装,车身改色,透镜,刹车,避震,进气,排气
健康,幽默,微博,新闻,创业,旅行,平顶山,医学美容
其他生活
hifi,耳机,C5,宝华,b,w
文艺,狮子座
娱乐,美食,游戏动漫
美食,投资理财,军事,美女,体育
减肥,爱睡觉,学生一枚,模特,猫,美食控,懒
体育
职场招聘
服装搭配,小牧女,colorrun,摄影
上海生活,游戏动漫
旅游
娱乐
文艺,旅游,星座命理,美食
搞笑幽默
文艺,旅游,健康,美食,时尚
健康,文艺,时尚,美食,娱乐,投资理财
IT 数码
投资理财,美女,新闻资讯,军事,文艺
投资理财,娱乐,美食,IT 数码,美女
新闻趣事
美食,旅游,娱乐,文艺,体育
IT 数码,文艺,娱乐,游戏动漫,体育
星座命理
帅哥,美容,开朗,逛街,美食
互联网,瑜伽,80 后的尾巴,星座控,人生,孩子气,tvxq
IT 数码,娱乐,旅游,美食,文艺,星座命理,时尚
名人明星,学生一枚
美食
投资理财,游戏动漫,旅游,IT 数码,文艺
文艺
平顶山生活
新闻资讯,军事,教育就业,健康
娱乐,搬厂,清洁清洗,搬家
投资理财,新闻资讯,文艺,IT 数码,旅游

游戏动漫
娱乐,美食
书,音樂,星座命理,吴世勋,sehun,exo
新闻资讯,旅游,军事,IT 数码,投资理财
时尚
文艺
视频音乐,美食,美图摄影
新闻趣事,上海生活
旅游,教育就业,娱乐,健康,时尚
bigbang,神经大条,生活大爆炸,汽車,旅游
娱乐,汽车
军事,新闻资讯,投资理财,体育,游戏动漫
搞笑幽默
cos,悬疑推理,仙剑,古风控,动漫,野生 CV,声控,盗墓
娱乐,美食,星座命理
汽车,文艺,体育
美女,玩游戏逗小姑娘
文艺
搞笑幽默,美女
星座命理,IT 数码,娱乐,体育,汽车
娱乐,文艺
微博奇葩,新闻趣事,搞笑幽默,星座命理
投资理财,娱乐,旅游
旅游,健康,新闻资讯,教育就业
美食,军事,IT 数码,美女
减肥 ing 宅女,外貌协会会长,爱狗一族,疯子,汽车,旅游,时尚
旅遊,赚钱,美女,游戏动漫,旅游,汽车,IT 数码
囧,神经质,美食,听歌,狮子座,偶稀饭睡觉觉,学生,90 后,音乐
教育就业,健康,时尚,文艺
冷笑话,文字,体育
娱乐
坚强,吸血鬼日记,意大利,康熙来了,美食,新闻资讯,星座命理,健康,旅游
旅游
游戏动漫
美食,新闻资讯
摩羯座,搞笑
上海生活
桌球迷,篮球,唱歌,游泳,射手座,90 后
健康,娱乐,星座命理,文艺,体育
教育就业,文艺,IT 数码,美女,投资理财
体育
娱乐,美食
旅游,VIP
娱乐,游戏动漫,星座命理,IT 数码

文艺,投资理财,星座命理,美食
太普通啦,人力资源,感性,广州,85 后,我就是我,o 型血,敏感,平常心,天秤座
星座运势
视频音乐
文艺,HeeChul,巨蟹座,旅游,80 后
旅行,电影,听歌,美食,音乐,80 后
旅游,健康,美食,时尚
投资理财,IT 数码,军事,星座命理,新闻资讯
游戏动漫,IT 数码,户外,完美主义,足球迷,90 后但我不脑残,睡觉睡到自然醒,热爱生活,玩儿,电影爱好者
连接服务器失败,互粉,90 後
星座命理
星座命理
游戏,广州,爱美食,唱歌,娱乐,摄影,户外,电影
萌宠
美图摄影
文艺
动漫
新闻趣事
IT 数码,文艺
军事,娱乐,新闻资讯,美女,体育
IT 数码
娱乐
娱乐
娱乐,游戏动漫
美女
教育就业
搞笑幽默
娱乐
体育,汽车,游戏动漫,IT 数码
新闻趣事,处女座
旅游,汽车,IT 数码,娱乐,体育
体育,汽车
投资理财,游戏动漫,时尚,美女,新闻资讯,健康
发呆,听音乐,游戏动漫,旅游,美食,娱乐
时尚潮流,深圳,休闲,室内设计,旅游,音乐
娱乐,体育
高中生,音乐，发呆
游戏动漫,IT 数码
电影,旅游,美食,音乐
教育就业,投资理财,旅游,健康,星座命理,美食
梅西,岩井俊二,起床困难户,DOTA
健康,军事,文艺,新闻资讯,美女,投资理财
美食

美食,旅游,娱乐,时尚
体育
设计,汽车,摄影,电影,文学,音乐,手机,新闻,爱情,双鱼座
大学,吃货,自由,学生,90后,电影,听歌
娱乐,陈翔控
投资理财
美女
汽车,时尚,星座命理,美食,旅游
星座运势,读书分享,娱乐
旅游
旅游
健康,时尚
娱乐
IT数码,旅行,电影,90后,听歌,音乐
星座命理,娱乐,IT数码,时尚,美女,二货
重口味,视频音乐,校园生活,美女,搞笑幽默
腐女一枚,蓝家女人,爱玩爱笑爱热闹,死忠SUJU,赫海真爱,篮球本命,SUPERJUNIOR
八卦,交友,美女,美剧,90后
美女
文艺,旅行,IT
星座命理
美食,星座命理,IT数码,天蝎座
娱乐
成熟,淡定
旅游
IT数码
萌宠,起床困难户,泡不好咖啡,苦逼学生党,电影迷,宅女一枚
娱乐,汽车,健康,时尚,体育
爱音乐,努力,高三,音乐发烧友,商业,IT数码,新闻资讯,投资理财,教育就业,体育
教育就业
游戏动漫
星座命理
搞笑幽默
旅行,自由,电影,听歌,音乐
可爱,阳光,少女时代,爱幻想,90後,强迫症,音乐控,fashion,神经大条,玩儿
军事,IT数码,文艺
娱乐
安阳生活,学生一族
穿衣美容,美女,河南生活,名人明星,视频音乐
娱乐,体育,文艺
美食
搞笑幽默
搞笑幽默
美女,游戏动漫,汽车,娱乐

美食,时尚,美女,星座命理,娱乐
IT 数码,旅游,星座命理,汽车,健康
娱乐
音樂,健康,时尚,美女,美食,娱乐
宅,自由,学生,90 后,腐女,花痴外向
健康,美女,美食,军事,IT 数码
编辑,旅游,读书,攝影
娱乐
搞笑幽默
摇滚,旅游,体育
新闻趣事
娱乐
旅游,看书,吃,宅
娱乐
娱乐
旅游
体育
独立,旅游摄影,天蝎女,多愁善感,星座控,娱乐
游戏动漫
时尚,美食,娱乐
时尚,旅游,投资理财,美食,娱乐
星座命理,文艺,投资理财,教育就业,娱乐
文艺
星座命理
美食
海绵宝宝,偶稀饭睡觉觉,读书,旅游
投资理财,旅游,美食,文艺,娱乐
娱乐
IT 数码,美食,游戏动漫,时尚,娱乐
投资理财,军事,美食,新闻资讯,游戏动漫
幽默,营销,睡觉,学生,足球
健康
游戏动漫
体育,美食,娱乐
新闻趣事,时尚
维他命,乐天派,随性,猫咪控,喜欢听歌,时尚,健康,美女
娱乐
旅游
娱乐
旅游,娱乐,美食
星座运势,读书分享,美图摄影,美食,视频音乐
重口味,八卦杂谈,校园生活
IT 数码
游戏动漫

军事,文艺,汽车,体育,美女,事业,宅,时尚,旅游,音乐
时尚,健康,旅游,美食,娱乐
体育,教育就业,新闻资讯,投资理财,军事
时尚,健康,文艺,娱乐
视频音乐,搞笑幽默
5sing,陈翰,洛熙,娱乐
美食,IT 数码,旅游
名人明星
文艺
旅游
娱乐
迷糊,神经质,美术,偏执狂,胡思乱想,旅游
创业,吃,上网,唱歌,睡觉,自由,听歌,音乐
投资理财,时尚,IT 数码,80 后,宅,听歌,旅游
星座命理,娱乐
欧美,听音乐,淘宝,双重性格,管理,耽美,爱自由,星座控,购物狂,电影迷
娱乐
杰娜控,张杰控,张杰,学生一族,坚强
新闻资讯,汽车,游戏动漫,娱乐
IT 数码
旅游
时尚
健康,美食,新闻资讯,旅游,汽车
娱乐
星座命理,体育
喜欢音乐,80 后的尾巴,开心,美食
美食
旅游,美女,时尚,娱乐,汽车
旅游,IT 数码,汽车
美女,汽车,游戏动漫,军事,旅游
双鱼,偶像剧,大学,爱睡觉,可爱,游戏动漫,IT 数码,旅游,星座命理
摩羯座,交友,巨蟹座,囧,互联网
旅游
新闻资讯,旅游,美食,美食,宅,自由,时尚,90 后,学生
游戏,浪漫,英语,巨蟹座,上网
美食,娱乐,健康,星座命理,旅游,自由职业,射手座,乐观,睡觉,音乐
游戏,游戏动漫
搞笑幽默,美食
文艺,美食,旅游
美食
腐,懒,生活,动漫,手机,游戏,90 后,音乐,电影,自由
美女,汽车,创业,交友,篮球,上网,听歌,音乐,电影
搞笑幽默
体育

娱乐,文艺,美食,旅游,时尚
娱乐
投资理财,旅游
体育
旅游,健康,美食
读书分享,美图摄影,新闻趣事,文艺,星座命理,游戏动漫
娱乐
搞笑幽默
美食
美食,旅游,娱乐
娱乐
重口味,活泼开朗,时尚,旅行,穿衣美容,任性,古灵精怪,美食爱好者
汽车
投资理财,时尚,旅游,美食
歐美音樂
新闻资讯,亦舒,经典歌曲迷,吃货,老友记,爬山
自由,时尚,旅行,旅游,90 后,听歌,电影,身未动,心已远
美食
娱乐
美食,新闻资讯,时尚,教育就业,旅游
美食
教育就业,游戏动漫,健康
IT 数码,军事,新闻资讯,游戏动漫,文艺,体育
旅游,文艺
搞笑幽默,萌宠,F1 汉密尔顿,皇马,尤文图斯
旅游
美食,健康,投资理财,时尚,娱乐,自由,唱歌
健康,IT 数码
游戏动漫,美女,新闻资讯,汽车,体育
健康,教育就业,投资理财,军事,星座命理
文艺
娱乐,文艺,游戏动漫
ロバ丸,上田竜也,丸上,HeySayJUMP,凉知,知念侑李
娱乐
娱乐
旅游,美食
美女
穿衣美容
娱乐,90 后,听歌,美食
音乐控,爱睡觉,电影爱好者,爱猫,网购,吃货,时评,看小说,闷骚
三浦春马,前田敦子
游戏动漫,健康,美食,旅游
宅,听歌,90 后,音乐
娱乐

投资理财,教育就业,时尚,娱乐
时尚,教育就业,娱乐,美食
美食,星座命理,娱乐
星座命理,游戏动漫,体育,文艺,淳米
健康,美食,文艺,娱乐
IT 数码
游戏动漫,美女
健康,美食,IT 数码,新闻资讯,投资理财
健康,教育就业,电工技师,电气技术,中医爱好者
美女,视频音乐,搞笑幽默,娱乐
八卦杂谈,校园生活,成都生活,搞笑幽默,美女,娛樂
旅游
美食
旅游
IT 数码
时尚,美食,星座命理,宅女,天蝎座,小说,音乐控,强迫症,神经质,执着
游戏动漫,星座命理,文艺
游戏动漫,IT 数码,时尚,汽车
萌宠
新闻资讯,时尚,军事,IT 数码,投资理财
娱乐,时尚,天秤座,吃货
穿衣美容,旅游,美食
时尚,娱乐
旅游,美女,微博奇葩,美食,搞笑幽默
汽车
汽车,旅游,体育,娱乐
星座命理,健康,时尚,投资理财,汽车
美女
娱乐
不要靠近我,我是一个小刺猬,怕欺骗,怕孤单,爱幻想爱做梦,偏执冲动情绪化,严重缺乏安全感,敏感略带神经质,90 后,狮子座
文艺
旅游,时尚,娱乐,星座命理,新闻资讯
IT 数码
投资理财
美食
新闻资讯,时尚,美食,投资理财,健康
萌宠
汽车
旅游
搞笑幽默
娱乐
美食
教育就业

投资理财,旅游,美食,IT 数码,体育
登山鞋,徒步鞋,天伦天,盛威鸟,户外鞋,休闲鞋,户外服,户外包,运动鞋,运动服
IT 数码
EXOM,EXOK,EXO
安静,电脑,幻想,冷笑话,发呆,爱音乐,看电影,星座,健康
时尚,旅游,星座命理,美食,娱乐
微博奇葩
IT 数码,文艺,星座命理,游戏动漫,美女
汽车,美食,文艺,IT 数码,体育
我就是我,旅行,80 后,白羊,美食
旅游,努力
游戏动漫,星座命理,IT 数码
旅行,听歌
厦门生活
娱乐
新闻资讯
旅游,星座命理,游戏动漫,军事
新闻资讯,美女,军事,IT 数码,娱乐
娱乐
旅游
健康,旅游
旅游,电影,摩羯座,音乐
微博,狮子座,电影,80 后
旅游
娱乐
军事,美食,旅游,汽车
娱乐
美女,体育
文艺
娱乐,美食,时尚,文艺,星座命理
IT 数码
体育
羽毛球,阅读,创业,乐观,看书,动漫,自由,美食,电影,音乐
汽车,军事,文艺,娱乐,体育
交友,网络,同志,插画,电视,郑允浩,tvxq,dj,宅女加腐女,梅西
00 后,时尚,潮流,小吃货,学生,巨蟹座女孩,爱心天使,舞蹈培训,窈窕,小萝莉
搞笑幽默
动漫,篮球,军事,IT 数码,文艺,体育
娱乐
自由,时尚
体育
IT 数码
美食
睡觉

校园生活
旅游,星座命理,时尚,娱乐
自由职业,偶喜欢玩,网络,游戏,吃,看书,动漫,旅游,美食,电影
美食
旅游,星座命理,娱乐,美食
旅游,美食,美女,时尚,搞笑,吃,囧,处女座,乐观,学生
校园生活,我是大学生,喜欢听歌,看电影。
萌宠,湖南生活,美食
娱乐
旅游
旅游,健康,新闻资讯,文艺,美食
IT 数码
穿衣美容
娱乐
自由职业,处女座,美食,好性格,时尚,音乐,游戏,生活,唱歌,电影
成熟稳重哈,温文尔雅,享自由,幽默控,摄影爱好者,末路 HR
美女
新闻趣事,微博奇葩,美食,河南生活,星座命理
搞笑幽默
旅游
校园,动漫,西班牙国家队,足球,手机,处女座,好书,运动,90 后,篮球
星座命理,IT 数码,美食,时尚,美食
天秤座
IT 数码,旅游
美食,娱乐,游戏动漫
军事,投资理财
听歌,狮子座,90 后
体育,星座命理
美女,苏州生活
游戏动漫,娱乐,美食,星座命理,IT 数码
娱乐,健康
80 后,生活,好性格
投资理财,健康,汽车,娱乐,时尚
时尚
车世界,体育资讯,搞笑幽默,视频音乐,莆田生活
搞笑幽默,巨蟹女,腹黑,吃货一枚,大叔控,梁静茹,青春
IT 数码,美女
健康
娱乐,教育就业
健康,投资理财
济宁生活,电影,旅行
萌宠
旅游,娱乐,时尚,文艺,星座命理
时尚,旅游,美食,健康,娱乐

穿衣美容
搞笑幽默
健康,时尚,旅游
旅游,音乐
投资理财,汽车,健康,IT 数码,体育
新闻资讯
搞笑幽默
时尚,星座命理,健康,包包
假发制造
汽车,美女,新闻资讯,军事
投资理财,新闻资讯,娱乐
教育就业
名人明星,旅游,穿衣美容,青岛生活,美食
校园生活
游戏动漫,旅游,文艺,娱乐,美食
星座运势,名人明星,情感生活,美食,视频音乐,音乐,电影,听歌,90 后,学生
旅游,文艺,娱乐,游戏动漫,美食
搞笑幽默
文艺,时尚
娱乐
视频音乐
许嵩控
美女,文艺,旅游,娱乐,体育,篮球,什么什么的
旅游
汽车,IT 数码,文艺,网球,羽毛球,旅行
健康
体育
文艺
美女,汽车
学生,生活,媒体,k 歌,电影迷,欧美
美食,美女,体育
旅游,美食,体育,IT 数码,文艺
文艺,新闻资讯,旅游,美食,投资理财
IT 数码,美食,星座命理,军事,体育
浙江生活,网络,新闻,投资,IT 互联网,策划
星座命理
游戏动漫,军事,IT 数码,体育
健康,IT 数码,体育
健康,游戏动漫,美女,娱乐,体育
重口味,读书分享,视频音乐,名人明星,襄阳生活,VIP,文艺,重口味腐女,爱海贼
时尚,星座命理,美女,军事,IT 数码
健康,游戏动漫,汽车,星座命理,体育,宅,80 后,听歌,电影,音乐
旅游,移动互联网
美女,娱乐

体育,娱乐
美食,军事,文艺
美国生活
汽车,美女,IT 数码,体育,娱乐
美女,军事,新闻资讯,健康,旅游,时尚,教育就业,IT 数码,娱乐
健康
视频音乐
教育就业
美女
美食,娱乐
旅游,新闻趣事,搞笑幽默,美容服饰,北京生活
听歌,平常心,摩羯座,手机,偶稀饭睡觉觉,吃货,美女,旅游,上网,宅
美女
汽车,时尚,游戏动漫,娱乐,体育
IT 数码
新闻资讯
旅游
武汉生活,健康
IT 数码,汽车,美女,自由,时尚,美食,旅游,80 后,电影,音乐
新闻资讯
游戏动漫,旅游,健康,娱乐
IT 数码
军事,星座命理,健康
体育
体育,汽车,IT 数码
新闻资讯,IT 数码
美女
看电视,爱美食,淡定,爱玩,汽车,体育
美食
旅游,体育
小说
爱自由,旅游
旅游
星座命理
时尚
体育
苦、踢足球、无业
魔羯座,看电影,搞笑幽默,浙江生活,美食,旅游,音乐,单车
娱乐,文艺,游戏动漫,星座命理
济宁生活
健康,投资理财,旅游,IT 数码,娱乐
征服
旅游
娱乐

八卦,语录,阅读,音乐
听歌
投资理财,新闻资讯,健康,旅游,娱乐
娱乐,体育,星座命理
星座命理
旅游,投资理财,汽车,教育就业,体育
搞笑幽默
游戏动漫,星座命理,美食,文艺,娱乐
新聞資訊,健康,旅遊,美食,娛樂
体育
健康
健康,文艺,天秤座
IT 数码
美女,娱乐,美食
美女,体育
新闻资讯,美食,旅游,健康,体育
健康,教育就业,投资理财,体育,IT 数码,旅游,星座命理,游戏动漫,娱乐
文艺,时尚,旅游,健康,娱乐
文艺,IT 数码,军事,游戏动漫,美女,狮子座,CF
IT 数码
健康,军事,旅游,美食,IT 数码
娱乐
美容,减肥,時尚,宅女一枚,健康,娱乐,文艺,IT 数码,旅游
时尚
真实力,帅哥,激情创业者,爱吃,喜欢睡觉,爱车,随性不随意,阳光,开朗
喜欢旅游,听音乐,设计,IT 互联网,拍照,快乐,美食控,创意,爱生活,爱睡觉
睡觉睡到自然醒,手機控,小說控,旅游
文艺,星座命理
美食,健康
旅游,美食,娱乐,吃,文字,闲
美食
视频音乐,星座命理,游戏,音乐,爱玩,小说,安静,动漫,宅,学生
美女,星座命理,体育
娱乐
新闻资讯,IT 数码,美食,投资理财,游戏动漫
微博,音乐,网购,文字,浪漫
双鱼座,音乐
视频音乐,搞笑幽默
娱乐,美食,旅游,体育,美女
武汉生活,文艺,新闻资讯,健康,娱乐,投资理财
游戏动漫,旅游,美食
体育,娱乐
健康,投资理财,旅游
旅游,美食,美女,穿衣美容

旅遊,汽車,文藝
宅女,美食,旅游,时尚,80 后,电影,音乐
娱乐,旅游
美国生活
新闻趣事,体育资讯,视频音乐,搞笑幽默,惠州生活
汽车,新闻资讯,旅游,军事,IT 数码,新闻,数码
旅游,美食,IT 数码,娱乐
娱乐
軍事
游戏动漫
旅游
军事,夜,文字,旅行
健康,时尚,旅游
健康,旅游
健康,旅游,美食,葡萄控,活泼开朗,善良,爱美食,爱旅游,爱生活,双鱼座
汽车,汽车爱好者
健康,旅游,美食,电子商务,海鲜零食,美味海鲜屋,贝壳,海鲜礼品,海鲜控,海鲜
美女,投资理财,IT 数码
名人明星,美女,视频音乐,新闻趣事,搞笑幽默
旅游
游戏动漫
旅游,搞笑幽默,美食,无锡生活,美剧,淘宝,天蝎女,咖啡,听音乐,B 型血
娱乐,美食
旅游
名人明星
无聊,时尚
文艺
旅游,新闻资讯,教育就业,美食,投资理财
文艺,娱乐
美食,娱乐,健康,文字,偶稀饭睡觉觉,摄影
星座命理
时尚,美食
健康
新闻资讯,文艺,投资理财,教育就业,旅游
娱乐,星座命理,游戏动漫,美女
军事
美食
美女
娱乐
健康,新闻资讯,美女,投资理财,汽车
旅游,70 后,睡觉
美食,搞笑幽默,视频音乐,名人明星,萌宠,汪酱控
健康,军事
娱乐

军事,IT 数码,体育,文艺,投资理财
穿衣美容,投资理财
旅游
美食
军事,IT 数码,文艺,体育,娱乐
娱乐
创意,体育,IT 数码,汽车,星座命理,投资理财,军事,旅游
视频音乐,像个,英雄,一样,活着
投资理财,教育就业,健康,文艺
文艺
美女
美食
一幅画!
文艺,健康
美容服饰,健康
时尚,美女,星座命理,美食,旅游
健康,美食,时尚,文艺,娱乐
星座命理,娱乐
IT 数码,娱乐,星座命理,美食
文艺,旅游,时尚
星座命理,娱乐
其实
IT 数码,旅游,美食,体育,教育就业
健康
旅游,美食,娱乐
娱乐
健康,时尚,文艺
美食
体育,NBA,90 后,游戏,睡觉,校园,学生,篮球
投资理财,新闻资讯,军事,IT 数码,体育
娱乐,旅游,水瓶座
开封生活,投资理财,教育就业,IT 数码,体育
囧,摩羯座,娱乐,游戏,看书,听歌,旅游购物
穿衣美容,健康,投资理财
娱乐,体育,旅游,美女
投资理财,旅游,健康,文艺,美食
旅游
随性,高中生,花痴,爱吃,宅女一枚,美食
美食
新闻资讯,健康,旅游,汽车,IT 数码
美食
美女,微博奇葩
星座命理,旅游
游戏动漫,娱乐,旅游,星座命理

多变,星座命理
旅游,文艺,新闻资讯,美食,体育
娱乐,旅游,文艺,时尚,体育
旅游,星座命理,美食,美女,健康
娱乐
时尚,美食,汽车,军事,娱乐,互联网,80后,NBA
娱乐
向日葵,猫,摄影,奋斗ing
80后,体育,羽毛球,自由,网络,吃货,文学,旅游
美食
体育
新闻资讯,教育就业,文艺,旅游,健康
娱乐,教育就业,旅游,IT数码,军事,美女,游戏动漫
旅游
90后,画画,欧美音乐,文艺
阜阳生活,健康,体育
90后,学生
娱乐
娱乐,美女,星座命理,美食
教育就業,文藝,娛樂,時尚,健康
游戏动漫
手机,美女,艺术,文字,天蝎座
旅游
文艺,旅游,美女,时尚,美食
吃,宅女
汽车,时尚,旅游,健康,新闻资讯,投资理财
旅游
娱乐,文艺,新闻资讯,健康,旅游
汽车,旅游,时尚,美食,娱乐
时尚,健康,文艺,美女,投资理财,教育就业
IT数码
教育就业,星座命理
美食
微博奇葩,娱乐
90后,篮球,创业,历史,听歌
旅游,娱乐,时尚
IT数码,美女,娱乐
美女,体育,动漫，吐槽,运动
美图摄影
微博奇葩
体育
旅游
体育资讯
娱乐

广东生活
星座运势,美食,八卦杂谈,情感生活,佛山生活
新闻资讯,旅游,娱乐
美食,阅读
娱乐
体育,美食,健康,新闻资讯,美女
娱乐,新闻资讯,文艺,健康,IT 数码
旅游
微博奇葩
星座命理,美食,教育就业,时尚,娱乐
上饶生活
娱乐
巨蟹座,吃货,旅游,宅女
美女,军事
萌宠
搞笑幽默,美女,穿衣美容,微博奇葩,北京生活
喜欢唱歌,胡思乱想,古灵精怪,90 后非脑残,文字控,文艺
文艺,美食,星座命理,健康,时尚
娱乐
游戏动漫
体育,文艺,IT 数码,时尚,军事
单身,90hou,体育,文艺
美女,播编表专业学生
健康,时尚,美食,教育就业,投资理财
辽宁生活
时尚,健康,美食,文艺,娱乐
美女,军事,娱乐,体育
健康
IT 数码
健康,体育,娱乐
美女,时尚,投资理财,教育就业,旅游,美食
教育就业,投资理财,IT 数码
旅游
新闻趣事
旅游
游戏动漫
美图摄影,星座运势,情感生活,新闻趣事,搞笑幽默
唱 K,户外,周杰伦,交朋友,外貌协会,韩剧,新闻资讯,投资理财,时尚,娱乐
旅游,游戏动漫,健康,美食
军事
名人明星,拾荒者
娱乐
美食
名人明星,教育就业,向往成功,优质青年

美女
旅游,美食,时尚,健康,娱乐
星座命理
游戏动漫,美女,星座命理,IT 数码,娱乐
love,娱乐
旅游,美女,IT 数码
穿衣美容,旅游,健康,时尚,新闻资讯,教育就业,投资理财,文艺,娱乐,美食
名人明星
投资理财,新闻资讯
美食
娱乐,听歌
美女,音乐,唱歌,传媒,上网,听歌,摄影,艺术,设计
娱乐
教育就业,健康,旅游,娱乐,美食
时尚,新闻资讯,健康,旅游,美食
美剧,文学,水瓶座,宅女,旅行,自由,听歌,90 后
文艺,体育,娱乐,旅游
时尚
处女座,英语,股票,网络,八卦
美食
美食,旅游,健康,时尚,游戏动漫,美女
星座命理,体育
体育资讯
娱乐,宅,篮球,大学生,玩儿,上班族,汽车,美食,创业中,吃货
美食,新闻资讯,汽车,旅游,体育
视频音乐,耳机发烧友,吉他控,篮球,学生,咖啡控,音乐控,音乐爱好者,真实力,奋斗
新闻趣事,搞笑幽默,娱乐
发奋青年,屌丝,旅游
军事,旅游
美女
娱乐,文艺,IT 数码,旅游,摄影,台球
军事
旅游
医生
美女
搞笑幽默
健康
军事,IT 数码
搞笑幽默
猫咪控,IT 数码,美食
星座命理
床头柜,休闲椅,餐椅,茶几,电视柜,布艺沙发,客厅家具
上海生活,投资理财
美食

文艺,教育就业,旅游
宅,90后,乐观,设计,旅行,IT,双鱼座,音乐
娱乐,投资理财,汽车,时尚,美女
游戏动漫
美陈,创意,公共空间,艺术,装置,雕塑,景观,策划,传媒,设计
文艺,体育,娱乐
投资理财,美女,IT数码
美食
文艺,金牛,巨蟹,音乐,电影,阅读,旅行,美食,钓鱼,乒乓
IT数码,柏林之声
搞笑幽默,体育
读书
健康,教育就业,旅游,美食,文艺
时尚,汽车,偶喜欢玩,白羊座,射手座,小说,90后,音乐
娱乐,星座命理,汽车
娱乐
美食,旅游,体育,奋斗,80后,马刺,好书
IT数码,文艺,娱乐,美食
美食
新闻趣事,搞笑幽默,美女,视频音乐,IT数码
星座命理,旅游
名人明星,校园生活,旅游,体育资讯,视频音乐
旅游,微博奇葩,搞笑幽默
外向,漂亮,可爱
乐活族,游戏ING,85後
星座命理
健康
星座命理,娱乐
投资理财,处女座
星座命理,美食,军事
旅游,新闻资讯,娱乐,体育,IT数码,听歌
旅游,美食,娱乐
体育,IT数码
美食,时尚
健康,新闻资讯,娱乐
旅游
时尚,健康,旅游,美食,娱乐
搞笑幽默
旅游
娱乐
新闻趣事
娛樂
美食,购物,淘宝
娱乐,投资理财,星座命理

服务员,文化,手工匠,红楼梦,驴友,DIY,好书,爱自由,文字,体育
爱生活,心理,爱自由,睡觉,媒体,我就是我,美剧,爱电影,旅游摄影,面食族
美食
视频音乐
A 股,动漫,电影,英语,水瓶座,学生,睡觉,读书,好性格,宅男
美女,星座命理
旅游,美食,萌宠,视频音乐,微博奇葩
新闻趣事
科学探索,读书分享,三明生活,新闻趣事,教育,书法,数学,思想
健康,汽车,游戏动漫,IT 数码,旅游
汽车,80 后
喜欢听歌,看电影,多愁善感,爱幻想,爱美食,敏感的天平女,宅女一枚,星座命理
神经大条,偶像剧
旅游
搞笑幽默
室内设计,收藏,怀旧,拍照,设计,教育就业,汽车,旅游,军事,文艺
名人明星
美女,星座命理,体育
教育就业
星座运势,美容服饰,名人明星,八卦杂谈,长春生活
大连生活,睡睡,爱旅游,美食家
美女
娱乐
三郡,葡萄酒,红酒,法国
教育就业,投资理财,汽车,健康,美女
其他生活,体育
娱乐,体育,美食,IT 数码,游戏动漫
名人明星,巨蟹座,中医
水瓶座,日本,lol,wow,紫色
游戏动漫,新闻资讯,旅游,美食,军事
体育
旅游,美女,搞笑幽默,IT 数码
娱乐,学生,电影
北京生活,体育
文艺,娱乐
旅游
娱乐
IT 数码,MES 项目管理
娱乐,宅
旅游,体育,足球
微博奇葩,旅游,美食,搞笑幽默
美食
乐观,羽毛球,搞笑,网购,交友,双鱼座,听歌,幽默,篮球,体育
游戏动漫

爱睡觉,美食爱好者,热爱生活,文艺,娱乐
时尚
微博奇葩
旅游,旅行
星座命理
汽车,游戏动漫,旅游,美食,军事
新闻资讯,美女,游戏动漫,IT 数码,体育
军事,新闻资讯,旅游,汽车,体育,美食
韶关生活
健康
汽车
IT 数码,美食,娱乐,体育
健康
美容服饰
教育就业,娱乐,文学,好书,宅女,音乐,动漫,时尚,旅行
80 后,经济,旅游,看书,体育,电影
游戏动漫
90 后,爱吃,上网,乐观,天秤座,大学,听歌,开心畅怀
德阳生活
旅游
美食
无聊,巨蟹,自由,爱生活,可爱,自然,笑,音乐发烧友,时尚,美女
真人 CS,打鬼子,极限运动
娱乐,旅游,文艺,时尚,教育就业
微博奇葩,视频音乐,新闻趣事,湖北生活
美女
游戏动漫
体育
娱乐
阅读,瑜伽
金牛座,偶喜欢玩,篮球,囧,睡觉,善良,大学生,90 后,好性格,自由
旅游
搞笑幽默
搞笑幽默,娱乐
名人明星
文艺
教育就业,投资理财,体育,汽车,美女
新闻趣事,体育资讯,搞笑幽默,上海生活
娱乐,美发达人,体育,美发,篮球,音乐,网络,美食,旅游
旅游,星座命理
旅游,星座命理,美食,娱乐,文艺
旅游,美食,文艺
新闻趣事,视频音乐,美女,微博奇葩,搞笑幽默
娱乐,文艺,星座命理,旅游

美食
美食,IT 数码
旅游,星座命理,美食,健康,时尚,娱乐,IT 数码
摇滚,格子控,喜欢音乐,情绪化,偏执狂,魔羯座
时尚,旅游,文艺,处女座
投资理财,健康,文艺,时尚
广州生活,旅游,美容服饰,90 后
吥夠成熟,懒瘾控,投资理财
旅游,美女,游戏动漫,娱乐
娱乐
游戏动漫
游戏动漫,旅游
星座命理,健康
旅游,文艺,花艺师,鲜花控,插花,花艺,鲜花,花店,送花,鲜花速递
文艺,体育
汽车,体育
新闻资讯,健康,旅游,美食,时尚
健康,星座命理,散打,90 后但我不脑残,处女座
美食,旅游,娱乐
军事,汽车,IT 数码,娱乐,体育
旅游,文艺,教育就业,健康,投资理财
健康,美女,投资理财
苏州生活
新闻,幽默
娱乐
吃喝玩乐,宅,睡觉,球迷,处女座
旅游,健康,时尚,汽车,娱乐,文艺
星座命理,文艺,娱乐,旅游,健康
美女,娱乐
文学,80 后
投资理财
娱乐
IT 数码,时尚
穿衣美容
缺乏安全感,起床困难户
微博奇葩,看电视,书,教师
体育
星座命理,健康,投资理财,时尚,娱乐
我爱 UC 浏览器
IT 数码,桌球控,苹果控,完美主义者,真诚直白,成长 ing,数码控
美食
水瓶,购物狂,电影控,80 后宅女,爱睡觉,自拍,苹果控,宅女加腐女,玩,上网
健康,美食,文艺,旅游,星座命理,娱乐
双鱼座

體育,美食,旅遊,健康,投資理財
夜猫子,旅游美食爱好者,淡定
营销,IT,数码,广告,设计,媒体,财经
教育就业,健康,旅游,文艺
游戏动漫
时尚,美食,健康,新闻资讯,旅游,美女
美食,天蝎座,文学,吃货
体育,军事
玉米,文字控,双重性格,双子女
美食,星座命理,IT 数码,文艺,娱乐
体育
娱乐,教育就业,时尚,汽车,投资理财
娱乐
教育就业,美食,新闻资讯
游戏动漫
听歌,数码,足球,新闻,旅游,90 后,电影,大学生,NBA,音乐
喜欢唱歌,娱乐
小说,动漫,幽默,睡觉
微博奇葩
文化产业管理,西班牙语,法语,日语,会计,esperanto
美食，游戏，朋友,电影，篮球，电脑
文艺,军事,体育
广东生活,新闻趣事
旅游,星座命理,美食,娱乐,文艺
旅游,新闻趣事
动漫,哈韩,仙后,妖精,爱 EXO,鳗鱼,90 后,爪机党
旅游
旅游,乐观,学生,动漫,美食,腐女一枚,英语,90 后,小说,睡觉
游戏动漫
娱乐
乐器,琴行,视频音乐,徐州生活,摇滚,吉他,驻唱,UKULELE,FUNK,PUNK
文艺
娱乐
上网,创业,篮球,学生,哥要得是激情
体育
美食
星座命理
旅游
美食
新闻资讯,游戏动漫,汽车,IT 数码,娱乐,体育
投资理财,旅游,星座命理,文艺,美食
文艺,美食,旅游
新闻资讯
IT 数码

娱乐,文艺,健康,旅游
爱猫爱狗,幾米,摄影,画画,设计,音乐,电影,书,旅行,90 后
旅游,投资理财,健康,汽车,文艺
说走就走的旅行
美女
星座命理
宅男,交友,自由,上网,音乐,80 后,篮球,双鱼座,电影,幽默
宅女,80 后,睡觉,网购
搞笑幽默,穿衣美容,拖油瓶一个
体育资讯,八卦杂谈
大学
游戏动漫,IT 数码,星座命理,投资理财
娱乐
旅游,江南古镇,小桥流水人家,简单,爱幻想,快乐,处女座,大学
健康,投资理财,旅游,IT 数码
游戏动漫,时尚,IT 数码,军事,汽车,体育
娱乐,旅游,健康,军事,游戏动漫
夜店,酒吧,音乐,时尚
文艺,娱乐,周杰伦铁杆粉丝,诺基亚手机,爱狗狗,设计,天秤座,微博,历史,读书
娱乐
健康,新闻资讯,投资理财
文艺
投资理财
IT 数码,文艺,体育
汽车
旅游
IT 数码,新闻资讯,健康,美食
时尚
美女
健康,时尚
娱乐,旅游,美女,美食,IT 数码
旅游,投资理财,文艺,美食
娱乐
美食
时尚
投资理财,娱乐
搞笑幽默
时尚,娱乐,文艺,旅游,健康
旅游,时尚
IT 数码,文艺
文艺,旅游,时尚,星座命理
美食,新闻资讯,投资理财,健康,旅游
美食
体育,娱乐,游戏动漫,时尚,文艺

新闻趣事,love,电影控,汽车,新闻资讯,旅游
旅行,娱乐,旅游,吃,生活,时尚,美食,游戏
旅游,星座命理,文艺,娱乐,美食
娱乐
旅游业,搜索引擎,酒店预订,酒店,旅行,渡假,网上酒店搜索,住宿,酒店优惠,旅游
IT 数码,时尚,星座命理,汽车,美女,健康,旅游,娱乐
美女,汽车,时尚
体育,美食,文艺,游戏动漫
娱乐,美食,时尚,投资理财,健康
娱乐
新闻趣事
时尚,星座命理,旅游,文艺,娱乐
旅游,美食,时尚,娱乐
湖州生活,健康
美女
旅游
健康,新闻资讯,体育
IT 数码,娱乐,健康,微博,好书,巨蟹座,90 后,玩儿,传媒,新闻
文艺,体育,娱乐
美女,投资理财,汽车,娱乐,体育
美食
星座命理
汽车,旅游,娱乐,美食
文艺,美女,时尚,游戏动漫,健康,娱乐,旅游
新闻资讯,军事,娱乐
微博奇葩,天蝎的,小吃货,大小胖
旅游,健康,时尚
新闻资讯,旅游,IT 数码,娱乐,体育
善良,90 后,电影,好性格,篮球,交友,体育,足球
游戏动漫
星座命理,时尚
沉默,摩羯座
微博奇葩,搞笑幽默,南京生活,流浪,军事,新闻资讯,教育就业,健康,旅游,体育
浙江生活,微博奇葩,穿衣美容,新闻趣事,健康,时尚,美食,IT 数码,娱乐
搞笑幽默,新闻趣事,旅游,广东生活,美食
文艺,娱乐
揭阳生活,完美主义,天蝎
美女
美食,新闻资讯,健康,娱乐
星座命理
新闻资讯,投资理财,教育就业
IT 数码,体育资讯,科比
冷笑话,幻想,体育
美食

游戏动漫,文艺,贴吧,英雄联盟,穿越火线
八卦杂谈,旅游,深圳生活
时尚,旅游,娱乐
时尚,投资理财,体育
美女,教育就业,星座命理,军事
汽车,娱乐,体育,投资理财,IT 数码
旅游,新闻资讯,娱乐
投资理财
星座命理
娱乐
健康,游戏动漫,汽车,魔兽世界,自行车,动漫,重口味,公益,减肥
汽车
健康,娱乐,游戏动漫
神探夏洛克,宠物控,园艺,DIY 爱好者,柯南,爱音乐,陆毅,Westlife
吉林生活
汽车,时尚,IT 数码,美女,娱乐,吃,听歌
旅游
IT 数码
投资理财,娱乐,旅游,健康,美女
旅行,汽车爱好者,汽车
旅游,投资理财,汽车,健康,美食
电脑,迪庆香格里拉,白族,洱源牛街,土木工程,昆明冶金,文艺
娱乐
美食,星座命理,健康,自由,天蝎座,音乐,玩儿
健康,旅游,星座命理,娱乐,IT 数码
乐观,吃货,健康,时尚,星座命理,美食,娱乐
旅游,美女,汽车,投资理财,美食
星座命理,金牛座
IT 数码,旅游
微博奇葩,穿衣美容,美食,娱乐,听歌,旅游
美食,等
娱乐
爱电影,睡觉,唱 k,巨蟹座,喜欢听歌,摄影爱好者
医药,淡定
美食
旅游
广州生活,穿衣美容,美女,汽车
睡懒觉,湖人
VF,SF
潮流,减肥,生活,搭配,美容,时尚
投资理财,汽车,美女,游戏动漫,旅游,海贼王,听音乐,宠物,90 後
娱乐
娱乐,星座命理,游戏动漫,体育
旅游

文艺,体育,军事
美食,视频音乐,搞笑幽默,星座命理,旅游
美食,旅游,健康,新闻资讯,汽车
穿衣美容,新闻趣事,搞笑幽默,美食,旅游,文艺,听歌
美食,娱乐
文艺,体育,娱乐
看电影,爱狗,逛街,旅游
90 後
体育资讯,真的爱她,酷爱军事实践
时尚,汽车,美女,健康
投资理财,文艺,美食,时尚
IT 数码
网络,玩儿,寂寞
汽车,军事,文艺,体育,小说,电影,听歌
娱乐,爱笑,喜欢唱歌
IT 数码,汽车
健康,娱乐,汽车,时尚,美食,旅游
欧美控,吸血鬼日记,会计,暮光之城,自由
军事,平常心,A 股,旅行
美食
娱乐
时尚
娱乐,美女,IT 数码,健康,新闻资讯,体育,星座命理,美食,军事
娱乐,网络,汽车
美食,健康,旅游
体育,美女,军事
娱乐
穿衣美容,娱乐
汽车,时尚,旅游,体育,80 后,足球,美食,偶稀饭睡觉觉,天秤座
投资理财,时尚,健康,旅游,美食
读书分享,美食,美图摄影,台湾生活
MUG,音游,rb,天然呆
旅游,汽车,体育
时尚
旅游,IT 数码,文艺,体育
旅游,娱乐
美女
娱乐
淑女
投资理财
美女,深圳生活,搞笑幽默,视频音乐,天道酬勤,自由
娱乐,狮子座
新闻资讯,早教,情商,教育,心理学
偶稀饭睡觉觉,吃货,幽默,旅游

手机
游戏动漫
旅游,娱乐,健康,新闻资讯,文艺,时尚
美食,体育
微笑
穿衣美容,美食,重庆生活,旅游,90后,白羊座,爱小说,爱摄影,爱旅游
时尚,IT数码,旅游,美食,文艺
文艺
军事
星座命理
篮球
娱乐,时尚
娱乐
娱乐
体育,文艺
娱乐,音乐
健康,军事,教育就业,新闻资讯,旅游,汽车
搞笑幽默
穿衣美容,海南生活,美食,旅游,服饰,情绪化,旅游美食爱好者,90后
念旧偏执,唱歌,起床困难户,宅,萌主,天然呆,二货,双鱼座,吃货,90后
旅游,美食,IT数码,游戏动漫
投资理财,新闻资讯,健康,旅游,美食
文艺
维尼夫妇,2PM,尼坤,健康,星座命理
搞笑幽默,体育
投资理财,美女
恋爱
搞笑幽默,微博奇葩,旅游,新闻趣事,新闻资讯,体育,教育就业
旅游,霜霜
旅游,美食,穿衣美容,微博奇葩,搞笑幽默
游戏动漫,IT数码
时尚,健康,投资理财
军事,健康,文艺
健康
帅哥,街拍,宝宝,萝莉控,星座命理,美食
by2,逛街,爱睡觉,美食
健康,旅游,投资理财,文艺,娱乐
新闻资讯,微博,美食,听歌,旅行,交友,处女座,80后
娱乐,时尚,健康,旅游,美食
新闻趣事
香港生活,穿衣美容,时尚,网购,猫控
娱乐
健康
交朋友,睡懒觉,懒人,音乐爱好者,休闲

军事,文艺,娱乐,游戏动漫,汽车,新闻资讯,IT 数码,体育
爱音乐,闷骚,懒,龟梨和也,O 型血,创意,画画,美国,金牛座,重口味
教育就业,时尚,健康,旅游,投资理财,美食,文艺,IT 数码
时尚,旅游
游戏动漫,美食
追求完美,感性,简单,台球
星座命理
美食,时尚,娱乐
美食,游戏动漫,体育,IT 数码
娱乐
健康,美食,军事,投资理财,IT 数码
汽车,创业,善良,游戏,音乐
IT 数码
旅游
IT 数码,娱乐,美女,旅游,时尚,文章同学
IT 民工,网络营销,电子商务,购物,京东商城,时尚
汽车
看书,投资理财,教育就业,军事,IT 数码,文艺
游戏动漫,文艺,体育,娱乐,IT 数码
美女,时尚,文艺,体育,星座命理,美食,娱乐,汽车
健康,文艺,体育,娱乐
美食
旅游,新闻资讯,汽车,IT 数码,娱乐
时尚,娱乐
投资理财,教育就业,新闻资讯,娱乐,美食,旅游
时尚,很粘人滴,双子座
汽车,军事,体育
投资理财,美食,时尚,旅游
健康,美食,新闻资讯,汽车,旅游,IT 数码,娱乐
汽车,体育
心灵宁静,旅游,策划设计,动漫狂热型,狮子座
时尚
时尚,旅游,娱乐
汽车,IT 数码
旅游
法国留学生,法国红酒进口商,法国文化,法国旅游,法语
美女
休闲
热爱生活,星座命理,电影,美食
文艺,投资理财,IT 数码,旅游,健康,美食
动漫
蘑菇美容,蘑菇保健
汽车
文艺

时尚,美女,爱睡觉
90,摄影,旅游
投资理财,旅游,IT 数码,文艺,娱乐
娱乐
旅游
健康,投资理财,旅游,娱乐,IT 数码
健康,IT 数码,体育,美女,美食
旅游,穿衣美容,新闻趣事,搞笑幽默,健康,星座命理
搞笑,舞蹈,音乐,美食,天秤座
萌宠,狮子座,小清新,90 后但我不脑残
旅游,金牛座
旅游,美食,新闻资讯,美女
搞笑,美食控,萌宠,美图摄影,fashion,旅游,天秤座
南京生活,新闻趣事,视频音乐,旅游,穿衣美容
孩子气,神经大条,小女人,星座命理,娱乐,美食,军事,旅游
IT 数码
旅游,健康,投资理财,IT 数码,文艺
新闻趣事
美食
教育就业,投资理财,体育,新闻资讯
摄影,营销,媒体,传媒
旅游
IT 数码,新闻资讯,游戏动漫,娱乐
体育,娱乐
新闻资讯,游戏动漫,旅游,文艺,美食
宅女,90 后,自由,学生,囧,艺术,弹钢琴,电绘,制图,看书
文艺,体育,娱乐,教育就业,投资理财
新闻资讯
健康,旅游,游戏动漫,体育
美食
游戏动漫,娱乐,90,爱猫,冷笑话,动漫控,宅男
汽车,军事,IT 数码
美女,体育,健康,教育就业,IT 数码
娱乐,时尚,美女,健康,美食
军事,娱乐,投资理财,新闻资讯,IT 数码
偶喜欢玩,校园,八卦,语录,微博,读书,电影,浪漫,网络,90 后
美食,美女,娱乐
旅游,自由
美食,游戏动漫,娱乐,旅游
时尚,娱乐,健康,星座命理,美食
时尚
星座命理
文艺
旅游

深圳生活,听歌
温柔,足球迷,汽车,文艺
会是那一个
旅游,星座命理,美食,文艺,娱乐
大连生活
汽车,投资理财,娱乐,IT 数码,美女
体育,美女
健康,时尚,娱乐
军事,投资理财,娱乐,健康
娱乐,旅游
体育
星座命理,美食,军事,IT 数码,文艺
新闻资讯,投资理财,美食,文艺,宅女,吃货,大学生,天蝎座,90 后,设计
文艺,美食,健康,娱乐,时尚
娱乐
营销
娱乐
文艺
睡觉,运动,双子座,学生
微博奇葩,新闻趣事,视频音乐,搞笑幽默,名人明星
游戏动漫,文艺,IT 数码,UI 设计,喜欢设计,热爱设计,共同进步
美食
新闻资讯
穿衣美容
娱乐
新闻趣事
美食,旅游,文艺,健康,时尚
搞笑幽默,体育资讯,双鱼,90 后非脑残,喜欢音乐,nba,闷骚,随性,怀旧
新闻资讯,文艺,娱乐
美食
钓鱼,火影忍者,music,篮球爱好者,娱乐,美女,旅游,IT 数码,文艺,体育
三国杀,美食,健康,旅游,军事,IT 数码
武汉生活
汽车
健康,教育就业,体育
IT 数码
阿尔,哔哩哔哩,bilibili,普爷,眉毛子,APH,黑塔利亞,黑塔利亚 APH,aph,游戏动漫
文艺
美食
時尚,星座命理,娛樂,體育
网络,宅男
娱乐
IT 数码
美图摄影

文艺,时尚,健康
发呆,巨蟹,内向,Music,幻想,单纯,巧克力,喜欢唱歌,漫画,校园
健康,时尚,文艺
旅游,教育就业,汽车,文艺,娱乐
文艺
时尚
旅游,阅读,足球,旅行,音樂
旅游,汽车
IT 数码,美食
游戏动漫
投资理财,游戏动漫
娱乐
旅游,创业,乐观,IT,巨蟹座,运动,80 后
娱乐
健康,新闻资讯,体育
幽默,巨蟹座,搞笑,善良,美剧,自由,90 后,好性格,吃货
汽车,美女,美食,娱乐,军事
健康,汽车,时尚,新闻资讯,军事
体育
汕头生活
旅游
自由,设计,上网,电子商务,美食,微博,生活
时尚
旅遊
旅游
体育
新闻资讯,投资理财,健康,时尚
美食
时尚
设计,美剧,狗狗,爱旅行,美女,投资理财
投资理财,星座命理,游戏动漫,时尚,汽车,旅游,IT 数码,体育
旅游,新闻趣事,搞笑幽默,武汉生活,美食
旅游,搞笑幽默,K 歌,乐观,游泳,白羊,SPA,美食,电影控,购物
教育就业,健康,投资理财,旅游,美食
视频音乐,美食
新闻资讯
时尚,旅游,娱乐
搞笑幽默,军事,游戏动漫,体育
看电视
其他生活
健康
美食,唱歌,没心没肺乐天派
健康,文艺,星座命理
娱乐

军事,娱乐,数码,旅游,美女,IT 数码,汽车
搞笑幽默,娱乐
娱乐,时尚,旅游,星座命理,美食
语言学,考研
旅游,娱乐
美女,时尚,教育就业,星座命理,军事
旅游,搞笑幽默,专一,snooker,吃货,偶稀饭睡觉觉,运动,囧,音乐
娱乐
阿妹粉丝,马伊琍粉丝,李小璐粉丝,装修爱好者,爱幻想,购物狂,吃货爱美食,音樂,减肥瘦身,臭美但懒得打扮
时尚,星座命理,游戏动漫,IT 数码,文艺
福建生活
淘宝控,爱唱歌,it,手机
健康,旅游,军事,娱乐,新闻资讯
星座命理
吃,搞笑,乐观,爱玩,热爱生活,爱幻想,搞笑幽默
文艺
娱乐
旅游,90 後,爱旅游,爱电影,听音乐
娱乐,文艺
旅游,IT 数码,汽车
IT 数码
台州生活
你的粉丝
旅游,美食,健康,小眼睛,爱笑
IT 数码,时尚,新闻资讯,投资理财,教育就业,娱乐
单身,购物,爱睡觉,名人明星,微博奇葩,搞笑幽默,穿衣美容
汽车
IT 数码
篮球用不熄!!!
微博奇葩
游戏动漫
旅游
宅男
旅游
星座命理,游戏动漫,娱乐
娱乐
美女
宠物图集,美食,美容服饰,搞笑幽默,徐州生活
文艺,美食,旅游
IT 数码,星座命理,美女,旅游,健康,投资理财,时尚,娱乐
旅游
旅游
美女

微博奇葩,娱乐,幸福罗,爱祥,猪迷,舞极限
游戏动漫,美食,娱乐,文艺,时尚
旅游
处女座,爱自由,简单,旅游,文艺
星座命理,旅游,健康,娱乐,体育
IT 数码
美食,健康,旅游
体育,投资理财,娱乐,健康,游戏动漫
视频,广播,黑莓,散步,时尚,健康,旅游,美食,文艺
巨蟹座
教育就业,娱乐
健康,旅游,耐心,爱心,体育,美女,游戏动漫,文艺,娱乐
时尚
新闻趣事,穿衣美容,美女
教育就业,IT 数码,娱乐,文艺,健康
时尚,游戏动漫,旅游,IT 数码,星座命理
街拍,粤语,欧美,美食爱好者,动漫,音乐
旅游,微博奇葩,新闻趣事,湖北生活
投资理财
视频音乐,新闻趣事,穿衣美容
美食,视频音乐,美女,旅游
汽车
娱乐,吃饭饭,水瓶座,幽默,睡觉,学生,时尚,90 后,听歌,音乐
旅游,美食,娱乐
时尚,旅游,娱乐
汽车,体育,IT 数码
美女
美食,游戏动漫,健康,娱乐,文艺
美食
娱乐,投资理财,美食
青春,瘦身,水瓶,美容护肤,美食,宅,校园
娱乐
旅游,微博奇葩,星座命理,美食,穿衣美容
自由,生人闷，熟人疯。
动漫迷,怀旧,宅女加腐女,漫畫,纸模型,橡皮章子
美食
文艺,娱乐,体育
新闻资讯,娱乐,美女
健康,美女,军事,IT 数码,体育
汽车,IT 数码
旅游,读书分享,新闻趣事,校园生活,星座命理,文艺,娱乐
娱乐,旅游
听音乐,街拍,唱 K,女人,品牌,O 型血,美食,健康,文艺,娱乐
微博奇葩,视频音乐,穿衣美容,旅游,搞笑幽默

健康,汽车,旅游,美食,娱乐
美食,汽车
游戏动漫,美食,新闻,吃,平常心,摄影,娱乐,唱歌,旅游,听歌
新闻资讯
文艺
游戏动漫
新闻趣事,穿衣美容,微博奇葩,旅游,搞笑幽默
时尚
湖人,80 后,林峯,交友,篮球,音乐,浪漫,IT,美食
旅游,穿衣美容,广东生活,新闻趣事
美食
新闻资讯,美女,时尚,汽车,IT 数码
校园生活,体育
IT 数码,健康,旅游,星座命理,投资理财,军事,娱乐
新闻资讯
学生,林丹,羽毛球
汽车
自然,英语,写字,矛盾体,宠物,执着,感性,简单
健康,IT 数码,美食
搞笑幽默
健康,美食,旅游
健康
萌宠,微博奇葩,旅游,搞笑幽默,90 后但我不脑残
睡觉,幽默,旅游,冷笑话,天蝎座,90 后,听歌,唱歌
一中,华农,广大
微博奇葩,新闻趣事,浙江生活
偶稀饭睡觉觉,90 后,汽车,写作,唱歌,交友,美食,旅游,自由,财经
健康
星座命理
科幻,Music,美食,IT 数码,体育,娱乐,游戏动漫
美女,健康
时尚,军事,旅游,汽车,娱乐
美女,社会工作专业,90 后射手,书法,乒乓球菜鸟,星座命理,教育就业
游戏动漫
爱笑,爱旅游,单身,简单,爱生活,文艺
军事,汽车,美女,旅游,体育
萌宠,微博奇葩,搞笑幽默
健康,教育就业,投资理财,星座命理,美食
济南生活,微博奇葩,搞笑幽默,美女,美食
旅游
文艺,时尚,旅游,健康,娱乐
健康,新闻资讯,时尚,投资理财,美食,社会责任
美女,旅游,军事,汽车,IT 数码
爱音乐,淘宝,购物,博客,夜猫子,唱 K,Music,旅行,狮子座,看书

美女
压力
美食,旅游
时尚
张根硕,娱乐
游戏动漫,IT 数码,美食
体育
投资理财,旅游,美食,IT 数码,娱乐
网络推广,游戏控,服饰潮流,网游玩家,网络创业,原创,美女,军事,IT 数码,娱乐
文艺,娱乐,时尚
旅游
汽车,游戏动漫,美女,IT 数码,健康,投资理财,娱乐
教育就业
文艺,偶喜欢玩
睡懒觉,安静,神经大条,朋友,宅女一枚,爱幻想,家居,书,看电视,美食
文艺
IT 数码,体育
体育
美女
美食,搞笑幽默
娱乐
视频音乐,新闻趣事
娱乐走向,韩语,养鱼养花,好书,街舞,音乐
健康,IT 数码,文艺,教育就业,星座命理,娱乐
健康,投资理财
健康,游戏动漫,时尚,娱乐,新闻资讯
微博奇葩,游戏,偶稀饭睡觉觉
健康,旅游,时尚,娱乐
新闻资讯,美女,娱乐,时尚
搞笑幽默,军事,体育,宅男
萌宠
游戏动漫,自信,美术,游戏,喜欢旅游,喜欢音乐,高中生
文艺,体育,美女,游戏动漫,汽车,教育就业,投资理财,健康,新闻资讯,娱乐
新闻资讯
音乐,狮子座,宅女,吃货,乐观,A 股
汽车
娱乐,IT 数码
时尚,健康,旅游,美食,IT 数码,80 后,爱睡觉,爱美食,爱我的宝宝,爱我的家
娱乐
娱乐
投资理财
美食,文艺,时尚,星座命理,投资理财
星座命理
娱乐,文艺

时尚,新闻资讯
美食
自由,时尚,旅游
教育就业,娱乐,文艺,美食,好书,平常心,听歌
教育就业,文艺,健康,时尚,旅游
锦州生活,体育资讯
游戏动漫,娱乐
美国直邮,支持支付宝,打折购物,美国代购
新闻资讯,IT 数码,体育
教育就业,投资理财,健康,时尚
旅游
健康,旅游
穿衣美容
视频音乐,旅游
美食
星座命理,IT 数码,娱乐,体育
汽车,时尚,美食
真诚,阳光,爱生活
美食
IT 数码,羽毛球,听歌,处女座
美图摄影,青岛生活,汽车,IT 数码,游戏动漫,美食,美女,时尚,新闻资讯,教育就业
时尚,娱乐,美食
体育
健康,新闻资讯,体育
视频音乐
星座命理
旅游,星座命理,军事,IT 数码,游戏动漫,美女,汽车
娱乐,美食,文玩,得瑟,80 后,白羊座
汽车,体育
学生,动漫,徐浩,硬币,文艺
穿衣美容
水瓶女,TVB,娱乐,时尚,星座命理,美食,旅游
上海生活
旅游,南京生活,萌宠,新闻趣事,微博奇葩
旅游,体育,宅
体育
双鱼座,爱自由,坚强
美食
穿衣美容,旅游
电脑,冷笑话,新闻
动画,杭州,NBA,小说,汽车,娱乐,幽默,足球,美剧,建筑设计
微博奇葩,健康,星座命理,文艺,娱乐,减肥,小说,唱歌,睡觉
汽车
投资理财,星座命理,军事

美女
足球迷,播音主持在校生,摩羯座,90 后
IT 数码,星座命理,美食,时尚,健康
娱乐
旅游,跳舞
汽车,新闻资讯,健康,美食,军事
教育就业,时尚,游戏动漫,文艺,娱乐
体育
80 后
游戏动漫
音乐
互联网,美食,健康,新闻资讯,旅游
时尚,健康,军事
体育
美女,娱乐,时尚,健康,投资理财
购物,唱歌,睡觉,吃
旅游,旅游美食
喜欢听歌,桌球,喜欢唱歌,体育,篮球
旅游
体育
美食控,懒
新闻资讯
时尚,健康,旅游,娱乐,文艺
IT 数码,旅游,新闻趣事,搞笑幽默,美女,大学生
娱乐,姜敏赫,郑容和,tiffiny,CNBLUE
健康
汽车,时尚,健康,旅游,美食,温暖与快乐,力量与热情,Coffee
武汉生活
化妆师造型师,彩妆,化妆师,平面设计,健康,听歌
减肥,吃喝玩乐,公益,巨蟹座,摄影,迷糊,学生一枚,随性,旅游,美食
懒人,90 后但我不脑残
娱乐,健康,旅游
视频音乐,新闻趣事,微博奇葩,广东生活,美食
视频音乐
散步,诗歌,书法,心灵,博客,纪录片,徒步,舌尖上的滋味
美女
旅游,穿衣美容,视频音乐,水瓶座
美女,美食
旅游摄影,梦想,青春,IT 数码,时尚,娱乐
旅游
游戏动漫,娱乐,投资理财,美女
搞笑幽默
游戏动漫
情感生活,育儿百科,新闻趣事,名人明星,搞笑幽默

娱乐,文艺,时尚,星座命理,健康
猎头顾问,Executing,Search,人力资源专家,高级人才寻访,委托招聘
健康,娱乐,投资理财,美食,旅游
汽车,时尚,美女,IT 数码
IT 数码,美女,自由
名人明星,视频音乐,旅游,80 后,篮球,处女座,NBA
军事,IT 数码,体育,手机,羽毛球,搞笑
体育,IT 数码,美食
旅游
健康,时尚,星座命理,旅游,文艺
IT 数码
娱乐
旅游,搞笑幽默,星座命理,广州生活,新闻趣事
教育就业,汽车
娱乐
天蝎座
时尚
旅游,娱乐
理性的文科生,考研党,吃货
美女,游戏动漫
文艺,健康,教育就业,旅游,体育
美女
旅游
IT 数码,投资理财,体育,美食,旅游
旅游
时尚
文艺,游戏动漫,时尚
MH4
云南生活
旅游,美食,萌宠,微博奇葩
来到这平台,请老师多指教
交友
游戏动漫,时尚,美食,健康,IT 数码,娱乐
时尚,旅游,美食
体育资讯,sneaker,NBA
有时犯二,喜欢睡觉,活泼可爱
星座命理,文艺,美食,投资理财,娱乐
新闻趣事
投资理财,新闻资讯,旅游,美食,文艺
美女
军事
美食,教育就业,健康,旅游,文艺
旅游,美食
投资理财

读书分享,卑微小丑
李嘉恒,kris,吴亦凡
谢娜,主持,滑雪,广播剧,浪漫,贵州生活,美食,军事
星座命理,唱歌,NBA,篮球,音乐
健康,时尚,美食,娱乐,旅游
军事,时尚,文艺,娱乐,校园,听歌,幽默,射手座
新闻趣事
美食,娱乐
偏执狂,fashion,看电影,旅游爱好者,hiphop,单身,篮球,幽默,网络,娱乐
福州生活,爱音乐爱睡觉
IT 数码
美食,游戏动漫,旅游,时尚
教育就业,时尚,文艺,美食,健康,旅游
喝喝,偶喜欢玩
河北生活
山东生活
教育就业,健康,投资理财
交友
90 后,旅行,格子控,理财,医生,白羊
傻,星座,矛盾体,shopping,随性,上网,学生,睡觉,美食爱好者,双重性格
健康,新闻资讯,娱乐
音乐爱好者,电影控,户外,设计师,动漫控,新闻资讯,美女,游戏动漫,时尚,汽车
娱乐
美女
文艺,美食,娱乐,时尚,旅游,健康
娱乐
娱乐,美食,时尚
神经病
体育,时尚,汽车,美食,文艺,健康
军事,汽车,IT 数码,体育,旅游,大学,学生
娱乐,健康,新闻资讯,听歌,看书
体育,汽车,IT 数码,投资理财,娱乐
娱乐
旅游,娱乐
娱乐,汽车,时尚,旅游,美食
90 后
其他生活
美女
IT,自由职业,电子商务,数码,创业,营销,双子座,汽车,看书,财经
娱乐
微博奇葩
军事,汽车
健康,文艺,娱乐
穿衣美容

军事,IT 数码,游戏动漫,动漫
体育,文艺,汽车
体育
爱
娱乐
IT 数码,文艺,投资理财,旅游,星座命理
旅游,萌宠,运城生活,搞笑幽默,美食
肥欢笑
游戏动漫
萌宠,穿衣美容,微博奇葩,搞笑幽默,旅游
网络营销,爱吃,摩羯座,90 後,胡歌的胡椒粉
IT 数码
足球迷
旅游,投资理财
娱乐
搞笑幽默,简单,天蝎座,炎亚纶,飞轮海
娱乐
旅游
时尚,汽车,健康,紫色,小说,学生,书籍,人生,体育,羽毛球
旅游
健康,旅游,IT 数码,美食,娱乐
健身,爱玩,宅,懒人,美食控,吃货一枚,水瓶,沉默,绘画,潮人
美女,军事,文艺,旅游,健康,双子座
社会的批判者
魔兽世界,画画,体育
娱乐
大连生活
萌宠
IT 数码
射手,羽毛球
健康,美食,旅游
美食,旅游,时尚,汽车,体育
汽车,体育
闷骚,狮子座,摄影,模特儿
强迫症,摄影,自然,购物,音乐迷,神经质,旅游,健康,美食
游戏动漫
视频音乐,自由职业,旅行,唱歌,动漫,上网,玩儿,设计,音乐,羽毛球
体育
旅游,美食
教育就业,星座命理,时尚,健康,旅游
美食,名人明星,新闻趣事,搞笑幽默,视频音乐
东东海,最爱汪东城
美食,旅游,体育,篮球,台球,羽毛球,dotaer
文艺

旅游
娱乐
美食
广东生活,穿衣美容
浙江生活,摄影,汽车,自由
美食
旅游,体育,教育就业,时尚,IT 数码
IT 数码,新闻资讯,体育,游戏动漫
美食
文艺,逛街,阅书,品茶
美食,游戏动漫,教育就业,健康,美女,军事
美食,投资理财,娱乐,汽车,旅游
投资理财,健康,旅游,美食,娱乐
大学生,好性格,游泳,快乐,tvb,火影忍者,医生,疯狂,葡萄牙
汽车,文艺,娱乐
旅游,体育,汽车
教育就业,投资理财,军事,文艺
健康,游戏动漫,IT 数码,旅游,星座命理
摇滚,新闻资讯,文艺,娱乐
幽默,宅
游戏动漫
娱乐
旅游,美食,娱乐
90 后,果粉
搞笑幽默
娱乐
健康,星座命理
美食,时尚,健康,娱乐,旅游
广告机,液晶拼接,网络广告机,数字标牌,触控一体机,液晶拼接墙,显示方案供应商,监视器
健康,新闻资讯,汽车,投资理财,旅游
IT 数码,投资理财,健康,体育
搞笑幽默,美食,视频音乐,美女
搞笑幽默,娱乐,手机,吃货,水瓶座
时尚
星座命理
IT 数码
微博奇葩
90 后,文字,读书,唱歌
哈尔滨生活
安妮海瑟薇,军事,新闻资讯,投资理财,游戏动漫
smile,Do,what,makes,you,happy,Be,with,who,享受生活
娱乐,美食,旅游
天秤座,Henry,吃货一枚,宅女,懒人,爱唱歌,美食
文艺,体育

健康,娱乐
时尚,美女,美食,游戏动漫
娱乐
体育资讯,搞笑幽默,泸州生活,搞笑,音乐
美女,八卦杂谈
旅游,教育就业,健康
健康,时尚,游戏动漫,新闻资讯,旅游
游戏动漫
知识产权代理
萌宠,美食,旅游
旅游,娱乐,投资理财,文艺,美食
校园生活,射手座
视频音乐,文艺
游戏动漫
旅游
娱乐,健康,星座命理
娱乐
IT 数码,美女,星座命理
旅游,看电影,聊天,逛街,购物
旅游,健康,汽车,军事,娱乐
娛樂
名人明星,听歌,吃货,唱歌,电影,时尚,网购,生活,巨蟹座,睡觉
时尚
体育
新闻资讯,美女,旅游,体育,娱乐
娱乐
娱乐
游戏动漫
游戏动漫,时尚,旅游,体育
新闻趣事
娱乐
梦想,有点二,爱笑,旅游摄影,自由,旅游
旅游
投资理财,美女,娱乐,美食,星座命理,时尚
旅游
校园生活,美食
IT 数码,时尚,新闻资讯,旅游,健康
新闻资讯
体育,文艺
新闻趣事,美食,旅游,成都生活,开朗,眼镜,看电影,听歌,微博,淘宝
娱乐,投资理财
旅游
娱乐
腹黑控,双鱼座,家庭教师,同人腐腐同人,奇基的时代,盗墓笔记,实力协会,悲观主义,6609,

桂林市宝贤中学
矛盾体,旅游,美食,文艺,体育,娱乐,听歌
时尚,星座命理,旅游,娱乐
文艺
汽车,旅游,美食,娱乐
健康,娱乐,美食,投资理财
新闻趣事,美图摄影,读书分享,IT 数码,星座运势,星座命理,军事,汽车,新闻资讯,时尚
天平座,喜欢音乐,吃喝玩乐,自由,营销,星座命理,娱乐,新闻资讯,投资理财
文艺,IT 数码,体育,娱乐
美食
游戏动漫
IT 数码
爱旅游,小女人,传媒,成都
穿衣美容
汽车,健康,投资理财,旅游
时尚,汽车,体育,旅游,军事
新闻资讯
旅游,新闻资讯,教育就业,IT 数码,美食
时尚,文艺,体育,娱乐
单身,学生,狮子座
人力资源,潮人,les,music,驴友,罗志祥
上网
搞笑幽默
娱乐,新闻资讯,旅游,时尚,星座命理
健康,美食,军事,文艺,体育,汽车,IT 数码
文艺,游戏动漫,美女,IT 数码,体育
军事,新闻资讯,教育就业,汽车,文艺
文艺,CPP 软件工程师
旅游
文艺,美食
大学生,听歌,90 后,音乐,音乐迷,时尚达人,活泼开朗,笑
搞笑幽默
新闻趣事,美食,校园生活,盐城生活
意大利,喜欢音乐,喜欢旅游,爱旅行,世界杯,媒体人,军事
纠结,内向,敏感,天平座,化妆品,美食,时尚,投资理财,娱乐
军事,文艺,娱乐,投资理财,星座命理
浙江生活,萌宠,搞笑幽默,健康,旅游,美食
教育就业
学生,睡觉,乐天派,旅游美食爱好者,幻想,小说
娱乐
文艺
美女
星座命理,美女,搞笑幽默,江苏生活,时尚
旅游,青岛生活

体育,新闻资讯,旅游,IT 数码,时尚
大学,旅行,学生
IT 数码,IT 互联网
穿衣美容
文艺
北京生活
美食,默默,2412,90 后,瓶子
投资理财,健康,星座命理,汽车,旅游
街舞,上海
穿衣美容
南京生活
90 后,网络,紫色,电影,旅游,好性格,唱歌
军事,汽车,美女,娱乐,旅游
健康
军事
美食,旅游,健康
Music,汽车,摩羯座,科比,自由,nba
星座命理
健康,时尚,游戏动漫,文艺,娱乐
旅游,偶稀饭睡觉觉,听歌,大学,运动,玩儿,羽毛球,善良,交友,创业
旅游,听歌,旅行,爱生活,坚强,笑
教育就业
宅女,吃,偶稀饭睡觉觉,射手座,90 后
娱乐,美食,星座命理,投资理财
投资理财,旅游,健康,新闻资讯,时尚
娱乐
体育,投资理财,汽车,旅游,IT 数码
视频音乐
摄影爱好者,好性格,看书
星座命理
美食,汽车,游戏动漫
体育
新闻资讯,军事,时尚,健康,娱乐
做自己想做的,走自己想走的,爱自己所爱的
新闻资讯
星座运势
旅游,美食,Britpop,洁癖,多重人格未分裂,Cartoon,摩羯,整理控,Indie,强迫症
体育
曼联拥趸,摩羯座,姚笛,90 后,荷兰
娱乐
美食
星座命理,旅游,健康,文艺
美女,体育,娱乐
旅游

娱乐
星座命理,教育就业,旅游,IT 数码,娱乐,体育
娱乐,旅游,军事
搞笑幽默
穿衣美容,时尚,宅
美图摄影
文艺
游戏动漫,娱乐
文艺,星座命理
投资理财,健康
旅游
自行车旅行,4 加 2 旅游,登山,潮车,死飞,自行车运动,运动,体育,健康,旅游
烹饪,语录,热情,吃货,90 后,旅游,天蝎,美食,潮流
投资理财,教育就业,健康
娱乐,旅游
健康,旅游
大叔受激萌,大叔控腹黑控,美男正太控,耽美控,二次元症候群,腐女宅女耽美狼,偶稀饭睡觉觉,好书,动漫,吃货
健康,新闻资讯,IT 数码,文艺,体育
美女
新闻趣事,广州生活,IT 数码,美食,旅游,病理,医学生
行星控,exo
投资理财
投资理财,80 后的尾巴,烹饪,财经
张敏,社会工作者,青少年教育,心理咨询,NGO,小组工作,个案工作,宜宾学院,社会工作
看书
体育,运动,听歌
星座运势,偶稀饭睡觉觉,音乐,天蝎座,电影
投资理财,新闻资讯,旅游,军事,汽车
游戏动漫,教育就业,投资理财,美食,娱乐
健康,娱乐,时尚,旅游,美食
娱乐
星座命理,游戏动漫,教育就业,文艺,娱乐
体育,娱乐,美食,星座命理,游戏动漫,IT 数码,投资理财,文艺
旅游,汽车,美女,娱乐,体育
娱乐
汽车,时尚,IT 数码,美食,新闻资讯,紫色,听歌,生活,处女座
娱乐,汽车,时尚,健康
时尚,美食
汽车,旅游,娱乐,IT 数码
时尚,旅游,健康,娱乐,美食
时尚
新闻资讯,汽车
娱乐

教育就业, 汽车, 军事, IT 数码, 体育
旅游
旅游, 汽车, 游戏动漫, IT 数码, 体育
军事
汽车, 军事
健康
健康, 美食, 娱乐, 时尚, 美女
简单生活, 旅游, 穿衣美容
新闻资讯
名人明星
旅游, 教育就业, 投资理财, 军事, 美食
娱乐
美女
健康, 投资理财
美甲控, 养生美容, 酷爱兰博基尼, 完美主义, 外貌协会, 巧克力, 文艺, 时尚, 旅游, 90 后
教育就业, IT 数码, 娱乐, 文艺, 美食
旅游, 投资理财, 旅行, 90 后, 电影
IT 数码, 美食, 新闻资讯, 投资理财, 娱乐
IT 数码, 美食, 旅游, 星座命理
动漫, 学生一枚
文艺
旅游, 军事, 时尚, 投资理财, IT 数码
恐怖电影控, 宅, 爱舞蹈, 无厘头, 学生
新闻趣事, 自由, 创业, 善良, 吃, 偶稀饭睡觉觉
搞笑幽默, 游戏动漫
生活, 阅读, 投资, 法律, 哲学, 律师, a 型血, 驴友, 美食爱好者, 天蝎女
房产, 星座运势
体育
军事, IT 数码, 游戏动漫, 汽车, 体育
微博奇葩
时尚, 娱乐, 健康, 旅游, 美食
新闻资讯, 健康, 旅游, 美食, 文艺, 喜欢听歌
娱乐, 体育, 星座命理, 美食
新闻资讯, IT 数码
旅游, 美食
旅游, 星座命理, 美食, 文艺, 娱乐, 新概念, 文字
星座命理, 自由, 睡觉, 听歌, 宅, 电影, 上网, 旅游, 玩儿, 吃货
名人明星
IT 数码
星座命理
八卦杂谈
睡懒觉, 腹黑, 沉默, 画画, 爱音乐, 冷笑话
90 後
教育就业, 旅游

军事,社会学,北京大学
摄影
健康,教育就业
教育就业,IT 数码
娱乐,体育,美女,游戏动漫,教育就业
旅游
飞行爱好者,乒乓球爱好者,歌曲控
时尚
美女
美食,文艺,旅游,娱乐
福建生活,星座命理,美食,穿衣美容
星座命理
腐女宅女耽美狼,广告,间歇性犯二,爱猫,游戏动漫
健康,新闻资讯,星座命理,军事,体育
体育
上海生活
无锡生活
宁波生活
美食
健康
旅游,主持人
娱乐
新闻资讯,旅游,娱乐,军事
星座命理,军事,JAVA,IT 数码,android
广东生活,游戏动漫
投资理财,军事,健康,新闻资讯,IT 数码
汽车
搞笑幽默,体育
娱乐
投资理财
文艺,美食,星座命理,旅游
微博控,乐观,幽默,K 歌,微博,互粉
自由职业,美食,听歌,时尚,摄影爱好者,电影,乐观,宅,旅行,射手座
健康,时尚,星座命理,美食,文艺,娱乐
游戏动漫,星座命理
美女,IT 数码,体育,娱乐
天蝎座的 90 后,TaylorSwift,高三,陈奕迅,音乐迷,好书,gossipgirl,英国,旅游
教育就业,投资理财,旅游,美食,军事,体育
文艺
汽车,教育就业,军事,IT 数码,投资理财,旅游,新闻资讯
娱乐,时尚,汽车,游戏动漫,IT 数码,体育,执着,爱玩,电脑,感性
投资理财
新闻趣事
时尚,文艺

新闻资讯,健康,旅游,星座命理,娱乐
IT 数码,汽车
美容服饰
美图摄影,娱乐
电脑,吃喝玩乐,学生
勤劳的 1B,累 B,苦 B,2B,SB
健康,娱乐
投资理财,星座命理
设计,80 后,乐观,音乐,摩羯座
健康,投资理财,美食,星座命理,吃货,手工,旅游,护肤
Android,大学,数码,80 後,B 型血,医学,音樂,平常心,运动,摄影
游戏动漫,时尚,IT 数码
娱乐
娱乐
萌宠
穿衣美容
体育,娱乐
旅游
健康,文艺,体育,娱乐
体育,娱乐,时尚
游戏动漫,时尚,旅游,美食,娱乐
学习 ING,旅游,果粉,萌,手机控,小萝莉,粉红控,k 歌
IT 数码,文艺,体育,娱乐,汽车
体育
旅游
美食,新闻趣事,搞笑幽默,池州生活,写作,小说,宅女,80 后,动漫,文学
体育
文艺
旅游
军事,好书,玩儿,IT,电子商务,汽车,教育就业,时尚,体育
文艺,时尚,健康,教育就业
美食,旅游,时尚,娱乐,爱笑,喜欢睡觉。
娱乐
旅游,宅女,吃货,睡觉,旅行,美食,双鱼座
美食
美女,美食,IT 数码,体育,娱乐
健康,交朋友,网购,音乐，90 後
游戏动漫,IT 数码,文艺,时尚,美食
湖北生活,囧,自由,亲情,金牛座,八卦
旅游
旅游,体育,美食,汽车,军事,美女
大学,IT 数码,美食,时尚,健康,娱乐
萌宠,电影,狗
游戏动漫

IT 数码
视频音乐,健康
汽车
时尚,汽车,星座命理,旅游,美食
健康,星座命理,美食,文艺,娱乐
吉林生活
文艺,娱乐,健康,新闻资讯,体育,美食,军事,IT 数码
韩剧,听音乐,美食,90 后,完美主义,小说控
娱乐,时尚,健康,旅游,美食,IT 数码
娱乐,时尚,健康,旅游,美食
时尚,娱乐,文艺,手绘
星座命理,娱乐
投资理财
电影
新闻资讯,健康,星座命理,旅游
游戏动漫
微博奇葩
健康,投资理财,汽车,文艺,美食
娱乐
零度新浪
教育就业,军事,旅游,美女,汽车,体育
娱乐
体育
投资理财
唱歌
旅行,时尚
IT 数码,美女,健康,旅游,军事
搞笑幽默,文艺,时尚,IT 数码
演奏
美女
投资理财,汽车,健康,新闻资讯,美女
搞笑幽默,搞怪,K 歌,爱唱歌,学生
巨蟹座,电影,美剧,旅行,玩儿,娱乐,体育
游戏动漫
美食,军事,IT 数码,娱乐,体育
视频音乐,娱乐
美女,游戏动漫,娱乐,体育
美食
新闻资讯,旅游,体育
军事
新闻资讯
权志龙,BIGBANG,狮子座,自由,听歌,学生,90 后
文艺,体育
淘宝控,宅男,好性格,旅行,偶喜欢玩,狮子座,交友,新闻,数码,音乐

新闻趣事,搞笑幽默,微博奇葩
新闻资讯,教育就业,军事,体育,文艺
美食,新闻趣事,体育,IT 数码,时尚,娱乐
美女
美食
美女,体育
娱乐
时尚,健康,文艺,美食,旅游
水瓶座,旅行,电影,星座,音乐
视频音乐,无狗不能活,跑调依旧唱,潮人一枚,苦逼高三党,搞怪无厘头,O 型水瓶女,音乐发烧友
星座命理,大学生,音乐,娱乐,吃货,水瓶座
文艺
星座命理
健康
健康
美女
体育
文艺,IT 数码,美女,娱乐
娱乐,星座命理
美食,旅游,美食,美女,汽车,体育
娱乐,美食,旅游,星座命理
游戏动漫,时尚,IT 数码,体育,娱乐
娱乐,文艺,天秤座,历史,小说,偶喜欢玩,吃,旅行
视频音乐,名人明星,穿衣美容,夜猫族,90 后,旅游,吃货,射手座,听歌,音乐
搞笑幽默,历史
IT 数码
美女
美食
投资理财,时尚,汽车,IT 数码,旅游
美女,体育
搞笑幽默
新闻趣事,穿衣美容
视频音乐
投资理财,美女,时尚,娱乐
娱乐
投资理财,旅游,新闻资讯
穿衣美容,星座命理,美食,微博奇葩,新闻趣事,游戏动漫
体育
健康
新闻资讯,美女,游戏动漫,时尚,美食,军事,IT 数码,健康,体育,娱乐
旅游
体育
Mysteryland,TomorrowWorld,基督教徒,EDC,Creamfields,Tomorrowland,EDM,Ultra

其他生活
旅游
天平座
90 后
旅游,教育就业
健康,时尚,动漫迷,拍照,music,小清新,日本,漫画,爱美食,爱幻想
教育就业,文艺,娱乐,大学,苹果控,90 后非脑残,英语,时尚潮流,休闲,手机看小说
追求完美,简单快乐,巨蟹座,旅行,听歌,星座,美食
健康
旅游,新闻资讯,投资理财,吃,80 后,幽默,金牛座,亲情,财经,新闻
汽车,星座命理,新闻资讯,旅游
天秤座,名人明星,睡觉
娱乐
健康
旅游,音乐,傻里傻气,偶喜欢玩,偶稀饭睡觉觉
娱乐
教育就业
胡思乱想,爱美食,咖啡,美食
搞笑幽默
旅游
IT 数码,健康,旅游,美食,游戏动漫,自己哭,自己笑,自己看着自己闹
旅游
旅游,奋斗,文学,喜欢旅游
教育就业,投资理财,体育,新闻资讯,健康,旅游
美食
新闻资讯,游戏动漫,体育,娱乐
娱乐
搞笑幽默,巨蟹
绘画,音乐,教育,美食,文艺,健康,旅游
教育就业,呆,木,笨
美食,星座命理,旅游,健康,娱乐
体育
健康,时尚
自由职业,赚钱,奋斗
游戏动漫,星座命理,体育,旅游
美食
bigbang,hiphop,羽毛球,大学生,90 後,奋斗,学习,music,猫咪,美食
文艺
穿衣美容,视频音乐
执着,学生一枚,完美主义,爱幻想,热爱生活
美女
健康,美食,星座命理,娱乐
90 後,手机,咖啡,聊天,android,神经大条,自恋,无聊
美食,旅游,星座命理

汽车,游戏动漫,旅游,体育,娱乐
娱乐,旅游
自由,90 后
游戏动漫
名人明星,宅
文艺,新闻资讯
美女,新闻趣事,搞笑幽默
美食,本命张若昀
体育
f（x）,天蠍女,时尚,美食
咖啡控
旅游
美女,幽默
游戏动漫
穿衣美容,搞笑幽默,新闻趣事,旅游,龙岩生活
搞笑幽默
娱乐,动漫,上网,听歌,宅,学生,90 后
娱乐,美食,时尚,健康,旅游
时尚,新闻资讯,旅游,健康,美食
星座命理
星座命理,IT 数码,军事
体育,新闻资讯,健康,旅游,美女
星座命理,体育,投资理财,教育就业,健康
旅游
萌宠,音乐,星座,林俊杰
旅游,健康
旅游
游戏，群体活动
教育就业,汽车,文艺,健康,投资理财
IT 数码,星座命理,新闻资讯
星座命理,健康,囧,自由,狮子座,篮球
IT 数码,旅游,汽车,军事,体育
IT,互联网,文学,动漫,网购,善良,宅男,创业,阅读,80 后
詹姆斯,星座,体育
教育就业,旅游,新闻资讯,投资理财,娱乐
星座命理
星座命理
娱乐,游戏动漫,文艺
游戏动漫,美女,旅游,军事,娱乐
游戏动漫,健康,星座命理,美食,文艺
名人明星
娱乐
健康,新闻资讯
旅游,无

时尚,旅游,星座命理,美食,文艺
拍照,吃喝玩乐,忧郁,任性,设计,潮人,淘宝控,bigbang,宝宝,单纯
微博奇葩,旅游,美食,穿衣美容,搞笑幽默
视频音乐,名人明星,美食,旅遊,穿衣美容
穿衣美容,微博奇葩,搞笑幽默,视频音乐,名人明星
时尚
娱乐
校园,囧,爱情
美食
我想要一个家,凌晨两点,九月十一,很容易满足,害怕一个人
体育
游戏动漫
爱自由,思考,射手座,偶稀饭睡觉觉,幽默,Music,偶喜欢玩,NBA
星座命理
美食
电影,听歌,自由
典型双子男
旅游,美食,文艺,娱乐,体育,关于高中学习,高考大学
时尚
文艺,时尚,健康,旅游,体育
汽车,游戏动漫,IT 数码,曾经的职业玩家,微博
90 后
游戏动漫,娱乐
80 後
美女,搞笑幽默,视频音乐
IT 數碼,美女,旅遊,美食,汽車
美食,娱乐,时尚
西安生活
健康,旅游,美食,娱乐
无厘头,摄影控,娱乐
艺术,水瓶座,设计师,时尚潮流,闷骚,影视,热情,性感,自由职业,大学
新闻趣事
娱乐
新闻资讯,IT 数码,美女
旅游,南京生活,自恋,猫,南京
美食,胡歌,西游,周星驰,美女,体育,娱乐
娱乐
星座命理,体育
美食
时尚
星座命理
美食
美食
文艺,健康,美食,旅游

军事
IT 数码,军事,汽车,星座命理,游戏动漫
上海生活
IT 数码
健康,新闻资讯,娱乐,时尚,美食
听歌,90 后,美容护肤,执着,吃喝玩乐,旅游爱好者,学生一族,时尚,星座命理
投资理财
体育,IT 数码,新闻资讯,军事
喜欢幻想和发呆,笑点很奇怪
美食,旅游,投资理财
搞笑幽默,美女
90 后,张翰,网球
汽车,旅游,体育,娱乐
安徽生活,双子座,会计,广播,吃喝玩乐,音乐迷,爱生活,创意,听歌
健康,体育,娱乐
校园生活,阳江生活,舞蹈,羽毛球,狮子座,好性格,学生,善良,音乐
读书分享,宅
娱乐,星座命理,美女,时尚,文艺
汽车,星座命理,娱乐,体育,健康
连接服务器失败
旅游
健康,美食,文艺,星座命理,娱乐
IT 数码
视频音乐,微博奇葩,星座命理,搞笑幽默,天蝎,星座控,移动互联网,IT 数码,汽车
体育,BIGBANG,NBA,少女时代,美国,喜欢唱歌,不会只是传说,大学,摩羯座,音乐
情感生活
吃货
双鱼座,发呆,柯南,无聊,美食
旅游
旅游,新闻资讯,文艺
跳舞,听歌,宅女,处女座,电影,玩儿,吃货,美食,游戏,旅行
健康,IT 数码,美食,星座命理
IT 数码
美女
旅游,健康,时尚,文艺,娱乐
山东生活
游戏动漫
我爱 UC 浏览器
旅游,星座命理,美食,文艺,娱乐
新闻趣事,微博奇葩,名人明星,视频音乐,美女
旅游,时尚
星座命理,新闻资讯,健康,时尚,投资理财,文艺
娱乐
军事,游戏动漫

娱乐,美食,汽车,时尚,健康
汽车,娱乐,体育,IT 数码,美女
数码,财经,大学,校园,感性激情,乐观,开朗……
双鱼座,90 后,微博,音乐,偶稀饭睡觉觉,自由职业,平常心,交友,电影,听歌
时尚
旅游
星座命理,旅游
健康,体育,娱乐
旅游,美食
刘恺威,忠实威化,娱乐
上善若水
IT 数码
娱乐,将宅进行到底,心静无惊
新闻资讯,汽车,文艺,时尚,教育就业
军事,体育,羽毛球
军事,投资理财,旅游,娱乐
游戏动漫
健康
微博奇葩,古典音乐,电影,看书,宅,足球
健康,旅游,星座命理,美食,娱乐
k 歌,80 後,低调,房地产,爱电影,星座命理
体育,文艺
美女,名人明星,美食,星座命理,穿衣美容
搞笑幽默
旅游,汽车,游戏动漫,苦逼学生党,吃货,大叔,动漫,篮球
星座命理,新闻资讯
汽车,美女,IT 数码,娱乐,体育,90 后,台球爱好者
文艺,旅游
赤西仁,钟汉良,荷兰,旅行,汽车,画画,时尚潮流,双鱼,淘宝控,商业
星座命理,汽车,教育就业,体育,健康,IT 数码
旅游,健康
美女
爱电影,爱旅游,周杰伦
旅游
美食
投资理财,游戏动漫,新闻资讯
美食
星座命理,IT 数码,健康,教育就业
美女
山口山,游戏动漫,灰常懒,爱美人儿,吃货,睡觉,读书
美女,军事
动漫,视频音乐,搞笑幽默,校园生活,娱乐,小说迷,古风迷,cosplay,宅女,摩羯座
星座命理,美食,投资理财,文艺,娱乐
娱乐

游戏动漫
游戏动漫,动漫迷,瓶邪,喜欢吐槽,喜欢暴漫,钟爱王爷,吸血鬼,懒人
文艺
平常心,八卦,听歌,唱歌
名人明星,旅游,微博奇葩,新闻趣事,搞笑幽默
京剧,媒体
娱乐,旅游,健康,美女,时尚
手机,新闻资讯,美食,IT 数码
玩
健康,人本万象,万事皆通
游戏动漫,时尚,星座命理,美食,娱乐
星座命理,美食,时尚
大连生活
美食
佛山生活,双子座
投资理财,教育就业,IT 数码
文艺
爱旅游,爬山,爱情,讷讷的眼睛
美食,健康
星座命理
女人,爬山,设计师,胡思乱想,语录,自行车,微博奇葩,旅游,新闻趣事,搞笑幽默
文艺,美食,娱乐
汽车,军事,IT 数码,游戏动漫
娱乐
偶稀饭睡觉觉,浪漫,上网,创业
奥黛丽赫本,free,张国荣
投资理财,美食,健康,旅游
IT 数码
美女
体育
羽毛球,语录,80 后,宅
美女
微博奇葩,美食,体育
娱乐,旅游,IT 数码,健康,教育就业,美食
文艺
教育就业,体育
娱乐
爱旅行,多愁善感
上网,善良,听歌,营销,新闻,篮球,看书,A 股
汽车
全心全意为人民
新闻趣事,纠结,ACG,白羊座,学生,生活
时尚,汽车,教育就业,美食,军事,美女,游戏动漫,星座命理
美食,文艺,娱乐

美食,旅游,游戏动漫,体育
旅游,搞笑幽默
看电视,旅游摄影,环保,热情
体育
旅游
写杂文,看闲书,研究植物
美女,汽车,军事,文艺,娱乐
亲情
搞笑幽默,新闻资讯
销售,爱自由,孩子气
体育,旅游,美食,健康
时尚,IT 数码,文艺,健康,军事,美食,美女,游戏动漫,投资理财
美女
投资理财,旅游,体育
健康,时尚,旅游,星座命理,体育
IT 数码
广东生活,汽车
不要迷恋哥,哥现在是死胖子
时尚,健康,旅游,美食,文艺
文艺,娱乐
娱乐
萌宠,名人明星
西安生活,健康
娱乐
军事,汽车,旅游,金牛座,运动
摄影,德国,北京,多肉植物,美食,旅游
娱乐
娱乐
旅游摄影,音乐达人,文艺,安徽卫视,电视节目制作,媒体人
游戏动漫,美食,星座命理,健康,文艺
萌宠
投资理财,汽车,娱乐
星座命理,美食,文艺,时尚
爱乐贝贝
军事
文艺,美食,投资理财,新闻资讯,健康,旅游
旅游摄影,欧美控,90 后但我不脑残,新闻资讯,娱乐,健康,旅游
娱乐
美食
文艺
体育
投资理财,游戏动漫,星座命理,体育,健康,IT 数码,文艺,美女,教育就业,军事
娱乐
美女

购物狂,古典艺术,休闲,穿衣美容,潮
星座命理,娱乐,健康,新闻资讯
视频音乐,微博奇葩,搞笑幽默,新闻趣事
摩羯座,80 后
游戏动漫,星座命理,美食,体育,娱乐
娱乐,美食,军事,IT 数码,旅游
旅游,穿衣美容
军事
体育
星座命理
呼和浩特生活,运动和旅游
娱乐
吃货,情绪化,懒人,睡懒觉,偶稀饭睡觉觉,天秤座,重庆
旅游,娱乐,投资理财,IT 数码,汽车
苹果控,唱歌,电影,美剧,处女座,看书,睡觉,音乐,90 后
体育
爱情
美食
旅游
景德镇生活
Lakers,Chelsea,RealMadrid
汽车,美食,军事,IT 数码
校园生活
美女,健康,IT 数码,汽车,体育
90,搞怪,投资理财,旅游,感性,美食
体育资讯,IT 数码,新闻趣事,校园生活
投资理财,新闻资讯,军事,文艺,娱乐
美食,军事
汽车
投资理财,旅游,军事,体育,娱乐
IT 数码
星座命理,美食,娱乐,投资理财
投资理财,游戏,新闻,金牛座,90 后,交友,IT,听歌,旅行,旅游
闲人,Sunseeker,支持教育革命,反对应试教育
美食,军事,投资理财,旅游
旅游
IT 数码
星座命理
体育
健康,美食
文艺,宣传片,广告,微电影,网络视频,病毒传播,短片,小品
美食,穿衣美容,微博奇葩,视频音乐,搞笑幽默,街舞控
旅游,吃货,旅行,多愁善感
投资理财

娱乐
体育
时尚,星座命理
宅女
四川生活
IT 数码
娱乐
汽车,健康,旅游,星座命理,美食
IT 数码,旅游
寻物器,双向防丢器,蓝牙防丢器,手机防盗器,手机防丢器,汽车黑匣子,撞车党,碰瓷,自驾游,汽车
时尚,游戏动漫,文艺,新闻资讯,旅游
娱乐,文艺,时尚,健康,旅游
时尚,健康,旅游,星座命理,娱乐
Rainababy 兒,李敏鎬,小澤之亜,Kaitou 少女
体育
云南生活
微博奇葩,搞笑幽默,青岛生活
美食,宅女
美食,星座命理
文艺,美食
投资理财
健康,美食
汽车
一些事一些情,娱乐
美食
新闻资讯,旅游,教育就业,游戏动漫,体育
美女
投资理财
投资理财,汽车,美食,体育
文艺,火影忍者
时尚
美女,旅游
新闻趣事,自由
娱乐
美女,体育
旅游,时尚
娱乐
美食,游戏动漫,时尚,文艺,娱乐
美图摄影,星座运势,情感生活
游戏动漫,娱乐
时尚,美食,健康,军事,IT 数码,文艺
IT 数码
时尚,星座命理,美食

美食,汽车,体育,自由,生活,亲情
健康,美食
健康,旅游,投资理财,汽车,美食
美食,旅游,美女,视频音乐
体育
娱乐,汽车
美女,游戏动漫,教育就业,文艺,IT 数码
文艺,健康,美食,旅游,IT 数码
LVFashion
时尚
健康
汽车,旅游
媒体,写作,财经,时尚
名人明星
游戏动漫
搞笑幽默,新闻趣事,旅游,星座命理,美食,娱乐
星座命理,穿衣美容,美女,新闻趣事,搞笑幽默
美女
新闻资讯,健康,IT 数码,时尚
IT 数码,足球,运动,宅
文艺,星座命理,旅游,健康,教育就业,娱乐
狮子座,爱睡觉,旅游,文艺,美食
美女
健康,时尚,星座命理,美食,娱乐
85 后 AB 型天蝎女,书,金融,电影爱好者,矛盾体
文艺,娱乐
人无我有,我有我优,善良,看书,旅行,听歌,电影
旅游
养生,活泼,乐观,阅读,日语,水瓶座,看电影,医学,投资理财,朋友
宅女一枚,时尚,music,家居,娱乐,美食,爱自由
旅游,搞笑幽默
旅游
IT 数码
美女
旅游,教育就业,星座命理
游戏动漫
投资理财,旅游
文艺,艺术
娱乐
汽车,旅游,体育
娱乐
宣传,建议或意见,税收,政策,咨询,投诉
爱睡觉,90 后,听歌,水瓶座,唱 K,Music,文字控
文藝

80 后
音乐,回忆加妄想,抵制玛丽苏无敌,95,漫画,爱二新 kids 一只,古言迷,宅女加腐女,刘诗诗,霍建华
健康
八卦杂谈,需要梦想,需要方向,需要眼泪,需要人陪
绘画,IT 数码,汽车,手机,JAY,巨蟹男,学习,微博,爱唱歌,喜欢听歌
旅游,星座命理,娱乐
手机挂件,手机,手机链,琉璃,饰品
文艺
读书,电影,幽默,历史,美食,旅游,亲情,睡觉
IT 数码,文艺
IT 数码,旅游,星座命理,美食,文艺,体育,健康,新闻资讯,娱乐
体育资讯
美女,星座命理,健康,美食,游戏动漫,时尚,娱乐
体育
IT 数码
星座命理,旅游,教育就业,新闻资讯
思考,音乐,成长 ing,自由,动漫,旅游
萌宠,美食,旅游
新闻趣事
体育,文艺,汽车,时尚,小野猫,性感,吃货,手机,传媒,锦州
游戏动漫
新闻资讯,健康,军事,娱乐,旅游
新闻资讯,美食
投资理财,旅游,游戏动漫
视频音乐,搞笑幽默,贵阳生活,娱乐,美食,星座命理,健康,时尚
旅游,水瓶
IT 数码
文艺
爱吃,幽默,天蝎座,看书,听音乐,发呆,健康,热爱生活
自由职业,摩羯座,90 后
美食,娱乐
美食,星座命理
教育就业,新闻资讯,文艺
旅游
健康
娱乐
广东生活
花样美男,情感生活,美容服饰,旅游,美食
星座命理
旅游
文艺,旅游爱好者,单身,阳光
天平座,80 後,幸福,健康,旅游
IT 数码,投资理财,健康,娱乐,时尚

娱乐,美食,旅游
星座命理,投资理财,教育就业,IT 数码,体育
美食,IT 数码,文艺,体育,娱乐
时尚
星座命理,90 后
星座命理
娱乐
游戏动漫,娱乐
科学探索
武汉生活
旅游
美女
军事,新闻资讯,体育,IT 数码,汽车
新闻资讯
教育就业,新闻资讯,投资理财,汽车,IT 数码
2 女子,吃货,美剧,双鱼座
微博奇葩
天蝎座,足球,乐观,娱乐
时尚
其他生活
名人明星,美女
IT 数码
美食,娱乐
时尚,旅游,健康,星座命理,文艺
军事,新闻资讯,时尚,教育就业,娱乐,星座命理,文艺
美食,时尚,投资理财,娱乐
时尚,娱乐
体育,投资理财,美女,IT 数码,文艺,教育就业,健康,星座命理
旅游
貌美如花
投资理财
美食,狮子座,校园,90 后,浪漫,亲情,好书,搞笑,睡觉,听歌
文艺,体育,娱乐,LES,無所謂,街舞,沉默,爱笑,懒,北京
美食,旅游,名人明星,美女
旅游,娱乐
汽车,旅游,美食,体育
唯一爱好睡觉
星座命理
A 米,意大利,皇马死忠,IED
体育
娱乐,美女,体育
时尚
搞笑幽默,篮球,看美女
娱乐,文艺,体育

文艺,美食,游戏动漫
体育
教育就业
IT 数码,文艺
美食,胡思乱想
IT 数码,旅游,投资理财,美女,娱乐
70 末的悲哀,没赶上一件好事
汽车
热爱足球
美女,娱乐
娱乐
福建,周毅
视频音乐,BIM 工程师,revit 讲师,revit 研究员,快乐,健身,微博控
名人明星
新闻趣事,搞笑幽默,美女
美女,文艺,美食,旅游,娱乐
校园生活
健康,时尚,旅游,美食,文艺
美女
旅游,IT 数码,体育
生活就是去生活
IT 数码
美食
旅游
文艺
美食
美食
娱乐,旅游,美食
健康,教育就业,文艺,恋爱择偶问题,离异家庭问题,婚姻家庭咨询师
娱乐,吃货
军事,IT 数码,龙湾华艺不锈钢
文字,历史,摄影,巨蟹座,新闻,财经,网络,营销,旅行,时尚
健康,美女
美食
娱乐
新闻趣事,广东生活,美女
投资理财
健康,旅遊,美食,美女,文藝
学生
新闻资讯
投资理财
音乐迷,90 后非脑残,恐怖片,桌游,努力,旅游美食,汽车,美食,旅游,体育
处女座
旅游,美食,健康,娱乐,文艺,射手座,瞿玮

游戏动漫
娱乐
搞笑幽默,四川生活
文艺
时尚,投资理财,娱乐,文艺,军事
工科男
投资理财,新闻资讯,军事,IT 数码,体育,娱乐
娱乐,星座命理,新闻资讯,游戏动漫,体育
新闻资讯,游戏动漫,投资理财,旅游,美食
喵星人,双重性格,爱旅行,最近爱上运动,不善交际,健忘,粉嫩黄绿都爱,蓝色,美食
旅游
游戏动漫
投资理财,体育
娱乐,时尚,旅游,健康,美食
健康,旅游,娱乐
足球,德国控,摄影师,设计,Photoshop,拜仁慕尼黑,旅行
体育,汽车
旅游
美食
体育,汽车
旅游,偶喜欢玩,读书,摄影爱好者,摩羯座,文字,乐观,90 后,生活
八卦,自由,游戏,90 后,手机,音乐,天秤座,历史,网络
文艺
新闻资讯,美女,星座命理,文艺,娱乐
文艺,娱乐
向日葵,香港,桌球,淡定,美女,军事,星座命理
美女
汽车,星座命理,文艺,体育,娱乐
汽车,文艺,娱乐
星座命理
时尚,文艺,美食,健康
旅游,自由,运动,电影,音乐,听歌,处女座
新闻资讯,文艺,美食,游戏动漫,体育
时尚,星座命理,游戏动漫,军事,娱乐
游戏动漫
娱乐
星座命理
美食,旅游,汽车,IT 数码,文艺
ALL 伸,阳炎厨,银土,APH
文艺,美食,旅游,健康,时尚
旅游
游戏动漫
视频音乐,八卦杂谈,情感生活,名人明星,武艺,动漫迷,音乐,华晨宇,文艺
搞笑幽默

90 后但我不脑残
游戏动漫,韶关学院,娱乐,运动,宅男,睡觉,80 后
时尚,投资理财,健康,旅游,美食
旅游,投资理财,IT 数码,新闻资讯,时尚
时尚
体育,教育就业,投资理财
宠物图集
旅游
美食
汽车,投资理财,体育,娱乐,游戏动漫
体育资讯
星座命理
健康,游戏动漫,时尚,星座命理,娱乐
体育,新浪读书
老鹰画室人
搞笑幽默,新闻趣事,旅游,微博奇葩,视频音乐
投资理财,旅游,星座命理,美食,文艺
时尚,娱乐,美食
时尚,游戏动漫,新闻资讯,健康,美食,美剧控,家居,双重性格,喜欢旅游,看电视
文艺,旅游,美食,新闻资讯,体育
紫色,浪漫,娱乐
热情,胆大心细,含金男,通信,通讯,美食,娱乐
视频音乐,听音乐,90 後,重口味,Music
江门生活
美食
汽车
美食,军事,文艺,体育,娱乐
美食,投资理财,军事,IT 数码,时尚
美食,旅游,健康,娱乐,文艺
体育
IT 数码,格子控,双鱼,爱旅行
娱乐
名人明星
旅游,汽车,IT 数码
文艺
游戏动漫
北京生活
汽车,军事
美女,体育
汽车
新闻资讯,健康,时尚,美食,投资理财
娱乐,IT 数码
星座命理,IT 数码,游戏动漫
娱乐,时尚,旅游,美食,文艺

军事
美女
教育就业,投资理财,旅游,美食,文艺
汽车,时尚,旅游,美女,体育
教育就业,IT 数码,健康,游戏动漫,军事
名人明星,旅游,新闻趣事,北京生活
搞笑幽默
视频音乐,萌宠
微博奇葩,星座命理,旅游,美女,穿衣美容
搞笑,玩儿,吃货,幽默,乐观,汽车,运动,处女座,旅行,自由
名人明星
体育
善良,狮子座,睡觉,吃,90 后,幽默,网络,乐观,唱歌,音乐
美食
娱乐
IT 数码
诚信互听
旅游,娱乐,美食
旅游
星座命理
娱乐,羽毛球,上网,玩儿,睡觉,吃,爱情,听歌,旅游,美食
旅游
简单,音乐控,游戏动漫
娱乐
美女
娱乐
文艺
体育
军事
美食,娱乐
时尚,娱乐,文艺,美食
时尚,新闻资讯,健康,美食,娱乐
星座命理,健康,时尚,娱乐,教育就业
军事
名人明星
体育,NBA,大学,等着我!
体育
爱安卓,爱手机,为安卓疯,为刷机狂,绝对安卓控,环球安卓汇
读书分享,美图摄影,美食,名人明星,宠物图集,娱乐
娱乐
完美主义,麦霸,新闻资讯,教育就业,健康,娱乐,文艺
体育,NBA,篮球,音乐
视频音乐,穿衣美容
美女

学习
汽车,健身,美食
搞笑幽默,金牛座,摇滚,运动,旅行
星座命理
投资理财,IT 数码,时尚,文艺,娱乐
娱乐,星座命理,时尚,投资理财
萌宠
美图摄影,宠物图集,名人明星,苏州生活,美食
其他生活
网络营销,自由,学生,视频音乐
IT 数码,狂吃不胖,射手座,动漫,上网,宅,学生,自由,90 后
新闻资讯,旅游,IT 数码
体育
旅游,读书分享,八卦杂谈,名人明星,上海生活
交友,看电视,喜欢旅游,麦霸,吃货一枚,愤青少女不文艺,浪漫,听歌,水瓶座,乐观
健康,旅游,美食,星座命理,文艺,娱乐
娱乐,时尚
时尚,健康,文艺,娱乐,美食
文艺,星座命理,娱乐
河南生活,搞笑幽默,新闻趣事,微博奇葩,美食
投资,自然,时尚达人,教育就业,投资理财,旅游,平常心,电影
旅游
娱乐
旅游
微博奇葩,萌宠,美食,娱乐,健康,军事
IT 数码
学习,娛樂,新聞資訊
娱乐
新闻资讯
搞笑幽默,微博奇葩,新闻趣事,穿衣美容,文艺
军事,旅游
美食
山西生活,双鱼座,听歌
游戏动漫,汽车,IT 数码,体育,双子座,世界杯,漫画,玩,球迷,90
体育
文艺,汽车,星座命理,旅游,娱乐
新闻趣事,穿衣美容,旅游,美食,广西生活
娱乐
娱乐
娱乐,美食,星座命理,游戏动漫
军事
美女
投资理财,美女,旅游,娱乐,体育
汽车

体育资讯
爱旅行,90,穿衣美容
手机,新闻,摄影爱好者,文学,爱唱歌
體育
娱乐
时尚
狮子座,听歌
搞笑幽默,微博奇葩,健康,娱乐,IT 数码,游戏动漫
星座运势,花样美男,美容服饰,八卦杂谈,搞笑幽默
投资理财,旅游,新闻资讯,汽车,美食
教育就业
时尚,娱乐,旅游
新闻资讯,旅游,IT 数码,体育,娱乐
名人明星
时尚,健康,旅游,美食,文艺,阅读,游戏,天蝎座
娱乐
娱乐
音乐爱好者,户外
娱乐
时尚,游戏动漫
体育
NBA,愤青,打篮球,体育
执着
IT 数码
搞笑幽默
旅游,星座命理
旅游,时尚,汽车,娱乐
旅游,文艺,娱乐,新闻资讯,教育就业
投资理财,教育就业,汽车,健康,文艺
娱乐
娱乐
文艺
吴世勋,朴灿烈,黄子韬,吴亦凡,EXO,宅女加腐女,萌宠,微博奇葩
美食,娱乐,时尚,汽车,游戏动漫,文艺
旅游,健康,教育就业,美食
健康,美食
娱乐
文艺
健康,体育,教育就业
美食,世界因我而精彩
旅游,军事,游戏动漫,投资理财,博彩,中山大学,澳門,Toastmasters,大学生
时尚,健康,旅游,美食,星座命理
IT 数码,汽车,时尚,体育
懒,吃喝玩乐,双子,吃货,娱乐,新闻资讯,旅游,美食

八卦杂谈
校园生活
时尚,娱乐
美食
体育,游戏动漫,IT 数码
深圳生活,奋斗,校园,投资理财
星座命理
体育,健康,军事
健康,旅游,文艺,娱乐
娱乐,学生,校园,音乐
健康,美食,IT 数码,娱乐,体育
旅游,时尚,美食,文艺
音乐控,强迫症,吃货,天秤,学生一枚
萌宠
生活,网购,新闻,电影
旅游
体育
美食,新闻资讯,健康
巧克力,美剧,90 后,唱歌,旅游,英语,巨蟹座
娱乐,自由,听歌,动漫
美食
天蝎座,音乐,电影,90 后,手机,NBA,滑板
羽毛球,阳光,五月天,笑,自然,微笑,爱电影,水瓶座,乐观,吃货
娱乐
澳大利亚生活,羽毛球
学生,奋斗,学习,dota,幸福,神经大条,喜欢听歌,游戏动漫
娱乐
汽车,美女,IT 数码,娱乐
搞笑幽默
旅游
美食
搞笑幽默,星座命理,新闻趣事
育儿百科,篮球,小说,听歌,好性格,大学生,吃货,射手座,NBA,动漫
美食
文艺,娱乐,美食,汽车,游戏动漫
美食,我要去旅行
名人明星
游戏动漫,IT 数码,娱乐,星座命理,美女
新闻资讯,美女,文艺,时尚,汽车,教育就业,健康,体育,娱乐
IT 数码,娱乐,游戏动漫,时尚,体育
旅游,文艺,时尚,矛盾,美术,游戏动漫,星座命理,美女,娱乐
健康,星座命理,美食,汽车,娱乐,体育
教育就业,体育
娱乐

旅游,娱乐
宅女,心屬廣外,舞蹈
新闻资讯,体育
娱乐
文艺,健康,时尚,星座命理,娱乐
体育
体育
车世界,旅游,视频音乐
旅游,狮子座,足球,游泳,健身,美术,o 型血,厦门,韩国
旅游
娱乐,游戏动漫
篮球
多愁善感,思考,吃喝玩乐,爱自由,追星,傻,o 型血,小清新,娱乐,文艺
游戏动漫
健康,时尚,文艺
美女,狮子座
乐活族,美食
文艺
游戏动漫
旅游,美食,军事,IT 数码,投资理财
美食,IT 数码,投资理财,时尚,新闻资讯
美食
旅游,新闻资讯,投资理财,健康,时尚
体育
娱乐,IT 数码
美女,旅游
强迫症,大学生,懒人一枚
IT 数码
陕西生活
萌宠
平常心,浪漫
体育
旅游,美食,穿衣美容,星座命理,新疆生活
旅游,时尚,爱旅行,小文艺,自拍自恋自乐,学生,天秤座,摄影,好性格,网购
娱乐
旅游,乐观,美食,偶稀饭睡觉觉,读书,宅,80 后,听歌
搞笑幽默
时尚,娱乐
IT 数码,美食,这里,没人?!
娱乐
美女
娱乐,文艺,体育,时尚,旅游,健康
蚌埠生活,梅西脑残迷、
幽默

新闻资讯,旅游,汽车,投资理财,军事
郭敬明,2NE1,权志龙,bigbang
时尚
教育就业,美食,文艺
时尚,文艺
旅游
看电影,玩游戏,吃零食
娱乐
时尚,娱乐
名人明星,维尼夫妇,李准基
EXO,吴世勋,HUNHAN
娱乐,游戏动漫,旅游,美食
NBA,湖人,科比,杜兰特,打败詹姆斯,广州恒大
游戏动漫,美女,IT 数码
乐观,吃货,幽默,小说
利物浦,双重性格,纠结,唱歌,星座命理,游戏动漫,投资理财
视频音乐,听歌,摩羯座,电影,玩儿,上网
音乐,文字控,90 后
一切尽在不言中
美女,游戏动漫,军事
娱乐
娱乐
囧,插画,旅游
娱乐
健康,新闻资讯,时尚,军事,投资理财
美食,旅游,星座命理
新闻资讯,娱乐,体育,游戏动漫
体育
旅游,健康,时尚,星座命理,美食
美女,军事,IT 数码,数码,水瓶座,汽车,听歌,电影,音乐,80 后
文字控,老秦人,自然爱好者
汽车,新闻资讯,健康,军事,IT 数码
旅游
游戏动漫,美女,教育就业,旅游,文艺
体育
IT 数码
体育,游戏动漫,宅
金牛座,静静呆着,没啥,学生
旅游,美食,健康,娱乐,IT 数码
星座命理,美食,时尚,文艺
天蝎座
投资理财,文艺,教育就业
旅游,星座命理,文艺,体育
健康,旅游,美食

旅游
娱乐
娱乐
时尚,文艺
新闻资讯
文艺,娱乐
时尚,娱乐,美食
90 后,欧美音乐,MORE,宅,游泳,好莱坞,睡懒觉,快乐
新闻趣事
酸奶控,帅哥控,购物狂
穿衣美容,四川生活
娱乐,游戏动漫,数码,摄影爱好者,搞笑,听歌
名人明星,笑,白羊座,日语
健康,时尚,旅游,美食,IT 数码
娱乐
美女
搞笑幽默,新闻趣事,IT 数码,视频音乐
娱乐
健康,美女,IT 数码,教育就业,娱乐
体育
娱乐
旅游,健康
教育就业,美女,游戏动漫,时尚,娱乐
爱猫控
健康,投资理财,美女,时尚,IT 数码
美食
美食
时尚,美食,文艺
单身,强迫症
体育
吃货
IT 数码
投资理财,娱乐,体育
体育
IT 数码,汽车,体育,美食,旅游
娱乐
游戏动漫
丽鬼冤仇,幽灵猎人,燃情男狙击,真没想到,摩登世界,中国的小皇,深海长眠,半夜鬼上床
星座命理,健康
娱乐,文艺,IT 数码,军事,星座命理
体育
美女,汽车,投资理财
小昔米,纤之影,葵静香,九月的高跟,月桂女神,再见中国海,敬酒歌,怪音
飞天小侠,永不沉没,血防线,风尚,之乎者也,获救,钢铁海龙兽,摩登天师

投资理财
美食
新闻趣事,旅游,IT 数码,职场招聘,茂名生活
棒打薄情郎,笠原弘子,水木年华,着迷,粉红色的回,铜头铁罗汉,爱上单眼皮,甲斐田裕子
体育,IT 数码,健康,汽车,美食
汽车
娱乐,外貌协会,追星迷,花痴
娱乐,汽车,军事,IT 数码,文艺
美食
搞笑幽默
美食
旅游
盗名窃誉,雅爵,我爱我夫我,狼族盟约,愿郎重吻妾,跟我一起疯,捍卫美国,我愿为你戒
三姑嫂,桑島法子,生化危机之,娇凤狂鸾,冬天的故事,白纸,暴龙兽,仓田佐祐理
文艺
吃喝玩乐,思考,天蝎座,宅女,交友,美女,时尚,汽车,投资理财,IT 数码
旅遊,文藝,遊戲動漫
吃货。
视频音乐
时尚,星座命理,美女,游戏动漫,体育
游戏,创业,摄影,钓鱼
健康,旅游,美食,文艺,娱乐
文艺,新闻资讯,健康,旅游,娱乐
汽车
搞笑幽默,园林假山,鱼池假山,英德黄腊石,园林景观石,流溪驳岸景观石,假山石,景观石工程,塑石
星座命理,美食
时尚,娱乐
娱乐
娱乐
投资理财,健康,旅游,文艺
星座命理,新闻资讯,IT 数码,游戏动漫,文艺
旅游
超级宅女
交友,户外,黑苹果青年,公益,足球,90 后,摄影爱好者,水景,美食,旅游
时尚
睡觉睡到自然醒,留学生,我就是我,读书,处女座,教育就业,时尚,文艺,娱乐,星座命理
时尚
旅游
读书分享,美食,新闻趣事,星座运势,北京生活
旅游
美女
娱乐
搞笑幽默

音乐,语录,乐观,时尚,英语,90后,阅读,自由
IT数码,娱乐,旅游,美女,军事
疯子一枚,90后宅女,时尚,健康,美食,汽车
文艺,时尚,星座命理,旅游,娱乐
旅游,新闻资讯,时尚
微博奇葩,旅游,美食,萌宠,江苏生活
新闻,英语,羽毛球,教育就业,健康,时尚,旅游,娱乐
IT数码,我爱佳凝
美图摄影
搞笑幽默,系好学生,文字控,金牛座,不是很乖,完美主义,未成年啦,幽默,乐活族,刀子嘴豆腐心
旅游,自由,浪漫
IT数码
文艺,新闻资讯,旅游,健康
健康,美食,娱乐,美女,游戏动漫
星座命理,美女,微博奇葩,搞笑幽默,肇庆生活
ChuckBass
娱乐
星座命理,IT数码,体育
摩羯,发呆,体育
搞笑幽默
体育
旅游
投资理财
我爱UC浏览器
文艺
娱乐
健康
军事
IT数码
深圳生活
好书,投资理财,军事,新闻资讯,旅游,时尚,汽车
娱乐
单身待解救,90后但我不脑残
健康
娱乐
健康
美女,教育就业,投资理财,新闻资讯,军事
游戏动漫
文艺,娱乐,汽车,新闻资讯,美女
美食,旅游
IT数码,体育,娱乐,汽车,慢,搞笑,游戏,乐观,运动,天蝎座
时尚,IT数码
娱乐

偶喜欢玩,小说,唱歌
武汉律师,武汉法院,武汉检察院,武汉公安局
搞笑幽默
IT 数码,新闻资讯
运动,美食,财经,音乐
专处,商标,著作权,幽默
义乌刑事律师
游戏动漫,投资理财,文艺,美食,娱乐
企业法律顾问,交通事故律师,刑事辩护律师,合同律师,法律咨询,法律顾问,法律服务,律师
湘潭律师,全国优秀律所
搞笑,羽毛球
律师,律师帮您维权,法律服务,法律顾问
新闻资讯
营销,摄影爱好者,创业,文学,生活,幽默,睡觉,旅行,旅游,听歌
投资理财,健康,教育就业
投资理财,旅游,美食,健康
文艺,旅游,IT 数码,娱乐,体育
游戏动漫
美食,军事,文艺,娱乐,体育
深圳生活
爱笑,睡觉睡到自然醒,无厘头,水瓶女,睡懒觉
美食,看书
旅游,健康,投资理财,文艺,娱乐
SuperJunior,蔡依林,shinee,双子座,少女时代,exo 粉,爱音乐,旅行,电影
文艺,新闻资讯
文艺,设计,电影,摄影爱好者,旅游,体育,美剧,婚礼灯光,婚礼,音乐
教育就业,汽车,游戏动漫,星座命理,文艺
军事,IT 数码,汽车,体育,文艺
时尚,体育,娱乐,美食,文艺
k 歌,吉他,纯音乐，越狱
娱乐,虾米一枚,天秤座 O 型女,宅
星座命理,旅游,健康,体育,文艺
时尚,娱乐,汽车,教育就业,星座命理
新闻趣事
感性,看书,天蝎女,旅游美食爱好者,喜欢听歌
娱乐
星座命理
美食
天津生活
体育,美食,动漫,音乐
体育
苦逼学生党,偏执,走读高中生
美图摄影,旅游,情感生活,美女,校园生活,徒步，探险,飞行，骑车
体育

穿衣美容
文艺
娱乐
体育
星座命理,游戏动漫,娱乐,体育,学生,NBA,足球,90后,吃
广东生活,时尚,书籍
娱乐
健康,旅游,美食
玉环婚纱摄影,新闻资讯,军事,美女,时尚,汽车
时尚
吃,看书,旅游,亲情,微博,摩羯座,自由,爱情,大学生,紫色
军事,健康,旅游,美食,娱乐
新闻资讯
美食
军事
娱乐,新闻资讯,教育就业,文艺,体育
新闻资讯,健康,文艺,旅游
投资理财,旅游,星座命理,美食,娱乐
视频音乐,星座命理,新闻趣事,搞笑幽默
汽车,旅游,美食,体育,娱乐
IT数码,娱乐,文艺,旅游,星座命理
爱自由,耽美,杂志,音乐控,90后但我不脑残,花痴
90后,林峯
80后的尾巴,爱自由,随性,美剧,健康
汽车
星座命理,胸有惊雷
美女,娱乐
军事,娱乐,体育
IT数码,80后,网络,电子商务,听歌,电影
娱乐,游戏动漫,时尚,星座命理,文艺
新闻资讯,IT数码,汽车
文字控,唱k,教育就业,时尚,美食,旅游
体育
娱乐
囧,语录,偶稀饭睡觉觉,体育,80后,宅,天蝎座,运动,美剧,校园
旅游
文艺,汽车
摩羯,淘宝控,护肤,80后,o型血,手机看小说,宅女一枚,孩子气,网购,乐观
健康
美女
时尚,投资理财,新闻资讯,旅游,文艺
娱乐
搞笑幽默
军事,星座命理,文艺,体育,IT数码

神经大条,90 后非脑残,天秤,娱乐,服装,高中生,书,诗歌,美食,旅游
宅女,唱歌,旅游
美女,投资理财,旅游,美食,军事
时尚
教育就业,IT 数码,世界杯,发呆,足球
纯仙团饭,本命允浩
时尚,美食
美图摄影
三分钟热度,处女座,90 后,学生一枚,海贼王
投资理财
美食,旅游,健康,美女,时尚
音樂,幽默,篮球,娱乐,唱歌,林俊杰,喜欢听歌,旅行,上网,动漫
新闻资讯,旅游,美食
旅游,IT 数码,汽车
美女
视频音乐,小二,吃货,狮子座,张杰控,唱歌
原创,music,JAY,杰伦
娱乐,旅游,文艺,汽车,时尚
摄影
旅游
健康,美女,娱乐,IT 数码,游戏动漫
星座运势
IT 数码
时尚,星座命理,教育就业,新闻资讯
听音乐,90 后
星座命理,旅游,娱乐,美食
搞笑幽默,娱乐
投资理财
旅游,风尘之子
美女,新闻资讯,教育就业,投资理财,旅游
les,旅游,80 后,上网,文字
健康,旅游,星座命理,美食,文艺
美食
娱乐,90 后
娱乐
时尚,上网,羽毛球,唱歌,游戏,八卦,搞笑,交友,乐观,旅游
IT 数码,娱乐,新闻资讯,美食,美女,体育
。只要唯一
搞笑幽默,乐观,校园,偶稀饭睡觉觉
上网,艺术,历史,腐,情绪化,韩国,任性
新闻资讯,旅游,投资理财,健康
冷笑话,感性,简单,旅游,新闻资讯,军事,文艺,听歌,搞笑,学生
视频音乐
投资理财,文艺,娱乐,旅游

星座命理,新闻资讯
回味秋日：昂看,谁在染缸里看到
美女,视频音乐,新闻趣事,搞笑幽默,微博奇葩
旅游
双鱼女,爱旅游,懒,看电影,时尚潮流,街拍,宅女一枚
电子商务,b2c,广告,商业摄影,摄影师
娱乐,旅游,体育,健康,美女
教育就业,游戏动漫
汽车,健康,投资理财,教育就业,旅游
文艺,星座命理,宅,乐观,互联网,听歌,90 后,游戏,读书,自由
游戏动漫
八卦
美食,偶稀饭睡觉觉,维尼夫妇,海贼王,喜欢听歌,傻,任性,美食控,玩儿
旅游,美食,萌宠,搞笑幽默,美女
健康,投资理财,旅游,军事,新闻资讯
汽车,时尚,娱乐
美食,吃货,IT 民工
cosplay,游戏动漫,闷骚,画画,腐女宅女,天蝎女,动漫控
美女,时尚,娱乐,校园,交友
教育就业
IT 数码,游戏动漫,娱乐,健康,美女
穿衣美容
旅游,摄影,羽毛球,美剧,电影,篮球
旅游,健康
游戏动漫
文艺,处女座
love
军事
旅游
搞笑幽默,新闻趣事,名人明星,美食,旅游
新闻资讯,美食,健康
汽车,教育就业,旅游,星座命理,文艺
文艺
穿衣美容
偶喜欢玩,天蝎座,八卦,吃货,上网,自由,校园,爱情,幽默,好性格
体育
旅游
投资理财,娱乐,IT 数码,汽车,体育
美食
娱乐,文艺
美女,娱乐
围巾专业设计师
广东生活
娱乐,时尚,投资理财,健康,旅游

投资理财,健康,美食,IT 数码,体育
新闻资讯,教育就业,投资理财,娱乐,体育
美食,星座命理,旅游,娱乐
名人明星
投资理财,娱乐,汽车,旅游,美女
旅行,汽车,运动,足球,游戏,篮球,唱歌,手机,听歌,90 后
美女,时尚,体育
军事
星座命理
汽车,体育,玩游戏,音乐,篮球
游戏动漫
旅游,美女,军事,文艺,体育
游戏动漫,体育
宅男,忠犬攻
快乐,微笑,90 后但我不脑残,爱电影,睡懒觉,时尚
美食,健康
交友,时尚,自由,浪漫,白羊座,艺术,大学生,幽默,动漫,电影
时尚,美女,旅游,教育就业,星座命理,娱乐
美女,时尚,投资理财,体育,旅游
美食
娱乐
时尚,好性格,宅,射手座,80 后
旅游,美食,文艺,娱乐
新闻资讯
游戏动漫,体育,罗外初二 7,90 后,C 罗,梅西,足球,托雷斯
IT 数码,时尚,汽车,美食,娱乐
健康,美女,体育
美食,IT 数码,军事
自由,唱歌
美食
星座命理,娱乐,文艺
时尚,娱乐,健康,旅游,美食
时尚
美食,投资理财,文艺,IT 数码,军事
娱乐
投资理财
IT 数码,美食,体育,投资理财,新闻资讯,汽车,军事,娱乐,旅游
旅游,新闻资讯,娱乐,美食,健康
爱 pchy4ever,pchy
囧,偏执狂,音乐爱好者,巨蟹座,网购,网络营销,电子商务,自信,80 後,爱电影爱音乐
娱乐
八卦,汽车,搞笑,幽默,旅行,旅游,美食,80 后
游戏动漫,文艺
房产

文艺
美食,投资理财,星座命理,时尚,健康
开朗,帅哥,计算机,IT 互联网,完美主义,简单,笑话,音乐控,自信,投资理财
美女
娱乐
搞笑幽默
数码,媒体,传媒,A 股
体育
旅游
上海生活,新闻趣事,穿衣美容,IT 数码,娱乐,旅游,美食,汽车,时尚
美食,娱乐,文艺,星座命理,体育
紫色
美女,游戏动漫
简单,自由职业,真诚,看电视,健康,听音乐,热爱生活,淡定,书籍,喜欢唱歌
体育
校园生活
美女
体育
时尚,健康,美女
新闻资讯
新闻资讯,投资理财,旅游,美食,娱乐
新闻资讯
搞笑幽默,文艺,文字
穿衣美容,美食,动漫,双鱼座,艺术,网购,旅游,学生,好书,音乐
辽宁生活
美食
旅游,吃,旅行,射手座,乐观,80 后
校园生活
健康,时尚,旅游,星座命理,美食
娱乐
投资理财,军事,IT 数码
科学探索
WOW 什么的,bilibili
时尚,投资理财,健康,新闻资讯
旅行,网购
好书,金牛座,听歌,乐观,羽毛球,好性格
娱乐,时尚,旅游,体育,汽车
健康,游戏动漫,美女,文艺,体育
新闻资讯,90 后,音乐
娱乐,美食,星座命理,允在 YoonJae,开白 KAIBAEK,腐,CP 党,天蝎座
美女,美食
时尚,教育就业,新闻资讯,美食,娱乐
时尚
汽车,星座命理,新闻资讯,军事,投资理财,阅读

娱乐
旅游
美女
IT 数码,汽车,游戏动漫
IT 数码,娱乐,体育,汽车
美食
新闻资讯,健康,旅游,美食,美女
旅游
体育
美女
乐橙,娱乐
体育
教育就业
娱乐
IT 数码,运动
军事,汽车
旅游,时尚,健康,美食,娱乐
时尚,投资理财
教育就业
娱乐
旅游,星座命理
宠物图集,搞笑幽默,情感生活,星座运势
放荡不羁爱自由,徒步狂热分子,滑板抢回街道,耳机发烧,摄影发烧,李准基死忠,切尔西死忠
时尚,投资理财,文艺,IT 数码,汽车
娱乐
玉石爱好,收藏,健康
美食,旅游
名人明星
健康,美食,旅游,玩儿,网购,宅女,小说
旅游美食,听歌,电影,善良,自由,处女座
时尚,偶喜欢玩
篮球,听歌,狮子座,90 后
美食,旅游,健康,时尚,文艺,娱乐
娱乐,体育
IT 数码
体育,90 后,NBA,天秤座,睡觉,运动,宅,上网,乐观
名人明星
穿衣美容
文艺
教育就业
美食,旅游
健康
军事,IT 数码,健康
体育

名人明星
娱乐
娱乐,旅游,健康,游戏动漫,美食
教育就业,时尚,健康,文艺,娱乐
时尚,健康,旅游
旅游
80后,电影
汽车,旅游,美女
美食
体育
游戏动漫,美女,娱乐,汽车,IT数码
健康,美食,体育,娱乐
微博奇葩
娱乐
星座命理
搞笑幽默
时尚,健康,旅游,美食
发呆,听音乐,娱乐,旅游,文艺
网购,美食,迷糊,电影,天蝎,平模,小脸
文艺
文艺,旅游,时尚,投资理财,汽车,健康,美食
娱乐
健康,文艺
文艺,体育,娱乐
时尚,汽车,IT数码,文艺,娱乐
学生一枚,胡思乱想,猫控,宅女,双鱼座
军事,投资理财,教育就业,时尚,新闻资讯
穿衣美容
文艺,旅游,美食,投资理财,健康
时尚,健康,旅游,美食,娱乐
旅游,军事,IT数码,美女,美食,游戏动漫,体育,汽车
投资理财,IT数码,体育,时尚
小说
名人明星,穿衣美容,微博奇葩,山东生活,搞笑幽默
美女
美女,娱乐,游戏动漫,教育就业,军事,星座命理,旅游
投资理财,健康,旅游,星座命理,美食,娱乐
搞笑幽默,双鱼座,微电影,音乐控,梦幻西游指挥,秦时明月,游戏,搞笑
美女,游戏动漫,汽车,IT数码
吃货一枚,健康,娱乐,美食,双鱼座
新闻资讯,娱乐
i,睡觉,上网,吃,摩羯座
旅游
穿衣美容

旅游
美食,旅游,投资理财,IT 数码,汽车
罗志祥,学习,画画,搞笑,乐观,美食,星座命理,旅游,娱乐,游戏动漫
旅游,星座命理,美食,IT 数码,娱乐
星座命理,游戏,高三,学习,吃喝玩乐,动漫
娱乐,教育就业,军事
偏执狂强迫症,闹眼子
教育就业
看书,搞笑
星座命理
美食,时尚
娱乐
娱乐
IT 数码,爱旅游,低调,体育,手机,星座命理,随性,科技,金融,梅西
美食
旅游
娱乐
美女
娱乐,时尚,IT 数码,旅游,美食
娱乐
游戏动漫,汽车,体育,湖人
体育,文艺,娱乐,旅游,教育就业,军事
美食,娱乐
星座命理,旅游,名人明星,穿衣美容,视频音乐
军事,文艺,健康,新闻资讯,旅游
旅游,自由,篮球,白羊座,汽车,时尚,动漫,吃
数码,生活,睡觉,音乐
体育,旅游
短发的长发少年
投资理财
健康,娱乐,旅游,美食,时尚
娱乐
视频音乐
健康,新闻资讯,汽车,体育,军事
舞蹈,运动,手工
摩托车,模具
娱乐
娱乐
旅游
投资理财
星座运势
策划,IT 互联网,旅游摄影
旅游,美食
健康,时尚,美食,娱乐

美食
美国生活
自由,亲情,电影,水瓶座,听歌
翻唱,手绘党,ACG,学生党
美女,新闻趣事,搞笑幽默,书法,70后,农民,阅读,和纪录片,爱玩游戏
腐宅耽美狼,动漫控日剧饭,爱E神爱生活,萌系物体收集控,强迫症
文艺,健康,旅游,星座命理,美食
体育
美女,时尚,新闻资讯,教育就业,军事
旅游,教育就业,美食
巧克力,古典艺术,爱美食
搞笑幽默,微博奇葩,美女,星座命理,新闻趣事
庚饭,妖精,双子座,射手座,英语,演员,追星,火影,娱乐
娱乐
90,金牛,幸福,喜欢音乐,街拍,军事
娱乐
名人明星
租赁,足球,置业,学区房,买房,海景房,房产新闻,抵押贷款,二手房
你就不要想起我
健康,旅游,星座命理,美食,娱乐
旅游
紫色,狮子座
IT数码
羽毛球,小说迷,90后,温柔,学生,动漫,听音乐,美女
健康
萌宠
美女,汽车,投资理财,旅游,娱乐
体育,旅游,健康,90后,乐观,狮子座
海贼王,动漫,渣画
美女
新闻资讯,文艺,旅游,美食,游戏动漫
健康,时尚,娱乐,美食
读书分享
娱乐,文艺,健康,教育就业,时尚
体育
旅游,天蝎座,半个吃货,纯P粉,男神沈煜伦
摩羯女,娱乐,时尚,旅游,文艺,喜欢听歌,摄影,动漫,小说,青春
IT数码,美食,游戏动漫,美女,体育
新闻资讯,美食,教育就业,军事,文艺,大懒虫,囧货
爱美食,巴萨,三国杀,体育
爱旅游,军事
旅游,美食,健康,娱乐
文艺,IT数码
时尚,旅游,美食

电影控,天平座,玩,快乐,美食控,旅游,时尚,游戏动漫,美女,IT 数码
星座命理,旅游,文艺,健康,新闻资讯
穿衣美容
读书分享,视频音乐,娱乐
双子座
体育,游戏动漫
文艺,健康,时尚,星座命理,IT 数码,发呆,爱睡觉,喜欢音乐,美食
投资理财,新闻资讯,美女,宅男
冷笑话,交朋友,遊戲動漫,工作,独立,娱乐
穿衣美容
新闻趣事,校园生活
美女,军事,宅,80 后,90 后,美食,旅游
美食,旅游,逛街购物,热爱网购,80 后妈妈
漫画,日语,发呆,天蝎,动漫控,柯南
教育就业
视频音乐,美食
健康,星座命理,军事,IT 数码
新闻趣事,吃,美剧,宅女
健康,美食,旅游
美女,幽默,看书,美食,巨蟹座,善良,旅游
旅游,宅女
体育,娱乐
文艺,美女,美食,娱乐,旅游
闷骚,汽車,西班牙,旅遊,爱睡觉,爱音乐,看电影,登山,诗词,散步
通信,健康,星座命理,美食,军事,娱乐,诚实
美食,健康,游戏动漫,旅游,娱乐
IT 数码,旅游
美女,时尚,旅游,文艺,体育
美容护肤,化妆品,团购,美容,时装,网购,服装,外贸,旅行,时尚
教育就业
音乐控
美食,新闻趣事,名人明星,校园生活,搞笑幽默
文艺,游戏动漫,美食,旅游
山西生活
娱乐
游戏动漫,美食,娱乐
旅游,美食,视频音乐,搞笑幽默,西安生活
健康,美食,IT 数码,文艺,娱乐
美食
娱乐,体育
娱乐,美女,游戏动漫,文艺,体育
星座命理
投资理财,军事,新闻资讯
美食,旅游,教育就业,投资理财,文艺

爱美食,书籍,旅遊,听音乐
美食
娱乐,旅游,文艺
旅游
娱乐
旅游
军事,美食,健康,美女,游戏动漫
娱乐
天秤座,幽默
英语,狮子座
美食,时尚
视频音乐
汽车,娱乐
美女,努力,宝宝,孩子气
神经质,射手座,向日葵,阳光,爱生活,星座命理,美食,旅游,健康
娱乐
文艺,游戏动漫,新闻资讯,IT 数码
狮子座
摄影,固执,追求完美,挑食,读书,天蝎座,乐观,80 后,睡觉,旅游
爱生活,开朗,逛街,暮光之城,摩羯女,吸血鬼日记,时尚
时尚
体育
穿衣美容,健康
双子座
TomHiddleston, 蛰 礼 , 文 字 ,BellGrills,TamasWells, 五 月 天 , 二 点 五 次 元 , 李 霄 云,HarryPotter
美食,微博奇葩
心理学,宠物,懒,爱电影,旅行,紫色,我就是我,手机控,爱自由,爱音乐
新闻趣事
时尚,文艺,投资理财,游戏动漫,教育就业
游戏动漫
IT 数码
强迫症,赚钱,读书,善良,巨蟹女,双重性格,完美主义,爱猫,上班族,90 後
减肥 ing,护肤控,购物狂
健康,星座命理,美食
娱乐,体育
星座命理,投资理财
汽车,美食,投资理财,时尚,健康,旅游,文艺
旅游,射手座,幽默,写作,文学,网络,看书
游戏动漫,时尚,美女,IT 数码
教育就业,娱乐
新闻资讯,教育就业,时尚,文艺,娱乐
娱乐,IT 数码,健康,时尚
娱乐,文艺,星座命理

投资理财,健康,旅游,美食,星座命理,文艺,IT 数码
体育,投资理财,旅游,星座命理,娱乐,时尚
娱乐
体育
军事,娱乐,教育就业,投资理财
文艺
搞笑幽默
善良,小清新,旅游,学生
娱乐,旅游,音乐,水瓶座
四川生活,搞笑幽默
时尚,韩国,随性
文艺,娱乐,时尚,美食
文艺,汽车,旅游,星座命理,娱乐
投资理财
IT 数码
文艺
新闻资讯,旅游
美食
游戏动漫,美食,体育
旅游
健康
体育,IT 数码,好脾气,上进心责任感,待解救男青年,周董 fans,爱阅读,网购控,影视控,三星控
美女,游戏动漫
娱乐
设计,音乐控,文艺,娱乐,健康,时尚,星座命理,画画的双子座
萨瓦兰,荷兰,武汉,腐
旅游
文艺
ACGN 能力者
娱乐
游戏动漫,体育,IT 数码,星座命理,乐幽默,爱交友,喜助人,运动蜜,三分钟,无知难
娱乐
IT 数码
投资理财,汽车,时尚,IT 数码,娱乐
搞笑幽默,大学,平常心,篮球,自由,好性格,动漫,90 后
美食,旅游,时尚,娱乐
人追求什么,健康就好
体育
健康,旅游,娱乐
娱乐
娱乐,唱歌,偶喜欢玩,狮子座
一个人 sinian,听歌爱歌词,舍不得,时而冷漠,很二很二,处女座,90 后
钢琴,街舞,体育

旅游,IT 数码
IT 数码
投资理财,IT 数码
时尚,娱乐
星座命理
教育就业,旅游
旅游,文艺
游戏动漫,投资理财,美女,旅游,体育
IT 数码
时尚,美食,文艺,娱乐
汽车,新闻资讯,游戏动漫,军事
生活
新闻资讯,美女,游戏动漫,美食,娱乐
穿衣美容
名人明星,文艺
旅游,美女
星座命理,旅游,文艺,新闻资讯,健康
娱乐,美食,唱歌,画画,吃货,自拍,重庆妹儿,夜猫子,90 后非脑残,摩羯座
名人明星
体育
游戏动漫
投资理财
娱乐
搞笑幽默
投资理财,新闻资讯,文艺,体育,军事
美食,自由,90,交朋友,爱玩,冷笑话,幽默,交友,IT 数码
各种爱好
视频音乐
美食,阅读,处女座,旅游,听歌,运动,旅行,电影
文艺,体育,娱乐
娱乐
美女
旅游,美食,健康,投资理财,汽车
微博奇葩,美食,星座命理
IT 数码
情感生活,新闻资讯,美容护肤
娱乐
搞笑幽默
天津生活
新闻趣事,搞笑幽默,名人明星,旅游,视频音乐
体育
时尚
亲情,电子商务,自由职业,好书,美食,时尚,旅游
美女,军事,教育就业,文艺,投资理财

娱乐
时尚,IT 数码,娱乐
八卦杂谈
文艺,空想
文艺
IT 数码,美食,旅游
文艺,旅游,新闻资讯,时尚,娱乐
汽车,体育,IT 数码,A 股,设计,广告,传媒
时尚
娱乐
教育就业
手机达人,旅游
视频音乐,穿衣美容
情绪化,射手女,夜猫子,90 后非脑残,汽车,时尚,投资理财,疯子,2 货,宅女
游戏动漫,军事,娱乐,新闻资讯
新闻资讯,时尚,星座命理,美食,文艺
名人明星
旅游
旅游,美食,搞笑幽默,安徽生活,微博奇葩
娱乐
美食
健康
美食
美女,娱乐
健康,旅游,90 后
游戏动漫,星座命理,IT 数码,文艺
美女,旅游,微博奇葩,搞笑幽默
IT 数码
教育就业,旅游,IT 数码
军事,体育,游戏动漫,IT 数码,汽车
搞笑幽默
美食,娱乐
旅游,新闻资讯,文艺,体育,美食
游戏,吃货
健康,美食,新闻资讯,娱乐,教育就业
旅游,健康
娱乐,井井井井井
文艺
潮,娱乐
文艺,军事,IT 数码,庚澈,朝鲜,小提琴
美女,交友
美食,旅游,体育,娱乐,美女
旅游,星座命理,军事,IT 数码,体育
旅游,美食,娱乐

投资理财
微博奇葩,旅游,新闻趣事,搞笑幽默,萌宠
懒人,疯狂,吃货,90后
搞笑幽默
时尚,健康,旅游,美食,娱乐
IT数码
美女,游戏动漫
萌宠,新闻趣事,美食,旅游,星座命理,开朗，学生，聊天
旅游
汽车
美食
纠结,90后
健康,美女,旅游,美食,星座命理
美女
时尚,文艺,爱笑,我就是我,爱旅行
神经大条,神经质,摩羯座,娱乐,时尚,逛街,淘宝
星座,淘宝,听音乐,美食,平常心,吃货,90后,小说,搞笑,双子座
娱乐
投资理财
新闻资讯,健康,时尚,游戏动漫,旅游,美食,IT数码,娱乐
90后,时尚,小说,天秤座,吃货,校园,音乐,网购
穿衣美容,美食,旅游
美食,旅游,体育
IT数码,新闻资讯,时尚,健康,旅游,军事,体育
天蝎,微博控,爱生活,音乐DJ,美术,电影
文艺,新闻资讯,游戏动漫,美女,娱乐,体育
时尚
美食,旅游,穿衣美容
娱乐
V迷,永不嵩手,嵩鼠,因为有V,音乐纯粹,爱V绝对,Vae,听音乐,许嵩,90后
旅游,娱乐,唱歌,语录,玩儿,媒体,美剧,爱情,美食,听歌
娱乐,投资理财,教育就业,美食
新闻资讯,健康,旅游,星座命理,军事,真实力
娱乐,文艺
新闻资讯
微博奇葩,单身,爱生活
娱乐,美女,IT数码,投资理财,旅游
美食
娱乐
名人明星,美食,搞笑幽默,视频音乐,星座运势
电影
文艺
星座命理,娱乐,体育,IT数码,游戏动漫
美食

体育
星座命理,IT 数码,教育就业
时尚,美女,体育
健康
旅游
生活,搞笑,时尚
汽车,时尚
游戏
投资理财
睡觉
旅游
旅游,好性格,营销,射手座
娱乐
时尚
旅游
名人明星,新闻趣事,旅游,职场招聘,热情,电影,法律,感性,创意
健康,教育就业,新闻资讯,时尚,旅游
美女
教育就业,电脑控
娱乐
视频音乐
汽车
IT 数码,IT,电子商务,手机,数码,篮球,双鱼座
美女,汽车,旅游,美食,体育
90,music,天秤,中医,朋友,美食,旅游摄影,心理,夜猫子
英国生活
军事
搞笑幽默,旅游,美女,广东生活,视频音乐
旅游
美食
八卦杂谈
美女,艺术,生活,上网,旅行
IT 数码
旅游,美女,时尚,汽车,娱乐,宠物,台球,音乐控
体育
追星,腹黑,爱音乐,腐女
IT 数码
手机,大学生,90 后,音乐
IT 数码,设计,网购,看书,运动
健康,美女
美食
IT 数码
新闻趣事
旅游

企业发展,赢利执行模式,总裁培训,企业管理
健康
美食,旅游,健康,汽车,时尚
搞笑幽默
文艺,天蝎女,耽美,游戏动漫,美食
娱乐,文艺
星座命理
甲醇,靖边县,定边县,乌审旗,鄂托克前旗,银川,延安,安塞,吴起,志丹
新闻趣事,旅游,穿衣美容
汽车,时尚,游戏动漫,新闻资讯,美女,投资理财,娱乐,星座命理,美食
美女,旅游,星座命理,美食,娱乐,爱美食,臭美,睡觉睡到自然醒,射手女
投资理财,星座命理,美食,娱乐
穿衣美容
交朋友,时尚,汽车,美女,体育,娱乐
娱乐,时尚,艺术,睡觉,搞笑,音乐,学生,电影,乐观,90 后
游戏动漫,体育
星座命理
文艺,动漫
新闻资讯,时尚,星座命理,美食
IT 数码
微博奇葩,穿衣美容,美食,旅游,文艺,疯子,伪文艺,偶尔情感泛滥
爱情
健康
穿衣美容
新闻资讯,娱乐,文艺,星座命理,美食
文艺,汽车
文艺
军事,IT 数码,体育
星座命理,旅游
文艺,教育就业,健康,旅游,美食
上海生活
体育
娱乐,体育
体育,游戏动漫
体育,IT 数码,美食,时尚
天坪座,跑步
爱吃,美食,音乐,水果,发呆,长发
娱乐
旅游
旅游
娱乐
腐女,旅游,文艺,玩儿,宅女
打篮球,桌球,大学生,生活,笑,坚强,自行车,爱电影,健康,新闻资讯
娱乐

文艺
哲学,吉他弹唱,古典钢琴,桥牌,投资,量子物理,中医,拉丁舞,美食,爵士
90 后,淡定,跑步,喜欢音乐,文艺
体育
旅游,军事,文艺,体育,娱乐
美食
美食,旅游,美女,穿衣美容,微博奇葩
文艺
欧美控,乐天派,双 J 迷,时尚,健康,旅游,美食,文艺
学生,听歌,大学
北京生活
搞笑幽默
宅,低调,文艺,电影,大学生,处女座,睡觉,看书,90 后
文艺,新闻资讯,星座命理
篮球爱好者,体育
创造,书法,喜欢阳光,热爱生活,大一,潮汕人,美食,IT 数码,文艺,体育
申东佑是理想型,郑真英一生 ww,渡边麻友单推
旅游,美食,时尚
美食
娱乐
美食,娱乐
时尚,旅游,新闻资讯,健康,美食
游戏动漫
娱乐
娱乐
跆拳道,愤青,医学,爱生活,情绪化,健康
游戏动漫
体育
起床困难户,双子座
IT 数码,投资理财
娱乐,平常心
名人明星
自由
好性格,幽默,白羊座,90 后
体育
美食,军事,旅游
时尚
搞笑幽默,北京生活
美食,文艺,时尚,教育就业,健康
星座命理,学生
美食
星座命理,投资理财,美女,时尚
旅游,文艺,IT 数码,体育,娱乐
旅行,听歌

健康,星座命理,文艺
星座命理
新闻资讯
连接服务器失败
爬山,皇马,真诚,青春,足球,人生,睡觉睡到自然醒,石头,自由,军事
搞笑幽默
视频音乐
娱乐
美食,健康,旅游,文艺
旅游,美食,汽车
汽车
健康,星座命理,旅游,美食,文艺
拍照,户外,自行车,音乐爱好者,80 后的尾巴,绘画
汽车
文艺
汽车,娱乐,军事
学生一枚,热爱生活,房产,外贸,大学,喜欢听歌,低调,IT 数码,设计,运动
旅游
女人,听音乐,淘宝,开心,爱,喜欢音乐,懒人,双子座,任性,狗狗
体育
军事,新闻资讯,投资理财,旅游
游戏动漫
游戏动漫
90 后,Les,Tina,天秤座,Aomiz,yesorno
旅游,汽车,体育,娱乐,军事
动漫
娱乐
娱乐
失去了！才真痛!
美女
文艺
搞笑幽默,篮球
娱乐
水瓶女,宅女
时尚,动漫,处女座,自由,80 后
星座命理,旅游,健康,美食
旅游,美食,新闻资讯,文艺
娱乐
旅游,新闻资讯,军事,汽车,星座命理
旅游
LAY,伯贤,艺声大本命,EXO
娱乐,文艺,IT 数码,体育,汽车,旅游,健康,新闻资讯,投资理财
美食
游戏动漫,军事,IT 数码

时尚,旅游,IT 数码,文艺,娱乐
宅女一枚,文字控,插画,睡觉睡到自然醒,个性,瘦身,k 歌,旅游,美食,娱乐
新闻资讯,时尚,旅游,美食,文艺,好性格,宅女
娱乐
娱乐
体育
佐鸣王道,瓶邪,发呆,cosplay,美食爱好者,金牛,宅女加腐女,旅游,娱乐,星座命理
强迫症,微博
旅游,投资理财,汽车,时尚,美食
教育就业
游戏动漫,美女
教育就业,汽车,IT 数码
学生,时尚达人,旅游美食爱好者,社会,阿根廷,黑莓,谢娜,林峯
旅游
文艺
体育,篮球
健康,汽车,IT 数码,文艺,美食
爱情,时尚,数码,旅游,听歌,运动,小说,宅
娱乐
健康,新闻资讯,旅游,军事,IT 数码
娱乐
睡懒觉,工作,IT 数码,新闻资讯,教育就业
旅游,新闻资讯,美女,投资理财,健康
教育就业
旅遊
新闻资讯,旅游,IT 数码,健康
美食
美女,体育,军事
体育
美食,微博奇葩,搞笑幽默,安徽生活
美女,汽车,时尚,娱乐,IT 数码
校园,内向,看电视,学生
旅游
体育
旅游,文艺,美食,娱乐,生活,美女,自由,90 后
旅游
军事
街拍控,想当学霸,天然呆
IT 数码
旅游,游戏动漫,IT 数码,娱乐,星座命理
汽车,游戏动漫,IT 数码,文艺,娱乐
IT 数码
星座命理,微博奇葩,宝鸡生活,新闻趣事
搭配控,翡翠控,玉石,和田玉,翡翠珠宝,新闻资讯,文艺

大非洲,随性,笑,自然,90,健康,美食,文艺
美女
低调,90 後
时尚
娱乐
羽毛球,狮子座,宅,平常心,乐观,吃,运动,美食
美食
娱乐
教育就业,健康
IT 数码,星座命理,体育
娱乐,健康,文艺,军人情结,85 后,爱唱歌,音乐,吸血鬼日记,愤青,宅
文艺,旅行,吉他,音乐
美女
娱乐,时尚,游戏动漫
双鱼座,新闻,网购,80 后,电影,阅读,语录,听歌
旅游,军事,IT 数码,体育,娱乐
新闻资讯,旅游,星座命理,投资理财,教育就业
宠物图集,旅游,泉州生活,时尚达人,时尚
腐,欧美剧,游戏动漫
萌宠,美食,睡觉睡到自然醒,90 后,睡觉,吃货,动漫,电影,小说,双鱼座
美食
美食,新闻资讯,旅游,星座命理,时尚,娱乐
娱乐,金牛,V 迷
育儿百科,美容服饰,北京生活,旅游
娱乐,时尚,美女,IT 数码
善变
校园生活,读书分享,美图摄影,法国,猫咪,宅女一枚,懒人,古典艺术,睡懒觉,动漫
自由,新闻,爱笑,绘画,午饭,中医,随性,金牛
时尚
建筑设计,汽车,交友,自由,玩儿,校园,电影,80 后,大学,摄影爱好者
娱乐
BIGBANG,电影,旅游,美食,时尚
汽车,旅游,美女,文艺,娱乐
视频音乐,体育资讯,搞笑幽默,校园生活,西安生活
爱幻想,陈奕迅,摄影,看书,双鱼女,单纯,星座命理,时尚,文艺
体育,娱乐,投资理财,美女,军事
新闻趣事,文艺,IT 数码,健康,教育就业,投资理财
星座命理,志愿者,雙子座男生
娱乐,健康,体育,盖房,篮球
视频音乐,美女,美图摄影,娱乐,好性格
时尚,旅游,健康,美女,体育
美食,体育
IT 数码,体育
梦想,文字控,学习,咖啡,摩羯座,玩儿,文字,手机,心情,90 后但我不脑残

旅游,星座命理,美食,教育就业,文艺
体育,游戏动漫
美食,健康,投资理财,娱乐,时尚
投资理财,旅游,美食,健康,时尚
视频音乐,搞笑幽默,微博奇葩,新闻趣事
平常心
文艺,吃货,Dance,HIPHOP
搞笑幽默
时尚
美女
体育,教育就业,IT 数码,美食
萌宠,羽毛球,动漫,亲情,睡觉,唱歌,射手座,囧,留长发 ing
汽车,旅游,美食,娱乐,体育
体育,时尚,游戏动漫,IT 数码,娱乐
视频音乐
娱乐
IT 数码
军事,娱乐,美女,游戏动漫
旅游,健康
游戏动漫
旅游
爱幻想
旅游,娱乐
偶稀饭睡觉觉,随性,逛街,拍照,爱旅行,怀旧,白羊座,猫控,90,麦霸
偶喜欢玩,读书,大学,互联网,好性格,电影,偶稀饭睡觉觉,90 后
白羊座,星星,神经大条,星座命理
美食
美食
穿衣美容
文艺
新闻趣事,文艺,汽车,娱乐,健康,旅游
健康
星座命理,新闻资讯,健康,旅游,汽车
汽车,教育就业,IT 数码,文艺,娱乐
天蝎,射手,淘宝,唱 k,天秤,心情,家居,Music,娱乐
浪漫,学生,文字,摩羯座,巨蟹座,双鱼座
娱乐
电子商务,英语,80 後,互粉,喜欢听歌,奋斗,吃货一枚,狮子座
美女,摄影,睡觉,宅男
投资理财,体育
星座命理
90,偏执狂,胡思乱想,宅女加腐女,矛盾体
娱乐,健康,美女,广告,新闻,80 后
文艺

健康
汽车,美食
搞笑幽默,美食,旅游,河南生活,微博奇葩
世界杯,德国,怀旧,平常心,善良,爱自由,F1,意大利,爱生活,简单
健康
搞笑幽默
旅游
简单,shopping,互粉,心理学,笑话,音乐控,android,网购控,淘宝控,淘宝
读书分享
互粉吧。亲~
微博
名人明星
美女
文艺,自由,幽默,乒乓球,吉他,音乐,娱乐,体育,上网,旅行
娱乐
娱乐
网购,购物,潮流,时尚,流行,吉他自学,弹指,吉他控,小提琴,音乐迷
美食
娱乐
IT 数码,健康,新闻资讯
新闻资讯,教育就业,星座命理,娱乐,军事
娱乐
美食,IT 数码,时尚,文艺,体育
体育,游戏动漫
体育
美食
汽车,游戏动漫,军事,健康,投资理财,教育就业
数码,爱睡觉,广州,影视,敏感,恐怖片
新闻趣事,旅游,美食,穿衣美容
旅游,美食
搞笑幽默,娱乐,汽车,体育,火影,艾弗森，头文字 D,篮球，dota
投资理财
穿衣美容,教育就业
健康,美食,旅游,时尚,娱乐
名人明星
军事,新闻资讯
健康
军事,美女
娱乐,旅游,体育,美女,投资理财
强迫症
旅游
星座命理,唱歌,吃货
娱乐
文艺

文艺
旅游
美食,IT 数码,时尚,旅游,星座命理
时尚,旅游,文艺,娱乐,IT 数码
吃货一枚,巨蟹,军事,汽车,健康,旅游,美食
双子座,独立,努力,志愿者,赚钱,美食,教育就业,投资理财,时尚,军事
娱乐
奋斗 ing,90 后
游戏动漫,军事,我就是我,个性,手机看小说,搞笑幽默,阅读
山东生活,亲情
旅游,体育,星座命理
健康,时尚
汽车,新闻资讯,游戏动漫,美食,军事
新闻资讯
疯子
新闻趣事,娱乐
新闻资讯,美女,游戏动漫,教育就业,时尚,旅游,IT 数码
名人明星
美食,星座命理,旅游,娱乐
宅女加腐女,韩寒,看小说,夜猫子
娱乐
娱乐,健康
巧克力,动画,囧,水瓶,双重性格,完美主义,瓶邪,宅女加腐女,美食
健康,美女,汽车,军事,IT 数码
IT 数码
娱乐,IT 数码
资讯,投资理财,旅游,美食,体育,娱乐
娱乐,游泳,旅游
奋斗,喜欢听歌,开朗,艺术,善良,bigbang
旅游,汽车,投资理财,时尚
哆啦控
IT 数码
星座命理
新闻趣事
星座命理,汽车
网商,努力,互联网,教育就业,旅游,健康,美食
旅游
文艺,时尚,健康,军事,娱乐
旅游
体育,教育就业
游戏动漫
偶喜欢玩,新闻,艺术,乐观,电影
娱乐
教育就业

军事
搞笑幽默,浙江生活
搞笑幽默
星座命理,美食,娱乐
执着,自由,橙汁,我就是我,吃货,白羊座,旅行,听歌
游戏动漫
足球
教育就业,时尚,游戏动漫,健康
美女,游戏动漫,文艺
时尚,体育,美食,旅游,汽车,美女
新闻资讯,文艺,投资理财,教育就业,IT 数码
新闻资讯,教育就业,星座命理,文艺,健康
健康,旅游,美食,文艺,体育
星座命理
投资理财
时尚
罪心,みうめ女神,ニコニコ動画,全能学习中,宅,隶属翼域友人屋,隶属 LAD 社,隶属羽翼舞团
爱球,爱吃,爱唱歌
美食,娱乐,旅游,时尚,健康
健康,娱乐,模型,看书,动漫,运动,电影,音乐,旅游
健康,美食,时尚,新闻资讯,旅游,文艺
时尚,旅游,星座命理,美食,文艺
美女
穿衣美容
创业,汽车,自由,90 后非脑残,旅游
美女,汽车,游戏动漫,新闻资讯,军事,IT 数码
IT 数码,新闻资讯,健康,美食,娱乐
汽车,IT 数码,旅游
军事,IT 数码
NBA,体育
IT 数码
健康
郑州生活
星座命理,穿衣美容,旅游
旅游
旅游摄影,跑步,健康,新闻资讯,IT 数码,体育
旅游
文艺,娱乐
新闻资讯,健康,星座命理,IT 数码
涂鸦,名人明星,娱乐,90 后,感性
美食,动漫,学生
美食,视频音乐,穿衣美容,旅游,搞笑,活泼开朗,有点傻里傻气,善良,吃货,看书
时尚,投资理财

美女,美食,军事,IT 数码,时尚,娱乐,星座命理,新闻资讯,游戏动漫,文艺
娱乐
娱乐,认真负责,喜欢听歌,文艺
教育就业,美女,投资理财,健康,IT 数码
健康,文艺,美女,娱乐
美女
美女,体育,学习永无止境
军事
美食
旅游
旅游,美食,文艺,好性格,动漫,音乐
新闻趣事
体育
星座命理,新闻趣事,视频音乐,美食,旅游
文艺,汽车,时尚,IT 数码,娱乐
听歌
汽车,体育
睡觉睡到自然醒,小说,懒,喜欢唱歌,笑,听音乐,星座命理
美食,教育就业
娱乐,美女,教育就业,新闻资讯,体育
星座命理,健康,旅游
享受生活
娱乐
娱乐,游戏,巨蟹座,90 后,仙后
健康,文艺,时尚
美食,搞笑幽默,旅游,穿衣美容,美女
健康,新闻资讯,文艺,投资理财,旅游,星座命理
汽车,教育就业,游戏动漫,时尚,星座命理
时尚,军事
军事,美食,汽车,游戏动漫,体育
新闻,自由职业
美女,文艺,体育,娱乐
易建联死忠,中国男篮,易建联,外貌协会,90 後,娱乐,体育
娱乐
宠物图集
星座命理
星座命理
个股
美女
旅游
B 型血,内向,宅女一枚,天秤座,听音乐,85 后,多愁善感,旅游美食,海贼王
汽车,娱乐,时尚
体育资讯
投资理财,新闻资讯,游戏动漫,军事,IT 数码

娱乐,文艺
游戏动漫
微博奇葩,健康,娱乐
健康,汽车
美食
和谐，健康即美
搞笑幽默,那些年,对不起!
视频音乐,美食,新闻趣事,旅游,穿衣美容
旅游,娱乐
新闻趣事,视频音乐,旅游,微博奇葩
游戏动漫
IT 数码
读书分享,宠物图集,新闻趣事
美女,摄影,学生,旅行,音乐爱好者,蔡依林
IT 数码
文艺
健康,教育就业,文艺,体育,娱乐
汽车
旅游,美食,军事,娱乐,体育
新闻趣事,美图摄影,旅游,动漫,搞笑幽默,吃
文艺
听歌,狮子座,80 后
娱乐,文艺,美食
健康
美食,穿衣美容,微博奇葩,搞笑幽默,贵州生活
健康
旅游
时尚,新闻资讯,旅游,星座命理,美食,文艺,娱乐
IT 数码,投资理财,健康,旅游,军事
体育,游戏动漫,旅游,IT 数码,文艺
娱乐
开朗女生,爱广播,爱音乐
市藏,哔哩哔哩,UTAU
旅游,新闻资讯,时尚,娱乐
体育
汽车,娱乐
健康,时尚,旅游,IT 数码,文艺
娱乐,健康
IT 数码
进城务工,逛街男,庄主,前现代
游戏动漫,IT 数码
杭州生活
美食
时尚

娱乐
个性,摄影师,shopping,美食,时尚,文艺,体育,娱乐
美食,军事,体育,娱乐
星座命理
巨蟹
偶稀饭睡觉觉,听歌,囧,唱K,宅女,90后,吃
美女,旅游,美食,IT数码,娱乐
娱乐,时尚,旅游,美食
美食
文艺,体育
旅游
娱乐
星座命理
旅游,美食,星座命理,文艺,娱乐
娱乐
IT数码,健康,新闻资讯,美女,娱乐
体育
萌宠
游戏动漫,时尚,文艺,娱乐,美食
金牛座,爱贴吧,爱祖国,团员,吧主,发呆,逆袭,励志帝,美术生,小愤青
黑白控,摇滚乐,恐怖电影,大爱五月天,猫咪控,动漫控,喜欢音乐
微博奇葩
微博奇葩,美女,陕西生活,新闻趣事
星座命理
旅游,星座命理,军事,IT数码,娱乐
娱乐,投资理财,旅游,美食,文艺,健康
美食,学生,睡觉,巨蟹座
看书
美食,汽车,投资理财,健康,IT数码
美食,美图,意大利,汽车
校园,潮流,金在中,香橙,意大利,单纯,葡萄牙
上网不知要干啥,超级无敌宅,游戏达人,唱K
美食,旅游,教育就业,IT数码,娱乐
娱乐
星座命理,美女,时尚,娱乐,爱武艺
汽车,娱乐,IT数码,游戏动漫
娱乐
搞笑幽默,新闻趣事,视频音乐,八卦杂谈,美女,90宅男一枚
旅行,财经,美女,历史,旅游,运动,好性格,天秤座,篮球,80后
旅游
动漫
体育
美食
旅游,淘宝网店铺童装,动漫迷,亲子育儿,追韩剧,美剧控,好吃,80后妈妈,鑫宝萌主帮

游戏动漫,娱乐,旅游,文艺,IT 数码
开朗,发呆,文艺,旅游,美食,健康,美女,时尚,投资理财
重庆生活
80 后
穿衣美容
健康,旅游,时尚
教育就业
穿衣美容
投资理财,健康,汽车,旅游,美食,90 后但我不脑残
旅游,文艺,美食
娱乐
90 後,单身,nba,北京,体育
投资理财
旅游,美食,文艺,时尚,美女
娱乐
教育就业,健康
穿衣美容,美食,射手座,大学生,化妆,k 歌,吃货,幽默,感性
学生,玩儿,睡觉,电影,大学,幽默,水瓶座,浪漫,美食
搞笑幽默,微博奇葩,体育
IT 数码
美容服饰,网购,吃货,狮子座,神经质
汽车,篮球,巨蟹座,微博,运动,电影,摄影,动漫,自由
时装,电影迷,创意,90,欧美控,狗狗,交友,幽默,娱乐,唱歌
娱乐,IT 数码,游戏动漫
娱乐,健康,时尚,汽车,体育,美食
IT 数码
IT 数码,文艺,军事,体育,娱乐,休闲游戏,打工一族,宅
爱情,浪漫,平常心,80 后
美女,汽车
魔兽,时尚,娱乐,汽车,游戏动漫,美女
IT 数码,美食
视频音乐,其他生活,搞笑幽默,新闻趣事,穿衣美容
体育
汽车,健康,IT 数码,娱乐
美食
白羊座,微博奇葩
IT 数码
新闻趣事,旅游,视频音乐,校园生活
体育
军事
军事
投资理财,旅游,健康,美食,娱乐
陈奕迅,天秤座,80 后,唱歌,宅男
美食

新闻趣事,星座运势,健康养生,汽车,军事,IT 数码,旅游,星座命理
淘宝女装掌柜,平常心
文艺,春心荡漾,喜欢玩,旅游
娱乐
游戏动漫
美食,娱乐
娱乐
天秤座,江阴,保健,双鱼座,建材,骑车,财会
新闻资讯,汽车,旅游,IT 数码,体育
新闻资讯,健康,旅游
文艺
美女,旅游,军事,投资理财,教育就业
文艺
体育,IT 数码
新闻资讯,IT 数码
IT 数码,美食,旅游
旅游
文艺青年
体育
其他生活
新闻趣事,视频音乐,美食,旅游,穿衣美容
健康,旅游,星座命理,美食
健康,新闻资讯,汽车
娱乐
幽默,天蝎座,90 后一追求淡定,文字,紫色,大学,网络,上网,睡觉,动漫
投资理财,营销分析,营销策划,微博营销,SEM,社会媒体化营销,SEO,网络营销,电子商务,互联网
节奏控,美食,时尚,娱乐,汽车
美食,新闻趣事,视频音乐,校园生活,搞笑幽默
镇江生活
时尚,健康,旅游,美食,娱乐
娱乐,旅游,美食
美女
美女
娱乐
微博奇葩,想减肥的吃货,狮子座,张根硕
文艺,娱乐
时尚,美食,投资理财,美女,汽车
娱乐
美食,健康,旅游,汽车
IT 数码,美食,手机,微博,美女,游戏,校园,爱情,宅女,动漫
搞笑幽默
娱乐
娱乐

娱乐,旅游,美食
重庆生活,校园生活,美食
创意,毅力,旅游,创业,亲情,80 后,篮球,平常心,水瓶座,自由
体育,星座命理
娱乐,汽车,时尚,游戏动漫,旅游
汽车
新闻,互粉,微博控,网络
cosplay,游戏动漫,星座命理,美食,娱乐,吸血鬼控,咬咬
星座命理,汽车,娱乐,教育就业,体育
宅女加腐女,火影迷,回笼觉,耽美狼,愚人
旅游
军事
美女,汽车,文艺,娱乐,旅游
浙江生活
军事,健康,新闻资讯,投资理财
时尚,新闻资讯,简单,爱玩
穿衣美容,郑州生活,旅游,健康,星座命理,美食,体育,娱乐
军事,时尚,汽车,旅游,文艺
美食,时尚,旅游,文艺,健康
投资理财,时尚,旅游,美食,文艺
教育就业,美女,健康,美食,投资理财
郑州生活,新闻趣事,微博奇葩,搞笑幽默,美女
健康,新闻,传媒,音乐,宅女,平常心,校园,善良
游戏动漫,IT 数码,文艺,美食,娱乐
金毛,美女
投资理财,星座命理,娱乐,文艺,IT 数码
健康
娱乐
汲取正能量,看新闻非说点啥,爱中医中药,植物控,文字控
时尚,军事,文艺
健康,历史,搞笑,幽默,睡觉,文字,动漫
旅游,娱乐,美女,时尚,美食,摩羯座,少女时代
美女
美食,教育就业,IT 数码,新闻资讯,投资理财
美食,娱乐,IT 数码,旅游,汽车,巨蟹座
娱乐
美食,旅游
旅游,美食,萌宠,搞笑幽默,穿衣美容
IT 数码
旅游
乐观,美食,运动,篮球
汽车,快乐,单身,奋斗
IT 数码,娱乐
旅游,美食,IT 数码,体育,娱乐

青春,矛盾体,巨蟹女,真诚,交朋友,外贸,工作
IT 数码
时尚,教育就业,汽车,军事,娱乐,体育
宜搜,手机搜索,移动顶告
时尚,文艺,新闻资讯,健康,美食,娱乐
时尚
搞笑幽默
IT 数码,教育就业,健康,娱乐,新闻资讯,美食
教育就业,旅游,星座命理
时尚
体育
健康,旅游,汽车,军事
传媒,美食
我就是我,投资理财
微博奇葩,美食,搞笑幽默,穿衣美容,演员,吃喝玩乐
美食,健康,旅游,娱乐,水瓶女
文艺,时尚,健康,美女,体育
美剧,80 后,音乐,浪漫,天蝎座,文学,爱情,偶喜欢玩,美女,天秤座
萌宠,搞笑幽默
微博奇葩,穿衣美容
IT 数码,汽车,星座命理
娱乐,美女,IT 数码,游戏动漫
文艺,军事,星座命理,IT 数码,体育
美食
美食
90 後
军事
体育
星座命理,娱乐,喜欢音乐,狮子座
旅游,星座命理,美食,娱乐
旅游,娱乐,新闻资讯,游戏动漫,教育就业
健康,投资理财,体育,教育就业
俄罗斯生活,IT 数码
music,shopping,乐天派,努力,美食,娱乐
汽车,旅游,美食,体育
原创文学,大学生创作平台
IT 数码
娱乐,动漫
巨蟹座,80 后,吃货
娱乐,旅游
时尚摄影师,淘宝摄影,时尚,艺术,摄影
萌宠,视频音乐,美食,旅游,搞笑幽默
旅游
文艺

旅行,电影,爱俊杰,美食,音乐
汽车
时尚,投资理财,娱乐,IT 数码,汽车
教育就业,健康,旅游,文艺,星座命理
相声,90 后非脑残,偏执狂,处女座,上海生活,摄影控,怀旧,周杰伦,海贼王,胡思乱想
军事,IT 数码,娱乐,投资理财,文艺,体育,汽车
体育
旅游,星座命理
美女
wow,很宅的 80 后,地产设计,品牌设计,文艺,娱乐,健康
美图摄影
娱乐,美女,游戏动漫
新闻资讯,星座命理,旅游,娱乐,文艺
新闻趣事,健康,投资理财
视频音乐
星座命理,游戏动漫
烦恼抛脑后
游戏动漫,美食
美女,美食,IT 数码,文艺,娱乐
IT 数码
SEM,火影忍者,上网
陇南生活,校园生活,新闻趣事,美食,名人明星
搞笑幽默
射手座
美女,健康,娱乐
新闻资讯,文艺,体育,娱乐,健康
其他生活,旅游,视频音乐
美图摄影
穿衣美容
时尚,美女,新闻资讯,健康,美食
韩剧,90 后非脑残,化妆品,韩国,健康,摩羯座 B 型血
新闻资讯,美女,IT 数码,娱乐,星座命理
拍照,平常心,射手座
时尚,健康,文艺,美食,旅游
旅游,星座命理,美食,皮卡丘,微博控,法语,英剧,美剧,Pchy,林宥嘉
旅游,新闻资讯,体育
美女,美食,时尚
测绘教育工作者
旅游,IT 数码,健康,投资理财,娱乐,美女,新闻资讯,教育就业
军事,传媒,网络
美食
美食
古诗词,美食,微博奇葩,95 后,大大咧咧,心无城府,古典文学,自由职业,天秤座
旅游,体育

日本,love,摩羯,i,追星,性感,jyj,怀旧
动漫,宠物图集,星座命理,游戏动漫
張惠妹,重口味,活泼开朗,学生,双鱼座,iphone,笔亲,爱音乐,IT 数码
游戏动漫
文艺
江苏生活,爱情,摄影爱好者,羽毛球,乐观
投资理财,新闻资讯,美食,星座命理
美食
爱吃,学生一枚,活泼开朗,90 後,睡觉睡到自然醒,爱旅行,o 型血
时尚
体育,IT 数码,军事
时尚
听歌,95 后,半宅,天蝎女,校园
时尚
教育就业
文艺,星座命理
校园
文艺,娱乐
美食,星座命理,健康,旅游,时尚
美女,重庆生活,偶喜欢玩
娱乐,旅游
善良,旅游,平常心,电影,音乐,紫色,手机
游戏动漫,体育
健康,投资理财,旅游,星座命理,娱乐
刘心,欧美,刘同,李程远,90 后非脑残,幻想,上海,电影迷,音乐迷,韩庚
电影,音乐,旅游,历史,幽默,财经,运动,A 股,好书,狮子座
名人明星,美女,视频音乐,星座命理,搞笑幽默,宅,音乐,娱乐,小说
大学,旅行,音乐,偶稀饭睡觉觉,八卦,唱歌,英语,小说,自由职业
萌宠,龙宝儿爸,龙宝儿妈
游戏动漫,体育,娱乐
自由,天蝎座,les,acg,心理,魏晨,人生
听音乐,四川生活,囧,互联网,紫色,文字
教育就业
投资理财,体育
美图摄影
健康
IT 数码,体育,星座命理,美食
美女,汽车,IT 数码,体育,娱乐
游戏动漫,体育,娱乐,美女
听音乐,画画,运动
游戏动漫,IT 数码
IT 数码
美食,创业,文学,NBA,白羊座,浪漫,八卦,手机,玩儿,数码
旅游

名人明星,美食,旅游,微博奇葩,新闻趣事
娱乐
体育
美食,星座命理,健康,时尚
新闻资讯,旅游,美女,时尚,娱乐
湖州生活,吃货
旅游
美女
娱乐
时尚,星座命理,健康,娱乐
游戏动漫,IT 数码,娱乐
毕业 ing,找工作 ing,篮球,中医药,美剧,音乐,医疗卫生,CRA,生物制药
娱乐,小说,幽默,音乐,旅行,文字,动漫
娱乐,体育
视频音乐
情感生活
名人明星
文艺,教育就业,投资理财,美食,IT 数码,军事
视频音乐,新闻趣事,穿衣美容,星座命理,美食
美女,美食,美食,旅游,时尚,游戏动漫,娱乐
心理,高中生,生活,平常心,自由,旅游,爱摄影,处女座,学生族,爱自然
美食,娱乐
时尚,美食,摄影,音乐
名人明星
宅女一枚,小清新,动漫控,文藝,萌宠,微博奇葩,湖南生活,美女,穿衣美容
感性,爱旅游,单身,五月天,阳光
文艺,美食,旅游,健康,时尚
旅游
娱乐,英语,电影,旅行
旅游
游戏动漫,美女,汽车,美食,体育
自由,宅,摩羯座,时尚,宅女,美食,上网,小说,90 后,阅读
游戏动漫
娱乐
上海生活
IT 数码
旅游
星座命理,美食,军事
文艺
美食,娱乐
游戏动漫,旅游
美女,旅游,汽车,IT 数码,游戏动漫
时尚,IT,电子商务,网络,90 后,旅行
搞笑幽默

开朗,新闻趣事
娱乐
旅游
移动办公,OA,协同管理,办公自动化
文艺,90 后,奋斗
视频音乐
视频音乐
餐饮管理策划,广告标识工程,美国美安产品,艺术培训,古琴制作,传媒
投资理财
加油,努力
文艺,广告,动漫迷,文字,上网
游戏动漫,娱乐
娱乐,篮球,写作,双鱼座,唱歌,看书,美食,80 后
军事,投资理财
汽车,教育就业,投资理财,IT 数码,文艺
娱乐,时尚,健康,旅游,美食,电影,80 后,读书
大学,爱自由
美食,超喜欢指甲油,摄影控
善良
汽车,美女
时尚
新闻资讯,文艺
美食,新闻趣事,旅游,广东生活,健康
娱乐
旅游,星座命理,微博奇葩,搞笑幽默,名人明星
新闻资讯
汽车,新闻资讯,健康,IT 数码,美食
旅游,美食,体育
教育就业,旅游,美食,IT 数码,娱乐,美女
微博奇葩
游戏动漫
时尚
古典
投资理财,时尚,娱乐,体育,IT 数码
美食
旅游,美食,娱乐,投资理财,美女
美食,穿衣美容,星座命理,江苏生活,微博奇葩
投资理财
时尚,星座命理,美女,旅游,娱乐
时尚,文艺,美食,娱乐
时尚,游戏动漫,军事,旅游,健康
娱乐
名人明星
娱乐,游戏动漫,文艺,投资理财,教育就业,健康

视频音乐,新闻趣事,江苏生活,时尚,旅游,健康
体育,呦呦奶茶
酒店预订,旅游顾问
时尚
旅游,美食
娱乐,唱歌,跳舞,爱鹿晗,巨蟹座,爱唱歌,爱跳舞,90 后
娱乐,时尚,星座命理,旅游
音乐,扯淡的态度!
时尚,娱乐,星座命理,旅游,美食
旅游,体育,文艺,时尚,汽车,军事,IT 数码
旅游,文艺,娱乐
美食
搞笑幽默,海贼王,听音乐,宅,动漫
美女,旅游,军事,娱乐,体育
IT 数码,汽车,旅游
旅游,看书
娱乐
偶稀饭睡觉觉
旅游
教育就业,文艺,健康,旅游,美食,营销
Smile
体育
军事,IT 数码,体育,娱乐
体育
娱乐,健康
漂一代,追梦人,操卖白粉的心,拿卖白菜的钱,80 后,淘宝
健康,投资理财,旅游,美食,文艺
八卦杂谈,大连生活,搞笑幽默,校园生活,听歌
穿衣美容
名人明星,90 后,水瓶座,文艺青年
文艺,娱乐
旅游,萌宠,新闻资讯,教育就业
时尚,旅游,新闻资讯,IT 数码,投资理财
娱乐,美食
IT 数码,投资理财
旅游,星座命理,美食,天秤座,80 后,听歌,玩儿,爱情,唱歌
美女
综合,服饰,生活,时尚潮流,购物,美容
电影
微博奇葩,搞笑幽默
教育就业,新闻资讯,旅游,军事,体育
游戏动漫,美食,时尚,投资理财,星座命理,文艺,娱乐
体育
新闻资讯

教育就业
健康,体育,娱乐,美女,游戏动漫,军事
山东生活,视频音乐,美食,旅游
新闻资讯,健康,旅游,星座命理,美食
搞笑幽默,投资理财,时尚
教育就业
娱乐
投资理财,汽车,游戏动漫
IT 数码
娱乐,新闻资讯,投资理财,汽车,IT 数码
星座命理,健康,旅游,体育,娱乐,美食
身后基佬一大群,哈士奇控,搞笑幽默
美食,健康,吃货,博学,理性
军事
穿衣美容
军事
新闻趣事
娱乐,汽车
健康,时尚,美食,娱乐
娱乐,美食,文艺
投资理财,时尚,美食,我是吃货,吃
体育
美女
旅游
新闻资讯,教育就业,星座命理,IT 数码,体育,90 后
旅游,美食,军事,体育
平常心,双子座,偶稀饭睡觉觉
游戏动漫,美女
娱乐,游戏动漫
双鱼座
湖北生活,穿衣美容
健康,新闻资讯,教育就业,投资理财,旅游,娱乐,IT 数码,文艺,体育
爱唱歌,布丁,周杰伦,搞笑,强迫症,摩羯女,腐女,星座,看电影,海贼王
文艺,娱乐,教育就业,旅游
90 后,摩羯座,外向,爱玩,学生。
广西生活
睡觉,微博,小说,90 后,好性格,双鱼座,吃货,电影
美女
美食,文艺,教育就业,娱乐,喜欢看小说,喜欢睡觉和做梦,旅游
健康,旅游,星座命理
投资理财
85 后,网球,孙燕姿,许嵩,神经大条,厦门,表演,卡卡,自然
星座命理
美女,投资理财,美食,美食爱好者,怀旧,星座命理

美食
旅游
新闻资讯
重庆,淘宝,旅游
名人明星
广东生活
书店,旅行,戏剧表演,音乐,料理,园艺花草,摄影,美食
123
新闻趣事,星座命理
旅游
名人明星
教育就业,美食
投资理财,汽车,娱乐,IT 数码
旅游,美食,娱乐,射手座,吃货,睡觉,好性格,旅行,搞笑
娱乐
娱乐
奥明曼福,企业标识,橱窗设计,活动策划,Agency,Event,开业典礼,时尚与奢侈品,福建公关,OMF
体育
星座命理,投资理财,美食,IT 数码
神经质,浪漫,新闻资讯,文艺,健康,旅游,喜欢音乐
美女,旅游,文艺,娱乐,美食
美女,美食,文艺,时尚,娱乐
偶稀饭睡觉觉,大学生,宅女,学生
文艺
资讯,赛车,星座命理,穿衣美容,美女,新闻趣事,搞笑幽默
投资理财
娱乐,新闻资讯
美女,八卦杂谈,美图摄影,IT 数码,名人明星
健康
穿衣美容
体育,娱乐,美食,文艺,美女,单身求解脱
亲情
娱乐,IT 数码
英國,旅行,电影
微博奇葩,视频音乐,名人明星,旅游,其他生活
美食
IT 数码
旅游,时尚
這個是小號,辛賈一生推,賈法爾俺嫁
美食,娱乐
娱乐,自由职业
体育
投资理财,体育,游戏动漫

努力 ing,宅,阅读,巨蟹座,吃货,90 后,听歌,睡觉
文艺
视频音乐
体育
IT 数码
微博奇葩,没有什么爱好,,
体育
服装面料
听歌,搞笑,动漫
新闻资讯
上海生活
动漫,音乐
体育
体育,天蝎座
娱乐
文艺,健康,新闻资讯,美女,幽默,小说,网络,游戏,音乐,媒体
八卦杂谈
時尚,美食,星座命理,旅遊,汽車
IT 数码,旅游,汽车,游戏动漫
娱乐
教育就业,时尚,星座命理,美食,娱乐
旅游
时尚,美食,新闻资讯,健康,娱乐
文艺,吃货,许嵩 vae,摩羯座,旅行
美女
娱乐
体育
美食,娱乐,汽车
教育就业,旅游,军事,文艺,体育
表演,插画,欧美,幽默,Music,涂鸦,独立,思考,演员,美术
美女
宅,上网,动漫,微博,小说
美女,时尚,唱歌
星座命理
美食
佛冈奇闻趣事,佛冈人,佛冈新闻,佛冈新鲜事
美食,健康,时尚,星座命理,娱乐
娱乐,热爱电视剧,90 后一追求淡定,摩羯座
80 后,时尚
爱自由,奋斗,睡觉,烹饪爱好者,射手座,90 後
娱乐,美女
旅游,搞笑幽默,湖北生活
文艺
投资理财,美食

治愈系,文字,控,潮牌,旅行,美食,时尚,嘻哈,街舞,朋克
搞笑幽默
宠物图集
交友,搞笑,幽默,足球,篮球,旅行,生活
新闻资讯,IT 数码,美女,游戏动漫,娱乐
美食
时装,美容,购物,拍照,90,公务员,旅游
美食,星座命理,健康,美女,时尚
射手座,电子商务,乐观,90 后
美女,游戏动漫,汽车,娱乐,IT 数码,大学
美食,时尚,性格活泼开朗,喜欢听音乐,Licky,Digens
娱乐,游戏动漫,美女
旅游,搞笑幽默,星座命理,微博奇葩
文艺,运动,爱玩,动漫迷
文艺
体育,娱乐
投资理财
时尚,搞笑
娱乐,时尚,体育,美食,旅游
娱乐
美食
时尚,新闻资讯,旅游,美食,娱乐
时尚
新闻资讯,游戏,运动,爱电影,网球
学生,阅读,睡觉,90 后,宅女,动漫,紫色,偶喜欢玩
搞笑幽默,娱乐,健康
星座命理
投资理财,旅游,星座命理,IT 数码,时尚
旅游,温柔，旅游，普工
娱乐
生活,笑话,高考,我就是我
时尚
金牛
旅游
活泼,阳光,90 后非脑残,交友,幽默,热爱生活,喜欢唱歌,微笑,喜欢听歌,游戏动漫
爱美食,爱摄影,爱自己
美食,穿衣美容,星座命理,微博奇葩,搞笑幽默
文艺
体育
囧
旅游
时尚,教育就业,投资理财,健康,娱乐
山东生活
投资理财

睡懒觉,星星,怀旧,做梦,散步,动画,星座命理
娱乐,旅游,文艺,星座命理
美食,旅游,游戏动漫,军事,投资理财,文艺,娱乐
河北生活
投资理财
视频音乐
IT 数码
军事,投资理财,汽车,娱乐,美女
赣州生活,7239
旅游
健康,旅游,IT 数码,娱乐,文艺,星座命理
天秤座 B 型女
投资理财
名人明星,腐女,无厘头,疯狂,正太控,矛盾体,爱吃
教育就业
米修
军事,新闻资讯,高考,旅游美食,手工艺品,丝带绣,时尚,馨艺手工坊
旅游,剑三,交友,大学生,宅
旅游
娱乐
旅游,星座命理,美食,文艺,娱乐
游戏动漫,娱乐
旅游,IT 数码,外向,上网
90 后非脑残,学生一族,神经大条
军事,美女,娱乐
新闻资讯,美女,游戏动漫,军事,IT 数码,IT,80 后
时尚潮流,时装秀场,高级定制,购物,奢侈,潮流,设计,搭配,服饰
美女,穿衣美容,时尚,旅游,美食
时尚,娱乐,健康
新闻趣事,健康,文艺,时尚,娱乐
教育就业
星座命理
美女
星座命理
体育
美食,新闻资讯,健康,旅游,投资理财
文艺,体育,时尚,美食,娱乐
教育就业
交友,爱情,80 后
旅游,健康,投资理财,美食,娱乐
娱乐,美食,时尚
旅游
IT 数码,体育,美女,汽车,军事
肿瘤,外科,肺,食管,纵隔,胸腔镜

搞笑幽默
美食,时尚,娱乐
我的秘密花园
艺术,吃,摄影,时尚,听歌
微博奇葩
爱自由,好性格,足球,巨蟹座,诗歌,打篮球,朋友,人生,旅游爱好者,影视
娱乐
睡觉,天蝎座,乐观,吃,学生,看书,好性格,听歌
美食
萌宠,湖北生活
旅游
游戏动漫
感性,孩子气,狗狗,开朗,逛街,音乐爱好者,喜欢听歌,淘宝控
星座命理,美食,娱乐,文艺,游戏动漫
风景摄影,风景,宁静,自由,平凡,爱情公寓,海贼王,旅游爱好者
电影
乐观,美食,数码,旅游,微博,90 后
美食
体育
白羊座,平常心,善良,乐观,听歌,自由
视频音乐,自拍狂
伪环保主义者,伪愤青
美女,游戏动漫,时尚,健康,军事,体育
梦想,足球,90
粤语,体育
美女,小说
网络,篮球,旅游,摄影,幽默,新闻,射手座,生活,运动,电影
旅游
文艺,旅游
80 后,摩羯座,电影
体育
新闻资讯,教育就业,旅游,美食,IT 数码
80 后的尾巴,仓鼠控,手机,双重性格
美女
美食
娱乐
教育就业
娱乐
文艺,体育,娱乐
汽车,体育
福尔摩斯,樱井孝宏
体育,文艺
美食,时尚,新闻资讯,健康,文艺
娱乐

星座命理
美女,体育,游戏动漫,爱电影
其他生活
电视剧,旅游
娱乐,时尚
投资理财,汽车,新闻资讯,旅游,军事
美女,美食,IT 数码,娱乐,游戏动漫
扬州生活,名人明星,美食,旅游,新闻趣事
汽车,时尚,旅游,美食,IT 数码
星座命理,美女,新闻趣事,搞笑幽默,深圳生活
新闻趣事,星座命理,娱乐
IT 數碼
90 后,玩儿,交友
娱乐
文艺,星座命理,美女
旅游,星座命理,美食
处女座
新闻资讯,军事
宅
盗墓笔记
80 后,电影,动漫,狮子座,财经,汽车,运动,电子商务,游戏,手机
娱乐,时尚,美女,健康,星座命理
亲情,校园,文艺
娱乐,IT 数码
美女,娱乐,浪漫,平常心,看书,睡觉,双鱼座,亲情
健康,汽车,军事,体育
军事,游戏动漫,新闻资讯,美女,美食
时尚
投资理财
旅游
健康,体育
旅游,美食,娱乐,健康,文艺
娱乐
视频音乐,美食,旅游,微博奇葩
美食
大学生
体育
教育就業,旅遊,美食,軍事,IT 數碼
星座命理
旅游
美食,旅游,搞笑幽默,潍坊生活
星座命理
交友
美女

体育
爱龟龟想养龟
文艺,体育,娱乐,旅游,游戏动漫
八卦杂谈
汽车,美女,健康,星座命理,美食
IT,动漫
旅游
娱乐
唱歌,天秤座,80 后,玩儿,游戏,体育,吃货,睡觉,网络,亲情
美女,娱乐
漫步者
新闻资讯,时尚,投资理财,娱乐,IT 数码
星座命理,时尚,健康,美食,娱乐
交友,乐观,学生,娱乐,摩羯座,90 后
体育,游戏动漫
旅游
娱乐,星座命理,健康,时尚
旅游,美食,北京生活,萌宠
名人明星
时尚
美食
时尚
旅游
美食
搞笑幽默
药药药,拖延症患者,強迫症患者,摩羯座
时尚,旅游,美食,文艺,娱乐
时尚,美女,IT 数码,文艺,娱乐,美食,投资理财,健康
13474657284
星座命理,美食,旅游,健康
教育就业
投资理财,旅游,美食
汽车
视频音乐,新闻趣事,搞笑幽默,新疆生活,投资理财,教育就业
健康,美女
健康
美女,文艺,游戏动漫
时尚,美食,星座命理,旅游,娱乐
旅游,汽车,IT 数码,文艺,体育
旅游,星座命理,文艺,教育就业,娱乐
美女,IT 数码,时尚,旅游,星座命理,文艺,娱乐
娱乐,鹿晗
美食,时尚,学生
体育

旅游
幽默
名人明星,美女
美女,微博奇葩
娱乐,上网,学生,美食控,手机,微博,唱 K,学生一枚,电视,杂志
游戏动漫,IT 数码,健康,旅游,美女
读书分享,宠物图集,搞笑幽默,美食
搞笑幽默
体育
星座命理,动漫,电影,宅,游戏,天蝎座
新闻趣事,IT 数码
美女,新闻趣事,合肥生活
校园生活
时尚,美食,逛街,睡懒觉,腐女,看电影,星座,80 后的尾巴,淘宝控,外貌协会
军事
学生,吃货
游戏动漫,文艺
江苏生活,汽车
美食,旅游,星座命理,IT 数码
星座运势,美食,重口味,情感生活,搞笑幽默
投资理财,旅游,古典音乐,好书,狮子女,爱自由,旅游摄影,电影,爱情,运动
文化,笑话,新闻资讯,健康,文艺,历史,文学,羽毛球,乐观,80 后
自拍,快乐无忧
浪漫,美食爱好者,时尚,双子座,自由,学生,偶稀饭睡觉觉,八卦,音乐,电影
投资理财,时尚
搞笑幽默
IT 数码,汽车,天枰座
娱乐
足球,台球,娱乐
游戏动漫,爱自由,天秤,阅读,冷笑话,睡懒觉,沉默,散步,喜欢听歌,潮人
Metersbonwe,PHANTACi,林峯,Chok,布丁,杰迷,jay 迷,90 后,双子座,周杰倫
新闻资讯,时尚
情感,自由,学生,上网,网络,宅男,无敌丑男,囧,宅,巨蟹座
新闻趣事
娱乐,IT 数码,美女,时尚,游戏动漫
游戏动漫
体育,娱乐,文艺,军事
户外,绘画,音乐爱好者,文艺
美图摄影
旅游,麦霸,动漫控,坚强,自行车
游戏动漫
美食,时尚,娱乐
开心,80 后的尾巴,梦想,休闲,简单,神经大条,钓鱼,减肥,爱睡觉,娱乐
娱乐

美食
娱乐
吃
时尚,IT 数码,健康,体育,娱乐
美女
旅游,游戏动漫,健康
娱乐,商业演出,庆典礼仪,节目创编,开盘奠基,招商订货会,婚礼策划,广告制作,会议会展,礼品定制
舞蹈,健康,可爱,歌手,交朋友,音乐控,魔兽世界,交友,幽默,80 后
85 后,网络,体育,Music,唱 K,逛街,音乐,篮球,看书,天秤座
微博控,星座命理,宅女,娱乐,手机,上网,艺术,唱歌,游戏,双子座
星座命理,娱乐
游戏动漫,旅游,星座命理,IT 数码,娱乐,自由职业,自由,音乐
搞笑幽默,娱乐
军事,IT 数码,美女,投资理财,汽车
体育,游戏动漫,娱乐
紫色,听歌,小说,吃,上网,睡觉
交朋友,移动互联网,市场营销,互粉,腐女,文艺,室内设计,网络营销,时尚潮流,互联网
娱乐
军事天地,搞笑幽默,IT 数码,八卦杂谈,90 後,活泼开朗,DOTA,平常心,网购,善良
美女
星座命理,健康,旅游,娱乐
狗狗,美食
投资理财,时尚,健康,旅游,娱乐
游戏动漫,IT 数码,军事
微博奇葩,浙江生活
健康,文艺,娱乐
娱乐
大学,电影,旅行,宅女,吃货,大学生,搞笑,美剧,交友,紫色
新闻趣事,搞笑幽默,视频音乐,星座命理,旅游
成都生活
文艺,娱乐,游戏动漫,星座命理,军事
美女,投资理财,汽车,娱乐,体育
文艺,时尚,旅游,美食
文字,摩羯座,微博,网购,双鱼座,巨蟹座,学生
发笑图片,搞笑影像,专题消息,明星偶像,时尚潮流,美容美体护肤,饮食健康,韩国整形,世界旅行
美食
娱乐
新闻趣事,视频音乐,搞笑幽默,美女,新闻资讯,游戏动漫,健康,娱乐
体育
文艺,旅游,星座命理,时尚,娱乐
文艺,娱乐
媒体,宅女,网购,80 后,上网,天秤座

美女
星座命理,美女,美食,娱乐,时尚,旅游
时尚,电影迷,爱玩,爱美食,美食
体育,旅游,美食
健康,娱乐,星座命理,旅游,美食
旅游
搞笑幽默,新闻趣事,星座命理,香港生活
旅游
旅游,读书分享,宠物图集,美图摄影,紫色,交友,上网,汽车,时尚
文艺
旅游
文艺,旅游,投资理财
彩妆,婚纱,香薰,婚禮策劃,地产,时尚,手工,室内设计,美容护肤,旅游摄影
摄影,美食,德国,比利亚,西班牙,巴萨球迷,旅游
文艺
穿衣美容
娱乐
穿衣美容
文艺,游戏动漫
电影,动漫,小说,旅游,吃货,天蝎座
校园,傻,听歌,90 后
星座命理,大学生
Be,your,sunshine
微博,营销,网络,好性格,80 后,汽车,游戏,时尚
游戏动漫,娱乐
时尚,美食,文艺,娱乐,星座,看电影,爱音乐,听音乐,淘宝,购物
文艺,体育,军事,星座命理,IT 数码,旅游,美女,汽车
新闻资讯,健康,旅游,美食,体育
文艺,娱乐,美食,星座命理,旅游
自由,娱乐,羽毛球,听歌,处女座
新闻资讯,文艺,星座命理,美食,投资理财
台球,唱歌,汽车
文艺,旅游,游戏动漫,努力
新闻资讯,亲情,旅行,生活,吃货
视频音乐,汽车,体育
新闻资讯,美食
魂淡,2B,幼齿控,御姐控,萝莉控,漫控,小宅
娱乐
音乐控,天秤座,玩,钢琴,NBA,大爱 poppin,90 后,搞笑,爱音乐,学生
投资理财
大学生,校园,创业平台,团购网站
文艺,时尚
娱乐
美食

美女,美食,其他生活
汽车,阅读,英语,好书,营销,交友
游戏动漫,美女,美食,旅游,文艺
军事,IT 数码,文艺,体育,娱乐
健康,天秤座
健康,旅游,星座命理,美食,文艺
娱乐
时尚
北京生活,善良
投资理财
122,海口十中,NBA,篮球
90 后沉默
美食,大学生,看书,90 后,善良,浪漫,好性格,偶稀饭睡觉觉
文艺
星座命理,旅游,文艺
娱乐
娱乐,星座命理,美食,文艺,Eason,曲婉婷,王若琳,根硕,容和,弘基
娱乐,酥糖会,微刊,吾爱文卓,赵文卓影迷,卓迷,赵文卓
宅女,羽毛球,善良,音乐
娱乐,投资理财,健康,美食,星座命理,军事,IT 数码
纠结女,白羊女,文艺,时尚,旅游,教育就业,娱乐
体育
新闻趣事
美女,星座命理,健康
星座命理,萌宠,旅游,微博奇葩,新闻趣事
投资理财,汽车,旅游,美食
陕西生活,新闻趣事,美女
健康,美食
成都生活,萌宠
李宇春
美食,娱乐
天生反骨
文艺,娱乐
爱自由,有为青年,旅游美食爱好者
萌宠,新闻趣事,视频音乐,微博奇葩
羽毛球,好性格,体育,美女,运动
校园生活,科学探索,读书分享,教育,情感生活
美食,时尚,旅游,娱乐
韩庚,时尚,ELF
IT 数码,新闻资讯,旅游,教育就业,投资理财,军事
健康,教育就业,旅游,文艺
时尚,新闻资讯,健康,星座命理,文艺
时尚,IT 数码
名人明星

美女,文艺
娱乐
美食,时尚,教育就业,汽车,文艺
娱乐
星座命理,小巫女,国学爱好者,经济学爱好者,厨娘,小资女,美食控,B 型血,HR,狮子座
军事,体育,旅游
汽车
旅游,搞笑幽默
旅游
成都生活
名人明星,帅哥控,宅女,小说控
娱乐,IT 数码,健康,汽车,旅游
音乐
教育就业,健康,新闻资讯,投资理财
娱乐,旅游
80 后,善良,八卦,天蝎座,吃货,网购,游戏,宅,看书,偶喜欢玩
美女
时尚
健康
时尚,投资理财,体育,军事,汽车
星座命理,新闻资讯,美女,教育就业,健康
香橙一枚,宅女一枚,天枰座,听音乐,文艺,娱乐,星座命理
美女
健康
投资理财
吃货一枚,无节操无下限,文艺装逼犯,多重人格待分裂,二次元宅,星座控
游戏动漫,军事
时尚,汽车,IT 数码,美食,娱乐
个性抱枕广告框,企业相册定制,个人照片制作,菜谱设计制作,设计,艺术,名片设计,建筑模型制作,海报设计,logo 设计
时尚,文艺,娱乐,教育就业,美食
我向往自由平等,宝宝,烹饪,忧郁,快乐大本营,美食,旅游,IT 数码,娱乐,体育
旅游
白羊座,美食,80 后,时尚,旅行
穿衣美容
娱乐
军事,体育,汽车,美女,IT 数码
汽车,军事,体育,娱乐,文艺
军人,忠于祖国,忠于人民,幽默,水瓶座,旅游,时尚,亲情,善良,宅女
文艺,娱乐
体育
纤体,我们都要做瘦纸
娱乐
新闻资讯,投资理财,IT 数码,军事

文艺,体育
游戏动漫,军事
文艺
旅游
军事,投资理财
娱乐
娱乐
搞笑幽默
娱乐
娱乐
双鱼座,天然呆
动漫,时尚,吃货,上网,囧,处女座,数码,娱乐,90 后
搞笑幽默
军事,新闻资讯,旅游
娱乐,听歌,音乐,睡觉,艺术
娱乐
美食,乐乐先生,学生一族,户外,摄影爱好者,体育,赚钱,90 後,爱美食,奋斗
新闻趣事,我是苏永鹏
新闻趣事,美食,上海生活
星座命理,时尚,汽车,娱乐,IT 数码
星座运势
八卦杂谈,不吐槽会死星人,第六感超强,毒舌腹黑,天然呆 T 口 T,低调的华丽,游戏迷,宅男,猫
自由,摩羯座,设计,个性,喜欢音乐
网络,上网,吃货,90 后,写作,处女座,美食,睡觉,看书,NBA
美容服饰,微博,听歌,看书
吃喝玩乐,执着,90 后但我不脑残,文艺,旅游,汽车
健康,美食
娱乐,天蝎座,听歌,幽默
营销
旅游,萌宠,视频音乐,搞笑幽默
美食,美女
育儿百科,美容服饰,美女,教育就业,星座命理
娱乐
萌宠,搞笑幽默,新闻趣事,北京生活,穿衣美容,旅行,吃货,金牛座,善良
IT 数码
校园生活
视频音乐
星座命理
敏感,爱吃,简单
全职写手,自由撰稿,水瓶女子,青丝文案
娱乐
娱乐,星座命理,时尚
重口味,Music,O 型血

IT 数码
美食,体育
娱乐,新闻资讯,健康,美食,吃货
美女,IT 数码,新闻资讯,游戏动漫,美食,文艺,娱乐
健康,美食,文艺,体育,娱乐,姑娘,好姑娘,体育的世界
娱乐
美女,天蝎座,自由,平常心,90 后
星座命理,活泼开朗,学生
旅游,星座命理,美食,文艺,娱乐
健康,旅游,文艺,小氢锌,人文男青年,瞎扯淡,1987,考古
旅游
投资理财,星座命理,旅游,娱乐,美食
旅游,游戏动漫,星座命理,美食,娱乐
文艺,游戏动漫,旅游,美食,娱乐,教育就业
巨蟹,音乐控,逛街,看电影,旅游,美食,娱乐
教育就业,投资理财,娱乐,时尚,健康,文艺,旅游
穿衣美容
娱乐
时尚,军事,美食,美女,投资理财
美女
写作,读书,财经
美食
美食,星座命理
健康,新闻资讯,投资理财,军事,文艺
穿衣美容
娱乐
知识,服务,旅游,80 后
游戏动漫,汽车,美女,IT 数码
汽车,旅游,娱乐,时尚,美食
娱乐
体育
赚钱,军事,IT 数码,文艺,旅游,体育
旅游
爱玩
旅游,穿衣美容
时尚,星座命理,娱乐
新闻资讯,娱乐,旅游,美食,时尚
游戏
旅游
星座命理,娱乐,美食,汽车,体育
名人明星
美食
娱乐
旅游,星座命理,文艺,美食,时尚

宠物图集
娱乐
投资理财,文艺,军事,娱乐,汽车
美食,旅游,太原生活,音乐控,诗词
健康,美食,电影,二郎腿,亲情友情爱情,社会闲杂人士,减肥中请勿喂食,80后,音乐
美女,投资理财,新闻资讯
健康,旅游,时尚,汽车,教育就业
猫咪,间歇性话痨,肉食系不挑食,OP
健康,投资理财,旅游
视频音乐
健康,娱乐,教育就业,美食,星座命理
娱乐
教育就业
娱乐
教育就业,旅游,美食,娱乐,体育
娱乐
视频音乐
历史,文字
娱乐,传奇私服,80后
搞笑幽默
娱乐,体育,旅游,美食
浪漫,欧美音乐,宠物,旅游美食,粤语
微博,旅行,平常心,乐观
搞笑幽默
健康
笑,文学,90后但我不脑残,猫咪
娱乐
文字,羽毛球,搞笑幽默,山东生活,穿衣美容,文艺,健康,投资理财,时尚,美食
文艺,新闻资讯,汽车,教育就业,旅游
下野紘
娱乐,游戏动漫,健康,美女,IT数码
努力,坚持,运动,rain,美食,隆多,NBA,k歌,篮球,学生一枚
投资理财
上海生活
文艺
手机,天蝎座
军事,IT数码
营销,交友,听歌,品味
娱乐,旅游
电影,美食,睡觉
时尚,美女,IT数码,汽车,健康
SUJU,TVXQ,东海希澈在中
星座命理
体育

娱乐
其他生活
体育
健康,星座命理
文艺,美食,旅游,投资理财,健康
旅游,游戏动漫,星座命理,体育,娱乐,IT 数码
新闻资讯,娱乐,教育就业,时尚,文艺,旅游,美食
美容服饰
天津生活
钢琴,音乐
旅游,穿衣美容
职场招聘,情感生活,旅游,北京生活,美容服饰,文学,旅行,玩儿
文艺
宅女,阅读,看书,睡觉,90 后,听歌,美食,网购,上网
女汉子,不吐槽会死星人,aline,directioner
游戏动漫
体育
教育就业,旅游
文艺,睡觉,90 后,平常心,吃货,囧,玩儿,双鱼座,看书,听歌
穿衣美容
独立,执着,感性,幻想,咖啡,爱美食,摄影,猫控
喜欢听歌
八卦杂谈
美食,电影,音乐
美食
崔雪莉,爸爸去哪儿,田亮,张悦轩,田雨橙
浙江生活,美食
旅游
汽车
文艺
体育
娱乐
旅游,美食
娱乐,健康,文艺
数码,努力,登山,坚强,汽车,IT 数码,健康,旅游
娱乐
旅游
旅游,IT 数码
文艺,时尚,游戏动漫,旅游,美食
投资理财
美食,文艺,健康,旅游
投资理财,旅游,美食,IT 数码,娱乐
其他生活
懒人,睡觉睡到自然醒,手机看小说,轮滑,天蝎座,网络,运动

健康,投资理财,教育就业,旅游,美食
晋中生活
娱乐,军事,体育
美女
美女
休闲,浪漫,营销,好书,历史,时尚,文艺,投资理财,新闻资讯,旅游
音乐迷,穿衣美容
海南生活
美食,娱乐,自由职业,旅游
汽车,美女,娱乐,体育,IT 数码
健康,星座命理
健康,时尚,投资理财,美食,文艺
视频音乐,搞笑幽默,西双版纳生活,名人明星,美女,时尚,自由职业,爱情
体育,娱乐
新闻资讯,美女,娱乐
娱乐
IT 数码,军事
娱乐,体育,健康,旅游
美食
美女
游戏动漫,娱乐,美食,星座命理
以父之名 1983
桌游,粤语,上网,旅游爱好者,瑜伽,睡懒觉,喜欢听歌,美食,动漫
娱乐
乐天派,美国,阳光,驴友
体育
军事,投资理财,健康,IT 数码,游戏动漫
四川生活
新闻资讯,教育就业,IT 数码
外貌协会,大叔控,美女
广东生活
旅游,投资理财,时尚
萌宠,搞笑幽默,微博奇葩,视频音乐,穿衣美容
时尚,星座命理,旅游,体育,听歌,运动,我喜欢看海,手机,微博,幽默
星座命理,IT 数码
美食
旅游
上网,摄影爱好者,语录,娱乐,自由,听歌,双子座,爱情,浪漫,汽车
星座命理,范爷粉,百吃不胖,高等吃货
投资理财,健康,新闻资讯,美女,旅游
文艺,新闻资讯
文艺,美女
文艺,旅游,真眸,善心,美言
游戏动漫

70 后,90 后,80 后,90,羽毛球,创业,睡觉,旅游,交友,自由
江苏生活
90 后但我不脑残,矛盾体,强迫症,音乐控,瘦身,旅行,双重性格
旅游,时尚,投资理财,新闻资讯,体育
90 后,看书,巨蟹座,张卫健
吃,好性格,娱乐,自由,听歌,交友,文字,时尚,天蝎座,电影
nonentity
旅游
时尚,偶稀饭睡觉觉
美食,健康,汽车
70 后,狂吃不胖,后知后觉
我随他爱旅游
羽毛球
投资理财
美食,娱乐,健康,时尚,星座命理
娱乐,时尚,旅游,文艺,投资理财
IT 数码
微博奇葩,美食,萌宠,穿衣美容,星座命理,执着,天蝎座,喜欢音乐
摄影
山西生活
美食
新闻趣事,IT 数码
娱乐
投资理财,体育
娱乐
汽车
网络
文艺,体育,娱乐,投资理财,美女
新闻趣事
健康,旅游,美女,军事,新闻资讯
文艺,旅游
旅游
自由,孙燕姿控,怀想,音乐,旅行,梦想成真
文艺,时尚,美女,投资理财,军事
娱乐,腐女,宅女,学生,90 后,自由,艺术,乐观,处女座,听歌
汽车,教育就业,健康,美食,IT 数码
美食
文艺,美食,美女,娱乐,旅游,癫,奇
星座命理
广东生活
视频音乐
健康
萌宠,生活
汽车,星座命理,体育,IT 数码

旅游
健康,美食,IT 数码
发呆,游戏动漫,双鱼座,90 后
健康,星座命理,娱乐,美食,时尚
微博奇葩,新闻趣事,搞笑幽默
旅游,教育就业,游戏动漫,文艺,美食
游戏动漫,娱乐
游戏动漫,IT 数码
投资理财,IT 数码,体育
情感生活
旅游,美食,汽车,投资理财
双子座,星座,飞行
唱歌
娱乐
体育
动漫,数码,音乐,90 后,美食,双子座
体育,IT 数码,旅游,美食,娱乐
健康,投资理财,星座命理,美食,时尚
美女,穿衣美容,旅游,搞笑幽默
爬山,紫色,坏人,平常心,写作
校园
文艺,听歌,看书,旅行,乐观,八卦,修练内心
旅游
娱乐
投资理财,教育就业,新闻资讯
玩儿,善良
娱乐,美食
狮子女,小女人,完美主义,情绪化的小女人,85 后,shopping
IT 数码,娱乐
新闻资讯,教育就业,投资理财,文艺
美食
时尚
健康,好书,娱乐,天秤座,美食,英语,平常心,旅行
搞笑幽默
星座命理,时尚,军事
时尚,教育就业,星座命理
生活,电影
旅游,游戏动漫,美食,体育,娱乐
美女
娱乐
美食,时尚,旅游,IT 数码,娱乐
健康
旅游,健康,美食
娱乐

时尚,旅游,美女,美食,娱乐,广告,爱萌,吃货,猫女
星座命理
体育
美女,旅游,星座命理,IT 数码
星座命理,美食,时尚,文艺,健康
美食,旅游,健康
工程管理,地产职业经理人,营销,羽毛球
新闻资讯,数据化团队建设,SEO 优化,天猫运营,淘宝客,直通车,钻展,精准营销,风夕马甲,数据化营销
娱乐
美女
体育
游戏,篮球,历史,旅行,水瓶座,90 后,交友,NBA,吃,玩儿
美食
时尚
微博奇葩
文艺,新闻资讯,时尚,艺术,设计,天蝎座
新闻资讯,教育就业,旅游,文艺,体育,爱情,篮球
旅游,文艺,娱乐,健康
娱乐
处女座,旅行,影视,爬山,美食,自由,摄影
90 后非脑残,美剧控,重口味,欧美音乐,美食
文艺,体育,娱乐
游戏动漫
游戏动漫,健康,旅游,IT 数码,娱乐
美食,星座命理,文艺
来不及 say,hi,吃货,自由,玩儿,音乐
汽车,双子座
娱乐
美女
体育
水瓶座
职场招聘
星座命理,游戏动漫,娱乐
娱乐
挖掘机,装载机,叉车,推土机,压路机,拖拉机,东方红,一拖,工程机械,路德机械
游戏动漫
娱乐
星座命理,射手座,90 後,许嵩迷,动漫控
汽车,体育
美食
汽车,游戏动漫,新闻资讯,娱乐,文艺,健康,旅游,星座命理,IT 数码,体育
教育就业,旅游,美食,文艺,娱乐
玩,吃货一枚,90 后但我不脑残,冷笑话,睡觉睡到自然醒,欧美控,爱美食,购物狂,电影迷

时尚,旅游,美食,娱乐
新闻趣事
努力向右,绝不自恋,西关小姐
时尚,网购
旅游
健康
美女
星座命理
体育,汽车,军事
汽车,娱乐
健康,教育就业,投资理财,星座命理,娱乐
投资理财,娱乐,IT 数码,文艺
体育
游戏动漫,新闻资讯,体育,投资理财,军事,教育就业,文艺
搞笑幽默,其他生活
健康,游戏动漫,投资理财,IT 数码,体育
体育,唱歌,运动,狮子座,电影,学生
情感生活,新闻趣事,美食,名人明星,视频音乐
搞笑幽默
文艺,投资理财,旅游,健康,美食
健康
视频音乐
旅游
体育,IT 数码,学生
新闻趣事,金牛座,吃货一枚,交友,时事,时评杂谈,新闻,学雷锋,网络
健康
汽车
美图摄影,健康,时尚,旅游,娱乐
电影爱好者,金融,单车骑行,逛街,感性,时尚,文艺,旅游
娱乐
搞笑幽默
时尚,投资理财,星座命理,美食,娱乐,学生
星座命理,健康,娱乐,旅游,文艺
大连生活
韩国小清新,欧美范儿,美食,superjunior,电影控,仙后,SM 家族饭,摩羯,维尼夫妇热血饭,
旅行
旅游
善良,吃,交友
美食
汽车,军事,旅游,健康,美女
旅游,赤峰生活,美食,穿衣美容,汽车,娱乐
健康,旅游,美食
汽车,IT 数码,娱乐
投资理财,新闻资讯,IT 数码,游戏动漫,星座命理,军事,文艺

汽车
名人明星,穿衣美容,微博奇葩,美食,搞笑幽默,宅女
IT 数码,美食
音乐,生活,游戏,电影,平常心,玩儿,美食,好书,文学
旅游,音乐,天秤座,时尚,看书
新闻资讯
星座命理,爱幻想,90 后但我不脑残,蝎子,完美主义,音乐发烧友
娱乐
健康,时尚,星座命理,旅游,娱乐,学生一枚
穿衣美容
游戏动漫
体育
热情,拍照
音乐,偶稀饭睡觉觉,美女,时尚
投资理财,IT 数码,体育
拒绝雷同
美食,健康,星座命理,娱乐,文艺,宅女,音乐控,双鱼座,摄影爱好者,囧
游戏动漫
体育
旅游
投资理财,健康,旅游,历史,东周,动漫,电影,景德镇,上海
旅游
体育
好书,偶稀饭睡觉觉,旅行
北京生活,架子鼓手,UKULELE,吉他,校园音乐,处女座
时尚
文艺,睡觉,呼和浩特,射手,90 后,包头
搞笑,学生一枚,文艺
偶稀饭睡觉觉
搞笑幽默
大学
体育
娱乐,游戏动漫
星座命理,美食
无聊
时尚,天蝎座,唱歌,60 后,亲情,善良,文学,交友,平常心
浙江生活,广告设计,品牌策划,平面设计,网站设计
视频音乐,搞笑幽默,南宁生活,星座运势,新闻趣事
去张根硕身边,赚钱,工作,张根硕,上网,羽毛球,天蝎座
投资理财,美食,健康,时尚
汽车,体育,文艺,IT 数码
美食,旅游,投资理财,汽车,健康,星座命理,IT 数码
music,体育,娱乐
揭阳生活,艺术,美术教育

爱笑,交朋友,向日葵,爱生活,星座,喜欢音乐,o 型血,天枰座,自然,摄影爱好者
旅游
投资理财,IT 数码,旅游,游戏动漫,汽车,体育,美食,军事
IT 数码,OA,办公软件,网络办公
美食
投资理财,星座命理,旅游,白羊座
小妮呓语,相信缘分
美食,乐观,善良,幽默,电影,旅游,音乐
汽车,游戏动漫,美食,军事,IT 数码
美女
时尚,IT 数码,体育
微博奇葩
娱乐
旅游,体育,军事,汽车
新闻资讯,健康,星座命理,娱乐
IT 数码,美食,旅游,星座命理,娱乐
旅游,健康,时尚,文艺,美食
军事
北京,房地产,营销
新闻资讯,军事
美女,投资理财,娱乐,文艺,时尚
穿衣美容
销售,市场,孩儿他爸,激光
健康,旅游
星座命理
健康
体育
星座命理,娱乐,健康,投资理财,旅游,偶稀饭睡觉觉,80 后,白羊座,阅读
汽车
娱乐,宅女
体育
名人明星,视频音乐,旅游,美食,搞笑幽默
新闻资讯,娱乐,美食,体育
美食
新闻趣事
旅游,美女
健康
旅游,二手房
时尚
IT 数码,单车旅行,爱旅行,汽车爱好者
文艺,美食,旅游
IT 数码,美女
旅游,军事
娱乐

文字控,叛逆,旅游,电影控
宠物图集
娱乐
篮球
设计,自由,文字,摄影爱好者,正能量,想象力特别强大,北京妞儿,射手座
时尚,娱乐,体育,我很出名
游戏动漫
阿森纳,旅遊
新闻资讯,时尚,娱乐,文艺,星座命理
健康,时尚,文艺,星座命理
投资理财,新闻资讯,星座命理,美食,娱乐,B 型血,乐观
喜欢音乐,搞笑!
旅游,健康,时尚,美食
巨蟹座,吃货,听歌,善良,玩儿,睡觉,网购
睡觉
旅行,睡觉,生活,电影,大学生
体育,夜猫子,死神
京剧,评书
娱乐
新闻趣事
沈阳生活
娱乐,美食,美女,汽车
文艺,旅游,美食,游戏动漫
强迫症,柯南,陈奕迅,蓝色,粤语,天蝎女,爱幻想,爱电影爱美食,爱音乐爱旅行,爱心理学
教育就业,新闻资讯,娱乐,吃货,处女座,想减肥又超爱吃,宅男 90 后
娱乐
美食
80 后,水瓶座
钢琴演奏,钢琴教师
美食,旅游,健康,时尚,娱乐
听歌,唱歌,睡觉
高中生
视频音乐
汽车
独家,明星访谈,娱乐,明星,最 8 卦的 8 卦,时尚,专栏,娱乐圈
视频音乐
星座命理,投资理财,吃货,自由,时尚
汽车,游戏动漫,时尚,旅游,IT 数码
微博奇葩,美女,搞笑幽默,旅游,名人明星,聚在一起开心玩
旅游
名人明星
时尚,星座命理
投资理财
艺术,欧美旧杂志,收藏鉴赏,古董珠宝,广告狂人,电影,时尚

绘画,钢琴,唱歌,设计,摄影,LOGO
新闻资讯,汽车,体育
汽车,星座命理,军事,文艺
娱乐,巨蟹座
健康
美食
时尚,美女,娱乐
健康
军事,IT 数码,星座命理,健康,投资理财
花样美男,旅游,美容服饰,美图摄影,健康,星座命理,美食,娱乐
摩羯座
旅游
美食,旅游,时尚,创业
旅游,娱乐
娱乐
美女,IT 数码,文艺,体育,娱乐,时尚,幽默,80 后
军事,投资理财,健康,教育就业,新闻资讯
IT 数码
营销,音乐,游戏,幽默,80 后,创业,善良,吃,看书
穿衣美容,名人明星,萌宠,微博奇葩,视频音乐
健康
军事
好书,音乐,90 后
美食
美食,汽车,体育,健康,美女
美食,旅游,健康,教育就业,文艺
旅游
游戏动漫
体育,文艺,游戏动漫,健康
Cashback,MichaelJackson,里屋,海豚,Dinosaur
健康,文艺,计算机,旅游摄影,拍照,街拍,文化,设计,绘画,摄影
军事
投资理财
军事,IT 数码,文艺,体育,娱乐
健康,星座命理,旅游,随性,维他命,粤语
旅游,穿衣美容,搞笑幽默,视频音乐,名人明星
新闻资讯,善良,自由,平常心,微博,乐观,手机,自由职业,电影
周觅后援会,周觅
娱乐
汽车,健康,军事,旅游,IT 数码
新闻资讯,美女,旅游,教育就业,投资理财
搞笑幽默,美食,星座命理,视频音乐,旅游
娱乐
IT 数码

新闻资讯
投资理财,游戏动漫,体育
90 后
魔羯座,爱幻想,校园,语录
新闻趣事
搞笑幽默,军事
游戏动漫,星座命理,旅游,美食,娱乐
微博控,巨蟹座,听歌,学生,唱歌,吃货,宅
穿衣美容
旅游,微博奇葩,穿衣美容,视频音乐,江苏生活,赖床族,想改变,爱美食爱电影,金牛座 MM
美食,时尚
美食
微博奇葩,白羊座
自虐,新闻,大学,历史,媒体,学生,天蝎座,动漫,美剧
听歌,交友,运动,美食,音乐,小说
微博奇葩
文艺
资产管理证券化,证监会,金融律师,体育
美女,IT 数码,旅游,军事
婚礼跟拍照片,婚庆摄影,婚礼摄影技巧,高端婚礼跟拍,婚礼纪实摄影,纪实婚礼摄影,太原,
婚礼摄影,婚礼跟拍
健康,教育就业,旅游,美食,娱乐
旅游
旅游,娱乐,美鞋世界,万千变换,快来选购
美女,游戏动漫,汽车,娱乐,体育
电影,喜欢听英文歌,90 后,插画爱好者,爱笑,爱玩,游戏动漫
汽车,时尚
双子座,80 后
健康
文艺,旅游
典型金牛座
健康,旅游,星座命理,唱歌,亲情,吃货
星座命理
教育就业,娱乐,新闻资讯,文艺
星座命理,微营销,电子商务,射手座,听歌,读书,英语
双鱼女,独立,主持人,看电影,听音乐,爱音乐,媒体,传媒,巨蟹座
穿衣美容,小女人,双重性格,护肤,天秤座,网购
处女座,旅游,dota,男人,健身,投资理财,汽车
教育就业
美食
文艺,校园,好书
腐女一只,吃货一枚,无厘头,矛盾体,摩羯女
时尚
微博奇葩,上海生活,新闻趣事

文艺,旅游
美食
健康
新闻资讯,星座命理,美食,旅游
旅游,IT 数码,投资理财,游戏动漫,体育,贪玩,乐观,体育不错,成熟,90 后
起床困难户!
时尚,新闻资讯,健康,星座命理,IT 数码
汽车
体育,听歌,上网,爱情,汽车,历史,电影
听歌,好性格,狮子座,偶喜欢玩,音乐,文字,90 后,吃,三分鐘熱度,三分钟热度
搞笑幽默,美女,动漫迷,小说
娱乐
教育就业
时尚
维尼夫妇,EXO,BIGBANG,吴亦凡,张根硕,权志龙,鹿晗
游戏动漫
美图摄影,情感生活,摄影,小说,娱乐
文艺,时尚,美女,美食,军事
体育
投资理财
IT 数码,星座命理,文艺,娱乐
娱乐
学生,艺术,好性格,摄影爱好者,NBA,篮球
搞笑幽默
星座命理
NBA,上网,学生,喜欢音乐,自信,美食控,快乐,微博控,体育
旅游,小辣椒
旅游,健康,IT 数码,时尚,星座命理
幽默
体育
健康
美食
美女,时尚,美食,文艺,娱乐
健康
新闻趣事
新闻资讯,娱乐
体育
投资理财,娱乐,健康,美食,美女
体育,娱乐,美女,健康,星座命理
游戏动漫
文艺,旅游
旅游,星座命理,不是 90 后的 90 后,天蝎座
视频音乐,校园生活,新闻趣事,美图摄影
健康,旅游,时尚,汽车,娱乐

谁没龙游潜水日
其他生活
娱乐,天秤座,英语,吃货,时尚,艺术,旅游,音乐,听歌,90后
美女,旅游,文艺,游戏动漫,美食
娱乐
投资理财
搞笑幽默,时尚,多动症………
体育
军事,时尚,娱乐,星座命理
美食,投资理财,旅游,新闻资讯,汽车,热爱生活,敏感,旅游美食
体育
游戏动漫,美女,汽车,美食,体育,旅游
唱歌,爱情
娱乐,时尚,健康,旅游,美食
娱乐
游戏动漫,文艺
经济,打篮球,旅游爱好者,购物,美食,政治,美女,新闻资讯,军事,体育
睡觉,娱乐,宅
汽车,游戏动漫,旅游,IT数码,娱乐,自驾游,摄影
星座命理,军事,娱乐,文艺
80后
娱乐
时尚
汽车
职业coser,网配CV
娱乐
健康,美食,时尚,教育就业,娱乐,乐观,吃货
健康
艺术,八卦,幽默,看书,听歌
旅游,美食,健康,乒乓球,斗地主,宅女
奋斗,时尚,汽车,娱乐,文艺,投资理财,跑步
汽车,美女,娱乐
文艺
星座命理,IT数码,文艺,美食,旅游,健康,娱乐
娱乐
美女
看电视,吃货一枚,流浪
教育就业,文艺
游戏动漫,文艺,汽车,旅游
关岛,旅游
电视人,新闻工作者,房地产记者,新闻,90后,传媒,电影,读书
星座命理,娱乐
教育就业
星座命理

80 后
美食,旅游,健康,投资理财
理财公司,大融小贷,网上贷款公司,投资理财公司,安全理财公司
娱乐
爱自由
IT 数码,旅游,美食,时尚
静临,野神,画画,神谷浩史,DRRR,折原临也,吐槽星人,电影,吃,动漫
娱乐
健康,橘子,火锅,环评工作者,80 后,双鱼座
音乐,睡觉,气度影响格局,性格左右命运
星座命理,娱乐
游戏动漫,旅游,美食,IT 数码,淘宝控,旅行,吃货,自由,游戏,天蝎座
美食,双子座,吃货,美食人生,为一切创造美食
成长,共青团
教育就业
汽车,时尚
80 后
健康,投资理财,旅游,美食
工作,人生,疯狂
微博奇葩
时尚,新闻资讯,旅游,体育,娱乐
IT 数码
军事
美女,文艺
旅行,羽毛球,股票,网购,运动,自由,美食
爱电影,心理学,跳舞,爱旅游,时尚
娱乐
娱乐,体育
KobeBryant,MichealJordan,MichealJackson,学生,KarlMalone,MagicJohnson,篮球爱好者
投资理财,摩羯座
90 后但我不脑残,闲聊
游戏动漫,美女,美食,IT 数码,娱乐
自认小清新。,矫情。,欠揍。,典型天秤。,小阴暗。,伪文艺。
时尚,美食
体育
聊天,学习,星座命理,旅游,体育,读书,大学生
星座命理,巨蟹座
傻得可爱,起床困难户
游戏动漫
星座命理
搞笑幽默
时尚
交友
自由控,宅女,吃,校园,射手座,90 后,小妞子,文字,电影,囧

喜欢旅游,时尚潮流,随性,天蝎,策划,重庆生活,动漫迷,梦想,青春,心理学
淘宝控,幻想,电影迷,听音乐,時尚,计算机,耽美,生活
旅游
时尚,娱乐
IT 数码
健康,手机,音乐
娱乐
幽默,小说
看电影,爱音乐,广东生活
微博奇葩,IT 数码
健康,时尚
汽车,体育
地主婆,腐女一枚,music 狂,HelloKitty 控,双重性格,乖乖女
美女
星际 dsa,星际争霸,dsa,rpg,星际
名人明星
新闻趣事
美食,博客,购物,媒体,语录,汽车,旅游,娱乐,新闻资讯
娱乐
星座命理
新闻资讯,军事
IT 数码,囧,吃货,上网,小说
旅游
设计
吃,汽车,宅,乐观,白羊座,旅游,新闻,美剧
旅游,美食,时尚,娱乐
教育就业
旅游,摩羯座,90 后,紫色,学生
新闻资讯,一刻钟社区,规范化社区,社区党支部,社区居委会,社区服务站
娱乐,手机,睡觉,学生,听歌
美食
名人明星
文艺
娱乐
旅游,体育,汽车,80 後,主持,dota,白羊座,行情,西班牙,篮球
穿衣美容
表演,搞怪
汽车用品,电子商务
巨蟹,巡演,Live
投资理财,文艺,娱乐,军事,新闻资讯
汽车,美女,健康,旅游,美食
健康
大学生,艺术,瘦身,爱情,好书,摄影爱好者,双子座,网购
新闻趣事,爱旅游,英超球迷,偶尔也学术,单身宅男,爱听歌,射手座

旅游
星座命理,美食,文艺,生活,听歌,阅读,NBA,白羊座,读书
爱情,123
美女
IT 数码,汽车
教育就业
搞笑幽默,投资理财
恐怖片大爱,喜欢科幻相关,涂鸦,听歌,读书
美食,娱乐,时尚
短袖,泳裤,男装,服饰,服装
123,游戏,网络,浪漫,亲情
美女
旅游,游戏动漫,时尚,汽车,军事
旅游,白羊座,小说,平常心,紫色,好性格,80 后,偶稀饭睡觉觉,巨蟹座
新闻趣事
IT 数码,游戏动漫
狮子座,街舞,睡,玩,hiphop,吃,听歌
微博奇葩
时尚,娱乐
顽皮鬼,吃货,90 后
旅游,80 后
旅游,视频音乐
投资理财,旅游,美食,娱乐
萌宠
娱乐
体育资讯
教育就业,投资理财,娱乐,美食,体育,大爱 ldylickem,大爱阿姆,摩羯,AB,爱篮球
娱乐
旅游
双子,唱歌,上网,玩儿,海贼王,喜欢音乐,体育,游戏动漫,美食
幽默,八卦,善良,微博,自由,我爱旅游
体育
健康,旅游,美女,美食,体育
投资理财,健康,美食,星座命理
旅游,新闻趣事,视频音乐,搞笑幽默
IT 数码,新闻资讯,军事,健康,投资理财
美女,游戏动漫
文艺,喝喝茶,晒晒太阳
娱乐,美食,健康,睡觉觉,摩羯座 B 型血,小说
健康,投资理财,汽车
IT 数码,建筑,爱笑,听歌,语录
娱乐
自然,街舞,书籍,爱,时装,销售,紫色,偶稀饭睡觉觉,艺术,水瓶座
钟小哇,钟汉良 Wallace,时尚,柯南,学化妆,电影,家居,平常心

文艺
旅行,生活,创业,大学生村官
投资理财,新闻资讯,军事,文艺,美食,美女,时尚,IT 数码,娱乐
旅游,投资理财
搞笑幽默
名人明星
娱乐
三国杀,生活,搞笑,吃货,八卦,处女座
美食,超爱狗狗,射手座
微博奇葩,穿衣美容,搞笑幽默,娱乐,宅女,偶稀饭睡觉觉,90 后
文艺,时尚,美食,旅游
美食,汽车,旅游,军事
美食
旅游,骚年,不定期刷屏,抵制傻 b,竹马最高,翔润不拆不逆,JP 离我远点,A 团
爱自由,看电影,爱音乐
美女,娱乐
电影,音乐,八卦,天蝎座,听歌
旅遊,BIGBANG,90 后,吃货,咖啡控,古典音乐
宗教,猎人,德国,天文,白羊座,外语,WOW
IT 数码
恐怖片,手机,完美主义,沉默,爱玩,怀旧,驴友,神经质
时尚
幽默,交友,摄影,旅游
宅,美食,好性格,上网,生活,双鱼座
旅游
星座命理,美女,美食,娱乐,体育
旅游,军事,体育,文艺,新闻资讯
旅游,健康,投资理财,IT 数码,军事
时尚,汽车,歌词控,music,拍照,旅游美食,幸福的地图
笑话
体育
时尚,新闻资讯,旅游,娱乐
体育,美食,教育就业,投资理财,新闻资讯,旅游
喜欢蓝色,白色
Music,善良,幽默,活泼可爱,文字,气质女,学生一族,处女座,有个性,90 後
旅游
旅游,美食
可爱,高中生,90 后非脑残,正太控,购物,O 型血,唱 K,时尚,美食,娱乐
游戏动漫,理论物理
时尚,IT 数码,文艺,美女,美食,旅游,体育
新闻资讯,美女,汽车
名人明星,穿衣美容,美女,搞笑幽默,视频音乐
时尚,生活,文学,电子商务,汽车,微博,阅读,羽毛球,写作,新闻
旅游

新闻资讯,时尚,军事,娱乐
巨蟹,音乐,BIGBANG
体育
时尚,娱乐,美食,健康
美食
IT 数码
娱乐
湖北生活,美食,星座命理,穿衣美容,搞笑幽默,好书,动漫,唱歌
旅游,美食,萌宠,微博奇葩,美女,健康,IT 数码
美食
时尚,女装
文艺
教育就业
小说,猫控,游戏动漫
旅游
星座命理,美食,文艺,健康,游戏动漫
名人明星
精品酒店,酒店预订,连锁酒店,时尚,绿色,旅游,商务
时尚,美食,旅游,健康,娱乐
金钱,欲望,九零
娱乐,时尚,体育,IT 数码
名人明星
奇门兵刃,游戏时代,智枭,千门,武侠小说,方白羽
好书,八卦,生活,吃,宅
娱乐
军事,旅游,80 后,足球,历史,新闻
旅游,穿衣美容,星座命理,微博奇葩,美食
新闻资讯,健康
旅游,大学,足球,善良,乐观,运动,上网
学生一族,90 後,hiphop,音乐控,爱旅游,爱电影,双鱼,偶稀饭睡觉觉
娱乐,旅游,美食
美图摄影
星座命理,娱乐
时尚,IT 数码,美女,军事,娱乐
海高,海宁市高级中学,留学生,天秤座
游戏动漫
搞笑,听歌,时尚,自由职业
低调,慢热,对外汉语,东方神起,suju,音乐,美剧,异次元
狗狗,摩羯座,法律,音乐控,台球,喜欢旅游
旅游,健康,美女,时尚,娱乐
旅游,健康,美女,美食,文艺
电影,足球,娱乐,篮球,自由
旅游
美食,教育就业,新闻资讯,文艺,健康,艺术,大学,大学生,网络

时尚
美食,健康
美食,教育就业,文艺,长大,重汽
投资理财
美食控,志愿者,公益活动,旅游,名人明星,微博奇葩,新闻趣事,浙江生活
好书,美食,旅游,电影,水瓶座,音乐
新闻资讯
娱乐
体育
汽车
自由,学生,上网,睡觉,宠物,90後,爱电影,爱幻想,购物狂,爱美食
旅游,上海生活
旅游,美食
旅游,星座命理,健康
军事,旅游,体育,互关注,互粉丝,投资理财,教育就业
Aph不毕业,已弃疗,声控末期已没救,伪文青,立花慎之介,壮哉我大不列颠
文艺,旅游,美食,羽毛球,宅女,90后,艺术
星座命理,美食,旅游
双鱼座,旅游,好性格,乐观,80后,美食,电影,生活,吃,唱歌
娱乐
游戏动漫,娱乐
快乐,约瑟爹利,约克夏
杭州生活
汽车
体育
教育就业,健康,旅游,美食
体育
文艺
微博奇葩
美食,旅游,穿衣美容,广州生活
fashion,羽毛球,90后,爱金融,女会计
文艺,汽车,旅游
旅游
浪漫
游戏动漫
迷糊,任性,自由,旅行,上网,睡觉,美剧迷,狗狗,爱吃,80后
旅游,健康,投资理财,美食
穿衣美容,星座命理,搞笑幽默,视频音乐,旅游
旅游,军事,娱乐
星座命理,IT数码,军事,游戏动漫
健康,旅游,星座命理,美食,娱乐
健康,文艺,旅游,体育,美食,爱睡觉。
健康,时尚,美食
美女,娱乐,IT数码

手机,校园,90后
FXTZ
娱乐
上海大众,唐都燕华,汽车,旅游
体育,美食,健康,美女,时尚
艺术,敏感,美术,完美主义,双重性格,文字,90後,原创,游戏动漫,天秤座
娱乐,旅游
美女
喜欢旅游,书,music,美食
旅游,健康,新闻资讯,文艺,体育,吃货,运动,娱乐,篮球
文艺,帕客,素食主义者
文艺,IT数码,娱乐
旅游,星座命理,时尚,娱乐,IT数码
浪漫,自由,设计,艺术,小说,音乐,运动,听歌,90后,网购
读书
读书,羽毛球,90后,吃货,白羊座,乐观
美女,娱乐
投资理财
金融,贸大,实践,传媒,大学生,吃货
投资理财,快乐的小女子,时尚,摄影
iphone,时尚潮流,时事,培训,Music,诗歌,格子控,音乐,股市,美国
旅游
星座命理,文艺,美食,健康,游戏动漫
IT数码,文艺
投资理财,娱乐,旅游,星座命理,文艺
微博奇葩,美食,旅游
IT数码,美图摄影,名人明星,唐山生活
新闻资讯,教育就业
健康,旅游,娱乐
呆,懒,冷
星座命理,美食,旅游,军事
安防,大数据,全民创业,网络游戏,软件,服务外包,科技,孵化
美食,搞笑
搞笑幽默
军事
汽车,IT数码,健康
娱乐
体育
旅游
荧光绿,吃货,刘忻
新闻资讯
Sser
美女,小清新
娱乐

娱乐
星座命理
旅游,健康,体育,投资理财
健康,星座命理,美食,文艺,娱乐
时尚,双鱼,上升处女
体育
时尚,白羊座,宅女,偶稀饭睡觉觉,宅,浪漫
新闻资讯,娱乐,体育,IT 数码,时尚
体育
IT 数码,娱乐
文艺
健康,旅游,新闻资讯,星座命理,美女
旅游,微电台,音乐控,感伤的天蝎
简单,偶稀饭睡觉觉,金牛座,傻
时尚,旅游,星座命理,美食,娱乐
娱乐
旅游,健康,时尚,娱乐,美食
美女,娱乐,体育
历史,吃货,天蝎座,唱歌,汽车,网络,80 后
巨蟹女,动漫控,完美主义,体育,娱乐,星座命理,IT 数码,游戏动漫
宅,文字,90 后,小说,偶稀饭睡觉觉,射手座,学生,听歌,写作,阅读
视频音乐,潮
动漫,处女座,日语,低调,猫咪,生活,健康,IT 数码
名人明星
文艺
新闻资讯,美女,军事
萌宠,微博奇葩
军事,时尚,星座命理,娱乐,体育
汽车,时尚,美食,健康,旅游
听歌,旅游,吃货,幽默,唱歌,偶喜欢玩,90 后
旅行,宅女,80 后,双鱼座,音乐,自由,幽默,搞笑
美食,旅游,娱乐
爱睡觉,美术,乐天派,文字控,逛街控,美食控,魏晨控,宅女,天蝎座,学生
时尚,星座命理,文艺
文艺
LOVECHILD,DREAMER,WILDFOX
体育
健康,星座命理,美食,娱乐
美食,星座命理,穿衣美容
搞笑幽默
美女,体育,娱乐
健康,旅游,投资理财,美食,娱乐
投资理财,汽车,美女,时尚,游戏动漫
娱乐,快乐的生活

文艺,美食,教育就业,健康,旅游,娱乐,美食
新闻资讯,帽子选择,帽子围巾控
80 後,狮子座,矛盾,情感,文字,爱旅游,活泼开朗,淘宝控
上网,巨蟹座,时尚,80 后,浪漫,善良,旅游
娱乐,美食
简单,健康
搞笑幽默,90 后,音乐,电影,金牛座
星座命理,美食,文艺,教育就业,投资理财
新闻资讯
靠谱,文艺青年,金牛座
娱乐
时尚,娱乐,美食,旅游,妞寶兒,豬比,絕唱一段芊芊,最愛爸爸,天枰座的妞妞
健康,美食,教育就业,文艺
美食,IT 数码,文艺,体育,娱乐
美食
美食,星座命理,时尚
狗狗,大学,于和伟,90,学生一枚,张泽群,陈道明
拍照,K 歌,美食,娱乐,文艺
娱乐
发呆 ing,宅,篮球,手机,自由,搞笑,善良,偶喜欢玩,NBA,90 后
青岛生活
咖啡控,历史,娱乐,艺术,巴黎,金牛座,美食,音乐
善良,旅行,电影,足球,看书,运动,历史
健康,文艺,星座命理,美食
北京生活,穿衣美容,美食,旅游
美女,IT 数码
时尚,星座命理,唱歌,网购,音乐,吃货,90 后
美女,娱乐,体育
吃
健康
健康,美食,旅游,娱乐,IT 数码
美女,体育,教育就业,旅游,军事
萌宠,旅游,郑州生活
美食
星座命理,娱乐,游戏动漫
微博奇葩
旅游
80 后
星座命理
星座命理
西北农林科技大
娱乐,90 后开始的一代,自由
旅游
微刊,时尚

文艺
美容服饰
微博奇葩,搞笑幽默,视频音乐,巨蟹座,90 后
时尚
美食,摄影,微博,旅行
娱乐
微博控,85 后,music
时尚,化妆,水瓶座,动漫
职业画家
旅游,健康,时尚,投资理财,IT 数码,Drama 控,HipPop,engineer
新闻资讯,美女,时尚,娱乐,体育
娱乐,热爱生活,摩羯座,80 后
娱乐,旅游,文艺,投资理财
游戏动漫
微博奇葩,星座命理,新闻趣事,旅游,美食
美食
星座命理
视频音乐
美食,平面设计,90 后,金牛座
古剑奇谭
cosplay
BIGBANG,盗墓笔记,自由,唱歌,射手座,90 后,学生,好性格,宅女,玩儿
旅游
健康,星座命理,IT 数码
时尚
娱乐,小清忻,双子女,神经大条
娛樂,吃货,天秤座
IT 数码
旅游
IT 数码
穿衣美容
正义,法律,幽默,亲情,英语,宅
文艺,旅游,美食
穿衣美容
游戏动漫,投资理财,IT 数码,娱乐
美女,小说,胡思乱想
星座命理
体育
旅游,汽车,IT 数码
文艺
汽车,时尚,星座命理,文艺,娱乐
偶喜欢玩,偶稀饭睡觉觉,音乐
投资理财,健康,旅游,星座命理,美食
星座命理,IT 数码

文艺
新闻资讯,文艺
名人明星
电视剧
娱乐
星座命理
军事
美女
投资理财,美女,健康,星座命理,美食
新闻资讯,健康,文艺,投资理财
娱乐,旅游,逛街,K 歌,开朗,搞笑
娱乐
体育
体育
体育,游戏动漫
其他生活
校园生活,时尚
投资理财
体育
搞笑幽默
教育就业,新闻资讯,健康,星座命理,娱乐
美食,金牛座,偶稀饭睡觉觉,善良,80 后,吃货,听歌,网络,宅女,电影
喝酒玩牌踢足球
新闻趣事
娱乐,军事,文艺
旅游
购物,自信
美女,搞笑达人,dota 酱油党,天秤座男人,听歌,囧,游戏
伍毫籽的意义。
校园生活,星座运势
旅游,音乐　电影　旅游
旅行,宅女
旅游,美食
新闻趣事,穿衣美容,星座命理,搞笑幽默,美女
旅游,健康,投资理财,文艺
文艺,coser,诱受,猫控
时尚
娱乐,旅游,教育就业,新闻资讯,美好的事物,美食,爬山
美女
美食,娱乐
娱乐
娱乐
旅游,爱情,唱歌,时尚,篮球,校园,好书,语录,八卦,文字
时尚

狮子座,紫色,90 后
旅游,苏州生活
教育就业,新闻资讯,旅游,旅游爱好者,大叔控,仙剑,学习,电影迷
钻井工程,海洋石油,天津泰达开发区,棒子完蛋操去,韩庚加油,女赵薇男韩庚
7V,娱乐,投榜
美食,西安生活,读书分享,校园生活,搞笑幽默
搞笑幽默,健康
白羊座,自由,新闻,平常心
自由职业,旅游,逛街,上网,数码,睡觉,音乐
美女
绘画,美食,游戏动漫
户外,摄影,旅游
文艺,追女孩子,想她
生活,吃,唱歌,玩儿,射手座,宅
游戏动漫
IT 数码
互联网,网游,CF,DJ,游戏动漫,文字,巨蟹座,小说,音乐,微博
IT 数码,军事
娱乐
投资理财,旅游,健康,新闻资讯
看书,旅行,音乐,生活,运动,交友,摄影,金牛座
军事,娱乐
星座命理,交友,音乐,汽车,90 后,旅行,艺术
奕生为欢
IT 数码,美食,星座命理,娱乐,游戏动漫
穿衣美容
旅游
星座命理
健康,文艺,娱乐
教育就业,美食,投资理财,健康,新闻资讯
时尚,旅游,美女,教育就业,文艺
游戏动漫,文艺,IT 数码,星座命理
美食
游戏动漫
体育
快乐大本营,摩羯女,艺术,上海生活,舞蹈,旅游爱好者,韩剧,星座命理,健康,时尚
汽车
八卦
名人明星
创业,幽默,80 后,历史,艺术,旅游,阅读,自由,摄影,唱歌
美女,时尚,投资理财,旅游,健康
秒客,桌球,广告人,桌游,好性格,偶喜欢玩,创业,投资理财
军事,体育,IT 数码,运动,美食,听歌,读书,学生,狮子座,音乐
娱乐,新闻资讯,时尚,投资理财,文艺

时尚
文艺,游戏动漫,军事,IT 数码
旅游,文艺,娱乐
淘宝店铺,旅游
体育
新闻趣事,搞笑幽默,微博奇葩,旅游,美女
美女,健康
体育
吃货一枚,爱笑,love,街拍,宅女一枚,大学,时尚
晒幸福！2011 第
投资理财,时尚,娱乐
广州生活,80 后,吃货,听歌,宅女
新闻趣事
星座命理,电子商务,营销,睡觉,宅
睡觉
爱玩,美食,旅游,健康,汽车,体育
教育就业,投资理财
好性格,美食,笑话,强迫症,自由,平常心,大学生,天蝎座,玩儿,吃货
巨蟹座,偶稀饭睡觉觉,美食,玩儿
星座运势
藏地之旅——拉
文艺
嫩萌萌学长,萌萌学长,小嫩嫩,好奇宝宝,小可爱,小淘气,90 后的小 P 孩儿,大吃菜小坑货,熊孩子,90 后
美女,体育,时尚,旅游,娱乐
日剧控,恐怖片,学生一族
旅游,星座命理,娱乐,美女,体育
篮球
IT 数码,美食,文艺
新闻资讯,汽车,IT 数码,游戏动漫,体育
文艺
叼烟的女孩：做
旅游,教育就业,IT 数码,80 后宅女,孩子气,学习,市场营销,新闻资讯
旅游,健康,汽车,娱乐,体育
娱乐
娱乐
美食,旅游,游戏动漫,娱乐
世界上有这么一
新闻资讯,美食
射手,篮球爱好者,台球,吃货一枚,会计,闷骚,体育
娱乐
时尚,星座命理,娱乐
美女
IT 数码

星座命理
健康,投资理财
汽车,更爱生活,爱音乐,爱电影,爱车,90 后,白羊座
水钻,Iphone,粉嫩,保护壳,可爱,奶油,冰激凌,芭比,手机壳,DIY
娱乐
原谅我,这样一
瑶山来的喜帖:
花痴妹自曝:我
星座运势
健康,文艺,体育
娱乐,体育
水瓶,90 后一追求淡定,淘宝店主
娱乐
美女,美食
名人明星
娱乐,体育,IT 数码,文艺
美女
文艺
90,画画,创意,爱自由,乐天派,独立
军事,美女
陈奕迅,阿根廷,志愿者,天秤,睡觉,体育,美食控,玩
星座命理,旅游,新闻趣事,天津生活,搞笑幽默
时尚
旅游,吃货,巨蟹座,90 后
IT 数码
火影忍者,篮球爱好者,低调,90 後,欧美,打球,休闲,体育
文艺,美食,健康,古典音乐,健身,三国杀,单身,武艺,学生一枚,喜欢听歌
萌宠
神经质
体育
背包族,蜜蜂,双鱼座,宅,音乐,校园,学生,爱犬族,旅行,00 后
时尚
music,旅游
笑话,搞笑,冷笑话,糗事,恶搞,段子
艺术,麦霸,学生一族,矛盾体,美食控,互粉
快乐大本营,90 后的宅女,大学学生,星座,巨蟹座,动漫,张根硕
星座命理
坚强,自信!
商业地产,房地产
合肥生活
金融,活泼,偶稀饭睡觉觉,善良,电影爱好者,自然,音乐达人,水瓶女,旅游
女人,化妆,少女时代,臭美,自拍,交友,娱乐,上网,淘宝控,80 后的尾巴
旅遊,美食,文藝,娛樂
健康

穿衣美容
娱乐,文艺,旅游,听歌,水瓶座,上网,摄影,小说,美食
漫画,化妆,美剧控,水瓶,摩羯女
微博奇葩,美女,萌宠,星座命理,搞笑幽默
时尚,娱乐,学生,动漫
娱乐,汽车
体育资讯
投资理财,旅游,创业
娱乐,旅游
旅游
潮爆,可爱,日系混欧美,美女
娱乐
体育,天蝎座
你懂的
健康,美女,教育就业,娱乐,旅游
文艺
美女
屌丝
美食
教学,漫画创作,囧,校园,音乐
投资理财,时尚,旅游,美食,文艺
旅游,美女,体育,汽车,IT 数码,玩儿
江西生活,体育,美女,军事,时尚
投資理財,美食,星座命理,IT 數碼,旅遊
健康,投资理财,星座命理,美食,娱乐
旅游,美食,旅行,天秤座,80 后
IT 数码,美女,游戏动漫,娱乐,军事
搞笑幽默
中日混血,北京,天津,神户,中国,日本,混血
微博奇葩,旅游,穿衣美容,搞笑幽默
旅游
时尚
IT 数码,汽车
大学,大学生,协会,中国,985 重点大学,职业发展协会,求职,职业,天津,南开大学
新闻资讯,健康,美食,旅游
搞笑幽默
交友,乐观,90 后,唱歌,小酒窝,偶稀饭睡觉觉,吃,偶喜欢玩,自由,处女座
美食
星座命理,IT 数码,文艺,时尚,上网,睡觉,处女座,善良,看书,自由
旅行,职业人
娱乐,90 后宅男
新闻资讯,游戏动漫,体育
IT 数码,美食,娱乐,星座命理,新闻资讯,军事
体育

无语 ing,反感 ing,乐观 ing,叛逆 ing,海绵宝宝,彼得兔,努力 ing,缺爱 ing,纠结 ing,无奈 ing
自赏黑金属,邪三昧,中古漫,驾笼真太郎,邪典,猎奇控,cult,手绘
名人明星
娱乐
健康,美女,投资理财,体育,军事
新闻趣事
娱乐,美女,旅游
汽车,文艺
手机,玩儿,电影,羽毛球
娱乐,搞笑
文艺,健康,善良,音乐,吃货,90 后
投资理财
军事
帅哥,睡觉睡到自然醒,看小说,90 后但我不脑残,我就是我,情感,金牛,新闻,文艺
休闲,钢琴,跳舞,敏感,拍照,文字,互联网,八卦,玩儿,情绪化
娱乐
穿衣美容,娱乐,健康,乐观,90 后,天蝎座
校园生活
星座运势
美食
享受生活,追逐快乐
新闻资讯,健康,教育就业,星座命理,旅游
旅游,文艺,健康,美食,游戏动漫
美食
上海生活
美女,其他生活
Entertainment,Video
星座命理,美食,IT 数码,汽车,健康
努力 ING,吃货,旅游,AB 双鱼,可爱多,麦霸
游戏动漫,90 后,动漫,天秤座
大学,我来啦
美食
新闻资讯,健康
星座命理,健康,娱乐,美食
游戏动漫,美女
教育就业,投资理财
游戏动漫
新闻资讯,健康,娱乐,旅游,美食
投资理财,新闻资讯,健康,时尚
工作狂,钓鱼,低调,营销,吃货一枚,爱睡觉,广告,社会,足球迷,投资理财
美食,健康,教育就业,时尚,娱乐,宅,善良,巨蟹座,好性格,听歌
阅读,美剧,娱乐,水瓶座,天秤座,微博,生活,123,幽默,90 后
健康,旅游,文艺

好书,青春,随性,幸福,旅游美食爱好者,疯子
玩儿,金牛座,唱歌
娱乐
旅游,新闻资讯,娱乐,军事,教育就业
美女,视频音乐,搞笑幽默
胡思乱想
旅游,时尚,游戏动漫,健康,美食
娱乐,星座命理,美食,时尚
旅游,文艺,美食
娱乐,美食,旅游
名人明星,美食,视频音乐,搞笑幽默
视频音乐
健康,星座命理,旅游,IT 数码,美食,娱乐
搞笑幽默,视频音乐,美女,旅游
萌宠
娱乐
新闻资讯
校园生活,天蝎女,龟梨和也,人生
旅游
美女
体育,热爱篮球
名人明星,娱乐
IT 数码
80 后,天蝎座
30 歲,台灣,台中市
旅游
体育
美食
护肤保养,家装设计,居家装饰,旅游控,爱情,家居装饰,装修,旅游,旅行
时尚,美女,投资理财,汽车,IT 数码
AtlantaFlacons,AtlantaBraves,Hotspur,Madridista,网球和高尔夫,花滑、艺体中和,NFL、MLB、NHL,自行车和健身,NCAA
美食,文艺
文艺
美食
旅游,穿衣美容,搞笑幽默,深圳生活
装潢艺术设计,军事爱好者,旅游,视频音乐
美女,游戏动漫,军事,IT 数码,文艺
ELF 妖精,绘画,美术,cosplay,吸血鬼,淡定,ACG,射手座
时尚
游戏动漫,星座命理,娱乐,体育
睡觉,旅游,天秤座,玩儿,宅女,爱玩,喜欢旅游,喜欢漫画,独立
娱乐
大学生,偶喜欢玩,IT,篮球,水瓶座,阳光,直率,奋斗 ing,健身,90 后

游戏动漫
美食,文艺
娱乐,游戏动漫,汽车,IT 数码,体育
投资理财,健康,军事,游戏动漫
工艺品,收藏品,奢侈品,老挝红酸枝,明清家具,仙游红木家具,红木家具,仿古家具,古典艺术家具,仙作产品
书籍,历史,财经,散步,养生,心理学
娱乐
美食
美食,美女
美食,教育就业,健康,80 后好妻子,好妈妈,自由,自由职业,80 后,吃货
创业,学生一枚,方大同
击剑,飞行,通用航空,OSU,北京二中
微博奇葩
娱乐,旅游,文艺
拍照,爱自由,爱音乐,任性,爱情,街舞,欧美控,健康,美食,娱乐
投资理财,健康,美食,IT 数码,文艺
投资理财,文艺,矛盾,喜欢音乐,动漫,摄影,小说,看书,爱睡觉,夜猫子
娱乐
护理
IT 数码
爱音乐,看电影,发呆,新闻资讯,美食,体育
美食
视频音乐
文艺,时尚,美女,娱乐,体育
漢語文學,興趣心理學,交友,Venice,油畫、掃描
文艺
娱乐
ELF 妖精,SUPERJUNIOR
健康,旅游,IT 数码,娱乐,汽车
文艺
天蝎座
育儿百科,吃货,90 后,宅,双鱼座
星座命理
健康,旅游,爱幻想,水瓶座加 O 型血,80 后宅女,爱情,时尚,美食
体育资讯
IT 数码,文艺
教育就业
90 后但我不脑残,彩妆,梁静茹,服装,自恋,化妆品,双子,设计,娱乐,时尚
微博奇葩,搞笑幽默
电商,音乐,电影
军事,新闻资讯
美食
美食,旅游,健康,时尚,娱乐,休闲,爱吃,宅女一枚

星座命理
新闻趣事,搞笑幽默
看各种美景,尝各种小吃,发各种呆,要加油,天秤座,80 后,旅行
旅游,广东生活,微博奇葩,狗狗,小女人,80 後,有洁癖
娱乐
夏目,LOL,虎牙控,霹雳,瓶邪,OP,吃货,真三,银魂,基三
厦门生活
娱乐,时尚,旅游,文艺,投资理财
赛夏党,耽美控
健康,时尚,旅游
哈韩但爱国,张艺兴,旅游,星座命理,娱乐,潮流,exo,射手座,90 后
游戏动漫
超严重精神洁癖,重口味,獅子座
游戏动漫
IT 数码,美女
美食,旅游
健康
娱乐
胆子大,犟脾气,固执
囧
健康
星座运势,解梦,中国术数,解梦师,紫微斗数,西洋占星,易经八卦,佛道双修
美食
美女,星座命理,美食,军事,文艺
旅游
新闻资讯,汽车,投资理财,体育,娱乐
旅游,健康,投资理财,新闻资讯,美女,娱乐,汽车
星座命理
江苏生活,美食,旅游,穿衣美容,名人明星
独立,心理,自信,思考
唔烟,唔蒲,唔饮酒,“好学生”,石岐中学
吉林生活
旅游
旅游,健康
健康,娱乐
体育
旅游,旅游美食爱好者,阳光,创意,教育,IT 数码,文艺,体育,娱乐
音乐,吃喝玩乐
天蝎座,90 后,睡觉
美食,军事,文艺,体育,娱乐
军事,新闻资讯,健康,投资理财,旅游
美食,娱乐
体育
视频音乐

文艺,体育,娱乐,健康,美食
旅游,娱乐
美食,八卦,时尚,摄影,自由,宅女,旅游,偶稀饭睡觉觉,动漫,天秤座
河南生活,微博奇葩,星座命理
天秤座
偶稀饭睡觉觉,90 后,浪漫,摩羯座,宅女
视频音乐,90 后,音乐控,微博控,手机控,互听
读书,宅,天秤座
美女
教育就业
减肥去
幽默,睡觉,偶喜欢玩,美女,90 后,阅读,自由,娱乐,吃
音乐发烧友,追星
新闻趣事,听歌
写作,电影,网购,艺术,足球,射手座,音乐,浪漫,生活,旅行
穿衣美容
文艺,玩儿,幽默,交友,乐观,好性格,唱歌,音乐人,音乐,四川音乐学院
时尚,汽车,美食,IT 数码,娱乐
吃货,处女座,游戏,动漫
星座命理,游戏动漫,娱乐,体育
时尚,宅,小说,射手座,好性格,90 后,学生
汽车,新闻资讯,游戏动漫,投资理财,旅游,时尚,IT 数码
美女,军事
美食
汽车
视频音乐,游戏动漫,IT 数码,娱乐
旅游,健康
教育就业,旅游,美食,文艺,娱乐
军事
电影爱好者
时尚,美食
穿衣美容
美食
文艺
安防监控,监控摄像机,监控摄像头,安防产品
文艺,IT 数码
旅游,美食,视频音乐,成都生活
创业
娱乐,体育
美食
健康
IT 数码
旅游
新闻资讯,教育就业

时尚,娱乐
游戏动漫
好性格,生活,音乐,手机,运动
美食,金牛座,直率,能睡,爱吃,爱狗狗,爱兔子
旅游,新闻资讯,娱乐,时尚
汽车
投資理财,旅游,上海生活
文艺
健康,旅游
娱乐
教育就业
文艺,美食,旅游,星座命理
视频音乐,美女,大学,媒体,好性格,亲情,微博,设计,旅行,电影
文艺,娱乐,美女
美食,游戏动漫,旅游,体育,娱乐
汽车,IT 数码,星座命理,美食,旅游
旅游
幽默,电影,旅行,摄影爱好者,新闻,网购,美食,好书,设计,搞笑
星座命理,教育就业,投资理财,时尚,文艺,娱乐
小小动画人,大学生
爱电影,日语,爱生活,美食控,日剧控,时尚,旅游
美女,新闻资讯,文艺,美食,汽车
星座命理,新闻资讯,健康,美食,娱乐
安静,爱电影,玩,美女
星座命理,教育就业,健康,体育
旅游,体育
文艺
文艺,健康
视频音乐,名人明星,美食,星座命理
娱乐
文艺
玩,管理,潮流,心理学,安静,听音乐
投资理财,体育,汽车
美女
文艺,星座命理
星座命理,旅游,新闻资讯,健康,时尚
教育就业,星座命理,健康,美食,娱乐
旅游,健康,美女,美食,娱乐,平常心
美食,生活,上网,90 后,羽毛球,好书,美剧,听歌,巨蟹座
名人明星,懒,孩子气,音乐控
怀旧,美剧控,古典艺术,欧美控,美女,健康,旅游,时尚
汽车,IT 数码,娱乐
养生,淡定,浪漫,爱自由,梦想,爱旅行
摄影爱好者,上网,营销,动漫,90 后,唱歌,吃货,穿越火线

星座命理,时尚,娱乐
娱乐
好像很快乐,爱钻牛角尖,情绪化,吃喝玩乐
健康,旅游,美食,文艺,汽车
旅游
快乐,读书,游戏,书法,旅游,电影,学生,游泳
旅游
名人明星,旅游,微博奇葩,搞笑幽默
萌宠,搞笑幽默
文艺
时尚,交友,旅行,亲情
教育就业
汽车,娱乐,幽默,90后,金牛座
游戏动漫,文艺
时尚,娱乐,游戏动漫,美女
美女
视频,会计,旅游,投资理财
旅行是种游离,天秤座,美剧
学校,School,魔法师,Magic,微博
星座命理
打绿粉,dota,足球
听音乐,健康,美食,投资理财,旅游,体育,打球,80后的尾巴,单身,自由
旅游
新闻资讯,美食,IT数码,文艺,娱乐
唯爱源
美女,娱乐,IT数码
时尚,旅游,军事,美食,投资理财,汽车,IT数码
美食
娱乐
娱乐
文艺,娱乐,旅游,IT数码
旅游,时尚,娱乐,美食,巨蟹座,上网,80后,看书,电影,听歌
新闻资讯
游戏动漫
美食,旅游,斯诺克,篮球,体育
佛学,养生,中医
美食,新闻资讯,时尚,文艺
文艺,旅游,健康,时尚,娱乐,自由
瘦身,shopping,爱笑,钢琴,汽车,旅游,健康,新闻资讯,投资理财
投资理财,健康,美女,时尚,娱乐,游戏,旅行,足球,85后
娱乐
教育就业,美女
健康,旅游,娱乐,汽车,睡觉,射手座,80后,宅女,上网
美食

美食,星座命理,北京生活,上网
娱乐,健康,时尚
娱乐
健康
美食,娱乐,投资理财
体育
高三党,肖肖滴灿白党
时尚,服装设计师
吃货,偶稀饭睡觉觉,大学生,平常心,90后,巨蟹座
搞笑幽默
微博奇葩
时尚,健康,星座命理,娱乐,美食
汽车,音乐,交友,旅行,时尚,摄影,创业,吃货,90后,天秤座
求固定组,挂件党,云裳只为一人,秀爷,基三情缘更新版,高三,吃货,倒斗,瓶邪黑花,宅腐双修
健康
音乐爱好者,外贸,玄彬,狗狗,王力宏,时尚,新闻资讯,文艺
投资理财
时尚,数码,IT,电子商务,游戏,互联网,手机,电影
IT数码
旅游,处女座,平常心,偶稀饭睡觉觉,善良
美女
星座命理,旅游,时尚,娱乐,文艺
美食
美食,巨蟹座,摩羯座,大学
游戏动漫,健康,美食
文艺,娱乐,时尚,旅游,美食
二逼,脑残,痴呆,死宅,旅游
上海生活
健康
搞笑幽默
游戏动漫,时尚,投资理财,文艺,娱乐
健康,投资理财,湖北新华
善良,美食
健康,美女,时尚,汽车,旅游
旅游
旅游,游戏动漫,IT数码,健康,新闻资讯,美女,文艺,体育,娱乐
美食
美女
体育
娱乐
体育
IT数码,美食
娱乐,旅游,新闻资讯,时尚,军事

时尚
文艺,体育,汽车,旅游,军事
IT 数码,新闻资讯,电视,自由,乐观开朗,风度翩翩,进取心,责任心强,工作认真,有思想的人
美食
健康,新闻资讯,娱乐,旅游,美食
美女,穿衣美容
摄影,穿衣,搭配,服饰,性感,街拍,美女,潮流,时尚
美食控,单身,校园,喜欢唱歌,90 后但我不脑残,摄影控,独立,交朋友,听歌,旅游
亲情,财经,手机,网购,游戏,乐观
星座命理
偶稀饭睡觉觉,美食,英语,玩儿,学生,小说,电影,宅女,射手座
音乐,囧,时尚,搞笑,旅游,IT,文字,大学生,射手座,语录
旅游
旅游,美图摄影,新闻趣事,星座运势,校园生活
健康,旅游,星座命理
穿衣美容
潮流爱好者,藝術愛好者,摄影爱好者
教育就业,文艺,星座命理,美食,旅游
娱乐,文艺,时尚,美食,旅游
娱乐,美食,IT 数码
时尚
健康,体育,旅游,娱乐,投资理财
写作,营销,睡觉,运动,汽车,电影,囧,水瓶座,幽默,宅
军事,文艺,体育
听歌
电脑,电影迷,电子商务,军事,IT 数码
美女,旅游,军事,IT 数码
投资理财,汽车
娱乐
美食,游戏动漫
喝酒
文艺
旅游
美食,投资理财,健康,时尚,旅游
军事,美食,健康,美女,汽车,阅读,吃货,历史,旅行
娱乐
美食,微博奇葩,美女,新闻趣事,搞笑幽默
旅游,时尚,汽车,投资理财,娱乐
麦霸就是我,经纪人
娛樂
新闻资讯,体育,美女,IT 数码,游戏动漫
运动,旅行,亲情,创业,NBA
娱乐
宅,网络,美食

娱乐
健康,美食,IT 数码,娱乐,80 后,上网,宅女,游戏,摩羯座,听歌
金牛,90 後,矛盾体,旅游爱好者,完美主义,公益,强迫症
娱乐
新闻趣事,广东生活,星座命理,穿衣美容,文艺
美食
荧光色控,甜點控,阳光抑郁型少女,恐怖片女王,外貌协会主席,闷骚的金牛座,漫画,电影,面条控,摄影爱好者
新闻资讯
投资理财,体育
美食,娱乐,教育就业,健康,浪漫,亲情,搞笑,听歌,旅游,音乐
美食,时尚
IT 数码
美容护肤,髮型師,造型师,美发造型师,护肤美容
美食
娱乐
游戏动漫
汽车
美食,娱乐
投资理财
汽车,游戏动漫,旅游,星座命理,军事
美食,睡觉睡到自然醒,健康
旅游,星座命理,美女,时尚,美食
体育
旅行,閱讀,音樂
文艺,星座命理,投资理财,运动,金牛座 O 型,旅游,80 后,听歌
时尚,摩羯座
娱乐
体育
军事
健康,旅游
投资理财
健康,时尚,投资理财,新闻资讯
可爱,美容,减肥,喜欢旅游,看电视,双重性格,娱乐
新闻趣事,旅游,军事,美食,汽车,游戏动漫
微博奇葩,新闻趣事,户外,茶,冷笑话,诗词,爱旅行,快乐,低调,文艺
娱乐
美食,IT 数码
健康,美食,娱乐,时尚,美女
美食
新闻资讯,娱乐,美食,时尚,汽车,广告,摄影爱好者
文艺,自由职业,旅行,交友,好书,体育,看书,幽默,浪漫,平常心
旅游
文艺,IT 数码,时尚

美女,很幼稚,我喜欢唱歌,我是一个咤男噢
游戏动漫,娱乐,宅
海绵宝宝,崔胜铉,摇滚,爱自由,随性,katyperry
浙江生活
揭阳大学生,就业,爱心,教育,公益
生活,上网,90后,阅读,双子座,搞笑,电影,文学,小说,看书
美女
IT数码,体育,健康,文艺,娱乐
汽车
谈心,食,唱K,兼职,乐观,旅游,大学生
娱乐
美女,军事
文艺,时尚,投资理财,旅游,健康,军事
少儿舞蹈,瘦身,街舞,舞蹈
矛盾体,水瓶座,星座控,文字控,纠结体,发呆ing,网购,吃货,睡觉,八卦
娱乐,韩剧,水瓶座加O型血,爱笑,快乐,孩子气,偶像剧
时尚,健康,旅游
多愁善感,文艺,古典,才女
IT数码
萌宠
文艺
时尚
好性格,IT,处女座,电影,90后,上网,唱歌,校园,互联网
老北京粉条,北京特产,老手艺粉条,食品加工,食品生产,粉丝生产,粉条生产,出口贸易,德润通,京一根
幽默,互联网,汽车,旅行,善良,双鱼座,自由职业,宅,美食,80后
搞笑幽默,视频音乐,游戏动漫,汽车,军事,IT数码,随机随遇而安,随和随心随缘
网购,历史,汽车,互联网,处女座,电影,生活,摄影爱好者,小说,旅行
文艺,新闻资讯,健康,美食,投资理财
旅游,美食,星座命理,健康,时尚
旅游
旅游,玩儿
欧美控,我就是我,文字控
文艺,时尚,美食,娱乐
穿衣美容,搞笑幽默,微博奇葩,新闻趣事
体育
花样美男
时尚,美食,教育就业,星座命理,文艺
文艺,时尚,旅游,星座命理
投资理财,美食,星座命理,旅游,健康
宅
时尚,美女,旅游
汽车,投资理财
美食

美食
IT 数码,文艺,娱乐,体育
新闻趣事
张艺兴
娱乐,音乐,80 後,人来疯,豆豆
鄙视男人,海贼迷,有点 2,大胃王一只
90 后,上网,小说,动漫,设计,看书,音乐,摄影爱好者
时尚,娱乐,旅游,美食,文艺
娱乐,天天向上,清爽干净,瑜伽,80 后,天秤座,宅女,看书
视频音乐,新闻趣事,旅游,穿衣美容,名人明星
杂志设计工作,崇尚艺术,热爱生活,向往自由,喜欢旅行,淘宝控,音乐迷,跟随时尚,潇洒浪漫,温馨的家
娱乐
健康
美女,名人明星,搞笑幽默,职场招聘,车世界,体育
七字党
汽车
柴油机发电机组,潍坊柴油机,潍坊华章动力
时尚,健康,旅游,美食
星座命理
新闻资讯,时尚,健康,美食,文艺
手机,微博,吃货,看书,篮球,亲情,创业,动漫,旅游,电影
娱乐,旅游
微博奇葩,新闻趣事
狮子座,游戏,娱乐
美女
汽车,IT 数码
美食,旅游,萌宠,宅女,狮子座,听歌,文字
娱乐
教育就业,投资理财,旅游
美食,旅游,健康,美女,体育
投资理财,美食,旅游,时尚,文艺
内衣,投资理财
名人明星
时尚
休闲,视频音乐,快乐大本营,跑步,热情,大叔控
美食
新闻趣事,八卦杂谈,美图摄影,旅游
美食,娱乐,健康,时尚
电影,听歌,美剧,汽车,善良,旅行,乐观,好性格,小说,读书
体育
运动,狮子座,天蝎座,bigbang 权志龙,学生,宅女,听歌,乐观,吃货一枚,偶稀饭睡觉觉
汽车,美食,军事
搞笑,天秤座,教育,购物狂,育儿,孙燕姿,双鱼女

名人明星,情感生活,成都生活,搞笑幽默,星座运势
连接服务器失败,居里夫人
美食
娱乐,时尚,美食
文艺
娱乐
娱乐
娱乐,美女
娱乐,IT 数码
健康,时尚,文艺,娱乐,游戏动漫
旅游
娱乐
旅行,宅女,吃货
动漫,图片控,语录,传媒,游戏,宅男,新闻,音乐
汽车
教育就业,时尚,星座命理,美食,游戏动漫
电影控,娱乐,美女,游戏动漫
学生,唯美语录,感情语录,美图,文字控
时尚
IT 数码
娱乐
穿衣美容,一腔孤勇
时尚,健康,旅游,美食,娱乐
星座命理,旅游,娱乐,偶稀饭睡觉觉,生活,双鱼座
娱乐
星座命理
欧美控,360 圈红人 YAN,蔡妍 YAN,自拍女王混血控,性感女皇欧美范,性感蕾丝豹纹控,美腿黑丝控,爆乳美臀控,潇艳女皇 YAN,重口味乳神 YAN
扒皮吧,网红扒皮
教育就业,健康,娱乐,美食,旅游
视频音乐
军事
热爱生活,驴友,时尚达人,美食爱好者,教育就业,汽车,投资理财
微博奇葩,旅游,山东生活,新闻趣事
天蝎座 80 后宅男,美女,运动
旅游,时尚,星座命理,娱乐
美食
名人明星
名人明星
吃货,90 后,狮子座,旅游,美食
微博奇葩,陕西生活,搞笑幽默
美食,视频音乐,美女
美女,89 年的 80 后,大学,任性,双鱼女,平常心,善良,双鱼座,努力,爱唱歌
敏感,单纯,爱幻想,爱睡觉,单身,任性,傻

IT 数码,健康
迷茫的小青年
美食
投资理财,汽车,健康,旅游,IT 数码
体育,娱乐
旅游,文艺
投资理财
自由,音乐,旅行,电影
IT 数码
健康,星座命理
美食
心理,旅游,旅行,美食,音乐,电影,听歌,90 后,潮流时尚,音乐爱好者
娱乐
游戏,体育,IT 数码
阅读,宅,文艺,军事
旅游,文艺,时尚,美食,娱乐
星座命理,娱乐
美女
睡懒觉
IT 数码
美女
娱乐
娱乐,网购,处女座,90 后,自由,电影
文艺,教育就业,娱乐
娱乐
美女
泰国生活,新闻趣事,美女,穿衣美容,新闻资讯,汽车,教育就业,旅游
IT 数码
星座命理,健康,旅游,时尚,投资理财
娱乐,狮子座,唱歌,自由
娱乐
娱乐,文艺,美食,时尚
日本,Arashi,嵐
搞笑幽默,新闻趣事,微博奇葩,台州生活,穿衣美容,射手座
互联网,NBA,90 后,搞笑,囧,营销,网络,IT,电子商务,学生
名人明星,旅游,微博奇葩,视频音乐,哈尔滨生活
旅游
娱乐
娱乐
娱乐
电脑,教育就业,爱睡觉,自然,乐天派,心情,时尚
教育就业,健康,旅游,星座命理,文艺,体育,文字,阅读,摩羯座,音乐
投资理财
音乐艺术,文化创意产业,古筝女,艺术学院音乐系,厦大硕士研究生,星河古筝中心,音乐文化

艺术,古筝
娱乐,时尚,健康,新闻资讯,美食
名人明星
美食,旅游,娱乐,体育
幽默,时尚,军事,健康,旅游,文艺
旅游,美食,穿衣美容,广东生活,健康,投资理财,时尚
美女,游戏动漫,时尚,娱乐,旅游
艺术,天秤座,旅游,幽默,90后,音乐,交友,羽毛球,摄影爱好者,大学生
新闻资讯
体育,游戏动漫
汽车
军事,文艺,体育,时尚,IT数码
混合小清新,电影,吃货,网球,文学,美剧,阅读,历史,音乐,宅
彩妆,摄影控,美容护肤,写作,旅游
看书,听音乐
体育,美女
美容服饰
旅游
自由,金牛座,足球,90后
游戏动漫
娱乐
美女,游戏动漫
时尚
军事,robot,movie,sports,Apple,DIY,Arduino,CV,VFX,CG
音乐达人,情感生活,名人明星,武汉生活
健康,旅游,军事,IT数码,体育
旅游
游戏动漫,旅游,美女,美食,娱乐
乐观,摄影,时尚,电影,90后,大学生,宅女,设计,双鱼座,动漫
娱乐
旅游,美食,游戏动漫
体育,新闻资讯,健康,投资理财,旅游,军事
美食
IT数码,美图摄影,校园生活,搞笑幽默,新疆生活,滑板
旅游,微博奇葩,搞笑幽默,广东生活,穿衣美容
娱乐
旅游,新闻趣事,视频音乐,读书分享,动漫
韩庚,superjunior
新闻趣事
SD敢达OL,学习,阅读,书籍,漫画,未定义
文艺
教育就业
时尚,美食,星座命理,娱乐
美女,游戏动漫,IT数码

娱乐,IT 数码
美食
网球,旅游,美食,时尚,真诚,自由
搞笑幽默
教育就业,娱乐,体育,美女
新闻资讯,美女,投资理财,旅游,健康
军事
时尚,教育就业,新闻资讯,军事,娱乐,汽车,投资理财
新闻资讯
星座运势
宠粮,集邮,茶城,古玩,首饰,鲜花,水族,绿植,玉器,珠宝
美食
娱乐
时尚
穿衣美容,微博奇葩,名人明星
美女
文艺
旅游,IT 数码
时尚,正直,诙谐,待解救男青年,夜店喝酒不泡妞
电视迷,紫色
游戏动漫,星座命理,文艺
IT 数码
偶喜欢玩,吃,吃货,90 后,科比湖人
美食,美女
美食
娱乐
旅游
吃,IT 数码
美女,旅行,乐观,80 后,摄影爱好者,天蝎座,好性格,幽默,电影
囧
体育
旅游,双重性格,喜欢旅游,爱,开心
游戏动漫
旅游,健康
美图摄影,爱睡爱宅爱收集,间歇性整理控,三分钟热度
IT 数码,娱乐,美女
娱乐
懒
体育,美食
健康,时尚,旅游,文艺,体育
新闻趣事,搞笑幽默,萌宠,旅游
爱幻想,学习,努力,追梦,减肥,普通青年,娱乐
美女
美食,游戏动漫

电影迷,海贼王,天蝎
星座命理,军事,IT 数码,文艺,娱乐,美女
军事
健康,开朗
微博奇葩,文艺
娱乐,金牛座,偶稀饭睡觉觉
萌物无抵抗,阿猫,阿狗,搞笑
90,游戏动漫,动漫,宅,学生,天枰座,听音乐,宅男,历史,单身
天秤座,90 后,偶喜欢玩,电影,紫色,生活,听歌,亲情,大学生,自由
美女
吃货,天秤座,搞笑,美女,睡觉,80 后,美食,自由,听歌,旅行
体育
娱乐
娱乐
汽车
娱乐,投资理财
娱乐
投资理财
投资理财
美食
美食,IT 数码,文艺,旅游
宅男,吃,囧,睡觉,看书,双子座,足球,动漫,八卦
爱睡懒觉,阅读,吃货
臭算账的,吃喝玩,Leo,爱拍拍,娱乐,旅游
游戏动漫
旅游
旅游
好性格,听歌,幽默,白羊座,乐观,90 后,宅
美食,电影
電影
旅游,文艺,娱乐,时尚,IT 数码
健康
体育
文艺,美女,旅游,美食,娱乐,时尚
时尚,星座命理,文艺,旅游,美食
教育就业
IT 数码
善良,玩儿,吃,娱乐,电影
军事
军事,快乐大本营,设计师,独立,学生一族,健康,偶喜欢玩,娱乐,旅游,体育
汽车,文艺,体育
文艺,闷骚,早睡早起的老人
IT 数码
小说,孩子气

军事
星座命理,旅游,宅女,娱乐,音乐,美食,亲情,阅读,网购,90 后
娱乐
鏃呮父
星座命理,摩羯座
游戏动漫,美食,IT 数码
文艺,体育,美食,旅游,健康,时尚
时尚,健康,星座命理,文艺,娱乐
旅游美食爱好者,交朋友,体育,健康,时尚潮流,书籍,人生,爱美食,市场营销,风行
娱乐
美女,时尚,美食,娱乐,体育
健康,旅游,IT 数码,文艺,时尚
娱乐
名人明星
美女,汽车,投资理财,娱乐,IT 数码
IT 数码,军事,美食
云南中青旅行社,云南旅游,云南中青旅,云南旅游咨询,云南旅游报价
音乐,音乐天才
历史,营销,梦想之路,大美之悦
娱乐,动漫
美容服饰,星座运势,情感生活,搞笑幽默,腐女,大屁股
娱乐
旅游,时尚
旅游
教育就业
美女,游戏动漫,体育
美食
美食,金牛座
浙江生活
处女座
美食
新闻趣事
搞笑幽默
处女座,80 后,宅男
微博奇葩,搞笑幽默
星座命理,娱乐,体育,IT 数码,手机,90 后,乐观,听歌,搞笑,好性格
安静,篮球,唱歌,体育
旅游,时尚
投资理财,美食,健康,旅游,时尚
名人明星,新闻趣事,美食,萌宠,搞笑幽默,我就是我,活泼开朗,看书,摩羯
旅游
攝影,爱电影,吉他,创意,85 后,天秤座,运动,旅游,文艺,体育
爱音乐,爱自由
偶喜欢玩,游戏,美食,90 后

体育
IT 数码,美食,军事,星座命理,旅游
美食,娱乐
时尚,美女,健康,旅游,娱乐
美图摄影
学生,90 后,偶像剧,美食,宅,自由
时尚,教育就业,新闻资讯,健康,美食
旅游,白羊座
娱乐,军事,IT 数码,文艺,体育
体育
娱乐
娱乐
娱乐,亲情
美食,军事
吃
平常心,80 后,大学,读书
体育
游戏动漫,美女,IT 数码
美女,投资理财,军事,旅游,星座命理
娱乐
健康,文艺,美食,投资理财,时尚
健康
微博奇葩,旅游,美食,穿衣美容,吉林生活,时尚
偶稀饭睡觉觉,财经,营销,新闻,吃货,手机,旅游
娱乐
文艺,游戏动漫
娱乐
美食,游戏动漫,时尚,新闻资讯
音乐,吃,网购,手机,90 后
娱乐
游戏动漫
IT 数码
健康,新闻资讯,时尚,教育就业,美食
美食
旅游,美食
創意,健康,美食
花花,华晨宇,iphone,张根硕,狮子座,羽毛球,平常心
游戏动漫
旅游,美食
文艺
会计,坚强,迷糊,赚钱,巨蟹女,幽默,努力,独立,奋斗,爱笑
健康
星座命理
旅游,教育就业,健康,投资理财,时尚

旅游
无聊,高中生,体育,羽毛球,90 後
投资理财,美女,汽车,时尚,体育
旅游,时尚,健康
红楼梦,美食,娱乐,时尚
穿衣美容,搞笑幽默,其他生活,微博奇葩,新闻资讯,健康,游戏动漫,投资理财,旅游
时尚,美食,90 后,白羊座
八卦杂谈,名人明星,视频音乐,校园生活,哈尔滨生活
军事
娱乐
健康,时尚,文艺,军事,投资理财,平常心,吃,巨蟹座
旅游
双鱼座,偶尔神经质,心地善良,宅女一枚,肉食派,唱歌,动漫,旅游
旅游
娱乐,好书,上网,射手座,音乐,旅游,吃货,紫色,幽默,90 后
星座命理,娱乐,时尚,健康,体育
视频音乐,旅游,星座命理,搞笑幽默,穿衣美容,音乐,吃货,传媒,运动
动漫,笑点低泪点低,讨厌欺骗,选择障碍,强悍女,学生
美图摄影,名人明星
搞笑幽默
美女,游戏动漫
投资理财
汽车,投资理财,健康,旅游,娱乐
时尚,教育就业,美食
新闻资讯,教育就业,美食,娱乐,健康
足球,巴萨,魔羯座
新闻资讯,时尚,投资理财,IT 数码,军事
囧,宅女,狮子座,自由,睡觉,吃货,90 后
美食,时尚,健康,星座命理,娱乐
dota,bigbang,爱唱歌,看电视,杭州,志愿者,金牛
美食美酒,米其林餐厅,旅行故事,私人游艇,私人飞机,海岛游,奢华旅游,奢华酒店,时尚生活
游戏动漫,娱乐
时尚
双子女,电影,90 后,向日葵
时尚
娱乐
娱乐
旅游,游戏动漫
美食
IT 数码,旅游,新闻资讯,美女,娱乐,体育,军事
时尚,美食,健康,旅游,文艺
微博奇葩
旅游,游戏动漫,文艺,体育,IT 数码
投资理财,新闻资讯

娱乐
美食
星座命理,健康,教育就业
汽车
游戏动漫
旅游,文艺
娱乐,美食
游戏动漫
体育
天生我才必有用
旅游,读书
军事,教育就业,时尚,投资理财,健康
健康,服饰,减肥,80后的尾巴,淘宝控,古典艺术
美食,娱乐,文艺,投资理财,教育就业
游戏动漫,汽车
美食,新闻资讯,美女,游戏动漫,IT数码
新闻资讯,健康,星座命理
时尚,新闻资讯,健康,教育就业,文艺
时尚
美食,健康,时尚
娱乐
旅行,营销,天秤座
美食
蚊子思密达
爱自己,好书,DIY,睡懒觉,90后但我不脑残,Music,低调,宅女,金牛,90後
手机,动漫,吃货,音乐,网络,体育,游戏,运动,自由,90后
娱乐
美女,旅游,汽车,星座命理,IT数码
新闻资讯,旅游,IT数码
低调,北京,IT互联网,猎头
文艺,游戏动漫,IT数码,体育
游戏动漫,IT数码,汽车,旅游,星座命理
读书分享
技术宅,巴萨控
娱乐
娱乐,IT数码,新闻资讯,旅游,体育
新闻资讯,旅游,IT数码,美女,星座命理,文艺
汽车,旅游,IT数码
广东生活,穿衣美容,旅游,美食
娱乐
汽车,旅游,IT数码,投资理财,美食,文艺
娱乐
汽车,时尚,旅游,IT数码
SEHUN,行星饭,EXO,吃货一枚,90后,海绵宝宝,射手,娱乐

健康, 星座命理, 文艺, 新闻资讯, 旅游, 美食
文艺, 历史爱好者, 历史, 学生
娱乐
游戏动漫, 旅游, IT 数码
娱乐
新闻趣事
教育就业, 时尚, 旅游, 健康
汽车, IT 数码, 新闻资讯, 教育就业, 星座命理
娱乐
娱乐
汽车, 星座命理, IT 数码
汽车, 军事, 娱乐
汽车, 星座命理, IT 数码
投资理财, 旅游, 汽车, 娱乐, 星座命理
名人明星
微博奇葩, 美女, 搞笑幽默
美食, 时尚, 旅游, 投资理财
时尚
健康, 时尚, 新闻资讯
娱乐, 健康, 教育就业, 汽车, 旅游, 美食
娱乐
法国, 爱自由, 打球, 浪漫, 搞笑, j 家, 亲情
新闻资讯, 美食
健康
娱乐
IT 数码, 美女, 新闻趣事, 搞笑幽默, 成都生活
游戏动漫
娱乐
汽车, 旅游, IT 数码
浪漫, 爱情, 校园, 公关, 主持, 广告, 传媒, 大学生, 交友节目, 北京高校
微博奇葩
创业
旅游, 美食, 穿衣美容, 搞笑幽默, 湖南生活
娱乐
娱乐
天然呆, 受, 卡姆辛, 傲娇属性, 萌物无抵抗, 不要脸, 单蠢, 轻音乐, 不靠谱骚年, 腐女
娱乐
娱乐
时尚
美女, 旅游, 投资理财, 美食, 体育
娱乐, 摩羯水瓶座, 吃货, 动漫, 美食
娱乐
时尚, IT 数码, 新闻资讯, 旅游, 美食
时尚, 美食, IT 数码

教育就业,投资理财,美食,IT 数码
娱乐
文艺,美女,时尚
美女,美食,体育,健康,星座命理,文艺
娱乐
娱乐
星座命理,娱乐,时尚,旅游,IT 数码
娱乐
娱乐
娱乐
娱乐,美食,时尚
娱乐
娱乐
娱乐
娱乐
汽车,新闻资讯,旅游,IT 数码
健康,时尚,IT 数码,旅游,军事,文艺
娱乐
娱乐
娱乐
IT 数码,新闻资讯,汽车,旅游
旅游,体育,时尚,星座命理,IT 数码
娱乐
时尚,军事,娱乐,新闻资讯,旅游,星座命理
娱乐
娱乐
时尚,旅游,新闻资讯,汽车,IT 数码
新闻资讯,汽车,IT 数码,健康,星座命理,文艺
新闻资讯,IT 数码,汽车,旅游,军事
娱乐
娱乐
娱乐
旅游,军事,体育,游戏动漫,投资理财,IT 数码
深大学生家教,兼职
教育就业,健康,IT 数码,文艺,投资理财,旅行,电影,80 后
娱乐
胡思乱想,幻想,宅女加腐女
时尚,旅游,汽车,星座命理,IT 数码
新闻资讯,旅游,IT 数码,游戏动漫,美食,体育
娱乐
娱乐
娱乐
娱乐
娱乐

旅游,新闻资讯,汽车,IT 数码
时尚,美食,文艺,新闻资讯,美女,旅游,IT 数码
娱乐
新闻资讯,旅游,IT 数码,健康,星座命理,文艺
双子,微笑,旅游
娱乐
新闻资讯,旅游,IT 数码,健康,教育就业,美食
健康,时尚,旅游,IT 数码
旅游,美食,文艺,时尚,投资理财,IT 数码
时尚,IT 数码,旅游,美食,文艺
投资理财,健康
汽车,美食,旅游,美女,体育
娱乐
新闻资讯,游戏动漫,军事,娱乐,体育
美食,旅游,穿衣美容,视频音乐,新闻趣事
萌宠,微博奇葩,搞笑幽默
萌宠,视频音乐
娱乐
文艺
射手座,80 后,音乐,篮球,足球,搞笑,汽车,投资理财,现货黄金投资,财经
娱乐
视频音乐
软件,wow,生活大爆炸,猫咪,科幻,小清新,人生,疯狂,腹黑,娱乐
新闻资讯
星座控,处女座的 MM,韩剧控,皮肤护理专家,美食控,瑜伽达人
装逼狗
密室,网球,赛车,懒,睡觉睡到自然醒,爱吃,动漫
娱乐,游戏动漫
文艺
娱乐
新闻趣事
福建生活
游戏动漫,汽车,旅游,新闻资讯,体育
安静,旅游,设计,忧郁的双鱼
文艺,IT 数码
狮子座,自由
娱乐,旅游,美食,体育,游戏动漫
旅游
美女
美食,旅游,新闻趣事
旅游,微博奇葩,搞笑幽默,新闻趣事
汕头生活,宠物图集,美容服饰
旅游,汽车,足球,双子座,皇马,Music
旅游摄影,看电影,幻想,聊天,军事,美女,旅游

投资理财,军事,娱乐,健康,旅游,IT 数码
娱乐
旅游,IT 数码
娱乐
新闻资讯,旅游,星座命理,健康,汽车,军事
健康,星座命理,IT 数码
娱乐
娱乐
娱乐
娱乐
新闻资讯,时尚,IT 数码
星座命理,文艺,旅游,体育,汽车,美食,IT 数码
历史,矛盾,大学生,90 後,科技,网购,三国杀,文化,心理学,宅
娱乐
娱乐
时尚,汽车,旅游,娱乐,文艺,创意设计,设计师,宅男,狮子女
投资理财,美食,文艺
野性,钢琴,教师
教育就业,美食,体育
娱乐
健康,时尚,教育就业
娱乐
时尚,美食,新闻资讯,IT 数码,文艺
汽车,星座命理,新闻资讯,旅游,IT 数码
娱乐
健康
新闻资讯,汽车,旅游,IT 数码
美食,娱乐,游戏动漫,时尚,旅游,健康,美食
娱乐
娱乐
娱乐
文艺,旅游,Love,向往美好,个性使然
流汗,允儿,Nba,F1,睡觉,零食,幽默,音乐,篮球,足球
时尚,文艺,文字,偶喜欢玩,吃货
加拿大生活
星座命理
体育
新闻资讯,汽车,时尚,旅游,IT 数码
游戏动漫
穿衣美容,美食
娱乐,旅游,汽车,时尚,星座命理
娱乐,游戏动漫,美女
投资理财
新闻资讯,旅游,IT 数码

投资理财,美食,IT 数码
bigbang,BIGBANG
文艺,IT 数码,投资理财
文艺,体育
娱乐,体育,听歌
娱乐
IT 数码
健康,娱乐,时尚
美食
高中生,射手,旅游,美食,时尚,娱乐
火影忍者,三国杀,玩,快乐,旅游,游戏动漫,投资理财,IT 数码
娱乐
娱乐,IT 数码,军事,星座命理,美食
摄影控,武艺,麦霸,写字,体育,其他生活,美食,旅游,视频音乐
爱时尚,爱生活,时尚,自由,音乐,宅女,阅读,电影
美食
IT 数码
军事
济源生活
体育
星座命理,娱乐
爱情,听歌,好性格,善良,文字,音乐,静
娱乐
天秤座,90 后,好性格,宅女
文艺,收藏,文化
投资理财,运动,旅游,爬山,音乐,创业
阅读,吃货,旅行
旅游
时尚
汽车
旅游,美食,视频音乐,微博奇葩,搞笑幽默
健康
娱乐
时尚,腐女,同志,時尚,肌肉控,刷图党,纹身 tatto,欧美风
美食,旅游,健康,时尚,娱乐
娱乐
教育就业,健康,美女,时尚,娱乐
游戏动漫,军事
自由,舞蹈
美女
美食
微博控,创意,音乐,电影,浪漫,财经,英语
美食
文艺

美食,旅游,微博奇葩,新闻趣事,搞笑幽默
军事
文学,美女,活泼开朗,狮子座
星座命理
时尚,旅游,星座命理,美食,娱乐
音乐,美食,旅游
静默,散文,历史,偶稀饭睡觉觉
娱乐
旅游,娱乐,唱歌
体育
游戏动漫,美食,汽车,IT 数码,娱乐
娱乐
文艺,娱乐,体育,旅游,美食
穿衣美容
军事
搞笑幽默,旅游,美食,新闻趣事,摩羯座,无肉不欢
美女
微博奇葩,IT 数码
IT 数码
旅游,投资理财,文艺,体育,篮球 NBA,90 后,自由,偶喜欢玩
娱乐,美剧
美图摄影,读书分享,美食,新闻趣事,搞笑幽默
视频音乐,美女
星座命理,RTA,听歌,音乐,水瓶座,宅,学生,90 后
新闻资讯,体育,IT 数码,汽车,投资理财
娱乐,游戏动漫
游戏动漫
河北生活,旅游
北京生活,校园生活,体育资讯,体育,美女
视频音乐,旅游,名人明星,穿衣美容,微博奇葩
搞笑幽默,微博奇葩,美女,其他生活,旅游
汽车人变形,昌国路车神,射手座 O 型血
体育,娱乐
美女,健康,IT 数码,文艺,娱乐
娱乐
娱乐
萌宠,维尼
美食
旅游
汽车
健康,旅游,处女座,乐观
文艺
文艺,娱乐,时尚
娱乐,自由,美食,旅游,大叔控,生活大爆炸,爱幻想

娱乐
茶,孙燕姿,烹饪,睡觉睡到自然醒,学生一族,独立,音乐爱好者,热情,旅游摄影,K 歌
旅游
文艺,旅游,美食,爱吃鬼、懒虫。
视频音乐,名人明星,微博奇葩,北京生活,美女
旅游,O 型血,水瓶座
搞笑,平常心,旅游,微博,90 后,好性格
美食
衬衫控,不睡醒会死星人,处女座,看书,音乐
游戏动漫,双鱼座
健康
信息安全,电子商务,互联网,电子政务,产权交易行业,Java 研发工程师,田径运动员,业余摄影爱好者,网络安全,Java 软件工程师
科士威,成功
健康,IT 数码,美食
汽车
娱乐,双鱼座的幻想女,歌嫣,杰娜,美食,90 后
星座命理,健康,旅游,娱乐
八卦,幽默
娛樂,周杰倫
美女,美食,苍井空,娱乐
娱乐,IT 数码
游戏动漫,教育就业,旅游
体育
娱乐
广州生活
物流,旅游,IT 数码,美食,汽车
时尚
时尚,健康,旅游,美食,娱乐
时尚,好书
文艺
时尚
音乐,旅游,文艺,体育
现代时尚生活,绿能建筑,新能源开发,节能环保低碳,建筑材料,绿色生态健康
A 股
文艺,新闻资讯,星座命理,娱乐,懒,沉默
旅游,健康,军事,体育
平常心,时尚,90 后,听歌,唱歌,运动
纠结
星座运势,美食
瘦身,美容护肤,化妆品,购物,听音乐,潮流,感性,时尚,投资理财
体育
时尚
美剧控,摄影,睡觉,穿衣美容,微博奇葩,名人明星,时尚,旅游,美食,星座命理

汽车,军事
星座命理,旅游,健康
校园生活,旅游,阳光
健康
体育,音乐,宅,传媒,摩羯座,历史,运动,NBA
汽车,投资理财,健康,美女,旅游,娱乐
新闻趣事,微博奇葩,搞笑幽默,听音乐,胡思乱想,电影爱好者
投资理财
闷骚,无聊,o 型血,体育
IT,好性格
美女,IT 数码,时尚
健康
文艺,天秤座,搞笑,电影,旅行,吃货,时尚,音乐
网购,90 后
美图摄影,搞笑幽默
旅游美食爱好者
时尚
死 loli 控
健康
消防兵,山东,武警,军人
娱乐
读书分享
深圳生活,钢笔画,动漫画背景,跆拳道
星座命理
游戏动漫
我用一手心的温
健康
健康,娱乐,教育就业
韩国,EXO,看书上网听音乐,韩国音乐,钢琴,北京,娱乐
美女,学生
娱乐
旅游,文艺,娱乐
投资理财
娱乐
音樂,诗词,美食,向日葵,LM,鍾漢良
视频音乐,夜猫子,学生一族,小说,IT
淡干海参,拉缸盐海参,盐干海参,蓬莱长岛海参,山东长岛,海鲜,海参,长岛,野生
媒体
健康,星座命理,旅游,体育
美食,叭爪鱼,小咪
游戏动漫,IT 数码,投资理财,汽车,时尚
时尚,星座命理
时尚,123,美食,宅女,紫色,微博,听歌,水瓶座,吃货,90 后
微博奇葩,美食,旅游,星座命理,上海生活,电影

时尚,模特控,旅行,摩羯座,80 后
大爱 2ne1
旅游,美食,娱乐
诗歌,美剧控,双重性格,文艺,美食,旅游
投资理财,时尚
魔羯座,心情,瘦身
搞笑幽默
旅游,健康
看着月亮好睡觉,微小的狂热,机车
健康,时尚,新闻资讯,星座命理,旅游,娱乐
浪漫,善良,交友,摄影,上网,文学,双子座,好性格,幽默,旅游
文艺
游戏动漫,美女,健康,星座命理,娱乐
健康,星座命理,IT 数码,金牛座宅男
设计,手机
情感生活,名人明星
游戏动漫,星座命理,军事
搞笑幽默
体育
上网,双鱼座
投资理财,交友,设计
星座运势,视频音乐,新闻趣事,美容服饰,名人明星
健康,教育就业,投资理财,军事,IT 数码
猫咪控得很厉害,伪文艺宅女,睡觉,旅游,平常心,摄影,双鱼座,文字,美食,音乐
IT 数码
IT 数码,文艺,体育,旅游,汽车
娱乐
双鱼座,自由,乐观,睡觉,偶喜欢玩,数码,上网,校园,90 后,微博
娱乐,游戏动漫
生活,睡觉,阅读,美女,唱歌,读书,旅行,宅,90 后,巨蟹座
阳光,散步,服饰,美食,时尚,听歌,自由,投资理财,健康,旅游
娱乐,IT 数码,军事,美女
上网,搞笑,篮球,睡觉,90 后,手机,巨蟹座,幽默
美食,IT 数码
文艺,旅游,美食,时尚,娱乐,睡觉,音乐
搞笑幽默
IT 数码
旅游,美食,文艺
新闻资讯
娱乐
娱乐
星座命理,时尚
投资理财,新闻资讯,旅游,IT 数码
旅游,名人明星,微博奇葩,新闻趣事

旅游,美女,穿衣美容,娱乐
军事,IT 数码
手机,白羊座,游戏,90 后,平常心,听歌,音乐,小说,F1 最爱阿隆索,上网
视频音乐
南京生活
国学文化,DIY,翻译口译,乐活 LOHAS,素食环保低碳,电影,音乐,旅游
呼叫中心,ContactCenter,客户关怀
游戏动漫
娱乐
动漫,123
动漫,视频音乐,校园生活,搞笑幽默,潮州生活
穿衣美容
时尚,星座命理,美食,健康,旅游
健康,亲情,浪漫,美女,吃,好性格,幽默,90 后,旅游,娱乐
旅游
旅游,星座命理,美女,时尚,健康,读书,射手座
新闻资讯,教育就业
娱乐,星座命理,健康,旅游,美食
健康
美食,旅游,健康,娱乐,投资理财
美女
台湾生活,3C 達人,夜店,時尚,自然醒,麻豆
文艺
旅游,IT 数码,文艺,娱乐,时尚
面相风水学,星座控狮子女,淘宝控,旅游,美食
睡觉,学生,宅,自由,90,宅男,山东生活,搞笑幽默
时尚,旅游,健康,娱乐,美食
健康
健康,旅游,文艺,娱乐,星座命理,音乐爱好者,写作,善良,文学,天蝎座
星座命理
时尚
穿衣美容
名人明星,美食
贵阳生活
宅女,紫色
旅游,健康,美食,文艺,双子座,文学,90 后
体育
IT 数码
高跟鞋控,A 型天平女,易喜易怒易纠结
紫色
IT 数码,娱乐
体育
平常心
投资理财,时尚,文艺

健康,管理,情感,美术,会展策划,摄影爱好者,娱乐八卦
购物,睡觉睡到自然醒,幻想,室内设计,生活,低调
自然,爬山,休闲,旅游摄影,环保,绘画,好性格,足球,幸福,看电影
文艺,新闻资讯,健康,投资理财,星座命理
IT 科技,移动科技,移动设备,电脑,互联网创业,移动互联网创业,移动互联网,创业者,创业
娱乐
网购
汽车,旅游,美食,军事,IT 数码
投资理财
娱乐
爱旅游,90 後,陈奕迅,科幻
文艺,星座命理,旅游,时尚,娱乐
新闻资讯,汽车,IT 数码,军事,体育
旅游
娱乐
娱乐
旅游,文艺,娱乐
健康,文艺,旅游,时尚,美食
旅游,户外,时尚达人,咖啡,旅游爱好者,自行车,设计,旅游美食,旅游摄影,爱音乐
旅游
游戏动漫,美食,汽车,IT 数码,体育
汽车
星座命理
旅游,投资理财,时尚,健康,星座命理
新闻资讯
紫色
旅游,星座命理,时尚,美食,娱乐
健康
名人明星
娱乐
文艺,方言,昆虫,诗词,书法,国学
耳目精灵
IT 数码,体育
新闻资讯,美女,娱乐,时尚,美食
娱乐,体育,美女
娱乐
投资理财
微笑,射手女,游戏动漫,吃货一枚
美食
旅遊
旅游,汽车
宅,天秤座,较内向,外温柔内坚强,爱家人,慢性子,懒会看时候,喜欢网购,不追星
美食,淡定
新闻趣事,旅游,美食

学习,健康,旅游,文艺,平常心
汽车
游戏动漫
营销
学生一族,爱音乐,听音乐
文艺,时尚
射手座,爱音乐,爱自由,烹饪,恐怖片,快乐大本营,静静的听歌,望着天空发呆,散步,涂鸦
工作
阅读
旅游,美食,视频音乐,搞笑幽默,体育
爱笑,玩,紫色,聊天,电脑,音乐爱好者,蓝色,学生一族,美食,娱乐
搞笑幽默
军事
娱乐,教育就业,文艺,汽车
旅游
穿衣美容
IT 数码,时尚,汽车,美女,娱乐
美女,时尚,文艺,健康,娱乐
健康,健康饮食,健康达人,健康生活,养生,健康知识,父母保健
时尚
旅游,文艺,投资理财,游戏动漫,健康
新闻资讯,旅游,文艺,吃,金牛座
文艺,美食,细节控,胡思乱想星人,爱玩爱闹爱折腾,二次元,恋物癖,音乐,囧,天秤座
足球
体育
时尚,健康,美食,星座命理,娱乐
健康,旅游,时尚,文艺,美食
偶稀饭睡觉觉,摩羯座,宅女,网购,电子商务,吃,偶喜欢玩
投资理财
浪漫
微博,幽默
美食,时尚,娱乐,健康,星座命理
股票,80 后
南京生活,日剧
美食,学生,汽车,偶喜欢玩,吃,90 后,白羊座
只嗳她
浪漫,自由
营销,好性格,自由,80 后,汽车
文艺
时尚,美女,美食,健康,娱乐
文艺,听歌,动漫,好书,宅男
娱乐
视频音乐
投资理财,美食,文艺,时尚,健康,星座命理

微博,90 后,看书,搞笑,善良,时尚
单身,90 後
时尚,射手座
汽车,时尚
游戏动漫,健康,旅游,懒,迷糊,简单
时尚,巨蟹座
穿衣美容
美食
时尚,健康,投资理财,教育就业
手表,新闻资讯,游泳,篮球,白羊男,旅游美食,心理学,星座命理,汽车,时尚
健康,美食,文艺,游戏动漫,旅游
教育就业,英语,对外经贸大学,财经,创业,平常心,旅游
爱好极其广泛,工作狂人,时尚新潮,自信坚强乐观,善良有爱心,娱乐,投资理财,IT 数码,美食,自由
健康,健康饮食,健康达人,健康生活,养生,健康知识,父母保健
语录,校园
搞笑幽默,IT 数码
健康,旅游,星座命理,美食
大学,旅游爱好者,投资理财,新闻资讯,教育就业,军事,IT 数码
美食
美食,房产,美女,宁波生活
旅游
汽车,文艺,军事,娱乐,体育
旅游
偶喜欢玩,自由职业
历史
美女
广州生活
文艺,健康,新闻资讯,星座命理,美食,美食
投资理财,军事,美食,体育,美女
美女,汽车,IT 数码,娱乐,历史,英语,搞笑,时尚,90 后,音乐
交友,爱电影,搞笑,工作,旅游,体育
体育,军事,娱乐,投资理财
b 型血,蓝色,许嵩,music,美食,游戏动漫
娱乐
旅游
旅游
美女,娱乐
情绪化,美食,运动,小说,浪漫,好性格,爱情,诗歌,青岛生活,疯狂
123
美食,旅游,娱乐
旅游
娱乐
游戏动漫

时尚
美食,文艺,娱乐,星座命理,时尚
搞笑幽默,语录,美剧,新闻,小说,123,电影
狮子座,小说
晒货,真人秀,时尚街拍,ELLE,YOKA,VOGUE,时装周过客,models,newyork,爱时尚
教育就业,文艺,健康,时尚,娱乐
旅游,美食,体育,IT 数码
fashion,赚钱,胡思乱想,睡觉睡到自然醒,小资,时尚潮流,偏执狂,桌球,iphone
宅
搞笑幽默,山东生活,美食,新闻趣事,名人明星,爱睡觉,双重性格,电视剧,人力资源,处女座
看书,电影,英语,旅游,音乐,唱歌,听歌
健康饮食,健康达人,健康生活,养生,健康知识,父母保健
娱乐
名人明星
创业,旅行
b 型血
体育
体育
微博奇葩,星座命理,安徽生活,旅游,视频音乐
搞笑幽默,星座命理,名人明星,美食,穿衣美容
美女,健康,投资理财,汽车,IT 数码
星座命理
二次元症候群,爆破手,初三党,特摄控,假面骑士,各種宅,渣绘大师,同人无敌
旅游
娱乐,IT 数码
娱乐,阅读,大学,吃货,90 后
手机,狮子座
游戏动漫
体育,汽车,旅游
旅游,美食,文艺,睡觉,偶喜欢玩,紫色,听歌,乐观,90 后,天秤座
娱乐,劲跑
视频音乐
娱乐
新闻资讯
英语
华夫饼,咖啡,饮品
搞笑幽默,星座命理,美女,书,喜欢旅游,江苏生活
汽车,体育,美食,星座命理
健康,时尚
旅行,校园,网络,宅
游戏动漫
汽车
美食
胡夏,许嵩,音乐,00 后,上网

重口味
健康饮食,健康达人,健康生活,养生,健康知识,父母保健
开心最重要,羽毛球,足球,幽默
文艺,语录,善良,写作,互联网,好性格,创业,文字
吃货
军事
健康,时尚,旅游,娱乐
星座命理,穿衣美容
微博奇葩
梦想终将会实现
山东生活,文艺
汽车
旅游
金在中我爱你,music,金希澈,艺术,朴有天,音乐达人,音乐爱好者,东方神起,爱幻想
美女,健康
健康,旅游
微博奇葩,搞笑幽默,旅游,视频音乐,广东生活
旅游,美食,文艺
星座命理
美女
美女,投资理财,IT 数码,新闻资讯
视频音乐
看书
搞笑幽默,军事,体育,新闻资讯
视频音乐,汽车,搞笑幽默,90 後,爱音乐,娱乐
游戏动漫,星座命理,美食,娱乐
品牌,服装搭配,服装行业,服装批发,服装网店,创业,fashion,旅游
星座命理,健康
旅游,好性格
偶稀饭睡觉觉,手机,吃货,123,搞笑
美女
互联网
摩羯座,八卦,旅游,吃,80 后
美食,天枰座,朴灿烈,李东海,李赫宰,kris,偶喜欢玩,听歌,90 后,微博
娱乐
游戏动漫,各种宅,白羊座,无节操的货,coser
微博奇葩
爱 2PM,爱 Nichkhun,偏执狂,时尚
娱乐
游戏动漫,IT 数码
投资理财
IT 数码,汽车,游戏,手机,数码,IT,psp,学生
体育,幽默,善解人意开心果,美食,相声,聚会控,音樂,体育迷,篮球爱好者,足球迷
娱乐

体育
体育
星座命理
美食
旅游
美食,旅游,健康,时尚
旅游,新闻资讯,文艺,星座命理,教育就业
美女,旅游,美食,体育,娱乐
IT 数码
旅游,星座命理,魔羯座
90 后,音乐,汽车,娱乐,篮球,网络,运动,宅
美食
健康,汽车,投资理财,体育,娱乐
美食
西安交大,运动,幽默,娱乐,旅游
星座命理
体育馆,羽毛球场,练习场软件,高尔夫软件,高尔夫俱乐部,练习场,高尔夫,体育
娱乐
美食,时尚,娱乐
时尚
娱乐,旅游,美食,时尚,美女
星座命理
军事,美食,投资理财,娱乐,健康,时尚,体育
旅游,美食
美女,体育,IT 数码,汽车,娱乐
美食,旅游,矛盾体,巨蟹女,迷糊,敏感
文艺
IT 数码,健康,美女,时尚,娱乐
IT 数码
金属,黑白,胶片,摄影,白天的背面
梦想,学生,努力,90 後,爱旅游,爱电影,玩,旅游
天枰座,快乐大本营,手机看小说,文艺,娱乐
旅游,汽车,娱乐
搞笑
体育
体育,文艺,军事,教育就业,健康
平常心,善良,美食,自由,听歌,互联网,80 后,乐观,数码,网购
美女,娱乐,幽默,音乐
旅游,穿衣美容,萌宠,美食,新闻趣事
新闻资讯,汽车,娱乐,旅游,IT 数码
名人明星
淡定,敏感,纠结,跑步,旅游,时尚,汽车,娱乐
文艺,军事,游戏动漫
爱笑,简单

美食,旅游,体育
娱乐,美女
投资理财,文艺,健康
吉安生活,美女,搞笑幽默,时尚,游戏动漫,体育,娱乐
美食,旅游,宫崎骏,美剧
IT 数码,美食,时尚,教育就业,游戏动漫,健康,娱乐,动漫控,看书
美容服饰
时尚,投资理财,军事,IT 数码,文艺
健康,旅游,美食,体育,投资理财
新闻,时尚,自由职业,乐观,文字,营销,创业,交友,财经,文学
星座命理,美女,娱乐
文艺,星座命理,旅游,美食
司法行政
看电影,听音乐,发呆,水瓶女,爱自由,吃喝玩乐,美食控,互粉,美食,星座命理
伟业我爱我家,我爱我家,房产经纪人,房地产,二手房,西城,5i5j,itme,HITFM,SUM41
美女
美食
娱乐
旅游,狮子座,学生,历史,运动,90 后
健康,军事,体育
娱乐,体育,偏执狂
旅游,美食,汽车,体育,投资理财
星座命理
体育
IT 数码,军事
交友,动漫,自由,吃货,听歌,吃,白羊座,音乐
娱乐
美食,汽车,娱乐,旅游,生活大爆炸,金牛,shopping
搞笑幽默
搞笑幽默
娱乐
投资理财,旅游,军事
名人明星
美食,美女
交友
体育
爱音乐,双鱼,英语,五迷
时尚,IT 数码
旅游,感性,90,睡懒觉,团购,无聊,爱音乐,发呆,腐女,美食
旅游
四川生活
娱乐
娱乐,军事,时尚,汽车,IT 数码
汽车

娱乐
美女,娱乐
艺术,网购,生活
天蝎,同志,生活大爆炸,看电视,Music,爱吃,睡懒觉,睡觉睡到自然醒,娱乐
水瓶座
工作,文字控,浪漫控,真心控
娱乐
汽车
娱乐,健康,旅游,美食
巨蟹座,财经
新闻趣事,创业,大学生,乐观,校园,90 后
汽车,新闻资讯,时尚,IT 数码,文艺
好书,偶稀饭睡觉觉,天蝎座
IT 数码,游戏动漫
旅游,新闻资讯,美食,军事,文艺
新闻资讯,教育就业,汽车,体育,军事
游戏动漫,旅游
娱乐,健康,旅游,美食,体育
娱乐,美食,体育
健康
IT 数码,教育就业,汽车,军事,娱乐
体育
娱乐
旅游,娱乐,文艺,投资理财
美食,旅游
玩,IT 数码
汽车
旅游
文艺,边看萌芽边听歌
体育,旅游,投资理财,事业男,天蝎座,汽车,NBA
新闻资讯,投资理财,时尚,美食
娱乐
星座命理,文艺,投资理财,教育就业,旅游
健康,美食,文艺,体育,娱乐
爱情,我爱睡觉,偏执狂傻妞,射手,偏执狂,情绪化,iphone,健康,美食
数码,IT,體育,音乐控
天蝎座,健康,美食,文艺,旅游
健康,旅游,军事,体育,游戏动漫
投资理财,军事,美食,星座命理,美女
听歌,a 半开朗
投资理财
体育资讯,电影迷,爱美食,欧美音乐,体育,宅男,听歌
美女,娱乐
体育,IT 数码,美食,健康

浪漫,大学生,巨蟹座,80 后,运动,体育,唱歌,校园,幽默
健康
旅游,教育就业
处女座,读书,80 后,英语,幽默,旅游,语录,上网
健康
文艺,体育
旅游,美食,投资理财
星座命理,时尚,旅游,美食,文艺,娱乐
娱乐,囧,交友
视频音乐,偶稀饭睡觉觉,音乐,宅,阅读,运动,平常心,看书
旅行,校园
娱乐
美食,自由控,文字控,旋律控,情绪控
娱乐
时尚,美食,娱乐
教育,河北生活
文艺
星座命理,美食,歌手
历史,英语
娱乐,IT 数码,美食,旅游,星座命理
湖南生活
情感生活
娱乐
乐天派,BIGBANG,单身,爱睡觉,舞蹈演员,90 后,电影,吃货,天蝎座,艺术
穿衣美容
网购
晚睡强迫症,旅游,山地车,人力资源,奢侈品,创业,水瓶座
旅游
吃货,天蝎座,开朗,爱美食哦
IT 数码,玩儿
宠物图集,强迫症,天蝎座,文字,八卦女一枚,单眼皮小眼睛
星座命理
旅游,美食,名人明星,视频音乐,娱乐,体育,躲在角落看世界
星座命理
娱乐
娱乐
新闻
奢侈品,香港代购,90 后,购物达人
新闻资讯,汽车,旅游,军事,IT 数码
美食,星座命理,时尚,教育就业,娱乐
看电影,旅游,美食,文艺
娱乐
可惜我是金牛座
体育

娱乐
投资理财,军事,IT 数码,文艺,体育
美女,IT 数码
电影
美女,娛樂,倾计(聊天),识朋友,玩
新闻资讯,游戏动漫,时尚,体育,娱乐
新闻资讯,时尚,星座命理,美食,娱乐
微博奇葩
星座命理
天蝎座,90 后,北京,互粉,开朗
互联网,广告
美食,汽车,时尚,旅游,IT 数码
美食,时尚,星座命理,喜欢听歌,纠结,睡觉,娱乐,偶像剧,神经大条,游戏
希饭,poppin
旅游,水瓶座
单身,活泼,创意,旅游爱好者,打篮球,幸福,开心,美食,健康,聊天
教育就业,旅游,健康,军事,新闻资讯
娱乐
美食,娱乐,唱歌,音乐,热爱主持,语录,生活,读书,数码,动漫
旅游
美食,军事
时尚,文艺,星座命理
娱乐
IT 数码
投资理财,教育就业
耽美,中国风,古风,古风音乐,动漫,听歌,看书,90 后,天蝎座
投资理财,美食
娱乐
美食,其他生活
美食
美食,新闻趣事,视频音乐,搞笑幽默
旅游,星座命理,游戏动漫,时尚,美食
时尚,娱乐,EXO 控,偶稀饭睡觉觉,双子座,幽默,睡觉,吃货
文艺,听歌
名人明星
围巾控,MAX 控,破产姐妹迷,吃货扁扁
旅游
投资理财,游戏动漫,美女,星座命理,娱乐
搞笑幽默
美食,新闻趣事,旅游,搞笑幽默,体育,美女
旅游,文艺,时尚,新闻资讯
密集恐慌症,强迫症,幻想症,神经质
旅行,动漫
NBA,双鱼座

gay,八卦杂谈
美食,搞笑幽默
体育,游戏,平常心,听歌
体育
旅游
易经,Android,打篮球,法律,美国,律师
四川生活
军事,美食,健康,汽车,娱乐
美食,娱乐,VIP,GD,bigbang,韩剧迷,YG 妹子
美食,游戏动漫,美女,星座命理,娱乐
健康,时尚,投资理财,文艺
新闻趣事,篮球,NBA 迷,修行路上
旅游,教育就业,美食,时尚,投资理财,金融系在读生
波雅汉库克,妮可罗宾,80 后,历史,囧,旅游,天蝎座
双子座 A 型,开朗,轻音乐,投资理财
名人明星,幻想,香港,笑,钢琴,EXO,迷糊,自由,校园,爱情
东莞生活,美剧迷,孩子气,偶像剧,天秤座
娱乐
旅游,文艺,新闻资讯,时尚,娱乐
汽车
美食
旅游
娱乐
广告,策划,插画,红酒,服饰,英语,紫色,旅游
巨蟹座
创业,网络
体育,旅游,美食
健康,娱乐
旅游,美女,时尚,游戏动漫,IT 数码
星座命理,文艺,娱乐
投资理财
美食
IT 数码,体育,娱乐,汽车
旅游,名人明星,穿衣美容,新闻趣事,视频音乐
时尚,旅游
shopping,美女,体育,投资理财,IT 数码
双鱼座,偶喜欢玩
旅游,美食,搞笑幽默
美食,星座命理,IT 数码,娱乐
美食,IT 数码,娱乐,体育,汽车
校园生活
时尚,星座命理,教育就业,健康,旅游
财经,天秤座
名人明星

娱乐
旅游,美食,星座命理,山东生活,英语,羽毛球
美甲师,手工爱好者,森,原木家具,钟爱复古,天秤座,偏执狂,电影爱好者,爱生活,爱自由
星星
健康
新闻趣事
神经质,偶喜欢玩,射手女
时尚
娱乐,时尚
粉红控,搞笑幽默
星座命理
体育,汽车,美食
八卦杂谈,新闻趣事,名人明星,搞笑幽默,桂林生活
游戏动漫,双鱼座,音乐,篮球,Dota,漫画
星座命理,旅游,娱乐
美食,娱乐
美女,游戏动漫,娱乐,IT 数码
囧,爱情,浪漫,娱乐,摄影,听歌
美女,娱乐,时尚,健康,旅游,美食,文艺
旅游,娱乐
汽车,美女,旅游,时尚,娱乐
爱情,双鱼座,羽毛球,电影,听歌,紫色,唱歌,90 后
娱乐,电影,唱歌,乐观,动漫,张杰,听歌,宅男,狮子座
军事,IT 数码,文艺,体育,汽车
娱乐
阅读,双子座,手机,自由
投资理财,IT 数码,格斗,李小龙,羽毛球,交互设计,用户体验,UI 设计,移动互联网,经济
穿衣美容,星座命理
娱乐
情感生活
美食
名人明星,听歌,美食
美食
新闻资讯
旅游,健康,双鱼座,80 后
IT 数码
汽车,IT 数码,时尚,体育
体育
健康,旅游,美食,IT 数码,体育
名人明星,娱乐
美食
广东生活
游戏动漫,投资理财,娱乐,音乐
娱乐,旅游,时尚,汽车,美食

星座命理,汽车,新闻资讯,健康,旅游,美食
创业
搞笑幽默,汽车
旅游
星座命理
军事
美食,汽车
视频音乐,游戏动漫
美女
美女
旅游,美食,微博奇葩,新闻趣事,搞笑幽默,新闻资讯,时尚,投资理财
囧
健康,旅游,时尚,文艺,娱乐,平面设计师
游戏动漫
娱乐
韩范儿,大铜板,微笑,乐观
IT 数码,体育,汽车,旅游,美女
游戏动漫,娱乐,汽车
美术,水瓶座,动漫,上网,电影,艺术,搞笑,旅游,吃货,学生
旅游
游戏动漫,宅男
平常心
星座命理,汽车
视频音乐,名人明星,洛阳生活,校园生活,美女
娱乐,旅游,时尚
处女座,听歌
旅游,教育就业,美食,军事,体育
娱乐
投资理财,美女,娱乐,汽车,IT 数码,正太控,蓝色,旅游
浙江生活
星座运势,视频音乐
福建生活
时尚,90 后,双鱼座,唱歌,网购
时尚,奋斗 ing,美食,旅游,音乐控,读书,90 后
教育就业,IT 数码,娱乐,新闻资讯,体育
文艺
健康
娱乐
营销
时尚,睡觉,宅,健康
娱乐
时尚,旅游,星座命理,美食
教育就业,美食,美女,健康,旅游,文艺
EXO

美食
音乐,搞笑,运动,旅游,艺术
浙商,义乌小商品城,财经,财富,商业,人物,宏观,公司,新闻,思想
旅游,星座命理,美食
游戏动漫
时尚,偶稀饭睡觉觉,平常心,宅
文艺
上网,听歌,好书,善良,电影,搞笑,传媒,文学,读书,美食
汽车,IT 数码,旅游,娱乐,体育
美食,旅游,文艺,科研,抗体,生物,流式
美食,星座命理,旅游,美女,娱乐
娱乐
美女,写散文,读书,唱歌,主持节目,朗诵爱好者,做饭是一种享受,摄影爱好者,音乐
游戏动漫,IT 数码
文艺
其他生活
美食,新闻资讯,文艺,娱乐,旅游
SNYD,旅游,星座命理
美食
星座命理
视频音乐
时尚,旅游,文艺,星座命理,娱乐
游戏动漫,娱乐
旅游,投资理财
星座命理,穿衣美容
体育,游戏动漫
娱乐
健康,文艺,美食,旅游
艺术,爱情
美食,IT 数码,文艺,体育,娱乐
简单,深圳生活,爱旅游,时尚,教育就业,健康,旅游,电影,音乐,美食
音乐,看小说,美食,电影,运动,旅游
美食
星座命理,精油爱好者,美容护肤,体育,美剧,水瓶座
处女座,自由
新闻趣事
发呆 ing,美食,天蝎座
多愁善感,低调,90 後,手机,怀旧,偶喜欢玩,看书,睡觉,音乐,旅游
新闻趣事
健康,旅游,美食,时尚
科斯的粉丝,经济法学研究生
美食,汽车,健康,旅游
睡觉
文艺

文艺
健康,旅游,美食
黑莓,军事,旅游
美女,足球,运动
美食,娱乐
动漫,学生,宅,自由,懒人,爱唱歌,睡懒觉,微博,白羊座,阅读
星座命理,文艺,健康,宅,校园,偶稀饭睡觉觉,睡觉
双鱼座,幽默,看书,旅行,财经,自由,音乐,平常心
原创,电视,彩妆,乐天派,神经大条,生活,幸福,淘宝,发呆,购物
游戏动漫,IT 数码
投资理财,新闻资讯,旅游,军事,IT 数码
旅游,健康,投资理财,IT 数码,体育,娱乐
健康,游戏动漫,汽车,投资理财,文艺
美食
搞笑幽默
绝壁声控,最近迷上霹雳了,岛国动漫,网翻,苦逼的理工女,伪文艺,建筑学
投资理财,IT 数码,新闻资讯,健康,旅游
巨蟹座,天秤座
媒体
爱电影,懒,狮子座,吃,恐怖片,神经大条
旅游,名人明星,微博奇葩,江苏生活,新闻趣事
文艺,娱乐,时尚,汽车
我就是我,哈哈,电影爱好者,交朋友,旅游美食爱好者,吃,网络
紫色
时尚,旅游,健康,投资理财,文艺
时尚,宅男,处女座,90 后,电影,唱歌,数码,体育,宅,听歌
娱乐,IT 数码
时尚的见证者
游戏动漫
健康
汽车,IT 数码,游戏动漫
穿衣美容
游戏动漫
娱乐
学生,网络
旅游
萌宠,教育就业
美食,娱乐
视频音乐
旅游
80 后
教育就业,旅游,宅
美食
写作

体育,車路士,森林狼,天蝎座,90 后
健康,新闻资讯,美食,星座命理,时尚
体育
教育就业,新闻资讯,IT 数码,投资理财,体育
娱乐
新闻资讯
旅游,美食,福建生活
爱幻想,睡觉睡到自然醒,懒人,宅女一枚,法国,双重性格,时尚,90 后,看书,天秤座
体育
南京生活,旅游,名人明星,美食,穿衣美容
娱乐,健康,旅游,IT 数码
搞笑幽默,视频音乐,美图摄影
时尚,双子座学生妹子
美食,美女,文艺,游戏动漫,旅游
星座命理,文艺,新闻资讯,美食,娱乐
搞笑幽默,爱自由,吃货一枚,宅女加腐女,爱玩,懒人,90 後,搞怪,睡觉,学生一枚
娱乐,游戏动漫,文艺,体育,旅游
娱乐,美食,旅游,IT 数码
娱乐,体育
新闻资讯,教育就业,健康,文艺,体育
美女
IT 数码
美人,美味,美食
星座命理,游戏动漫
旅游
美女
穿衣美容
汽车,IT 数码,美食,娱乐
IT 数码,娱乐,教育就业
娱乐
服装,时尚,购物狂,时尚潮流,团购,淘宝,摩羯女,单身
游戏动漫,幻影,冒险岛
新闻资讯,文艺,军事,游戏动漫,校园,历史,微博,宅,游戏,动漫
旅游
文艺,星座命理
旅游
娱乐,旅游,健康,美食,汽车,游戏动漫,投资理财
星座命理,娱乐
旅游
IT 数码,投资理财,时尚,旅游,星座命理,自由,宅女
娱乐,星座命理,搞笑,好性格,乐观
文艺
销售,汽车,营销,搞笑,美女,水瓶座
旅游,美食,文艺,体育,娱乐

时尚,汽车,教育就业,投资理财,旅游
时尚,健康,文艺,美食,教育就业,旅游
动漫,游戏,NBA,自由,狮子座,宅男,90 后,体育,手机,听歌
汽车,投资理财,健康,星座命理,军事
IT 数码
搞笑幽默
投资理财
旅游
旅游
美食
娱乐
美女,财经
美食,穿衣美容
娱乐
美食,旅游,健康,汽车,时尚,历史,神话
体育
星座命理
看书
美女,IT 数码,星座命理
旅游,文艺,体育,娱乐
新闻资讯,教育就业,军事,文艺
星座命理,90 后,搞笑,理性,感性
投资理财
网络
广告
旅游
星座命理
自拍,苹果控,淘宝,爱音乐,天蝎座,唱歌,购物狂,连接服务器失败,潮人
美食,旅游
娱乐,健康,旅游,星座命理,美食
军事,文艺,新闻资讯,教育就业,投资理财
偶喜欢玩,金牛座
健康
旅游,美食,文艺,娱乐,美女,游戏动漫
教育就业,新闻资讯
文藝,星座命理,娛樂
美女,汽车,旅游,文艺,娱乐
IT 数码
投资理财
梦醒开始飞
娛樂
体育,娱乐,汽车,时尚,美食,IT 数码
人生若只初相见,旅游,电影
美食

美女
旅游,偶喜欢玩,阅读,天秤座,媒体
娱乐,宅女,读书
娱乐,文艺
美食
宁夏生活,新闻趣事,萌宠,星座命理,搞笑幽默
00 后
新闻趣事
投资理财,乐观,沉默
穿衣美容
IT,囧
健康,美食
大学生
体育
娱乐
浪漫
旅游,健康,时尚,美食,娱乐,戒烟神人,创业先锋,单身花美男,金牌指挥
娱乐
游戏动漫
姜河那,魏千翔,游戏动漫,娱乐,天龙八部 OL,艾斯比帮,百度张韶涵吧,韶先队,张韶涵
娱乐
穿衣美容,向往纯粹生活,大爱保定,舞蹈
浙江生活
健康,美食,旅游,投资理财,时尚
娱乐,天蝎座
文艺,IT 数码,汽车,游戏动漫,体育
钢琴
搞笑幽默,新闻趣事,广东生活,星座命理,旅游
投资理财
汽车
美食,军事,娱乐,体育,自由,旅游
玩儿
IT 数码
名人明星,旅游,美食,穿衣美容,微博奇葩
汽车
美女,娱乐,IT 数码
名人明星
广东生活,美食
乐观,80 后,射手座
旅游,宅,旅行,90 后,手机,睡觉,水瓶座
游戏动漫,教育就业,军事
美食,军事
健康,旅游
美女,汽车,IT 数码,文艺,体育

投资理财,旅游,美食,军事,IT 数码
培训,教育,教学,家教,课外辅导,语文补习,作文辅导,家庭教师,小学生,初中生
教育就业,健康,旅游
足球,欧美乐,实况足球,巨蟹,90 后
娱乐,美食,游戏动漫,军事,汽车
旅游
小说
学生一族,小说迷,孩子气,时尚,爱自己
运动
善良
哈韩族,休闲,旅游,美食,老师,宅女,北京人在狮城
歌手,听歌,诗歌,梦想,化妆,音乐迷,音乐爱好者,爱唱歌,自拍,娛樂
投资理财,旅游,星座命理,美食,文艺
军事,体育
娱乐
时尚,紫色,crissangel,杨千嬅,梁朝伟,尼古拉斯凯奇,魔笛,水瓶座
新闻资讯
游戏动漫
好性格,娱乐
美食,星座命理,微博奇葩,吃,平常心,音乐,好性格,乐观,听歌
美女
文艺
写作看小说,音乐,射手座,旅游,美食,唱歌,设计
亲情,90 後,低调,好性格,音樂,营销,聊天
教育就业,文艺,新闻资讯,新闻,语录,历史,读书,电影,上网,学生
动漫,许嵩迷
旅游,新闻资讯,文艺
文艺,体育,娱乐,浪漫,亲情,睡觉
旅游,星座命理
文艺,健康,美食,时尚,娱乐
微博奇葩
新闻趣事
美容服饰,做梦
微博,爱情,娱乐,新闻资讯
美女,新闻资讯,投资理财
手机,宅男,微博,诗词,社会,书籍
爱旅行
宅
美食,多愁善感的天秤,吃货,宅女,90 后
IT 数码,军事
旅游
美女,游戏动漫,汽车,旅游
搞笑幽默
新闻资讯,手机门户

视频音乐,穿衣美容
健康,旅行,语录,紫色,设计,天蝎座
美女
美食,旅游,文艺,娱乐,健康,星座命理,IT 数码
娯樂
IT 数码
读书,宅,吃,校园,娱乐,巨蟹座,小说,互联网,听歌,学生
萌宠
游戏动漫
穿衣美容,搞笑幽默
星座命理,娱乐,文艺,IT 数码,旅游
体育
东莞生活
旅游,美食,文艺,IT 数码,娱乐
旅游
游戏动漫,体育
偶稀饭睡觉觉,天蝎座
时尚,抱着无所谓态度,会生活的很好,乐观
文艺,旅游,星座命理,投资理财,健康
新闻资讯,娱乐
手机控,中性有点小女人,活泼开朗外向,服装,美食,电影,幽默
文字,环保
美食,翻译,电影,文学
星座命理,时尚,美女,新闻资讯,健康
唱歌,微博
哈利波特,双鱼座,动漫,宅
梦想,学生,喜欢音乐,爱唱歌,o 型血,双子女,低调,90 後,乐观,健康
时尚
美食,旅游
星座命理,时尚,体育,娱乐
健康,文艺,星座命理,教育就业,娱乐
体育
文艺,投资理财,美女,旅游,军事
睡觉
旅游,健康
美食
美食,新闻资讯
IT 数码
andy,junjin,申彗星,金炯完,李玟雨,eric,shinhwa,团饭,神话 fan,橙子
其他生活
艺术,亲情
健康,新闻资讯,旅游,军事,美食,微博控,乐活族
狮子座,喜欢有天啊,讨厌下雨
新闻资讯,星座命理,军事,旅游,文艺

体育
穿衣美容,视频音乐,名人明星
娱乐,体育,忧郁
游戏动漫
学生,偶稀饭睡觉觉
游戏动漫
微博奇葩,新闻趣事,旅游,穿衣美容
江苏生活
美食,健康,吃货,看小说,客服,宅女猪猪一只,80 后
美食
游戏动漫
时尚
IT 数码,汽车
coser,玩儿,K 歌,时尚,娱乐
乐观,交友,学生
搞笑
瘦身,爱吃,校园,双子座,平常心
时尚,星座命理,美食,健康,旅游
体育
羽毛球,Shazam,Kafka,Storm
时尚
文字
穿衣美容
文艺,汽车,健康,军事,时尚,教育就业,投资理财
时尚,天秤座,蓝色
穿衣美容,听歌,爱电视剧,小女人,爱唠嗑,各种宅,90 后,购物,睡觉
美女
健康
你若不离不弃,我必生死相依,小白羊,固执,好玩,爱笑
健康,旅游,美食,90 後,幻想,学生一枚,拍照,music,网购,睡觉
水瓶座,90 后非脑残,强迫症,新闻资讯
教育就业,汽车,投资理财,娱乐,体育
旅游
旅游
时尚
旅游,美女,信阳生活,IT 数码,视频音乐
做梦,旅游,娱乐,听歌,篮球
新闻资讯,健康,星座命理,美食,娱乐
汽车,娱乐
穿衣美容,美食,旅游,广东生活
娱乐,健康,时尚,狮子座,音乐,动漫,唱歌,玩儿
喜欢听歌
英语
动漫迷,游客

IT 数码,军事,投资理财,教育就业,新闻资讯
体育,新闻资讯,旅游
A 股
娱乐
美女,文艺,娱乐,旅游,时尚
汽车,娱乐,体育
游戏动漫
时尚
天蝎座,吃货,电影,小说,音乐,旅游
体育资讯,财经资讯,健康,文艺,体育
穿衣美容
美女,视频音乐,搞笑幽默,穿衣美容
旅游
视频音乐
动漫,广州生活
旅游,时尚,星座命理
新闻资讯,IT 数码,文艺,星座命理,体育,唱歌听歌,悬疑片控,篮球爱好者,足球迷,台球发骚友
2013 快男,于湉,华晨宇,爱笑,善良,幽默,唱歌,篮球,白羊座
时尚,自由职业
星座命理,囧,射手座,天蝎座,校园,吃货,上网,浪漫,唱歌
宅
星座命理,美女
娱乐
军事,体育
星座运势
文艺,旅游,新闻资讯,军事,体育
微笑
旅游,汽车,体育
体育
天蝎座
旅游
健康
文字,A 股
时尚,投资理财,美食,旅游,文艺
娱乐
教育就业,旅游,营销,摄影爱好者,狮子座,自由
健康
文艺
娱乐
教育就业,时尚,娱乐
教育就业
新闻趣事
娱乐,健康,旅游,星座命理,投资理财

美女,文艺,美食,健康,旅游
新闻资讯,健康,汽车,军事,IT 数码
旅游,游戏动漫
教育就业,投资理财,体育
文艺,健康,星座命理,养生,美容,周易,服饰,美食
自由,学生,奋斗,爱幻想,各种冷知识,爱电影,推理控
汽车
山东生活
动画,hiphop,影视,爱吃,服装,视频音乐
游戏动漫
美食,星座命理,健康,文艺,娱乐
星座命理,旅游,健康,美食,娱乐
汽车,时尚,旅游,新闻资讯,美食
文艺,美食,星座命理
乐观
健康,美食,娱乐,文艺,教育就业
娱乐
新闻资讯,军事,IT 数码,体育
新闻资讯,时尚,教育就业,娱乐,IT 数码
教育就业,80 后
旅游
健康
旅游,好书,睡觉,偶喜欢玩,乐观
新闻趣事
教育就业
旅游,手机,搞笑,80 后,动漫,天秤座,新闻,幽默,吃货,读书
星座命理
美图摄影,体育资讯,校园生活,新闻趣事
星座命理,游戏动漫
足球,历史
睡觉,80 后,时尚,吃货
新闻资讯,IT 数码,时尚,投资理财,美食,娱乐
娱乐,依赖也是爱
美食
人生何处不进击,欢脱,傻缺,美食,双鱼座
旅游,文艺,音乐,旅行,美食,做梦,书法,摄影
SQL
旅游
美女
旅游,教育就业,新闻资讯,游戏动漫,IT 数码
美食,宅女,处女座,自由,吃货
美女,时尚,娱乐,星座命理
投资理财,足球,股票,宅男
美食,投资理财,娱乐

IT 数码,星座命理,游戏动漫,娱乐
怀旧
旅游,无聊,K 歌,射手座,个性,学生
游戏动漫,动漫,吃
IT 数码,水瓶座,手机,吃,听歌,平常心,美食,电影,喜欢吃零食…
体育,校园
减肥 ing,腹黑,宅女一枚,巨蟹女,学生一族
射手座,90 后,微博,美食
休闲,美食,娱乐,健康,时尚,旅游
旅游,星座命理,美食,军事,IT 数码
美女
IT 数码
文艺
摄影师,台球,90 後,幽默
名人明星,美食,穿衣美容,旅游,辽宁生活
名人明星,湖北生活,微博奇葩,新闻趣事,美女
文艺,健康,旅游,星座命理,美食
健康,旅游,美食,游戏动漫,娱乐
交友,上网,美女,汽车,娱乐,旅行,旅游,90 后
游戏动漫
时尚
娱乐,体育
投资理财,教育就业,娱乐,体育
IT 数码,美食
健康,美食
听歌,设计,上网,90 后,唱歌,艺术,交友,娱乐,时尚,音乐
山下智久,梁静茹,少女时代
白羊座,80 后,羽毛球,幽默,写作,自由,手机,平常心,听歌
娱乐
吃喝玩乐,乒乓球,简单,养生,爬山,音乐控,台球,时尚,汽车,IT 数码
萌宠
健康,美女,时尚,IT 数码
旅游,双鱼座,音乐
美食,旅游,07 年的那个夏天
百色生活
娱乐,旅游,时尚,星座命理,美食
名人明星
善良
90,非单身,射手座
名人明星
艺术,善良,浪漫,学生,乐观,唱歌,上网,爱情,天蝎座,宅女
时尚
交友,宅男
游戏动漫,时尚,汽车,体育,IT 数码

IT 数码,投资理财,旅游,星座命理,健康
艺术
美食,视频音乐,美女,旅游,搞笑幽默
娱乐
体育
穿衣美容
李准基的粉丝,张根硕的鳗鱼,boyfriend 珉宇,光旻,荣旻,白羊座,动漫,听歌,宅女,上网
吃货
美女
时尚
新闻资讯,旅游,军事,文艺
文艺,八卦,纯真,文字
乐观,营销,幽默
健康,星座命理,美食,娱乐,文艺
投资理财,游戏动漫,IT 数码,90 后,宅,阅读,双鱼座
MUJI,原宿,欧美控,漫画
电子商务,设计,宅女,天蝎座,听歌,幽默,摄影
投资理财,IT 数码,文艺,体育
游戏动漫
时尚,教育就业,游戏动漫,健康,星座命理,娱乐,大学生,90 後,摩羯座
美食,健康,时尚,教育就业
生活,善良
文艺
健康,美女,时尚,教育就业,旅游
时尚新鲜人,旅游,美图摄影,视频音乐,Freedom
。我
军事
新闻资讯
娱乐,体育,生锈的铁杆球迷
IT 数码
美食,新闻资讯,黑眼圈,吃好睡好玩好,睡觉睡到自然醒,吃,90 后
八卦
健康
美食,美女,旅游,星座命理
星座命理,视频音乐
旅行,梦想,音乐控,仙剑,海贼王,阳光,青春,热爱生活,武术
篮球,动漫,时尚,潮男,起床困难户,80 后,正太,K 歌
时尚,娱乐
游戏动漫,隐身党
新闻资讯,旅游,体育,汽车,军事
健康,美女,IT 数码,文艺,体育,娱乐,游戏动漫,时尚,军事
娱乐
教育就业
汽车

摄影爱好者,美食控
投资理财,没心没肺,小白,小欠儿,贪吃鬼
搞笑幽默,心在,志在
娱乐
娛樂
IT,设计
爱情,生活
IT 数码
搞笑
舞蹈,懒人,紫色控,宅女一枚,摩羯
教育就业,健康,文艺,新闻资讯,旅游
军事,篮球,摩羯座,偶稀饭睡觉觉,90 后
教育就业
文艺
军事,电影,成长 ing,学习 ing,缺爱 ing,奋斗 ing,珞丹控,宅男,睡觉
旅游,文艺
星座命理,军事,文艺
旅遊
时尚
健身,篮球,伪基佬,肌肉控,屌丝男,绍兴佬,肉食者,皮具控,失语症,欲行者
旅游
猫控,向日葵,爱音乐,偏执狂,偶喜欢玩,干物女,娱乐,神经大条,自由,古怪
投资理财
军事,体育,星座命理,时尚,娱乐
情感生活,羽毛球,校园,处女座,电影爱好者
名人明星,旅游,穿衣美容,视频音乐,星座命理
旅游,娱乐,体育
文艺,旅游
八卦,射手座,唱歌,美食,大学生,宅,80 后,听歌
文艺,CAFA 实验艺术
助听器,西门子
名人明星
天蝎座
乐观,随性
搞笑幽默
旅游
美食
娱乐
美女,军事,IT 数码,体育
双鱼座,90 后,吃,体育,睡觉,小说
美女,书法,绘画,莎拉波娃,诗词,喜欢音乐,美术,哲学
视频音乐,美图摄影,情感生活,校园生活,喜欢逛逛世界!
游戏动漫,新闻资讯,旅游,IT 数码,娱乐
文艺

旅游,时尚
旅游
健康,新闻资讯,文艺,娱乐,美食
教育就业
美女
北京生活,美食,萌宠
囧,文字,新闻,时尚
教育就业,娱乐,新闻资讯,美女,军事
红酒,健康,投资理财,汽车,旅游
健康,时尚,娱乐,旅游,美食
北京生活
时尚,音乐,旅游,电影,平常心,双子座,美食,90后,足球,微博
篮球,爱音乐,陈奕迅,睡懒觉,汽车,IT数码,体育
IT数码,娱乐
教育就业,文艺
旅行
体育
摄影
星座命理,旅游,偶稀饭睡觉觉,天秤座
旅游,旅行,摄影
poping,美女
疯子,自恋
新闻趣事,微博奇葩,星座命理,搞笑幽默
健康
IT数码,体育
金黄色,黑白,音乐,善良,信仰,爬山,文字,囧,搞笑
80后
心情
美食,文艺,健康,时尚
巨蟹座O型血,EXO
教育就业,健康,投资理财
吃货,街拍,同志
星座命理
电影,90后
游戏动漫,旅游,娱乐,美食,文艺
娱乐
美食,时尚,健康,娱乐
军事
白羊座,平常心,营销,微博,小说,电影
汽车,狮子座男生
自由,广告
道学奥天术,EVE,自由,偶稀饭睡觉觉,好书,小说,吃货,上网,交友,平常心
80后,美剧,篮球,偶喜欢玩,体育
版权,版权维权,知识产权保护,数字出版,版权保护,电影反盗版,软件反盗版,大卫版权网

狮子座,电影
拍照,汽车,文字控,想去旅行,臭美,双鱼座,浪漫,偶稀饭睡觉觉,宅女,音乐
星座命理,新闻资讯,时尚,汽车,IT 数码
星座命理
狮子座,90 后
汽车,随着心情做事,做自己喜欢的
娱乐
时尚,健康,文艺,美食,娱乐
视频音乐,星座命理
娱乐
旅游
旅游
看电影,阳光,爱生活,旅游
娱乐
广东生活
足球,数码
90 后,默颜
体育,A 股
自由
平常心
穿衣美容
情感生活,八卦杂谈,新闻趣事
美食
旅游
射手座,美女
时尚,美食,手机,睡觉,听音乐,发呆
文艺
娱乐
美图摄影
彭郁雅,善良,宅女,90 后,唱歌,吃货
时尚
文学,乐观
文艺,游戏动漫
Entertainment
宅女,双重性格,自由,shopping,执着,小女人,学生一枚,我就是我
体育
游戏动漫,体育,汽车
好性格
吃,历史
军事,娱乐,健康
游戏动漫,体育
摩羯座,90 后
时尚,新闻资讯,旅游,IT 数码,娱乐
星座运势,娱乐

时尚,娱乐
彩妆,养生,商业,美容护肤,穿衣美容,玉器人培训,中国玉文化,和田玉收藏投资,玉器鉴别鉴定,浪漫旅行
英语
文艺
美女,游戏,NBA,设计,大学,90 后
美食
娱乐
财经,小说
星座命理,旅游
娱乐,听歌,宅女,自由,亲情,巨蟹座,80 后
IT 数码
张艺兴,繁星,EXO,SMTOWN,剩下你猜
心情,交朋友,独立,旅游,美食,美食爱好者
双子座
娱乐
健康
文艺,娱乐,巨蟹座
058214,IT
喜欢旅游,双重性格,小清新,插画,南京,美食
偶喜欢玩
文艺,教育就业,旅游,新闻资讯,军事
上海生活
搞笑幽默
双鱼座
体育
星座运势
美食,旅游,文艺,健康,星座命理,热爱生活,怀旧,80 後,坚强,笑
萌宠
浪漫,乐观
投资理财
搞笑幽默
潮人,文章,胡歌,美食,时尚,文艺,JAZZ
投资理财,教育就业,IT 数码,健康,时尚
搞笑幽默
美食,健康
星座命理,摩羯座,自行车,吃货一枚,陈奕迅
健康,时尚,旅游,新闻资讯,教育就业
搞笑幽默,喜欢看恐怖片
娱乐
浪漫,爱情
娱乐
基督徒,书法绘画爱好者,化妆造型,英语
旅游

娱乐
美女
英语
文字,娱乐
健康,北海里面洗过澡,沙漠里面睡过觉,长大艺术学院,宜昌,电影,80 后,艺术,摄影,旅游
汽车,星座命理,游戏动漫,娱乐,军事
美女,汽车,财经,摄影,数码,历史,运动,学生,新闻,体育
美食
健康,美食,娱乐,教育就业,文艺,美食,旅游
娱乐
星座命理
连接服务器失败,电影迷,IT 数码,游戏动漫,汽车,娱乐
美女,美食,篮球,偶喜欢玩,90 后,双子座,游戏,体育,搞笑,运动
吃
健康,旅游,美食,娱乐
娱乐
文艺
星座命理
IT 数码,娱乐,汽车,美女
健康,娱乐,时尚,美食
文艺
教育就业
音乐,大学
牛皮纸袋,首饰盒,超大型彩盒,礼品袋,礼品盒加工,营销
旅游
设计
搞笑
美女
偶稀饭睡觉觉,旅游
军事
军事
游戏动漫,美食,美女,IT 数码,文艺
篮球,听歌,电影,NBA
娱乐,历史,偶喜欢玩,旅游,音乐
英语
微博奇葩
小说,互联网
时尚,美女,星座命理,旅行,吃货
狮子座,媒体,90 后,音乐,设计,NBA,上网,听歌,唱歌,幽默
美食
旅游
健康,人力资源,败家,起床困难户,乐观,80 后,浪漫,听歌,金牛座,宅女
体育
时尚

文艺,游戏动漫,美女,娱乐,星座命理
生活
娱乐
IT 数码
电影爱好者,喜欢听歌,美剧迷
偶喜欢玩,手机
旅游
美食,营养,健康
新闻趣事,穿衣美容,星座命理
文艺,娱乐,旅游,星座命理,IT 数码
星座命理
IT 数码
美女
吃,爱情
娱乐,新闻资讯,时尚,投资理财,美食
旅游,美女,新闻趣事
美食控,android,bigbang,布丁,球迷,个性,神经大条
娱乐,汽车
学别人最好的,做最好的自己
文艺,时尚,汽车,旅游,IT 数码
健康
美女
看书,广告
文艺,搞笑
星座命理
新闻资讯,娱乐,IT 数码,美食,时尚
美女
营销,音乐
文艺
星座命理,旅游,IT 数码,上网,吃货,美容,乐观,学生,生活,电子商务
自由,阅读,交友,生活,善良,美食,处女座,电影,游戏,上网
健康
娱乐
我喜欢 Michael
星座命理
星座命理,旅游
金牛座
读书分享
投资理财
视频音乐
美女,游戏动漫,娱乐
美食,军事,体育
旅游,美食,娱乐
时尚,旅游,IT 数码,汽车,星座命理,美食

射手男
摩羯座
时尚
娱乐,体育,投资理财,汽车,时尚
娱乐
游戏动漫
时尚
天秤座
减肥,吃货一枚,魔兽世界,发呆,淘宝,冷笑话,矛盾体,梁静茹,心情,花痴
囧,射手座
新闻资讯,体育,娱乐,美食,踢球,啤酒,聊天
美食
魏晨的粉丝哦
名人明星
娱乐,体育
时尚,汽车,IT 数码
上网
大学生,宅男
游戏动漫,文艺,投资理财,星座命理
汽车
美女,IT 数码,旅游
旅游,视频音乐,美食
旅游,投资理财,时尚,美女,美食
唱歌
旅游,美食
微博奇葩,搞笑幽默
IT 数码,视频,运动,健身爱好者,搞笑,动漫,游戏,电影
游戏
名人明星
美食,美女,娱乐,旅游,汽车
美食,娱乐,健康,时尚
汽车,美食,IT 数码,听歌,紫色,水瓶座,吃,数码,旅游,电影
新闻趣事
射手座,o 型血,85 后
有点宅,大爱王力宏,星座控,小清新,文字控,90 后
穿衣美容
游戏动漫
亲情,平常心
投资理财,教育就业,新闻资讯,健康,美食
紫色,宅女
文艺,我还是会想你,星座命理,美食,时尚,娱乐
天蝎座,善良,打篮球,旅游爱好者,幸福,学生
IT 数码,娱乐,汽车,时尚,美女
新闻趣事

设计,运动,宅,双鱼座
巨蟹座
美食,篮球,英语,阳光,户外,shinee,高尔夫,疯子,水瓶女
运动,平常心
嘿嘿,天秤座,90后,手机,吃货,学生,好书,八卦,宅女,上网
天秤,真实力,摄影,幽默,开朗,大学生,天枰座O型女
搞笑,摄影,学生,时尚
游戏动漫
大学
娱乐,宅,白羊座
旅游,军事,文艺,体育,娱乐
娱乐
娱乐
萌宠,星座命理,穿衣美容,微博奇葩,视频音乐
宠物图集,读书分享,美食,新闻趣事,搞笑幽默
体育,IT数码
军事
营销
娱乐
IT数码,文艺,奔三的80后
星座命理,投资理财,旅游,健康
娱乐,教育就业
美食
美食控,芳香疗法,时尚,健康
美食
微博
娱乐
投资理财,健康,IT数码
星座命理,旅游,美食
鸡尾酒,创意设计,游戏动漫
萌宠
旅游
娱乐,韩剧
娱乐,旅游,星座命理,美食,动漫
生活,羽毛球
90后,音乐,艺术,设计,时尚,广告,自由,电影,大学,交友
美女
摩羯座,音乐
媒体,gay,五月天,爱电影,动画,法语,粤语,傻,单纯
时尚
医疗,心理,保健,美容,养生,健康减肥,时装,街拍,化妆
时尚,美食,IT数码,娱乐,巨蟹座
穿衣美容
娱乐,军事,汽车,美女,游戏动漫,IT数码

IT 数码,健康,美食,新闻资讯,娱乐,体育
睡觉,生活
媒体,金牛座
娱乐
娱乐
投资理财
动漫,创业,养生,天秤座,政治,旅游美食,干物女
美食
著作权,版号,ICP,SP,游戏开发,双鱼座,文网文,游戏策划,吃货,时尚
视频音乐,搞笑幽默,穿衣美容,投资理财,时尚,旅游,健康,美食
乐观,上进青年,爱生活
视频音乐,旅游,美食,萌宠,微博奇葩,偶稀饭睡觉觉
美食,娱乐
90 后
行街睇戲食飯,唱 K,上网,80 后
投资理财,星座命理,美女,娱乐,体育
名人明星
旅行
健康
文艺,紫色,时尚
巨蟹座,爱情
美食
星座命理
健康,星座命理
校园,八卦,天秤座,学生,美食,宅,吃货,上网,偶喜欢玩,90 后
文艺
大学生,吃货
美女,汽车,IT 数码,星座命理,体育,娱乐
星座命理,美女,娱乐
文艺,金牛座
好药,好医生,亚健康,医院,减肥
娱乐,汽车,健康
投资理财,健康,旅游,星座命理,宅
休闲卖场业顾问,休闲宾馆业顾问,休闲养生业顾问,休闲健身业顾问,旅游业顾问,餐饮业联合顾问
教育就业,时尚
数码
旅行
文学,宅男,好书
星座命理,美食
娱乐
视频音乐,新闻趣事,微博奇葩,星座命理,搞笑幽默
美女,军事
强迫症女王,执着,任性冲动,手机控,喜欢看书,喜欢发呆,喜欢听歌

投资理财,娱乐,时尚,汽车
体育
星座命理
美食
娱乐
军事,娱乐,星座命理
新能源,太阳能,照明,LED,电子元器件,网购,汽车,微博,生活,IT
娱乐
游戏动漫,偶喜欢玩,双子座O型血,宅女,学生,校园
旅游
时尚,旅游,美食,美女,娱乐
美女
军事,美女,游戏动漫
音乐控,双鱼座,电影,摇滚
囧,电影
星座命理,军事,汽车,时尚,美女,健康,股票,阅读,微博,好性格
教育就业
巨蟹座,新闻
娱乐,星座命理
汽车,射手座,电影,音乐,设计,90后,生活,财经,唱歌,网络
文字,乐观
娱乐
新闻资讯,健康,美食,娱乐,投资理财
旅游,美食,设计,设计来源于生活,艺术
思考,猫咪控,music
IT数码
创业,时尚,旅行,电影
文艺
旅游,搞笑,校园,乐观
新闻资讯,旅游,娱乐,IT数码,时尚
投资理财
好性格,汽车,宅,F1,旅游,美食,体育
投资理财,美女,军事,新闻资讯
文艺,投资理财,游戏动漫,美食,健康
IT数码,旅游,美食,星座命理,健康
旅游,美女,时尚,文艺,娱乐
美女
星座命理,娱乐,美女
旅游,军事,IT数码,体育,娱乐
汽车,游戏动漫,星座命理,IT数码,体育,娱乐
体育
时装,街拍,时尚,健康,减肥
偏激,强迫症,自虐,洁癖,天蝎,夜猫,嗜睡,变态,二逼文艺青年,懒
求男友,求兼职,很屌丝

会计,星座控,时尚潮流,投资理财
网购,吃
娱乐
北京生活
游戏动漫
IT 数码
时尚,星座命理,旅游,美食,文艺
大嘴巴,星座,旅行,经典,糗事,囧事,冷笑话,搞笑,幽默
偶稀饭睡觉觉,唱歌
教育就业,时尚,旅游,娱乐
旅游
新闻趣事,电影迷,浪漫,感动
星座命理
健康,新闻资讯,美食,教育就业,投资理财
IT 数码,我叫唐景天,糗友,90 后非脑残,电影
娱乐,文艺
犬夜叉,新兰党,夏目友人帐,美食,听音乐,文字控,日韩音乐控,钢琴
美食
美女,旅游,IT 数码,时尚,娱乐
新闻趣事
美女
购物
好性格,偶稀饭睡觉觉,宅
东莞生活,简单而开朗的我,交友,羽毛球,平常心,白羊座,音乐
美容,养生,时尚,街拍,化妆
穿衣美容
数码,阅读,上网,生物质气化
校园,巨蟹座
游戏动漫
游戏动漫,体育,摄影爱好者,电影,吃货
爱音乐,射手座
娱乐
羽毛球,健康,旅游摄影,美剧迷,80 後,宅女,爱幻想,奋斗,网购,处女座
网购
逛街,淘宝控,模特,化妆,情感,多愁善感,欧美控,幻想,爱玩,时尚潮流
美食
唱 K,乐天派,随性,韩剧,户外,品牌,小说迷,美女,乐观,音乐
时尚,健康,投资理财,星座命理,IT 数码
穿衣美容,宅,爱美男,小说,学生,青春
视频音乐
动漫
旅游,懒,高考,休闲,想去青岛。
美女,宅男
娱乐

旅游
新闻资讯,投资理财,教育就业
旅游
羽毛球
车世界,湛江生活,旅游,新闻趣事,美图摄影
汽车
幽默,狮子座,偶稀饭睡觉觉,羽毛球,90 后
旅游
虽小道,必有可观者焉
手机,80 后,幽默,爱情,创业,新闻,运动
美女
天蝎女,大学生,旅游美食爱好者
旅游,军事,娱乐,文艺,体育
健康,体育,新闻资讯,教育就业,IT 数码
旅游,美食,娱乐
偶稀饭睡觉觉,天秤座,亲情,80 后
旅游,美食
财经
狮子座
娱樂
新闻资讯,游戏动漫
理想家,偶喜欢玩,文艺女青年,慵懒,写作是我的生活,哲学家,旅行,摄影,紫色,浪漫
热爱生活,乐观
教育就业,健康,美食,文艺,娱乐
手写文字控,创业,营销,经济,旅遊,看书,青春
动漫,苦逼学生党,烦躁又癫狂,讨厌世界,吃,睡觉,上网
军事
视频音乐
减肥,亚健康,好医院,医生,好医生
宅男,爱情
媒体
搞笑幽默
时尚,浪漫,电影,汽车,乐观,旅行,体育,好书,动漫
搞笑幽默
美女,汽车,军事,旅游,娱乐
美食
旅游
旅游,穿衣美容,美食,萌宠,视频音乐
旅游,大学生,听歌
娱樂
亲情
健康,睡觉,旅行,自由,紫色,处女座,安静但又狂热
唐山生活,旅游,美食,吃货,臭美小辣椒,彩妆,护肤美容,电视剧,电影,消费
旅游,游戏动漫

养生,时尚,减肥,美容,健康
投资理财
美食
叹
胡思乱想,妖精,天蝎座,爱情,睡觉,电影
沉稳兼具孩子气,善变,潜力股,处女座
星座命理
健康,旅游,星座命理,美食,娱乐
苏打绿,金浩森,文子
新闻资讯,投资理财,娱乐
教育就业,时尚,旅游,星座命理,美食,文艺,娱乐
时尚,广告人
美食
新闻趣事
美女,时尚,体育
公务员培训,事业单位培训,公职类考试
亲情,交友,摄影爱好者,狮子座,小说,语录,八卦,创业,大学,宅
文学,艺术
娱乐,生活,广告,巨蟹座,交友,自由,电影,音乐
IT 数码,文艺
旅游,新闻资讯,游戏动漫,美食
娱乐
宅,学生,读书,摩羯座,胡思乱想
美食,新闻资讯
文艺
IT 数码
体育
美食,星座命理,旅游,新闻资讯,军事,投资理财,时尚
文艺
新闻资讯,教育就业
偶稀饭睡觉觉
IT 数码
美食
美食
体育,娱乐,汽车,文艺,IT 数码
健康,汽车,美食,IT 数码,体育
投资理财
羽毛球,大学生,90 後,射手座,雕塑艺术,旅游摄影,运动健身,校园
视频音乐
娱乐,体育,汽车,手机,NBA,音乐,电影,篮球,上网,听歌
娱乐,时尚,美女,健康,美食,旅游,游戏动漫,文艺
互联网,美剧
花样美男,宠物图集,美食,美容服饰,搞笑幽默
时尚,健康,旅游,娱乐,美食,80 后

旅游,教育就业,文艺,健康,美食
时尚,街拍,美容,减肥,亚健康
旅游
旅游,上网,80后,英雄联盟,怀旧,偶稀饭睡觉觉
爱菲如命,犯二
旅游
美食
娱乐,紫色,听歌,吃货,金牛座
名人明星,旅游,娱乐
摩羯,新闻,猫咪,美图摄影,宅女一枚,音乐爱好者
游戏动漫
新闻资讯
旅游,投资理财,健康,美食,星座命理
看电视,shopping,美食,玩儿,90后,宅,双子座
搞笑
时尚,星座命理,健康,旅游,美食
投资理财,旅游
凯文加内特
青海生活
体育资讯
娱乐
睡觉,爱情,运动,手机,交友,音乐,浪漫,巨蟹座
教育就业,投资理财,军事,美食,小说,动漫,旅游,电影
旅游
美食,新闻趣事,搞笑幽默,微博奇葩
运动,自由,美女,80后,旅行,音乐
睡觉
美女
美食,时尚
旅游,美食,健康,星座命理,娱乐
IT数码,文艺,体育,健康,新闻资讯,娱乐
平常心
视频音乐
视频音乐
教育就业,投资理财,军事
设计
时尚,教育就业,旅游,美食,文艺
新闻资讯,时尚,文艺,白羊座
星座命理,猫控,宅女,爱生活
网购,囧
游戏动漫,娱乐,文艺,IT数码,体育
美食
美女,投资理财
美食

IT 数码,游戏动漫,娱乐,汽车,美女
娱乐
旅游
汽车
文艺
投资理财,娱乐,美女,旅游,IT 数码
汽车,旅游,军事,IT 数码,体育,A 股,篮球,美女,生活,电影
设计
穿衣美容
教育就业,新闻资讯,旅游,文艺,希望心灵透彻,希望水源纯净,希望天空蔚蓝
射手座,八卦
看书
IT 数码,体育
Supernatural,电影,尼古拉斯凯奇,休杰克曼,张智霖,JensenAckles,HughJackman
娱乐
摄影
天蝎座
文艺,其实我很宅
文学,自由
网购
体育
天蝎座,90 后,时尚,音乐,旅行,乐观
娱乐,文艺
射手座,处女座
火影迷,制服控警察控,动漫
健康
美女
体育
汽车
视频音乐
写作
美女,汽车,体育
旅游,名人明星,搞笑幽默,校园生活,视频音乐,fx,2PM,交友,90 后,维尼夫妇
游戏动漫,美食,汽车,旅游,时尚
投资理财,双子座,大学生,90 后
音乐
时尚,文艺,旅游,星座命理,美食
IT 数码
娱乐,写作
美食,搞笑幽默,随性,搞笑,笑得等待
善良,双子座,学生,动漫
IT 数码,健康,新闻资讯,教育就业,旅游,军事
搞笑幽默,萌宠
白羊座

健康,美食,文艺
健康,时尚,汽车,投资理财,娱乐
处女座
娱乐
健康,白羊座,娱乐,平常心,大学生,自由,听歌,学生
星座命理,旅游,美食,娱乐
创业,携手求发展,创作求共赢
旅游,北京生活
星座命理,音乐迷
单身,爱睡觉,娱乐
偶喜欢玩
处女座
视频音乐,穿衣美容,美食,旅游,名人明星
自由
娱樂,无厘头
旅游
NBA,双鱼座
简单,娱乐,文艺,美食,羽毛球,亲情,自由,水瓶座,学生,善良
视频音乐,猎头,互联网
游戏动漫,二维动画原画师,创意
我就是我,学生,喜欢音乐,爱好钢琴,爱上网,微笑很重要,爱自己
IT 数码
旅游,汽车,教育就业,美女,军事
新闻资讯,娱乐
星座命理,美女,军事
旅游
IT 数码
穿衣美容
韩剧,BEAST,bap,fx,EXO,superjunior,旅游,美食,文艺,体育
旅游爱好者
美食
音乐,旅行,紫色,欧美音乐,暮光之城
旅游,IT 数码,文艺,体育,娱乐
喜欢音乐,拍照
微博奇葩
双子座
娱乐
文艺,体育,娱乐,游戏动漫,汽车,教育就业
天蝎座,吃货
育儿,亲子,旅游,美食
亲情
娱乐,IT 数码,汽车,美女
网络
穿衣美容,微博奇葩,星座命理,视频音乐,搞笑幽默,重庆生活

摄影爱好者
防水原材料,摄影,电子商务,防水施工,家装防水,防水卷材,防水涂料,防水材料,防水堵漏,防水
新闻资讯,游戏动漫,星座命理
双鱼座
名人明星
电影,浪漫,宅,2B 女青年,双子座,神经质
视频音乐
娱乐,旅游
萌宠
军事,IT 数码,文艺,娱乐,体育,NBA,90 后,学生
吃货,金牛座
星座命理,微博,偶稀饭睡觉觉,吃,乐观,天秤座,音乐,90 后,睡觉,听歌
汽车,旅游,户外,美食
娱乐,体育,汽车
迈迷,愤青,双子男,学生
CF,吃,唱歌,数码,平常心,爱的蜜方
娱乐,文艺,教育就业
篮球,小说
美食
设计,八卦
美食
娱乐
上网
新闻趣事,萌宠,美食,搞笑幽默
八卦,90 后,囧,听歌,文字,手机,学生,水瓶座,偶稀饭睡觉觉,动漫
军事
中医,养生,钢材
浪漫,艺术
娱乐,美食,旅游,健康,教育就业
娱乐,开朗
体育
美食,文艺,旅游,健康,投资理财
美女,双子座
旅游,星座命理,文艺,娱乐
军事
旅游,美食,娱乐,文艺,吃货
旅游
蜗居,动漫,看古装剧,认植物
运动,旅游
旅游,视频音乐,搞笑幽默,美女
财经,网购
新闻资讯,旅游,美食,文艺,娱乐
文艺

美食
IT 数码
八卦,设计
美女,娱乐
投资理财
健康,文艺
文艺,游戏动漫,声控,秦迷,COS 迷,外貌协会,读书,吃货,睡觉,文字
投资理财
美女,投资理财,体育
睡觉
紫色,篮球
音乐
NBA
旅游
汽车,听歌,游戏,吃货,双子座,艺术,读书,美食,宅男,电影
英语,互联网
时尚,双子座
羽毛球
睡觉,好性格,听歌,天秤座,交友
美图摄影,校园生活,美食,视频音乐,安顺生活
IT 数码
游戏动漫,娱乐,军事,文艺,投资理财,美食,健康,星座命理,IT 数码
娱乐
梦想,阳光
新闻趣事,广西生活
美食,时尚,汽车,文艺,IT 数码
内向,双鱼座,温柔,听音乐,宅女
美图摄影,星座运势,美食
校园,广告
美女
游戏动漫,新闻资讯,教育就业,星座命理,体育,健康,美食,娱乐
浪漫
巨蟹座,设计
游戏
健康,文艺,运动,旅游,喜欢音乐,看电影,听音乐,爱音乐,谢娜
健康
传媒
校园生活,旅游,八卦杂谈,搞笑幽默,武汉生活,体育,文艺,军事,娱乐
美剧,摄影爱好者
看书
娱乐,文艺,体育,时尚,游戏动漫,旅游,美女
学生,吃货,文学,文字,美女,军队,战争,IT
四川生活,旅游,美食,星座命理,水瓶座,偶稀饭睡觉觉,吃货
星座命理

听歌, 宅女, 吃货
双鱼座
好性格
旅行, 吃
游戏动漫
时尚
旅游
游戏动漫, 健康, 旅游, 投资理财, 文艺, 健身, 自行车
美女
电子商务, 好书
娱乐, 美女
娱乐
重口味
运动
文字, 浪漫
游戏, 金牛座
体育
善良, 文字
美食, 健康
搞笑幽默, 娱乐
搞笑
语录, 校园
视频音乐, 美食, 名人明星, 穿衣美容, 学生, 鳗鱼, 微博, 90 后, 睡觉
新闻资讯
广州生活
时尚
星座命理
忧愁, 青年, 诗词, 文艺
好性格
新闻
星座命理, 文艺, 汽车, 新闻资讯, 旅游
教育就业, 文艺
读书
电子商务
双鱼座
快乐, 漫画, 活泼, 计算机, 跑步
时尚, 美食, 旅游, 娱乐
自由职业, 80 后
美女, 投资理财, 娱乐
动作, 表情, 思维, 语言, 行为, 五感, 人类, 探索, 心理学
成都生活
睡觉
游戏动漫
汽车, 军事, 游戏动漫

娱乐,音乐
娱乐,美女,时尚,汽车,IT 数码
音乐
美食,跳舞,低调,diy,射手女,花痴,恐怖片,谢娜
新闻资讯,游戏动漫,美女,旅游,IT 数码
王力宏,飞轮海,90 后非脑残,股市,生活大爆炸
娱乐,旅游
时尚,星座命理,文艺,健康
星座命理,体育
文字,摄影
阳光宅男
数码,英语
文艺,娱乐
美食
双鱼
亲情,语录
偶喜欢玩,宅女
健康,水瓶座,搞笑,乐观,八卦,玩儿,美女,偶喜欢玩,娱乐,睡觉
历史
大学,时尚,吃货,自由,平常心,写作,微博,玩儿,旅游,巨蟹座
教育就业,健康,美食,文艺,娱乐
教育就业
汽车,教育就业,旅游,文艺,娱乐
娱乐
文学,好书
倔强,吃,好性格
美食,爱情,吃,幽默,亲情
购物狂,酒吧,电玩城,电影,散打,搏击,健身,时尚,格斗,健身爱好者
娱乐
美女,摄影师,笔亲,简单,建筑,爱玩,爱美食,绘画,户外,设计师
体育,美食,旅游
美食,旅游
双子座
旅游,文艺,教育就业,娱乐
美图摄影
美女
小说,旅行,文字,英语,读书,自由,美剧
微博奇葩,电影
娱乐
美食,文艺
好性格,上网
时尚,美女,投资理财
时尚,星座命理,美食,文艺,娱乐
上网,学生,旅游,美食,爱情,教育就业,投资理财,时尚,星座命理,娱乐

校园生活,搞笑幽默,贵阳生活,情感生活
徐州生活
文字
旅游
视频音乐,新闻趣事
星座命理,旅游,美食,娱乐
教育就业,时尚,美食,六百,动漫,宅女,睡觉,摩羯座
投资理财
时尚,IT 数码,娱乐
美食
时尚,教育就业,健康,美食,娱乐
IT 数码
新闻,看书,自由
投资理财
游戏动漫
游戏动漫,美女,美食,娱乐,IT 数码
新闻资讯,美食,文艺,娱乐
篮球,吃
投资理财,旅游,星座命理,美食,IT 数码
游戏动漫,美食,星座命理,美女
睡觉,双鱼座,娱乐
美食,娱乐,旅游,声音控,电影,音乐,乐观
娱乐
搞笑幽默,穿衣美容,视频音乐
时尚
新闻资讯,教育就业
时尚,游戏动漫,美食,娱乐,动漫,天蝎座
搞笑幽默,旅游,美食
汽车,时尚,旅游,星座命理,旅行,音乐,偶喜欢玩,双鱼座,90 年的 80 后
美食
投资理财
看书,动漫,天蝎座,校园
旅游,苏州生活
喜欢工作
体育
视频音乐,旅游,IT 数码,校园生活,榆林生活,有为青年
美女,旅游,文艺,汽车,军事
文艺,旅游,景观
电子商务,数据挖掘,数字营销,鞋子比天高,头发怨水长
搞笑幽默,80 后,动漫,乐观,吃,水瓶座
汽车,美女,美食,健康
宅
语录
汽車,體育,旅遊,美食,IT 數碼

汽车,IT 数码,健康,投资理财,旅游
英语,自由,宅,听歌,文字,乐观,NBA,大学生,历史,90 后
美食
摄影,文字
温柔,music,爱旅行,快乐,活泼,阳光,苹果,85 后,爱生活,电视剧
健康
美食,游戏动漫,动漫,善良,游戏,小说
麦霸,打篮球,真诚
汽车,时尚,IT 数码,娱乐,美女
善良,囧
健康
游戏动漫
美食
天秤座,吉他,吃货,90 后,乐观,校园
旅游,星座命理,文艺,娱乐,美食
美食
娱乐,体育,IT 数码,游戏动漫,美食
娱乐,时尚
游戏动漫,文艺,美食
娱乐
游戏动漫
文艺
新闻资讯,看书,听歌
旅游
旅游,萌宠,新闻趣事,穿衣美容,山西生活
健康,体育
娱乐,80 后,爱唱歌的疯子,喝点小酒
IT 数码,星座命理,新闻资讯,健康,教育就业,旅游,文艺
IT 数码
教育就业,健康,旅游,美食,文艺,音乐,绘画,生活
娱乐
育儿百科,视频音乐,美容服饰,美食,潮州生活
旅游
善良
娱乐
汽车,军事
美食
时尚,旅游,星座命理,文艺,娱乐
GTDer,程序员
文艺,星座命理,旅游,健康,游戏动漫
团购网站,摄影旅游,美女,互联网,交友
时尚,星座命理,美食
時尚,教育就業,娛樂,学生,90 后,唱歌
文艺,健康,旅游,美食,游戏动漫

汽车,美食,健康
星座命理,娱乐
旅游
宅男,历史,80后
穿衣美容,90后,旅行,亲情,善良,大学生
善良,搞笑,电影,上网,运动,双鱼座
文艺,美食,军事,旅游,新闻资讯,美女,汽车
唱歌,射手座
教育就业,健康,美食,娱乐,文艺
体育
网络
搞笑幽默
广告人,房地产,篮球爱好者,家居
文艺
旅游,唱歌去旅行,睡觉
上海生活
IT数码,教育就业,时尚,新闻资讯,健康,旅游,文艺
听歌,123
体育
舞蹈爱好者,善良,上网,自由,音乐,好性格
文艺,猫和老鼠,漫画,英语,文字
文艺,IT数码,爱陈翔,神经质。
美食
校园,网络,美女,阅读,吃,娱乐,看书,90后
游戏,宅,乐观,音乐,交友,电子商务,摄影爱好者,吃,时尚,90后
体育
手机
音乐,葫芦丝,小号,萨克斯风,汽车,摄影,旅游,游泳,射击
狮子座,交友,亲情,80后,美女,善良,八卦,吃货,运动
娱乐
娱乐,宅女一枚,生活
教育就业
星座命理,吉他控,天蝎座,90后
娱乐,文艺,美食
娱乐
文艺
美女
射手座,睡觉,搞笑,80后,宅,创业,旅游,时尚
健康,新闻资讯,娱乐
星座命理,新闻资讯,旅游,时尚,汽车,教育就业,娱乐
时尚,娱乐,90后非脑残
不太信邪的80后,相声,音樂,汽车,爱美食
汽车,游戏动漫,IT,水瓶座,幽默,上网
旅游,爱笑

娱乐
营销,时尚,美食,搞笑,电影,吃,幽默,上网,网购
医生,时尚
数码
美食,美女,新闻趣事,搞笑幽默,湖北生活
投资理财,旅游
星座命理
文艺
美女,乐观,80后
军事,美女,娱乐,体育,旅游,汽车
游戏动漫,美食
打篮球,看美女
星座命理,健康,军事,旅游,娱乐
美女
好书,生活
搞笑幽默
旅游,美女,投资理财,IT数码,新闻资讯
健康,投资理财,文艺,IT数码,旅游
娱乐
美女
名人明星,青岛生活,视频音乐,新闻趣事,搞笑幽默,体育
羽毛球,旅游,双子座
上海生活,美食
名人明星
时尚,健康,旅游,美食,文艺
善良,文学,自由,摄影,网络,摩羯座,历史,爱情,看书,唱歌
时尚,旅游,健康,偶稀饭睡觉觉,娱乐,大学,天蝎座,宅男,好性格,80后
娱乐
动漫,双子座
汽车
旅游
体育
新闻资讯,娱乐,旅游,美食,文艺
游戏动漫
旅游,购物狂
星座命理,穿衣美容,美食
IT数码,星座命理,游戏动漫,体育,娱乐
体育,游戏动漫
旅游,IT数码,汽车
娱乐
旅游,美食
美食,时尚,娱乐
读书,平常心,摩羯座,自由,好书,紫色,宅,亲情,看书,旅行
游戏动漫,时尚,旅游,星座命理,IT数码

时尚,文艺,娱乐
星座命理
美食,文艺,娱乐,时尚,听歌,学习 ing,荷花,巨蟹座,宅,95 后
投资理财,美食,军事,文艺,体育
微博
教育就业,游戏动漫,军事
体育
宅女,财经
宅女,自由职业,唱歌
游戏动漫
旅游
体育
理财,淡定,浪漫,双子女
文艺
Jessica 郑秀妍,YoonA,TaeYeon,SONE
汽车,时尚,IT 数码,美女,娱乐
投资理财,美食,星座命理,旅游
娱乐
森林风,原宿风,夏天控,黑白控,小清新,摄影,学生,摩羯座 B 型女,90 后
自由,旅行,写作,听歌,文字,双鱼座
文艺,财经,音乐,动漫,善良,读书,搞笑,校园,艺术
游戏动漫,时尚,IT 数码
健康,娱乐,投资理财,IT 数码,旅游
旅游,名人明星
搞笑幽默,微博奇葩
旅游美食爱好者,旅游
健康
新闻趣事
新闻趣事,搞笑幽默
娱乐
听歌,网购
善良的存在着,乐观好心态,90 后学生,处女座
时尚,汽车,健康,投资理财,旅游
网购,微博
新闻资讯
体育
星座命理,天蝎女
羽毛球,电影
吃,爱情
娱乐
时尚,美女,美食,旅游,娱乐
汽车,军事,健康,教育就业,苏大,微笑,皇马
美容服饰,宅女,吃货,双鱼座
文艺

广州求助,广州飘,八卦
微博奇葩,萌宠,美女,新闻趣事,视频音乐
搞笑幽默
自由
娱乐
文艺,美食,汽车,IT 数码,美女,新闻资讯,体育
美食
每天都有好心情,宅女
体育资讯
娱乐,IT 数码,旅游,游戏动漫
偏执狂,新闻资讯,健康,教育就业,星座命理,娱乐,IT 数码,旅游
小清新,温柔,浪漫,模特,瑜伽,韩剧,独立,80 後,美容,文艺
时尚,投资理财,健康,旅游,文艺
不自信,爱军事,爱国,关心时政,求关爱,胖纸一枚
乐天派,美食爱好者,努力,双鱼,o 型血,妈妈团,玩具
文艺,时尚,教育就业,旅游,IT 数码,投资理财,军事
教育就业,美食
名人明星,游戏动漫,娱乐
星座运势,疼自己爱自己
低调,善感
健康,教育就业,时尚,文艺,美食,阅读,旅行,水瓶座加 AB 型,宅生活,学生
游戏动漫
美食,爱吃
Tee,levis,lee,北美代购
美食,新闻资讯,健康,教育就业,投资理财
娱乐
娱乐
美食
星座命理
美食,街舞控,学生一族,搞怪,狗狗,欧美控,巨蟹座,唱 K,o 型血,吃货
健康,旅游,美食,文艺
名人明星
娱乐,文艺
娱乐
穿衣美容,旅游,上饶生活
偶稀饭睡觉觉,巨蟹座,善良,听歌,学生,交友,浪漫,偶喜欢玩,英语,旅行
学生,小说,偶喜欢玩,天蝎座,紫色,吃,宅,偶稀饭睡觉觉,听歌,90 后
时尚,娱乐,美食
新闻资讯
芯片,Bigbang 的 VIP,刘忻,bigbang,金牛座,吃货,听歌
动漫,学生,创业
时尚,游戏动漫
星盘,塔罗,艺术,私房烘培
可爱,爱笑,巨蟹,向日葵,旅游爱好者,吃货一枚,健康,美食,青春

旅游
新闻趣事
IT 数码,娱乐
校园,大学
娱乐
教育就业,游戏动漫,美女,旅游,IT 数码
星座命理,80 后,音乐,善良,好性格,宅女,艺术,电影
美食
旅游,文艺
IT 数码,欢迎大家关注我
娱乐
时尚,游戏动漫,汽车,娱乐,IT 数码
广东生活
睡觉
旅游
旅游
健康
军事,体育,IT 数码,数码,网络,学生
搞笑幽默,文艺
美食,唱歌,吃货,小说,摩羯座
美食,IT 数码,体育
传媒,网购
星座命理,文艺,新闻资讯,教育就业,汽车,投资理财,影视制作,养生美容,营销策划
潮人,生活大爆炸,游戏动漫,时尚
宅女一枚,自由,疯子,天平座
浪漫的滋味
汽车,游戏动漫,时尚,健康,文艺
IT 数码,平常心
IT 数码
摩羯座
美食,搞笑幽默,上海生活,星座命理,旅游
穿衣美容
游戏动漫,美女,娱乐
会理财的金牛座,金牛座
QQ421256586,读心,娱乐,魔术,音乐,IT
军事
美食
互联网,写作
美食,偶喜欢玩,80 后,水瓶座
做人以信仰为贵,做事以尽心为贵
魏晨,金钟大,摩羯座,学生一枚,旅游
平常心
新闻趣事,旅游,美食,穿衣美容,四川生活
时尚,听歌,美剧,美食

时尚,汽车,旅游,美食,读书,电影,偶稀饭睡觉觉,听歌,吃货
美图摄影,旅游,美女,文艺
广东生活,旅游,美食
美食
时尚
海贼王,低调,自由,天秤座
娱乐
美食,投资理财,旅游,健康,梦想,真诚,活泼,金融,阳光,爱生活
网游玩家,音樂,颜饭,漫画控,exo,驯鹿
广州爱情,广州求助,广州飘,八卦
娱乐
文学
娱乐
星座命理
视频音乐,娱乐
交友,篮球,睡觉,90 后
文艺,哈哈,偶喜欢玩,双鱼女
视频音乐,穿衣美容,旅游,江苏生活,星座命理
教育就业,文艺,军事,星座命理,娱乐
健康,睡觉,乐观,自由
健康,新闻资讯,IT 数码,自由,金牛座
旅游,美女
新闻趣事,语录,音乐,双子座
美女,美食,旅游,汽车,健康
娱乐,浪漫
金牛座,吃货,恐怖片,星座命理,美食,娱乐
喜欢唱歌,郑容和,偶像剧,唱 K,Music,o 型血,韩国,摇滚,时尚,娱乐
时尚,淘宝,美白,减肥,美容,互粉
IT 数码,军事,健康,文艺,体育
八卦
投资理财
文艺,美食,旅游,健康,投资理财
健康,新闻资讯
美女
美食,名人明星,星座命理,美女,穿衣美容
娱乐,旅游,时尚
文艺,体育,娱乐
美食,体育,娱乐,睡觉是头等大事,大大的吃货,电影,音乐,金牛座,搞笑,90 后
多才多艺好青年,美剧,巨蟹座,学生,感性与理智兼并,超级好心情,完美无瑕,智慧幽默,粉嫩桃子,动漫
IT 数码,自由,听歌,唱歌,旅游,电影
健康,美食
娱乐
娱乐

湖北生活
健康,教育就业,旅游,美食,娱乐,视频音乐,名人明星
新闻资讯,时尚,文艺,美食
时尚,娱乐,汽车
旅游
文艺,摇滚乐,刺青,刺青电影,狗狗，二货,心理学,爱美食,神经质,海贼王,欧美控
名人明星
传媒
星座命理,旅游
健康,时尚,IT 数码,体育,娱乐
体育,打篮球，,踢足球
美女,军事,IT 数码,体育,娱乐
黑牡丹,自由职业,睡觉,旅行,动漫,美女,篮球,射手座,囧
时尚,宅女
体育
娱乐
娱乐
執著,樂觀,听歌,90 后,篮球,小说
健康
军事,娱乐,体育
体育
搞笑幽默,巨蟹座
八卦,玩儿
活力工场,HLGC 小黑
娱乐,文艺,天秤座,唱歌,看书,书法,听音乐。
美女,体育,娱乐
娱乐
文艺
汽车
新闻资讯,时尚,美食,IT 数码,文艺
旅游,天秤座
狮子座,囧,90 后
运动,旅游,纽约,天津,文学,新闻,摄影爱好者,美食,历史,电影
搞笑幽默
学生一族
金牛座,宅女,美女,交友,自由职业,时尚,上网,听歌,旅游,80 后
娱乐
娛樂
美食
娱乐
双鱼座
IT 数码,时尚,投资理财,汽车,体育,80 后,篮球,听歌,上网,电影
青春,真诚,巨蟹座女生,幽默,旅行,宅,音乐,善良,看书,听歌
敏感,矛盾体,我就是我,交朋友,心情,喜欢唱歌

体育
军事,新闻资讯,教育就业,美女,健康
美女,游戏动漫,宅男,玩儿,生活,睡觉
娱乐
娱乐
名人明星,欧美疯疯疯,宅,旅行
娱乐,时尚,健康,星座命理,美食
视频音乐
娱乐,星座命理,文艺
爱幻想,安静,宅女一枚,室内设计,娱乐,小说,设计,90 后,双鱼座,音乐
健身,瑜伽,天蝎座
星座命理,旅游,健康,美食,文艺
唐山生活
娱乐
睡觉
娱乐
考研,绿色,双鱼座,90 后
健康,美女,整形美容专家,美丽变身
处女座,旅游,运动,旅行
名人明星
自信,幻想,爱睡觉,赚钱,幸福,独立,开心,喜欢旅游
爱玩,街舞,笑话,郑允浩,学生一枚,臭美,活泼,单身,阳光,旅游
时尚,文艺,娱乐,投资理财
IT 数码
时尚,星座命理,美食,投资理财,娱乐
慈善,白羊座,园林业,艺术,荆州,思考,湖州,成都,济宁,装饰装潢
军事,文艺,IT 数码,游戏动漫,汽车
热爱生活,住宅系统方案,绿色建筑,集成住宅,预制房屋
游遍中国,三峡大学,篮球迷,美剧控
手机,好书,校园,好性格
教育就业,健康,投资理财,文艺,星座命理
古代控,汉服君
星座命理
汽车
旅游
职场招聘,美食,旅游,美容服饰,育儿百科
幽默
车世界,美女,新闻趣事,搞笑幽默
旅游,星座命理
娱乐
时尚,教育就业,旅游,星座命理,文艺
体育资讯
搞笑幽默
美食,处女座,电影,听歌,紫色,喜欢天空

旅游,八卦,旅行,吃货,美剧,阅读,美食,幽默,巨蟹座,音乐
名人明星
星座命理
日本,文学,善良,90 后
晚睡强迫症,射手女,纠结的矛盾体,拖延症重症患者
北京生活
美食,投资理财,汽车,体育,美女
娱乐
健康,星座命理,新闻资讯,教育就业
旅游,美食,体育
娱乐
IT 数码
娱乐
汽车,教育就业,投资理财
90 后,游戏,网络,幽默,玩儿,八卦,英语,宅男,小说,白羊座
数码,美剧,网络,狮子座,偶喜欢玩,平常心,80 后
健康,旅游,星座命理,美食,文艺
创业,善良,吃货,生活
美食,互粉,,爱听歌,睡觉,唱歌,时尚,读书,囧,好性格,天枰
IT 数码
星座命理,教育就业,文艺
娱乐,健康,旅游,美食,教育就业
娱乐,星座命理,爱笑,公益志愿者,幻想,懒,沉默,听音乐,天秤
美食
足球,80 后
游戏动漫,听歌,自由,巨蟹座
广告,英语
名人明星
美女,交友,90 后,音乐,大学,英语,运动,平常心,宅
旅游
旅游,IT 数码,星座命理,娱乐
IT 数码
旅游,纯粹,自由,大学,校园,写作,好书,文学,文字
新闻资讯,旅游,健康,美食,时尚
文艺,新闻资讯,时尚,健康,旅游
健康,旅游,摄影,好性格,自由,天蝎座,旅行,交友,时尚
娱乐
健康
新闻资讯
成长 ing,星座命理,旅行,胡思乱想双鱼女,时尚,音乐,吃,善良,设计,旅游
投资理财,汽车,美食,新闻资讯,美女
电影,时尚,潮男,运动,美食,购物,搭配
健康,时尚,游戏动漫,星座命理,美食,文艺,娱乐
电影狂,体育狂,大男孩,闷骚,疯狂,细腻,饭后呆

搞笑幽默,汽车,益智,音乐,温州,85 后,三国杀,电影
汽车,IT 数码
新闻资讯,时尚,美女,IT 数码,体育
搞笑幽默
IT 数码,八卦,上网
旅游,美食,文艺,娱乐
游戏动漫,动漫,最爱黄山
军事
教育就业,汽车,体育,新闻资讯,IT 数码
美女,娱乐
我爱诗爷
微博奇葩,萌宠,美食,旅游,视频音乐
媒体,NBA,营销,新闻,幽默,创业
萌宠
旅游,投资理财
90 后,白羊座,Bigbang
电影,交友,旅游
美女,90 后,学生,音乐,玩儿,吃
时尚,80 后,网络
汽车,文艺,时尚,美食,娱乐
投资理财,汽车,游戏动漫,健康,娱乐
星座命理,萌宠
牙齿矫正,口腔正畸,教育就业,健康
汽车,时尚,健康,旅游,娱乐
生活,善良,搞笑
美女,宅,美剧,处女座
娱乐
爱旅行,房地产,天蝎座,80 後,nba,无厘头,哈哈,阳光,汗水,运动
娱乐
自己选择自己,宅,生活,乐观,唱歌,时尚,善良,上网,幽默,金牛座
投资理财
吃货一枚,学生,爱自由,处女座,爱旅游,活泼开朗,唱歌,睡觉,上网,娱乐
游戏动漫,文艺,枪弓枪最高,森川智之,铃村健一,汪酱
新闻资讯
时尚,旅游,美食,投资理财,美女
美食
新闻资讯,时尚,美食,星座命理,健康
Android,北京移动,CMCC,中国移动,Google,IT 数码,网络
汽车,星座命理,美食
时尚
星座命理
体育,旅游
美食,娱乐
旅游

懒,安静,爱笑,双子座,吸血鬼日记,喜欢听歌,睡懒觉,猫咪,热爱生活,旅游美食
好性格
美食,名人明星,视频音乐,校园生活
时尚,游戏动漫
美食,赤坎党
美食
娱乐
安静
旅游
天蝎座
合肥生活,情感生活,美容服饰,交友,天蝎座,爱情,大学生,互粉
旅游
健康
IT 数码
美女,文艺
旅游,亲情,吃货,囧,巨蟹座
IT 数码,美女,游戏动漫,汽车,韦德
新闻趣事,文艺女青年,条纹控,宅女,搞笑,欧美
视频音乐
鞋店
教育就业,IT 数码
室内设计,电影,深圳,慎重,杭州
股票,水利,农业,苹果,常熟,狮子座,法律,哈尔滨,网络电子商务
旅游
军事天地,游戏动漫,军事,文艺,体育,娱乐
美女
美女
娱乐
体育
娱乐
IT 数码,文艺,体育,娱乐,旅游
旅游
睡美人
家具行业,家装建材,实木家具,家居,书房家具,餐厅家具,客厅家具,卧室家具,家具
娱乐
阿森纳
我一直都在,双鱼座
文艺,90 后,大学生,娱乐,听歌,旅游,天蝎座
游泳,读书,打球,旅游
旅游,健康,文艺
美食,狮子座,美女,设计
星座命理,微博奇葩,搞笑幽默,新闻趣事
投资理财,旅游
微博奇葩,穿衣美容,新闻趣事,搞笑幽默,名人明星

游戏动漫,星座命理,IT 数码,娱乐
文艺,娱乐,新闻资讯
英国生活,英国大学,同学会微博,英国留学,伦敦城市大学
美食,新闻趣事,搞笑幽默,福建生活
娱乐,体育,游戏动漫,旅游,文艺
健康,教育就业,军事
塔罗牌,理性白日梦,摩羯座,完美主义,不会去死症,美食
旅游
巨蟹座,唱歌,NBA,学生,羽毛球,电影,摄影爱好者,听歌,宅男,篮球
体育,时尚,娱乐,文艺
体育,娱乐,教育就业,军事
视频音乐
各種宅,吃货
娱乐,文艺,星座命理
娱乐,IT 数码,时尚,星座命理,美女
唱歌
孩子气,文化,自信,胡思乱想,阅读,潮流,感性,音乐控,喜欢音乐,爱唱歌
羽毛球,NBA,90 后,微博,上网,看书,麦蜜,英语,足球,篮球
旅游
教育就业,新闻资讯,美食,旅游,娱乐
娱乐
搞笑,冷笑话,James,篮球
游戏动漫,汽车,投资理财,文艺,娱乐
上海生活
体育,时尚,文艺,旅游,美食
星座命理
娱乐
教育就业,骑车旅游
美食,娱乐
IT 数码
搞笑幽默
星座命理,娱乐,时尚
教育就业,投资理财,健康,文艺,美食
教育就业,教育
搞笑,汽车,宅
游戏动漫,火黑党,爱手绘,爱动漫,初三党
新闻资讯,房地产评估,新闻,汽车
吃货,睡觉,狮子座,旅游,浪漫,搞笑,小说
投资理财,娱乐
娱乐
三明纹身,尤溪纹身,福州酒吧,纹身图案,洗纹身,时尚纹身,福州刺青,纹身爱好者,福建纹身,
福州纹身
爱睡觉,猫控,计算机,IT 数码,游戏动漫
时尚

美女,汽车,旅游,IT 数码,教育就业,美食,军事
文艺
做饭,运动
美食,汽车,军事,IT 数码,体育
赣州生活
旅游
汽车
美女,健康,新闻资讯
搞笑幽默
军事,新闻资讯
宠物图集,娱乐
星座命理,旅游
IT 数码
搞笑幽默,新闻趣事,其他生活,美食,旅游
旅游
娱乐,体育
美图摄影,VIP,李胜贤,TOP,权志龙,GD,BigBang
文艺,时尚,美食,娱乐
教育就业,汽车,星座命理,新闻资讯,美女
文艺
游戏动漫,动漫宅,腐女宅女耽美狼,宅女,巨蟹座,美食,创业,娱乐,睡觉,90 后
双重性格,我就是我,学生一枚,娱乐
美食
娱乐,模特
90 后
学生,随手拍,旅行,爱美食爱睡觉,轻微白衣洁癖,爱猫爱狗
职场招聘,汽车,星座命理,新闻资讯,军事
美食
阅读,爱情,亲情,幽默,美食
教育就业
体育
游戏动漫,军事,教育就业,旅游,文艺,自由,体育,小说,电影
高三,汽车,游戏动漫,新闻资讯,IT 数码,体育
动漫控,天蝎座,阅读,90 后,校园,吃货
交友,偶稀饭睡觉觉,吃,水瓶座,听歌,90 后,学生
美食,懒人,善良,简单
娱乐,乐观开朗的女孩,傻里傻气
教育就业,健康,旅游,IT 数码
娱乐
萌宠
娱乐,水瓶座
白羊座,手机,玩儿,好性格,搞笑
娱乐
健康,美食

新闻趣事
建筑,抖森,loki,RDJ,全职高手,三集片,银魂,POI
IT 数码
我就是我,学生一族,韩剧,微笑,美食,时尚
旅游
文藝
体育
重口味,音樂,矛盾体
美食,会计,90 後,睡懒觉,吃货一枚,乐天派
处女座
时尚
美女,美食
学习,动漫迷,搞笑,狮子座,网球
健康管理,乐观,玩儿,旅游,摄影爱好者,汽车,时尚,美食,阅读
美女
名人明星,看书,看电视
时尚,旅游,文艺
美女,萌宠,搞笑幽默
星座命理,文艺,娱乐,游戏动漫
旅游,美食
美女,投资理财,军事,美食,星座命理,文艺,体育
德阳,爱德阳,德阳爱情,德阳求助,德阳飘,八卦,吃喝玩乐
娱乐
篮球
旅游
IT 数码
军事,时尚,健康,娱乐,星座命理
英语
陕西生活
星座命理
新闻资讯,忧郁,美国
求基友,爱媳妇,纯情小男生,90 后,交朋友,爱生活,天蝎座
星座命理
偶稀饭睡觉觉
拍照,旅游
微博奇葩,星座命理,穿衣美容,名人明星
美女
美食
IT 数码
美女,幽默
跑步,执着,睡觉睡到自然醒,强迫症,爱自由,爱音乐,随性,看小说,淡定,娱乐
新闻趣事
济宁,保护他人,想象力,慈善,审计,牧业
旅游

娱乐
旅游,健康
投资理财,时尚,旅游,娱乐
旅游
美食,时尚,娱乐
尼伯龙根的指环,照相,音乐,财迷,内科,呼吸与危重症
星座命理
娱乐
时尚,旅游,美食,文艺
体育
旅游,星座命理,投资理财,娱乐
旅游,双子座女生,80 后的末班车
文艺,IT 数码,旅游
娱乐
文艺
健康,时尚,旅游,美食,文艺,旅行,电影
巨蟹座,电影爱好者,旅游
美食,摄影师,饭店,广告
平面设计,软件,大学生,设计,dota,品牌,游戏动漫,文艺
情感生活,新闻趣事,美食,搞笑幽默
美女,汽车
冰淇林,篮球,游戏动漫
新闻资讯
教育就业
微博奇葩,搞笑幽默
新闻趣事,旅游,名人明星,美食,安徽生活
唐禹哲,小说,动漫
体育篮球,自由,NBA,大学生,90 后,双鱼座,运动,宅,上网,音乐
文艺,投资理财,旅游
寒暑假工,求职,90 后,80 后,招聘
音樂,美食,强迫症,电影控
游戏动漫,汽车,体育,IT 数码,美食
骨灰级 dota 玩家,top,BIGBANG,中国第一 vs
健康,旅游,体育,娱乐,时尚
吉他,天秤座,动漫,宅,90 后
时尚
体育
旅游,健康,时尚,美食,娱乐
云南生活
平安创建,服务民生
旅游,美食,萌宠,穿衣美容,星座命理
搞笑幽默,微博奇葩
Maroon5,dota
北京生活

文艺,美女
微博奇葩,娱乐
游戏动漫,娱乐,旅游,美食,体育
汽车
上网,篮球,运动,学生
太原,爱太原,太原爱情,太原求助,太原飘,八卦
好书
教育就业,时尚,美食,文艺
时尚,IT 数码,设计,90 后,电影,双鱼座,旅行,睡觉,上网,听歌
文艺
搞笑幽默
旅游,汽车,体育
投资理财,星座命理,军事,游戏动漫,教育就业,IT 数码
娱乐,文艺
娱乐,旅游,健康
新闻资讯,健康,旅游,军事,时尚
休闲,天枰座,旅游爱好者,健康
白羊座,搞笑,睡觉,90 后,吃货
IT 数码
娛樂,新聞資訊,美女,美食,旅遊
生物,双鱼座,旅游,深圳,牺牲奉献,计算机软件,海口
娱乐
搞笑幽默,北京生活,美食,旅游
吃货,睡觉,90 后,时尚,生活,网购
投资理财
美食
旅游
时尚
视频音乐
健康
体育,娱乐
体育
娱乐
时尚,投资理财,旅游,娱乐,IT 数码
游戏动漫,娱乐,星座命理,美食,文艺
汽车
好脾气,哈尔滨,义乌,执着
汽车,健康,新闻资讯,时尚,旅游
交友,八卦,摩羯座
爱情,白羊座,平常心,生活,交友,乐观,善良,大学生,校园,学生
爱睡觉,画画,娱乐
投资理财,美食,星座命理,健康,时尚
旅游,健康,新闻资讯,文艺,体育
旅游

文艺
穿衣美容,名人明星,旅游
白羊座,听歌,英语,幽默,搞笑,运动,交友,美剧,唱歌,时尚
新闻趣事,Zshengs,LightVision,光源视觉设计,光源潮品,光源映画
旅游
美女,娱乐
美食,唱歌,营销,睡觉,善良,摄影,吃货,水瓶座,旅游
文艺
娱乐,教育就业
游戏动漫
娱乐,文艺
80 后
美食
数码,浪漫
游戏动漫,娱乐,桌游控,自由,篮球,好性格,睡觉,吃货
美女
娱乐
文艺,时尚,旅游,娱乐
旅游,健康,美食
投资理财,旅游
投资理财,体育
文艺
星座命理,IT 数码,游戏动漫,军事,体育
娱乐,健康,投资理财,汽车,美女
体育
旅游
教育就业,旅游,美食,投资理财,新闻资讯
美女,好书
娱乐
新闻资讯
美女,游戏动漫,时尚,IT 数码,早教,落六者,站长
读书分享,搞笑幽默,视频音乐,美女,美食,娱乐,体育,旅游,军事,DOTA
旅游,80 后,摄影,旅行,双鱼座
强迫症,懒人吃货,爱自由旅游,宅腐双修,双重性格,银魂,摄影爱好者,购物,游戏动漫
美食,娱乐,汽车,体育
少女时代,横山由依,松井珠理奈,渡边麻友,篠田麻里子,AKB48,宫泽佐江
新闻资讯,健康,教育就业,投资理财,娱乐
游戏动漫,汽车,体育
汽车
旅游
美食,娱乐,Ohhh 热恋小摩羯
军事,文艺,体育,娱乐
军事,时尚,IT 数码,文艺,体育
视频音乐,微博奇葩

星座命理
幽默,白羊座,爱情,小说,听歌,时尚,宅女,电影,音乐,90后
星座命理
地铁拉手,广告人爱时尚,主持,80后的尾巴,策划,微笑,法语,双子女,搞怪,旅游
太原,爱太原,太原爱情,太原求助,太原飘,八卦
体育
阅读,音乐,天蝎座,90后,动漫,宅女,学生,吃货,吃,小说
旅游,星座命理,健康,时尚
投资理财,健康,时尚,文艺
星座命理,旅游,美食
投资理财
旅游
无厘头,双重性格,手机看小说,吃货一枚,爱吃,宅女,小说,吃货,神经质,美食
美食,健康,旅游,左溢,菜鸟,简单,听歌
星座命理
星座命理,汽车,时尚,旅游,体育,娱乐
时尚,搞笑,玩儿,白羊座,囧,平常心,80后
微博奇葩,游戏动漫
旅游
IT数码,美食,娱乐
美女
健康,美食,旅遊
美食,短发控,制服西装控,正装控,男男爱
摄影爱好者,123
体育,军事,时尚,游戏动漫,美女
星座命理,健康
鞍山生活
游戏动漫,美食,玩儿,摩羯座,旅游
视频音乐,热爱,反省并自由
星座命理
旅游
新闻资讯,时尚
健康,投资理财,旅游
体育
文艺,旅游
情绪化,矛盾体,宅女加腐女,爱玩,强迫症,单身,小女人,巨蟹座,爱狗狗,杭州
军事
文艺,军事,IT数码,体育,娱乐,美食
新闻资讯,美女,游戏动漫
亲子,教育,育儿,深圳南山,剑桥英语,英语爱好者,英语培训,外教口语,英语口语,英语学习
活泼可爱的傻瓜,偶稀饭睡觉觉,时尚,娱乐
旅游,谛听风的轻吟
娱乐
美食

品味,时尚,自由,星座,娱乐,看书,旅行
白羊
文艺
美食,美女,军事
微博奇葩
健康,旅游,美食,娱乐
美女,游戏动漫,时尚,文艺
美女
爱吃,星座命理
视频音乐,微博奇葩,名人明星,新闻趣事,搞笑幽默
美食,星座命理,视频音乐,旅游,搞笑幽默
特别宝贝金英云,西皮如戏不嗜好,不饭饭圈,不饭巨星,贤敏暖心饭,最近偏好敏旭,李赫宰男神,初心兼大宝贝敏,不唯数字,SUJU 为底线
星座命理,天秤座,搞笑,时尚,互联网,创业,90 后,微博,唱歌,动漫
计算机爱好者,微博控,科技控,简单,黑客,网络一哥,手机控,软件编程师,搞网站,IT 数码
乐观,狮子座,微博,吃货,自由,网络,旅游,电影,音乐,唱歌
时尚,军事
星座命理,旅游,健康,美食,教育就业
睡觉,音乐,乐观,校园,80 后
网游,环保
娱乐
微博奇葩,旅行
体育,创业,美剧,美食,NBA,时尚,汽车,旅游,篮球
旅游,文艺,时尚,美食,囧,90 后
新闻资讯
摄影,偶喜欢玩,吃货,90 后,水瓶座,偶稀饭睡觉觉,交友,网络,好性格,听歌
时尚
时尚,美食,文艺,娱乐,汽车
美食,健康,旅游,娱乐,文艺,搞怪,电影,影人,2B 青年欢乐多
新闻趣事
转悠,爱幻想,怀旧,旅游美食
美食,体育,美女,汽车
穿衣美容
美食
自由,大学
美食,健康,旅游,美食,星座命理,文艺
体育
汽车,旅游,投资理财,体育,时尚
旅遊
旅游
娱乐
视频音乐
双子座女生,爱阿狸,八卦,读书,时尚
美女

健康,文艺,时尚,投资理财,旅游,美食,娱乐
旅游
美食,上网,宅,睡觉,90 后,狮子座,体育,动漫
娱乐,体育
旅游,时尚
IT 数码
爱睡觉,高中生,爱情,唱歌,上网,听歌,娱乐,小说,美剧,电影
旅游,自由,旅行
美女
娱乐,钟汉良,小哇,良民,蒙古族,水瓶座,旅游
名人明星,二宫和也颜饭,大宫严重洁癖饭,arashi 团饭
时尚
美女,时尚,健康
教育就业,时尚,IT 数码,文艺,娱乐
健康,星座命理,时尚,美食,体育
美食,旅游,教育就业,健康,时尚,娱乐,咖啡,睡觉,喜欢旅游,90 後
道具控,常年挂连拍,射精狂,僞设计,僞艺术,反骨作风,游离状,照相
穿衣美容,娱乐
娱乐,90 后,爱狗,爱猫,金牛座,吃货,学生,闷骚
文艺
视频音乐
IT 数码
娱乐,星座命理,旅游
旅游,游戏,交友,上网,摩羯座,自由职业,自由
IT 数码
旅游,新闻资讯,健康,教育就业,投资理财,娱乐,美食
旅游
星座命理,美食
健康,娱乐
投资理财,时尚
旅游,娱乐,美女,美食,浮夸
旅游
娱乐
建筑,热情,乐天派,牛哄哄
小说,八卦,手机,绘画,时事,收藏,双鱼女
宅,吃
汽车,星座命理,健康,投资理财,娱乐
敏感,情绪化,孩子气,学生一枚,任性,浪漫,时尚,旅游,美食
军事
娱乐,用心做好每件事,真诚对待每个人
美女
游戏动漫,娱乐,IT 数码
美女,时尚,汽车,教育就业,文艺
搞笑幽默,文字,电影,看书,睡觉,吃货,宅

韩寒,星座控,情感,基督徒,医学,天秤,死神,谢娜
文艺,连接服务器失败,陈奕迅,双鱼座,听歌
健康
茶农,喜欢喝茶,养壶,茶友,壶友,武夷岩茶,大红袍
娱乐
健康,美女,旅游,美食,娱乐
可爱,音乐爱好者,爱幻想,交朋友,高三
IT 数码,美食
双子女,云南生活,娱乐
钢琴,相声,意大利,猫咪,音乐控,养生,艺术,古典音乐,法国,健康
文字,电子商务,无聊,双子女,工作狂,皇马
游戏动漫
紫色,化妆,书,电影爱好者,孩子气,武汉
吃货,SHINeeWorld,B 型血,狮子座,小说控,80 后的尾巴,麦霸
投资理财,军事,美女
文艺
体育
娱乐,时尚
美女,汽车,娱乐
美食,囧
投资理财
星座命理
数码
美女,时尚,文艺,游戏动漫,成长,ing
娱乐
读书,搞笑,网络
李玉刚,幸福,旅游爱好者,服装
卖萌
娱乐,汽车,美女,美食,军事
娱乐
美女,乐观,音乐,NBA
语录,微博控,日语,动漫控,日本,欧美控,卡卡,随性
视频音乐,体育
旅游,美食,游戏动漫,狮子座,动漫,睡觉
美女,游戏,上网,吃货,偶喜欢玩,射手座,小说,偶稀饭睡觉觉,宅,90 后
时尚,汽车,旅游,美女,IT 数码
动漫,电影
美女,健康,新闻资讯,汽车,游戏动漫,旅游,美食,投资理财,娱乐
汽车,游戏动漫,美食,军事
娱乐
巨蟹座 O 型血,美剧,新闻,设计,看书,阅读,通颜觉罗氏,镶蓝旗,满洲族,旅行
美食
大学生,爱睡觉,许嵩,林宥嘉
北京生活

手机,天蝎座
新闻趣事
健康,Pong迷,吃货,宅,旅行
双鱼女,爱睡觉,重口味,足球,美剧控,敏感,星座,旅游,八卦
美食
旅游
神经大条,时尚,旅游
美食,旅游
游戏动漫,文艺,体育,娱乐
民生,社会,运动,历史,读书,音乐
新闻资讯,美女,IT数码,文艺,文艺青年,音乐,时尚,90后,娱乐,周杰伦
篮球,养生,摩羯,歌手,卡卡,科幻
新闻资讯
宅女一枚,吃货,天蝎,美食,文艺
平常心
新闻,爱生活,简单,宅女一枚,情绪化,摄影控,吸血鬼日记,生活大爆炸,魔域
星座命理
文艺
搞笑,股票,微博,体育,星座,fashion,桌球,赛车
搞笑幽默
娱乐,80后,篮球,摩羯座,旅游,美食
军事,喜欢修东西,IT,搞笑,电影,设计,宅男,玩儿,汽车,摩羯座
投资理财,教育就业,美食,游戏动漫,IT数码
汽车,学生,美食,乐观,吃,看书,幽默,紫色,动漫
星座命理,美女,IT数码,游戏动漫
汽车,海淀区,美术老师,儿童美术,创意,儿童教育,家教,画画
运动
乐正绫,动漫
文艺,旅游,时尚,健康,投资理财
娱乐
游戏动漫
时尚,美食,健康,教育就业,体育
美食,健康,投资理财,英语
美女,80后
娱乐
射手座,文学
娱乐
單車,宅,照相
星座命理,时尚,旅游,美食,娱乐
视频音乐,名人明星,美食,旅游
娱乐
娱乐
浪漫,爱英语,爱美女
新闻资讯,旅游,娱乐,汽车,军事

强迫症女王,资源控,哲学感悟,听歌,旅游,电影,阅读,设计,自由职业,摄影爱好者
娱乐
运动,咖啡,强迫症,情感,法语,死神,jyj,炎
教育就业
美食,娱乐
视频音乐
紫色
新闻资讯,星座命理,IT 数码
小说,80 后
美图摄影,视频音乐,情感生活,美食
真实力,人力资源,重庆,公关
体育
财经,电子商务,宅男,八卦,美女,创业,媒体,明星,婴儿,自由职业
健康
温柔,小女人,电视,学生一族,宅女一枚,演员,爱吃,唱 K,美女,好性格
文艺,偶喜欢玩,旅行
搞笑幽默,微博奇葩
减肥,帅哥,浪漫,双重性格,旅游美食爱好者,偶喜欢玩,自由,纠结,音乐,天秤座
体育,娱乐
爱情,囧
汽车,美食,健康
投资理财,IT 数码,旅游,汽车
汽车,美食,IT 数码,文艺,娱乐
军事爱好者
时尚,旅游,星座命理,娱乐,美食
视频音乐,天然呆,90 后宅女,唱歌
娱乐
娱乐,星座命理,美食,投资理财,健康
游戏,腐,摇滚,低调,elf,搞怪
星座命理
新闻趣事,文艺
丹东生活,玩,美食控,懒
吃货
时尚
文艺,看书,小说,动漫
篮球,it,厦门,微笑
时尚
时尚,娱乐
健康
旅游,体育
微博,搞笑,音乐,八卦,幽默
文艺,娱乐,教育就业,新闻资讯,星座命理
娱乐
IT 数码,美食,娱乐

投资理财,体育
搞笑幽默,读书分享,星座运势,宠物图集,新闻趣事
投资理财
为您们做的更好
旅游
娱乐
看书,摄影,喜欢听歌,热情,创意,阳光,写作,90後,爱旅游,开朗
90后,旅游,浪漫,娱乐,搞笑,幽默,唱歌,时尚,音乐,听歌
新闻资讯,游戏动漫,军事,教育就业,美食,文艺
视频音乐
投资理财,军事,旅游
美女,八卦杂谈,星座运势,新闻趣事,搞笑幽默,90后,摩羯座,室内设计师,奋斗ing
香酥,香脆,的大鸡排
海外生活
吴世勋,朴灿烈,krisyeol
娱乐,双子座,电影,吃
八卦
文艺,娱乐
美女,军事
学生,平常心,宅男,吃,音乐
边村村民,灿白,文艺女青年,小清新,卷福,神探夏洛克,吃货,函数妹纸,双子座,宅女
旅游,文艺,娱乐
美食,旅游
星座命理,旅游,新闻资讯,娱乐
旅游
搞笑幽默
美女
好性格,看书
娱乐
穿衣美容,文艺
娱乐
美食,星座命理,娱乐
汽车,星座命理,美食,旅游,健康
大学生,美女
娱乐
文艺
美食,健康,时尚,旅游
小说,自由,乐观,好性格,双鱼座,唱歌,运动,篮球,动漫
心理学,电脑,电影迷,爱,基督徒,热情,日剧控
投资理财
睡觉,发呆,吃
网络
美女,视频音乐,搞笑幽默,淮南生活,文艺,体育,娱乐,IT数码
汽车

健康
快乐,闷骚,cosplay,欧美,天秤
星座命理,文艺
娱乐
星座命理
微博,吃货,gay,书法,陈翔,我就是我,苹果控
羽毛球
旅行,美食,浪漫,唱歌,上网,听歌,90后美男
美食,大学,宅,宅女,80后,睡觉,好性格,乐观
IT数码,IT
旅游,文艺,军事
星座命理
娱乐
穿衣美容
体育,旅行,白羊座,阅读,NBA,80后,美食,篮球,运动
美女
山东生活
星座命理,自由,吃货,偶喜欢玩,亲情,小说,羽毛球,听歌,水瓶座
旅游,美剧
天秤座,写作
娱乐
体育
摄影,音乐,读心,自由
看小说,沉默,o型血,汽车,摩羯座,旅游
宅
娱乐
健康,旅游,时尚,星座命理,娱乐
娱乐
健康
娱乐
微博奇葩,星座命理,搞笑幽默,内蒙古生活,自由,时尚,旅行,听歌,音乐
搞笑幽默
上海生活
美图摄影,偶稀饭睡觉觉,网购,听歌,电影
美食,娱乐,健康
音乐,善良
听歌,搞笑,偶稀饭睡觉觉,手机,巨蟹座
游戏动漫,IT数码
让自己能开心,自由,天秤座,爱情
军事
育儿,减肥达人,瘦身,高端妈妈
时尚
娱乐,交友
娱乐

游戏动漫
重庆生活
80 后,上网,天平座,商业,金牛
完美主义,音乐爱好者,音乐控,吃货一枚,音乐发烧友,睡懒觉
娱乐
美图摄影,娱乐
湖南生活
潮流,跆拳道
病态的变态
互联网
时尚,投资理财,教育就业,健康,娱乐
时尚
爱笑,烹饪,无限正能量,青春,摄影爱好者,美食,旅行,个性摩羯女,我爱江直树,快乐 90 后
新闻资讯,IT 数码,体育,乐观,旅游,旅行,80 后,天秤座,篮球,听歌
萌宠
九零后,苦逼青年
时尚,健康,旅游,教育就业,投资理财
娱乐,体育
90 後,海贼王死粉,台球,白羊座,WOW,psp,音乐控,汽车
体育
羽毛球
美食
投资理财,新闻资讯
业余老师,高中英语,高中,思维导图,记忆,记忆力超常,英语学习,英语爱好者,背单词,单词
我是水瓶座
汽车,为人民服务,A 咖,渴望独立,天秤座,小本本想飞行
睡觉睡到自然醒,时尚,咖啡,休闲,90,逛街,听音乐,爱生活
音乐,动漫
美女,娱乐
萌宠,美食,名人明星,潮州生活,新闻趣事
双子座,新闻资讯,Cynthia
美女,游戏动漫,二点五次元,无下限吴杰超,ALL 黑党,黑子哲也,宅腐双修
喜欢童趣
美女,汽车,文艺,体育,时尚
健康,游戏动漫,星座命理,娱乐,文艺,90 后,宅男,天秤座
新闻趣事
旅游
娱乐
上海生活,新闻趣事
视频音乐,美食,旅游,微博奇葩,搞笑幽默
微博奇葩,爱搞怪,爱嘻嘻哈哈,爱休闲,爱大笑,90 后
处女座,医学,宅女加腐女,喜欢听歌
汽车,时尚,游戏动漫
亲情,巨蟹座,语录,创业,90 后,浪漫,音乐,自由,平常心,文学

时尚,汽车,文艺
视频音乐,天蝎座,自由,90 后,旅行
游戏动漫
唱歌,宅,看书,睡觉,电影,吃,平常心,宅女,听歌,80 后
睡觉睡到自然醒,爱旅游,广州,90 後,互粉,奋斗,好性格
成都,gossipgirl,谢娜,相声
时尚
搞笑幽默
军事,星座命理
IT 数码
时尚
旅游
IT 数码,游戏动漫
好性格,狮子座,浪漫,学生
娱乐,K 歌,健身,音乐迷,欧美,大学,金牛座,NBA,幽默,宅
时尚
美女
美容服饰,旅游,美食,米秀珉,东方神起 SJ,死团饭,允在天道,金在中,东方神起,JYJ
娱乐
平常心,听歌,美剧
汽车,文艺
时尚,健康,IT 数码,文艺,娱乐
二逼青年,娱乐,健康,文艺,时尚
游戏动漫
搞笑幽默,艺术,射手座,摄影
投资理财
新闻趣事,视频音乐,旅游,搞笑幽默
健康,美女,汽车
游戏动漫,IT 数码,自尊心,建筑,农业,天秤座,柏拉图式的爱,制药,廉耻心,江阴
民工甲
IT 数码
旅游,美食,星座命理,投资理财,娱乐,异想天开,狮子座,宅女
疯子,城市猎人,绘本控,白羊座
摄影爱好者
韓劇,繁星蛋白,清晨的小鹿,边伯贤咸咸 QAQ,张艺兴本命
星座命理
旅游,美食,娱乐
娱乐,游戏动漫,IT 数码,小说,摩羯座,90 后,囧,电影,手机,宅
游戏动漫,体育
校园生活
浪漫
微博,宅女,营销,白羊座,旅游,娱乐,创业,网购,广告,美女
旅游
爱情,睡觉,唱歌,80 后,双鱼座

射手座,好书,学生
健康,旅游,新闻资讯,娱乐,美食
军事,IT 数码,娱乐
微博奇葩,军事,历史,文学
娱乐
美女,新闻资讯
张根硕的鳗鱼,夏茗悠
星座运势
美食,强迫症,90,学生
新闻趣事,视频音乐,读书分享,摩羯座,动漫,小说,学生
文艺
唱歌歌,电影,骑车,好人,汽车,游戏动漫,美女
旅游
时尚,军事,文艺,新闻资讯,健康
星座命理
文艺
娱乐,时尚
遊戲動漫,动漫,双子座
星座命理
股票
旅游
文艺,音乐,电影,双子座,儿童节,随性
文艺
投资理财,文艺,娱乐,军事
足球
旅游
体育,时尚,新闻资讯,文艺,旅游
滨州生活,自酿葡萄酒,葡萄酒酿造师,酿葡萄酒,葡萄酒爱好者,娱乐
听歌,90 后
星座命理
乐观,双鱼座,动漫,听歌,旅游,艺术
文艺
自由,音乐,浪漫,运动,汽车,旅游,摄影爱好者
娱乐,美食
音乐,水瓶座,小说,旅游,营销
旅游
时尚
渗透测试,信息安全,互联网,Web,网络安全
美女
娱乐
游戏,车世界,上海生活,搞笑幽默,旅游
穿衣美容,摄影爱好者,爱自由,妖精,微博控,80 后,摄影控,街拍,K 歌,麦霸
文艺
IT,旅游,唱歌,微博,音乐,学生,网络,宅男,网购,大学生

多愁善感,看电影,听音乐,金牛座,好性格,睡觉睡到自然醒
时尚
游戏动漫,IT 数码
娱乐
文艺
乔丹迷,马尾控
动漫,猫,盗墓笔记,懒人
文艺
旅游
汽车,娱乐
娱乐,唱歌
看电影,动漫,幽默,看书,睡觉,火影忍者,原创,superjunior,喜欢音乐,文化
游戏动漫,星座命理,新闻资讯,时尚,娱乐
偏执综合征,摄影小角,景观设计,文艺
音乐,北约,星座命理,游戏动漫
搞笑幽默
浪漫,巨蟹座,吃货,宅女,80 后,爱情,时尚,美食,偶稀饭睡觉觉,生活
新闻趣事,旅游
美食,体育
娱乐,汽车,投资理财,美女,IT 数码
体育,文艺,美食,时尚,美女
娱乐
爱尹先生,九零後,宅,金牛座,長髮控,吃货
美女,IT 数码,汽车,游戏动漫
星座命理,旅游
体育
COS,大学生,动漫,画漫画
娱乐,游戏,语录
视频音乐,狮子座,阿狸控,exo
服装设计,完美主义,天蝎,旅游,娱乐
四川生活
武汉生活,湖北生活
旅游,文艺,健康,教育就业,星座命理
搞笑幽默
动漫,腹黑大叔心,外貌协会,德意志战车 NC 粉
游戏动漫
游戏动漫
新闻资讯
那英,娱乐
娱乐
美食,军事
时尚,娱乐,文艺,美食,旅游
旅游,时尚,美食,IT 数码,文艺,吃货,摩羯座
旅游,美食,美女,时尚,汽车

名人明星
减肥瘦身,陈信宏,云南大理,宅,五月天,85 后,发呆,爱音乐,维尼夫妇,五迷
文艺,汽车,时尚,娱乐,美食家,治愈系,泰迪熊
羽毛球,听歌,平常心,动漫,体育
够朋友,微信营销,刘一秒,徐鹤宁,杨涛鸣,陈安之,有为青年,营销鬼才,成功学
招聘,新闻资讯,IT 数码
发呆,爱幻想,旅游摄影,水瓶,梁静茹
体育,新闻资讯
旅游,可爱,90 后,搞笑,吃,好性格,睡觉
娱乐
美食,体育
金牛座
搞笑幽默
名人明星
美女,音乐发烧友
旅游,星座命理
旅游
娱乐,vae,金牛座,90 后
新闻趣事
旅游,穿衣美容,视频音乐,新闻趣事,搞笑幽默
体育,汽车,旅游,IT 数码
汽车
星座命理,旅游,美食,军事,IT 数码
看书写字听音乐,边走边停,边走边拍,温暖,微笑,友好,爱生活,别扭,古典艺术,安静
体育
时尚,星座命理
时尚,游戏动漫,IT 数码
美食
新闻资讯,教育就业,星座命理,军事,体育
娱乐,旅游,星座命理,美食,时尚,爱笑
旅游,英语
浪漫,唱歌
娱乐
美食
美女,游戏动漫,IT 数码
网购,浪漫,爱情,宅女,娱乐
时尚
娱乐
美食
汽车,传统文化爱好者,吃,听歌,电影,平常心,看书,生活,历史
新闻资讯,NBA
时尚,文艺,星座命理,旅游,美女
体育,娱乐
旅行

搞笑幽默
视频音乐,星座命理,射手座,学生,玩儿,音乐,天秤座,校园,善良,90 后
教育就业,军事,游戏动漫
游戏动漫
美食,其他生活
时尚
爱唱歌,主持,旅游
80 后
新闻资讯,投资理财,教育就业,旅游
耽美腐宅女一枚,吃货,学生
新闻趣事
美食
休闲,法律,室女座,足球,旅游,渔业,乒乓球
90 後,女裝网店
美容,格子控,ab 型,爱猫,山下智久,努力,乐天派,唐禹哲
健康,时尚,投资理财,娱乐
新闻资讯
美女,汽车,宅女,上网
上网,旅游,90 后,吃货
游戏动漫
健康,时尚
文艺,娱乐
汽车
新闻资讯,投资理财
运动,品着茶,端着书,奋青
电影,唱歌,狮子座,吃货,自由,旅行
听歌,浪漫
动漫,好性格,天秤座小面瘫,下垂嘴,睡觉
旅游,文艺,王菲,西藏西藏,猫性女人
旅游
旅游
美女,体育资讯,旅游,武汉生活
懒,星座命理,美食,娱乐,时尚,游戏动漫,美女
娱乐
篮球,交友,一些事一些情,宅男,美女,手机看小说,世博志愿者,茶
旅游,美食
广州,爱美食,幻想,健康
偶稀饭睡觉觉,好性格,上网,新闻,天秤座
美容服饰,美女,时尚,文艺,小清新,旅游,健康
微博奇葩,福建生活
NZ,睡觉,Film,TT,Game,韩国,美食,旅游
偶稀饭睡觉觉,学生一枚,宅女一枚,动画,青春,旅游,自由,处女座,向日葵,90
爱笑,爱旅游,时尚潮流
郑州生活,郑州婚车租赁,郑州礼仪公司,郑州婚礼策划,媒体,80 后,时尚

IT 数码,投资理财,军事
白羊座
娱乐
yz,宣管
健康,旅游,星座命理,美食,娱乐
文艺,旅游,星座命理,美食,时尚
济南,肇庆,活泼,单身,苹果
健康
登山,打台球,骑车
健康,教育就业,军事,时尚,投资理财,文艺
旅游,时尚,网购
文艺,游戏动漫,星座命理,美食,学生,吃货,宅,偶稀饭睡觉觉,听歌,囧
宅女一枚,偶稀饭睡觉觉
矛盾体,校园,情绪化,天蝎女,神经质,旅游美食,学生一枚,电影爱好者,90
娱乐
娱乐
情感生活,杂志,摩羯
体育,汽车,新闻资讯,IT 数码,文艺
狮子座,懒人,听音乐,美食
汽车,美女,美食,娱乐
娱乐,军事
娱乐,睡觉,90 后,篮球,唱歌,动漫,音乐,双子座
旅游,微博奇葩,新闻趣事,搞笑幽默,穿衣美容,白羊座
美食,文艺,IT 数码,教育就业,旅游
IT 数码,体育,手机,天蝎座,动漫,90 后,自由,运动,宅男,篮球
文艺,美女,新闻资讯,军事,游戏动漫,汽车
视频音乐,穿衣美容,星座命理,美食,旅游
美食爱好者,生活大爆炸,快乐大本营,设计师,电影控,麦霸,偏执狂,iphone,服饰,b 型血
美食
投资理财
搞笑幽默
美食
旅游,时尚,娱乐
健康,时尚,星座命理
爱玩,爱幻想,购物狂,爱美食
时尚,星座命理,体育,新闻资讯
微博奇葩,网络,宅男,爱音乐,学生一枚,天蝎座,真诚
不定期躁狂症,十分钟励志青年,选择性眼瞎耳聋
搞笑幽默
娱乐,白羊座,摄影爱好者,偶稀饭睡觉觉,旅游
健康,古城香业网销
个性,青春
时尚,军事,IT 数码
娱乐

美容服饰,肇庆生活
湖北生活
学生一枚,疯子,爱自由,服饰,喜欢听歌
搞笑幽默,囧,设计,吃货,双鱼座,旅行,动漫
体育,吃,射手座,80 后,宅
时尚,游戏动漫,舞蹈培训,hiphop,爵士,街舞
吴亦凡
体育,骑的王八撵大奔
娱乐,浪漫,听歌,90 后
短发妹控,眼镜娘控,爱耍小脾气,胆子特别小
孙燕姿,烹饪,迷糊,夜猫子,拍照,登山,浪漫,自然,笑,美食
美女,慎重,淄博,房地产,成都,公关,园林业,财会
游戏动漫,旅游,体育,娱乐,有点小网虫,游戏魔,拿心交朋友,用心服务,情歌王,麦霸
美女
学生,唱歌,动漫,上网,遊戲動漫
投资理财,健康,旅游,美食,文艺
旅游
教育就业,旅游,美食,军事,IT 数码
听歌,宅男,吃,好性格,双鱼座,旅游,睡觉,浪漫,乐观,电影
微博奇葩
汽车,时尚,旅游,星座命理,美食,文艺,娱乐
美剧控,孩子气,IT 数码,交友,吃货,腹黑,果粉,狂吃不胖,gay,90 后
星座命理
时尚,娱乐
娱乐
低调,时尚
感性,微博控,写字,摩羯,乐观,音乐迷,随性,流浪,自由,学生、杰迷、K 歌
时尚,娱乐,文艺
时尚,文艺,旅游,美食
时尚,摄影爱好者
娱乐
IT 数码,体育
新闻资讯
娱乐
水瓶座加 O 型血,美剧,动漫,偶稀饭睡觉觉,大学,运动,囧,偶喜欢玩
娱乐,美食
星座命理,文艺,美食,善良,浪漫,双鱼座,宅女
生活小饰品,善良,艺术,水瓶座,马口铁盒,储蓄盒,储纳盒,收纳盒,储存盒
旅游,新闻资讯,汽车,军事,美食
搞笑幽默
广东生活,吹水
文艺
搞笑幽默
文艺,美食,娱乐,星座命理

时尚
娱乐
演员
营销,听歌,幽默,巨蟹座,互联网,平常心,游戏,唱歌,旅游
三中人,荔城三中,历史,双子座,古筝女,羽毛球爱好者,90后,荔江人,增中人
心理学,绘画,耽美,死神,星座命理,健康,游戏动漫,旅游,美食
IT数码
手机看小说,时尚潮流,看书,摄影,80後,娱乐,美食,时尚,健康,旅游
媒体,吃货,狮子座,传媒
肇庆生活,美食
军事
娱乐,美女,体育,搞笑
爱情,校园,黑莓,美食,汽车,健康,旅游,星座命理,美食,娱乐
娱乐
投资理财,娱乐
汽车
搞笑幽默
美食,星座命理
投资理财,文艺,教育就业,音乐,交友,90后,阅读,大学生,狮子座
萌宠
汽车,军事,娱乐
体育
旅游,新闻资讯,教育就业,娱乐,IT数码
星座命理
娱乐
时尚
新闻资讯
体育资讯,私募基金经理
微博奇葩,搞笑幽默,美女
游戏动漫,娱乐
美食
视频音乐
投资理财
娱乐,时尚,美食
重庆,忠诚心,海口,权利,湛江,网络电子商务
江苏生活
游戏动漫
旅游,美女,汽车,健康,游戏动漫
摩羯座A型血
体育
健康,旅游,教育就业,美食,美女
IT数码,旅游
美食,新闻资讯,体育,娱乐
健康,旅游,星座命理,美食,娱乐

汽车,健康
唱歌,NBA,处女座,篮球,运动,吃,90 后
王力宏,搞笑,新闻,乐观,运动,金牛座,亲情,羽毛球,手机
乐观,自由,交友
美女,处女座,紫色
美女,旅游,IT 数码
浪漫,睡觉,交友,数码,创业,自由
军事,美食,IT 数码,汽车,旅游
汽车
电子商务,汽车,文艺,体育,搞笑幽默,星座命理,IT 数码,时尚
简单,湖人,体育
军事
投资理财
新闻资讯,教育就业,美女,军事,体育
美女
健康,汽车,旅游,美食,娱乐
山西生活
健康,教育就业,娱乐,美食,爱自由,读书,设计,双子座,旅游,文艺
娱乐
篮球
武汉生活,大四党,睡觉睡到自然醒,青春,时事,摄影控
济宁,反应佳,呼和浩特,帅哥,家具,广告,电影
金牛座,运动,摄影
互联网,腐女,电脑,服饰,瘦身,意大利
旅游
健康,美食,娱乐,时尚,投资理财,文艺
体育
生活,美食,广州美食,澳门,港式奶茶,猪扒包
游戏动漫
旅游,美食,星座命理,穿衣美容,新闻趣事
新闻资讯,文艺,健康,体育,篮球爱好者,美女,看书,设计,美食
文艺,健康,旅游
娱乐
健康,旅游,美食,文艺
星座命理,美食
旅游,上网
美食,体育,娱乐
娱乐
旅游
美剧,摄影爱好者,自由,摩羯座,平常心,校园,动漫
健康,旅游,星座命理
美食,美食爱好者,收藏,高尔夫,志愿者,驴友
恐怖片,唱 K,低调,善良,偶稀饭睡觉觉,看电影,发呆,海贼王,萌宠,旅游
健康,旅游,时尚,娱乐,睡觉,美食

旅游
娱乐
旅游
宅,90,北京生活,娱乐
新闻趣事,旅游,湖州生活
健康,设计,photoshop,psmenu
读书分享,美食,宠物图集,新闻趣事,乌鲁木齐生活
陕西生活,游戏动漫
游戏动漫
爱美食,吃货一枚,狗狗,偶稀饭睡觉觉
投资理财
体育,足球
姿多彩 1999OK,看书,浪漫,80 后,设计
军事
搞笑幽默,运动,财经,新闻趣事,好书,外汇,黄金,期货投资,股票投资
海外生活
美食,天秤座
娱乐,时尚,健康,旅游,美食,新闻,宅女,看书,自由,搞笑
游戏动漫
听歌,玩儿,亲情,善良,吃,偶稀饭睡觉觉,旅游,交友,自由
高达,CLANNAD,夏尔,韩流,Beast,SHINee
上海生活
娱乐
百合控,百万亚瑟王,一般动漫,轻小说,galgame,天凤麻雀
嘟噻秀,吴世勋,张艺兴,吴亦凡,朴灿烈,边伯贤,鹿晗,EXO
小说,交友,网络,语录,摄影爱好者,时尚
文艺,体育,旅行,文字,足球,音乐,摩羯座,历史,读书,宅女
娱乐
教育就业,旅游,美女,游戏动漫
汽车,游戏动漫,美食,IT 数码,娱乐
新闻资讯,美女,时尚
星座命理,美食,投资理财,体育,旅游
时尚,美食,旅游,健康,新闻资讯,历史,文字,电影
教育就业,汽车,健康,旅游,IT 数码
校园
体育,汽车,美食,IT 数码,文艺
时尚,新闻资讯,健康,文艺,美食
金牛座,学生,听歌,微博,搞笑
健康,旅游,星座命理,教育就业,新闻资讯,娱乐,美食,文艺,军事,体育
美女
游戏动漫,时尚,美女
起床困难户,萝莉控,老愤青
俱乐部,汽车按揭,汽车金融,汽车信贷,理财,投资,移动互联网
时尚,汽车,IT 数码

时尚,健康,文艺
游戏动漫,体育
健康,新闻资讯,旅游,美食,IT 数码,飞行
美食,时尚,健康,教育就业
美女,汽车,动漫,上网,娱乐,篮球,吃,旅行
時尚
健康,公益水晶球,公益路线图,公益图谱,效率,幽默,坚强,公益天平
90 后,偶喜欢玩,偶稀饭睡觉觉,搞笑,音乐,吃货,摄影爱好者,电影,看书,美食
娱乐
美食,娱乐
旅游
娱乐,美食,星座命理,军事,游戏动漫,金牛座
美食,娱乐
美女
穿衣美容
美女
新闻资讯,教育就业,军事,汽车,星座命理,体育
美女,娱乐
投资理财
娱乐
旅游,摄影,乐观,睡觉,90 后,上网,吃,电影,宅,听歌
美食
何澤勝,游戏动漫
游戏动漫
美食,IT 数码,体育,汽车,投资理财,文艺
投资理财
搞笑幽默
教育就业,文艺,旅游,健康,爱生活
IT 数码,旅游摄影,O 型血,旅行,平常心,亲情,白羊座,80 后,音乐
80 后天蝎座,茶,苹果控,松下相机 LX7,复古,设计是一种态度,小大人,abc,看电影,爱音乐
文艺,时尚,旅游,美食,娱乐
情感生活,巨蟹座
美女
旅行,网络,幽默,文学
育儿,互粉,欧美控,美国,软件,钟汉良
体育
服饰风尚,服饰潮流,服饰搭配,时尚
体育
视频音乐,健康
娱乐
星座命理
娱乐
IT 数码
美女

文艺,娱乐,时尚,IT 数码
美食,射手座,小说,趴趴走
水瓶座,平常心,快乐,聊天,摄影控,医生,努力,龟梨和也,张翰
娱乐,游戏动漫,美食,旅游,文艺
运动,自由职业,娱乐,财经,修仙从养心开始
星座命理,健康,娱乐
文艺
汽车
教育就业,投资理财,美食,旅游,健康
旅游,娱乐,美食,美女,体育
网购,足球,射手座,历史,电影,生活,乐观,80 后,音乐
萌宠,新闻趣事,穿衣美容,江苏生活,旅游
曲爹,小说,颜控,古风,声控,小号
90 后,大学生,旅行
时尚,健康
笑话,养生,地产,经济,管理,投资理财,新闻资讯,美女,开心,书籍
时尚,记录生活,完美主义者
新闻趣事
IT 数码,娱乐,美女,汽车
美女
美女,游戏动漫,体育
娱乐
疯,活泼,安静
不绣钢首饰
新闻资讯,游戏动漫,星座命理,IT 数码,教育就业,美食,文艺
教育就业,娱乐,文艺
美食,娱乐
汽车
娱乐
旅游,娱乐,时尚,星座命理,文艺
旅游,体育
星座命理,文艺,娱乐,暮光之城,旅行,自由,健康,亲情
黑框眼镜男控,文字,动画片,电影,旅游,宅
戏剧,旅游,投资理财,周杰伦,执着
善良,双子座,狮子座,音乐,90 后,篮球
体育
游戏动漫,旅游
健康,文艺,新闻资讯,游戏动漫,汽车
健康,音乐,平常心,水瓶座
生活
娱乐
投资理财,美食
美食
娱乐

穿衣美容
旅游,穿衣美容
健康
文艺
美图摄影,视频音乐,BenWhishaw,安妮小明一生推,静茹郭静听不厌,新男神古川雄辉,刘惜君的细菌,巨蟹座B型血
善良,90后
les,耽美,韩寒,追星,行情
美女
上海生活
娱乐,投资理财,健康,旅游,军事,体育
游戏动漫,双子座,豪放,腐女,学生
微博奇葩,新闻趣事,星座命理,旅游,重庆生活,格子控,爱交朋友,爱笑,阳光开朗
穿衣美容,吃货
感性,活泼开朗,美食,睡觉睡到自然醒,魔羯座,旅游
美女,IT数码,时尚,汽车,娱乐
时尚,游戏动漫,教育就业,文艺
娱乐
唱歌,123
时尚
IT数码,时尚,亲情,白羊座,90后,吃货
新闻趣事,名人明星
新闻资讯,健康,旅游,IT数码,文艺
时尚,美食
汽车,军事,IT数码
时尚
娱乐
淘宝购物,淘宝店,潮牌,原创潮牌
旅游,美食,星座命理
美女,娱乐,时尚
美食
美女,美食,娱乐
健康
大学
汽车,美女,投资理财
星座命理,健康
羽毛球
90后,学生,上网,双子座,听歌,吃
时尚
花样美男,双子座,郑容和
游戏动漫
文艺
教育就业
美女,搞笑幽默,宅男

名人明星,旅游
时尚,娱乐,文艺,体育,开朗
美女,体育,IT 数码
娱乐
萌宠
玩,射手,学习
体育
搞笑幽默
时尚,旅游
广西生活
游戏,校园,淡定,湖人,卡卡
美食
时尚,偶喜欢玩
游戏动漫
打鼓,白羊座,旅游,旅行,音乐,足球,体育,运动,听歌
校园生活,美食,听歌,狮子座
体育,美女,军事,新闻资讯,旅游,汽车
新闻趣事,天津生活
美食,文艺
娱乐
教育就业,文艺
时尚
体育
联众深圳,联众城市 2,1419,深圳美食,深圳人,深圳,美食
艺术,海贼王,魔兽世界,腹黑,医学
娱乐
书法,军事
金牛座,睡觉睡到自然醒,陈奕迅,吸血鬼日记,犯二青年,游戏动漫
辽宁生活
时尚,美食,旅游,星座命理,投资理财,知性,感性
心理学,职业生涯发展,求职,微招聘,职场,8090,公益
旅游
旅游
星座命理
旅游
好性格
星座命理
师范,丽江,丽江师专,文艺,美食
心理
校园,好书,亲情,文字控,唱 K,健康
新闻资讯
魔兽世界,安安静静
星座命理
偶喜欢玩,美食

文艺
军事
宅女
旅游,美食,穿衣美容
体育,教育就业,游戏动漫,军事,汽车,时尚
时尚,娱乐,萌男控,处女座
体育
美食
80后,a股,校园,85后,动漫迷,正太控,偏执狂,球迷,疯狂,留学生
美女
乐天派,小说迷,腹黑,无厘头,星座命理,水瓶座B型血,90后
健康,星座命理,美食,时尚,娱乐
汽车,时尚,体育,星座命理,娱乐
旅游,旅行,美剧,吃货,电影
动漫
体育
汽车
娱乐
时尚,健康,星座命理,美食,娱乐
摄影爱好者,电视剧,爱音乐,服装设计,ac米兰,diy,学生一族,臭美
文艺
健康,教育就业,文艺,体育,幽默,80后,美女,摄影爱好者,财经,听歌
游戏动漫,娱乐,时尚,星座命理,文艺
自由职业,手机,数码
数码,IT,生活,吃,交友
孙燕姿,娱乐,体育
天平座
美容服饰,美食,健康,时尚
娱乐,美食,矛盾体,懒,敏感,90,双重性格,爱美食,水瓶座,吃货
文艺,美食,星座命理,旅游,健康
时尚
娱乐
美食,健康,汽车,时尚,旅游
搞笑幽默
IT数码,文艺,体育,娱乐
IT数码
时尚,游戏动漫,美女,娱乐
娱乐
管理,坚强,聊天,执着,射手,网络营销,看电视,人生,网购,80後
动作片,欧美电影,欧美剧,科幻大片,国产电影,好莱坞大片,港台电影,动漫,电影电视,电影
爱旅行,喜欢音乐,完美主义,处女座,90后但我不脑残,咖啡,it互联网,自由,美食,汽车
娱乐
穿衣美容
游戏动漫,投资理财,军事

投资理财
健康,时尚,军事
搞笑幽默
美女,音乐,浪漫,运动,睡觉,旅游,美剧,旅行,美食
校园,好性格,大学生,生活,阅读,自由
美女
健康
潮宝,时尚辣妈,育儿,伪文学爱好者,宝宝,爱旅游,美食
体育,娱乐
美食
星座命理
天秤座,唱歌,学生,娱乐,动漫
新闻趣事
时尚,健康,旅游,美食,文艺
金融
娱乐
123,财经,娱乐,汽车,金牛座
权利,互粉,杭州,江门,扬州,广告,嘉兴
搞笑幽默
阅读
星座命理,娱乐,健康,旅游,美食
美食
文艺,旅游,时尚
娱乐
文艺,天秤座,囧,睡觉
旅游
时尚,游戏动漫,美食,旅游,娱乐
IT 数码
搞笑幽默
旅游,最爱 shopping,标准白羊座
美女,体育,汽车,游戏动漫
文艺
平常心,写作,搞笑
睡觉,善良,80 后,交友,运动,听歌,旅游,吃
IT 数码
宅,御姐,自由,处女座,吃货
微博奇葩
文艺,美女,游戏动漫,星座命理,军事,神经质
美食
教育就业,旅游,军事
娱乐,旅游,星座命理,摩羯座,篮球,紫色,游戏,小说,唱歌,音乐
四川生活,搞笑幽默,文艺,孤独患者,安妮宝贝
体育,汽车,军事,新闻资讯,IT 数码
牙套妹,穿衣美容,健康,兔子情结,宅宅宅女,狮子座,看书

IT 数码
星座命理,旅游,穿衣美容,名人明星,视频音乐,时尚,娱乐
游戏动漫,时尚,星座命理
创业,吃,囧,娱乐,音乐,股票,善良,乐观,时尚,旅行
美女
新闻趣事,投资理财,新闻资讯
眉山生活
娱乐,健康,旅游,美食,文艺
幻想症,八卦分子,我有病,双鱼座,娱乐,新闻资讯,旅游,星座命理,美食
美食,视频音乐,新闻趣事,穿衣美容,名人明星,生活
游戏动漫,投资理财,IT 数码
时尚,教育就业,健康,旅游,文艺
时尚,汽车,文艺
旅游,美食
娱乐
山地车爱好者,唱 k,双子座,吃货
旅游
体育
投资,绘画,心理,社会,我就是我,服饰,人生,星座控,听歌,散步
欧美控,正太控,听歌,好性格,小说,Bielieber,天蝎座,吃,时尚,学生
单眼皮
八卦,宅女,睡觉,娱乐,好性格,自由
汽车
娱乐,汽车
自觉,江门,忠诚心,市场,盐城,牧业
听歌,游戏
海贼王,美剧
时尚
美女,投资理财
健康,旅游,美食,美女,娱乐
旅游,军事,文艺,体育,娱乐
名人明星,新闻资讯,IT 数码,汽车,睡觉,白日梦,爱好女,爱 RMB,喜欢车,90 后
投资理财
海贼王,睡懒觉,射手座,桌球,动漫迷,爱玩,执着,感性,服饰,喜欢听歌
美食,体育,旅游,健康,游戏动漫,文艺
娱乐
美食
美食,娱乐,投资理财,时尚,健康,旅游
旅游,婚纱控,生活,手机,天平座,看书,时尚
运动,紫色,理财,家居,成都,乐橙
时尚,旅游,美食,娱乐
介
体育
深圳生活

汽车
美食,90 后,历史,美剧
在路上,美食,相濡以沫,天蝎金牛,睡觉,旅游,80 后
舞动人生,静心品诗,走遍万水千山
投资理财,美食,星座命理,时尚
每天都在祈祷
健康,体育,娱乐
爱生活,韩庚,瑜伽,影视,电影爱好者,音乐迷,高三,巴西
新闻资讯,教育就业
音乐,上网,80 后
欧美电影,向往美好,爱情,亲情,行走,美食,书,自由
美食,旅游爱好者,双子座,偶稀饭睡觉觉,学生,笑,爱自由,吃货
军事,美食,美女,汽车,时尚
美女,时尚,旅游,娱乐,美食
旅游,双鱼女
微博,互联网,幽默,摄影
旅游
美食
健康,自由
教育就业
文艺,教育就业,星座命理,美食,娱乐
便是晴天,行到水穷处,坐看云起时
星座命理
娱乐,摄影,学生,旅游,看书,时尚,狮子座
福建生活,管理者,哲学,心理学,厚黑学
古川雄辉,减肥,海贼王,游戏动漫,娱乐
新闻资讯,美女,娱乐,体育
穿衣美容
新闻资讯
时尚,教育就业,健康,美食,IT 数码
旅游,星座命理
教育就业
自由,摄影爱好者,听歌,读书,音乐,偶稀饭睡觉觉,旅行
时尚,娱乐,美食,旅游
室女座,公关,农业,自觉,保护他人,房地产
娱乐
80 后的尾巴
宅女,数码,娱乐,小说,语录,唱歌
文艺
教育就业,旅游,美食,娱乐
朴有天,美少女的谎言,林宥嘉,angelababy,柯震东
旅游,穿衣美容,美食
健康,投资理财,新闻资讯
时尚,美食,星座命理,旅游,娱乐

时尚,宅
演员,互联网,高手,浪漫
新闻资讯,孩子气,证券,公关,爱电影,IT 互联网
广州,思考,执着,休闲,发展力,家具,餐饮
小说,睡觉,大学生,听歌
星座命理,娱乐
时尚,文艺,健康,旅游,美食,娱乐
旅游,健康,星座命理,文艺
投资理财,健康,旅游,娱乐,IT 数码
娱乐
美食,摩羯座,旅游,自由,交友,90 后,羽毛球,乐观,大学生,创业
文艺
爱生活,减肥,听音乐,白羊,美容护肤,猫咪控,美食,IT 数码
旅游
新闻趣事,佛山生活,搞笑幽默,穿衣美容,星座命理
体育
求交往
娱乐
搞笑幽默
萌宠,名人明星,搞笑幽默
八卦,摩羯座,偶稀饭睡觉觉,偶喜欢玩,小说,爱情,吃货,电影,娱乐,阅读
体育
教育就业,美女,健康,旅游,美食
美食,IT 数码
旅游
旅游
服饰,潮人,喜欢听歌
乐呵,得瑟,听歌,学生
园林业,毅力,巨蟹座,恋爱,义乌,主观
江苏生活,投资理财
旅游
体育
娱乐
旅游,汽车,汽车达人,吃货,桌球,80hou 小单身,赛车,美食,无聊,爱车一族
爱玩,咖啡,旅游美食,简单,音乐控,爬山,个性,美食,旅游,文艺
旅游
新闻资讯,时尚,旅游,美食,娱乐
足球,计算机软件,制药,热情,互粉,西安,湛江
穿衣美容
美女
微博奇葩
健康
娱乐
健康,旅游,星座命理,美食,娱乐

时尚,健康,旅游,美食
娱乐
美女,体育,IT 数码,汽车,生活,80 后,自由
Max,Xiah,Micky,Hero,UKnow,郑允浩是我骄傲,东方神起最高
星座命理
美女,旅游,IT 数码,体育
睡懒觉,八卦,学生,搞笑,巨蟹座,听歌,微博
文艺
星座命理,努力,魔羯座,音乐迷,k 歌,90 后非脑残
视频音乐,新闻资讯,好想独立,新闻
新闻资讯,IT 数码
星座命理,时尚,健康,美食,娱乐
健康,微笑,天蝎座,电影,听歌,时事,学习
旅游,宅,水瓶座,游戏人生
游戏动漫,旅游,IT 数码
新闻资讯,美女,美食,娱乐
星座命理,旅游,美食,搞笑幽默
游戏动漫,旅游,IT 数码
军事,八卦,电影,动漫
美食
军事,偶喜欢玩
张根硕,鲁敏宇,电影控
宅女,宅男,睡觉,吃货,幽默,巨蟹座,80 后,摩羯座,电影,平常心
穿衣美容
睡觉,吃货,90 后,狮子座,大学生
穿衣美容,时尚
娱乐
汽车,美女,健康
美食,军事
游戏动漫
新闻趣事,旅游,搞笑幽默,萌宠,财经,八卦
教育就业,星座命理,文艺
Barbara,90 后
游戏动漫,军事,娱乐
教育就业
自由,新闻,摩羯座,生活,80 后
时尚,旅游,娱乐,体育,文艺
LuHan
娱乐
电影,紫色,网络,爱美食,喜欢音乐,音乐控,话剧,神经质,我就是我,名人明星
旅游,偶喜欢玩,美食,偶稀饭睡觉觉,吃货,狮子座,羽毛球,手机,网购,唱歌
K 歌,音乐,90 后,完美主义
汽车,娱乐
星座命理,娱乐

体育
囧,80后的尾巴,林志颖忠实粉丝,白羊女一枚
现场翻译,采访,路演,谈判翻译,会议翻译,培训翻译,项目翻译,交传,口译,英语翻译
北京生活,教育,军事,文艺,上网,双子座
体育,游戏动漫,军事,汽车
体育
文艺
汽车
文艺,汽车,军事
汽车,娱乐,军事,IT数码
美食
旅游,娱乐,文艺,美食,教育就业,游戏动漫
湘潭生活
娱乐,天蝎座,生活,网购
旅游
投资理财
时尚,娱乐
娱乐
时尚
深圳生活,呆
文艺,游戏动漫
体育
游戏动漫
视频音乐,美食,娱乐,白羊座,音乐,吃货,90后
娱乐
军事,体育,文艺,IT数码,投资理财
美女
美食,游戏动漫,旅游
微博奇葩
娱乐
新闻资讯,文艺,旅游,美女
动漫,性感
运动,篮球,新闻,体育,上网,时尚,语录,羽毛球,搞笑,NBA
健康,旅游,游戏动漫,体育,军事,低调,历史
旅游
名人明星
体育,交朋友。
娱乐
星座命理,娱乐,军事,IT数码,体育,健康,汽车,新闻资讯,教育就业,投资理财
文艺,NINO,嵐,岚,ARASHI
美食
娱乐
les,鳗鱼
星座命理

健康
投资理财,汽车,旅游,军事,体育,变异的摩羯座,吃货,睡觉,运动
文艺,投资理财,新闻资讯,旅游,星座命理,美食
游戏动漫,时尚,IT 数码,娱乐,星座命理
旅游,体育,学生,A 股,金牛座,篮球
睡觉,篮球,自由,校园,处女座
健康,美食,游戏动漫,军事,新闻资讯,美女,汽车,美食,娱乐
浪漫,交友,寂寞的冷拳,火热的心
chess,fics,音乐,日语,懒,真遥本命
娱乐,运动,护士,射手座,美食
娱乐,新闻资讯,教育就业,IT 数码
方大同,摩羯座,厦门,高三,苦逼文科生
健康,星座命理,体育,娱乐,美女
视频音乐,美食,广东生活,星座命理,搞笑幽默
动漫迷,上网
名人明星,微博奇葩,旅游,美食,旅游美食爱好者,欧美,喜欢唱歌,自拍,海绵宝宝
游戏动漫,文艺,娱乐,教育就业,美食,体育
时尚,汽车,娱乐,美食
旅游,编织爱好者,淘宝,钩针,编织
喜欢旅游,白羊座,偶喜欢玩,90 后
小文艺,小二逼,宅女一枚,看书
猫咪,健康,时尚,星座命理,IT 数码,90 后
体育
吃货,玩儿,乐观,音乐,上网,90 后,天蝎座
爱生活,电影,听歌,唱 K,旅游
演员,名模
建材,足球,合肥,苹果,执着,运动,慎重
旅游
娱乐,IT 数码,美食,旅游,游戏动漫
教育就业,旅游,健康,文艺
IT 数码,文艺,体育,星座命理,美女,军事,娱乐
美女,吃货,八卦,处女座
娱乐,罗志祥
新闻资讯,美女,汽车
娱乐
军事
游戏动漫,娱乐,体育,星座命理
文艺,汽车,体育
旅游,健康,时尚
电影,娱乐,运动
汽車
游戏动漫,星座命理,军事,新闻资讯,汽车,教育就业
美食
宅,瓶邪本命,人格分裂

吃货,上网,好喜欢旅游,美食
乐观,天蝎座,清远,主观,爱情,足球,哈尔滨,律师
美食,教育就业,文艺,健康,美食,新闻资讯
游戏动漫,IT 数码,体育,魔兽世界,天秤座,看书,读书,80 后,音乐,电影
IT 数码
游戏动漫,宅,双鱼座
动漫,搞笑,金牛座,偶稀饭睡觉觉,旅行,幽默,宅
新闻资讯,教育就业,娱乐,健康,IT 数码
星座命理,旅游
文艺,娱乐
IT 数码,星座命理,娱乐,军事,汽车,体育
娱乐,美食
MartinFreeman,Benedict,腐腐腐腐腐女子
教育就业,健康
体育,美女,投资理财,旅游,美食
同性恋,天蝎座
投资理财
爱马,娜梓纯杰的爱恋,火影忍者,快乐家族,亲情,巨蟹,吃,爱音乐
旅游,狗狗,爱旅行,设计,创意,爱生活
爱音乐,听音乐,看电影,疯狂,赚钱,音乐,文艺,乐观,小说,唱歌
法律,高考,炎
爱狗一族,牛奶妹,吃货,旅游,天秤座
娱乐
美食,吃货,看电视,低调,宅,90 后,旅游,睡觉,唱歌,天秤座
教育就业,文艺,时尚,旅游,美食
搞笑幽默
美食,娱乐
美食
健康
新闻趣事,广西生活
教育就业
旅游
星座命理,电影
时尚,娱乐,旅游,星座命理,美食
教育就业,时尚,健康,美食,文艺,投资理财,星座命理,IT 数码
新闻趣事,穿衣美容,南京生活,娱乐,投资理财,健康,旅游,美食
时尚,游戏动漫
文艺
时尚,美食
美容服饰
上网,快乐大本营,暮光之城,吃货,古川雄辉,90 後,小说迷,处女座,宅女,互粉
娱乐
汽车,娱乐,文艺,军事,拿破仑,爱国者,网络,90 后
游戏动漫

搞笑幽默
新闻资讯,教育就业,军事,健康,游戏动漫,文艺,投资理财,美食,IT 数码
娱乐
健康
娱乐
开心吃,不开心看情况吃,上网,旅行,听歌,水瓶座
搞笑幽默
篮球,足球,排球
粉红控,神经大条,科比,懒,医生,看电视,奋斗,音乐控,偶喜欢玩,任性
旅游,美食,游戏动漫
明星,造梦者,李炜,唱歌,娱乐,北京,校园,听歌,90 后,好性格
旅游
美容服饰,美食,平常心,时尚,听歌,电影,口腔正畸,金牛座,80 后
名人明星
体育
美食,好书,玩儿,宅男,手机,多愁善感,小清新,幽默,阳光,处女座
娱乐
娱乐,文艺,军事,旅游,游戏动漫
娱乐
自由,时尚,美食,A 股,新闻,手机,文字,传媒,语录,博客
游戏动漫,萌妹纸,张国荣,科比
教育就业,旅游,军事,汽车,美食,娱乐
新闻资讯,汽车,游戏动漫,旅游,军事
健康,美女,旅游,IT 数码
汽车,美食,文艺
文艺
星座命理,游戏动漫,文艺,美女,教育就业,投资理财,IT 数码,健康,汽车,旅游
美食,健康
旅游,美食
新闻资讯
时尚,音乐,汽车,阅读,偶喜欢玩,财经,紫色,自由职业,媒体,语录
美女
自然,b 型血,单身,旅游美食爱好者,我就是我,工作,乐天派,睡懒觉,奋斗,music
自由,美剧,创业,金牛座,摄影爱好者,浪漫,财经,囧,体育,亲情
娱乐,时尚,健康
疯狂,汽车
健康,美女,文艺,体育,娱乐
搞笑幽默,新闻趣事,视频音乐,星座命理,微博奇葩,运动,体育,搞笑,吃货,天秤座
美女,星座命理,枫若泪,泪飘零
娱乐,新闻资讯,健康,旅游,美食
旅游
计算机
90 後,梦想,哲学,校园,大学,流浪,巴萨
名人明星,美食,星座命理,睡觉,唱歌,吃货

美女,时尚,教育就业,IT 数码,娱乐
幽默,直率,歌神
八卦杂谈,美女,视频音乐,双鱼座,摄影,上网,宅,自由,小说,90 后
体育,美食,星座命理,新闻资讯,文艺,娱乐
旅游,美食,体育,军事,投资理财,美女
教育就业,军事,文艺
新闻资讯,投资理财,美女,旅游,IT 数码
les
美女
搞笑幽默,旅游,美食,微博奇葩,萌宠
娱乐,汽车,投资理财
孩子气,夜猫子,爱音乐,90 後,娱乐
娱乐
旅游
汽车
时尚,敏感,文学,网络,情绪化,温柔,完美主义,天蝎座,时尚潮流,爱音乐
无锡生活,户外,古典音乐,交友,双重性格,傻傻的善良,孩子气,多愁善感
交友,摄像,卖茶,互粉,文艺,养生,旅游,时尚,品茶
教育就业,投资理财,体育
旅游,微博,运动,游戏
娱乐
体育,苦逼住院总
教育就业,健康,旅游,美食,时尚,娱乐,唱歌,吃货
北京生活,新闻趣事,名人明星
时尚,汽车,健康,旅游,文艺
个性,奋斗,学习,暮光之城,娱乐,新闻资讯,IT 数码
星座命理,北京地下说唱,MC,HipHop,狮子座,足球,90 后
体育
教育就业,健康
天秤座,英语,偶稀饭睡觉觉,乐观
健康,娱乐
收集控,音乐疯子
情绪化,电影深入调查者,顺其自然,独立音乐控,轻微强迫症,恋书癖,宅
韩国生活
娱乐
健康,美食,囧,自由职业,吃,摄影,娱乐,创业
旅游
IT 数码,娱乐,时尚,健康,摄影,美剧,好书,英语,乐观,新闻
军事,教育就业,IT 数码
时尚
娱乐
美食,双子,睡觉,大意,偶喜欢玩
旅游
外貌协会,音乐迷,书籍,时尚,健康,旅游,文艺,娱乐

娱乐
新闻资讯,旅游,军事,汽车,体育
游戏动漫,娱乐
音樂,文學,体育
娱乐
IT 数码,美食,健康,新闻资讯,游戏动漫
健康,娱乐,旅游,美食,时尚
文艺
健康,投资理财,美女,星座命理,IT 数码
娱乐
爱狗狗,完美主义,狮子座,旅游,爱自由,桌球
分享,校园俱乐部,高考,知识先锋,百科
美食
体育
娱乐,旅游,星座命理,文艺,时尚,美女
娱乐,狮子座,90 后,平常心,音乐,宅女,吃
旅游
娱乐
娱乐,IT 数码,喜欢安静的生活,爱 K 歌,双鱼座
娱乐
80 后,听歌,美剧,八卦,网络,电子商务,IT,营销,玩儿,媒体
星座命理,五月天,盗墓笔记,海贼王,处女座,篮球
旅游
旅游,美食
旅游
时尚
农业,宅男,园林业,重庆,顺德,双鱼座,可爱
娱乐
IT 数码,娱乐,体育,新闻资讯,时尚
宅女,搞笑,90 后,网络,自由,玩儿,校园,美食,美容
IT 数码,八卦杂谈,美女,新闻趣事,唐山生活
汽车,体育,IT 数码,文艺,娱乐
名人明星,穿衣美容,微博奇葩,美食,旅游
新闻资讯,娱乐,军事,体育,教育就业
美食
喜欢帅哥,自由,安静,偶喜欢玩,90 后,电影,听歌
健康,旅游,美食,文艺,娱乐
体育
健康,星座命理,军事,文艺,美女,教育就业,IT 数码
游戏动漫,文艺,IT 数码
游戏动漫
爱幻想,动漫迷,宅女,大爱鹿晗,k 歌,神经大条
旅游,娱乐
旅游,美食,搞笑幽默,腐女,腐宅集中营

火影忍者
美女
娱乐,时尚,旅游,星座命理,美食
上海生活
徐州生活
天秤座
娱乐,星座命理,健康
美女,搞笑幽默
IT 数码,健康,网购,白羊座,小说,音乐,吃货,旅行,看书,90 后
白羊座,法律,江门,发展力,咨询,烟台,公关,篮球
娱乐
美食,广东生活,因为爱情
画画,腐,动漫,平常心,游戏,数码,宅,学生,音乐,搞笑
体育
投资理财,娱乐,游戏动漫,时尚,旅游,新闻资讯,美女,健康,星座命理,体育
时尚,星座命理,美食,IT 数码,文艺,体育
财务会计
自由,网络,自由职业
健康,NBA,体育
旅游,射手座
游戏动漫,旅游,体育
投资理财,理财,O 型血
音乐
星座命理,美食,军事,IT 数码
您的朋友,学习交流,影楼成长顾问,培训顾问,步步为赢,婚纱摄影,影楼最新资讯,网络营销课程,团队管理,总裁模拟舱
教育就业,娱乐,美食,旅游,游戏动漫
娱乐,90 后
游戏动漫,投资理财,军事
娱乐,美女,文艺
乐观,运动,幽默,吃,蓝色,白羊,彩妆,冷笑话,吃货一枚,星座命理
IT 数码
喜欢听歌,体育,IT 数码,旅游,健康,娱乐
搞笑
IT 数码,看小说,高中生,宅。
爱美食,学习,育儿
军事,新闻资讯,美女,娱乐
浙江生活,无敌靠谱妞,小身材大味道,Friends 迷,爱生活,爱植物,爱动物
娱乐
体育
美食
学生
美食,娱乐,游戏动漫,旅游
美食

投资理财
时尚,街拍,代购,买手,vintage,复古
运动,90 后,宅男,上网,自由,宅
游戏动漫,银他妈,SHINee
美食
执着,fashion,90 後,烹饪,寂寞,睡觉睡到自然醒,时尚,听歌,gay,单纯
娱乐
教育就业,时尚,投资理财,美食,旅游
埃及生活
搞笑幽默,星座命理
美食,旅游,健康,投资理财,时尚
新闻趣事,美食,微博奇葩,搞笑幽默
文艺,旅游
娱乐,无聊,随性,感性,多愁善感,海贼王,发呆,听音乐,动漫,90 后
旅游,娱乐,星座命理
时尚
时尚,健康,美女,喜欢听歌
搞笑幽默
喜欢唱歌
旅游,乒乓球,苹果,可爱,制药,天秤座
k 歌,爱电影,爱旅游,星座命理,睡觉,自由,狮子座,八卦,美食,电影
湖北生活,新闻趣事,微博奇葩,旅游,NBA,好性格,交友
名人明星,新闻趣事,美女,搞笑幽默,辽宁生活
时尚,投资理财,教育就业,新闻资讯,文艺
游戏动漫,美食
教育就业
文藝
神经质,喜欢听歌,摩羯女,跳舞
美女,汽车,新闻资讯,教育就业,军事
大同美女,大同美食,大同摄影,大同时尚,大同城市建设,大同潮流资讯,大同夜生活,云冈石窟,山西大同,大同
旅游
室内设计
文艺,体育,娱乐
美食
娱乐
读书分享,视频音乐,校园生活,厦门生活
美食
自尊心,主观,园林业,慎重,成都
新闻资讯,文艺
穿衣美容,睡觉,水瓶座
旅游,健康,时尚,美食,文艺
文艺
旅游

穿衣美容
生活,85 后,IT,微博控,移动互联网,网络,android,品牌,shopping,资讯
体育,美食,低调,朱古力,平头男,听音乐,有木有
时尚,旅游,健康,星座命理,娱乐

EXOK,Sehun,오세훈,吴世勋,Baekhyun,변백현,边伯贤,卞白贤

美食,汽车,游戏动漫,体育,娱乐
天秤
台球
娱乐
娱乐,星座命理
广东生活
军事天地,美女
穿衣美容
微博奇葩,双鱼女,学生，动漫，美食
汽车,时尚
细心,宜昌,水利,江门,武汉,合肥
旅游
旅游,美食
体育,娱乐,投资理财
旅游,自由职业
豆豆跟妈妈混,妈妈给你买糖吃
美食
投资理财,旅游,教育就业,娱乐,美食,蒂娜吉塔勒拉,周杰伦
时尚
体育
旅游
搞笑
汽车
篮球,创业
时尚,健康,美食,体育
美食
体育
运动,看书,乐观,摄影,射手座,旅行,美食
IT 数码
生活,美食,旅游,唱歌,浪漫,小说,水瓶座,自由,80 后,足球
90 后但我不脑残,美食,旅游,向日葵,欧美控
娱乐,健康,90 后,平常心,金牛座,自由,学生,乐观
人力资源,天蝎,自由,学生,旅行,90 后,厦门,猫咪,时尚
健康
旅游,健康,投资理财
游戏动漫,美食,星座命理
娱乐
健康,教育就业,正能量,健康生活,未来成长,亲子,教育,男宝宝,母婴,育儿

狮子座,宅男,紫色,音乐,幽默,大学生,财经,英语,美剧,睡觉
美食控,军事,单身,户外,打篮球,室内设计,自拍
IT 数码,游戏动漫,娱乐,教育就业,体育
体育
手机,IT 数码,时尚,文艺,教育就业
星座命理,娱乐,小说,上网,耽美
美食,健康,娱乐,旅游
平常心
单纯,追求完美,幽默,善良,坚韧,摩羯座
旅游,美食,时尚
搞笑,睡觉,上网,偶喜欢玩
时尚
宠物图集
时尚,健康,新闻资讯,善良,游泳爱好者,处女座,完美主义者,萝莉以上,御姐未满,85 后
宠物,韩剧,90 後,帅哥,逛街,臭美,宅女加腐女,天蝎女,hiphop,时尚潮流
厦门生活,星座命理
投资理财,文艺,教育就业
时尚,新闻,读书,宅男,80 后
美食
旅游,偶稀饭睡觉觉,天蝎座,唱歌,90 后,小说,音乐,搞笑
旅游
自由,双鱼座
新闻资讯
汽车,文艺,娱乐,星座命理,游戏动漫
名人明星,美食,搞笑幽默,江苏生活,文艺
美食
旅行,大学生,自由,偶稀饭睡觉觉,处女座,音乐,文字,美食
汕头生活,90 后,交友
美女,体育
美食
发呆,五月天,无厘头,偶稀饭睡觉觉,平常心,游戏动漫,美食,听歌,水瓶座
欧美控,麦霸,活泼开朗,水瓶女,善良,吃,旅游
视频音乐
动画,双鱼女,爱吃,浪漫,蓝色,旅游,羽毛球控
广东生活
星座命理,投资理财
视频音乐,穿衣美容,名人明星,旅游,美食
娱乐
IT 数码
娱乐
文艺
小说迷,军事,游戏动漫
旅游,文艺,娱乐
汽车

美女,体育,汽车,文艺,黑莓,游戏,吃,运动,校园
汽车,娱乐,投资理财
其他生活
NBA,羽毛球,天秤座,乐观,亲情,旅游,幽默,看书,电影,音乐
浪漫,学生一个,中意奶茶,双鱼座,动漫
游泳,喜欢音乐,旅游,美食,娱乐,健康,时尚
汽车,新闻资讯,美女,时尚,IT 数码
旅游,星座命理,美食
健康
军事,新闻资讯,健康,投资理财,旅游,美食
好性格,我就是我,喜欢听歌,shopping,旅游美食,爱自由,钢琴,爱电影
旅游
装饰材料,家居建材
科技,文艺,教育就业,健康,军事,历史,电影,90 后,摩羯座,摄影
摄影师,小清新,日系,写真,婚纱摄影,旅游,文艺,时尚
娱乐
视频音乐
美女
手机,艺术,时尚,美食,爱情
偶稀饭睡觉觉,吃货
搞笑幽默,新闻趣事,IT 数码
星座命理,狮子座,乐天派
IT 数码
囧,看书,偶稀饭睡觉觉,数码,动漫
娱乐,新闻资讯,旅游,星座命理,美食
美食
游戏动漫
娱乐
搞笑幽默
餐饮,股票,乐观,烟台,电影,想象力,热情
健康,新闻资讯,美女,时尚
美食
搞笑幽默
建材,保护他人,呼和浩特,杭州,牺牲奉献,兜风
游戏动漫
自行车,旅游,交友,面膜,美食,美肤,摄影,美容服饰
旅游,教育就业,体育
旅游美食爱好者,书法爱好者
教育就业,健康
棋手,三国杀迷,程序猿,80 后,天秤座,吃货
新闻趣事,泉州生活
美食,星座命理,健康,娱乐,时尚
健康,美食,文艺,娱乐
随性,健康,旅游

IT 数码
幽默,乐观,搞笑,90 后,天蝎座
IT 互联网,电脑爱好者,电脑游戏,戴尔,市场促销,合作授权,IT,促销优惠,电脑,售后
文艺,美食,投资理财,体育,IT 数码
微博奇葩,美食
娱乐
体育
忘记烦恼
狗狗,仙后,个股
爱电影爱美剧,爱护肤,爱舞蹈,爱音乐
健康,旅游,美食,体育
娱乐
安静,基督徒,ac 米兰,80 后宅女,微笑,干物女
健康
搞笑幽默
时尚,美食,健康
平常心,博客,方大同,胡歌,梁静茹,庚澈,小资
星座命理,健康,旅游,美食,娱乐
旅游,星座命理,健康,美食,时尚,娱乐
IT 数码
娱乐
学生一枚,90,女人,小清新,爱音乐,逛街,看电影,文字控,彩妆,摩羯
时尚,投资理财,娱乐,健康
听歌,巨蟹座,IT,阅读,设计,吃,旅游
星座命理
旅游,IT 数码
摄影,婚礼摄影
爱旅行
投资理财,时尚,新闻资讯,健康,体育
娱乐
IT 数码,娱乐,汽车,旅游
90 后,游戏动漫,小说,宅男,旅游,新闻趣事,搞笑幽默,杭州生活,懒,单身
美女,儒道佛,哲学,读书
美女
天蝎座
时尚,汽车,健康,美食,娱乐
游戏动漫
旅游,学生
旅游,上网,听歌,音乐
体育
爱篮球的 90 后,好性格,囧,听歌,大学生,篮球,唱歌
体育,幽默,写作,八卦,吃货,动漫,旅行,旅游
时尚
汽车,时尚,文艺,美食,军事,文字控,Music,重口味

室内设计,电影,家具,西安,济宁,狮子座,柏拉图式的爱
娱乐
搞笑,手机
娱乐,星座命理,旅游,美女,时尚
时尚,娱乐,新闻资讯,IT 数码,体育
娱乐,星座命理
星座命理
投资理财,健康,时尚,教育就业,娱乐
美食,娱乐,游戏动漫
唯美的,宝瓶座,热情,广州,建筑,淄博,苹果
动漫,娱乐,文艺
时尚,美食
汽车
汽车,IT 数码,旅游,新闻资讯,健康,投资理财,美食
星座运势,健康,娱乐,时尚,吃货
穿衣美容,美食,旅游,间歇性整理控,绿色植物,夜景,摄影,紫色,看书,摩羯座
星座命理,穿衣美容,名人明星,北京生活
视频音乐
娱乐
时尚,健康,汽车,旅游,IT 数码
军事,美食,IT 数码,文艺,体育
斑泉,火影,动漫,二次元,渣,家教,银魂,宅,白羊座
美图摄影,美女,视频音乐,搞笑幽默,汕尾生活,听音乐,文字,乐观
音乐,网购,80 后,电影
文艺,美食,投资理财,游戏动漫
郑允浩,睡懒觉,葡萄牙
摄影爱好者,美剧,旅游,听歌
Music,O 型血,唱 K,教育就业,美食,旅游,健康,时尚
体育
娱乐
萌宠
游泳,天蝎,冷笑话,咖啡,时尚潮流,销售,热情,90 后非脑残,萝莉控,西安
娱乐,天蝎座,八卦,网购,玩儿,动漫,宅,90 后,美食,旅游
体育
旅游
娱乐
健康
游戏动漫,玩儿,天蝎座,吃货,宅,写作,动漫
游戏动漫,美女,IT 数码
美女,双鱼座
时尚
娱乐,爱可倾城亦可毁,冰天雪地,谁是,连接服务器失败
安逸,轻文艺,休闲,电台,杂志
阿根廷生活

体育,军事,教育就业,IT 数码
美女,汽车,IT 数码,文艺,体育
文艺,军事,新闻资讯,教育就业,投资理财
体育
天平座,矛盾,情绪化,独立,瘦身,美食控,电影迷,感性,旅游美食爱好者,小说迷
囧,天秤座,听歌
视频音乐,星座命理,美女,美食,旅游,娱乐
双重性格,心情
宠物,大学,互粉,科比,旅游摄影,绘画,街拍,旅游美食爱好者,欧美音乐,文艺
军事,旅游,美食,IT 数码
搞笑幽默
汽车,旅游,美食,娱乐,体育
美食,娱乐,文艺,时尚,IT 数码
旅游,时尚,健康,星座命理,文艺
穿衣美容
美食
美容服饰,旅游,美食,视频音乐
要减肥,感情丰富,爱尼坤,追韩,爱幻想,阅读,90 后,听歌
微博奇葩
IT 数码,美食,时尚,娱乐,张根硕
军事,星座命理,文艺
美食,紫色,80 后
视频音乐
娱乐,文艺,美女,体育,教育就业
新闻趣事,旅游,美女,搞笑幽默,上海生活
本人开网店,QQ1041615343
文艺,体育,娱乐,IT 数码
好书
美食,…
时尚,写作,文字控,绝对 KRIS,微博
额,天蝎座,无聊
诗经唐诗宋词丶,甄嬛传丶,宜喜宜忧丶,宜骄宜纵丶,宜谦宜卑丶,秦时明月丶,汉服丶,无虐
不欢丶,死心眼丶,兔子控丶
美食
时尚,旅游,军事,文艺,娱乐
文艺,美食
新闻资讯,娱乐,时尚
美食,投资理财
搞笑幽默
唯美,个性,婚礼策划
娱乐
美食,娱乐,白羊座,英语,偶稀饭睡觉觉,八卦,睡觉,网购,搞笑,乐观
文艺
汽车,音乐,好书

穿衣美容,装修美图,家居装饰,装修平台,室内设计,80 后创业,装修加盟,装修公司,装修设计,装修顾问
旅游
娱乐
时尚,旅游,星座命理,美食,文艺
娱乐,电影,办团队旅游 L 签
时尚,射手座,80 后,旅行,网购,生活,八卦,娱乐,交友,唱歌
台球用品及配件,奥秘台球杆,飞利台球杆,互粉互注
搞笑幽默
娱乐
健康,美食,睡觉,摄影,设计,旅行,阅读,处女座,80 后,电影
慈善,农业,卫生服务,家具
名人明星,死忠粉,骨灰粉,杰迷,Jay,宅,腐女
吃货,娱乐,处女座
营销,天蝎座
体育
懒人,搞怪,游戏动漫,吃货,游戏,动漫,学生
体育,投资理财,必须保本
军事
乐观,玩儿,吃,交友,亲情,狮子座,宅女,校园
IT 数码
娱乐,美食,星座命理,美女,游戏动漫
旅游,爱自由,爱生活,爱寂寞,潜意识
名人明星
旅游,美食,娱乐
健康,娱乐,星座命理,投资理财,时尚,搞笑,运动,旅游,听歌,电影
娱乐
文艺
香港草莓家,美食
娱乐,狮子座
体育
耽美,爱猫,腐女,宅女加腐女,睡觉,动漫
广东生活
个人写真,古装摄影,旗袍摄影,设计,电影,摄影,艺术
仙后 Cassiopeia,ELF13,动感 101,凯慧中学,韓流,处女座
美食,星座命理,娱乐
90 后
美女
新闻资讯,交友,音乐
娱乐
体育资讯
桶装水零售,桶装矿泉水批发,淮口全兴水店
投资理财,旅游
听歌,淡定,爱自由,我就是我,健康,处女座,平常心,睡觉

贵州生活
旅游,军事,文艺,教育就业,星座命理,美食,IT 数码,体育
时尚,摄影,傻,DIY,陈奕迅,狮子女,潮,室内设计专业,欧美控,火影
美女,新闻,文字,语录,读书,电影,交友,唱歌,上网,听歌
苹果,运动,篮球,盐城,江阴,房地产,美女,嘉兴
旅游,投资理财,美食
白羊座,帅哥,艺术,律师,乐观,扬州
八卦杂谈
搞笑幽默,萌宠,美食,星座命理,视频音乐,健康,新闻资讯,时尚
我爱张杰,旅游,学生,睡觉,平常心,巨蟹座,幽默,90 后,乐观,宅女
汽车,体育
投资理财
美食
娱乐
IT 数码
游戏动漫
时尚
体育,旅游,自由,双子座
娛樂
美食
IT 数码,汽车,娱乐,体育,自由
电影,生活
允在,男同,动漫,游戏动漫
较懒,饭团,最近柯震东,欢喜韩娱,红薯夫妇,洪钟贤不错看,男神李钟硕,理想型金钟云,本命徐贤,SONE
娱乐
新闻,南宁,艺术,汽车,交友,旅游,摄影,美食,时尚,夜店
吃货,宅女,天蝎座,奋斗,孩子气,微博,八卦,睡懒觉,爱玩,电脑
娱乐,天秤座
体育,运动,美食
旅游
上海生活,汽车,吃货,音乐
计算流体动力学,CFD,制冷仿真,暖通空调
上海生活,美食,旅游,美女
游戏动漫,星座命理
娱乐,旅游
美食,儿童摄影
时尚
文艺
美食
动漫,游戏,吃货,狮子座,宅,听歌,小说,学生,睡觉
处女座,宅
时尚
娱乐

汽车,美食
单身,旅游,汽车,娱乐
睡觉
文艺,时尚
汽车,火影忍者,欧美控,睡觉,宅,水瓶座,爱吃,唱 k
游戏动漫
美食
娱乐
合肥生活
爱美食,时尚,时装,爱音乐
体育
名人明星
娱乐
健康
旅游
生活
IT 数码,视频音乐,搞笑幽默,校园生活,音乐,宅,上网,搞笑,IT,90 后
电影迷,宅女一枚,旅游
旅游
好性格,射手座,大学生
美食
新闻资讯,健康,时尚,星座命理
偶稀饭睡觉觉,网购,80 后,电影,吃
电影,营销
娱乐
阳光,奋斗 ing,闷骚,吃货,80 后
时尚,音乐,金牛座,电影,设计
IT 数码,体育
时尚,旅游,星座命理,美食,文艺
时尚,文艺,旅游
水瓶座,苗兄
娱乐,乐观,生活,时尚,摄影,爱情,大学,英语,浪漫,亲情
旅游,美食,娱乐
玩儿,听歌,电影,文学,摄影爱好者,宅,旅游,数码,动漫,生活
DN 推推 05,DN 推推
健康
美食,旅游
投资理财,旅游,星座命理,时尚,娱乐
旅游,星座命理,美食,军事
90 后非脑残,军事
时尚女装,穿衣美容
时尚,文艺,旅游
文艺,美食
警察,派出所

处女座
穿衣美容,微博奇葩,旅游
电脑
健康
闷骚,娱乐
美食,娱乐
时尚,健康,旅游,星座命理,娱乐
投资理财,旅游,健康,汽车,体育,狮子座
校园生活,娱乐
海底囚人,自然之敌P,VOCALOID,终焉之栞,ib,弹丸论破,梦日记,阳炎project,音乐,游戏
娱乐,军人控,喜欢唱歌
体育
汽车,新闻资讯,健康,旅游,星座命理
美食,娱乐,旅游,设计,摄影爱好者,动漫,水瓶座
美食,时尚,汽车,投资理财,娱乐
星座命理
娱乐,唱歌,电影,音乐
文艺,投资理财,时尚,美女,美食
美食,小熊座少女
游戏动漫,投资理财,星座命理
文艺,美食,旅游,听歌,天蝎座,文字,吃,小说,电影,旅行
旅游,美食,新闻资讯,投资理财,娱乐
宅女一枚,我就是我,电影爱好者,发呆,听音乐,学生,吃货,睡觉,金牛座
健康,体育,教育就业,文艺
歷史爱好者,电影迷,爱幻想,足球迷,篮球迷,A型血,摩羯座,乐天派,选择恐惧症,海贼王
旅游
私人派对,高尔夫,文化传播,游艇,商务休闲,高端娱乐,全球,PARTY,YVS,美女
心理学,爱电影,周杰伦,低调,90後,神经质,跳舞,爱旅游,房地产,IT数码
投资理财,汽车,娱乐,旅游,军事,体育
娱乐
天枰座,羽毛球爱好者,羽毛球,听歌,善良
时尚,娱乐,健康,星座命理,美食
体育资讯,搞笑幽默,星座,打球,工作,放轻松就好,阳光,运动
美食,新闻资讯,健康,旅游
娱乐,美女,睡觉
美食,一直幸福着
视频音乐
专业推销三十年,3U党,猎奇爱好者,土豪,伪游戏宅,恋声癖,唱见厨,颜艺
上网,运动,乐观,天蝎座,生活,动漫,美女,看书,文学,90后
网络,旅行,搞笑,艺术,微博,娱乐,看书,互联网,时尚,动漫
臭美
体育,汽车
体育
健康,汽车,美女,体育

旅游,军事,美女,汽车
军事,计算机,时事,喜欢听歌,时评,奋斗,google,热爱生活
娱乐
名人明星,微博奇葩,萌宠,美女,视频音乐,文字
时尚,小腿控,射手座
旅游,吃货,水瓶座,宅
IT 数码
旅游,星座命理,美食,军事,文艺
IT 数码
游戏动漫,美女
文艺,娱乐
superjunior,阳光,钓鱼,腹黑,时事,公关,恐怖片,相声
爱情,美女,偶像剧,神经大条,宅女一枚,闷骚,护士,市场营销,音樂,视频
健康,娱乐,文艺,汽车,旅游,IT 数码
美女
文艺,游戏动漫,星座命理
新闻资讯
娱乐
娱乐,生活,电影,好书,网购,游戏,80 后,宅,旅游,听歌
娱乐,时尚,前沿,美女,江苏,好生活,摄影
娱乐
健康,新闻资讯,娱乐
汽车,健康,文艺,IT 数码,旅游,美食
健康,旅游,时尚,美食,文艺
新闻趣事,视频音乐,名人明星,微博奇葩,減肥中,吃货一枚,电影,听歌
搞笑幽默
吃,睡觉,听歌
时尚
天秤座,冷笑话,看电影,精神病人,骑行,名侦探柯南,动漫,音乐,宠物
运动,美女,休闲,时尚潮流,室内设计,时尚,星座命理,旅游,美食,体育
旅行,手机,电影,美食,摄影,互联网
娱乐
旅游,文艺,星座命理,贪玩,宅女,学生,英语,狮子座,90 后
IT 数码
游戏动漫,动漫,学生,处女座,宅,搞笑
自由职业,偶喜欢玩,平常心,摄影爱好者,搞笑,美食,生活,乐观,交友,旅游
人力资源
旅游,搞笑幽默,新闻趣事
IT 数码
旅游
时尚,军事,汽车,旅游,美食,文艺,体育
摄影爱好者,音乐爱好者,运动健身,看书,交友
美食,星座命理,90 后,网络,娱乐,文学,语录,新闻,音乐,校园
游戏动漫,IT 数码

公司战略与风险,经济法,税法,财务成本管理,审计,会计,雅思,澳洲,ACCA,CPA
视频音乐
旅游
北京生活,旅游,摄影爱好者
健康,旅游,投资理财,星座命理,美食
美女,宅女
美食,星座命理
IT 数码
天蝎座,渣剑三中,电四,蝶恋花,军爷一枚
教育就业,时尚,星座命理,美食,娱乐,双鱼座,90 后,艺术
体育,90 后但我不脑残
旅游,健康,时尚
时尚
娱乐,体育,美女,文艺,健康
体育,微博,电影,自由,音乐,浪漫,交友,新闻,玩儿,听歌
IT 数码
游戏动漫,军事,自由
星座运势,狮子座,90 后,吃货,听歌
视频音乐
美女,体育
旅游,娱乐,文艺,美食,爱音乐,幻想
汽车,旅游,IT 数码,娱乐,越野爱好者,音乐爱好者,满洲里,内蒙古,文化,原创
旅游,时尚,美食,IT 数码
投资理财
惠州生活,游戏动漫,基友控,90 小正太
天秤座,善良,紫色,偶稀饭睡觉觉,80 后
网络,手机,游戏,宅男,微博,偶稀饭睡觉觉,睡觉,摩羯座,音乐,80 后
娱乐
美食
娱乐
游戏动漫
文艺,星座命理
娱乐
体育,蓝色,篮球爱好者,学生,运动,天蝎座,自由职业,平常心,搞笑,幽默
娱乐
军事,IT 数码,文艺,体育,娱乐
新闻资讯,军事,体育,文艺,汽车
李霄云
手机,幽默,羽毛球,交友,宅男,睡觉,网购,偶喜欢玩,双鱼座,90 后
游戏动漫,星座命理
军事
IT 数码
名人明星
汽车,美食,文艺,健康,旅游

IT 数码
旅游
娱乐
新闻趣事,美食
星座命理
健康
浙江生活,自由,听歌,户外,狮子座
咖啡控,巨蟹,星座命理,任性,荷西,孙少安,健康,旅游,美食
文艺,旅游,美食
奋斗,咖啡,巨蟹座
星座命理,时尚
名人明星,belieber,爱 1D,90 后,英语,宅女,天蝎座
游戏动漫,星座命理,文艺
汽车
视频音乐
新闻资讯,教育就业,文艺,体育,摩羯座
美食,新闻资讯
宅女,80 后,娱乐,新闻,财经,创业,睡觉,时尚,浪漫,语录
沈阳生活,天蝎座
教育就业,投资理财,IT 数码,娱乐,文艺
汽车,游戏动漫
A 股,运动,听歌,玩儿,自由
游戏动漫
旅游
扬州生活,体育资讯
江苏生活
环保
旅游,美食
军事,汽车,体育
娱乐
美女
平常心
汽车
旅游,狮子座
新闻资讯,开朗,感性
时尚,投资理财,旅游,健康,美食,听歌,80 后,宅女
宅女,乐观,听歌,美剧,语录,英语,90 后,平常心,天秤座
穿衣美容
时尚
美女,娱乐,80 后,偶喜欢玩,旅游,电影,天蝎座
杭州生活,旅游,主题活动,原版绘本阅读,幼儿教育,英语启蒙
体育资讯
篮球,摩羯座,唱歌,90 后,乐观,学生,自由
美女

星座命理
美女,美食
学生一族,游戏动漫
微博奇葩,搞笑幽默
IT 数码
新闻趣事,荆州生活,搞笑幽默,星座命理,穿衣美容
听歌
玩儿
汽車,美女
文艺
o 型血,宅男,双鱼座男生,历史,好书,美剧,手机,80 后,听歌,英语
玩儿,游戏,小说,幽默,吃,电影
IT 数码
投资理财,旅游,美食,文艺,娱乐
IT 数码,美女
美食,旅游,电影
旅游,美食,健康
健康,美食
矛盾综合体
IT 数码,教育就业,娱乐
时尚,美女,军事,IT 数码
美食
搞笑幽默
游戏动漫
时尚
娛樂,音乐,听歌,学生,校园
娱乐
旅游,美食,文艺,新闻资讯,星座命理,体育
娱乐
微博奇葩
娱乐
时尚,美食,健康,文艺,旅游
投资理财,汽车,健康,旅游,美女
旅游
平常心,好性格
美食
广告,交友,阅读,听歌
游戏动漫,文艺
新闻资讯,淡定,平常心
旅游,星座命理,美食,文艺,娱乐
时尚
旅游,校园生活,视频音乐,体育资讯,搞笑幽默
美女
育儿百科,宠物图集,美食,学习

星座命理
摄影,好书,乐观,文学,文字,阅读,交友,大学,金牛座,90 后
乐观,游戏,睡觉,天秤座,吃货
IT 数码,美女,汽车
文艺,IT 数码,旅游,美食,美女
搞笑幽默
美食,教育就业,文艺,健康
游戏动漫
娱乐,教育就业,旅游,健康,文艺
娱乐
搞笑幽默,感性,高,瘦
投资理财
美食,登山
文艺,健康,新闻资讯
天蝎座
娱乐
美食
萌宠
美女,健康,IT 数码,娱乐,体育
体育
篮球,唱歌,看书,自由,运动
北京生活
广告
时尚
娱乐,时尚,健康
宅男,英语,手机
汽车
名人明星,娱乐
萌宠,旅游,美食,微博奇葩,穿衣美容,美食控,睡懒觉
势空才能化大鹏
星座命理,旅游,健康
体育,90 后
李宇春的玉米,李宇春控
投资理财,军事,旅游,新闻资讯,汽车
星座命理
动漫
爱吃,懒人,高中生,A 型血,欧美控,巨蟹娃,比伯控,90 还算后
温州生活
时尚,文艺
亲情
军事
旅游
IT,学生
旅游

健康,娱乐,美女,时尚,星座命理
视频音乐
各种宅
新闻趣事,音乐,文字,运动,旅行
旅游,汽车
美食,娱乐,游戏,吃货,新闻,八卦,爱情,听歌,自由,上网
文艺
宠物图集,狮子座,阳光
美食,萌宠,视频音乐,旅游
时尚,娱乐,美食,游戏动漫
新闻趣事
旅游,美食
娱乐,睡觉
陕西生活,发呆,旅行,平常心,偶喜欢玩,吃
自行车,品牌,fashion,旅游
爱玩,90后非脑残,BIGBANG,发呆,爱睡觉,EXO,双子座,吃
星座命理,美食,旅游,娱乐,IT数码
旅行,小说,音乐,睡觉,吃货,摄影爱好者
美食
慢热,旅行,体育,金牛座,音乐,电影,善良,文字,吃,90后
美食,视频音乐
军事
水瓶座
新闻资讯,汽车,旅游,文艺,娱乐
星座命理,旅游,娱乐,时尚
韩国代购,日本药妆,内衣裤打底袜,代购进口护肤品,代购进口化妆品
军事,新闻资讯,汽车,IT数码,文艺
处女座,好性格,音乐,好书,唱歌,听歌,语录,90后,爱情,电影
爱阿狸,晒不黑,吃不胖,情绪控,爱文字,爱电影,90后,洁癖,很白,讨厌爱装的人
投资理财,新闻资讯,美食
星座命理,投资理财,时尚,吃
囧,星座命理,游戏动漫,IT数码,美女,娱乐
时尚,汽车,IT数码,体育,娱乐
娱乐,IT数码,旅游,星座命理,美食
娱乐
体育,傻,赛车,潮
中国移动通信
投资理财
旅游,新闻资讯,军事
善良,偶稀饭睡觉觉,上网,英语,自由,搞笑,娱乐
微博,吃货,听歌,美食,羽毛球,音乐,水瓶座,90后
喜欢网购,看电影听音乐,留学生一枚,有点小宅,天蝎座的90后,小说控,动漫,美食
微博奇葩,穿衣美容,星座命理
美食,萌宠,视频音乐,星座命理,双子座,游戏,魔法,异能,动漫,COS

新闻趣事
旅游
游戏动漫,汽车,投资理财,新闻资讯,健康,IT 数码
游戏动漫,汽车,健康,旅游,美女
爱情
PS,创意,修图师,修图,广告,合成,retouch
湖人,逛街,疯狂,狗狗,玩,学生一族,唱 K,散步,科比,宅女
旅游
善良,互粉,狗狗,护肤,爱睡觉,美食
美女
旅游
杭州生活,奋斗,紫色,HK,gmat,ielts
名人明星,青春,真诚,音樂
新闻资讯,时尚,旅游,美食,娱乐
体育
宅
媒体人,地产,资讯,广告人,执着,天黑请闭眼,真三国无双,爱音乐,艺术,好性格
星座命理,美食,娱乐
视频音乐,chicano,健康,西藏命运,哲学,欧美音乐控,黑人说唱,牙买加文化,街头篮球
文艺,教育就业
旅游
美食
健康
文艺,时尚,星座命理,旅游,健康
搞笑幽默,90 後,篮球
名人明星,美女,校园生活,高中生,林峰,LOL
军事
富顺二中,富顺,自贡,18 岁,富顺交友,时尚,生日 6 月 22,dota,幽默,交友
体育
穿衣美容
美食,听歌
投资理财,旅游,美食,IT 数码,文艺,90 后
健康,娱乐
投资理财,健康,旅游
漫画,猫咪,萌宠,爱吉他,小说绘,胡椒粉,白羊座,90 后
名人明星,玩儿,射手座,吃
旅游
新闻趣事,搞笑幽默,好书,娱乐,吃货,学生,自由,90 后,巨蟹座,东方神起
时尚,韩国代购
娱乐
爱音乐,校园,文字控,星座,文艺,爱旅游,微博控,巨蟹座,历史,文字
上海生活
80 后
巨蟹座,80 后,宅男,自由职业,浪漫,网络,乐观,互联网,汽车,吃货

教育就业,星座命理,健康,新闻资讯,投资理财
娱乐
Swiftie,阿狸控
插头插座试验机,电源线弯曲试验,IEC 检测设备,单档多档冲击锤,盐雾试验机,UL 探针,IEC 试验指,安规检查机械
旅游
健康,时尚,旅游,美食,娱乐
教育就业
英语,易感动,学生族,水瓶座女生,宅
美食,健康,教育就业,娱乐,旅游
星座命理,旅游,时尚,娱乐,文艺,喜欢干净,单身,幽默搞笑,喜欢唱歌跳舞
娱乐
自由,睡觉,吃货,娱乐,偶喜欢玩,听歌,美食,音乐,天秤座
娱乐
游戏动漫
美食,jolin,时尚
时尚
网络
射手座,怀旧
名人明星
时尚
旅游,游戏动漫
旅游,美女
美女,投资理财,健康,旅游,体育
娱乐
时尚,新闻资讯,星座命理,健康
投资理财,旅游,IT 数码,体育
投资理财
投资理财,时尚,旅游,星座命理,娱乐
小说,动漫
IT 数码,数码
安徽生活,萌宠,美食,小说,动漫,睡觉
星座命理,娱乐
搞笑幽默
时尚,IT 数码
美图摄影
健康,美食
乐观,交友,旅行,美食
投资理财,文艺,时尚,健康,美食
旅游
健康
旅游,美女,娱乐,IT 数码
IT 数码
80 后

教育就業,娛樂,投資理財,旅遊,星座命理
美食
旅游,美食,星座命理
娱乐,星座命理,投资理财,白羊座宅女,浪漫,睡觉,90后,听歌,媒体
娱乐,美女,时尚,投资理财
美食,遊戲動漫,旅遊,文藝,娛樂
娱乐
时尚,死飞
鹿晗
娛樂
投资理财,健康,美食,娱乐,射手座
旅游,文艺,投资理财,IT数码,军事,汽车
文艺
时尚,健康
汽车,体育
80后,迷糊,天秤,学生一枚,旅游美食,寂寞,高考,话剧,方大同,电视
娱乐,时尚,文艺,美食
旅游,健康,美食,新闻资讯,时尚
旅游,星座命理,睡觉,摄影,美食,大学生
婚纱摄影工作室,广州个人写真,时尚,广州婚纱摄影
美食,旅游,健康,时尚,娱乐
IT数码,娱乐,教育就业,新闻资讯
星座命理,美食,健康,旅行,吃,宅
教育就业
时尚,文艺,娱乐
IT数码,汽车,健康,体育,娱乐
娱乐
爱玩,发呆,疯子,吃货
娱乐
四川生活,美食
游戏动漫,音乐,看书,动漫,宅女,睡觉,小说,金牛座,吃货
美女
电影,娱乐,历史,动漫,看书,幽默,善良,音乐,吃,乐观
汽车
新闻趣事,搞笑幽默,穿衣美容,星座命理,微博奇葩
旅游,巨蟹座,可爱
营销,旅行,听歌,上网,好性格,平常心
80后,迷糊,天秤,学生一枚,旅游美食,寂寞,高考,话剧,方大同,电视
旅游
娱乐
时尚,美食
新闻趣事
新闻趣事,旅游,名人明星
新闻资讯,旅游,时尚

旅游,娱乐,生活,美食,听歌,传媒,音乐,读书,平常心,天秤座
美容服饰
军事,美食,美女,游戏动漫,文艺
喜欢听歌,美食控,懒,名人明星,旅游,动漫,吃货,校园,交友
爱小帅,善良,艺术,音乐,电影,幽默,吃货,玩儿,动漫,90 后
上海生活
电影,笔控,音乐,手机控,旅游
健康,娱乐,星座命理,游戏动漫,军事
美食
游戏动漫,时尚,娱乐,美食,IT 数码
旅游
扬州生活
美食,娱乐
外贸,男人,杂志,济南生活
旅游,微博奇葩
美女
娱乐
漫画,朋友,爱情,我就是我,彩妆
文艺
文艺
80 后,小说,亲情,读书,自由,平常心,乐观,文学,天蝎座
旅游,娱乐
星座命理,美食
投资理财,健康,星座命理,旅游,娱乐
文艺
天秤座,搞笑,音乐,玩儿,上网,90 后
跑步,电影,看书,娱乐,美食
汽车,军事,娱乐,体育
电影,体育,天蝎座,爱情,NBA,运动
星座命理
娱乐,宅,搞笑,好性格,音乐,幽默,吃货,唱歌
囧
白羊座,发呆,美食,乒乓球,强迫症,神经质,A 型血,环保,计算机
旅游,美食,时尚,美女,娱乐
游戏动漫,体育,星座命理,IT 数码,宅,娱乐
游戏动漫,腐女,宅女,小说,动漫
汽车
娱乐
美食
名人明星,自由,八卦,体育,运动
娱乐
新闻资讯,旅游,文艺,娱乐
IT 数码
旅游,外贸业务,外贸

旅游
视频音乐,新闻资讯
美女,游戏动漫,IT 数码
旅游,读书分享
新聞資訊,旅遊
设计,听歌,90 后,吃货,电影,好性格,幽默,音乐,善良
自由
美食,文艺,娱乐,旅游,游戏动漫
吃货,自由,80 后,偶稀饭睡觉觉,电影,音乐,天蝎座,美剧,宅女,旅游
天蝎座,吃货,海绵宝宝,瘦身,偏执狂,英国,向日葵
狮子座,八卦,吃货,音乐,自由
美食,军事
旅游,体育,文艺,水瓶座,篮球,文字,电影,交友,好书,NBA
张根硕,张根硕的鳗鱼,愛根碩,天秤座
IT 数码,体育,美女,娱乐
文艺,美食
健康,新闻资讯,时尚,投资理财,教育就业
2,淘宝
健康
小鬼刘忻,爱刘忻,芯片,刘忻控,潮忻,刘忻
音乐,时尚,娱乐,IT
行一件好事,心中泰然,行一件歹事,心中惭愧,善良,音乐,自由
新闻资讯,汽车,体育,美女
美女
美食,时尚,娱乐,星座命理,唱歌,天蝎座,吃货,自由,上网,听歌
台球,随性,怀旧,体育,摄影爱好者,创业,交友,旅行,电影,微博
文艺,健康,旅游,体育,投资理财
微博奇葩,视频音乐,新闻趣事,搞笑幽默,满足,乐观,温暖
双鱼座
配音,唱 K,主持人,旅行,传媒,时尚,美食,英语
视频音乐
汽车,游戏动漫,文艺,体育,娱乐
吃货一枚,美食控,看书,室内设计,囧,摄影,新闻,历史
靠谱好青年,时尚达人
北京生活
旅游,星座命理,美食
美食
小说
名人明星
懒,居家,臭美,踏青,妖精,西门子,施耐德奇胜,吸血鬼日记控,旅游,美食
广东生活
新闻资讯,健康,娱乐
旅游,创业,阅读,语录
体育

美食
旅游,星座命理,文艺,美食,时尚
娱乐
文艺
星座命理,瓶男,O 型
游戏动漫
福建生活,单身青年,麦霸,宅,屌丝,金牛座,80 后
水瓶,词曲作者,宅男一枚,网络歌手
时尚
体育
文艺
自由职业
军事
文艺,国学,传统文化,云南师范大学,汉服
电影,篮球,靠谱小青年,旅行
睡懒觉,美食,听歌,逛街,游玩,逛街或者公园,骑单车,喜欢溜冰,天秤座,90 后
搞笑,电影,音乐,旅游,怀旧,财经,金牛座,自由
爱生活爱自己,时尚,自由
游戏动漫,动漫迷,会书法,最近在学小提琴,喜欢画画
考研,宅,动漫爱好者一只,网购,英语,上网,偶稀饭睡觉觉,吃货,旅游,听歌
军事,新闻资讯,旅游,IT 数码,汽车
名人明星,狮子女,减肥,逛街,开朗,90 后非脑残,吃货,乐观,宅女
看电视,销售,自由,宅,动漫,看书,睡觉,上网,娱乐,时尚
篮球爱好者,最爱大北京,台球发骚友,天蝎座,电影爱好者,户外,爱唱歌,理财
感性,乐观,听音乐,陈绮贞,旅游爱好者,吃货,运动,自由,天秤座
搞笑幽默
7758258,娱乐,音乐,水瓶座
IT 數碼,投資理財,體育,体育
游戏动漫
娱乐
羽毛球,摄影爱好者,旅游爱好者,IT 数码,英语
90 后
体育
文艺,美食,游戏动漫,娱乐
IT 数码,文艺
旅游,科学探索,读书分享,视频音乐
投资理财
视频音乐,北京生活
美女,摄影
搞笑幽默,宅男,音乐
美食
星座命理,美女
娱乐
克拉玛依生活,大学生,运动,互联网,大学,双子座,好性格,90 后,篮球

美女,时尚,汽车,运动
真实力,银魂,许嵩,小清新,海绵宝宝,笑话,潮流,心理学,爱电影,听音乐
穿衣美容
健康,旅游,美食,文艺,娱乐
时尚,游戏动漫,射手座,90后,小说
文艺,新闻资讯,健康,旅游,美食
旅游
娱乐
健康,旅游
网购,偶稀饭睡觉觉,白羊座,上网,微博,偶喜欢玩,好性格,善良,亲情,娱乐
旅游
星座命理
搞笑幽默
娱乐
美女,K歌,音乐迷,时事
穿衣美容
娱乐,大学生
娱乐
天蝎座
美女
荆州,写作,吉林,兜风,餐饮,爱情
体育
文艺,大学
时尚
视频音乐,星座运势,名人明星
穿衣美容,舞蹈,it互联网
体育,时尚
上网,宅,数码,音乐爱好者,麦霸
健康,设计,玩儿,看书
伪飞友,F1车迷,铁粉,科研民工
80后,游戏,看书,天秤座
娱乐
健康,文艺,时尚,娱乐,旅游
旅游,美食,穿衣美容
汽车,文艺
单身,吃货一枚,咖啡控,拍照,电影迷
热爱生活,健康,旅游,星座命理,美女,投资理财,旅行,自由,白羊座
美女
贪吃,萝莉型,善良单纯是好人,搞笑派,水瓶座,小清新,O型血,Music,唱K
摄影
帅气逼人,黑白色,搞笑,动漫爱好者一只,狮子座
教育就业,时尚,文艺,娱乐,白羊座,音乐,旅游,看书,吃货,宅女
我是个好人
文艺,星座命理

娱乐,IT 数码
时尚
健康,jolin 粉,在校学生 90 后,上网
旅游,广东生活,没意思,羽毛球,微博,听歌,音乐,紫色
模特,模特经纪,杭州经纪人,杭州模特,杭州礼仪,杭州兼职,杭州大学生兼职
宅女,处女座,旅行,动漫,听歌,90 后
投资理财,旅游,美女,IT 数码,汽车,娱乐
巨蟹座,汽车,数码
健康养生,阳台种菜,健康知识,养生常识
教育就业,汽车,美女,军事,娱乐,搞笑
健康
健康
娱乐
娱乐,投资理财,CFA,交流观光团,NUS,浙江大学,色影无忌,直排滑,溜冰
美食,美女,旅游,时尚,小说,交友,唱歌,娱乐
台州生活
微博奇葩
IT 数码,娱乐,时尚,文艺
文艺
时尚
八卦,吃,交友,学生,天蝎座,旅游,偶喜欢玩,自由,90 后,乐观
美食,名人明星,搞笑幽默
美女
新闻趣事,搞笑幽默,湖北生活,穿衣美容,微博奇葩
美食,旅游,游戏动漫
互粉,美食控,单身,美食,体育
体育,IT 数码,美食,星座命理,投资理财,新闻资讯
八卦杂谈
天蝎座,时尚,90 后,广告,小猪罗志祥,炎亚伦,张檬
上网
运动,旅行
新闻资讯
好性格,乐观
教育就业,娱乐,时尚,健康
處女座,上网,运动,90 后,宅,摄影爱好者
美食爱好者,动漫控,傻,音乐发烧友,乐观,搞笑,神经大条,手机看小说
健康,80 后
IT 数码
健康保健,养生文化,中医中药,中医美容,五行,针灸
懒,情绪化,简单,喜欢听歌,医学,音乐达人,游戏,天秤座,搞笑,善良
旅游,视频音乐,房产,搞笑幽默,南京生活,运动,数码,网络,音乐
娱乐
淘宝达人,淘宝客 vip
IT 数码,新闻趣事

军事,美食,时尚,新闻资讯,文艺
穿衣美容,新加坡生活,大学生,美剧,天秤座
搞笑幽默
美食
新闻趣事
新闻资讯,旅游,游戏动漫,美食
许嵩,忧郁,夜猫子,猫咪控,腐女宅女耽美狼,瓶邪王道,黑執事,双鱼座,旅游
广告,创意,美食,女人,购物,美妆
新闻资讯
游戏动漫,IT 数码,街舞控,Rapper,WOWer,电脑爱好者,小漫迷
文艺,娱乐
搞笑幽默,视频音乐,体育资讯,IT 数码,旅游
星座命理,新闻资讯,美食,娱乐,体育,美食,健康,美女
亲情,新闻,90 后,校园,善良,文学,创业,语录,宅,旅行
微博奇葩
娱乐,新闻资讯,IT 数码,教育就业,投资理财
美食,新闻趣事
语录,足球
星座命理,自由
娱乐,星座命理,美食
投资理财,健康,旅游,美食
微博奇葩,美女,视频音乐,搞笑幽默,新闻趣事
时尚,健康,美食,娱乐,文艺
美食
搞笑,网购,时尚
娱乐
迷恋星空,爱 k 歌,学生一族,射手座,喜欢笑,神经大条
IT 互联网,喜欢音乐,经济,执着,时尚
宅宅宅宅宅宅女,90 後
爬山,新闻资讯,娱乐,护肤,化妆品,青春,爱自由,吃喝玩乐,猫,双子座
情感,心理学,语录,亲情,喜欢旅游,浪漫,文字控,古典音乐,心灵深处的风景
旅游美食爱好者,健康,游戏动漫,汽车,平常心,亲情,时尚,看书,自由
美女,NBA
娱樂,美食
星座命理
投资理财,美女
穿衣美容,神经质,做梦,胡思乱想,疯子,三分钟热度
旅游
八卦杂谈
美食,投资理财,时尚,娱乐
美食
设计,IT 数码,商业,美术,文艺
旅游
投资理财,新闻资讯

娱乐
新闻趣事,宠物图集,美食,北京生活
大学生,吃,幽默,电子商务,读书,白羊座,生活,旅游
文艺,健康,时尚,旅游,美食,学生
旅游
复古,原单,中旅商业城,淘宝店,实体店,欧美日韩女装
游戏动漫,美女,教育就业,投资理财,汽车,IT 数码
体育
娱乐,健康,旅游,星座命理,美食
穿衣美容
时尚,健康,美食,旅游,星座命理
电子商务,绿色食品,健康生活
娱乐
汽车
娱乐
80 后,处女座,偶稀饭睡觉觉,吃,娱乐,爱情,生活
体育
旅游,美食,健康,睡觉,游戏,手机,娱乐,听歌
旅游,体育
射手座,80 后,交友,幽默,睡觉,美食,网购,旅游,时尚
娱乐,游戏动漫,星座命理,美食,体育
游戏动漫
偶稀饭睡觉觉,123
健康
紫色
多元化,时尚养生,新开元,杭帮菜
唱歌,吴世勋,高三,美食,旅游,健康
传媒,大学生
美食,汇聚
搞笑幽默
美食,2B 青年欢乐多
旅游
游戏动漫
吃,旅游,时尚,白羊座,美食,80 后
名人明星,福今茶叶
游戏动漫,IT 数码,投资理财
80 后,白羊女
幽默,旅游,美食,人生,健康
80 后,育儿,学生,福建,宁德,同城,生活,狮子座,淘宝,母婴用品专卖
健康,旅游,美食
羽毛球
浙江生活
娱乐
白羊座,时尚

為前途,拼人生,贏未來,我無悔
奋斗,IT 互联网
疯狂,坏脾气,双鱼女,自由,音乐,唱歌,浪漫,爱情,睡觉,时尚
娱乐,我是我,希望被在乎的我
健康
自拍
学习,音乐迷,自然,喜欢音乐,天秤,简单,天秤座,美食,文艺,娱乐
汽车,LesT
时尚,教育就业,IT 数码,文艺,投资理财,B3 文艺青年,困货,篮球,平常心
旅游,美食,娱乐,喜欢网络歌曲,无厘头宅女,偶稀饭睡觉觉,电影,巨蟹座,90 后,搞笑
军事
健康,美食,汽车,写作,媒体,运动,财经,处女座,看书,旅游
健康,互联网,就爱吃,80 后,幽默,美食
美食,时尚,娱乐
娱乐,睡觉,电影,偶喜欢玩,射手座,自由,小说,听歌,吃货
搞笑幽默
旅游,娱乐,星座命理,美女
娱乐,网络,电影,睡觉,听歌
美食
桌球,书籍,运动,旅游美食爱好者,旅遊
水瓶座,吃,123,电影,睡觉
美食
游戏动漫
美食
辽宁生活,搞笑幽默,新闻趣事
投资理财,汽车,游戏动漫,旅游,体育
怀旧,懒人,电视剧,自拍,喜欢唱歌,美食,旅游,娱乐
紫色,唱歌,浪漫,娱乐,美食,旅行,听歌,金牛座,乐观,时尚
设计,小说,90 后,宅女,音乐,八卦,吃货,睡觉,美食
文艺
平常心,时尚
游戏动漫,新闻资讯,文艺,IT 数码
室女座,可爱,审计,人马座,写作
看书,传媒
欢迎关注,互粉,恩施利,青岛朗维斯,木器家具保养,木蜡油,柚木油
歌神，烹饪
名人明星,美食,八卦杂谈,新闻趣事
美女,搞笑幽默
IT 数码
福建生活
旅游
天蝎座,巨蟹座,双鱼座
美食,游戏动漫,美女,时尚,娱乐
星座命理

旅游
星座命理
新闻资讯
时尚,旅游,星座命理,美食,娱乐
A 股,写作
体育
美食
健康
娱乐
时尚,娱乐
时尚,星座命理,旅游,游戏动漫,文艺
新闻趣事,休闲,爱吃,管理,时尚潮流,公益
新闻资讯,美食,娱乐,文艺
娱乐,任督二面,问题收容所
美食,囧,吃货,紫色,宅女,听歌,90 后,文字,处女座
游戏,美食,电影,网购,偶喜欢玩,玩儿,紫色,天蝎座,娱乐,幽默
娱乐,美食,旅游
旅游,娱乐
旅游
校园生活,视频音乐
随手拍,美食达人,羽毛球,暴走,金融,大小非减持,军迷,股票,宅,看书
IT 数码
新闻资讯
搞笑幽默
温州生活,2B 青年,双子座,炎亚纶
英语教育,培训
新闻资讯,教育就业,投资理财,健康,旅游,金牛座
电影爱好者,欧美音乐,旅游美食爱好者,爱猫,遊戲動漫,美食,文艺,旅游,娱乐,小说
健康
湖北生活
赚钱钱钱钱钱钱,汽车
娱乐,体育,游戏动漫
名人明星,搞笑幽默,微博奇葩,视频音乐
广州恒大足球队,粤语,网络营销,足球,幽默,互粉,音乐爱好者,IT 数码,体育
看电影,团购,看小说
健康,体育
新闻趣事,微博奇葩,搞笑幽默,游戏动漫,旅游,军事,体育
旅游
乐天派,音樂,笑话,神经质,看小说,电视剧,娱乐,美食,好人卡收藏家,听歌
学习国学,儒释道,健康生活,快乐工作
娱乐,旅游,健康,美女
美食,旅游,教育就业,汽车,IT 数码
新闻趣事,搞笑幽默,微博奇葩,囧,明星,网游
星星,学生一枚,漫画,美食控,旅游,游戏动漫,教育就业

时尚, 爱杂志款, 第一站美甲, 青田五十街
旅游, 美食, 栎迷, 芋头, 90 后
电子商务
旅游, 星座命理, 美食, 娱乐
健康, 娱乐, 旅游, 营销, 生活
音乐, 骑行, 自行车, 摄影, 电子商务
文艺
娱乐
呼伦贝尔生活
旅游
新闻资讯
懒, 笑, 微笑, 游戏动漫, 动漫控, 强迫症, 星座控, 90 後, 巧克力, 狮子女
娱乐
微博奇葩, 偶喜欢玩, 自由, 紫色, 双鱼座, 摩羯座, 娱乐, 英语, 吃货, 囧
时尚, 90 后
双子座
搞笑幽默
娱乐
健康, 旅游, 美食, 文艺, 娱乐
文藝, 奋斗, 强迫症
跳舞, 听歌, 看书
体育
名人明星
体育资讯
投資理財, 娛樂, 文藝, 時尚, 旅遊
投资理财, 时尚, 旅游, 文艺, 健康, 爱自由
星座命理
新闻资讯, 旅游, 美食, 文艺
娱乐
搞笑幽默, 羽毛球, 校园, 巨蟹座, 财经, 紫色
体育
娱乐
足球, 游戏, 汽车, 篮球, 看书
文艺, 教育就业, 新闻资讯, 健康, 旅游
体育, 平常心
体育
体育
游戏动漫, 体育
文艺, 旅游, 吃货
悲惨
大大咧咧
昆明生活, 徒步旅行, 自行车爱好者, 体育, 偶喜欢玩, 80 后, 电子商务, 网络, 善良
微博奇葩, 浙江生活, 穿衣美容
娱乐, 美食, 旅游, 星座命理, 健康, 任性, 星座控, 吃货, 唱歌, 电影

听歌,摩羯座 O 型
视频音乐,好好爱爱我的人,对我好的人
熬
电影,乐观,体育,创业,吃,金牛座,80 后,交友,文学,运动
体育
自由,搞笑,自由职业,广告,吃货,偶稀饭睡觉觉,玩儿,90 后,双鱼座,微博
时尚,90,街舞,爱音乐,笑,自然,幸福,音乐爱好者,阅读
旅游
娱乐
美女,时尚,投资理财
旅游
教育就业,旅游,文艺,时尚
偶稀饭睡觉觉,宅,偶喜欢玩,射手座,七叶草,家人,五迷
唱歌,吃货
视频音乐,名人明星,旅游,穿衣美容,美女
娱乐,军事
体育
90 后,天秤座,吃货,亲情,八卦,偶稀饭睡觉觉,学生,紫色,动漫,电影
文字
湖北生活,旅游,微博奇葩
健康
游戏,八卦
美食,旅游,搞笑幽默
娱乐
IT 数码
重庆生活,读书分享,情感生活,名人明星,新闻趣事
为爱走天涯,诗词,听戏,魔羯座女子,走路看风景,文字,西湖志愿者,历史地理民俗,品茶,阅读
旅游,文艺
搞笑幽默
游戏动漫
教育就业,时尚,投资理财,旅游,美食
奔三族
校友交流,企业宣讲招聘,创业指导,经济学系,就业指导,漳州师范学院
娱乐
时尚
美食
体育
投资理财
懒,美食,旅游
文艺,汽车,新闻资讯,时尚,IT 数码
Coffee
健康,时尚,教育就业,星座命理,娱乐
游戏动漫

时尚
搞笑幽默,80后,巨蟹座,篮球,音乐
文艺,娱乐,健康,旅游,美食
双子座
CF,广科,广东科技学院,南博,松昌,潮州,宅男,大学生,90后
大学
美女
旅游,学生,篮球,汽车,上网,旅行
旅游
名人明星,视频音乐,读书分享,搞笑幽默
涂鸦,音乐,摩羯座
星座命理,健康,投资理财
娱乐
体育,娱乐,篮球,看书,天秤座,90后,羽毛球,好性格,运动,听歌
美女
娱乐
美食,投资理财,新闻资讯,IT数码,健康
新闻资讯
股票,传媒,A股,文学,网购,电子商务,广告,汽车,营销,网络
幽默,双鱼座
娱乐,投资理财,旅游,军事,文艺
穿衣美容,青岛,造型师,模特,帅哥,音乐,幽默,巨蟹座,另类90后
健康
新闻资讯
娱乐
微博,娱乐
文艺
娱乐
旅游
星座命理,汽车,军事,IT数码,健康
文艺,IT数码
射手座,仙剑,历史,运动
上网
美食
娱乐
文艺
游戏,90后,旅游,美食,音乐,爱情,运动,网购,唱歌,天蝎座
旅游美食爱好者,小资,咖啡控,淘宝控,宅女,烹饪,狮子座,生活
艺术,动漫
美食
搞笑幽默
搞笑幽默
语录
旅游,原创,平面设计

旅游,IT 数码,娱乐,美女,汽车
娱乐
汽车,新闻资讯,天蝎座,好性格,睡觉,搞笑,生活,财经,上网
法語,動漫,文艺
美女
时尚
金牛座
校园生活
时尚
IT 数码,宅,睡觉,90 后,听歌
动漫,宅男,平常心,白羊座,NBA,校园,囧,娱乐,游戏,看书
体育
健康,星座命理
爱生活
军事,体育
文艺,游戏动漫,时尚,美食
体育
旅游
文艺,IT 数码
美食,文艺
美食,美图摄影,时尚,健康
喜欢听歌,WESTLIFE,westlife,Westlife
美食,旅游
娱乐,星座命理,旅行,平常心,水瓶座
健康,天蝎座
旅游,学生
新闻资讯,汽车,美食,娱乐,IT 数码,旅游,时尚
摩羯座,自由
校园,心理学,睡懒觉,文艺,投资理财,旅游,星座命理,美食
幽默,射手座,自由,金牛座,听歌,音乐
新闻资讯,美女,美食,军事,娱乐
旅游,发呆
文物博物馆,传统文化,艺术,国画,篆刻,书法
美女,大学生
娱乐
旅游
时尚,健康,旅游,美食,娱乐
阅读
时尚,美食,文艺,游戏动漫,星座命理,吃货,天秤座
美食
旅遊,時尚,娛樂
健康,旅游,星座命理,军事,汽车
摩羯座,英语
长沙生活

美食
音乐,天蝎座
异国恋,次奥,欧美控,时尚
名人明星
健康,汽车,星座命理
摩羯座,囧
美食
时尚,星座命理,军事
旅游
军事,IT 数码,文艺,娱乐,体育
文艺
时尚
善良,搞笑,交友
娱乐
游戏动漫,天蝎座
美食,旅游,娱乐
艾尚真分享群
星座命理
音乐,好性格
搞笑幽默
情感生活,新闻趣事,校园生活,惠州生活,多愁善感,沉默,微博
健康
娱乐,时尚
IT 数码,时尚,军事,星座命理,娱乐,手机,90 后
体育,娱乐
健康,美食
健康,体育,网球
美女,新闻资讯,IT 数码,军事
娱乐,健康,美女
双子座,游戏
时尚,教育就业,旅游,美食,星座命理
穿衣美容
android,登山,羽毛球,幽默,k 歌,IT 互联网,健身,汽车
微博
星座命理
旅游,IT 数码,军事,汽车
音乐,动漫
IT 数码,网球,宅男,音乐,双子座,数码,微博,爱情,幽默
吃喝玩乐,NBA,旅行
IT 数码,文艺,体育,娱乐,美女
爱生活,陈奕迅,流浪,强迫症,天秤座,看电影,旅游美食爱好者,美剧控
天蝎座
篮球,读书
南京生活

时尚,星座命理,美食,旅游,健康
星座命理,美食
穿衣美容,美食,丹东生活,善良,天秤座,浪漫
旅游,健康,娱乐,星座命理,美食
美食
美图摄影,动漫迷,高考,懒,银魂,伪宅,摄影,吃,美食,旅游
娱乐
旅游,星座命理,美食,时尚,娱乐
娱乐
美食
A 型血,宅女,微博,学生,娱乐,上网,音乐,小说,幽默,双鱼座
80 后
爱幻想,团购,健康
视频音乐
小胡椒要高考了,胡歌,
美女,教育就业,IT 数码
音乐,学生,摄影,天蝎座,动漫,自信乐观可爱,90 后
娱乐
体育,娱乐,星座命理
新闻资讯
时尚,旅游
教育就业,时尚
时尚,旅游,娱乐,星座命理,美食,IT 数码,美女,投资理财,军事
IT 数码,体育
军事,IT 数码,文艺,体育,汽车,时尚,美女,美食,娱乐
体育
旅游,娱乐,文艺
天秤座,偶稀饭睡觉觉,90 后,动漫,宅男
美女
旅游,穿衣美容,美食,萌宠
汽车,旅游,IT 数码
美食,军事
新闻资讯,游戏动漫,时尚,汽车,教育就业
宅女,平常心,电影,音乐
文艺,健康,美食,旅游
游戏,听歌,金牛座,美食,旅游,羽毛球,偶稀饭睡觉觉,90 后
文艺,娱乐,美食
天蝎座
新闻趣事
校园生活
美食,健康,汽车,文艺,娱乐
体育
星座命理,美食
I,am,just,an,ordinary,teenage,girl

少女时代
美食
投资理财
穿衣美容
宠物图集,读书分享,搞笑幽默,视频音乐,美食
旅游,美食
搞笑幽默,新闻趣事
旅游
搞笑幽默
生命在于折腾
教育就业,投资理财
体育
健康
旅游
偶稀饭睡觉觉
文艺,娱乐,水瓶座,吃货,偶稀饭睡觉觉,文字,听歌
美食
电子商务
健康,星座命理,美女,游戏动漫,时尚,美食,新闻资讯,汽车,文艺
美食
投资理财,健康,汽车,娱乐,教育就业,美食,IT 数码,军事,文艺,体育
我就是我
游戏动漫
浪漫,乐观,巨蟹座,善良,摄影爱好者,听歌,自由,写作,历史,电影
美女,视频音乐,校园生活,成都生活,搞笑幽默,SPORTS,幽默
新闻资讯,汽车,旅游,IT 数码,经常无言以对,偶喜欢玩,懂我才知道我好,射手座,90 后
穿衣美容
美食,教育就业,星座命理,军事,游戏动漫
看书,旅游,处女座,吃货,摄影爱好者,听歌,90 后
时尚
美剧
紫色,语录
股票,互联网
搞笑幽默,视频音乐,萌宠,星座命理,美女,学生一枚,懒人,爱唱歌,文艺,看电影
星座命理,数码,平常心,摩羯座,语录,运动
美女,游戏动漫,旅游,美食,时尚,娱乐,水瓶座
体育
辽宁生活
睡觉,o 型血,摩羯座,连接服务器失败,美食
诸葛亮,泰坦尼克号,暮光之城,天涯明月刀
时尚
文艺,旅游,美食
旅游
旅游,厦门一中,动漫,美食,天秤座,文学,羽毛球

健康,美食
历史,80 后
健康,旅游,旅行,瑜伽,双鱼,上网
藤井 lena,MUSIC,VIP,TOKYODream,BigbangGD
星座命理
旅游
旅游,时尚,美食,娱乐,音乐
旅游
英国,韩国,咖啡,上海,冷笑话,星座命理,美食,文艺,旅游,新闻资讯
新闻趣事,情感生活,八卦杂谈,名人明星,校园生活
健康
广东生活,双子座
时尚,IT 数码,娱乐
喜欢德国,喜欢香港,旅游,周杰伦铁杆粉丝,听歌,游戏,双子座女生,幽默,乐观,美食
时尚
喜欢周杰伦
娱乐
不善表达,内敛
教育就业,健康,旅游,文艺
体育资讯
狗狗,外貌协会,金庸,完美主义者,三分钟热度,睡觉,看书
射手座
文艺
体育,发呆,火影忍者,热情,无聊,宅,唱歌,上网,品牌,孩子气
游戏动漫,星座命理,军事
偶稀饭睡觉觉,摄影,80 后
Music,喜欢唱歌,时尚
投资理财,汽车,健康,美食,旅游,天秤座,爱旅游,爱生活,爱学习,有追求
IT 数码,时尚,汽车,娱乐
时尚,星座命理,健康,教育就业,吃货,搞笑,宅
星座命理,健康,娱乐,游戏动漫,旅游,教育就业
文艺,娱乐,体育
好性格
旅游,时尚,投资理财,IT 数码,美食
唱歌,金牛座
健康
汽车,教育就业,投资理财,健康
睡觉
网上开店,休闲女装专卖店,自由职业,营销,网购
娱乐
美食,健康,旅游,美女,教育就业,大学生,听歌,90 后
一摔,最爱鸡肉卷
IT 数码
微博奇葩

大学生
美女
创业,动漫,好书,旅游,电影,校园,营销,读书,自由,NBA
美食
哥哥,宅男,宅
飞机稿,TVC,广告文案,广告创意
羽毛球,平常心,旅行,自由,偶喜欢玩,上网,手机,偶稀饭睡觉觉,吃,听歌
美食,营销,偶喜欢玩,听歌,电影,80后,语录,玩儿
美食,教育就业,文艺,娱乐,旅游
新闻资讯,投资理财,旅游,汽车,美食
美食
旅游,体育
娱乐
体育
美女,时尚
视频音乐
娱乐
娱乐,文艺
流苏,鬼万,忻玲
名人明星
搞笑幽默,bigbang,NBA,90后,处女座,手机,篮球,游戏,上网,听歌
和田地毯,新疆地毯,艺术品,艺术品投资,波斯地毯,奢侈品,投资品,收藏级,手工制作,家居装饰
健康
文艺
金牛座,爱好法语,紫色
美女
美食,娱乐,IT数码
体育
游戏动漫,娱乐,文艺
周杰伦
IT数码,文艺,汽车,时尚,游戏动漫
美女,旅游,文艺
娱乐
娱乐
爱玩,电脑,乒乓球,游戏动漫
体育
新闻资讯
闷骚4级,宅腐入门级,冷漠3级,猫鱼结合体,爱幻想
新闻资讯
时尚,旅行,历史,财经,音乐,旅游,自由,生活,网购,创业
娱乐
视频音乐,金属,后摇,cult,死宅
游戏动漫,星座命理,美食,健康,时尚,投资理财

娱乐, 时尚, 星座命理, 美食, 疯狂
上网, 90 后
游戏动漫
星座命理
广州生活
体育, 汽车
娱乐
好性格, 看书, 亲情, 听歌, 动漫, 学生, 乐观, 小说, 囧, 微博
旅游
帅哥, 美女, 锻炼, 音乐, 谈恋爱, 逛街, 看电影, 旅游, 看书
旅游, 星座命理, 教育就业, 娱乐, 健康
宠物图集, 美食, 健康, 旅游, 星座命理, 娱乐, 看书, 网购, 处女座, 90 后
中高考辅导, 艺考直通车, 音乐, 交友, 教育培训
时尚, 体育
时尚, 文艺, 健康, 旅游, 星座命理
新闻资讯
穿衣美容
名人明星, 吃货
云南生活
娱乐, 旅游, 时尚, 体育
历史
体育
美女
美女
新闻资讯
体育
自拍控, 钢琴, 吃货
八卦杂谈
美食, 娱乐
健康, 教育就业, 新闻资讯, 投资理财, 军事
汽车, 自由, 运动, 旅游, 射手座
运动旅行阅读, 萌宠控, 自由随性小狮子, 淡泊宁静平常心, 偶稀饭睡觉觉
名人明星, 新闻趣事, 搞笑幽默, 微博奇葩
IT 数码, 文艺, 时尚, 健康, 旅游
搞笑幽默
穿衣美容, 娱乐, 宅女, 吃货一只, 90 后, 吃货
全剧终, 一个, 你还欠我
天蝎座, 80 后, 善良, 亲情, 唱歌, 电影, 旅行, 宅, 乐观, 上网
90 后非脑残, 双子, 神经质, 强迫症, 旅游
大学生
巨蟹座
星座命理
旅游, 教育就业, 军事
历史

旅游,健康,军事,游戏动漫,好性格,好书,广告,运动
时尚
健康,旅游
澳大利亚生活,体育,IT 数码
美食
八卦杂谈
体育
时尚,教育就业,文艺,IT 数码,健康
IT 数码
娱乐,美女,天秤座,网购,搞笑,篮球,亲情,语录,音乐
教育就业
健康,时尚,文艺,旅游,无奈的天秤
80 后,音乐,摩羯座
音乐发烧友
宅男,美女
体育资讯,学生,游戏,双子座,听歌,体育,搞笑,校园,动漫,交友
游戏动漫
文艺
感性,各种玩,爱睡觉,随便,韩庚,文字,旅行,吃货,帅气,天秤座
星座命理
娱乐
天蝎座
星座命理,教育就业,旅游,美食,娱乐,唱歌听歌
LADYGAGA 美图控
宠物图集,校园生活,新闻趣事,美食,搞笑幽默,待解救女青年
投资理财,美食,健康,时尚,娱乐
乐活,打球发呆听音乐,游泳滑雪,美剧,文字,浪漫,摄影爱好者,旅行,天秤座,读书
萌宠,美食
星座运势,读书分享,美食,校园生活,搞笑幽默
娱乐,矮丑穷姑娘,死阿宅,炎阿布控
交友,美女
交友,动漫,好性格,校园,白羊座,艺术,小说,听歌,自由,学生
美食
新闻资讯
搞笑幽默,初二学生,宅男,狮子座,文艺青年,乐观
打篮球,90 后,白羊座
视频音乐,宅女,90 后,音乐,跳舞,校园,上网,听歌,巨蟹座
视频音乐,校园生活,搞笑幽默
健康
聊天,ColinMorgan,偶稀饭睡觉觉,音樂,英格兰,动漫控,腐女,摩羯座,大学生,看书
IT,手机
投资理财,旅游,健康,娱乐
旅游,旅行
星座命理

旅游,时尚,健康,投资理财,美食
新闻资讯
美食
穿衣美容
护肤品,化妆品,日系,美容
健康,旅游,游戏动漫,娱乐,文艺
旅游
新闻资讯,休闲生活,咖啡,吃喝玩乐
恋声
微博
奋斗,完美主义
巨蟹座,发呆,瑜伽,情感
穿衣美容
校园,音乐
旅游,健康,时尚
美食,吃货,自由,旅行,设计,80 后,睡觉,小说
旅游美食爱好者,交朋友,帅哥,爱唱歌
娱乐,梁静茹鱼迷,听歌唱歌自由宅,看书,睡觉
河池生活,美图摄影
音乐,阅读,平常心,生活,旅游,90 后
新余脑补少年,仰天岗车神,新纺吐槽狂魔,摩羯座
健康,文艺,教育就业
营销,双子座,唱歌,90 后
旅游,美食,IT 数码,文艺,娱乐
新闻资讯,在线振动监测,故障诊断,ISO 国际,振动分析师培训
文学
吃货,微博
旅游
旅游,娱乐,体育
同志,咖啡,简单,摇滚,笑话,音乐控,时尚,旅游,文艺
体育
游戏动漫
白羊座
美食
军事,娱乐,旅游,时尚,美女
星座命理
体育
美食,军事,娱乐,时尚,美女
JazzDancer,爱音乐爱唱歌,浙江
美食,汽车,体育
时尚,娱乐,健康,美女,旅游,平常心
美食
穿衣美容,美容护肤,摄影爱好者,读书
娱乐

娱乐
星座命理,娱乐
娱乐,摩羯座
体育
娱乐
美食,娱乐
新闻趣事
天秤座,单身,浪漫,懒,玩,双重性格,唱K,奋斗,爱旅游,90後
狮子座,好性格
搞笑幽默,睡觉,宅,学生,漫画,快乐,爱笑,玩,互粉,简单
文艺,时尚,美食,健康,旅游
星座命理,商务英语,唱歌,宅,音乐,射手座
新闻资讯,健康,旅游,美食,军事
肥秋推,s1,好人,阿宅,才不是阿宅,南外
旅游,时尚,美食,IT数码,娱乐
美女,娱乐,时尚
汽车,IT数码
游戏动漫,军事,IT数码
美食,李成种,金明洙,李成烈,李浩沅,南优贤,张东雨,金圣圭,INFINITE
文艺,时尚
名人明星,新闻趣事,视频音乐,美食,旅游,自由,平常心,宅女,巨蟹座
萌宠
旅游,时尚,文艺,投资理财,娱乐
体育
90後,迷茫,准毕业生
IT,时尚
文藝,體育,娛樂,知念侑李,山田凉介,HeySayJUMP,巨蟹座,学生
江苏生活
文艺,求勾搭,原宿原宿原宿,多风格,LOLITA爱好者,淘宝店主,在校大学生
80后,双鱼座
娱乐
强迫症,美食,听歌,90后,偶稀饭睡觉觉
象屿商城
旅游
健康,自由,90后,玩儿,财经,处女座,乐观
星座命理,旅游,睡觉,摩羯座O型,90后
美食
吴海波
双子控,电影,自由,外貌控,千年正太吧,射手座,自信
旅游,美食,微博奇葩,名人明星
大学城,手撕鸡,健康,美食,随手香,特色熟食,特色卤菜
名人明星,美食,旅游,美女,穿衣美容
美食,IT数码,健康,美女,游戏动漫
健康,新闻资讯

健康,旅游,美食,娱乐,星座命理
美女
美食
213,金牛座
WEB 前端,草根站长,GEEK,IT,杂志控,SEOer
娱乐,美女,星座命理,美食
时尚,投资理财,旅游,美食,娱乐
湖南生活,美食,搞笑幽默
金牛座,学生
娱乐
旅游,美食,投资理财,文艺,新闻资讯
IT 数码
投资理财,游戏动漫,旅游,娱乐
熬夜强迫症,彩虹糖,RTA 少年组,刘俊麟,徐浩,朱元冰,溢声军,左溢,金牛座,吃货
旅游,健康,娱乐,时尚,星座命理
体育,娱乐
旅游,福建生活
娱乐,非诚勿扰,日语控,天蝎 00 后,学生,吃货,小说,动漫,cosplay
天蝎座
SK 初心,山夫妇大好,天然长末低调萌,双蛋逼 O 退散,资源只服务基友,不混饭群
健康,新闻资讯,旅游,军事,体育
汽车
浪漫
TIMEV 赢时代,SEO,电子商务,网络营销
搞笑幽默
旅游,教育就业,浪漫
健康,文艺,新闻资讯,娱乐
无所谓的乐天派,三分钟热度,选择恐惧症
美女,旅游,美食,文艺,体育
处女座
投资理财
巨蟹座
张国荣 Leslie,美剧控,神经质的射手座,奋斗中的 90 后
美食,新闻资讯,健康,文艺,教育就业
汽车,投资理财,娱乐,文艺,星座命理,天秤座,O 型血,爱情,小说
电影,计算机软件,写作,乒乓球,苹果
数码
娱乐
IT 数码
星座命理,HR
娱乐,时尚
健康,时尚,星座命理,美食,文艺
娱乐,时尚
娱乐

大学,吃喝玩乐,画画,双重性格,向日葵
游戏动漫,健康,旅游,星座命理,美食,娱乐
美食,泉州生活
娱乐
旅游
汽车
娱乐
美女,娱乐,文艺,IT 数码
体育
香港,狮子女,玩儿,时尚,开朗,购物,旅游美食爱好者,爱笑的女孩,睡觉
旅游,名人明星,美女,穿衣美容,星座命理
旅游
NBA,湖人,军事,健康,汽车,投资理财,体育
军事
双鱼座,生物,金牛座,帅哥,反应佳,牺牲奉献,西安
美图摄影,唯庚
新闻资讯,健康,旅游,投资理财,时尚
视频音乐,囧,宅男,偶稀饭睡觉觉,学生,吃货,音乐
123,幽默
娱乐,投资理财,教育就业,健康,星座命理,文艺,听歌,语录,天秤座
體育,健康,遊戲動漫,旅遊,投資理財
读书分享
旅游
自由职业
游戏动漫,时尚,美食,娱乐,文艺,正太控,杯子控,微笑控
健康,新闻资讯,娱乐,星座命理
游戏动漫
平常心
时尚,娱乐,游戏动漫,浪漫,听歌
文艺
摩羯座
时尚
星座命理
游戏动漫
睡覺
汽车,自由,财经,大学,上网,摄影,射手座
乐观,旅游,艺术,美食,时尚
体育,娱乐
时尚,投资理财,美食,星座命理,健康
佛牌链,佛牌代请,招财女神,古曼童,蝴蝶牌佛牌,药师佛佛牌,四面神佛牌,必打佛牌,崇迪佛牌,泰国佛牌
娱乐
电脑,摄影爱好者,旅游,美食,星座命理,游戏动漫,IT 数码
宅,运动,亲情,足球,上网,美食,电影

新闻资讯
新闻资讯,旅游,时尚,文艺
读书
旅游,教育就业,汽车,健康,投资理财
连云港生活,文艺
时尚,新闻资讯,旅游,文艺,娱乐
搞笑幽默,NBA,音乐
金院,幽默,善良,宅女
体育
睡觉,吃货
军事
无欲无求
旅游
时尚
体育
IT 数码,文艺,体育,游戏动漫,新闻资讯
旅游,汽车,美女,娱乐
文艺
美容服饰
美食
体育
视频音乐,语录
动漫
名人明星,旅游,萌宠,搞笑幽默,福建生活
体育,小说,游戏,电影,上网,篮球,网络,双鱼座,天蝎座
时尚,平常心,吃货,摩羯座,大学生,玩儿,网购,好性格
羽毛球,网购,交友,电影,双子座
我一直都在,青春不死
娱乐
投资理财,健康,时尚,旅游,IT 数码
旅游,美食,文艺,娱乐
旅游
80 后,浪漫
时尚,旅游,星座命理,美食,娱乐,网购,亲情,美食控,吃货
穿衣美容,浪漫,双鱼座,善良,娱乐,自由,宅女
清爽环境,优雅的音乐
旅游,军事,IT 数码,投资理财,体育
美女,时尚,汽车
美图摄影
娱乐
新闻资讯,时尚,游戏动漫
90 后但我不脑残,文字,90 后
IT 数码,玩儿,吃货,90 后,网购
营销,听歌,阅读,平常心,乐观

巨蟹座,娱乐,听歌,90 后,唱歌,网购
娱乐,90 后,狮子座,自由
生活,美女,阅读,乐观,自由
星座命理,美食,文艺
穿衣美容,旅游
80 后,偶稀饭睡觉觉,宅
双鱼座
乐观,自由,90 后,玩儿,羽毛球,设计,自由职业,吃货,搞笑,射手座
个人
快乐控,全球控
体育资讯
游戏动漫
星座命理,美食,文艺,娱乐,狮子座,肉包控,爱独自听歌
军事
美食
汽车,旅游,文艺,体育,思考,书,发呆,打球,喜欢旅游
没动漫就死星人,宅
囧,浪漫,宅女
美图摄影,90 后,摩羯座,善良,听歌,臭美的懒人,爱唱歌的女生
美食,星座命理,旅游,健康,时尚
文艺
美食
投资理财
天蝎座,猫咪控,爱自由,穿衣美容
美女
美食
文字
90 后
旅游,名人明星,星座命理,视频音乐,搞笑幽默
美图摄影
旅游
创业,文化,公关,淘宝,博客,爱音乐,健康,广告,媒体,传媒
旅游
音乐,90 后,历史,NBA,浪漫,好性格,偶稀饭睡觉觉,宅女,自由
健康
娱乐
美食,时尚,健康,旅游,娱乐
水瓶座
宅男
萌宠
紫色
投资理财,新闻资讯,娱乐,健康,体育
文艺,教育就业,新闻资讯,健康
娱乐,时尚,美食,旅游,星座命理

美食,绿色控,玩儿,自由,囧,吃货,天蝎座的 90 后,宅女
旅游
exo,李宇春,白羊女,懒
旅游
旅游
湘潭生活
星座运势
健康
水墨画,绘画,書法,书法,盆景,艺术
旅游
旅游,投资理财,汽车,娱乐
时尚,90 后,文字,学生
美女,汽车,IT 数码
遊戲動漫,文藝,美食
时尚,投资理财,旅游
笑
娱乐
无爱人士,疯子,射手座,宅男
神经质,搞笑幽默
大学生,玩儿
新闻资讯,娱乐,军事,美食,旅游
旅游,娱乐,健康,军事,体育,金牛座,音乐,伪摇滚青年,旅行
黑木耳,射手座,90 后,苹果族,小说,动漫,游戏,成长 ING,起床苦难户
游戏动漫
旅游,美食,星座命理
自由职业
旅游,娱乐
美女
偶喜欢玩,囧,美食,吃,学生
情感,跑步,80 后,好性格,文字,自由,摩羯座,电影,好书,美女
学生一族,简单
名人明星
烘焙爱好者,吴彦祖,王力宏,爱狗,花草控,摄影,电影,读书,旅游,咖啡
顾弄选虚,粉丝控,你好我安,互粉成灾,怪谈热线,嗨歌,伊蚊猪,猪迷,80 后,读书
旅游
文艺
文艺,娱乐,投资理财,时尚,美女
旅游,美食,游戏动漫,文艺
张根硕河北后援,鳗鱼,张根硕后援会,张根硕
旅行
历史
文艺,娱乐,健康,游戏动漫,星座命理
宅
IT 数码,文艺,体育,娱乐

汽车,投资理财,旅游,美食,IT 数码
星座命理
娱乐
健康,美食,教育就业,汽车,文字,生活
穿衣美容,90 后
水瓶座
微博奇葩
冬天
旅游,文艺
体育
小说,韩剧,环保,高尔夫,justinbieber,阿森纳,快乐大本营,工作狂,少女时代,炎
旅游,娱乐
名人明星,微博奇葩
旅游,吃货
强迫症,美术,唱歌,动漫控,爱笑
星座命理,投资理财,健康,游戏动漫,IT 数码
毒舌,光头,痞子,老青年,文艺,卖萌,双子座
旅游,电影,足球,运动,80 后,平常心,小说,宅
搞笑幽默
动漫迷,音乐爱好者
奢侈品,代购,香水,时尚,成熟优雅,女人,法国巴黎
旅游,汽车,美女,游戏动漫,90 后
美食
新闻资讯
新闻资讯
搞笑幽默
旅游
健康,囧,平常心,八卦,双子座,宅
旅游
90 后的老太婆,K 歌,金牛,腐女,宅,大学生
處女座,聽歌,旅游
IT 数码,旅游
美食
游戏动漫,娱乐
游戏动漫,幽默
娱乐
视频音乐,读书分享,美食,新闻趣事,校园生活
文艺
教育就业,投资理财,娱乐,体育,IT 数码
网络键盘,玩转人生,狮子座,萌控,格斗,跑酷
美女,游戏动漫,教育就业,娱乐,文艺
美剧,网购,小说,游戏,80 后,宅男,音乐,吃,动漫,水瓶座
娱乐,囧,吃喝玩乐,K 歌,双重性格,钓鱼,双子座,设计,90 后,旅游
廣東人,世界杯

游戏动漫,教育就业,IT 数码
爱紫色薰衣草,射手座
美食,娱乐,动漫,音乐,旅游,偶喜欢玩,摄影,自由,电影
旅游,健康,美食,娱乐
娱乐,体育,睡觉,90 后
健康,新闻资讯,文艺,军事,娱乐
教育就业,旅游,健康,时尚,文艺,游戏动漫,投资理财,IT 数码
星座命理
体育资讯
健康,文艺,时尚,星座命理,美食
外貌协会
美女,穿衣美容,美食
白羊,旅游摄影,Music,吃喝玩乐,投资理财,IT 数码
旅游,微博奇葩,新闻趣事,搞笑幽默,广东生活
游戏动漫
军事
投资理财,娱乐,体育,文艺,美食
旅游,时尚
文艺,健康
搞笑幽默,上网,乐观,旅行,宅男,交友,文学,音乐,新闻,网购
搞笑幽默,自行車,曼联,单反
娱乐,戒骄戒躁
美国生活
美食
娱乐
娱乐,IT 数码
文艺,美食,星座命理,被遗忘,音樂,97 天秤
健康,新闻资讯,美食
美食,执着,善良,运动,唱歌,爱狗,睡觉,看书,自由,宅
体育
时尚,美食,娱乐,西安
娱乐
娱乐,爱玩,幻想,浪漫,美女,摄影爱好者
唱歌
Supreme,讲中文
90 后
新闻趣事,搞笑幽默,美容服饰
宅女,处女座,90 后,动漫,善良,好性格,吃货,听歌,上网,旅游
军事,游戏动漫,汽车,体育
旅游,文艺,健康,美食,娱乐
健康,时尚,英语,旅游,听歌,交友,美食,双子座,吃货
军事,体育
美女
加拿大生活

旅游,体育
星座命理
手机,电影,金牛座,上网,80 后
IT 数码,汽车,星座命理,投资理财
美女,娱乐
美女,IT 数码
星座命理,没事发下白日梦
投资理财,教育就业
时尚,娱乐,美食,投资理财,旅游,星座命理,文艺
游戏动漫,投资理财,IT 数码,军事,体育
在读博士
旅游
股票,80 后
睡觉
娱乐,文艺,体育
看书
时尚
宅男,运动
兰大
单车一族,单车旅行,珠三角,广州天河,广州驴友,湛江,乐观,徐闻,中国大陆最南端
娱乐,非典型处女座,三无青年,腐女,90 后
旅游
星座命理,旅游,美食,健康,娱乐,文艺,听歌,美剧
时尚,吃货,双鱼座
新闻趣事,生活,好书,游戏,上网,传媒,自由,音乐,历史,篮球
美食,音乐
健康,美食
新闻资讯
时尚
美容服饰
美女
美女
搞笑幽默
青春,个性,时尚
时尚,汽车,美女,娱乐,体育
旅游,植物,旅行,音乐
时尚,健康,旅游,星座命理,美食
IT 数码
新闻趣事,宅女
90 后,偶稀饭睡觉觉,平常心,手机,交友,偶喜欢玩,美食,音乐,白羊座,乐观
体育
体育,教育就业,投资理财,军事,IT 数码
体育
广告,娱乐

校园生活,新闻趣事
自由,旅行
健康,旅游,时尚,汽车,投资理财
听歌
星座命理,体育,娱乐,汽车,军事
游戏动漫,军事,IT 数码,新闻资讯
娱乐
娱乐,体育,游戏动漫,天蝎座,游戏,偶喜欢玩,吃,搞笑
IT 数码,沉默
伪腐女真宅女,NBA 控,90 后,喜爱小包子,狮子座
汽车
美剧控,双重性格,吃货,神经大条,旅游
新闻资讯,游戏动漫,汽车,体育
篮球,一些事一些情,摩羯座,电子商务,自信,表演,狮子女,流浪
娱乐
游戏动漫,动漫爱好者
开心就好
美食,日语,内外兼修,時尚,大外女,粉色控,处女座
新闻资讯,旅游,美食
美食
投资理财
小说迷,小感动,睡觉,宅中宅,旅行,喜欢腹黑的主,动漫,小孩子
旅游,废柴君
时尚,健康,旅游,军事,娱乐
看书
微博奇葩
努力,吃货一枚,宠物,shopping,快乐大本营,休闲,宅女一枚,善良,小清新,听歌
穿衣美容
IT,帅哥,强迫症,减肥,旅游美食爱好者,学生一枚
娱乐,游戏动漫,文艺
时尚
健康,旅游,军事,IT 数码,娱乐
读书
唯梓,鱼葱,百合,姐控
篮球迷,爱艾儿,爱幻想,爱傻笑
黄石生活
语录,科技,新闻资讯,健康,教育就业,IT 数码
体育
旅游
旅游,娱乐,体育,美女,投资理财
迷糊,天秤,学生一枚,旅游美食,寂寞,高考,话剧,方大同,电视,80 后
旅游,美女,IT 数码,军事,体育
游戏动漫,美女
美食

扬州生活,上网,唱歌
自行车,爱,基督徒,90 后非脑残,同志,睡懒觉,爱吃,旅游爱好者,爱自由,爱情
旅游,时尚,游戏动漫
读书分享,美食,美图摄影,宠物图集,视频音乐
美食
动漫,动漫迷,设计,cosplay,猫,O 型血
123
IT 数码,汽车,音乐
游戏动漫
英语,新闻
游戏动漫
旅游,文艺,娱乐
旅游,健康,文艺,美食,娱乐
文艺,娱乐,体育,oo 后,清纯可爱,水瓶座,囧,传媒,上网,音乐
娱乐,星座命理
旅游,美食,IT 数码,文艺,体育
教育就业,娱乐,军事,新闻资讯,美女,健康,乐观,善良,金牛座,营销
旅游
IT 数码,旅游
IT 数码
投资理财,美女,星座命理,健康,娱乐
时尚,美食
旅游
娱乐
NBA
星座命理,个性,音乐迷,love,爱睡觉,减肥,傻,音乐爱好者,孙燕姿
吃货,傻缺,爱电影,旅行
娱乐
健康,星座命理
美食,听音乐,淘宝,宅,90 后
天蝎座
游戏动漫,时尚,娱乐,星座命理,美食
美图摄影
美女,旅游
积极向上好青年,大学生,读书
学生,自由
投资理财
时尚
萌宠,微博奇葩
健康,教育就业,旅游,新闻资讯,时尚,文艺,美食
新闻资讯,游戏动漫,星座命理,体育,时尚,娱乐
汽车,投资理财
健康
视频,搞笑,时尚,优惠促销,购物,生活,微博,90 后

旅游
美食,小说,宅女,音乐
传媒,时尚
医生不是神,电影迷,愤青,IT 人
90 后,好性格,偶喜欢玩,吃,自由,美食,娱乐,听歌,上网,射手座
美食,时尚
旅游
写作
体育
听歌,偶稀饭睡觉觉,美食,偶喜欢玩,电影,双鱼座,睡觉
90 后,天秤座,宅女
旅游
军事,旅游,文艺,体育,音乐,运动,天蝎座,偶稀饭睡觉觉,阅读,篮球
偶稀饭睡觉觉,篮球
时尚,健康,旅游,美食,星座命理
时尚,美食,旅游
军事,时尚,健康,旅游,美食
娱乐
娱乐
八卦杂谈,星座运势,情感生活,美图摄影,校园生活
美食
名人明星,穿衣美容,星座命理,搞笑幽默,微博奇葩,双鱼座
健康,旅游
娱乐,双子座,学生,游戏
旅游
娱乐,星座命理,美女,IT 数码,文艺
交友,设计,美食,玩儿,旅游,乐观,大学生,自由,网购,睡觉
手机,天秤座,学生,小说,文学,好性格,看书,听歌,宅,娱乐
旅游
狮子座
汽车
美剧
新闻资讯,健康,星座命理,娱乐
文艺,娱乐,90 后,浪漫,阅读,小说,好性格,狮子座,文字,学生
90 後,手工
娱乐
星座命理,健康
时尚
水产,阳澄湖,阳澄湖大闸蟹,美食
时尚,游戏动漫,美女,旅游,美食
幽默,娱乐,90 后,NBA,自由,吃货,汽车,好性格,处女座,听歌
投资理财,娱乐,文艺,IT 数码,美食
美食,旅游
80 后,旅游,文字,亲情,白羊座

IT 数码
星座控,清新菜鸟,平常心,艺术,白羊座,80 后
香橙,陈翔,娱乐
美食
旅游,体育,娱乐,时尚
新闻资讯,时尚
投资理财,教育就业,健康,旅游,娱乐
IT 数码
美食,迟小秋
巨蟹座,法律,家具,义乌,广州,律师,湖州
星座命理
夜猫子,神经大条,吃,旅游,爱电影,蓝色,90 后但我不脑残,美食,天蝎座
投资理财,营销,天蝎座,创业,交友,小说,90 后,电影,阅读,旅游
新闻资讯
美图摄影
设计,吉他,音乐,动漫
汽车
KOBE,90 后,运动,篮球,音乐,水瓶座,听歌,旅行
名人明星
动漫,狮子座,音乐,90 后,旅游,羽毛球,宅女,上网,看书,学生
广东生活,搞笑幽默
江苏生活,健康,旅游,美食
文艺
美食,健康,旅游,IT 数码,体育
宅女
英音,日音,宅,日漫,英剧,日剧,二宫和也,Arashi,AvrilLavigne
游戏动漫,汽车,体育,IT 数码
礼品供应,礼品批发,美容套装,美容套,蟹具,蟹工具,礼品生产专家,户外各类刀具,修脚刀套装,指甲刀套装
摄影爱好者
体育,娱乐,军事
时尚,新闻资讯,健康,星座命理,美食
萌宠
新闻趣事,宁夏生活,搞笑幽默
美女,游戏动漫
瑜伽,健康,娱乐,听歌,偶喜欢玩,音乐
美女,IT 数码,文艺,体育,娱乐
美女
学生,时尚,天蝎座,娱乐,音乐
娱乐,健康,军事,时尚,体育
RTS,国产 RPG,学酥,萌宠控,死宅,重度拖延症患者,二次元星人,仙剑
娱乐
狮子座,音乐,吃货,90 后,搞笑,平常心,文字,宅
体育

娱乐
购物,星座命理,娱乐,时尚,旅游,美食
游戏动漫,投资理财,旅游,娱乐,文艺,看书,天蝎座,学生
美食
穿衣美容,视频音乐,名人明星
旅游,美女,时尚,美食,投资理财,新闻资讯,体育
文艺,健康,旅游
愤青,笑话控,电器迷,音乐控
体育,汽车,军事,旅游,星座命理
娱乐
游戏动漫,IT 数码,文艺,体育,娱乐
娱乐
游戏动漫,汽车,星座命理,IT 数码,娱乐
汽车
新闻资讯,汽车,IT 数码
深圳,90 後,爱生活
文学,奋斗,旅游
美食
体育
情感生活
体育,游戏动漫,美女,星座命理,美食
白羊座,90 后
时尚
时尚,投资理财
时尚,怀旧,宅女
旅游,体育
IT 数码,游戏动漫,时尚,汽车,文艺
小说,宅,玩儿,吃
美食
搞笑幽默
洋葱少女玻璃心,宜室宜家好菇凉,万岁万岁万万岁,冷漠也热气腾腾,不折不扣缺点控,神仙姐姐不 lady,杜益民的脑残粉,阴暗纯情同比例,MR 是你也是我,晕头转向茫茫然
美食
军事
星座命理,新闻资讯,健康,旅游,美食
旅游
视频音乐
吃货
摄影控,汽车,健康,旅游,IT 数码
娱乐
重口味,美图摄影,视频音乐,新闻趣事,体育资讯
文艺
文艺
搞笑幽默

美食,时尚,文艺,健康,娱乐
娱乐
视频音乐
投资理财
娱乐
十连等云等凯旋,奋斗,吃喝玩乐,爱自由,美食控,活泼,学生一族
校园
求治愈,转战撸啊撸,心碎中,半宅男,魔术爱好者,游戏动漫,怪物猎人,爱看小说,Dota 菜鸟,
狮子座
IT 数码
娱乐
娱乐
足球,汽车,搞笑,NBA,唱歌,白羊座,90 后,乐观,听歌,旅游
手机,听歌
IT 数码
游戏,善良,自由,运动,创业
穿衣美容
旅游,星座命理,文艺,娱乐
折页,宣传册,名片彩页,广告设计
健康
娱乐,唱歌,弹琴
吃,娱乐,生活,偶喜欢玩,交友
旅游
星座命理,文艺,旅游,IT 数码,狮子座,网购,大学生,偶稀饭睡觉觉,偶喜欢玩,听歌
体育,文艺,IT 数码
文艺
游戏动漫,时尚,体育,娱乐,美女
体育资讯,IT 数码,体育,玩
投资理财,汽车,体育,健康,旅游
处女座
名人明星,搞笑幽默
新闻资讯,旅游,健康
时尚,睡觉
娱乐,文艺,美食
时尚,娱乐,美食
星座命理,双鱼座,娱乐
旅游
时尚,旅游,美食,文艺,旅行,音乐,电影,狮子座
旅游
健康,旅游,星座命理,美食,狮子座,时尚,好书,娱乐,游戏,小说
萌宠,微博奇葩,美食,浙江生活,搞笑幽默
搞笑幽默,魔羯座,90 後,喜欢音乐
星座命理
娱乐

教育就业,投资理财
美食,偶稀饭睡觉觉,吃货,听歌,天秤座
时尚,娱乐,旅游,美食,体育
军事,游戏动漫,时尚,美食
玩儿,文学
金贤重,exo,日韩欧美疯,爱吃不胖呵呵,外貌協會,摩羯座,牛灿,就爱我仔锅锅
爱情
旅游
美食,历史
微博奇葩
旅行
视频音乐
文字控,文艺
美女
小说,摩羯座 A 型女,动漫,宅女,吃货
美容美体,乐观,运动,射手座
80 后创业,金牛座,诚实可靠小郎君
水瓶座,游戏
双鱼座,摩羯,休闲,宅女加腐女,狮子女
运动
游戏动漫,娱乐,巨蟹座,好性格,偶喜欢玩,学生
文艺
IT 数码
旅游,美食,IT 数码
游戏动漫,IT 数码,文艺
时尚,旅游,健康
享受生活,理解人生,技术,无所不能,囧,艺术,吃货,幽默
健康,娱乐
文艺
时尚,旅游,美食,文艺,娱乐
很傻很天真,音乐,足球,吃货,动漫,唱歌,白羊座,90 后
美图摄影,视频音乐,名人明星,2PM,听歌,香橙
阅读,写作
美食,娱乐
文艺,娱乐
娱乐,美食,游戏动漫,美女
贤妻良母,听歌,上班族,80 后,等爱 ing
游戏动漫,体育,教育就业,IT 数码,娱乐
娱乐,星座命理,旅游,美食,文艺,无语
时尚,美食
娱乐
巨蟹座,股票,读书,唱歌,宅
娱乐
白贤,kris,瓶邪黑花,三分钟热度,盗墓笔记

萌宠,美食,穿衣美容,视频音乐,搞笑幽默
胡思乱想,神经大条,宅女一枚,多愁善感,护士,白羊女,睡觉睡到自然醒
娱乐
咖啡控,潮人,服饰,娱乐,汽车,文艺,时尚
健康
宅,动漫,小说
网购,汽车,搞笑,乐观,运动
文艺
处女座
游戏动漫,美女
双子座女生
广东生活
娱乐
搞笑幽默
新闻趣事
时尚
我就是我,哈哈,情绪化,偏执狂,体育
旅行,时尚,善良,财经,浪漫,网购,听歌,紫色,娱乐,唱歌
娱乐,旅游,美食
美食
歐美控,睡覺,愛旅行,自由,唱歌,愛音樂
相声,自信,大学生
旅游,文艺
双鱼座,向日葵,文字控,90後,爱幻想,书,学生一族,文艺,美食,旅游
游戏动漫,娱乐
体育,娱乐
美食
享瘦美好人生,健康瘦身,绿色减肥,最好的减肥药,减肥
娱乐,汽车
湖南生活,美食,星座命理,健康,汽车,旅游,军事,文艺,娱乐,体育
狮子座,阳光,梁静茹
微博奇葩,新闻趣事,旅游,美食,上海生活
娱乐
健康
美食,游戏动漫,新闻资讯,健康,美食,体育,听歌,NBA,上网玩游戏,看书
安徽生活
游戏动漫
爱吃,唱K,时装,音乐,宅女,美食,摩羯座,旅游,大学,时尚
旅游
娱乐
美女,体育资讯
教育就业,游戏动漫,星座命理
娱乐
美食

健康,写诗,老年人,失地农民,拆迁
上网,宅,听歌,睡觉,吃,娱乐,平常心,射手座
自信坚强乐观,执着,钢琴,90後,摩羯女,宅女
TOP
看小说,动漫控,游戏动漫
体育
视频音乐,新闻趣事,穿衣美容
旅游,IT 数码,刘杰毅
美食,旅游,名人明星
周杰伦,半个宅男,90後
笔控,NFL
体育,汽车,IT 数码,教育就业,投资理财
狮子座,交友,90后,旅行,上网,时尚
莆田生活,穿衣美容,天蝎座,90后,唱歌
健康
娱乐
坑爹,幽默,网控,吃货,屌丝男,狮子座
游戏动漫,体育
美食
游戏动漫
校友新闻,校友会,校园文化,教师动态,北京外国语大学
旅游美食爱好者,书法,逛街,音乐控,爱玩,80后,散步,购物狂,陈奕迅,旅游摄影
00后,妖精的尾巴,时尚潮流,休闲,动漫控,摩羯女,电视剧,兽王书迷,教育就业,星座命理
美女,美食,军事
唱歌
独立
文艺
美容服饰,乐观开朗,有一颗善良的心,没工作,没相貌,没身材,没钱,没车,没房,80后
金牛女,吃货一枚,小说,音乐迷,睡觉睡到自然醒,美食,文艺,娱乐
娱乐,游戏动漫,时尚
美女,遊戲動漫
投资理财,健康,星座命理,旅游,美食
车迷,教育家,体育迷,游戏达人
体育
游戏动漫
遊戲動漫
偶喜欢玩
文艺,娱乐,游戏动漫
娱乐
漫画,设计师,音乐,狮子座,好书,美食,动漫,小说,玩儿,电影
体育
洛阳生活,穿衣美容,新闻趣事,搞笑幽默,微博奇葩
文艺
时尚,娱乐,美食,乐观,阅读,摩羯座,睡觉,微博,文字

新闻资讯,旅游,文艺,美食,娱乐
青少年,网购没钱娃娃,11 班,水瓶座
时尚
好书,生活,娱乐
美女,旅游,美食,军事,娱乐
生活,电子商务,看书
体育
星星,胡思乱想,天秤,心理学,情绪化,健康,奋斗,笑,穿衣美容,搞笑幽默
娱乐
娱乐
新闻趣事,视频音乐,名人明星,体育资讯
搞笑幽默,美食,半糖梦境,薰衣草控的紫,爱林先生,唯一的唯一,偶稀饭睡觉觉,90 后,宅,摩羯座
新闻资讯,旅游,星座命理,时尚,娱乐
军事,IT 数码,体育
旅游,美食
投资理财,旅游,摄影,宅,音乐,诗歌,看书,生活
房产专家,音乐控,军事迷,笑话控
美食控,旅游,吃货一枚,爱睡觉,爱唱歌,懒人,魔兽世界
汽车,IT 数码,娱乐,数码,射手座,创业,生活,80 后
时尚,健康,旅游,星座命理,文艺
美食,旅游,文艺,时尚,星座命理
恋爱通告,幸福,星星控,医疗,菜鸟,黄鹂新村
美食,时尚,文艺,娱乐,星座命理,天蝎座,摄影爱好者,摄影
汽车,投资理财,美女,IT 数码,体育
校园生活,搞笑幽默,游戏动漫,美女,娱乐
汽车,体育,美食,IT 数码
娱乐
娱乐
IT 数码,汽车,美女,娱乐
吉他爱好者,花式调酒,poppin 爱好者,音乐,90 后
星座命理,游戏动漫,闷骚,听音乐,巨蟹,星座,动漫
旅游
旅游,天枰座,80 后,宅 T,限制级血腥片,Les,T
娱乐
投资理财,新闻资讯,健康,星座命理
军事
张国荣 fan
娱乐
旅游
时尚,健康,旅游,美食,娱乐
旅游,星座命理,美女
星座命理,文艺,娱乐
旅游,时尚,星座命理,娱乐,美女,文艺,投资理财,汽车

娱乐,美食,星座命理,旅游,IT 数码
时尚,教育就业
笔亲,摄影控,女人,ab 型,书籍,潮,金俊秀,盗墓笔记
微博奇葩,看电视
体育,娱乐,美女,IT 数码,新闻资讯,投资理财,汽车,星座命理,军事
bigbang
搞笑幽默
美食,萌宠,穿衣美容,星座命理,搞笑幽默
汽车
校园生活
娱乐
娱乐,时尚
健康
微博奇葩
旅游,美食,穿衣美容
广东生活,旅游,穿衣美容,动漫,乐观,电影,运动
IT 数码
星座命理,美女
游戏动漫,动漫
健康,体育,娱乐
时尚,游戏动漫,娱乐,美食,星座命理,狮子座小妹,紫色
穿衣美容
95 后,娱乐,微博,上网,电影,听歌,NBA
美女
娱乐
文艺
汽车
旅游
历史,读书,电影
体育
美食,微博奇葩,名人明星,萌宠,穿衣美容,狗狗
巴萨,五迷,胡思乱想,快乐,安静,懒,玩
时尚
娱乐,IT 数码,文艺,旅游,星座命理
射手座,IT 数码
体育,金牛座,篮球,NBA,动漫
时尚,娱乐,艺术派,麦霸,冷幽默,文艺女青年,唱歌,射手座,好性格
娱乐,旅游,时尚
微博奇葩,音乐,小说,电影
减肥达人,水瓶座,时尚,自由,偶喜欢玩,玩儿,好性格,运动
体育
巨蟹座,懒人,主持人,喜欢唱歌,90 后但我不脑残,上网,自由,学生,娱乐
星座命理
吉他,美国,苹果,随性,公务员

教育就业,美食,健康,娱乐,天秤座O型女,宅女一枚,快乐至上
心情,健康
穿衣美容
教育就业,娱乐,投资理财,健康,90后,天秤座,时尚,睡觉,听歌
运动,平常心,搞笑,宅男,金牛座,娱乐,美食,旅游
星座运势,美图摄影,读书分享
体育资讯
美食,囧人囧事,少言寡语,自由职业,射手座,交友,玩儿,幽默,搞笑,宅女
健康,投资理财
美女
体育资讯,体育,81,81精神
睡懒觉
新闻资讯,军事,IT数码
微博奇葩,听歌
星座命理,游戏动漫
喜欢安琪
校友新闻,校友会,校园文化,教师动态,北京交通大学
娱乐
美图摄影
广州生活
新闻资讯,娱乐,美食,IT数码,体育
浙江生活,2,海绵,傻笑,酒窝
教育就业,文艺,历史,阅读,读书,水瓶座
时尚潮流,服装,聊天,美食
娱乐,我是个开朗的人,睡觉,电影,游戏,学生
火影忍者,乐观,篮球,学生一族,90後,自由
时尚,唱歌,吃货,上网,听歌
偶稀饭睡觉觉,宅女,平常心,美剧,80后
八卦杂谈
吃,00后,看书,唱歌,玩儿,天秤座
娱乐
摩羯座
教育就业,文艺,健康,美食,旅游
篮球,典型巨蟹座,正直男,90后
军事,文艺,体育,IT数码,娱乐
韩国代购,护士
旅游,星座命理,娱乐,教育就业,健康
时尚,投资理财,旅游,健康,美食
上网,吃货,宅
小说控,家庭教师Reborn,逍遥自在,恐怖片控,地下城与勇士,天秤座
美食,萌宠,新闻趣事,穿衣美容,微博奇葩
美食,IT数码,汽车,旅游,体育
文艺
搞笑幽默

军事,逛街,看电影
美食
星座命理,IT 数码,文艺
文艺
教育就业
星座命理,娱乐,文艺,时尚,健康
名人明星,文艺
美女
善良,水瓶座
手机,交友,偶喜欢玩,紫色,90 后,网络,囧,宅,好性格,睡觉
爱电影
美女
娱乐
萌宠
睡觉,唱歌,音乐,上网
健康,新闻资讯,娱乐,旅游,美食
校友新闻,校友会,校园文化,教师动态,北京科技大学
文艺,美食,星座命理,健康,旅游
文艺,体育
美食
视频音乐,宅男
90 後,交友
原创,电影,90 后,设计,旅行,自由,听歌,广告,摄影
睡觉,电影,大学生,旅行,音乐
90 后,文字,美食
投资理财,教育就业,健康,美食,星座命理
星座命理,金牛座
星座命理,美食
微博奇葩,搞笑幽默
新闻资讯,军事,美女,数码,美剧,校园,大学生,电影,网购,听歌
健康,游戏动漫,教育就业,文艺,动漫控
美容服饰
唱歌
萌宠,新闻趣事,旅游,美食,搞笑幽默,吃货,汽车,射手座,90 后
投资理财,娱乐,汽车,IT 数码,美女
遊戲動漫,90,大学,海贼王,善良
美女
抠字眼大叔,律政不归路,求品质不凑活,madao,捉急狂,时尚,娱乐
投资理财,旅游,美女,体育
看书,爱旅行,搞笑,乐观,运动,新闻资讯,生活,围观群众
投资理财,文艺,军事,阅读,美食,营销,好书,80 后,创业
娱乐,汽车
娱乐,文艺,时尚,美食,旅游
旅游

军事
应急管理学院,预防为主,普及应急知识,社会安全,公共卫生,事故灾难,自然灾害,应急管理
旅游
美女
教育就业,投资理财,旅游,娱乐
游戏达人,音乐控,旅行,军事迷
娱乐,健康,时尚,生活,搞笑,乐观,美食,旅游
留学
生活,听歌
教育就业,健康,新闻资讯,文艺,时尚
美食,旅游,印剧,菲剧,NaYa,英剧,IPKKND,泰剧
娱乐,阅读,90 后,吃货,历史,巨蟹座
美食,旅游,体育
星座命理,IT 数码
IT 数码
星座命理
美食,军事,健康,娱乐,体育,游戏动漫,旅游,IT 数码
美女
体育
设计,文字,新闻,创业,看书,财经,自由职业
爱吃,旅游爱好者,随性,时尚,美食,文艺
健康
新闻资讯,商业地产资讯,市场营销策划,市场研究,市场调研,商业地产
名人明星,双子座男生
文艺
微博奇葩
购物狂,服装,淘宝,创业,淘宝控,电子商务,营销
旅游,美女,时尚,汽车,娱乐,偶稀饭睡觉觉,学生
美食
星座命理
搞笑幽默,美女,旅游,篮球,平常心,时尚,好性格,网购,汽车,处女座
微笑,爱狗狗,唱 k,爱笑,跳舞,自由职业
IT 数码
旅游
旅游,时尚,教育就业,投资理财,娱乐
文艺
自由
广东生活
旅游,健康,IT 数码
旅游,美食,IT 数码,文艺,娱乐
AnitaMui
搞笑幽默,新闻趣事,微博奇葩,南京生活,旅游
美食
点点控,娱乐,宅女一枚,可爱,小清新,爱帅哥美女,萝莉,狮子座,睡觉,学生

动漫,乐观,数码,上网,吃,旅游,80 后,汽车,手机,宅
时尚,星座命理,健康
视频音乐,美女,微博奇葩,搞笑幽默,大学生,诗词,美食,互联网,游戏,白羊座
娱乐
瑜伽,臭美,闷骚,找星星
游戏动漫
旅游,教育就业,文艺,娱乐
娱乐
旅游,健康,美女,时尚,美食,娱乐,IT 数码
美食,星座命理,教育就业,投资理财,健康
星座命理
新闻资讯,汽车
搞笑,好书,吃,唱歌,网购,看书,美食,金牛座,小说,幽默
游戏动漫,美女,美食,军事,IT 数码,亲情,囧,手机,宅女,篮球
健康,时尚,体育,忠实粉,游戏动漫,感慨,情话,小心情,孤独,憧憬
健康
娱乐
穿衣美容
娱乐
娱乐
娱乐,新闻资讯,美食,游戏动漫
IT 数码,90 后,读书
文艺
搞笑幽默
柯震东,花痴,白羊座,文艺
体育
游戏动漫,军事
校园生活,财经,交友,大学,学生
股票,双子座,汽车
娱乐
我的美丽日记,奶粉
新闻资讯,旅游,军事,健康
美女
体育,娱乐
情感,休闲,娱乐,艺术
体育,搞笑,自由职业
美食,星座命理,旅游,健康,娱乐
健康,美女,游戏动漫,军事,美食
凤台一中,安徽医科大学,八零后,80 后,B 型血,摩羯座,医护工作者,医护人员,医护
美食
时尚,投资理财,旅游,文艺
旅游,小说,听歌
诚信互粉,汽车,90 后,宅男,123
狮子座

投资理财
搞笑幽默,校园生活
娛樂
懒
名人明星,娱乐
旅游
名人明星
美食
健康
爱狗狗,思索人生,家居,music,娱乐
游戏动漫,动漫迷,Music,柯南迷,美剧,宅男,偶稀饭睡觉觉,吃货,巨蟹座,动漫
IT 数码
娱乐
美女,游戏动漫,时尚
美食
娱乐,看书,时尚,生活
天秤座 B 型男,喜欢旅游,80 后
娱乐,游戏动漫,美食,星座命理,旅游,吃货,数码,时尚
旅游,美食
体育
穿衣美容,新闻趣事,视频音乐,美食,旅游
视频音乐
旅游摄影,梅西,留学,柯南,吃货,90 后,音乐,学生,羽毛球
体育,文艺,美女,娱乐
旅游,星座命理,美食,文艺,娱乐
美食,健康,星座命理
投资理财,羽毛球,宅
星座命理,穿衣美容,旅游,美食,三亚大学
投资理财,时尚,星座命理,美食,娱乐
搞笑幽默,视频音乐,美女
玩儿,健身,新浪读书,电影迷,看小说,护肤,金希澈,a 型血,陈绮贞,留学生
娱乐
IT 数码,我可能不会爱你,微博,小说,听歌,电影,睡觉,宅女,巨蟹座,上网
桌游,吃喝玩乐,喜欢听歌,白羊女,电视剧,微博控,90 后,上网,吃货,学生
娱乐,体育,文艺,美食,游戏动漫
娱乐,体育,文艺,IT 数码,军事
校园,学生
终极忍者,且行且远,内心探索,简单快乐,BusinessWoman
军事,古典艺术,elf,双鱼,计算机,电影爱好者,韩国,苹果
浪漫,胡思乱想,快乐大本营,时装,睡觉睡到自然醒,购物,包包控,K 歌达人,逛街族,淘宝控
娱乐,健康,旅游,时尚
唱歌达人,留学咨询,购物狂,吃货一枚减肥中,学生
其他生活
旅游,美食,娱乐,汽车,体育

运动
学生,听歌,平常心,娱乐,优点多多啊!
体育资讯,郑州生活,校园生活,视频音乐,美女
好性格,宅,手机,时尚,旅行,音乐,萌物,猫控,森系,乙女
旅游
娱乐,健康
健康,美食,看书,90后,创意,DIY,處女座
娱乐,艺术,紫色,音乐,90后
娱乐
时尚,美食,娱乐
电脑,简单,健康,旅游,文艺,娱乐,搞笑
体育,足球
IT数码
军事
互联网,电影,摄影爱好者,新闻,幽默,90后,音乐,英语,数码,乐观
旅游
Nichkhun,微博,自由,90后,听歌,宅,美食,动漫,张根硕的鳗鱼,双鱼座
健康
健康,香橙,爬山,茶
喜欢每天的日落
星座命理
军事
生活,自驾,骑行,旅行,摄影,车友,聚会,汽车
时尚
文艺,娱乐,IT数码,电影,港乐,篮球,海贼王
游戏动漫
完美主义,星座命理,娱乐,至上励合,摩羯座
旅游
睡觉,dota,环保,钓鱼,彩妆
美食,新闻资讯,旅游
IT数码,体育资讯,搞笑幽默,汽车
新闻资讯,旅游,游戏动漫
健康,交友
健康,星座命理,IT数码,体育,娱乐
摄影爱好者,街拍,爱,意大利
旅游,星座命理
街拍时尚,杂志控,走秀,大牌控,欧美时尚,明星街拍,明星,娱乐,时尚,文艺
娱乐
时尚
微博奇葩,体育,娱乐
美食
IT数码,美女,游戏,天蝎座,学生,上网,乐观,自由,设计,动漫
娱乐,时尚,听音乐,宅男,自由,天秤座,创业,电子商务,80后,动漫
新闻趣事,视频音乐

娱乐
时尚
星座命理
穿衣美容,美食
体育,游戏动漫
美食,文艺,娱乐
摩羯,吸血鬼日记,80 后,电影,IT,宅男,旅游,篮球,音乐,时尚
游戏动漫
名人明星
娱乐
苏州生活,治愈系,文艺
美食
星座运势,校园生活,吃货一枚,宅,学生,睡觉,动漫,狮子座,音乐,电影
健康,汽车,旅游
娱乐,新闻资讯,文艺,军事,时尚
爱电影,纠结,动漫控,旅游,文艺
美食,娱乐,游戏动漫,美女,体育
美女
时尚,美女,健康,美食,娱乐,宅,吃货
美食,娱乐
汽车
美食
旅行,电力,温州,热爱生活,历史,看书,真实力,思考,爱自由,健康
汽车
无厘头的小疯子,电子发烧友,90 后宅女,吃辣杭州人,闷骚偏执狂,AB 型摩羯座,强迫症女王
唱歌,手机,时尚,浪漫,美食,网购,旅行,90 后
娱乐,教育就业,投资理财
娱乐
星座命理,狮子座
美食
羽毛球,摄影爱好者,娱乐,体育,文艺,IT 数码
旅游,汽车,音乐
娱乐,游戏动漫
衢州生活
军事,游戏动漫,IT 数码,娱乐,美女
美食
美食
视频音乐
旅行,自由,汽车,乐观,平常心,创业,白羊座,80 后
新闻资讯,美食,军事,旅游,星座命理
学生,旅行,听歌,小说,电影,校园,手机,摩羯座
星座命理,美食,体育,二逼怪咖,欣赏诗诗,柯南迷
美食,文艺,体育,汽车,娱乐,无爱情论,半完美主义
美食,文艺,体育,军事

女王,Cynicism,TED,Gintama,日剧,Jogging,Russel
新闻资讯
萌宠
IT 数码,体育,时尚,娱乐
考研,篮球,孩子气,k 歌,爱旅游,音乐,汽车,游戏动漫,体育
自由职业
体育,教育就业
健康
舞蹈爱好者,柔术
娱乐,时尚,健康,星座命理,美食
旅游,娱乐,体育
新闻资讯,娱乐,教育就业,IT 数码,体育
旅游,美女
时尚
学生
IT 数码
美女
没有控!
美食,星座命理,旅游,健康,投资理财
时尚,旅游,美食,娱乐
萌宠,美女,旅游,搞笑幽默
美食
游戏动漫
搞笑幽默
旅行,好书,小说,音乐
健康
星座命理,美食,娱乐
股票,囧,自由
投资理财,旅游,花
军事,体育
动漫
娱乐,美食,90 后,中学生,上网,偶喜欢玩,唱歌,善良,双鱼座
娱乐,好性格,平常心,金牛座
名人明星
游戏动漫
名人明星,娱乐
娱乐
汽车
星座命理,水瓶座
搞笑幽默
时尚,旅游
体育
娱乐
旅游,夜猫子,吃货,天蝎女,辛夷坞,汉服,拉美西斯二世

健康,教育就业,美食,美女,娱乐,历史,网购,阅读,大学
沉默,奋斗,微笑,真诚,低调,强迫症,80後,努力,IT 数码
穿衣美容,摄影,爱时尚,处女座
美食,狮子座 90 后,动漫爱好者一只,羽毛球,宅
星座命理,游戏动漫
广东生活,民工
文艺
旅游
手机,大学,吃,美女,双鱼座,爱情,自由,80 后
旅游,星座命理,娱乐,时尚,美食
IT 数码
读书,白羊座,网络,篮球
美女,教育就业,军事,IT 数码
美食,娱乐
美食
美食,行星团 12 只,底线鹿鹿,女神茜茜
吃货,睡觉,天秤座,音乐,80 后,网购,电影
投资理财,旅游,体育,汽车,美女
健康,投资理财
旅游,最爱浩纸,彩虹糖
旅游,健康
名人明星,体育
娱乐
健康,平常心,美食,偶喜欢玩,网购
遊戲動漫,時尚,轻音乐,养生,摩羯座,设计,沉默,爱唱歌,单身,漫画
旅游,投资理财,星座命理
健康
广东生活
军事,旅游,爱狗,历史,阅读,新闻,IT,80 后,旅行控,摄影
教育就业,汽车,时尚,游戏动漫,IT 数码,吃,偶是懒猪,学生,90 后
文艺
文艺,健康
美食
娱乐,自由职业
临沂生活,IT 数码,旅游,临沂新儒拉丁,新儒拉丁舞,拉丁舞,临沂舞蹈,临沂拉丁舞
90 后
体育
淄博贷款网,无抵押贷款
看电影
贵州生活
娱乐
IT 数码
健康
旅游,娱乐,体育,射手座,篮球,音乐,学生

投资理财,新闻资讯,旅游,健康,文艺
星座命理
旅游
搞笑幽默
美女
广东生活
吃,运动,80后
狮子座,吃货,偶稀饭睡觉觉,大学生,喜欢旅游,爱,韩剧,音樂,90后,爱睡
开朗,汽车,军事,星座命理,体育
旅游,IT数码,美食,80后,吃货,看书,旅行,平常心
视频音乐
体育
微博奇葩
星座命理
植物杀手,强迫症患者,tamaswells,Doraemon,时事,科技,看字,音乐,技术宅
搞笑幽默
听歌,旅游,文学
时尚,健康,美食,星座命理,双重性格魔蝎座
美女,游戏动漫,汽车,美食,体育,吃货,处女座,好性格
自由
体育,IT数码,文艺
军事,IT数码,体育,投资理财,教育就业,美女
投资理财
新闻趣事,囧
旅游,IT数码,金牛座,宅
时尚,教育就业,投资理财,星座命理,旅游
娱乐,文艺,星座命理,美食
微博奇葩,爱情,听歌
体育,篮球爱好者
娱乐
健康,娱乐,体育,旅游
娱乐,汽车,旅游,美食,军事
双子座
新闻资讯,旅游,投资理财,娱乐,健康
汽车,新闻资讯,旅游,军事
新闻资讯,IT数码
健康,星座命理,娱乐,时尚,美食
旅游,爱旅行,学生,设计,吃货,时尚,好性格,生活,90后,金牛座
听歌,NBA
娱乐
睡觉睡到自然醒,武汉纺织大学,哆啦A梦控
90后,动漫宅,技术宅,Google粉,宅男,ACG宅,动画,cosplay
广东生活
体育

汽车,娱乐,体育,文艺,IT 数码
新闻趣事,搞笑幽默,旅游
文艺,吃货一枚,摩羯座,娱乐,美食
军事,IT 数码
美女,时尚,美食
美食
小说,吃,学生,偶稀饭睡觉觉,90 后,手机,动漫,宅,电影,天蝎座
IT 数码,创业
搞笑幽默
娱乐,90 後,各种傻,各种憨,各种无语
游戏动漫
情爱专家,飙车浪女,知性女屌丝
星座命理,文艺
星座命理,投资理财
旅游,健康,文字,听歌,吃,阅读,孤独
美食,吃,交友,90 后
名人明星
时尚,健康,旅游,美食,文艺,美剧,旅行,在中控,90 后,金牛座
美女
恐怖片爱好者,辣妈,母婴,时尚,宝宝
视频音乐,搞笑幽默,白羊座,欧美音乐,篮球
健康,时尚,娱乐,文艺
育儿百科,旅游,八卦杂谈,美容服饰,星座运势
娱乐
搞笑幽默,动漫控,情绪化,音乐控,磁灶中学
文艺
旅游
健康,新闻资讯,美食,汽车,军事
浪漫,紫色,穿衣美容,旅游,上海生活,美食,时尚,汽车,投资理财
美食,高三,小说,水瓶,吃货
听歌,时尚,90 后,音乐笛子口琴
旅游
娱乐,文艺,游戏动漫
新闻资讯,IT 数码,军事,旅游,娱乐
娱乐
娱乐
体育,游戏动漫,娱乐
天蝎座,吃,英语,吃货,玩儿
星座命理,健康
搞笑幽默,新闻资讯
星座命理
96 年,巨蟹座
汽车,摩羯座,90 後
健康,娱乐,文艺,IT 数码,体育

宅,营销,音乐
旅游
IT 数码
美食
旅游,星座命理,美食,娱乐,文艺,玩儿,小说,自由,宅,吃货
文艺,教育就业,健康,星座命理,美食
化妆品,美容护肤,韩剧,休闲,爱吃,时尚潮流,朴有天,跳舞,淘宝控,Music
游戏,搞笑幽默,视频音乐,旅游
八卦杂谈,毅力星人,游戏,技术,萌,宅,偶稀饭睡觉觉
旅游
美食,IT 数码,体育
旅游,美食,娱乐,健康,自由,旅行,吃,宅
医生不是神,游戏达人
江苏生活
时尚,情绪化的小女人,知性美女,偶喜欢玩,善良,处女座
幽默,韩寒,love,seo,心情,红楼梦
投资理财,教育就业,健康,旅游,美食
健康
美食,军事,娱乐,213 美骚年,变态,吃货,偶稀饭睡觉觉,网络,旅游
搞笑幽默,張敬軒 Fans,自行车爱好者,玩,电影,处女座
时尚
美食爱好者,热爱生活,娱乐,吃货
自由,音乐,网购,旅游
游戏动漫,投资理财,旅游,IT 数码,体育
微博奇葩
IT 数码,旅游
星座命理
时尚,娱乐,健康,美食,旅游
时尚
执着,命硬,务农,篮球,创业,好性格,体育
星座命理,娱乐
体育,营销,自由职业,吃,冷笑话,射手座,足球
美食,游戏,上网,体育,网络,白羊座
娱乐
九號路賭場,娛樂,貴賓,温哥华,星光,九號路,賭場,百家樂,牌九,廿一點
婚礼策划,宴会设计,婚礼摄影摄像,婚礼司仪,婚庆花艺,婚礼专家,杭州,花逸良品,婚礼顾问
哈哈哈哈哈哈哈
连接服务器失败,闷骚,迷糊,睡觉,懒,吃货一枚,小说,唱歌,宅女,游戏动漫
娱乐,幽默,听歌
新闻资讯
新闻资讯,投资理财
文艺
娱乐,文艺,美食,唱歌,旅行,80 后
娱乐,玩游戏,看电影,逛街

上海生活
童装,淘宝卖家,母婴
汽车,健康,时尚,娱乐,星座命理
名人明星
婚礼策划,执行,创新,独一无二,旅游
美食,旅游,健康,美女,时尚
搞笑幽默
美食
游戏动漫,文艺
游戏动漫
星座命理,文艺
文学,历史,国学,经济,传媒,移动互联网,社会观察员,新闻评论家,理想主义者,中国公民
白羊座,追星,娛樂
星座命理,游戏动漫,健康,时尚,文艺,娱乐
善良,温柔体贴,双鱼座,温文儒雅,成熟稳重
健康,体育,娱乐
汽车,健康,旅游
旅游,星座命理,时尚,美食,娱乐
文艺
时尚
投资理财
美食
美食
时尚
旅游,星座命理,美食,体育,娱乐
时尚,军事,新闻资讯,健康,旅游
健康,旅游,时尚,体育,娱乐
旅游,美食,文艺
乐天派,听歌,爱动物,平常心,时尚,浪漫,好性格
新闻趣事,金牛座,宅女,偶稀饭睡觉觉,看书,平常心,时尚,电影
美女,IT 数码
时尚
视频音乐,娱乐
美女
时尚,时尚达人,电脑,逛街,金牛,品牌,shopping,美容护肤
视频音乐,新闻趣事,美食
游戏动漫,金牛座,动漫,游戏,时尚
星座命理,游戏动漫,摩羯座,宇智波鼬,动漫,音乐
旅游
傻 B，疯子
娱乐,健康,旅游,星座命理,美食
汽车
电影控,艺术,听音乐,梦想
星座命理

穿衣美容
星座命理
旅游
时尚
娱乐
傻不啦叽,旅行,成长 ing,追求梦想,爱音乐,爱旅游美食摄影,爱生活,90 后
军事,健康
美女
文艺
文艺,教育就业,健康,美食,娱乐
娱乐
搞笑幽默
文艺,旅游,美食,自由,90 后,宅,双子座,小说
TVB,汽车,时尚,IT 数码,体育
新闻资讯,文艺
微博奇葩,旅游
巨蟹座
美食
射手座,爱笑
搞笑幽默,旅游,美女,穿衣美容,宝鸡生活
旅游,星座命理,美食,文艺,娱乐
设计师,体育,星座命理,娱乐,时尚,美女
文艺,80 后,宅,摩羯
星座命理
旅游,美食,健康,时尚
娱乐,美食
我就是我,舞蹈,微笑,情感,搞怪,书籍,活泼开朗,美术,篮球爱好者
搞笑幽默
时尚,星座命理,旅游,文艺,娱乐
电影,文字,浪漫,摄影
娱乐
文艺
汽车,时尚,IT 数码,新闻资讯,星座命理,旅游,体育
70 后,电器迷,音乐控
典型的射手座
旅游
外贸业务员
好书,汽车,互联网
奋斗
旅游
旅游
旅游
娱乐,文艺,吃,语录,自由,旅行
名人明星,美食,新闻趣事,旅游,郑州生活

美图摄影,读书分享,美食,视频音乐,名人明星
健康,旅游
星座命理,投资理财
偶稀饭睡觉觉,吃货,90后,宅女
教育就业,美女,IT数码
古风,音乐,篮球,茶
视频音乐
视频音乐,微博奇葩,名人明星
美食
美食,体育,文艺,时尚,IT数码
电影,篮球,唱歌,上网,旅行,NBA,游戏,交友,百善孝为先,众情义为重
生活,时尚,摄影,财经,旅游,音乐,自由职业,天蝎座,网络,文学
新闻趣事
体育
网络营销
交友,自由,幽默,营销,搞笑
文艺,星座命理,时尚,娱乐
Seed空间,雕塑,80后,艺术,水墨,油画
IT数码,90后,摄影爱好者,网购,旅行,听歌
美食,美女,视频音乐
双鱼座
留学,死神,傻,流浪
时尚潮流,咖啡,文字控,吃货一枚,喜欢听歌,爱电影,旅游
穿衣美容
美女
美女,游戏动漫,汽车,军事
娱乐,时尚,健康,旅游,文艺
狮子座女生,80后
美食,汽车
旅游
时尚,美食,学生一个,巨爱权志龙,帅哥控,韩娱控,巨蟹女,懒人,各种玩儿,宅女猪猪一只
娱乐
新闻资讯
音乐
搞笑幽默
发呆,抽风,自娱自乐,文字,历史,文学,旅行,NBA,读书
名人明星,时尚,广告,媒体,艺术,听歌,宅女,咨询,最新动态,绯闻
二货
名人明星
cosplay,散步,偶像剧,摄影师,music,旅游摄影,美食爱好者,处女座,平常心,艺术
坚持自己的原则,KKKk歌，麦霸,不算是腐女,想去旅行,喜欢大狗狗,梦想去普罗旺斯,铁塔控,美食控,金牛座
音乐爱好者,快乐,低调,喜欢旅游,强迫症,无厘头
美女

天蝎座
盐城生活
游戏动漫,美食
美食,文艺,娱乐
动漫,听歌,校园
名人明星,美食,旅游
游戏动漫,时尚,IT 数码
玉溪生活
星座运势,美食,视频音乐,搞笑幽默
娱乐,美食,旅游
偶稀饭睡觉觉
娱乐,体育
娱乐
星座命理,美女,旅游
体育
娱乐,新闻资讯,时尚,健康,文艺,游戏动漫,星座命理
星座命理,美食,文艺,娱乐,旅游
时尚,美女
军事,旅游
宅男,天秤座,游戏,90 后
执着,宅到不能宅,文字控,电脑迷
教育就业
时尚
新闻资讯,健康,美食,小提琴,钢琴,时尚,旅游
微博奇葩,美食,星座命理,搞笑幽默,新闻趣事
网购,明星,公益,许嵩,魔兽,摩羯女,红楼梦
睡觉,善良,上网,爱情,吃货
设计,幽默,購物控
娱乐
上网,羽毛球,旅行,微博,摄影,金牛座
健身,淡定,跑步,怀旧,自由,睡觉,星座控,新闻趣事
美食
新闻资讯
娱乐
游戏动漫,娱乐
美食
时尚,新闻资讯,旅游,健康,娱乐
投资理财
服装,电影控,音乐爱好者,完美主义,幸福
帅哥,exo,睡觉,白羊座,唱歌,紫色,90 后,音乐,吃,上网
星座命理,旅游,美食,游戏动漫,健康,吃货,巨蟹座
IT 数码
动漫
旅游

星座命理
美食,我爱他,喜欢笑,学生,喜欢吃好吃的,敏感
健康
时尚,吃货,白羊座,旅游,80后,电影,摩羯座,听歌
体育
睡觉睡到自然醒,学生,新闻资讯,军事
军事
文艺,健康,星座命理,时尚,娱乐
腐女一枚
IT数码,文艺,时尚,汽车,美女,轻轻女子,清清心
偶喜欢玩,水瓶座,连接服务器失败,文字控,吃货一枚,懒,music,无厘头,偶稀饭睡觉觉
房产
星座命理,美食,00后,学生一族,张根硕控,自由
IT数码,日語,双眼geass,梶浦由纪
IT数码,时尚,文艺
星座命理
时尚
宠物图集,星座运势,星座命理
登山,音乐,小说,篮球
时尚
美食
星座命理,校园,搞笑,动漫,金牛座,善良,学生
小宇宙强大
八卦,90后,生活,艺术,历史,80后,校园,浪漫,文学,幽默
美女,IT数码,文艺,体育
星座命理
成都生活
娱乐,时尚,星座命理
登山,管理,nba,爱旅行,随性,军事,美女,体育,投资理财,旅游
厦门生活,搞笑幽默,坚强,原创,完美主义,真实力
北京生活
大夫,金牛座,财经,旅行,美剧,紫色,听歌,自由,偶稀饭睡觉觉
河北生活
汽车,睡觉,娱乐,时尚,音乐,幽默,八卦
唱k,吸血鬼,化妆,音乐达人
投资理财,摩羯座,旅游,时尚,音乐
新闻资讯
名人明星
健康,游戏动漫,星座命理,美食,娱乐,手机看小说,宅女一枚
游戏动漫,美女,军事,IT数码,娱乐
时尚,健康,投资理财,旅游,美食
教育就业,健康,旅游,美食,文艺
校园,教育,育儿,爱,香橙,林俊杰,80后宅女,苹果控,睡懒觉,志愿者
宅,摩羯座,90后,吃货

新闻资讯,美女,旅游,星座命理,军事
文艺,新闻资讯,娱乐,IT 数码,星座命理,旅游,美女,教育就业,军事
学生一枚,活泼,青春,画画,开朗
健康
美食
娱乐
参禅,已婚人士
时尚,偶稀饭睡觉觉,天秤座,吃货,囧,宅,旅游,90 后
旅游,美食,时尚,娱乐
旅游,傻婆,校园,平常心
文艺,淘宝店主,自由,乐观,80 后
何琦琦,定时炸弹,人来疯一枚
文艺
旅游,健康,星座命理,投资理财,新闻资讯
美食
搞笑幽默
健康
视频音乐,体育,音乐篮球
搞笑幽默
娱乐
培训,养身,旅游
娱乐,时尚,投资理财
体育
旅游,娱乐,时尚,执着,陈奕迅 Eason,听歌
认识帅哥美女,好性格,听歌
新闻资讯,旅游,健康,美食
唱歌,处女座
文艺,娱乐,星座命理
搞笑幽默,营销
娱乐,吃货,乐观,巨蟹座,宅,时尚
美食,幽默,80 后
校园生活,视频音乐,星座运势,搞笑幽默,美图摄影
减肥,天蝎,柯南,摩羯,gossipgirl,广州人,金牛
背包客,摩羯座
微博奇葩,体育
微博,交友,苗迷,90 后,自由
最新,实拍,幽默搞笑,新闻,奥运,网罗天下,90 后,时尚,美食,80 后
文艺,军事,美食,教育就业
电脑,天枰座,武艺,文化,旅游美食爱好者,资讯
重庆生活,处女座,摄影爱好者,生活,旅行
娱乐
投资理财
健康
时尚,娱乐,教育就业,文艺,星座命理

读书分享
幽默,音乐,阅读,吃货,旅游,网络,天蝎座,紫色,乐观,睡觉
健康,旅游
文藝,美食,旅遊,娛樂,大野智,SS 山组最高,翔智,樱井翔,SS 山組,大野智 ohno
闷骚,90 后非脑残,金牛座,懒人,爱唱歌,打篮球,体育
汽车,文艺,娱乐,体育,美女
美女,健康,文艺,狮子座,上网,90 后,小说,宅,好书,吃货
旅游
90 后,音乐,交友,善良
星座命理,美食,健康
旅游
旅游
星座命理
名人明星
新闻资讯
美女
健康
新闻资讯
新闻资讯
游戏动漫,时尚,美女,旅游,娱乐
美女
时尚
投资理财,教育就业,汽车,IT 数码
沉默,矛盾,养生,生活,心情,家居,发呆达人,宅女加腐女,胡思乱想,旅游美食
娱乐
时尚,美食
美食,摩羯座,浪漫,英语,乐观
娱乐
时尚
时尚,美食
时尚,文艺,美食,星座命理,娱乐
幻想,学习,90 後
宿迁团市委书记
美女
IT 人,电视迷,时尚达人,笑话控
娱乐
美食
旅游,搞笑幽默,星座命理,穿衣美容,重庆生活
女人,遊戲動漫
新闻资讯
礼品,定制,团购,骨瓷,贵金属,藏品,游泳
双子座,学生
星座命理
娱乐

新闻资讯,时尚,教育就业,娱乐
娱乐
旅游
其他生活
上网,时尚,偶稀饭睡觉觉,宅女,游戏,旅游,八卦
时尚,乐观,美食,旅游,玩儿,巨蟹座,吃货,电影
汽车
健康养生,新闻趣事,旅游,美食
新闻资讯,娱乐,旅游
旅游美食,温柔,影视,旅游爱好者,经济,诗词,软件,社会,登山
游戏动漫
新闻资讯,旅游
娱乐,IT 数码,美食,旅游
汽车,体育
时尚
美女
星座命理
新闻资讯
综艺,音乐
娱乐
健康,星座命理
手机,双鱼座,听歌,生活,动漫,时尚,90 后,上网,电影,美食
穿衣美容,音乐,90 后,乐观,看书
文艺,投资理财,旅游
音乐,书,电影
小说,电影,80 后,汽车,偶稀饭睡觉觉,交友,摩羯座,听歌,美食,旅游
健康,文艺,军事,旅游,娱乐
时尚,美食,健康,娱乐
时尚
dota,基督徒,潮人,天平座,散步
游戏动漫
体育,时尚
音乐,善良,时尚,搞笑,微博,好书,网络,睡觉,娱乐,90 后
宠物图集
美食,汽车,旅游
浪漫,小说,学生,旅行
读书分享,搞笑幽默,文艺
爱情,善良,生活,乐观,美食
萌宠,名人明星,微博奇葩,搞笑幽默,穿衣美容
旅游,健康,美食,娱乐
搞笑幽默
娱乐
名人明星,旅游,美食,穿衣美容,运城生活,偶稀饭睡觉觉
军事

职业 HR,健身爱好者,双鱼,摄影,O 型血
时尚,音乐,睡觉,电影,娱乐,英语,文字,偶喜欢玩,90 后,听歌
文艺,娱乐,旅游,自由,旅行,好书,吃,90 后
娱乐
文艺
娱乐
蒋劲夫,生物工程,美食
娱乐,偶稀饭睡觉觉
军事,IT 数码
投资理财
汽车,电子商务,搞笑,偶稀饭睡觉觉,宅男,旅行
教育就业,星座命理,娱乐
C 罗控,金牛座,托福考试准备中,粉色控玫红控,活泼可爱的疯子,90 后,爱车族,爱车爱玩爱
音乐,运动,爱狗一族
娱乐,时尚,美食
体育
情感生活,简单,爱心
起床困难户,體育,囧,宅男
军事,星座命理,旅游,文艺,游戏动漫,美食
旅游,新娘,wedding,浪漫,婚礼
音乐
美食,星座命理,投资理财,睡懒觉
旅游,投资理财,汽车,IT 数码,娱乐
教育就业,健康,新闻资讯,旅游,文艺
互粉,爱电影爱旅行,学生,选择恐惧症患者,自恋狂,爱帅哥,萝莉,无厘头
IT 数码,无产阶级伪小资,唯恐天下不乱
搞笑,乐观,幽默,吃,健康,投资理财
萌宠,小白
美女,视频音乐,新闻趣事,搞笑幽默,微博奇葩
情感生活,爱情,唯美,精选,语录,热门,90 后,80 后,心语,情感
音乐
娱乐
二青年,运动范儿
视频音乐,多想
乐观,浪漫,美食,听歌,看书,玩儿,时尚,旅游,双子座
瘦身,大学生,偶稀饭睡觉觉,笑,自然,文化,健康
微博奇葩,娱乐
旅游,健康,偶稀饭睡觉觉,90 后,平常心,好性格
新闻资讯,美女
文艺,旅游,摄影,美食,语录,旅行,宅,微博,天蝎座,90 后
旅游
旅游,美食,小工作狂,爱好自由
购物狂,美剧控,学生一枚,文藝,星座命理,娄艺潇迷,爱。张根硕,双子座
新闻资讯

文艺
河南生活,穿衣美容,睡觉
文艺,时尚,星座命理,美食,娱乐
穿衣美容,美女,搞笑幽默,旅游,哈尔滨生活
美女
文艺
宅女
视频音乐
穿衣美容
旅游
美女,游戏动漫,星座命理,体育
健康,文艺,娱乐,时尚,美女
文藝,摄影爱好者,执着,狮子女,媒体人,神经质
巴萨,曲怀觞,岁月推理,温瑞安,不见荷,崎路人,剑子本命,荣迷,白羊座
IT 数码
文艺,旅游,游戏动漫,汽车,屌丝
娱乐
时尚,游戏动漫,美女
娱乐,游戏动漫
萌宠,名人明星,穿衣美容,星座命理,微博奇葩
名人明星
旅游
投资理财
视频音乐,军事,健康,IT 数码
搞笑幽默,旅游,美女,视频音乐,微博奇葩
喜欢 JJ
唱歌,电影,音乐,90 后,旅行,游戏
交友,宅男,电影,摩羯座,90 后
健康,娱乐,星座命理,美食,美女
旅游
IT 数码,汽车,投资理财,娱乐,体育
游戏动漫
吃货,笨女人,小文艺,旅游,听歌,英语,电影
娱乐
江苏生活,搞笑幽默
投资理财,旅游,娱乐
旅游
搞笑幽默,小小烦恼控丶
娱乐,星座命理,文艺,体育,新闻资讯,美女
星座命理,八零后
时尚,美女
美食,旅游,穿衣美容,其他生活
教育就业
重庆生活,娱乐

投资理财
体育,时尚,娱乐
教育就业,南山大学,志愿填报,专业介绍,高考分数线,招生咨询,烟台南山学院
名人明星,80 后,互联网,广告,社媒营销专家
星座命理
美食,80 後,美剧控
微博,善良,网络,好性格,宅女,白羊座,时尚,平常心,娱乐,上网
时尚,旅游,健康,新闻资讯,美食
微博奇葩,美剧控,闷骚,街拍,强迫症,音乐控,少女时代
理财,投资理财,旅游,美剧,时尚,音乐,财经,汽车,语录
爱玩,星座命理
电影,语言,篮球,自由,生物,化学,美剧,乐观,文学,足球
汽车,美女,无聊
健康,旅游,美食,文艺,星座命理
北京生活,微电影,话剧,影视戏剧,李振彬,李振彬工作室
美女,玩儿,幽默,唱歌,听歌
聊天,电影迷,90 后非脑残,拍照,迷糊,潮流,笑话
美容服饰,旅游,宠物图集,新闻趣事,美食,电影
重庆生活,旅游,酒店业
健康,汽车,星座命理,娱乐
时尚,健康,娱乐,美食
旅游
美食
直接,大大咧咧,喜欢看帅哥,玩,听歌,学生
活泼开朗,篮球爱好者,笑,青春,上网,吃货,篮球,射手座,星座命理,娱乐
健康,新闻资讯,投资理财,旅游,美食
动漫,周杰伦,双鱼座
重庆生活
时尚,娱乐,体育,IT 数码,汽车
旅游,时尚,汽车,文艺,娱乐,男士时尚资讯,女士时尚资讯
娱乐
娱乐
投资理财,娱乐,新闻资讯,旅游,美食
美食,健康
健康,个性,胡思乱想
旅游
新闻资讯,旅游,娱乐,美女,投资理财
90 后,动漫,学生,听歌,钢琴演奏,长笛 player,古筝达人,善良,紫色,亲情
名人明星,体育
傻真系番禺之耻
我爱的人不爱我
深圳生活,汽车,善良,手机,历史
汽车,美食,文艺,新闻资讯,教育就业,军事
化妆,天枰座

汽车,创业,营销,旅行,小说,文学,80 后,美食,电影,互联网
投资理财,英语,穿衣,时尚
时尚,美食
健康,娱乐,美食,美女,IT 数码
文艺,IT 数码,投资理财,健康,旅游,军事,新闻资讯,教育就业
校园生活
教育就业,星座命理
旅游,文艺,时尚,美食,IT 数码,体育,美食
健康,汽车,体育,投资理财
体育,美女
IT 数码
时尚,美食,新闻资讯,健康,旅游,文艺,娱乐
搞笑幽默,爱拍照,萌,吃货一枚,90 后但我不脑残,双鱼座
IT 数码
旅游,IT 数码
搞笑幽默
娱乐
IT 数码,国防部,国家安全,科学研发,信息安全,安全顾问,网络安全
军事,娱乐,健康,美女,投资理财
投资理财,美女,IT 数码,娱乐,吃喝玩乐
巨蟹座,吃货,学生,紫色
开朗,爱漂亮,爱唱歌，创业者
文艺
美女,军事
IT 数码,搞笑,足球,上网,音乐,旅行
时尚
旅游,文艺,时尚,水瓶座
军事
屌丝,处女座
电影,电视,导演,编剧,剧情,纪录片
旅游,星座命理,美食,新闻资讯,娱乐
双鱼座
爱玩,美食爱好者,做梦,我就是我,music,射手女,学习,爱睡觉,阳光,美食
旅游
体育,IT 数码
娱乐,写作,睡觉,校园,音乐,善良,听歌,90 后
旅游,游戏动漫,美食
真诚,旅游美食,乐观,低调,娱乐,健康,新闻资讯
娱乐
动漫
娱乐,健康,时尚
健康,旅游,星座命理,IT 数码
穿衣美容,时尚
旅游,美女,投资理财

旅游
创业是我的梦想,囧,浪漫,旅行,生活,幽默,玩儿,爱情
时尚,汽车,体育,吃,上网,唱歌,打篮球,看足球,收球鞋,写东西
天蝎座,手机控,有点小忧郁,有点小叛逆,偶像剧迷,只听好歌
娱乐
文艺
成都生活,娱乐
时尚
乡土,村儿,城乡结合部范儿
军事,IT 数码,投资理财,娱乐,健康,教育就业,新闻资讯,旅游,星座命理
新闻资讯,美女,军事,体育,娱乐
美女
美食
娱乐,旅游
搞笑幽默
搞笑幽默,穿衣美容,教育就业
星座命理
恐怖片,猫控,听歌,A 型血,心理,电影迷,爱幻想,好书,散步,摩羯
旅游,星座命理,搞笑幽默,新闻趣事,视频音乐
旅游,运动
美食,爱笑,蓝色,看书
游戏动漫
健康
新闻,美女,期货配资公司,期货配资,金融投资,金融,期货开户
娱乐
双子,体育,军事,LOL,音乐、歌曲、DOTA,球,睡觉,宅
娱乐
密集恐惧症,强迫症偏执狂,水瓶女,爱旅行,美剧,搞笑,吃货,原创,神经质,努力
吃货一枚,重口味,动漫控,外貌协会,耽美,宅女加腐女,懒人
高中生
体育资讯
专卖店,旅行,摄影爱好者
八卦杂谈,IT 数码,新闻趣事,搞笑幽默,美女
名人明星
投资理财,美食,健康,新闻资讯,旅游,乐观,吃,射手座,平常心
新闻资讯,美女,美食
好看的衣服,好吃的食物,青涩的初恋,真心的友谊
投资理财,美女,旅游,咖啡控,天蝎女,睡觉睡到自然醒,爱自由,B 型血,我就是我
投资理财,新闻资讯,时尚,旅游,美食
健康,宅,音乐,80 后,白羊座
穿衣美容,娱乐
体育,美女
娱乐
美食,娱乐

美食,旅游,新闻资讯,时尚,教育就业,娱乐
南京生活,穿衣美容,偶喜欢玩,爱情,上网,白羊座
微博奇葩,视频音乐,广东生活,美食,旅游,吃喝玩乐,吉他,爱生活,志愿者,市场营销
天秤座,学生,奋斗 ing,开朗
军事,美食,星座命理,旅游
唱歌,艺术,青春,投资理财,健康,文艺
娱乐
IT 数码
体育
文艺
美女
旅游
时尚,美食,健康
美食,军事,旅游,健康,新闻资讯
IT 数码
娱乐,星座命理,美食,文艺
娱乐
旅游,星座命理,美食
健康
体育资讯,投资理财
IT 数码
美女
新闻资讯
初三党,娱乐
教育就业
美女,新闻趣事,IT 数码
八卦杂谈
美女
IT 数码,军事,美食,星座命理
健康,娱乐
美食,IT 数码,娱乐
情感生活,健康,美女,美食,星座命理
笑话,军事,投资理财,健康,IT 数码
美女
微博奇葩,穿衣美容,新闻趣事,幽默,摄影,美食
DIY 配珠,佛头,手串,蜜腊,小叶檀,菩提子,佛教
娱乐,健康
美食
文艺,健康,旅游,教育就业,投资理财,星座命理
名人明星
旅游
新闻资讯
娱乐,时尚
健康,新闻资讯,时尚,星座命理,美食

美食,旅游,军事,IT 数码
美女,体育资讯,校园生活,合肥生活
体育
旅游,笑,安静,音乐,文字,90 后,狮子座,大学生,英语,旅行
美食
旅游
娱乐,星座命理,love,户外,蓝色,梦想,时尚
IT 数码,交友,我就是我,宅,学生,宅男,连接服务器失败,奋斗,学习
时尚,紫色,汽车
微博奇葩
美女
旅游,汽车,IT 数码
时尚,文艺,娱乐,美食,Music,80 后宅女,低调,爱旅游,独立,爱美食
笑,梁静茹,梦想,化妆,紫色,赚钱,手机,电脑,汽车,旅游
读书分享,校园生活,情感生活,新闻趣事,搞笑幽默
穿衣美容
星座命理,路痴,迷糊,200 的领导人,小盆友碾压者
衬衫控,耽美控,动漫控,花痴,双子座,外貌协会,爱吃,娱乐,文艺,旅游
美食,IT 数码,视频音乐,美女,体育
娱乐
影视,手机看小说,90 後,爱旅游,睡觉睡到自然醒,乐观,梦想,上网,活泼开朗,偶像剧
星座命理,体育,娱乐,汽车
处女座 O 型,自由,篮球,足球,唱歌,音乐,历史
各种坑,摩羯座,80 后,发呆
吃货一枚,拍照,K 歌,学生一族,笑话,韩剧,服装,电影控,爱生活,电视剧
时尚
美女,新闻资讯,游戏动漫,健康,娱乐,IT 数码,文艺,体育
文字控,内裤控,应该不傲娇,文艺小青年,受受,男同
動漫,金牛座,学习中,耽美控
搞笑幽默,穿衣美容,新闻趣事,重庆生活
娱乐,游戏动漫,军事,IT 数码,体育,喜欢听歌,网游
吃货,手机,宅女,狮子座,90 后,上网,睡觉,电影
游戏动漫
体育,健康,汽车,IT 数码,娱乐
诗歌,心情,美食,美女,羽毛球,宅男,读书,旅游,摄影爱好者,听歌
余杭,双鱼座
模特,唱歌跳舞,WOW,体育
娱乐
军事,娱乐,体育
旅游
视频音乐,微博奇葩,新闻趣事,旅游
旅游,健康,时尚,娱乐,美食
美食
新闻资讯,时尚,旅游

文艺
时尚,文艺,美食,星座命理,娱乐
美食
语录,旅游
四川生活,微博奇葩,新闻趣事,搞笑幽默,IT 数码,游戏,搞笑,好书,双子座,宅
微博奇葩
旅游,美食
汽车
旅游,健康,星座命理,美食,娱乐
校园生活
健康,新闻资讯,文艺,旅游,汽车
听歌,阅读,白羊座
射手座
美女,湖北生活
吃,音乐
偶稀饭睡觉觉,EXO 迷,天蝎座,90 后,偶喜欢玩,学生,吃货,听歌,囧,音乐
旅游
盐城,医疗
旅游
新闻资讯,军事,美女,游戏动漫,体育
游戏动漫,美女
唱歌,睡觉
视频音乐,动漫,美图摄影
时尚
游戏动漫
旅游,阅读,娱乐,动漫,广告,美食,设计
星座命理
汽车,射手座,射手
娱乐
娱乐,旅游,健康,美食,文艺
IT 数码
美食
娱乐
娱乐
搞笑幽默,名人明星
新闻趣事,视频音乐,教育就业,投资理财,时尚,旅游,娱乐
娱乐,文艺,星座命理
新闻资讯
淡定,美食,健康,星座命理
娱乐
IT 数码
星座命理
体育,你就是我的
投资理财,健康

美食,旅游
美食,文艺,健康,IT 数码,体育
爱幻想,睡懒觉,爱吃,快乐
Python 培训,Mysql 培训,Oracle 培训,html5 培训,php 培训
曹楠杍,音乐,处女座
新闻资讯,时尚,教育就业,投资理财,找工作,人力资源,apple,iphone,路桥,快乐
新闻资讯
穿衣美容
爱电影,爱旅游,体育
游戏动漫
时尚,娱乐,美食
文艺,读书,旅行,90 后,双鱼座,NBA,幽默,阅读,游戏,运动
教育就业,旅游,时尚,美食,娱乐
文艺,美食,旅游,二逼青年欢乐多
美食,动漫控,文字迷
IT 数码,娱乐,星座命理,时尚,八卦,宅女,绘画,小酒窝,白羊座,摄影
简单,爱生活,喜欢音乐
美食,好书,双子座,摄影爱好者,音乐
简单,音乐控,市场营销,考研辅导,出国留学
视频音乐
健康
双鱼座,90 后,大学
旅游,健身达人,饮料狂,购物狂,水果狂,烹饪达人
美食
摩羯座,大学生,90 后,宅
旅游
浪漫,读书,网购,乐观,听歌,美食,网络,天蝎座,旅游,时尚
旅游,动漫,校园生活,美食
文艺,娱乐,星座命理
文艺,美食,健康,娱乐,汽车,旅游,星座命理
旅游
猫咪,发呆,腐女,臭美,随性,爱睡觉,神经质,睡懒觉,沉默,好性格
教育就业,体育,娱乐
文艺,新闻资讯,健康,旅游,星座命理,美食
娱乐
旅游,文艺,娱乐,体育,军事
帅哥,吉林,电影,自尊心,旅游,义乌,写作
旅游,体育
旅游
时尚
娱乐
搞笑幽默,游戏动漫
搞笑幽默
音乐

搞笑幽默,linux,网站,站长
娱乐
投资理财
文艺,游戏动漫,IT 数码,投资理财
健康,幸福,巨蟹座,喜欢听歌,拍照,90 后,诗歌,音乐迷,杨丞琳
视频音乐
美食
名人明星
汽车,健康,美女,星座命理,军事
娱乐
健康,文艺,时尚,娱乐,星座命理
健康
穿衣美容,健康
TFboys,凯源,牛桃,入江夫妇,troublemaker
娱乐
游戏动漫,文艺,美食,IT 数码,旅游
营销,电影,85 后天蝎,足球达人,阅读
电影控,视频音乐,江苏生活
体育
上海生活
学生,互联网
美食
微博奇葩,文艺
星座命理,健康,文艺,娱乐
娱乐,游戏动漫,星座命理,IT 数码
美剧,舞蹈,天秤座,旅行
美食,旅游,健康,时尚,文艺
搞笑幽默
新闻趣事,自由,唱歌,足球,好性格,白羊座,善良,交友,音乐,电影
旅游,时尚,健康,军事
旅游,健康,汽车,时尚,娱乐
微博互粉,微博控,有点胸,有点凶
90 後,羽毛球,自由,交友,学生,摄影,天秤座,音乐
搞笑幽默,手机,大学生,听歌,宅男,90 后,文字,时尚,看书,音乐
IT 数码,汽车,星座命理,娱乐,游戏动漫,旅游
IT 数码,设计,羽毛球,摄影爱好者
IT 数码,军事,汽车,美女
健康,投资理财,时尚,旅游,美食
旅游,健康,教育就业,投资理财,80 后,PPT,演讲,互联网,设计
搞笑幽默,视频音乐,名人明星,旅游,星座命理
时尚,娱乐
穿衣美容
时尚,健康,星座命理,旅游,美食
旅游,时尚,星座命理,美食,娱乐,彩色控,Kitty 控,水瓶座

喜欢旅游,美食爱好者
时尚,娱乐,体育,美女,水瓶座,品牌,90 後,NBA,手机,摄影爱好者
旅游,汽车,美食
旅游
投资理财
旅游
重口味
美食,IT 数码
娱乐,星座命理
美女
投资理财,健康,美食,旅游
搞笑幽默
娱乐
体育,娱乐,文艺
美食,美食
娱乐
美食
汽车,新闻资讯,IT 数码
新闻趣事,星座命理,美女,穿衣美容,旅游
美女,娱乐
健康,旅游
时尚,娱乐
时尚,游戏动漫,文艺,美食
狮子座,动漫,摄影,听歌,读书,美食,旅游,电影
美食
名人明星,旅游,星座运势,视频音乐
旅游,文艺
微博奇葩,不会想起
北京生活
旅游,美食,娱乐,星座命理
时尚
开心
新闻资讯,星座命理,时尚,娱乐
新闻资讯
沉默,爬山
体育,旅游
旅游,美食,军事,体育,娱乐
美食
旅游,体育资讯
健康,美女,游戏动漫,娱乐,金牛座
视频音乐,萌宠
双鱼座,爱幻想,90 后但我不脑残,旅游,伪文艺女青年,宠物爱好者
唱歌,90 後,听歌,浪漫,运动,学生,网络,旅行,射手座
星座命理

视频音乐,体育
80 后,文字,音乐,旅行,媒体,唱歌,公务员,娱乐,小说,电影
文艺
军事,健康,平常心,吃货,幽默,天蝎座,篮球,上网,80 后,NBA
舞蹈,美容,手机看小说,宅女一枚,巨蟹,shinee,记者
游戏动漫,爱宝贝,双鱼男
教育就业,火柴人,生命赢家
娱乐
美女
电脑,懒,美食控,漫画,shopping,游戏动漫,神经大条,看小说,美食
旅游
萌宠
美食,旅游,健康,时尚,娱乐
搞笑幽默,南京生活
美女
军事,星座命理
旅游,星座命理,美食,军事,IT 数码,文艺
萌宠,搞笑幽默,穿衣美容,视频音乐,星座命理
波尔多红酒
孤独者,写作,文艺控,阅读,自由,吃,好书
美食
旅游,体育
时尚
游戏动漫,IT 数码,体育,旅游,汽车
搞笑,交友,美剧,英语,吃,电影,宅,娱乐,乐观,读书
狗哥小白愛上癮,伏見癡漢超有愛,室長尊哥誰小受,墨明棋妙粉,河图大人是王道,古风混金屬,五月天是底線,尊哥室長誰小受
教育就业,时尚,美食,旅游,星座命理,娱乐
旅游
教育就业,军事,文艺,IT 数码,健康,美女,投资理财
游戏动漫,火影,海贼王,那朵花,动漫,DNF
星座命理,健康,时尚,娱乐
教育就业
漫画,插画,涂鸦,旅游,文艺,游戏动漫
星座命理,旅游,微博奇葩,搞笑幽默,视频音乐
名人明星
天蝎座,篮球,美食,数码
健康
搞笑幽默
新闻趣事
文艺,娱乐,创意设计,电影,摄影
汽车,打球,军事,我命由我不由天
时尚,文艺,旅游,体育,娱乐
健康,汽车,旅游,新闻资讯

汽车,星座命理,IT 数码,多愁善感,怀旧,听歌,游戏,90 后,唱歌,上网
美女,旅游美食爱好者,死神,萝莉控,历史,柯南,街舞,时尚达人,美食控,时装
军事
新闻资讯,星座命理,游戏动漫,教育就业,投资理财
军事
旅游
文艺,美食,健康,时尚,音乐,吃,旅游,电影,80 后,白羊座
体育资讯,体育
娱乐
广州,牺牲奉献,帅哥,金牛座,篮球,成都
喜欢听歌,爱幻想,听音乐
江西生活,情感生活,IT 数码,美容服饰,旅游
游戏动漫,美食,体育,莼,大爱 NBA,LOL,偶稀饭睡觉觉
体育
四川生活
美女
穿衣美容
吃货,宅女,巧克力,美食
搞笑幽默,文艺
文艺,原宿系,森林系
萌宠
美女
90 后,听歌,好性格,网购,搞笑,自由,睡觉
胡思乱想,夜猫子,吃货
投资理财
星座命理
娱乐
美食
独立,美食,旅游,新疆生活,娱乐,时尚,健康
汽车,游戏动漫,美女,旅游,巨蟹座,听歌
时尚,娱乐,90 后
羽毛球
星座命理
TJ,微博,听歌,体育
美食,游戏动漫,健康,军事,体育,篮球,写作,时尚
时尚,汽车,教育就业
娱乐,军事
网店装修,网店货源,网店店主,小说,新闻,交友,游戏,IT,大学生,创业
学生一族
健康,旅游,美食,军事,体育,足球
美食
娱乐,偶喜欢玩,文艺
上网,历史,巴萨,创意,服饰,笑话,爱电影,视频音乐
车世界,美女

时尚
游戏动漫,汽车,IT 数码,体育,吃货,旅游
娱乐
文艺,美食,周恩来忠实粉,狮子座,数学恐惧症,伪文艺青年
美食,汽车,美女,时尚,文艺
旅游,IT 数码,文艺,体育,娱乐
体育,娱乐
健康,时尚,文艺,娱乐,学生,好性格,玩儿
亲情,营销,电影,上网,电子商务,阅读,听歌,好性格,宅,处女座
耽美
洋装,叔控,bjd,LO 娘,娃妈
娱乐
旅游
IT 数码
名人明星,搞笑幽默,新闻资讯,游戏动漫,文艺
加拿大生活
美食
搞笑幽默
旅游,看书,发呆
娱乐,时尚
语录,上网,自由
视频音乐,美食,旅游,搞笑幽默
游戏动漫
娱乐
体育,我喜欢篮球,风流倜傥,花见花开,车见爆胎,人见发财,EXO 组合
新闻资讯,投资理财,教育就业,体育,娱乐
美食
美食,旅游,健康,新闻资讯,星座命理
篮球
汽车
教育就业
善良,摩羯座,音乐,看书,电影,自由,篮球,幽默,90 后,NBA
粤语,时尚,微博,校园
咚咚
健康
网购
体育
粉丝团,菜包,Nester,蔡平
文艺,旅游,健康,美食
新闻资讯,时尚,IT 数码,文艺,娱乐
教育就业,时尚,投资理财,星座命理,健康
星座命理
旅游,星座命理,美食,娱乐
旅游美食,留学生,摄影爱好者,自由,爱玩,新闻资讯,军事

其他生活
文艺,IT 数码,新闻资讯,旅游,美食,健康
旅游,睡觉,天蝎座,90 后,乐观,读书
自由,旅游,电影,生活,宅男,睡觉
搞笑幽默
娱乐
萌宠,微博奇葩,搞笑幽默,新闻趣事,视频音乐
80 后,公益,昆明
吃货,美食,健康,娱乐
80 后,英伦控,无厘头,微醺,爱做梦
搞笑幽默,新闻趣事,宠物图集,美食,星座运势
穿衣美容
美食
美食
好性格,汽车,幽默,电影,天秤座,游戏,80 后,旅游,网络
旅游
娱乐
文艺,旅游,流浪,射手座,写作,强迫症
视频音乐
网购,偶稀饭睡觉觉,吃
美女
旅游
时尚,体育,教育就业
娱乐,美食,游戏动漫
萌宠
酷酷,自由,吃,上网,偶喜欢玩,旅游,网购,美食,娱乐,水瓶座
徐正曦
美女
汽车,时尚
旅游,文艺,旅行,睡觉,80 后,自由,美食
娱乐,健康,军事,IT 数码,体育
萌宠
旅游
星座命理,娱乐,时尚,美食,健康
旅游
完美主义
美女,体育,时尚,旅游,美食,娱乐
文艺,星座命理,美食
星座命理
宅女,听歌,90 后
投资理财,时尚,美女,旅游,文艺,体育
美女,游戏动漫
聚会,酒吧,咖啡,音乐,玩儿
90 後,搞笑女,動漫控,犬夜叉迷,蜡笔小新控,电影狂人

山东生活
80 后
烟台生活
体育资讯,八卦杂谈,视频音乐,校园生活,搞笑幽默,体育
摇滚,娱乐
干物女
娱乐
自由,水瓶座,交友,文学,游戏,听歌,幽默,80 后
重口味,美容服饰,八卦杂谈
浪漫,好性格,乐观,微博,摄影爱好者,幽默,平常心,善良,睡觉
浙江生活,新中式,装饰画,陈设,软装配饰,工业造型,平面,插画,艺术,设计
汽车,Netizen,Workaholic,CarHobbyist,Perfectionist
新闻资讯
搞笑幽默
体育
星座命理,美食,体育
微博奇葩,体育
娱乐,汽车
旅游,IT 数码,游戏动漫,健康,投资理财,军事
旅游,星座命理
吃,语录,幽默,学生,时尚,体育,旅游,唱歌,摄影,90 后
新闻趣事,微博奇葩,视频音乐,搞笑幽默,穿衣美容
游戏动漫,体育
娱乐
音乐
文艺
星座命理
星座命理,游戏动漫,分裂,双子座
IT 数码,旅游,宅,汽车,阅读
媒体,美女,时尚,婚礼 MV,微电影,电影
90 后非脑残,书,交朋友,唱 K,逛街,奋斗,美食控,吃货一枚,喜欢听歌,好书
旅游,健康
搞笑幽默
搞笑幽默,旅游,视频音乐,娱乐,健康,投资理财
音乐发烧友,坚强,美食
时尚,旅游,美食,体育,娱乐
南极人保暖内衣,雅鹿羽绒内胆,千仞岗羽绒服,红豆内裤
八卦杂谈
文艺,新闻资讯,汽车,健康,投资理财
旅游,产业,金融,数据,平台
旅游,运动,双鱼座,唱歌,搞笑,吃,NBA
汽车
文艺
听歌,手机,90 后

穿衣美容,视频音乐
娱乐
校园,历史,羽毛球,电子商务,听歌
90后,善良,唯美,听音乐,逛街,旅行,读书,上网,吃,沉默
香港,深圳,purewhite,Designer,搭配控,Fashion
其他生活
军事,IT数码,文艺,体育
吃货!
微博奇葩,淘宝控
旅游
娱乐
娱乐,教育就业
托福 ing,吃货一枚,张杰滴学生星,张杰,极度衬衫控,偶稀饭睡觉觉,处女座,高中生,格子控,星星
文学,艺术,旅行,自由,美剧,摄影爱好者,电影,小说,水瓶座
IT数码,汽车,军事,努力,午饭,沉默,人生
IT数码
文艺
IT数码
宅,美食,娱乐
文艺,新闻资讯
时尚,投资理财,旅游,健康,娱乐
私人定制,芳香疗法
童装批发,好书,北京土著,网购,摩羯座,80后
网购,互联网,生活,沁园,净水器,电子商务
娱乐,旅游,金牛座,喜欢音乐,爱睡觉,街舞,爱自由
穿衣美容,星座命理,旅游,视频音乐
星座命理
喜欢简简单单……
旅游,星座运势,桂林生活,读书分享,美食
娱乐
健康
旅游,美食,文艺,娱乐
IT数码
文艺,吃,90后
汽车,80后,听歌
投资理财,时尚,健康
读书,交友,旅行,旅游,看书,上网,天秤座,唱歌,平常心,宅
美女
投资理财,美食,旅游,IT数码,文艺,时尚,AYXA,SHOP
上海生活,美图摄影,宠物领养,宠物救助,流浪狗救助,流浪狗领养,流浪猫救助,流浪猫领养,公益慈善,爱
社会实践,公益志愿者,校园,大学生,学生
新闻资讯,公交车,南宁轻轨,公共交通,南宁地铁,南宁公交,公交轶事

旅游,萌宠,视频音乐,IT 数码,汽车,游戏动漫,体育
玩转生活,自由
娱乐,熊出没
娱乐
烟雨江南,死神,魔兽争霸 3,诺基亚,梅西
娱乐,时尚
投资理财,时尚,美女,健康,星座命理
娱乐,文艺,自由,电影,玩儿,吃货,艺术,生活,游戏,听歌
明星,网游,爱旅行,高尔夫,中医,80 后,金融,互粉,旅游,投资理财
游戏动漫
汽车,美食,回族,清真,新疆
狮子老虎,80 后,音乐,搞笑,篮球,美食
汽车,旅游,美食,IT 数码,娱乐
善良,平常心
爱美女,睡觉 zZ,懒人一枚
游戏动漫,体育,新闻资讯
星座命理,美食,文艺
时尚
旅游,娱乐,IT 数码
射手座
汽车
IT 数码,汽车,新闻资讯,教育就业,美食
打篮球
游戏动漫,时尚,娱乐
文艺,娱乐,IT 数码,星座命理,游戏动漫
重庆生活
我简简单单的我
校园生活
广东生活,穿衣美容,星座命理,新闻趣事,搞笑幽默,旅游爱好者,电影迷,K 歌,文字
IT 数码,八卦杂谈,搞笑幽默,校园生活
时尚,IT 数码,星座命理
星座命理,美食,娱乐
艺术工作者,互联网,艺术,音乐,媒体
健康,旅游,IT 数码,美食,文艺
旅游,投资理财
名人明星,穿衣美容,星座命理,美食,视频音乐,小小盆友,天秤座,吃货
教育就业,体育,娱乐,投资理财
健康,文艺
旅游,文艺,游戏动漫,健康,美食
视频音乐
娱乐
文学,NBA,A 股,好性格,八卦,财经,体育,亲情,媒体
文学,白羊座,金牛座,浪漫,微博,IT,好书,广告
吃货,范二,爱睡觉

美食,游戏动漫
80后,摩羯座,摄影爱好者,浪漫,八卦,财经,玩儿,大学,语录,博客
健康,旅游,IT数码
娱乐,人生得意须尽欢
爱唱歌,lovefashion,偏执狂,爱猫,爱生活,music,偶喜欢玩,疯子,文艺,打排球
旅游,IT数码,美图摄影,广州生活,搞笑幽默
90后,NBA,白羊座,英语,A股,互联网,文字,写作,校园
旅行,创业,八卦,紫色,历史,囧,自由职业,传媒,语录,广告
自由,时尚,80后,创业,A股,八卦,手机,文字,体育,大学
旅游
原创,往年,扒拉扒拉,控型,汽车,星座命理,IT数码
IT数码,体育,看电影,听歌,打篮球,喜欢旅游
美图摄影,IT数码,双鱼座,开心,梦想,美术,设计,90後
游戏动漫,善良,上网
双鱼座,善良,90后,生活,小说,吃货,听歌
教育就业,体育,汽车,旅游,新闻资讯
旅行,创业,爱情,NBA,金牛座,A股,好性格,体育,校园,媒体
自由,美食,听歌,偶稀饭睡觉觉,爱情,金牛座,摄影爱好者,网络,传媒,博客
旅行,美食,80后,汽车,创业,英语,金牛座,好性格,写作,广告
自由,听歌,汽车,创业,英语,营销,写作,校园,传媒,广告
汽车,摄影爱好者,微博,手机,文字,囧,体育,电子商务,好书,语录
唱歌,狮子座
自由,80后,美剧,A股,摄影爱好者,八卦,玩儿,写作,亲情,语录
娱乐
动漫
娱乐
时尚,美食,爱情,摄影爱好者,八卦,网络,微博,文字,亲情,博客
搞笑幽默
电影,美食,90后,美剧,羽毛球,金牛座,浪漫,历史,好书,大学
娱乐,汽车,投资理财,旅游,时尚
美食,星座命理,小说,K歌,随性,神经质,看电视
美食,美女,时尚,体育,娱乐
电影,美食,爱情,阅读,偶喜欢玩,八卦,微博,文字,囧,写作
紫色,学生
旅行,美食,微博,紫色,互联网,文字,体育,IT,亲情,媒体
自由,美食,听歌,偶稀饭睡觉觉,NBA,羽毛球,英语,历史,自由职业,亲情
潜力股,学生
宅女一枚,爱吃,爱玩,好书,天枰座,唱k,流浪,音樂,低调,心理学
搞笑幽默
汽车,摩羯座,金牛座,偶喜欢玩,摄影爱好者,网络,历史,互联网,IT
90后,听歌,汽车,爱情,英语,微博,历史,体育,好书,传媒
视频音乐
八卦杂谈
电影,好性格,美食,羽毛球,听歌,新闻,自由,唱歌,搞笑,天秤座

旅行,时尚,美剧,摩羯座,偶喜欢玩,八卦,手机,历史,互联网,传媒
美女
军事,大学,听歌
80 后,听歌,汽车,白羊座,微博,电子商务,营销,数码,写作,自由职业
娱乐
旅游美食爱好者,向日葵,音乐发烧友
读书分享,时尚,闷骚女,无聊,宅,睡觉,巨蟹座,大学生
时尚
音乐,爱情,NBA,A 股,新闻,偶喜欢玩,亲情,广告,博客
美女
水瓶女,爱吃,时事,足球迷,创业,臭美,心情,女人,证券,理财
星座命理,美食,新闻资讯,旅游,游戏动漫,娱乐
时尚达人,唱 k,爱音乐,品牌,女人
美食
唱歌。打篮球
广西生活,旅游
星座命理,美食,新闻资讯,游戏动漫,旅游
文艺
电影,音乐,爱情,白羊座,英语,摄影爱好者,好书,数码,媒体,博客
体育
天枰座,钓鱼,平常心,爱唱歌
搞笑幽默
美女,时尚,文艺,健康
穿衣美容
美食,自由,旅行
投资理财,旅游,美食
80 后,音乐,美剧,偶稀饭睡觉觉,金牛座,网络,玩儿,数码,自由职业,广告
宅女
新闻趣事,厨房达人,减肥食疗,食材,养生,煲汤
娱乐
游戏动漫
旅游
健康,时尚,美女,娱乐
美女,投资理财,游戏动漫,IT 数码
A 股,浪漫,囧,电子商务,IT,数码,校园,博客,金牛座,财经
旅游,80 后,摩羯座,文学,NBA,网络,历史,玩儿,自由职业,大学
育儿百科
跳舞
自由,时尚,旅游,90 后,创业,微博,体育,大学,媒体,广告
好性格,睡觉,金牛座
教育就业,时尚,旅游,美食,文艺,欧美剧,欧美电影,做吃的
时尚,娱乐
游戏
投资理财

偶稀饭睡觉觉,文学,八卦,手机,IT,好书,写作,校园,媒体,博客
文艺
体育
娱乐,美女
校园,双重性格,看电影,爱音乐,阳光,可爱,舞蹈,画画,美食,文艺
时尚
健康,体育
电影,自由,美食,羽毛球,英语,好性格,偶喜欢玩,浪漫,紫色,营销
旅游
文艺,娱乐
美食,汽车,创业,偶稀饭睡觉觉,文学,羽毛球,金牛座,好性格,微博,玩儿
美食
旅游
时尚,汽车,文学,好性格,浪漫,历史,文字,IT,大学,传媒
游戏动漫
时尚,双鱼座,白羊座,好书,吃,旅游,浪漫
o 型血,爱自由,日本,旅游,绘画,健身
偶稀饭睡觉觉,摩羯座,羽毛球,好性格,八卦,微博,囧,自由职业,大学,语录
美食,孤独门生,苗迷,妄想派,完美主义者
星座命理
好孩纸
微博奇葩
娱乐,健康,新闻资讯,旅游,时尚
听歌,摄影爱好者,手机,紫色,IT,营销,好书,数码,语录,广告
IT 数码
游戏动漫,IT 数码,体育,汽车,娱乐
电影,时尚,90 后,创业,A 股,财经,紫色,囧,校园,大学
美食,偶稀饭睡觉觉,文学,英语,A 股,财经,电子商务,好书,数码,语录
汽车
90 后,9,宅宅,吃货,水瓶座
娱乐,旅游
旅行,美食,90 后,创业,NBA,偶喜欢玩,网络,手机,电子商务,玩儿
电影,美剧,汽车,创业,英语,体育,电子商务,IT,玩儿,大学
美食,星座命理,娱乐
财经,大学生,90 后,数码,互联网,体育,新闻,吃,动漫,狮子座
好性格,旅行,听歌,幽默,双子座,运动,读书,电影,新闻,善良
摄影,旅行,瑜珈,钢琴,伯明翰,伦敦,英国,温哥华,加拿大,普华永道
美食,旅游,文艺,爱死神,金牛座最后一天,90 后,偶稀饭睡觉觉
新闻资讯
IT 数码,汽车
旅游,健康,娱乐,投资理财,时尚,看书
自由,网络,游泳,偶喜欢玩,囧,幽默
视频音乐,搞笑幽默,美食
军事,健康

艺术,偶喜欢玩,浪漫,汽车
游戏动漫,娱乐
微博奇葩,其他生活,新闻趣事,视频音乐,搞笑幽默
新闻资讯
双鱼座,足球,创业,交友,音乐,80 后,股票
旅行,美食,90 后,文学,白羊座,紫色,营销,自由职业,亲情,博客
旅游,投资理财
搞笑幽默
80 后,创业,偶稀饭睡觉觉,阅读,白羊座,金牛座,新闻,摄影爱好者,文字,校园
文艺,旅游,美食,时尚
美女
视频音乐
美女,娱乐,军事
萌宠,美女,星座命理
名人明星
游戏动漫
自由,阅读,白羊座,英语,金牛座,新闻,体育,自由职业,大学,传媒
爱情,NBA,好性格,新闻,偶喜欢玩,浪漫,手机,历史,互联网,IT
听歌,美剧,创业,文学,白羊座,网络,财经,好书,博客
时尚,旅游,汽车,爱情,阅读,NBA,互联网,体育,玩儿,自由职业
汽车
体育
体育
娱乐,唯爱 SuJr
旅游爱好者,追星,音乐发烧友,娱乐
80 后,创业,摄影爱好者,网络,囧,体育,电子商务,好书,亲情,博客
动漫,书,上网，睡觉，网游
羽毛球,财经,手机,紫色,营销,自由职业,校园,旅游,美剧,八卦
时尚,健康,旅游,美食,自由,看书,80 后
电影,80 后,听歌,音乐,NBA,金牛座,好性格,IT,玩儿,校园
时尚,旅游,美食,文艺,娱乐
美食,时尚,旅游,娱乐,体育
新闻趣事
旅游,天蝎,80 后
时尚,娱乐,星座命理
阅读,英语,A 股,财经,历史,体育,玩儿,好书,语录
娱乐
健康,旅游,时尚,星座命理
健康,时尚,投资理财,娱乐,IT 数码,美食
八卦杂谈,美图摄影,江苏生活,校园生活,搞笑幽默
娱乐
美食,留学生食谱,家做美食,简易健身,生活妙招,省钱
美女,汽车,娱乐,体育,IT 数码
爱玩,天蝎座,偶稀饭睡觉觉,吃货

经济,投资理财
新闻资讯,时尚,娱乐,00 后的小魔女,看书,宅,美食控,钢琴,旅游美食爱好者
生物制药,时尚,彩妆护肤
搞笑幽默,文艺
时尚,爱情,白羊座,好性格,新闻,摄影爱好者,八卦,微博,互联网,亲情
时尚,汽车,爱情,NBA,A 股,摄影爱好者,电子商务,写作,媒体,博客
时尚,文艺,娱乐,内向,喜欢自作多情,喜欢胡思乱想,喜欢自拍,狂吃不胖,爱笑,喜欢听歌
视频音乐
时尚,小说,旅行,美食,音乐
教育就业,娱乐,体育,美女,汽车,文艺
80 后,90 后,听歌,偶稀饭睡觉觉,阅读,金牛座,A 股,财经,营销,博客
健康,旅游
名人明星,新闻趣事,视频音乐,旅游,美女
80 后,时尚,吃,宅男
娱乐,时尚
音乐达人,舞蹈,电视剧,娱乐,表演,深圳生活,旅游
娱乐
自由,旅游,宅,睡觉
星座命理,体育,天蝎座,自由职业,90 後,桌球
旅游,汽车,摩羯座,白羊座,羽毛球,偶喜欢玩,手机,营销,媒体,语录
时尚
健康
学习
美食
游戏动漫
教育就业,旅游,美女,新闻资讯,健康,时尚
体育资讯
娱乐,IT 数码
电影,旅游,90 后,创业,摩羯座,网络,互联网,数码,写作,广告
红魔,床上钉子户,巨怕热,曼联死忠
星座命理
玩儿
娱乐
英国生活,汽车,腐女
苹果控,时尚控,数码,旅行,汽车,娱乐
搞笑幽默
娱乐,冷酷
IT 数码
90 后,羽毛球,金牛座,浪漫,微博,历史,互联网,囧,体育,传媒
电影,自由,爱情,阅读,文学,白羊座,羽毛球,手机,校园,广告
典型的处处
IT 数码
IT 数码,梦想,活力,财经,平常心,金牛座
艺术,平常心,创业,读书,音乐

汽车,创业,爱情,NBA,英语,紫色,历史,文字,体育
90 后,羽毛球,A 股,历史,囧,玩儿,自由职业,校园,语录
听歌,文学,金牛座,偶喜欢玩,互联网,文字,体育,好书,自由职业,校园
星座命理,汽车,娱乐,旅游
旅游
生活,游戏,吃
娱乐
旅行,摩羯座,NBA,英语,新闻,微博,玩儿,好书,写作,传媒
美食,星座命理,时尚,栽花,烘培
军事
投资理财,美女,体育
娱乐,汽车,新闻资讯,游戏动漫,美女,美食,旅游,星座命理
游戏动漫
听音乐看电影,旅遊,散步,美食
小兔,可爱,活泼
生活是把杀猪刀
搞笑幽默
教育就业,健康,美女,军事,体育
时尚,爱吃,游戏动漫,健康,美食
小清新,读书,文艺,旅游,美食
体育
美食
大学,美食,猫咪控,天蝎座,爱音乐,90 后
旅游,美食,美女,福建生活
新闻资讯,娱乐
美女
四年级小学生,读书,跳舞,9 岁
旅游,股票,网购,天蝎座
视频音乐,名人明星
教育
射手座
美食,旅游
其他生活
星座命理
娱乐
旅游,娱乐
文艺,时尚,娱乐,旅游,美食
新闻趣事,搞笑幽默,浙江生活
娱乐
韩国生活
文艺,星座命理,时尚,汽车,设计,K 歌,逛街,旅游,瑜珈,电影
IT 数码,旅游,美食
美食,游戏动漫
娱乐

情感生活,温州生活
汽车,时尚,自由,浪漫,设计,电影,双子座,艺术,旅游
搞笑幽默
IT 数码,美女,星座命理,时尚,军事,汽车
爱玩,爱唱歌,跆拳道,娱乐,游戏动漫
诗
90 后非脑残,爱电影,懒,音乐控,网购
娱乐,IT 数码,美食
IT 数码
安康科士威,渭南科士威,宝鸡科士威,咸阳科士威,汉中科士威,运城科士威,延安科士威,铜川科士威,西安科士威,创业
美食,萌宠,旅游
美食,萌宠,天秤座,自由,音乐
华晨宇,花花
游戏动漫,娱乐
爱菜头,听歌,唱歌,看书,射手座,幽默,玩儿,美食,音乐,动漫
美食
不辜负此生
时尚,文艺,健康,教育就业,娱乐
懒入骨
搞笑幽默,新闻趣事,视频音乐,微博奇葩,萌宠
时尚
IT 数码,星座命理,娱乐,语录,123,数码
娱乐,健康,星座命理
视频音乐,微博奇葩,穿衣美容,旅游,美食
80 後,服饰,新闻趣事,微博奇葩,穿衣美容,旅游,美食,时尚,健康,娱乐
开朗,幽默,偶稀饭睡觉觉,宅女,唱歌,上网,交友,设计
唱歌
投资理财,美食,旅游
投资理财
80 后,手机,浪漫,自由职业,上网
体育,IT 数码
旅游,美女,视频音乐,搞笑幽默,江苏生活
囧,白羊座
学生,听歌
创意策划,酒吧,夜店,圆素 CLUB,摄影,生活,交友,摄影爱好者,90 后,媒体
旅游,健康,时尚,教育就业,投资理财
旅游
星座命理,个性,TTL,完美主义,音乐,天蝎,T,时尚,跳舞,吃货
游戏动漫,体育
美食,投资理财,娱乐,时尚
军事,IT 数码,文艺,体育,娱乐
时尚
自信

娱乐,文艺,时尚,水瓶座
美女,军事,娱乐
宇宙饮食,唐安麒
星座命理
游戏动漫
搞笑幽默
新闻资讯
文艺,健康,旅游,军事,星座命理
游戏
健康,时尚,军事
水瓶座,唱歌,好书
美女
学生,狮子座,摄影爱好者,唱歌
名人明星,吃,交友
美食,萌宠,美女,新闻趣事,搞笑幽默,娱乐
星座命理,时尚
睡懒觉,爱自由,吃喝玩乐,舞蹈,爱生活,爱睡觉,电视剧,艺术,电影
汽车,时尚,旅游
娱乐
旅游
雾霾之都,旅游爱好者,摄影爱好者,玛莎拉蒂,英雄联盟,爱刺激,周杰伦忠实粉丝,射手座
回粉,美食,吃货一个
多宝,爱多亮,喜欢旅游,喜欢音乐,搞笑,巨蟹座女生,旅游,美食,80 后,网购
健康,文艺,娱乐,新闻资讯,美食
宅男
游戏动漫,IT 数码,文艺,娱乐
GAY
时尚
娱乐
美食
旅游,娱乐,健康,星座命理,美食
名人明星,美食
历史,科学
旅游,IT 数码,投资理财
美食
时尚,娱乐,旅游,健康
娱乐,IT 数码,美食,健康,投资理财
听听音乐"
创业,美剧,上网,美食,钩编
美食
四川生活
娱乐
娱乐,文艺,星座命理,萧亚轩,涂鸦,吃,双子座,爱音乐,活泼,洒脱
投资理财,时尚,新闻资讯,IT 数码

旅游
娱乐
文艺,汽车,新闻资讯,IT 数码,娱乐,军事
寻仙,白羊座,运动,学生
娱乐,时尚
旅游
美食,IT 数码,军事,旅游,体育
旅游,新闻趣事
美食,矛盾体,90 后
旅游
文艺
至天使，茂美党
发呆,散步,日语,90 後,吃货一枚,双重性格,学生一枚,滑雪,好书,健康
绍兴生活,摄影旅游,化妆造型师
行走在路上,喜欢阳光,热爱旅行
IT 数码,体育资讯,八卦杂谈,校园生活
搞笑幽默
星座运势
偏执狂,夜猫子,高中生,睡觉睡到自然醒,天蝎女,喜欢旅游,盗墓笔记,小清新,校园,旅游
体育,娱乐,IT 数码,游戏动漫
时尚,健康,旅游,星座命理,美食
健康,旅游,教育就业,娱乐,90 后
新闻资讯,美女,娱乐
吃货,历史
美食,唱歌,交友,白羊座,摄影,宅女
八卦杂谈,幽默,摄影
文艺,体育,娱乐
搞笑幽默,体育,生活,善良,文字,电影
旅游
惠州生活
母婴,育儿,孕妇,怀孕,妈妈,宝宝,胎教
军事,投资理财,汽车,旅游,美食
游戏动漫,美女,星座命理,美食,IT 数码
健康
星座命理,美食
美食
新闻资讯,教育就业,投资理财,旅游,体育
娱乐
旅游,时尚,文艺,IT 数码
名人明星
新闻资讯
美食
游戏动漫,美食,娱乐
游戏动漫,军事,美食,体育

时尚,IT 数码
双子座
美食
星座命理,娱乐,健康,时尚,教育就业,工科女,人来疯,白羊座的女生,文学,90 后
美剧迷,上海生活,购物狂,唱 k,爱情,腐,女人,穿衣美容,吃货,90 后
动漫
IT 数码,娱乐
搞笑幽默,情感生活
自由,听歌
娱乐
创业,运动,双鱼座,亲情,90 后,吃,乐观,睡觉
旅游,幽默细胞一麻袋,天生乐观派,摇滚,外贸,旅行
浪漫,快乐,体育,旅游美食
浙江生活
视频音乐
星座命理,美食
旅游
星座命理
星座命理,美女,体育,娱乐,走着,乐观好心态,电影,巨蟹座,篮球,平常心
时尚,旅游,美食
教育就业
我是楼井家二筝,我是萌萌
IT 数码,美食,美女,时尚,汽车
军事,美食,新闻资讯,投资理财,美女,文艺
健康,投资理财,星座命理,美食,娱乐
娱乐,文艺,星座命理
搞笑幽默
汽车,股票
美女,时尚,娱乐,游戏动漫,军事
体育
星座命理,健康,美丽天使,爱吃,雙魚座,80 后
投资理财
投资理财,美女,娱乐,IT 数码,体育
军事,游戏动漫,体育,旅游,汽车,美女
户外,交朋友,我就是我,旅游
新闻资讯,美食,时尚,娱乐,旅游
购物,星座命理,美肤,时尚,交友,听歌,处女座,努力 ING,90 后,音乐
新闻趣事,电影,网购,历史,微博,吃货,财经,大学生,睡觉,90 后
娱乐,旅游,体育
军事
娱乐
美女,娱乐,公益,电影,光华志愿者协会
活泼,海绵宝宝,未来
嗜睡,逛街,买衣服,养猫,K 歌,大爱吴彦祖

旅游,军事,IT 数码,文艺,娱乐
名人明星
美食,投资理财,时尚,游戏动漫,旅游
美女
文艺
穿衣美容,美食
美女
文艺,魔羯座,DIY 达人
爱笑,日本,喜欢音乐
旅游,美食,文艺,娱乐,健康
视频音乐,八卦杂谈,IT 数码,新闻趣事,搞笑幽默
汽车,文艺,健康,旅游,投资理财
美食控,苹果控,听歌,美剧,美食,时尚,睡觉,90 后
NBA
爱听做你的公主,狮子座,唱歌,吃货,睡觉,电影,爱阿布,爱魏晨,90 后,美女
娱乐
文艺,时尚,旅游,Dreamlike,TVB 控,SunShine,宅女一朵,baby 小女生
穿衣美容
美女
健康,时尚,舞蹈爱好者
文艺
四川生活
三明生活,服务行业,学雷锋,金融
文艺
宅女,体育,80 后
投资理财,美食
军事,娱乐,美食,IT 数码,教育就业,投资理财,体育
music,喜欢旅游
健康,时尚
教育就业
乐观,自由
睡觉,旅游,美食
时尚,旅游,IT 数码,文艺,娱乐
90 后
萌宠,美食,美女,视频音乐,辽宁生活
美食
娱乐,旅游,健康
娱乐
视频音乐,搞笑幽默,美食,萌宠,名人明星
时装,吃货,双子座,Music,爱猫,情绪化,听歌
星座命理
艾薇儿,music,搞笑幽默,湖人
美食,穿衣美容,体育
健康,旅游,美食,军事,娱乐

文艺,娱乐
星座命理,游戏动漫,投资理财,保险理财,教育基金,养老保险,车险,团险,平常心
摄影,安静,喜欢旅游,水瓶女,90 后非脑残,收藏
旅游
情感生活,我就是我,90 后,好奇宝宝,五月天,害怕孤独,沉默
视频音乐,吃,平常心,90 后,美剧,小说,学生,双鱼座
裴秀智,韩国控,金明洙,小说迷,爱唱歌,90
美食
性别男，愛好女。
搞笑幽默
睡觉,唱歌,学生
星座命理
上海生活
体育
西安生活
娱乐
学问
体育
娱乐
新闻资讯,教育就业,旅游,娱乐,文艺
名人明星,美食,穿衣美容,Ftisland
忻芯相印,清城中学学生,美特生,上网,看书,天秤座,90 后,懒虫,爱听歌,宅
粉红控,薰依草,00 后,搞笑,英语,学生,狮子座,善良,偶喜欢玩,看书
健康,动漫,睡觉,美食,宅,自由,旅游,娱乐
旅游
旅游,狮子座,音乐,偶稀饭睡觉觉,玩儿,吃货
时尚
美食,搞笑幽默
文艺
星座命理,时尚
杭州生活
娱乐,文艺,IT 数码,美女,游戏动漫
旅游,娱乐
搞笑幽默,旅游
美食,电影,好书,平常心,网购,亲情,微博,营销,交友
美食,时尚,健康
视频音乐,搞笑幽默,新闻资讯,美食,星座命理,健康,文艺,旅游
旅游
娱乐
游戏动漫,汽车,美食,星座命理
IT 数码,娱乐,新闻资讯,时尚,体育
文学,亲情,浪漫,自由,90 后,美剧,手机,k 歌,上网,听歌
文艺
娱乐

吉林生活
圭贤控，SJ 迷
爱,坚强,完美主义,法国,爱笑,安静,快乐,90 後,做梦,文艺
视频音乐,嗯嗯嗯,好性格,音乐
IT 数码,教育就业,健康,军事,汽车
西安生活,设计师,别墅装修,别墅设计
摄影达人,摄影爱好者
文艺
搞笑幽默,IT 数码
美食
微博奇葩,名人明星,穿衣美容,新闻趣事,搞笑幽默
双子座,烹饪,ET,90 后,电影控,微博控,陈赫,曾小贤,欣赏幽默的人
活泼开朗,逛街
旅游
让自己忙碌起来
旅游,健康,军事,文艺,娱乐
时尚,美食,星座命理,文艺,娱乐
新闻资讯
美食
八卦杂谈,校园生活,东莞生活,有点傻,减肥,爱电影,热爱生活
星座命理
娱乐
90 後,音樂,音乐,自由,历史,阅读,乐观,水瓶座,90 后
美食,自由
体育
杭州生活
星座命理,时尚,美食,IT 数码,文艺
askayang,鸽子,楊宗緯,杨宗纬
搞笑幽默,其他生活,微博奇葩,星座命理,穿衣美容
电影,小说,宅女
名人明星,搞笑幽默
梦想,音樂,奋斗,篮球,个性,发呆,海贼王
星座命理,摩羯座,运动,微博,上网,美剧
教育就业,时尚,汽车,体育
时尚
名人明星,穿衣美容,新闻趣事,健康,汽车,旅游,星座命理,娱乐
美食,星座命理,IT 数码
游戏动漫
视频音乐,美食,旅游,名人明星
美食
视频音乐,萌宠,旅游,写作,浪漫,美食,艺术,摄影,善良,搞笑
新闻趣事,搞笑幽默,美女,新疆生活,投资理财,新闻资讯,IT 数码,游戏动漫,旅游
游戏动漫,投资理财
娱乐,游戏动漫,IT 数码,星座命理

有点小冲动,90 后,三分热度,朦胧
乐天派,天蝎,感性,时尚,健康,平常心,美女
网络,摄影,数码
电影,好性格,天秤座,吃,上网,睡觉
美食
北京生活,旅游
宅,吃
娱乐,搞笑,乐观,爱情,美食,音乐,时尚,动漫,好性格
美食
期望越大,失望越大
旅行,学生,自由,名人明星,摄影，美术
搞笑幽默
四川生活,活泼,大大咧咧,开朗,90 后,金牛座,乐观
星座命理,娱乐
美食爱好者,喜欢听歌,热爱生活,敏感,休闲,星座命理,视频音乐,旅游,广东生活,偶是打酱油的!
投资理财,旅游,体育,美食,军事
美女
美女,教育就业,健康,娱乐,文艺,美食,旅游
健康,教育就业,旅游,体育
微博奇葩,搞笑幽默,美女,视频音乐,宅男
美食,樱桃小丸子
时尚,投资理财
娱乐
IT 数码,美女,娱乐
摄影爱好者,车迷,喜欢运动和唱歌
投资理财,旅游,美食,军事,IT 数码
旅游
宅女,睡觉
旅游,星座命理,文艺,IT 数码,处女座,好性格,唱歌,数码
旅游
八卦杂谈
美食
新闻趣事,搞笑幽默
IT 数码
旅行,吃货,美剧,电影
游戏动漫
运动,摄影控,美食,投资理财,娱乐,IT 数码,体育
美食
时尚,游戏动漫,文艺
美女
娱乐
其他生活
文字

时尚,投资理财
旅游,娱乐,教育就业,军事
文艺,娱乐
文艺,新闻资讯,游戏动漫,时尚,旅游,娱乐,美食
美食,美女,健康,旅游
文艺,汽车,健康,星座命理,娱乐
登山,电视,爱美食,购物狂
时尚,娱乐
美女,名人明星,穿衣美容,美食,萌宠
美女
美女,游戏动漫
听歌
IT 数码
旅游
旅游,美食,游戏动漫,教育就业,娱乐
旅游,90 后,吃货,听歌
美食,爱唱歌,水瓶座
娱乐,游戏动漫,文艺
名人明星,旅游,美食
足球迷
美食
上海生活
欧美控
汽车
游戏动漫,文艺,IT 数码,汽车,爱情,睡觉,听歌,上网
冬季,雪花,篮球
美食
娱乐
美食
吃货,新的邮箱和微博,新的梦想跟开始,读书,天秤座,文字,交友,唱歌,旅游
天秤座,上网
新闻资讯,健康,文艺,投资理财,星座命理
摄影,时尚,狮子座,旅游,吃货,生活,80 后,设计,幽默
五月天,爱睡觉,拍照,怀旧,矛盾,文字控,文学,完美主义,努力,音乐控
微博奇葩
旅游
游戏动漫,上网,家庭教师 Reborn,柯南迷新兰控,死神,海贼王
我不服社会
时尚
名人明星,娱乐,文艺
体育,游戏动漫
旅游
艺术,玩儿,吃货,自由
表演,宅,吉他钢琴,迟钝型,90 后,水瓶座,音乐,唱歌

美食, 旅游, 新闻趣事, 搞笑幽默, 湖南生活, 健康, 新闻资讯, 投资理财, 文艺, 娱乐
广西生活, 搞笑幽默, 微博奇葩
旅游, 娱乐, 星座命理, 新闻资讯, 美食
名人明星
投资理财, 波浪理论, 主升浪, 涨停, 交友, 宅男, 创业, 股票, 财经
动漫, 搞笑幽默
游戏动漫, 文艺
娱乐, 投资理财, 美食, 文艺, 时尚
校园生活
校园生活
美女
旅游, 健康, 时尚, 娱乐
美食
学生
搞笑幽默
时尚, 娱乐, 新闻资讯, 投资理财, 体育
文艺, 娱乐, 体育, 美女, 游戏动漫
合肥生活, 翰爽、武艺控
武威生活
视频音乐, 萌宠
新闻资讯, 星座命理, 美食, 美女, 时尚
健康
穿衣美容, 旅游, 美女, 新闻趣事, 惠州生活
搞笑, 乐观, 90 后但我不脑残, 听歌, 时尚, 音乐, 白羊座, 幽默, 自由
美图摄影, 美食
萌宠, 沉默是金丶
文艺
娱乐, 汽车, 游戏动漫, 健康, 美食
体育
旅游
名人明星, 穿衣美容
汽车, 517890994, 偶稀饭睡觉觉
美食, 星座命理
穿衣美容
美食, 娱乐
军事
声音控, 双鱼座, 强迫症, 玩, 上网, 购物, 娱乐, 唱歌, 自由, 宅
80 后宅女, 爱电影, 玩, 服饰, 喜欢唱歌, 化妆, 爱生活, 电视剧, 爱睡觉, 减肥
文艺, 娱乐
八卦杂谈, 文艺, 娱乐
星座命理, 健康
投资理财, 游戏动漫, 新闻资讯, 美食, 军事, 电影, 读书, 买乐购商城, 团购, 购物
萌宠, 星座命理
美食, 宅女, 交友, 旅行, 吃货, 阿暮

牧业,保护他人,单身,金华,武汉,旅游,成都,恋爱
奋斗,爱幻想,青春,喜欢听歌,哈哈,火影,苹果,新闻,宅男,羽毛球
IT 数码
新闻趣事
娱乐,宅女,巨蟹,吃货,90 后
IT 数码
搞笑幽默
强迫症,情绪化,音乐达人,香橙,体育,娱乐,旅游
健康,旅游
热情,猫咪,睡懒觉,好性格,85 后,美食控,互粉,活泼,时尚,娱乐
微博奇葩
星座命理,时尚,娱乐
时尚,美女,健康,娱乐,旅游
IT 数码
娱乐,星座命理,游戏动漫
游戏动漫
投资理财,军事,时尚,IT 数码,娱乐
喜剧,创业,自恋,闷骚,科技,工作狂,原创,胡思乱想,搞怪,阅读
生活,善良,旅行
娱乐
IT 数码
旅游,时尚,娱乐,80 后,看书,宅
职场招聘,搞笑幽默
典型的二货,吃货,睡货
美食
旅游
汽车
其他生活
娱乐,时尚
时尚
星座命理,美食
娱乐
娱乐,时尚,美食,旅游
恐怖片,孩子气,购物狂,瑜伽,艺术,阅读,喜欢旅游,水瓶座,美食,时尚
新闻趣事
经典,男士裘皮,男士皮衣,德国,vllrno,皮草,商务,威尔诺
微博奇葩,美食,90 后非脑残,music,健康,娱乐,文艺
语录,囧,宅,小说,帅哥,偶稀饭睡觉觉,爱情,天平座,音乐,90 后
娱乐
新闻资讯,文艺,娱乐
投资理财,美食,时尚
职场招聘,美女,视频音乐,滨州生活,体育,教育就业,健康,投资理财,星座命理,文艺
游戏动漫
星座命理,旅游,娱乐,文艺,时尚

星座命理,IT 数码,娱乐
新闻趣事,IT 数码
时尚,IT 数码,美食,旅游
旅游,疯疯癫癫
体育
健康,新闻资讯,文艺,体育,双子座,浪漫,英语
游戏动漫
娱乐,健康,旅游,汽车,体育
美食,健康,军事
视频音乐,勉強唔來。
游戏动漫,新闻资讯,星座命理,美食,文艺
文艺,旅游,美食
帅
美食,星座命理
汽车
美女,移动互联网,娱乐,微博控,90 后,处女座,交友
星座命理,新闻资讯,时尚,IT 数码,娱乐,学生
汽车,军事,健康,IT 数码,体育
阅读,听歌,90 后,吃货,摄影,白羊座,自由,旅游
娱乐
美女,娱乐,游戏动漫
Cosplay 爱好者,古风音乐,宅腐双修考据党,瓶邪
美食,穿衣美容,美女,新闻趣事,搞笑幽默
旅游
军事,新闻资讯
健康,游戏动漫,教育就业,军事,文艺
听音乐,唱歌,开朗的小会计
视频,动漫
军事,投资理财
微博奇葩,游戏动漫,体育
体育资讯
2013,体育,汽车,网购,吃货,乐观
时尚,旅游,星座命理,美食,娱乐
美女,IT 数码,投资理财,苹果控,电脑,心理,火影忍者
美食
健康,IT 数码,星座命理,新闻资讯,文艺
视频音乐
上班族,游戏控,火锅控,烧烤控,爱睡觉,爱吃,a 型血,摩羯座,80 末,音乐
教育就业
旅游,美女,健康,星座命理
时尚,健康,美女,娱乐,美食
体育
体育
校园生活

投资理财
文艺,旅游,健康,教育就业
汽车,IT 数码
新闻趣事,搞笑幽默,安阳生活,宠物图集
沉默,疯狂,漫画,安静,偶喜欢玩,无厘头,书籍,杂志,茶
听段段的歌
健康,娱乐,教育就业,时尚,体育
自由,时尚,双鱼座,90 后
中国男篮,中国女篮,运动,中国女排,90 后,旅游,幽默,浪漫,白羊座,听歌
时尚,教育就业,旅游,美食,投资理财
动漫,乐观,微博,听歌,好性格,爱情,天蝎座,90 后,睡觉,八卦
旅游
校园生活,搞笑幽默,美食,星座运势,情感生活
美女
游戏动漫,体育
文艺
视频音乐,电影,交友,90 后,听歌,爱情,浪漫,看书,旅游
浙江生活
时尚,文艺,IT 数码,娱乐
美食,唱歌,90 后,网购,旅游,音乐,微博,金牛座,娱乐
投资理财
Leehyukhae,EunHae
星座命理,旅游,美女,娱乐,文艺
体育,军事,IT 数码
旅游,教育就业
搞笑幽默,穿衣美容,星座命理
河北生活,时尚
新闻资讯,健康,旅游,汽车,军事,美食
视频音乐,足球
教育就业,新闻资讯
美食
美食,美女
搞笑幽默
美食
軍事,時尚,投資理財,文藝,娛樂
善良
汽车,时尚,星座命理,美食
浪漫,好性格,乐观,80 后
知性而不失理性
美女,娱乐
90 後,动漫迷,猫咪控,潮,广州人,狮子女,音樂,学生一枚,星座控,娱乐
IT 数码,教育就业,时尚
时尚,汽车,文艺,旅游,娱乐
美食

吃喝玩乐,赛车,水瓶座,旅游,听歌
美食
鲁南大厨
浙江生活
学生,爱唱歌,爱玩……
驴友,时尚达人,巧克力,化妆品,会计,阅读,休闲,爬山,K 歌,购物狂
阿狸的情书,最爱 jj 林俊杰,阿狸迷。,语录,90 后
汽车,好性格,狮子座,互联网,音乐,阅读,90 后,营销,微博,游戏
朴灿烈,吴世勋,exo,旅行,听歌
金融,科技,电影迷,时事,美食,自由,旅游
旅行,水瓶座,电影,NBA,羽毛球,音乐
教育就业,星座命理,IT 数码
美女
新闻趣事,旅游
军事,娱乐
健康,旅游,美食
失物招领,生活部,生活维权部,木棉菁英,广东工业大学,广工,计算机学院,学生会
娱乐,健康,文艺,美食,旅游
文艺,娱乐,美女,IT 数码,体育,单纯
文艺
时尚
唱歌,交友
汽车,旅游,体育,美女,游戏动漫
汽车
军事,教育就业,投资理财,新闻资讯,文艺
文艺,教育就业,健康,星座命理,娱乐
娱乐,旅游,健康,时尚,手工,diy,网购,美食
旅游,娱乐
亲情,幽默
美女
健康,旅游,星座命理,美食,时尚,娱乐
萌宠
美食,游戏,篮球,幽默,旅行,羽毛球,狮子座
看书
NBA
水瓶座
教育就业,娱乐,体育,健康,游戏动漫,旅游,美食
娱乐
做我自己,柯南,双子座,音乐,偶喜欢玩,平常心,体育,吃,囧,娱乐
旅游,星座命理,娱乐,双子座,八卦,吃货,音乐,睡觉,白羊座,语录
时尚,投资理财,新闻资讯,旅游,美食
酸奶控,范范迷,粗线条,坏脾气,学生,游泳,摄影,纯音乐,率真
时尚
健康,星座命理,旅游,美食,娱乐

名人明星
健康,投资理财,军事
时尚
娱乐
星座命理
女,女装,女装服,女装服装,服装批发,服装店,服装
旅游
美食
时尚,星座命理,娱乐
其他生活
看电视,喜欢音乐,双重性格,旅行,网购
石家庄生活,新闻趣事,创新的思维,实力代表一切,策划,媒体,户外广告,设计
白羊座,宅,90 后
游戏动漫,美女,IT 数码,投资理财,汽车
搞笑幽默,新闻趣事,天平座,探险,读书,轻音乐
情感生活,微博奇葩
教育就业,旅游,健康,娱乐,新闻资讯,投资理财
游戏动漫,音乐,左溢,溢声军,彩虹糖,冰糖,朱元冰,白羊座,RTA
美食,旅游,星座命理
平常心,幽默,时尚,水瓶座,搞笑,睡觉,自由
囧,奋斗,爱幻想,爱美食,文艺,娱乐,旅游,美食
IT 数码
文艺,旅游,时尚,投资理财,健康,纠结
搞笑幽默
新闻资讯,美女,军事
天秤座
爱音乐,喜欢旅游,爱自由,吃喝玩乐,学生,旅游,健康,星座命理
健康
娱乐
美图摄影,体育
宅女,听歌,90 后,吃,学生
美女,游戏动漫,双子座
时尚,美食,IT 数码,娱乐
投资理财
微博奇葩,萌宠,其他生活,穿衣美容,美女
汽车
时尚,美食,体育
美女,游戏动漫,健康
健康,星座命理,文艺
体育
美食
健康,汽车,军事
娱乐
体育

旅游
娱乐
文艺,娱乐,星座命理,旅游,美食
军事,游戏动漫,投资理财,文艺,旅游
旅游,IT 数码,新闻资讯,军事,体育
电影
学习,时尚
美食,美女,IT 数码,汽车,体育
幽默,自由,唱歌,听歌
星座命理
IT 数码
慢慢走吧
文艺
电影,生活,自由职业,好性格,水瓶座,部队,摄影,80 后,好书,旅行
宅男
微博奇葩
吃货
旅游,美食,军事,美女,篮球，跑步，等
美女
IT 数码
游戏动漫,文艺,娱乐,体育
娱乐,美食,旅游,IT 数码,时尚
娱乐
美食
体育,学生
娱乐,萌男控，追星族
体育
旅游
娱乐,宅,80 后,神经大条,强迫症,任性,天蝎女,吃货
娱乐,星座命理,时尚
视频音乐
娱乐,健康,旅游,美食
旅游,IT 数码,文艺,体育,娱乐
文艺
美女
娱乐
程蝶衣,哥哥,张国荣,重莲,瓶邪,王爷,肖倾宇,河图,公子,古风
娱乐,体育,IT 数码,军事,美女
搞笑幽默
遂宁生活
星座命理,旅游
音乐,网游,旅行
时尚,娱乐,文艺,IT 数码
娱乐

萌
体育,文艺
霸气,影視,汽车,美食,音樂,旅行,艺术,时尚
石家庄生活,文艺
校园,学生
时尚,美女,旅游,美食,娱乐
游戏动漫
爱电影,爱旅游,爱看书,爱听音乐,孩子王
新闻,网络,手机,IT 数码
时尚,美食,健康,旅游,星座命理
动漫
投资理财,健康,时尚,IT 数码,娱乐
文艺
美食,小說控,海贼迷,白羊座,乐观
搞笑,营销,金牛座,旅游,好性格,90 后,好书,文字
搞笑幽默
时尚,上网,手机,听歌,90 后,音乐,睡觉,生活
唯 13,SUJU,艺旭王道,金钟云是命
单身,微博控,减肥,自拍,发呆,看电影
娱乐
旅游
单身,阳光,IT 数码
神起正装控,外貌协会,射手座,苦逼学生党,仙后,东方神起团饭,腹黑爱发呆
文艺,体育,健康,新闻资讯,娱乐
美食,看电视,旅行,摩羯座
文艺,时尚,旅游
游戏动漫,娱乐,星座命理
娱乐,处女座,宅,90 后,自由,唱歌,上网
八卦杂谈
时尚,健康
娱乐
文艺
有点小脾气,k 歌,街舞,娱乐,旅游,美食
美女,健康
旅游,星座命理,美食,时尚
美女,好性格
时尚,娱乐,旅游,健康,美食
健康,娱乐,新闻资讯,美食,旅游,星座命理
健康,旅游,文艺,娱乐,汽车
网购,娱乐,处女座,羽毛球,自由,平常心,旅行,手机,好性格
健康,文艺,旅游,美食,交友,金牛座,90 后,小说,紫色,乐观
游戏动漫,健康,娱乐
读书分享
旅游,幽默,唱歌

福建生活
军事,IT 数码,文艺,新闻资讯,健康,体育,娱乐
校园
善良,大学生,天秤座,爱吃,爬山,台球,music,90 后但我不脑残,NBA,微博
80 后
体育,文艺,新闻资讯,娱乐
囧,幽默,电子商务,宅女,羽毛球,亲情,平常心,网购,90 后,营销
旅游,健康,新闻资讯,投资理财,汽车
学生一族,随性,90 后但我不脑残,简单,天蝎,努力
美食
旅游
文艺
游戏动漫,教育就业,体育,IT 数码
体育,IT 数码,汽车,天蝎男,偶稀饭睡觉觉,增特奥迪
IT 数码
文艺
新闻资讯,娱乐,旅游,投资理财
健康,旅游,体育,美食,娱乐
军事,美食,娱乐,时尚,美女
美女
喜欢鹿晗,比较追星,比较开朗
善良,吃货,音乐,幽默,浪漫,自由职业,偶喜欢玩,美食,偶稀饭睡觉觉,旅游
投资理财,星座命理,新闻资讯,时尚,娱乐
旅游,美食,娱乐
娱乐
时尚,投资理财,健康,美食,娱乐
汽车
时尚
游戏
投资理财,健康,新闻资讯,旅游,美食
生活,宅,服装,女装网店,女装批发,女装潮流,女装店主,女装零售,艺术
娱乐,游戏动漫
数码,手机,吃货
军事,游戏动漫,IT 数码,娱乐,体育
体育,美女
时尚,娱乐
美女
好书,看书,旅行,你不玩生活,生活就会玩你
中二病患者,Monk 和尚,KENN 王爷,cosplay,火影忍者,仙剑,瓶邪,游戏动漫,萌宠,星座命理
娱乐,时尚,旅游,星座命理,美食
文艺
卷福大表姐,英剧美剧,sk8er,魔方,滑板,权力的游戏,基本演绎法,梦龙钱婆霉霉,冰与火之歌
IT 数码,时尚,健康,旅游,美食

时尚,IT 数码,新闻资讯,健康,汽车,旅游,娱乐
金浩森,廖劲锋,权志龙,天蝎座,Bigbang
娱乐
文艺,旅游美食爱好者
搞笑幽默
电影,网购
文艺,时尚,美食,健康
汽车,体育,娱乐,美食,微博控,阳光,篮球爱好者,学生一族
游戏动漫,IT 数码,体育
健康
睡觉
体育,睡神
游戏动漫,汽车,IT 数码,娱乐
时尚,美食,健康,星座命理,娱乐
游戏动漫,IT 数码,汽车,教育就业,投资理财,旅游,星座命理,文艺,体育,娱乐
星座命理,是我一生的宿命
星座命理,喜欢孙艺洲
娱乐
星座命理,美食,时尚
游戏动漫
汽车,时尚,娱乐
教育就业,时尚,文艺,娱乐
IT 数码,哈尔滨,武汉,艺术
美图摄影
娱乐,IT 数码
美女,体育
美食
美女
自由
娱乐
游戏动漫
星座命理,美食,教育就业,娱乐,游戏动漫
手机,大学生,看书,网购,宅女,好性格,白羊座,生活,唱歌,吃货
微博奇葩,新闻趣事,搞笑幽默
搞笑幽默
名人明星,摄影,睡觉,看书,电影,吃货
情感生活
美女,体育,旅游,新闻资讯,汽车,投资理财,IT 数码,军事
健康,新闻资讯,军事,汽车
睡觉
吃货,美食,旅游,80 后,电影,音乐
抚顺生活
健康,投资理财,美食
娱乐,电影,小说,双子座,文学,唱歌,吃货,好书,动漫,搞笑

美食,汽车,文艺,时尚,投资理财
娱乐,时尚,汽车,体育
娱乐
其他生活
手机,校园,微博,水瓶座,睡觉,平常心,偶喜欢玩,宅
吃货,巧克力,处女座,90,笑,李弘基,爱美食,爱旅游
开心,双子座,123,吃,80 后
体育资讯
体育,IT 数码,娱乐,时尚,旅游
时尚,美食,娱乐,金牛座,90 后,睡觉,上网
看电视,喜欢旅游,开心,随性,爱电影,爱旅游,体育
体育,军事,旅游,美女,汽车
投资理财,新闻资讯,旅游,美食,文艺
爱旅行,教育就业,投资理财,美食,军事,IT 数码
娱乐
文艺
旅游
IT 数码,搞笑幽默,Unchangeable,sun,and,the,moon,time,of,same
新闻资讯,美食
动漫,摄影
娱乐,旅游,健康,美食,星座命理
娱乐
体育,娱乐
搞笑幽默
娱乐
星座命理,美食,文艺,娱乐,旅游,心情,唱歌,宅,自由,旅行
游戏动漫,IT 数码,美女
湖北生活,旅游,美食,微博奇葩,穿衣美容
美女
旅游
汽车,时尚,文艺,旅游,星座命理
投资理财,新闻资讯
上海生活,自由
自信
汽车,美女
水瓶座
旅游,萌宠
幽默
娱乐
读书分享
逛街,海贼王,开朗,星座
旅游,文艺
旅游,健康,星座命理
IT 数码

射手座,自由职业,80 后,阅读
健康
吃,阳光
名人明星
天秤座,动漫
艺术,随性
大学生了,篮球,90 后,创业,股票,双子座,运动,大学生,传媒,旅游
善良,美食,娱乐,电影,唱歌,学生,听歌,水瓶座,90 后,旅行
动漫,妖精的尾巴
游戏动漫,电影,网络,篮球,羽毛球,玩儿,90 后,摩羯座
爱狗,爱猫,我就是我
90 后,吃,大学,学生,玩儿,双子座,电影,小说,听歌
IT 数码
IT 数码,动漫
巧克力,旅行,爱睡觉,心理学,爱旅游
时尚,美食,旅游,娱乐
广东生活
K 歌,游山玩水
穿衣美容
旅游,娱乐,文艺,时尚
旅游,美食,时尚,教育就业,娱乐
游戏动漫,美女
美食
旅游,水瓶座,偶稀饭睡觉觉,音乐,吃货,搞笑,玩儿
旅游
IT 数码,美女,游戏动漫,娱乐,军事
健康,体育,文艺
南宁生活,美食,旅游,新闻趣事,星座命理
IT 数码,文艺,健康,旅游,投资理财,美食
体育,文艺,娱乐,时尚
搞笑幽默,广东生活
美食
萌宠,旅游,体育
沉默不是我的错,旅行,吃货一枚,90 后非脑残,愤青,爱美食,懒人,巧克力,法国,双鱼座
投资理财,文艺,美食,IT 数码,时尚
时尚,汽车,旅游,军事,体育
投资理财,天蝎座,书籍,有梦想爱幻想,超级个性者,海贼控,喜欢写点小东西,睡不醒星人,美食,旅行
时尚,健康,旅游
IT 数码
生活,幽默,旅游,玩儿,听歌,乐观,90 后,偶喜欢玩
星座命理
白羊座
时尚,健康,文艺,美食

星座命理,时尚,美食,旅游,娱乐
星座命理,投资理财,新闻资讯,美女,军事,IT 数码
美女
体育
名人明星
美食,视频音乐,名人明星,旅游,搞笑幽默
游戏动漫,IT 数码,文艺,好书,互联网,动漫,听歌,电影
娱乐
语录,创业,旅游
爱情,网络
军事,投资理财,新闻资讯
娱乐,吃货,天秤座,80 后
体育
投资理财,时尚,健康,美食,星座命理,吃货
文艺,IT 数码
军事
旅游
文艺
文艺,健康,时尚,娱乐,旅游
搞笑幽默,新闻趣事,穿衣美容,偶喜欢玩,旅游,偶稀饭睡觉觉,交友,玩儿,浪漫,吃货
情感生活
美女,IT 数码
星座命理
微博奇葩
营销,85 后,书籍
体育,娱乐
IT 数码,教育就业,健康,美食,游戏动漫
美女
旅游
教育就业
科幻,天秤座,上网,随性,学习,电影迷,85 后,IT 数码
娱乐
搞笑幽默,美图摄影,苏州生活,IT 数码,体育,娱乐,文艺,时尚
健康
读书,自由,创业
IT 数码,体育
音乐,网络,语录,手机,动漫,90 后
文艺,军事,IT 数码,汽车
旅游
新闻资讯,时尚,军事
文艺,健康,旅游,美食
旅游,体育
情感生活,搞笑幽默,美食,视频音乐
传媒策划

时尚,旅游,IT 数码,美食,娱乐
幽默,羽毛球,旅行
军事,新闻资讯,体育
旅游,文艺,游戏动漫
游戏动漫
爱情心理咨询师,睡觉,音乐
体育
教育就业
体育,汽车,旅游,美食,军事
金牛座 MM,吃货,网络,80 后,美女,善良,读书,乐观,网购,旅行
汽车,宅女,吃,天蝎座,90 后,看电影,海贼王,听音乐,文艺,健康
旅游
娱乐,星座命理
旅游
IT 数码
时尚,体育
娱乐,IT 数码,美食,文艺
时尚,文艺,娱乐
星座,美食
旅游
搞笑幽默
穿衣美容
教育就业,投资理财,娱乐,健康,玩儿,生活,音乐
萌宠
巨蟹女
平常心,阅读,听歌,80 后,低调
微博奇葩
运动,篮球
娱乐,FERHFGDCNJ
文艺,教育就业,IT 数码,美女
体育,旅游,游戏动漫
新闻趣事
冷笑话,简单,爬山,强迫症,笑话,宠物,淡定,猫控,娱乐,旅游
教育就业,汽车,时尚,旅游,IT 数码
搞笑幽默,穿衣美容,名人明星
视频音乐,旅游,萌宠,搞笑幽默
娱乐,文艺,健康,旅游,时尚
喜爱下国际象棋
教育就业,文艺,老外汉语班,对外汉语
爱狗狗爱猫猫
娱乐,健康,美食
校园生活,娱乐
文艺,娱乐,旅游,双鱼座,90 后,传媒,听歌,学生,电影,美剧
听歌,文学,吃货,善良,美食,唱歌,旅游,数码,紫色,射手座

健康,旅游,星座命理,美食,娱乐
旅游,娱乐,健康,美食
学生
新闻资讯,投资理财,军事,IT 数码
IT 数码,游戏动漫
军事,IT 数码,体育,娱乐,汽车
文艺
时尚,娱乐
黑咖啡,半明媚半忧郁,文艺青年,黑白色苦咖啡,特立独行,天秤仔子,横竖都二,娱乐自己,自由,美食
数码,动漫,体育资讯,视频音乐,校园生活
文字,娱乐,运动,微博,天蝎座,好性格,自由,浪漫,旅行
新闻趣事,体育
工作狂,广告,随性,强迫症,午饭,平常心,策划,品牌
健康,时尚,旅游,美食
娱乐,旅游,文艺,星座命理,健康
90 后
体育,教育就业,新闻资讯,美女
名人明星,小狮子
冷笑话
白羊座,偶稀饭睡觉觉
美食
时尚潮流,休闲,手机看小说,情感
旅游
美食,旅游
美食,校园生活,视频音乐,时尚,旅游,文艺,娱乐
美食
军事,新闻资讯,健康,娱乐,美女
时尚,娱乐,体育
金牛座,宅,动漫,美食爱好者,活泼开朗,孩子气,偶稀饭睡觉觉,网购,b 型血,90 后但我不脑残
娱乐
美女,搞笑幽默,其他生活
周杰伦,唐禹哲,看小说,爱幻想,魔羯座,书籍,喜欢音乐
新闻资讯,健康,旅游,美食
美食,90 后
名人明星,美食,旅游
星座命理
旅游,新闻资讯,教育就业,文艺,美食
汽车
新闻资讯,健康,旅游,星座命理,美食
美食,爱猫,白色板鞋,苦逼理科生,笛安,郭敬明,suju,张翰,五月天,旅行
游戏动漫,IT 数码,时尚,汽车
汽车

读万卷书,行万里路
IT 数码
美女,旅游,IT 数码,娱乐,美食
Bitchams,跳水,GayStar,奥运会,澳大利亚,GoldMedalist,马修米查姆,MatthewMitcham,悉尼,布里斯班
音乐
愤青,车迷,电视迷,游戏达人
IT 数码
微博奇葩
IT 数码
视频音乐
爱美食,时尚潮流
心理学,真诚,开朗,乐观,狮子座,拍照,K 歌,娱乐,文艺,旅游
台球,听音乐,向日葵,茶,fashion,旅游美食,电影,80 后,自然,双子座
时尚
宅
美食
善良,文字,写作
陈奕迅,写字,IT 数码
美女
足球,电影,湛江,毅力,建筑,盐城,细心
视频音乐,车世界,厦门生活,汽车,玩儿,音乐,娱乐,时尚,幽默
娱乐,活泼,安静,健身,金融
视频音乐,搞笑幽默
美女
济南生活,80 後,gay,医学,大学生
体育
娱乐,健康
旅游,美食,星座命理,文艺
夏目友人帐,90 後,神奇宝贝,数码宝贝,孙燕姿,四川生活,爱旅游,音樂,电影控,动漫迷
投资理财
汽车,旅游,美食,娱乐,投资理财
休闲,顺德,恋爱,嘉兴,餐饮,装饰装潢,运动,想象力
旅游,美女,微博奇葩,新闻趣事,搞笑幽默
娱乐,健康,旅游,美食,时尚
小说
新闻
汽车,游戏动漫
旅游,新闻资讯,教育就业,投资理财,文艺
帅得不明显,雄性人类,广工
健康
穿衣美容
文艺
美剧中毒,卡库,CP9,陈奕迅一生黑,养乐多粉碎机

动漫
旅游,新闻资讯,美食,文艺
旅游
双子座
文艺,美食,旅游,娱乐,汽车
搞笑幽默,视频音乐,时尚,星座命理,游戏动漫,娱乐,心理,看电视
生活,旅行,电影,音乐,90 后
咖啡,笑,钓鱼
自由职业
美食,古镇控,毫无疑问的吃货,一下雨就委屈,不安分的小情绪,骄傲的小自尊,摩羯座 AB 型血,90 后
游戏动漫
教育就业,美食,旅游,健康,汽车
体育
健康
IT 数码,军事,汽车,体育
视频音乐
80 后,乐观,睡觉,旅行,摄影,体育,听歌,处女座
微博奇葩,名人明星,福建生活,王珂王新
天秤座,睡觉
美食
时尚达人,淡定,内向,努力,游泳,跑步,旅游爱好者,登山,闷骚,工作狂
美食,旅游,穿衣美容,吃货,偶喜欢玩,文字,90 后,乐观,学生
娱乐
新闻资讯,汽车,旅游
汽车
旅游
睡觉
旅游
美食,穿衣美容,读书,动漫,电影,历史,旅游,巨蟹座,英语,运动
时尚,教育就业,美食,新闻资讯,娱乐
穿衣美容,美食,萌宠
时尚,美食,投资理财,娱乐
只做我自己!
娱乐
我就是我,宅女,美食
IT 数码,文艺
玩儿,浪漫,汽车,美食,NBA,自由职业,吃货,宅男,交友,幽默
爱幻想,霸道,文学,狮子座
投资理财
保健,金牛座,宝瓶座,骑车,恋爱,摩羯座
重庆生活
穿衣美容
投资理财,北经城,房地产开发

健康,游戏动漫,娱乐,旅游,星座命理
微博奇葩
军事,新闻资讯,旅游,美食,时尚,健康
娱乐,美食
体育
IT 数码,好书
美食
新闻资讯,时尚,旅游,美食,娱乐
90 后,爱蝴蝶结,粉红控爱 kitty,旅游,美食,娱乐
军事
学习
IT 数码,旅游,投资理财,军事,娱乐
娱乐
娱乐,时尚,健康,旅游,美食
浙江生活
美食,旅游
健康,投资理财,新闻资讯,军事,旅游
旅游,美食,IT 数码
90,les,music,吃货,电脑,淘宝控,摩羯座
射手座
汽车,旅游,美女,IT 数码,体育
网络
教育就业,娱乐,健康,旅游
90 后
文艺
娱乐
视频音乐
健康
娱乐
娱乐
美图摄影,星座运势,名人明星,游戏动漫
投资理财,新闻资讯,教育就业,游戏动漫,文艺
美女,IT 数码
音樂,汽车,游戏动漫,旅游,体育
u,smile,i
星座命理,教育就业,健康,时尚,娱乐
80 后的尾巴,写字,睡懒觉,情绪化,音樂,胡思乱想,心理学,旅游美食,疯子,打篮球
美食
健康
篮球,乒乓球爱好者,羽毛球强项,林俊杰的 fans,崇拜詹姆斯,听歌,执着,低调,潮州,体育
K 歌,IT
新井瞳,东京女子流,恋瞳癖
盐城,宝瓶座,上海,细心,法律,扬州,嘉兴,保健,柏拉图式的爱
娱乐

美食
游戏动漫,娱乐
时尚,汽车
山东生活
健康
旅游
搞笑幽默
搞笑幽默
电影迷,电脑,聊天,时事,体育
娱乐
美女
自由职业,吃货
微博奇葩,IT 数码
娱乐,美食,汽车
文艺
名人明星,微博奇葩,新闻趣事,搞笑幽默,教育就业
微博奇葩,星座命理,视频音乐,搞笑幽默,新闻趣事
新闻资讯
上海生活,旅游,萌宠,星座命理,CUBE 家族崛起!,BEAST 死忠,龙俊亨,金泫雅,张贤胜,GNA
星座命理,时尚,旅游,美食,文艺
文艺,星座命理
美女
搞笑幽默
游戏动漫
军事,体育
文艺
美食,IT 数码
IT 数码
VAE,篮球,吃,宅,大学生,射手座,看书,紫色,搞笑,体育
微博奇葩,视频音乐,美女,搞笑幽默
新闻资讯
娱乐,新闻资讯,旅游,美食
星座命理,微博奇葩,新闻趣事,视频音乐,美女
美食
文艺,娱乐
星座运势
宅女加腐女,宅女,动漫控,动漫迷,动漫
IT 数码,健康
房产
新闻资讯,娱乐
穿衣美容,微博奇葩,萌宠,名人明星,搞笑幽默
单身,旅游美食爱好者,健康,时尚,音乐,吃货,狮子座,生活,大学生
星座命理,健康,文艺,旅游,美食
文艺,时尚,美食,游戏动漫,健康,星座命理,投资理财

美食, 文艺, 娱乐
狮子座, 电影
军事
美食, 星座命理, 健康, 旅游, 娱乐
娱乐
娱乐, 唱歌, 玩儿, 生活, 白羊座, 吃货, 好性格, 搞笑, 睡觉
美女
爱幻想, 绘画, 旅游, 穿衣美容, 美食, 萌宠, 看雨听雨, 自由, 听歌, 音乐
旅游, 八卦杂谈, 美女, 校园生活, 搞笑幽默, 单身待解放, 音乐, 天秤座, 90 后
娱乐, 李斯丹妮我爱你
美食
美食, 文艺
双子座, 音乐, 吃货, 互联网, 囧, 时尚
爱看小说
娱乐
人生, 外向，爬山，服装
穿衣美容
讨厌做作!
潮流, 喜欢听歌, 文字控, 个性, 瘦身, 胡思乱想, 天蝎女, 欧美控, 音樂, 重口味
教育就业, 美食, 娱乐, 健康
文艺
旅游, 娱乐, 体育
动漫, 80 后, 广告, 游戏, 玩儿, 听歌, 美剧
体育
体育, 游戏动漫, 娱乐
听歌, 摄影, 唱歌, 旅行
新闻资讯, 教育就业, 文艺
校园生活, 美图摄影, 视频音乐, 昭通生活, 美剧, 乡村, 摇滚, 吉他, 文字, 篮球
公益, 交友, 搞笑, 幽默, 广告, 摄影爱好者, 80 后, 旅行, 设计, 好书
文艺, 旅游, 美食, 娱乐, 时尚, 网购
娱乐
星座运势, 新闻趣事
娱乐
健康, 旅游, IT 数码, 美食, 教育就业
星座命理, 时尚, 文艺
搞笑幽默, 新闻趣事, 视频音乐, 微博奇葩, 美女
儿童英语阅读, 英语演讲, 读书, 阅读, 少儿英语教育, 典范英语, 英语剧
美食
紫色, 校园, 90 后, 大学
娱乐, 教育就业, 投资理财, 旅游, 健康
娱乐
时尚, 汽车
찬백당, 音乐控, 灿白, EXO, SMTOWM

娱乐
时尚,IT 数码,体育
旅游
娱乐
美食,IT 数码,时尚,教育就业,健康,豆豆妈妈
旅游
看书,听音乐,发呆,动漫,衰人
金融,吃喝玩乐,美食控,媒体,数码,电子商务,互联网,玩儿,历史,艺术
我就是我,美食,健康,文艺,时尚
善良,射手座,自由,时尚,篮球,运动,好性格,娱乐,爱情,电影
旅游,美食
萌宠,美食,穿衣美容,微博奇葩,搞笑幽默
娱乐
健康,投资理财
旅游
善良
旅游,平常心,运动,音乐,数码,阅读,汽车,大学,足球,天秤座
美女
娱乐
体育
穿衣美容
旅游,名人明星,视频音乐,星座命理,浙江生活
时尚,旅游,美食,IT 数码,汽车,娱乐
娱乐,有时吧,像个男生,电影,处女座
健康,文艺,游戏动漫,星座命理
吃货,猫控,旅游,美食
学生,白羊座,睡觉,语录
体育
搞笑幽默,微博奇葩,萌宠,旅游,美女
美食
射手座,吃货,水瓶座
IT 数码,新闻资讯,时尚,星座命理,美女
体育资讯,旅游,新闻趣事,美女,搞笑幽默
新闻资讯,文艺,时尚,美食,娱乐
汽车,文艺
情感生活
吉他手,吉他爱好者,美女
音乐,90 后,自由,吃,双子座
旅游
新闻趣事
娱乐
书，乐观
旅游
投资理财,健康,星座命理,文艺,娱乐

新闻资讯
腐女,萌宠,穿衣美容,美女,看书,睡觉,洗澡
搞笑幽默
健康,美女,娱乐,时尚,汽车
教育就业,汽车,旅游,IT 数码,娱乐
文艺
健康,美食,旅游,体育,时尚,汽车,娱乐
舞台设计,舞台灯光,舞台,舞蹈,传媒,表演艺术,演艺公司,时尚,文艺,娱乐
健康,新闻资讯,汽车,旅游,投资理财
美女
梁静茹,吃货,新闻,历史,睡觉,看书,音乐,唱歌,学生,艺术
游戏动漫
文艺
游戏动漫
旅游
星座运势
幽默
星座命理,旅游
教育就业,新闻资讯,健康,投资理财,时尚
游戏动漫
唱歌
新闻资讯,教育就业,时尚,美女,体育
体育
娱乐,体育
排骨精,忠实的歌晨党,Kpop 控,天枰座 O 型女,90 后
吃,美女,旅游,电影
营销,自由,音乐
自由,偶稀饭睡觉觉,吃货,听歌,旅行,摩羯座,宅女,80 后
时尚,体育,教育就业,健康,汽车
文艺,时尚,旅游,健康,美食
游戏动漫,娱乐,汽车,文艺
简单的幸福,娱乐
随性,开朗,爱睡觉,冷笑话
爱唱歌,小忧郁,爱笑,白羊座
河南生活
时尚
星座命理,美女,Music,打篮球,体育,文艺,娱乐
幸福,服装,完美主义,真实力,IT 数码,美女,时尚,汽车,新闻资讯
文艺,留学,育儿
心情语录,绝美语句,唯美语句,唯美语丝
文学,奋斗,读书,自拍,可爱,旅游美食,浪漫,财经,会计,娱乐
体育
名人明星,美食,星座命理,微博奇葩,搞笑幽默
北京生活

穿衣美容
萌宠,名人明星,文艺
幽默
文艺,好书,处女座
偶稀饭睡觉觉,听歌,交友,宅男,自由职业,偶喜欢玩,美女,爱情,吃
健康,教育就业,美女,体育,娱乐
星座命理,军事,文艺,娱乐,时尚
娱乐
名人明星,银川生活
自拍,快乐,美食控,互粉,O 型血,Music,爱旅游,爱电影,爱笑,青春
军事,汽车
美食
娱乐
时尚,星座命理,美食,文艺,娱乐
灿白洁癖,exo,吃货
健康,旅游,星座命理,娱乐
永远睡不醒。。。,饿了就不开心。。
健康,旅游,IT 数码,文艺,体育
搞笑幽默
音乐
历史
电影圈工作者,初心韩小胖,煎蛋凡凡么么哒,萝莉御姐
爱自己,彩妆,偏执狂,努力,时尚,经济,篮球爱好者,美剧,大学
搞笑幽默,星座命理
旅游
微博控,娱乐,上网,青春,爱睡觉,电视剧,团购,淘宝,购物,广州
时尚
健康,星座命理,文艺,娱乐,小清新,90 后,双鱼座
写作,旅游,阴晴不定
文艺,教育就业,健康,旅游
音乐,睡觉,巨蟹座,上网
科学探索
臭写字的,死画画的
投资理财
IT 数码
心情,服饰
投资理财,美食,IT 数码,娱乐,汽车
娱乐
相互关注,五月天,射手座,MJ,旅行,单车,网购,无愁,无绪
手机,90 后
读书分享
自由,宅,亲情,学生,搞笑,双子座
娱乐
星座命理,美食,善良,自由,听歌,旅游,80 后

幽默,上网
健康,游戏动漫,娱乐
美食
视频音乐,美食,旅游,穿衣美容,星座命理
时尚,星座命理
搞笑幽默
美食
宠物图集,NBA,设计,时尚,运动,美剧,电影,吃货,英语,乐观
宠物图集,视频音乐,美容服饰,新闻趣事,美食
旅游,大叔控,高三,随性
小说,传媒,新闻,网络,财经,媒体,摄影
美食,感性,强迫症,完美主义,水瓶,偏执狂,双重性格,随性
娱乐
旅游,军事
星座命理,旅游,美女,游戏动漫
名人明星
健康,文艺,体育,娱乐
投资理财
穿衣美容,新闻趣事,视频音乐,搞笑幽默,美女
音乐控
时尚,娱乐,旅游,文艺
睡觉睡到自然醒,NBA,网络,手机,体育,美女,娱乐,IT 数码
体育
创业
处女座,巨蟹座
美女,自由职业,自由,好性格,时尚,80 后
高三,浪漫,心情,好性格
梦想,偶喜欢玩,相貌控,桌球,文字控
江苏生活,时尚,汽车,健康
游戏动漫
旅游
游戏动漫,体育
体育
射手座,EXO
汽车,教育就业
军事
自然卷控,电波女,冒失娘,中二,宅女,学生,看书,巨蟹座,双子座,90 后
穿衣美容
健康,体育
娱乐,文艺
文艺
旅游,时尚,文艺,娱乐
愤青,淘宝控,柯南,外貌协会,创业
体育,IT 数码,时尚,星座命理,亲情,处女座,好性格,紫色,宅女

天津生活,胡思乱想,狮子女,美食
旅游,星座命理,美食,健康,时尚
河南生活,萌宠,微博奇葩,穿衣美容,淘宝
情感生活,娱乐
美女,美食,旅游
搞笑幽默,其他生活,星座命理,萌宠
DIY 扎染,水下摄影,深度大理,客栈,私家菜
旅游
疯子
旅游,星座命理,美食,文艺,娱乐
娱乐,天津快板,曲艺,相声,大鼓书,评书,民族乐器,音乐,葫芦丝,快板教学
IT 数码
旅游
教育就业,汽车,游戏动漫,体育,文艺
搞笑幽默
狮子座
投资理财,健康,睡觉,时尚,搞笑,宅女,乐观
柯南
美女,时尚
健康,旅游,星座命理
美食
体育资讯,低调,网球
时尚,电影,散步,爱幻想,购物狂,吃货,烹饪,自拍,服饰
文艺
文艺
时尚,文艺,听音乐,爱音乐,购物
健康,旅游,美食,娱乐
IT 数码
北京生活,军事
旅游,偶喜欢玩,游戏,天蝎座,篮球,摄影,阅读,手机,上网
旅游
旅游,美食,星座命理,文艺
文艺
时尚,新闻资讯,健康,美食,旅游
食肉动物,美剧狂,强迫症患者,辣手夺命小护士
教育就业,时尚
娱乐
广西生活,名人明星,穿衣美容,时尚,文艺,美食,旅游,健康
美女,旅游,娱乐
文艺,星座命理,健康
设计师
豆瓣友邻,行走的人,猫的宠物,植物花痴,音乐不听会死
娱乐,文艺
90 后,偶稀饭睡觉觉

娱乐,体育
娱乐
教育就业
文艺
无聊
健康,天秤座,电影,善良,吃货
文艺,投资理财,健康,美食,旅游
旅游,美食,体育
美食,时尚,天秤座,疯子,怪脾气,不聽话,不爱做事,喜欢一个呆家
美食
美女,星座命理,军事,体育,娱乐
新闻趣事,穿衣美容,美食
IT 数码,军事,文艺,体育,娱乐
低调,90 後,文艺,音乐控,旅游美食,学生一枚,天秤
搞笑幽默,新闻趣事,玩手机,运动
健康,旅游,星座命理,美食,时尚
新闻资讯,旅游,时尚,汽车,IT 数码
娱乐
水瓶座,睡觉睡到自然醒,心理,music
时尚达人,购物狂,孩子气,微笑,自拍,情感,化妆,网络,手机,做梦
微博,阅读,平常心,自由,音乐,白羊座,90 后
文字
园林业,慎重,杭州,搞笑,济宁,运动,美女,看书,休闲,江门
美女,游戏动漫,文艺,时尚,星座命理,娱乐
外贸童装,日单潮品童装,日单 zoomic 特卖,淘宝,败家
创业,生活,自由,设计,时尚
旅游
广东生活
太原生活,人人好
教育就业,男人
艺术工作者
星座命理,美食,IT 数码,娱乐
娱樂,射手座,囧,吃,睡觉,动漫,八卦,学生,手机,美食
小说,吃,八卦,宅,看书,80 后
娱乐
时尚,娱乐
视频音乐,搞笑幽默,美食,旅游,穿衣美容
星座命理
舞蹈,篮球,英雄联盟
游戏动漫,旅游,美食,IT 数码
情绪化
其他生活,穿衣美容,旅游,美食
IT 数码,健康,投资理财,教育就业,美食
美女

最爱 GD,爱 BB,爱尹二允,星座命理,美食,游戏动漫,文艺,娱乐
视频音乐,美女,搞笑幽默
星座命理
学生,外貌协会,书籍,水瓶女,时尚,健康,旅游,文艺,娱乐
考研,考博,强军,教育,韧达,盛世清北,清华,北大,高端辅导,985 高校
江苏生活,搞笑幽默
汽车,新闻资讯,投资理财,教育就业,文艺
游戏动漫
周杰伦,我就是我,大学生,白羊座,摄影,唱歌
街拍,文学,手机,文字,奋斗,交友,看书
游戏动漫
美食
美女,旅游,画画,2B 青年
新闻资讯,美女,文艺,娱乐,教育就业
职场招聘,娱乐
旅游,吃货
娱乐,宅,音乐,水瓶座,紫色,吃货,80 后,唱歌
唱歌,运动
羽毛球,设计师,小女人,旅行,穿衣美容,团购,时装,健康养生,购物,执着
喜欢安静的发呆
新闻资讯,旅游,星座命理,美食,娱乐,文艺
北京生活
旅游,健康,星座命理,文艺
旅游
IT 数码
体育,汽车,游戏动漫
名人明星,微博奇葩,萌宠,旅游,IT 数码,美食,娱乐,文艺,体育
听歌,美食,阅读,爱情,娱乐,旅行,80 后,双子座
黄步云
搞笑幽默,游戏动漫,IT 数码,体育
星座命理,游戏动漫
娱乐,张杰,刘忻
时尚
名人明星
时尚
小说,娱乐,听歌,自由职业,音乐,游戏,搞笑,吃货,微博,双子座
游戏动漫
军事
新闻资讯
唱歌,待解救男青年,双鱼座
吃,搞笑
微博奇葩,星座命理
财经,文学,宗教,中医,摄影
封子,封神演义,藤崎龙,封神演义仙界传

军事
彩虹,梦游者,陈奕迅,BIGBANG,睡觉睡到自然醒
视频音乐,娱乐
宅…各种宅!
娱乐
90 後,金牛座,笑话,音乐控,文学
投资理财,时尚,健康,旅游,美食
娱乐,星座命理,亲情,羽毛球,平常心,艺术,网购
新闻资讯,美女,IT 数码,投资理财,旅游,星座命理,文艺,健康,体育,娱乐
汽车
旅游,游戏动漫,健康,美食,娱乐
搞笑幽默
文艺
台州生活,娱乐
时尚
IT 数码,娱乐
摩羯女,欧美音乐控,电影,美剧,90 后,文学,听歌
睡觉,水瓶座,爱音乐,旅游,上网,努力,看电影,完美主义
IT 数码
星座命理,外貌协会,双子,爱自由,喜欢听歌,可爱
体育
游戏动漫,古剑奇谭,仙剑奇侠传,轩辕剑
广东生活
安徽生活
爱唱歌,游泳,时尚,旅游,美食
萌宠
星座命理,天秤座,摄影爱好者,羽毛球,宅男,偶稀饭睡觉觉
重感情,旅游美食爱好者,阳光，热情，直爽
小酒窝
旅游,文艺,IT 数码
新闻资讯
健康,旅游,美食,娱乐,时尚,生活,爱情,亲情,大学,设计
体育
美食,军事,美女,IT 数码,汽车
搞笑幽默,微博奇葩,旅游,视频音乐,星座命理
星座命理
旅游美食,发呆,爱音乐,文学,跳舞,钢琴,写字,文艺,画画,音乐，写作，看书
电影爱好者,诗词,音樂,爱情,文学,健康,旅游,文艺,教育就业,娱乐
淘宝控,减肥,服饰,彩妆,欧美控,穿衣美容,韩国,美容护肤,时尚潮流,健康
娱乐
市场,理性
IT 数码,旅游,视频音乐
旅游,星座命理
新闻趣事

江苏生活,烹饪,体育
美食,旅游,健康,时尚
街舞,电影
美食
不了解自己,运动,音乐,小说,大学生,睡觉,巨蟹座
宅
游戏动漫,娱乐
新闻趣事
游戏动漫,星座命理,美食,上网,偶稀饭睡觉觉,穿越火线半职业,双子座,90后
IT数码,健康,新闻资讯,文艺,体育,娱乐
投资理财,新闻资讯
汽车,自由,旅行
旅游
美女,健康
视频音乐
游戏迷
双鱼座,文艺,时尚
新闻资讯,投资理财,文艺,旅游,体育
旅游,健康,娱乐,文艺,星座命理
旅游,美食,军事,IT数码,体育
时尚,健康,投资理财,美食,星座命理
90後,体育
视频音乐
旅游
视频音乐,穿衣美容
美食,萌宠,美女,视频音乐,穿衣美容,神经质,强迫症,心理学,宠物
穿衣美容
IT数码
健康,新闻资讯,旅游,星座命理
猫咪,摩羯女,宅,摄影,唱歌
搞笑幽默,新闻资讯,汽车,IT数码
微博奇葩,美女
娱乐
新闻
娱乐
其他生活,旅游,搞笑幽默,穿衣美容,新闻趣事,教育就业,时尚,新闻资讯,健康
旅游,时尚
美食,淡定
时尚,文艺
美食,文艺
IT数码,美食,娱乐,时尚,文艺
八卦杂谈
游戏动漫,健康
Britney,网王

投资理财,军事,体育,娱乐,游戏动漫,旅游,IT 数码
美女
健康,军事
微博,娱乐,听歌,90 后
穿衣美容
美女
娱乐,文艺
旅游
IT 数码,吃睡觉,爱吃
美食
娱乐
美女
穿衣美容
摄影爱好者,时尚,爱情,手机,电子商务,自由,处女座,音乐,搞笑,双子座
娱乐
美食
旅游,美食,美女,星座命理,穿衣美容
时尚
赚钱,心理学,手工,小清新,英语,新闻资讯,健康,旅游,星座命理,美食
娱乐
旅游
玩儿,投资理财,健康,旅游,娱乐,文艺
saber,全职猎人,犬夜叉
游戏动漫
娱乐
投资理财
时尚,健康,旅游,星座命理,美食
汽车,赚钱,爱生活,愤青,强迫症,shopping,爱旅游,德国,神经大条
旅游
美女
读书,新闻,写作,玩儿,射手座,美剧,生活,天秤座,幽默,音乐
娱乐,健康,投资理财,时尚,美食
星座命理,吃货,～神经～大条
新闻资讯,游戏动漫,健康,美女,体育,娱乐,军事,IT 数码,文艺
音乐,天秤座,旅游
星座命理
旅游,星座命理,新闻资讯,美女,美食,军事,IT 数码,文艺,体育,娱乐
轻松熊奶油熊,巧克力吃多头晕,你要知道我闷骚,强迫妄想中二病,初音未来 miku,偶尔无聊
DIY,我腐不是我的错,小提琴小治愈,森女部落清森系,漫画家高木直子
时尚
娱乐
动画,睡觉睡到自然醒,笑话,宠物,恐怖片,快乐大本营,健康,旅游,美食,娱乐
文艺,偶喜欢玩,八卦,听歌,学生,校园,紫色,偶稀饭睡觉觉,动漫
美食,时尚,游戏动漫,文艺,健康

星座命理
郑州生活,爱
旅游
视频音乐
美食
美女,IT 数码,科学探索,车世界,新闻趣事
旅游
90 后,偶稀饭睡觉觉,吃,宅女,亲情,大学,宅,校园,自由,音乐
健康,新闻资讯,军事,体育,IT 数码
时尚
旅游,健康,时尚,文艺,娱乐,二货,音乐控
自由职业,囧,天秤座,唱歌
看小说,吃喝玩乐
时尚
手机,宅,音乐,校园,学生,游戏,体育
微博奇葩
宅
金牛座
娱乐,美食,星座命理
玩儿
文艺
吃货,上网,90 后,偶喜欢玩,睡觉,娱乐,唱歌,天蝎座,宅女,看书
金牛座,时尚,平常心,汽车,艺术,摄影爱好者,广告,媒体,旅行,80 后
偶稀饭睡觉觉,校园,学生,巨蟹座,读书
旅游,健康,汽车,美食
军事,娱乐
娱乐,美女,宅男,金牛座,时尚,宅,唱歌,艺术,搞笑,音乐
教育就业,星座命理,时尚,文艺,娱乐
美食,微博奇葩,星座命理,穿衣美容,搞笑幽默
汽车,IT 数码
美食
芜湖国际快递
文字,最亲爱的女孩儿
汽车,新闻资讯,健康,旅游,美食
向日葵,小女人
健康,旅游,文艺,体育,IT 数码
旅游,健康,美食,汽车,教育就业,投资理财
名人明星,视频音乐,校园生活,搞笑幽默,西安生活
旅游
新闻资讯
自由职业,电子商务,网络营销,it 互联网
娱乐
搞笑幽默
美食

搞笑幽默,视频音乐,旅游,微博奇葩
溜冰
信佛,朋亲
90 后但我不脑残
美容服饰,明基模力女孩,时尚
保护动物反虐杀,潮流時尚
active,talent,student
IT 数码
万紫千红窗帘
上海生活,游戏动漫
运动,学生,狮子座,羽毛球,听歌,音乐
娱乐,时尚,健康,文艺,体育
美食,娱乐
美食,新闻资讯,美女,IT 数码,时尚,娱乐,购物
游戏动漫,网游,Music,IT 互联网,青春,游戏,WordPress,博客,网络,设计
健康
新闻趣事,微博奇葩,视频音乐,旅游,四川生活
美食
旅游
时尚,文艺
搞笑幽默,军事,文艺,投资理财
新闻资讯,投资理财,旅游,美女,汽车
体育
诗词,看小说,時尚,电影迷,星座命理,军事,健康,旅游,娱乐,美食
视频音乐,新闻资讯
美女,音乐,篮球,情种,忧郁王子,情歌王子,军迷,police,疯子,迷一样的男人
旅游,法国,80 后,红酒,美食
星座命理,搞笑幽默,新闻趣事,穿衣美容,广东生活
星座命理,美食,娱乐,唱歌,舞蹈,玩
庄培贤
体育,娱乐,教育就业,IT 数码,游戏动漫
巨蟹座,起床困难户,小宅女,单身待解救,偶稀饭睡觉觉
湖州生活
自由职业,体育,旅游,新闻,好书,自由,生活,美女,历史,80 后
旅游
旅游,完美主义,90 后但我不脑残,旅游摄影,双鱼座
美食
美食
80 后,善良
投资理财
游戏动漫,时尚,IT 数码,健康,新闻资讯,文艺,体育,娱乐
汽车,游戏动漫,美女,旅游,娱乐
IT 数码,文艺,娱乐,体育
穿衣美容

文艺,星座命理,美食,军事,健康,体育,娱乐,汽车,教育就业,投资理财
学生
汽车
娱乐,张根硕控
投资理财,教育就业,时尚,健康,旅游
乐观
星座命理,情绪化,心理,语录
体育
娱乐,IT 数码
时尚,健康,旅游,娱乐
旅游,投资理财,时尚,军事,美食,星座命理,美女,IT 数码,娱乐
美容服饰
80 后
新闻趣事,军事,游戏动漫
健康,旅游,新闻资讯,时尚,美食
旅游,萌宠,穿衣美容,视频音乐,搞笑幽默
军事,新闻资讯,教育就业,体育,游戏动漫
文艺,IT 数码,军事,体育,游戏动漫
星座命理
星座命理,游戏动漫,IT 数码,文艺,娱乐
星座运势,校园生活,星座,情感
视频音乐,睡觉,旅游,看书
名人明星,星座命理,萌宠,微博奇葩,娱乐
美女
汽车,IT 数码
汽车
爱人如己!,随和,被动者,弹吉它,基督徒,自恋,安静,交友,喝酒,唱歌
时尚,星座命理,健康,文艺
汽车,文艺,娱乐,游戏动漫,美女
娱乐
娱乐,体育,80 后,篮球,音乐
计算机,好性格,创业,电脑,阅读,教育就业,音乐,摄影爱好者,电影,读书
硫酸镁,硫酸铵,硫酸亚铁,农业级硫酸镁,工业级硫酸镁,医药级硫酸镁
体育
营销,八卦,旅游,睡觉,自由职业,听歌,浪漫,善良,狮子座,偶喜欢玩
星座命理,文艺,娱乐,美食,旅游
教育就业,时尚,健康,旅游,文艺
动漫,美食,旅游,吃,看书,天蝎座,90 后,校园,好书,娱乐
好书,时尚,英语,娱乐,电影
开心果,90 後,听歌,音乐
穿衣美容,90 后,狮子座
时尚,巨蟹座 B 型血,硕士研究生,逛街达人,舞蹈达人,馋嘴妞,新鲜有趣的东东
时尚
时尚,娱乐,美食,旅游,星座命理

时尚,娱乐
美食,健康,时尚,投资理财,娱乐,旅游
旅游
美女,平常心
林志颖,双重性格,柯南,我就是我,星星,双子女,吃货,偶像剧,爱玩,shopping
投资理财
旅行,校园,幽默,篮球,写作,创业,双鱼座,交友,大学,听歌
投资理财,健康,文艺
文艺
旅游,娱乐
游戏,搞笑,娱乐,时尚
美女
篮球
时尚,星座命理,娱乐
吉他,学生
投资理财,IT 数码
体育
旅游
体育,陈升
旅游,投资,慢走
湖南生活,视频音乐,微博奇葩,名人明星,萌宠
体育,我是猪头,吃货,篮球,数码,善良,80 后
体育
时尚
幽默,运动,美食,音乐,感性
阅读,善良,旅行,上网,运动,美食
时尚,星座命理,天平座,娱乐,摄影爱好者
健康
游戏动漫,娱乐,星座命理
星座命理
听歌
投资理财,教育就业,旅游,IT 数码,文艺
旅游,美食,文艺
其他生活,美图摄影,八卦杂谈,搞笑幽默
美女,文艺,游戏动漫
数码,写作,旅游,善良,平常心,好性格,篮球
摩羯座,双鱼座
娱乐,美食,街舞,舞蹈,摩羯座,动漫,吸血鬼日记,随性,法国
搞笑幽默
体育
旅游
星座命理,旅游,视频音乐,穿衣美容,美食
旅游
时尚

游戏动漫,时尚,旅游,美食,文艺
教育就业,旅游,IT 数码
视频音乐
文艺
车世界,美图摄影,旅游
健康,时尚,娱乐
星座命理,旅游,美女
旅游,健康,美女,美食,IT 数码
IT 数码,军事
搞笑幽默,游戏动漫,娱乐,IT 数码
韩剧,第三医院,崔秀英,金承佑,吴智昊,金敏贞
旅游,美食,萌宠,偶稀饭睡觉觉,善良,双鱼座,听歌,大学生
微博奇葩
八卦杂谈
汽车
巨蟹座,吃,看书,文字,睡觉,90 后,电影,宅女,小说,音乐
天蝎座,90 后,吃货,偶稀饭睡觉觉,旅行,搞笑,紫色,宅,学生,善良
文艺,娱乐,时尚,IT 数码,体育
双鱼座,逍遥,坐忘,心斋,归零,艾儿悦吟心
教育就业
蘑菇星人,学生
新闻资讯,汽车,时尚,旅游,美食
星座命理,娱乐,90 后,吃货,动漫,大学生,小说,学生,乐观,双子座
时尚,健康,星座命理
旅游,星座命理,健康,教育就业,投资理财
MAYA 服饰,床上用品,富豪新村
旅游,教育就业,星座命理,投资理财,健康,网购,旅行,天蝎座
最爱猫咪
搞笑幽默
娱乐,旅游,健康,美食,星座命理,90 后,学生
可爱善良
名人明星
军事
星星,插画,疯子,武汉,文艺,旅游
星座命理,新闻趣事,时尚,娱乐
湖人,吸血鬼日记,动漫迷,死神,猫,运动,吃货,篮球,看书,美食
交友,玩儿,动漫,大学,写作,双鱼座,音乐,90 后,时尚,电影
其他生活
狮子座 90 后
手机,新闻,好性格,篮球
文艺,娱乐,时尚
美女
星座命理
名人明星

执着、情绪化
80后,章鱼,文章,时尚,足球,历史,事迹,笑話,双子座
娱乐,不是我自寻死路,吃货,爱音乐,爱玩,我就是我,冷笑话,爱幻想
娱乐
手表爱好者,化妆品代购,美食,电影,汽车
汽车,美食,健康,IT数码,摄影,旅游,电影,阅读,IT
星座命理,游戏动漫
平面设计,投资理财
室内装饰,胡思乱想,营销,爱睡觉,创意,可爱
汽车,投资理财,星座命理,军事,IT数码
旅游,军事,体育
靠谱,天马行空,幽默,开朗,感性与理性并存,善良,娱乐,交友,足球,好性格
美女
娱乐,投资理财,游戏动漫,体育,IT数码
活着要快乐
汽车,速度与激情,公务员,80后
娱乐
名人明星
美女,游戏动漫
美图摄影,美女,视频音乐,校园生活,搞笑幽默
星座命理,美女,山东生活,健康
游戏动漫
交友,偶喜欢玩,学生,吃,听歌,微博,唱歌,旅游,金牛座,80后
投资理财
星座命理
IT数码,文艺
旅行,好性格,互联网,吃货,紫色,娱乐,幽默,汽车,宅,摩羯座
教育就业,新闻资讯,健康,旅游,星座命理,美食
音乐迷,学生一族,高考,音乐达人,交朋友,爱情,ac米兰,水瓶座
IT数码,旅游,其他生活
视频音乐,90後,k歌,外貌协会
旅游
搞笑幽默,新闻趣事,美女,旅游,美食
星座命理,美女,90后,囧
娱乐
IT数码,美女
南平生活,搞笑幽默,吃货
旅游,星座命理,娱乐,健康
星座命理,旅游,娱乐
爱旅行,护肤,多愁善感,睡觉,唱歌,上网,自由,宅,时尚,旅行
教育就业,阅读,旅游,唱歌
腐,动漫控,睡懒觉,美食
星座命理
美食,平常心,校园,英语,乐观

视频音乐
视频音乐,时尚
IT 数码
视频音乐,旅游,投资理财,体育,文艺,娱乐
简单生活简单爱
互联网
搞笑幽默,美女
美女
新闻资讯
个性,喜欢唱歌,娱乐
旅游
新闻趣事,投资理财,健康,时尚,旅游
体育
处女座,搞笑
看书
健康,旅游,星座命理,体育,娱乐
美食,美食
旅游,IT 数码
教育就业,健康,美食,星座命理
美食,吃,处女座
娱乐
重口味,天秤,随性,欧美控,原创,睡觉睡到自然醒,美食控,互粉,个性,自拍
乐观,交友,篮球
呱呱影院,最好看的电影
美食,名人明星,服装,完美主义,低调,90 後,外貌协会
动漫,视频音乐,校园生活,搞笑幽默,河池生活
90 后,工程机械,晃悠,漂泊,旅行
娱乐
游戏动漫,娱乐,体育,IT 数码,冷笑话,九零後　摇滚乐
爱情,知性女青年
视频音乐,吃货,音乐,旅游,90 后,动漫,时尚,紫色,射手座,摄影爱好者
寂寞,迷糊,学生一枚,发呆,娱乐,文艺,时尚,90 后,乐观,善良
游戏,唱歌
美食,时尚
游戏动漫,旅游,体育,IT 数码,教育就业,健康,新闻资讯,娱乐
健康,汽车,旅游,美食,星座命理
游戏动漫,IT 数码,体育
美食,文艺
星座命理,美食,旅游,手机,睡觉,旅行,动漫,娱乐
娱乐,星座命理,IT 数码,文艺,乐观,双子座,电影控,音乐,ly
体育
娱乐
娱乐,it 互联网
健康

多愁善感
娱乐,汽车,旅游,文艺
搞笑
美图摄影
画画艺术生,天秤,巧克力
汽车,教育就业,健康,时尚,美女
娱乐
健康咨询专家,官方微博
新闻趣事,体育,健康,军事
足球,白羊座,听歌,创业
美食
旅游,娱乐,时尚,健康
福建省教师招考,师范生,招考考纲公告,法律法规时事,教育综合真题,莆田代课教师,教师资格证试讲,两学普通话考试,莆田幼师招考,莆田市教师招聘
广东生活
时尚,IT 数码
家族总宣传
旅行
音乐爱好者,爱吃,可爱,睡懒觉,谢娜,时尚,健康,旅游,娱乐,美食
美食,旅行,白羊座,善良,睡觉,平常心,80 后,听歌,偶稀饭睡觉觉,音乐
投资理财,旅游
旅游,乐观,数码,音乐,90 后
投资理财
新闻资讯,旅游,美食,军事,文艺
搞笑幽默
美女
文字控,宅女一枚,欧美,留学,阳光,奋斗,justinbieber,玩儿,学生一枚,90 后但我不脑残
此微博停用
新闻趣事
游戏动漫,时尚,文艺,冷門電影,後期,攝影
旅游,微博奇葩,新闻趣事
教育就业,健康,痴呆,大头,郁郁寡欢,沉迷游戏
无厘头,音樂,音乐达人,法语
平常心
传媒
旅游
桌球,萝莉控,新闻趣事,文艺,汽车
旅游,文艺
医学美容整形,医学美容,瘦脸针,美白
体育,篮球迷,90 后,伤员,水货高手,运动成瘾
星座命理
美食,旅行,军均控,文学,简单,艺术
健康,白色控,粉紅控,音乐,旅行,吃货,善良,美食
亲情,童鞋

美食
美女
网络,爱唱歌,懒人,睡懒觉,小清新,喜欢音乐,钢琴,爱旅游,爱笑,bigbang
搞笑幽默,IT 数码
名人明星,新闻趣事,视频音乐,八卦杂谈,校园生活
爱美,k 歌,好学,财经
IT 数码,时尚,娱乐,投资理财,健康,摄影爱好者,大学生,听歌
娱乐
视频音乐,名人明星,新闻趣事,搞笑幽默,广东生活,新闻资讯,时尚,汽车,IT 数码
教育就业
娱乐,健康,旅游,美食,潮州,城基,金中,电影,星座
艺术,双鱼座,唱歌,媒体,旅行,吃
旅游
娱乐
读书分享
娱乐
穿衣美容,财经,天蝎座
动漫,校园生活,视频音乐
時尚
汽车,文艺,IT 数码,投资理财,教育就业,新闻资讯,军事
美食,娱乐,体育
普通话考试,两学考试,福建省教师招考,法律法规时事,教育综合真题,教师资格证试讲,三明市幼师招考,师范生,三明代课教师,三明市教师招聘
军事
时尚
娱乐
长春生活,名人明星,时尚
音乐,娱乐
微博奇葩,搞笑幽默,美女
健康,娱乐,投资理财,IT 数码,游戏动漫
电影控,美食爱好者,新闻资讯,娱乐,教育就业,美食,军事
游戏动漫
名人明星
时尚,娱乐,新闻资讯,旅游,美食
星座命理
娱乐
睡觉,上网,其他生活,视频音乐,搞笑幽默,IT 数码,旅游
游戏动漫,星座命理,旅游,美食,好奇猫,胆小鬼,爱哭鬼,处女座
名人明星
美食,旅游,IT 数码,健康
音乐
体育
大爱力宏,电影控,旅游美食,白羊女
汽车

创新工作坊,我们的城市,生活的准则,JA 志愿者,员工志愿者,改变明天,安斯泰来
旅游,英语
微博奇葩,星座命理
90 后,互联网,乐观,爱美控,娱乐,交友,营销,电子商务,时尚
旅游,投资理财,汽车,美食,体育
教育就业,旅游,星座命理,美食,娱乐
美食
游戏动漫
屌丝,seo 爱好者,wower,直通车车手,天猫推广,淘宝推广,电子商务,淘宝掌柜
健康,旅游,文艺,教育就业,投资理财
投资理财,旅游
媒体,美食
星座命理,music
投资理财,IT 数码,健康,文艺,教育就业
娱乐
教育就业
美女
文艺
文艺,新闻资讯,游戏动漫,IT 数码,军事
旅游,体育,滑板手套,幽默,运动,自由
旅游,健康,文艺,星座命理,娱乐
文艺,00 后,糖豆,学生
时尚
旅游,摄影
健康,体育,吸迷,数字,美食
游戏动漫,军事,文艺
时尚,旅游,文艺,体育,娱乐
体育,80 后,平常心,NBA
美女
执着,活泼开朗,傻,80 後,爱笑,非诚勿扰,懒
搞笑幽默
新闻资讯,汽车,搞笑,运动
旅游
搞笑幽默
新闻趣事,玩儿,吃,水瓶座
教育就业,美女,旅游,星座命理,军事,娱乐
旅游
汽车,体育,娱乐,金牛座,90 后,继科迷
分享,蓝色,梦想,摄影,收藏,看书,旅行
文艺,娱乐,IT 数码
美食,体育,健康,旅游,文艺
名人明星
时尚,健康,星座命理,美食,文艺
会计

美食,文艺,娱乐,创业
星座命理
名人明星
美女
上网,幽默,搞笑,英语
DOTA 爱好者,体育
新闻趣事,星座命理,美食,穿衣美容,搞笑幽默
新闻趣事
健康,军事
汽车
广州生活
其他生活
westlife,户外,狗,新闻资讯,军事,IT 数码
娱乐
旅游,娱乐,吃货,美食,90 后,白羊座,摄影爱好者,旅行
名人明星
新闻资讯
星座命理,微博奇葩,视频音乐,穿衣美容,旅游
吃喝玩乐
体育
天蝎女,球迷,低调,80 后宅女,电视
文艺,美食,旅游,健康,投资理财
旅游,水瓶座,吃货
美食,体育
名人明星,重庆生活,美食
星座命理,美食,婚纱控,漫画,动漫,爱吃,睡懒觉,旅游美食,发呆
汽车
星座命理
新闻趣事
好想在宿舍养喵,吃货大部队队员,猫咖小分队
旅游,娱乐,投资理财,军事
投资理财,新闻资讯,感恩,绽放,红楼梦,自行车旅行,青春,文学,自由,追求
张杰,笑,音乐发烧友,星座命理,娱乐
我爱容垣,萌瓶邪,爱古风,爱小说,爱玩,射手,温暖治愈系,腐女,软妹子,我承认我是吃货
美女,体育
美容服饰
视频音乐,美食,萌宠,搞笑幽默
情感生活,读书分享,校园生活,新闻趣事
游戏动漫,美女,娱乐,文艺,时尚
湖南生活,囧,A 型血,90 後,自由散人,巨蟹座
文艺
文艺,IT 数码,美食,美女,游戏动漫
健康,动漫爱好者,副业妈妈,偶稀饭睡觉觉
听歌,偶稀饭睡觉觉,美食,小说,历史,乐观,自由,水瓶座

紫色,偶喜欢玩,矛盾,好性格,幽默
旅游,设计
跳舞,吃货,紫色,蓝色,听歌,音乐
星座运势,搞笑幽默,宠物图集,美食,名人明星
爱睡觉,可爱,减肥,舞蹈,教育
旅游
美食
校园生活,搞笑幽默,娱乐
90 后,宅女,偶稀饭睡觉觉,摩羯座,学生,吃,偶喜欢玩,好性格,唱歌,音乐
旅游
狮子座
军事,娱乐,美女
健康
文艺
健康
英汉双语微言,IT 数码,新闻资讯,看电影,我就是我,林俊杰
文艺
机床工具,数控刀片,CNC,数控刀具
其他生活,IT 数码
星座运势,读书分享
游戏动漫
名人明星
汽车,音乐,运动,摄影,旅行,创业,生活,善良,幽默,自由职业
健康,体育,娱乐
广东生活
美女,体育
旅游,天蝎座,90 后
美食
公关,唯美的,装饰装潢,计算机软件,硬件
文艺
娱乐
星座命理,娱乐,投资理财
汽车
动漫
旅游,美食,求瘦身
IT 数码
黄山生活
美食,星座命理,文艺,时尚,娱乐
美女,搞笑幽默
教育就业,时尚,游戏动漫,健康,娱乐
娱乐
北京生活
娱乐,体育,NBA,哎哟,不错哦
娱乐

电影,平常心,浪漫,白羊座,宅,吃
体育,茶,90 後,爱电影,音樂,文艺,旅游,健康
新闻资讯
美图摄影
投资理财,时尚,文艺,美食,娱乐,无敌音乐工场,企业管理,心理学,唱歌,听歌
汽车,时尚
新闻资讯,教育就业,美女,美食,军事,疯子,旅游美食爱好者
时尚,文艺,体育,娱乐
娱乐
90 后,宅男,吃货,感性,偶像
娱乐,星座命理,音乐,宅
成都地产圈,高端物业,旅游地产,商业地产,房地产,地产,房地产营销策划
积极奋发,乐观向上
新闻资讯,美女,汽车,IT 数码
美女
幽默,新闻趣事,广东生活,美食
校园生活,艺术
星座命理
IT 数码
娱乐
水瓶座,散步,汽车
一根筋,傻兮兮,好吃懒做,小脾气,娱乐
体育
游戏动漫,娱乐,时尚
文艺
美女,处女座,自由职业,阳光
军事,新闻资讯
偶稀饭睡觉觉,白羊座,免费试用,试客
星座命理,文艺,体育,美女
九只,隆诗,苏干支,Jolin
美食
宅女一枚,平常心,吃货
新闻资讯,健康,旅游
文艺,程序猿,无三观
新闻资讯,时尚,投资理财,星座命理,美食
90 后,白羊女
星座命理,旅游,游戏动漫
美食
搞笑幽默,穿衣美容
时尚
健康,娱乐
格子控
视频音乐
文艺,体育,美女,IT 数码

新闻资讯,美女,时尚,娱乐,体育
旅游,健康
体育
k 歌
旅游
教育就业,文艺
体育
交友,睡觉,时尚,爱情,不爱学习的学生,处女座,宅男,爱听歌爱唱歌,苹果控,90 后
健康
星座命理,旅游,健康,美食,时尚
更爱源源,爱源源,吃货
旅游,新闻趣事
文艺,体育,娱乐
星座命理
麦霸,日剧,幸福,韩剧,善良,自由职业
广东生活,旅游,美食,搞笑,看书
投资理财,教育就业,汽车,健康,新闻资讯
健康,体育,娱乐
体育,娱乐,汽车,时尚,美女
娱乐
囧
名人明星
旅游,爱猫,矛盾无知,天文地理,宿命,动物,历史,军事,体育,射手座
娱乐,军事,旅游,美食
体育
旅游
美食,时尚,新闻资讯,旅游,健康
旅游,美食,文艺
健康,新闻资讯,娱乐,教育就业,美食
体育
美女,摄影控,淡定,梦想,爱狗
微博奇葩
穿衣美容,手表控,瑜伽爱好者,宅
80 后,游泳,唱歌,自由,睡觉,宅,music,钓鱼,狮子座,游戏
旅游,学生
好性格,阅读,读书,漳州生活,新闻趣事,视频音乐,微博奇葩
读书分享,名人明星
文艺,天秤座,学生,听歌,90 后
吃,双子座,幽默,美食,90 后。,爱听歌爱唱歌。
新闻资讯,时尚,投资理财,旅游,美食
惠州生活
美女,星座命理,文艺
宅女
名人明星,白羊座的吃货,听歌,唱歌,电影,好书,自由,90 后

教育就业,投资理财,健康,旅游,美女
娱乐,游戏动漫
时尚,娱乐,美食,旅游,健康
玩儿,手机,自由,八卦,游戏,学生,动漫,音乐,天秤座,搞笑
星座命理,旅游,美食,摩羯座,时尚,80后,娇生惯养,购物狂
健康,教育就业,军事,文艺
健康
哆啦A梦车饰,哆啦A梦
军事,旅游,美食,美女,体育
娱乐
论文代发,期刊编辑
乖学生乖儿子
新闻趣事,搞笑幽默,南宁生活,奋斗,旅游美食,学生一枚,交友,玩儿,好书,看书
星座命理,美食
计算机,幽默,电子设备,杰伦
户外,爱生活,奋斗,学习,考研,人生,爱自由,梦想,思考,公益
美女,IT数码,体育,娱乐,喜欢听歌,台球,麦霸,唱k
美食
娱乐
娱乐
旅游
欧洲,探险,时尚,美食,度假,休闲
军事,美食
文艺,妖精的尾巴,学生
时尚
投资理财,IT数码,汽车,电话2082788
娱乐,投资理财,时尚,IT数码,汽车,新闻资讯
健康,游戏动漫,旅游,美女,汽车,玩美艺术,摩羯座
娱乐,投资理财,教育就业,IT数码,在学游泳中,慢热,小说,睡觉,90后,羽毛球
时尚,娱乐,投资理财,健康
微博奇葩,时尚
视频音乐,旅游
新闻资讯,健康,教育就业,投资理财,娱乐
旅游,珍惜,懒散,恋旧,摩羯,三分钟热度
教育就业,健康,美女,汽车,美食,文艺,娱乐
健康
时尚
旅游,文艺
相叶雅纪,大野智,二宫和也,樱井翔,松本润,模特闪瞎眼,大宫 SK 点赞,总武线走起,末子踢馆中,红紫是正解
体育
美丽说,时尚潮流,音乐,喜新厌旧,没有毅力,扯淡,开放,冷笑话,电脑
时尚
射手座,汽车,创业,生活,自由,艺术,摄影爱好者,网络,时尚,电子商务

时尚,娱乐
美食,娱乐
时尚,娱乐,上网
时尚,体育,娱乐
健康
美食,IT 数码,吃货,白眼狼,学生
时尚,教育就业,健康,美食,娱乐
汽车,健康,美女,IT 数码
娱乐,游戏动漫,美食,时尚,旅游,体育,文艺,健康
美食,交友,睡觉,唱歌,旅游,上网,运动,篮球
cosplay,苏云凡,仙剑奇侠传,淅雨听风配音社
吃货,美女,IT,阅读,看书,唱歌,90 后
IT 数码
新闻趣事,教育就业
好性格,旅行,体育,汽车,微博,音乐
美女,游戏动漫,健康
文艺
电影
文艺,娱乐,美食,善良,听歌
美女,时尚,汽车
行政人事,管理,房产,行政管理
娱乐
新闻资讯,体育,游戏动漫,军事,健康
美食,喜欢音乐,做梦,天蝎,时尚,动漫,矛盾体,爱唱歌
投资理财,新闻资讯,时尚,IT 数码,汽车
美女,娱乐,投资理财,体育,美食,旅游,时尚
IT 数码,美食,健康
美食
军事
旅游,美女,星座命理,体育,娱乐
美食,穿衣美容,视频音乐,搞笑幽默
旅游,健康,IT 数码,结交广大网友,自由职业,创业,微博,美女,IT,宅
美食
搞笑幽默,夕颜,纯粹
襄阳窗帘,晾衣架,椅子套,沙发套,隐形纱窗,百叶帘,竹帘,卷帘,布帘,窗帘
美女,投资理财,旅游,体育,IT 数码
旅游,健康,时尚,汽车,游戏动漫,体育,IT 数码,军事
投资理财
娱乐
微博奇葩
校园生活
穿衣美容,基督信仰,美丽,健康,NUSKIN,营销
金牛座,宅,平常心,汽车,游戏,时尚,听歌
蒋劲夫,快乐大本营,明星,杂志,忧郁,夜猫子,生活,减肥,主持人

旅游,美食,摄影,电影
娱乐,美食
游戏动漫
旅游,美食,IT 数码,娱乐,游戏动漫
视频音乐,搞笑幽默
体育
娱乐
EXO 挚爱朴灿烈,EXO 挚爱金钟大,EXO 挚爱黄子韬,EXO 挚爱白贤,EXO 挚爱鹿晗,EXO 挚爱世勋,EXO 挚爱艺兴,对人真诚、善良
搞笑幽默
体育,文艺
投资理财,新闻资讯,旅游,美食,文艺,娱乐
新闻资讯,投资理财
时尚,教育就业,文艺,娱乐,美食
偏執狂,夢想家,文藝,旅行,電影,音樂,看書,五月天
时尚,IT 数码,教育就业,投资理财
宅男,交友,网络,睡觉,宅,爱情,听歌,囧,平常心,时尚
美女
游戏动漫
游戏动漫,IT 数码
佛山生活
旅游
军事,汽车
旅游,娱乐,健康
娱乐,汽车,体育,游戏动漫
浪漫,囧,娱乐
美食
娱乐,体育,文艺
名人明星
双子座,80 后
娛樂,星座命理,旅遊
时尚,新闻资讯,教育就业,文艺,军事,闷骚,懦弱
名人明星
新闻资讯,教育就业,文艺,旅游
娱乐
旅游,睡觉,大鼻子,音乐,青春,疯狂,纠结
美食
萌宠,亲情,偶稀饭睡觉觉,美剧,电影,好书,看书,吃货,射手座,80 后
读书,交友,文字,动漫,天蝎座,美食
美女,文艺
体育
星座命理,体育
娱乐
星座命理,美食,旅游

娱乐,美女,体育
娱乐,健康,美食,旅游
美食
美食,旅游,文艺,双鱼座,90 后,旅行,英语,亲情
游戏动漫,美食,旅游,娱乐,音乐达人
美女
搞笑幽默
射手座
星座命理,小洁癖,小心软,小感性,小敏感,轻微外貌协会,强迫症,热感触,起床困难户,忽冷忽热
新闻资讯
美食,旅游
汽车,时尚,健康,美食,旅游
美食,交友,音乐,唱歌,90 后,爱情,旅游,宅男,娱乐,听歌
娱乐
旅游,星座命理,美食,文艺,娱乐
汽车
搞笑幽默,新闻趣事,星座命理,微博奇葩
健康
娱乐
泾中,南京大学,泾中校友,宝应泾河,泾河,泾河中学
娱乐
健康,旅游,星座命理,IT 数码,娱乐
星座命理,娱乐
游戏动漫,军事,IT 数码,美女
美女
新闻趣事
深圳生活,时尚,文艺,星座命理,娱乐,健康
吃货,梦想家
娱乐,游戏动漫,文艺
吃货。,纠结
双鱼座,校园,NBA,看书,交友,财经,羽毛球
旅游
星座命理,美食,时尚,文艺,娱乐
电子商务,网购,80 后,旅游,射手座,宅
看书
美女,娱乐,汽车,IT 数码,旅游,我就是我,交朋友
体育,军事
VIP,GDragon,天蝎,BIGBANG
香港
公安,澄海,严格执法,为人民服务,救火,消防
动漫,水瓶女,爱猫,爱睡觉
财经与股市,读书用心
时尚

游戏动漫,健康,篮球,单身单纯的人！,单身单纯的人
IT 数码,文艺
体育
搞笑幽默
时尚
搞笑幽默
美食,视频音乐,搞笑幽默,北京生活,旅游
艺术品,书画,画家,书法家,艺术,古玩,古董,节能环保,画画
宁波大红鹰
萌宠
IT 数码
微博奇葩
IT 数码
旅游,时尚,新闻资讯,美食,文艺
教育就业
旅游
娱乐
游戏动漫,IT 数码,军事,美女,旅游,爱旅游,90 後
娱乐
文艺,教育就业,新闻资讯,健康,军事
手写
80 后
休闲,时尚潮流,手机看小说,时装,爱电影,心理学,新闻资讯,A 股,医学,投资理财
小女人,娱乐
IT 数码
名人明星,IT 数码,旅游,美女,视频音乐
校园生活
名人明星
好性格
健康,红烧肉
名人明星,娱乐
健康,天秤座
娱乐
健康,旅游,时尚,新闻资讯
美女
時尚,娛樂
生活,历史,玩儿,90 后,NBA,运动,好性格,搞笑,美女
穿衣美容
美食
新闻趣事,八卦杂谈,搞笑幽默,旅游,美女
微博,偶稀饭睡觉觉,宅,时尚,乐观,网络,营销,电影
投资理财
时尚
星座命理

摄影爱好者,爱玩,搞笑,高中生,睡神,吃货,2青年,90后,天蝎座
旅游
手绘,CG,筝,文字,写曲,钢琴,旅行
睡觉,美食
微博奇葩
穿衣美容,旅游,双鱼女
唱K,爱音乐,交友,运动,喜欢旅游
缺爱！邪恶！
游戏动漫,时尚,汽车,教育就业,投资理财
成都生活
文艺,体育,汽车
旅游,军事
健康
游戏动漫,美女,美食,娱乐
重庆生活,娱乐
美女
IT数码,娱乐,美女
新闻资讯
体育
游戏动漫
时尚,旅游,娱乐,自由,听歌,宅
视频音乐
体育
好书
互相尊重
39710530,欧冠,球迷,武汉,同仁会,德甲,德国队,拜仁慕尼黑,体育
自由,休闲,陈奕迅,努力,看电影,喜欢旅游,射手座,善良,购物狂,90後
汽车,美女,旅游,IT数码
旅游,美食,文艺
爱吃,东北银,PKUer,85后,搞笑幽默,萌宠,体育
IT数码,文艺,娱乐,摄影,睡觉,时尚,吃货,小说,美食
萌宠
车世界,科学探索,美女,搞笑幽默,体育,汽车,军事,旅游
游戏动漫,文艺
游戏动漫,新闻资讯,投资理财,军事
自由
IT数码,军事,汽车,投资理财,新闻资讯
游戏动漫
动漫
动漫
美食,自由,校园,游戏,艺术,篮球
穿衣美容,视频音乐
IT数码,汽车,娱乐
体育,新闻资讯,美女

IT 数码
投资理财,文艺,IT 数码,健康,旅游,美食
美女,星座命理,体育
健康,美女,美食,体育,军事
美食
时尚
游戏动漫,健康,旅游,星座命理,娱乐
管理软件,IT 互联网,IT 行业,网络营销,IT 培训,CRM,ERP,企业管理咨询,企业信息化,信息技术
看书,自由,电影,浪漫,唱歌,天蝎座,乐观
健康,教育就业
文艺
旅游
投资理财,时尚,军事,IT 数码,文艺,娱乐
新闻趣事
投资理财
单身,济宁,重庆,江阴,爱情,白羊座,网络电子商务,海口
娱乐,纠结,拍照,K 歌,敏感
旅游,视频音乐,新闻趣事,搞笑幽默,陕西生活
游戏动漫
娱乐,文艺
美食,旅游,娱乐
小屁孩,❤,大苹果,胡思乱想
搞笑幽默
游戏动漫,新闻资讯,美女,IT 数码,汽车,娱乐,时尚,文艺,体育
哈尔滨,重庆,兜风,人马座,生物,慎重
双鱼女
美食,文艺,教育就业,体育,游戏动漫
心理咨询,婚姻家庭辅导,真诚,热情
时尚
旅游,健康,游戏动漫,健身
汽车,文艺,体育,星座命理
美食,美女,时尚,文艺,娱乐
美食,IT 数码,金牛座
旅游,娱乐,美食,IT 数码,汽车
旅游
时尚,ing
旅游,美食
美食,旅游
真诚,快乐,爱笑,安静,努力,活泼开朗,淡定,乐观,艺术
美女
投资理财,YY
旅游
好性格,微博,时尚,阅读

湖北生活
搞笑幽默
微博奇葩
旅游,健康,时尚,星座命理
情感生活,文艺
旅游,军事,IT 数码,体育
视频音乐,美食,星座命理,新闻趣事,搞笑幽默
设计,爱设计,学设计的学生
美女,教育就业,投资理财,云南人
白羊座
旅游,星座命理,教育就业,美食,时尚
名人明星
美女,文艺,教育就业,军事,投资理财
体育
旅游,自个疼
音乐,娱乐
名人明星,90 後,天蝎座
美女
音乐爱好者,打乒乓球
旅游,教育就业,体育
体育
80 后,唱歌,上网,听歌,睡觉,电影,美剧,生活,天蝎座,旅游
星座命理
美女
紫色,上网
喜欢旅游,古巴吉士纳,龙婆培,龙婆坤,四面神,掩面佛牌,必打佛牌,蝴蝶佛牌,崇迪佛牌,泰国佛牌
教育就业
摄影爱好者,睡觉,自由,摄影,听歌,宅女,摩羯座,语录
萌宠
游戏,Randy,Rhoads,重金屬,音樂,獅子座,吃,Metallica
微博奇葩,搞笑幽默,新闻趣事
视频音乐,体育
美女,体育
星座命理,健康,旅游,IT 数码,汽车,体育,大学,电影,美剧
穿衣美容
旅游,星座命理
白色控,海贼迷,双子座,美剧迷,90 後,看书,摄影,唱歌,动漫,东野圭吾
法国,星座命理,健康,娱乐,文艺,海绵宝宝,文静,
旅游,星座命理,美食,文艺,娱乐
苏打绿,宠物图集,偶稀饭睡觉觉,懒,喜欢听歌,情绪化,手机,摄影爱好者,收藏,汽車
艺术,小说迷,湖北生活,新闻趣事,美食
教育
健康,旅游,美食,娱乐

军事,娱乐,体育,美女
健康,游戏动漫,星座命理
搞笑幽默,健康
游戏动漫,健康,旅游,星座命理,军事
穿衣美容,视频音乐,美食,搞笑幽默,爱笑话,懒,自信,爱美食,好性格
IT,财经,投资理财,旅游,星座命理,美食,IT 数码
吃喝玩乐
娱乐,星座命理,投资理财,美食,美女,时尚
萌宠
搞笑幽默,名人明星,美容服饰,美食,育儿百科
投资理财,旅游,新闻资讯,教育就业
娱乐,上进青年,阳光控,低调内敛,专抢你的菜哦
健康,游戏动漫,IT 数码,体育
美食
新闻资讯,军事,体育,运动,洒脱,文学
动漫,运动,吃,微博,小说,旅游,电影,音乐,汽车,80 后
游戏动漫,IT 数码
游戏动漫
时尚,美食,健康,新闻资讯,教育就业
美食,旅游
旅游
健康,真诚,傻,浪漫,散步,怀旧
娱乐,羽毛球,90 后
军事,汽车,游戏动漫,时尚,美女,娱乐,体育
教育就业
教育就业,文艺,星座命理,IT 数码,娱乐
健康,爱,喜欢旅游,美女
健康,军事,体育,听音乐,，看电影,，互粉,，互听
星座命理,旅游,新闻资讯,投资理财,游戏动漫
旅游,美女,时尚,美食,星座命理,游戏动漫,娱乐
湖州生活
IT 数码
时尚
美食
娱乐
新闻资讯
星座命理,娱乐,美食,旅游
90 後
爱吃爱玩爱嘉嘉
旅游,健康,时尚,教育就业,娱乐
时尚,娱乐
美食,娱乐,时尚
90 后,打工族,美食,驴行
穿衣美容,娱乐

汕头生活
星座命理
视频音乐,微博奇葩,萌宠,星座命理,文艺,游戏动漫,娱乐
艺术,天蝎,休闲,喜欢听歌,热爱生活,淡定,时事,影视,体育,fashion
时尚,体育,IT 数码,美食,美女,汽车,娱乐
时尚,星座命理
旅游,汽车,骨 z1 里的傲气,浪漫,宅男,巨蟹座,微博,90 后
微博奇葩,Moment,十里烟华
90 后,创业,摄影爱好者
美食
旅游,汽车,IT 数码,体育,双子座,80 後,电影爱好者
美食,名人明星
萌宠
美食
微博奇葩
蓝色,吸血鬼
爱自由,爱音乐,爱旅游,学生一枚,自由,喜欢音乐,诗词
美女
时尚
新闻资讯,天蝎座,微笑,o 型血,狗狗迷,爱旅游,音樂
搞笑幽默,IT 数码,军事,时尚
旅游,听歌,善良,动漫,音乐,电影
旅游,美食
美食,健康,新闻资讯
吃货,justinbieber,射手
旅游,投资理财,美女,健康,时尚
新闻趣事
音乐迷,思考,爱自由,文艺
教育就业,IT 数码,汽车
旅游,美食,教育就业,文艺
射手座
娱乐,健康,IT 数码
吃货,娱乐,网购,双子座,爱情,睡觉,大学,学生
体育,娱乐,旅游
体育,军事,健康
搞笑幽默
体育
IT 数码
汽车,媒体
美食
游戏动漫,静待缘分,爱猫爱狗,囧,动漫,听歌,摄影爱好者,80 后,电影,宅男
星座命理
INchocolate,巧克力,李成钟,金明洙,李成烈,李浩沅,南优铉,张东雨,金圣圭,INFINITE
文艺

搞笑幽默
旅游,美食
旅游,游戏动漫,美女,汽车,IT 数码
甘榜情,万福楼,走进走出,主妇的假期,爱的掌门人,昆仑奴,对对碰
健康,旅游,IT 数码,文艺,娱乐
双鱼女
文艺
星座运势
健康
娱乐,星座命理,新闻资讯,体育
画画,热爱生活,爱吃,李民浩,泰勒斯威夫特,夜猫子,睡懒觉,文字控,胡思乱想
新闻资讯,教育就业,美女,投资理财,IT 数码
IT 数码,娱乐,投资理财,新闻资讯,体育,旅游,时尚
美食,小說,音樂,動漫,宅女非我所愿
军事,IT 数码,娱乐,投资理财,新闻资讯
宅男,睡觉睡到自然醒,游戏动漫,汽车,体育,娱乐
娱乐
汕头生活
教育就业,游戏动漫,IT 数码,体育
搞笑幽默,新闻趣事,健康养生,美食,自由职业,创业,80 后,天秤座,旅游
绵阳生活
萌宠,微博奇葩,泉州生活,搞笑幽默,视频音乐
游戏动漫,美食
教育就业,旅游,美食,IT 数码,文艺
搞笑幽默,文艺
美食,新闻资讯
投资理财
文艺,星座命理
游戏动漫,新闻资讯,旅游,美食,体育
汽车,旅游,体育,时尚,娱乐
搞笑幽默,宅,好动
教育就业,体育
旅游,星座命理
投资理财,美女,汽车
音乐爱好者,射手座
军事,星座命理,游戏动漫
看书,上网,动漫,天秤座,学生一族
处女座,散步,工作,音乐迷,安静,宅,80 后,爱生活
汽车,体育,旅游,吉他,喜欢音乐,日本,旅游美食,杂志,西班牙
时尚
娱乐
美食
汽车,投资理财,时尚,文艺,IT 数码
朋友,青春,中医

IT 数码
搞笑幽默
旅游,时尚
巨蟹,发呆,娱乐,游戏动漫,个性,文字,喜欢唱歌,90,猫,网络营销
健康,时尚,旅游,文艺,娱乐
美食,体育,娱乐,时尚,星座命理
美女
娱乐
教育就业
时尚,教育就业
汽车,军事
旅游
健康,新闻资讯,军事,星座命理,IT 数码
旅游,IT 数码,军事,体育,文艺
新闻资讯,健康,美食,时尚,教育就业,投资理财
文艺
健康
时尚,旅游,健康,文艺,体育
美图摄影,沉默,睡懒觉
娱乐,电影,偶喜欢玩,体育,唱歌,音乐
娱乐,汽车,IT 数码,军事,游戏动漫,体育
军事,IT 数码
美女
美食
时尚,教育就业,美食
洛洛,我真的好想你
视频音乐,新闻趣事,旅游,教育就业
旅游,军事,文艺
星座命理,旅游,美食,萌宠
三国杀,历史,动漫迷,建筑,小说,幽默,看书,新闻资讯,游戏动漫,文艺
时尚,娱乐,IT 数码,游戏动漫
小野友樹,小野賢章,野島裕史
娱乐,体育,旅游,投资理财,时尚,IT 数码
旅游,娱乐
健康
名人明星
名人明星,娱乐
旅游
投资理财,新闻资讯,健康,旅游,体育
教育就业,投资理财,新闻资讯
射手座,自由,看书,偶稀饭睡觉觉,80 后,动漫,宅女
穿衣美容,萌宠,K 歌,音乐,巨蟹女,多愁善感,爱唱歌,活泼,青春,个性
北京生活,视频音乐,穿衣美容,美食,旅游
美女

美食,健康,时尚,投资理财,美食,旅游
军事,文艺,健康,美食,娱乐
新闻趣事,视频音乐,名人明星,美女,搞笑幽默
时尚,教育就业,投资理财,健康,体育
新闻趣事,搞笑幽默,旅游,穿衣美容,微博奇葩,健康,游戏动漫,娱乐
微博奇葩,旅游,美女,新闻趣事,搞笑幽默
穿衣美容,旅游,美食
旅游
美食
福州生活
汽车,IT 数码
美女,健康
美女
健康,旅游
娱乐
音乐,睡觉
体育
文艺,健康
旅游
西安,时尚,旅游,美食,娱乐,体育
文艺,体育,笑,学生一族,懒,k 歌,台球,桌球,傻,吸血鬼日记
美食,文艺
美女
写字,健身,读书,音乐爱好者,体育
时尚,美女,娱乐
时尚,娱乐,健康,旅游,文艺
职场招聘,读书分享,美食,济南生活,育儿百科,星座命理,文艺,健康
星座命理
体育
游戏动漫
新闻资讯
健康,投资理财,教育就业,IT 数码
美食
投资理财,体育,汽车
旅行,channel4,BBC,设计
视频音乐,美女,萌宠,旅游,穿衣美容
娱乐
偶喜欢玩,游戏动漫,健康,美食,娱乐,时尚,电视剧,双子女,动漫
娱乐,文艺,时尚
星座命理
文艺
文艺,旅游,健康,文学,网络,自由,美食,微博,亲情,时尚
旅游,微博奇葩,新闻趣事,美女
体育,文艺,IT 数码,Durant 球迷

时尚,健康,美食
时尚,网购,健康,澳大利亚,潮流,瘦身,美容护肤,化妆品,日系美妆
穿衣美容,星座命理,健康,美食,旅游,文艺
穿衣美容
湖北生活
游戏动漫,时尚,娱乐,文艺,美食
视频音乐,爱时间机器,爱盗梦空间,爱源代码,爱蝴蝶效应,爱幻想,爱哥特,爱理论,爱我所爱,爱自由
小說,笑话,红楼梦,乒乓球
IT 数码,游戏动漫,旅游,美食,娱乐
文艺,旅游,健康,星座命理,娱乐,时尚
搞笑幽默,穿衣美容,新疆生活
旅游
学生,b 型血,恐怖片,暮光之城,冷笑话,爱玩,90,旅游,星座命理,美食
新闻趣事,娱乐
名人明星,星座命理,娱乐
美女,喜欢旅游,NBA,感性,简单
水瓶座,A 型血
美食,文艺
穿衣美容,汽车,军事
投资理财,教育就业,健康,美女
美女,新闻资讯,体育
母婴
時尚
时尚,文艺,教育就业,美食,大学生
美食
k 歌,游戏动漫,80 後
IT 数码,旅游,健康,教育就业,投资理财
游戏动漫,美女,娱乐
穿衣美容,河南生活,搞笑幽默
校园生活,星座命理,美女,微博奇葩,高帅缺一富,自由人,双重性格,路上行者,宅,浪
新闻资讯,健康
娱乐
汽车,游戏动漫,美女
作死星人,自我矛盾体,南京人,面食控,美图摄影,金钟大,Tiffany 黄美英
体育
投資理财
娱乐
良民,汉堡
星座命理,睡觉,学生,安静,玩,快乐,健康,时尚,旅游,美食
体育
动漫控,腐,爱电影,旅游
星座命理,时尚,美女,文艺,娱乐
新闻资讯

游戏动漫
完美主义者,电视剧控,独立音乐,跑步
美女,旅游,投资理财
美食,旅游,身不在旅途,心亦在旅途,两者总有其一
旅游
金牛座,艺术
旅游,健康
旅游
美食
游戏动漫
娱乐,旅游,星座命理,文艺
名人明星
美食
IT 数码,时尚,美女,新闻资讯,娱乐
搞笑幽默
健康
健康,时尚,汽车,投资理财,旅游
健康,美食,投资理财,教育就业,文艺
IT 数码,娱乐
娱乐
娱乐
学生一枚,90,青春,逛街,看小说,爱幻想,娱乐
爱太阳,爱旅游,爱音乐
IT 数码,旅游,汽车,健康,体育
文艺
美图摄影,视频音乐
汽车,美女,游戏动漫,文艺,体育
旅游,娱乐,美食
娱乐
微博奇葩
美食,星座命理,旅游,娱乐
文学,狮子座,运动,80 后,睡觉,自由,阅读,电影,音乐
美食爱好者,忧郁,敏感,睡觉,电影控
星座命理,旅游,文艺,娱乐,美食
旅游,时尚
维尼饭,宅女,K 歌
美食
娱乐
军事,文艺
新闻资讯,健康,投资理财,教育就业,时尚
摄影控,性感,欧美控,设计师,射手座,高跟鞋,网购,自由,90 后,旅游
旅游
汽车
天蝎座,吃货,80 后

时尚
视频音乐,星座命理
健康,美女,汽车,IT 数码,体育
旅遊,新聞資訊,摩羯女
美食,游戏动漫,健康
新闻趣事,美食,星座命理,旅游,搞笑幽默
汕尾生活
年轻型航模教练,园林,公园飞手,航模,无人机应用,无人机平台开发,无人机飞控师,90 后
教育就业,旅游,时尚
时尚,军事,健康,投资理财,IT 数码
游戏动漫,时尚,IT 数码,军事,体育
湖南生活,星座命理,穿衣美容,旅游,美食
旅游
美食,体育,汽车
旅游,IT 数码,美女,新闻趣事,搞笑幽默
音乐,羽毛球,宅女,射手座,睡觉,旅行,电影,偶喜欢玩,时尚,80 后
体育,商业
旅游,美食,娱乐
自然,手机看小说,90 后非脑残,胡思乱想,学生,90 后,宅,自由,睡觉,爱美食
体育
新闻资讯
时尚,娱乐
美食,幸福,甜食动物,酷爱面食
娱乐
幽默,自由,偶喜欢玩,唱歌,美食,爱情,大学生,睡觉,白羊座,90 后
处女座,HR,听音乐,热情,安静,o 型血,时尚,旅游,美食,娱乐
文艺
双子座,减肥,北京,活泼,重庆,天秤,干物女
抹茶红豆控,美食吃不够
学习,奋斗,爱幻想,娱乐
娱乐,文艺,旅游,时尚
健康
星座运势
旅游
星座命理,低调奢华,精品,巴黎,网购,范冰冰同款,周迅同款,原单外贸,明星同款,欧美大牌
时尚,体育
设计,向日葵,O 型血,旅游
电视
健康,旅游,军事,体育
穿衣美容,旅游
健康,教育就业
美食,星座命理,旅游,娱乐
八卦,手机,简单,单身,发呆,淘宝,做梦
动漫,文字,中医

新闻趣事,新闻资讯
微博奇葩
美食,文艺
美食,搞笑幽默,新闻趣事,微博奇葩,旅游,时尚,太黑,爱猫
美食,军事,健康,新闻资讯,美食,投资理财,旅游
娱乐
美食
星座命理
IT 数码
时尚
IT 数码,美女,时尚,旅游,美食,娱乐
美食
汽车,星座命理,健康,娱乐,游戏动漫,投资理财,文艺
娱乐,健康,时尚
我就是我,电影,90 后,双重性格,旅游美食,音樂
娱乐,健康,美食,旅游,教育就业
IT 数码,文艺,旅游,汽车,军事,娱乐,新闻资讯
美食
星座命理,娱乐
娱乐,唱歌,上网,旅行,旅游,80 后,美食,生活,偶稀饭睡觉觉
新闻趣事,搞笑幽默,星座命理,河南生活
美女,汽车,体育,上网,自由,睡觉,娱乐,唱歌,学生
苏打绿,沉默,白羊,网购,书籍,文字控,我就是我,90,爱笑,安静
星座命理
90 后
体育
美食
旅游,星座命理,投资理财,娱乐,时尚
健康
时尚,IT 数码,旅游,娱乐
美食
IT 数码
体育
文艺,旅游,娱乐
文艺
微博奇葩,囧
旅游,娱乐,体育
美食,时尚,汽车
八卦杂谈,时尚,星座命理,军事,文艺,娱乐
教育就业,IT 数码,娱乐
投资理财,体育,美食,游戏动漫,汽车
交友,微博,投资,理财,电子商务,现货交易
体育
旅游,穿衣美容

军事,文学,新闻,微博
文艺
美容服饰,读书分享
时尚,健康,旅游,星座命理,文艺
体育
娱乐
金牛座,潮人,星座,爱音乐,淘宝,B 型血,80 後,小清新,时装,喜欢唱歌
名人明星,90 后
旅游,穿衣美容,搞笑幽默,星座命理,微博奇葩
美食
美食
微博奇葩
其他生活
娱乐
美食
投资理财
汽车,IT 数码,游戏动漫,美女,新闻资讯
美食,娱乐,时尚,健康,旅游
上海生活,搞笑幽默,上海精神病医院,精神卫生中心,上海精神卫生,上海精神科医院,精神病,精神分裂
美食,健康,美女,军事,汽车
健康,时尚,投资理财,文艺,娱乐
旅游
美图摄影
时尚
爱电影,Shopping,旅游,摄影,淘宝,星座命理,时尚
时尚
蜡笔小新,NBA,水瓶座,音乐
雷人雷事雷到家,搞传销,双鱼座,旅游,囧囧,搞笑,网络,20 后,汽车狂,震撼
汽车,健康,时尚,教育就业,IT 数码,文艺,听歌,IT,篮球
陕西生活,新闻资讯,汽车,IT 数码,体育
美食,军事
体育,Nerazzurri,国际米兰球迷,三国,电影,NIKE,足球
时尚
星座命理,神经质
美容服饰,名人明星,美食,搞笑幽默,新闻趣事
旅游
体育
新闻资讯,军事
文艺
健康,文艺,美女,体育,娱乐
梁静茹,自信,心理学,王力宏,互粉,阳光,微博控,郭敬明,创业,上网
IT 数码
健康,旅游,文艺

搞笑幽默
美食,白羊座
投资理财,教育就业,美女,军事
游戏动漫
投资理财,旅游
星座命理,偶稀饭睡觉觉,巨蟹座
美食,微博奇葩,萌宠,搞笑幽默,北京生活,舞蹈
汽车,美女,健康,军事,娱乐
投资理财
游戏动漫
美食,宅
文艺,美食,健康,时尚
体育
金牛,爱自由,购物,90 後,唱 K,电脑,逛街,喜欢听歌,福建生活,吃货一枚
健康,教育就业
汽车
美女
爱音乐,音樂,夜猫子,汽车,体育,娱乐
跆拳道,90 后但我不脑残,宅女一枚,看小说,庚饭
游戏动漫,健康,美女,军事,IT 数码,文艺,娱乐
新闻资讯,IT 数码,体育
游戏动漫,星座命理,娱乐,美食,体育,爱音乐,爱零食,爱睡觉
咖啡控,湖人,双重性格,喜欢旅游,旅游,视频音乐
星座命理
旅游,美食
美食
投资理财
美食,文艺
搞笑幽默
文艺,娱乐,美食,美女
旅游
新闻资讯,娱乐,时尚,旅游,美食,文艺
旅游,微博奇葩,湖北生活
旅游,体育,娱乐,游戏动漫,美女
娱乐,新闻资讯,时尚,汽车,IT 数码
汽车,时尚,旅游,美食,娱乐
敏感,美食,娱乐,新闻资讯,随性,奋斗,睡懒觉,青春,神经大条
文字控,韩庚,快乐大本营
体育,娱乐,游戏,篮球,看书
体育,旅游,摄影爱好者,紫色,听歌,文字,90 后,偶稀饭睡觉觉
教育就业,旅游,军事,体育,娱乐,生活,游戏,搞笑,乐观,运动
旅游
美食,美女,旅游,美食,教育就业,文艺,时尚,健康
自由职业,偶稀饭睡觉觉,时尚潮流,双鱼女

美女,汽车
IT 数码,军事,美女
美食,娱乐,时尚,文艺
美食,星座命理,文艺,健康
美食
时尚,旅游,美食,文艺,娱乐
娱乐
娱乐
营销,好书,瑜伽,时尚潮流,新闻,理财,古典艺术
股指期货,广交所招商加盟,白银投资,招商加盟,白银 TD,黄金投资咨询,体育,自然,坚强,赚钱
美女
星座命理,IT 数码,娱乐
旅游,八卦杂谈,美食,校园生活,搞笑幽默
美女,视频音乐,IT 数码
旅游
新闻资讯,健康,美女
自由
新闻趣事,旅游
IT 数码,黄靖伦,林宥嘉,完美主义,处女座,谷歌控
汽车
游戏动漫,娱乐,文艺
胡思乱想,影视
美女,娱乐
等!
体育,健康,美食,青春,喜欢听歌,执着,爱幻想
文艺,投资理财
体育
娱乐
其他生活
美女,汽车,文艺,健康,时尚
健康,汽车,旅游
各种青年,白羊座,学生
IT 数码,投资理财,文艺,旅游,教育就业,时尚,新闻资讯
新闻资讯,星座命理,娱乐
星座命理,娱乐,新闻资讯
美容服饰,福建生活
搞笑幽默
缺爱 ing,起床困难户,微博控，美女控
美食,文艺,游戏动漫,情绪化,韩剧,天蝎座,宅女,看书,吃货
健康,投资理财,教育就业,新闻资讯,IT 数码
上海生活
旅遊,听音乐
体育

美女
文艺
游戏动漫,时尚,体育
健康,时尚,军事,娱乐,体育
时尚,旅游,美食
新闻趣事
旅游,美食
路痴,好性格,偶喜欢玩,听歌,搞笑,美食,自由,睡觉,亲情,宅
体育,文艺,娱乐
名人明星
视频音乐
教育就业
美食
教育就业,健康
旅游
游戏动漫,投资理财,娱乐
游戏动漫,军事,娱乐
八卦
投资理财,军事,体育
游戏动漫,投资理财,军事
健康,美食,军事,IT 数码
汽车,娱乐
写作,思考,江门,理性,财会,搞笑,会计,湖州,游戏,西安
IT 数码
旅游
娱乐
健康
搞笑幽默,娱乐,军事
美食,娱乐
男人,旅游爱好者,登山,软件,学生一枚,新闻资讯,旅游,美食,IT 数码,军事
军事,美女,游戏动漫
投资理财,军事,新闻资讯
娱乐
美食,体育,旅游
财经
美剧,动漫
单身贵族
幽默笑话,笑话,幽默,搞笑,IT 数码,美食,军事,文艺,娱乐
时尚
游戏动漫,时尚,美食,文艺
旅游,美食,汽车,体育,娱乐
看小说,时评,美食,娱乐
我真棒
健康,时尚,文艺,旅游

旅游,尚品
微博奇葩
时尚
教育就业,时尚,美食,娱乐,文艺
体育
娱乐
旅游,美食
星座命理,偶喜欢玩,射手座,好性格,校园,电影,音乐,听歌,90后
校园生活,搞笑幽默,星座运势
星座命理,汽车
文艺
时尚,体育,美食
文艺
娱乐
浅口鞋,adidas,正品保证,代购,时尚
武汉,唱歌,喜欢听歌,玩
微博奇葩,美食,穿衣美容,星座命理,新闻趣事
健康,教育就业
时尚
美食
IT数码,美食,旅游,体育,娱乐,电影
旅游,美女
教育就业,美女,时尚,旅游,文艺
红酒,培训,独立,留学,自由,美剧,创业,设计,读书,旅游
80后
旅游
文艺,娱乐,健康,美食,旅游
旅游,美女,新闻资讯,军事,体育
文艺
星座命理,我就是我,吃货
微博奇葩,吃货一枚,大学生,动漫控
旅游
玩儿,汽车
旅游,美食,军事
娱乐
美食
投资理财
90后非脑残,宅男,爱睡觉,好性格,单纯,喜欢听歌,低调,单身,天蝎,热爱生活
文艺,体育,麦迪,艾弗森,C罗,胡思乱想
爱星座,爱宅女
投资理财,时尚,健康,旅游,美食
文艺,时尚,健康,旅游,体育
游戏动漫,IT数码,拍照,K歌
时尚,星座命理,健康,投资理财,美食,音乐,大学

健康,旅游
游戏动漫
娱乐,美食,游戏动漫
萌宠
IT 数码,时尚,汽车,体育
80 后,时尚,李维斯,潮流,川久保玲,阿迪达斯三叶草,耐克 NIKE,处女座,创业
时尚,旅游,美食,军事,娱乐
旅游,文艺
媒体,紫色
汽车,旅游,投资理财,IT 数码
文艺,旅游,时尚,新闻资讯,娱乐
香港生活
美食,旅游
娱乐,旅游,美食
忠诚心,呼和浩特,金牛座,餐饮,江门,思考,淄博,理性,发展力,活泼
体育,健康,IT 数码,美食
双子座,音乐,数码,八卦,手机,交友,幽默,美女,大学,搞笑
文艺
活泼开朗
汽车,IT 数码,娱乐,体育,投资理财
视频音乐
旅游
自干五,美女,文艺
美食,文艺,美女,IT 数码,新闻资讯,体育
硬件,慈善,活泼,宅女,单身,卫生服务,审计,扬州,餐饮,呼和浩特
IT 数码
无趣的人
汽车
体育
美食,文艺,美食
重口味,娱乐
旅游,青春,睡觉睡到自然醒,财经
开心,自拍,爱笑,娱乐
西安,金华,网络电子商务,湛江
美女,IT 数码,体育
美食,体育,娱乐,文艺,美女
文艺,游戏动漫,时尚,IT 数码,娱乐
星座命理,时尚,汽车,健康,文艺
健康,教育就业,体育
健康,时尚,娱乐,美食,美食,旅游
文艺,娱乐,旅游,星座命理,美食,游戏动漫
名人明星
美女,微博奇葩,搞笑幽默,星座命理,视频音乐
时尚,健康,旅游,美食,娱乐

搞笑幽默,宅,学生,活泼开朗,服装
人力资源,热爱生活,投资理财,美食
名人明星,星座命理,旅游,美食
娱乐,星座命理,美食
美食,星座命理,文艺,健康
吉林生活,教育就业
星座命理,美女
文艺
文艺,游戏动漫,美女
文艺
校园生活
美食,就这么过
游戏动漫
美女,文艺,健康,旅游,体育
旅游,汽车,星座命理,美食,体育
美食
多愁善感,傻,红楼梦,笑,爱自由,驴友,爱生活,军事
时尚
A 股,唱歌,看书,偶稀饭睡觉觉
穿衣美容
教育就业,文艺,美食
游戏动漫,新闻资讯,教育就业,军事,体育
摩羯座
美女,新闻资讯,文艺,旅游,健康
旅游
娱乐
体育
娱乐,IT 数码
美女,旅游,娱乐
娱乐
新闻趣事,美女,视频音乐,搞笑幽默,保姆吸尘器,无线吸尘器,机器人吸尘器,智能吸尘器
旅游,新闻资讯,广告,传媒,媒体,策划,教育就业
时尚,投资理财
教育就业,军事,文艺
内向，喜欢作菜
星座命理,旅游,娱乐
互联网,乐观,看书,美剧,好书,电子商务,平常心,旅行,汽车,听歌
美女,娱乐,游戏动漫,健康
健康,时尚,旅游,美食,娱乐
娱乐
娱乐
军事,听歌,写作,白羊座
交易策略,国际期货,股指,期货,股票,外汇,黄金,投资,理财,金融理财
名人明星,微博奇葩,旅游,视频音乐

娱乐
体育,奋斗,Android,汽车,70753E
文艺,娱乐,星座命理
美食,娱乐
穿衣美容
星座命理
星座命理,时尚,娱乐,文艺
玩,懒,快乐,北京,体育,投资理财,旅游,文艺,娱乐
健康,娱乐,美食
自由,羽毛球
多愁善感,善良,开心,青春,梦想,A 型血,双子座,90 后
汽车
美食
时尚
美女,闷骚男
健康
财迷,90 后,包包控,强迫症
文艺,IT 数码
投资理财,美女
汽车,IT 数码
书,喜欢旅游,文化,投资理财,时尚,旅游,文艺
时尚
时尚,汽车,新闻资讯,娱乐,体育
体育,娱乐,小疯狂
娱乐,星座命理
名人明星
旅游
文艺,美女,新闻资讯,健康,娱乐
体育
产品视频,动画设计,视频设计,影视制作,企业宣传片制作,影视广告
旅游,娱乐,文艺,美食,星座命理
自然,时尚,健康,旅游,美食
星座命理
读书分享
健康,时尚,星座命理,IT 数码,文艺,娱乐
健康
名人明星
爱笑,懒,教育就业
美食,娱乐,美女,汽车,体育
游戏动漫,星座命理
汽车,旅游
新闻趣事,搞笑幽默,美食,星座命理
汽车,健康,星座命理,美女,旅游,体育
旅游

星座命理,90後,处女座,完美主义
新闻资讯
时尚,健康,文艺,美食,娱乐
美女,外贸协会,没兴趣没爱好
健康,星座命理,娱乐
汽车,IT 数码,美食,旅游,美女
其他生活,美食,搞笑幽默,新闻趣事,美女
星座命理
动漫控,麦霸,健康,亲情
时尚
名人明星
视频音乐
美食,健康,时尚,教育就业,娱乐
搞笑幽默
星座命理,美食,IT 数码,文艺
旅游,时尚,投资理财
射手座,善良,美食爱好者,喜欢听歌,热爱生活,开心,喜欢旅游,朋友,好书,校园
旅游,文艺,娱乐,美食
搞笑幽默
汽车
搞笑幽默,健康,新闻资讯,时尚,旅游,文艺
体育,IT 数码,游戏动漫
IT 数码
O 型血
萌宠,视频音乐,新闻趣事,旅游,美食
健康
美食
美女,旅游,军事
美女
新闻资讯,游戏动漫
神经质,80 后的尾巴,电子商务,WOW,射手座,魔兽世界,狗狗
时尚,健康,旅游,星座命理,美食
萌宠,名人明星
追星,双鱼座,文字控,星座控,努力,情感,音乐迷,傻,拍照,孩子气
新闻资讯,投资理财,军事,健康
宠物,心理学,真实诚信,养生保健,公益,单身,独立第三方理财,美食
红酒,时尚,电影,旅游,平常心,浪漫,偶喜欢玩,乐观,睡觉,…
汽车,自动变速箱维修,自动变速箱
美食,娱乐
旅游
IT 数码
时尚
教育就业,新闻资讯,投资理财,美食,笑话
名人明星,新闻资讯,时尚,文艺,教育就业,娱乐

名人明星,美图摄影,视频音乐
汽车,教育就业,体育
河童,骸云,双鱼,Cosplay,云雀恭弥,家庭教师
旅游,健康,新闻资讯,汽车,投资理财
汽车,体育
娱乐
旅游
萌宠
投资理财,文艺,旅游,美女
搞笑幽默
微博奇葩,穿衣美容,新闻趣事,搞笑幽默,健康,新闻资讯,时尚,旅游,娱乐,巨蟹座
娱乐,旅游,美食
还好、还好
校园生活,摄影
娱乐
热爱生活,时尚达人,娱乐
美食
旅游
娱乐
娱乐
教育就业,旅游,美女,健康
美食
时尚,游戏动漫,军事
名人明星
旅游,视频音乐
IT 数码,文艺,体育
旅游
新闻资讯,旅游,健康,体育,IT 数码
文艺
美食,篮球,NBA,游戏动漫
四川生活,娱乐
热爱生活,喜欢旅游,体育,娱乐
旅游
健康,菩提,空义,大乘,因缘,人生,龙树菩萨,文殊菩萨,佛学
旅游
名人明星,娱乐
美食
玩儿,网购
旅游
美食,旅游,羽毛球,小说
健康,生活,80 后
健康
时尚,旅游,星座命理,美食,娱乐
新闻资讯,教育就业,娱乐

投资理财,旅游,文艺,音乐爱好者,开心党,乐天派,真诚,豪爽
微博奇葩
新闻趣事,IT 数码
体育
美食,星座命理
健康,娱乐,汽车,旅游,投资理财,乐观,创业,体育
美食,新闻资讯,教育就业,汽车
投资理财
娱乐
白羊座,动漫
时尚,旅游,美食,文艺,娱乐
星座命理
游戏动漫
旅游,穿衣美容,搞笑幽默,新闻趣事,微博奇葩
笨蛋属性天然呆,死宅,吃货一枚减肥中,矫情
其他生活,新闻趣事,星座命理,穿衣美容,美食
时尚,旅游,新闻资讯,教育就业,投资理财
体育资讯
新闻趣事,校园生活,IT 数码
音乐控,金融,看小说,b 型血,财经,跳舞,90 後,心理学,爱旅游,爱电影
投资理财,K 歌,拍照,听歌
旅游
军事
美食,水瓶座,电影,读书,玩儿,吃货,娱乐,乐观,睡觉,听歌
美女,IT 数码,体育,旅游,星座命理,有想法,90 后,乐一族,O 型血,喜欢音乐
文艺,游戏动漫
星座命理,娱乐,时尚,美女,健康
娱乐,游戏动漫,狮子女,动漫控,仙剑,摄影,宅女,腐女,原创,格子控
时尚,星座命理,美食,文艺,娱乐
游戏动漫
娱乐,幸福简单之家
旅游,时尚,娱乐,美食,汽车
娱乐,美女
美食,教育就业,健康,新闻资讯,旅游
美食
穿衣美容
娱乐
旅游,新闻资讯,教育就业,汽车,文艺,宠物,爱笑爱撒娇嗨皮
时尚
健康,新闻资讯
单身,爱睡觉,娱乐,美食,听音乐,创业,汽车,爱唱歌
搞笑幽默
时尚
旅游

懒,睡觉睡到自然醒,低调,90后但我不脑残,林俊杰
投资理财,汽车,教育就业,IT数码,电影,宅,听歌,善良,交友,90后
娱乐
美食,健康,时尚,娱乐
旅游,投资理财,教育就业,健康,美食
汽车,时尚,游戏动漫,美食
时尚,轻微洁癖,爱购物,爱音乐,爱看书,完美主义者,k歌
美女
文艺
视频音乐,时尚,文艺,娱乐
健康,美食,旅游
水瓶座,蓝色,爱狗族
平常心,看书,动漫,音乐,羽毛球,摩羯座
旅游,星座命理,娱乐,文艺
美女,時尚,爱幻想,我就是我,爱玩
美食,星座命理
aesop,julique,悉尼,代购,保健品,UGG,奶粉
健康,星座命理,美食,娱乐
爱笑
时尚
90后非脑残,互粉互注,红尘,星座命理
IT数码,文艺,旅游,健康,新闻资讯,时尚
健康,新闻资讯,投资理财,文艺,娱乐
新闻趣事,搞笑幽默,名人明星,穿衣美容
游戏动漫
偷懒时听广播
水瓶,动画,夜猫子,向日葵,努力,书,喜欢旅游,茶,西班牙,梦想
毅力,武汉,程序员,硬件,审计,计算机软件,单身,装饰装潢
热爱生活,体育
游戏动漫,文艺
新闻资讯
旅游,健康,时尚,星座命理,娱乐
旅游,美食
视频音乐,萌宠,美食,旅游,搞笑幽默
跆拳道,文艺,健康,星座命理,美食,听歌,电影,日志,睡觉,旅游
健康,美女,时尚
文艺,娱乐
游戏动漫
旅游,服装,完美主义,音乐爱好者,IT数码,时尚,娱乐
娱乐
教育就业,旅游,美食
星座命理,IT数码,体育
旅游,美食,文艺
文艺,娱乐,星座命理,时尚,受,熊,处男

旅游,美食,IT 数码
IT 数码,投资理财,星座命理
新闻资讯
娱乐
搞笑幽默
创业,平常心,娱乐,紫色,偶喜欢玩,手机,阅读,宅,美食,电影
新闻资讯,军事,体育
笑,自行车,运动
旅游,投资理财,教育就业,健康
旅游,南通生活,感情动物,小说控,双鱼座,善良
爱幻想,陈奕迅,购物狂,欧美控,古典艺术,宅女一枚,家居,看电视,双重性格,小清新
其他生活,神经质,幻想,建筑,睡懒觉,迷糊,吃货,拍照,微笑,亲情
旅游,哈哈,音樂
旅游,IT 数码,游戏动漫,汽车,体育
旅游,穿衣美容,星座命理,视频音乐,吃喝玩乐,多愁善感
美食,旅游
旅游,健康,时尚,投资理财,军事
健康,新闻资讯,汽车,投资理财,文艺
军事,投资理财,时尚
旅游,阳光,向日葵
娱乐,新闻资讯,星座命理,美食,文艺
美食,游戏动漫,旅游
娱乐
孩子气,靠谱,港台剧,旅游,巨蟹座,海贼王,好性格,美食,90 后,听歌
美图摄影,动漫,搞笑幽默
摄影,天蝎座,旅行,时尚,娱乐,90 后,吃货,睡觉
电脑,淘宝,美食爱好者
美女,游戏动漫,健康,星座命理,娱乐
穿衣美容
健康,旅游,新闻资讯,星座命理,美食
美女,时尚
新闻资讯
娱乐,旅游,明星,耽美
美女,IT 数码,星座命理,旅游,美食
旅游,星座命理,娱乐
电音控
穿衣美容,微博奇葩,星座命理,健康,旅游,美食
新闻资讯,娱乐
娱乐,教育就业,新闻资讯,文艺
一点也不蠢,妖魔党,兵短 NC 粉
写作,幽默
汽车,投资理财,教育就业
娱乐
体育

美食,文艺,教育就业,低调,交朋友,生活,吃
星座命理,体育
名人明星
星座命理
美女
美食
90 后非脑残,喜欢旅游,广州人,胡思乱想,志愿者,卡卡,任性,宅男
旅游,搞笑,金牛座,旅行
烹饪,看戏,平常心,听歌,美食,读书,摄影爱好者,英语,旅行
新闻趣事,旅游,萌宠,微博奇葩,搞笑幽默
宅
娱乐
新闻资讯
旅游,娱乐
视频音乐
处女座,羽毛球,电影,网络,动漫
健康,旅游,星座命理
投资理财,健康,娱乐,善良,80 后,交友,宅男
美女,旅游
健康,美食,新闻资讯,文艺,旅游,IT 数码,教育就业,军事,体育
IT 数码
体育
广东生活
时尚,娱乐,汽车
教育就业
金华,室女座,乒乓球,湛江,合肥,权利,建材,慎重
教育就业
美食
游戏动漫,时尚,娱乐,健康,旅游
教育就业,文艺,新闻资讯,健康,旅游
名人明星
上网,睡觉,美食控,韩剧,双鱼女,淘宝
体育,娱乐,时尚,美女,军事
新闻 910,时尚,爱音乐,淘宝控,购物,创业者,90 后,美食控,美容,网络
健康
美食,美食,时尚,文艺,娱乐,旅游,星座命理
娱乐
星座命理,美食,名人明星,微博奇葩,搞笑幽默
新闻资讯,美食,娱乐,健康,教育就业
旅游,房产,车世界,潍坊生活,搞笑幽默
投资理财,娱乐
健康,时尚,旅游,娱乐
娱乐,游戏动漫,美女,星座命理,美食
宠物控,电影迷,电器迷,吃货

体育
文艺,体育
搞笑幽默
娱乐
汽车
星座命理
美图摄影
爱幻想,懒人,爱吃,画画,强迫症,睡觉睡到自然醒,爱旅行,萌宠,看书
旅游,搞笑,八卦
文艺
时尚,旅游,美食
单身
旅游,美食,搞笑幽默,云南生活,时尚,文艺,娱乐
体育
美食
旅游,河南生活
国产片,旧物,人文,青花瓷,老城区,猫,大理,菲林相機,影像,陈升
星座命理,旅游,美食
文艺,星座命理,健康,旅游,美食
娱乐
旅游
睡觉,处女座,90后,偶喜欢玩,大学生,体育,音乐,美食,校园
娱乐,体育
宅女,90后,电影,吃,语录,偶喜欢玩,手机控,执着
读书分享
汽车
新闻资讯,健康,美食,娱乐,文艺,星座命理,时尚,游戏动漫
健康,游戏动漫,美食,旅游
汽车,军事
成都生活,投资理财,爱唱歌,懒人,成都,新闻资讯,美食,旅游,教育就业,健康
旅游,娱乐
美食,星座命理,娱乐
美食
健康
新闻资讯,健康,时尚,文艺,IT数码
游戏动漫,军事,美食,娱乐,美女
美女,美食,时尚,娱乐,体育,爱动漫,旅游爱好者,喜欢摄影,街舞观者,爱音乐
搞笑幽默,视频音乐,新闻趣事,美食,动画设计,蒂姆伯顿,乙一,黑暗文学
投资理财,健康,星座命理,美食
游戏动漫
体育
投资理财
健康
星座命理,汽车,时尚,文艺,娱乐

旅游
时尚
新闻趣事
TVB,爱自由,桌球,天蝎,电影控
自由
娱乐,美女,军事,IT 数码
间歇性精神异常,白羊座 A 型血,有生活品位,Passion,超完美主义,经典 80 后
自由,学生,唱歌
旅游,星座命理,美食,时尚
大连生活,IT 数码,旅游,视频音乐,体育资讯
旅游
书迷
娱乐
脾气怪的妞
美食
搞笑幽默
旅游,阅读,摄影,看书,o 型血
美女,军事,IT 数码,旅游,汽车
时尚,旅游,星座命理,美食,娱乐
晨不离则橙不弃,乐橙
娱乐
汽车,文艺,教育就业,体育
美图摄影,军事
娱乐
美女,IT 数码,视频音乐,搞笑幽默
娱乐
健康
唯美的,义乌,广州,慎重,市场,电影
美食,爱猫,双重性格,90 后非脑残,内向
美女,旅游
美食,文艺,娱乐,健康,时尚
旅游
时尚,健康,旅游,美食,娱乐
星座命理,游戏动漫
IT 数码,射手座,上网,手机,宅,音乐,自由,吃
体育,娱乐
教育就业
新闻
搞笑幽默,时尚,90 后,偶喜欢玩,吃货,偶稀饭睡觉觉,听歌,文字
听音乐， jay
韩剧,沈昌珉,仙后,jyj,tvxq,射手座,金俊秀,郑允浩,朴有天,金在中
健康,唱歌，运动
美食
时尚,旅游,美食,星座命理,娱乐

美容服饰,名人明星,视频音乐,美食,西安生活
娱乐
旅游,美食,搞笑幽默,军事,娱乐
美食,健康,教育就业,旅游,星座命理
美食,健康,新闻资讯,时尚,汽车
体育资讯,体育
80 后
美食
时尚,美食,星座命理,文艺,娱乐
双鱼座,好性格,囧,旅行,80 后
铂爵,钟爱一生,高端婚纱摄影,鼓浪屿婚纱摄影,婚纱,摄影
视频音乐
囧
小说,水瓶座
睡觉,宅,生活,校园,美剧,自由,听歌,旅行,动漫,时尚
搞笑幽默
投资理财
美女
时尚,旅游
旅游,IT 数码,美食
日式,日式料理,装修顾问,升降桌,日式装修,格子门,小空间装饰,榻榻米
汽车,体育
星座命理
90 后但我不脑残,EXO,互粉,高三,医学,吃货,世勋,鹿晗
美女
敏感,独立,欧美控,名人明星
文艺,时尚,娱乐,美食,汽车,健康,旅游
幽默,90 后
摩羯座,学生,吃货,听歌,宅女
游戏动漫,塞夏,小黑子,佐鸣,土银,黑花,瓶邪
旅游,微博控,单身,天秤,90 後
旅游,游戏动漫,美食,军事,娱乐
旅游,美食,文艺
旅游
游戏动漫,健康,汽车,时尚,美食
星座命理,80 后
单身,85 后,时尚,傻,书籍,猫咪,睡懒觉,心理
文艺
IT 数码,文艺
教育就业,文艺,时尚,美食,娱乐
旅游
教育就业
汽车,新闻资讯,健康,投资理财
星座命理

萌宠,穿衣美容,旅游,美食,星座命理,时尚
广东生活
美食,IT 数码,体育
教育就业
搞笑幽默
星座命理
娱乐
娱乐
汽车,美女,新闻资讯,健康,军事
海绵宝宝控,娃娃控,旅游
新闻趣事,单曲循环控,淡淡的忧伤,神秘感,低调的华丽,成长 ing,河图控,原创小说,网络文学,古风音乐
唱 K,处女座,偶稀饭睡觉觉,手机控
美女,汽车,娱乐,健康,IT 数码,新闻资讯,时尚,投资理财
娱乐
美女,美食
穿衣美容
文艺
旅游
文艺
旅游,星座命理,时尚,美食,娱乐
新闻趣事
星座命理
美女
旅游
吉他,爱笑
穿衣美容,新闻趣事,搞笑幽默,视频音乐,旅游
搞笑幽默
娱乐
娱乐
时尚,美食,星座命理,美女,若战并肩作战,若败共赴黄泉,吃货一枚,莫失莫忘,莫离莫弃
摄影
文艺,吃货
小说,林宥嘉,吃货,乐观,音乐控,90 后但我不脑残,花痴,微笑,白羊
旅游
双鱼座
美食
美女,旅游,美食,军事,体育,娱乐
时尚,游戏动漫,美女,文艺,美食,健康,娱乐
听歌,偶稀饭睡觉觉,娱乐,噢噢坚持嗯哼
美食,搞笑幽默,微博奇葩,爱吃,看书,天秤座,睡觉
台球,公益,爱情,体育,喜欢音乐,愤青,柯南,爱唱歌,广交朋友,为人和善
美食,娱乐,汽车,军事,旅游
娱乐

美食
重庆生活
娱乐
健康,八零后,摇滚,孩子妈
时尚
游戏动漫
微博奇葩,爱生活,爱板砖,爱 DOTA,没背景,没未来
娱乐,玉米，苞谷，90 后
旅游,星座命理,美食,IT 数码,娱乐
旅游,美食,文艺,时尚,娱乐
美食,文艺,健康,旅游
时尚,新闻资讯,旅游,美食,娱乐
财经
内向，打台球，
体育
星座命理,学生一族,双子女,吃货一枚,运动
美食
投资理财
偶喜欢玩,美剧,文艺,看电影,爱音乐,阳光
体育资讯
美女,游戏动漫,旅游,体育,汽车,IT 数码
美食
好书,运动,旅游,美剧,写作,广告,网购,文字,吃货,自由
仙剑,插画,运动,宅女,视频音乐,搞笑幽默,新闻资讯,星座命理,娱乐,文艺
体育,新闻资讯
游戏动漫
美食
旅游
游戏动漫,体育
小说,数码
星座命理,时事,活泼,营销
IT 数码,娱乐
美女
90 后,创业
北京生活,搞笑幽默,旅游,微博奇葩,萌宠,汽车
星座命理,美食,IT 数码,娱乐,旅游
美食
娱乐,文艺
美食,文艺,好书,羽毛球
汽车
时尚,旅游,IT 数码,娱乐,天蝎座,浪漫,吃货,睡觉,旅行,90 后
美食,花花草草,天秤座
时尚
玩儿,网购,好性格,吃货,双鱼座,游戏,美剧,英语,宅,旅游

星座命理,星座,发呆,听音乐
健康,游戏动漫,汽车
名人明星,星座命理,其他生活,新闻趣事,文艺
时尚
健康
IT 数码,游戏动漫,QQ799659027
美食
游戏狂
美女
美女,网购,时尚,90 后,音乐
爱美食,时尚,汽车,娱乐,体育,美食
旅行,白羊座,篮球,好书,自由,体育,偶喜欢玩
娱乐,体育
旅游,美女,汽车,美食,IT 数码
娱乐,文艺
魔蝎座,Music,学生,体育,汽车
萌宠,新闻资讯,时尚,文艺,教育就业
韩国生活,应聘请私信皮下,以下条件不招聘,年龄小于 16 岁,高三党,初三党
曼联 fans,偶喜欢玩,摩羯座,篮球,伪五迷,吃货,90 后,NBA,学生,体育
文艺,时尚,娱乐,投资理财
双鱼座,休闲,勇敢,主观,上海,电影
旅游
娱乐
投资理财,汽车,IT 数码,美女,时尚,电影,美剧,美食,动漫,狮子座
摇滚,音乐,涂鸦,街舞,旅遊
电视剧,爱睡觉,90 後,小清新,拍照
校园,双子座,唱歌,偶稀饭睡觉觉,音乐
听歌,80 后
搞笑幽默,穿衣美容,微博奇葩,美女,旅游,美食
大学生,校园,爱情,网购,旅行,听歌,语录,摄影,90 后,音乐
娱乐,健康
旅游
文艺,新闻资讯,健康
汽车,体育,娱乐,旅游,军事
投资理财,旅游,体育
旅游,美食,文艺,体育,娱乐
时尚
美女,军事,IT 数码
娱乐,新闻资讯
旅游
音乐爱好者,游戏达人,平常心,玩儿,互联网,微博,睡觉,电影
旅游
娱乐
奋斗，旅游。

爱旅游, 小提琴, 投资理财, 金牛座, 健身
校园生活, 学生一族
足球, 财经
娱乐
IT 数码, 娱乐, 汽车, 时尚, 美女
校园生活
娱乐, 旅游, 星座命理, 美食
汽车, 投资理财, 美女, 处女座, 喜欢睡觉
旅游, IT 数码, 娱乐, 时尚
动漫迷, 幽默, 吃货, 旅游
微博奇葩
投资理财, 文艺, 教育就业, 新闻资讯, 军事
汽车, 旅游, 娱乐, 体育
美食, 宁波生活
汽车, IT 数码, 体育, 打球
旅游, 文艺
健康, 旅游, 星座命理, 美食, 投资理财
娱乐
美食, IT 数码, 宅女
上网, 无聊, 喜欢旅游, 户外, 听音乐, 魔羯座, 幸福, 影视, 经济, 娱乐
IT 数码
名人明星, 校园生活, 视频音乐, 搞笑幽默
星座命理, 健康
IT 数码, 时尚, 旅游, 健康, 美食
美食, 星座命理, 睡觉
时尚
荆州, 勇敢, 金牛座, 帅哥, 反应佳, 恋爱, 双鱼座, 游戏, 硬件, 济宁
健康, 旅游, 美食
视频音乐
投资理财
健康, 文艺, 星座命理
微博营销, 中小企业, 分享, 交流
IT 数码, 文艺, 军事, 游戏动漫, 体育, 娱乐
美食
体育
IT 数码
搞笑幽默, 娱乐, 健康, 新闻资讯, 军事, 游戏动漫
星座命理, 游戏动漫, 军事, IT 数码
美女
名人明星, 搞笑幽默, 新闻趣事, 微博奇葩, 美女
篮球, 玩儿
美女, 时尚, 美食, 健康, 投资理财
星座命理, 美食, 时尚, 健康, 旅游
美食, 星座命理, 娱乐, 疑似自闭症患者, 后知后觉, 宅女, 90 后, 吃货, 小说, 玩儿

星座命理,娱乐,美食
娱乐
旅遊,文藝,健康,星座命理,美食
美女,幽默,好书,小说,电影
射手座,动漫,学生,音乐,吃货,网购,90后,看书
玩儿
娱乐
江门,餐饮,咨询,建筑,清远,审计,顺德
教育就業
美国生活
文艺
美食,学海无涯乐作舟
游戏动漫,美食,文艺
美女
星座命理,新闻资讯,健康,时尚
军事,IT数码,体育,新闻资讯
文字,偶喜欢玩,生活,天蝎座,80后,旅游,广告,艺术,音乐
汽车,军事
教育就业
星座命理
90后,天秤座,电影,好性格,学生,电子商务,摄影,游戏,浪漫,音乐
奋斗,搞笑,吃货,古灵精怪
美食
游戏动漫
旅游,文艺,健康,美食,星座命理,娱乐,游戏动漫
自信,美食
娱乐
文艺,娱乐
旅游,星座命理,时尚,文艺,娱乐
新闻趣事
文学,重庆,内向,爱旅行
美食,运动,交友,电影
娱乐,旅游,新闻资讯,教育就业,投资理财
IT數碼
传媒,网络
体育
陈奕迅,海绵宝宝,金牛座
史迪仔控,柯南柯南,交友,大爱费德勒,美食,吃,90后,旅行,双子座
星座,看电影,钓鱼,美食,摩羯座,媒体,文字,历史,好书,自由
美食,美女,时尚,汽车,体育
旅游
好性格,80后
外贸数据,外贸营销,游戏动漫,健康,星座命理,美食,时尚
美食,军事,IT数码,娱乐,起床困难户,奋斗ing,体育,听歌

娱乐
IT 数码,交友,设计,时尚,摄影爱好者,天秤座,广告,生活,搞笑,听歌
摩羯座,艺术,财会,湖州,细心,足球
羽毛球
新闻资讯,游戏动漫,时尚,教育就业,娱乐
旅游,穿衣美容,萌宠,微博奇葩,美食
IT 数码
旅游
读书分享
美食
旅游,美食
狗狗,懒人,学生一枚,法律
投资理财,新闻资讯,IT 数码,娱乐,旅游
自古最右,最右神回复,最右,最右神,神最右,最右君
健康
新闻资讯,文艺
爱幻想,看小说,爱音乐,驴友,敏感
唱歌
however
娱乐,健康,美食,星座命理,娱乐八卦,吃,生活,游戏,天秤座
交友,广告
IT 数码
名人明星,户外,音乐爱好者,服装,韩剧,日剧,新闻资讯,购物,听音乐,蓝天白云
名人明星,武艺控,水瓶座,郭敬明,90 后
文艺
旅游,文艺小青年,写作,个性 Ing,运动，,娱乐
美食
星座命理,双子座,偶喜欢玩,听歌,囧,羽毛球,大学生
穿衣美容
体育
旅游,财经,无敌最寂寞,实德,现货黄金,纸黄金,纸白银
旅游,穿衣美容,微博奇葩,视频音乐,搞笑幽默,时尚
喜欢看小说,双子座女生
穿衣美容,搞笑幽默,微博,美食
微博奇葩,乐观,好性格,平常心
美食,游戏动漫
旅游,文艺,时尚,IT 数码,娱乐,85 后
设计
美食
IT 数码,美女,汽车,旅游
美食
文艺,教育就业,健康,英语,天蝎座,体育,旅行
旅游,投资理财,美食,新闻资讯,健康
军事,新闻资讯,体育,美女,娱乐

我就是我,双鱼,爱吃,自由,唱歌,上网,看书,动漫,睡觉,学生
热情,无聊,努力,跑步,忧郁,喜欢唱歌,健康,大学
户外,旅游,交友,娱乐
武汉大学
宠物图集,狗狗,我就是我,看电视,爱笑,懒,安静,90 后,吃货,宅
文艺,美食,投资理财,教育就业,健康
健康
生活
名人明星
娱乐,时尚,旅游,星座命理,美食
美食,IT 数码,健康,旅游,游戏动漫,时尚,教育就业,新闻资讯
天枰座,搞笑幽默,游戏动漫,体育
新闻资讯
美女,旅游,星座命理,娱乐,汽车,健康
军事,美女
学生,单纯,汽车
美图摄影
搞笑幽默
视频音乐
星座命理,游戏动漫
旅游
新闻资讯,投资理财,娱乐,教育就业,IT 数码
健康
旅游,时尚,汽车,娱乐
娱乐
旅游,文艺,娱乐
娱乐
123,读书
其他生活,新闻趣事
娱乐
旅游,文艺,天秤座,自由,80 后
游戏动漫
健康,共生共赢,拒绝平庸,魅力服务,有效执行,整合资源,智造品牌
美食
时尚
低调,房地产,武汉,天平座,随性
美食
文艺,体育,IT 数码,美食,美女
汽车,摇滚,自由,个性,商业,英语,管理,皇马,投资理财,真诚
企业培训,企业家培训,北京大学,经济学院
广州生活
投资理财
娱乐
美容服饰

文字控,O 型血
纠结,宅女一枚,无聊,美食
平常心,巨蟹座
双子座,新闻,NBA,财经,八卦,英语,看书,听歌,电影
爱笑
美食,旅游,微博奇葩
娱乐,旅游,健康,星座命理
搞笑幽默,微博奇葩,星座命理,穿衣美容,美食
美食,艺术,数码,时尚,摄影爱好者,90 后,设计,天蝎座,坚强的女生会哭,不会输
健康
雙子女,娱乐,自由,时尚,宅,美食,上网,学生,睡觉
娱乐
大连生活
星座命理,新闻资讯,美食,时尚,娱乐
宅
爱生活爱上大,笑点低哭点更低,纠结的矛盾体,追求完美,爱钻牛角尖,90 後
动漫,海贼迷
军事
娱乐
娱乐,星座命理,健康
美食,旅游,健康,时尚,英语,浪漫,偶稀饭睡觉觉,阅读,旅行,吃货
教育就业
时尚,旅游,娱乐
旅行,乐观,80 后,足球,运动,篮球,幽默,美食,玩儿,NBA
娱乐
IT 数码,星座命理,美女,游戏动漫
美食
娱乐
电视迷,IT 人,时尚达人
时尚,汽车
娱乐
IT 数码,八卦杂谈,名人明星,美女,敏感,善良,吃,90,时尚,懒人一枚
视频音乐,校园生活,体育资讯,美图摄影,旅游
娱乐
星座运势
娱乐
浙江生活,教育就业,游戏动漫
陈奕迅,青春,感性,美食,90,漫画,爱自由,音樂,学生一族,搞笑幽默
旅游
天秤座,团购,美女,军事,健康,网购,交友,旅游
文艺,美女,网购,交友
文艺,熊猫控,文字,阅读
星座命理
美食,穿衣美容,旅游,健康,娱乐,投资理财,教育就业

上海生活
自尊心,上海,反应佳,运动,深圳,恋爱,肇庆,乒乓球
教育就业,投资理财,健康,IT 数码,文艺,紫色,吃货,90 后,羽毛球,天蝎座
旅游,美食,IT 数码,文艺,娱乐,TVXQ,仙后
时尚,星座命理,美食,文艺,游戏动漫
新闻趣事
娱乐
健康
单身
IT 数码,游戏动漫,旅游,文艺,娱乐
星座运势
商业地产,房地产营销,昆明万科
健康,新闻资讯,游戏动漫,汽车,体育
美食
90 後,A 型血,射手座
娱乐
旅游
美食,娱乐,时尚
总裁职场,语录,职场专家,职场语录,职场,总裁
萌宠
愤青,军事迷,80 后,70 后
宠物图集
星座命理
美图摄影,宁波生活,美食,听歌
电影迷,打球
教育就业,旅游,投资理财,军事,文艺,游戏动漫,美食,新闻资讯,美食,体育
时尚,投资理财,星座命理,美食,娱乐,偶稀饭睡觉觉,吃货,电影,听歌
巨蟹座,网购
时尚
爱老婆,就爱老婆,只爱老婆
健康,美女,汽车,旅游,IT 数码
新闻趣事
90 後,低调,单身,阳光,懒,快乐,安静,music,爱旅行,简单
大学,平常心,音乐
白羊女,les
体育
喜欢音乐
时尚
健康,游戏动漫,美女,娱乐
视频音乐
微博,宅男,90 后,幽默,上网,音乐
眼镜一族
娱乐
星座命理,投资理财,IT 数码,文艺,美女

大学生,美食
时尚,美女,美食,投资理财,旅游
旅游,美食,星座命理,互联网
娱乐
重庆,新闻趣事,新闻资讯,教育就业,IT 数码
幽默开朗
吃货,幽默,处女座,搞笑,听歌,读书,上网,唱歌,英语,90 后
旅游,宜昌生活,平常心,善良,旅行,80 后,囧,宅
视频音乐
新闻资讯
时尚,文艺,闷骚,小女人,肉肉脸
亲情
天秤座,摄影,文学,听歌,美食,微博,新闻,生活,自由
视频音乐,穿衣美容,名人明星
四川生活
音乐,亲情,小说,看书,听歌
旅游,游戏动漫,IT 数码,娱乐
IT 数码,健康,投资理财,教育就业,军事,文艺
时尚,娱乐
IT 数码
吃货
健康,旅游,星座命理,文艺,娱乐
娱乐,时尚
搞笑幽默
爱生活,爱自己,爱他人
教育就业,游戏动漫,健康,旅游,文艺
美食,亲情
星座命理
美女,广东生活,投资理财
健康,新闻资讯
狮子座,旅游,听歌,音乐
美食
教育就业,美食,时尚,娱乐,文艺
宠物控,电器迷,游戏达人,吃货
IT 数码,新闻资讯,游戏动漫,投资理财,娱乐,闷骚二货,木头,骑行男淫,不懂爱,不懂怜香惜玉
娱乐,新闻资讯,游戏动漫
江门,制药,哈尔滨,人马座,江阴,宅女
偶稀饭睡觉觉,电影控,潮汕的,中大南方学院,普宁勤建
健康,投资理财,美食,美女,旅游
娱乐,旅游,美食
贵州生活,旅游,美食,微博奇葩,搞笑幽默
新闻趣事,成都生活,文艺,笑,学生,摄影
哈尔滨,思考,广告,西安,艺术,肇庆,盐城,慎重

旅游
玩儿,电子商务
怀旧,美食,自由,听歌,书,电影控,茶,善良,睡觉睡到自然醒,旅游
文艺,IT 数码,时尚,娱乐
军事
天平座,快乐,爱吃,傻,朋友,睡觉,时尚,美食,健康
听歌,浪漫,90 后,偶喜欢玩,自由,处女座,数码,艺术,设计
懒人,德国,天枰座,自由,宅女一枚,文艺
科学探索,教育,读书分享,美女,文艺
开朗幽默阳光
律师,教育就业
星座命理
IT 人,90 后,电视迷,电影迷
梅格妮,小布丁,唱歌,时尚,音乐,幽默,学生,天秤座,90 后
美食
娱乐,处女座,听歌,宅女,八卦,旅行,90 后
西安,淡定,跑步,科比,计算机,军事,美食,旅游,健康,美女
军事
移民,香港生子,坐月子,月子中心,赴美生子,二胎,母婴
好性格
健康,梦想徒步旅游,自由,运动,射手座,幽默,80 后
美食,娱乐,健康,旅游
时尚
娱乐,体育,爱篮球爱健身,单身
娱乐
游戏动漫
教育就业
娱乐,时尚
娱乐,星座命理,美食,处女座,营销,乐观,睡觉
旅游
游戏动漫
汽车,体育,旅游,足球
娱乐
娱乐,明基模力女孩,网购,电子商务,唱歌,亲情,音乐,乐观,平常心,吃
星座命理
文艺,旅游,娱乐,美食,时尚
投资理财,新闻资讯,文艺
上海生活
足球,校园
内涵帝,BL,美食,旅游
情感生活,善良,八卦,艺术,娱乐
时尚
幽默,自由
美食

军事,汽车,旅游,投资理财,房产
淡定,cosplay,巧克力,吸血鬼,游戏动漫,星座命理,双鱼座,90 后
美食,旅游,健康,时尚,娱乐
汽车,时尚
美女,健康,星座命理,汽车,娱乐
真实力
美女
足球,小说,上网,金牛座,90 后
校园生活
新闻资讯,健康,旅游,美女,汽车
娱乐,美女,健康,时尚,旅游,美食,双重性格
游戏动漫,美女,健康,星座命理,IT 数码
美女
新闻趣事,萌宠,旅游,穿衣美容,美食
投资理财,美女
时尚
校园,娱乐
看书,唱歌,听音乐,宅
美女
教育就业,时尚,新闻资讯,文艺,娱乐,健康,旅游
娱乐,旅游,美食
影视,美腿,航空,电影
强迫症,重口味,文字,吃货
萌宠,旅游,徐州生活,视频音乐,美女
投资理财,美食,时尚,旅游
美食,健康,星座命理,投资理财,旅游,雙魚女,b 型血,旅游美食爱好者
韩饭,电视迷
微博奇葩,星座命理,新闻趣事,美食,美国生活
语录,听歌
科比
娱乐,教育就业,健康,旅游,文艺
文艺,时尚,美食,游戏动漫,娱乐
微博奇葩,娱乐,IT 数码
游戏动漫
听音乐,爱音乐,看书,电影爱好者
星座命理,新闻趣事,美食,旅游,穿衣美容
平常心,听歌,语录,玩儿,天蝎座,汽车,好性格,睡觉,手机,90 后
游戏动漫,IT 数码
文艺,体育
范 er,美食
美食,星座命理,时尚,教育就业,娱乐
时尚,汽车,旅游
视频音乐
新闻趣事,穿衣美容,美女,旅游

IT 数码,汽车
美食
体育资讯,搞笑幽默,美女
教育就业,美女,游戏动漫,军事,投资理财
星座命理
旅游,汽车,投资理财,体育,IT 数码
教育就业,娱乐,新闻资讯
娱乐
时尚,星座命理,美食,IT 数码,娱乐
文艺,投资理财,教育就业,新闻资讯,美食
旅游,新闻资讯,体育,汽车,军事
搞笑幽默
生物,狮子座,自觉,建材,海口,嘉兴
旅游
美食
美食
新闻资讯,健康,教育就业,投资理财,娱乐
理财,坚强,喜欢听歌,开朗,阳光,家居,心情,美食,文艺,旅游
偶喜欢玩,运动
视频音乐,搞笑幽默,汽车,游戏动漫,旅游,美食,文艺
情绪化,蓝色,自由职业,看书,旅游美食爱好者,射手
体育
睡觉睡到自然醒,旅行,韩剧,快乐,学生,看电视,吃
懒人 QAQ,属性单一,努力成为小透明,98line
南京生活,广播,music,小清新,南京,蓝色,强迫症,方大同,学生一枚
旅游
文艺,旅游,体育
美女,游戏动漫,时尚,美食,文艺,娱乐
时尚,美食
娱乐
读书分享,情感生活,美容服饰,动漫,星座命理,穿衣美容,微博奇葩,新闻趣事
美女
文艺,游戏动漫,星座命理,新闻资讯,时尚
旅游,美食,体育,时尚,娱乐
体育
新闻趣事,时尚,搞笑,水瓶座,吃货,美食,传媒
上网
时尚,健康,旅游,美食,娱乐
投资理财,时尚,健康,星座命理,娱乐
星座命理
新闻资讯
浪漫
娱乐,美食,健康,新闻资讯,投资理财,旅游,星座命理
星座命理,新闻趣事,搞笑幽默,旅游,陕西生活

吃货,懒,狮子座
游戏动漫,投资理财,旅游,军事,IT 数码,浪漫,新闻,搞笑,天蝎座
投资理财,美食,旅游,星座命理,娱乐
90 后非脑残,恐怖片,睡觉睡到自然醒,学生一枚,爱睡觉,帅哥,爱旅行,多愁善感,双鱼,吃货
美女
旅游,新闻趣事,搞笑幽默
文艺,健康,旅游
时尚
我吵我闹我骄傲
军事,IT 数码
投资理财
汽车
教育就业,星座命理
美图摄影
娱乐
美食
投资理财,旅游,星座命理,游戏动漫,美女
名人明星
奋斗 ing
穿衣美容
美食
90 後,游戏动漫
美女
健康
名人明星
旅游,星座命理
名人明星,仙后
美女,玩儿,90 后
会计,扬州,宝瓶座,财会,烟台,宅女,乒乓球
美女,美食,军事,体育
文艺
军事
体育
书法,茶,瑜伽,爱电影,武艺,偶像剧,高尔夫,吃喝玩乐,佛教
汽车,美女,娱乐
穿衣美容,旅游,爱旅游,拍照控,时尚搭配,淘歌达人,双鱼座
IT 数码,军事,娱乐,体育,新闻资讯
搞笑
游戏,美女
微博奇葩
吃货,浪漫幻想,善良,旅游
美食,球球球球
微博奇葩,星座命理,名人明星,喜新不厌旧,各种分裂,白羊座,猴子控,偏执狂
画画,韩国,摄影,爱旅游,90 後,双鱼座,艺术

娱乐
美食,萌宠,臭袜子,长得俊,喵星人,偶喜欢玩
旅游,星座命理,文艺,美食,娱乐
教育家,音乐控,笑话控,旅行
时尚,健康,娱乐,投资理财,旅游
娱乐,吃货,巨蟹座
新闻资讯,文艺,美食,军事,娱乐
军事,旅游,投资理财,娱乐,汽车
文艺
美食
看电影
美食
汽车
唱歌,玩儿,听歌,电影,天秤座
星座命理
美图摄影,娱乐,健康,汽车,时尚,教育就业
美女,Specialized
搞笑幽默,新闻趣事,视频音乐,美食,星座命理
时尚
时尚,娱乐
旅游,美食,体育,文艺
体育
美女
旅游
健康,时尚,旅游,娱乐,美食
安静,爱笑,唱 K,星座命理,娱乐
音樂
视频音乐
时尚,美食,健康,旅游
宅,自由
刺激,探险,摄影,电影,自由,旅游
星座命理
美食
旅游,深圳,b 型血,80 後,爱旅游,爱电影
时尚
时尚
用毅力战胜一切,古典音乐,滑雪,小提琴,工作狂,活泼,快乐,漫画,游戏动漫,偶喜欢玩
体育
旅游,阅读
学习 ing
新闻资讯,娱乐
广州,毅力,思考,常熟,牺牲奉献,搞笑
搞笑幽默
游戏动漫,投资理财,体育,军事

新闻资讯,看书,运动,旅游,体育,历史
美女,游戏动漫,娱乐
娱乐,旅游,文艺,星座命理
我就是我,简单,动漫控,亲情
简单,努力 ing,爱笑,比较宅,学生,假小子,电视剧,傻的可爱,囧,90 后非脑残
旅游,暮光之城,那些花儿,红色高跟鞋
健康
娱乐,时尚,星座命理
听音乐,娱乐
星座命理,喜欢音乐,粉色控,蝴蝶结控,白羊座
美食,旅游,新闻趣事,搞笑幽默,新乡生活,网购,娱乐,上网,看书
穿衣美容,萌宠,旅游
时尚,V 迷,偶稀饭睡觉觉,处女座,听歌,90 后
新闻趣事,校园生活,搞笑幽默,名人明星,体育资讯
时尚,美食
美女,名人明星,旅游,微博奇葩,新闻趣事
美食,游戏动漫,IT 数码,娱乐,旅游
娱乐
娱乐,IT 数码,星座命理,美女,游戏动漫
健康
宅,爱情
投资,设计师,k 歌,IT 数码,android,旅游美食爱好者,原创,文化,自信,休闲
教育,读书分享,新闻趣事,搞笑幽默,IT 数码
IT 数码,篮球,科比
网游
星座命理,历史
旅游
搞笑幽默
善良,游戏,电影,音乐,天蝎座,吃货
美食,王力宏,中考女一枚
娱乐
时尚
汽车,新闻资讯,美女,体育,IT 数码
健康
星座命理,美食,娱乐
单身,阳光,双子,自由,简单,视频音乐,设计,爬山
加内特,懒人,AC 米兰,纳什,意大利,卡卡
时尚,星座命理,美食,娱乐
娱乐,美食
投资理财,健康,旅游,星座命理,美食,娱乐
健康,星座命理,旅游,文艺
弟控 X 兄控,钟汉良,初次
军事
娱乐,体育,旅游,音乐,幽默,美食,吃货

体育
娱乐,旅游,美食,IT 数码,汽车
旅游,星座命理,新闻资讯,健康,教育就业,宅,互联网
体育
IT 数码,旅游
星座命理
星座命理,穿衣美容,视频音乐,美女,旅游
游戏动漫,文艺,体育,娱乐,音乐,旅行,运动,游戏,小说,看书
新闻资讯,时尚,IT 数码,投资理财,娱乐
游戏动漫
搞笑幽默
吃货,享受,小懒惰
体育,旅游,汽车,健康,游戏动漫
文艺,星座命理,旅游,健康,美食
健康,娱乐
美女
美女
星座,十二生肖
星座命理,偶稀饭睡觉觉,哈迷,萝莉,御姐,腹黑,巨蟹座
自由,电影,美食,摄影,旅游
K 歌之王,营养师,幽默睿智,喜欢浪漫,形象设计师,喜欢买车,赛车爱好者,护肤达人,chok
动漫,时尚,萌女,吃货,美妆,减肥,星座,八卦,吐槽,恶搞
美食,天蝎座
MTB,XC,AM,DH,速降,山地车,户外,自行车,骑行,旅游
记忆犹如橡皮擦,呆货,爱动漫
善良,摩羯座,美食,摄影爱好者,时尚,旅游
视频音乐
星座命理,娱乐
军事
星座命理
健康,旅游,美食,文艺,娱乐
娱乐
偶稀饭睡觉觉
天蝎座 80 后,唱歌,天蝎座,听歌,旅行,交友
看书,摩羯座,听歌,体育,旅游,语录,汽车,搞笑,幽默,美食
娱乐
校园生活,新闻趣事,视频音乐,搞笑幽默,杭州生活
奋斗,双子女,矛盾体,孩子气,沉默,DIY
钢琴
投资理财,美食,文艺,娱乐
新闻资讯,旅游,军事,体育,美女
健康,IT 数码,新闻资讯,旅游,投资理财
娱乐
美食,军事,IT 数码,文艺,体育

搞笑幽默
游戏动漫,投资理财,教育就业,星座命理
代购,奶粉,药品
IT 数码,星座命理,美食,文艺,体育
星座命理,美食,军事,动漫,旅游,吃,媒体,交友,听歌
美食
it 互联网,娱乐
新闻资讯,汽车,IT 数码
爱吃
文艺,健康,星座命理,美食,娱乐
娱乐
文艺,娱乐
娱乐,美食,时尚,文艺
张掖生活
星座命理,旅游,汽车,美食,IT 数码
娱乐,美女,投资理财
健康
军事,投资理财
热情,重庆,嘉兴,兜风,宅女,艺术,白羊座
名人明星,旅游,美食,台湾生活
健康,时尚,投资理财,星座命理,娱乐,摩羯
时尚,旅游
体育
美食,体育
美女
汽车,时尚,IT 数码
休闲,天蝎座,体育,自由职业,睡觉
投资理财,牡丹江婚纱摄影,牡丹江影楼,牡丹江写真,牡丹江,情缘
美食,娱乐
投资理财
旅游,撸管手淫,文爱,各种爱爱,寂寞爱骚货
体育,IT 数码,旅游,时尚,娱乐
时尚,星座命理,旅游,教育就业,文艺
时尚
射手座
娱乐
汽车,健康,新闻资讯,旅游,军事
射手座,武汉
健康,时尚,旅游,美食
视频音乐
海南生活,看书,网购,射手座,新闻,小说,手机零售业
IT 数码,体育,教育就业,健康
星座命理
时尚

健康,美女,投资理财,旅游,军事
乐观,玩儿,电影
时尚,游戏动漫,旅游,美食,军事,体育,娱乐
星座命理
健康,星座命理,文艺,旅游
美食,游戏,看书,语录,天蝎座,幽默,电影,写作,摄影爱好者,动漫
娱乐,文艺,健康,旅游
娱乐,体育
旅游
成都生活,校园生活,IT 数码,看书,学生一枚,音乐控,简单,娱乐,游戏动漫,美食
四川生活,体育
星座命理,游戏动漫,时尚,旅游
星座命理
江都,专题片,宣传片,MV 制作
文艺,娱乐,美女,游戏动漫
娱乐
军事
体育,军事,IT 数码
游戏动漫,宅腐基,许嵩控,动漫爱好者一只,K 歌达人
文艺
时尚,星座命理,军事,IT 数码,文艺
时尚,娱乐,健康,教育就业,美食
美食,娱乐
白羊座,小说,旅行
猫,美术,公益
音乐,偶喜欢玩,平常心,吃货,天秤座,写作,自由,90 后,学生,宅
美食,视频音乐,搞笑幽默,教育就业,时尚,文艺,困觉觉,大爱大爱陈奕迅,美剧,天蝎座
90,天平座,哈哈,电影爱好者,交朋友,桌游,小说迷,活泼开朗
汽车,庞大奇瑞,绝对不贵
美容服饰,BIGBANG,唱 K,日語,旅遊,玩,吃貨,跳舞
健康,中西医结合,身心平衡,营养顾问,康复师,健康管家,运动康复,健康管理
星座命理,时尚,体育
美图摄影
IT 数码
时尚,美食,文艺
小说
视频音乐,时尚,射手座,微博,偶喜欢玩,听歌,浪漫,上网,宅女,音乐
旅游,娱乐
军事,美食,健康,IT 数码,旅游,汽车,美女
娱乐
时尚
校园生活,搞笑幽默,起床困难专业户,宅,伟大的吃货,巨蟹座,轻音乐,of,睡觉族,星座控
文艺,时尚,娱乐,健康,美食,星座命理
新闻趣事,微博奇葩,穿衣美容

听歌上网宅美食,小说
星座命理,健康,美食,旅游,娱乐
时尚
健康,旅游,星座命理,时尚
IT 数码
游戏动漫,时尚,汽车,IT 数码,投资理财
美女
情感生活
搞笑幽默,娱乐,音乐,善良,时尚,宅
文艺,娱乐,旅游,星座命理
旅游
游戏动漫,文艺,娱乐,IT 数码
微博
时尚
柯南 FANS,BigBang,美食爱好者,权志龙 GD
NBA,英语,偶稀饭睡觉觉,互联网,微博,历史,网络,吃,篮球,动漫
娱乐
美食
健康,时尚,娱乐,旅游,美食,珠宝设计,高级珠宝定制,钻石,珊瑚,水晶
小说,手机,睡觉,旅游,80 后
90 后,平常心,玩儿,生活,体育,自由,旅游,历史,善良,篮球
美食,星座命理
健康,旅游
蓝色,猫,游戏动漫
摄影爱好者,电子商务,旅行,娱乐,射手座,时尚,网购,偶喜欢玩,紫色,90 后
旅游,善良,白羊座,美食,好性格,运动,读书
星座运势
穿衣美容,微博奇葩,新乡生活,美食
旅游,美食,文艺,娱乐,投资理财
美食,不听话的孩子
投资理财
做事真：做人糊!
名人明星,旅游,美食
看小说，听歌
美图摄影
互联网
IT 数码
文艺,时尚,健康
旅游,美食,星座命理,时尚,娱乐
深圳大学生公寓,小迷糊,处女座,宅,美食,新闻,营销,上网,80 后,创业
恋爱,双鱼座,股票,天蝎座
文艺,疯子,睡觉,吃,游戏,看书,白羊座,平常心,宅女,唱歌
汽车,IT 数码
营销,爱情,媒体,A 股,水瓶座,体育,浪漫,上网

娱乐
足球
体育
IT 数码
文艺
视频音乐
时尚,娱乐
娱乐,时尚,模特,流行时尚,广告,传媒,经纪人,摄影
汽车,健康,游戏动漫,投资理财,美食,旅游
星座命理
旅游,漳州生活
旅游,娱乐,体育
旅游
体育,篮球狂徒
娱乐
旅游,新闻资讯,健康,美食
陕西生活
许嵩,学生一枚,吃货一枚
投资理财
星座命理,旅游,时尚,娱乐,美食
双子座,吃货,时尚,唯爱 Vae,发呆,90 后,听歌,睡觉,学生,文字
旅游
教育就业
紫色,处女座,美食,自由,运动,幽默,80 后,旅游
健康,教育就業,文藝,IT 數碼
美食,星座命理,时尚,健康,旅游,偶喜欢玩,宁静,悠闲,自在,想拥有自己的店
懒,神经质,电影迷,麦霸,白羊座,美食
游戏,动漫,电视剧,军事,历史,小说,自由,旅行,音乐
微博奇葩
体育,宝宝保暖泳衣,网店店主,爱泳宝贝,婧淇妈妈,英语,创业,平常心,80 后
微博奇葩,美女,视频音乐,搞笑幽默
军事
娱乐
健康
文艺,旅游,美食,娱乐
星座运势,双子女,爱旅游,发呆,画画,睡懒觉,听歌,写字,咖啡,爱幻想
文艺,旅游,投资理财,体育
IT 数码,娱乐,美食
星座命理,旅游,投资理财,美女
神经大条,nba
娱乐
新闻趣事,校园生活,情感生活,宠物图集,搞笑幽默
逛街,文艺,发呆,浪漫控,唯美控,哈韩哈日哈欧美,学生,90 后,处女座
娱乐,游戏动漫,旅游,体育

美食,星座命理,旅游,教育就业,投资理财,乐观,偶喜欢玩,设计,大学生,狮子座
军事
名人明星
旅游
旅游
美女
星座命理
美食
娱乐
时尚,娱乐
情感生活,美图摄影,星座运势,美食,宠物图集
放生,禅,成都华藏香堂,代理加盟,佛子,佛教,纯天然加持殊胜,厂家直销,佛香,藏香
娱乐
军事
文艺
娱乐
欧美,插画,时尚,星座命理,健康,新闻资讯,美食
微博奇葩
电影控
汽车,军事,体育,娱乐,白羊座,电影,美食
教育就业,美女,新闻资讯,星座命理,文艺
车世界
福建生活
南昌生活
时尚
健康
游戏动漫,IT 数码,美食
投资理财,美食
时尚
文艺
福建教师招聘
钢管舞,肚皮舞教练,热爱运动,生活
体育
娱乐
美食
游戏动漫,IT 数码,手工爱好,伪虹饭,声音控,伪娃娘,WOW,军爷赛高,吃货,睡觉
新闻趣事
星座控,美食,微博奇葩,搞笑幽默,名人明星
游戏动漫,金牛座,游戏,小说,音乐
美女,牺牲奉献,白羊座,看书,兜风
视频音乐,娱乐
哇水仙,初次同人,杨慕初 X 杨慕次,一触即发
星座命理
名人明星,情感生活,校园生活,搞笑幽默,深圳生活

美食爱好者,喜欢听歌,时尚达人,新浪读书,看书,时尚,睡觉,上网,自由,娱乐
欢迎大家关注我,唱歌
健康
美食
星座命理
文艺
旅游,美食,文艺,体育,陈奕迅,桌球,女人,nba,微笑,k 歌
美图摄影
美食
新闻资讯,时尚,汽车,美女,军事,娱乐,体育
美食
美食,娱乐
睡觉睡到自然醒,爱自由,吃喝玩乐,腐,海绵宝宝,爱吃,90 后但我不脑残
体育,NBA,足球,射手座,搞怪,搞笑,运动
学生一族,爱生活,巨蟹座,美食爱好者
新闻趣事,搞笑幽默,福建生活,旅游,美女
紫色,平常心
旅游,新闻资讯,娱乐,教育就业,体育
美食,乒乓球,植物,羽毛球,爱听音乐
视频音乐
矛盾体,喜欢胡歌,喜欢白日做梦
Iamsherlocked
时尚,教育就业,旅游,新闻资讯,文艺,好性格,睡觉,摄影
名人明星
健康,运动,娱乐,篮球,80 后,上网,新闻,画地为牢
任性、执着。
国际物流,日本航线,贸易,幽默,吃货,驴友,户外,生存,2012,急救
时尚,健康
懂得珍惜与感恩,真诚待人,营销策划,创业者,老板,大学生
健康,时尚,星座命理
宅,上网,听歌,音乐,囧,好性格,睡觉,艺术,80 后,自由
美术,服装,睡懒觉,小说,大学生,动漫迷,上网,佛科医学院,龙山中学,吃货
游戏动漫,健康,文艺,投资理财,旅游
新闻资讯,军事
美食
名人明星
美女
旅游
福建生活
星座命理
时尚,健康,新闻资讯
美食,旅游美食
娱乐
情感生活,星座运势,新闻趣事,视频音乐,搞笑幽默

搞笑幽默
旅游,看电影,听音乐
奋斗,旅游美食爱好者,superjunior,开朗,看小说,80 后的尾巴,love,爱旅行,小女人,臭美
星座命理
美食
旅游
教育就业,美食,美女,游戏动漫,娱乐
破蛋臭蛋,冰激凌
娱乐
IT 数码
时尚,旅游,星座命理,文艺,娱乐,偶像剧
健康
旅游
微博奇葩,michael,jackson,我爱睡觉,爱 MJ,喜欢健身,怪人一个
开开心心,开心就好
美图摄影,汽车,旅游,健康,文艺
射手座,校园,90 后,听歌,读书,善良,交友,宅,好性格,学生
美食,IT 数码,娱乐,星座命理,旅游
维尼夫妇,文字控,手机,猫,爱自由,麦霸,唱 k
美食
时尚,娱乐
90 后但我不脑残,搞笑,乐观,自由,学生
娱乐
旅游
健康,体育,娱乐
文艺,体育,娱乐
十堰生活
廉耻心,慎重,美女,义乌,制药,乒乓球,武汉
教育就业
星座命理
娱乐
旅游,星座命理,美食,军事,娱乐
宣传片,微电影,广告人,营销,地产,金牛,网络,策划,电影控,设计师
教育就业,游戏动漫,体育
旅游,美食
射手座,00 后
星座命理,美食,娱乐
星座运势
健康,投资理财,体育,文艺,军事
新闻资讯,时尚,健康,旅游,文艺
军事
时尚,时尚达人
娱乐,都是朋友,囧,善良,音乐,唱歌,90 后,学生,吃,偶喜欢玩
时尚,IT 数码,娱乐,美女,法律,乒乓球,爬山,简单,执着,天蝎

文艺,体育,娱乐
军事,IT 数码,文艺
搞笑幽默
文艺
健康,体育,娱乐
旅游
八卦杂谈
健康,体育,娱乐
呼和浩特,烟台,重庆,济南,江阴
汽车
狮子座,90 后,英语
文艺,体育,娱乐
游戏动漫
投资理财,游戏动漫,健康,旅游,美食
健康,新闻资讯,娱乐
游戏动漫,美女,体育,娱乐
健康,体育,娱乐
星座命理,健康
视频音乐,成都生活,m
IT 数码,美食,军事
时尚
健康,美食,旅游,狮子座
星座命理,用辉煌了却此生
游戏动漫
美食,星座命理,其他生活,穿衣美容,旅游
娱乐
超越年龄心灵,幽默,仅是个人观点
汽车,美食,文艺
游戏控,绘画爱好者,体育篮球,美食,娱乐,体育,睡觉睡到自然醒,高三
湛江,慈善,幻想,房地产
星座命理,美食,IT 数码,文艺,娱乐
时尚
文艺
健康,新闻资讯,时尚,美食,IT 数码
游戏动漫
娱乐
视频音乐,名人明星,美女,體育,娛樂,微博,唱歌,学生,体育,篮球
IT 数码
视频音乐,萌宠,美食,美女,搞笑幽默
英语,时尚
体育
IT 数码,美食,旅游,美女,汽车,体育
新闻资讯,旅游,体育
旅游,新闻趣事,视频音乐,搞笑幽默

健康,娱乐,时尚,星座命理,文艺
汽车
文艺
美女,時尚,汽車,IT 數碼,體育
时尚,美女
名人明星,美女,穿衣美容,旅游,文艺
健康,乐观
IT 数码
名人明星,娱乐
健康,旅游,美食,文艺,娱乐
FUCK,巨蟹座
军事,IT 数码,体育,漫迷,阅读控,自行车爱好者,果粉,资深变态,愤世青年,学生
美食,旅游,文艺,娱乐
文艺,体育,娱乐
新闻资讯
美食,旅游,军事,健康
文艺
天蝎,篮球爱好者,大学生,互粉,单身,游戏,幽默,听音乐,爱唱歌,外貌协会
我爱 497,听歌,睡觉,电影
军事
旅游,发展力,想象力,可爱,园林业,牧业,牺牲奉献
名人明星
健康,新闻资讯,娱乐
电信运营商,通讯行业
健康,旅游,星座命理,文艺
爱钻牛角尖,矛盾体,懒惰
服装,怀旧,创意,音乐爱好者,爱旅行,低调,强迫症,游泳,独立,Music
时尚,文艺,旅游,健康,军事,跑步,弹琴
美女,军事,投资理财,游戏动漫
新闻趣事
时尚
游戏动漫,茶文化原创动画
善良,听歌,电影,囧,看书,金牛座,狮子座,平常心,美食,上网
微博奇葩,美食,旅游,西安生活,新闻趣事
巨蟹座,唱歌,乐观,艺术,交友,游戏,电影,90 后,运动,宅
星座命理
健康,旅游,星座命理,文艺,娱乐
美女,爱情,Android,聊天,90,苹果
恋爱,义乌,细心,市场,牺牲奉献,活泼
各种运动
娱乐,美食,旅游,新闻资讯,游戏动漫
友情基情闺蜜情,聚会趴地,cosplay,重口味,小清新,个性展现,娱乐放松,电影迷,自拍
星座运势
健康,时尚,汽车,美食,旅游,85 后,射手座,自由

旅游,健康
军事
吃喝玩乐,時尚,星座,旅遊,理財
文艺
美食
美食
游戏,音乐,电影,我自己
名人明星
周杰伦,音乐无国界,爱杰永不懈
时尚
财经,囧,动漫,80 后,看书,自由职业,微博,糊涂,愚蠢,傻瓜
洛杉矶旅游,洛杉矶一日游,洛杉矶地接,迪士尼,拉斯维加斯,环球影城,洛杉矶美食,美国旅游
游戏动漫,健康
玩儿,宅男,大学,自由,游戏,篮球,学生,善良,双鱼座,电影
娱乐,處女座,睡觉觉
其他生活
美食,健康,时尚,教育就业,投资理财,星座命理
执着,义乌,公关,骑车,重庆,电影
娱乐
汽车
摇滚,偶稀饭睡觉觉,90 后,山地车爱好者,单反情结,吃货,爱吃冰,monster 怪獸,单身待解救,白羊女一枚
历史,好性格,阅读,生活,美食,自由职业,善良,娱乐,交友,时尚
体育
美食,双鱼座,羽毛球,宅女,音乐,动漫,网购,白羊座,吃
美女,汽车
旅游
成都生活,互联网,校园,新闻资讯,时尚,教育就业,体育,娱乐
阅读,幽默,动漫,音乐
穿衣美容
美食,娱乐,旅游
福州生活,教育就业
新闻资讯,投资理财,星座命理
好性格,女人,烹饪,上网,睡觉,唱歌,护士,健康,旅游,美女
健康,投资理财,汽车,军事,IT 数码
旅游,名人明星,美食,穿衣美容
美食
重口味,心理学,欧美控,娱乐,旅游,时尚
90 后,天秤座,猫控
旅游,娱乐
体育
美女,健康,新闻资讯,投资理财,旅游
权利,会计,摩羯座,运动,乐观,股票,细心

创意,家居,设计风格,装饰,时尚家居生活,装修设计,沙发,灯具,时尚,创意生活
处女座,读书
IT 数码
军事,教育就业,投资理财
旅行,唱歌,吃
搞笑幽默
美食,搞笑幽默,萌宠,视频音乐
投资理财,游戏动漫,旅游,文艺,体育
旅游,军事,投资理财,汽车
投资理财
旅游
娱乐,吃
读书分享
IT 数码,旅游,游戏动漫,喜欢旅游,萝莉控,吃货,喜欢爱情公寓,水瓶座,90 后
娱乐,学生,天蝎座,搞笑
娱乐,旅游,体育
汽车,体育
旅游,星座命理,时尚,娱乐,美食
旅游
健康,美食
投资理财,健康,旅游,文艺,体育
搞笑幽默,广东生活,低调
美食,旅游,健康,新闻资讯,投资理财
时尚,美女,投资理财,文艺,IT 数码
美食
美女
旅游
IT 数码
校园生活,新闻趣事,IT 数码,八卦杂谈,黔西南生活,美食,听歌,金牛座,网购
军事
搞笑幽默,娱乐
网络,自由职业,偶喜欢玩,营销,体育,美女,亲情,80 后
体育
娱乐,吃货一枚,晟斗士,睿士糖
体育,偶稀饭睡觉觉,运动,80 后,摩羯座,吃货
美女,时尚,汽车,娱乐,IT 数码
体育
文艺
可爱,扬州,广州,苹果,乒乓球,运动,湖州
健康
教育就业,娱乐,新闻资讯,健康
军事,新闻资讯,美女,投资理财,汽车
穿衣美容
新闻资讯,星座命理,文艺

旅游,健康,投资理财,美食
上海生活
胡思乱想,宅女,画画,坚强,古典音乐,购物狂,爱睡觉,时装,南京,任性
健康,汽车,旅游,军事,体育
汽车
宠物图集
时尚,汽车,90后,吃货
旅游,娱乐,文艺
时尚,文艺,旅游,美食,星座命理
羽毛球,美剧控,90後,狮子女,电影,学生
旅游,娱乐
体育
体育
美食
宠物控,旅行,电器迷,车迷
美食,旅游
健康,星座命理,军事,文艺,体育
文艺,旅游,美食
历史,校园,学生,好性格,90后,摩羯座,读书,音乐
IT数码,乖小孩,听歌,电影,文学,学生,90后,偶喜欢玩,吃,好书
新闻资讯,星座命理,美食,文艺,娱乐,狮子座
旅游,健康,新闻资讯,美女,游戏动漫,动画
爱睡觉
美食,视频音乐,拉萨生活,微博奇葩,搞笑幽默
体育,健康,旅游,美食,游戏动漫
文艺,IT数码
娱乐
旅游
微博奇葩
美食
巨蟹座,盐城,酒店,骑车,思考,生物,荆州
娱乐
广场恐惧症,外向孤独症,推理悬疑控,八月长安铁粉
旅遊,美食,IT數碼,文藝,娛樂
娱乐
韶关生活,美图摄影,美容服饰,美食
游戏动漫
旅游,时尚,娱乐,IT数码,美食,汽车,体育
汽车,娱乐,投资理财,军事,美女
美图摄影
时尚,新闻资讯,美食,军事,IT数码
娱乐
摄影
娱乐

文艺,娱乐
美女,游戏动漫,美食,文艺,体育
时尚,美食,星座命理,娱乐,健康,美食,投资理财
搞笑幽默,巨蟹座,起床困难户
读书分享
旅游
美女,帅哥,双子座,网络电子商务,白羊座,义乌
美食
《我的公主》,宋承宪,《当男人恋爱时》,时尚,新闻资讯,健康,旅游,美食,学生,羽毛球
美图摄影
汽车,娱乐,体育,IT 数码,军事
体育,美女
旅游,星座命理,美食,娱乐
体育
文艺,军事,娱乐,吃,听歌,音乐,新闻,巨蟹座,美食,抗日青年
美女
貌似双鱼座,猫咪,咖啡控,强迫症,唯蓝,SuperJunior
娱乐,SLG 工作室
体育
文艺,娱乐,汽车,游戏动漫,美女
其他生活,文艺
娱乐
美女
旅游,健康,文艺
美食
健康,体育,娱乐
IT 数码,投资理财
旅游,时尚,美食
美食
文艺
金牛座,善良,听歌
美食,军事
时尚,健康
IT 数码
健康,新闻资讯,文艺
美女
美图摄影
娱乐
IT 数码
新疆生活
健康,旅游
星座命理,搞笑,偶稀饭睡觉觉,宅女,自由
新闻资讯
其他生活

萌宠
搞笑,善良,80 后,学生,睡觉,校园,交友,浪漫,动漫,金牛座
我就是我,欧广源,绘画
视频音乐,搞笑幽默
美食,游戏动漫
文艺,英国代购,混血儿,英国,妈妈宝宝用品,奢侈品,海外购,DIOR,Celine,YSL
娱乐,星座命理,旅游,健康,美食
娱乐,金牛座
美食,美女,游戏动漫,体育,军事
喝茶,发呆
情感生活,半懵半懂半成熟
IT 数码
浙江生活
湖北生活
游戏动漫
魔羯座,黑莓,80 后,篮球,政治,经济,大叔,旅游
星座运势
心理健康,心理咨询与治疗,健康
美女,投资理财
高技能骑士大人,傲娇公主殿下,小豆汤与大板车,板车夫妇,19CM 身高逆差萌,鹰之眼,秀德高校篮球部,緑間真太郎,高尾和成,高绿
旅游,投资理财,文艺,美食
搞笑幽默,视频音乐
体育
体育资讯
IT 数码,娱乐
时尚
美食,汽车,健康,旅游,IT 数码
教育就业
其他生活,视频音乐,美食,旅游
美食
娱乐
时尚,健康,娱乐,美女,美食
警察,机械工程师,科技,爱旅行,北京,足球迷,德国,汽车,IT 数码
美食,减肥,画画,游泳,旅游摄影,旅游美食,深圳,水瓶女,烹饪,数码
河北生活
美食,教育就业,健康,旅游,文艺
宝贝儿,成长
文艺
胡思乱想,安静,妖精,低调,爱电影,爱旅游,美食,摄影
游戏动漫
新闻资讯,军事,美食,体育
教育就业,美食,旅游,IT 数码,健康,汽车,时尚,体育,娱乐
体育,美食,IT 数码,投资理财,健康

情感生活,育儿百科,八卦杂谈,新闻趣事
体育资讯,傻傻分不清楚,电子商务,文学,唱歌,自由,90 后,电影
文字,善良,艺术,睡觉,上网,自由,90 后,笔亲
汽车,IT 数码,娱乐
旅游,宅男,浪漫,睡觉,电影,吃货,80 后
文艺
篮球
军事迷,车迷,电器迷,IT 人
旅游,汽车,美食,文艺,体育
旅游,穿衣美容
爱睡觉,星座命理,美食,旅游
健康,新闻资讯,投资理财,美食,旅游
微博奇葩,视频音乐,搞笑幽默,南充生活
体育,旅游,投资理财,汽车,美食
汽车,军事,健康,旅游,新闻资讯,独立,我就是我,平常心,偏执狂,爱自由
文艺,金牛座,自由,宅,学生,足球,听歌,游戏,上网,旅游
汽车,时尚,美女,美食,IT 数码,宅男,微博
星座命理
军事,美食
游戏动漫,旅游,军事,娱乐,郭恒,魔兽世界,LOL,邯郸,吉他社,吉他
时尚,音乐
星座命理,文艺,美食
睡觉…
看书,历史爱好者,足球迷,篮球爱好者,CR7 崇拜者,曼联死忠,NBA
我就是我,爱美食,书,动漫
美食,娱乐
搞笑幽默,其他生活,微博奇葩,新闻趣事
随性,读书,巧克力,爬山,心理学,music,爱旅行,我就是我,活泼开朗,狮子座
汽车
搞笑幽默
教师,培训,爱自由,口语学习,翻译,IT 互联网,英语,软件开发,体育,教育就业
星座命理
名人明星,体育,奋斗 ing,体育迷,网虫,微博控
星座命理,美食,时尚,健康,文艺
时尚,汽车,旅游,娱乐,美女
校园生活
教育就业
等退休
娱乐,文艺,星座命理,健康,纠结,完美主义,电子商务,狮子座
更爱妈妈,爱旅行,爱篮球,单纯双子男
新闻资讯,文艺,旅游
山水间结庐,一卷书,一杯茶,微凉
文艺,白羊座,艺术,动漫,篮球,幽默,音乐,设计
文艺,健康,星座命理,美食,娱乐

旅游
美食,八卦杂谈
微博奇葩,搞笑幽默
美食,旅游,穿衣美容,男人,皇马,摩羯座,生活,90后,平常心
游戏动漫,音樂
投资理财
游戏动漫,狮子座90后,吃货
投资理财,时尚,娱乐,营销,游戏,双子座,文字,90后,摄影,吃货
娱乐
名人明星,穿衣美容,视频音乐,新闻趣事
微博奇葩,IT数码
美女
娱乐,处女座,80后
广东生活
文艺
星座命理,体育,娱乐,投资理财,IT数码
时尚,旅游,美食,文艺,娱乐
体育
娱乐,教育就业,体育,健康,新闻资讯
美食
IT数码
吃货,乐观,吃,坚强,旅游
建筑,执着,渔业,清远,深圳
时尚,汽车
健康
美食
时尚,新闻资讯,健康,旅游,美食
投资理财,旅游
旅游,自由,阳光,单身
美食,星座命理,时尚,体育,娱乐
旅游,军事,IT数码,体育
汽车
游戏动漫,时尚
电影MV,H族,不断走走的旅粉,懒人一枚,学习学习再学习,尚秀,瑜珈
睡觉,上网,80后,音乐,宅女,创业
微博奇葩,视频音乐,新闻趣事,石家庄生活,旅游
IT数码,校园生活,视频音乐,八卦杂谈,搞笑幽默
星座命理
体育
视频音乐
时尚,文艺,美食,娱乐,宅,双鱼座
双鱼座
时尚,新闻资讯,健康,旅游,美食
时尚,健康

穿衣美容
体育
旅行,吃货,体育迷,电影迷
新闻资讯,旅游,汽车
时尚,文艺
健康,我恨我爱你,好姑娘永垂不朽,PISCES
健康,时尚,美食
射手座,80 后,吃货,自由,羽毛球
文艺,体育
娱乐
美食,美食
汽车,新闻资讯
星座命理
A 型血,大学生,财经,思考,乐观,90,爱音乐,运动,诗词,微笑
文艺
星座命理,游戏动漫
投资理财,美女,钓鱼、汽车
美食
河北生活,微博奇葩,萌宠,旅游,搞笑幽默
小清新,各种宅,音乐,狮子座
搞笑幽默
吃货,吃,巨蟹座
体育
穿衣美容,美食,偶稀饭睡觉觉,偶喜欢玩,吃,90 后,听歌,处女座
美食
游戏动漫
美女,体育,娱乐,时尚,爱情
文艺,娱乐,体育,旅游,美食
fashion,健康,星座命理,娱乐
体育资讯,新闻趣事,重庆生活,电影,体育,读书,旅游,历史
军事,汽车,美女,旅游,娱乐
娱乐
美食,星座命理,娱乐,善良,阅读,90 后,狮子座,英语,学生
microsoft,android,java,电脑,软件迷,艺术绘画,IT 技术
新闻资讯,汽车,旅游,军事,IT 数码
投资理财,旅游,文艺,体育,娱乐,暨南大学
旅游,文艺,时尚,健康,投资理财
巨蟹座
美女
娱乐
上海生活,NBA,看书,动漫
娱乐
时尚
旅游,深圳生活,搞笑幽默,美食,美女

IT 数码
微博奇葩
新闻资讯
美食,旅游,广东生活
體育
狮子座,拍照,听歌
娱乐
原创,阳光,五月天,爱睡觉,玉米,小记者
学生一枚,完美主义,军事天地,新闻资讯,游戏动漫,汽车,投资理财
旅游,旅游美食爱好者
时尚
娱乐
爱美食,爱吃,主持
时尚,娱乐
美女,教育就业,旅游,军事,体育
萌宠,摄影控
美女,健康,军事,IT 数码,文艺
文艺,旅游,美食,健康
文艺
体育
新闻资讯,汽车,美女
笑话控,体育迷,游戏达人,电影迷
新闻资讯
旅游
娱乐
时尚
时尚,娱乐
IT 數碼,汽車,美食,體育
时尚,教育就业,投资理财,娱乐,IT 数码
摩羯座,自由职业,音乐,善良,旅游,电影,语录,新闻,微博
投资理财,健康,娱乐,时尚,旅游
天蝎座,80 后,动漫
宅女一枚,shopping,旅游
旅游,星座命理
动漫,自由,篮球,台球,奋斗 ing,金牛座,90 后
新闻资讯,旅游,教育就业,投资理财,军事
穿衣美容
车迷,房产专家,军事迷,愤青
水瓶,搞笑幽默,游戏动漫
平常心
健康,美食
军事
星座命理
旅游

健康,星座命理,美食
旅游,美甲师,化妆师,妆面造型,化妆
新闻资讯,美食,军事,娱乐,体育
星座命理,文艺
健康
爱吃
旅游,天蝎座,好性格,乐观,90 后
魔兽世界,渣手绘,遊戯王,游戏动漫
游戏动漫,军事
美女
时尚,白羊座
星座命理,健康,美食
搞笑幽默
旅游,枣庄生活,搞笑幽默
时尚,投资理财,新闻资讯,美女,星座命理,美食,教育就业,健康
美女
视频音乐,洛阳生活,星座运势,校园生活,music
体育资讯,体育
体育
穿衣美容
娱乐,投资理财,时尚,美女,IT 数码
愤青,音乐控,体育迷
健康,体育,新闻资讯,汽车,文艺,娱乐,IT 数码
投资理财
宫锁沉香,宫电影版,娱乐
IT 数码,体育
军事,新闻资讯
时尚,健康,新闻资讯,星座命理,教育就业,文艺,投资理财,旅游,IT 数码
名人明星
妖精
旅游
娱乐,IT 数码
旅游,美女,健康,汽车,时尚,娱乐,体育
旅游,文艺,娱乐,体育
电影,海外生活,游戏动漫,幽默,海贼迷,天秤
文艺,看书,双鱼座,radioman,历史,幻想族
旅游
文艺
历史,80 后,动漫,旅行,善良,双子座,幽默,时尚
时尚,汽车,美食,娱乐
美食
IT 数码
旅游,娱乐,新闻资讯,教育就业,健康,社工,海布,NGO
广东生活,汽车

穿衣美容
IT 数码,文艺
福建生活,视频音乐,旅游,创业,汽车,平常心,听歌,时尚
体育
济宁,主观,湛江,旅游,硬件,勇敢,农业
体育,健身,看电影,时尚,NBA,爱旅行
旅游,美食,娱乐,星座命理
星座命理
80 後,小清新,话唠,有才,善解人意,时尚,新闻资讯,健康,旅游,IT 数码
体育,娱乐
游戏动漫
星座命理,旅游,教育就业,娱乐
IT 数码
旅游
娱乐
美食
美食,旅游,其他生活,视频音乐,名人明星
IT 数码,新闻资讯,星座命理,娱乐,体育
时尚,旅游,文艺,娱乐,教育就业,海绵宝宝,分裂,水瓶座
时尚
互粉,运动
运动和旅游,美食,电影
娱乐
体育
旅游
体育,娱乐
成都,唯美的,盐城,室内设计,杭州,济宁,会计
美女
IT 数码,美食,星座命理,时尚,文艺
汽车
美女
旅游,美食,文艺,娱乐,乐天派
娱乐
娱乐
美食
Towiko 组新人,学生一枚,偶尔的 coser,渣渣新人 mader,新人 mad 制作者,B 站新人 UP
时尚,娱乐
橙子,vip,健康,文艺,美食,旅游,娱乐
教育就业,时尚,健康,文艺,娱乐
搞笑幽默,睡懒觉,创意
游戏动漫,汽车,投资理财,体育,娱乐
美女,新闻资讯,IT 数码,文艺,娱乐,五月天
爱吃,健康,新闻资讯,投资理财,美食
教育就业,投资理财,新闻资讯,健康,旅游

时尚,健康,旅游,美食,娱乐
梦想,广告人,工作狂,传媒,广告,时尚,新闻资讯,美食,军事,文艺
无锡生活
汽车,美女
汽车
美甲 DIY,美甲师,美甲控
IT 数码
IT 数码
搞笑幽默
看电影,偶稀饭睡觉觉,巨蟹座,爱生活,游戏动漫
IT 数码,军事
美容服饰,宜宾生活
旅游,文艺,IT 数码,娱乐
萌宠
选择障碍,人群恐慌
健康,吃喝玩乐,娱乐
咖啡,文学,时装
时尚,文艺,旅游,健康,教育就业,写作,公文,批改,申论,公务员考试
吃貨
新闻趣事,视频音乐,搞笑幽默,微博奇葩,美女
旅游,投资理财,文艺,娱乐,90 后但我不脑残,个性无法拷贝,k 歌,国外旅游,摄影,AB 型
旅游,美食,娱乐,文艺
美食,时尚
读书分享,动漫,搞笑幽默
电视剧,旅游,阳光,体育,文艺
汽车
旅游
投资理财,星座命理,美食,体育,娱乐
星座命理,美食,双重性格,吃货,疯子,强迫症,巨蟹,单身,神经质,90 後
品牌,心理学,市场营销,商业,电子商务,营销,上海,时尚,旅行,美食
投资理财
娱乐
时尚,娱乐
旅游
文艺,IT 数码,汽车,美女,娱乐
美女,体育,喜欢听歌,吃喝玩乐睡,胡歌,湖人,拍照,篮球爱好者
星座命理
体育
瘦,身材,减肥药,瘦身,减肥,美女
福州,福州生活,福州媒体,福州校园,福州爆料,福州美食,新浪福建,福建身边事,福州同城会,
福州民生
名人明星
重口味,美食,听音乐,爱音乐
健康,旅游,投资理财,汽车,游戏动漫,插画,画画,摄影,动画

新闻趣事
时尚,游戏动漫,星座命理
美女
低调,O 型血,90 后
新闻资讯,美食
投资理财,星座命理,语录
旅游,健康,教育就业
美女,汽车,IT 数码
微博奇葩,旅游,视频音乐,搞笑幽默,穿衣美容
游戏动漫
美食
旅游,新闻资讯,时尚,IT 数码,汽车
娱乐
美女,紫色,艺术,水瓶座,音乐
感性,简单,西班牙,向日葵
文艺
文艺
我爱篮球,教育就业,美食,文艺,体育,我爱玩
旅游,漫画美男控,腐女宅女耽美狼,腐女,宅女
投资理财,旅游,教育就业,健康,IT 数码,美食
美食,IT 数码,汽车,投资理财,娱乐
时尚,投资理财,体育,文艺
美女
旅游
娱乐
教育就业,体育,IT 数码,健康
时尚
娱乐
旅游,随性,大学
新闻资讯
美食,军事,IT 数码,汽车,文艺,体育
游戏动漫,偶喜欢玩,学生一枚,阳光,听歌
文艺,逛街爱时尚,抒情摇滚歌曲,爵士舞
名人明星
时尚,美食,娱乐,体育
摄影爱好者,旅游,财经,股票,狮子座
旅游,穿衣美容
健康,新闻资讯,教育就业,投资理财,军事
美女
旅游,健康,美女,文艺,军事
旅游
娱乐
旅游
娱乐

投资理财,教育就业,文艺,美食,娱乐,90后
娱乐
星座命理
健康
文藝
娱乐,文艺,健康,新闻资讯,爱家人,爱钟汉良
视频音乐
军事,新闻资讯,娱乐,汽车,投资理财
睡觉睡到不想起,宅
娱乐,投资理财,时尚,健康,星座命理
搞笑幽默
时尚,旅游,娱乐
健康,娱乐,IT数码
星座命理,娱乐
军事,投资理财,旅游,汽车
IT数码,旅游,美食,游戏人生
体育,时尚,健康,美食,旅游
军事,娱乐
足球迷,记者,运动,电脑,心理学,开心,传媒,看书,策划,计算机
汽车,投资理财,新闻资讯,军事
八卦杂谈
汽车
商业模式创新,资源整合,电子商务网,络赚钱机器,网络推广实战,营销型网站,品牌定位,微博营销,直复式营销,网络营销
普罗旺斯薰衣草,多愁善感,善良,水瓶座,活泼,幽默,逛街,爱唱歌
美女
娱乐,美女,汽车,IT数码,旅游
IT数码,时尚,文艺,健康,美食
教育就业
IT数码,文艺,体育,娱乐,音乐,80后,唱歌,篮球,运动,乐观
健康
文艺
娱乐,美食,IT数码,游戏动漫
美食
文艺,酷爱双节棍,天秤座
个性,旅游摄影
体育
娱乐
Ruby,RubyOnRails,IT民工,软件工程师,加利福尼亚,美国,加拿大,温哥华
星座命理
娱乐,文艺,时尚,唱歌，跳舞，活泼
校园生活
新闻趣事
美食

爱我别想走,美女,娱乐
穿衣美容,旅游,新闻趣事
教育就业,懒人
文艺,娱乐,IT 数码
健康,旅游,美食,体育
旅游
我是祛痘专家
汽车,健康,文艺,娱乐,体育
星座命理
百姓生活,美食控,美食,食品安全,健康品质,天津风味,包子,烟台,灌汤包,健康饮食
張芸京,周杰倫,古典音乐,哲学
运动,廉耻心,卫生服务,北京,合肥
单身,活泼,爱生活,阳光,旅行
星座运势,爱墨镜到爆,王者傲气,不受约束,爱时尚,爱音乐,专一,阳光,创意,美食
星座命理,娱乐,投资理财,健康,旅游,美食
名人明星,搞笑幽默,美食,新闻趣事,穿衣美容
美女,旅游,IT 数码
微博奇葩
星座命理
美女,游戏动漫,IT 数码,体育,娱乐
微博奇葩
投资理财
家居,柯南,美食
湛江,哈尔滨,济宁,宜昌,慎重
军事,美女,美食,IT 数码,体育,游戏动漫
爱幻想
TMT,天使投资,户外,自驾游,创业,教育,消费,移动互联,互联网
搞笑幽默
旅游,汽车,美食
星座命理,健康,美女,军事,体育
美食
视频音乐,教育就业,美女,IT 数码,纫
旅游,美女,时尚,美食控,健身,健康,汽车,投资理财,美食
时尚,旅游,娱乐,军事,美食
旅游,娱乐
教育就业,星座命理,娱乐
文艺
健康,美女,IT 数码,学生,ab 型,爱情,思考,郁闷。篮球
肇庆生活
旅游
福建生活,睡觉睡到自然醒,摩羯,欧美音乐,我就是我,强迫症,感性,喜欢音乐,愤青,健康
旅游,游泳，旅游
旅游
美女,汽车,旅游,军事,IT 数码

朋克,包包控,悦己,悦读,时尚,宅,文艺,复古,旅行,音乐
新闻资讯,游戏动漫,时尚,文艺,娱乐
时尚达人,热爱生活,美食爱好者,喜欢听歌,经典语录收集,广州,校园,情感
旅游,娱乐,美食,汽车
时尚,射手座,90 后
吃喝疯乐购,美食,时尚,旅行,听歌,健康,旅游
创业投资,新闻资讯,音乐迷,互联网,时尚,大学生,IT 互联网,中关村动态,电子商务,创业
体育,投资理财,文艺,IT 数码,军事,美食
旅游
幻想,建筑,江门,保健,硬件,卫生服务
娱乐
美女,餐饮业
旅游,美食,视频音乐,搞笑幽默,北京生活
爱笑,我喜欢上网聊天
美食,文艺,美女
游戏动漫,旅游,美食,文艺,娱乐,无厘头,吃货
吹水
新娘跟妆,无敌超爱化妆,婚纱造型馆,造型设计师,婚纱,美女,吃货,桌球
旅游
旅游
新闻趣事,穿衣美容,名人明星,搞笑幽默
游戏动漫,美食,文艺
旅游
数码,传媒,网络
IT 数码,文艺,军事,体育,娱乐
娱乐
寂寞,足球,睡觉睡到自然醒,看小说,低调,怀旧,宅男,手机,A 型血,执着
旅游,健康,美食,娱乐
梅格瑞恩,尼古拉斯凯奇,张曼玉,水瓶座,旅行,电影,时尚,摄影
美食,文艺,白羊
爱旅行,旅游
敏感
旅游
自由,旅行
时尚,旅游,健康,美食,游戏动漫
旅游美食,动画,青春,学生一枚
游戏动漫
IT 数码
时尚,美食,金牛座,90 后,宅
其他生活,体育资讯
亲情,聊天,夜猫子,偶稀饭睡觉觉,爱玩,执着,听音乐,90 後,许嵩,蓝色
健康,文艺,体育,娱乐,开朗,星座,看电影,发呆,爱音乐
育儿百科
我是天蝎座女孩,我是 90 后女生,玩儿,乐观,羽毛球,学生,浪漫,90 后,网络,听歌

旅游,美食,汽车,投资理财,娱乐
游戏动漫
农业,休闲,牧业,成都,幻想,兜风,公关,搞笑
娱乐,IT 数码,游戏动漫,健康,体育
新闻趣事
执着,宜昌,唯美的,常熟,艺术,清远,律师
双鱼座,互粉,写作,咨询,执着,绵阳,农业,济宁
游戏动漫,教育就业,旅游,军事,体育
娱乐
视频音乐
魔兽,旅游美食爱好者,打球,IT 互联网,80 后的尾巴,IT 数码,游戏动漫,体育,娱乐
美食,旅游,大方热情,喜欢旅游,爱笑的女孩,90 后,傻傻的,单纯
体育,美食
法律,自尊心,白羊座,杭州,生物,常熟,狮子座,思考
健康
时尚,旅游,美食,娱乐,健康
健康,星座命理,娱乐
开朗,活泼快乐,看电视,看书,会计
美食,睡觉睡到自然醒,唱歌,学生,搞笑,狮子女,猫咪,做梦,音樂,巧克力
娱乐
美食,旅游
无所谓,笑点低,看电影
新闻资讯,美女,游戏动漫,时尚,汽车
星座命理,娱乐
体育,美食,游戏动漫,美女,汽车
射手,猫咪控,大叔控及控大叔,大叔控,时尚,游戏动漫,美食,90 后又怎么了,我不是同性恋,
吃喝玩乐宅腐呆
时尚,娱乐,美女,美食,90 后
娱乐
宠物图集,情感生活,星座运势,唐山生活,美食,夜猫子,古灵精怪,唱 K,八卦,囧
健康,游戏动漫,旅游,美食,IT 数码
健康
湖人,爱执信,粤语,阿仙奴死忠,懒,羽毛球
运动,旅游,微博,偶稀饭睡觉觉,交友,时尚,双子座,娱乐,90 后,乐观
健康,投资理财,体育
回音哥,旅游
娱乐,旅游
搞笑幽默
新闻资讯,文艺,汽车
星座命理
IT 数码,汽车,体育,娱乐
射手座,疯狂,睡懒觉,爬山,美食,旅游,健康
90,投资理财
娱乐,奶油小生

海绵宝宝,英语,90後,教育就业,娱乐,美食,健康,旅游,厦门,夜猫子
汽车
美女,发展力,室女座,想象力,公关,股票,成都
旅游,体育,娱乐
旅游,星座命理,美食,娱乐,体育,摄影,宅,搞笑,萌
搞笑幽默
体育
时尚,文艺
名人明星
旅游,美食
文艺,80后
美食,闷骚,无聊,巨蟹,懒猪,爱吃,宅女一枚
游戏动漫,文艺
自由,亲情,吃货,90后,动漫,处女座,宅女,八卦,偶喜欢玩,听歌
80后,90后,宅女,户外,宅男,柯南,游戏,恐怖,侦探,密室
娱乐,新闻资讯,星座命理,游戏动漫,美食
娱乐,IT数码
美图摄影,视频音乐,名人明星,读书分享,旅游
旅游
不水
旅游,视频音乐,科幻,心理,星星,腐女,爱音乐,金牛,游戏动漫
旅游
美食,娱乐
文艺
美女,投资理财,军事,IT数码,体育
娱乐
电影,营销,音乐,幽默,上网,80后,听歌
爱睡觉,阳光
体育
星座命理
美食,旅游,吃货,高三,水瓶座,BIGBANG,听音乐,我就是我,卡卡,justinbieber
澳门回来,关门最晚的甜品,80后,彩公鸡,蛋糕,奶茶,港式甜品
教育就业,投资理财,新闻资讯,娱乐,健康
名人明星,新闻趣事,旅游,搞笑幽默,萌宠
美女,IT数码,旅游,汽车,体育
美食
美女
娱乐,美女,宅女一个,三分钟热度,90后,简简单单
时尚,旅游,星座命理,美食,文艺
娱乐
娱乐
文艺,美食,旅游,时尚,娱乐
吃货
美女,体育

五月天,我就是我,跑步,旅游,教育就业,体育
娱乐
美食
市场,渔业,乐观,肇庆,生物,发展力,帅哥
旅游
旅游
健康,时尚,旅游,美食,娱乐
旅游,娱乐,美食
美食
90 后,学生一枚,爱玩,学生一族
娱乐,健康
娱乐
莆田生活
新闻资讯,教育就业,旅游,文艺,娱乐
娱乐,起丝,企鹅,张起政粉丝会
搞笑幽默
天秤座,审计,主观,装饰装潢,哈尔滨,酒店,运动
思考者,思考,美景丰收,完美主义,沉默,午饭,执着,不拒绝音乐,不拒绝美食,偶喜欢自由
廉耻心,哈尔滨,权利,财会,恋爱,细心,农业,济南
星座命理
旅游
IT 数码
蘑菇,幸子
美食
汽车,健康,旅游,星座命理,IT 数码
江门,理性,硬件,恋爱,勇敢,自尊心
星座命理,美食,IT 数码,娱乐,时尚,游戏动漫
娱乐,美女
东方神起
旅游,体育
星座命理
美食,游戏动漫,美女,娱乐,吃货
微博,网络,好性格,读书,文学,创业,设计,唱歌,睡觉,自由
IT 数码,旅游
江门,天秤座,餐饮,广告,北京
视频音乐,美食,重庆生活,体育
世博志愿者,花痴,90 后但我不脑残,狮子座,仙后
文艺,体育
新闻资讯,文艺,投资理财,体育,健康,旅游
乒乓球,搞笑,成都,发展力,广州,篮球,旅游,金牛座
时尚,娱乐
巨蟹座,乐观,淄博,牧业,电影,可爱,江门,乒乓球
美食,汽车,旅游
家具,宅女,篮球,宜昌,发展力,双子座

美食,邱雁是我大爷,吃货,90后,旅行,大学,双鱼座
吐槽,宝石,热缩片,手工,动漫,宅,美食,文艺,IT数码
校园生活,军事
旅游,教育就业,美食,投资理财,游戏动漫
旅游,美食,微博奇葩,穿衣美容,遵义生活,音乐,时尚
文艺
美食
健康,教育就业,旅游,星座命理,美食
新闻趣事,名人明星
星座命理,娱乐,汽车,美食,篮球爱好者
IT数码
搞笑幽默,新闻趣事,学生,水瓶女,搞笑,旅行,校园,90后
美食
文艺,旅游,健康
新闻资讯,健康,游戏动漫,时尚,汽车,体育,娱乐
娱乐
陈奕迅,奋斗,美剧,阅读,驴友,喜欢听歌,时尚达人,古典艺术,文艺
睡觉,双重性格,英雄联盟,双子女
在路上
健康,星座命理,美食,娱乐,旅游
旅游,时尚
静想一乐
视频音乐,美容服饰,宠物图集,旅游,美食
投资理财,时尚,美食,星座命理,健康
善良,巨蟹座,偶稀饭睡觉觉,双鱼座,星座命理
IT数码,休闲,喜欢运动,N724,设计师,纽约中文网
唱K,互联网,玩儿,搞笑,吃,游戏,吃货,我就是我,爱音乐,娱乐
美食
时尚
IT数码
天蝎女,星座控,散步,健康,旅游
新闻资讯,love
美食
娱乐,文艺,旅游
时尚,旅游
投资理财,星座命理,旅游,健康,文艺
旅游
旅行,美食,双子座
搞笑,80后,唱歌,化妆师造型师,囧,幽默,音乐,摄影爱好者,平常心,巨蟹座
情若能自控
视频音乐,娱乐,J迷,龙妹,90,林2
文艺,健康,旅游,时尚,娱乐
常熟,江阴,济宁,烟台,想象力
视频音乐

娱乐,体育,旅游,时尚,健康,星座控,好性格,K歌达人,射手座,80后
时尚,旅游,美食
陈翔
时尚,汽车
明基模力女孩,文艺
投资理财,汽车,旅游,文艺,体育
新闻资讯,教育就业,IT数码,体育,娱乐
微博奇葩
健康,旅游,美食,娱乐
视频音乐,美女,美食,浙江生活
投资理财,美女,时尚
体育,美女,旅游,军事,IT数码
游戏动漫,星座命理
新闻趣事
IT,90后,旅行,游戏,电影
体育
美女,体育,IT数码,中医
娱乐
宅男,校园,唱歌,听歌
旅游,军事,文艺,体育,好书,历史,文字
杭州,摩羯座,写作,休闲,建筑,反应佳,肇庆,会计
美女,健康
美女
双鱼座,幻想,海口,财会
时尚
浪漫
星座命理,文艺
美图摄影,体育资讯,IT数码,美女,商丘生活,教育就业,游戏动漫,军事,体育
星座命理,健康,文艺,娱乐
美食,萌宠,微博奇葩,星座命理,名人明星
视频音乐,新闻趣事,搞笑幽默
时尚
名人明星
旅游,健康,美食,汽车,体育
幽默,搞笑,旅游,摄影,生活,娱乐,美食,新闻,石柱,重庆
江阴,想象力,建材,恋爱,唯美的
游戏动漫
美女,游戏动漫,美食,文艺,投资理财
旅游
萌宠
星座命理,游戏动漫,健康
教育就业,文艺,新闻资讯,体育
广州生活
广东生活

宅男,房地产,广告,恋爱,北京
好性格,历史,数码,微博,80后
IT数码,娱乐,汽车
文艺
牡羊座,藝術,外國人
水利,济宁,双鱼座,兜风,思考,财会,海口
美食,时尚
懒觓,闷骚,F1,gay
旅游,快乐,安静,喜欢听歌,烹饪,吃货一枚,摄影爱好者,狮子座
时尚
疯狂,人生,房产,新闻资讯
毅力,合肥,忠诚心,休闲,公关,写作,湖州
教育就业,旅游,娱乐,健康,美食
投资理财
美女
交友,吃,乐观好心态,自由职业,射手座
娱乐
美女,名人明星,微博奇葩,星座命理,搞笑幽默
IT数码,体育,军事,汽车,美女
娱乐
娱乐,旅游
爱生活,好性格,读书,文字控,健康,文艺,美食
娱乐,军事,投资理财,汽车,旅游,IT数码
体育
IT数码
萌宠
新闻资讯,旅游,文艺,美食,娱乐,SupplyChain,利物浦大学,供应链管理
美食,吃货、睡货
蓝色,正太控,小说,电影,听歌,宅女,完美主义者,睡觉,吃货,动漫
体育,IT数码,烟酒生,篮球
美女,美食
90后,西安控,星座控,AB型血,金牛座
星座命理,投资理财,健康,娱乐,亲情,旅行,好性格,电影
听歌,90后,射手座
美食
文艺,娱乐,时尚
体育
新闻资讯,疯子
健康,美食,旅游
文艺,希,二次元君,美图控,2B青年欢乐多,书籍
美食
文艺,健康,旅游,美食
NBA球迷,将乐四中,将乐一中,吃货,体育
健康,新闻资讯,旅游

时尚
星座命理
旅游,新闻资讯,教育就业
美食,神经大条,个性,喜欢唱歌,科比,无厘头,双重性格,吃货
投资理财,时尚,美女
美女
新闻资讯,爱美食,老歌控,懒虫,麦霸
轮滑爱好者,90 後,体育,投资理财
篮球,nba,篮球爱好者,湖人,投资理财,体育,90 后非脑残
wower,魔兽世界
健康,星座命理,美食,文艺,娱乐
80 后的尾巴
穿衣美容
perfect
狮子座,自由,旅游,偶稀饭睡觉觉,网购,唱歌,宅女,时尚,偶喜欢玩,吃货
穿衣美容
新闻趣事,娱乐
热爱乒乓球
电影
文艺,游戏动漫
搞笑幽默,微博奇葩,视频音乐,星座命理,旅游
幽默,唱歌,睡觉
文藝
时尚,健康,旅游,美食,娱乐
文艺,美女,时尚
新闻趣事,搞笑幽默,邢台生活,美食,投资理财,娱乐,新闻资讯,教育就业
健康,旅游,美食,投资理财,娱乐
活泼,快乐,健身
新闻资讯,旅游,娱乐,投资理财
文艺,旅游,体育,星座命理
外貌协会,时尚,健康,汽车,旅游,IT 数码
美食
文艺,寂寞党
陈绮贞,爱蔡健雅,爱王若琳,Adele,钟爱小丸子
美食,娱乐
自由,动漫,艺术,旅行
娱乐
旅游
名人明星
美食,汽车,健康
唱歌,听歌,00 后,玩儿,美食,娱乐,双鱼座,学生,宅
IT 数码
新闻资讯
星座命理

新闻,娱乐圈,静雯世家,成功励志,减肥,美容护肤,爱情箴言,哲理感悟,星座爱好者,互粉
美食
欧美控,交友,搞笑,AV 女优,仓老师,苍井空,小子玛利亚,美剧,睡觉,双子座
军事,美食,旅游
宠物图集
娱乐
汽车,投资理财,美食,IT 数码,娱乐
娱乐
坚强,娱乐
LOL,宅,美食,游戏动漫,画画,团子,夏洛克,动漫
审计,宅男,建筑,装饰装潢,帅哥,主观,自尊心
萌宠
军事
星座命理,90 后
火影海贼一家亲,搞笑幽默
90 后,宅男,射手座,音乐,善良,学生,玩儿,动漫,幽默,吃
文艺
旅游
文艺,行走。
2,乐观,活泼,可爱,天然呆,天平座
美食,新闻资讯,旅游,军事,文艺,娱乐,双子女,喜欢听歌,学生一枚,我就是我
夜猫子,腐女,指环王,林宥嘉,古川雄辉,美剧控,文艺,Eason 陳奕迅
单纯,90,阳光
军事,IT 数码,文艺,体育,娱乐
星座命理,微博奇葩,名人明星,搞笑幽默,广州生活
IT 数码
90 后
90 后,好性格,听歌
乐观,宅男
忻玲
投资理财
体育
爱旅游,养生,笑话,爱电影,宠物,心理学,低调
大学,篮球,自由,音乐
投资理财
新闻趣事,江苏生活,搞笑幽默,视频音乐,美食,新闻资讯
美女,娱乐
游戏动漫,旅游,娱乐,时尚,美女,军事
旅游,健康
体育,美女,新闻资讯,IT 数码,军事
旅游,90 后,巨蟹女
拉丁舞,古筝,学生,巨蟹座,乐观,90 后,校园
搞笑幽默
美食

外貌协会,青春,乐观,吃货,美食
旅游,教育就业,投资理财,文艺,娱乐
美食,文艺
娱乐,交友,幽默,自由,微博,听歌,偶喜欢玩,吃货,电影
娱乐,美食,美女
蓝色,音乐旅游电影,读书和思考,简单风趣,理想与生活,创业,90後
时尚
健康,情绪化,投资理财
投资理财,时尚,健康,体育,娱乐
星座命理,美食,小说迷,喜欢听歌,跆拳道,无厘头,疯狂,90后但我不脑残
文艺
新闻资讯
旅游
IT数码,文艺,美食,星座命理,游戏动漫
嘉兴,装饰装潢,理性
热爱生活,美食爱好者,呆
吃啊吃,睡觉,爱世勋,exo
娱乐,双子座
旅游,星座命理,处女座,音乐,小说,偶稀饭睡觉觉,宅女,90后,学生
伪音乐人
娱乐
山东生活
青春,fashion,爱自由,新歌,娱乐
娱乐
美食,娱乐,天秤座,音乐,90后,听歌,吃货,宅,睡觉,旅行
动漫
want,to,find,you,shouted,name
视频音乐
旅行,听歌,偶稀饭睡觉觉,平常心,网购,囧,爱情,羽毛球,射手座,80后
旅游,美食
娱乐,体育,IT数码
军事,安静
星座命理,健康
名人明星
游戏动漫,囧,神经大条,水瓶男,95后,学生一枚,幻想,爱玩
穿衣美容
娱乐,宝马X3车友,陆龟控,陆龟,80后
新闻资讯,娱乐,美食,时尚,美女
游戏动漫,玉米玉米玉米,
娱乐,语录收集,大学生,听歌,摩羯座O型,音乐,宅
新闻资讯,美食,星座命理,旅游,娱乐
美食
新闻资讯,美女,投资理财,IT数码,军事,语录
杭州生活

巨蟹座,摄影爱好者,动漫,90 后,宅女,历史,生活,文学,设计
娱乐,文艺,处女座,文学,减肥,睡觉睡到自然醒,完美主义,美食,购物,星座
体育
美食爱好者,电影控,TVB,自由,阅读,网络,睡觉,宅女,学生,小说
豪爽,咪电影,咪星座,安静的疯子,小说,90 后
IT 数码,汽车,教育就业,投资理财,军事
娱乐
新闻资讯,娱乐,吃,唱歌,巨蟹座,90 后
文艺,画画,设计,零食依赖症,爱 cosplay,宅女加腐女,原创,睡觉睡到自然醒,美食,游戏动漫
美食
健康
投资理财,旅游,教育就业,时尚,健康
游戏动漫,美女
艺术,运动,思考,金牛座,可爱,乒乓球
汽车,新闻资讯,娱乐,体育
娱乐
IT 数码
旅游,喜欢音乐,看电影,游戏动漫
旅游
娱乐,时尚,文艺,旅游
名人明星
萌宠,动漫,狮子座,音乐
新闻资讯,投资理财,时尚
娱乐,美食,游戏动漫
旅游
校园生活,宠物图集,视频音乐,搞笑幽默,读书分享
新闻资讯,娱乐
星座控,彩妆,完美主义,音乐发烧友,双子,爱猫,时尚,美食,文艺
执着,可爱,宜昌,股票,制药,广告,单身
娱乐
摩蝎座,动静
搞笑幽默,少火的人
阿狸控
娱乐
搞笑幽默
新闻趣事,美女,搞笑幽默
新闻资讯,IT 数码,美食,游戏动漫,体育
IT 数码,汽车,发呆,幽默,玩,快乐,校园,迷糊,怀旧,打篮球
星座命理
处女座
小哥控,V 迷,娛樂,愛宥嘉,愛美男,萌男控,嘉人,动漫控,耽美狼,宅女
艺术,摩羯座,骑车,苹果,忠诚心
强迫症,服装,猫控,娱乐,文艺,旅游,美食,时尚

文艺
心理学,宠物,宅女一枚,学生一枚,天蝎座,时尚
运动,听歌,紫色,处女座男生,唱歌,篮球,小说,手机,90后,吃货
射手女,美食控,时尚达人,吃货,乐观,幽默
娱乐
旅游
旅游,搞笑幽默,汕头生活,文艺,美食,时尚,游戏动漫,健康
投资理财,健康,时尚,美食,娱乐
教育就业,投资理财,旅游,美食,娱乐
游戏动漫
娱乐,文艺
搞笑幽默,新闻趣事,微博奇葩,旅游,美食
美食,旅游,快乐大本营,旅游美食,爱旅游,睡觉,自由,双子,看电视,80后的尾巴
旅游
小萌妞,小麻豆,小模特,小童星,小萝莉,可爱宝贝,小美女,大眼睛,纪姿含
健康,新闻资讯,美女,文艺
时尚
旅游,美食,娱乐,时尚,汽车,写字,自由职业
教育就业
体育
旅游
文艺,旅游,美食
IT数码
美女,汽车,旅游,投资理财,体育
搞笑幽默
娱乐,体育,新闻资讯,美食,文艺
娱乐,美食
自由,唱歌,交友,吃货,天秤座,文字,音乐,90后,旅游,动漫
新闻资讯,游戏动漫,IT数码,体育
健康,文艺
星座命理,旅游,纠结,双鱼座,偏执狂,美食爱好者,宅,冷笑话,傻,双鱼女
IT数码,时尚,文艺,体育,星座命理,娱乐
文艺,荣迷
旅游
娱乐
汽车
娱乐,IT数码,90后,天蝎座,永远不用减肥,明骚易躲,天蝎座的90后
娱乐,旅游,体育
美女,游戏动漫,美食,娱乐,旅游,星座命理,IT数码
新闻趣事
投资理财,新闻资讯,旅游,军事,体育
汽车,教育就业
体育,军事
时尚,美食,体育

新闻资讯,教育就业,美食
美女
投资理财,军事,新闻资讯,旅游,IT 数码
教育就业,美食,体育
旅游
IT 数码,旅游,新闻趣事,美女,校园生活
游戏动漫
美女
投资理财,旅游,时尚,文艺,娱乐,美食
乐观,游戏,摩羯座,音乐,文学,体育,爱情,读书,唱歌,宅男 80 后
搞笑幽默,安卓,90 后
新闻趣事
星座运势
美食,视频音乐,旅游,美女,时尚,IT 数码,娱乐,体育
体育
IT 数码,时尚,美食,游戏动漫
时尚
Jedward,謝霆鋒,娱乐
金牛座,乐观,自由,平常心
新闻资讯,健康,娱乐,时尚,星座命理
低调,睡懒觉,狗狗
娱乐,游戏动漫,新闻资讯,旅游,IT 数码
健康
汽车,IT 数码,体育,海绵宝宝,it,k 歌,IT 互联网,爱唱歌,科比
娱乐,文艺,游戏动漫,绘画,动漫迷,爱自由,动漫
穿衣美容
时尚
旅游,游戏动漫
微博奇葩,游戏动漫
新闻资讯,军事,旅游
体育,投资理财,军事,娱乐,IT 数码
美食,星座命理,游戏动漫,娱乐,体育
读书分享,情感生活,星座运势
射手座,体育,美食
美食,做个好人
投资理财,娱乐,文艺,星座命理,旅游
笑
新闻资讯,投资理财,教育就业,美食
健康,旅游,星座命理,时尚
喜欢旅游
没厘头,活泼开朗,矛盾体,看漫画,臭美
时尚,文艺,娱乐,旅游
IT 数码,90 后非脑残,双鱼,新闻,文字,音乐控
新闻资讯,汽车,旅游

游戏动漫
萌宠,名人明星,穿衣美容,时尚,娱乐,文艺,美食,旅游
娱乐,投资理财,健康,星座命理
娱乐,教育就业,文艺,美食,旅游,时尚
旅游,娱乐
旅游,娱乐
健康
娱乐,星座命理,文艺,矛盾体,猫和老鼠,爱睡觉,J迷,90后,睡觉,宅女一枚
别人没必要知道
微博奇葩
体育资讯
健康
美食
宠物图集,宜宾生活,读书分享,许嵩,学生一族,完美主义,开心
江阴,咨询,装饰装潢,细心,盐城,广告,建筑,肇庆
体育
天蝎,bigbang
IT数码,娱乐,投资理财,游戏动漫,旅游
视频音乐,新闻资讯,文艺
时尚,美食,健康,娱乐
时尚,娱乐,親情,品味人生百态,无束缚,玩樂,樂天派,美食,小说,校园
搞笑幽默,宅,睡觉,看书,动漫,健康
游戏动漫,美女,时尚,汽车,健康,IT数码,体育
搞笑幽默
文艺
汽车,教育就业,美女,新闻资讯,体育,IT数码
文艺
美女,游戏动漫,体育,乔飞人,2K系列,处女座,篮球,足球,皇马死忠
美女,微博奇葩,穿衣美容,北京生活,文艺,娱乐,健康,时尚,IT数码
穿衣美容,搞笑幽默,美女
美食,旅游,新闻资讯,投资理财,IT数码
文艺
娱乐,体育,热火球迷,科幻,打篮球,三国杀,NBA,学生一枚,许嵩,火影
汽车,旅游美食爱好者,军事
SuperJunior,bl,O型血,重口味,Android,Music,i,炎,F1,sj
谢娜,张杰
律师,幻想,荆州,嘉兴,程序员,旅游
娱乐
读书分享
星座命理,美食,健康,时尚,娱乐
投资理财,旅游,IT数码
游戏动漫
星座命理,搞笑幽默,视频音乐,美食,游戏动漫,汽车,旅游,时尚
勇敢,成都,人马座,篮球,运动,金华

娱乐
美女,美食,IT 数码,游戏动漫,时尚
娱乐,囧
草泥马是吉祥物,好屌非丝,中国好呻吟,很傻很天真,狮子座,朗尼先生,歌手鼓手吉他手
娱乐
慈善,可爱,硬件,审计,好脾气,乒乓球
新闻资讯
微博奇葩,江西生活,新闻趣事,搞笑幽默,旅行,吃货
旅游,美食,微博奇葩,视频音乐,搞笑幽默
时尚
穿衣美容,星座命理,时尚
许巍,爱自由
时尚
时尚,旅游,教育就业,投资理财,美食
旅游,游戏动漫,美食,体育
美食,听歌,好书,80 后
投资理财,星座命理
美女,旅游,美食,穿衣美容,视频音乐
美女,体育
兽医,动漫,天秤座,宅男,宠物控,网球爱好者
快乐,美食,微博奇葩,穿衣美容,视频音乐
义乌,成都,园林业,重庆,反应佳,艺术,程序员
美女,IT 数码,摄影爱好者
旅游,美食
美女,汽车
文艺
游戏动漫,文艺,IT 数码,旅游,美食
旅游,美食,【BIGBANG】
狮子女,小清新,自然,90 後,看电影,文艺,爱自由,时尚,美食,旅行
职场招聘
睡觉,90 后,吃,旅游,美剧,巨蟹座
强迫症,高中生,吃货
体育,新闻资讯,80 后的尾巴,深圳,旅游美食,校园,喜欢听歌,养生,笑话,篮球
时尚,汽车,美女,军事,IT 数码,教育就业,体育
上网,篮球
娱乐,美食,时尚,健康
旅游
星座命理
财经资讯
娱乐
文艺,体育,微笑,社会
新闻资讯
时尚,旅游,美食,文艺,娱乐
卡通控

动漫, 双鱼座, 游戏, 高中生, 活泼开朗, 孩子气, 校园
教育就业
健康, 旅游, 星座命理, 听歌, 安静, 悲伤, 自卑
健康, 美女, 娱乐, 体育
揭阳生活
文艺, 纯音乐, 文字, 美女, 睡觉
健康, 汽车, 文艺, 军事, 新闻资讯, 旅游
旅游
娱乐
IT 数码, 娱乐, 星座命理, 美食, 体育
黑龙江生活, 教育就业
健康, 美女, 军事, 语录, 听歌
美食, 旅游, 微博奇葩, 视频音乐
健康
陕西生活
美食, 军事, IT 数码, 文艺, 娱乐
文艺, 旅游, 娱乐
旅游
游戏动漫
游戏动漫
体育
90 後, 旅行, 自由, 管理, 梦想
健康
游戏动漫
时尚
双鱼座, 紫色, 旅行, 手机, 搞笑, 互联网
游戏动漫
美食
视频音乐, 相爷本命, 阿春男神, 墨明棋妙粉, 瓶邪, 广播剧, 声控, 河图本命, 古典音乐, 文艺
娱乐, 旅游
旅游, 健康, 美食
旅游, 健康
星座命理
新闻资讯
旅游
星座命理
时尚
娱乐, 时尚, 旅游
娱乐
旅游
健康, 挑战自我, 汗水, 拼搏, 欢乐, 爱好, 娱乐, 休闲运动, 体育运动
娱乐
美食, 微博奇葩, 新闻趣事, 搞笑幽默, 河南生活
美女, 时尚, 教育就业, 星座命理, 投资理财, 90 後

旅游,时尚
天秤,电影,音乐,90 後,人生,电视,自然,坚强
投资理财,健康,旅游,娱乐
名人明星,喜欢听歌,90 后,唱歌
美食
IT 数码
旅游,投资理财,游戏动漫,美食,体育
广告,书籍,爱旅行,内向
时尚,美女,娱乐,唱歌，看书，
文艺,爱写作,爱睡觉,听音乐
星座运势,旅游,星座命理,娱乐,看电影,星座,神经质,低调
穿衣美容,美食,四川生活
新闻资讯
搞笑幽默,视频音乐,旅游,穿衣美容,紫色,自由,水瓶座,90 后,看书,电影
星座命理
汽车
娱乐
美食
娱乐
设计,123,网络,网购,电影,电子商务,微博,听歌,搞笑,营销
美食,文艺,投资理财,教育就业,娱乐
日剧,Rocker,吃,双鱼座,美剧,动漫,写作,90 后,电影,旅游
旅游,汽车,投资理财,娱乐,IT 数码
投资理财,听话的孩子,小乖,萌宠,乖乖女,咪咪,喵星人,萌
健康
制药,西安,兜风,牧业,游戏,金华,淄博,骑车
保护他人,盐城,荆州,可爱,吉林,忠诚心,室女座
娱乐
文艺,旅游
娱乐
娱乐
旅游,娱乐,投资理财,军事,汽车
美食,健康,旅游,教育就业,吃货,跳舞
美食,网购,电影,音乐,网络,旅行,艺术,淘宝卖家,淘宝店主,时尚
旅游
时尚,星座命理,娱乐
美食,旅游
美女
体育,汽车
投资理财
篮球,打游戏,购物,台球
健康,美食,IT 数码,汽车,时尚
文艺,新闻资讯,娱乐
旅游

星座命理
体育资讯
星座命理,娱乐,游戏动漫,汽车,吸血鬼
IT 数码,文艺,体育,教育就业
汽车,文艺,体育,娱乐
制药,搞笑,湖州,骑车,忠诚心,南京,杭州
我爱帅哥,晒不黑
美女,美食,军事,体育
娱乐
看书,家具,思考
娱乐,购物,手机控,上班族,水瓶座
文艺,娱乐
娱乐
游戏动漫,乐天派,射手座
游戏动漫,体育
好性格,交朋友,工作,低调,工作狂,单纯,音樂,我就是我,坚强
军事,时尚,旅游,健康,IT 数码
信仰,自由职业
文艺
游戏动漫,文艺,娱乐
shopping,感性,唱歌,吃货,幽默,大学生,宅,电影,80 后,时尚
小说,天蝎座,电子商务,教育就业,新闻资讯,军事,文艺,娱乐
教育就业
成都,活泼,金华,游戏,乒乓球,执着,金牛座,忠诚心
新闻趣事,微博奇葩,旅游,江苏生活,汽车,投资理财,美食,军事,娱乐
毅力,网络电子商务,艺术,北京,骑车,股票
旅游,时尚,投资理财,星座命理,健康
旅游
星座运势,星座命理,星座,美食,听音乐,看电影,海贼王,爱音乐
游戏动漫,娱乐,美女
文艺,美食
宠物图集,南京生活
美食
娱乐
新闻资讯,投资理财,军事,娱乐,汽车
萌宠
IT 数码,体育,投资理财
新闻资讯,星座命理,教育就业,健康,时尚
体育,旅游,IT 数码,汽车
旅游
荆州,审计,制药,好脾气,义乌,上海,济宁
新闻资讯,旅游,娱乐,美女,体育
淄博,毅力,运动,双子座,美女,狮子座,自觉,法律,水利
旅游

娱乐,时尚,美食,健康,旅游
旅游,游戏动漫,汽车,IT 数码
八卦杂谈
穿衣美容
美食
投资理财
单身,猪,路痴,购物狂,外貌协会,宅女,小说,傻,巴黎梦,迷糊
体育资讯
游戏动漫,美食,IT 数码,体育,娱乐
自由,文字,听歌,旅游,摄影,水瓶座
guitar,画画,骑行,锦衣夜行的痛楚,呵呵,甲同学
搞笑幽默
美女
体育资讯,篮球, 台球,
时尚,教育就业,美女,娱乐,体育
娱乐
時尚
IT 数码,时尚,游戏动漫,旅游,文艺,娱乐,玩,90,计算机
新闻资讯,娱乐,汽车,体育
娱乐,文艺
游戏动漫,文艺,教育就业,娱乐,体育
时尚,小说,吃货,上网,旅游,偶稀饭睡觉觉,音乐,摩羯座
视频音乐
星座运势,古灵精怪,偶喜欢玩,爱唱歌
摄影爱好者,旅游,搞笑,看书,乐观,紫色,自由
旅游
教育就业
娱乐,游戏动漫
健康,美食
葡萄牙语,教育就业
时尚,IT 数码,教育就业
IT 数码,时尚,娱乐
文艺
职来职往,欧洲杯,世界杯,美术,音乐,运动和旅游,照相爱好者,足球迷,健身,体育
健康,娱乐,教育就业,旅游,美食
游戏动漫
美食
美食
旅游,美食,穿衣美容
娱乐
广东生活
livehouse,Club,酒吧,酒吧控,夜店,娱乐圈,娱乐
健康,新闻资讯,教育就业,投资理财,IT 数码
美女,旅游,娱乐

健康,游戏动漫,旅游,军事,体育
娱乐,星座命理
搪瓷,新疆,旅游,经济贸易,足球,篮球,紫色,读书,善良,乐观
IT 数码,八卦杂谈,搞笑幽默,音乐
金牛座,电影,音乐,羽毛球,上网
汽车,旅游
淡定,爱自由,爱电影,爱旅游,动漫控,双子座,咖啡,爱美食,自由主义乐天派,自由散漫
美女,汽车
搞笑幽默
汽车,旅游,美食,体育,文艺
摩羯座,小说,90 后,电影,羽毛球,宅男,吃货,自由,交友
娱乐
健康,美食,旅游,投资理财
平常心,英语,自由,唱歌,网购,爱情,生活,时尚
旅游,体育
游戏动漫,汽车
武术,美食,教育就业,军事,新闻资讯
太阳,骑单车,旅行,唱歌,听歌,dota,摄影,音乐,金牛座,吃货
运动,南京,写作,美女,慎重,网络电子商务
炎亚纶的布丁
写作,传媒人,安徽师范大学,文艺范儿,旅游,90 后,文学,每天读点文学吧
范二青年,8 卦女,音乐控,强迫症,义气,直爽,95 后,魏晨控,铁橙,乐橙互听
美女,新闻资讯,投资理财,IT 数码,娱乐
博客,爱音乐,听音乐,咖啡控,涂鸦,夜猫子,忧郁,臭美,歌手,活泼开朗
微博奇葩,穿衣美容,美食,名人明星,浙江生活
旅游
娱乐
月光仙子
汽车,IT 数码
美食,旅游
白羊,新闻资讯
美食,文艺,简单不庸俗
篮球,摇滚,梦想,室内设计,美术,时尚,汽车,军事,旅游,娱乐
旅游,健康,美食,投资理财
美食
健康,新闻资讯,娱乐
穿衣美容,时尚
搞笑幽默
蓝牙,无线技术,巨蟹座,攻城狮一头,行到时间尽头,旅到世界尽头,学习是一种信仰,爱生活
爱产品
新闻资讯,旅游
旅游,传媒,双鱼座
狮子座,偶稀饭睡觉觉,爱唱歌,网络,手机,90
旅游,美食,星座命理,穿衣美容,上海生活

时尚
娱乐,体育
仙后一枚,东方神起
搞笑幽默,体育,健康,新闻资讯
文艺,靠谱,认真,向上,自信,温暖,乐观,自由,爱
星座命理,娱乐,时尚
健康,时尚,美食,星座命理
旅游,学生,偶稀饭睡觉觉,自由,90 后,上网,宅女
星座命理,文艺
教育就业,星座命理,投资理财,健康,娱乐
娱乐
美食
美女,军事
旅游,军事,娱乐
白羊座,善良,宅女,睡觉,80 后,音乐,英语,看书
星座命理,美食,娱乐,体育,狮子座 T
汽车,体育
文艺,时尚,旅游,健康,娱乐,唱歌,双子座女生
时尚,吃
工程照明,智能家居,家居照明,LED,LED 照明,商业照明,投资理财,IT 数码
时尚
星座命理,旅游
时尚
美食,美图摄影,读书分享,视频音乐
八卦杂谈
投资理财,游戏动漫,美食,体育,IT 数码
游戏动漫,古风控,cosplay,动漫发烧友,二次元社团
SuperJuniorM,爱夏,汪茗洋,旅游,用心灵感动世界,乐橙
娱乐,爱陈奕迅,吃货
旅游,健康
时尚,汽车,旅游,文艺,娱乐,茶道,美术绘画
潮人,服装,心理学,宝宝,听音乐,购物,星座命理,娱乐,美女,时尚
美食,健康,时尚
旅游摄影,读书分享,美图摄影
健康
健康,美女,娱乐
穿衣美容,新闻趣事
射手座,上网,运动,体育,90 后,手机,宅男,游戏,偶喜欢玩,音乐
娱乐
90 后,看书,游戏,好脾氣,成熟,阅读
欧美控,潮流,减肥,星星波点控,蓝色控,广州面交店,唱 K,双子女
时尚
搞笑幽默
旅游

时尚,美女
旅游,健康,新闻资讯,投资理财,工作狂,马大哈,情绪化,矛盾
美女,游戏动漫,爱音乐,爱美食,爱自由,懒人
IT 数码
时尚,健康,旅游,星座命理,美食,游戏,电影,摄影,奔三自信的女人
cos 后期,动漫,VOCALOID,ACG,二次元
旅游
IT 数码,文艺,娱乐
娱乐
娱乐
娱乐,旅游,游戏动漫,军事
美女,羽毛球,90 后,爱,开心
美食
旅游,美食,星座命理,视频音乐,穿衣美容
美食,抽奖控,街跑控
时尚
汽车
电影,慈善
文艺,体育
健康
旅游,IT 数码,美食,游戏动漫,娱乐
设计师,巨蟹座
我自豪,我是 00,学生
视频音乐
旅游
星座命理,健康,美食,体育,娱乐
旅游,美女,游戏动漫
汽车
军事,好性格,创业,设计,低调
健康,汽车,旅游,文艺,体育
懒人一个,爱哭,无奈,纠结控,喜欢一个人,冷笑话,射手,夜猫子,吃货,文艺
90 后,小说,历史,美剧,美食,大学生,吃货
美食,星座命理,IT 数码,搞笑,数码,篮球,乐观,自由,时尚,音乐
旅游,美食,萌宠,穿衣美容
军事
交友,唱歌,睡觉,旅行,篮球,摄影,游戏,创业
校园生活
新闻资讯,美食,健康,旅游,游戏动漫
汽车,体育,IT 数码
创业是我的梦想,吃货一枚,很多人,交友
文艺,娱乐
金牛座
医学,快乐大本营,O 型血,交友,看书,唱歌,运动,英语,学生,90 后
游戏,幻想,英语,网络,宅男,摄影,篮球,上网,睡觉,仙剑

书,music,投资理财,教育就业,健康,军事
时尚,美食,娱乐,投资理财,健康
游戏动漫
话还是我先说吧,筹码还不够
文艺,旅游,星座命理,时尚,美食,娱乐
忠诚心,深圳,写作,肇庆,乒乓球,苹果,狮子座
创业,水瓶座,美食,80 后,自由职业,IT
时尚,娱乐,汽车,体育,IT 数码
蘑菇头,格子衬衫控,黑框眼镜控,死理性派,学院派,后印象派绘画,垮掉派文学,影评乐评,节奏强迫症患者,古典吉他
爱情,反应佳,室内设计,互粉,运动
美食,旅游
体育,爱笑,快乐,玩,漫画,笑话,爱吃,打篮球,三国杀,足球
求关注,90 后非脑残
娱乐,旅游,星座命理
体育,健康,汽车,美食,时尚,文艺
吃,睡觉
娱乐,对未来感到迷茫
旅游,时尚,体育,美食,投资理财
文艺,情绪化,90,宠物,淘宝,懒,安静,爱吃,天蝎座
单身
星座命理,河南生活,搞笑幽默,新闻趣事,名人明星
旅游,娱乐,投资理财,健康,时尚
体育
喜欢紫色,星座命理,旅游,美食,00 后,巨蟹座,学生
体育,美女,旅游,投资理财
旅游,娱乐,体育
首富,吴诗心,鹿晗,卞白贤,致美丽的爱咳嗽,杯子杯子酱,咳嗽阿姨洗铁路,大家族永在,牛奶白永在心头,灿白一生推
游戏动漫,IT 数码,时尚,教育就业,健康,旅游,文艺
美女
新闻趣事,微博奇葩,旅游
娱乐
美食
美食,汽车,美女,健康,时尚,文艺
汽车,美女,旅游,娱乐,体育,篮球
文艺
帅哥,游泳,桌球,90,猫,B 型血,狗狗,演员,美剧迷
汽车,游戏动漫
名人明星,校园生活,美图摄影,体育资讯,美女
悠久之翼 EF,家族 CLANNAD,PSP 党,罪恶王冠,双鱼座
旅游
美食,美女,广东生活,新闻趣事,搞笑幽默
星座命理,IT 数码,教育就业,美食,娱乐

美女
公益,新闻趣事,校园生活,搞笑幽默,娱乐
星座命理,美食
体育
时尚,IT 数码,文艺,娱乐,投资理财
娱乐
写作
游戏动漫,美食,IT 数码,娱乐,体育,动漫,网络,吴尊的粉丝,金牛座,游戏
游戏动漫
旅游,新闻资讯,体育,娱乐,军事
瘦身,简单,吃货,生活,90 后,善良,囧,搞笑,电影,天秤座
执着
搞笑幽默,美图摄影
娱乐,星座命理,美食,旅游,旅行,天蝎座,90 后,校园,音乐,手机
娱乐,美食,旅游,体育
健康
时尚,旅游,娱乐,文艺,星座命理
娱乐,体育,美食
名人明星
学生一枚,爱幻想,交朋友,坚强,紫色,互联网,沉默,香橙,听音乐,美食
双子座,娱乐
名人明星,星座运势,听歌,看书,宅,90 后
名人明星
旅游
IT 数码,汽车
新闻趣事,爱旅行,旅游美食,开心,柯南,喜欢音乐
新闻资讯,教育就业,IT 数码,旅游,健康
美食
萌宠
游戏动漫,军事
健康
时尚,天蝎座,善良,囧,篮球
体育资讯,佛山生活,IT 数码,视频音乐
娱乐
美女,健康,美食,IT 数码,军事,娱乐,体育,文艺
文艺,笑
健康,旅游,星座命理,娱乐,投资理财
文艺,汽车
IT,唱歌
汽车,IT 数码
军事,新闻资讯,旅游,教育就业,体育
小说,偶稀饭睡觉觉,射手座,90 后,上网,学生,囧,阅读,交友,听歌
游戏动漫
投资理财,军事,体育,动漫控,吃货一枚,90 后,美食

视频音乐
时尚,投资理财
美食,美女,时尚,娱乐,体育
美食
娱乐
校园生活
体育,旅游
新闻资讯,投资理财,汽车,美女,军事,IT 数码
微博奇葩,娱乐,新闻资讯
时尚
旅游,健康,IT 数码,文艺,娱乐
游戏动漫
星座命理,娱乐,游戏动漫,旅游,美食,文艺
tvb,宅,自由,时尚,射手座,美食,music,爱旅行,陈奕迅,简单
大庆生活
旅游,新闻资讯,投资理财,军事,星座命理
校园,媒体联盟,城市媒体,旅行
喜欢旅游,睡觉,动漫,热爱生活,美容,逛街,减肥,时尚,美食,爬山
时尚,教育就业,健康,星座命理,新闻资讯
好想去旅游哦
上海生活
读书分享,星座运势,美食,90 后,金牛座
新闻资讯,文艺,时尚,健康,偶尔神经质,双鱼座,90 后,美剧,唱歌,玩儿
文艺,游戏动漫,娱乐
自由职业,好性格,吃货,天秤座,80 后
新闻资讯
唱歌,吃,旅游,游戏,手机,动漫,紫色,巨蟹座,听歌,羽毛球
宅男,摩羯座
娱乐,美食
狮子座,旅游,美食
IT 数码
游戏动漫
星座命理,狮子座,90 后,听歌
体育
娱乐
美食,健康,旅游,文艺,投资理财
自由主义乐天派,医学生
新闻资讯,IT 数码
教育就业,投资理财,星座命理,文艺
名人明星
美容服饰
搞笑幽默
娱乐
美女,汽车,音乐,偶喜欢玩,双鱼座,乐观

听歌,90 后,宅,上网,摄影爱好者,白羊座,好嘛,我又长高了,音乐
美女,娱乐
娱乐,游戏动漫,星座命理
善良,设计,偶稀饭睡觉觉,喜欢音乐,爱笑,单身,阳光,电视剧,玩,快乐
旅行,语录,校园,交友,时尚
健康,时尚,旅游,美食,文艺
教育就业
美食
胡思乱想
星座命理,健康,美食,旅游,时尚,80 后,亲情,摄影爱好者
汽车
我本世间一菩提
新闻趣事,美食
娱乐
旅游
军事,娱乐
星座命理
体育
旅游,汽车,美女
娱乐,汽车,美食,听歌,天蝎座,幽默,旅游,学生
游戏动漫,IT 数码,新闻资讯,娱乐,军事,投资理财,美女
美食,蕾丝控,LOL,90 後
大学生,生活,游戏动漫
美食
艺术,天秤座
投资理财,文艺,健康,旅游,美食
星座命理,IT 数码,游戏动漫,美女,美食
萌宠,健康,新闻资讯,时尚,教育就业,文艺,娱乐
美食,文艺
双鱼座
新闻资讯,游戏动漫,时尚,旅游,文艺,摩羯座,运动,喜欢安静,看书
新闻资讯,游戏动漫,IT 数码,娱乐
美食
体育
豆瓣 video 控,减肥他妈的,适时热血,死飞,电影,kop,荣迷,女毅丝,技术宅
健康,快乐,安静,健身,活泼
文艺,健康,旅游,IT 数码,娱乐
娱乐
搞笑幽默,新闻趣事
时尚
美食,军事,汽车,时尚
游戏动漫,娱乐,体育,军事,IT 数码
旅游,新闻资讯,娱乐
娱乐

美女
时尚,游戏动漫,文艺,旅游,美食
重口味,情感生活,视频音乐,搞笑幽默,娱乐
北京生活
浙江生活
旅游
体育,旅游
旅游,文艺
游戏动漫,文艺
忧郁
ios 游戏,手机游戏,3D 游戏,手机游戏开发,动作游戏,ios,IT 数码
健康
新闻资讯,娱乐
汽车,IT 数码,美女
IT 数码
仙剑,漫画,喜欢听歌,小说迷,健康,文艺,美食,游戏动漫
星座命理,金牛座
体育
自由,旅行,宅女,吃货,双鱼座,网购,90 后,音乐
娱乐,活力,健康
星座命理,文字记录生活,听歌,巨蟹座
娛樂
健康
旅游
体育,IT 数码
喜欢旅游,直接 2 惨,文艺
娱乐
宅女,体育
体育
星座命理,美食,体育,娱乐,美女
吃货一枚,懒,胡思乱想,怀旧,疯狂,无厘头,爱饺子
梦想,吃货,Music,热情,无聊,电影爱好者,打球,蓝色,矛盾体,nba
旅游,星座命理,穿衣美容,搞笑幽默,美女
K 歌达人,KTV 低调分子,音乐爱好者
北京生活
美食,军事,娱乐
星座命理,新闻资讯,健康,时尚,汽车
娱乐,美食,旅游,时尚
时尚,美食,美女,IT 数码,投资理财,文艺
游戏动漫,爱玩,电影迷
娱乐
情感生活,美图摄影,搞笑幽默,美容服饰
文艺
旅游,IT 数码,体育

文艺
巨蟹座
扎巴依,计划赶不上变化,失眠与嗜睡同在,恋家,阿鲁巴,起床失败爱好者,科比,湖人
快乐大本营,天蝎座,星座命理,娱乐
娱乐
蓝色性格,大学生,旅行,闲不下来的人儿,篮球,美食,双鱼座
旅游,娱乐
娱乐,新闻资讯,游戏动漫,军事
娱乐
休闲,生活,善良,水瓶座,服饰,健康,时尚,星座命理,美食,娱乐
星座命理,娱乐,旅游
文艺,何炅,宋茜,尼坤,阅读,翰爽控,维尼饭,水瓶座 B 型血
摄影爱好者,吃货,天秤座,乐天派,咖啡控,旅游,健康,美食,时尚,汽车
美女
浪漫
星座命理,游戏动漫,美食
搞笑幽默
旅游,美女,教育就业,军事,美食,体育
韩剧,BIGBANG,维尼夫妇,美食控,娱乐,时尚
购物狂,奋斗,育儿,天蝎女,美食,自由职业
体育
健康,教育就业,军事
懒虫,爱 eason,吃货,射手座,乐观
体育
健康
咖啡,电影迷,旅行,音乐,坚强,教育就业,军事,IT 数码,旅游,投资理财
娱乐
体育
名人明星,河南生活,穿衣美容,星座命理
美食,娱乐
澳大利亚生活
美剧,摇滚,猫咪,魔兽世界,吸血鬼
时尚
文艺,IT 数码,旅游,星座命理,美食
海贼王,健康,微博奇葩,旅游,美食,美女,新闻趣事
汽车,美女,体育
旅游,健康
生命诚可贵,爱情价更高
星座命理,娱乐,快乐
新闻趣事,视频音乐,美食,旅游,美女,喜欢旅游
时尚,游戏动漫,星座命理,旅游,投资理财,文艺,娱乐
游戏动漫,电影迷,85 后,自然,玩儿,旅游
旅游,健康,新闻资讯,教育就业,投资理财
大学,娱乐,篮球,90 后,学生,动漫,乐观,好性格,交友,宅

广东生活
投资理财,汽车,IT 数码
文艺
搞笑幽默
旅游,手机,善良,阅读,媒体,语录
办公环境,人体工学,室内设计,办公系统家具,电影爱好者,时尚,投资理财
运动,篮球,二手车,深圳,汽车
写字,唱 K
新闻资讯,旅游,美食
名人明星,娱乐
新闻资讯,健康
健康,美食,星座命理
旅游
游戏动漫
文艺,新闻资讯
体育资讯,大学,篮球,运动
搞笑幽默,美图摄影
视频音乐
游戏动漫,娱乐,旅游,美食
双子座,海贼王,BIGBANG,小说迷,动漫宅,GD,权志龙
美食,汽车,旅游
体育
星座命理
教育就业,投资理财,汽车,体育,娱乐
游戏动漫,星座命理,美食
美食,萌宠,搞笑幽默,娱乐
美女,体育
静静的一个人
文字
动漫迷,90 后,厦门,快乐大本营,海音报,电脑,开朗,发呆,爱音乐
美食,娱乐,旅游,时尚,游戏动漫,美食,文艺
大爱尼坤
健康
游戏动漫,爱自由
美食,佛山二中,爱猫爱吃
搞笑幽默
文艺,时尚,旅游,美食,娱乐
健康
法律,网络营销,营销,财经,传媒,心理学,地产
时尚
美容服饰,情感生活,视频音乐,新闻趣事,校园生活,双鱼女,爱猫一村,Cym
广告
微博奇葩,健康
旅游,投资理财,化妆,白羊座,美容护肤,低调,爱旅游,90 後

星座命理
娱乐
保密
健康,娱乐,体育
娱乐
搞笑幽默,美食,新闻趣事,穿衣美容,双鱼女
搞笑幽默
文艺
军事,IT 数码,教育就业,体育
任性,小清新,强迫症,搞笑,双重性格,疯子,完美主义,时尚,旅游,文艺
健康,娱乐
1122,【蓮】,ふぁねる,あけねる,きー助,晃要
时尚,美食,旅游,体育,娱乐
时尚,旅游
美女
澳大利亚生活
娱乐,卡纳基控,英语,学生
娱乐
星座命理,IT 数码,健康,美女,娱乐,时尚,体育
体育,科蜜
健康,娱乐,游戏动漫,星座命理
时尚
游戏动漫
株洲生活
美女,旅游,游戏动漫
桂林生活
搞笑幽默,小资
旅游
较劲金牛女,无厘头,爱旅行,迷糊,旅游,美食
搞笑幽默,矛盾体,胡思乱想
萌宠,90 後,娱乐,美食,一般宅,小文艺
名人明星
文艺,投资理财,健康
新闻资讯,学生一枚,活泼,古灵精怪,天蝎女,校园,娱乐,时尚,健康,旅游
美食,韩剧,娱乐
教育就业,美食,时尚,旅游,文艺
美女
娱乐
文艺
星座命理
星座命理
搞笑幽默,体育
健康,时尚,旅游,美食,娱乐
狮子王,彭于晏,王力宏,朱莉,美少女战士,二叔,德普,神奇宝贝

旅游
旅游,80 后,love,魔羯座,淘宝,购物,美食
旅游,军事
服饰,购物,gxgJeans,Gstar,GXG,KOYO,时尚,娱乐
微博奇葩
健康,IT 数码,旅游,娱乐,时尚,吃货爱美食,购物狂,购物达人,素描,建筑
娱乐,孩子气,逛街
军事,IT 数码,教育就业,健康,美食
汽车,游戏动漫,体育,文艺,新闻资讯,投资理财,军事,健康
旅游,时尚,文艺,娱乐,体育
星座命理,江苏生活
努力奋斗工作,爱篮球的 90 后,吉他爱好者,减肥,吃货,天蝎座的 90 后,爱文字,忧伤
IT 数码
教育就业,IT 数码,军事,体育,大学生,旅游,篮球,天秤座,NBA,听歌
时尚,完美主義者,大愛貝克漢姆
娱乐
教育就业
娱乐,夜猫,睡懒觉,吃,听歌
美女
健康
幽默的我,篮球爱好者,交友,唱歌,娱乐,自由
娱乐,美食
无厘头
星座命理,教育就业
娱乐
时尚,自由,乐观,宅女,创业,美食,文学,生活,设计,旅行
娱乐,汽车,时尚,星座命理
星座命理
时尚,爱情,摩羯座,睡觉,上网,旅游,听歌,学生,宅
时尚
自由,文字,摄影,艺术,文学,摄影爱好者,爱情
美食
美食
股票,wow,纠结,午饭,自信,坚强
搞笑幽默
娱乐
旅游
学生,电影,听歌,网络,媒体,校园,爱情,大学生,NBA,旅游
北京生活,懒,快乐,休闲,体育
投资理财
军事,体育
耗子啃猪脚杆,耗子啃西瓜
美食,自由,红酒,宠物
美食,健康

娱乐
汽车,旅游,睡觉,上网
文艺
娱乐
IT 数码,时尚,娱乐
星座命理,愤青,恐怖片,美剧迷,双子座,电脑控制狂,起床困难户,LES
军事,体育
发呆,文艺,开朗,军事,自然
美食
健康,美食
娱乐
星座命理,新闻趣事,微博奇葩,搞笑幽默,广州生活
美食,健康
睡觉,健身,宅女一枚,演员,科比,火影忍者
美女
娱乐,IT 数码
美食
八卦杂谈,吃货一枚,哈哈,军事
健康,旅游
萌宠,雪纳瑞
游戏动漫,娱乐,军事
美食,穿衣美容
健康,体育,汽车
娱乐
旅游,90 後,低调,爱自由,单身,汽车,体育
萌宠,搞笑幽默,新闻趣事,游戏动漫,IT 数码,文艺,娱乐,美食
努力不一定成功,放弃就一定失败
美食,娱乐,游戏动漫
旅游,美女
梦想,情感,美术,室内设计,电影迷,电脑,聊天,幻想,冷笑话,执着
新闻资讯,旅游,军事
傻,乐天派,外向,小说控
旅游,体育,交友,运动
潮,随性,服装,时尚,rock,HIPHOP,adam,bigbang
旅游,美食,IT 数码,娱乐
银川生活
旅游,时尚,美食,星座命理,娱乐
旅游
白羊座,微博,自由,90 后,网络,平常心,旅行,交友
旅游
IT 数码,体育
星座命理,时尚
旅游
汕尾生活,新闻资讯

汽车
美食,星座命理,旅游,投资理财,健康
穿衣美容,自由
健康,爱生活，爱自己
娱乐
梦想,执着,小清新,文艺,听歌,90后,摄影,旅游,文字
美食,文艺,健康,旅游,军事
山东生活
足球迷
旅游,娱乐,吸血鬼日记,狗狗,love,k歌
美食
教育就业,投资理财,汽车
IT数码
星座命理
篮球,双子座
娱乐,文艺,美食,旅游,时尚
教育就业,IT数码,投资理财,美食,旅游
体育
游戏动漫,时尚
游戏动漫,星座命理,IT数码,文艺,教育就业
高中生,简单,学生一族,坚强,看电影,好性格,美剧,文艺
旅游,IT数码,时尚,新闻资讯,奋斗，旅游逛逛,天枰座,手机,乐观
游戏动漫,体育,娱乐
游戏动漫
搞笑幽默,娱乐
时尚
强迫症,吃货,娱乐
搞笑幽默,IT数码
学生一枚,笑,篮球爱好者,运动,乐观,双鱼座,爱玩
美女,娱乐
教育就业,健康,美食,娱乐
视频音乐,新闻趣事,旅游,微博奇葩,北京生活,美剧迷
新闻资讯,时尚,美女,体育,娱乐
长春生活
汽车,文艺,体育,娱乐,军事
旅游
睡懒觉,懒人,爱自由,梦想,文艺
游戏动漫
美食
蓝色,欧美音乐,泰勒斯威夫特迷,吃货
军事
体育资讯
北京生活,时尚,健康,中国山水画,书法
体育

游戏动漫
时尚,星座命理,美食
IT 数码,健康,星座命理,美食,娱乐
游戏动漫,娱乐
美女
摄影,汽车,新闻资讯,文艺,IT 数码,娱乐,人力资源,动漫迷,奋斗,笑
娱乐
新闻资讯,美女,旅游,IT 数码,娱乐
旅游,时尚,爱酒啊,一点点二,婚纱,缺爱 ING,吃货一枚减肥中,KITTY 控,金牛女,爱睡觉
新闻资讯,健康,时尚,美食,娱乐
军事
汽车,游戏动漫,娱乐,IT 数码,星座命理
娱乐
旅游,娱乐,军事,新闻资讯,健康,投资理财,美女
娱乐
健康
时尚,健康,教育就业,娱乐
浪漫,偶喜欢玩,电影,好性格
星座命理,搞笑幽默,爱芒果台,双鱼座,淘宝网
喜欢音乐,天蝎座,武艺,Kenny,Music,旅游,学生,完美主义,音乐爱好者,沉默
懒人,柯南,星座命理,时尚,健康,美食,娱乐
美食,名人明星,视频音乐,校园生活,美图摄影
体育,白羊座
驾校
美食
旅游,美食,房产
旅游,感性,电影,热情,苦涩,随性,gay
娱乐
旅游
健康
看小说,EXO,林俊杰,幻想,爱睡觉,五月天,唱歌,宅女,90,天平座
汽车,美食
星座命理,教育就业,健康,旅游,美食
文艺
美食,自由,吃货
时尚
IT 数码
汽车
时尚,美女,旅游,美食,娱乐
旅游,交友,摄影,娱乐,囧,游戏,偶稀饭睡觉觉,90 后
娱乐
汽车,时尚,健康,娱乐,大学生,听歌,唱歌,旅游,宅女
游戏动漫,IT 数码
爱琳网,淘宝返利,服装搭配,气质,美丽,逛街狂,时尚

时尚,美女,旅游,美食,娱乐
汽车,时尚,旅游,军事,娱乐
汽车,旅游,娱乐,军事,美女
IT 数码
IT 数码,娱乐,体育
美食,美女,娱乐
时尚
体育
美女,教育就业,投资理财,IT 数码,文艺,靠谱青年就是我
情感生活,星座运势,美图摄影,读书分享,金牛座
宁波生活
双子善变性格、,九零后、,爱自由、,电视迷、,BEYOND 迷、
娱乐,IT 数码,美食,汽车,体育
体育
娱乐
爱吃,爱睡,爱玩,爱学习
体育,健康,旅游
JJProject,李東海,李赫宰,金厲旭
娱乐
体育,娱乐,IT 数码,旅游,文艺
猫控,90 後,巧克力,魔羯座,O 型血
娱乐,汽车
游戏动漫,张根硕,小清新,动漫控,狗狗,欧美控
微博奇葩,时尚
麒麟我命,游戏渣渣,相爷声美,最爱妖君,声控,叔控,猫咪先生最高,深腐半宅,社会工作,广外
IT 数码,娱乐
星座命理
美女,时尚,旅游,美食,娱乐
娱乐
美食,投资理财,时尚,新闻资讯,旅游,健康
教育就业
健康,时尚,教育就业,旅游,文艺
音乐发烧友,欧美,看书,O 型血,80 后的尾巴
搞笑幽默
汽车
旅游
文艺,教育就业,时尚,旅游,星座命理
旅游,教育就业,汽车,健康,新闻资讯
文艺
美女,幽默,开朗,音乐,绘画
美女,娱乐
旅游,美食,健康
星座命理

音樂,咖啡,吉他,艾薇儿,TaylorSwift,自由,吃货
教育就业,汽车,健康,美女,时尚
90 后,摩羯座,乐观加小感性
名人明星
游戏动漫,美食,星座命理,娱乐
美女,体育
美食
视频音乐,旅游
常州生活
投资理财,美食,时尚,健康,文艺
微博奇葩,广东生活,旅游
娱乐
美女
娱乐,教育就业
娱乐,旅游
文艺
旅游
农业,IT,爱情,羽毛球,好书
爱自由,猫,休闲,粤语,学生一枚,电视剧,星座命理,外貌协会,交朋友
搞笑幽默,旅游,美女,新闻趣事,四川生活,诗词,学生一族,摄影师,欧美音乐
娱乐
娱乐
美女,体育
教育就业,健康
名人明星,新闻趣事
水瓶座,贪玩,有点小暴力,爱说话
发呆,矛盾体,旅游,军事,新闻资讯,星座命理,爱笑,双子座,讨厌数学,宅
无聊 ing,hjy,巨蟹座 O 型血
美食,新闻资讯
新闻趣事
旅游,新闻资讯,投资理财
银魂,bigbang,文字控,文艺,日本,游戏动漫
旅游
搞笑
汽车
搞笑幽默
时尚
星座命理,教育就业,健康,投资理财,娱乐
娱乐
旅游
体育
美女,游戏动漫
美图摄影
音乐,宅男

体育
星座命理
名人明星
旅游,美食,文艺,健康
新闻资讯,美女,健康,军事,旅游,汽车,娱乐,文艺
时尚,文艺,IT 数码,娱乐
星座命理
星座命理
美食,健康,文艺,星座命理,玩儿
睡懒觉,闷骚,影视,美剧迷,无厘头,小说迷
娱乐,美食,教育就业,新闻资讯,IT 数码
体育
健康,爱情,微博,偶喜欢玩
美食,教育就业,时尚,新闻资讯,健康,娱乐
美食
娱乐,教育就业,体育,星座命理,新闻资讯
投资理财,美食,汽车,娱乐
美食,旅游
投资理财,健康,旅游
时尚,旅游
体育,谁让我吃豆腐,我就跟谁走
BIGBANG,k 歌,海绵宝宝,风行
体育
游戏动漫,时尚,娱乐,体育,星座命理
星座命理,游戏动漫,体育
汽车
柯震东,天平座,听音乐,EXO,高中生
萌宠,视频音乐,搞笑幽默,新闻趣事,微博奇葩
美食,健康
美食
娱乐
名人明星
美女,军事,做你喜欢的
旅游,星座命理,文艺,新闻资讯
娱乐
文艺,旅游,美食,娱乐
体育
体育,娱乐
爱美食
爱旅行,music
美食
旅游
美食
巨蟹座,宅女,动漫控,瓶邪,90 后,游戏

时尚
MIS,管理学院,信息技术,软件,电子商务,互联网,IT 俱乐部,复旦大学,IT,MBA
萌宠
旅游
投资理财
汽车
美食,旅游,星座命理,文艺
文艺,美食,旅游,健康,娱乐,时尚,教育就业
旅游
文艺,有点儿任性,90 后,时尚,游戏动漫,星座命理,美食,娱乐
营销专家
投资理财,美食,军事
美食,文艺,娱乐,时尚,健康,旅游,美食
卡拉马佐夫兄弟,复活,百年孤独,狂爱三毛文字,余华作品集,路遥作品集,狂爱宫崎骏动漫,傲慢与偏见,日语爱好者,文学阅读者
90 后,亲情,好性格,平常心,吃,英语,吃货,幽默,唱歌,旅游
读书,运动,自由,乐观,吃货
娱乐
美食
体育
萌宠
宠物图集
健康,投资理财
美女,学生
时尚
健康
高三的孩子
正骨,爱萌物,养生,天蝎女,读书,腐女,80 后
处女座,爱音乐,搞笑,O 型血
星座命理,巨蟹
游戏动漫
SHE,吃货,动漫控,妖精,新闻资讯,音樂,淡定,工作,吸血鬼日记,时尚
小女人,旅游,穿衣美容,美食,星座命理
时尚,汽车,美食,IT 数码,吃,唱歌,旅游,电影
星座运势,深圳,谢娜,八卦,偶稀饭睡觉觉,90 后非脑残,喜欢武艺的
旅游
阅读,写作,读书,巧克力,郭敬明,爱生活,画画,巨蟹座,喜欢听歌
星座命理
旅游,美食,westlife,阳光,三国杀,台球,海贼王,DOTA,狮子座,无厘头
旅游,星座命理,时尚,娱乐
旅游
电影迷,爱旅游,时尚达人,公务员
上网,射手座
电影迷,汽车,美食,游戏动漫,健康

咖啡,多愁善感,狗狗,吃喝玩乐,大学生,美女,IT 数码,体育,娱乐,汽车
娱乐,文艺
财经,动漫
妮娜,FXCB 天雷,牛灿
爱唱歌,白羊座,随性,旅游,美食,娱乐
星座命理,旅游,时尚,投资理财,文艺
大学生,好性格,90 后,网络,亲情,吃货,旅行,睡觉,巨蟹座,电影
江苏生活
旅游,新闻资讯,汽车,娱乐,IT 数码
文艺一丢丢,靠谱小青年
微博奇葩
时尚
体育
娱乐,美食,星座命理
旅游,美食
文艺,体育,娱乐,android,爱旅游
文艺,美食,旅游,文学,偶喜欢玩
狮子座,睡觉,听歌,偶喜欢玩,好性格,学生
娱乐,星座命理
沉默
宅女,看书,文字,90 后,好性格,摩羯座,睡觉
化妆品,法国,奢侈品,旅游,新闻资讯,文艺,娱乐,时尚
文艺,学生
爱自由
微博奇葩
旅游
八卦杂谈,策划,音乐发烧友,温柔
品牌营销,战略规划
汽车,军事,IT 数码
汽车,美食,体育
娱乐,文艺,星座命理
星座命理,娱乐
美食,天蝎座
强迫症,星座控,购物狂,港剧迷,芝士控,港粉控,学生
娱乐,时尚
电影爱好者
CAM,CAD,IT 数码
文艺,体育,娱乐
双子座,平常心
萌宠,美食
星座命理,时尚,美食,娱乐
旅游
视频音乐
体育资讯

可爱,减肥,gay,游戏动漫,宅女,美术,室内设计,天枰座,腐,懒
校园生活
穿衣美容
文艺
体育,旅游
新闻资讯,健康,旅游,美食,文艺,流浪,自由,播音主持,支教
足球,吃喝玩乐,科比,李宇春
gossipgirl,孩子气,巨蟹女,宝宝
大房東家,大房主公,百地聖美,一縷飄香,大神帝國
城市休闲车,死飞,山地车,小轮径,公路车,折叠车,狼途,自行车
时尚,美食,旅游,娱乐,体育
文艺,游戏动漫
健康,娱乐
搞笑幽默,健康
游戏动漫,美女,美食,文艺,娱乐
穿衣美容
旅游
建材批发
美食
新闻资讯,健康,文艺,娱乐,平常心,时尚,美食
游戏动漫
什么都喜欢搞搞
军事,IT 数码,文艺,体育,娱乐
内向
鹰潭生活
娱乐
星座命理
新闻趣事
美女
体育
军事,新闻资讯
体育
文艺,体育,娱乐
旅游,星座命理,美食
美女,睡觉睡到自然醒,桌球,篮球,学生一枚
新闻资讯,美女,文艺
娱乐
文艺
网购,天蝎座,睡觉,汽车,电影,吃货,80 后
宅,怀旧,网购,爱睡觉,减肥,逛街,迷糊,旅游美食,天秤,时尚
搞笑幽默
文艺
教育就业,游戏动漫,军事,IT 数码,体育
皇马,柯南,足球,C 罗,体育资讯

教育就业,投资理财,娱乐
新闻趣事,时尚,旅游
体育,小学,足球
旅游
娱乐
健康,体育,娱乐
汽车
美食,名人明星,旅游,视频音乐
情感生活
星座命理,文艺,乐观,善良,自由,音乐,睡觉,玩儿,搞笑,微博
洁癖
健康,投资理财
美食,健康,旅游,时尚
娱乐
美食
旅游,星座命理,IT 数码,文艺,娱乐
浙江生活,新闻资讯
美食
美女,养生,周杰伦,听音乐,音乐爱好者,健康,美食,时尚,游戏动漫,娱乐
星座命理,娱乐
爱美丽
音乐
美食,健康
美食
文艺
教育就业
娱乐
90 後,海贼迷
娱乐
广西生活
阳光,互粉,美食,娱乐,旅游,美女,时尚
游戏动漫
美女,游戏动漫,美食,文艺,娱乐
耽美腐宅女,白羊座,白羊座 A 型血
娱乐,游戏动漫
文艺
美女,美食,文艺,时尚,星座命理,旅游,健康,美食
投资理财,新闻资讯
娱乐
游戏动漫
文艺,美食,投资理财,娱乐,星座命理
汽车,文艺,教育就业,新闻资讯
旅游,双鱼座,好性格,玩儿,吃货,文字,亲情,手机,读书,看书
旅游

投资理财,娱乐,星座命理,IT 数码,体育,音乐,狮子座,90 后
娱乐
美女,娱乐,教育就业,投资理财,军事
三分钟热度
教育就业
美女,旅游,微博奇葩,搞笑幽默
爱玩,韩剧,学习,90 後
文艺
美女
大学
时尚
美女
娱乐,星座命理
游戏动漫
美食
学生一族,校园,执着,魏晨,许嵩,蓝色,白羊座,单身
八卦,手机,军事,文艺,汽车,娱乐,IT 数码
教育就业,时尚
美女,搞笑幽默,音樂,聊天
星座命理,游戏动漫
IT 数码,吃货,好性格,乐观
军事
苏州生活
健康
文艺
搞笑幽默,旅行,电影,唱歌,玩儿,双层性格
搞笑幽默,美食
星座命理,视频音乐,穿衣美容,湖北生活,新闻趣事
健康
美女
美女
文艺,体育,娱乐
文艺,英国,睡觉睡到自然醒,吃货,思考
娱乐
苏州生活,娱乐
时尚,投资理财,健康,娱乐
旅游,美食,微博奇葩,搞笑幽默,美女
搞笑幽默,时尚,娱乐
美男,小说,韩星,星座命理,文艺,娱乐,美食
美女,投资理财,星座命理,IT 数码,体育,游戏动漫,军事,教育就业,旅游,文艺
遊戲動漫
健康,汽车,旅游,星座命理,美食
体育,娱乐,健康,美女,游戏动漫,汽车,投资理财,文艺
美食,爱生活,创业,朋友,教育就业,健康,旅游,IT 数码,体育

旅游
白羊座
体育
娱乐,学生,音乐,射手座,唱歌,睡觉
娱乐
健康,美食,星座命理,教育就业,IT 数码
教育就业
体育,美女
美食
文艺,旅游,时尚,娱乐,健康,IT 数码
美食
投资理财,汽车,美食,美女,娱乐
娱乐,旅游,健康
健康
投资理财,文艺
健康,星座命理,IT 数码,旅游,吃货
微博奇葩
旅游
娱乐
美食
娱乐
时尚,旅游,健康,投资理财,汽车
安静,健身,美食控,爱自由,情感,时尚,娱乐,旅游,喜欢吃美食,喜欢纯音乐
美女
健康
其他生活
美食,电影,旅行
文艺
教育就业,时尚,好吃懒做,懒人一坨,淘宝控,水瓶座,吃货
游戏动漫
旅游,视频音乐
IT 数码,旅游
旅游,健康,时尚,娱乐
时尚,美食,星座命理
美食
自由
旅游,星座命理,美食,文艺,娱乐
星座命理
名人明星
体育,美女
健康
神经大条,苏打绿,偏执狂,学生,宅女
健康,新闻资讯,娱乐,时尚,教育就业,文艺
文字,摄影,晚安

娱乐
美女,教育就业,时尚,娱乐,文艺
爱唱歌,天蝎,文艺,偏执固执臭脾气,十几年也没改掉,精神洁癖狂,不称职的天蝎座,灵魂洁癖
名人明星,巨蟹,爱笑
时尚,睡觉,写书,玩游戏,娱乐解说,羽毛球,等等,散步
娱乐,篮球爱好者,music,双重性格,陈奕迅,孩子气,吃喝玩乐,交朋友,阳光
NBA,篮球,囧,美食,听歌,阅读,宅男,电影,玩儿,时尚
游戏动漫
时尚
体育,军事
游戏动漫,娱乐,体育,美食,汽车
忻州生活
名人明星,IT 数码
娱乐
健康,美食,文艺,校园,吃货
旅游
时尚,星座命理
时尚,健康,投资理财,文艺,热情,心理,财经,室内设计,哲学,欧美
萌宠
星座命理
时尚,美食
娱乐
处女座,美女,娱乐
健康,星座命理
旅游,娱乐,美女,健康,星座命理,美食
星座命理,游戏动漫,IT 数码,汽车,美食
微博奇葩
视频音乐,美女,新闻趣事,搞笑幽默
娱乐
文艺,教育就业,IT 数码
星座命理,军事
娱乐,美女,体育
江苏生活
江门生活,法国,bigbang,猫,化妆,星座命理
文艺,IT 数码,旅游,时尚,娱乐
读书分享
篮球
时尚,星座命理,美食,娱乐,健康
投资理财
教育就业
时尚,星座命理,美食
时尚,健康,旅游,娱乐,游戏动漫,体育,90 后但我不脑残,小说控、电影控,Music
美女

健康
IT 数码, 娱乐
文艺, 时尚, 健康, 美食, 星座命理
安徽生活, 投资理财, 体育, 天秤座, 游戏, 旅游, 电影, 睡觉
健康, 旅游, 美食, 美女, 汽车
美女, 体育
健康, 新闻资讯, 时尚, 汽车, 体育
汽车, 文艺, 娱乐, IT 数码
完美主义, 神经质, 低调
娱乐
旅游
IT 数码, 游戏动漫
猫咪, 敢达, 动漫
汽车
教育就业, 投资理财, 健康, 旅游, 美食
汽车
平常心, 睡觉, 旅行, 美食, 自由
时尚潮流, 阳光, 创意, 90, 爱音乐, 娱乐
娱乐, 美女
十字绣, 政券投资, 风险管理, 个人理财, 公司信贷, 个人代款, 星座, 女士真皮包, 爱尚百变包包屋, 会计
娱乐, 体育
汽车
娱乐
娱乐
搞笑幽默, 学生, 开朗, 玩, 爱音乐
体育
星座命理, 美食
医学检验
娱乐, 星座命理, 美女, 汽车, 时尚
吃货一枚, 漫画, 活泼, 学生一族, 爱睡觉, 爱生活, 乐观, 搞笑, 摩羯座
新闻资讯, 投资理财, 美食, 旅游, 体育
微博奇葩, 搞笑幽默, 成都生活, 美食
投资理财
娱乐, 时尚
旅行, 安静
旅游
时尚, IT 数码, 星座命理, 旅游, 娱乐
娱乐, 投资理财, 旅游, 星座命理, 美食
视频音乐, 电影
文艺
游戏动漫, 星座命理
汽车, 投资理财, 美女
IT 数码, 美食, 投资理财, 娱乐

游戏动漫,文艺,娱乐,体育
凤游龙门,文艺,微笑,演员,电视剧
娱乐,健康,美食,旅游
游戏动漫
韩国生活,娱乐
教育就业
旅游
时尚,娱乐,星座命理,旅游,健康
穿衣美容
教育就业
美食,健康,娱乐
搞笑幽默
萌宠
IT 数码,旅游,教育就业,汽车,新闻资讯
旅游,教育就业,健康,新闻资讯,美食
呆瓜石头石头,石头
健康,娱乐,宅,80 后,教育就业
视频音乐
旅游
娱乐
狮子座,90 后
娱乐
腹黑,陈奕迅,咖啡,夜猫子,绘画,星座控,小说迷,猫,设计师,娱乐
游戏动漫
健康,旅游,美食,军事,游戏动漫
美食,娱乐,水瓶座加 O 型血
穿衣美容,微博奇葩,萌宠,星座命理,搞笑幽默,怀旧,独立
军事
娱乐
游戏动漫,IT 数码,汽车,健康,体育
教育就业,健康,星座命理,时尚,文艺,娱乐
文艺
军事
美女
文艺
旅游
娱乐,文艺,时尚,健康,星座命理
星座命理
k 歌,手機控,乐观
文艺,美食,旅游,健康,娱乐
娱乐,军事,美食,时尚,教育就业,健康,旅游
旅游,美食,爱电影,囧,懒人
娱乐
旅游

河南生活
电影迷,听歌,90 后,疯狂,花痴,吸血鬼日记,文艺,时尚,娱乐,美食
娱乐,体育,NBA,生活,时尚
雀雪莉,sulli
IT 数码,娱乐
美食,职场招聘,爬山,笑话
旅行,90 后,交友,音乐,运动,天秤座,摄影,自由,生活,小说
健康
美食,游戏动漫
80 后创业者
时尚,美女,美食,娱乐
微博奇葩,山东生活,娱乐
新闻资讯,健康,时尚,娱乐,文艺
文艺
游戏动漫,文艺
美女,新闻资讯,健康,娱乐,教育就业,军事,体育
多愁善感,购物狂重症患,看电影,睡懒觉,选择综合症,纠结体
健康
老法师,净土,愿解如来真实义,慈悲,因果
美食,文艺,旅游,健康,游戏动漫
穿衣美容
时尚,旅游,美食,健康,娱乐
娱乐
购物,萝卜价
搞笑幽默,游戏动漫
旅游
美食,新闻趣事,视频音乐,穿衣美容,搞笑幽默
旅游,时尚,美食,娱乐,文艺
新闻资讯,旅游,娱乐
星座命理,美食,旅游
体育,自由,好性格,运动,听歌,上网,交友,看书,幽默,90 后
乐活族,手机控,美甲控,技术宅,起床困难户,单身懒散女青年,减肥 ing,减肥永不成功党,人格分裂,少女时代 sone
旅游,娱乐
旅游,美食,娱乐
IT 数码,游戏动漫
酷爱玩丢丢,麻麻的小怂包,双子男,尿王,大水桶,想尝尝骨头,最爱狗粮,小正太
美食
娱乐,时尚,旅游,美食,文艺
美女,IT 数码,娱乐,游戏动漫,星座命理
犯 2，思维跳跃，
美图摄影
娱乐,旅游
吃货一枚,睡懒觉,射手女

美女,汽车,时尚,IT 数码
宅女加腐女,吃货一枚
小说,音乐,游戏动漫,旅游
教育就业,新闻资讯,美食,时尚,娱乐,投资理财,汽车,摩羯女,星座命理
穿衣美容
美女,娱乐
文艺,美女,健康,新闻资讯,投资理财,星座命理,网购商城
体育,教育就业
文艺,体育
旅游,新闻资讯
美食,双重性格,善良,摩羯座,懒
囧
旅游,投资理财,美食,星座命理,汽车,文艺
天秤,体育
旅游,文艺
涂鸦
美食,旅游,汽车,娱乐,时尚
美食
健康
投资理财,健康,教育就业,文艺,新闻资讯,时尚
北京生活
IT 数码
娱乐
车世界
美女
美食,健康,文艺,时尚,娱乐
时尚,美鞋控
军事,时尚,投资理财,IT 数码,娱乐
健康,旅游
教育就业
美食,娱乐
星座命理
娱乐,投资理财,教育就业,健康,美食
美食
教育就业,投资理财,新闻资讯,游戏动漫,IT 数码
娱乐
旅游,军事,文艺,娱乐
旅游
星座命理
浙江生活
穿衣美容,时尚
IT 数码
教育就业,新闻资讯,健康,文艺
时尚,新闻资讯

潮州生活,梦想
思考,人力资源,交朋友,旅游美食爱好者,摄影师,心理,电影爱好者,语录,足球达人,天文爱好者
钱串儿,乱七八糟的思想,喜欢妖孽男,爱狗成痴,非标准 90 后,偶稀饭睡觉觉
旅游,新闻资讯,汽车,星座命理,军事,IT 数码,文艺
文艺,时尚,健康,旅游,美食,娱乐
娱乐
其他生活
汽车
娱乐
文艺,新闻资讯
穿衣美容
教育就业,时尚,游戏动漫
旅游
时尚,IT 数码,旅游,新闻资讯,文艺
美容服饰,情感生活,八卦杂谈,新闻趣事,名人明星
旅游,美剧控,IT 数码,美食
搞笑幽默,视频音乐,河南生活,美食,星座命理,喜欢文字
旅游,文艺,爱情,文字,看书,旅行,写作,生活,自由
美食
IT 数码
体育
游戏动漫,星座命理,娱乐,文艺,开朗,八卦,美食,听音乐,星座,海贼王
娱乐
穿衣美容
汽车
娱乐,健康,新闻资讯,投资理财,教育就业,文艺,时尚
投资理财,新闻资讯,旅游
美食
星座命理
历史,玩儿,校园,听歌,奋斗 ing,时尚,唱歌,创业,乐观,90 后
军事,汽车,游戏动漫
投资理财,娱乐,新闻资讯,旅游,IT 数码
搞笑幽默
健康,旅游,星座命理,娱乐
星座命理,旅游,名人明星,搞笑幽默
搞笑幽默,育儿,处女座
游戏动漫,美食,旅游,健康,新闻资讯,时尚
旅游,美食,90 后,服装
游戏动漫,美女,新闻资讯,军事,娱乐
健康,游戏动漫,汽车,IT 数码,娱乐
游戏动漫,娱乐
微博奇葩,美女,新闻趣事,搞笑幽默
萌宠,狮子座,乐观,吃货,游戏,淡定,热爱生活,内向

星座命理,文艺
时尚,游戏动漫,投资理财,娱乐,星座命理,溜冰爱好者,唱歌,吃货,狮子女,化妆师一枚
娱乐,旅游,体育,美食,军事,IT 数码
名人明星,美女,视频音乐,河北生活,健康,汽车,娱乐,体育,外向，篮球，学生
娱乐,美女
教育就业
守护,快乐
经验丰富,学 C1,考证快,老教练,C1,和蔼
旅游,体育,投资理财,游戏动漫,健康,新闻资讯,星座命理,军事
旅游,IT 数码,美食,汽车,体育
穿衣美容
看书,旅游,文艺,设计,哲学,绘画,奋斗,户外,平常心
旅游,美食,文艺,游戏动漫,幽默,善良,学生,双子座
旅游,时尚,娱乐
时尚
健康,新闻资讯,美女,时尚,娱乐
时尚
IT 数码,外语学习,家庭保健,学习方法,亲子教育
新闻资讯
健康,美食,80 后,选择控,喜欢游泳,唱歌,处女座 A 型
游戏动漫
指导,培训,网店,淘宝
教育就业
娱乐
健康,新闻资讯,摩羯座,乒乓球
微博奇葩
时尚
武汉生活
新闻资讯,军事
健康,新聞資訊,軍事
美食,吃货,怪盗基德,旅游美食爱好者,巨蟹女,文艺,时尚,健康,旅游,娱乐
美食
美女,美食,文艺,娱乐,时尚,体育
旅游
泉州生活,微博奇葩,搞笑幽默
汽车
新闻资讯,健康
游戏动漫,健康,旅游,美食,IT 数码
狮子座,社会,搞怪,仙剑,游戏,吃,搞笑,汽车,时尚,娱乐
健康
最好的男科医院,专家,前列腺肿大,龟头炎,男科,早泄,前列腺,生殖系统,阳痿,前列腺炎
激情,军人,帅哥,我是 1,猛男,GAY,GV,肌肉控,短发控,军警
健康,新闻资讯,星座命理,时尚,IT 数码
娱乐,游戏动漫

时尚
幽默,好性格,双鱼座,广告,设计,电影,新闻,营销,大学生
文艺,军事,娱乐,健康,台球控
娱乐,星座命理
秋田犬
美食,健康,娱乐,吃货,网购,读书
健康,旅游
健康,游戏动漫,时尚,美食,微博控,漫画,80 後,旅游
音乐,时尚,复古,恋旧,偏执狂,坏女人
娱乐,外国语学院,美国,购物狂,粤语,音乐达人,康熙来了,美容护肤,爱电影
美食,爱猫,手机控,金牛座
搞笑幽默
文化,忧郁,医学,茶,旅游
文艺
健康
美女,时尚,军事
娱乐
娱乐
娱乐,新闻资讯,汽车,健康,美女
星座命理,偏执狂,爱美食,暮光之城,爱旅行,花痴
汽车,IT 数码,新闻资讯,教育就业,投资理财
新闻资讯,体育,游戏动漫,美食
美女
旅游,新闻资讯,娱乐
打球,爱自由,移动互联网,电脑,wow,感性
娱乐,汽车,文艺,健康,体育
新闻资讯,健康,教育就业,美食,投资理财,娱乐
汽车,IT 数码
名人明星,搞笑幽默,视频音乐,旅游,穿衣美容
创意,孩子气,娱乐,时尚,星座命理,旅游,文艺
体育,投资理财,新闻资讯,军事
名人明星
旅游
投资理财,军事,IT 数码,时尚,新闻资讯,文艺,体育,娱乐,韩国,我喜欢一个人
星座命理,新闻资讯,文艺,时尚,旅游,美食
单身待解放,建筑,娱乐
投资理财,娱乐,旅游,IT 数码,美食,美女,时尚
体育
美食,新闻趣事,穿衣美容,旅游,萌宠
音乐，运动
教育就业,时尚,美女,文艺,投资理财,IT 数码,新闻资讯
旅游,汽车,美食,IT 数码,体育
娱乐
体育

读书分享,宠物图集,名人明星,内江生活
宠物图集,星座运势,名人明星,其他生活,情感生活
娱乐
校园生活,90 后但我不脑残,自由,学生,搞笑,娱乐
搞笑幽默,娱乐,旅游
郑州生活
娱乐,华语第一天团,天籁传奇,2012,最炫民族风,凤凰传奇,太原演唱会
娱乐
杭州,旅游爱好者,古典音乐,摄影爱好者,手机
美食,旅游
时尚,金牛座宅女,爱吃,快乐大本营,看电视,喜欢听歌,活泼,微博控,单身,睡懒觉
新闻资讯,营销咨询,财经,营销理念,市场定位,品牌策划,营销策划,销售与市场,营销,职业经理人
文艺,星座命理
健康
娱乐
音乐控,搞笑,游戏,85 后,足球,奋斗,新闻资讯,文艺,游戏动漫,娱乐
文艺,游戏动漫
投资理财,美食,健康,文艺
80 后
时尚,旅游
IT 数码,新闻资讯,投资理财,旅游,健康,美食
IT 数码,文艺,美食,新闻资讯,时尚,汽车,娱乐
星座命理,美食,IT 数码,军事,娱乐
游戏动漫,文艺
娱乐,。
游戏动漫,汽车,娱乐,IT 数码,旅游,电影迷,音乐发烧友
娱乐,健康
旅游,美女,IT 数码
教育就业
美食,微博奇葩,视频音乐,听歌,生活,五月天,NBA,休闲
梦想,情感
健康,旅游,美女
美女
美容服饰,旅游,美图摄影,视频音乐,美食
体育,教育就业,时尚,健康,旅游
新闻趣事
健康,星座命理,美食,时尚
体育
相声,爱笑,咖啡控,狮子座,吃货
军事,美食
教育就业
投资理财,美食,IT 数码,时尚,健康
旅游,美食,视频音乐,娱乐

护肤品店主
游戏动漫
教育就业,娱乐,新闻资讯,游戏动漫,美女,美食,IT 数码
健康,美食,旅游,文艺,娱乐
新闻资讯,游戏动漫,IT 数码,体育,美女
IT 数码,美女,游戏动漫,娱乐,内向,Music,学生一枚,玩,搞怪,幻想
娱乐
美女,军事,IT 数码,投资理财,新闻资讯
IT 数码,旅游,娱乐,美女
文艺
时尚,娱乐,健康,美食,90 後,活泼,爱生活,多愁善感
军事,IT 数码,文艺,娱乐,时尚,新闻资讯
健康,旅游,投资理财,军事,美食
星座命理
美食,爱幻想,看小说
外貌协会,封印终结者,星之所在,永远的一只信蜂
体育
美食,星座命理,健康,投资理财,体育
设计师,绘画,服饰,宅,听歌,时尚,星座命理,娱乐
娱乐,旅游,投资理财,IT 数码
武术,双节棍,双节棍爱好者
文艺
文艺,体育
IT 数码
旅游,健康
美女,体育
阳光,舞蹈,爱音乐,校园,我就是我,旅游美食爱好者,宠物
美食,健康
时尚,娱乐
摄影,阳光,王菲,思考,驴友,旅游摄影,朝鲜,古典艺术,自行车,旅行
美女
时尚,星座命理,体育,娱乐,爱笑
星座命理,旅游
体育
吃货一枚,天蝎
视频音乐
IT 数码
军事
星座运势
星座命理,教育就业
投资理财,星座命理,健康,汽车,教育就业
教育就业,投资理财,军事,汽车,IT 数码
娱乐
文艺,健康,游戏动漫,星座命理,教育就业

娱乐
PaaS,SaaS,云计算,服务管理,资产管理,设施管理,智慧地球,智慧城市,智慧建筑
娱乐
出差,销售行业
旅游,美食,投资理财,娱乐
娱乐
时尚,娱乐
星座命理
健康,旅游,军事,IT 数码,体育
逛街,快乐,互粉,偶像剧,喜欢唱歌,K 歌,双子,开朗,感性,潮流
IT 数码,军事
美图摄影
健康,至少我还在努力,金牛,任性,爱睡觉,阳光,星座命理
时尚,汽车,张玮控,交朋友,运动,喜欢听歌
娱乐
健康,旅游,美食,爱睡觉,减肥
星座命理,时尚,旅游,美食,文艺
文艺,尝鲜族,K 歌小王,天枰座
视频音乐
星座命理,文艺
盗墓笔记,O 型血,90 后,双重性格
汽车,文艺,健康,投资理财,娱乐
健康
健康
健康
美食
开心,爱,喜欢音乐
微博奇葩,旅游,美食
四川生活,娱乐
熊交友,单身熊,找不婚熊,福州中熊,长久熊,找熊中,胖熊,福州熊
名人明星
投资理财
美女
时尚,文艺,汽车,健康,新闻资讯
旅游,汽车,美女
爱宠一族,宅人加懒人一枚,甜点控,摩羯座 B 型血,法律服务,法律咨询,法律人,健康,星座命理
体育,时尚,美食
名人明星
星座命理
新闻资讯,投资理财,汽车,娱乐,旅游
旅游
美女
汽车,健康,文艺,体育

健康,爱吃,旅游爱好者,独立,shopping,学生一族,红楼梦,星座命理,团购,动漫迷
星座命理
旅游
天蝎男
旅游,娱乐
美女
汽车
美食
军事,健康,IT 数码,文艺,娱乐
时尚,汽车,新闻资讯,娱乐,旅游
美女
自然,热爱生活,家居,创意,阳光,爱生活,广州,花卉,花卉园艺,环境艺术
旅游,星座命理,娱乐,健康,美食
健康,旅游
IT 数码,旅游,时尚,投资理财,健康,新闻资讯,娱乐,汽车
新闻资讯
军事,美女,娱乐,IT 数码,健康,旅游,体育
娱乐
爱音乐,摄影,健康,旅游,美食
体育,文艺,游戏动漫,IT 数码,美女,娱乐
新闻趣事
斯琴高娃,羊胎素注射,活细胞,瑞氏国际,CSH,瑞士健康中心,瑞士羊胎素,羊胎素
柯南,学生,旅行,Music,星座命理,健康,美食,IT 数码,文艺
时尚,美食,旅游,娱乐,文艺
健康,时尚
美食,军事,IT 数码,文艺
游戏动漫,美食,星座命理,新闻资讯,时尚,旅游
汽车,旅游,时尚,投资理财,娱乐
IT 数码,美女
汽车,旅游,军事,保钓人士,钓鱼岛,保钓,上网,80 后
美女,体育
新闻资讯
游戏动漫
花样美男,新闻趣事,搞笑幽默
娱乐
美食,美女,汽车,IT 数码,游戏动漫
旅游,汽车
美女,时尚,健康,旅游,新闻资讯
爱玩,聊天,简单,感性,时尚,美食,星座命理,娱乐
萌宠,名人明星,水瓶座,90 后,校园
文艺,音乐
生活,网购,旅游,投资理财,健康,娱乐
国米,大漩涡,新闻资讯,旅游,体育
教育,阅读,图书租赁,私人专享,电话英语,早教,漯河

文艺,新闻资讯,旅游,美食,时尚
体育,逛街,网球,篮球,运动,汽车,网购,sneaker 文化,潮流文化,服装设计
旅游,体育
美食
娱乐
体育
汽车
娱乐,教育就业,健康,星座命理
体育
福建生活
萌宠,嘉兴生活,看书,爱睡觉,爱侦探悬疑小说,学生,90 后
神经大条,b 型血,独立,巧克力,敏感,纠结,简单
穿衣美容
文艺
旅游,军事,教育就业,汽车,新闻资讯
诚信为本
视频音乐
穿衣美容
娱乐
游戏动漫,旅游,IT 数码,美食,娱乐
星座命理,游戏动漫,健康
星座命理,娱乐
时尚
新闻资讯,健康,旅游,文艺,娱乐
娱乐,汽车
娱乐
搞笑幽默,朋友
旅游
独立,善良,摩羯座,阅读,读书,时尚,星座命理,新闻资讯
健康
旅游,教育就业
时尚,投资理财,健康,新闻资讯,美食
时尚,新闻资讯,健康,旅游,星座命理
体育
文艺,教育就业,旅游,美食,时尚
星座命理,娱乐,舞蹈
IT 数码
娱乐,旅游,美食,星座命理
杂志,营销,书籍,孩子气,90 后,音乐控,听歌,散步,运动,内向中的外向
美食
星座命理
旅游,美食,娱乐,神经控,小任性、小情绪
投资理财,时尚,美食,健康,旅游
娱乐,旅游,游戏动漫,美食,文艺

文艺,投资理财,汽车,新闻资讯,旅游
新闻趣事,搞笑幽默
旅游,健康
IT 数码,美食,健康,旅游
文艺,健康,旅游,美食
體育,遊戲動漫,文藝,汽車
时尚,美女,星座命理,娱乐
娱乐
IT 数码
美食
新闻资讯,旅游,星座命理,娱乐,文艺
神经质,腐,爱电影,美食
汽车,旅游,美食,IT 数码,体育
星座命理,美食
时尚,健康,旅游,美食
旅游,美食,星座命理,文艺,娱乐
懒,新闻资讯
视频音乐,新闻趣事,搞笑幽默,山西生活,穿衣美容
美食
星座命理
视频音乐,穿衣美容,旅游,美食,云南生活
美食
娱乐
IT 数码
娱乐
旅游
新闻资讯,旅游,美食,文艺,娱乐
健康,旅游,时尚,星座命理,娱乐
IT 数码
视频音乐,搞笑幽默,IT 数码,名人明星,八卦杂谈
移动互联网,安卓开发
新闻资讯,美食,投资理财,教育就业,旅游,娱乐
美女
文艺
健康
旅游
美食
娱乐,健康,美食,星座命理,旅游
健康
美女
娱乐
旅游,汽车,星座命理,美食
娱乐,电影,音乐,时尚
娱乐

初级达人,健康,旅游,美食,娱乐
IT 数码,投资理财,教育就业,美女,体育
视频音乐,时尚,体育,幽默,，,暗淡，,运动
IT 数码,读书分享,美食,美容服饰,成都生活
娱乐
幽默,文学,睡觉,动漫,电影
独立,青春,个性,瘦身,美容护肤,微博奇葩,旅游,穿衣美容,搞笑幽默,江苏生活
新闻资讯,文艺,静书
美女,搞笑幽默,汽车
文艺,瓶邪党,宅腐,山殿
新闻趣事,新闻资讯
美食
上网,唱歌,自由,感性
教育就业,新闻资讯,健康,旅游,娱乐
时尚
教育就业
星座命理,美食,IT 数码,娱乐
旅游,星座命理
投资理财,教育就业
体育,游戏动漫
画画,摄影,宅女,个性,学生,时尚,听音乐,懒,吃货一枚减肥中,拍照
投资理财,时尚
投资理财,2B,单纯,小清新,文艺
娱乐,健康,新闻资讯,文艺,星座命理
时尚
搞笑幽默
娱乐,旅游,文艺
美,美逼,大美逼,大大美逼,大大大美逼,大大大大美逼,大大大大大美逼,结巴
旅游,美食
时尚,娱乐,旅游,美食,文艺
美食
星座命理
文艺
旅游,美食,娱乐,美女,体育,汽车
游戏动漫
旅游
美食,爱电影,学生,90 後,吃货一枚,双鱼女
旅游,生活,Music,k 歌,香港,社会,历史,粤语,游戏
视频音乐,新闻趣事,旅游,福州生活,搞笑幽默
喜欢听歌,乐观,射手座,唱歌,陈翔,听音乐,香橙,青春,吉他,时尚
星座命理
娱乐,文艺,星座命理,美食,体育
星座命理
投资理财,教育就业,时尚,健康,旅游

健康,旅游,投资理财,美女,新闻资讯,星座命理
星座命理
汽车,军事,IT 数码,体育
健康,美女
许昌生活,河南生活
美女,游戏动漫,军事
娱乐
娱乐
健康
娱乐,星座命理,张根硕
B 型血,八卦,学生一族,旅游美食爱好者
搞笑幽默,IT 数码,娱乐,体育
健康,80 后,巨蟹座,美食控,淘宝控,寂寞,经济,幸福,真实力
军事
文艺,睡懒觉,狮子女,动漫迷,90 后非脑残
k 歌,游戏动漫,天蝎,高中生
旅游,蒙古海军总司令,世界级好人认证,变态萝莉控,FFF 团,AKB 饭,东方 project
新闻资讯,星座命理,军事,体育,美女
娱乐
学生一枚,瑜伽,懒,做梦,唱歌,旅行,摄影,音乐控,减肥,逛街
旅游,美女,文艺,爱幻想,狗狗,真诚,科比,海贼王,喜欢唱歌,简单
西水,汉森服务,锦湖,会员,健康咨询
微博奇葩
投资理财
生活,开心
搞笑幽默,娱乐
IT 数码,旅游
体育
美食,旅游,吃货
爱睡觉,看电影,历史,美术,天蝎女,设计,时装,美容护肤,美食
美食
时尚
投资理财
幽默,电影,音乐,IT 数码,IT 互联网
娱乐,宅女,乐观,偶喜欢玩,上网,90 后,听歌
娱乐
星座命理,美食,文艺,娱乐
腐,东方神起,旅游,美食,星座命理,时尚,游戏动漫
军事
时尚,新闻资讯,星座命理,美食,健康
游戏动漫,时尚,投资理财,文艺,军事
旅游,四川生活
萌宠
娱乐

足球,社团,运动
新闻资讯,投资理财,美食,旅游,美女,健康
旅游
健康
旅游
新闻资讯,健康
时尚,美食,旅游,文艺
电影,听歌,双子座
星座命理,娱乐
娱乐
孙燕姿,TaylorSwift,双重性格,外貌协会,学生一枚,完美主义,网球,时尚
幽默,看书,唱歌,文艺,开朗,军事,投资理财,健康,美女
名人明星
娱乐
时尚,汽车,军事,美食,文艺,双重性格,梦想,90后但我不脑残,欧美音乐
时尚,旅游
文艺
美女,IT数码,文艺,体育,游戏动漫,时尚,美食,健康,新闻资讯,娱乐
美食,美女,投资理财,旅游,星座命理,文艺,娱乐,健康,新闻资讯,体育
文艺,旅游,美食,时尚,游戏动漫
教育就业
新闻资讯
美女,投资理财,旅游,星座命理,美食,健康,娱乐,IT数码,文艺,体育
文艺
时尚,星座命理,旅游,美食,娱乐
逛街,开朗,爱电影,低调
旅游,星座命理,美食,美女,军事,文艺,体育,健康,新闻资讯,娱乐
娱乐
游戏动漫,娱乐,星座命理
美食
IT数码,娱乐
游戏动漫,新闻资讯,旅游,健康,体育,娱乐,投资理财,时尚,汽车,教育就业
时尚,投资理财,星座命理,文艺,教育就业
星座命理
需找称职的工作
旅游,健康,美食
学生一族,狗狗,外貌协会,潮,白羊座,自行车,欧美音乐,我就是我,电视剧,爱睡觉
新闻资讯,游戏动漫,旅游
投资理财,健康,时尚,文艺,娱乐
散步,平常心,处女座,爱生活,旅游,123,情绪化
搞笑幽默
娱乐
美食,IT数码,上班族
娱乐,教育就业,健康,旅游

娱乐,美女,游戏动漫,汽车
新闻趣事,萌宠,穿衣美容,星座命理
美女
美女,旅游,星座命理,美食,健康,体育,娱乐,军事,IT 数码,文艺
旅游,健康
美女,旅游,体育,汽车,IT 数码
游戏动漫,时尚,汽车,娱乐,健康,文艺,体育
控制系统服务,异形灯设计,酒店照明设计,照明管理顾问,照明系统服务,资深照明设计师
星座命理,旅游,健康,文艺,娱乐
文艺,投资理财,健康,旅游,美食
美女,旅游,美食,娱乐
时尚,文艺,汽车
旅游,潮人,喜欢听歌,吃喝玩乐,射手座,巨蟹座,大学生,游戏动漫
文化,完美主义
创业
健康,文艺,娱乐
IT 数码
美食
旅游
银狐
时尚
旅游,四川生活
军事,新闻资讯
宠物图集,美图摄影,美食,摄影,天秤,旅游美食爱好者,爱生活,90
文艺,时尚,旅游,娱乐
娱乐
欧美音乐,爱音乐,无厘头,腹黑,小说迷,英语,网络,娱乐,文艺,美食
IT 数码
游戏动漫
旅游,娱乐,体育,文艺,IT 数码
浙江生活,美食,旅游,搞笑幽默,穿衣美容
投资理财
常州生活,娱乐,生活,运动,热点,购物,时尚,摩羯座,交友
星座命理
包子,度白,SHINee,开泰,开白,克里斯,shinhwa,郑允浩
北京生活,搞笑幽默
旅游
腐女,上网,娱乐,学生,音乐,动漫,东方神起,春春
旅游,健康
投资理财
旅游
投资理财,娱乐,文艺,教育就业,健康
穿衣美容
时尚

搞笑幽默
星座命理,旅游,美食,学生仔,時尚,狮子座,时尚
历史,美女,爱情,旅游,自由,摄影爱好者,天蝎座,音乐,写作,语录
军事,财经,新闻,市场营销,网购,娱乐,旅游,新闻资讯,美女,时尚
旅游
凤凰广电系统,旅游,文艺
美食
旅游,文艺,新闻资讯,教育就业,投资理财
娱乐,星座命理,健康,时尚
IT 数码
新闻趣事
教育就业,旅游,文艺,美食
矛盾,懒,爱吃,宅女一枚,游戏动漫,教育就业,健康,美食,投资理财
星座命理
星座命理
娱乐
美食
美食
文艺,沉默,隐忍
健康,美食,旅游,时尚,投资理财
娱乐,时尚,旅游,星座命理,美食
娱乐
郑允浩,银魂控,土银党,腐女,cosplay,東方神起,摩羯座,瓶邪,美食,游戏动漫
娱乐,美食,搞笑,学生,旅游,舞蹈,时尚
美女
旅游
旅游
唱歌
美食,旅游,星座命理,健康
体育
新闻资讯,汽车,投资理财,军事,IT 数码
娱乐,星座命理,文艺,宅女加腐女,盗墓笔记
教育就业,星座命理,健康
美女,军事
健康
文艺,娱乐
IT 数码,美女
健康
美女,文艺,娱乐,军事
听音乐,海贼王,旅游美食,吃货一枚,减肥,舞蹈,胡思乱想,文艺,体育,投资理财
新闻资讯,风格各异,大搜罗,权威,生活记录,家居设计,家具品牌
汽车
娱乐,体育
旅游,文字,发呆

八卦杂谈,名人明星,娱乐
美女,游戏动漫,旅游,星座命理,美食,娱乐
休闲,会计
健康,旅游,星座命理,时尚,娱乐,旅游摄影,古典艺术,狗狗,双子座
IT 数码,新闻资讯,健康,教育就业
宅男,80 后,旅行,文艺,美女,健康
星座命理
汽车,90 後,爱旅游,旅游,教育就业
搞笑幽默,学生一族,养生,投资理财,新闻资讯,登山,魔羯座,跑步
娱乐
娱乐,美女,健康,文艺,体育
轻度强迫症,巨蟹,纲丝,辣妈,美甲控,80 后
娱乐,教育就业,游戏动漫,美食
减肥,海绵宝宝
旅游,电影爱好者,旅游美食爱好者,购物
文艺,IT 数码,星座命理,旅游,健康,娱乐
新闻资讯,真诚热情,专业,顶级公司,家政服务,教育就业
文艺,娱乐
投资理财,旅游,健康,汽车,教育就业
新闻资讯,教育就业
投资理财
小说,乐天派,80 后的尾巴,处女座,偶稀饭睡觉觉,时尚,健康,旅游,美食,娱乐
健康,娱乐,文艺,美食
篮球,摄影,植物爱好者
小说,看书,睡觉,爱旅行,吃货,阅读,k 歌,喜欢音乐
健康,美女,汽车,体育,娱乐
美食,旅游,星座命理
美食,小说,唱歌,听歌,吃,90 后,偶喜欢玩,偶稀饭睡觉觉,校园,娱乐
阳光,乐观,射手,阳光， 乐天派
娱乐
美食,美食
美食
桌球,手机
新闻资讯,教育就业
美食,军事,文艺,金牛座,平常心,摄影,文字,编辑
汽车
娱乐
游戏动漫
河南生活,新闻趣事,微博奇葩,奋斗,潜力股
教育就业
健康,旅游,星座命理,文艺,娱乐
文艺,教育就业,健康
旅游！,90 後
文艺

其他生活
美食
健康,新闻资讯,时尚,汽车,旅游
娱乐
美女
娱乐
低调的愤青,TVB 控,夜猫子愈夜愈欢,摄影旅游,强迫症倾向,双重人格未分裂,流浪的心,穷游背包客,淘宝掌柜,营销菜鸟
游戏动漫,教育就业,IT 数码
玩儿,时尚,文字
体育
户外,销售,驴友,F1,旅游,汽车
旅游,新闻资讯,游戏动漫,汽车,IT 数码
穿衣美容,南京生活,视频音乐,新闻趣事,微博奇葩,健康,投资理财,教育就业,娱乐
IT 数码
旅游,时尚,健康,星座命理,美食
旅游,健康,投资理财,天蝎座 80 后,会计
旅游
B 型血
星座运势,陈奕迅
旅游
搞笑幽默
健康,体育
投资理财
娱乐
投资理财,文艺
IT 数码,军事
搞笑幽默,视频音乐,美食,旅游,健康,时尚
星座命理,美食,IT 数码,游戏动漫
爱生活,70 后,女人,小资,宅女
美食
美食
娱乐
旅游
军事
奢侈品,设计师品牌,代购,时尚,买手
新闻资讯,健康,旅游,美食,文艺
娱乐
IT 数码,教育就业,旅游,星座命理
投资理财
汽车
新闻资讯
星座命理
新闻资讯

军事
旅游
健康
小清新,幸福,爱自由,玩,安静,快乐,活泼,互粉,微博控,宅女一枚
新闻资讯,时尚,旅游,美食,健康
新闻趣事,旅游,搞笑幽默
云南生活,美食
旅游,健康,时尚,投资理财
旅游
时尚,健康,旅游,星座命理,文艺
快乐,开心
娱乐
星座命理
时尚,游戏动漫
旅游
旅游,娱乐,健康,星座命理,奋斗的小豆子,水瓶座
投资理财
爱宇宙,JYJ,动漫,摄影爱好者,F（X）函数,森系
娱乐
天枰座,人生,喜欢音乐,教育就业,投资理财,新闻资讯,旅游,IT 数码
时尚
旅游,名人明星,星座命理,天津生活,穿衣美容
游戏动漫,美食,旅游
汽车
健康,星座命理,蓝色,星座,听音乐,苏打绿,魏晨
好书,看书,篮球,科幻,完美主义,汽车,文艺,体育
旅游,爱音乐,感性,宅男,好书,音乐
投资理财,旅游,星座命理,健康,新闻资讯,体育
星座命理,文艺,娱乐,红楼梦,胡思乱想,80 後
上海生活,IT 数码
美女
新闻资讯,健康,旅游,IT 数码,文艺
新闻趣事
新闻趣事
美剧迷
美女,体育
旅游
文艺
体育
军事
娱乐,美食,美女,投资理财,健康
教育就业,体育,星座命理,新闻资讯,军事
平常心,羽毛球,游戏,幽默,浪漫,好性格,自由,自由职业,娱乐,美食
旅游

娱乐,旅游
娱乐
军事
旅游,时尚,文艺,星座命理,娱乐
爱嘚吧,爱国学,咖啡机,咖啡,网购,吃,乐观,吃货
旅游,健康
美食,旅游,文艺,军事
星座命理,时尚,新闻资讯,投资理财,娱乐
k 歌,爱美食,河南生活,吃货一枚,外贸,自拍,任性,懒人,淘宝,购物
娱乐,时尚
文艺
汽车,游戏动漫,新闻资讯,旅游,IT 数码
旅游,处女座,吃,睡觉,宅女,80 后
时尚,法律,驴友,旅游
美食
军事,美食,新闻资讯,旅游,健康
iphone,懒人,幻想,学习,活泼,看电影,爱音乐,喜欢唱歌,双子座,娱乐
矛盾体,我就是我,金牛,蓝色,汽车
搞笑幽默,星座命理
游戏动漫,星座命理,旅游,美食,娱乐
宅,追星
搞笑幽默,乐观,细心,领导力,大学,电影控,爱旅行,上网,唱歌,文艺
美剧,大学生,运动,篮球,看书,双鱼
文艺,美女,军事,IT 数码,体育,娱乐
健康,IT 数码,教育就业,美食
视频音乐
旅游
时尚
星座命理,懒,健身,学生一族,恐怖片,编辑
遊戲動漫,星座命理
读书分享
娱乐
体育
穿衣美容,旅游,健康
美图摄影,IT 数码,美女,旅游,体育资讯
NBA,囧,体育
美食
绘画,射手座,欧美音乐,电影爱好者,设计师,动漫迷,陈奕迅,时尚,文艺,娱乐
搞笑幽默
娱乐
新闻资讯,娱乐,文艺,IT 数码,军事
健康,旅游,星座命理,娱乐
健康
娱乐

教育就业,健康,旅游
河北生活,游戏动漫,体育,娱乐
娱乐
新闻资讯,健康,旅游,美食,娱乐
美食,新闻资讯,教育就业,旅游,健康,投资理财
八卦杂谈,读书分享,名人明星,校园生活
新闻资讯
星座命理,娱乐
娱乐
穿衣美容,视频音乐
星座命理,健康,文艺,娱乐
IT 数码,星座命理,旅游
美女,时尚,游戏动漫
新闻趣事
投资理财,时尚,星座命理,新闻资讯,美食,文艺
文艺,娱乐
健康,娱乐,星座命理
星座命理
搞笑幽默
新闻资讯,旅游,星座命理,美食,文艺
萌宠,微博奇葩,新闻资讯,健康,文艺,游戏动漫
美女,体育,游戏动漫,军事
健康
GDBABY,BIGBANG
IT 数码,健康
搞笑,热爱生活,学生,互联网,囧,摩羯座,90 后非脑残,幸福,交朋友,80 后的尾巴
随性
IT 数码
娱乐,新闻资讯,旅游,美食,星座命理,时尚
美女
旅游,新闻资讯,爱唱歌,乐观,狮子座,奋斗,活泼,听音乐,唱歌,大学生
文艺
汽车,军事,体育
教育就业,旅游
海绵宝宝,内向,大叔控,快乐大本营,胡思乱想,购物狂,吃,建筑,奋斗,狮子女
娱乐
微博,游戏动漫,小清新
游戏动漫
营销,爱生活,销售,化妆品,中医,淘宝,美食,健康,文艺,时尚
娱乐
健康,旅游,美食
体育,教育就业,星座命理,健康,娱乐
时尚,IT 数码
健康,教育就业,旅游,美食

美女,IT 数码,文艺,体育,娱乐
教育就业,健康,演讲,励志
IT 数码,文艺
IT 数码,美女,时尚,汽车,娱乐
娱乐
星座命理,偶喜欢玩,大学生,上网
游戏动漫,美食,时尚,汽车,娱乐
IT 数码
健康
旅游
美食
新闻资讯,游戏动漫,美食
新闻趣事,资质,招标造价,动漫迷,人力资源,工程管理
娱乐
懒洋洋,旅游,健康,娱乐,美食,投资理财
投资理财,娱乐
我还要努力
旅游,美食,星座命理,穿衣美容,视频音乐
微博奇葩
证券,影视,爱吃,旅游爱好者,幸福,投资理财,新闻资讯,旅游,美食,娱乐
饭行星,李弘基,金钟仁,EXO
美女,投资理财,美食,文艺,体育
健康,时尚,汽车,旅游,体育
旅游, 快乐
情感生活,校园生活,新闻趣事,搞笑幽默,旅游,健康,美女,娱乐,时尚
嘿嘿,呵呵,哈哈,女的,矛盾体,双鱼女,紫色,o 型血,纠结,爱幻想
娱乐
娱乐,健康
旅游,旅行,听音乐,疯子,爱自由,法国,爱生活,网络,摄影爱好者,时尚
新闻资讯,美食,文艺,投资理财,旅游,娱乐,健康,时尚,军事,美食
名人明星
内蒙古生活,IT 数码
投资理财,新闻资讯,教育就业,汽车,军事,文艺
独立,巧克力,瓶邪,吃货,技术宅,古风,剑网三,日本文学
云南生活,美女,视频音乐,摄影爱好者,NBA,新闻,娱乐,军事,汽车,文艺
名人明星,文艺,IT 数码,娱乐
投资理财,教育就业,新闻资讯,体育,IT 数码,娱乐,文艺
视频音乐,搞笑幽默,穿衣美容,时尚,健康,教育就业,娱乐,王力宏我的最爱,幽默,新浪读书
视频音乐,微博奇葩,搞笑幽默
新闻资讯,旅游,时尚,健康
新闻趣事
娱乐
美食,时尚
教育就业

文艺,健康,新闻资讯,时尚,美食,军事,娱乐,IT 数码
娱乐,汽车,体育,IT 数码,时尚,游戏动漫,旅游
IT 数码,娱乐,美女,游戏动漫,星座命理,文艺
校园生活,美女,教育就业
文艺
新闻资讯,美食,娱乐,健康,文艺
娱乐,美食
时尚
娱乐,游戏动漫,美食,文艺,时尚
投资理财,娱乐,时尚,旅游
文艺
体育
旅游,时尚,星座命理,美食,游戏动漫
旅游,娱乐
爱自由,偏执狂,爱情,青春,闷骚,神经质,美食控
校园生活
汽车,娱乐
时尚,星座命理,美食,游戏动漫,娱乐
美女,娱乐
幻想未来
时尚,文艺,美食,娱乐
旅游
美图摄影
新闻趣事
星座运势,动漫,美图摄影,美食,游戏动漫
美食,汽车,健康,时尚,旅游,IT 数码,娱乐
美食,星座命理,健康,时尚,娱乐
文艺,IT 数码,汽车,美女,投资理财,娱乐,体育
汽车,文艺,星座命理
校园生活,美图摄影,新闻趣事,搞笑幽默,美女
美食,新闻资讯,文艺
校园生活,潘朵拉之心
理财,美女,旅游,美食,穿衣美容
美女
健康
投资理财,星座命理,健康,时尚,教育就业,美食,旅游
时尚
IT 数码,投资理财,时尚,健康,旅游
健康,投资理财,新闻资讯,教育就业,文艺
教育就业,时尚,娱乐,旅游,美食
娱乐,文艺,旅游,星座命理,美食
娱乐
星座命理,旅游,教育就业
鸭子控,码字党,忻斯,摄影,音乐,动漫

文艺,投资理财,健康,时尚,游戏动漫,汽车,旅游,美食
喜欢听歌,IT 数码
IT 数码
教育,矛盾体,看书,娱乐
星座命理
美食,IT 数码,旅游,体育,娱乐
娱乐,旅游
汽车,时尚
旅游,美图摄影,搞笑幽默,视频音乐,湖州生活
健康
微博奇葩
眉山生活,娱乐
体育
旅游,时尚,文艺
娱乐,投资理财,美女,健康,旅游
文艺
美女,文艺,旅游
文艺
娱乐,IT 数码
日本,阳光,搞怪,微笑,淡定,热爱生活,时尚达人,潮人,驴友,Music
汽车,游戏动漫,美女
体育
健康,IT 数码,旅游,新闻资讯,投资理财
娱乐,旅游,美食
娱乐
时尚,教育就业,美食,健康,旅游
爱睡觉,军事,旅游,健康,娱乐,游戏动漫
旅游,视频音乐,感性理性游离间,随心,思考,看书,喜欢听歌
体育,IT 数码
时尚,旅游,美食
旅游
汽车,娱乐,投资理财,教育就业
佛教,体育
美食
美食,教育就业
游戏动漫,娱乐
健康,旅游
天外 enactus,空巢老人,公益
美食,IT 数码,娱乐,文艺
旅游,文艺,军事,美女
旅游
健康,新闻资讯,投资理财,旅游,军事,IT 数码
娱乐,游戏动漫,新闻资讯,旅游,美食,军事,IT 数码
其他生活

时尚,美女,美食,文艺
游戏动漫,旅游
视频音乐
军事
旅游,文艺,IT 数码,美食
美食,旅游,时尚,文艺
海绵宝宝,处女座
搞笑幽默,吃,交友,数码,语录,文学,书,家居,钓鱼,汽车
搞笑幽默,偶稀饭睡觉觉
旅游,文艺
萌宠,浪漫玛丽,张根硕
名人明星
搞笑幽默,IT 数码,文艺,娱乐,汽车,时尚
文艺,娱乐,健康,时尚
娱乐,游戏动漫,体育
文艺,星座命理,时尚,娱乐
名人明星,微博奇葩,新闻趣事,搞笑幽默,成都生活,新闻资讯,健康,时尚,娱乐
投资理财,教育就业,健康,美食,汽车,IT 数码
篮球,原创,幻想,时事,诗歌,柯南,游戏动漫,IT 数码,娱乐,体育
文字控
美女,美食,军事,IT 数码,文艺
健康,旅游,文艺,娱乐,美食,生化
简单,强迫症,狮子座,旅游美食爱好者,读书,文艺,健康,旅游,星座命理,美食
旅游,单身,怀旧,淡定,爱睡觉,乐天派
视频音乐
视频音乐,健康,美女,美食,旅游,娱乐
时尚,旅游
投资理财
军事
新闻资讯
沉默,文艺,奋斗,陈奕迅,吉他,简单,旅游,时尚,美食
旅游,时尚,美食,健康,娱乐
美女,游戏动漫,IT 数码,文艺,娱乐
时尚潮流控,旅游,k 歌,学生一枚
娱乐
90 后,爱旅游,爱拍照,爱音乐
搞笑幽默
英语作文批改,英语学习,作文批改,中考英语作文,高考英语作文,中招作文,英语作文,英语辅导
教育,读书分享,体育资讯,美女,搞笑幽默
搞笑幽默
汽车,文艺,旅游,星座命理,美食
旅游
教育就业,投资理财,汽车,美女,娱乐

时尚
健康,教育就业,军事
时尚,健康,旅游,娱乐,文艺
娱乐,新闻资讯,旅游,星座命理,健康,时尚
K 歌,摄影控,我就是我,旅游美食爱好者,打球,舞蹈,大学,汽车,时尚,娱乐
美食,美女,娱乐,旅游,文艺,音乐,想念,情感,美好
美食,健康,旅游,文艺,汽车
星座命理,健康,文艺,军事
汽车,时尚,旅游,IT 数码,体育
名人明星
美食
美食
穿衣美容,文艺,娱乐
投资理财,教育就业,健康,旅游,军事
美容服饰,微博,人生,工作,水瓶女,音乐达人,娱乐
生活大爆炸控
娱乐,美女,旅游,美食
星座命理,健康,代理菲琳,艾思德妮,欧诗蒂等品牌,合作 QQ,82270992,736558422
吴邪本命,文艺,杂食动物,墙头多,受控
游戏动漫,美食,IT 数码,娱乐,体育
娱乐
星座命理,时尚
穿衣美容
军事
星座命理,新闻资讯,时尚,美女,投资理财
免费 App,App 推送,手机游戏,Android,iPad,iOS,App,Apple,iPhone
google,娱乐
茉莉,音乐爱好者,日语,英语,韩语,狮子座,美食,敏感,完美主义,电影爱好者
旅游,时尚,文艺
美女,健康
读书分享,校园生活,美图摄影,美食,旅游,文艺,文学,四川美术学院,文学社
游戏动漫
旅游,穿衣美容
射手女,完美主义,shopping,乐观,music
时尚
教育就业,美女,星座命理,旅游,娱乐
花样美男,读书分享,新闻趣事,情感生活
娱乐,时尚,健康,文艺
旅游,睡懒觉
旅游,星座命理,游戏动漫,新闻资讯,美女,美食,军事,IT 数码
健康,旅游,文艺,体育,娱乐
体育,文艺,旅游
视频音乐,美女,美食,搞笑幽默,新闻趣事
星座命理,美食

旅游
美女,新闻资讯,天蝎座,吸血鬼日记,越狱,Eason,MAYDAY,海贼王
体育,汽车,游戏动漫,军事,IT 数码
时尚,美食,健康,文艺,娱乐
健康,美食,文艺,时尚,娱乐
星座命理,美食,健康,军事,体育
河北生活
旅游,健康
游戏动漫,体育,汽车,IT 数码
IT 数码,E,海贼王 ONEPIECE
娱乐,彩妆
北京生活
娱乐,美食
视频音乐
旅游,90 后,电影迷,大学,开朗,户外,网络,手机,青春,个性
会所,红木,瓷器,字画,红酒
微博奇葩,美食,穿衣美容,旅游,意大利生活
体育
cosplay,韩国,多愁善感,外貌协会,夜猫子,天蝎座
睡觉睡到自然醒,爱睡觉,巧克力,乐观,搞笑,天蝎座,交友,吃货
文艺
IT 数码,游戏动漫,星座命理,时尚,娱乐
旅游,新闻趣事,视频音乐,搞笑幽默,微博奇葩
汽车
奋斗
旅游
旅游,娱乐,时尚,星座命理
安徽生活
视频音乐,娱乐,情绪化
体育,汽车
健康,旅游,星座命理,IT 数码,美食
新闻资讯,IT 数码,娱乐,投资理财
娱乐,时尚,健康
旅游,IT 数码,投资理财,时尚,文艺
投资理财,健康,新闻资讯,时尚,汽车
美食
娱乐
体育
美女,游戏动漫,汽车,体育,无聊
体育
投资理财,健康,汽车,旅游,美食
汽车
文艺
视频音乐,搞笑幽默,星座运势,八卦杂谈,山西生活

旅游,娱乐
幻想　抽抽
开朗,美容护肤,苹果控,古灵精怪,金牛座,爱睡觉,健康
新闻资讯,健康,旅游,军事,IT 数码,美食
视频音乐,孩子气
旅游,重庆生活,视频音乐,名人明星,搞笑幽默
名人明星
娱乐
IT 数码
穿衣美容
美食,旅游,搞笑幽默,萌宠,美女
室内设计,陈列设计,软装设计
发呆 ing,幻想盲,双鱼,美食控,自由,情绪化,文学,旅游
高中生,穿衣美容,娱乐
美女,美食
名人明星
美女,汽车,投资理财,时尚,旅游,美食
旅游
美食
游戏动漫
旅游,星座运势,车世界,新闻趣事,情感生活
娱乐
健康,娱乐,旅游,美食
朴甜甜,美食,星座命理
微博奇葩,穿衣美容,搞笑幽默
广东生活
星座命理,IT 数码
一个人的世界
新闻资讯,美女,汽车,旅游
其他生活,搞笑幽默,美食,旅游,体育,汽车
时尚,娱乐,星座命理,旅游,美食
旅游,军事,IT 数码,汽车,体育
视频音乐
军事,IT 数码
旅游,星座命理,汽车,文艺,IT 数码
名人明星,穿衣美容
星座命理,美食,游戏动漫,旅游,美食,娱乐
娱乐,健康,投资理财,时尚,新闻资讯,军事
美食
美食,文艺
健康,汽车,旅游,军事,体育,娱乐
视频音乐,搞笑幽默,IT 数码,美女,游戏动漫,军事,体育
胡思乱想,宅女一枚,欧美控,手机看小说,文化,美剧迷
美食,投资理财,娱乐,健康,体育,旅游,骑行、户外、旅游

旅游,大学生,摄影爱好者,军事
新闻资讯,旅游,IT 数码,投资理财,文艺,奋斗 ing,自由,乐观,幽默,阳光
美食,文艺,娱乐,美食,旅游,健康,星座命理
穿衣美容
体育,娱乐,美女,健康,游戏动漫
美食,穿衣美容,旅游
文艺,时尚,投资理财,娱乐,旅游
文艺,娱乐,时尚,星座命理
教育就业,娱乐,新闻资讯,投资理财,文艺,星座命理,健康
宅女,家居,囧,北京,恐怖片
健康,美食,星座命理,娱乐
IT 数码,时尚,娱乐
投资理财,新闻资讯,教育就业
美食,星座命理,游戏动漫,文艺,IT 数码,娱乐
娱乐
美食,新闻趣事,视频音乐,穿衣美容,微博奇葩
教育就业,汽车,健康,文艺,旅游
健康,时尚
时尚,旅游,游戏动漫,星座命理
IT 数码,汽车
文艺,新闻资讯,健康,军事,体育
美图摄影,健康
萌宠,深圳生活,穿衣美容,时尚,温柔
汽车,文艺,IT 数码,新闻资讯,时尚
新闻资讯
旅游,时尚,美女,IT 数码,体育
美女,时尚
旅游,动漫,读书分享,美容服饰,美图摄影
汽车,游戏动漫,军事
书,music,双子,篮球爱好者,西安,世界杯,健康,体育
美食,IT 数码,体育,文艺
文艺,健康,时尚,美食,旅游
文艺,IT 数码
娱乐
汽车,文艺,投资理财,旅游,新闻资讯,时尚,教育就业,健康,IT 数码,娱乐
投资理财,娱乐,美女,时尚,美食
旅游,美食
星座命理,文艺
教育就业,时尚,健康,投资理财,旅游,文艺
健康,文艺,投资理财,旅游,IT 数码
文艺,IT 数码
娱乐
旅游
新闻资讯,军事,体育,娱乐

游戏动漫
娱乐
军事,IT 数码,体育,星座命理,美食,文艺,娱乐,旅游,教育就业
Nichkhun,文艺,娱乐
名人明星,旅游,娱乐,时尚
娱乐
娱乐,健康
军事,文艺,游戏动漫
旅游,军事,体育,健康
IT 数码
健康,美女,游戏动漫,时尚,文艺,体育
IT 数码,文艺,娱乐,体育
星座命理
星座命理
娱乐
IT 数码,汽车,体育,美食,旅游,星座命理,投资理财,娱乐
教育就业
IT 数码,旅游,星座命理,汽车,游戏动漫,娱乐
时尚,娱乐,投资理财,健康,教育就业
文艺,新闻资讯,教育就业,健康,投资理财
时尚
健康,旅游,美食,时尚,娱乐
休闲,瘦身,购物狂,喜欢听歌,浪漫
汪苏泷。星弟。,曾沛慈。
游戏动漫,汽车,旅游,军事,体育,玩
健康
教育就业
旅游,文艺,90 后但我不脑残,K 歌,拍照,狮子女,music,星座命理
美食,游戏动漫
郑州生活,穿衣美容,旅游,星座命理,美食,娱乐,文艺
时尚,游戏动漫,军事,美女,模特,美食,浪漫,游戏
旅游
美图摄影,新闻趣事,名人明星,视频音乐,搞笑幽默
搞笑幽默,文艺
名人明星,微博奇葩,新闻趣事,搞笑幽默
真实,最真实的自己!,爱好有点多
娱乐
旅游
娱乐,美食,文艺,旅游
搞笑幽默,萌宠,旅游
创业,读书,时尚
美女,娱乐
游戏动漫,IT 数码
游戏动漫,时尚,健康,军事,新闻资讯

新闻趣事
星座命理,美食,时尚,教育就业,健康
新闻资讯,星座命理
旅游,健康,娱乐,新闻资讯
汽车,娱乐,体育
微博奇葩
时尚
悲伤主义者
搞笑幽默
娱乐
文艺,健康,美食,旅游,IT 数码,体育
微博奇葩,游戏动漫
美女,文艺,汽车,旅游,美食,军事,体育
时尚,娱乐,健康,旅游,美食
文艺
美食,星座命理,宅女一枚,金牛座,70 后,吃货一枚,正太控,矛盾体,财经
音乐控,爱睡觉,学生一枚,懒人,爱幻想,连接服务器失败,星座命理
星座命理,娱乐
美食,旅游
喜欢听歌,爱电影,唱歌,星座命理,大学生,艺术,水瓶座
游戏动漫
文艺
够潮你就来
游戏动漫
IT 数码,文艺
新闻资讯,时尚,星座命理,文艺,娱乐
德普迷,电影
欧美音乐,经济,街拍
娱乐,教育就业
汽车,时尚,投资理财,健康,新闻资讯,文艺,娱乐
搞笑幽默,新闻资讯,美女,军事,娱乐
文艺,体育,娱乐
娱乐
投资理财,新闻资讯,健康,旅游,星座命理
旅行,闷骚,美食,时尚,IT 数码
美食,娱乐
旅游
新闻趣事,视频音乐,搞笑幽默,新闻资讯,汽车,旅游,IT 数码,娱乐,体育
视频音乐
北京生活,微博奇葩,穿衣美容,搞笑幽默,新闻资讯,时尚,美食,文艺
美食,汽车
健康,文艺,美食,旅游,娱乐
湖北生活
护肤,美白控,美容护肤,美容,旅游,时尚,娱乐,美女,健康

汽车,时尚,美食,娱乐,文艺
零售,便利店,小孩,教育,讲师,旅游
广东生活
文艺,娱乐,体育,旅游,时尚
肇庆生活,美食,娱乐,体育
画画
新闻资讯,教育就业,军事,投资理财,娱乐,体育,文艺,IT 数码
投资理财,IT 数码,健康
IT 数码
旅游,文艺,美食
健康,娱乐,美食
投资理财,IT 数码
汽车,美食,军事
军事,文艺,IT 数码,体育,娱乐
处女座 O 型,處女座
体育
娱乐
旅游
旅游
文艺,健康,新闻资讯,旅游,娱乐
旅游美食爱好者,交朋友,我就是我
娱乐,美女,游戏动漫
时尚,旅游,星座命理,美食,娱乐
美女
美食,旅游,健康,教育就业,游戏动漫
美食
体育
时尚,汽车,娱乐,体育,文艺
文艺,游戏动漫,美女,旅游,健康
新宇礼仪协会,篮球爱好者,体育
教育就业,健康,星座命理,旅游,美食
星座命理,健康,游戏动漫,时尚,投资理财
新闻资讯,娱乐,教育就业,健康,旅游,美食
无聊,80 后
健康
美食,健康,旅游,投资理财,体育
娱乐
娱乐,美食,欧美控,双子座,爱吃不胖,双重性格,90 后非脑残,神经大条
体育
时尚,旅游,健康
美女,新闻资讯,教育就业,娱乐,健康,文艺
时尚,健康,星座命理,旅游,美食,文艺,体育,娱乐
游戏动漫
微胖一枚,宅男 80 后,巨蟹座,码农,北漂

健康
美食,娱乐,宅女加腐女,杂志,射手座,大学生,乐天派,海绵宝宝,90 后但我不脑残
娱乐,创业,睡觉
星座命理,投资理财,娱乐,IT 数码,美女,疯扯扯
美女
娱乐,时尚,健康,旅游,美食
美女,汽车,IT 数码,军事,娱乐
旅游
体育,爱幻想,看小说,音乐爱好者,三国杀,o 型血,浪漫
O 型血,任性,学生一族,喜欢听歌,音乐控,吃货一枚,安静,减肥,傻
泰安生活
娱乐
健康,旅游
美女
穿衣美容
新闻资讯,投资理财,旅游,星座命理,娱乐
有军人本色……,军事
美食,时尚,吸血鬼日记
视频音乐,游戏动漫,娱乐,体育
high 歌,
互粉,美食,娱乐,健康,美女,投资理财
猫控,攝影,劍三,IT 数码,美食,游戏动漫
美食,学习永无止境,热爱生活每一天,红色性格,乐观好心态,谦和
直率
美食
微博奇葩,星座命理,娱乐
移动互联网,文学,爱情,阅读,潮,时事,爱旅游,爱电影,90 後,IT 数码
教育就业,体育,娱乐
汽车,旅游,美食,娱乐,文艺,体育
穿衣美容
投资理财,时尚,美食
八卦杂谈,健康,投资理财
健康
体育,文艺,IT 数码,军事
投资理财,汽车,时尚,新闻资讯,美女
文艺
旅游爱好者,游戏动漫,向日葵,单纯
旅游,星座命理,新闻资讯,时尚,投资理财
美食,娱乐
IT 数码,投资理财
健康,旅游,美食,星座命理
旅游,体育,娱乐,IT 数码
宋茜 Victoria,fx,雪莉,krystal,郑秀晶
娱乐,投资理财,美食,旅游,文艺,新闻资讯,健康

AB 型血,music
时尚,游戏动漫,军事
狮子座,90 后但我不脑残,美食控,爱自由
旅游,星座命理,时尚,健康,美食,文艺
娱乐
志愿者,生活大爆炸,政治,科幻
淘宝网女装,时尚潮女,潮女,时尚,时尚潮流,热销,热销女装,服饰搭配,梦炫天店铺,梦炫天店主
星座命理,美食,名人明星,视频音乐,搞笑幽默
旅游
游戏动漫
自由,大白菜,健康,美食
娱乐
教育就业,娱乐,健康,美食,投资理财
健康,旅游,美食,文艺,娱乐,家居,电影
文艺
军事,游戏动漫,美女,娱乐,星座命理
娱乐
时尚,美食,娱乐
旅游,美食
文艺,时尚,投资理财
体育资讯,视频音乐
文艺
文艺
搞笑幽默
教育就业,汽车,旅游,体育,娱乐
文艺
时尚
星座命理
IT 数码
健康,新闻资讯,教育就业
美女
旅游,医学,自由,赚钱,桌球,双子女,学生一枚
搞笑幽默
游戏动漫
新闻资讯,健康,体育,星座命理,美女,游戏动漫,时尚,IT 数码
美容服饰,视频音乐,八卦杂谈,美食,娱乐,文艺,IT 数码,汽车
微博奇葩
美女
狮子座,情绪化,摄影,旅游,自由
旅游
设计,梦想,休闲
娱乐,旅游,美食
时尚,小说,汽车,旅游,美食,文艺,娱乐

旅游
时尚
动漫,星座运势,文静， 爱听歌啦
旅游,汽车,时尚
星座命理,健康,旅游,游戏动漫,美女
江苏生活
听歌,上网,睡觉,自由,旅行,90 后,娱乐,时尚,美食,文艺
星座命理,射手,孩子气,幻想,执着,懒人
军事,旅游,游戏动漫,娱乐,体育
教育就业,IT 数码,美女,文艺
体育
投资理财,教育就业,娱乐
简单,爱生活,爱自由,90 後,影视,旅游爱好者,游戏动漫
穿衣美容,旅游
穿衣美容
美女,宅女,快乐,福州,单身
美女,IT 数码
旅游,美食,体育
穿衣美容,娱乐,时尚
健康,活泼开朗,拍照,射手女,简单,爬山,营销
搞笑幽默,游戏动漫,咖啡控,伪攻,待进化,吃货,柯南
美女,爱生活爱方媛,美食
健康
八卦杂谈,新闻趣事,名人明星,美食,视频音乐
美食
美女
星座命理,美食
新闻趣事,美食,星座命理,微博奇葩,搞笑幽默
娱乐,健康,旅游,教育就业,时尚
登山
90 后但我不脑残,喜欢旅游,暮光之城,自由,微博,星座命理,时尚,娱乐,旅游,健康
校园生活
游戏动漫,娱乐,IT 数码
多愁善感,网游,爱旅行,旅游,美女
旅游
娱乐,投资理财
美图摄影
平平淡淡才是真
smile,music
淘宝控,90 後,k 歌,韩剧,电影控,双鱼,电视,旅游美食,学生一枚,苹果
文艺
旅游,安静的骚男
欧美音乐,健康,时尚,文艺,娱乐,星座命理
体育,好动

健康,教育就业,美女,新闻资讯,体育
李宇春,玉米
懒,爱笑,快乐,美食控,娱乐
游戏动漫
游戏动漫
时尚,健康,旅游,美食,娱乐
娱乐
游戏动漫,星座命理
新闻资讯,星座命理,美食,文艺,娱乐
新闻趣事
美食
旅游,星座命理,美食,时尚,娱乐
搞笑幽默
旅游美食爱好者,水瓶女,护士,音乐迷,时尚达人
健康,文艺
视频音乐,旅游,名人明星,美食,穿衣美容
汽车,投资理财,教育就业,旅游,军事
投资理财
游戏动漫,旅游,汽车,体育,新闻资讯,时尚,军事
视频音乐,教育,读书分享,美女,校园生活
健康饮食,火锅达人,旅游爱好者,美食,85 后
旅游,美食
爱自由,公益,梦想,猫,钢琴,爱电影,90 後,腐,爱笑,文艺
投资理财,时尚,IT 数码
IT 数码,旅游,美食,新闻趣事,美容服饰
游戏动漫,IT 数码,汽车,技术宅,爱面子,腼腆,乐观
星座命理,时尚,美女,娱乐,体育
溜冰
文艺,旅游,时尚,星座命理,汽车
美食,美女,视频音乐,搞笑幽默,微博奇葩,娱乐
美食,文艺,教育就业,时尚,健康,旅游
旅游,视频音乐,广东生活,微博奇葩,健康,新闻资讯,军事,美食
时尚,美女,娱乐,体育,文艺
健康,美女,美食,军事,娱乐
时尚
游戏动漫,新闻资讯,体育,文艺,汽车,教育就业,投资理财,美食,军事,IT 数码
新闻资讯,游戏动漫,军事,IT 数码
新闻资讯,星座命理,文艺,教育就业,投资理财,爱音乐爱旅游,单纯双鱼女,喜欢前卫又传统
星座命理,游戏动漫,时尚,美食,军事,健康,娱乐,IT 数码,文艺,体育
美食,娱乐,时尚
文艺
美图摄影,喜欢听歌
重庆生活,娱乐
IT 数码,星座命理,美食,娱乐,文艺

健康,星座命理
美食
军事
名人明星
文艺,娱乐
美女,体育资讯,长沙生活,搞笑幽默,校园生活
新闻趣事
旅游,美食
体育
宝贝
时尚,投资理财
娱乐,旅游,体育,IT 数码,游戏动漫
美女,娱乐,旅游,军事,游戏动漫,文艺
彩妆,幽默,护肤,旅游,健康
汽车,军事,文艺,娱乐,体育
视频音乐
星座命理
IT 数码
美食
军事,健康,旅游,美女,美食
时尚,教育就业,新闻资讯
军事,美食,健康,旅游,文艺,体育,美食
娱乐,教育就业,旅游,健康
吃遍天下,全爱好
娱乐
视频音乐,旅游
美食
文艺,星座命理
游戏动漫
时尚
健康,军事,文艺,娱乐,美食,体育
新闻资讯,教育就业,星座命理,健康,汽车,旅游
开心,敏感,热爱生活,爱吃,懒人,学习,幻想,胡思乱想,宅女一枚
娱乐
军事,教育就业,健康,时尚,新闻资讯,投资理财,星座命理
IT 数码
Badgirl,前奏伴奏旋律控,钢琴,爱 miao 一族,巨蟹女
娱乐,旅游
无聊
爱花草,音乐,美文美图
汽车,教育就业,投资理财,健康,旅游
新闻资讯,教育就业,军事,文艺,体育
新闻资讯,娱乐,健康
新闻资讯,健康,美食,军事,教育就业

旅游,IT 数码,新闻资讯,时尚,体育
穿衣美容,文艺
重庆生活,新闻趣事,搞笑幽默,美女
健康
萌宠,健康
教育就业,健康,新闻资讯
八卦杂谈,美食,新闻趣事,校园生活,搞笑幽默
新闻趣事,魔羯座,旅游美食爱好者,简单,执着,爬山,80 後
搞笑幽默,萌宠,美食,星座命理,体育,紫色,学生,休闲,打篮球,喜欢旅游
IT 数码,博,网络,手机,八卦,打球,跑步,旅游,编程
旅游,微博奇葩,视频音乐
睡觉,看书,自由,宅,美食
穿衣美容
IT 数码,娱乐
美食,吃货一枚,盗墓笔记,时尚,健康,美女,投资理财
军事
广东生活
军事,把可笑当好笑
摄影控,室内设计,旅游,美食,文艺
健康,美食,IT 数码,娱乐,文艺
娱乐
旅游美食爱好者,学生一族,插画,小资,电视
游戏动漫,健康
开心,喜欢音乐,体育
游戏动漫
美图摄影
美食
文艺,教育就业
星座命理,娱乐,文艺,时尚,旅游,天秤,喜欢旅游
看电视,基本上都喜欢
美食
健康
体育,游戏动漫,IT 数码,寻爱
娱乐
美女,搞笑幽默,新闻趣事,旅游
星座命理
美女
IT 数码,旅游,娱乐,体育,文艺
旅游,美食,新闻资讯,投资理财,娱乐
健康,游戏动漫,时尚,娱乐,星座命理
体育
『用心微笑』
时尚,IT 数码,旅游,美食,文艺,娱乐
文艺,汽车

美女
娱乐
名人明星,时尚,新闻资讯,健康,星座命理,美食
健康,新闻资讯,时尚,投资理财,教育就业
文艺,健康,美食
吃货一枚,爱美食,时事
强迫症,music,家居,看电视,双重性格,健康,时尚,新闻资讯,美食,娱乐
手机看小说,阅读,摩羯座,学生,自由,想一个人去旅行
旅游,时尚,教育就业,投资理财
时尚,娱乐,星座命理
视频音乐,娱乐,吃货，玩货。,旅游！旅游!!!
。。。。
美图摄影
美食
军事,IT 数码,文艺,娱乐,体育
健康,美食,游戏动漫,文艺,体育
旅游,健康,美食,娱乐,体育,时尚
视频音乐,美食,IT 数码
娱乐,文艺
健康,美食,旅游
文艺,健康,时尚,美食,韩国,购物,听音乐,双重性格,英语,90 後
健康
双重性格,爱电影,古典艺术,原创,AC 米兰,美食,时尚
健康,旅游,投资理财,娱乐
旅游
大学生,爱幻想,动漫,宅,咖啡,手机看小说,白羊,电视,学生一枚,感性
美食,健康,娱乐
旅游,旅游，自由，美食
美女,学生
动漫,酷爱火影
电影爱好者,策划,英语,浪漫,爱笑
旅游
汽车,健康,美食,娱乐
时尚
时尚,美女,旅游,美食,IT 数码,文艺
音乐迷,美食,军事,新闻资讯,旅游,文艺
美食,美容服饰,视频音乐,台湾生活,搞笑幽默
旅游
美食,娱乐
娱乐,美女
高一 21 班
旅游
时尚,美食,旅游,健康
时尚,旅游,文艺

新闻资讯,投资理财,教育就业,军事,娱乐,健康,美食,IT 数码
游戏动漫,IT 数码,汽车,军事,新闻资讯,投资理财,体育
投资理财,体育,军事,新闻资讯,教育就业,星座命理
萌宠,旅游,新闻趣事,其他生活,星座命理,体育
文艺,健康,旅游,美食
娱乐,美食,IT 数码
体育,时尚
健康,汽车,体育,娱乐
游戏动漫,旅游,星座命理,投资理财
穿衣美容
旅游,时尚,娱乐,美食
美女,IT 数码,军事,娱乐,体育
娱乐
军事,美女,星座命理,看小说,美食控,活泼,手机,维他命,聊天,休闲
汽车,健康,军事,体育,娱乐,旅游
娱乐
穿衣美容
美食,旅游
情感生活,玉溪生活,旅游,美食,吃货,吃喝玩乐,看小说,吃货一枚,爱旅行,睡懒觉
娱乐
乐天派,爱唱歌
旅游,萌宠,视频音乐,星座命理,美食
腹黑,爱睡觉,闷骚
视频音乐,美女,旅游,汽车,IT 数码
电视,爱电影,k 歌,旅行,文艺
名人明星,搞笑幽默,穿衣美容,美女,微博奇葩
新闻资讯,军事,体育,投资理财,教育就业
时尚,旅游
美女
其他生活
名人明星
IT 数码,娱乐
名人明星
文艺,美食,娱乐
搞笑幽默,投资理财,美食,文艺,星座命理,娱乐
微博奇葩,文艺
娱乐,遊戲動漫
星座命理,健康,旅游,美食,文艺,娱乐
星座命理,多愁善感
陈奕迅
美食,娱乐,旅游,星座命理,时尚,教育就业
美女,体育
娱乐,体育
健康,旅游,星座命理,IT 数码,文艺

健康
游戏动漫,投资理财,美食
投资理财,美食,IT 数码
时尚,军事,文艺
娱乐
旅游,时尚,文艺,娱乐
时尚,投资理财,IT 数码
美食,搞笑,吃货,偶稀饭睡觉觉,音乐控,喜欢音乐,爱美食
搞笑幽默,娱乐
体育
美食,看小说,许嵩,平常心,善良,睡懒觉,摩羯女
健康,美食,星座命理,搞怪,微笑
娱乐,美食,时尚,健康,旅游
单身,创业,奋斗,乐观,低调
健康,娱乐,美食,汽车,美女,游戏动漫
文艺,娱乐
美食
学习，运动
视频音乐,健康,鸿辉超市,进口食品,中山学院
学习
美女,旅游,健康,军事,娱乐
魔羯座,棋牌游戏,动漫玩具,音乐美术,金融理财,史地军事,天体航模,花鸟鱼虫
IT 数码
疯子,旅行,爱美食,吃货一枚,胡思乱想,简单,怀旧
旅游,其他生活,校园生活
山东生活,时尚,汽车,健康,美女,文艺,体育,娱乐
萌宠,军事,文艺
粤语,爱吃,冷笑话,矛盾,爱旅行,微笑
狮子女,淘宝,美食,学生一族,90,旅游美食爱好者,睡懒觉
时尚
新闻资讯,汽车,IT 数码,健康,旅游,星座命理
时尚,体育,娱乐,旅游,汽车
旅游,IT 数码
IT 数码,旅游,汽车
旅游
教育就业,美食,旅游,IT 数码,体育
汽车,美食,旅游,娱乐,体育
时尚
军事
健康,小女人,聊天,旅游爱好者,电影控,懒,玩,美食控,大学生,偶稀饭睡觉觉
美食,时尚,汽车,IT 数码,美女
名人明星
教育就业,文艺,旅游,美食,时尚,健康
健康

美发造型,设计,时尚
体育
健康,IT 数码,时尚,旅游,美食
健康,旅游,IT 数码,体育,娱乐
B 型血,神经大条,恐怖片,文艺,娱乐
旅游
美食
新闻趣事
美食,旅游,娱乐,汽车,时尚
星座命理,新闻资讯,时尚
星座命理,教育就业,健康,文艺,新闻资讯,旅游
汽车,时尚,文艺,娱乐
美女,健康,投资理财,汽车,文艺,美食,旅游
美女,时尚,娱乐,酒吧,健身,高尔夫,8090,时尚潮流,泡吧,无锡人
游戏动漫,文艺
随性,文艺,音樂,宅女一枚,旅游爱好者,真诚,青春,跑步,心情,爱自由
娱乐
汽车,美女,军事,IT 数码,娱乐
投资理财,美女,美食,文艺,军事,体育,娱乐
美女
文艺,游戏动漫,IT 数码
美女,汽车,投资理财,娱乐
旅游,微博奇葩,视频音乐,萌宠
娱乐
美食,搞笑幽默,美女,穿衣美容
旅游
旅游,文艺
娱乐,双鱼女,胡思乱想,爱旅行
微博奇葩,娱乐,游戏动漫,90 后,动漫,双鱼座,睡懒觉,情绪化
小正太,龙太子,10 后新生代
旅游,爱玩,安静,自由,听歌,双子座,暮光之城,爱旅行,音乐爱好者
健康,旅游,教育就业,新闻资讯
时尚,星座命理,旅游,投资理财,新闻资讯,健康
美食,娱乐,美女,教育就业,时尚
健康
湖北生活,娱乐
美女,体育,幽默
健康,时尚,投资理财,文艺,娱乐,低调
旅游,游戏动漫,动漫,妖精,小说迷,高中生,爱音乐,手机看小说
投资理财,美女
旅游,娱乐,蛋逼,文艺,主持人,视觉,广告,创意,极限,音乐
文艺,健康,教育就业,投资理财,汽车,新闻资讯,军事
旅游,星座命理,美食,时尚,娱乐,汽车
娱乐,美食,美女,军事,时尚

游戏动漫,星座命理,美食
军事,旅游
文艺,健康,旅遊,小清新伪文艺,五月天
正品保证,物美价廉,小西北团购,团购,网购,阿拉尔,塔里木大学
IT 数码,健康,旅游,美食,娱乐
汽车
文艺
简单,天蝎,自然,吃货一枚,懒,美食控
时尚,新闻资讯,旅游,IT 数码,体育
娱乐,认真的执著
名人明星,视频音乐,微博奇葩,搞笑幽默,穿衣美容
体育
时尚,旅游,美食,体育,娱乐
旅游,美食
美食,旅游,文艺
美食
美食,娱乐
投资理财,旅游,文艺,体育,汽车
新闻资讯,时尚,健康,旅游,美食
时尚
运动,移动互联网,听歌,旅游
北京生活,娱乐
美女,健康,娱乐,游戏动漫,投资理财,时尚,美食,星座命理,吃喝玩乐
美食
教育就业,投资理财,健康,美食
美食,健康,汽车,游戏动漫
娱乐,游戏动漫
美女,娱乐,时尚,美食,健康,旅游
军事
汽车,美女,旅游,军事,娱乐
健康,投资理财,美食,旅游,星座命理
健康
校园生活,教育,新闻趣事,八卦杂谈,搞笑幽默
汽车,新闻资讯,美食,IT 数码,娱乐
新闻资讯,美食,旅游,教育就业,IT 数码
微笑,爱吃,滑雪,情绪化,散步,美食爱好者,偶喜欢玩,小清新,喜欢旅游,学生
视频音乐,其他生活
旅游,娱乐,体育,IT 数码,美女
名人明星
娱乐,旅游
军事
游戏动漫,汽车,军事,IT 数码,体育
新闻资讯,教育就业,文艺,IT 数码,军事
文艺

旅游
搞笑幽默
娱乐
旅游,文艺
旅游
星座命理,娱乐,健康,时尚,文艺,IT 数码
新闻趣事,视频音乐,穿衣美容,名人明星,沧州生活,时尚,健康
小说迷,配音,慵懒随意,小清新伪文艺,古风控
穿衣美容,娱乐
美食
视频音乐
汽车,体育
搞笑幽默,娱乐,健康,文艺
美食,美女,时尚,汽车,娱乐
娱乐,健康,游戏动漫,旅游
美图摄影,视频音乐,济南生活,汽车,时尚,旅游,IT 数码
文艺
文藝,搞笑幽默,视频音乐,美女,做事情与思考,金融与投资
时尚,文艺
时尚,美女,旅游,美食,汽车
微博奇葩,搞笑幽默,广西生活,文艺,娱乐,旅游,美食
美剧,林宥嘉,五月天,微博控,美食控
IT 数码,娱乐
美食
遊戲動漫
自由职业,娱乐,喜欢音乐
舟山生活
视频音乐,名人明星,旅游,忽冷忽热,强迫症,间歇性耳聋
看电视,
美食,旅游,娱乐
星座命理
四川生活
美女,体育
搞笑幽默,军事
娱乐,美食
游泳,k 歌,苹果,电影迷,体育
美食,军事,IT 数码,文艺,娱乐
其他生活,星座命理,搞笑幽默,微博奇葩,新闻趣事
电影控,服装,音乐爱好者,韩剧,旅游爱好者
文艺
文艺,知足常乐,胖胖,知足常乐；胖胖；
游戏动漫,星座命理,美食,娱乐
文艺
星座命理

娱乐,美食
IT 数码
旅游,美食
美女
新闻资讯,美食,旅游,健康
新闻资讯
美术,星座命理
寓意深刻,短小精悍,一分钟故事,小故事,小小说,微小说控,小说故事,小说爱好者,小说控,小说迷
浙江生活
新闻资讯,美女,教育就业,投资理财,军事
投资理财,时尚
体育,美食,游戏动漫
娱乐
教育就业
文艺
美女
时尚,汽车,投资理财,旅游,美食
健康,美食,投资理财
星座命理,旅游,游戏动漫,时尚,美女
娱乐
穿衣美容,韩国代购淘宝店,时尚
IT 数码,健康,投资理财,体育,娱乐
时尚,汽车
旅游
猛一抬头,什么也看不见
白羊座,音乐迷,80 后,交朋友,生活,销售,养生,休闲,男人,健康
投资理财
娱乐
娱乐,投资理财,汽车,美女,文艺
文艺,旅游,体育
旅游
旅游,健康,美食,投资理财
旅游,美食,游戏动漫,漫迷，爱自由,漫迷,爱自由
文艺,冰雪聪明
新闻资讯
武汉,台球,爱旅行,体育,电脑
广东生活,投资理财
美食
旅游
美食
视频音乐,美图摄影,美食,名人明星,搞笑幽默
文静,文艺,时尚,健康,星座命理,娱乐
娱乐

娱乐
美女,美食,IT 数码,体育,娱乐
时尚
体育
美食,娱乐,时尚,星座命理
穿衣美容,旅游
上海生活
投资理财,教育就业,汽车
娱乐,旅游,IT 数码,游戏动漫
游戏动漫,汽车,美食,旅游,娱乐
美女,汽车,游戏动漫,时尚,IT 数码,娱乐
健康,旅游,美食
时尚,星座命理,文艺
旅游,军事,星座命理,投资理财
美食,星座命理,游戏动漫
军事,健康,旅游,体育
星座命理
名人明星,娱乐
健康,旅游,美食,时尚
萌宠,穿衣美容
娱乐
美容服饰
青春从未走远,投资理财
娱乐
时尚
IT 数码
IT 数码,时尚,汽车,体育
自由主义
健康,旅游,新闻资讯,娱乐,时尚
星座命理,新闻资讯,文艺,旅游,健康
新闻资讯,汽车,娱乐,体育,军事
时尚,美食
娱乐
旅游
IT 数码
旅游,人在做、,天在看
时尚,旅游,汽车,娱乐
娱乐,时尚,美女,美食
广州曾晨,热转印,数码印花
投资理财
健康养生
健康
名人明星
游戏动漫,文艺

心理学,爱幻想,旅行,搞笑幽默,90 后,经济,音乐,新闻资讯,星座命理
情感生活,旅游,美食,邯郸生活,名人明星
旅游,美食
娱乐
旅游,肇庆
游戏动漫,时尚
萌宠,视频音乐,名人明星,搞笑幽默,汽车
投资理财
文字控,软件,学生一族
IT 数码,星座命理
动漫
体育资讯,美女,视频音乐,篮球,动漫,游戏,天枰座,电影,羽毛球,科比
时尚,健康,服装设计,巨蟹座,画画,吃货,喜欢旅游,浪漫
新闻资讯,时尚,娱乐,文艺,旅游
游戏动漫
美食,星座命理,文艺
新闻资讯,娱乐,睡觉睡到自然醒
IT 数码,美食,教育就业,新闻资讯,娱乐
军事,美食
健康,旅游,星座命理,教育就业,美食
投资理财,教育就业,旅游,娱乐
娱乐,旅游,美食,文艺,时尚,星座命理,美食
娱乐
旅游,体育,文艺
旅游
健康,娱乐
韩剧,欧美,粉红控,时尚潮流,美图摄影,视频音乐
文艺,睡觉睡到自然醒,自由,睡觉,宅,90,无聊,看小说,听音乐,发呆
娱乐,汽车,IT 数码,体育
军事,IT 数码,新闻资讯,教育就业,旅游,文艺
文艺,健康
美女,军事,IT 数码
旅游
自然,吸血鬼日记,谢娜,少女时代,妖精,古典艺术,护肤,狗狗,喜欢听歌,热爱生活
文艺,汽车,健康,娱乐,星座命理,时尚,旅游,美食
听歌,打网球
粤语,时尚潮流,孩子气,纠结,时尚达人,爱旅游,陈晓东
时尚,旅游,美食
健康,娱乐,体育,文艺
健康,美女,游戏动漫,美食
文艺,IT 数码,健康,娱乐,时尚,旅游
搞笑幽默
健康,美女,旅游,美食,娱乐
穿衣美容

汽车,军事
时尚
游戏动漫,美食,军事
健康,娱乐,时尚,旅游
旅游,健康,星座命理,IT 数码,教育就业
美食,旅游
娱乐
游戏动漫
美女
美女
娱乐,美女,美食
心如止水,学生,射手,淡定,三分钟热度
文艺,星座命理,军事
时尚,美女,瘦身
投资理财,美食,旅游,军事
爱旅行,微博,拍照,90 后非脑残,新闻资讯,bigbang,大学,白羊座,听音乐
旅游,文艺,星座命理
美女
拍照,紫色
吉林生活,健康
人生哲理,经典段子,一分钟小说,一分钟小故事,微小说达人,微小说控,小小说爱好者,小说控,文学,小说迷
娱乐,体育
美食
文字控与自由控
严肃的八卦,爱自由,爱玩,音樂,美食控,时尚达人
娱乐,星座命理,健康
美图摄影,旅游,视频音乐,校园生活,泉州生活,阳光
娱乐,旅游,星座命理,美食,文艺
旅游
旅游
音乐
广州,IT 互联网,狮子座
柯南,美食,时尚,新闻资讯,健康,文艺
美图摄影
美食,粗粮,膨化食品,玉米食品,新闻资讯
美食
视频音乐,武汉生活,美图摄影,搞笑幽默,校园生活
时装,天蝎,热爱生活,喜欢听歌,睡懒觉,健身,活泼,电影爱好者,欧美音乐,时尚
游戏动漫
微博奇葩
美食,IT 数码,投资理财,时尚,美女
校园生活
时尚

星座命理
努力,健康,旅游,投资理财,娱乐,体育,IT 数码
文艺
星座命理
旅游,美食,游戏动漫,时尚,娱乐,巨蟹,格子控,动漫,自由
娱乐
军事,健康,体育
临沂生活
旅游,美食,健康,教育就业
娱乐
体育,旅游,单身,独立,小女人
娱乐
夜猫子,陈翔,大叔控,爱笑
文艺
爱食茶
蓝色,自由,平常心,水瓶座
娱乐
星座命理
新闻资讯
新闻趣事,穿衣美容,时尚
游戏动漫,旅游,美食
唱歌,DIY,双子座
八卦杂谈
美食,福建生活,搞笑幽默,军事,IT 数码,漫画,减肥,大学
爱笑,爱旅游,爱电影
旅游,美食,IT 数码,军事
石磊,美食
投资理财
娱乐
娱乐,汽车
健康
傲娇
时尚,娱乐,星座命理,搞笑,幽默,做梦
香港生活
新闻资讯,时尚,IT 数码
穿衣美容
萌宠
BaekHyun,边伯贤
宠物图集
游戏
美食
时尚,美食,旅游,星座命理,文艺,娱乐
美食,旅游
汽车

军事
时尚,星座命理,游戏动漫,美女,IT 数码
美食控,探险爱好者,音乐,甜点 DIY,阅读,养花,阳光,乐观,运动,绘画
文艺
星座命理
美女
美食,摩羯,安静,美术,杭州,爱,死神,寂寞,漫画,单身
穿衣美容,星座命理,游戏动漫
新闻资讯,时尚,投资理财,汽车,旅游,娱乐
投资理财,美食,旅游,新闻资讯,时尚,娱乐,爱就要坚持下去
汽车
文艺,娱乐,美食,汽车
微博奇葩,搞笑幽默,新闻趣事,山东生活,穿衣美容,文艺
美食,金融
军事,新闻资讯,健康
娱乐
星座命理,美食,时尚,娱乐,吃东西、睡觉、
娱乐
很疯,游戏,小说
美食,美女,汽车,娱乐,体育
美食,旅遊,投資理財,文藝,體育
美食
美食,娱乐
健康
文艺,时尚
旅游,娱乐,美食
投资理财
投资理财
神经质,文艺
民工
音乐爱好者,新闻资讯,外冷内热
文艺,教育就业
文艺
旅游,星座命理,娱乐
健康,旅游,星座命理,娱乐,时尚,美食
时尚,教育就业,新闻资讯,旅游,文艺
美食,娱乐,文艺,美女,时尚
神经质,腐,绘画,化妆,自由,摄影,喜欢听歌,淡定,创意,胡思乱想
军事,娱乐,星座命理,时尚
投资理财
投资理财,IT 数码,旅游,汽车
情感生活
时尚,美食
文艺

军事
娱乐,旅游
萌宠
投资理财
新闻资讯,军事,IT 数码,文艺,体育,娱乐
新闻资讯,娱乐
旅游
娱乐
娱乐,O 型血
娱乐
娱乐
娱乐
微博奇葩
IT 数码,游戏动漫
美食,微博奇葩
无聊,旅游摄影
旅游,美食
IT 数码,游戏动漫
Beliber,旅游,韩寒,微博,90 后
新闻资讯,旅游
搞笑幽默,游戏动漫
资讯控,时尚,80 后,段子,电商,笑话,语录,糗事,幽默搞笑,淘宝
旅行
美食,游戏动漫,时尚,IT 数码,娱乐,文艺
健康,美食,娱乐
服装设计,日本文化,日文,初级瑜伽,喜爱看动漫,内向
旅游
星座命理
动漫
美食控,安静,90 后,交朋友,爱电影,网球,学生一族,爱睡觉,成熟,天秤座
萌宠
新闻趣事,美女,娱乐,星座命理,美食,旅游
读书分享,文艺,简单
减肥茶,女人养身,美容护肤,祛痘印,祛痘,减肥瘦身
美食,旅游
dota,模特,旅游
娱乐,IT 数码,健康,时尚,教育就业
美食
IT 数码
美食,游戏动漫
运动,自然,军事,新闻资讯,健康
娱乐,游戏动漫
游戏动漫,旅游,军事,IT 数码,文艺
时尚,娱乐

美女
搞笑幽默
搞笑幽默,微博奇葩,游戏动漫,IT 数码
欧美音乐,旅游
时尚,体育,文艺,美食,游戏动漫,娱乐
视频音乐
K 歌
校园生活,武术,music,新闻,90 後,娱乐,健康,体育
军事
文艺,教育就业
美女
娱乐
阳光,爱生活,重口味,萝莉控,体育,fashion
搞笑幽默
新闻趣事
健康
文艺
文艺,健康,军事
投资理财,汽车,IT 数码
健康,美食,旅游,美女,时尚
穿衣美容,时尚
军事,体育,娱乐,双子座
新闻趣事,美食,搞笑幽默
海外生活,视频音乐
搞笑幽默
娱乐
美女,游戏动漫,娱乐,体育,IT 数码
娱乐,健康,美食,文艺,时尚
健康
投资理财,娱乐,体育,汽车,军事
IT 数码,投资理财,教育就业,新闻资讯
搞笑幽默,美图摄影,动漫,花样美男
茶,多愁善感,寂寞,it 互联网
宅女,狮子女,腹黑,神经大条,傻姑凉。。
新闻资讯,美女,文艺
文艺,娱乐
恐怖片,赚钱,销售,喜欢听歌,手机看小说,工作,90 後,寂寞 ing,电脑,金牛座
文艺,美食
旅游,星座命理,美食,娱乐,新闻资讯,游戏动漫
旅游,娱乐
时尚
娱乐,汽车,投资理财,旅游,军事,IT 数码
钓鱼,篮球,运动,听歌
文艺

军事
游戏动漫,哲学,爱自由,偶像剧,安静,玩,爱电影,大学生,90后
投资理财,军事,娱乐,游戏动漫,时尚,体育
美女,旅游,星座命理,新闻资讯,IT数码,体育
游戏动漫,星座命理,新闻资讯,汽车,IT数码,教育就业,美食,健康,文艺,体育
教育就业,IT数码,娱乐,游戏动漫,时尚,文艺
微博奇葩
搞笑幽默,新闻趣事,美女,微博奇葩
处女座,幻想,爱,时尚,娱乐,星座命理
江西生活
娱乐,美食
旅游,星座命理,微博奇葩,搞笑幽默,浙江生活,娱乐
投资理财,游戏动漫,体育,IT数码
新闻资讯,IT数码,娱乐
时尚,旅游,IT数码
新闻资讯,教育就业,旅游,星座命理,文艺
美女,旅游,体育
美食
军事,美食,时尚,旅游,文艺
音乐爱好者
美食
名人明星,新闻趣事,旅游,广西生活,搞笑幽默
自动车床加工,机械加工,奋斗,随性
时尚
动漫,投资理财,文艺
娱乐,IT数码
星座命理
娱乐
星座命理,旅游,美食
健康,体育,娱乐,汽车,教育就业,军事
旅游
旅游,星座命理,美食
台球,潮流,IT数码
搞怪
疯子,快乐大本营,90,简单,WOW,偶稀饭睡觉觉,爱睡觉,懒人,欧美控,justinbieber
娱乐
健康,旅游
瘦身,蓝色,摄影师,摄影控,时尚,健康,旅游,娱乐,文艺
娱乐,大学生,自由,上网,宅,睡觉
睡觉睡到自然醒,心理学,AC米兰,自拍,天蝎女
音乐
文字,插画,喜欢旅游,漫画,狗狗,吃喝玩乐,书籍,唱K
美食
新闻资讯

美女
时尚,投资理财,新闻资讯
美食
娱乐
投资理财,旅游,娱乐,游戏动漫,篮球,简单,忧郁,音樂,乐观
90後,摩羯座,爱睡觉,傻,笑,拍照,吃货,设计,大学生
星座运势,星座命理
体育
美食,娱乐,旅游,搞笑,摄影爱好者
美食,汽车,美女,娱乐,IT数码
武汉生活,时尚
教育就业,美食,娱乐,IT数码,健康,美女,新闻资讯,游戏动漫,文艺
文艺
旅游,大学,电视剧,爱笑
90后,睡懒觉,旅游,游戏动漫,娱乐
阅读,音乐,娱乐,旅游,时尚
健康,IT数码,体育
美食,体育,美女,时尚,旅游,IT数码
微博奇葩,旅游,美食,星座命理,新闻趣事
IT数码,职场招聘,视频音乐,泰安生活,搞笑幽默,军事,健康,星座命理,汽车
旅游
娱乐,世界以痛吻我,我却回报以歌,只要我有,只要你要,没有不可能,世界对你来说,桃子
天气,孙中山,民主政治,英语,培训,诗词,跳舞,真诚,健康,星座命理
旅游,美食,娱乐,时尚
娱乐,游戏动漫,美女
美食
投资理财
时尚,娱乐
旅游
新闻资讯,投资理财,健康,星座命理,时尚,娱乐,文艺
活泼,健身,美食控,漫画,金融
旅游
文艺,时尚,美食,星座命理,娱乐
美女
美食,娱乐,EXO
动漫,猫控,幻想,美食
德州扑克,两性关系,体育,投资理财
旅游
90後,个性，纯粹，开朗
其他生活
娱乐,游戏动漫
美食,时尚,汽车,教育就业,娱乐
音乐迷,文艺,健康
美食

美女,美食,投资理财,旅游,汽车,游戏动漫,军事
健康,时尚,旅游,美食,娱乐
旅游,视频音乐,搞笑幽默,美食,IT 数码,娱乐
游戏动漫,旅游,IT 数码
文艺,时尚,旅游,星座命理,美食,多情,矛盾综合体,懒人,胡子控,强迫症
旅游,美食,军事,娱乐,体育
健康,时尚,美食
游戏动漫
教育就业,时尚,娱乐,文艺
健康,投资理财,IT 数码
游戏动漫,军事,IT 数码,文艺,美女
CSOL,微博控
新闻趣事,搞笑幽默,游戏动漫
文艺,娱乐,游戏动漫,军事
汽车
时尚,教育就业,旅游,美食,娱乐
美食,时尚
星座命理,游戏动漫,旅游,美食,娱乐
健康,文艺,娱乐
美女
健康,时尚,文艺,汽车,旅游,美食,教育就业,投资理财,IT 数码
美食,军事,IT 数码
汽车,时尚,游戏动漫,旅游,IT 数码,投资理财,军事,文艺,体育,娱乐
看小说,动漫迷,写作,动画,双鱼座,音乐迷,时尚,健康,星座命理,美食
娱乐
军事,娱乐,汽车,IT 数码,投资理财,旅游,星座命理
穿衣美容,视频音乐,星座命理,微博奇葩,新闻趣事
IT 数码,汽车
投资理财,教育就业,文艺,美食,星座命理
美食,文艺,时尚,娱乐
活泼开朗,美剧迷,音樂,简单,文字控,唱 k,吃喝玩乐,爱自由,时尚,娱乐
体育,少女时代,哈哈大笑,抽风无时无刻
美食,新闻资讯,娱乐,游戏动漫,美女,星座命理
文艺
健康,星座命理
体育
时尚,投资理财,游戏动漫,健康,旅游,文艺
文艺
汽车
运动,交朋友,我就是我,乐观,热爱生活,淡定,内向,旅游,健康,美食
游戏动漫
旅游
投资理财,新闻资讯,IT 数码
幸福,微笑,购物,热情,爱吃,热爱生活,开心,音乐控

时尚,星座命理,旅游,健康,娱乐
汽车,军事,IT 数码,文艺,娱乐
文艺
读书分享,星座运势,校园生活
美女
k 歌，斗地主………,品牌
游戏动漫
吴世勋,鹿晗,90 後,处女座
新闻趣事
足球,湖人,NBA,韩国,狗狗,健康,旅游,体育,美食,娱乐
政治经济,爱电影,O 型血,翻译,爱自由,爱旅行,看书
萌宠
时尚
美食,汽车
娱乐,旅游,星座命理
投资理财,IT 数码,新闻资讯,旅游
美容护肤,淡定,星星，娜米,星座命理,娱乐
穿衣美容,时尚
简单,爬山,音乐控
星座命理,音乐,新闻资讯,美女,文艺,旅游,八卦杂谈,重口味,名人明星,动漫
真实力,户外,麦霸
时尚,娱乐,体育,IT 数码,机电一体化,有时很衰,奋斗中,幸运,幽默开朗,有梦想
美图摄影,美食,贵州生活,搞笑幽默
时尚,打工仔,韩寒
旅游
新聞資訊,時尚,健康,旅遊,投資理財
时尚,IT 数码
军事,时尚
旅游,健康,体育
穿衣美容,完美主义,巨蟹座 AB 型
IT 数码,娱乐,健康
新闻资讯,时尚,旅游,美食,娱乐
汽车,军事,旅游
教育就业,健康,新闻资讯,旅游,文艺
娱乐
军事,各种交流
美食
爱美食,爱音乐,爱旅行,游戏动漫,星座命理
健康,时尚,投资理财,教育就业
美食,星座运势,视频音乐,╰→爱音乐
文艺,健康,体育,游戏动漫,娱乐
穿衣美容
穿衣美容
美食,文艺,时尚,汽车,游戏动漫

新闻资讯
投资理财,旅游,星座命理,新闻资讯,IT 数码,体育
文艺,时尚,娱乐
美食,视频音乐,搞笑幽默,旅游,美女,娱乐,吃货，欣赏美好。
重庆生活
投资理财
体育
视频音乐,体育,汽车,时尚,娱乐
动漫,美食,偏执腹黑本命,瓶邪塞夏党,贾童鞋 bieber,B 站二次元,重口味小清新,盜墓筆記,灵异神秘学,COSER 控
星座命理
IT 数码
体育,娱乐
想到你会笑
音乐达人,浪漫,睡懒觉,偶喜欢玩,宅男,美女,星座命理,游戏动漫,体育,娱乐
魔羯座,咖啡控,跆拳道,成都,房产,执着,简单,咖啡,游戏,乐观
健康,IT 数码,文艺,体育
星座命理
娱乐,文艺
文艺,健康,旅游,时尚,娱乐
情感生活,狮子座的 80 后,情感,家居,喜欢听歌
旅游
dj
美食,文艺,IT 数码,娱乐
健康
投资理财,旅游,美食
陕西生活,微博奇葩,旅游,健康,时尚
旅游,投资理财,汽车,健康,军事,体育
时尚,美食,新闻资讯,旅游,文艺,游戏动漫,星座命理
星座命理,微博奇葩,视频音乐,名人明星,萌宠
旅游
娱乐
星座命理
欧美控,爱旅行,自由,自拍,红酒,金贤重,B 型血,真诚,青春,小资
美女
休闲,星座,双子,宅,安静,感性,音乐控,健康,美食,IT 数码
文艺,游戏动漫,汽车
汽车,旅游,IT 数码
新闻资讯,体育,娱乐
阳光,插画,原创,爱玩,文艺,旅游,手绘,漫画,动漫
旅游
美女,健康,文艺,时尚,星座命理,美食,娱乐
娱乐,旅游,逛街，折纸,美食
健康,旅游,文艺,娱乐,美食,美女

旅游,娱乐,投资理财,新闻资讯,健康,星座命理
金华生活,娱乐
时尚
科学探索,读书分享,星座运势,体育资讯,爱自由,梦想
旅游,娱乐,汽车,文艺,体育,音乐，篮球，文艺
文艺
娱乐
美图摄影
穿衣美容
搞笑幽默,阳江生活,IT 数码,美女
投资理财,娱乐,教育就业,新闻资讯,时尚,文艺,健康
IT 数码
美食,旅游,娱乐
娱乐,美食,矛盾体,胡思乱想,幻想
旅游,美食,文艺
旅游,健康
旅游
旅游
教育就业
娱乐
教育就业,美食,健康,旅游
体育,汽车,IT 数码,娱乐
时尚,娱乐
娱乐
穿衣美容
娱乐,文艺
育儿百科,创意设计,情感生活,时尚
健康,时尚
健康
健康
娱乐,IT 数码,小白贤,灿灿,小鹿,小白久
穿衣美容,娱乐
moonlight,雅痞,85 后,文艺
文艺
微时尚,网购,团购网购,淘宝天猫控,时尚潮流,美容美体,化妆护肤,减肥瘦身,购物达人,潮流服饰搭配
名人明星
旅游,体育,IT 数码,美食
玩儿,炎亚纶,奋斗,坚强,吸血鬼日记,柯南,淘宝控,书,心理,爱唱歌
搞笑幽默,新闻趣事,视频音乐,汽车,军事,IT 数码,文艺,体育
阳光,旅游美食,天秤,迷糊
美女,美食,旅游,娱乐,体育,文艺
娱乐
娱乐

旅游,体育
美食,时尚
星座命理
单身,摩羯座,小女人,睡觉,爱音乐,Music
健康,星座命理
旅游,娱乐,美食,时尚,健康
美女
健康
体育
旅游
旅游,军事,文艺,体育,娱乐
美食
B 型血
文艺,汽车,旅游
投资理财,旅游,教育就业,健康,军事
睡觉,音乐,古风
微博奇葩,美食,视频音乐,新闻趣事,搞笑幽默
微博奇葩,星座命理,穿衣美容,视频音乐,搞笑幽默
美食,宠物图集,美图摄影
美女
IT 数码,时尚,军事,汽车,旅游
其他生活
娱乐,时尚,偶喜欢玩,90 後,拍照,美女,小说,香水控
旅游
汽车,文艺,体育,单纯
搞笑幽默
稳重,运动
时尚,旅游,健康,美女,体育
星座命理
文艺,时尚,健康,旅游,娱乐
时尚
娱乐
星座命理,美食,军事
健康,体育,娱乐
深圳生活
娱乐
时尚,旅游,健康,美女,文艺
健康,体育,娱乐
游戏动漫,时尚,IT 数码
文艺,体育,娱乐
健康,体育,娱乐
动漫,篮球
文艺,体育,娱乐
玩儿

旅游,星座命理
萌宠
投资理财,美食,健康,旅游,IT 数码
看电视，听音乐
时尚,汽车,娱乐
游戏动漫,时尚,军事
健康
健康,时尚,人力资源从业者,疯子,海蛎抱蛋,美剧,旅行,团购,简单,B 型血
娱乐,时尚,投资理财,旅游,健康,美食
美食
娱乐,美食,文艺,星座命理,时尚,IT 数码,游戏动漫
旅游
健康
健康
美女,教育就业,汽车,IT 数码,体育
其他生活
美食
娱乐
时尚,旅游,美女,娱乐,健康
星座命理
美食,健康,教育就业,时尚,旅游,IT 数码,娱乐,体育
星座命理,健康,文艺,时尚,娱乐,音樂,吸血鬼日记,电影迷,时装
游戏动漫
新闻资讯,体育
娱乐
健康,旅游
旅游
旅游,爱觉觉,狂吃不胖,美食
美女,时尚,汽车,娱乐
互粉,减肥,爱美食,喜欢听歌,开朗,旅游,微博奇葩,辽宁生活,美女,IT 数码
娱乐
汽车
旅游,星座命理,时尚,文艺
奋斗,文艺
教育就业,汽车,时尚,IT 数码,娱乐,社会
美女,时尚
新闻资讯,文艺
投资理财
星座命理,娱乐
时尚潮流,街拍,欧美控,天蝎女,旅游,星座命理
旅游,投资理财,汽车,文艺
军事,美女,船东代理,日本线,印巴线,中东线,货代,国际货代,业务新手,求货
IT 数码,美女
淡定,矛盾体

美女
娱乐,IT 数码
健康,娱乐,文艺,美食,星座命理
新闻资讯,健康,时尚,娱乐
娱乐
新闻趣事,美女,娱乐
美食,时尚,美食,健康,教育就业,投资理财
微博奇葩,时尚
名人明星
视频音乐,美女,名人明星,美食,搞笑幽默
健康
微博奇葩,穿衣美容,搞笑幽默,旅游,IT 数码,游戏动漫,汽车,娱乐
旅行
美食,穿衣美容,微博奇葩,湖南生活,新闻趣事
微博奇葩,视频音乐,名人明星,旅游,搞笑幽默,湖北生活
文艺,星座命理,美食,旅游,游戏动漫
星座命理,IT 数码,体育,汽车,时尚
護膚成份,美容护肤,美容保养,護膚,精华素,美丽
娱乐
睡觉睡到自然醒,爱旅行,怀旧,文字,努力,学生
美食,旅游,汽车,军事
娱乐,00 后、爱英语
魔羯座,快乐大本营,爱幻想,爱美食,粤语,开朗
旅游
搞笑幽默
美食,汽车
汽车
白羊女
李锦威,健康,美食,文艺,娱乐,美女,心都凉了
旅游
美食,军事,文艺,投资理财,新闻资讯
文艺,健康,新闻资讯,星座命理,时尚
教育就业,时尚,健康,星座命理,IT 数码
游戏动漫,娱乐
时尚,文艺
健康,文艺,IT 数码,活动策划,微电影,旅游摄影
美女,星座命理,IT 数码
美食
新闻资讯,健康,旅游,美食,娱乐
时尚
小执着,极端
时尚,健康,星座命理,美食,文艺
IT 数码,文艺,军事,美女,游戏动漫,游戏,看书,电脑,dota
星座命理

穿衣美容
穿衣美容,美女,时尚,健康,美食
敢爱敢恨,自由,护士,爱黑夜
体育,游戏动漫
臭美,街舞,音乐发烧友
穿衣美容
电影,抒情,IT 数码,文艺,投资理财,健康,新闻资讯,蝴蝶飞飞
军事,美食,新闻资讯,投资理财,娱乐
名人明星,娱乐
美女,健康,体育
新闻趣事,娱乐
忧郁,矛盾,天蝎座,宅女
星座命理,投资理财,新闻资讯,健康,娱乐
游戏动漫,体育
美食,旅行,善良,网购,偶稀饭睡觉觉
投资理财,金融,学生一族,自由,90 後,梦想,财经,校园,书籍,人生
金牛座,绘画,经济,投资理财
娱乐
娱乐,教育就业,新闻资讯,文艺,旅游,健康
健康,双子座,动漫迷,看小说,爱幻想
动漫,自由,宅,旅游,80 后,美食,IT 数码,文艺,体育,时尚
新闻趣事,读书分享,星座运势,名人明星,视频音乐
投资理财,时尚,IT 数码,体育,汽车
娱乐,健康
美女,文艺,星座命理
美食,旅游,美女,陕西生活
搞笑幽默
旅游,健康
健康,时尚,投资理财,文艺,星座命理
新闻资讯,游戏动漫,IT 数码,文艺,娱乐
文艺,古钱,历史文化,收藏
文艺,娱乐
IT 数码
IT 数码,美女
爱无情
娱乐
娱乐
旅游,文艺,娱乐,教育就业
上海生活
汽车
星座命理
星座命理,美食,旅游
健康,文艺
星座命理

美食,微博奇葩,搞笑幽默,旅游
游戏动漫,IT 数码,文艺
文艺
孙静雅,内涵,腐女,XXOO,90 后
IT 数码
名人明星
星座命理,时尚,美女,美食,文艺
美女,体育
星座命理,旅游,体育,娱乐,IT 数码
视频音乐,浙江生活,穿衣美容
大爱男神,学生,作者,水瓶座,文字
娱乐,新闻资讯,教育就业,军事,星座命理
美女,娱乐,汽车,体育,文艺
游戏动漫,军事,文艺,体育,娱乐
投资理财,文艺,金融,创业,哲学,自由,电影,财经,房地产,编辑出版
校园生活
旅游,美食,健康,时尚,文艺
IT 数码,美食,旅游,健康,美女
美食,IT
健康,游戏动漫,旅游,文艺,娱乐
文艺
美女,美食,旅游,游戏动漫,体育
成都生活
星座命理,旅游
美食
k 歌～看电视～
彩妆,时装,服装设计,旅游摄影,音乐爱好者,电影控,时尚,美食,星座命理,文艺
海东生活
二缺
软件,投票,热门事件,QQ,娱乐
娱乐
时尚,星座命理
教育就业,美食,健康,旅游,宅女
科学探索,娱乐
萌宠
IT 数码
旅游,游戏动漫,时尚,汽车,娱乐
旅游
文艺
名人明星,八卦杂谈
玩,快乐,爱笑,军事,新闻资讯,时尚,文艺,娱乐
摩羯,90 后非脑残,好书,看书,大学生,学生一族,music,双重性格
美女,军事
90 后、

视频音乐,旅游,名人明星,美食
网络,新闻,健康,军事,美女
校园生活,军事
资阳生活,宅,天秤
旅游
个性,青春,瘦身
健康,新闻资讯,美食,投资理财
时尚,文艺,新闻资讯,旅游,投资理财
文艺,娱乐,美食,旅游,健康,新闻资讯,时尚,教育就业
时尚,星座命理,健康,美食,美女
娱乐,90 后,开朗,军事
文艺,健康,教育就业,美食,旅游
IT 数码
美女
体育
搞笑幽默
90,开心,懒人,90 後,吃喝玩乐,团购,好性格,巨蟹座,喜欢旅游,看电视
美食,文艺,新闻资讯,健康,投资理财
文艺,IT 数码
艺术,旅游
娱乐
银饰品,星座命理,新闻资讯,美女,IT 数码,时尚
美食
IT 数码,投资理财,汽车
美食,娱乐
旅游,时尚,文艺,娱乐
名人明星,旅游,视频音乐,新闻趣事,美食
搞笑幽默,时尚
安静,英语,文字,旅游
体育,打篮球
新闻资讯,时尚
偶稀饭睡觉觉,健康,90 后非脑残,北京
旅游,星座命理,美食
投资理财,体育
绘画,建筑,天蝎,娱乐,美食,美女,游戏动漫
投资理财,旅游,军事,IT 数码,体育
睡懒觉,吃和睡
读书分享,美图摄影,美容服饰,旅游,穿衣美容,星座命理,视频音乐
搞笑幽默
美女,IT 数码
情感生活,八卦杂谈,微博控,80 後,职场
HXY4949,294,95,笑起来就这样
时尚
美食,健康

健康
名人明星,文艺,军事,IT 数码,体育
文艺
新闻资讯,娱乐
美食
星座命理,美女,健康,时尚,投资理财
河南生活
娱乐
游戏动漫,旅游,军事
文艺青年
投资理财,汽车,健康,美女
美食
新闻资讯,文艺,体育,娱乐,军事
星座命理,汽车,美食,健康,IT 数码,军事
美食,时尚,娱乐,文艺,旅游
搞笑幽默
星座命理,IT 数码,体育
时尚
娱乐
美食,八卦杂谈,旅游,其他生活,搞笑幽默
视频音乐
健康,新闻资讯,体育
时尚,汽车,教育就业
汽车,星座命理,美食
教育就业,星座命理,文艺
时尚,教育就业,星座命理
娱乐
IT 数码
体育
教育就业,健康,时尚,体育,星座命理
美图摄影,读书分享,搞笑幽默,视频音乐
娱乐
新闻资讯
美食
娱乐
学生,爱睡觉,爱旅游,山东生活,湖南生活
游戏动漫,新闻资讯,投资理财,娱乐
心情
时尚
新闻趣事
娱乐
IT 数码,阅读,美剧,手机,电影,听音乐,科技
视频音乐,旅游
胡思乱想

娱乐,数学,旅行者,蜘蛛纸牌,软件开发,探索,历史文学,科幻电影
旅游,体育
汽车
快乐,美术,90
星座
美食,旅游,健康
IT 数码,娱乐,美女,星座命理
汽车,健康,旅游,IT 数码,娱乐
娱乐,旅游,健康,美食
投资理财,IT 数码,新闻资讯
娱乐,投资理财,星座命理,美食,美女
一路向西
时尚
慢热大猫酸辣控
星座命理,娱乐,教育就业,新闻资讯,旅游,投资理财
幼教，舞蹈,梁耀燮,教育就业
畫畫,二次元症候群,動漫,腐宅双修,甜食狂热分子,抹茶控,咖啡,旅游,摄影
名人明星,搞笑幽默,微博奇葩,穿衣美容
耽美,攻控,资源强迫症,既宅又腐,中抓控
旅游
美食
教育就业,汽车,IT 数码
投资理财,健康,教育就业
汽车,文艺,娱乐
交友,运动,乐观,宅女
新闻资讯
名人明星
汽车,时尚,投资理财,旅游,教育就业
时尚,娱乐
时尚
美瞳,假发,手机壳 DIY,芭比娃娃,指甲油,假睫毛,口红,手包,BLINGBLING,骷髅
热爱生活,财经,情绪化,打篮球,书,体育,投资理财
旅游,文艺,体育,娱乐
搞笑幽默,旅游,美食,星座命理,视频音乐
娱乐
动漫迷,内向,海贼王,学生
游戏动漫,旅游
旅游,萌宠,穿衣美容,减肥,时尚,娱乐,星座命理,美食,健康
美食,星座命理,娱乐
美食,IT 数码,文艺,吃吃，睡睡，玩玩
旅游,美食,军事,娱乐,汽车
娱乐
投资理财,新闻资讯
美女

美女,陕西生活,搞笑幽默,汽车,军事,IT 数码
穿衣美容
娱乐
美食,时尚,居士服,茶叶,燕窝,虫草,沉香,饰品,佛教用品
娱乐
爱绝成痛,至爱成殇
健康,旅游,汽车,娱乐,体育
星座命理,微博奇葩,新闻趣事,搞笑幽默,广州生活,汽车,天蝎座
军事,教育就业,星座命理,时尚,健康,美女
星座命理
游戏动漫
搞笑幽默,娱乐
IT 数码,体育
美食,爱笑,吃货,夜猫子
旅游,游戏动漫,西西里岛,爱坚果爱妖孽,双子座,宅女加腐女
搞笑幽默,读书分享,星座运势,三亚生活,美食
旅游,投资理财,体育
投资理财,教育就业,星座命理,美食,旅游,书籍,睡懒觉,人生,布丁
新闻资讯,IT 数码
运动,90 後,打球,体育
周杰伦,90 后,吃货,摩羯,睡觉睡到自然醒
娱乐,星座命理,健康
娱乐
体育,游戏动漫,旅游
吴亦凡,IT 数码,文艺,美食,旅游,游戏动漫
娱乐
搞笑幽默,抽奖控,小米,我就是我,学生一族,手机,亲情,音乐爱好者,娱乐
娱乐
旗袍,旅游,投资理财
体育,军事,美食
IT 数码
美女,美图摄影,体育资讯,视频音乐,搞笑幽默,美食,体育,娱乐
读书分享
时尚,娱乐,健康,美食
游戏动漫
体育
旅游,军事,汽车
美食
美女,80 后
自由
阳光,安静,简单,粉红控,文艺
搞笑幽默,朱元冰
时尚,90 后非脑残,恐怖片,偶稀饭睡觉觉,b 型血,孩子气
娱乐

时尚,美食
IT 数码
美食
娱乐,文艺,美食,兽医，护理宠物,贪吃，贪玩，贪睡
娱乐
旅游,文艺
娱乐,旅游,美食,文艺
星座运势
时尚
游戏动漫,星座命理,文艺
美女,军事,体育
时尚,教育就业,文艺
投资理财,美食,IT 数码
IT 数码,汽车,旅游,体育,娱乐
搞笑幽默,微博奇葩,旅游,美女,穿衣美容
美食,浪漫,90 后非脑残,独立
星座运势
美食
IT 数码,娱乐,美食,投资理财,教育就业,健康,旅游
旅游
娱乐
游戏动漫
体育
文艺,时尚
体育,美女,游戏动漫,健康,IT 数码
教育就业,文艺,汽车,星座命理,IT 数码
时尚,妖精
美食,娱乐
新闻资讯
IT 数码
游戏,足球,历史,军事
广东生活,娱乐
粉红控,爱唱歌,Music,文字控,爱旅行,多愁善感
美食,游戏动漫,旅游,文艺,星座命理,IT 数码
文艺,美食,娱乐
新闻资讯,投资理财,旅游,IT 数码,娱乐
新闻资讯,旅游,美食,体育,娱乐
广东生活,游戏动漫
美女
北京生活,娱乐
星座命理,教育就业,投资理财,健康,旅游,文艺,美食
投资理财,星座命理
甘肃生活,娱乐
娱乐

美女
K 歌之王
时尚
游戏动漫
时尚,汽车,体育,IT 数码
文艺,美食,旅游,健康,时尚
烹饪艺术家
微博奇葩
娱乐,汽车,时尚,美女,IT 数码
旅游
美食,金在中,娱乐
搞笑幽默,娱乐
游戏动漫
视频音乐,旅游,萌宠,文艺,健康,投资理财
汽车,美女,游戏动漫,军事,娱乐,旅游
成都生活,校园生活,搞笑幽默,新闻趣事
投资理财
娱乐
视频音乐
新闻资讯,投资理财,时尚,娱乐,健康
美食,活泼开朗,执着,单身,天秤,男人,购物狂,爱美食,心情,花痴
广东生活,体育
旅游,煲剧
娱乐
搞笑幽默
懒人,销售,淘宝控,宅女一枚,手机看小说,花痴,90 后但我不脑残,独立,韩国,娱乐
美女
文艺,星座命理,时尚,娱乐,投资理财
任性,旅游
娱乐,时尚,文艺,美女
人生
穿衣美容,名人明星,美食,萌宠
美食
健康,星座命理
新闻趣事
IT 数码
投资理财,文艺,教育就业,游戏动漫,秦时明月,九州
游戏动漫,微笑,爱旅行,军事,新闻资讯,IT 数码
美食,游戏动漫,IT 数码,美女
旅游
娱乐,星座命理
军事
汽车,军事,旅游,新闻资讯,投资理财
教育就业,体育,美食

八卦杂谈,福州生活,美女,搞笑幽默
喜欢听歌,旅行,美食,美剧迷,恐怖片,爱幻想,购物狂,陈奕迅,猫咪控,重口味
时尚
穿衣美容,美食
投资理财,教育就业,新闻资讯,军事,IT 数码
遊戲動漫
IT 数码
娱乐
游戏动漫,时尚,娱乐,星座命理,美食
美食
娱乐,军事,新闻资讯,汽车
旅游,投资理财
搞笑幽默,游戏,摩羯座,微博,NBA,汽车,新闻资讯,军事,IT 数码,体育
HipHop,街舞,音乐,说唱,时尚,90 后,电影,星座,娱乐,美食
购物狂,星座命理,手机
HipHop,街舞,音乐,说唱,时尚,90 后,电影,星座,娱乐,美食
HipHop,街舞,音乐,说唱,时尚,90 后,电影,星座,娱乐,美食
穿衣美容,娱乐
时尚,文艺,娱乐,健康
HipHop,街舞,音乐,说唱,时尚,90 后,电影,星座,娱乐,美食
HipHop,街舞,音乐,说唱,时尚,90 后,电影,星座,娱乐,美食
娱乐
新闻资讯,时尚,体育,文艺,娱乐
美女,美食,体育
对你微笑,纯属礼貌
HipHop,街舞,音乐,说唱,时尚,90 后,电影,星座,娱乐,美食
文艺,IT 数码,汽车,时尚,旅游
体育
娱乐
娱乐
美食,旅游,星座命理,新闻趣事,搞笑幽默,周杰伦,简单难懂,看书　睡觉　旅游
吃货一枚
美食
时尚,美女,新闻资讯,文艺,教育就业,星座命理,军事,汽车,旅游,IT 数码
搞笑幽默,培训,运动爱好者,读书摄影旅游,投资理财,健康,新闻资讯,军事
美食
美食,娱乐,文艺
双子女,旅游,美食
自由,宅,80 后,天蝎座,无聊,热情,傻,美女
娱乐
IT 数码,军事
教育就业,旅游,美食,IT 数码,时尚
双鱼女,无厘头,爱笑,玩,快乐
娱乐,谢娜

美女
小说,音乐,电影,动画,敏感
搞笑幽默
美女,自拍,淫妻,3P,绿帽,丝袜
体育
汽车,投资理财,旅游,健康,教育就业,美食
教育就业,以书会友,读者,广西建院,交友,读书爱好者,读书
游戏动漫,旅游,美食,娱乐,发呆,吃货,怀旧
穿衣美容
健康
开朗,思考,成熟,爱旅行,留学,梦想,独立,热情,自信,学习
教育就业,美食,旅游,时尚,娱乐
星座运势
娱乐,时尚,旅游,星座命理,美食
娱乐,游戏动漫,体育,學習、運動、娛樂
文艺
咖啡
汽车
健康,小女人,摄影爱好者,爱玩,IT 数码
名人明星
IT 数码
新闻资讯,时尚,健康,美食,旅游
新闻资讯
汽车,定向越野,自行车,轮滑
美女,汽车,旅游,时尚,星座命理,IT 数码
体育
美食
教育就业,旅游,军事,美食,文艺,星座命理
时尚,娱乐
星座命理,爱睡觉,白羊
狐狸君,B 型血,旅游,音乐控,陈奕迅,文艺,选择困难症,天秤女
时尚,旅游,星座命理,美食,娱乐
游戏动漫,体育
汽车,时尚,新闻资讯,健康,星座命理
游戏动漫,美食,旅游,汽车,IT 数码,娱乐,星座命理
乐橙,温柔
hold 不住,爱笑,娱乐,段子,趣图,搞笑,幽默,语录,冷笑话,80 后
活泼、幽默
投资理财,文艺,宣纸刺绣,玉玺牌
新闻资讯
健康,投资理财,美食,IT 数码,娱乐
旅游,投资理财
时尚,汽车,娱乐
HipHop,街舞,音乐,说唱,时尚,90 后,电影,星座,娱乐,美食

移动应用产品,数据采集终端,智能终端,视频监控,RFID,智能卡,云计算,物联网产业
穿衣美容
新闻资讯,星座命理,IT 数码
文艺,军事
会计
健康,时尚,IT 数码
HipHop,街舞,音乐,说唱,时尚,90 后,电影,星座,娱乐,美食
美食,旅游,IT 数码,娱乐,文艺
校园生活
美女,游戏动漫
游戏动漫,美食,IT 数码
游戏动漫,体育,文艺,娱乐
健康,旅游,文艺
游戏动漫,时尚,投资理财,星座命理,美食
时尚,健康,美食,看电影,幸福,爱吃,浪漫,紫色,喜欢音乐,看小说
时尚,健康,微笑,温柔,旅游美食
文艺,旅游,美食,时尚
体育
旅游,读书分享,情感生活,车世界,IT 数码
博客,听音乐,美食
游戏动漫,美食,军事,娱乐,文艺
时尚
游戏动漫,娱乐
微博奇葩,美女,新闻趣事,湖南生活
健康,时尚,教育就业,爱旅游,休闲,街拍,游泳,开朗,爱音乐,爱自由
个性杀手
体育,體育,軍事,健康,投資理財
名人明星,美容服饰,科学探索,花样美男,美图摄影,文艺,旅游,教育就业,娱乐,美女
旅游,美食,星座命理,穿衣美容,微博奇葩
军事
新闻趣事
视频音乐
文艺,IT 数码,健康,时尚,文字控,文字
香港生活,名人明星
娱乐,体育,美食,旅游,汽车
穿衣美容,神经大条
健康,美食,新闻资讯
摄影控,美食控、爱旅游、
其他生活,时尚
穿衣美容,sleeping
投资理财,时尚,娱乐,文艺,健康,美食,旅游
文艺,旅游
时尚,美女
90 后,淡定,幽默

汽车,军事,IT 数码
娱乐
视频音乐,时尚,搭配,美容化妆,美女,购物,职场女性,求关注
视频音乐,搞笑幽默,新闻趣事,穿衣美容,星座命理
体育,hiphop,数码
新闻趣事,搞笑幽默,视频音乐,穿衣美容,娱乐
投资理财,美食,军事,文艺,娱乐
旅游
新闻资讯,健康
美食
穿衣美容
西安生活,那一抹微笑
新闻趣事,健康
旅游，小说，
广东生活,基督徒,技术控,时事评论,美食爱好者,旅游爱好者,古典音乐,外语爱好者,跑步爱好者,骑行爱好者
婚纱摄影,旅游,美食,投资理财,时尚
娱乐
新闻趣事
睡觉,旅游,发呆,上网
广州生活,it,Music
娱乐,旅游
汽车,娱乐,文艺,美食,旅游
时尚,星座命理,投资理财,文艺,教育就业,旅游
娱乐
superjunior,魔域,o 型血,TVB,梁静茹,少女时代,梅西,沈昌珉,山下智久,体育
星座命理,考神,00 后,波蜜,小清新文艺范,双重性格,水瓶,蓝色性格
娱乐
旅游
美女
美食,时尚,教育就业
汽车,星座命理,娱乐
时尚,旅游,美女,健康,文艺
游戏动漫,投资理财,健康,IT 数码
健康
新闻资讯,投资理财,时尚
名人明星
杀漠粉,宅舞,略完美主义,只能出妹纸的货,古风万年坑,二逼,绘画,BJD,coser
时尚,投资理财,旅游
美女
教育就业,投资理财,军事,IT 数码,文艺
健康,旅游,天平座,看书,体育,教育就业,IT 数码
美食,粤华,礼物,格子铺,北师珠海
美女

时尚,星座命理,旅游,美食,娱乐
旅游
画画,看书,养动物
美食
旅游
美食,星座命理,IT 数码,时尚,娱乐
投资理财,健康
文艺
泉州生活,旅游,白羊,美食控,偶稀饭睡觉觉
星座命理,投资理财
娱乐,重口味
新闻资讯,文艺,健康,教育就业
睡觉,宠物,大叔控,爱唱歌,美食控,购物狂,康熙来了,吃货一枚,滑雪,喜欢听歌
新闻趣事
美食
娱乐,IT 数码
吃喝玩乐,活泼,音乐控
旅游,游戏动漫,爱摄影,IQ160,爱旅游,90 后但我不脑残
新闻趣事
美食,旅游
体育
健康,时尚,星座命理,美食,娱乐
旅游,健康
星座命理,搞笑幽默,微博奇葩,穿衣美容,美食,文艺,娱乐,时尚,美食
美女,健康,军事
旅游
星座命理,美食,旅游,时尚,喜欢音乐,吃喝玩乐,爱生活,潮流,随性,生活
穿衣美容,完美主义,85 后,海绵宝宝,双鱼座女生,燕姿迷,甜食爱好者,增肥党,向日葵
旅游,时尚,投资理财,娱乐,IT 数码,宅汉子
娱乐,美食,时尚,健康,旅游
搞笑幽默
健康,新闻资讯,教育就业,旅游,星座命理,法国,随性
美女,旅游,IT 数码
投资理财
美图摄影,搞笑幽默,IT 数码
IT 数码
苦行僧,全职下岗大学生,青春路,互联网,chinubbs,高校,门户,民工,ITer,论坛
教育就业,星座命理,美食
搞笑幽默,宅
美图摄影,情感生活,重口味,八卦杂谈,校园生活
文艺
校园生活
笑话、美食、女生
娱乐

美食
美女,美食,健康,星座命理
新闻资讯,游戏动漫,星座命理
娱乐
视觉传达设计,文艺,时尚
星座命理,文艺,健康,美食
美食
赚钱,it,执着,聊天,自由,旅行,美食控,单身,85后,美食
旅游,视频音乐,新闻趣事,搞笑幽默,穿衣美容
文艺
娱乐
时尚,娱乐,健康,旅游,美食
房产,旅游
美女,睡懒觉
娱乐,军事,IT数码,新闻资讯,投资理财
游戏动漫
黏人，舞蹈，医学
文艺,左撇子,感觉几好的,右脑发达,非攻即受
时尚,健康,旅游,美食,娱乐
视频音乐,体育
汽车,美食,IT数码
时尚,娱乐
时尚
天蝎女,90后,时尚
文艺
美食
美女
IT数码,美食,旅游,星座命理,唯爱家族的宣宣,良民LM,杨幂的蜜蜂,时尚潮流,宅女一枚,影视
星座命理,微博奇葩,美女,新闻趣事,搞笑幽默
娱乐,美食
美食,投资理财,时尚
娱乐
娱乐
随性
娱乐
娱乐
健康,旅游
视频音乐,美食,新闻趣事,搞笑幽默,武汉生活,娱乐
旅游
投资理财,IT数码,娱乐,美女
福建生活,娱乐
美食
新闻资讯,教育就业

名人明星
旅游
淘宝
教育就业,时尚,健康,旅游,文艺,我喜欢带着你玩
IT 数码,osu,格斗游戏,学生,東方,宅
和朋友爬山
电视,80 后宅女,互粉互注,爱美食,电影迷,化妆品,网购,狮子座,音乐控,服装
大业兴房产,90 后但我不脑残,单身,阳光,做梦,白羊,善良,小资
新闻趣事,美食,星座命理
旅游
旅游,文艺,IT 数码,汽车
游戏动漫,音乐迷
小小哈,爱老爸,爱老妈,何 B 仔,上帝之手
旅游,文艺
IT 数码
旅游,时尚,新闻资讯,文艺,美食
军事,美女,娱乐,IT 数码,体育
游戏动漫,时尚,体育,美食,军事,娱乐
游戏动漫,军事,娱乐
美食,IT 数码,娱乐,时尚
健康,美女,投资理财
美女,时尚,军事
新闻资讯,星座命理,IT 数码
美女,游戏动漫,时尚
美图摄影
IT 数码
文艺
时尚
新闻资讯
美食,健康,学养生,小小资,爱漂亮,是吃货,望美满
娱乐,文艺,IT 数码,美食,游戏动漫,星座命理
视频音乐,教育就业
美食
书籍,舞蹈,财经资讯,旅游美食,梦想,军事,教育就业,投资理财,时尚,健康
星座命理
德国
游戏动漫,星座命理,时尚,美食,旅游
IT 数码,娱乐
美食,娱乐,游戏动漫
旅游,美食,投资理财,文艺
教育就业
时尚
新闻资讯,教育就业,投资理财,健康,娱乐
美食

心情,创意,阳光
手卷烟,脑残志坚,囤货控,拉子,T
娱乐,旅游
旅游
旅游
生活，
娱乐
美食
正太
IT 数码
西安
IT 数码
校园生活
搞笑幽默
旅游,时尚
IT 数码,游戏动漫,旅游,美女,体育,汽车,美食,健康,教育就业,军事
汽车,美女,旅游,IT 数码,体育
旅游,健康,美食,神经，没心没肺
文艺
娱乐
娱乐
娱乐
娱乐
娱乐
娱乐
娱乐
IT 数码,美食
旅游,IT 数码,游戏动漫,新闻资讯,军事,时尚
汽车,投资理财,文艺
搞笑幽默
美食
名人明星,视频音乐,搞笑幽默,星座命理
时尚,教育就业,文艺,旅游,星座命理
旅游
星座命理,IT 数码,汽车
名人明星
娱乐
网编,传媒,品牌,域名,SEO,站长,公关,管理,广告,营销
微博奇葩
汽车,旅游
IT 数码
美女,星座命理,美食,IT 数码,体育
文艺
美食,电视剧,旅行,动漫,上网,学生,宅,喜欢听歌,摩羯座,读书

美食,睡觉,学生,爱吃,聊天,胡思乱想,朋友,睡懒觉,巨蟹,疯子
旅游,美食,山东生活
旅游
体育
时尚,旅游,健康,美食,文艺
励志,时尚,美女
新闻资讯,健康,美食,旅游,星座命理
旅游
文艺,娱乐,旅游,投资理财
旅游
IT 数码
投资理财,旅游,美食,军事,IT 数码
旅游,教育就业,投资理财,娱乐,体育
2013 为梦想而战
投资理财
美食
体育,娱乐,IT 数码,旅游
游戏动漫,教育就业,IT 数码
旅游,IT 数码,娱乐
时尚,美女,教育就业,90 后,没有了啦
旅游
哼哼哈嘿
投资理财,旅游,星座命理,美食,军事,IT 数码,健康,新闻资讯,娱乐
美食
游戏动漫,投资理财,旅游
搞笑幽默
爱自由,创意,星星,动漫迷,柯南,金牛座,服饰,耽美,F1,吸血鬼
独立
美食,健康
情感生活
游戏动漫,IT 数码,娱乐
汽车
旅游,时尚,文艺,娱乐
旅游
时尚,体育,汽车,投资理财,军事,星座命理,IT 数码,娱乐
跆拳道,体育,白羊,火影忍者,足球是我的最爱,tank,旅游摄影,暮光之城,爱唱歌
美女,旅游,美食,IT 数码,娱乐
winnie,4836,yellow,爱时尚,爱电影,极简,陈奕迅,懷舊,RMB
旅游
穿衣美容,搞笑幽默,星座命理,旅游,美食
军事
文艺
健康,美食,养野生土蜂,农村养殖,美容护肤,美容养生
娱乐

教育就业,时尚,健康,旅游,美食
新闻趣事,名人明星,河北生活,视频音乐,美女
CG
旅游,旅游摄影,巨蟹
旅游,教育就业
美食
健康
美食,旅游,美女,汽车,军事
名人明星,美食,旅行,处女座
终极一班 2,90 後,爱美食,宅,吃货,上网,学生,追偶像剧,爱交友,萌
旅游
视频音乐,健康,IT 数码,文艺
星座命理,美食,军事
IT 数码,健康,旅游,体育
新闻资讯,健康,美女,汽车,旅游,美食,IT 数码,娱乐,体育
星座命理,游戏动漫
健康,娱乐
健康
体育,汽车,娱乐,投资理财,旅游
萌宠
旅游,说啥呢,地产,音乐发烧友,管理,思考,摄影控,咖啡控,旅游美食
美女,旅游,微博奇葩,搞笑幽默,视频音乐,新闻资讯,健康,文艺,IT 数码
健康,汽车,新闻资讯
90 后非脑残,宅女加腐女,爱美食,学习,星座命理,爱音乐,处女座,小文艺,活泼,可爱
梦想, 幸福, 娃娃
旅游,文艺,健康,美食,娱乐
户外,团购,体育
娱乐,时尚,健康,旅游,美食,文艺
新闻资讯,教育就业,文艺
娱乐,美食
文艺
健康,美食
美女,旅游,美食
美食,文艺
美女,娱乐
穿衣美容,名人明星
美女,游戏动漫,时尚
文艺,巨蟹座,腐女,吃货一枚,大叔控,偏执狂
美女
美女,新闻资讯,时尚,体育
教育就业,时尚
自然,hiphop,好性格,阅读,情绪化,科技,爱生活,创意,摄影爱好者,爱情
体育
星座命理,活泼乐观爱说话

搞笑幽默
旅游,汽车
新闻资讯,教育就业,美女,星座命理,IT 数码
游戏动漫,汽车,文艺
美女,新闻资讯,星座命理,IT 数码
美食
美女,时尚,IT 数码
文艺,旅游,星座命理,美食,娱乐
时尚,投资理财,文艺
名人明星,搞笑幽默
美食
IT 数码
投资理财,旅游,军事,文艺,娱乐
游戏动漫
微博奇葩,新闻趣事,搞笑幽默,健康,星座命理,旅游,新闻资讯,文艺
旅游
娱乐,时尚,美女,美食,体育
IT 数码
台湾,钓鱼岛,解放军,舆论战,舆情,军事
娱乐
视频音乐,文艺
德国生活,新闻趣事,穿衣美容,教育就业,时尚
美食,旅游,文艺,体育,娱乐
旅游
军事
娱乐
美食,投资理财
广东生活
文艺,时尚,新闻资讯,投资理财,教育就业
美食
美食,旅游,文艺
旅游
时尚,投资理财,娱乐,旅游,新闻资讯
减肥永不成功党,天蝎女,购物狂,爱美食
文艺,体育
娱乐
娱乐
宠物图集
摩羯座
游戏动漫,IT 数码,体育
娱乐
旅游,娱乐
旅游,美食,时尚
旅游,健康,美食

江西宜春,教育就业,投资理财,娱乐,IT 数码,新闻资讯
娱乐,星座命理
娱乐,健康
娱乐,美食
娱乐
旅游
美食,旅游,娱乐
健康,美食,娱乐,时尚
爱电影,旅游,文艺
旅游,新闻资讯,健康,时尚,投资理财,美食
娱乐
旅游,美食,体育,文艺,新闻资讯,IT 数码
娱乐,健康,投资理财,旅游,美食
军事,体育
无厘头,快乐,美食,宅,旅游,交朋友
旅游,美容服饰,星座运势
新闻资讯,军事,娱乐,游戏动漫,体育
旅游,娱乐
名人明星,搞笑幽默,视频音乐,汽车,美女,IT 数码
时尚,旅游,星座命理,新闻资讯,娱乐
美食,活泼开朗,吃货一枚
IT 数码
军事,体育
流浪,旅游,健康
體育,娛樂,IT 數碼,時尚,汽車,教育就業,投資理財
汽车,娱乐,旅游,IT 数码,体育,我就是我,天平座,90 后但我不脑残,music,相声
爱睡觉,自拍,重口味,喜欢旅游,美剧控,麦霸,陈奕迅,中医,投资理财,化妆品
美食
健康,美食,星座命理,游戏动漫,娱乐
文艺
游戏动漫,IT 数码,星座命理,美女,娱乐
汽车
娱乐
乐橙,搞笑幽默,娱乐,喜欢音乐
美食
迟钝星人,抽风女,睡懒觉的夜猫,发呆 ing,火影,奇葩,CF,吃货
星座命理,文艺,旅游,健康,娱乐
体育,健康
娱乐
搞笑幽默
投资理财
时尚,娱乐,文艺,美食
八卦杂谈
体育,美食

军事
美女
旅游摄影,胡思乱想,爱自由,简单,爬山
搞笑幽默,视频音乐
旅游,时尚,娱乐
自由职业,数码,时尚,旅游,音乐
旅游
IT 数码
旅游
星座命理,体育,IT 数码,游戏动漫
旅游
吸血鬼日记,医学,自信,看电视
星座命理,文艺
美食,健康,投资理财,旅游,军事
IT 数码,时尚,健康,旅游,文艺,投资理财
游戏动漫,投资理财,IT 数码
江西生活,投资理财,文艺,教育就业
娱乐,汽车,IT 数码,时尚
健康
八卦杂谈
旅游,健康,投资理财
文艺
新闻资讯
娱乐,体育
爱旅游,神经质,90 後
时尚
娱乐,体育,beyond,黄家驹,黄贯中,黄家强,叶世荣,摇滚,音乐
星座运势,情感生活,八卦杂谈,旅游
没节操但善良,带我去旅行,超爱甜食,喜欢音乐,自拍,小洁癖,强迫症,龙山人,狮子女,英语
90 后,宅女,美食,购物,听音乐
健康,旅游
美食,游戏动漫
视频音乐
游戏动漫
游戏动漫,旅游,爱看动漫,慢性格、爱爬山,爱旅游
健康,美食
娱乐,时尚
旅游,游戏动漫,美女,文艺,军事
旅游,娱乐,文艺,军事,IT 数码
旅游,美食,娱乐
旅游
时尚,娱乐
健康,时尚
体育,天枰座

投资理财,旅游
新闻资讯,文艺,教育就业
佛法,大乘,密宗,保险理财,财经,亲子共同成长,瑜伽
娱乐
文艺
文艺
美容服饰
健康,娱乐,星座命理,文艺
IT 数码
教育就业,军事,文艺
旅游,日剧,猫咪控,天枰座,宅女加腐女,温柔,吃货一枚,时装,性感
美食,旅游
新闻资讯,星座命理,旅游,健康,美食,时尚,娱乐
时尚
健康
shopping,台球,多愁善感,90 后非脑残,网购,吃,宠物图集,美食
星座命理,美剧,游戏动漫,美食,娱乐
宅,美食
军事
体育,新闻资讯,文艺
美食,最爱苹果树,zzj 四六货,爱猫爱摄影
双鱼座
娱乐
健康,美女,美食,军事,文艺,妈妈,妈咪,怀孕,育儿
文艺
健康,文艺,教育就业,娱乐
娱乐
娱乐
旅游,健康,娱乐,时尚,美食
旅游,文艺,体育
时尚,IT 数码,旅游,星座命理,娱乐,美女,游戏动漫,健康,美食,军事
随性,双鱼,周杰伦,睡懒觉,篮球爱好者,80 后,DOTA,爱旅行,看电影
时尚
美食,b 型血
穿衣美容
投资理财,旅游,美食,汽车
美女,游戏动漫,汽车,文艺,娱乐,时尚,表演,唱歌
IT 数码,时尚,旅游,娱乐,文艺,美女
福建生活,搞笑幽默,新闻趣事,穿衣美容,戒酒,你没本事不屑,还小,新闻资讯,喜欢自贱,和贱贱的笑
健康
文艺
汽车,美女,体育,睡货!
旅游,美食,想用文字分享,我的感动,再把这感动传递,给下一个,看这些文字的人

文艺,投资理财,旅游,教育就业,体育
星座命理
旅游
投资理财,时尚,文艺,娱乐
爱幻想,文字控
神经大条,情绪化,阿信,安东尼
文艺,娱乐
健康,时尚,旅游,美食,娱乐,广告人
搞笑幽默,体育
旅游
名人明星,搞笑幽默,视频音乐,微博奇葩
IT 数码,文艺,体育,娱乐
白羊女,要奋斗,减肥,热爱生活,重庆,简单
bigbang,处女座,完美主义
狮子座 90 后男生,低调,游戏动漫
星座命理,新闻资讯,体育,IT 数码,健康,文艺,美食,旅游
体育
美食
健康,新闻资讯,游戏动漫,投资理财,美食
军事
星座命理,美食,游戏动漫,文艺
旅游
新闻资讯
健康,教育就业,旅游,体育
娱乐,投资理财,新闻资讯,健康,旅游
美食
投资理财,教育就业,新闻资讯,健康,文艺
文艺,星座命理,B 型血
搞笑幽默,微博奇葩,美女,矮丑龊
美女,时尚,IT 数码
文艺,娱乐
健康,美女,时尚
健康,体育,娱乐
健康,新闻资讯,娱乐
旅游,单身青年,摄影爱好者,户外穷游,户外徒步
上海生活
美食,娱乐
旅游
萌宠,微博奇葩,星座命理,穿衣美容,美食
文艺,美女
星座命理
小清晰,射手女,听歌,旅游
旅游,军事,IT 数码
娱乐,游戏动漫,最爱甜甜的草莓

星座命理,文艺
美食
军事
天津生活
游戏动漫,时尚,文艺,星座命理
汽车,旅游,美食,军事
旅游,智慧女人,成长 ing,幸福 ing,书籍,喜欢旅游,中医,投资理财,化妆品,旅游美食爱好者
游戏动漫
娱乐
美食,旅游,时尚,文艺,美女
新闻趣事
汽车,游戏动漫,IT 数码,体育,娱乐
美食
时尚,美食,星座命理,投资理财
娱乐,美食
美食
搞笑幽默
体育
旅游
IT 数码
美食,时尚
不信,怎么,你,有病,我,说了
巨蟹座,深圳,宅男,羽毛球,高中生,自行车,游戏,音樂,环保
时尚
美女,旅游,汽车,IT 数码,体育
新闻资讯
IT 数码
健康,新闻资讯
其他生活
美食
娱乐,时尚
美女,游戏动漫,体育
汽车,旅游,星座命理,娱乐,IT 数码,投资理财,游戏动漫
星座命理,美食,旅游,投资理财,健康
星座命理,旅游,娱乐,爱旅行,阳光,双子女,喜欢听歌,美食爱好者,热爱生活,活泼开朗
IT 数码,军事,美女
美图摄影,星座运势,旅游爱好者,夜猫子,活泼开朗,忧郁,交朋友,发呆,完美主义,敏感
校园生活,视频音乐,读书分享,90 後,插画,好书,矛盾体
销售,天秤座,外貌协会,旅游摄影
微博奇葩,陕西生活,游戏动漫
健康,美女,游戏动漫
娱乐
游戏动漫
星座命理

搞笑幽默,文艺
名人明星
健康
汽车,投资理财,教育就业
美食,新闻资讯
新闻资讯,时尚,教育就业,旅游,IT 数码,狮子座,爱电影,南京,大学
娱乐
军事,健康,投资理财,新闻资讯
星座命理,娱乐,健康,时尚
时尚,军事,旅游,文艺
游戏动漫,星座命理,军事
Davichi,金俊秀,沈昌珉,运动,吃货,看小说,仙后,爱电影,看足球,看剧
动漫,游戏动漫,小说控!!!!
星座命理
体育
游戏动漫
星座命理
旅游,军事
购物狂
活泼可爱，美容，
篮球
投资理财,美女,文艺,体育
美容服饰
娱乐,上网,看节目,上网 K 歌打台球
时尚,IT 数码,投资理财,星座命理,旅游
绘画,书,大学,写作,美食,文艺
娱乐
体育
健康,新闻资讯,美女,军事
新闻资讯,健康,旅游,星座命理,IT 数码
娱乐
美食,健康
星座命理,体育,娱乐,宅,摩羯座女孩,交朋友,迷糊,学生一枚
搞笑幽默
穿衣美容,萌宠,美食,旅游
搞笑幽默,新闻资讯
美食
驴友,摄影师,美食,IT 数码
旅游
自由职业,搞笑幽默,新闻趣事,旅游,IT 数码
健康,旅游,教育就业,矛盾,双鱼女,幻想,学生一枚
美食
搞笑幽默
星座命理,IT 数码,教育就业,时尚,娱乐,文艺

娱乐,单身,90
穿衣美容
军事,美食,美女,游戏动漫,IT 数码
美食
美女,名人明星,穿衣美容,时尚,娱乐,美食,星座命理
游戏动漫,汽车,体育
学生,摄影,跆拳道,音乐达人,书法,绘画,麦霸,真实力,音乐爱好者,设计师
时尚,美女,军事,体育,娱乐
漫画,低调,睡觉,动漫,自由,音樂,水瓶女
健康,游戏动漫,时尚,娱乐,努力,品牌,美女
美食,执着,90 後,K 歌,购物狂,爱生活,可爱
娱乐
美女
美女,游戏动漫
视频音乐,美女,微博奇葩,星座命理,搞笑幽默
新闻资讯,体育
美食
体育资讯
新闻资讯,健康,美女
体育
健康,娱乐
日语
搞笑幽默,游戏动漫,新闻资讯,狗狗,玩,手机看小说,动漫
新闻趣事
健康
市场营销,爱生活,情感,淘宝,健康养生,购物,真诚,夜猫子
娱乐
教育就业,文艺,投资理财,星座命理,娱乐
美女
美食,时尚,汽车,美女,新闻资讯,星座命理,体育,IT 数码
体育,娱乐
美食,投资理财,教育就业,汽车,健康,旅游
投资理财
娱乐
搞笑幽默,萌宠
娱樂,夜猫子,交友,天蝎座,个性
漫画故事,心情,几米童话,爱情,语录,心情签名,几米漫画,几米,文艺,旅游
旅游,军事,汽车,美女
教育就业,新闻资讯,娱乐,旅游,汽车,美女
EXO
体育
星座命理,情绪化,宅女一枚,双重性格,80 后的尾巴,魔兽,O 型血,恐怖片
美食
美食

闷骚,旅游摄影,科比,听音乐,发呆
星座命理,文艺
游戏动漫
穿衣美容
星座命理,设计,处女座,完美主义,90後,神经质,爱旅游,心理学
新闻资讯
IT数码,娱乐,汽车,军事,体育
军事,投资理财,时尚,美女,游戏动漫,旅游,IT数码,娱乐
健康,投资理财
文艺,美食,旅游,健康,时尚
美食
美食,军事,星座命理,游戏动漫,汽车
娱乐,纠结中…
旅游,娱乐,体育
健康,旅游,体育,美食,娱乐
网络,NBA
美食
旅行,宅女,吃,乐观,90,justinbieber,登山,小女人,爱美食,文艺
美食
美食,流行,音樂,旅遊,書迷,3C,電影,偶像劇,衣著,明星
善良,健康
文艺,IT数码,娱乐
文艺,时尚,健康
宠物
健康
星座命理,游戏动漫
健康,美食,旅游,时尚,文艺
看电影,白羊座,时尚,旅游,美食,健康
美食
星座命理,穿衣美容,健康,时尚
娱乐,白羊,吃
娱乐,IT数码,体育
汽车,新闻资讯,美女,体育,军事
汽车
其他生活,IT数码
户外
美食,美食
体育,娱乐,文艺,美女
时尚,智力竞技,冒险,学习,免费简餐,德州扑克
星座命理,娱乐
搞笑幽默
名人明星
美食,健康,教育就业,旅游
美食

教育就业,旅游,体育,新闻资讯,游戏动漫
文艺,健康,教育就业,旅游,美食,吃货,有一颗旅游的心,我就是我,电影爱好者,哈哈
新闻趣事,搞笑幽默,娱乐
音乐控,养生,浦,求解脱,缺爱
时尚,汽车,美女,游戏动漫,娱乐
时尚,购物,美容,服饰,潮流,折扣,优惠,打折,淘宝,娱乐
体育
旅游,时尚
时尚,教育就业,健康
投资理财,健康,旅游,美食,娱乐
时尚
旅游,健康
娱乐
执着,旅游美食,爱幻想,喜欢听歌,星座控,情绪化,爱睡觉,开朗,时尚,双鱼座
美食,星座命理,游戏动漫,娱乐
新闻资讯,游戏动漫,军事,IT 数码,时尚
健康,爱美食,购物狂
想飞的自由落体
健康,体育,美食,星座命理,游戏动漫
名人明星,娱乐
军事
旅游,健康,文艺,教育就业,体育,美食
投资理财,时尚,美食,星座命理,娱乐,新闻资讯,健康,教育就业
健康,美食,投资理财,美女,汽车
高调做事,低调为人,享受生活,珍惜身边人
美女
健康,旅游
IT 数码,娱乐,新闻资讯,旅游,健康
投资理财
几米,电影,柯南,听歌,哥们,爱女朋友
投资理财
軍事,旅遊,投資理財,汽車,健康
游戏动漫,星座命理
爱自由,摇滚,腐,猫控,驴友,音乐,动漫迷,美食,茶,旅行
体育,军事,美女,汽车,时尚,投资理财,旅游
旅游,娱乐
娱乐
娱乐
古典音乐,随性
金融,IT 数码,music,书,拍照
旅游,投资理财
星座命理
星座命理,健康,娱乐,no,pain,gain,微笑
IT 数码,娱乐

旅游,美女,IT 数码,文艺,娱乐
游戏
娱乐
文艺,时尚,健康,美女,星座命理,娱乐
搞笑幽默,新闻趣事,美食,旅游,美容服饰
美女
健康,按摩垫,按摩器,有位生活,80 后,按摩椅
搞笑幽默,体育
体育,麻将
旅游
娱乐,运动,大自然,创业,收藏,新闻资讯
美食,游戏动漫
健康,旅游,美食,娱乐
杂志,苹果控
美食
摩羯女 O 型
搞笑幽默,娱乐
爱睡觉,淘宝,爱自由,看电影,爱音乐,游戏动漫,旅游,美食,时尚,娱乐
体育,旅游,游戏动漫,投资理财,IT 数码
天秤座,跑步,高中生,奋斗,游泳,减肥
基罗,山狱,JDJC 仗露,盾妮,真凛
深圳生活
搞笑幽默
娱乐,坚持,任性,爱音乐,期待 ing,也可以很安静,活泼开朗,唯爱祥祥
美食
新闻资讯,博半古通半今
娱乐,旅游,美食
汽车
星座命理,教育就业
文艺,教育就业,美食,健康,时尚
都暻秀,劉永才,비에이피
旅游,美食,时尚,娱乐,星座命理
旅行,粤语,阅读
新闻资讯,军事,投资理财,游戏动漫,星座命理,欧美音乐,音乐,动漫
文艺
军事
美女
投资理财,游戏动漫,体育,娱乐
健康
旅游,投资理财,娱乐,时尚
搞笑幽默,娱乐
新闻资讯,健康,时尚,美食,文艺,娱乐
萌宠

娱乐
健康,新闻资讯
美女,90 後,青年
旅游
肇庆生活,搞笑幽默,新闻趣事,微博奇葩,体育,时尚,旅游,文艺,娱乐
搞笑幽默,旅游,体育
搞笑幽默,娱乐
娱乐,美食
健康,美食,旅游,文艺
体育
游戏动漫,星座命理
美食,星座命理,军事,娱乐,文艺
旅游
游戏动漫,时尚,90 后非脑残
名人明星,美女
林宥嘉
娱乐,k 歌,低调,淘宝控,天蝎女,爱玩,iphone,自信,Music
穿衣美容,美女
湖人,吃喝玩乐,个性,真诚,青春,交朋友,电影爱好者,恐怖片,IT 数码,喜欢听歌
IT 数码
新聞資訊
旅游
美女,汽车,投资理财,军事,健康
旅游,汽车
星座命理,美食,娱乐,美女,IT 数码
娱乐
旅游
旅游,唱 k
金融,黄金,股票,投资,投资理财,白银投资
汽车,美女
旅游,美食,穿衣美容
家居,IT 互联网,新闻资讯,设计,营销,市场营销,楼盘,房地产,房产
志愿者,体育
单身
情感生活,育儿百科,美食,新闻趣事,八卦杂谈,美女
新闻趣事,IT 数码,软件开发,软件测试,music,喜欢旅游,体育,互联网
名人明星
萌宠,旅游,搞笑幽默
萌宠
漫画,听音乐,天平座,电影爱好者,情绪化,维尼夫妇,宅,善良,柯南,旅游
娱乐
星座命理,娱乐,时尚,文艺,体育
游戏动漫
健康,时尚,IT 数码,新闻资讯,美女,娱乐,汽车,投资理财,体育

汽车,教育就业,投资理财,IT 数码,新闻资讯,时尚,文艺
穿衣美容,搞笑幽默,星座命理,名人明星,视频音乐
浙江生活,汽车
旅游
爱自己爱生活,美食,时尚,爱玩爱自由,爱他爱生活,各种宅,各种腐,手机控,天蝎座
文艺,时尚,美女,游戏动漫,IT 数码,小忧伤
健康,旅游,星座命理
IT 数码,投资理财,文艺,教育就业,旅游
温柔,听音乐,单身,吃喝玩乐,文化,小清新
哇咔咔
萌宠,微博奇葩,新闻趣事,揭阳生活,时尚,投资理财,文艺,健康
听音乐,奋斗,看电影,文学,美食控
其他生活,IT 数码
娱乐,投资理财,IT 数码,游戏动漫,时尚
汽车
福建生活,娱乐
旅游，吃，工作狂
娱乐
旅游
新闻趣事
星座命理,旅游,文艺
旅游,娱乐
教育就业,口才,心智训练,亲子教育,领导力,企业内训
美女,体育,娱乐,军事,IT 数码
旅游
IT 数码,文艺,教育就业
文艺,新闻资讯,汽车,军事,娱乐
旅游
文艺,羽毛球
娱乐
娱乐,汽车,军事,IT 数码,体育
游戏动漫,教育就业,汽车,文艺,IT 数码
娱乐,时尚,健康
娱乐,时尚,旅游,美食,星座命理
时尚
时尚
美女
美食,文艺,教育就业,健康,旅游,娱乐,游戏动漫
汽车,投资理财,体育
体育
设计,艺术,shopping,時尚,潮,欧美,爱自由,服装,室内设计,美术
美女,健康,军事,IT 数码
旅游,体育
旅游,健康

娱乐
娛樂,體育,熱血,假文青,電影,自由,美食,旅行
娱乐,同志,GAY
校园生活,搞笑幽默,新闻趣事,八卦杂谈,武汉生活,娱乐
娱乐
视频音乐,娱乐
游戏动漫
健康,爱笑,玩
美女,喜欢无拘无束
IT 数码
文艺,新闻资讯,旅游,美食
IT 数码,体育,游戏动漫,投资理财,文艺
时尚,健康
时尚,教育就业,IT 数码,游戏动漫,星座命理,文艺
旅游
体育
時尚,遊戲動漫,健康,新聞資訊,美女
美食,体育,美女,教育就业,IT 数码
游戏动漫,汽车,军事,美女,旅游,文艺
军事,文艺,娱乐,游戏动漫,教育就业,IT 数码
美食,IT 数码,体育,游戏动漫,投资理财,星座命理
新闻趣事
星座运势
美女,旅游,文艺,汽车,星座命理,体育
重庆生活
搞笑幽默
爱生活爱金黎,旅游
美食,IT 数码,体育,汽车,教育就业,文艺
文艺
体育,娱乐
影视
健康
美食
旅游,星座命理,贵州生活
美女
游戏动漫,教育就业,星座命理,新闻资讯,美食,文艺,美女,汽车,IT 数码
美女,美食,体育
美食
美女
游戏动漫
90,流浪,旅游
爱旅游
自由职业,上网
新闻趣事,搞笑幽默

IT 数码
微博奇葩,视频音乐,穿衣美容,美食,名人明星
娱乐
旅游,IT 数码,教育就业,新闻资讯,文艺
美女,旅游,星座命理,美食
娱乐
微博奇葩,四川生活
搞笑幽默
IT 数码
娱樂
萌宠,旅游,美女,微博奇葩,搞笑幽默
重口味,文字控,90 后
旅游,时尚
名人明星,娱乐
旅游
汽车,体育
美容护肤,水瓶
新闻资讯,娱乐,旅行,写作,广电,新闻系,漳州师范,睡懒觉,80 后,天蝎座
健康
旅游,星座命理,时尚,美食
IT 数码,汽车,娱乐
文艺,新闻资讯,游戏动漫,投资理财,美食,军事,体育
广东生活
美食,文艺,新闻资讯,军事,美女,游戏动漫,星座命理,时尚,教育就业,体育
教育就业,美食,文艺,娱乐
军事,汽车
美女,旅游
星座命理,文艺,k 歌,摄影,看书,睡觉,cosplay,动漫控,喜欢听歌,爱自由
睡觉睡到自然醒,瓶邪,小说,音乐控,猫咪,射手女,懒人,90 後,学生一枚,游戏动漫
游戏动漫
美食,娱乐,时尚
校园生活
旅游
美食
文艺
视频音乐,美女,时尚,汽车,军事,娱乐,宅、睡、听歌
娱乐
喵教,基三,八块腹肌,巨人小天使,艾利,cos
养生,美食,新闻趣事,搞笑幽默,其他生活
美食,b 型血
天蝎,搞怪,唱 K,O 型血,宅,爱睡觉,可爱,90 後,爱玩,娱乐
星座命理,健康,时尚,旅游,美食
微博奇葩,星座命理,美女,穿衣美容,新闻趣事
艺术,旅游摄影,英语,爱生活,创意,我就是我,自信

汽车
娱乐,文艺,军事
文艺,IT 数码
旅游,广东生活,娱乐
穿衣美容,时尚
文艺,娱乐,体育,游戏动漫,漫画,吉他,动漫迷,音乐迷,留学,吃喝玩乐
文艺
武汉生活,旅游,视频音乐,八卦杂谈,搞笑幽默
美女,游戏动漫
教育就业,投资理财,娱乐,体育
军事
星座命理,文艺
投资理财,旅游,IT 数码,娱乐,时尚
娱乐
健康,汽车,旅游,美食,体育
美食,游戏动漫
旅游,美女,美食,投资理财,文艺,京华时报,艺术品投资周刊
美食
美食
旅游,美食,文艺
体育
IT 数码,教育就业,美女,娱乐
投资理财,时尚
投资理财
游戏动漫,IT 数码,美食,汽车,体育
美食
星座命理,90 后,O 型血,双子女
教育就业
IT 數碼,汽車,娛樂,汽车,IT 数码,文艺,美食,军事,体育,娱乐
星座命理,浙江生活,视频音乐,新闻趣事
体育,监理 121
时尚
搞笑幽默,视频音乐,星座命理,游戏动漫,美食,娱乐
戏,书,行走的力量,粤语,电影旅行小情绪,瓶邪小哥长白山
美食,音乐,旅行,电影,台球,留学
娱乐
文艺
美食,星座命理
西安生活
美食
时尚
游戏动漫
娱乐,美食,健康,旅游
文艺

90 后但我不脑残,搞笑幽默,双鱼,双重性格,幻想,神经质,低调
爱笑
旅游美食爱好者,执着,散步,平面设计,名人明星,魏晨控
星座命理,自然,美女,爱生活
娱乐,大眼睛,懒人,淘宝控,喜欢音乐
旅游
文艺,时尚
新闻资讯,旅游,军事,文艺,游戏动漫,动漫,梦想,海贼,死神,搞笑
IT 数码
文艺,时尚,娱乐,健康,美食
搞笑幽默
热情,赚钱
动漫,视频音乐,星座运势,宠物图集,搞笑幽默,游戏动漫,星座命理
汽车
美图摄影,美女低调,文艺小青年,单反控,阅读控
by2 吧,小时代,by2 心语家族,BY2,By2 国际后援会
健康,娱乐,游戏动漫,新闻资讯,美女,体育
新闻资讯,健康,娱乐,体育,军事
教育就业,星座命理,文艺,体育,娱乐
IT 数码,文艺,体育,健康,新闻资讯,娱乐
时尚,娱乐,旅游摄影,美食爱好者,睡觉睡到自然醒,音乐发烧友,朋友,时尚潮流,偶像剧
旅游
娱乐
美女,体育
体育资讯
美女
骑行
文艺,娱乐,美食,时尚
新闻资讯,体育
新闻资讯,星座命理,军事
娱乐
体育
旅游
健康,游戏动漫,美食,娱乐,体育
星座命理,汽车,体育
穿衣美容,福州生活,搞笑幽默,旅游,娱乐,美食,健康,游戏动漫
旅游,体育
时尚,星座命理,美食,投资理财,IT 数码,娱乐
娱乐
星座命理,新闻资讯,时尚,美食,娱乐
美女,旅游,游戏动漫,美食
体育
娱乐
旅游,娱乐,星座命理,文艺,游戏动漫

军事
文艺,娱乐,新闻资讯,健康,旅游
旅游,IT 数码
体育,娱乐
搞笑幽默,旅游,西藏生活,IT 数码
健康,投资理财,教育就业,时尚,旅游
娱乐
体育,市场营销,喜欢听歌,打球,懒人,爱睡觉,足球迷,睡觉,宅,梅西
星座命理
投资理财,汽车,时尚,健康,旅游
旅游
旅游
美食,旅游,时尚,文艺
娱乐,汽车,时尚,美食
八卦杂谈,广州生活,情感生活,视频音乐,新闻趣事
新闻趣事
时尚,美食
体育
汽车,星座命理,旅游,军事
时尚
文艺
马来西亚生活
教育就业,新闻资讯
宠物图集,温州生活,美食,游戏动漫
游戏动漫,美女,时尚,汽车,娱乐,悲催,倒霉,耍家,吃货,90 后
美女,IT 数码,数码,平常心
美女
新闻资讯,旅游,美食,星座命理,娱乐
新闻资讯,旅游,投资理财,美食
旅游
IT 数码
美女
旅游
投资理财
新闻资讯
新闻趣事,穿衣美容,旅游,天津生活,义工,志愿者,公益
旅游,美食
简单,五月天,安静,懒,90 后,沉默,阅读,白羊座,篮球,湖人
娱乐,健康,美食,汽车,旅游
IT 数码,旅游,美食
新闻资讯,星座命理,文艺,时尚
教育就业,新闻资讯,军事,体育,娱乐,文艺
时尚,军事,美食,文艺,教育就业,IT 数码,体育,美女,汽车,娱乐
文艺,娱乐,旅游

游戏动漫,IT 数码,体育,旅游
汽车,美食,时尚,IT 数码,娱乐
活泼开朗
投资理财,时尚,旅游,美食,娱乐
时尚,投资理财
IT 数码,娱乐
娱乐,旅游,IT 数码
旅游
游戏动漫
娱乐,旅游
惊蛰,带我回家,换你十年天真无,用我一生,小天真,吴邪,张大爷,张起灵,闷油瓶,瓶邪
体育
人生
文艺,汽车,投资理财
街舞
健康
娱乐
美女
娱乐
健康,星座命理
美食,旅行,看电影,阅读,摄影
微博奇葩,游戏动漫
游戏动漫,家庭教师,火影忍者,妖精的尾巴,动漫迷,梦想
军事,美女
娱乐,游戏动漫,美食,星座命理
星座命理
娛樂
健康
游戏动漫
星座命理,单身,水瓶座,购物狂,无厘头,玩儿
星座命理,娱乐,IT 数码,文艺,旅游
宁夏大学,国学,汉服
美食
旅游,微博奇葩,穿衣美容,视频音乐,搞笑幽默
文艺
上海,郭敬明,漫画,学生一枚,英国,读书,青春,文艺
美女,手机墙纸,无水印,android,apple,iPhone,旅行,唯美,文艺,小清新
海南生活,搞笑幽默,星座命理,旅游,穿衣美容
健康
时尚
美食
时尚,星座命理,娱乐
星座命理,教育就业,新闻资讯,时尚,旅游,娱乐
体育

新闻资讯,汽车,娱乐,IT 数码,喜欢听歌,热爱生活,幽默,上网,奋斗,咖啡
美食,娱乐,旅游,新闻资讯
文艺,美食,时尚,美女,娱乐
美食,时尚
娱乐,文艺,时尚
时尚,星座命理,健康
旅游,时尚,健康,星座命理,美食,娱乐
新闻资讯,文艺,娱乐,汽车,IT 数码
穿衣美容,健康,时尚,文艺
美食,娱乐,健康,星座命理,时尚,军事
时尚,娱乐,星座命理
投资理财,健康
美食,娱乐
搞笑幽默
文艺,体育
娱乐,旅游,健康,美食,IT 数码
微博奇葩,娱乐
时尚,美女,旅游,美食,打篮球,美国,心情,烹饪,体育
旅游
美女,睡觉,上网,学生,宅
旅游,葫芦丝
汽车,体育,娱乐
投资理财,娱乐,美食,旅游,IT 数码,军事,文艺,体育
红酒,女人,爱生活,天平座,风趣幽默
旅游,学生一枚,喜欢听歌
娱乐,文艺
旅游,健康
新闻资讯,旅游,星座命理,文艺
唱 K,水瓶座,单车,西安,威尔谢尔,孙燕姿,亨利,上海宁,足球,阿森纳死忠
教育就业,投资理财,健康,美女,文艺
游戏动漫,新闻资讯,美食,娱乐,旅游,巨蟹女,英语,traveling,大学生
美食,投资理财,宅,赚钱,吃货一枚,瑜伽,王力宏,偶稀饭睡觉觉
摇滚不死,真人秀重症患者,美剧重症患者,Post90
保定生活
时尚,旅游,美食,文艺,娱乐
时尚,健康,美食
美食
新闻资讯,健康,军事,体育
体育
时尚,汽车,美女,体育,娱乐
动漫,唱歌,摄影,看书,娱乐,小说,篮球
文艺
旅游,军事,娱乐,汽车,体育
投资理财,文艺,时尚,IT 数码,游戏动漫

体育,美女
美女,时尚,娱乐,健康,文艺,摄影
视频音乐,动漫,校园生活,美女
健康,时尚,汽车,投资理财,IT 数码
星座命理,文艺,健康
美食,健康
科幻
健康,投资理财,旅游,美食
时尚
美女
旅游,IT 数码,投资理财,时尚,文艺
投资理财,旅游
旅游,星座命理,娱乐,美女
娱乐
搞笑,吃货,好性格,摩羯座,宅女一枚,手机看小说,双重性格,美剧控,开心,80 后的尾巴
星座命理
星座命理,新闻资讯,奋斗 ing,爱 Minho,爱 silence,爱小四
游戏动漫,军事,IT 数码,乐逗一族,反恐精英 Online
旅游,健康,娱乐,文艺
体育
懒人
游戏动漫
娱乐,美女,汽车,投资理财,体育
旅游
文艺
体育
美食,旅游,健康,时尚,娱乐
獅子座,巧克力,旅游,CNBLUE 鄭容和,韩国文化,韩语学习,健康
新闻资讯,娱乐,美食,文艺,欧美,名著,摩羯女
时尚,体育,健康,投资理财
90 後,玩儿
文艺
艺术家黄先启,北京深圳,黄先启视频,画家黄先启,书法家黄先启
新闻资讯,游戏动漫
IT 数码,美图摄影,名人明星,校园生活,搞笑幽默
新闻趣事,星座运势,韶关生活,爱旅行,睡觉睡到自然醒,手机
星座命理,情绪化,狮子座
搞笑幽默,体育,时尚,理财,谢娜,何炅
搞笑幽默
B 型血,宅男,敏感,90
旅游
新闻资讯
汽车
时尚,热爱生活

体育
旅游,星座命理,健康
旅游美食爱好者,微笑,孙燕姿,小清新,健康,时尚,旅游,美食,文艺,娱乐
时尚,美女
时尚,美食,IT 数码
投资理财,旅游,美女,教育就业,星座命理,文艺,体育,娱乐
超 2 小眺眺,星座命理,健康,游戏动漫,娱乐,文艺
星座命理
我喜欢钓鱼
文艺,娱乐,时尚,星座命理,美食
文艺,时尚,星座命理,美食,健康,旅游
爱笑,我就是我,台球,科幻,军事
NBA 球迷,旅游爱好者,文学创作,文学爱好者,文学小青年
游戏动漫
旅游,娱乐
美食
游戏动漫
新闻资讯,汽车,IT 数码
旅游
时尚,狮子座 90 后女生
教育就业,军事,投资理财,体育,汽车
文藝
搞笑幽默,新闻资讯,旅游,美食,体育,汽车,爬山,k 歌,幽默,篮球
时尚
新闻资讯,汽车,IT 数码
旅游
时尚,游戏动漫,美女,星座命理,旅游
制作中国毛笔,传承千年文化
时尚
娱乐
新闻资讯,美食
长沙生活
美食,投资理财,旅游,文艺
IT 数码
新闻资讯,旅游,IT 数码
新闻资讯,汽车,IT 数码
健康,旅游,星座命理,时尚,娱乐
星座命理,旅游
旅游,体育,美食
游戏动漫,娱乐
新闻资讯,旅游,IT 数码
林更新,朴有天,夜猫子,双重性格,宅女一枚,天平座,死神,爱睡觉,90 后但我不脑残,蒋劲夫
健康
IT 数码,健康,星座命理,美食,游戏动漫

体育,文艺
健康,星座命理,投资理财,美食,游戏动漫
健康
娱乐
美女,广州生活,新闻资讯
娱乐,IT 数码,汽车
体育,新闻资讯
搞笑幽默,美食,视频音乐
游戏动漫,娱乐,体育,汽车,旅游,男人,学习,奋斗,A 型血,阳光开朗
美女,新闻资讯,汽车,体育,打篮球,阳光,房地产
健康,游戏动漫,教育就业,新闻资讯,文艺
体育,星座命理,军事,IT 数码
娱乐
潮流,时尚达人,表演,校园,团购,笑话,宅女一枚,时尚
旅游
美食
教育就业,健康,时尚,星座命理,美食,娱乐
穿衣美容,旅游
旅游
娱乐
文艺,美女,星座命理,旅游,投资理财
旅游
新闻趣事,新闻资讯
星座命理
旅游,星座命理,美食,文艺,娱乐
投资理财,时尚,健康,旅游,美食,文艺,娱乐
投资理财
美女,健康
管理
游戏动漫
IT 数码,文艺
旅游,文艺,游学,冬夏令营,带薪实习,健康,旅行,手机控,微博控,90 后
旅游,美食,IT 数码,娱乐
美食,微微一笑很倾城
体育
文字控,旅游,90 后,听歌,冷笑话,天蝎女,户外
游戏动漫,美食,文艺
教育就业,健康,军事,教师,电脑游戏爱好者
娱乐,文艺,健康,旅游,星座命理
IT 数码,文艺,时尚,教育就业
美女,时尚
美女
旅游
娱乐,游戏动漫

体育
开朗,愤青,懒人,90後,吃货,臭美,奋斗,爱213,相亲相爱,北京爷们儿
旅游
工业设计,家居设计,广告创意,建筑设计,室内设计专家,创意设计,旅游,美食,文艺,时尚
娱乐
游戏动漫
体育
娱乐,旅游,美食,汽车,体育
时尚,旅游,娱乐,文艺,投资理财
文艺
美女,娱乐,美食,游戏动漫
音樂
美食
IT数码,军事,旅游,游戏动漫
明基模力女孩,美女,文艺
美女
新闻资讯,健康,教育就业,旅游,文艺
新闻资讯,美女,游戏动漫
约会,美空,学生,模特,商务伴游,娱乐
旅游,军事,IT数码
美女,投资理财,IT数码,体育,娱乐
健康
旅游,文艺,教育就业
娱乐
娱乐
投资理财
时尚达人,购物狂,服装,淘宝,购物,美容护肤,性感,舞蹈,化妆品,宅女
文艺
健康,文艺,汽车,投资理财,体育
智慧女人,酒吧,高跟鞋控,跳舞,音乐,电影,时尚,汽车,旅游
美食
萌宠
美食
游戏动漫,教育就业,旅游,时尚,娱乐,美食,文艺
美女
时尚
美食
旅游
旅游
美食,游戏动漫
教育就业,旅游
美食,旅游,美女,体育,娱乐
蓝色控,游戏迷,宅女,爱杰伦
健康

健康,旅游,美食,娱乐
娱乐
笑,EXO,好性格,双重性格,90 后但我不脑残,画画,爱猫,写作,音乐发烧友,低调
新闻资讯,时尚,投资理财,文艺,娱乐
新闻资讯,教育就业,投资理财
文艺,素描,水粉画,井岸,美术培训,摄影师,旅游
健康
体育
美食,军事,汽车,娱乐,旅游
瑜伽,科幻,英格兰,欧美音乐,小说迷,好书,美容护肤,咖啡控,旅游美食,翻译
重庆生活
美女
文艺
旅游
名人明星
母乳喂养,职场妈妈
军事
体育
游戏动漫
体育
文艺,时尚,星座命理,美食,娱乐
美食,旅游,不想上班,购物狂,奋斗,新闻资讯,健康,教育就业,汽车,文艺
名人明星,缺爱 ING,90 后,爱艺兴,EXO,爱音乐
时尚
游戏动漫
旅游,时尚,文艺,美食,新闻资讯
shopping,朋友,娱乐
旅游,文艺,军事,美食,美女,沉默,奋斗
时尚,娱乐,体育,汽车,美女
文艺
旅游
时尚,文艺,美食,星座命理,娱乐
IT 数码
湖南生活,小女人,双子女,淡定
美女,搞笑幽默
时尚
美食
微博奇葩,旅游,视频音乐,搞笑幽默,穿衣美容
健康,美女,文艺,教育就业,IT 数码,体育
旅游,游戏动漫,体育
宝宝
旅游
贵州生活
军事

游戏动漫,幻想,双子,小说迷
时尚,娱乐
微博,偶像剧,摩羯座,青春
健康,汽车
摄影
健康,美食
美容服饰
写作,好书,80后宅女,语录,腐女,聊天
新闻资讯,健康,娱乐,旅游,IT数码
娱乐
金牛座,学生一族,孩子气,游戏动漫,体育,娱乐,旅游
投资理财
游戏动漫
文艺,旅游,健康,美食,娱乐,IT数码
美女
健康,美食
健康,时尚,旅游
娱乐,文艺,IT数码
美女,娱乐
汽车,健康,旅游,美食,军事
看电影,自由,music,法国,怀旧,时装
健康
文艺
DIY,创业,古典音乐,狮子座,摄影爱好者,奋斗,o型血,淘宝控,旅游,汽车
星座命理
执着,矛盾体,双子座
美食,星座命理,多愁善感,英语,教育就业,沉默,music
IT数码
双子座,敏感,美食爱好者
可爱,减肥,偶像剧,听音乐,淘宝,媒体,时尚,文艺,旅游,娱乐
IT数码,娱乐
健康,旅游,投资理财
时尚,文艺,健康,旅游,美食,教育就业
美食
时尚,文艺,娱乐
时尚,旅游,健康
计算机,旅游摄影
视频音乐,湖北生活,旅游,文艺,体育,IT数码
游戏动漫,动漫,读书
汽车,美食
未来作家,音乐,心情
时尚,军事,IT数码
美食,校园生活,搞笑幽默,重庆生活,旅游
时尚

旅游
健康,星座命理,军事,IT 数码
冷笑话迷,伪文艺,美食,吃喝玩乐,爱自由
美食
搞笑,哲理,感性,语录,娱乐,宅男,生活,快乐
微博奇葩,美女,视频音乐,搞笑幽默
文艺,星座命理,美食,旅游,射手座,吃喝玩乐,星座控,爱自由,公益,爱睡觉
美食
新疆生活,萌宠,金牛女
玩儿,小清新,可爱,动漫,文艺
旅游
旅游,娱乐
文艺,投资理财,教育就业,新闻资讯,时尚,星座命理,美食,体育
健康
游戏动漫,军事
中华文化习俗,中华传统文化,快乐,摄影,娱乐,媒体,文化,美食,新闻
IT 数码,美女,体育
教育就业
汽车,娱乐,美食,旅游
娱乐
新闻资讯,军事,IT 数码,游戏动漫
娱乐
美食
健康,美食,教育就业,时尚,IT 数码,文艺,娱乐
交朋友,安静,双鱼座,爱美食
星座命理,时尚
游戏动漫,星座命理
美食,游戏动漫
新闻资讯
旅游,新闻趣事
热爱生活
时尚,美食,文艺
游戏动漫,青年,上进,追求,汽车,文艺,体育
穿衣美容,娱乐
篆刻,典雅
心情
美食,星座命理,美女,独立,IT 数码,健康,体育
健康,时尚,娱乐,体育
旅游,爱旅行,阳光,爱生活
教育就业,旅游,文艺
体育
旅游
美女,时尚,汽车,IT 数码,娱乐
娱乐

娱乐
电视,搞怪,健康,文艺,旅游,美食
健康,新闻资讯,美女,军事,旅游,大胖子
体育资讯,体育
时尚,娱乐
投资理财
教育就业,健康,星座命理,投资理财,旅游,文艺
美容服饰,旅游,星座运势,八卦杂谈,搞笑幽默
娱乐
性感,时装,团购,时尚潮流,吃货,健康,拍照,O 型血,网购
2B 青年,屌丝,强迫症,矛盾体,生活大爆炸,exo,学生党,时尚,IT 数码,星座命理
时尚,80 后,宠物达人,宠物用品,美国代购
游戏动漫
教育就业,旅游,美食,星座命理,新闻资讯,健康
上海生活,星座命理,美食,萌宠,美女
健康,爱生活,时尚
读书
娱乐
恋旧，专一
90 后,暗恋
汽车
旅游
娱乐
体育,陈奕迅,动漫迷,水瓶座,单身,五月天,温柔,足球,公益,爱唱歌
星座命理
IT 数码,娱乐,新闻资讯,合同纠纷,保险索赔,婚姻家庭,交通事故,怀化律师
娱乐,旅游
美女,美食,娱乐,文艺,旅游
健康
IT 数码
美女
教育就业,新闻资讯,娱乐
视频音乐,花样美男,校园生活,名人明星
穿衣美容
娱乐,游戏动漫,美食
美图摄影,IT 数码,科学探索,重口味,旅游
IT 数码
文艺
投资理财,IT 数码,星座命理
微博奇葩
美女,旅游,IT 数码,娱乐,体育
唱 k,双鱼女,shopping,时尚,星座命理,美食,文艺,娱乐
健康,旅游,娱乐,逛街只逛不买型,学生一族,胡思乱想,爱,沉默,吃货,天蝎座
旅游

文艺
娱乐
电影控,耽美,时尚,天蝎座,文学,梦想,书,music
游戏动漫,教育就业,IT 数码,军事,新闻资讯
微博奇葩,文艺
娱乐
娱乐
视频音乐,娱乐
穿衣美容,山西生活,微博奇葩,旅游,文艺,星座命理,娱乐
美食
美食,游戏动漫,健康,美女
新闻资讯,旅游,IT 数码
美容服饰
星座命理,宅,笑,吃货一枚
体育
财经资讯
心理
IT 数码
手工,小吃货,傻到可爱的二货
文艺
游戏动漫
美女
投资理财,体育,白银,黄金,投资,金融
穿衣美容,新闻趣事,旅游,美食
娱乐,星座命理
美女,健康
健康,旅游,美食,文艺
k 歌,李宇春
名人明星
其他生活
教育就业,新闻资讯,娱乐,旅游
新闻资讯,旅游,美食,军事,IT 数码
星座命理,时尚,想不断的唱歌。
旅游,读书分享,西安生活
文艺,美食,旅游
游戏动漫,时尚
旅游,星座命理
星座命理,娱乐
海外生活
体育资讯
旅游
乒乓球,聊天,交友,军事,IT 数码,帅哥,男人,寂寞,淡定
福州生活,时尚
旅游,文艺,时尚

文艺
投资理财
翻译,金牛座,睡懒觉,魔羯座,努力,小资
文化,历史,交响乐,敲钟,祈福,跨年
军事,汽车,星座命理,旅游,文艺
游戏动漫
娱乐,文艺
时尚
时尚
旅游,美女
星座命理
新闻资讯,IT 数码
旅游,美食,娱乐,时尚,文艺
旅游,新闻趣事,美女,名人明星,穿衣美容,投资理财,IT 数码
体育
微博奇葩,娱乐,心情随笔
文艺
健康,旅游,军事,汽车,投资理财
文艺,旅游,爱玩爱笑爱闹
娱乐
投资理财,旅游,教育就业,汽车,娱乐
美女
时尚,健康,新闻资讯,旅游,投资理财
吃货一枚,乐天派,服装设计,爱自由,爱睡觉
旅游,穿衣美容,美食
交朋友,文艺,健康,旅游,体育
汽车,投资理财,军事,IT 数码,文艺,体育,娱乐
IT 数码
新闻趣事,搞笑幽默
宠物图集,视频音乐,新闻趣事
美食,星座命理,时尚
军事
娱乐,文艺
投资理财,IT 数码,时尚,健康,星座命理,美食
IT 数码,美女,时尚,娱乐
时尚,美食
美食
文艺
旅游
新闻资讯,教育就业
IT 数码,文艺
时尚,娱乐,健康,星座命理,美食
新闻资讯,健康,旅游,美食,娱乐
时尚,文艺

时尚,军事,新闻资讯,旅游,美食
旅游
穿衣美容
星座命理,旅游,健康,时尚,投资理财
新闻资讯,健康,美食,星座命理,腐女,神经质,90 後,涂鸦
读书分享,八卦杂谈,美图摄影,新闻趣事,打球,旅游美食,心理学,巨蟹,文艺
时尚
园林景观设计,城市建设,住宅景观设计,别墅景观设计,公园设计,园林设计,城市规划,城市设计,建筑设计,景观设计
健康,旅游,汽车,投资理财,IT 数码
娱乐
时装
健康,美食,旅游,教育就业
教育就业,文艺,新闻资讯
时尚,星座命理,娱乐
双鱼座,爱生活,80 后,爱马苏
美食
娱乐
投资理财,军事,健康,汽车,汽车电喇叭
娱乐
汽车,娱乐
广西生活
军事,体育,吸血鬼日记,ELF,快乐大本营
时尚,汽车
汽车,美女,美食,游戏动漫,体育,娱乐
体育
旅游,美食
文艺,法国代购,美食
体育
旅游,娱乐,投资理财,时尚,文艺
娱乐,军事
游戏动漫,娱乐
娱乐,投资理财,美食,旅游,健康,时尚
健康
旅游,星座命理,IT 数码,文艺,娱乐
时尚,新闻资讯,投资理财,汽车,健康,旅游,美食
汽车
美食,军事
文艺
IT 数码,健康,美食,文艺,体育
正太,许嵩迷,英雄联盟,桌球,校园,狮子座,上海,90 后非脑残,音乐迷,学生一族
教育就业
时尚
美女,星座命理

汽车
旅游
搞笑幽默,游戏动漫,体育
汽车,体育
文艺
时尚,教育就业,旅游,娱乐,文艺
进击的巨人,黑执事,家庭教师Reborn,火影忍者,摄影爱好者,cosplay,动漫控
健康,心情,文艺,IT数码,美食,旅游
IT数码,游戏动漫
美女,娱乐
看小说,爱幻想,音樂,学生一枚,金牛座
娱乐
投资理财,游戏动漫,时尚
文艺
娱乐,星座命理,游戏动漫,新闻资讯,美食,游戏,吃货,猫控
美女,健康,新闻资讯,游戏动漫,旅游
新闻趣事,搞笑幽默,旅游,美女,穿衣美容,时尚
时尚,美食,星座命理,投资理财
美食,教育就业,时尚,文艺,娱乐,健康
娱乐
IT科技,IT数码
美女
星座命理,军事
美食
健康,PINKFLOYD,PINK,娱乐
投资理财
游戏动漫,体育,娱乐
时尚
星座命理,游戏动漫,文艺,体育,娱乐,冬天来了
天蝎女,矛盾体,旅游,健康
娱乐,文艺,健康,星座命理,汽车
教育就业
穿衣美容
时尚,游戏动漫
美食,时尚,新闻资讯,文艺,体育
美女,美食
微博奇葩,美食,体育,游戏动漫
星座命理,美食,旅游
体育
旅游,体育,运动,喜欢听歌,热爱生活
娱乐
星座命理,文艺,娱乐,健康,美食
游戏动漫,美女
娱乐

视频音乐,八卦杂谈,娱樂
游戏动漫
旅游
美女,体育
美食
教育就业,自行车
旅游,健康,时尚,美食,娱乐
体育
星座命理
白羊座,淡定,心理学,旅游,学生一族,娱乐,教育就业
汽车,时尚,IT 数码,星座命理,美女
美食,吃货
外贸
美食
美食,投资理财
汽车,投资理财,教育就业,新闻资讯,IT 数码
江苏生活
娱乐
健康
时尚,娱乐,美食,星座命理,健康
美女,四川生活,穿衣美容,时尚,星座命理,美食,文艺
新闻资讯,美食,文艺
星座命理,娱乐
美食
囧,吃喝玩乐,听歌,90 后,大学生,偶稀饭睡觉觉,双子座,善良,好性格,睡懒觉
星座命理,体育,美女
健康,娱乐,时尚,星座命理,旅游,美食
听歌,旅行,90 后,唱 k,服装,星座控,美食,喜欢音乐,艺术,猫控
星座命理
星座命理,娱乐
文艺,美食
投资理财,时尚,小说,造价,美食,旅游
体育
旅游,美食,汽车
时尚,旅游,美食,文艺,娱乐
美食,旅游
星座命理,美食
穿衣美容
娱乐,游戏动漫,体育
时尚,恐怖片
游戏动漫,娱乐,文艺
新闻资讯,娱乐,音樂
淘宝控
美女

湖南生活,健康
美食
体育,旅游,美食
教育,情感生活,视频音乐,时尚,汽车,美女,旅游,美食,体育,娱乐
TaylorSwift,英美剧,美国,猫咪,水瓶女,听音乐,英国,欧美,小清新,美食
投资理财,新闻资讯,军事,汽车,健康,旅游,美食,体育
体育
IT 数码,汽车,美女
美女,文艺,游戏动漫,体育
教育就业
娱乐
电影爱好者,交朋友,王力宏,唱 k,it,汽车,投资理财,IT 数码,体育,娱乐
娱乐,文艺
体育,健康
美女,娱乐,旅游
美女,游戏动漫
美食,旅游,游戏动漫,新闻资讯,星座命理
星座命理
IT 数码,体育
强迫症,娱乐
足球迷
汽车,教育就业,投资理财
娱乐
投资理财
旅游
美女
旅游
汽车
娱乐
IT 数码,狗狗
投资理财,旅游,汽车,IT 数码,军事
星座命理
美食
健康
美食
投资理财,体育,交友,看书,篮球,创业
Love 本营迷,星座命理,健康,旅游
美食,搞笑幽默,湖南生活
时尚
星座命理,时尚,美食,旅游,文艺
旅游
旅游,星座命理,投资理财,汽车,美女
做梦
娱乐,星座命理,狮子女

教育就业,新闻资讯,健康,IT 数码,投资理财
投资理财
时尚,体育,娱乐,游戏动漫,汽车
娱乐,时尚,旅游,美食
文艺,健康,美食
旅游,文艺
穿衣美容
旅游,IT 数码,时尚,文艺,体育
阿根廷
搞笑幽默
娱乐,玩,懒,爱笑,互粉
美食,游戏,音乐,狮子座
华美,泉州,安溪,福建,闽南人
娱乐,体育
健康
娱乐,汽车,美食
ac 米兰
文艺,IT 数码
文艺,美食,娱乐,健康,时尚
时尚
娱乐,旅游,神经质
健康,时尚
美食,旅游,美女,游戏动漫
体育,娱乐
游戏动漫,动漫控,吃货腐败,科幻,吸血鬼,安静,神经质,美剧迷,射手,腐女党
大庆生活,娱乐
美食
文艺
旅游
娱乐
娱乐,健康,教育就业,投资理财,旅游
音乐,武术
推理之神,技术宅,90 后,水瓶座
健康,教育就业,投资理财,体育,美女
旅游,IT 数码
体育,秀秀,自拍,前卫,旅游,时尚,娱乐,美食,文艺
时尚,文艺,投资理财,教育就业
美女,体育
江西崛起,江西路新社,走进赣鄱,看江西,发现江西,今日微江西,江西新闻
美食,娱乐
摩羯女,音乐控,乐观,宅女,爱音乐,健康
健康
体育
微博奇葩,游戏动漫

体育,新闻资讯,娱乐,旅游,汽车,文艺
名人明星,娱乐,星座命理
创意,动漫控
美女,文艺,体育,美食,星座命理,游戏动漫
新闻资讯,星座命理,旅游,军事
IT 数码,游戏动漫,教育就业,我是广告人
星座命理,时尚,娱乐
娱乐,美食
美食,美女,云南生活,新闻趣事,搞笑幽默,汽车,教育就业,投资理财,娱乐,IT 数码
游戏动漫
汽车
美女,时尚
文艺,软通学院,江西财经大学
美食
体育
IT 数码,娱乐,新闻资讯,健康,时尚
美食,旅游
微博奇葩
双子座女生,腹黑,维尼夫妇,动画,吃货,韩国,看电影
美食,爱打扮,热爱生活,心情,fashion,听音乐
搞笑幽默,新闻资讯,教育就业,投资理财,汽车,IT 数码
时尚,美食,健康,旅游,美食,娱乐
新闻趣事
时尚,健康,旅游,美食,娱乐
视频音乐
微博奇葩,旅游,意大利生活,娱乐,减肥会失败星人,爱美剧,吃货
娱乐,强迫症候群,电影控,皇马,足球迷,双重性格,起床困难户,天秤座,上班族
健康
宠物图集
徐贤,容和,亚纶,红薯夫妇
新闻资讯,美女,美食,IT 数码,娱乐
健康养生,旅游,品酒,健康,娱乐,美食
娱乐,星座命理
爱自由
健康
美食
美食,旅游,新闻资讯,时尚,娱乐
娱乐
IT 数码,星座命理,健康,美食,文艺
娱乐
娱乐,新闻资讯,游戏动漫,军事,IT 数码,汽车
美女,教育就业,投资理财,美食,健康
星座命理
军事,美食,投资理财,美女,时尚

传递正能量
服装设计师,购物,爱搭配,爱美丽,海报时尚,服装品牌,服装搭配,服装行业,时尚
人到哪乱到哪,偏头疼,晚睡强迫症,万年隐身帝,怕鬼,电影狂人,扫雷达人,宅系生物,文艺
喜欢音乐,爱睡觉,巨蟹,简单,感性,听音乐,忧郁,我就是我,睡觉,宅
美食,汽车,时尚,IT 数码,娱乐
星座运势,名人明星,镇江生活,教育就业,健康,旅游,美食,娱乐
体育,美食,星座命理,娱乐
娱乐,时尚
教育就业
娱乐,游戏动漫,汽车,军事,体育
视频音乐,二哥路人粉,教授路人粉,蝴蝶 cp 粉,茨威格,阿森纳,费纳,费德勒
健康,时尚,美食,维密控,小清新,爱原单,水瓶座加 O 型血,吃货,爱淘,爱得瑟
创意,乐橙,幸福,旅游爱好者,三国杀,喜欢音乐,传媒,媒体,广告,旅游
搞笑幽默,娱乐
教育就业,IT 数码,娱乐,汽车
90 后但我不脑残,大学生,艺术,水瓶座,动漫控,文艺
汽车,教育就业,投资理财,娱乐,体育
旅游,投资理财,美食,时尚,娱乐
娱乐,IT 数码,美女,文艺,军事
美食,健康,旅游,体育,爱美食,咖啡
旅游,娱乐
搞笑幽默,傻,睡觉睡到自然醒,胡思乱想
IT 数码,汽车,教育就业,美女
娱乐
旅游
美食
文艺,美女,游戏动漫,娱乐,美食,旅游
健康,星座命理,新闻资讯
新闻资讯,时尚,体育
健康,时尚,美食,教育就业,投资理财
搞笑幽默,体育,学生一族,90 后,神经质,爱旅游
IT 数码
娱乐
名人明星,时尚,摄影
搞笑幽默,教育就业
娱乐,美食
潜力股、奋斗 ing
IT 数码
旅游
健康,美食,旅游
美女
游戏动漫,星座命理,美食,体育,娱乐
美女
萌宠

教育就业,文艺,IT 数码
旅游
文艺,健康,投资理财,时尚,IT 数码,星座命理,军事,米虫 and 吃货
教育就业,健康,旅游,美食,军事,文艺
娱乐,体育,旅游,天蝎,学习,爱自由
时尚,文艺,旅游,娱乐,星座命理
名人明星,上海生活,娱乐
时尚
美食,旅游,时尚,娱乐,体育
美女,美食,星座命理,文艺
媒体人,文藝,体育,教育就业,文艺
汽车,游戏动漫
旅游,美食,文艺
健康,文艺
美食,旅游
时尚,教育就业,IT 数码,健康
手机看小说,桌球,感性,love,90 后非脑残
IT 数码,美食,时尚,旅游,文艺,娱乐
军事,旅游
美食,投资理财,教育就业
军事
时尚,星座命理,投资理财,娱乐,美食,臭美
投资理财,娱乐,健康,旅游,美食
餐饮,吃货,美食,投资理财,汽车,IT 数码
星座命理
旅游,健康,新闻资讯,美女,汽车
娱乐
文艺
星座命理
搞笑幽默,新闻趣事,视频音乐,旅游,汽车,教育就业,投资理财,娱乐,星座命理
新闻资讯,美食,旅游,文艺,体育
视频音乐,河南生活,旅游
教育就业,健康
美食,游戏动漫
时尚,美食,旅游
汽车,美食
时尚
娱乐,游戏动漫,健康
美食,旅游,新闻资讯,体育,娱乐,文艺
美食
IT 数码,旅游,游戏动漫,娱乐,军事
汽车,军事,美食,旅游
汽车,星座命理,时尚,娱乐
郭敬明

90 后非脑残,射手座,WOW,文艺,游戏动漫,美食,军事
旅游,IT 数码
娱乐
娱乐,时尚
IT 数码
视频音乐
穿衣美容,新闻趣事,旅游,微博奇葩,时尚,文艺,教育就业
时尚
美食
文艺,星座控,猫控,爱旅游,爱笑,简单,随性,独立,历史,文字
爱音乐,腐女,听音乐,淘宝,购物,美剧控,吃货一枚,宝宝,网购,唱 k
投资理财,旅游,新闻资讯,美食,军事
小说迷,写字
文艺,娱乐
美食
90 後,双重性格
情感生活,读书分享,星座运势,美食,校园生活,摩羯座,读书,执着
视频音乐,吸血鬼日记,偶稀饭睡觉觉,cosplay,火影忍者
旅游
新闻趣事,投资理财,新闻资讯,汽车,教育就业
旅游,星座命理,教育就业,时尚,美食,健康,IT 数码,体育
健康
投资理财,美食,旅游,星座命理,教育就业,文艺,娱乐,健康,时尚,军事
文艺
游戏动漫,美食,IT 数码
旅游,军事,娱乐
android,安卓,apple,苹果,apps,应用推荐,outlets,奥特莱斯,megamills,米格天地
健康
体育
美女,星座命理,汽车,旅游,美食,健康,文艺,IT 数码,体育,娱乐
健康,时尚,旅游,体育,美女,汽车,IT 数码,投资理财,星座命理,娱乐
美食,旅游,游戏动漫,娱乐,文艺
游戏动漫,军事,教育就业,文艺,体育,娱乐
游戏动漫,美女,汽车,旅游,健康,文艺,体育
微博奇葩,鸡涌,巨蟹座,O 型血,BIGBANG,90 后
汽车
新闻资讯,时尚,汽车,星座命理,娱乐
KARICARE,貝拉米奶粉,奶粉,保健品,UGG,時尚,澳洲代購
新闻资讯,健康
汽车
汽车,娱乐,旅游
体育
时尚,健康,旅游,美食,娱乐
新闻资讯,美女,文艺,星座命理

汽车,时尚,投资理财,旅游,IT 数码
时尚,汽车,文艺,体育,娱乐
军事,IT 数码,健康,娱乐,文艺
吃货一枚,拖延症,肉多多植物
汽车,体育,新闻资讯,旅游,IT 数码
投资理财,旅游,汽车,军事,IT 数码
微博奇葩
健康,美女,投资理财,星座命理,体育
美女,新闻资讯
股票,AvrilLavigne,孙燕姿,旅游摄影,购物,唱 k,睡觉,动漫,水瓶座,爱美食
教育就业,健康,旅游,美食,文艺
汽车,旅游,军事,IT 数码,娱乐
旅游,文艺,美食,娱乐
汽车,新闻资讯,游戏动漫
星座命理,投资理财,文艺,新闻资讯,健康
教育就业,娱乐,汽车,投资理财,健康,美女,时尚,美食,文艺
新闻资讯,投资理财,文艺,旅游,美食,健康
汽车,健康,旅游,美食,娱乐
美食爱好者,新闻资讯,体育,军事,健康,旅游,汽车
美食
名人明星
健康,旅游
IT 数码,娱乐,广州租房,人才招聘,二手发布,生活服务,搞笑趣味,社交网络,重口味,时事新闻
投资理财,时尚,汽车,IT 数码,体育
军事,旅游,时尚,汽车,IT 数码,美食
新闻资讯,健康,旅游,文艺,娱乐
商务包,公文包,商务礼品,商务合作,礼品行业
IT 数码,体育,娱乐
旅游
爱狗狗,爱笑,美食,学生,爱玩,冷笑话,微博控
名人明星,美女,教育就业
娱乐
时尚,军事,IT 数码,旅游,汽车
购物,音乐,钢琴,美食
娱乐
黑龙江生活,美食,不迁怒,不贰过
美女
娱乐,游戏动漫
体育
设计,装修,投资理财
星座命理,真诚,双子座,中醫,形意拳,厚朴一期,健康
美食,永远在一起
娱乐

文艺,汽车,时尚,游戏动漫,投资理财,教育就业
頭角峥嶸
娱乐,新闻资讯,美女,游戏动漫
健康
旅游
娛樂,文藝,旅遊
教育就业,投资理财,看小说,心理学,网球王子,言情小说
体育,投资理财,旅游
李宇春,孙燕姿,胡思乱想,摩羯,双鱼座,水瓶座,淘宝控,天秤座,星座命理
美女,教育就业,文艺
军事,娱乐,文艺,IT 数码,旅游,美女,游戏动漫,时尚,汽车
美食
视频音乐,搞笑幽默,穿衣美容,星座命理,美食
新闻趣事,搞笑幽默,美食
时尚,教育就业,旅游,文艺
体育,文艺,军事
广东生活,视频音乐,美女,体育,IT 数码
视频音乐,娛樂,遊戲動漫,nico,vocaloid
搞笑幽默,健康,时尚
游戏动漫
文艺,时尚,健康,旅游,美食,娱乐
投资理财
娱乐,健康,IT 数码,汽车,体育,双子
新闻资讯
旅游,视频音乐,微博奇葩,新闻趣事,搞笑幽默
投资理财,新闻资讯,娱乐,吸血鬼,搞笑,幽默,胡思乱想,吃货一枚
游戏动漫
搞笑幽默,穿衣美容
萌宠,文艺,宅女,睡懒觉,爱玩,喜欢音乐
穿衣美容,美食,旅游,爱旅行,巨蟹女,90,旅游美食爱好者
简单,爱自由,射手女,孩子气,微博,投资理财,旅游
星座命理
星座命理,游戏动漫,卜懂寂寞,学生
星座命理,美食
搞笑幽默,新闻资讯,体育,娱乐
体育
美女,文艺,IT 数码,军事,投资理财
搞笑幽默,游戏动漫
新闻资讯,美食,健康,旅游,星座命理,IT 数码
军事,健康,新闻资讯,时尚,娱乐
IT 数码,汽车,娱乐,健康,时尚,投资理财,美食
投资理财,时尚
美女,投资理财,娱乐
萌宠,美食,旅游

伪文艺女青年,獅子座,文字控,蘇打綠,陳綺貞,土肥圓黑二,吃货一枚减肥中,千葉涼平
旅游
星座命理,文艺
娱乐,星海音乐学院,热爱唱歌
游戏动漫,娱乐
美食
游戏动漫
美女,体育,星座命理
星座命理
古典音乐,苹果控,唱歌,旅游,美女
新闻资讯
美女,旅游,微博奇葩,搞笑幽默,萌宠
潮流美鞋,潮服饰,搭配,街拍,潮物,时尚,减肥,时尚达人,饰品,文艺
星座命理,时尚,美食,健康,旅游
美食,旅游,军事
三体,科幻,风景美食,鲁邦三世,另类宠物,植物昆虫,EVA,茶道
健康
美食,体育,汽车
旅游,娱乐,星座命理,文艺
美食
星座命理,福建生活,搞笑幽默
开心,淘宝控,睡觉睡到自然醒,学生一枚,爱吃,方大同,八卦,红楼梦,星座命理,双重性格
搞笑幽默,旅游,美食,情感生活,宠物图集,娱乐,星座命理,新闻资讯
名人明星
健康,美食
旅游
电影控,旅游,生活,网购
美食,IT 数码
美女,美食,军事,IT 数码
娱乐,美食
美食,美女,绍兴生活,搞笑幽默,娱乐,文艺,IT 数码
投资理财,文艺,体育,游戏动漫,IT 数码
教育就业,星座命理,健康
健康
村上春树迷,萝莉,萌神,二次元症候群
旅游,聊天,音乐发烧友,爱音乐,平常心,艺术
旅游,投资理财,健康,IT 数码,文艺
娱乐,体育
文艺,时尚,新闻资讯,健康,投资理财
健康,时尚,旅游,星座命理,美食
美食
奋斗,双重性格,偏执狂,高中生
体育,星座命理,军事,美食
旅游

美食,美女,喜欢唱歌,科比,街舞
文艺,体育
时尚
美食
投资理财,时尚,健康,美食,文艺
时尚
体育
文艺,IT 数码,星座命理,娱乐,心理,动漫,学生,宅,喜欢音乐,互联网
健康,汽车,游戏动漫,军事,体育
娱乐,创业,火影,养生,微笑,林峰,赚钱,音乐爱好者,电影控,动漫控
体育,旅游
体育
考研,篮球,品牌,语录,时尚,开朗乐观孩子气
美食,摩羯座,o 型血,随性,85 后,爱睡觉,矛盾体
美食,娱乐
健康,星座命理,旅游,美食,文艺
时尚
喜欢音乐
财经,养生
时尚,新闻资讯,文艺,IT 数码,美女
天蝎座
健康,教育就业,旅游
美食,新闻资讯,汽车,旅游,军事
执着,简单,笑,坚强,小清新,努力,开朗,时尚,旅游,娱乐
IT 数码,美图摄影,美女,名人明星,新闻趣事
投资理财,健康,旅游,美食
美食
美食,娱乐,游戏动漫,旅游,星座命理
低调 90 後,我行我素
爱情
健康,投资理财
新闻资讯
从此幸福
旅游,星座命理,美食,IT 数码,文艺
星座命理
旅游,体育
星座命理
文艺
体育,娱乐,汽车
90 后但我不脑残,爱旅游,爱电影,新闻资讯,美食,娱乐,k 歌
美食,时尚,健康,教育就业,星座命理
文艺
游戏动漫,美食
微博奇葩,美食,旅游

我们是 8090 后,游戏中感悟人生,新兴团队,建站团队
IT 数码
时尚,旅游,健康,新闻资讯,美食,娱乐
娱乐,体育
娱乐
爱吃,乐观,爱笑,艺术,吃,娱乐,时尚,星座命理,健康,美食
时尚,旅游
时尚,文化,欧美,喜欢音乐,舞蹈,shopping,美食,天秤座,青春,星座控
星座命理,健康,美食,娱乐,时尚
旅游,体育,文艺,美女,新闻资讯
汽车,教育就业,新闻资讯,健康,IT 数码
体育
美食,旅游
星座命理,搞笑幽默,旅游,军事,美食,教育就业,投资理财,体育
缇庡コ,浣撹偛
星座命理
旅游
烹饪
娱乐
投资理财,军事,健康,新闻资讯,IT 数码
美女,游戏动漫,汽车,星座命理,军事
美食
娱乐,新闻资讯,旅游,投资理财
微博奇葩,美食,穿衣美容,星座命理
旅游,星座命理
时装
时尚,旅游,美食,娱乐,水瓶座,正太控,学习,爱美食,爱玩
时尚
旅游
健康
名人明星,新闻趣事,搞笑幽默,娱乐
体育
室内设计
旅游,体育
健康,旅游,美食
投资理财,汽车,军事,IT 数码
搞笑幽默
体育
健康,时尚
美食,汽车,军事,体育
搞笑幽默,女人,时尚
星座命理,美食
脆弱,敏感,童话里的孩子,美食,爱自由,双鱼女
娱乐

星座运势,体育
心情,桌游,睡觉,123,旅行,美食,奋斗,玩
美食,娱乐,文艺,美食,星座命理,健康
全球購,奢侈品,个性独特,視覺,vintage,时尚,文艺
星座命理,健康,旅游,美食,水瓶座,睡觉,时尚,哲学,动漫,影视
娱乐,体育,美食,麦迪,运动,听音乐
新闻资讯,美女,游戏动漫,军事,娱乐
健康
娱乐
科学探索,新闻资讯,健康,文艺,娱乐,体育
投资理财
新闻资讯
IT 数码,时尚,汽车,投资理财,旅游
娱乐
汽车,投资理财
美女,IT 数码,旅游,汽车,游戏动漫,文艺,体育
教育就业,DOTA 女子协会,DOTA,桌球,拍照,旅游美食爱好者,打球,成都
吃货……
时事,小女人,新闻资讯,时尚,健康,星座命理,娱乐
体育,新闻资讯,军事
时尚,体育,旅游
旅遊,旅游
美女,新闻资讯,旅游,星座命理,美食,娱乐
娱乐,军事,旅游,午夜话题,情感空间,随性
娱乐
美女,文艺,喵,任性
旅游,星座命理,美食,文艺,娱乐
美女,教育就业,娱乐,时尚
汽车
旅游
旅游,美女,南川区,南城街道,团支部,南川区官地村
旅游,娱乐
时尚,健康,美食,文艺,教育就业
美食,汽车,体育,娱乐,时尚潮流,性感,时装,时尚
星座命理
旅游,娱乐,健康,时尚,投资理财,教育就业,星座命理
文艺
体育
穿衣美容,星座命理,名人明星,广东生活,视频音乐
美女,汽车,文艺,体育,娱乐,新闻资讯
新闻资讯,IT 数码,时尚,旅游,健康
时尚
时尚
文艺,教育就业,爱新闻,爱广播,爱摄影,爱拍摄,韩师 10 广电一

健康,娱乐,教育就业,新闻资讯,美食
新闻资讯,旅游,教育就业,文艺,IT 数码
体育,IT 数码
汽车,桌球,微笑,随性,怀旧,摄影爱好者,看电影,听音乐
汽车,体育,旅游
旅游,IT 数码,汽车
娱乐,文艺,星座命理,美食
美食,星座命理,时尚,投资理财,娱乐,IT 数码
星座命理
新闻资讯,美食,文艺,健康
新闻资讯,娱乐,投资理财,健康,教育就业
游戏动漫,猫,动漫,妄想症,幻想控
IT 数码,投影,展览设计,展览展示,发布会,多媒体,科技,广告,传媒
强迫症,美食,生活,吃货,吃,新闻趣事
美食,健康,时尚,汽车,娱乐,星座命理
IT 数码
美女,军事,文艺
体育
文艺,旅游,星座命理,健康,新闻资讯
读书分享,教育,深圳生活,健康,美女,汽车,IT 数码,娱乐
美女,娱乐
美剧迷,孩子气,逛街,无厘头,生活大爆炸,街拍,淘宝控,服装,旅游美食爱好者,欧美
天然呆,二货,腐女,大爱丶 MIC 男团,疯子,温柔,摩羯,音乐控,cosplay,吃货
旅游
网球,旅行,朋友,打篮球,亲情,music,心理,fashion,科技,美女
健康
旅游,台球,小说,看书,篮球,发呆,体育,睡懒觉,缺爱 ing
教育就业
投资理财
美食
旅游
娱乐
汽车,体育
健康,文艺,IT 数码
90 後,小说迷,无厘头,娱乐
美食,时尚,旅游,文艺
旅游,梦想,爱睡觉,可爱
文艺
娱乐
新闻资讯
体育
时尚
科幻
健康,吸血鬼日记,爬山,英语,看电影,玩儿,好书,怀旧,手机看小说,旅游

旅游
新闻资讯
文艺,投资理财,时尚,健康,旅游
娱乐,军事
健康,美食,IT 数码,文艺,体育
美女,娱乐,军事
美食
娱乐
时尚,健康,旅游,娱乐
文艺
美食,穿衣美容,微博奇葩,搞笑幽默,文艺,体育,娱乐
唱 k,HR,服装,旅游爱好者,经济,水瓶女,外贸,睡懒觉,销售,胡思乱想
游戏动漫
汽车,时尚,旅游,美食,IT 数码
旅游,名人明星,北京生活,穿衣美容,美女
文艺,娱乐
星座命理,健康,美食,体育,文艺,军事,时尚,IT 数码,游戏动漫
文艺
汽车,旅游,健康,军事,星座命理,IT 数码,娱乐
旅游
游戏动漫,时尚,文艺,军事,IT 数码
自由,双子,遊戲動漫,文藝
萌宠,娱乐
娱乐
汽车,娱乐,美食,IT 数码
美食,新闻资讯
娱乐
游戏动漫,蓝色,动漫
美女,美食,体育,娱乐
旅游,汽车,时尚,美食,IT 数码
美食,星座命理,美食爱好者,电视剧,团购,音樂,射手座,安静,爱电影,爱旅游
娱乐
美食,时尚,新闻资讯,旅游,娱乐
李胜贤,崔胜铉,姜大声,东永裴,权志龙
创业,投资,ios,互联网,移动通讯,android,日语,天使投资,vc,85 后
娱乐,IT 数码,军事,新闻资讯,时尚
文艺
娱乐
文艺
骑自行车乱逛,交友,旅游,护士,天枰座 A 型女
炎
时尚,新闻资讯,美食,投资理财,军事
游戏动漫
新闻资讯,美食,娱乐,美女,时尚,汽车

美食
汽车,美女,体育
星座命理
美食,旅游
娱乐
星座命理,娱乐
娱乐,文艺,美食,射手座,偶稀饭睡觉觉,善良,生活,爱自由,电影爱好者,旅游美食爱好者
新闻资讯,投资理财,旅游,文艺,娱乐
时尚
游戏动漫,美食,IT 数码,文艺,汽车
IT 数码,汽车,游戏动漫,旅游
美女
汽车,游戏动漫,美女,旅游,美食,文艺,体育
微博奇葩,文艺,时尚,健康,娱乐
教育就业,美食,文艺,健康,时尚,旅游
汽车,旅游,体育,娱乐
遗憾至此,我的歌声里,泡沫,沫沫,关注李代沫,广东泡沫联盟,如果没有你,末日不孤单,中国好声音,李代沫
微博奇葩,IT 数码
娱乐,投资理财,健康,旅游,汽车
教育就业
娱乐
体育,游戏动漫
文艺,旅游,健康,时尚,娱乐
健康,旅游,美食,体育,IT 数码,校园 110,伪 Geek,爱折腾的娃
宠物图集
旅游
文艺
东莞生活
新闻趣事
旅游,投资理财,汽车,IT 数码
星座,羽毛球，游泳，
视频音乐,投资理财
教育就业,健康,新闻资讯,娱乐,文艺
视频音乐,投资理财,新闻资讯,IT 数码
娱乐
IT 数码
汽车
时尚
音乐,看电影,听音乐,敏感,淡定,学生一枚,天秤,文艺
旅游
汽车,投资理财,旅游
体育资讯,美食,旅行,音乐,听歌,电影,破坏
视频音乐,旅游,穿衣美容,萌宠,搞笑幽默

健康,美女,旅游,美食,文艺
文艺
健康,新闻资讯,星座命理,旅游,美食,文艺,娱乐
星座命理,娱乐
星座命理,美食,娱乐
美女,时尚,体育,娱乐
商业,IT 数码
穿衣美容,文艺
时尚,星座命理
IT 数码,教育就业
浪漫,玩儿,健康,美食,时尚,娱乐
娱乐,体育,幻想主义,乐观,爬山,双鱼座,游戏,篮球,摇滚,K 歌
微博奇葩,文艺
汽车
天蝎座,暮光之城,教育就业,时尚,文艺,娱乐,喜欢音乐,大叔控,懒人,爱唱歌
旅游,文艺
美食,旅游,时尚
星座命理,文艺,娱乐
军事,娱乐,投资理财,汽车,旅游,喜欢旅游
顺其自然,一切随缘,美女,旅游,时尚,汽车,娱乐
体育,汽车
AJ,新百伦,马丁靴,潮物,美衣,韩国,化妆,护肤
汽车,美食,娱乐,时尚,教育就业,IT 数码,投资理财
投资理财,体育,韩流,韩剧,美女,娱乐,美食
微博奇葩,搞笑幽默,军事
美食,穿衣美容,健康,看书,英语,校园,坚强,自然,吃货一枚,喜欢音乐
健康,星座命理,旅游,汽车,军事
视频音乐,体育
体育,IT 数码
搞笑幽默
时尚,美食,文艺,健康,教育就业
新闻趣事,撕烂腹肌
娱乐,投资理财,KR
教育就业,文艺
美食,IT 数码,文艺,教育就业,健康
美女,IT 数码
文艺,IT 数码
萌宠,星座命理,娱乐
文艺,教育就业,新闻资讯
美女,时尚,体育,军事,IT 数码
投资理财,美女,汽车,IT 数码
新闻资讯
旅游,文艺
交友,处女座,B 型血,猫控,时尚,旅游,美食

旅游,健康,文艺,娱乐,美食
90 後,娱乐
投资理财
穿衣美容,文艺
处女座,爱电影,听歌,时尚,85 后
旅游,其他生活,搞笑幽默,微博奇葩,新闻趣事
娱乐
时尚,娱乐,教育就业,文艺,旅游,投资理财
旅游,文艺,娱乐,游戏动漫,鳗鱼,手机控
美女,娱乐
美女,体育
文艺,健康,旅游,娱乐
星座命理,文艺
电影,军事,体育
视频音乐,广州生活,新闻趣事
旅游,美食,娱乐
穿衣美容
健康,旅游,星座命理,美食,娱乐
投资理财,健康,美女,美食,军事,IT 数码
汽车,美女,文艺,美食,IT 数码,游戏动漫
美食,IT 数码,汽车,教育就业,体育,娱乐
健康,新闻资讯,游戏动漫,时尚
时尚,健康,美食,旅游
名人明星,娱乐,时尚,军事
投资理财,军事,汽车,IT 数码,体育
美食
吃货一枚,天蝎座,麦霸,动漫控,love,美食
娱乐
娱乐
时尚
娱乐
军事
音乐达人,吸血鬼,电影爱好者,神经质,爱电影,爱旅游,90 後,浪漫,音乐爱好者,麦霸
星座命理,娱乐
情绪化,投资理财,时尚,美食,星座命理
嵌入式 linux
游戏动漫,旅游
旅游,美食,娱乐
汽车,军事
教育就业,时尚,娱乐
旅游
星座命理,美食,旅遊,娛樂
穿衣美容
IT 数码

时尚,旅游,美食
汽车
萌宠,微博奇葩,新闻趣事,北京生活,旅游,健康,IT 数码,投资理财,文艺
娱乐
健康,吃货
汽车,游戏动漫,时尚,美女,旅游,娱乐
时尚,健康,旅游,星座命理,IT 数码
美食
太原生活,校园生活,IT 数码,投资理财
美食
创业,电子商务,红酒,培训,独立,退伍小兵,志愿者,闷骚,星座命理,军事
无聊,安静,懒,90 後,90 后非脑残,生活,网购,留学生,互粉,单身
健康
新闻趣事,搞笑幽默,时尚,文艺,教育就业,投资理财,旅游,健康,80 後,品牌
旅游,美食
健康,星座命理,旅游,美食
旅游
美食,巨蟹女
爱笑,爱电影,爱旅游
美女,星座命理,军事,体育,娱乐
时尚,娱乐
武汉生活,健康
星座命理
汽车
健康
美食,A 型血,我就是我,爱唱歌,睡懒觉,咖啡,爱美食
星座命理,IWANTyou,Imissyou,ILIKEyou,Ineedyou,ILOVEyou
IT 数码,娱乐,投资理财,旅游,星座命理
IT 数码
音乐控,矛盾体
狮子座,美食
体育,文艺
goagent,caoliu,美女
时尚
八卦杂谈,视频音乐,美女,美食,睡觉睡到自然醒,逛街,看电影,天蝎,音乐控,90 后但我不脑残
美女
语录,路在脚下,勇敢往前走,星座命理,新闻资讯,投资理财,文艺
新闻资讯,时尚,娱乐,美食,欧美音乐,文化,演员,爱自由,吃喝玩乐,交朋友
汽车
教育就业,投资理财,水瓶座
旅游
网购达人,淘宝控,购物狂,网络营销,85 后,热爱生活,随性,潮,团购,奋斗
星座命理,美食,银魂,水瓶,爱吃,宅女一枚

爱吃,动画,健康,简单,安静,听音乐,爱自由,哈哈
美女,IT 数码,汽车
娱乐,文艺,健康,旅游,星座命理,美食
名人明星,微博奇葩,穿衣美容,旅游,美食
星座命理
杀
新闻资讯,IT 数码,旅游,汽车,军事
二战史,NewAge,足球迷,上海,安静
健康
体育
汽车,旅游,文艺,娱乐
旅游
车世界,旅游,科学探索,读书分享,宝钢金属,宝钢,汽车,动感,青春
美食
时尚
羽毛球,文字,新闻
育儿,80 后宅女,A 型血
星座命理
美女,美食,文艺
时尚,教育就业,文艺
旅游,美食
旅游
时尚,文艺,新闻资讯
娱乐,美食,时尚
新闻资讯,时尚,汽车,旅游
穿衣美容,美食,旅游
美女,投资理财
汽车,IT 数码,旅游,美女
旅游,健康,体育,时尚,美食,娱乐
名人明星
健康
旅游
搞笑幽默,美食
美女
汽車,美女,體育
文艺
微博奇葩,美女,视频音乐,新闻趣事
时尚,文艺,娱乐,新闻资讯,体育
新闻资讯
星座命理,健康,旅游,时尚,娱乐
新闻资讯,时尚,旅游,美食,文艺
娱乐,文艺,游戏动漫
美女
时尚,旅游,健康,美女,娱乐

新闻资讯,旅游,美食,时尚,娱乐
星座命理,旅游,新闻资讯,文艺,娱乐
旅游
星座命理
星座命理
佛山生活,军事天地
旅游,美食,健康,投资理财
IT 数码,文艺,旅游,美食,体育
时尚,健康,汽车,军事
娱乐
新闻趣事,娱乐
文艺,贪睡,mayday,Y,固执摩羯女,旅行,文字
新闻资讯,时尚,IT 数码,美食,体育,文艺,军事,汽车,投资理财,美女
美食,星座命理,旅游,健康,IT 数码
穿衣美容,星座命理,搞笑幽默,旅游,美食
投资理财,旅游,美食,IT 数码,体育
游戏动漫,时尚,美女,文艺
美女
旅游,可爱,美食,金牛女,90 後,爱睡觉,星座命理,时尚
娱乐,游戏动漫,汽车
穿衣美容,旅游,美食,微博奇葩
校园生活
娱乐,00 後,靓女不得不追求,血可流,头可破
汽车,美女,健康,旅游,美食,军事,文艺,体育
时尚,美食
旅游
美女
美食
新闻资讯,星座命理,IT 数码
体育,文艺,娱乐,投资理财,营销,广告,中大南方,创意,策划
娱乐
投资理财,教育就业,体育,新闻资讯,汽车,IT 数码
搞笑幽默,IT 数码,投资理财
体育
新闻资讯,旅游,IT 数码
教育就业,美食,文艺
电影爱好者,旅游美食爱好者,健身,好性格,护肤,80 後,交友,健康,时尚
游戏动漫,吃货,动漫控
教育就业
汽车
美食,娱乐,IT 数码
美食,旅游,投资理财,新闻资讯,时尚,健康
强迫症,看电影,听音乐,交友,游戏,吃,潮,设计,网络,摄影爱好者
美食,娱乐

穿衣美容,美食
名人明星,美食,旅游
名人明星,IT 数码,文艺,娱乐
旅游,萌宠,美食,浙江生活,汽车,娱乐,IT 数码,体育
新闻资讯,体育
魔羯座,装坚强,爱时尚,购物狂,冰山美人
美食,投资理财
娱乐,健康,旅游
时尚,旅游,美食,娱乐,教育就业
新闻趣事,新闻资讯,汽车,教育就业,娱乐,投资理财
体育
美食
广东生活
投资理财,娱乐,军事,文艺,美女
搞笑幽默
娱乐
娱乐
旅游,汽车,美食,美女,时尚
健康,旅游,美食,文艺,娱乐
美女,旅游,军事
美女,教育就业,军事
新闻资讯,教育就业,军事
T9,切尔西球迷,英格兰,曼联,打篮球,体育,娱乐
军事,体育
娱乐,旅游,健康,美食,投资理财,美女
搞笑幽默
体育,娱乐,汽车,美女,IT 数码
星座命理,佛教,跳舞,新浪读书,宅女一枚,新闻资讯,文艺,投资理财,娱乐,健康
旅游,新闻资讯,教育就业,汽车,投资理财
K 歌,鬼步,DJ
福州生活,教育就业
新闻资讯,星座命理,时尚,娱乐,游戏动漫
娱乐,美食
搞笑幽默,时尚
娱乐,美食
星座命理,文艺
忧郁,广告人
旅游,美食,阿坝生活,穿衣美容,新闻趣事
汽车,健康,旅游,时尚,娱乐
旅游
网络,新闻,爱旅游,休闲,搞怪,美食主义者
时尚,美女,星座命理,文艺,娱乐
健康养生,健康保健,健康生活,中医养生,养生,保健,美食,新闻资讯,健康
陕西生活,健康,新闻资讯

美女,旅游,美食,体育
教育就业,新闻资讯,健康,美女,IT 数码
药师佛,蝴蝶牌,泰国佛牌
旅游,健康
安徽生活,共青团,安徽分公司,中国信保
体育,娱乐,法警,活泼开朗,搞笑,水瓶座,K 歌,男人,篮球
游戏动漫
美食,时尚
雁山区,桂林,漓江学院,文艺
汽车,美女,体育,娱乐,投资理财
美女,旅游,娱乐,时尚,美食,汽车
游戏动漫,汽车,IT 数码
新闻资讯
文艺
文艺,旅游,星座命理,美食,娱乐
文艺
旅游
汽车
美女,时尚
美食,教育就业,健康,旅游,文艺,娱乐
娱乐
旅游
娱乐,投资理财,新闻资讯,时尚,教育就业,旅游
IT 数码
美食
美食,新聞資訊
游戏动漫,娱乐
时尚,涂鸦,苹果,看书,睡觉,宅,爱自由,重口味
互粉,k 歌,体育,娱乐
游戏动漫,美食,军事,体育,旅游
校园生活
時尚,遊戲動漫,體育,美食,时尚,美女,游戏动漫,体育
独一无二
旅游,健康,IT 数码,军事,娱乐
美食,新闻资讯,教育就业,投资理财,旅游,军事
文艺,娱乐,汽车,美食,时尚
健康,新闻资讯,文艺,美女,美食
军事,IT 数码
旅游,美食,体育,娱乐
娱乐,旅游,健康,美食,文艺,汽车
旅游,时尚
汽车
好男人就是我,我就是好男人
旅游,健康

时尚,健康,新闻资讯,汽车,旅游,文艺
新闻趣事,旅游,视频音乐
旅游,美食
教育就业,美食,健康,娱乐,新闻资讯
爱时尚，爱睡觉
健康
IT 数码
IT 数码,投资理财,教育就业,美女,旅游,娱乐
新闻资讯,真诚,休闲,爱笑,爱自由,学生一族,独立,心情,冷笑话,简单
美食,健康,旅游
旅游,美容服饰,时尚,美食
美女,美食,健康
美女,健康,星座命理,时尚,旅游,美食,娱乐
搞笑幽默,八卦杂谈,新闻趣事
中医专家,健康
体育,旅游
军事
名人明星,娱乐
教育就业,投资理财,健康,时尚,旅游
体育资讯,IT 数码,旅游,视频音乐,搞笑幽默
80 後,旅游,文艺
美女,游戏动漫
体育
美食,投资理财,IT 数码,时尚,健康
我 2 但是我快乐,我爱心理学,发个呆,犯个傻,汽车,游戏动漫,健康,IT 数码,体育
时尚,旅游,美食,文艺,娱乐
投资理财,军事,IT 数码,体育,娱乐
新闻趣事,穿衣美容,时尚,旅游,健康,星座命理,美食
新闻资讯,投资理财,旅游,时尚,文艺,美食,健康养生,食品营养
穿衣美容,文艺,汽车
美食,娱乐,美女,星座命理,时尚,文艺
美女,美食
时尚,娱乐
文艺
视频音乐,水瓶座,喜欢旅游,美容护肤,音乐控
汽车,星座命理,娱乐,游戏动漫
体育,IT 数码,健康,旅游,美食
汽车,星座命理,文艺
新闻资讯,体育,健康,美食,游戏动漫
娱乐
健康,美女,游戏动漫,美食,娱乐
IT 数码,时尚,文艺,旅游,星座命理,健康
专业设计安装,音丽士影音系统,家庭背景音乐,私家影院构建,室内模拟高尔夫,智能控制系统,远程监控,QQ343713263

美女
健康
健康,旅游,星座命理,美食,娱乐
旅游,健康
影视,K 歌,玩儿,微笑,體育,美食,旅游,名人明星,美女,搞笑幽默
美食,军事,投资理财,文艺,娱乐,汽车,IT 数码
美食,文艺,娱乐,教育就业,时尚
汽车,美女,旅游,美食,娱乐
美食
旅游,IT 数码
搞笑幽默,娱乐,游戏动漫,美女
天秤,旅游
健康,旅游,文艺,体育,娱乐
IT 数码
新闻资讯,体育,汽车,教育就业,投资理财
时尚,教育就业,健康,投资理财,娱乐,美食,美女,文艺
教育就业,培训,语言培训,会计培训,成就培训学校,中小学生,成人培训,美容化妆,国学书法,教育培训
旅游,爱音乐的吃货
乐天派,收藏,美女,汽车,IT 数码,健康,旅游,游戏动漫,投资理财,文艺
文艺
美食,时尚,旅游,星座命理,美食,娱乐
娱乐
IT 数码,体育,娱乐
时尚,星座命理,健康,旅游,投资理财
教育就业,投资理财,娱乐
IT 数码
搞笑幽默,视频音乐,新闻趣事
体育资讯
美食,时尚,旅游
搞笑幽默,文艺
快乐,老当益壮,健康
旅游,美食
美女,教育就业,文艺
美女
淡定,o 型血,投资理财,美食,军事,娱乐,体育,台球,80 後
新闻资讯,娱乐,文艺
美女,旅游,IT 数码
新闻资讯,健康,时尚,教育就业,投资理财,旅游,美食
花样美男,美女
旅游,深圳生活
旅游,汽车,美食,娱乐
汽车,教育就业
娱乐

时尚,旅游,IT 数码,娱乐
投资理财,娱乐,时尚,教育就业
美食,星座命理,健康,时尚,娱乐
娱乐,天秤座,创意,爱生活
军事,体育
美食,健康,娱乐,旅游美食,迷糊
投资理财,健康
美女,教育就业
时尚,IT 数码,健康,投资理财
文艺
宠物,旅游,美食
文艺
娱乐,美女
娱乐,情感,美食
投资理财,汽车
美食,旅游,美女,娱乐
化妆品,文艺,美食,旅游,娱乐,时尚
新闻资讯,投资理财,健康,时尚,旅游,汽车
文艺
新闻资讯,旅游
时尚,健康,新闻资讯,文艺,旅游
时尚,娱乐,旅游,健康,美食
美食,旅游
美食
娱乐
穿衣美容,新闻资讯,时尚,娱乐
散打,宅生活,双鱼,冷笑话,罗志祥,睡觉睡到自然醒,活泼开朗,大学生,爱幻想,喜欢音乐
体育
时尚,星座命理,文艺,美食
投资理财
美女,新闻资讯,汽车,军事,IT 数码
游戏动漫,娱乐,IT 数码
我就是我,奋斗,个性,多愁善感,热爱生活,阅读,沉默,喜欢音乐,水瓶座,时尚
广东生活,新闻资讯,星座命理,美食,旅游,健康,娱乐
国际快递,仿牌,出口
美女,时尚,汽车,健康,百变,曲婉婷,姐妹一起淘,关注时尚潮流,旅游,养生美容
听音乐,电影迷,冷笑话,教育就业,diy,新闻资讯,投资理财,文艺,美食,娱乐
E 神,LOL,music,双鱼,90 后但我不脑残,爱美食,游戏动漫,旅游
时尚,健康
游戏动漫,娱乐,汽车,体育
投资理财,健康,旅游,美食
穿衣美容,星座命理,时尚,娱乐
新闻资讯,IT 数码,美食,汽车,投资理财
美食,娱乐

时尚,投资理财,星座命理
星座命理
至天使～
星座命理
健康,星座命理,美食,IT 数码,娱乐
军事,新闻资讯,时尚,汽车,文艺,体育
美食
娱乐,时尚,星座命理,教育就业,投资理财
微博奇葩,美食,时尚,IT 数码,文艺
汽车,健康,军事,娱乐,旅游
汽车
视频音乐,文艺,娱乐,体育
美食,娱乐
投资理财
穿衣美容,文艺
游戏动漫
星座命理,美食
商派网络,网络分销,电子商务 ERP,B2B,IT 数码,新闻资讯
新闻资讯,投资理财,教育就业,高等学历,MBA,在职研究生,专升本
旅游,健康,美食,文艺
健康
视频音乐,微博奇葩,搞笑幽默,旅游,体育
健康,星座命理,汽车
投资理财,健康,时尚,旅游,美食
军事,美女,游戏动漫,时尚,汽车,教育就业
娱乐
美女,IT 数码
旅游
IT 数码
80 後,努力,旅游美食爱好者
旅游,新闻资讯,投资理财,汽车
美女,贵金属,黄金外汇,时尚,投资理财,健康
动漫迷,看小说,奋斗,购物狂,学习
投资理财,军事,IT 数码
文艺,时尚,健康,星座命理,美食
体育
体育
旅游,健康,新闻资讯,时尚,教育就业,投资理财
安静,金融,新闻资讯,体育
旅游
微博控,数码控,我是新国货,华仔迷,80 后,IT 民工,购物狂,新闻资讯,星座命理,美食
健康,美食,汽车,旅游
健康,投资理财,旅游,美女,IT 数码
美食,健康,旅游,美食,文艺,娱乐

汽车,军事,教育就业,时尚,美女,健康,体育
美食
汽车,娱乐,健康,星座命理,旅游
名人明星,海贼王,白羊女
整形,赴台医美,赴台考察,两岸交流,医疗美容,两岸
新闻资讯,旅游,美食,IT 数码,娱乐
投资理财,健康,时尚,文艺
娱乐
养生,音乐控,宠物,投资理财,教育就业,健康,旅游,文艺
美女,IT 数码
美容,健康,时尚
旅游,美食
时尚,健康,娱乐,坚强,独立,双重性格,重口味,Music,爱自由,梦想
文艺,汽车
体育,新闻资讯,教育就业,投资理财,文艺
游戏动漫
游戏动漫,美女,教育就业,体育,娱乐
体育
美食,时尚,旅游,娱乐
美食,时尚,家居,看电视,文字控,梁静茹,80 后,宅女
健康,游戏动漫,时尚,旅游,美食
体育,美食,旅游
美女,美食,军事,体育
旅游,体育,IT 数码,娱乐,游戏动漫
星座命理,娱乐
健康,美女,游戏动漫
健康,美食,旅游,文艺
投资理财,健康,星座命理,旅游,文艺
投资理财
美食,娱乐,韩剧,音乐爱好者,星座命理
穿衣美容,潮流服饰,女装店主,时尚
星座命理
旅游,健康,体育,教育就业,IT 数码
美食,旅游,文艺,上进,青年,共青团
星座命理,搞笑幽默,穿衣美容,时尚,旅游,美食,文艺
星座命理,美食,娱乐,教育就业,健康
美文,经典,语录,情书,心理学,爱情,治愈系
文艺,星座命理,旅游,健康
星座命理,美食
娱乐,欧美电影控,金牛座 B 型,麦霸
MAD,健康,游戏动漫,娱乐,新闻资讯
健康,娱乐,时尚
萌宠,体育
娱乐,星座命理,文艺

投资理财
游戏动漫,IT 数码,军事,美食,时尚
健康,旅游,教育就业,娱乐
教育就业,美食,旅游,80 后,书法,辅导,英语
我不快乐
投资理财,IT 数码,旅游,体育
娱乐,健康
沉着
IT 数码,大学生,动漫控,魔羯座,小提琴,幻想,听歌,电影,90 后,善良
体育资讯
旅游
星座命理,美食
欧美控,篮球爱好者,独立,音樂,爱自由,电脑,旅游摄影,吃喝玩乐,执着,体育
娱乐,旅游
娱乐
云南生活,视频音乐,搞笑幽默,健康,旅游,美食,文艺,娱乐
文艺
旅游
娱乐,听音乐,爱笑,爱玩,香橙
旅游,文艺,时尚,美食
美食,美食,文艺,新闻资讯,投资理财,健康
旅游,IT 数码
新闻资讯
旅游,星座命理,美食
时尚,娱乐
文艺,健康,旅游
搞笑幽默,美女,名人明星
旅游,汽车,文艺,游戏动漫,体育
新闻趣事,文艺,新闻资讯
娱乐,投资理财
时尚
新闻资讯,pertroleum,科技二路,山东曲阜人,娱乐,健康,汽车,旅游,体育
娱乐
文艺,时尚
IT 数码,娱乐,汽车,时尚
体育
美食,旅游
美食,教育就业,投资理财,健康,旅游
美女
投资理财,新闻资讯,游戏动漫,IT 数码,教育就业
娱乐,美食,时尚,健康,旅游
IT 数码,汽车,体育
体育
投资理财

美食,体育
军事,IT 数码,文艺,体育,娱乐
健康,IT 数码
睡懒觉,海贼王,狗狗,爱旅行,狮子座,90 后,美食
文艺
旅游,新闻资讯,汽车,美食,体育
IT 数码,体育
美女
浪漫,体育,交朋友,主持人,电影爱好者,爨柏景区,明清古宅套院,老房子,旅游,爨底下
文艺
旅游,美食
时尚
旅游,体育,开不了口丶
体育
美容服饰,读书分享,购物,睡懒觉,热爱生活,善良的糊涂菇凉,爱美爱吃爱看书,有态度的吃货
健康
娱乐,体育
教育就业,星座命理,娱乐
萌宠
東方神起,cos,游戏动漫
星座命理,时尚,文艺,娱乐
时尚,娱乐,文艺,旅游
军事,文艺,游戏动漫,IT 数码,音乐迷,动漫迷
投资理财,旅游
喜欢唱歌,喜欢旅游,市场营销,90 后但我不脑残,0 型血,活泼,奋斗,爱美食,投资理财,时尚
汽车,美女,娱乐,体育
体育,美女
美食,星座命理
星座命理,青春,90 后但我不脑残,宅女,少女时代
健康,旅游,投资理财,娱乐
美食,旅游,健康,时尚,巧克力,单纯
时尚
体育,旅游
搞笑幽默,娱乐
娱乐
军事
名牌家居,品牌装修公司,室内设计,家装设计,实创装饰,装修
娱乐
军事,健康,娱乐,旅游,文艺,美食,汽车,新闻资讯
美食,娱乐,美女,体育,旅游
穿衣美容,跑步,热情
投资理财,旅游,星座命理,美食,健康,育儿亲子,育儿交流
美女,时尚

美食,旅游,娱乐
投资理财,时尚,美食,旅游,娱乐
时尚,文艺,娱乐,健康
教育就业,军事,IT 数码
时尚,旅游,IT 数码,文艺
教育就业,旅游,时尚,军事,IT 数码
娱乐
新闻资讯,投资理财,时尚,旅游,娱乐
爱生活,舞蹈,紫色,英语,体育,喜欢音乐,羽毛球,孩子气,活泼开朗,乐观
旅游,军事,游戏动漫,体育,原创,艺术,咖啡,影视,书籍,女人
娱乐,投资理财,文艺,时尚,美食,旅游
汽车,美食,娱乐,健康,时尚
汕头生活,体育,阳光,高考,创业,奋斗,学习
健康,时尚,文艺,汽车,美食,旅游,美女
名人明星,搞笑幽默,视频音乐,新闻资讯,游戏动漫,美女,IT 数码,文艺,体育,娱乐
文艺,烹饪达人,吃货一枚,服装,80 後
军事
文艺,美食,娱乐
星座命理
健康,文艺,时尚,旅游,美食
新聞資訊
娱乐
美食,旅游,星座命理,体育,娱乐,上网,阳光,大学生,奋斗
留学,投资,金融,A 股,财经,投资理财,星座命理
文艺
美食,星座命理
文艺
囧,旅游美食,旅游
娱乐
搞笑幽默,军事
健康,奋斗,善良,安静,金牛,低调,多愁善感,减肥美容养生
游戏动漫
教育就业,健康,旅游,军事,文艺
美女
游戏动漫
美女
IT 数码,文艺,投资理财,健康,旅游,美食
娱乐,汽车,体育,IT 数码
教育就业,投资理财
健康
教育就业,美食,军事,投资理财,IT 数码,文艺
旅游
新闻资讯,旅游
美食,投资理财,健康,新闻资讯,文艺

新闻资讯,投资理财,时尚,旅游,美食
旅游
旅游
时尚,健康,旅游,美食
蚌埠生活,文艺,游戏动漫,旅游,星座命理,美食
美女,投资理财,IT 数码,美食,健康,娱乐
旅游,汽车,新闻资讯,军事
旅游,娱乐
新闻资讯,旅游,文艺
新闻资讯,IT 数码,游戏动漫,娱乐
星座命理,游戏动漫
军事,星座命理,旅游,健康,娱乐,IT 数码,体育,投资理财
投资理财,军事,美食,时尚,教育就业,新闻资讯,文艺
信息技术革新,信息扩散,信息系统,加州大学伯克利,PhD
亲子育儿,投资理财,旅游,娱乐
教育就业,汽车,文艺,IT 数码,军事
娱乐
娱乐,无厘头,活泼开朗
时尚
花样美男,乐橙一枚,魏晨控
旅游,青春,商业,爱猫,A 型血,随性,巨蟹,小说
IT 数码,军事
美女
投资理财,时尚,健康,教育就业
美食,娱乐
美女
文艺,投资理财,娱乐,美食,旅游,教育就业,新闻资讯
汽車,遊戲動漫,軍事,IT 數碼,文藝,體育,娛樂,娱乐,美女,美食
娱乐
名人明星,娱乐,庚饭,天蝎座,火影忍者,90 後
IT 数码
娱乐,山东师范大学,山东人,西宁,鲁能泰山球迷,青藏高原,植物分类,中科院
健康,旅游,美食,IT 数码,体育
IT 数码
游戏动漫,星座命理,投资理财,时尚,健康,新闻资讯,教育就业,美食,IT 数码,娱乐
美食
开朗,广泛,47 操作者
文艺,IT 数码
死神,游戏动漫,汽车,娱乐,体育
星座命理
文艺,吃货,腐女,发呆,宅,90 后,孩子气,强迫症,天蝎女,美食
星座命理
怀旧,摄影,爱睡觉,懒,闷骚,宅女一枚
旅游

购物,music,时尚,旅游,娱乐
双鱼,宅女一枚,娱乐
时尚,健康,旅游,投资理财,娱乐
时尚,IT 数码
旅游,IT 数码
新闻资讯,健康,时尚,文艺,投资理财
健康,美食,旅游
健康,旅游,美食,娱乐,投资理财
玩
美食
娱乐
美食,游戏动漫
IT 数码,体育,汽车,美女,娱乐
星座运势,搞笑幽默,投资理财,IT 数码,旅游,星座命理,美食
教育就业,时尚,星座命理,体育,娱乐
旅游,娱乐,随性,学生一枚,夜猫子
搞笑幽默,北京生活,美图摄影,美女,健康,汽车,娱乐,IT 数码
旅游,时尚,游戏动漫
投资理财,美食,星座命理,旅游,时尚
投资理财
旅游,美食,穿衣美容,星座命理,新闻趣事
星座命理,汽车,健康,时尚
娱乐,疯子,格子控,无聊,K 歌,music,书,心理,星座命理,文艺
时尚,汽车,旅游,军事,IT 数码
时尚,美食,听歌,健康
旅游,美食
游戏动漫
时尚,读书,微博,爱情,创业,娱乐,美食,旅游,健康,新闻资讯
时尚
美女,美食,吃货,看小说,夜猫子,闷骚
旅游,开心果,很极端,白羊座,顶级吃货,90 后呀 90 后,我是吴毓颖啊
IT 数码
教育就业,美女,汽车,旅游,军事,文艺,体育
娱乐
健康,时尚,娱乐
旅游
美食,娱乐
时尚,健康,投资理财,旅游,美食
游戏动漫,文艺,军事,美食,旅游
美食
游戏动漫,军事,投资理财,娱乐
新闻趣事
学生,宅,听歌,90 后,IT 数码,健康,星座命理,美食,文艺,娱乐
美食

IT 数码,健康,时尚,投资理财,美食
美女,搞笑幽默
时尚
读书分享,搞笑幽默,校园生活,新闻趣事,八卦杂谈,健康
体育,美食,游戏动漫,旅游,桌游吧,娱乐
游戏动漫
梁静茹,摄影控,蓝色,画画,爱电影,足球,懒,安静,魔羯座,google
美食,日立电梯,80 后
体育,IT 数码
娱乐
游戏动漫,美妆控,婚纱控,动漫,彩妆,吃货,插画,爱玩,时尚潮流
健康,美女,旅游,汽车
搞笑,宅女,微博控,吃货,天秤座,任性,单纯,天秤,影视,爱吃
美女,新闻资讯,健康,旅游,美食,体育,娱乐
游戏动漫
双鱼,高中生,文字控,吃货
汽车,体育,阿迪控,篮球迷
健康,新闻资讯,投资理财,旅游,文艺
美女
教育就业,娱乐,军事
美食,IT 数码,军事,新闻资讯,投资理财,教育就业,时尚,美女
健康,旅游,美食
文艺,时尚,健康,美食
投资理财,娱乐,教育就业,时尚,旅游,新闻资讯,军事,文艺
娱乐,教育就业
视频音乐,搞笑幽默,浙江生活
旅游,游戏动漫,宅男 90 后,冒险,游行,动漫,小说,武术,张家界
健康,星座命理,旅游,美食
新闻趣事
投资理财,娱乐,美食,旅游,文艺,IT 数码
偏执狂,自拍,90 後,狮子座,小女人,睡觉睡到自然醒,美食
美食,汽车,健康
旅游,新闻资讯,健康,时尚,文艺
羽毛球,文艺,新闻资讯,投资理财,汽车,时尚
美食,健康
文艺,娱乐,教育就业,体育
星座命理,游戏动漫
美容服饰,情感生活,美食,美女,娱乐
动漫,文艺
投资理财,旅游,健康,星座命理,美食,娱乐
心理学,钢琴,音乐控,宠物,火影忍者,热爱生活,美食爱好者,幸福,韩剧,美食
文艺,体育
健康,娱乐,汽车,教育就业,时尚,旅游,军事,体育
新闻资讯,美食,体育,旅游,文艺,有点内向消极,向往自由与自然

旅游
时尚
时尚,娱乐,星座命理,文艺,天蝎女
游戏,乐观
军事,美食,娱乐,文艺,旅游
好好做人,旅游,美食,美女,体育
旅游,美食
旅游
美食,旅游,文艺
时尚,健康
旅游,健康,美女
游戏动漫
军事
健康,娱乐,文艺,时尚,旅游
星座命理,投资理财,旅游,美女,美食
健康,时尚,美女
IT 数码
娱乐,旅游
娱乐
美女
IT 数码,旅游
投资理财,健康
唱歌,健康,新闻资讯
娱乐,旅游
双鱼座的幻想女,写作随心,时装
旅游,美食,体育,娱乐
旅游,美食,军事,教育就业,新闻资讯
娱乐
文艺
新闻资讯,教育就业
牛栏,美赞臣,美素,射击,尿布,奶粉,港货代购,狩猎,真诚,美容护肤
旅游,星座命理,IT 数码,文艺,娱乐
时尚
新闻资讯,旅游,游戏动漫,文艺,体育
IT 数码,唱 k,海绵宝宝
新闻趣事,美食,育儿百科,旅游,军事,体育
教育就业,星座命理,爱自由
美食,娱乐,旅游,时尚,星座命理
喜欢旅游,Music,天蝎座,红酒,大学,单身,B 型血
游戏动漫
美食,健康,美女,时尚,娱乐
情感生活,健康
疯子
体育,娱乐,IT 数码,投资理财

新闻资讯,美女,时尚,体育,娱乐
网络营销,媒体,喜欢音乐,愤青,电影控,市场营销,电影爱好者,交朋友,唱K,o型血
娱乐
AIESEC,摄影,康熙来了,k歌,学习,天秤,爱美食,聊天,咖啡
时尚
星座命理,微博奇葩,搞笑幽默,新闻趣事,萌宠,时尚,健康,游戏动漫
健康,时尚
娱乐
娱乐,游戏动漫
娱乐
游戏动漫,星座命理,无厘头,爱笑,懒,听音乐,白羊
旅游
视频音乐,微博奇葩,娱乐
矛盾体,执着,善良,多愁善感,美女,时尚,IT数码,娱乐,投资理财
星座命理,娱乐,健康,旅游,体育
娱乐
军事,美女
美食
星座命理,名人明星
旅游,体育
文艺
娱乐
星座命理
IT数码
时尚,健康,教育就业,旅游
旅游,视频音乐,美容服饰,情感生活,时尚,美食,新闻资讯,健康,娱乐
美女,军事,旅游,投资理财,体育
教育就业,时尚,旅游,健康,美食
时尚,娱乐
测绘工程,交通运输,给水排水,工程管理,土木工程,阅读,学生,思考,辩论,体育
文艺
星座命理
教育就业,美女,新闻资讯,汽车,投资理财,旅游
游戏动漫
娱乐
游戏动漫,娱乐
文艺,美食,旅游
美食
星座命理,旅游,健康,美食
軍事,遊戲動漫,汽車,美女,體育,八卦
视频音乐
新闻资讯,游戏动漫,星座命理
文艺,IT数码,旅游,游戏动漫,汽车
美食

旅游,美女,视频音乐
旅游,新闻资讯,投资理财,军事,IT 数码
娱乐
健康,新闻资讯,时尚,旅游,星座命理
视频音乐,旅游
健康,文艺,时尚,汽车,美食,静动兼具
时尚,文艺,娱乐,旅游,美食
视频音乐,名人明星
旅游
文艺
星座命理,健康,娱乐
健康,旅游
IT 数码,文艺,求妹子关注阿,娱乐
汽车,投资理财,娱乐,健康,新闻资讯
教育就业,游戏动漫,美食,旅游
IT 数码,时尚,娱乐
天秤座,希望多交些朋友,娱乐
时尚
IT 数码
情感生活,健康,时尚,文艺,娱乐
IT 数码,娱乐
旅游,美食,视频音乐
健康,投资理财,时尚,美食
美女,时尚,文艺,娱乐,健康
时尚
美女
娱乐,教育就业,时尚,旅游,文艺
美食,游戏动漫
旅游,星座命理,汽车,美食,IT 数码
健康,美食,旅游
健康,快乐,健身,运动健康美丽,电影爱好者
旅游,教育就业,汽车,健康,新闻资讯,美食,娱乐
星座命理
旅游,星座命理,健康,游戏动漫,时尚,娱乐
投资理财,美食,健康,旅游
教育就业,健康,旅游,美食,娱乐
投资理财,军事
体育,娱乐
美食
体育
娱乐,IT 数码,新闻资讯,旅游,汽车
运动,娱乐,体育,汽车,IT 数码
娱乐
时尚,教育就业,星座命理,美食,美女,IT 数码,体育,新闻资讯,投资理财,文艺

新闻资讯,时尚,美食,游戏动漫,星座命理,文艺
体育,星座命理,旅游,娱乐,文艺,宅在二次元,混迹三次元
军事
军事,新闻资讯,汽车,美食,游戏动漫,教育就业,文艺,美女,星座命理,娱乐
娱乐
美食,时尚,旅游,健康,星座命理,娱乐
搞基,宅男,时尚,汽车,美女,星座命理,军事,健康,旅游,美食
投资理财
美女
健康,时尚,旅游,星座命理,美食
教育就业,投资理财,美食,游戏动漫,IT 数码,星座命理,文艺,新闻资讯,旅游,娱乐
娱乐
文艺,娱乐,健康,美食,旅游
PC 游戏控,萝莉控,我就是爱音乐,二货屌丝,狂热拜仁粉,德国队死忠,娱乐,体育,投资理财,文艺
美女,新闻资讯
游戏动漫,时尚,旅游,美食,娱乐,文艺
健康,时尚,美食,娱乐,IT 数码
燃烧的小宇宙,真实且不做作,笑点低哭点更低,纠结人生
IT 数码,新闻资讯,健康
军事
投资理财,旅游,文艺,IT 数码,体育
电视剧迷,文学,NBA,Music,散步,体育,新闻资讯,旅游,娱乐
旅游
健身爱好者,股票,开户,基金投资,期货投资,河南生活,股票账户开立,理财服务咨询,基金账户开立,投资理财
旅游,健康,美女,娱乐,时尚
澄海,澄海鹰眼,澄海交警,澄海交通
旅游,文艺,娱乐
善良幽默的吃货
娱乐,新闻资讯,美食,旅游,时尚,健康
IT 数码,旅游,美女
IT 数码,娱乐,听歌，睡觉,吸血鬼日记
旅游,军事
新闻资讯,娱乐,游戏动漫
旅游,健康
体育资讯,海贼王,天枰座,动漫迷,爱睡觉
星座命理,教育就业,美食,新闻资讯,文艺
娱乐
美女,娱乐
南宁生活,美食
教育就业,IT 数码
娱乐
幸福,完美主义,语录,广告,80 后宅女

娱乐,时尚,美女,健康
无知,无能,无为
汽车,美女,旅游,美食,娱乐
美食,星座命理,旅游
旅游,娱乐,美食,军事,体育
萌宠
文艺,美女,星座命理,健康,旅游,汽车,唱歌,健身,爱电影
旅游,娱乐,IT 数码
美女,IT 数码
名人明星
旅游,留学大马,马来西亚留学生,马来西亚生活,大马哈,美食,娱乐
娛樂
上网,臭美,插画,宅,美女,幸福,游戏动漫,星座命理,IT 数码,文艺
旅游
星座命理
时尚
游戏动漫
旅游,健康,时尚,投资理财,娱乐
新闻趣事
时尚
旅游,教育就业,美食,文艺,投资理财
游戏动漫,娱乐
娱乐,旅游
游戏动漫
美食,旅游
搞笑幽默,睡觉睡到自然醒,电视剧,旅游美食,爱自由,逛街,摩羯女
新闻资讯,体育,教育就业,投资理财,健康,一根网线,一台电脑,住家创业
娱乐
心理,自由,青春,旅游爱好者,射手座,music,正太控,学生一族,摄影控,90 後
时尚,娱乐,星座命理,美食,旅游
造型,时尚
新闻资讯
教育就业,IT 数码,文艺,旅游
维他命,健康,旅游
文艺,健康,星座命理,娱乐,旅游,美食
搞笑幽默,IT 数码
体育,娱乐,投资理财,IT 数码,旅游,美食
教育就业
健康
美术,画画,O 型血,k 歌,微博,NBA,金牛座,90 後,运动,篮球
服装,安静,喜欢音乐,坚强,B 型血,陈奕迅,文化,书籍,独立,旅游
新闻资讯,健康
时尚,旅游,美食,文艺,娱乐
娱乐

星座命理,旅游,投资理财,娱乐,美食
文艺,投资理财,时尚,美女,IT 数码
90 后
旅游,视频音乐,新闻趣事,搞笑幽默,武汉生活
教育就业,旅游
羽毛球,汪苏泷,五月天,音乐
狮子女,营销,旅游爱好者,浪漫,温柔,A 型血,基督徒,真诚,80 後,善良
游戏动漫,美食,娱乐,美女,本人钟壮,毕业于 33 中,现在的八中北区,真诚
健康
运动性感,潮流运动,运动时尚,热爱生活,时尚达人,健康,美女,IT 数码,时尚,娱乐
美食,娱乐
美食,旅游,健康,星座命理,投资理财
旅游
游戏动漫,健康,娱乐,IT 数码,体育
游戏动漫,体育,旅游,星座命理,美食
星座命理,时尚,娱乐,文艺,旅游
游戏动漫,体育,新闻资讯,文艺
教育就业,新闻资讯
汽车,旅游,健康,时尚,美食
汽车,IT 数码,美食,娱乐
新闻趣事
健康
新闻资讯,健康
娱乐
投资理财,汽车,时尚,美食,旅游
星座命理,军事,文艺,时尚,教育就业,新闻资讯
投资理财,IT 数码,体育,文艺,军事,美食
专业人士,经济管理,摄影,在重庆的成都人,電影,跳跃性思维,媒体人,白羊女,大学生,艺术
新闻资讯
娱乐,时尚,星座命理,健康,美食,投资理财,旅游
星座命理,投资理财,教育就业,健康,文艺,阿拉伯语,小说爱好者
教育就业,新闻资讯,投资理财,健康,时尚,娱乐,旅游
新闻资讯
体育
新闻资讯,健康,旅游,文艺,美食
娱乐,教育就业,星座命理
体育,IT 数码
旅游,健康,游戏动漫
IT 数码,文艺,时尚,健康,美食,爱生活
星座命理
美食,星座命理,娱乐
美食,旅游,名人明星,穿衣美容,搞笑幽默
教育就业
军事,新闻资讯,健康,教育就业,状态估计,新闻,汽车

美食,教育就业,文艺,瘦身,个性,健康,新闻资讯,旅游,娱乐,时尚
育儿百科,娱乐
美食,养生,读书和思考,育儿,旅游
时尚,教育就业
搬图,二次元,黄濑厨,动漫迷,渣画手,兵长厨,博爱党,动漫控,腐女,正太控
浙江生活
投资理财,美食,时尚,星座命理,文艺,娱乐
旅游,新闻资讯,星座命理,文艺,教育就业,投资理财
美女,汽车,投资理财,游戏动漫
微博奇葩,文艺青年,多愁善感,爱旅行,绘画,书法,90后但我不脑残,盗墓笔记,健康
吃货派,旅游
健康,文艺,投资理财,时尚,旅游,美食,星座命理
旅游,生活大爆炸,90后,娱乐,新闻资讯
教育就业,体育
茶,fashion,品牌,新闻趣事,汕头生活,旅游,美食,穿衣美容
IT数码,时尚,旅游,汽车,娱乐,投资理财,广州宠物狗,纯种,健康,新闻资讯
美食,旅游,游戏动漫,美女,IT数码
娱乐
热血控,東城不敗,腐
文艺
汽车,军事,美食,体育
娱乐,旅游
旅游,体育,汽车,美食,娱乐,时尚
时尚,美食,旅游
投资理财,汽车,新闻资讯,旅游,星座命理,IT数码
美食,IT数码,旅游
娱乐,体育,古典艺术,健康,70后,化妆
购物,美食,旅游,星座命理,健康,时尚
文艺,旅游,美食,时尚,健康
美食,IT数码,娱乐,汽车,体育
时尚,娱乐,美女,美食,体育,健康
穿衣美容
星座命理,美食,军事,IT数码,文艺
游戏达人,美剧爱好者,电影爱好者,幽默开朗,低调,娱乐,旅游,健康,美食,IT数码
娱乐,美女
游戏动漫,劳资就是单机控,天秤座是什么,宅男肿么了,乃萌这群傲娇受,你才无节操,万年总攻,基你一脸,我是好人妥妥的,这特么的不科学
旅游
旅游,文艺,汽车,时尚,美食
美食
寫詩,2014comeback,店,一年後的
美食,旅游,搞笑幽默,视频音乐
五月天
深圳生活

娱乐,星座命理,八卦,电影控,完美主义,美食爱好者,时尚达人,活泼开朗,80 后宅女,旅游
投资理财,健康,旅游,美食,IT 数码
娱乐,动漫控,爱吃,炎亚纶,犬夜叉,听音乐,爱音乐
旅游
娱乐,游戏动漫,体育
IT 数码,游戏动漫
美女
文艺,娱乐,时尚,旅游,美食,星座命理
新闻趣事,搞笑幽默,双鱼座
美食,体育,娱乐,汽车,旅游,星座命理,军事,IT 数码
文艺
文艺
星座命理,美食,娱乐
旅游,时尚,美食,娱乐,文艺
时尚,游戏动漫,投资理财,美食,星座命理
旅游
娱乐,健康
娱乐,旅游
90 后但我不脑残,我就是我,文艺,娱乐
新闻资讯,教育就业,健康,星座命理,美食
文艺
汽车,娱乐
美女,健康,游戏动漫,IT 数码
娱乐
美食,娱乐
旅游,娱乐
星座命理
安静,发呆,基督徒,韩国,钢琴,爱自由,内向,学生,90 后,听歌
旅游
娱乐
旅游,星座命理,美食,娱乐,文艺
美食,健康,时尚,IT 数码,新闻资讯
星座命理,新闻资讯,旅游,娱乐,游戏动漫
健康,娱乐,旅游,美食
星座命理,名人明星,搞笑幽默,重庆生活,视频音乐
娱乐
穿衣美容
教育就业
睡觉睡到自然醒
美女,美食,IT 数码,文艺,体育,娱乐
美女,健康,新闻资讯,美食
星座命理,娱乐
IT 数码
EXO,行星饭,兴鹿党,永爱兴鹿

军事,时尚,投资理财,IT 数码,市场营销
游戏动漫,旅游,体育,娱乐
名人明星,萌宠,搞笑幽默,新闻趣事,美女,新闻资讯,娱乐
文艺
星座命理
时尚,汽车,旅游,娱乐,健康
娱乐,星座命理,体育
游戏动漫
星座命理,健康,美女,美食
文艺,时尚,美食,旅游,娱乐
教育就业,健康,旅游
旅游,七杀,破军,贪狼
娱乐
佛學,美食,文艺
体育
时尚,健康,新闻资讯
IT 数码,健康,教育就业
游戏动漫
旅遊,意大利生活,成熟,暗恋,旅游,意大利,Fallen,堕落 Fallen,多重人格,努力
高中生,阳光,快乐大本营,浪漫,英语,音乐迷,美食,文艺,时尚,娱乐
双子座,belieber,美食
新闻资讯
娱乐,游戏动漫
体育,追星 JJ 林俊杰,娱乐,IT 数码
汽车,旅游,时尚,美食,文艺,娱乐
星座命理
穿衣美容
旅游,军事,投资理财,美食,体育
穿衣美容,游戏动漫
美女
时尚,健康,美容护肤化妆,宅,猫,天枰座
新闻资讯,健康,投资理财,文艺,旅游
吃喝玩乐
旅游
完美主义,吃货,90,潮,睡觉睡到自然醒,银魂,动画,爱猫,臭美,欧美控
美食
时尚,文艺,娱乐,健康,美食,活泼开朗,旅游
插画,原创,美女,时尚,旅游,美食,文艺
微笑,游戏动漫,健康,IT 数码
有爱的人,教师
时尚,美食,娱乐,星座命理,文艺
健康,汽车,教育就业,星座命理,体育
军事
美女

时尚,娱乐
游戏动漫
时尚,music,书,喜欢旅游,看电视,投资理财
教育就业,娱乐,文艺,旅游,美食
美容服饰,IT 数码,动漫,宠物图集,美食
新闻资讯,青春,动画,银魂,瘦身,娱乐,时尚,健康,教育就业,旅游
星座命理,娱乐
美女,IT 数码,军事
美食
体育,美女
视频音乐,微博奇葩,萌宠,娱乐,健康,旅游,星座命理,文艺
偶喜欢玩,美剧迷,巨蟹女,生活大爆炸,手工,西安,随性,吃货一枚,自然,吸血鬼日记
美食,文艺,新闻资讯,美女
美食
时尚,美食,旅游,娱乐,体育
美女,时尚,汽车,美食,旅游
搞笑幽默,新闻趣事,视频音乐,教育就业,美食,健康,新闻资讯,投资理财,娱乐,体育
健康,美女,旅游,文艺,美食
体育
旅游
汽车,美食,娱乐,健康,新闻资讯,旅游
旅游
简单,爬山,交朋友,志愿者,文艺,旅游
美食
健康
搞笑幽默,新闻趣事,美图摄影,美食,时尚,旅游,文艺,娱乐
情感生活,IT 数码,星座运势,八卦杂谈,搞笑幽默
投资理财
娱乐
文艺,健康,美食,旅游
娱乐
旅游
时尚
东莞
IT 数码,美食,娱乐,汽车,旅游
淡定,简单,爱美食,开心,健康,新闻资讯,旅游,美食,文艺
美食,文艺,体育,娱乐,健康
瑜伽,旅游爱好者,投资理财,健康,时尚,旅游,美食
视频音乐,体育
淘宝控,摩羯女,美容护肤,瘦身,育儿
美女,新闻资讯,娱乐,时尚,旅游
游戏动漫,体育
龙与地下城,休闲
美食

旅行,大学生
娱乐
时尚,投资理财,旅游,星座命理,懒人,逛街,简单,睡懒觉
娱乐
游戏动漫,娱乐
文艺,旅游,星座命理,美食,时尚
南无阿弥陀佛,南无观世音菩萨,大势至菩萨,印光法师文钞,水瓶座,素食,净土宗,放生,瑜伽
游戏动漫,吃货,宅女一枚,囧,发呆,90 後
美食,学生,宅,幻想,拍照,聊天,无厘头,宠物,神经质,敏感
罗志祥
时尚,星座命理
体育
游戏动漫,军事
军事,健康
游戏动漫,娱乐,美食
娱乐,敢爱敢恨
投资理财,时尚,旅游,美食,娱乐
二次元,日语初学者,PSP 控,90 后,柏木由纪,AKB48,东方 Project,处女座,旅游,游戏动漫
健康,教育就业,体育,军事
美食
投资理财
娱乐
投资理财,金融投资理财,金融投资
美女
星座命理,美食,文艺,娱乐
新闻资讯,星座命理,军事,体育,娱乐,美食,健康,投资理财,旅游
健康
娱乐,疯子
O 型血,水瓶女,美食,星座命理
视频音乐,旅游,读书分享,教育
时尚,教育就业,美食,文艺
视频音乐,娱乐,天秤座,听音乐,偶稀饭睡觉觉,爱吃,宅女一枚,时尚,上网,逛街
穿衣美容
健康,军事,文艺,汽车,时尚
美食,投资理财,新闻资讯,健康,旅游,美食
美食,健康,美女,娱乐,销售,玩,健身,快乐,微博控
文艺
游戏动漫,美女
投资理财,文艺,时尚,健康,旅游,新闻资讯
娱乐
投资理财,时尚,新闻资讯,健康,旅游,娱乐
教育就业,体育,美女,游戏动漫
文艺,健康,美食
投资理财

书籍,睡觉睡到自然醒,幻想,单纯,文艺,旅游
美女,美食,文艺,娱乐
新闻资讯,美女,文艺,娱乐,时尚
娱乐,海绵宝宝
游戏动漫,时尚,IT 数码,投资理财,新闻资讯,军事
星座运势,音乐控,美食,旅游
新闻趣事,宠物图集,健康,投资理财,时尚,星座命理,娱乐
美食,时尚,汽车,旅游,IT 数码,文艺
汽车,美食,星座命理,美女,娱乐,游戏动漫
教育就业
娱乐
汽车
旅游,美女,美食,疯疯癫癫
IT 数码,投资理财,新闻资讯,汽车,体育
视频音乐,体育
星座命理,美食,文艺,游戏动漫,娱乐,小清新,小说控
旅游,美食,美女,时尚,娱乐
NBA 迷,星座命理,旅游,体育
哈哈,白白的,天秤,90 后宅女
看小说,育儿,情绪化,神经质,90 後,活泼,阳光,爱生活,健康,美食
美女,体育
娱乐
校园生活
幽默,投资理财
其他生活,旅游,新闻趣事,美女
美食,旅游,新闻资讯,教育就业,时尚
军事,投资理财,新闻资讯
教育就业,健康,旅游,美食,文艺
搞笑幽默
新闻资讯,文艺,体育,旅游
健康,美食
健康,时尚,体育,美女,旅游,娱乐,汽车
健康,旅游,星座命理,IT 数码
军事
星座命理,投资理财,美女,时尚,娱乐
美食,旅游,时尚,娱乐,IT 数码
健康
动漫,健康,游戏动漫,文艺,体育,娱乐
娱乐,文艺,健康,新闻资讯,教育就业
美食,健康
梦想,美女
美食,旅游,教育就业,投资理财,文艺,娱乐
文艺
美女,IT 数码

健康,投资理财,娱乐,新闻资讯,旅游,时尚,汽车
绵阳生活,旅游,健康,星座命理,时尚,娱乐
想变得更好,快饿死的胖子,话唠属性,有些机智的说
军事
IT 数码
健康,体育,IT 数码,新闻资讯,旅游爱好者,爱吃,服装
旅游,美食,文艺,娱乐,星座命理
文艺,美食
健康,时尚,美女,美食,旅游
美食,娱乐
新闻资讯
汽车,健康,旅游,星座命理,娱乐
军事
旅游,星座命理,美食,时尚,文艺
游戏动漫,体育
旅游
魏晨
IT 数码,文艺,娱乐,时尚
时尚,投资理财,新闻资讯,IT 数码,娱乐,美食,旅游,星座命理
名人明星
娱乐
时尚,投资理财
名人明星,幽默,音乐,时尚
文艺,星座命理
游戏动漫,星座命理,美食
娱乐
美女,文艺
军事,新闻资讯,旅游
旅游,穿衣美容,娱乐
星座命理,旅游,美食,文艺,娱乐
文艺
基友,吃货,天蝎座,自行车旅行,旅游爱好者,活泼开朗,交友,宅,游戏动漫,旅游
旅游
星座命理,微博奇葩,视频音乐,新闻趣事,搞笑幽默
健康,时尚,娱乐
娱乐
星座命理,军事,健康,新闻资讯,时尚,旅游,美食
重庆生活,简单,吃,运动,交友,美女,健康,娱乐,投资理财,旅游
旅游
投资理财,旅游,文艺
郎军,致力音乐,祈求,我不是小丑,娱乐
汽车
娱乐,时尚
军事,美食,健康,美女,汽车,旅游

音樂
时尚,旅游,美食
美食,郭敬明,逛街,美容,爱情,新闻资讯,健康,旅游,星座命理,文艺
时尚,新闻资讯,游戏动漫,旅游,文艺
娱乐,本土,疯人,表演,博文管理学院,音乐人,自由,rock,乐队,摇滚
微童星,小帅哥,昊仔
新闻趣事,情感生活,美食,视频音乐,美容服饰
游戏动漫,娱乐
美女,娱乐,旅游,IT 数码,游戏动漫,美食
新闻资讯,健康,汽车
游戏动漫
时尚
健康,新闻资讯,旅游,美食,军事
美食
游戏动漫
时装
游戏动漫,体育,旅游
军事,IT 数码,文艺,体育,娱乐
娱乐,体育,文艺,旅游,健康,时尚
懒,武艺,烹饪,美食,旅行,电影,听歌,80 后,宅,浪漫
时尚
游戏动漫
游戏动漫,体育
新闻资讯,娱乐,美食,文艺,旅游
亲情,教育就业,军事,娱乐,健康,体育
娱乐,文艺,汽车
时尚,美女,IT 数码,文艺,娱乐
旅游,星座命理,体育
星座命理
美女,投资理财
教育就业,健康,新闻资讯,投资理财,星座命理,游戏动漫
美食,投资理财
穿衣美容,娱乐,文艺
教育就业,健康
时尚,旅游,美食,IT 数码,娱乐
娱乐
娱乐,投资理财,新闻资讯,汽车,IT 数码
名人明星,娱乐
汽车,时尚,文艺,体育,娱乐
星座命理,旅游,穿衣美容,视频音乐,美食,听歌,90 后,电影,音乐
旅游
旅游
音樂
娱乐

新闻资讯,娱乐,旅游,文艺,时尚
军事,美食,美女,汽车,娱乐
时尚,星座命理,美女,美食,欧美音乐
娱乐,健康,时尚,音樂
健康,美食,汽车,旅游
时尚
美图摄影,体育
娱乐
美食,旅游
兵长,cosplay,bjd,二次元
旅游
投资理财,旅游,汽车
IT 数码
汽车,健康
投资理财,IT 数码
娱乐,游戏动漫
健康,时尚,旅游,文艺,娱乐
体育,新闻资讯,时尚,投资理财,旅游
宅女,交友,投资理财,健康,美食,娱乐
美女,执着,银魂,个性
美食,军事,IT 数码,文艺,娱乐
穿衣美容,汽车,美食,娱乐,IT 数码,时尚
视频音乐,我就是我,学生,爱音乐,看电影
时尚
旅游,动漫迷,时尚达人,音樂,化妆
教育就业,投资理财,美食,新闻资讯,时尚,娱乐
太极拳,武术,巴西柔术,综合格斗（MMA,泰拳,散打
星座命理,IT 数码,设计,新闻资讯
汽车,美食,军事
家具定制,动画,旅游,文艺,游戏动漫
文艺,娱乐
学生一族
水瓶座,双重性格,感性,简单,爬山,坚强,吃货一枚,宅女,幽默,摄影
旅游,文艺,美食,投资理财
读书分享,美图摄影,星座运势,校园生活,视频音乐
微博奇葩,耽美腐宅女,回音,爷,广播剧控,cv
美女,许嵩,武艺,水瓶,美容护肤,shopping,蓝色,摄影控,独立,火影
新闻趣事,新闻资讯,教育就业
穿衣美容,视频音乐,美食,娱乐
体育,娱乐
搞笑幽默,健康,娱乐
体育,汽车
娱乐,军事
天蝎女,独立,杂志,吃货一枚,旅游摄影,懒,唱歌,上网,睡觉,宅

教育就业
IT 数码
美食
投资理财, 军事, 新闻资讯
星座运势
体育, 美食
新闻资讯
睡觉, 自由, 唱歌, 宅, 服装, 健康, 旅游, 时尚, 美女, 美食
时尚
IT 数码, 娱乐, 旅游, 星座命理, 美食, 文艺
星座命理, 娱乐, 投资理财, 时尚, 军事, 新闻资讯
星座命理, 娱乐
新闻资讯, 娱乐, 健康, 旅游
不吐槽会死星人, 专注于吃二十年, 空有一颗瘦子心, 梦想征服全世界, 第三只眼是相机, 心中有座永无岛, 文艺不靠谱青年
吃货, 游戏动漫
娱乐
星座命理
娱乐, 美食, 星座命理
健康, 游戏动漫, 旅游, 星座命理, 文艺
游戏动漫
星座命理, 时尚
教育就业
辣妈, 感悟, 心情, 爱情
投资理财, 教育就业, 美女, 健康, 星座命理
娱乐, 健康, 旅游, 美食
名人明星, 新闻趣事, 微博奇葩, 搞笑幽默, 喜欢听歌
娱乐, 时尚, 体育, 文艺
穿衣美容, 时尚
娱乐
游戏动漫
健康
医学, 羽毛球
时尚, 健康, 教育就业, 旅游, 美食, 军事
游戏动漫, 新闻资讯, 旅游, IT 数码, 体育
美食, 文艺, 健康
左旋肉碱, 减肥, 瘦身, 美女, 健康减肥, 安全减肥, 绿色减肥, 减肥药, 减肥产品, 减肥药排行榜
娱乐, 文艺, 美食, 旅游
美女, 体育, 健康, 投资理财, 军事
IT 数码, 汽车, 时尚, 健康, 投资理财
文艺
旅游, 文艺
新闻资讯, 美食, 时尚, 旅游, 健康
投资理财, 美食, 军事

缘,装饰画,工艺品,IT,健康,A 股,市场营销,淘宝,军事
美食,美女,时尚,文艺,娱乐,健康
体育
美女,体育
IT 数码,科技,跑跑卡丁车,电竞,外设,手机
教育就业,投资理财,游戏动漫,星座命理,IT 数码
美食,天秤座,电影,娱乐
美食
IT 数码
android
IT 数码
IT 数码,文艺
美女,军事,IT 数码,文艺,娱乐
IT 数码,汽车,时尚,游戏动漫,旅游
90 后但我不脑残,维他命,DOTA,乐天派,睡懒觉,音乐达人,时尚,美食,文艺,娱乐
新闻资讯,军事
第零句对白
文艺,时尚,IT 数码,娱乐
娱乐
时尚,健康,旅游,娱乐,美食
体育,美女,娱乐
美食,美食,o 型血
美女,健康
IT 数码,娱乐,体育
IT 数码,旅游,星座命理,美食,新闻资讯,游戏动漫,文艺,健康,体育,娱乐
VCC 舞蹈,街舞,机械舞,爵士舞,曳步舞,娱乐
娱乐
美食
K 歌,美食,体育
旅游,文艺,星座命理
娱乐
美女,时尚
体育
时尚,美女,IT 数码,娱乐
IT 数码
笑,冷笑话,购物狂,b 型血,拍照,爱玩,电影迷,健康,娱乐,星座命理
Music
汽車,遊戲動漫,新聞資訊,健康,旅遊,美食,體育
执着,法律,聊天,冷笑话,上海,爱玩
旅游,投资理财,美食,汽车
时尚,健康,美食,外地人在上海,上海雅荟,雅荟服装,蜂蜜摇滚,honeyrock,yalanka,雅兰卡
汽车,IT 数码,音乐,旅游,夜猫子,天蝎
坚强,摩羯,南京
健康

新闻资讯,时尚,汽车,娱乐,体育
军事,文艺,IT 数码
时尚,IT 数码,游戏动漫,美女,娱乐
汽车,旅游,军事,娱乐,体育
体育
娱乐,旅游
IT 数码,美食,娱乐,星座命理
娱乐
时尚,健康,美食,文艺,旅游,星座命理
美剧,爱情,偶喜欢玩,时尚,健康,美食,IT 数码,旅游,投资理财
80 後,文艺,投资理财,健康,时尚
IT 数码
游戏动漫,IT 数码,文艺,汽车
时尚,旅游,美食
娱乐,旅游,健康,时尚,美食,文艺
文艺,娱乐,投资理财,美食,旅游,健康,时尚
娱乐
娱乐
星座命理,投资理财,健康,时尚
任性,猫控,猫咪,执着,巨蟹座,独立,爱音乐,听音乐,发呆,文字控
爱睡觉,美食,教育就业,文艺,健康,新闻资讯
旅游,美食,文艺,娱乐,教育就业
体育
娱乐,时尚,旅游,文艺,IT 数码
美食,旅游
腦補一千萬,囉嗦一生推,Valvert,Newmann,天同怎麼這麼萌,清空這個彈匣,StarTrek,DoctorWho,叔控无鸭梨,環太平洋
旅游,体育,市场营销,创业,爱旅行,搞笑,善良,大学生,幸福,打篮球
90 后,网络
星座命理
文艺
娱乐,文艺,执着,射手座
教育就业,军事,汽车,IT 数码
视频音乐,星座命理,娱乐
健康,体育,娱乐
健康,体育,娱乐
钢琴,感性,简单,天蝎,宅女一枚,乐橙,听歌,电影,80 后,猫
娱乐
游戏动漫,体育
文艺,体育,娱乐
北京生活,新闻趣事,搞笑幽默,美食,美女,吃货,国际米兰
游戏动漫,旅游,IT 数码,汽车,体育
萌宠,时尚
旅游,老时光,旧香港,迷魂曲,冷情绪,美丽岛

偏执狂,我很傻也很温柔,90 後,善良,大学生,双子座
文艺,体育,健康
星座命理
旅游
投资理财,娱乐,旅游,体育,IT 数码
星座命理
娱乐,IT 数码,新闻资讯,健康,星座命理,美食,爱美食,幻想
文艺,美食
汽车,IT 数码
美食
瑜伽教练,健康,旅游,星座命理,美食,娱乐
汽车,IT 数码
美食
旅游,教育就业,美食
娱乐
星座命理,美食,时尚,教育就业,娱乐,文艺
旅游,健康,美女
读书分享,科学探索
星座命理,健康,旅游,投资理财,美女
IT 数码,游戏动漫
体育
教育就业,健康,旅游,娱乐,美食
新闻趣事,文艺
娱乐,美食,美女,游戏动漫
美食,教育就业
旅游
游戏动漫,美食,旅游,IT 数码
披肩,毛领,高级定制,皮草,时尚
美食,时尚,IT 数码,军事,旅游,健康
旅游,美食,星座命理,娱乐,文艺,游戏动漫
天秤,迷糊,学生一枚,笑话,猫控,快乐,健康,游戏动漫,美食,文艺
健康,投资理财,IT 数码
时尚,汽车
文艺,美食,娱乐,时尚
旅游,军事,文艺
娱乐
旅游
IT 数码,体育
星座命理,军事,IT 数码
健康,旅游,美食,文艺,娱乐
时尚,旅游,美食,星座命理,娱乐
吸血鬼日记,品牌策划,营销策划,广告人,汽车,旅游,IT 数码,体育,文艺
时尚,娱乐,服装,音乐爱好者
教育就业,星座命理,文艺

游戏动漫, 军事, 文艺
美女, 旅游, 体育
体育
文艺, 时尚, 新闻资讯, 旅游, 美食, 娱乐
体育, 汽车, 军事, 健康, 旅游
娱乐
IT 数码, 文艺
美女, 旅游, 汽车, 风行
爱旅游, 爱睡觉, 阳光, 林俊杰, 休闲
健康, 投资理财, 时尚
娱乐, 投资理财, 美女, 游戏动漫, 汽车, 大学
美食
体育, 文艺, 教育就业
旅游
双子
娱乐
IT 数码, 教育就业, 体育
投资理财, 旅游, 时尚
搞笑幽默, 时尚, 投资理财, 汽车, 健康
娱乐, 美食
穿衣美容, 娱乐
健康, 文艺
美食, 娱乐, 投资理财, 教育就业, 时尚, 健康, 新闻资讯
文艺, 娱乐, 旅游, 健康, 时尚, 新闻资讯, 投资理财
游戏动漫, 军事
健康, 旅游
健康
体育
睡觉睡到自然醒, 爱幻想, 唱 k, 偶喜欢玩, 旅游, 美食
星座命理, 旅游, 汽车, 时尚, 游戏动漫, 美女
武汉生活
星座命理
旅游, 美食, 美食, 学会善待自己
吉他演唱, 强迫症, 业余视频解说, 原创音乐
文艺
旅游, 健康, 投资理财, 时尚
汽车, 美女
文字, 爱自由, 孩子气, 陈翔, 自由职业, 语录, 80 后的尾巴, 喜欢音乐, 绘画, 设计师
娱乐, 星座命理
健康, 旅游, 星座命理, 美食, 娱乐
音乐, 旅游, 体育, 新闻, 读报, 交友
体育
星座命理, 游戏动漫
双重性格

爱美食,娱乐
娱乐
教育就业,汽车
校园生活
新闻资讯,体育
文艺
无聊,武术,好性格,音乐爱好者,冷笑话,电脑,聊天,游戏,善良,音樂
美食,游戏动漫,娱乐,军事,新闻资讯,体育
美食,旅游,美女
美女,汽车,时尚,娱乐,体育
游戏动漫,美女,娱乐,体育
旅游,美食
IT 数码
天蝎,高尔夫,文艺,体育
美食
美食
时尚,娱乐
健康,爱自由,赚钱,K 歌,执着,散步,旅游,听歌
新闻资讯,美女,文艺,体育,娱乐
搞笑幽默
微博奇葩
体育,NBA,球迷,水瓶,90 后非脑残,低调,疯子,打球
汽车
时尚,文艺,娱乐
星座命理,汽车
星座命理,健康
服饰
星座命理,旅游
时尚,汽车,娱乐,旅游
旅游
游戏动漫,健康
旅游,文艺
车世界,美图摄影,IT 数码,娱乐,汽车,旅游,休闲,街拍,散步
体育
教育就业,星座命理,军事
时尚,爱孩子,单身妈妈,爱学习,英伦控,旅行是种游离
游戏动漫,投资理财,体育
健康,新闻资讯,汽车
美女,体育
健康,时尚,投资理财
文艺,体育
娱乐,情绪化,爱吃,淡定,内向,敏感,安静
穿衣美容,美女
搞笑幽默,IT 数码

健康,天平女,90後,跳舞,孩子气,电视剧,爱生活,爱旅游
娱乐
新闻资讯
情感生活,游戏动漫
体育
IT 数码,时尚,美食,旅游,娱乐
美食,健康,旅游,教育就业,娱乐
健康,新闻资讯,体育
教育就业,健康,旅游,娱乐,爱音乐,美食,听音乐,睡懒觉,好性格
时尚,服装网店,服装搭配,服装批发,吃货,搞笑,IT 数码,淘宝控,销售,80 后的尾巴
旅游
看书,医学,正能量,爱美食,猫咪控,音乐控,水瓶座
投资理财,美食,健康,娱乐
听音乐,自由职业,财经
娱乐
娱乐,星座命理
汽车,教育就业,体育,美女,IT 数码,军事,娱乐
热爱生活
娱乐,旅游,新闻资讯,IT 数码
摄影,旅游,文艺,时尚
投资理财,创业
体育
娱樂
自由,动漫,死神,萝莉控,游戏动漫
体育
睡懒觉
教育就业,美女,军事,汽车
美食,星座命理,文艺,娱乐,旅游
军事,美女,旅游,文艺,体育
娱乐
欢乐,音乐
旅游
微博奇葩,穿衣美容,街舞,健康,旅游,时尚,娱乐
时尚,汽车,旅游,美食,新闻资讯
健康,IT 数码
健康,旅游,美食,娱乐,IT 数码
旅游,时尚,健康,星座命理
体育,皇马,努力,梦想,娱乐,双子,交朋友,西班牙,欧美控,足球迷
游戏动漫,美食
美女,IT 数码
美食,文艺,星座命理,健康,娱乐
体育,美女,美食,军事,旅游
游戏动漫,旅游,军事,IT 数码
娱乐,时尚,星座命理,游戏动漫,美女,IT 数码

文艺,AOA 粉丝团,AOA
旅游,文艺,娱乐,星座命理
旅游,新闻资讯,美女,时尚,娱乐
健康,文艺,星座命理,美食,旅游
美食
美女,游戏动漫,娱乐,旅游,美食
数码科技,农业科技
健康,旅游,美食,娱乐
文艺
90 后非脑残,热情,猫控,星座命理,新闻资讯,娱乐
旅游,星座命理,新闻资讯
美食,美女,美食,教育就业,汽车,投资理财,娱乐
体育
娱乐
宠物,善良,吃喝玩乐,摩羯女
旅游
汽车,游戏动漫,旅游,美食,娱乐
时尚,美女,星座命理,星座占卜,好闺蜜好基友,多重性格,人格分裂,双性恋,文艺
旅遊,汽車,IT 數碼
时尚,旅游,文艺
闷骚!宅!唱歌!
命运石之门,DarinZanyar,文艺,旅游,军事,IT 数码
军事,汽车,游戏动漫,IT 数码,体育
体育
IT 数码,旅游,汽车,娱乐,军事
IT 数码,时尚,美女
旅游,美食,天枰座,我就是我,90 后,爱自由
娱乐
星座命理,美食,美食
娱乐
65,高二(4)班,高一 17,六十五,雙魚女,爱说幼儿鸡,晨迷
星座运势,文艺,教育就业,新闻资讯,美食,时尚
美女,吃喝玩乐
以马内利,哈利路亚
星座命理,娱乐,古典音乐,时尚,游戏动漫,健康,美食,旅游
名人明星,娱乐
IT 数码,广告公司,室内装修,广告装饰,广告设计,广告制作
汽车,美食,IC 创业,IC,初创公司,二人转,歪诗,国学,Analog
时尚
美女,体育
新闻资讯,美食,娱乐,体育
旅游,穿衣美容,时尚
旅游,喜欢听歌,浪漫,乐天派,购物
军事,汽车

美女,新闻资讯,健康
IT 数码
美食
游戏动漫
健康,时尚,旅游
旅游,美食,文艺
时尚,旅游,健康,新闻资讯,文艺
娱乐
游戏动漫,星座命理
星座命理,旅游
游戏动漫
听音乐,广告,淘宝,媒体,古灵精怪,美食,美女,健康,娱乐,IT 数码
爱笑,水瓶座,校园,完美主义,韩剧,音乐爱好者,健康,时尚,旅游,星座命理
美食,汽车,健康,旅游,美女,时尚
旅游
时尚,娱乐
时尚,娱乐
娱乐
星座命理
旅游
时尚,汽车,游戏动漫,投资理财,娱乐,IT 数码
娱乐,美食,旅游,自拍,臭美
美女,elf
娱乐
时尚,健康,旅游,娱乐,美食
游戏动漫,军事,美女,星座命理,IT 数码
娱乐,文艺,教育就业,汽车,旅游
美食,体育,IT 数码
健康,旅游,文艺
遊戲動漫,IT 數碼,軍事,健康,美女
文艺
健康,时尚,星座命理,美食,娱乐
娱乐,美食,个性
星座命理,IT 数码,投资理财,军事
旅游,萌宠,微博奇葩,搞笑幽默,视频音乐
美食,健康,星座命理,时尚,旅游
娱乐
美食
旅游,新闻资讯
新闻资讯,娱乐,体育,投资理财
数学,英国,Statistics
军事,体育,娱乐
娱乐
广东生活,时尚

美食,健康的孩子,求子,促排,多囊
学生一枚,D8,詹蜜,体育
娱乐
游戏动漫,旅游,星座命理
星座命理,旅游,文艺,IT 数码,娱乐
tvb,军事,汽车,時尚,汽車,娛樂,IT 數碼,軍事,美女,娱乐
军事,文艺,体育,旅游
投资理财,等待 ING,奋斗 ing,发呆 ing,天蝎座,80 後,胡思乱想
视频音乐,旅游,萌宠,搞笑幽默,LOL,90,美食,IT 数码,文艺,娱乐
健康
微博奇葩,文艺,吃货,90 后,爱懒觉爱赖床,各种宅
名人明星,星座命理,视频音乐
时尚,游戏动漫
体育
时尚
旅游,美食,健康,文艺,星座命理,美食,娱乐
时尚,文艺,娱乐
游戏动漫,时尚,IT 数码,健康,体育,娱乐
台湾生活
重庆生活,教育就业,健康,IT 数码,投资理财,汽车
新闻资讯
处女座,狗狗,游戏动漫,IT 数码,娱乐
美女,健康,时尚
汽车,IT 数码,军事,时尚,娱乐,体育
健康,时尚,美食,娱乐
投资理财,时尚,美女,健康,旅游,IT 数码,娱乐
美食,时尚,娱乐,游戏动漫
旅游,时尚
新闻资讯,健康,文艺,体育,娱乐
IT 数码
娱乐,IT 数码,文艺,体育
旅游
时尚,星座命理,美食,娱乐,投资理财
体育
娱乐,IT 数码,体育
新闻资讯,IT 数码,军事
娛樂
IT 数码,健康,美食,旅游
上海生活,体育
教育就业
文艺,新闻资讯,旅游,美食,体育
星座命理,美食,健康,文艺,娱乐
健康
旅游,体育,娱乐

美图摄影,文艺
教育就业,时尚,美食,文艺,娱乐
时尚,旅游,美食,娱乐
旅游,文艺,时尚,教育就业,投资理财,健康,美食,娱乐
名人明星,娛樂
健康,时尚,文艺,美食,IT 数码
思考,时尚
意大利,吃货一枚,旅游美食爱好者,摄影控,电影迷,AC 米兰
IT 数码,娱乐,文艺,美食,旅游
体育
SuperJunior,重口味,文字控,音樂,狮子女,LUHAN,EXO,行星饭,爱鹿晗,神经大条
健康
时尚,文艺,娱乐,星座命理,旅游
娱乐,美食,军事
美食
旅游,体育,旅游爱好者,吃,运动,爱幻想,奋斗
美女,游戏动漫,汽车,IT 数码,娱乐
娱乐
汽车,新闻资讯,美女,投资理财,军事,星座命理,文艺,IT 数码
时尚,娱乐
美女
旅游,美食,IT 数码,文艺,娱乐
懒,矛盾纠结体,强迫症,爱睡觉,听音乐
美食
娱乐
旅游,星座命理,娱乐
体育
自然,爱音乐,军迷!
健康,汽车
时尚
美食,动漫,游戏
健康,娱乐,星座命理,军事,新闻资讯
游戏动漫
游戏动漫
90 后一追求淡定,大学,吃货一枚,阅读,羽毛球,奋斗,音樂,美食,健康,娱乐
文艺,星座命理,美食
房产
旅游,娱乐,教育就业,健康,星座命理
穿衣美容,活在当下。,娱乐
星座命理,美食,汽车
文艺,IT 数码,旅游,星座命理,美食
时尚,文艺
娱乐,教育就业,时尚,美食,星座命理
美女,IT 数码

娱乐
美食,游戏动漫,旅游,文艺,娱乐
IT 数码
娱乐,体育,美女
IT 数码,美食,军事,体育,游戏动漫,娱乐
星座命理
军事
旅游,穿衣美容,广东生活
旅游,星座命理,穿衣美容,网购,游戏动漫,影视
文艺,美食,娱乐,军事,IT 数码
IT 数码
投资理财,时尚,健康,旅游,美食
体育
教育就业,美食,影视,旅游爱好者,健康,汽车,体育,旅游,娱乐
传统文化,剑网 3,美食,星座命理,文艺
IT 数码,娱乐,投资理财,美女,体育
动漫,游戏动漫,娱乐,美女,旅游,体育
黑冰爆珠,华软,万宝路,港标黑冰,万宝路黑冰爆珠
娱乐,聊天,喜欢音乐
教育就业,时尚,新闻资讯,星座命理,娱乐
旅游,时尚
投资理财,时尚,健康,旅游,军事
美女,旅游
时尚,美食,旅游,娱乐
军事,文艺,体育,美食,IT 数码,时尚
IT 数码,教育就业,体育,游戏动漫
星座命理,美食,时尚
美女,健康,旅游,美食,娱乐,时尚
游戏动漫
旅游,美女,文艺,娱乐,星座命理
汽车,美食,军事,星座命理,文艺,睡觉睡到自然醒
IT 数码,军事,游戏动漫
旅游,娱乐,美食,时尚
美食,健康,旅游
新闻趣事,萌宠,微博奇葩,旅游,教育就业,新闻资讯,军事
体育,健康,投资理财,游戏动漫,新闻资讯
时尚,汽车,娱乐,文艺,IT 数码
旅游,军事,文艺,体育,汽车
旅游
教育就业,投资理财,健康,文艺,娱乐
孩子气,宅女,我就是我,看小说,爱幻想,紫色,懒,安静,快乐,健身
旅游,投资理财,IT 数码,汽车,娱乐
娱乐,军事
军事,IT 数码

微博奇葩,健康
美女
星座命理,双鱼,睡懒觉,五月天,双重性格,慢热
娱乐,星座命理
健康,教育就业,旅游,文艺,娱乐
娱乐
旅游,吃货,爱唱歌,喜欢听歌,减肥
文艺,旅游,美女,时尚,IT 数码
娱乐
文艺,健康,旅游
娱乐,文艺
新闻趣事,八卦杂谈,视频音乐,搞笑幽默,健康,星座命理,娱乐,文艺
旅游,微博奇葩,新闻趣事,江苏生活,搞笑幽默,汽车
时尚,旅游
乐观,美食,建筑,法律,书法,室内设计,心情,资讯,新闻资讯,娱乐
汽车,文艺,娱乐
动漫,随性,宅,吃货,起床困难户,色狼
娱乐,美食,时尚
娱乐
体育,军事,新闻资讯,娱乐
旅游,美食
旅游
萌宠,美食,文艺,偶稀饭睡觉觉
视频音乐
时尚
汽车,健康,旅游,美食
旅游,健康,体育,时尚,星座命理,娱乐
IT 数码,娱乐,健康,新闻资讯,教育就业
美女,军事,体育,旅游,iphone
美食,旅游,文艺
山西球迷,山西兴瑞,山西中宇
体育,交友,爱旅游,单身,玩儿,坚强,旅游,健康
IT 数码,美女,娱乐,旅游,汽车
文艺
时尚
旅游,娱乐,美食,健康,时尚
美食,仙剑,设计控,山东,绘画,书法,平面设计,89 年,巨蟹座,室内设计
星座命理,旅游
美女,文艺,体育
美食,健康,旅游,娱乐,文艺
投资理财,教育就业,健康,体育
IT 数码,健康,美女,旅游,游戏动漫,星座命理
美食
新闻资讯,游戏动漫,美女,IT 数码,军事

台球,IT,Music,情绪化,射手,钓鱼,喜欢旅游,双重性格,搞笑,游戏
旅游,电影,摄影,生活,白羊座,阅读,爱跑步,爱旅行
IT 数码
旅游,星座命理,游戏动漫,文艺,娱乐
韩国,简单,宝宝,品牌,陈翔,快乐大本营,性感,厦门,恐怖片,娱乐
旅游
美女,宅女加腐女,自拍,90 后但我不脑残,宅女一枚,睡觉,摩羯座,上海,les,插画
笑,自然,爱旅行,爱吃,美容护肤,shopping,矛盾体,我就是我,爱睡觉
新闻趣事,搞笑幽默,军事,新闻资讯,投资理财,IT 数码
看电影,帅哥,逛街,开朗,星座命理,美食,美女,游戏动漫,娱乐
文艺
新闻趣事,搞笑幽默,微博奇葩,美食,游戏动漫,娱乐
投资理财,新闻资讯,体育,娱乐
美食,时尚,文艺,娱乐,教育就业,新闻资讯
教育就业
新闻资讯,愤青过去式,三十没立,单板,投资理财
汽车,游戏动漫
健康
体育,星座命理,健康,旅游,美食,IT 数码
游戏动漫
体育
美食,健康,旅游
健康
新闻资讯,美食,汽车,旅游,星座命理,健康
旅游,投资理财,体育,时尚,汽车,娱乐
新闻资讯,军事,体育,娱乐
娱乐
体育
美食,文艺
文艺
星座命理,美食,健康,教育就业
旅游,星座命理,美食,娱乐
名人明星,旅游,时尚,游戏动漫,美食,文艺
IT 数码,文艺,心情,健康,新闻资讯
视频音乐,萌宠,旅遊,名人明星,穿衣美容,双子女,踢踏舞,音樂,舞蹈,娛樂
美食
文艺
教育就业,新闻资讯,游戏动漫,军事,文艺,体育
美食
文艺
新闻资讯
体育
搞笑幽默,体育
文艺

体育
新闻资讯,投资理财,军事,美女,娱乐,汽车,旅游
天蝎,游戏动漫,旅游,体育
IT 数码,时尚,美女,美食,娱乐,文艺
美食
新闻资讯
旅游,美食,娱乐
美食,文艺,旅游
教育就业,投资理财,娱乐
陕西生活,体育
广西生活,文艺,投资理财,时尚,健康
加拿大生活,美食,汽车,健康,旅游
美食,娱乐
娱乐,文艺
校园生活
云南生活,微博奇葩,旅游,新闻资讯,娱乐,星座命理,美食
美女
娱乐
旅游
体育,星座命理,美食,旅游,健康
体育
健康,旅游
健康,小资,育儿,潮妈,亲子,80 后,美食,文艺,时尚
美食
汽车,教育就业,文艺
旅游,健康,新闻资讯,娱乐,军事,体育
爱美剧,动漫迷,爱电影,爱旅游,自由职业
娱乐
新闻资讯
美食
娱乐,美食
健康
投资理财
尔等凡夫俗子,吾乃,出淤泥而不染,濯清涟而不妖,纯白无暇,清香芬芳,好一朵,纯洁的,大雕
美食,娱乐,健康
美女,体育,时尚,旅游,健康
汽车
投资理财,汽车,美食,军事,IT 数码
美女,健康,美食,投资理财,IT 数码
广东生活
文艺,旅游,美女
娱乐,美食,汽车,时尚,体育
娱乐,投资理财,新闻资讯,美食,IT 数码
美女

娱乐,星座命理
巨蟹座 O 型血,男,大学生,偶稀饭睡觉觉,90 后但我不脑残,偏执狂,看电视,喜欢旅游,双重性格,销售
美食
旅游,美食,IT 数码,娱乐,射手座,文字,无厘头
时尚,文艺
美女,时尚
穿衣美容,新闻资讯
娱乐
娱乐
新闻资讯
美食
健康,体育
娱乐,时尚
看电影,爱自由,射手,美食爱好者,喜欢听歌,纠结,音乐迷,娱乐
美女,旅游,健康,时尚,娱乐
听歌,巧克力,笑,聊天,射手座,健康,开心,喜欢音乐,娱乐
天蝎座,闷骚,林峰,咖啡控,安静,爱笑,深圳,开心,喜欢音乐,奋斗
旅游,健康,汽车,文艺
美女
星座命理,教育就业,文艺,体育,娱乐,旅游
娱乐
中泰翻译,家教,请佛牌,泰国正品代购,泰国导游,热情,K 歌,旅游,文艺
搞笑幽默,新闻趣事,旅游,微博奇葩
健康,美食,娱乐,IT 数码
美女,娱乐,汽车,军事,体育
体育
投资理财,文艺
教育就业,时尚,投资理财,健康,旅游
星座命理,军事,美食,旅游,健康
健康,教育就业,时尚,旅游,娱乐
教育就业,娱乐,文艺,旅游,新闻资讯
星座命理
文艺,美食,旅游
穿衣美容,时尚
时尚,美食,健康,娱乐,旅游
视频音乐
娱乐
文艺,投资理财,新闻资讯,旅游,时尚,家装画相框相册
IT 数码
健康,新闻资讯,旅游,美食,娱乐
美食
娱乐
旅游

IT 数码,美女
时尚,旅游,美食,娱乐
汽车,美食,IT 数码,军事,旅游,投资理财,娱乐,星座命理,文艺,体育
新闻资讯
英国生活,时尚,美食,星座命理,旅游
文艺,教育就业,旅游,娱乐,IT 数码
学生,拍照,粉红控
时尚,娱乐,健康,投资理财
穿衣美容
游戏动漫,星座命理,IT 数码,美女,时尚,BIGBANG
教育就业,投资理财
男生宿舍
旅游,美食
健康,时尚,旅游,娱乐
读书分享,科学探索,视频音乐,泉州生活,旅游
新闻趣事,校园生活,搞笑幽默,名人明星
健康,文艺,旅游
时尚,星座命理,美食,旅游,娱乐
旅游,美食,健康,舍,乐观,沉默,孤独且享受的,低调,90 后
旅游
美食
娱乐
美食,军事,体育,宅,90 后,连接服务器失败,学生一族,孩子气,nba
广东生活
旅游,健康,IT 数码,美食,体育,嗯………
胡思乱想,神经质,发呆,听音乐,书,水瓶座,情绪化
体育,美食
美图摄影,名人明星,新闻趣事,视频音乐,新闻资讯,星座命理,健康,时尚,娱乐
星座命理,美食,旅游
体育
时尚,健康,文艺
体育
IT 数码
美女,星座命理,旅游,美食
时尚
新闻资讯
美图摄影,旅游,名人明星,校园生活
新闻资讯,军事,娱乐,文艺,时尚,健康,投资理财,旅游
新闻资讯,健康,美食,文艺
旅游美食爱好者,摩羯座,感性,完美主义,爱幻想,阳光,喜欢音乐,逛街,矛盾体,健康
美女,新闻资讯,旅游,娱乐,体育
文艺,星座命理,旅游,汽车
寂寞,活泼开朗,美食控,登山
旅游,健康,时尚,美食,文艺

时尚,健康,星座命理,文艺,娱乐
时尚,健康,美食,文艺,旅游
时尚,本人全职是妈妈,在线时间不固定
美食,旅游,时尚,娱乐
美食,文艺
时尚,游戏动漫,IT 数码,文艺
军事,IT 数码,皮具之家
汽车,新闻资讯
美食,娱乐
美食,健康,新闻资讯
美食,旅游
星座运势
时尚,新闻资讯,IT 数码,文艺,娱乐,教育就业
文艺,娱乐,教育就业,旅游,美食
狗狗
养颜,护肤,抗疲劳,补血,养生堂,星座命理,美食,健康,时尚,美女
体育,美食,旅游,时尚,投资理财
娱乐
美女
美女,健康,IT 数码,娱乐,体育
健康
体育,娱乐,游戏动漫,星座命理,IT 数码
时尚,文艺,美食,游戏动漫,娱乐,体育
健康,旅游,时尚,摄影控,瘦身,美容护肤
佛山生活,体育,汽车
微博奇葩,投资理财
游戏动漫
其他生活,文艺
旅游,美食,美女,时尚
娱乐,IT 数码
星座命理,美女,时尚,娱乐
旅游,星座命理,时尚,动漫控,金牛座,宅男,孩子气,高中生,90 後,学生一枚
美食,文艺
宅女,天蝎女,沉默,臭美,90 後
搞笑幽默,名人明星
美食
体育
娱乐,时尚,美食
IT 数码,文艺,体育
娱乐
新闻资讯
睡觉睡到自然醒,胡思乱想,夜猫子,随性
美食,旅游
IT 数码,搞笑幽默

视频音乐,美食,旅游,电影,音乐,摩羯座
看小说,爱幻想,学生
新闻资讯,汽车,投资理财,旅游,体育
美女,汽车,投资理财,军事,IT 数码
美女,汽车,时尚,军事,娱乐
投资理财,教育就业,新闻资讯,美女,健康,旅游,体育
旅游
美女,游戏动漫,体育
娱乐
美图摄影,体育资讯,名人明星,新闻趣事,其他生活
销售,淘宝控,投资理财,健康,美食
投资理财
搞笑幽默
健康,时尚,汽车,教育就业,投资理财
投资理财,IT 数码
睡觉
旅游,星座命理
文艺,IT 数码,美女,时尚,汽车,游戏动漫
教育就业
IT 数码,游戏动漫,时尚,文艺,体育,旅游
视频音乐,时装,内向,喜欢听歌,时尚
体育,旅游,汽车,音乐迷,厦门,娱乐,新闻资讯,IT 数码
体育,文艺
美食,时尚,健康,旅游
娱乐
旅游,新闻资讯,健康,美食
90 後
时尚,旅游,健康,新闻资讯,美女
时尚,健康,旅游,美食,体育
搞笑幽默
文艺
美食,淡淡得执着
教育就业
微博奇葩,汽车
微博奇葩,穿衣美容,星座命理,美女,搞笑幽默
IT 数码,教育就业,时尚,娱乐,美食,健康
游戏动漫,投资理财,汽车,文艺,旅游
美食,穿衣美容,时尚,文艺,星座命理
文艺,旅游,健康,美食,时尚
体育,军事
美女,IT 数码,体育,游戏动漫
体育,汽车
粤语,怀旧,影视,历史,神经质,读书
时尚,星座命理,美食,娱乐

旅游
娱乐
时尚,IT 互联网,新闻资讯
IT 数码,美食,娱乐,文艺,体育
新闻资讯,IT 数码
美食
健康
穿衣美容
健康,文艺,IT 数码
军事
旅游,美食,娱乐
星座命理
健康,旅游,教育就业,美食
体育,旅游,听歌,电影,快乐大本营,男人,单纯
旅游,时尚,美食
汽车,游戏动漫,旅游,军事,IT 数码
星座命理,新闻趣事,萌宠,微博奇葩,搞笑幽默,文艺,汽车,娱乐
健康,投资理财,时尚,体育,旅游
开朗,休闲,偶像剧,校园,自信,快乐大本营,O 型血,90,电视,水瓶座
时尚,健康,旅游,美食,娱乐
星座命理,健康,时尚,娱乐
IT 数码,时尚,星座命理,美食,娱乐
娱乐,旅游
娱乐
旅游
美食,宅女一枚,新闻资讯,健康,旅游,文艺,时尚
旅游,健康
时尚
罗志祥百度,罗志祥微吧,罗志祥,珊子好喜欢祥子
美女,文艺,娱乐,体育,投资理财,军事,游戏动漫
HMEI 汇美,溶脂贴,蕾珂美,番茄派,凯蜜拉,普兰氏,猪鼻贴,巴诺巴奇
娱乐
娱乐,文艺,健康,旅游,时尚,教育就业
健康,新闻资讯,时尚,教育就业,美食
美食,IT 数码,时尚,旅游,投资理财
美女,音樂
文艺,美食
汽车,军事,教育就业,投资理财,旅游,健康,新闻资讯,娱乐
星座命理
音乐分享,星座命理,文艺
星座命理,美食,健康,旅游,新闻资讯,投资理财,娱乐
美食,教育就业,新闻资讯,时尚,游戏动漫,旅游,美食,文艺
时尚,美食,旅游
娱乐

文艺,军事,IT 数码,k 歌
美食,健康
名人明星,娱樂
无锡生活,体育资讯,视频音乐,美女,搞笑幽默
娱乐,爱吃爱睡,宠物控,宠物达人,宠物爱好者,韩剧
新闻资讯
文艺
汽车,旅游,文艺,体育
IT 数码
90 后非脑残,随性,向日葵,偏执狂,教育就业,时尚,健康,旅游,美食
体育,文艺,游戏动漫
美女
健康,旅游
IT 数码
健康,时尚,美食,旅游,娱乐
娱乐,旅游,美食,星座命理
娱乐,体育,文艺,新闻资讯,游戏动漫
IT 数码,新闻资讯,时尚,投资理财,娱乐,旅游
文艺,时尚,投资理财,美食
时尚,娱乐,文艺,旅游,散步,旅游爱好者,90 后非脑残,厦门,喜欢听歌
军事,旅游
旅游
上海生活,体育,疯狂,文艺,爱笑,90 後,投资理财
文艺,健康,投资理财,旅游,美食
听歌,90 后,视频音乐,体育,旅游,投资理财,美食,娱乐,文艺,健康
美食,娱乐,时尚
宜昌生活,IT 数码
视频音乐,旅游,美食,萌宠,名人明星
健康,文艺,旅游,星座命理,美食
教育就业,旅游,游戏动漫,娱乐,美女
美食,健康,旅游,文艺
娱乐
星座命理,旅游,健康,娱乐,新闻资讯
汽车
美食
IT 数码
美女,IT 数码
旅游,时尚
新闻趣事,旅游,美食,视频音乐,渭南生活
星座命理
文艺,新闻资讯,时尚,星座命理,娱乐
旅游
美女,娱乐
汽车,文艺,IT 数码

时尚,美食,文艺,娱乐,IT 数码
阳光,逛街,看小说,敏感
音乐教室,音响租赁,乐器租赁,游戏音乐,影视音乐,文化传媒,摇滚,俱乐部,排练房,乐队
军事,旅游,美食,文艺,娱乐
疯子,偶喜欢玩,腐女,学生一族,淘宝
自由,快乐,书籍,欧美控,娱乐,美食,旅游
时尚,爱美食,睡懒觉
美女,娱乐
产品摄影,婚礼跟拍,摄影,美食,文艺,时尚
美食
旅游,美食,游戏动漫,文艺
星座命理,旅游,教育就业,新闻资讯,文艺
同志,蓝色,星座命理
娱乐,微笑
体育,娱乐,文艺
丽水生活,文艺,教育就业
文艺
美女,游戏动漫
健康,新闻资讯,军事
投资理财
动画,瘦身,美食,动漫控,感性,简单,乐观,吃货,健康,亲情
独立,喜欢音乐
娱乐,旅游
健康,旅游,游戏动漫
看电视,沉默,安静,体育,科比
美食
军事
旅游,娱乐,美食,游戏动漫,星座命理,健康
游戏动漫,WOW,DOTA,美食,星座命理,娱乐
穿衣美容,娱乐
新闻资讯,旅游,IT 数码
健康,时尚,星座命理,美食,娱乐
新闻资讯,娱乐,旅游,星座命理,时尚,美食,双鱼,笑,吃货一枚
投资理财
古董收藏,明清家具,木质文化,传统文化
健康,摄影,干有创意的活,旅游
星座命理,投资理财
穿衣美容,教育就业,文艺
星座命理
旅游,健康,美女,军事,体育
美食
文艺,健康,汽车,旅游,美食
美食
旅游

影城
娱乐
健康,文艺
新闻资讯,健康
娱乐,潮,睡觉睡到自然醒,忧郁,shopping,不離不棄
游戏动漫,星座命理,美食,娱乐
吃货,爱,小清新,健康,旅游,星座命理,美食,娱乐
新闻资讯
投资理财,文艺,健康,旅游,星座命理,美食
努力,处女座,医学,影视,爱吃,幸福,汽车,健康,美食,文艺
旅游,汽车,时尚,健康,美女
旅游,娱乐,汽车,美食,文艺
投资理财,健康,美食,旅游,娱乐
IT 数码
美容,娱乐,淘宝控,睡觉睡到自然醒,喜欢旅游,音樂,健康
华邦集团,伊赛特家居汇,营销,家具营销,建材营销人
美食
娱乐,美食,IT 数码,健康,旅游,时尚,投资理财
投资理财,娱乐,美食,星座命理
美女,寂寞
健康,文艺,时尚,旅游,星座命理,新闻资讯,投资理财
旅游,时尚,投资理财,娱乐
军事,天蝎,房地产,喜欢唱歌,宅,80 后
投资理财,旅游,IT 数码,体育
IT 数码
体育,旅游,时尚,文艺,IT 数码
新闻资讯,旅游,美食,IT 数码,投资理财,健康
自由
时尚,健康,新闻资讯,旅游,军事,美食
旅游
美食,健康,IT 数码,旅游,星座命理,文艺
热爱生活,幽默,平常心,偶稀饭睡觉觉,学生一枚,神经质,低调,爱电影,会计,宅女一枚
健康
时尚,娱乐,健康,狮子座,日剧,旅游,IT 数码
健康,娱乐,新闻资讯,星座命理,旅游,投资理财,体育
旅游
美食
健康,星座命理,旅游,教育就业
汽车
狗狗,时尚潮流,网络,杂志,吃货一枚,暮光之城
汽车
吃货,辟谣联盟,自干五,C 青年脑残粉
美女,游戏动漫,体育
教育就业,健康,旅游,军事,IT 数码,爱,独立,射手座,新闻资讯

旅游,健康,星座命理,校园,喜欢音乐,交朋友
旅游,健康,时尚
健康,时尚,娱乐,美食
旅游,投资理财,星座命理,汽车
旅游
文艺,健康,旅游,美食,IT 数码
旅游,时尚,美食,娱乐
娱乐
美女,时尚,汽车,星座命理
投资理财,旅游,娱乐,IT 数码,新闻资讯
娱乐
购物,美容,服饰,潮流,折扣,优惠,健康,时尚,旅游
娱乐
搞笑幽默,吉他手,词曲作家,乐队主唱
DO,krisyeol
健康,旅游,星座命理,文艺
娱乐,美食,时尚,旅游
健康,美食
美食,体育
书,快乐,睡懒觉,常州生活,美食
音乐控,张根硕
健康,文艺,新闻资讯,时尚,星座命理,投资理财
美女,新闻资讯,IT 数码
文艺,娱乐,健康,星座命理,旅游
旅游
文艺,星座命理,娱乐,新闻资讯,游戏动漫,汽车
美食,时尚,新闻资讯,旅游,健康
文艺
IT 数码,旅游,健康,娱乐,时尚
统一通信,全球领航,IT 数码
旅游,星座命理,文艺,时尚,美女,健康,美食
育儿百科
唱 K,爱睡觉,爱笑,播音主持,艺术人生,天蝎座,小清新,小萌萌,完美主义,轻微外貌协会
美女,旅游,新闻,文学,沉默
体育资讯,美女,美食,游戏动漫,文艺,娱乐
娱乐,时尚,美女,星座命理,文艺
健康,旅游,星座命理,IT 数码,娱乐
萌宠,旅游,搞笑幽默,IT 数码
珠海生活,新闻趣事,旅游,健康,投资理财,新闻资讯
金牛,服装设计
投资理财,美食,新闻资讯,健康,旅游
健康,新闻资讯,投资理财,时尚,IT 数码
旅游,投资理财,健康,IT 数码,文艺,娱乐
武汉生活,新闻趣事,美食,校园生活,搞笑幽默

星座命理,旅游,娱乐,军事,文艺
娱乐
汽车,时尚,健康,旅游
娱乐
健康,星座命理,教育就业,时尚,娱乐,文艺
军事,汽车
搞笑幽默
游戏动漫
美食,时尚,星座命理,旅游,文艺
旅游,时尚,文艺
美食,投资理财,教育就业,星座命理,健康
文艺
娱乐,IT 数码
美食,金庸武侠,音乐,IT 互联网,体育
辽宁生活,美食,旅游,穿衣美容,美女
旅游
时尚,教育就业,健康,旅游,新闻资讯
教育就业,娱乐,健康,美食,投资理财
游戏动漫,旅游,IT 数码
心理,听音乐,潮人,爱电影,爱旅游
汽车,文艺,IT 数码,美食,体育,旅游,巨蟹女
娱乐,汽车,美女,健康,美食
体育
健康,时尚,投资理财,IT 数码,娱乐
健康,美食
游戏动漫
旅游
时尚,健康,旅游,美食,IT 数码,文艺
时尚,爱电影,周杰伦,汽车,球迷,坚强
时尚,汽车,星座命理,军事,娱乐
穿衣美容,娱乐
美女,军事,IT 数码,文艺,娱乐
科学探索
文艺
新闻资讯,旅游,时尚,体育,娱乐
汽车
旅游,星座命理,美食,娱乐
时尚,旅游
时尚,美女,汽车,旅游,娱乐
乐谱,吉他,摄影,开心,娱乐,看书,设计,紫色,艺术
美食,IT 数码,文艺,娱乐
美食,健康
美女,教育就业,体育,文艺,汽车
游戏动漫

美女,新闻资讯,军事,旅游,美食,IT 数码
健康,网络,发呆,星座命理,娱乐,旅游,时尚
体育
文艺,旅游,投资理财,美食,娱乐
旅游,其他生活
美食,旅游
健康,多肉植物,曹杨路地铁站,旅游,美食
健康,新闻资讯,娱乐
体育
重庆,hiphop
微博奇葩,搞笑幽默,重庆生活,旅游,星座命理,IT 数码,体育,娱乐
新闻资讯
新闻资讯,喜欢旅游,诗歌,开心
疯狂
健康,娱乐,旅游,新闻资讯
娱乐,时尚,新闻资讯
旅游,星座命理,军事,娱乐,美食,IT 数码
乐天派,高三文科,射手座
投资理财,军事
游戏动漫,旅游,娱乐,文艺
美图摄影
美女,军事,IT 数码,体育
旅游
GBOM,EXO,勋鹿,SEHUN,权志龙,朴春
文艺
搞笑幽默,健康,新闻资讯,文艺,投资理财
健康,军事,体育
娱乐,搞笑,小资,内涵,时尚
实事求是
娱乐
腹黑毒舌,霸气外漏美男子,Hiphop,Breaking,Locking,曲神,综合格斗,狂吃不胖桑脑筋,popping,各种运动达人
漳州生活,搞笑幽默,八卦杂谈,美容服饰,名人明星,娱乐
科学探索,BYOD,移动应用管理,MAM,MDM,移动设备管理,mobility,移动云计算,企业 APP,移动互联网
美食
游戏动漫
人力资源论坛,HR,背景调查,职业规划,HR 咨询,人才晋升,人才选拔,人才测评,人才管理,人力资源
古筝控,民族舞,养生,苏志燮,音乐控,狗狗,摄影控,双子座,拉布拉多,拉布拉多犬舍
游戏动漫,美食
娱乐
舞蹈,sj
视频音乐,搞笑幽默,中华时尚休闲鞋,新百伦,英格兰,军事,美女,旅游,娱乐,体育

坏数码,iPhone,三星,HTC,手机
K 歌,篮球，游戏
爱睡觉,偏执狂,孩子气,原创,睡觉睡到自然醒,沉默,看电视,喜欢旅游,天秤座,咖啡控
文艺,汽车,时尚,游戏动漫,投资理财,体育
星座命理,旅游,健康,汽车,专业主打,居家户外,旅游探险,LED 强光手电,头灯,自行车灯
娱乐,健康,宅女一枚,多愁善感,微笑,睡觉睡到自然醒,时尚,美食,星座命理,投资理财
时尚,旅游,娱乐
射手座,学生,篮球,爱旅游,低调
娱乐,美女,IT 数码
旅游
娱乐,旅游,健康,时尚,美食
时尚
新闻趣事
军事,射手座,大学生,跳舞,90 後,生活,旅游美食爱好者,休闲
IT 数码
美食
星座命理,美食,时尚,文艺
新闻资讯
旅游
圣诞宝宝,射手 vs 摩羯,甜食抵抗力低下,吃货一枚
北京生活,教育就业
视频音乐
健康
星座命理,美食
心理学,诗词,管理,网络,NBA,房地产,美食爱好者,军事,投资理财,美女
娱乐,时尚
娱乐
美食,健康,美女,投资理财,旅游
广西考试类,投资理财,教育就业,新闻资讯
AP 无解,这转转那逛逛,运动，,乐观自信，有爱,嗜睡，LOL 莎莎,爱睡觉
健康
游乐场,健康,娱乐,莆田
文艺,投资理财,健康,旅游,美食,军事
美女,体育
健康,海绵宝宝
IT 数码,汽车
体育,游戏动漫
星座命理,旅游,投资理财,健康,时尚,美食
娱乐,新闻资讯,教育就业,健康
体育
IT 数码
娱乐
金牛
旅游,投资理财,健康

军事,美食
军事,汽车,娱乐
体育
健康
热爱生活,喜欢听歌,时尚
星座命理,健康,时尚,娱乐,旅游,美食
军事,游戏动漫,旅游,娱乐,美女,体育
名人明星,视频音乐,新闻趣事,旅游,文艺,健康,美食,娱乐
美女
健康,军事,文艺,娱乐
军事,时尚
爱旅游,90 後,乐观,睡懒觉,电影控
军事,健康,旅游,旅游摄影,时事,投资理财,汽车,新闻资讯
健康,自拍控,吃货,二,有点小神经,小小胖纸,美食,娱乐
购物,美容,服饰,潮流,折扣,优惠,健康,时尚,投资理财
汽车,旅游
军事
IT 数码
微博奇葩,时尚
投资理财
美食,K 歌,处女座,美食,时尚,娱乐,健康,星座命理,旅游
视频音乐,旅游,投资理财
娱乐,低调,爱电影
寂寞,空虚
美女
旅游,美食
汽车
时尚
教育就业,外语
新闻资讯
体育,健康,时尚,美女,旅游,IT 数码,汽车,教育就业
美食
新闻资讯
旅游,星座命理,美食,时尚,健康
旅游,星座命理,IT 数码,娱乐,体育
娱乐
IT 数码,美食,体育
拼搏,创新,发展,实力
娱乐,健康,星座命理
时尚,投资理财,IT 数码,星座命理,旅游,美食
减肥,地产,DIY,养生,家居,护肤,旅游摄影,美食,淘宝,服装
体育,旅游
福州生活
科幻,诗词,美剧迷,乒乓球,爬山

投资理财,军事,体育
美食,星座命理
军事
星座命理,健康,时尚,文艺,娱乐
星座命理,娱乐
军事,IT 数码,体育
旅游,时尚,健康,美食,投资理财,IT 数码
娱乐,健康
混吃等死没追求,甜食,睡觉,纪录片,老女人和小男人
穿衣美容,微博奇葩
汽车
时尚,星座命理
军事,旅游,投资理财,体育
新闻资讯
时尚,美女,旅游,美食,军事
体育
健康,娱乐,旅游
旅游
娱乐
美食,健康
车世界,投资理财
文艺,体育
新闻趣事,美食,杭州生活,旅游,IT 数码,娱乐
画画,学生一族
美食,旅游,游戏动漫
旅游
美食,白羊
旅游,娱乐
哦都 K,90,娱乐,旅游
唱歌,摄影,幽默,上网,睡觉,听音乐,麦霸,旅游,IT 数码,娱乐
美食,旅游,星座命理,文艺,娱乐
微博奇葩,新闻趣事,辽宁生活,新闻资讯,军事
旅游,健康,星座命理,美食,文艺,娱乐
时尚,文艺,星座命理,美食,新闻资讯,教育就业
星座命理,美食
娱乐,投资理财,美女
健康,教育就业,体育,美食,投资理财
娱乐,美食,时尚
美女,娱乐,文艺,美食,投资理财
时尚,娱乐,新闻资讯,星座命理,健康
IT 数码,投资理财,星座命理,新闻资讯,娱乐
旅游,IT 数码,搞笑幽默,校园生活,美女,快乐至上,疯,学生
时尚,健康
健康,旅游

体育,时尚,美食,旅游
军事
时尚,体育,娱乐,新闻资讯,星座命理,IT 数码
吃喝玩乐,爱自由
时尚,娱乐,美食,文艺,小清新,东方神起,soulmate
脱下高跟,退了红尘,好姑娘永垂不朽
欧美音乐,美食,上网
重口味,小说,交友,幽默,爱生活,美食控,快乐,时尚达人,旅游美食,手工
文艺,旅游,教育就业,健康,投资理财,时尚,美食,娱乐
体育,健康,美食,汽车,旅游
文艺,IT 数码,体育
美女
时尚
偏执狂
道教,宅腐,建筑,翻唱,民俗,风水,插画
星座命理,美女,娱乐,体育,游戏动漫
护肤,美食爱好者,love
superjunior
美食,娱乐,时尚,游戏动漫,星座命理,体育
搞笑幽默,美女,新闻趣事,IT 数码,体育,娱乐
旅游
汽车
体育资讯,麻将,台球,高端休闲会所,德州扑克
汽车,IT 数码,文艺,体育,娱乐
新闻资讯
美食,健康
文艺,旅游,健康,美食,娱乐
学生,幽默,90 後,美食,新闻资讯,军事,健康,美女,IT 数码,投资理财
时尚,健康,旅游,美食
新闻资讯,健康,时尚,旅游,游戏动漫,娱乐,投资理财,美食
汽车,健康,投资理财
娱乐,旅游
星座命理,娱乐,时尚,美食
娱乐,美女
网购,文字,八卦,爱旅行,护肤,热爱生活,时尚达人,微笑,双子女,星座控
健康,娱乐,新闻资讯,旅游,美食
美食
娱乐,体育,时尚,星座命理
八卦,旅遊,手工,宝宝,资讯,新闻资讯,教育就业,旅游,美食,娱乐
健康,文艺
教育就业,投资理财,旅游,新闻资讯,娱乐
汽车,星座命理,新闻资讯,旅游,IT 数码
健康,汽车,投资理财,美食,文艺,新闻资讯,旅游,IT 数码
健康,星座命理,时尚,旅游,美女,教育就业,IT 数码

时尚,旅游,汽车,美食,文艺,游戏动漫,教育就业,IT 数码
时尚,IT 数码,美女,旅游,文艺
体育
旅游,健康,游戏动漫,汽车,体育
耽美,张根硕,浜尾京介,爱猫,旅游,美食
美食,体育
梅西,寂寞,爱幻想,看小说,自由职业,亲情
游戏动漫,体育,文艺,娱乐,IT 数码
时尚,军事,文艺
育儿百科,旅游
星座命理
体育,新闻资讯
自由,唱歌,睡觉,娱乐
星座命理,娱乐
旅游,娱乐
美食,文艺,体育,健康,旅游
健康,娱乐,旅游,IT 数码,游戏动漫,时尚,汽车,教育就业
文艺,军事,IT 数码,娱乐,美食
广东生活,娱乐,幸福,完美主义,韩剧,电影控
体育,娱乐,新闻资讯,军事
爱旅行,娱乐,投资理财,时尚,IT 数码,文艺
娱乐
美剧,阅读,好性格,读书,旅游,健康
教育就业,摄影控,时尚,健康,美食,旅游,美女,娱乐,文艺
健康,时尚,投资理财,娱乐
萌宠,美食,旅游,时尚,健康,娱乐
学生一族,学生一枚,90 後
游戏动漫,IT 数码
狮子座,新闻,网络,90 後,音樂,电影爱好者
Android
游戏动漫,文艺,睡觉睡到自然醒,爱音乐,情绪化,黑白控
旅游
星座命理
谨言慎行,喜欢音乐
星座命理
美食,旅游,健康,教育就业,娱乐,文艺
美女,游戏动漫,汽车
四川人在重庆,90 后,等爱者,简阳,医学生,权志龙,VIP,BIGBANG
娱乐
星座命理,娱乐,教育就业
娱乐,时尚,投资理财
健康,时尚,文艺,美食,旅游
星座命理
娱乐,游戏动漫

时尚,健康,新闻资讯,教育就业
投资理财
tvxq,宅女一枚,夜猫子,90后,星座,重口味,文字控,SuperJunior,F1,投资理财
投资理财,星座命理,美女
同志,笑话,体育
90後,枪械控,乐器控,初音控,萝莉控,女仆控,镜音控,动漫控,游戏动漫
时尚,天秤
美食,旅游,星座命理
体育
文艺,美食,星座命理,游戏动漫,时尚,娱乐
游戏动漫,体育,对不起我爱你
京剧,美剧日剧,涂鸦
时尚,文艺,健康,旅游,美食,娱乐,星座命理
旅游,时尚,美食,IT数码,娱乐
娱乐,时尚
旅游,IT数码,汽车,美女,娱乐
娱乐,体育
主持,平面设计,软件,时尚潮流,室内设计,哲学,美术,健康,教育就业,投资理财
体育
acg,80後,游戏动漫,文艺,IT数码
旅游,美食,健康,文艺
娱乐,星座命理,爱自由,吃喝玩乐,搞笑,吃,游戏,猫,天枰座
银魂,听音乐
微博奇葩,星座命理,其他生活,旅游,新闻趣事
旅游,军事,IT数码,文艺,体育,娱乐
体育,简单
体育
娱乐,文艺,投资理财,健康,军事
IT数码,时尚,文艺,娱乐,美食
娱乐,美女,IT数码,文艺,军事
美食,美女
文艺
旅游,汽车,投资理财
自我强迫,海盗,猫,靴子控,军备控,棋,甜食控,大叔控,旅游,健康
星座命理,健康
IT数码,娱乐,时尚,文艺
羽毛球
文艺,游戏动漫
娱乐,体育,军事
体育
投资理财,健康,游戏动漫,时尚,文艺
星座命理,文艺
时尚,旅游,美食,文艺,娱乐
健康

美食
新闻资讯,投资理财,文艺,教育就业,旅游
军事,投资理财,美女,新闻资讯,星座命理
娱乐
娱乐
时尚
求包养
体育,IT 数码
旅游,娱乐
IT 数码,旅游,健康,娱乐
健康,旅游,美食
旅游
健康
视频音乐,旅游,美食,美女
旅游,娱乐,时尚,美食,文艺
时尚,星座命理,文艺
时尚,游戏动漫,星座命理,IT 数码
娱乐,体育
星座命理,美食
微博奇葩,经济,体育
美食,旅游,IT 数码
文艺,游戏动漫,健康,美食,星座命理
游戏动漫
时尚,新闻资讯,健康,旅游
投资理财
文艺,娱乐,投资理财,旅游,健康,时尚
旅行
美食
娱乐
游戏动漫,文艺
文艺,教育就业,投资理财,军事,汽车,体育
娱乐,文艺,吃货一枚,音乐爱好者,爱旅行,双鱼,k 歌,诗词
美女,旅游,新闻趣事,搞笑幽默
美女,文艺,体育,投资理财
星座命理,娱乐
体育,美女,旅游,汽车
IT 数码,游戏动漫
穿衣美容,双鱼女,动漫迷,爱幻想,奋斗
愤青,热情
娱乐
教育就业,娱乐,文艺
新闻趣事,游戏动漫,教育就业,体育
时尚
体育

新闻资讯,体育
美女,娱乐,游戏动漫
时尚,星座命理,美食,娱乐
新闻趣事,投资理财,旅游,健康,IT 数码
IT 数码,文艺,星座命理
美食,健康,冷笑话,阅读,时事,喜欢音乐,看电视,喜欢旅游,广播,主持
健康,汽车,新闻资讯,旅游,文艺
汽车
校园,数码,亲情,自由职业
海贼王,家庭教师,妖精的尾巴 fans,漫画
美女,新闻趣事,微博奇葩,萌宠,搞笑幽默,娱乐
游戏动漫,健康
文艺
健康,星座命理
德国,巨蟹座
视频音乐
星座命理,其他生活
娱乐
教育就业
淡定,教育,蓝色,学生一族,shopping,许嵩,囧,英语,张翰,搞笑幽默
文艺
旅游,体育
旅游
美食,旅游
美食
情感生活,其他生活,旅游,读书分享
军事,体育
投资理财
投资理财
新闻资讯,健康,娱乐,旅游,美食
IT 数码
娱乐
美食,新闻资讯,健康,体育,军事
搞笑幽默,名人明星,IT 数码,视频音乐,体育资讯,服装设计,摄影控,主持人,交朋友
新闻资讯,转转更健康,关注时政,客观理性,自由主义,80 后,平常心,修身齐家
美食,教育就业,健康
旅游
旅游
新闻资讯
维尼夫妇
穿衣美容,娱乐
時尚,軍事
视频音乐
游戏动漫,旅游

军事,新闻资讯,文艺,IT 数码,投资理财
王菲,YG,VIP
游戏动漫,健康,旅游,美食
运动,金牛,新闻资讯,IT 数码,星座命理,体育,汽车
时尚,IT 数码,新闻资讯,汽车,星座命理
汽车,IT 数码,时尚,投资理财,旅游
文艺,娱乐,旅游,美食,团饭,邪教饭,东方神起
旅游
娱乐,体育
旅游
交友,心情,幻想,爱睡觉,热爱生活,敏感,80 后双鱼座,爱你更爱我,旅行,自由
文艺,星座命理,美食,IT 数码
娱乐
美容护肤,星座控,天秤,淘宝,金牛,健身,娱乐,时尚,新闻资讯,健康
新闻资讯,时尚,健康,娱乐,旅游,美食,星座命理,投资理财
爱自由,猫,梦想,情感,视频音乐,旅游,山东生活,名人明星,星座命理
美食
新闻资讯,旅游,美食,文艺
时尚
新闻资讯,美女,游戏动漫
娱乐,教育就业,健康,旅游,时尚,星座命理
常州生活,搞笑幽默,美食,IT 民工
旅游,美食,穿衣美容,美女,新闻趣事
健康,新闻资讯,美女,体育,汽车
星座命理,旅游,新闻资讯,美女,美食
娱乐,时尚,美女,健康,星座命理
枫叶,健康,文艺
健康,美食,新闻资讯,时尚,旅游
手机
美食,旅游,健康,新闻资讯,军事
文艺,体育,娱乐
体育,娱乐
星座命理,健康,美食,投资理财,时尚,新闻资讯,旅游,IT 数码
美女,体育
时尚,健康,投资理财,美食,娱乐
健康,时尚,星座命理,美食,娱乐,文艺
美食,健康,时尚,爱旅行,偶稀饭睡觉觉
文艺,军事
投资理财,美食,旅游,文艺,健康,时尚
魔域
搞笑幽默,IT 数码
搞笑幽默,娱乐
娱乐,美食,汽车,时尚,教育就业,旅游,健康
汽车,美食,看电影

娱乐
星座命理,美食,时尚,健康
文艺
旅游,美食,健康,军事
美食
美容,减肥,时尚,文艺,美食,旅游,娱乐
学生一枚,彩妆
娱乐
娱乐,体育,游戏动漫
o 型血,强迫症,绘画,美食
星座命理,娱乐
新闻资讯,汽车,旅游,军事,体育
军事,文艺,娱乐
汽车
星座命理,投资理财,文艺
名人明星
爱美食,幻想,时尚,安静,天秤座,旅游,90 後
tvxq,吃喝玩乐
美图摄影
文艺,星座命理
IT 数码,文艺,星座命理,美食
教育就业,旅游,体育
时尚
健康,新闻资讯,旅游,星座命理,美食
画画,90 后但我不脑残,爱猫,我就是我
美食,十大鑽石,鑽石十大品牌,鑽石,钻石品牌,十大品牌,婚戒,钻戒,钻石十大品牌,十大钻石
娱乐
娱乐,投资理财,新闻资讯,旅游,文艺
爱睡觉
体育,美女
旅游,星座命理,美食,文艺,游戏动漫
时事,温柔,浪漫,IT 数码,美食,娱乐,健康,新闻资讯,时尚,美女
美女,时尚,娱乐,电视剧,健康,美食,明星偶像,爱音乐
旅游
室内设计,文艺,新闻资讯,时尚,娱乐,美食,健康
健康,旅游,美食,IT 数码,娱乐
KTM,Aprilia,重机车,哈雷 Harley,铃木 Suzuki,杜卡迪 Dukati,宝马 BMW,川崎 Kawasaki,雅马哈 Yamaha,本田 HONDA
星座命理,IT 数码,美食,体育
星座命理,90 后,宅女,吃货,巨蟹座,发呆,开朗,看电影,神经大条
校园,军事,娱乐
旅游,健康
教育就业,美食,IT 数码,娱乐,体育

爱旅游,学生,小清新,白羊座,喜欢旅游,紫色,英语
健康,文艺,旅游
文艺,旅游
游戏动漫
美食
教育就业,投资理财
军事,星座命理,健康,时尚,体育
旅游,娱乐,时尚,健康
美女,旅游,投资理财,体育,军事
留学,O型血,随性,写字,古灵精怪,自拍,白羊女,自信
体育
美女,时尚,文艺,娱乐
投资理财
LARRY,雪恋夏。,网球,赛车,偶喜欢玩,水瓶座,90,打篮球,幸福
健康,投资理财,教育就业,美女,军事
汽车,娱乐,IT数码
新闻资讯
投资理财,美食,娱乐
旅游,星座命理,IT数码
健康,爱电影
旅游
娱乐,健康,游戏动漫,旅游,美食
爱美食,广州,搞笑,吃货,喜欢旅游,新闻资讯,星座命理,旅游,美女,投资理财
旅游,娱乐,美食
汽车,美女,时尚,美食,旅游,健康,文艺
投资理财,美女,游戏动漫,美食
萌宠
旅游,星座命理
美食,时尚,旅游,汽车,投资理财,娱乐
文艺,旅游,教育就业,健康,时尚
旅游
美食控,漫画,潮,随性,喜欢旅游,双重性格
美食,IT数码,文艺,娱乐,体育
时尚,星座命理,美食,文艺,娱乐
星座命理,文艺,游戏动漫,教育就业
a型血
IT数码,新闻资讯,时尚,旅游,娱乐,爱自由,学生
健康,娱乐,旅游
旅游
旅游,娱乐,美女,文艺,体育
娱乐
文艺,投资理财,旅游,美食,时尚
新闻资讯,文艺,美食,投资理财,时尚,健康,旅游
北京生活,新闻资讯

肇庆生活,白色,天蝎座,沉默,吃,安静,学习,紫色
军事
文艺,教育就业,娱乐
旅游
IT 数码
新闻趣事,时尚,健康,新闻资讯,娱乐
娱乐,IT 数码,体育
吃货一枚,简单,随性,拍照,处女座,军事,旅游,汽车,星座命理
文字控
美食,游戏动漫,体育,娱乐,旅游
体育
时尚
旅游
文艺,娱乐,新闻资讯,IT 数码,汽车,旅游,体育
美食
旅游,新闻资讯,娱乐
美女,时尚,旅游,汽车,娱乐,各种,游泳,唱歌
娱乐
健康,教育就业,美食
文艺,体育,娱乐,教育就业,时尚,旅游,IT 数码
投资理财
军事,星座命理,新闻资讯,美食,旅游
美食
美女,娱乐,汽车,健康,星座命理,新闻资讯,投资理财,文艺,美食,旅游
星座命理,美食,投资理财,美食
猫控
美食
新闻资讯,汽车,军事,IT 数码,体育
汽车,体育
娱乐,体育
美女,娱乐,星座命理,美食,体育
网络电台,podcast,播客
时尚,旅游,美食,娱乐
视频音乐,健康,旅游,美女
时尚,娱乐,健康,旅游,美食
文艺,IT 数码
娱乐,军事,生的平淡,活得开心
微博奇葩,其他生活,新闻趣事,搞笑幽默,健康,汽车,教育就业,娱乐,美食
朝鲜,学习
美食
教育就业,投资理财,旅游,健康,时尚,汽车
新闻资讯,美女,文艺,体育,娱乐
体育,美女
健康,时尚,旅游,娱乐

健康,美女,文艺,体育,娱乐
IT 数码,体育,时尚,汽车,文艺
微博奇葩
教育就业,IT 数码,旅游,美食
nba,新闻资讯,IT 数码,旅游,汽车,体育
娱乐,投资理财
健康,旅游
娱乐,美食,志愿者,做梦,赚钱
时尚,美女,娱乐,投资理财,IT 数码,美食,体育
文艺,娱乐
看小说,看电视,打电脑,高中生
旅游,汽车,游戏动漫,军事,文艺
星座命理,旅游,健康,娱乐
健康,教育就业,投资理财,旅游,美食
赤西仁,韩庚
美食,娱乐,IT 数码,时尚,新闻资讯
娱乐
新闻资讯,军事,体育,娱乐
IT 数码
新闻趣事
美食
文艺
自由,时尚,美食
娱乐,美食,时尚
名人明星,穿衣美容,新闻趣事,视频音乐,星座命理
健康,时尚,星座命理,教育就业
娱乐
投资理财,体育,军事
美食,娱乐,健康
公共政策
体育
人生
大学生,旅游,科幻,90,男人,军事,IT 数码,文艺,娱乐,体育
旅游
爱吃,安静,向日葵,新闻资讯,旅游,星座命理,美食,文艺
新闻资讯,星座命理,旅游,闲人
旅游,投资理财,娱乐,教育就业,军事,新闻资讯
善良,美剧,音乐控,完美主义,电影控,花痴,淘宝,学习,爱美食,书
健康,旅游,军事,文艺,IT 数码
微博奇葩,视频音乐,北京生活,搞笑幽默,新闻趣事,游戏动漫,娱乐,新闻资讯
游戏动漫,旅游,IT 数码
歌手
世界杯
时尚,星座命理,娱乐

新闻资讯,教育就业,军事
时尚,美食,文艺
新闻资讯,旅游,美食
游戏动漫,旅游,美食
时尚,投资理财,旅游,娱乐
体育
遊戲動漫,星座命理
时尚,星座命理,娱乐
娱乐
教育就业
美女
美食,神经大条,善良,散步
时尚,美食,文艺
游戏动漫,旅游,体育
游戏动漫,星座命理,文艺
美女,游戏动漫
摩羯女,王菲
90 後,起床困難戶
教育就业,美食,文艺
健康,体育
IT 数码,汽车,时尚,美女,体育
旅游,美食,时尚,娱乐,投资理财
广告人
旅游
星座命理,娱乐
视频音乐,IT 数码
旅游,体育
IT 数码
健康,旅游,娱乐,美食
爱
旅游,美食,美女,文艺,健康,汽车,新闻资讯,教育就业,IT 数码
投资理财,健康,新闻资讯,旅游,美食,IT 数码
内向,媒体人
健康,美食
IT 数码,文艺,体育
汽车,IT 数码,旅游,体育
健康
娱乐,美食,美女
新闻趣事,视频音乐,微博奇葩,搞笑幽默,美女
美女,旅游,美食
网购
旅游,娱乐
新闻资讯,文艺,美食,旅游
教育就业,旅游,健康,体育

时尚,游戏动漫,美女,美食,军事
敏感,策划,淡定
IT 互联网,手机,爱笑,新闻资讯,IT 数码
旅游
年轻无极限
娱乐
时尚,旅游,军事
星座命理,美食
时尚
美食,健康,新闻资讯,美女
美食,IT 数码,军事,旅游,汽车,投资理财,游戏动漫,健康
投资理财,教育就业,新闻资讯
娱乐,游戏动漫,汽车,投资理财,IT 数码,体育
美食,旅游,健康,文艺,新闻资讯
体育
时尚,健康,旅游,美食,娱乐
美食,健康,旅游,时尚,文艺
旅游,美食,时尚,文艺
旅游
美食,旅游,新闻趣事,江苏生活,新闻资讯
健康,时尚,投资理财,美食,娱乐
体育,汽车,投资理财,旅游,美食
旅游,时尚
游戏动漫
名人明星,娱乐
美女,汽车,星座命理,IT 数码
萌宠,吃,喝,拉,撒
福同享难同当 XD,扫文,推文,小说推荐,言情推荐,言情小说爱好者,言情小说控,言情小说,小说,言情
美女,健康,军事
新闻资讯,旅游,汽车,IT 数码,体育
纠结
文艺,体育,美食,军事
视频音乐,娱乐,时尚,美食,幸福 ing
健康
旅游,娱乐,汽车,IT 数码,体育
搞笑幽默,IT 数码
新闻资讯,美食,文艺,体育
娱乐,IT 数码
新闻资讯
星座命理
体育
旅游,IT 数码,时尚,美食,美女,游戏动漫
投资理财,时尚,新闻资讯,美食,娱乐

新闻资讯,投资理财,游戏动漫,旅游,文艺
美食,健康,文艺,娱乐
健康,新闻资讯,时尚,文艺,旅游,美食
体育
娱乐,重口味
微博奇葩,美食,旅游,视频音乐,茂名生活
IT 数码
汽车
IT 数码,军事,美食,旅游,星座命理
文艺,新闻资讯,娱乐,旅游
游戏动漫
美食,健康
旅游,美食,IT 数码
旅游,时尚,新闻资讯,教育就业,健康,美食
娱乐
拍照,上网,80 后的尾巴,淘宝控,语录,自由职业,IT 数码,旅游,健康,时尚
星座命理
娱乐
视频音乐
娱乐
星座命理,美女,军事,IT 数码,文艺
健康,软绵绵,小吃货,美食
游戏动漫
旅游,游戏动漫,文艺,美食
健康,旅游,美食,时尚
教育就业,健康,旅游,美食,星座命理
体育,新闻资讯
娱乐
体育,旅游,游戏动漫,军事,IT 数码
荷兰,90 后,法国,矛盾,宅,睡懒觉,水瓶座,香港,怀旧,爱旅游
新闻资讯,军事
投资理财,旅游,美食,健康,文艺
香港,奋斗,学习,育儿,吃货一枚,茶,交朋友,电影爱好者,旅游美食爱好者,天平座
娱乐
时尚,汽车,美食,公益,梦想,猫
执着
视频音乐
星座命理,美食
新闻资讯,健康,军事,IT 数码
IT 数码,娱乐,体育
美食,时尚,星座命理,文艺,娱乐
旅游
美女
旅游

娱乐,旅游,新闻资讯,星座命理,美食
美食
IT 数码,军事,美食
文艺
狮子女,有爱，期待，美好,有爱、期待、美好
健康,旅游,美女,美食,文艺,时尚
娱乐
教育就业,投资理财,美食,旅游,健康
美食
娱乐,体育
健康,旅游,美食,文艺,娱乐
新闻资讯,IT 数码,汽车,星座命理,体育
旅游,游戏动漫,汽车,美女,体育
时尚,军事
奢侈品,香港代购,化妆品代购,健康
健康
文艺,投资理财
旅游
新闻资讯,汽车,星座命理
旅游,娱乐,投资理财,教育就业,美食
美女
汽车
健康
校园生活
教育就业,娱乐,IT 数码,时尚
搞笑幽默
军事,汽车,旅游,IT 数码,美食,健康,教育就业
时尚
体育,投资理财
星座命理,美食,文艺
游戏动漫,旅游,文艺
娱乐
教育就业,健康,新闻资讯
视频音乐,新闻趣事,旅游,美食,文艺,体育
IT 数码,文艺,娱乐
游戏动漫,汽车,体育
健康,汽车,军事
游戏动漫,星座命理,娱乐
新闻资讯,时尚,美食
美食,旅遊,健康,時尚,娛樂
军事,文艺,教育就业,游戏动漫,美女,旅游,投资理财,体育
文艺
旅游,游戏动漫
美食,星座命理

健康,新闻资讯
游戏动漫,星座命理,娱乐
IT 数码
搞笑幽默
游戏动漫,投资理财,文艺
游戏动漫,文艺,娱乐
娱乐,星座命理,游戏动漫
旅游,健康,投资理财,时尚,美食
旅游
娱乐,旅游,星座命理,军事
旅游,美食,娱乐
广播剧,男人
旅游,美食,文艺
文艺,投资理财,健康,时尚
IT 数码,健康,汽车
时尚,游戏动漫,美女,bigbang,BBOY,爱跳舞爱音乐,90 后但我不脑残
爱幻想,学习,奋斗,男人,紫色,开心,历史,佛教,wow,爱电影
IT 数码,文艺,投资理财,新闻资讯,时尚,教育就业,健康,旅游,美食
游戏动漫,星座命理,体育
时尚,星座命理,文艺
美食,投资理财,文艺,时尚,旅游
健康
新闻资讯
北京,自拍,医疗,欧美,东庄,莆田
游戏动漫,文艺,娱乐,新闻资讯,星座命理,教育就业,投资理财,时尚
时尚,游戏动漫,美食,文艺
美食,IT 数码,文艺,体育,娱乐
红楼梦
时尚
新闻资讯
死宅,重口味,高达,游戏动漫
美食,健康,新闻资讯,娱乐,时尚
娱乐
文艺
军事,美食,旅游,IT 数码,时尚,汽车,体育
旅游
投资理财,美食
直率,旅游
娱乐,文艺,新闻资讯
IT 数码,投资理财,体育
投资理财,时尚,IT 数码,娱乐,军事
时尚,娱乐
旅游
娱乐

心理学,执着,爱,爱生活,世博志愿者,教育就业,旅游,星座命理,体育,军事
体育
汽车,体育
体育
文艺,美女,汽车,天蝎,标准的好男人,政治,真诚,科幻,执着,爬山
搞笑幽默,文艺
体育
旅游,IT 数码,美女,文艺,星座命理
文艺,健康,娱乐
美食,娱乐,星座命理,新闻资讯,时尚
游戏动漫,时尚,旅游,美食,星座命理
新闻资讯
包过,高等教育,本科,自考,网络教育,专升本,学历教育,教育就业
教育就业
娱乐,星座命理
新闻资讯,旅游
美食,文艺
时尚,健康,文艺
投资理财,行情,社会,钓鱼,喜欢旅游
娱乐
美女,汽车,IT 数码
健康,美食,娱乐
健康
文艺,娱乐
健康,新闻资讯,教育就业,旅游,娱乐
IT 数码,林俊杰,陈奕迅,看小说,篮球爱好者,书,拍照,心理,爱生活,可爱
新闻资讯,体育
新闻资讯,军事
設定偏執狂,绘画爱好者,游戏
星座命理,美食,娱乐
美食
旅游,美食,军事,文艺,体育
汽车,教育就业,美女,星座命理,军事,文艺,美食,IT 数码,体育
IT 数码
文艺,美食,旅游,娱乐
美食,汽车,旅游,体育,娱乐
体育
魏晨,小说迷
微博奇葩,娱乐
星座命理,新闻资讯,旅游,美女,美食
旅游,微博奇葩,新闻趣事,穿衣美容
时尚,美食,娱乐
汽车,投资理财,体育,文艺
美食

文艺,教育就业,美食,旅游
天蝎,音樂,美女,汽车,美食,健康,旅游,美食,娱乐,时尚
军事,投资理财,美女,旅游
旅游,教育就业,新闻资讯
体育
宜昌生活,娱乐
时尚
健康,旅游,娱乐
搞笑幽默,新闻趣事,微博奇葩,美食,台州生活
高一狗,黑子哲也,爱尔敏,艾笠,菊耀,APH 不毕业,通歆粉,狼,狐狸
旅游,健康,投资理财
娱乐,时尚
军事,旅游,体育,IT 数码,汽车
军事,投资理财,旅游,新闻资讯
时尚
发呆,美女,爱 magic,90 后,双子座,微笑,执着,学生一族,偶稀饭睡觉觉,交朋友
体育,健康,时尚,军事,汽车
新闻资讯,健康,文艺
美女,健康,文艺,新闻资讯,时尚,娱乐
动漫控
IT 数码
旅游,IT 数码,健康,美食,文艺
投资理财
健康,星座命理,新闻资讯,投资理财,IT 数码,军事,文艺
文艺,旅游,军事,IT 数码
旅游,美食,健康,时尚
娱乐,IT 数码,健康,汽车
视频音乐
旅游
娱乐
成熟,开朗阳光,娱乐,随和友善,理智,自然,小刘海,90 后,J 迷
美女,专业影视制作,三维动画,特效合成
旅游,健康,教育就业,美食,文艺
军事
文艺
宅男
星座命理,旅游,文艺,时尚,美食
星座运势
时尚,健康,星座命理,娱乐
美食
文艺,投资理财,时尚,IT 数码,健康,旅游,美食
娱乐
SM 家族饭,SHW
钢琴、武术。

听音乐,睡觉,学生
美食,投资理财,娱乐,体育,时尚
体育
IT 数码
投资理财,IT 数码,新闻资讯,娱乐,体育
美食,视频音乐,军事,IT 数码
时尚,旅游,美食,文艺,娱乐
体育,赖床,自由
旅游
时尚,文艺,娱乐
投资理财,时尚,汽车,体育,娱乐
时尚
旅游
视频音乐
潍坊生活,美女,健康,美食,旅游,IT 数码,睡觉
旅游,美食,投资理财,娱乐,时尚
DOTA2,娱乐,基友万岁,90 后,宅男,爱 pchy,暹罗之恋,纯 p 粉
IT 数码
健康,星座命理,时尚,旅游,美食,IT 数码,娱乐
星座命理,美食,娱乐
新闻资讯,时尚,美食,旅游,娱乐
IT 数码,游戏动漫
爱自由,星座控,吃喝玩乐
健康,朋友,真诚,白酒销售,桐乡,乌镇,善良,美食
美食,名人明星,music,双重性格,小说,爱吃,小清新,新闻资讯,文艺,娱乐
星座命理
IT 数码,投资理财
汽车
时尚,美食,娱乐
IT 數碼,遊戲動漫,體育
视频音乐,新闻趣事,微博奇葩,搞笑幽默,穿衣美容
游戏动漫
90 後,动漫控,o 型血,胡思乱想,手机看小说,宅男,微博,睡懒觉,魔兽,广播剧
名人明星
文艺,教育就业,健康,新闻资讯,军事,体育
沈阳生活,新闻资讯,投资理财
游戏动漫
豁达，低调，内秀
美食
情感生活,泉州生活
新闻资讯,健康,旅游,美食,星座命理
美女
美女,汽车,体育
游戏动漫

IT 数码,军事,80 后的末班车,巧克力,爱电影,爱自由,听音乐
时尚,体育,娱乐,游戏动漫
美女,游戏动漫,文艺
旅游,健康
美食
汽车,体育
健康,美食,新闻资讯,文艺
动漫,吳邪,張起靈,盗墓笔记瓶邪党,盗墓笔记瓶邪控,永远爱着老少年,赫海真爱死忠饭,SUPERJUNIOR
美食,汽车,体育,旅游
视频音乐,江西生活,旅游
旅游
美女,星座命理,新闻资讯,健康,娱乐
军事天地,美女,白山生活,视频音乐,IT 数码
文艺
旅游,美食,文艺,明信片,二逼青年
旅游,星座命理,文艺,美食,娱乐
健康,新闻资讯,时尚,文艺,汽车
IT 数码,新闻资讯
美食,旅游,时尚,文艺
美食,娱乐,时尚,体育,旅游
健康
文艺,体育,娱乐,健康
新闻资讯,投资理财,旅游,美食,健康
健康,旅游,美食
美食,军事,IT 数码
IT 数码,时尚,汽车
时尚
美食,娱乐,旅游,时尚
开朗
健康,旅游,投资理财
时尚,旅游
游戏动漫
新闻资讯,军事,教育就业,投资理财,旅游
娱乐
旅游,视频音乐,搞笑幽默,穿衣美容,湖南生活
时尚,游戏动漫,IT 数码,娱乐,教育就业
爱同盟,爱黑色,爱写文,旅游
傻瓜探戈,胡夏,胡夏虾米
星座命理,健康,时尚,旅游,娱乐
其他生活,新闻资讯
投资理财,时尚,新闻资讯,健康,旅游,美食
娱乐
文艺,汽车,时尚,投资理财,健康,星座命理

旅游,美女,健康,时尚,教育就业
IT 数码
游戏动漫,星座,盗墓笔记,瓶邪,黑子的篮球,海贼王,银他妈,静临,赤黑
美食,时尚,娱乐,旅游
视频音乐,文艺,星星,疯子
手工,逛街,舞蹈,健康,投资理财,旅游,美食
教育就业,体育
胡歌,魔兽
教育就业,新闻资讯,时尚,美女,体育,娱乐
体育,汽车,旅游,IT 数码,文艺
星座命理,娱乐,时尚,美食,潮人,服饰,美食爱好者,喜欢听歌
旅游,军事
汽车,旅游,IT 数码
散步,咖啡控
美食,天秤座,低调,爱美食,幻想,无厘头,文艺
娱乐,时尚
旅游
新闻资讯,投资理财,教育就业,时尚
IT 数码
陕西生活
文艺,娱乐
用心,爱你等于爱自己,好性格,汽车,旅游
K 歌,购物,美食,90 後,爱旅游,周杰伦,喜欢听歌,小说,上网
美食,新闻资讯,美女,娱乐
美食
时尚
星座运势,情感生活
美女
美女,IT 数码,文艺,娱乐,体育
军事
时尚
美女
旅游,时尚
娱乐
美食,摩羯女,宝宝,80 后,旅游美食,健康,体育,旅游,时尚,娱乐
健康,娱乐,美女
娱乐,教育就业
新闻趣事,读书分享,星座运势,视频音乐,星座命理,娱乐,IT 数码,文艺
军事,教育就业,体育,娱乐,文艺
新闻资讯,星座命理,旅游
汽车,星座命理,IT 数码
上海生活
美女,健康,时尚,汽车,旅游
星座命理

健康,旅游,星座命理,美食,娱乐
文艺,新闻资讯,游戏动漫,教育就业,旅游
IT 数码
学习
娱乐
穿衣美容,旅游美食,时尚
文艺,美女
星座命理
爱自由,思考,教育就业
汽车
汽车,时尚,体育,娱乐,美女
狮子座
IT 数码,游戏动漫,娱乐
娱乐
游戏动漫
健康,娱乐,时尚,新闻资讯,旅游
娱乐,美食
文艺,娱乐
90 后但我不脑残,旅游
汽车,投资理财,文艺,健康,时尚
旅游
视频音乐,健康,娱乐
星座命理,旅游,娱乐,时尚,文艺
美女
旅游,时尚
电影,投资理财,体育
旅游,时尚,美食,文艺,娱乐
新闻趣事,搞笑幽默,微博奇葩,旅游,穿衣美容,个性,美容护肤,shopping,摄影师
文艺
时尚
星座命理,时尚,军事,健康,IT 数码,投资理财,体育
投资理财,教育就业,时尚,健康,美食
汽车,体育
星座命理
搞笑幽默,旅游,投资理财,娱乐
自由职业,唱歌,随性,英语,射手座,吃货一枚,夜猫子,爱旅游,B 型血,男人
文艺
旅游,电影控
军事,IT 数码
旅游
完美主义,音乐爱好者,上网,看书,摄影,时尚,学生,朋友,真诚,健康
情绪化
健康,美食
健康,体育

时尚,游戏动漫,军事,文艺,娱乐
游戏动漫,美女
文艺,时尚,旅游,娱乐,星座命理
美女
体育,旅游
穿衣美容,新闻资讯,时尚,文艺
星座命理,健康,游戏动漫,体育
文艺,教育就业,汽车,娱乐,体育,军事
汽车,教育就业,军事,体育
视频音乐,军事
健康
IT 数码
广东生活,娱乐
美食,时尚,健康,旅游,娱乐
娱乐,时尚,健康,美食
时尚,美食,旅游,健康,文艺
娱乐,教育就业,投资理财,汽车,旅游,军事,IT 数码,文艺,体育
健康,时尚,汽车,新闻资讯,旅游
新闻资讯
文艺
魔羯座,强迫症,旅游,美食
娱乐,美食,IT 数码,文艺,体育,军事
旅游
星座命理,文艺,娱乐
K 歌,浪子
时尚,美食,星座命理,旅游,健康
健康,文艺
健康,军事
时尚
教育就业,旅游,文艺,新闻资讯
直率，开朗
90 后但我不脑残
体育
文艺,IT 数码
时尚,文艺,教育就业,新闻资讯,旅游
汽车,教育就业,娱乐,军事,IT 数码
星座命理
投资理财,新闻资讯,美食
教育就业,健康,娱乐
IT 数码
自由,上网,摄影,旅行,新闻资讯,汽车,旅游,IT 数码
文艺
微博奇葩,搞笑幽默,旅游,湖南生活,名人明星
名人明星,旅游

娱乐,文艺
情感生活,太原生活,搞笑幽默,时尚,美食,旅游,健康,教育就业
新闻资讯
旅游,新闻资讯,军事,娱乐
游戏动漫,娱乐,新闻资讯
新闻资讯,NGO,共营绿色,宜宾爱心网,环保,公益
微博奇葩,体育,IT 数码,军事
娱乐
星座命理,娱乐
双子女,娱乐,新闻资讯,星座命理,文艺
旅游,游戏动漫
旅游,军事,体育
军事,投资理财,健康,旅游,新闻资讯
旅游,娱乐
时尚,投资理财,军事,IT 数码,体育
旅游,汽车
江苏生活
美食,视频音乐,搞笑幽默,穿衣美容,时尚,旅游,娱乐
时尚,教育就业,军事,娱乐,游戏动漫
娱乐,旅游
拍照,看电影
美食,健康,旅游,文艺,娱乐
星座命理
健康,美女,投资理财,娱乐,IT 数码
校园,淘宝,购物狂,服饰,交朋友,电影爱好者,旅游爱好者,美食,美女,游戏动漫
汽车,美食
娱乐
情感生活
gossipgirl,tvxq,篮球,123,香橙,疯狂,人生,神经质,低调,90 後
美食,旅游,穿衣美容
又懒,又穷,又不老实
健康,时尚,投资理财,娱乐,IT 数码
羽毛球,排球,欧美流行音乐,金牛座,游泳,旅游,日剧,护肤,独身主义,同性恋
美食,文艺,美女,时尚,旅游,娱乐
娱乐,旅游,时尚,美食
美女
名人明星,肇庆生活,淡定,学生一枚,乐观,娱乐,双鱼
体育
美食,坚强,90 後,a 型血,爱旅游,我就是我,矛盾体
星座命理
时尚
军事,IT 数码,游戏动漫,娱乐,体育
旅游,Lucky
健康,娱乐,旅游,时尚

大条,嗜唾,搞怪,闷
喜欢旅游,诗歌,星座命理,时尚,娱乐,文艺
星座命理
游戏动漫,健康,旅游,美食,娱乐
健康,旅游,教育就业,文艺,娱乐
新闻趣事,福建生活,搞笑幽默,旅游,萌宠
星座命理,娱乐
娱乐
星座运势
娱乐,美女,军事,IT 数码
美食
体育,旅游,健康,新闻资讯,美食,娱乐
美食,新闻资讯,汽车,IT 数码,旅游,健康
美食,旅游
IT 数码,文艺
美食,体育,旅游,娱乐
新闻资讯,娱乐,美女,文艺
旅游
资产管理,时事资讯,金融,投资理财,期货开户,研发总部,弘业期货
旅游,美食,文艺
IT 数码,军事,娱乐,美女,健康,汽车,体育
小清新伪文艺,阅读,金牛座,星座命理,旅游,文艺
美图摄影,娱乐
时尚,新闻资讯,文艺,娱乐
娱乐,教育就业,星座命理,时尚,投资理财
SuperJunior,巨蟹座,偶像剧,青春,宅女一枚,淘宝,美食,购物,交朋友,娱乐
美女,时尚
EXO、相爱吧
POLICE,北市区公安分局,警察,网络安全,网警,保定市公安局
美食
军事,星座命理,汽车,娱乐
搞笑幽默
美食,美女,新闻资讯,军事,投资理财,教育就业,体育
鑫多党,90 后,大麦
武汉生活
美食,健康,文艺,娱乐,旅游,星座命理
教育就业,健康,新闻资讯,军事,旅游
厦门,购物,摄影爱好者,NBA
健康,游戏动漫,美食
美食
美女,体育
教育就业,投资理财
美女,娱乐,文艺,教育就业,新闻资讯
健康,旅游,美食,娱乐

时尚
汽车,军事,娱乐
娱乐,美食
游戏动漫,游泳,喜欢听歌,美食
星座命理
爱旅游,90 後,任性,逛街购物,时尚
少女时代,shinee,电视剧,爱睡觉,呆呆,侦查系,天蝎座,韩国,96 年,跳舞
时尚,军事,旅游,汽车,IT 数码,艾米莉
旅游,军事
旅游,健康
游戏动漫
健康,新闻资讯,旅游,IT 数码,时尚,娱乐
美食,娱乐
体育
投资理财,文艺,游戏动漫,娱乐,美女
美食,汽车
新闻资讯,美食,投资理财,健康,旅游,星座命理
旅游
IT 数码,美食,星座命理,时尚,教育就业
美食,旅游
军事,IT 数码,星座命理,健康,新闻资讯,旅游
绿箭侠,休斯顿火箭
美女,IT 数码
汽车
IT 数码,文艺
娃娃机控
娱乐
搞笑幽默,其他生活,旅游,微博奇葩
感性,学生一族,微笑,双子女
新闻趣事,美食,旅游
娱乐,美女
娱乐,美女
美食
旅游,美食,文艺,星座命理,新闻资讯
魏晨,感性,星座命理,美食,新闻资讯,听音乐,敏感,多愁善感,健康,旅游
IT 数码,汽车
简单的生活,男同,男男,不婚主义者,GAY,健康,旅游,美食,体育
健康
时尚
新闻资讯,治疗,美容,祛痘,创业,汽车,健康,旅游,IT 数码,文艺
旅游
美食,IT 数码
新闻资讯,美食,投资理财,健康,娱乐,时尚,旅游
汽车,时尚,美女,娱乐,体育

体育,娱乐
美食,娱乐
健康,旅游,IT 数码,天蝎座,八九滴
娱乐,IT 数码,文艺,体育
健康,旅游,时尚
90 后天蝎座。,爱自由。,爱玩爱聚会。
娱乐
旅游
娱乐,教育就业,新闻资讯,文艺,美食,IT 数码
IT 数码
娱乐,军事
旅游
时尚,星座命理,美食,文艺,娱乐
美食,视频音乐,名人明星
美女,军事,汽车,旅游,IT 数码
健康,美女,美食,体育,娱乐
投资理财,健康,旅游,美食,文艺
军事,IT 数码,文艺,新闻资讯,汽车,美食,娱乐,体育
美女
娱乐,双性恋
美女,时尚
旅游
军事,健康,文艺,美食
娱乐
旅游,投资理财,健康,销售,钓鱼,双鱼,江西九江
时尚,文艺,IT 数码,教育就业,美食,摄影
美食,时尚,娱乐
环保,音樂,公益
美食,新闻资讯,投资理财
娱乐,学习,HelloKitty 控
新闻资讯
娱乐,星座命理,健康
双面性格,美容,90
视频音乐,IT 数码
娱乐
文艺,投资理财,美食
广东生活
旅游,娱乐
淘宝,星座,发呆,腐女,巨蟹,猫控,DIY,游戏动漫
IT 数码,投资理财,体育,军事
娱乐,健康,美食
娱乐
星座命理
健康,爱家人

旅游,星座命理
文字,懒人,健康,快乐
美食,IT 数码,文艺,体育,娱乐
时尚,娱乐,投资理财,健康,美食
90 後,旅游
投资理财,时尚,新闻资讯,文艺,旅游,深职院
游戏动漫,美女,星座命理
玩,购物,旅游摄影,散步,唱歌,旅行,旅游,健康,军事,美食
娱乐,星座命理
投资理财,教育就业,管理,营销,交朋友,新闻资讯,军事,汽车,体育
投资理财,汽车,IT 数码,娱乐,体育
娱乐
美食,吃货,睡觉睡到自然醒,爱旅游,玩儿
旅游,健康,时尚
文艺
旅游,星座命理,投资理财,IT 数码,美食
投资理财,游戏动漫,美女,军事,IT 数码
游戏动漫,娱乐,军事,体育
懒,偶喜欢玩,无厘头,影视,偶稀饭睡觉觉,双鱼女,涂鸦,日本,猫咪控,游戏动漫
营销,80 后的尾巴,乐观,巨蟹,工作狂,汽车
娱乐
健康,娱乐,旅游,游戏动漫,星座命理
军事,IT 数码,娱乐
健康
宅,旅行,旅游,星座命理,娱乐,健康,文艺,美食
美女,时尚,旅游,文艺,美食,娱乐
萌宠,视频音乐
IT 数码
旅游,安静
旅游
游戏动漫,娱乐
摄影工作室
新闻资讯
时尚,健康,教育就业,IT 数码,体育
军事
独立,学生一族,文艺
旅游
IT 数码,体育,游戏动漫,文艺
IT 数码,时尚,旅游,星座命理,美食,娱乐,文艺
汽车,美女,旅游,文艺,娱乐
军事,低调
唱 K,文字控,游泳,开朗,星座,爱音乐,海贼王,Vae 迷,天秤座,90 后但我不脑残
情感生活,美食,娱乐,射手座,文字控,画画,敏感,生活,艺术
美食

搞笑幽默,娱樂
笑,我不爱我,我只是我,我是肉肉,I
IT 数码
文艺,体育,娱乐
投资理财,新闻资讯,教育就业,娱乐,体育,双鱼座
时尚,星座命理,美食
娱乐
微博奇葩,IT 数码,游戏动漫
文艺
娱乐,自由,上网,宅,时尚,潮流,简单,音乐控,影视,文艺
星座命理
旅游,星座命理,美食,时尚,娱乐
文艺,家居,户外,喜欢听歌,热爱生活,时尚达人,聊天,感性,旅游美食,迷糊
游戏动漫,教育就业,娱乐
文艺
时尚
游戏动漫
健康,游戏动漫
文艺
IT 数码,投资理财,时尚,美食,旅游
教育就业,投资理财
娱乐
娱乐,文艺,成都,爱情,好性格,胡思乱想
星座运势
情感生活,旅游,娱乐
梦想,神經病,Music,北京,努力,服装设计师,疯子,咖啡控,自由,涂鸦
八卦杂谈
游戏动漫,时尚,旅游,IT 数码,娱乐
IT 数码,动漫,魔兽忍村,IT
娱乐,美女,时尚,体育
真诚,律师,努力,安静,幸福,思考,喜欢音乐,健康
时尚
搞笑幽默
旅游,健康,美女,汽车,美食
删 PO 狂魔,现居 LA,家乡深圳,博爱党,fo 随缘,腐,九然,COSER
旅游
体育
营销,健康,教育就业,投资理财,星座命理,美食
东方神起
美女,旅游,军事,IT 数码
娱乐
时尚
文艺,美食,娱乐,旅游,健康
名人明星

旅游,健康,新闻资讯,教育就业,投资理财
陈翔,香橙,韩剧,娱乐
广东生活,教育就业,学生代表委员会,中药学院,广东药学院
爱自由,低调,爱旅游,小清新,涂鸦,文艺,新闻资讯,旅游,美食
美食
健康,新闻资讯,美食,文艺,娱乐
旅游,汽车,投资理财,文艺,星座命理
星座控,80后的尾巴,旅游,文艺,教育就业,投资理财,娱乐
时尚
健康,娱乐,旅游,美食
教育就业,旅游,健康,美食
文艺
娱乐
健康,游戏动漫,投资理财,时尚,娱乐
健康,美食,IT数码,时尚,美女
游戏动漫
杉田智和,尊礼伏八,米英普奥,瓶邪黑花,杉山纪彰,小西克幸,索香,鹿鞠,鼠猫,黑塔利亚
健康,校园,囧,时尚,汽车,IT数码,美食
时尚,娱乐,星座命理,旅游
美女
名人明星,投资理财
美食,时尚,游戏动漫,美女
军事
教育就业,旅游,投资理财,文艺,时尚,新闻资讯,健康
偶稀饭睡觉觉,微博控,单身,外貌协会,爱唱歌,模特,欧美控,网购,游戏,手机
娱乐
新闻资讯,体育
體育
旅游,美食
星座命理,旅游
投资理财,娱乐,文艺
文艺,新闻资讯,健康,旅游,美食
交朋友,帅哥,美食,游戏动漫,健康,时尚,娱乐
旅游,美女,健康,娱乐,文艺,汽车,IT数码
汽车,旅游,找吃,稳食,揾食,饿,订餐,点餐,外卖,叫餐
美食
旅游
游戏动漫,美食,星座命理,美女,时尚
娱乐
淡定派,文艺
体育
娱乐
娱乐
科学探索

电影迷,时尚潮流,爱美食,化妆,网络营销,韩国,星座命理,美女,时尚,娱乐
旅游,文艺
娱乐
星座命理
投资理财
娱乐,美食,时尚,投资理财,文艺
旅游,其他生活,视频音乐,挣钱,花钱,吃喝玩乐
游戏动漫,体育
美容,开朗,星座,发呆,安静,快乐,活泼,星座命理
旅游,Music,多愁善感
新闻资讯
健康
名人明星
娱乐
萌宠,美食,吸血鬼日记,矛盾体,欧美,疯子,爱玩,爱美食,冷笑话
美女,搞笑幽默
体育,投资理财,旅游,汽车
微博奇葩
文艺,美食
游戏动漫,星座命理,IT 数码,体育
星座命理
旅游
美女,美食,IT 数码
军事,游戏动漫,美食,IT 数码,娱乐
旅游,游戏动漫,美女,美食,IT 数码
投资理财,汽车,时尚,旅游
微博奇葩,潮人,美食爱好者,娱乐,黑莓,孩子气
校园生活,宠物图集,健康,新闻资讯,时尚,娱乐,美食
娱乐,体育,旅游,美女
美食,旅游,健康,文艺
读书分享,新闻趣事,美图摄影,搞笑幽默,文艺,IT 数码,军事,体育,娱乐
微博奇葩,时尚
投资理财,旅游,IT 数码,文艺,娱乐
美食,游戏动漫
穿衣美容,玩,吃喝玩乐,偶像剧,小女人,双鱼,花痴,90 后但我不脑残
美食,美女
美食,体育,旅游
美食,健康,时尚,旅游
IT 数码,文艺
娱乐
娱乐,体育
娱乐,时尚
美食
健康,旅游爱好者,单身,时尚,文艺,美食,娱乐

摩羯,星座,囧,浪漫,80 后,旅游,电影,篮球爱好者,喜欢听歌
旅游,健康,时尚,美食
美食,旅游
IT 数码
穿衣美容,娱乐
文艺,教育就业
新闻资讯,健康,旅游,星座命理,低调,爱旅游,学习
娱乐,文艺,星座命理,健康,美食
广东生活,旅游
美女,时尚,旅游,娱乐,文艺
电视剧,帅哥,星座控,吃喝玩乐,宅
娱乐
汽车,旅游
天蝎,内向,跑步,杂志,瘦身,电影爱好者,90,逛街族
体育
美食
文艺,美食,健康,旅游,星座命理
旅游,健康,投资理财,时尚,星座命理
美食,旅遊,教育就業,遊戲動漫,美女,體育
娱乐
游戏动漫,娱乐,体育
娱乐
新闻资讯,时尚,健康,星座命理,娱乐
美女,穿衣美容,搞笑幽默
IT 数码,军事,美女
水瓶女,90 后,爱笑,夜猫子,ELF,SuperJunior,发呆,健康,旅遊
文艺,IT 数码,时尚,旅游,美食
文艺
汽车
文艺,美食,IT 数码,时尚,健康,美食
娱乐
IT 数码,汽车,教育就业,投资理财,时尚
教育就业,星座命理,投资理财,旅游,健康,IT 数码
汽车,时尚,健康,旅游
娱乐,美女
美食,旅游
美女,娱乐
新闻资讯,军事,IT 数码
健康,旅游,星座命理,美食
教育就业
音乐爱好者,动漫控,美女
体育,碎懒觉,索普,游泳,小罗,足球
新闻趣事,穿衣美容,名人明星,旅游
文艺,旅游

美食,旅游,汽车,新闻资讯,体育
IT 数码,教育就业,新闻资讯,星座命理,旅游
名人明星,自信,电视
汽车,娱乐
日本生活,文艺
娱乐
娱乐
文艺,旅游,新闻资讯,美女,娱乐
美女,时尚,体育,旅游,美食,娱乐,美食,游戏动漫,健康,IT 数码
真诚,热爱生活,感性,休闲,亲情,校园,健康,教育就业,军事,体育
军事,IT 数码,新闻资讯
微博奇葩,游戏动漫
星座命理
时尚
教育就业
娱乐,文艺,旅游,星座命理
星座命理,体育
体育
萌宠,穿衣美容,视频音乐,时尚,旅游,文艺
旅游,娱乐,美食,健康,古灵精怪,90 后非脑残,搞怪,双重性格,笑,爱幻想
健康,旅游,美食,文艺,娱乐,军事,美女
文艺
娱乐,美食,文艺,星座命理,旅游
娱乐
汽车
美食,游戏动漫
美女
IT 数码,体育
星座命理,搞笑幽默,美女,旅游,娱乐,汽车,军事
文艺
军事
时尚
新闻资讯,时尚,健康,星座命理,游戏动漫,军事
汽车,时尚,IT 数码,健康,旅游
文艺,美食,旅游
娱乐,健康,文艺,教育就业,新闻资讯,投资理财
娱乐,体育,投资理财
健康,旅游,美食,动漫,复古照片,天蝎女,吸血鬼,爱音乐,发呆,星座
旅游,时尚,文艺,健康
游戏动漫,时尚,健康,美食,美女
文艺,教育就业,投资理财,健康,旅游
娱乐,爱生活,喜欢音乐,旅游美食爱好者,吃喝玩乐
新闻资讯,时尚,IT 数码,汽车
IT 数码

搞笑幽默
美女,宅男
美女
娱乐,美食
IT 数码,美女,游戏动漫,时尚,汽车,娱乐
投资理财,美食,健康,文艺,IT 数码
美食
旅游,美食,文艺,健康
旅游,星座命理,娱乐
军事,新闻资讯,体育,汽车
IT 数码,购物,减肥,淘宝,网购,宅女,双鱼座,时尚,美女
文艺
体育,汽车,旅游,健康,美食
娱乐,汽车,IT 数码
小说迷,动漫，cosplay
健康,美食,IT 数码,汽车,教育就业,美女,投资理财,娱乐
喜欢听歌,自然,爱自由,户外,萌宠,旅游,文艺
时尚
时尚
美女
汽车,美女,文艺,军事,体育
体育
投资理财,汽车
健康,星座命理,旅游,美食,娱乐
娱乐
美食,健康,娱乐,旅游
美食,军事,体育,文艺,IT 数码
娱乐,陈翔,电视剧,爱睡觉,听音乐
人生经不起折腾
旅游,星座命理,美食
湛江生活,校园生活,美图摄影,IT 数码,旅游,新闻资讯,军事
时尚,星座命理,健康,娱乐,学习,奋斗,爱旅行,天枰座,宅女
新闻资讯
投资理财,旅游,美食,IT 数码,娱乐
搞笑幽默,聊天,交朋友,旅游美食爱好者,军事,游戏动漫
宅,巨蟹,淘宝控,喜欢越剧,旅游,多愁善感,减肥
娱乐,星座命理
娱乐
美食,旅游,体育,爱旅游,活泼,白羊座
游戏动漫,IT 数码,美女,娱乐,文艺
健康
旅游,星座命理,小说,天蝎座,B 型血,旅游爱好者,影视,K 歌
开心，幸福…
娱乐

旅游
文艺,美食,时尚
景德镇生活,IT 数码
汽车,听歌,美食,电影,旅游
时尚
美食,美女,娱乐,教育就业,星座命理,健康,时尚
文艺
自由,星座
星座命理,娱乐,汽车,发白日梦,多愁善感,微笑,宅
娱乐
IT 数码,汽车,旅游,美食,时尚,投资理财,娱乐
娱乐
旅游,美食
汽车,自由,宅,心理学,健身,多愁善感,奋斗,大学生,设计,射手座
旅游
时尚,旅游,星座命理,美食,娱乐,安静、疯狂、、、
健康,时尚,娱乐,美食,星座命理,维尼热血饭,水瓶座,80 后,左撇子
美国生活,旅游
旅游,美女,健康,星座命理,美食,娱乐,体育
健康,新闻资讯,旅游,星座命理,美食,娱乐
旅游
手工,足球迷,电影迷,多愁善感,科学探索,音樂,体育,军事,新闻资讯,IT 数码
穿衣美容
看电视,喜欢旅游,钓鱼
新闻资讯
新闻资讯
娱乐
体育,文艺
健康,新闻资讯
旅游,汽车,美食,健康,军事
娱乐
搞笑幽默,k 歌,游戏动漫
娱乐
美食,穿衣美容,星座命理,视频音乐,新闻趣事,健康,时尚,新闻资讯,教育就业,投资理财
星座命理,汽车,投资理财,时尚,美女
艺术,娱乐,投资理财,文艺
喜爱夜蒲
美女,美食,娱乐
佛教密宗,佛教居士
新闻资讯,美食,娱乐
旅游,搞笑幽默,IT 数码
单纯,执着,双子,旅游摄影,美食爱好者,古灵精怪,任性,学习,电影爱好者
时尚
新闻趣事

科学探索
文艺, IT 数码, 军事, 游戏动漫
游戏动漫, 文艺
大连生活, 健康, 文艺, 娱乐, 体育
军事
美食
美女, 新闻趣事, IT 数码, 旅游, 南京生活, 娱乐
旅游, 新闻资讯, 体育, 娱乐, 军事
投资理财, 时尚, 星座命理, 旅游, 健康
文艺, 美食, 体育
军事, 时尚
健康
视频音乐, 美女
娱乐
新闻资讯, 汽车
IT 数码, 军事
旅游, 穿衣美容
投资理财, 教育就业, 新闻资讯, 文艺, 体育
IT 数码, 投资理财
星座命理, 健康, 汽车, 美女, 体育
健康
美食
投资理财, 健康, 旅游, 美食, 军事
投资理财, 军事, IT 数码, 旅游, 体育
投资理财
娱乐, 文艺, 教育就业, 旅游, 时尚, 投资理财, 健康, 新闻资讯
美食
体育, 学生, 睡觉睡到自然醒, 篮球
美食, 游戏动漫, 娱乐
美女, 新闻资讯, 月光族
搞笑幽默
文艺
旅游, 健康, 投资理财, 星座命理
游戏动漫
文艺, 体育
文艺, 美食, 时尚, 娱乐, 健康
新闻趣事, 搞笑幽默, 视频音乐, 穿衣美容, 时尚, 健康, 娱乐
美女, 星座命理, 时尚, 娱乐
美剧迷, 喜欢听歌, 医学, 狗狗, 爱旅游, 金牛, 生活, 电视, 广州, 吃喝玩乐
娱乐
健康, 体育, 娱乐
娱乐
美女, 新闻资讯
IT 数码

旅游,投资理财
IT 数码,娱乐,美食
健康,时尚,星座命理,旅游,美食
健康,投资理财,新闻资讯,时尚,娱乐
其他生活
IT 数码,文艺,体育
娱乐
汽车
性格极端
新闻资讯
旅游,投资理财,教育就业,IT 数码,新闻资讯,健康,文艺,体育,娱乐
旅游,IT 数码,游戏动漫,娱乐,吹水,看电影
新闻资讯,科技服务,科技创新,科技企业,科技新闻,科技
IT 数码,时尚,旅游,健康,美食,娱乐
IT 数码,旅游,时尚,健康,体育
时尚,新闻资讯,健康,教育就业,文艺,美女
新闻趣事,校园生活,视频音乐,搞笑幽默,名人明星
投资理财,军事,体育
巧克力,看书,吸血鬼日记,电影,宅,时尚,新闻资讯,健康,美食,旅游
健康
时尚,旅游,美食
投资理财,健康,美食,教育就业,时尚,娱乐
美食,星座命理,娱乐
体育
娱乐,美食,帆布鞋控,手机控
旅游,美女
体育,美女
苏打绿
时尚,游戏动漫
潮流服饰,时尚
娱乐
一切都是浮云,文艺
搞笑幽默,娱乐
天秤座
自拍,疯狂,懒人,成都,理财,爱唱歌,乐观,吃货,运动,旅游
搞笑幽默,新闻资讯,娱乐
星座命理
旅游
旅游,IT 数码,体育
星座命理,美食
军事,美食
娱乐,美女,时尚,游戏动漫
教育就业
IT 数码,游戏动漫

美食,汽车,体育,旅游,娱乐,篮球,交朋友,旅行,自由
娱乐,美食,时尚
IT 数码,文艺,新闻资讯,游戏动漫,时尚,军事,汽车,健康,投资理财,娱乐
健康,IT 数码,投资理财,新闻资讯,旅游,教育就业
名人明星,投资理财
教育就业
穿衣美容,投资理财
健康,美食,旅游
娱乐,文艺,健康,美女,汽车,旅游,IT 数码,体育
旅游
娱乐
时尚
旅游,美食,IT 数码,星座命理,新闻资讯,文艺
IT 数码,体育
美食
IT 数码
没错,就是这样,喵
娱乐,文艺,旅游,时尚,汽车
美女,微博奇葩,搞笑幽默,新闻趣事,名人明星
投资理财,旅游
投资理财,美食,新闻资讯,时尚,教育就业,文艺,旅游
星座命理,文艺
星座命理,娱乐
星座命理,游戏动漫,动漫宅，外星人
吃,旅游美食爱好者
四海为家,90 后非脑残,神经大条,TVB,矛盾体,韩国,美食,时尚,文艺,娱乐
美食
旅游
旅游,健康,偶尔宅,摄影,贪睡
星座命理,娱乐,美食,旅游,时尚
陈翔
新闻资讯,健康,星座命理
军事
美食
双重性格
娱乐
娱乐,投资理财,游戏动漫,美食,军事,健康,IT 数码
文艺
教育就业,投资理财,健康,娱乐,美食
搞笑幽默
活泼开朗
旅游,汽车,美食,IT 数码,体育
IT 数码,单身,wow,爱睡觉,怀旧,乐天派
新闻资讯,健康,军事

天秤座,插画
教育就业,娱乐
娱乐,时尚,星座命理
投资理财,汽车,军事,IT 数码,体育
IT 數碼
教育就业,文艺,健康,美女
梁静茹,双鱼座,文艺
投资理财,美女,文艺,娱乐,体育
体育
文艺,健康,新闻资讯,游戏动漫,IT 数码
健康,美食
时尚,健康
游戏动漫
体育
美女
金牛座,美食,呆呆呆。
台球,双鱼
体育
文艺,美女,军事,体育
星座命理
游戏动漫,军事,IT 数码
娱乐
星座命理,文艺
美食
美食
旅游,娱乐,美女,游戏动漫
美食
体育,IT 数码
军事
职场招聘,美食,新闻趣事,搞笑幽默,体育
其他生活
海绵宝宝
投资理财,旅游,汽车,健康,美食,IT 数码
汽车
购物达人,美食
微博奇葩
旅游,美食,穿衣美容,新闻趣事,搞笑幽默
旅游
娱乐
自由,旅游摄影,完美主义,幸福,爱吃,健康,时尚,旅游,美食,娱乐
军事,IT 数码,美食,娱乐,游戏动漫
旅游,文艺,新闻资讯,美食,星座命理
旅游
体育

娱乐
信息学院党支部
健康
星座命理,美食,旅游,时尚,娱乐
IT 数码
旅游,健康,体育
文艺
汽车
美女,新闻资讯,投资理财,美食,旅游
健康,美食,旅游
健康,时尚,投资理财,旅游,美食
新闻资讯
游戏动漫,体育,美女
游戏动漫
体育
星座命理,旅游,健康,美食,美女,娱乐
娱乐
游戏动漫
投资理财,旅游,娱乐,教育就业,时尚,IT 数码
美女,星座命理,文艺
旅游,IT 数码,游戏动漫,美女
娱乐
美食,美图摄影,读书分享,时尚,旅游,星座命理,娱乐
投资理财
星座命理,美食,文艺,汽车,娱乐,健康
文艺,教育就业,旅游,星座命理,投资理财
教育就业
投资理财,美食
游戏动漫
娱乐
星座命理,新闻趣事,其他生活,搞笑幽默
教育就业,投资理财,文艺,汽车,时尚,美女
吃货,玩,矛盾体,90 后但我不脑残,游戏动漫
爱旅游,摩羯女,小说,睡觉,学生,娱乐,美食,星座命理
星座命理
娱乐
冷笑话,love,喜欢音乐,古典音乐,无厘头,疯狂,美术,法语,美容护肤,摄影师
旅游,娱乐,游戏动漫,美食
体育,娱乐
星座命理
娱乐
幸福,浪漫,时装,时尚,旅游,星座命理,文艺
时尚,健康,文艺,娱乐,新闻资讯,投资理财
新乡生活,教育就业,美食,旅游,娱乐

时尚
旅游,美食,时尚,健康,星座命理,娱乐
旅游
IT 数码
美食
旅游,爱生活,爱睡觉,音乐迷,恐怖片,時尚,文藝,美食,旅遊,星座命理
美食,军事,投资理财,健康,旅游,时尚
时尚,健康,星座命理
健康
汽车
时尚,娱乐,文艺
美食,教育就业,健康,旅游,新闻资讯,文艺,投资理财,娱乐
汽车,军事
旅游,美食,时尚,IT 数码,娱乐
文艺,时尚,娱乐,旅游
娱乐,文艺,IT 数码
文艺,娱乐,旅游,健康,美女
文艺,美食,时尚,旅游,娱乐,星座命理
鹿晗
旅游,星座命理
沉默,天枰座,时尚
搞笑幽默,八卦杂谈,体育资讯,美女,星座命理,娱乐,体育
文艺
星座命理,美食,时尚,美女,娱乐
韶关生活,IT 数码
时尚,娱乐,健康,新闻资讯
汽车
旅游
IT 数码,娱乐,健康
体育资讯,动漫
旅游
体育资讯,体育,娱乐
军事,美食,投资理财,汽车,体育
体育,投资理财,教育就业,美女,健康
时尚,美食,游戏动漫
穿衣美容,健康,旅游,娱乐,时尚
体育,健康,旅游,娱乐,投资理财
旅游,时尚,体育,文艺
美女
娱乐,旅游,美食
美女
娱乐,美食,时尚,汽车,旅游
汽车,游戏动漫,美食,美女,旅游,吃好吃的
减肥,美食,狮子女,学生,开心,充实,忙碌,幸福,低调,善良

其他生活,新闻趣事,旅游,视频音乐,美女
只为等你,爱爱爱付辛博,狮子女,音樂,妖精,梅西
投资理财,健康
旅游
时尚
旅游
微博奇葩,游戏动漫
IT 数码
搞笑幽默,球迷,大学,篮球,简单
投资理财,美食
文艺
投资理财,娱乐
健康,军事,娱乐
政治,音乐达人,睡懒觉,人生,布丁,摩羯女,工作
搞笑幽默,新聞資訊,教育就業,文藝,軍事,健康,新闻资讯,教育就业,文艺,军事
暴走旅行,书,美食
旅游,时尚,投资理财,娱乐,文艺,美食,星座命理,教育就业,健康
无厘头,巨蟹女,苏打绿,小说迷,努力,魏晨,投资
时尚,旅游,娱乐
重口味,搞笑幽默,汽车,娱乐,广州人,时尚达人,学生一族,潮人,喜欢听歌,体育
星座命理,IT 数码,体育
教育就业,旅游,文艺
IT 数码,美食,旅游,游戏动漫,汽车
美食,娱乐
旅游,文艺,娱乐,时尚
汽车,星座命理,IT 数码
美女,汽车,时尚,IT 数码
星座命理
盗墓笔记,淘宝,喜欢听歌,文字,漫画,白羊座,时尚,旅游,星座命理,文艺
东方神起,宠物,周杰伦,强迫症,台球,笑话,美食
汽车,美食,健康,星座命理,旅游,美女,时尚
游戏动漫,健康,新闻资讯,娱乐
体育
美女
文艺,体育,娱乐
投资理财,星座命理,教育就业,时尚
美食
美食,旅游,文艺
书法,可爱
时尚,游戏动漫,星座命理,娱乐,文艺
体育
文艺,新闻资讯
娱乐
时尚,娱乐

汽车,星座命理,IT 数码
汽车,美食,旅游,娱乐
微博奇葩,新闻资讯
旅游
BIGBANG
旅游,美女,IT 数码,美食,娱乐
IT 数码,美食,教育就业,文艺,旅游,军事,新闻资讯,娱乐,体育
健康
娱乐,旅游
投资理财,星座命理
美女
文艺,投资理财,时尚,新闻资讯,健康
IT 数码,汽车,美女,游戏动漫,求交往,宅又囧,大学僧,没房没车,90 後,动漫迷
穿衣美容,河南生活
娱乐
文艺,体育
美女
文艺
休闲,时尚潮流,杭州,会计,腐,网球,爱笑
旅游,IT 数码
美女,军事,体育
健康,美食,时尚,汽车,文艺,唐禹哲
娱乐,新闻资讯,郑州大学,新闻传播,体育
网络,少女时代
科幻,热爱生活,游戏动漫,汽车,IT 数码
浙江生活,萌宠,星座命理,美食,爱旅游,懒,90 後
小孩子气,钢琴,绘画,STARBUCKS 控,学生一族,小资产阶级,苹果控
IT 数码,游戏动漫
汽车,雙魚座,二逼文艺范儿,苦逼穷学生
星座命理,军事,体育
美食,时尚,旅游,娱乐
80 后,喜欢听歌,电影迷
IT 数码
搞笑幽默
健康,投资理财,IT 数码
汽车,旅游,IT 数码
新闻资讯,旅游,IT 数码
娱乐
美食
星座命理
美女,娱乐,体育
金牛,单纯,世界杯,双鱼女,资讯,卡卡,任性
娱乐,80 後,b 型血,爱美食,热爱生活,睡懒觉,散步,爱幻想,乐观,天蝎座
搞笑幽默

听音乐,爱音乐,美术,心理学,笑话,交朋友,市场营销,时尚,文艺,投资理财
美食,微博奇葩,娱乐,时尚,健康,投资理财,旅游
韶关生活
健康,新闻资讯,旅游,星座命理,投资理财,教育就业
娱乐,美女
新闻资讯,军事,文艺,娱乐,体育
新闻资讯,旅游,健康
文艺,娱乐,教育就业,新闻资讯
旅游,文艺
星座命理,健康,文艺,新闻资讯
娱乐,天蝎,音乐达人,80 后的尾巴,湖人,美女,军事,时尚,汽车,体育
微博奇葩
美女,旅游,健康,时尚
教育就业
游戏动漫
美容服饰
教育就业,体育
投资理财,美食
旅游
搞笑幽默
星座命理,娱乐
军事,汽车,IT 数码,娱乐,旅游
教育就业,文艺,时尚,旅游,健康
新闻资讯
新闻资讯,军事,文艺,投资理财,娱乐
旅游
游戏动漫
娱乐,星座命理,汽车,时尚,健康
娱乐
星座命理,文艺,游戏动漫
文艺
旅游,健康,娱乐,美食,IT 数码
读书分享,健康,文艺
感性,简单,天蝎,爬山,摇滚,潮流,强迫症
汽车
美食
美女,星座命理,文艺
体育
文艺,娱乐
时尚
星座命理
游戏动漫,新闻资讯,教育就业,美食,IT 数码
娱乐
IT 数码

互粉,文字控,阅读,原创,旅游美食爱好者,时尚,汽车,文艺,旅游,美食
旅游
文艺,娱乐,时尚,健康
美食,娱乐,时尚,星座命理
新闻资讯,美女,体育
体育,娱乐,文艺
IT 数码,时尚,旅游,娱乐,美女,健康,美食
旅游,时尚,美食,娱乐
穿衣美容,时尚,星座命理
投资理财,教育就业,健康,旅游,时尚
汽车
新闻资讯,投资理财
游戏动漫,美女,体育
体育,投资理财,IT 数码
健康,投资理财,旅游,体育
娱乐,美女,游戏动漫
娱乐
投资理财,新闻资讯
娱乐
美女
时尚,美女
星座命理,娱乐
游戏动漫
旅游,体育
体育,汽车
体育,美女
游戏动漫,教育就业,IT 数码
美食,军事,IT 数码
汽车
星座命理,美食,娱乐
睡觉睡到自然醒,爱睡觉,学生一族,游戏动漫
视频音乐,娱乐
美女,军事,文艺,体育,娱乐
旅游,文艺,星座命理,美食,健康,娱乐
健康,投资理财
星座命理,娱乐,游戏动漫
美食,微博奇葩,萌宠,旅游,穿衣美容,时尚潮流,猫,巨蟹
军事,美女,时尚,美食,美满姻缘,幸福久久
IT 数码,健康,旅游,时尚,美食,娱乐
健康,娱乐
情感生活,搞笑,90 后但我不脑残,脾气火爆者,爱好唱歌,生性敏感,爱做梦,双鱼座
军事,娱乐,IT 数码,游戏动漫,美女
时尚,教育就业,旅游,美女,娱乐,新闻资讯
处女座,电影控,90,美食,体育,旅游,美食

新闻趣事,视频音乐,名人明星,搞笑幽默,南京生活
娱乐
投资理财,驾驶技巧,摄影,主持,活泼好动,喜欢交朋友,娱乐
IT 数码,体育,新闻资讯,美女,健康,文艺,娱乐,时尚,汽车,教育就业
娱乐,旅游,时尚,美食,健康,星座命理,哈哈，爱吃爱睡。
健康
娱乐,时尚,游戏动漫,健康,星座命理
情感,自行车
汽车,时尚,IT 数码,美女,文艺
投资理财,娱乐,教育就业,时尚,健康
文艺,旅游,星座命理,美食,健康,体育,娱乐,美食,军事,IT 数码
投资理财,健康,旅游,时尚,文艺
娱乐,文艺,军事,旅游,汽车
游戏动漫,星座命理,旅游,健康,美女,汽车
美食,健康
交友,潮流时尚生活,浪漫,感性,旅游美食,梦想,听音乐,购物狂,游戏动漫,星座命理
IT 数码,体育,娱乐,时尚,美食
娱乐
街拍,微博控
萌宠,穿衣美容,美女
吉他,游戏动漫,文艺,美食,军事,娱乐
幸福
军事
时尚,美食,文艺,娱乐,旅游,投资理财,健康
IT 数码,新闻资讯,旅游,美食
健康,美食,旅游,时尚,文艺
体育,游戏动漫,美食,旅游,汽车,文艺,娱乐
微博奇葩,体育
Music,汽车
美食,时尚
汽车
汽车
美食,娱乐,文艺
美女,美食,汽车
娱乐
时尚,健康,敖东鹿胎颗粒
健康,美食,投資理財
宠物图集,八卦杂谈,搞笑幽默,上海生活,新闻资讯,教育就业,时尚,投资理财,星座命理
搞笑幽默
星座运势,搞笑幽默
新闻资讯,健康,旅游,IT 数码,美食
善良,微笑,睡觉,自由,旅行,宅,新闻资讯,人生,网上创业,网上购物
汽车
游戏动漫,IT 数码

IT 数码,娱乐
星座命理
汽车,文艺,娱乐,旅游,美食,体育
旅游,娱乐,美食,文艺,健康,时尚
体育,娱乐
动漫
文艺
安徽生活,文艺
穿衣美容
汽车,娱乐,体育,星座命理,旅游,文艺
时尚,星座命理
时尚,美食,健康,教育就业,旅游
投资理财,旅游,美食,军事,IT 数码
新闻资讯,娱乐,教育就业,健康,星座命理,美食
微博奇葩,新闻资讯,娱乐,游戏动漫,军事,文艺,90,安静,宅
交友,教育就业,文艺,健康,新闻资讯,娱乐
娱乐
体育,新闻资讯
旅游,健康,投资理财
苹果
娱乐
天枰座,搞笑,团购,淘宝,学生,猫咪控,游戏动漫,教育就业,健康,美食
旅游,新闻资讯,星座命理
娱乐
健康
美食,时尚,星座命理,文艺,娱乐
时尚,汽车,体育,旅游
美女,文化教育,生活技能培训,文化教育培训,新生代打工青年,公益
微博奇葩,移动互联网,心情,游戏动漫
旅游,美容护肤
旅遊,BBS,華南師範大學,天平座,大学生,旅游,新闻资讯,IT 数码,文艺,教育就业
体育,汽车,娱乐
健康,娱乐
旅游
教育就业
美食,美食
IT 数码
旅游,娱乐
旅游,时尚
体育,健康,时尚,美食,旅游,星座命理
健康,新闻资讯,旅游,美食
旅游
校园生活,娱乐
健康,星座命理,娱乐

娱乐
体育,旅游,文艺,娱乐,军事,美食
美女,IT 数码,美食,健康,旅游
新闻资讯,军事
投资理财,IT 数码
旅游,健康,娱乐,投资理财
时尚,美食,星座命理,娱乐
健康,旅游,文艺,娱乐,时尚,美食
投资理财,娱乐
军事,汽车
游戏动漫,时尚,投资理财,星座命理,文艺
游戏动漫
宋词，茶，咖啡
额，我很闷～
汽车
旅游,处女座,学生,时尚,爱生活爱旅游,音乐控,潮流,简单,感性,爬山
游戏动漫,健康,星座命理,娱乐
乐观
娱乐,自戀,90 後,爱笑,美食控
新闻
娱乐
搞笑幽默,名人明星,校园生活,杭州生活,新闻资讯,美女,教育就业,投资理财,体育
美女,喜欢唱歌,烹饪,爱逛街，吃货。
美女
娱乐,美女,投资理财
新闻趣事,投资理财,新闻资讯,体育,娱乐
教育就业,汽车,健康,旅游,星座命理
穿衣美容,搞笑幽默,健康,娱乐,旅游,时尚
微笑,健康,时尚,旅游,教育就业,美女,新闻资讯,娱乐,军事
投资理财,汽车,美女,美食,旅游
星座命理,时尚
体育,美女,旅游,文艺,游戏动漫
军事,美食,美女,游戏动漫,时尚,娱乐,IT 数码
健康,时尚,军事
星座命理
健康
美食
爱笑,旅游
重庆,美食,健康
旅游,新闻资讯,游戏动漫,文艺,娱乐
IT 数码,游戏动漫,汽车,娱乐,体育
穿衣美容,娱乐,汽车,时尚,健康,旅游
健康,新闻资讯,旅游,新闻趣事,没事穷逛逛
娱乐

美食,投资理财,娱乐,新闻资讯,IT 数码
旅游,美食
点歌,品牌,旅游,美女,汽车,IT 数码,娱乐
美女,90 后追求淡定,金牛座,被处女座伤过,爱吃,爱睡
旅游
时尚,美食,教育就业,投资理财,健康,新闻资讯
看小说!
健康,投资理财,美食
旅游,香橙
紫色,乐观,吃货,韩剧,摩羯女,看电影,淘宝,热爱生活,休闲,闷骚
体育
文艺,旅游,时尚,健康,美食,娱乐
健康,美食,文艺,娱乐,时尚,新闻资讯,星座命理
旅游,娱乐,体育,文艺
新闻资讯,投资理财,文艺,时尚
时尚,健康,旅游,美食
星座命理,美食,娱乐
文艺
教育就业,投资理财,新闻资讯
娱乐,旅游,星座命理,美食,IT 数码,时尚,健康
健康,旅游
新闻资讯,军事,娱乐,游戏动漫
视频音乐
美食
美女,旅游,军事,体育,汽车
游戏动漫,美女,体育,文艺
时尚,文艺
健康
旅游,美女,体育,IT 数码,汽车
美食
新闻资讯,IT 数码
旅游,美食,教育就业,投资理财,时尚
文艺,娱乐,微笑
美食,教育就业,健康,IT 数码,娱乐
新闻资讯
旅游
新闻资讯,IT 数码,时尚
游戏动漫
健康,娱乐,新闻资讯,旅游,时尚
娱乐,美食,旅游,星座命理,时尚
IT 数码
旅游,健康,文艺
体育
体育,娱乐,IT 数码

娱乐
娱乐
美食,IT 数码,游戏动漫
健康,星座命理,美食,投资理财,娱乐
旅游,搞笑幽默,微博奇葩,其他生活,投资理财,新闻资讯,游戏动漫,汽车,健康
娱乐,时尚
星座命理,军事,游戏动漫,美女,娱乐
名人明星
巨蟹女,90 后,音乐迷,金贤重,k 歌,懒人,睡懒觉,紫色,萌宠,娱乐
投资理财,军事
汽车,美女,游戏动漫,IT 数码,投资理财,时尚
游戏动漫,美食,旅游,文艺
IT 数码,军事
体育
旅游,娱乐
文艺
美食,游戏动漫,旅游,娱乐,体育
投资理财
名人明星,校园生活,搞笑幽默,其他生活,新闻趣事
娱乐
文艺,军事
汽车,教育就业,投资理财,健康,美女,娱乐
健康,旅游,娱乐,IT 数码,体育,麦霸
新闻资讯,体育
新闻资讯,时尚,IT 数码,旅游,星座命理,美食,文艺,娱乐
新闻资讯
美女,文艺,IT 数码,娱乐
猫咪控
新闻资讯,美食,时尚,旅游,美食,汽车,体育
健康,旅游
星座运势,旅游,投资理财,军事,娱乐
美食,时尚,健康,旅游,美食,娱乐
美食,星座命理
新闻资讯,旅游
娱乐,军事,新闻资讯,游戏动漫,汽车
福建生活
健康,旅游,星座命理,美食,娱乐
我逞强,我多心,我害怕,我敏感,我神经,我倔强,我偏执
星座命理,军事,文艺,时尚,娱乐,游戏动漫
美女
娱乐
旅游,星座命理,新闻资讯,娱乐,教育就业
体育,汽车
体育

美女
体育
书画
娱乐
健康,时尚,娱乐
体育,文艺,军事,旅游,娱乐,IT 数码,汽车,教育就业
旅游
臭美,心情,旅游,新闻资讯,星座命理
腐女,吃货
游戏动漫,军事,旅游,美食,时尚,文艺
美女,教育就业,娱乐,体育,文艺
美食
娱乐,IT 数码
美女,时尚,健康,美食
时尚,健康
新闻资讯,游戏动漫,文艺,投资理财,旅游
瓶邪
萌宠,娱乐
穿衣美容,视频音乐,娱乐
IT 数码,体育,文艺,健康,旅游
健康,新闻资讯,美女,游戏动漫
旅游
美食
旅游,爱我的人都长命,直爽,爱恶搞,耍宝,颜控
美食
体育
美食,健康,星座命理,新闻资讯,旅游
旅游
美女
时尚
健康,美女
时尚
健康,文艺,娱乐
旅游
娱乐
美女,IT 数码,投资理财
中影集团剪辑师
美食
体育
投资理财,教育就业,汽车,旅游,体育
体育
旅游,美食,IT 数码
健康,英语,羽毛球,摄影,小说,文艺
IT 数码,时尚,旅游,美女,游戏动漫,电影爱好者,美食

经济实用
美食,星座命理,文艺,游戏动漫,时尚,娱乐
新闻资讯,美食,IT 数码,体育,娱乐
美食,时尚,IT 数码,体育,健康,旅游,美食
星座命理,旅游
教育就业,IT 数码
体育
视频音乐,游戏动漫
新闻资讯,文艺,娱乐
文艺,旅游,时尚
游戏动漫,娱乐,汽车,时尚,健康,美食
爱音乐,可爱,music,幸福,娱乐,时尚
美食
文艺,健康,星座命理,娱乐,时尚
汽车
时尚
健康,投资理财,娱乐
视频音乐
EXO,娱乐,狮子女,唱 k,咖啡控
体育,军事,文艺,美食,哲学,心理,篮球爱好者,执着,简单,单身
旅游,星座命理,文艺
IT 数码,体育,曼联球迷,电影迷,科幻,卡卡
新闻资讯,旅游,美食,娱乐,文艺,时尚
美女,娱乐
星座命理,健康,时尚,美食,娱乐,文艺,养成习惯,设计师
时尚,IT 数码
旅游美食,善良,偶稀饭睡觉觉,喜欢旅游,疯丫头,选择控,喜欢跳舞,喜欢听歌,唱歌,开朗
旅游美食爱好者,投资理财
投资理财,旅游
旅游,美食,时尚,健康,投资理财
美女,美食,娱乐,时尚,汽车
美图摄影,美食,新闻资讯,投资理财,健康,旅游
时尚,旅游,星座命理,文艺,娱乐,80 末 90 初
很有爱
跑步！篮球
我就是我,爱猫,电影爱好者,旅游美食爱好者
美女,教育就业,娱乐,健康
时尚,娱乐,健康,美女,美食,旅游
美女,娱乐,旅游
IT 数码,新闻资讯,健康,投资理财,汽车
星座命理,美女,游戏动漫,娱乐
美食,文艺,旅游,狮子女
旅游,教育就业,美食,文艺
IT 数码,美食,旅游,汽车

汽车
文艺
美食
美女,美食,文艺,时尚,投资理财,旅游,美食
投资理财
美食,旅游,时尚,星座命理,美食
旅游,星座命理,美食,投资理财
游戏动漫,汽车,美女,旅游,IT 数码,娱乐
时尚,教育就业,美食,旅游,健康
投资理财,新闻资讯
新闻资讯,IT 数码
游戏动漫
时尚
汽车,娱乐,星座命理
校园生活,情感生活,星座运势
星座命理
娱乐,无厘头
游戏动漫
美图摄影
健康,教育就业,新闻资讯,文艺,投资理财,娱乐
实证实修,极乐世界,阿弥陀佛,佛
美食,旅游,投资理财
IT 数码,汽车,美食,旅游
娱乐,旅游,美女,军事,文艺,IT 数码
体育,美女,游戏动漫,汽车,军事
投资理财
娱乐,美女
美女,遊戲動漫,汽車
新闻资讯,教育就业,时尚,星座命理,文艺,娱乐
美食,IT 数码,时尚
娱乐,文艺
文艺,开心,时尚,健康,旅游,美食,娱乐
时尚,教育就业,汽车,文艺,美食
教育就业,投资理财
娱乐,汽车,美食,旅游,沙扒湾,情感,嘉应学院,园林景观设计,兜风,吉他
电话营销,数据分析与挖掘,DM,eDM,SMS,TM,推广,CRM 系统开发,营销推广,网络营销推广
IT 数码,星座命理
美女
星座命理
新闻资讯
時尚,娛樂
教育就业
政治,新闻资讯,香港,考研,培训,读书,汽车,创业
旅游,美食

体育,娱乐
IT 数码,旅游,美食
娱乐
自拍,购物,美食,听音乐,上网,书,music,双子
旅游,新闻资讯,教育就业
游戏动漫,体育,IT 数码,军事
体育
时尚,旅游,美食,文艺
时尚,旅游,美食,文艺,娱乐
投资理财,旅游,娱乐,美女
星座运势,情感生活,幸福,青春,学习,奋斗,天秤座,爱音乐
游戏动漫
文艺,时尚,旅游,美食,娱乐
时尚,追求内心的快乐,崇尚自由,霸气,直爽,旅行,读书
美食,时尚
网游,寂寞,90 后
文艺
娱乐,投资理财
追星,音乐发烧友,汽车,游戏动漫,美女,IT 数码,文艺,体育
军事,时尚,汽车,新闻资讯,旅游,IT 数码,文艺
读书分享,美图摄影
旅游,娱乐,体育
旅游,健康,投资理财
美食,娱乐
魏晨,追星
时尚,健康
时尚,旅游
星座命理
星座命理,时尚,文艺,美食
投资理财
星座命理,旅游,健康,美食,娱乐
旅游,美女
文艺
奋斗 ing,学生党,巨蟹座,手机控,起床困难户
视频音乐,娱乐
时尚,健康
文艺,投资理财,教育就业
娱乐
时尚,旅游,健康,星座命理,投资理财
美食,旅游,时尚,投资理财,IT 数码
时尚,健康,星座命理,军事
汽车
时尚,文艺,军事,美食,星座命理,健康,旅游
IT 数码,军事,新闻资讯,美女

旅游,投资理财,汽车,新闻资讯,军事
北京 Lifestyle,Travel,娱乐
游戏动漫
美女,IT 数码,汽车,游戏动漫,军事,美食
星座命理,名人明星,萌宠
新闻资讯,游戏动漫,文艺,体育,娱乐
IT 数码,体育
文艺,游戏动漫
文艺,旅游,时尚,娱乐,美食,星座命理
新闻资讯,IT 数码,时尚
时尚,娱乐,美食,旅游,星座命理
娱乐
叫我 W
旅游,美食
天枰女,瘦瘦瘦,娱乐,投资理财,健康,星座命理
娱乐
文艺,爱唱歌,白羊女
爱吃,微博控
体育
搞笑幽默,美食,投资理财
时尚,美女,游戏动漫
健康
视频音乐
广东生活,微博奇葩,星座命理,新闻趣事,搞笑幽默
投资理财,旅游,文艺,时尚,美食
旅游
名人明星,娱乐
时尚,旅游,美女,娱乐,汽车,教育就业
IT 数码
美女,军事,IT 数码,体育
新闻资讯,健康,教育就业,美食,娱乐
旅游,汽车
旅游,星座命理,美食,娱乐,文艺,美女
美食,星座命理,娱乐
美食
陕西生活,体育
体育
旅游
汽车,游戏动漫,娱乐
IT 数码,教育就业,新闻资讯,体育,军事,旅游
新闻资讯,军事
IT 数码,汽车
星座命理
娱乐,健康,时尚,星座命理,美食,文艺

健康,处女座,90 後,喜欢音乐,火影忍者
健康,时尚
体育,投资理财,汽车,娱乐
时尚
90
巨蟹,外贸,网络营销,吃喝玩乐,创业,法语,英国,新闻资讯
美女,文艺,IT 数码
星座运势
美食,时尚,游戏动漫,星座命理
文艺,体育
游戏动漫,旅游,汽车,美食
游戏动漫,IT 数码,文艺,体育
美食,旅游,文艺,全职高手
美食
美女,旅游,汽车
旅游,投资理财
新闻资讯,旅游,投资理财
体育,文艺
穿衣美容
新闻资讯,投资理财,娱乐,体育,IT 数码
星座命理,娱乐,游戏动漫,美女,体育
星座命理,文艺,军事,体育,娱乐,投资理财,IT 数码,时尚,美食,游戏动漫
美食,娱乐,美食,文艺,旅游
美食,汽车,IT 数码,美女,娱乐
周杰伦
健康,旅游,星座命理,投资理财,娱乐
林俊杰,听音乐,睡觉,书
狗狗,平面设计
星座命理,娱乐
新闻资讯,美食,时尚,娱乐,旅游
时尚
时尚,星座命理,文艺,美食,投资理财,美女
时尚潮流,篮球爱好者,双重性格,上网,时尚,睡觉睡到自然醒,爱自由,随性,绘画
美食
体育,IT 数码
文艺
投资理财,汽车,IT 数码,新闻资讯
教育就业,IT 数码,新闻资讯
微博奇葩,女魔头
时尚
IT 数码,美食,星座命理,旅游
体育
军事,星座命理
美食

穿衣美容,旅游,美食,星座命理,搞笑幽默
星座命理
美女,健康
旅遊,時尚,文藝,娛樂,美食
教育就业,汽车,娱乐,文艺,IT 数码,体育
福建生活,旅游,体育
名人明星
旅游
娱乐
美女,。。。。。
娱乐
时尚
射手女
美女
教育就业
新闻资讯,投资理财,健康,美食
游戏动漫,星座命理,投资理财
旅游,汽车
美女,娱乐
爱美食,法国,90 后但我不脑残
体育
健康
娱乐,美食,游戏动漫,文艺,星座命理
旅游,星座命理,娱乐
投资理财
旅游,时尚,美食
时尚,游戏动漫
星座命理
健康,旅游,娱乐,美食,时尚
美食,娱乐,时尚,新闻资讯,健康,旅游,星座命理
美食,时尚
新闻资讯,健康,时尚
旅游
娱乐
娱乐
军事,IT 数码,文艺,娱乐,新闻资讯,旅游,美食,时尚
情感生活,新闻资讯,教育就业,娱乐,星座命理,文艺
动漫
星座命理
娱乐,美女
幻想,爱玩,聊天,电影迷,娱乐,健康,文艺,旅游,美食,星座命理
体育,游戏动漫
娱乐
旅游,娱乐,星座命理

美女
文艺
微博奇葩,体育
IT 数码
美图摄影
投资理财,健康,美食,新闻资讯
健康,投资理财,汽车,星座命理,美食
娱乐
娱乐,美女,文艺,体育,游戏动漫
美女,汽车,美食,旅游
美图摄影,美女,健康
美食爱好者
娱乐,军事
搞笑幽默
打篮球,我喜欢五月天,偶稀饭睡觉觉
军事,美女,美食,IT 数码,娱乐
新闻资讯,健康,文艺,娱乐,IT 数码,军事,旅游,体育
互粉
旅行,打篮球,新闻资讯,旅游,体育,娱乐,桌球,学生,唱歌,上网
银魂,,,,。。。
文艺,时尚
文艺,旅游
大学,校园,娱乐,时尚,新闻资讯,健康
汽车,美女,健康,旅游,IT 数码,80 后,看电影
宠物图集,美剧,足球,文艺,游戏动漫,健康
新闻资讯,投资理财,美食,星座命理,娱乐
搞笑幽默,游戏动漫
旅游,教育就业,投资理财,美食
时尚
游戏动漫
文艺,旅游,时尚,美食,投资理财
娱乐,教育就业,旅游,投资理财,美食,文艺
羽毛球,梦想,武术,猫咪控,music,演员,微博,奋斗,好性格,爱猫
名人明星,旅游,星座命理,搞笑幽默,河北生活
美食,起床困难户,Music,美食爱好者,浪漫,胡思乱想
体育
美女
水瓶座,执着,新闻资讯,旅游
健康,新闻资讯,时尚,教育就业,星座命理,美食
微博奇葩,名人明星,穿衣美容,时尚,美女,美食,文艺
水瓶女,美食控,欧美,宅女一枚,爱吃,发呆,爱生活,神经质
时尚,娱乐
IT 数码
游戏动漫

汽车,时尚,娱乐,体育,IT 数码
文艺,旅游,教育就业,军事,体育,游戏动漫
健康,旅游,美食
旅游,游戏动漫,娱乐
学习永无止境,喜欢旅游,很想去旅行,旅游摄影
文艺,健康,美食,时尚,投资理财,旅游
文艺,娱乐,星座命理
美食,时尚,文艺,体育,娱乐
旅游,健康,投资理财,IT 数码,文艺
善良,小女人,宅女一枚,90 後,IT 美女,平板,科技,单身,手机,IT 数码
love
IT 数码
名人明星,山东生活,娱乐,美女,投资理财,军事,旅游
新闻资讯,红色旅游,习仲勋,革命纪念馆,马栏山,旬邑县,旅游
娱乐,星座命理,美女,健康,旅游
喜欢旅游,诗歌,销售
军事,IT 数码,投资理财,体育,娱乐,时尚
星座命理,游戏动漫,旅游,美食,娱乐
娱乐
体育,文艺
投资理财,时尚,健康,旅游,美食,娱乐,学生听歌
证券研究分析,个股利好,出货内线,盘中分析,股票分析,涨停黑马,私募内参
星座命理
美女,文艺
穿衣美容,文艺,旅游
美女,健康,投资理财
文艺,时尚
汽车,美食,旅游,投资理财,星座命理
新闻资讯,美食,军事,时尚,娱乐
美女
大学生
体育,美女
娱乐,星座命理
星座命理,旅游,娱乐,文艺,美食
喜欢旅游,music,睡觉,学生,小清新
美女,娱乐,美食
游戏动漫,军事,汽车,美女,文艺,体育
教育就业,新闻资讯
星座命理,汽车,健康,娱乐
星座命理,IT 数码,美女,娱乐
名人明星,娱乐
旅游
时尚,投资理财,星座命理,美食,娱乐
娱乐

旅游美食爱好者,林宥嘉,bigbang,宅女加腐女,睡觉睡到自然醒
美女,体育,新闻资讯,星座命理,文艺,娱乐
投资理财,汽车,IT 数码
美食,健康,新闻资讯,汽车,旅游
内蒙古生活
朴有天,爱旅行
NBA
其他生活
时尚,旅游,文艺,娱乐
美图摄影,文艺,美食,教育就业,旅游,健康
旅游,k 歌,阳光,90,微博控,星座控
摄影,星座控,睡懒觉,90 後,爱美食,阳光,开朗,吃货一枚,星座,美食控
手机,摄影爱好者,小清新,休闲,IT 数码,娱乐,旅游,时尚,天蝎座,羽毛球
宝宝
健康
穿衣美容,时尚
旅游,游戏动漫,健康,美食
投资理财,军事,IT 数码,文艺
IT 数码,搞笑幽默
互联网
时尚
美食
娱乐,旅游,美食,星座命理,体育
教育就业,新闻资讯,健康,美食,文艺,媒体人,艺术,印刷人,印刷品,包装印刷
爱家人,白羊女,家具设计,90 後,亲情,大学
名人明星,娱乐
IT 数码
教育,艺术,健康,传媒,广告,书法,绘画,电影爱好者,文艺
美图摄影,健康
美食,娱乐
音乐控
文艺,IT 数码
娱乐
旅游,美食,体育
冬眠,秋盹,夏乏,春困,巧克力,成长 ING,贪吃,90 後
睡觉
手机看小说,看小说,新闻资讯,时尚,健康,娱乐,文艺
娱乐,汽车,星座命理,旅游,游戏动漫
时尚,文艺,教育就业,健康,美食
旅游
美食,体育,时尚,汽车,娱乐
穿衣美容,汽车
娱乐,旅游,美食
游戏动漫,体育

旅游
旅游
美食
旅游,军事,教育就业,健康,体育,娱乐
瘦身,减肥 ing
新闻趣事
游戏动漫,旅游
IT 数码,文艺,新闻资讯
美食,时尚,教育就业,投资理财,文艺,IT 数码
游戏动漫
投资理财,新闻资讯,旅游,IT 数码,汽车,娱乐,体育
汽车,时尚,投资理财,美女,IT 数码
旅游,星座命理,美食,娱乐
娱乐,健康,旅游,美食
动漫
娱乐
文艺,体育
旅游,搞笑幽默,视频音乐,新闻趣事,微博奇葩
搞笑幽默,汽车
妖精,金希澈,西班牙,音樂,矛盾体,影视,咖啡,欧美控,双重性格,学生一枚
娱乐,时尚
摄影,旅游
美女,娱乐
美女
美食
搞笑幽默,吃喝玩乐,重口味,爱自由
美食,文艺,旅游,时尚
淡淡的紫,蓝色,偶喜欢玩,青春,简单,睡觉睡到自然醒,刘恺威,美食,旅游
医学
文艺
做梦,爱电影,天秤,文字控,微博奇葩,萌宠,时尚,美食
美食,IT 数码,宅在家
旅游,IT 数码,时尚,文艺,娱乐
文艺,体育,健康,军事,娱乐
星座命理,新闻资讯,健康,投资理财,体育
旅游,美食,娱乐
文艺,美食,美女,IT 数码,游戏动漫
游戏动漫,体育
文字,睡觉,自由,真诚,沉默,书,喜欢旅游
美女,时尚
旅游,搞笑幽默,娱乐
健康,教育就业,美食,军事,IT 数码
军事,IT 数码,文艺,体育,娱乐
军事,IT 数码

新闻资讯,体育,娱乐,健康,旅游
投资理财,汽车,美女,健康,IT 数码
美食,新闻资讯,健康,文艺
仙剑,军事,IT 数码
文艺,健康
美女,文艺,苹果粉
旅游,娱乐,美食
娱乐,美女,IT 数码,深圳房地产,房屋买卖,金融贷款,房地产经纪人,文艺,时尚,旅游
新闻趣事,美图摄影
旅游,文艺,娱乐
篮球,画画,旅游,美食,文艺,游戏动漫
体育,军事
娱乐
美食,娱乐,新闻资讯,时尚,教育就业,投资理财
军事,新闻资讯
IT 数码
娱乐,美女,时尚,旅游,IT 数码,体育
美食
旅游,健康,时尚
双鱼女,多愁善感
乐观
文艺
美女
IT 数码,娱乐,体育
旅游,娱乐,新闻资讯,美食
美食,名人明星,美女,新闻趣事,搞笑幽默
投资理财,养生,美容,化妆品,护肤,时尚,健康
教育就业,美食,文艺,健康,新闻资讯,体育
美女
新闻资讯,汽车,时尚,美食,IT 数码
健康,汽车,美食,游戏动漫,IT 数码
教育就业,文艺,娱乐,旅游,星座命理
体育,IT 数码,时尚,娱乐,美食,美女,投资理财,健康
文艺,旅游
哈尔滨吃喝玩乐,哈尔滨美食,团购网,团购,大众点评网,健康,体育,美食
健康
健康
旅游,娱乐
学生一枚,爱情
美食,外贸童装批发,零售
娱乐,林宥嘉 YogaLin,书法,街拍,PhotoShop,宅女一枚,时尚,旅游,星座命理
旅游,美食
军事,美食
美食,健康,旅游

娱乐
浙江生活,汽车
美食
时尚
美女,时尚,体育,旅游
体育,美食
游戏动漫,娱乐,投资理财,旅游,健康,美食,文艺
美食,文艺,健康,旅游,交友,旅游美食,旅游摄影,孩子气,浪漫
投资理财,财务,旅游,轻音乐,抒情歌曲,美食
游戏动漫,星座命理
IT 数码,矛盾,收藏,看书
娱乐
游戏动漫,美女,星座命理,美食,IT 数码
搞笑幽默,微博奇葩,低调求生存
新闻资讯,美女,教育就业,文艺,投资理财
汽车,文艺
游戏
旅游,美食,文艺
服装,娱乐,IT 数码
娱乐
游戏动漫
旅游
名人明星
美女,新闻趣事,江苏生活
游戏动漫,文艺,旅游
娱乐
新闻趣事,搞笑幽默,微博奇葩
美女,时尚,旅游,健康,体育,新闻资讯,投资理财,美食,汽车,IT 数码
军事,数码
体育,健康,投资理财
爱生活,爱玩
美食
宅,自由,学生,驴友,美食爱好者,双子,家居,钓鱼,看电视,军事
萌宠
娱乐,美女,IT 数码
玩～,闲着发呆
美食,旅游,娱乐,时尚,文艺
娱乐
微博奇葩
游戏动漫,手機控,釣魚,旅遊,動漫,低調
健康,投资理财,星座命理,旅游,娱乐,IT 数码
文艺
游戏动漫,美食,体育,娱乐
IT 数码,其他生活,搞笑幽默,游戏,旅游

时尚,游戏动漫
搞笑幽默
时尚,美女
星座命理
懒,吃货一枚,美食控,校园生活,星座运势
双重性格,喜欢音乐
军事
教育就业,体育
娱乐
攀枝花生活
体育
美食,游戏动漫
教育就业,健康,旅游,美食,文艺
美女,美食
旅游,游戏动漫
福建生活,搞笑幽默,健康,旅游,文艺,体育
时尚,星座命理,投资理财,美食,文艺,教育就业,游戏动漫,娱乐
美食
健康,新闻资讯,旅游,美食,娱乐
篮球,娱乐,军事,汽车
娱乐
育儿百科,文艺,体育,娱乐
新闻资讯,游戏动漫,教育就业,IT 数码
美女,新闻趣事,搞笑幽默,视频音乐,其他生活
星座命理,游戏动漫,时尚,IT 数码,汽车
文艺,IT 数码,汽车,美食,娱乐
文艺
军事
投资理财,游戏动漫
健康,体育,娱乐
游戏动漫
旅游,海绵宝宝,感性,简单,强迫症,双重性格,双鱼女,随性,收藏,看电影
游戏动漫,教育就业,美食
游戏动漫,教育就业,星座命理
健康
其他生活
新闻资讯,时尚,美食,体育
美食,旅游,健康
时尚,星座命理,文艺
搞笑幽默,体育
时尚,文艺,体育
旅游,汽车
校园生活,视频音乐,十堰生活,搞笑幽默,美女
情感生活,旅游

娱乐
美食
投资理财,军事
上海大众,投资理财,美女,IT 数码,汽车
IT 数码,读书,傻,钓鱼
军事,旅游,娱乐,体育
微博奇葩,搞笑幽默
名人明星,娱乐
美女,IT 数码,新闻资讯,旅游,体育
娱乐,时尚,军事,游戏动漫
娱乐,减肥 ing,天平座,微博,星座命理,新闻资讯,游戏动漫,体育,时尚
喜欢听音乐
情绪控
美食,游戏动漫,文艺,娱乐,旅游
IT 数码,娱乐,美女,游戏动漫,教育就业
游戏动漫,投资理财,旅游
美女,美食,文艺
视频音乐,体育资讯,搞笑幽默
搞笑幽默,动漫控,粤语,90 后非脑残,吃喝玩乐,猫,日语,b 型血,浪漫,游戏动漫
旅游,健康,时尚,娱乐
视频音乐,清远生活,旅游,美女,新闻趣事
军事,美食,星座命理,健康
教育就业,游戏动漫
游戏动漫,娱乐
胡思乱想,摩羯,90 后非脑残,k 歌,玩,懒,搞笑,吃货,文艺,旅游
健康,旅游,文艺
美食
文艺
文艺,游戏动漫
美食,文艺,娱乐
健康,新闻资讯,娱乐
娱乐,体育,吃,旅游,音乐,电影,篮球和高尔夫,唱歌,电影控
八卦杂谈,IT 数码,体育资讯,美图摄影,搞笑幽默
体育
学生一枚,爱笑,爱电影,游戏动漫
健康
旅游,新闻资讯,健康,美女,时尚
时尚,游戏动漫
文艺
投资理财,军事,新闻资讯,健康,星座命理
旅游,美食,娱乐
时尚,美食,健康,汽车,文艺,娱乐,星座命理,旅游
旅游
萌宠,健康

涂鸦,笔亲,话剧,水瓶女,浪漫,时事,房产,疯狂,美食,健康
美食,文艺
旅游,新闻资讯
汽车
主持
旅游
投资理财,娱乐,军事,新闻资讯
军事,汽车,游戏动漫
IT 数码,教育就业,旅游,军事,文艺
旅游,美食,电影,游戏,汽车
军事,美食,星座命理,旅游
游戏动漫
美女,体育
穿衣美容
娱乐
冠希范儿。
体育资讯,车世界,视频音乐,新闻趣事,搞笑幽默
时尚,汽车,旅游,美食,娱乐
汽车,美女
投资理财,健康,汽车,新闻资讯,游戏动漫,时尚,文艺,娱乐
美食
微博奇葩
我就是我,爱睡觉,写字,学生一族,90
体育,娱乐
广东生活
军事,户外,设计师,建筑,汽车,美女,旅游,IT 数码,文艺
视频音乐
时尚,爱唱歌,k 歌,80 後,天秤
娱乐
广东生活
娱乐,美食
旅游,游戏动漫
汽车
健康,新闻资讯,时尚,投资理财,IT 数码
体育
美食,旅游,娱乐
教育就业,美食,新闻资讯,时尚,星座命理,文艺,娱乐
新闻趣事
游戏动漫,新闻资讯,美食,健康,美女,时尚,IT 数码
美食,搞笑幽默,美女,新闻趣事,娱乐
旅游,体育
健康,旅游,美食
美女,教育就业,IT 数码
IT 数码,娱乐,健康,旅游,美食

健康,投资理财,军事,娱乐,教育就业
读书分享,新闻趣事,湖北生活,美图摄影,旅游
名人明星
名人明星
新闻资讯,美食,文艺
健康,美食,娱乐
游戏动漫,教育就业,投资理财
新闻资讯,汽车,体育
美食,旅游,游戏动漫,南派三叔,黑执事塞夏党,盗墓笔记瓶邪党,死神,动漫
好性格,天秤,护士,双鱼,宅,看小说,睡懒觉,幻想,网络,青春
搞笑幽默
汽车,娱乐,文艺,美食,游戏动漫,健康,旅游,美食
名人明星,爱旅游,双子
相声,新闻资讯,美食,军事,娱乐,汽车
体育
健康,军事,体育
健康,时尚,星座命理,旅游,文艺
旅游,时尚
校园生活
时尚,旅游,游戏动漫,美女
旅游,汽车,体育,娱乐
体育,美女,旅游,星座命理
时尚,娱乐
旅游
旅游
娱乐,时尚
游戏动漫
星座命理,美食,游戏动漫,文艺,娱乐
健康,旅游,美食
时尚,汽车,游戏动漫,IT 数码,娱乐
旅游
体育
时尚,旅游,IT 数码
汽车,美食,文艺
新闻趣事,新闻资讯,文艺,体育
时尚,娱乐,美食,星座命理,旅游
新闻资讯,投资理财,IT 数码
娱乐,旅游,时尚
娱乐
体育,足球
旅游,教育就业,娱乐,投资理财,星座命理
新闻资讯,旅游,文艺,星座命理
丰香百货商店,上,东,峪,上东峪丰香百货,教育就业,汽车,健康,投资理财
音乐

投资理财,汽车,星座命理,旅游
星座命理
旅游,军事,体育
名人明星,文艺,娱乐
汽车,军事,娱乐,体育
游戏动漫,美女,汽车,时尚,IT 数码
iphone,服饰,娱乐,美女
电影,动漫,音乐,NBA
时尚,教育就业,IT 数码
美食,潮流,坚强,自然,执着,简单,时尚,娱乐
词曲创作,喜欢听歌,文艺,美食爱好者
美食,投资理财
健康,教育就业,旅游,美食,文艺,吃喝玩乐,爱自由,红酒,90,摄影爱好者
文艺,微博控
汽车,体育
房产
游戏动漫
娱乐
巨蟹,佛教,笑,爱旅游,爱电影,交朋友,相声,爱音乐,美食,公益
旅游,IT 数码,体育
健康,星座命理,美食,娱乐,体育
健康,时尚,文艺,美食,星座命理
体育资讯,教育就业,旅游,游戏动漫,体育,娱乐
新闻资讯,旅游,文艺
健康
娱乐
体育,IT 数码,军事,娱乐,投资理财,文艺
教育就业,时尚,汽车,旅游,娱乐,感性，思维简单
文艺,体育,娱乐
汽车,旅游,IT 数码,体育,娱乐
美食,体育,娱乐
健康,旅游,美食
美食,娱乐
穿衣美容,微博奇葩,搞笑幽默
旅游
健康,旅游,文艺,教育就业,娱乐
偶喜欢玩,互粉,无厘头,活泼开朗,爱笑,幽默,游戏,狮子座,阳光,90 後
健康,新闻资讯,投资理财,美食
星座命理,娱乐
游戏动漫,吃货,二货,宅女,小清新,逛街,文艺
汽车
寂寞 ing,减脂,健身
IT 数码
美食,体育,娱乐,新闻资讯,时尚

名人明星
穿衣美容,游戏动漫
游戏动漫,汽车,IT 数码
IT 数码
健康,体育,娱乐
新闻资讯,美女,旅游,IT 数码
IT 数码
时尚,IT 数码
电视迷
健康,新闻资讯,美食
娱乐
萌宠,投资理财,教育就业,新闻资讯,健康
美女,时尚
投资理财,时尚,游戏动漫,旅游,美食
教育就业,投资理财,时尚,汽车,娱乐
IT 数码,文艺
名人明星
旅游,waiting
旅游,美食,文艺
爱吃,娱乐,文艺
龙猫,宫崎骏,DIY,嗜睡,起床困难户,学生一枚,看小说,爱音乐,打篮球,看电影
星座命理
旅游,军事,IT 数码
投资理财,星座命理,体育,时尚
美女
健康,投资理财,IT 数码,旅游,军事
娱乐
娱乐,美食,时尚,健康,星座命理
旅游
视频音乐,开封,两岸早教,育婴,早教
文艺
教育就业,星座命理,健康,新闻资讯,投资理财
上海生活
娱乐
购物狂,网购,淘宝,女装,韩版
萌宠
体育
美食,新闻趣事,阳江生活,旅游
美女,健康,时尚,美食,娱乐
美剧迷,囧
体育,汽车,军事
幻想,吃货,写作,阳光,任性,爱睡觉,双重性格,双子,小说
搞笑幽默,微博奇葩,IT 数码
美食,北京生活

娱乐
卡卡,赚钱,资讯,任性
体育
旅游,时尚,汽车,娱乐,体育
健康,旅游,教育就业,投资理财,娱乐
IT 数码
爱美衣
美食
游戏动漫,看电影,火影忍者,宅男,NBA,音樂,打篮球,网购,游戏,狮子座
娱乐
娱乐
新闻资讯,健康,投资理财,汽车,旅游,IT 数码
旅游
美女,投资理财,军事
美食
军事,文艺,教育就业,汽车,新闻资讯,美女,时尚
IT 数码
名人明星,投资理财,IT 数码,时尚,体育,娱乐
军事,文艺,投资理财,爱电影,时尚达人
纠结,淡定,冷笑话,聊天,科幻,无聊
旅游,美女,时尚,汽车,娱乐
教育就业,美食,娱乐,文艺,时尚
美食
情感生活,福州生活,美容服饰,旅游,美食,教育就业,健康,体育
体育,IT 数码,美女,游戏动漫,娱乐
大学
美女,旅游,娱乐,投资理财,时尚
美食,时尚,星座命理
时尚,美食,星座命理,旅游,娱乐
新闻资讯,教育就业,文艺,旅游,美食
投资理财,教育就业,健康,军事,IT 数码
文史,收藏鉴赏,推理悬疑,计算机,模式识别,数据挖掘,人工智能,机器学习,体育,美食
90 后
漫画党,罗宾,杰森,DC,美漫
搞笑幽默
新闻资讯,投资理财
旅游
美食,娱乐
IT 数码,娱乐,文艺,健康,投资理财
新闻资讯,时尚,娱乐,星座命理
星座命理,健康,新闻资讯,教育就业,文艺,娱乐
喜欢唱歌,电视剧,美食,军事,体育,娱乐
美食,汽车,IT 数码,美女,娱乐
朋友,女人,美女,IT 数码,健康,旅游,星座命理

娱乐,吃,睡懒觉,双子座,90 後
美食,投资理财,旅游
时尚,教育就业,汽车,投资理财,娱乐
投资理财,美女,新闻资讯,文艺,体育,IT 数码
时尚,文艺,娱乐,投资理财
旅游,文艺,娱乐,水瓶座,搞笑,愛音樂，愛唱歌
IT 数码,文艺
美食,神经大条,爱情,金牛座,创业,开朗,文艺,发呆,星座
健康,新闻资讯,美女,时尚
教育就业,娱乐,新闻资讯,时尚,文艺
汽车,投资理财,IT 数码,时尚,星座命理,旅游,健康
汽车,娱乐,美女,潮人
旅游,健康,时尚
娱乐,体育,昆汀塔伦蒂诺,克里斯托弗诺兰,电影原声配乐,尼古拉斯凯奇,汤姆克鲁斯,美粉,
美剧迷,电影迷
美食,美女
美食
汽车,文艺
星座命理
美食,旅游,时尚
军事,投资理财,教育就业,旅游
文艺,健康,体育,旅游,教育就业
军事,文艺,新闻资讯,IT 数码
标准懒人一枚,疯狂,腹黑,随性,漫画,高中生,服饰,历史,听音乐,摄影控
军事,体育,游戏动漫
娱乐,星座命理
新闻资讯,汽车,旅游,IT 数码,体育
健康,旅游,美食,娱乐,教育就业
美女,投资理财,健康,IT 数码,体育,90 后非脑残
健康,时尚,文艺,旅游,美食,娱乐
music
星座命理,军事
娱乐
时尚,文艺,美食,健康,娱乐,汽车,旅游
美食
美女
健康,新闻资讯,时尚,教育就业,旅游
投资理财,游戏动漫,新闻资讯,星座命理,军事,文艺
香车,旅游,时尚,美食,穿衣美容
游戏动漫
IT 数码,美食,体育,娱乐,美女
爱唱歌爱音乐
时尚
娱乐

旅游,文艺,健康,汽车,游戏动漫
體育
旅游,时尚,汽车,美食
娱乐,美食,体育
天然呆自然萌,90 前,天蝎座,杰伦控
体育
爱幻想,拍照,单纯,水瓶女,好性格,多愁善感,旅游美食爱好者,爱旅游,星座命理,时尚
金牛,成都生活,欧美音乐,90 后非脑残,吃喝玩乐,动漫,旅游,文艺,娱乐
IT 数码,时尚,旅游,汽车,体育
时尚,娱乐,美食,旅游,体育,投资理财,新闻资讯,健康
汽车,旅游,美食
娱乐
旅游
时尚,旅游,美食
视频音乐,娱乐
水瓶,爱旅行,90 後,爱睡觉,爱生活,购物,看电影,爱自由,爱音乐,美容护肤
健康,文艺
文艺,体育,健康,美食,旅游
文艺,星座命理,旅游,美食,娱乐,体育
美女,体育
美食
动漫迷,看小说,music,篮球爱好者,强迫症,天蝎,DOTA,游戏动漫,汽车,体育
时尚,旅游,星座命理,娱乐,美食
美女
美食
旅游,星座命理,美食,健康
经济,旅游
搞笑幽默,时尚,健康,星座命理,美食,娱乐
美食,美食
懒,表演,娱乐,美食,旅游,体育
情感生活,旅游,美容服饰,搞笑幽默,美食
旅游,萌宠,星座命理,搞笑幽默,湖南生活
萌宠
游戏动漫,美食,旅游,文艺,体育
星座命理,教育就业,娱乐,时尚,文艺,新闻资讯
美食,时尚
视频音乐
娱乐,美女,游戏动漫,IT 数码,汽车
新闻资讯,游戏动漫,美女
时尚
体育,健康
宅,旅游,健康,80 后的尾巴,懒
美食
金牛座,股票,猫咪

教育就业,汽车
汽车
游戏动漫,美女
冷笑话,寒假求一工作
投资理财
娱乐,IT 数码,健康,旅游,美食
娱乐,自由,睡觉,茶
校园生活,完美主义,狮子座,好性格,淘宝,驴友,交朋友
耽美,夜猫子
时尚,投资理财
娱乐
文艺
性感,美女,天蝎
甜美,美妆,护肤,艾格,淘宝,美丽说,旅游,美食,娱乐,时尚
甘肃生活,搞笑幽默
投资理财
创业,爱情,投资理财,娱乐,旅游,运动,巨蟹座,影视,旅游爱好者
美食
汽车
娱乐,IT 数码
旅游,文艺,时尚
连云港生活
文艺
微博奇葩,星座命理,搞笑幽默,萌宠
新闻资讯,旅游,汽车,娱乐
游戏动漫,汽车,体育,IT 数码,伪球迷,数码控,摇滚控
美食,美女,体育
健康,时尚,文艺,新闻资讯,IT 数码
时尚,投资理财,IT 数码
新闻资讯,美女,美食
红酒文化,葡萄酒,红酒批发,红酒,美食
惠州生活,健康
穿衣美容,娱乐
爱笑,爱幻想,电影控,韩剧,睡觉,学生,shopping,天枰座,宅女一枚,双重性格
学生,篮球,汽车,旅游,体育
军事,文艺,新闻资讯,旅游,体育
星座命理,旅游,美食,美女,娱乐
军事,体育,娱乐
新闻资讯
吃喝玩乐,爱自由,体育
名人明星,娱乐
娱乐,文艺
军事
北京生活,星座,星座塔罗迷,星座达人,星座控,美女

新闻资讯,投资理财,健康,美食,娱乐
旅游,体育,文艺,新闻资讯,健康
美女
双鱼座,90,文艺
美食,时尚,星座命理,文艺,娱乐
娱乐,文艺
娱乐,星座命理
焦作生活,猫,强迫症,咖啡,90后非脑残,天平座,喜欢旅游
健康,文艺
汽车,体育
星座命理,旅游,美食
旅游
美食,旅游,体育,汽车,美食,美女
苏州生活,体育
星座命理,健康,旅游,IT数码,投资理财,体育
星座命理,旅游,投资理财,文艺
完美主义,孩子气,文字控
新闻资讯
健康
旅游,美食,文艺,体育,娱乐
健康,旅游,美食,时尚,娱乐
娱乐,星座命理,美食
学生一枚,热爱生活,自由,努力,水瓶
健康,汽车,游戏动漫,美女,军事,许嵩
K歌,旅游
视频音乐,娱乐,文艺,体育,游戏动漫
游戏动漫
健康,娱乐,美女,时尚,美食
娱乐
时尚,旅游
娱乐
改装,方向盘改装,壹工廠,汽车改装爱好者,汽车改装,oneworks
美食,游戏动漫,体育,投资理财,IT数码,文艺
穿衣美容,文艺,时尚,汽车,安静,学生
军事,投资理财,游戏动漫
美女
美女
星座命理
陈翔,双子座,偶稀饭睡觉觉,许嵩,学生,偶喜欢玩,活泼开朗,健康,文艺,娱乐
美食
体育,娱乐,游戏动漫
天蝎女
健康,新闻资讯
美女,健康,旅游,美食,IT数码

文艺
娱乐
美女,娱乐,星座命理,旅游,时尚
其他生活
媒体人,新闻资讯,记者,互联网,IT 互联网,律师,公务员,军事,IT 数码
美女
旅游,美食,IT 数码,时尚
时尚
娱乐
投资理财,美食,旅游,IT 数码,娱乐
IT 数码
旅游,宅女猪猪一只,做梦,爱睡觉,学生一枚,乐天派,听音乐,吃货一枚,90 後
旅游,文艺
新闻趣事,音乐迷,汽车
体育
腹黑，懒
八卦杂谈
美女,文艺,投资理财
新闻资讯
美女,旅游,体育
娱乐,美食,文艺,教育就业,健康,投资理财
文艺,音樂人
军事
娱乐
娱乐
娱乐,体育
新闻资讯,文艺
美女,体育
美食
旅游,唱唱唱,偶像剧
读书分享
新闻趣事
文艺,健康,IT 数码,教育就业,投资理财,HAPPY
美食
美食,旅游,搞笑幽默,视频音乐
美女,旅游,时尚,美食,健康
篮球裁判,体育
美女,重感情
萌宠,游戏动漫,k 歌,海绵宝宝,娱乐,文艺
美图摄影,美食,视频音乐,搞笑幽默,校园生活
名人明星,文艺,体育,娱乐
名人明星,娱乐,游戏动漫
健康,时尚,旅游,美食,娱乐
IT 数码

微博奇葩,旅游,体育,娱乐
喜欢听歌,吃货一枚,懒,爱笑,星座,听音乐,聊天,音乐达人,吃货,文艺
投资理财,教育就业,汽车,军事,IT 数码
健康,军事,美食,旅游,汽车
健康,纠结的矛盾体,快乐,神经大条,朋友,网购,水瓶座
时尚,娱乐,旅游,新闻资讯,健康,文艺
新闻资讯,游戏动漫,教育就业
文艺,IT 数码,军事,美食,美女
娱乐,军事,健康,新闻资讯,文艺,教育就业,美食,旅游
水瓶女,单纯,康熙来了,吸血鬼日记,美剧,喜剧,IT 数码,郑允浩
穿衣美容,旅游,美食,视频音乐,90 后,爱睡觉的懒妞
时尚,美食,文艺
娱乐
热爱生活,睡懒觉,爱吃,喜欢旅游,IT 数码
体育资讯,衢州生活,体育,射手
新闻资讯,体育,文艺,健康,教育就业,汽车,军事,星座命理
体育,文艺,游戏动漫,军事,美食
广东生活,时尚,投资理财
教育就业,娱乐,星座命理,游戏动漫
娱乐
旅游,美食
时尚,星座命理,美食,旅游
娱乐
小女人
时尚,新闻资讯,IT 数码,娱乐,文艺,投资理财,旅游,美食,教育就业
健康,投资理财,IT 数码,时尚,娱乐
美女,新闻资讯,健康,美食,文艺
娱乐
美女,美食,投资理财,健康,星座命理,娱乐,文艺
时尚,娱乐,美食
科学探索,新闻趣事,视频音乐,八卦杂谈,深圳生活,四会在线,袖珍网,一切我帮你,一条龙服务,时尚
娱乐
美食,娱乐
投资理财,旅游,美食,娱乐
文艺
90 後,K 歌,IT 数码,美食,旅游,汽车,娱乐
疯子,音乐发烧友,巧克力,狮子座,吃货
旅游
搞笑幽默,旅游,美食,时尚
IT 数码
时尚,投资理财,旅游,星座命理,美食
时尚,美女,星座命理,娱乐
读书分享,校园生活,潮州生活,穿衣美容,新闻趣事,星座命理,旅游

娱乐
军事,美食,汽车
游戏动漫
旅游,美食,娱乐
游戏动漫
时尚,汽车,娱乐,星座命理,旅游
旅游,美食,文艺
汽车
游戏动漫
美食,美女,新闻趣事,山东生活,搞笑幽默
体育
投资理财
娱乐
中介,厦门写字楼资讯,厦门写字楼租赁
星座命理,美食,美女,时尚,娱乐
星座命理,汽车,游戏动漫,旅游,娱乐
娱乐
娱乐,时尚,IT 数码
汽车,投资理财,教育就业,美食
新闻资讯,旅游,IT 数码,美食,投资理财
学生一族,shopping,喜欢听歌,睡懒觉,布丁
ALEX,小说迷,努力
健康,娱乐
军事
长沙生活,旅游摄影,梦想,青春,旅游美食,娱乐,美女,新闻资讯,健康,旅游
旅游,新闻资讯,美女,汽车,时尚
新闻资讯,健康,教育就业,娱乐,文艺
搞笑幽默
汽车
游戏动漫
新闻资讯,游戏动漫,IT 数码
新闻资讯,旅游,IT 数码,美女,游戏动漫,汽车,娱乐
美食,娱乐,文艺
动漫,学生,睡觉,上网,美食,星座命理,娱乐
文艺,高三
健康
美食,文艺,旅游,健康,游戏动漫,美食,IT 数码
旅游,健康,文艺
军事
娱乐,IT 数码,汽车,体育
记者
星座命理,体育,娱乐
娱乐,美女
心灵智慧,成都,古典艺术,文艺,健康,新闻资讯,旅游,美食

旅游,美食,微博奇葩,穿衣美容
健康,美食,旅游,娱乐
旅游,美食
汽车,旅游,星座命理,军事,体育,游戏动漫
IT 数码,物流,会计,电商,营销,管理,合工大
新闻资讯
美女,新闻资讯
文艺
汽车
萌宠
娱乐
健康,美食,IT 数码
美食
旅游,文艺,时尚
投资理财,娱乐,旅游,文艺
穿衣美容
苏州生活,IT 数码
文艺
文艺
文艺,教育就业,旅游
搞笑幽默,体育
美食,旅游,健康,IT 数码,美女
新闻趣事,微博奇葩,搞笑幽默
美食,娱乐,时尚,健康,旅游
娱乐,文艺
美女
美食
原宿,旅游
文艺
吃货,幽默,交友,看书,摄影,游戏动漫,旅游,美食,文艺,娱乐
新闻资讯,IT 数码,配图,GIS,天地图,数字城市,共享,运维管理,ArcGIS,OneMap
娱乐
IT 数码
星座命理,娱乐
美女
投资理财
星座命理
体育
游戏动漫,娱乐,星座命理
游戏动漫,娱乐,体育
IT 数码,美食,健康,时尚,娱乐
简单,音乐控,强迫症,同志,娱乐,水瓶疯子,手机控,小清新,月光族
教育就业,投资理财,健康,IT 数码,文艺,体育
IT 数码,美食,新闻资讯

娱乐
投资理财,时尚,旅游,美食,星座命理
投资理财
健康,新闻资讯,投资理财,教育就业,文艺
体育,游戏动漫,娱乐,美女,时尚,星座命理
娱乐
体育,娱乐,越野,恐怖片,Hyde,Radiohead,Muse,Jeep
美食,娱乐,文艺
新闻资讯,美女,体育
偶稀饭睡觉觉,大学,蓝色,90,汽车
散步,神经质,双子座,笔亲,喜欢唱歌
IT 数码,健康,娱乐,美女,体育
萌宠,美女,搞笑幽默
时尚
名人明星,新闻趣事,八卦杂谈,体育资讯
求职招聘,招聘,新闻资讯,教育就业,IT 数码,美食
娱乐
军事,投资理财,旅游,星座命理
旅游
美食
汽车,新闻资讯,时尚,文艺
新闻资讯
搞笑幽默
投资理财,娱乐,旅游,美食,时尚
时尚,游戏动漫
健康
健康,投资理财,美食,旅游,游戏动漫,时尚
星座命理,新闻资讯,游戏动漫,美食,体育,开朗,乐观,喜欢打羽毛球
健康
美食,旅游
娱乐
美女
星座命理
文艺,教育就业,旅游,美食,娱乐
相信自己和永恒,新闻资讯,健康,旅游,娱乐,军事
健康,美食
旅游,新闻资讯,游戏动漫
美女,投资理财,时尚,文艺,体育
搞笑幽默,美女
新闻趣事,汽车,军事,IT 数码,文艺,新闻资讯
自由,执着,天蝎,爬山,五月天,爱睡觉,music,心理,拍照,书
签名控,爱情,天猫,淘宝控,创业,美女,健康,IT 数码,娱乐,时尚
娱乐,健康
旅游,投资理财,时尚,星座命理,娱乐

军事,娱乐,新闻资讯,旅游,汽车,IT 数码,文艺
星座命理,旅游
娱乐,星座命理
安徽校媒
兰州生活,投资理财,体育,汽车
美女,汽车,IT 数码,娱乐,文艺
美女
娱乐
健康
听歌,数码,电影,澄海 3C
美食,美女,快乐,爱笑,美食控
搞笑幽默
军事,投资理财,旅游,新闻资讯,体育
音乐控,公益,公道，正义，,新闻,浪漫
娱乐
美女,旅游,美食,文艺,娱乐
游戏动漫,美女,娱乐,体育,时尚
搞笑幽默,视频音乐,教育就业,旅游,IT 数码,体育,娱乐
爱幻想,篮球,美食,武汉生活,搞笑幽默
军事
文艺,娱乐,美女,星座命理,时尚
名人明星
娱乐
新闻资讯,投资理财,健康,IT 数码,文艺
时尚,体育,文艺,娱乐
宅女一枚,娱乐,健康
旅游,时尚,IT 数码,文艺,游戏动漫
汽车,健康,娱乐,时尚
美食,星座命理,娱乐
旅游
游戏动漫,新闻资讯,健康,军事,娱乐
汽车,新闻资讯,旅游
娱乐
时尚,游戏动漫,星座命理,美食,娱乐
IT 数码,教育就业,投资理财
时尚,IT 数码,汽车,旅游,娱乐
广东生活,穿衣美容,健康,美女,时尚,娱乐,乐观,交友,护肤美容,减肥丰胸
多愁善感
体育
健康,时尚,投资理财,美食,娱乐
时尚,新闻资讯,星座命理,美食,文艺,娱乐
娱乐,旅游,时尚,健康,美食,淡定
汽车
文艺,时尚,健康,美食,旅游

IT 数码,汽车,新闻资讯,健康,旅游
美女
娱乐
文艺,游戏动漫,星座命理,娱乐
游戏动漫
旅游,Music,Android 控,双子座 AB 型,双子座,音乐控,文字控,Android
美食,娱乐,星座命理,体育,文艺
时尚,汽车
文艺,美食
娱乐,体育
游戏动漫
校园生活
旅游,美食,汽车,娱乐,IT 数码
时尚,星座命理,旅游,美食,美女
娱乐,旅游,体育
美食,北京生活,旅游,游戏动漫
新闻资讯,星座命理,美食,旅游
护鲸大战第四季,国足,体育
名人明星,微博奇葩
军事,IT 数码,文艺,体育,娱乐
美女,时尚,娱乐
游戏动漫,健康,星座命理,旅游,美食
旅游,名人明星,健康,星座命理,美食,文艺,娱乐
游戏动漫,旅游,娱乐,文艺,教育就业
时尚
星座命理,美食,文艺
旅游
美食,搞笑幽默,萌宠,微博奇葩,星座命理
看书,宅,自由
打球,原创
美女,投资理财,健康,新闻资讯,美食
发呆,小资,游泳,美容,看电影,星座,旅游,IT 数码,时尚,娱乐
美食,IT 数码,文艺,体育,娱乐,美女,时尚
纠结,高中生,看电影,逛街,腐女,可爱,爱睡觉,爱生活,坚强,爱旅行
娱乐
美女
IT 数码,军事,旅游,体育,汽车
旅游,健康,美食,星座命理
旅游,新闻资讯,星座命理
厦门印象,媒体创意人,闽台合作,台湾周播报
IT 数码,汽车,教育就业,娱乐,时尚,游戏动漫
军事
美女
美女

娱乐,奋斗,努力,狗狗,健康,投资理财,汽车,军事,美食
美女
爱幻想,看电影,逛街
旅游
电影,音乐,旅游,爱情
发呆,游戏动漫,娱乐
娱乐
文艺,旅游,星座命理
时尚,美女,娱乐,体育,健康
娱乐
时尚,星座命理
新闻资讯,旅游,体育
新闻资讯,美女,教育就业,美食,汽车
投资理财,娱乐,时尚,美女,IT 数码
美食,旅游,美女,娱乐,时尚
美食
娱乐,文艺,美食
安倍晋三,张召忠,宋晓军,对台军售,钓鱼岛,时尚,新闻资讯,健康,军事,娱乐
美女,游戏动漫
健康,体育,娱乐
爱音乐
文艺,体育,娱乐
教育就业,娱乐
IT 数码
文艺,体育,娱乐
健康,新闻资讯,娱乐
八卦杂谈
星座命理,游戏动漫,娱乐
河北生活,美女,旅游,搞笑幽默,微博奇葩
旅游,美食,看电影,爱音乐,听音乐,海贼王,游戏动漫
穿衣美容
文艺,体育,娱乐
文艺,体育,娱乐
旅游,时尚,体育
投资理财
投资理财,新闻资讯,时尚,星座命理,文艺
名人明星
体育,IT 数码
时尚,游戏动漫,美女,新闻资讯,健康
美女,健康,旅游,文艺,体育
美食
美食
游戏动漫,教育就业,军事
新闻资讯,美女,游戏动漫

健康,体育,娱乐
music
時尚
健康,新闻资讯,娱乐
文艺,体育,娱乐
体育资讯,连接服务器失败,体育
美食,新闻资讯,时尚,星座命理,娱乐
看小说,欧美控,无聊,美剧,军事,平常心,大学生
宅女加腐女,宅女一枚,收听会死么?,左溢,90後
广东生活,微博奇葩,视频音乐,旅游,美食,娱乐
投资理财,娱乐,体育,文艺,军事
高三,怀旧,追星,可爱,爱睡觉,帅哥,美容,汽车,军事,体育
微博奇葩,视频音乐,新闻趣事,搞笑幽默,河北生活
英语,老师,写字,好书,心理,写作,学生一族,完美主义,翻译,读书
美食,健康
健康,新闻资讯,体育
娱乐,文艺,新闻资讯,美女,游戏动漫
文艺,体育,娱乐
旅游
美女
时尚,旅游,星座命理
美食,吉他,化妆,购物狂,美容,旅游,美食,健康,娱乐,星座命理
汽车
美食
旅游
动漫迷,爱唱歌,CF,白羊座,jay迷,足球迷
时尚
汽车
健康,旅游
美女,新闻资讯,旅游,健康,时尚
体育
教育就业,汽车,文艺,体育,娱乐
微博奇葩,听歌呢
IT数码
唱歌,自由,旅行
宠物图集
投资理财,新闻资讯,时尚,健康,美食
投资理财,IT数码,旅游,美女,娱乐
美女,美食,旅游,搞笑幽默,微博奇葩,投资理财,新闻资讯,健康,军事,体育
美女,汽车,旅游,娱乐,文艺
健康
健康,旅游,星座命理,娱乐
自己要空杯,学习
80後,书,自恋,双重性格,足球,工作,游戏,宅,执着,旅游

汽车,新闻资讯
宠物,学生一族,减肥,美剧迷,吃货
娱乐,星座命理,美食,时尚
IT 数码,文艺,体育,娱乐
时尚,娱乐,旅游,美食,IT 数码
旅游
旅游,投资理财,时尚,娱乐
文艺,健康,时尚,美食,娱乐
美女,投资理财
IT 数码,游戏动漫
美女,文艺
游戏动漫,美食,旅游
美女,游戏动漫,时尚
活泼
时尚
汽车,旅游,星座命理,美食,军事
体育,新闻资讯
美食,制服控,黑塔利亚
旅游,新闻趣事,美女,教育就业,游戏动漫,新闻资讯,美食,娱乐
健康
宠物图集
美女
真诚,iphone,哈哈,情感,傻,坚强,听音乐,K 歌,美食爱好者,热爱生活
美食,军事
文艺,体育
美容服饰
大大咧咧
新闻资讯,健康,时尚,教育就业,投资理财
美食,旅游,时尚,娱乐
美食
旅游,投资理财,教育就业,新闻资讯,文艺
游戏动漫,美女,军事,IT 数码
IT 数码,旅游
江苏生活
投资理财,美食,文艺
90 后,男生,帅气,单身,没房,没车
旅游
文艺,体育,娱乐
文艺,时尚,旅游,美食,娱乐
健康,旅游,美食,文艺
穿衣美容
搞笑幽默,娱乐
游戏动漫
文艺,体育,娱乐

旅游,军事
美女,IT 数码
时尚,交友,唱歌,娱乐
甘肃生活,文艺,教育就业,娱乐
新闻资讯,星座命理,IT 数码,文艺,娱乐
美食
旅游
旅游,汽车,体育
美食
美食,IT 数码,体育
娱乐
美女,娱乐
工地男
IT 数码,学生,游戏狂
新闻资讯,娱乐,IT 数码,体育,旅游
汽车,娱乐
星座命理,娱乐,时尚
搞笑幽默,文艺,星座命理,娱乐
名人明星,旅游,美食
娱乐,美食,教育就业,文艺,军事,旅游,IT 数码
投资理财,娱乐,星座命理,时尚,健康
投资理财,军事,新闻资讯,美食,游戏动漫,体育
新闻资讯,星座命理,健康,时尚,投资理财
SJ,金秀贤,JYJ 金在中,SHINee 崔珉豪,天蝎女
爱自由
健康,新闻资讯,文艺,美食
美女
教育就业,健康,旅游,娱乐,美食
娱乐
时尚,文艺,感性,爱玩,聊天
旅游
旅游,双子座,80 后,风衣控,喜欢下厨,泰勒斯威夫特,明朝那些事儿,帆布鞋控,康熙来了,星座命理
时尚,美食,美女,投资理财,军事,健康,旅游,IT 数码
新闻趣事,搞笑幽默,穿衣美容,时尚,健康,旅游,文艺,美食
旅游,游戏动漫,汽车,体育
汽车
健康,体育,娱乐
名人明星
美图摄影
健康,旅游,星座命理,美食,娱乐
娱乐,旅游
穿衣美容,新闻趣事,微博奇葩,搞笑幽默,视频音乐
游戏动漫,文艺,体育

旅游
文艺,体育,娱乐
学生,金牛座
时尚,军事,娱乐
游戏动漫
旅游,健康
吃货,superjunior
美食
健康,旅游,美食
星座运势,星座命理,新闻资讯,投资理财
星座命理
投资理财,新闻资讯,文艺,美食,旅游
汽车,体育,游戏动漫
新闻资讯,军事,IT 数码,文艺,体育
健康
文艺,IT 数码
健康,新闻资讯,教育就业,投资理财,军事
体育,时尚,文艺,娱乐
星座命理
游戏动漫
流浪,音乐爱好者,麦霸
旅游,娱乐
旅游,名人明星,美女,视频音乐,时尚,新闻资讯,健康
喜欢唱歌,Music,O 型血,化妆品,潮人,服饰,罗志祥,美容护肤,娱乐
文艺,美食,吃货
时尚,旅游,娱乐
IT 数码,时尚,汽车
旅游,美女
旅游,美食,娱乐
体育,新闻资讯,汽车
时尚
旅遊,遊戲動漫,文藝
文艺
时尚,星座命理,美食,娱乐,文艺
旅游,健康,娱乐
美食爱好者,热爱生活,电影控,音乐爱好者,麦霸
旅游,美食,娱乐,文艺
教育,喜欢唱歌,90 后但我不脑残,电影爱好者,旅游美食爱好者,新闻资讯,时尚,健康,文艺,娱乐
投资理财,时尚,旅游,文艺
星座命理,时尚,教育就业,健康,娱乐,开朗…唱歌,
视频音乐,娱乐
美食,文艺
搞笑幽默

旅游
旅游,游戏动漫
搞笑幽默,旅游,动漫,情感生活,新闻趣事,名人明星
娱乐,健康,时尚
健康,旅游,娱乐,美食,星座命理
美女,美食,星座命理,体育,娱乐
体育,教育就业
军事
文艺,旅游,教育就业,投资理财,健康
汽车
文艺,娱乐,汽车,投资理财,新闻资讯,健康,IT 数码
时尚
游戏动漫,星座命理,文艺
健康,旅游,娱乐
军事
文艺,美食,IT 数码,娱乐
旅游
新闻趣事,美女
娱乐
汽车,美女
教育就业,IT 数码,文艺,体育,娱乐
穿衣美容
娱乐,健康
搞笑幽默,新闻趣事,旅游,美女,投资理财,教育就业
投资理财
游戏动漫
美女
时尚
教育就业,体育
游戏动漫
军事
体育
娱乐
时尚,旅游,美食,娱乐
娱乐,星座命理,IT 数码,时尚
娱乐,美食,旅游,时尚,汽车
投资理财
吃饭睡觉打豆豆
新闻资讯,娱乐,美女,军事,IT 数码,星座命理
知念侑李亲儿子,板野友美推,扣曼 T 君窝新欢,Nissy,Nero 唱见大本命,属性隐藏期,三观奇葩者,JP 双担棒粉滚粗
汽车,星座命理,美食,健康,旅游
旅游,娱乐,IT 数码,游戏动漫,美食
健康

旅游,美食,文艺,时尚,娱乐
旅游,娱乐
美女
维尼夫妇,韩剧,少女时代,大叔控,喜欢音乐,外貌协会,护肤,时尚,星座命理,娱乐
娱乐
美食
美食,旅游
音乐,自由职业
笑
教育就业,文艺,娱乐,旅游
时尚,旅游,IT 数码
新闻资讯,时尚,IT 数码
娱乐
新闻资讯,汽车,IT 数码
旅游,美食,体育
时尚,旅游,IT 数码
好书,文字
美女
看小说,茶,小清新,自由职业,新浪读书,爱旅行,多愁善感,天蝎女,美食,健康
文艺,旅游,娱乐
健康,美食,IT 数码,汽车,体育
明星,韩庚,娱乐
看小说,陈奕迅
爱猫,粉红控,爱旅游,上海,北京,爱美食,旅游
努力工作,坚强乐观,喜欢交朋友,活泼开朗,摩羯,听音乐,健康
穿衣美容,文艺,体育,娱乐
美食,美女,体育,汽车,娱乐
时尚,体育
健康,时尚,文艺,娱乐
健康,旅游,星座命理,美食
音乐，美容
旅游
新闻资讯,旅游,IT 数码
美食,体育,美女,IT 数码,汽车
文艺
健康
游戏动漫,新闻资讯
军事,娱乐
娱乐
旅游,IT 数码,时尚
游戏动漫,美食,体育,IT 数码,文艺
星座命理
旅游,星座命理,美食
新闻资讯,投资理财,娱乐,星座命理

美女
汽车
育儿百科,情感生活,宠物图集,美容服饰
体育
新闻资讯,时尚,旅游,体育,娱乐
时尚,娱乐,体育,旅游,文艺
周杰伦,娱乐,看电视,喜欢旅游,双重性格,养生,90後,个性,音乐控,台球
旅游,新闻资讯,美食
美食
健康,美女
文艺
旅游,文艺,时尚
美女,时尚,健康,旅游,星座命理
文艺,体育,娱乐
投资理财,旅游,军事
健康,文艺,体育
汽车,教育就业,文艺
旅游
健康,体育,娱乐
游戏动漫,健康
8586时尚女装,体育,娱乐
上网,睡觉,银魂,个性,蓝色,书法,喜欢唱歌,臭美,心情,文艺
新闻趣事,美食,旅游,星座命理,穿衣美容,学生一枚,书籍,爱吃,摩羯座
新闻趣事,美食,微博奇葩,搞笑幽默,名人明星
美食,旅游
美食,军事,IT数码,娱乐
听歌,电影,90后,处女座,真诚,矛盾体,旅游摄影,拍照,傻,笑
美食,旅游
美食,星座运势,美图摄影,情感生活,搞笑幽默,娱乐
旅游,时尚,新闻资讯,娱乐,自由,旅行,内向,主持人,真诚,青春
时尚,娱乐
娱乐,体育,美女,游戏动漫,美食
视频音乐,星座运势,旅游,美食,其他生活
时尚
时尚
文艺,时尚,旅游,美食,娱乐
旅游,音樂,IT数码
体育
娱乐
星座命理,娱乐
游戏动漫,美食,星座命理,文艺,娱乐
健康,新闻资讯
文艺,体育,娱乐
文艺,体育,娱乐

投资理财,旅游,星座命理
健康,军事,体育,文艺
娱乐
军事,健康,新闻资讯,时尚,旅游,娱乐
穿衣美容,新闻资讯,健康,文艺
教育就业,投资理财,文艺
美食
美食,星座命理,健康,时尚,旅游
娱乐
傻,音乐发烧友,文艺,娱乐,体育
健康,美食,旅游
美食
穿衣美容,美食,旅游,健康,时尚,娱乐
IT 数码,电子商务,民族风情,云南民族,民族饰品,民族风
康复医学科,健康
看电影,美食,文艺,旅游,星座命理
歌手,80 后
星座命理,娱乐
旅游,微博奇葩,搞笑幽默,新闻趣事,马鞍山生活
文艺,时尚,娱乐
美食,IT 数码,健康,动漫,善良,偶稀饭睡觉觉,旅游
美图摄影,八卦杂谈,搞笑幽默,校园生活,星座命理,时尚,文艺
时尚
健康
新闻资讯,时尚
旅游,美容服饰,新闻趣事,搞笑幽默,西双版纳生活
体育,娱乐
美女,时尚,汽车,体育,娱乐
旅游,健康
汽车,游戏动漫,娱乐,体育
宅男
简单,低调
搞笑幽默,娱乐
美女,星座命理,文艺,体育,娱乐
旅游,投资理财,健康,美女,军事
体育,IT 数码
青春,真诚,其他生活,新闻趣事,穿衣美容,美食,旅游
新闻资讯,教育就业,文艺
广东生活,喜欢温暖的阳光
美食
娱乐,游戏动漫
搞笑幽默
健康,娱乐,旅游,时尚
旅游,美女,游戏动漫,娱乐,IT 数码

水瓶座,易小布,星座命理,时尚,IT 数码,海绵宝宝,游戏动漫,旅游,美女,娱乐
投资理财,旅游,美食,军事,娱乐
同志,旅游,美食,体育,完美主义,真实力
投资理财,教育就业,时尚,汽车,文艺
浙江生活
游戏动漫
美食,旅游
美食
微博奇葩,迷糊,k 歌,完美主义,爱睡觉,80 後,宝宝
逛街,文艺,美食,爱自由,夜猫子,喜爱唱歌，跳舞,情绪化,爱幻想,起床困难户
90 后非脑残,恐怖片,怀旧,梦想,猫,化妆,情感,新闻资讯
教育就业
美女
旅游
美女,美食,IT 数码
文艺,体育,娱乐
教育就业,美食,文艺,体育,娱乐
动漫,校园生活,搞笑幽默,海南生活
文艺,汽车,教育就业,投资理财,时尚,军事,IT 数码,游戏动漫,旅游,健康
星座命理,文艺,娱乐,游戏动漫,教育就业,旅游,IT 数码
IT 数码,娱乐
媒体人,新闻资讯,开心,奋斗,美女,孝顺,勤快,体贴,活泼开朗,爱旅行
旅游,美食,文艺,娱乐
娱乐,体育
其他生活
美女
娱乐,游戏动漫
宅腐双修,一天一包裹,网购达人,求关心求关注,DOTA 控,科幻控,好心眼,爱音乐,淘宝,购物
投资理财,健康,体育
健康,时尚,星座命理,娱乐
遊戲動漫,汽車
游戏动漫
旅游
视频音乐
IT 数码,体育,新闻资讯,军事,旅游
美食,晋城生活
投资理财,健康,新闻资讯,教育就业,军事
美食
娱乐,旅游
健康,新闻资讯,娱乐
健康,体育,娱乐
时尚,美食,投资理财,新闻资讯
体育
视频音乐,内向小猴子。

娱乐,旅游,汽车,体育,美女
拍照,许嵩,大叔控,愤青,射手女,手机,摄影爱好者,文艺,旅游,IT 数码
搞笑幽默,游戏动漫
旅游
国标拉丁,投资理财,健康
文艺,体育,娱乐
我就是我,双子,吃,好性格,90,书籍,笑,文艺,娱乐,旅游
旅游,星座命理
IT 数码,文艺,健康,体育,娱乐
旅游
新闻资讯,体育,健康,文艺,娱乐
视频音乐,穿衣美容,新闻趣事,搞笑幽默,微博奇葩,IT 数码,军事
教育就业
爵士鼓,JYJ,TVXQ,金在中
IT 数码
时尚,健康,美食
养生,科技,金融,爱唱歌,茶,时事,古典音乐,留学,原创,星座命理
霸道的温柔,爱自由,音乐,旅游
读书分享
美食,旅游,娱乐
电影爱好者,美食,广告,旅游爱好者,旅游,军事
美食,健康,旅游,IT 数码,文艺,星座命理,投资理财,娱乐
健康,教育就业,新闻资讯,军事,投资理财
娱乐
微博奇葩,旅游,美食,萌宠,搞笑幽默
娱乐
IT 数码
星座命理,游戏动漫,文艺,军事,新闻资讯
娱乐
宅女一枚,服装,A 型血,娱乐,文艺,星座命理
时尚
旅游
旅游
星座命理,旅游
八卦杂谈,美女,搞笑幽默,游戏动漫,教育就业,文艺
旅游
fashion,美食,娱乐
旅游,娱乐,努力工作
娱乐
时尚
青春,旅游美食,宠物,宅女一枚,名人明星,微博奇葩,搞笑幽默,穿衣美容,星座命理
投资理财
新闻资讯,投资理财,教育就业,汽车,IT 数码,娱乐
IT 数码,汽车,新闻资讯,娱乐

娱乐
听歌,跳舞
微博奇葩,游戏动漫,IT 数码,娱乐
美食,时尚,旅游,娱乐
名人明星
星座命理
游戏动漫
游戏动漫,美食,体育
美女
白袜子,文艺,IT 数码,汽车,时尚
kindle,时尚,文艺,IT 数码
IT 数码
旅游,阳光,白羊座,表演
游戏动漫,火影
旅游,健康,美食,娱乐,听音乐,天秤座,吃
时尚
TDCN,ToppDogg
视频音乐,北京,双子座,法律,我就是我,吉他,k 歌,音乐控
文艺,星座命理,美食,旅游,爱幻想,狗狗,美食爱好者,娱乐,健康
汽车
娱乐
健康,美食
美食,游戏动漫,娱乐,旅游
重庆生活
星座命理,健康,旅游,美食,时尚,文艺,游戏动漫
UG,CREO,生活,网购,AUTOCAD,手板制作,PROE,mastercam,工业设计,深圳
投资理财,汽车
穿衣美容
新闻趣事,运动,情感,新闻,健康,看书
娱乐
视频音乐
微博奇葩,平常心,天枰座,好性格
羽毛球,我不是乐天派,心情,90 后但我不脑残,胡思乱想
娱乐,星座命理,健康,投资理财,乐天派,广告人,追星,音乐达人,市场营销,巧克力
搞笑幽默
军事,健康,美女,IT 数码,娱乐
健康,新闻资讯,娱乐,文艺,教育就业,体育
美食,健康,新闻资讯,时尚,星座命理
时尚,汽车,娱乐,投资理财,美女
体育,IT 数码
浪漫,健康,美食
萌宠,游戏动漫
军事,投资理财,教育就业
IT 数码,文艺,汽车,军事

汽车
军事,IT 数码,文艺,体育,娱乐
求关注,愤青,截瘫,新闻资讯,健康,旅游,星座命理
新闻资讯
文艺,新闻资讯,旅游,美食,健康,时尚
时尚,游戏动漫,文艺,美食,星座命理,旅游,娱乐
新闻资讯
旅游,AC 米兰,健康,时尚,娱乐
新闻资讯,IT 数码,游戏动漫
胡思乱想,考研,建筑,爱幻想,娱乐,文艺,投资理财,教育就业,体育
美食,娱乐
游戏动漫
时尚
摄影,旅游,投资理财
旅游,娱乐,IT 数码,健康,文艺
新聞資訊,時尚,旅遊
文艺,游戏动漫
新闻资讯,旅游,汽车
时尚,星座命理
新闻资讯
星座命理,美女,游戏动漫,体育,文艺
睡觉
时尚,教育就业,旅游,美食
星座命理,健康
游戏动漫
旅游
投资理财,旅游,娱乐,军事,IT 数码
游戏动漫
健康,新闻资讯,IT 数码,军事,旅游
娱乐
教育就业,旅游,新闻资讯,IT 数码,军事
新闻资讯,军事,文艺,娱乐
娱乐,汽车
钓鱼,周杰伦,甜瓜安东尼,克里斯保罗
新闻资讯,健康,旅游,美食,娱乐
新闻资讯,美女,娱乐,体育
娱乐
军事,时尚
星座命理
美女,穿衣美容,健康,时尚,旅游,星座命理
娱乐,美女,投资理财
健康
娱乐
新闻资讯,投资理财,教育就业,时尚,健康,文艺

乐观的小宇宙
IT 数码,新闻资讯,美女,文艺,体育,娱乐
IT 数码,健康,旅游
美食
星座命理,简单
时尚,汽车,娱乐,投资理财
教育就业,美食,娱乐,星座命理,游戏动漫,动漫,小说,电影,音乐,游戏
美女,旅游,美食,时尚
留学生,生活大爆炸,旅行,旅游,吃货一枚,德国,股市,美食控,单身,85 后
教育就业,汽车
文艺,旅游,新闻资讯
新闻趣事
美食,美女,娱乐,时尚,旅游,美食
支持韩老板,恋上鬼故事
自由职业,我就是我,理财,文艺,新闻资讯,IT 数码,旅游,美食
美食,旅游,星座命理
健康,军事
美食
美食,健康,娱乐,旅游
文艺,教育就业,时尚,旅游,健康
旅游,健康,投资理财,文艺,美食
军事,新闻资讯,时尚,投资理财
时尚,星座命理,旅游,教育就业,游戏动漫,健康,文艺
娱乐
旅游,文艺,商务礼品,工艺品,家居饰品,珠宝玉器,红木精品,陶瓷紫砂,设计师
宠物图集,娱乐
军事
文艺
教育就业,旅游,时尚,健康,美食
IT 数码
军事,旅游,健康,新闻资讯,汽车
健康,美食,娱乐,旅游
美女,美食,旅游,名人明星
体育,教育就业
旅游
性男
星座命理,美食,军事,文艺,娱乐,体育
IT 数码,娱乐
体育
娱乐,文艺,时尚,健康,旅游
星座命理,体育,娱乐
美女
IT 数码,美食
吃货,睡货

美食,时尚
萌宠,美女,名人明星,北京生活,旅游,游戏动漫,汽车,军事,体育
旅游,游戏动漫,时尚,娱乐,体育
旅游
视频音乐
旅游,时尚
瘦身,美容护肤,校园,美容,音乐,大学生,穿衣美容,化妆品,天平座
军事,旅游,IT 数码,美食,体育
美食,旅游
体育,时尚
美食
旅游
文艺
文艺
娱乐,梦里有你相共,时尚,旅游
旅游
魔蝎男,时尚
汽车,军事,IT 数码,教育就业,健康,新闻资讯,旅游,体育
娱乐,美食
游戏动漫,星座命理,军事,文艺,体育
娱乐
旅游,美食
辽宁生活,穿衣美容
旅游
美食
IT 数码,体育,娱乐,美女,旅游
旅游,美食,时尚,娱乐,健康
听歌,社会,向日葵,软件,微笑,游戏动漫,新闻资讯,教育就业,投资理财,IT 数码
时尚,娱乐
游泳,驴友,影视,打篮球,体育
游戏动漫,时尚,IT 数码
动漫迷一只,想去旅游的龙猫,吃货一枚
时尚,投资理财,健康,旅游,美食
各种货
健康,旅游,星座命理,美食,娱乐
美食,旅游,时尚,投资理财,娱乐,喜欢听歌,旅游爱好者,双子,内向，k 歌，
低调,健康,美食
美女
美食,教育就业,投资理财,新闻资讯,星座命理,IT 数码
投资理财
娱乐
欧美控,美食
美女,爱生活
时尚,旅游,文艺,IT 数码,娱乐

旅游
教育就业
桌球,美食
体育
娱乐,投资理财,建筑,防水
时尚,娱乐
健康,投资理财
汽车,游戏动漫,旅游,娱乐
微博奇葩,星座命理,穿衣美容,美女,视频音乐
搞笑幽默
娱乐,美食
时尚,健康,旅游,美食,娱乐
娱乐,美女,文艺
旅游,名人明星,八卦杂谈
娱乐,投资理财,时尚,健康,旅游
美女
时尚,美女,IT 数码,娱乐
睡觉,自由,学生,宅男,爱电影,爱笑,安静,娱乐,体育,新闻资讯
娱乐,美食,美女,旅游,时尚,健康
娱乐,交友,西安糗友,糗百
新闻资讯
名人明星,星座命理,微博奇葩,萌宠,搞笑幽默
军事,星座命理,游戏动漫,体育,娱乐
星座命理,时尚,美女,新闻资讯,娱乐
美食,娱乐,时尚
旅游
新闻资讯,美食,健康,投资理财,汽车
星座命理,旅游,文艺,美女,游戏动漫,时尚,IT 数码,娱乐
开朗幽默,跳舞,学生
美女
时尚
娱乐
文艺
旅游,美食,娱乐
体育,健康,旅游,星座命理,娱乐
音乐,家族饭,行星饭,狮子座,时尚,美食,娱乐
汽车
美食,天秤座
游戏动漫,美女
视频音乐
汽车,军事,新闻资讯,美女,IT 数码,投资理财
美女
旅游,健康,星座命理,设计,耽美,爱音乐,微笑
美容服饰,时尚,星座命理

健康, 新闻资讯, 美女, 体育, 军事
汽车, 投资理财
美女, 旅游, IT 数码
美食
蓝色, 独立, 欧美控
军事, 新闻资讯
射手女, 90 后非脑残, 爱吃, 休闲, 旅游, 美食, IT 数码, 娱乐
游戏动漫
巨蟹座, 喜欢音乐, 游戏动漫, 旅游
文艺, 游戏动漫
IT 数码, 娱乐, 旅游, 星座命理, 美食
IT 数码
书, 淘宝控, 互粉, 时尚潮流, 原创, 驴友, 自拍, 策划, 媒体人, 时尚
梦想, 传媒, 休闲, 时尚潮流, 射手座, 文艺, 美食, 星座命理, 军事, 娱乐
搞笑幽默
宅男, 网络, 手机, 新闻, 文学, 文艺, 游戏动漫, 教育就业, 投资理财, 新闻资讯
新闻趣事
娱乐, 游戏动漫
旅游, 美女, 投资理财, 美食, 娱乐
时尚, 旅游, 健康, 美食
健康, 旅游, 星座命理, 美食, 娱乐
淘宝控, 户外
体育, 娱乐
美女, 八卦, 情感, 公务员
体育
文艺, 体育, 娱乐
娱乐, 健康
美女, 投资理财
体育
星座命理, 旅游, 美食, 娱乐
娱乐
旅游, 美食, 娱乐, 投资理财, 时尚
新闻趣事, 美食, 穿衣美容, 旅游, 娱乐
汽车, 美女, 旅游, 军事, IT 数码
美食, 娱乐
新闻资讯, 投资理财, 旅游, 健康, 美食
IT 数码
文艺, 军事
时尚, 健康
美食, 娱乐, 教育就业, 军事, 体育
IT 数码, 娱乐, 游戏动漫, 体育, 新闻资讯
爱笑, 青春, 90 后, 爱美食, 喜欢听歌, 热爱生活, 旅游, 文艺, 娱乐, 美食
军事, 健康, 旅游, 星座命理, 文艺
娱乐, 时尚, 游戏动漫, 美女, 汽车, 星座命理

文艺,美女,旅游,时尚,娱乐
旅游
旅游,投资理财,体育,美食,IT 数码
旅游,视频音乐,搞笑幽默
闷骚,奋斗
新闻趣事,健康
娱乐
汽车,新闻资讯,文艺,体育,娱乐
游戏动漫,IT 数码,投资理财,美女,体育
美女
游戏动漫,教育就业,体育
美女,IT 数码
新闻资讯,时尚,体育,星座命理,健康,投资理财
90 后但我不脑残,心情,努力 ing,宅女 ing
IT 数码,新闻资讯,文艺,教育就业,旅游
时尚,游戏动漫,美女
旅游,美食,文艺,娱乐
娱乐,时尚,文艺,投资理财,旅游
美食,新闻资讯
汽车,IT 数码,娱乐
美食,教育就业,时尚,娱乐,健康
文艺
星座命理
文艺,IT 数码,健康,旅游,美食
IT 数码
萌宠,微博奇葩,美女,文艺,体育
绘画,音乐控,摄影,手工,强迫症,旅游摄影,吃货
健康,教育就业,时尚,旅游,文艺,娱乐,星座命理
狮子座,天牛,步甲,武夷山,深山,宠物,锹甲,甲虫,旅游,美食
娱乐
美女,微博奇葩,贵州生活,搞笑幽默,文艺,游戏动漫
文艺,IT 数码,军事,新闻资讯
微博奇葩
美食
健康
美食,投资理财,文艺,游戏动漫,娱乐,懒,美食控
美食
体育
军事,体育,娱乐,星座命理,IT 数码,COD 党
文艺,旅游
投资理财,IT 数码,A 股,世界杯,巨蟹,汽车,教育就业,新闻资讯,文艺
保定生活
搞笑幽默,视频音乐
娱乐

名人明星,旅游,美食,搞笑幽默,新闻趣事
IT 数码
时尚,美食,文艺,娱乐
好忧伤
美女,搞笑幽默,北京生活,娱乐,游戏动漫,体育
美食
游戏动漫
新闻趣事,搞笑幽默,黑龙江生活
美国,心情,歌手,美女
美食
旅游,美食
旅游,军事,新闻资讯,游戏动漫,体育
娱乐,时尚
健康,投资理财,美食,旅游,星座命理
IT 数码
八卦杂谈,娱乐
微博奇葩,游戏动漫,时尚
IT 数码
汽车,星座命理,娱乐
美女,美食,娱乐
健康,汽车,IT 数码
厦门,泉州,大学生,90 后,自主创业,华侨大学,文艺
美女,旅游,军事
健康,教育就业,体育,美食,美女,军事
体育
娱乐
教育就业,美食,IT 数码
游戏动漫,军事,娱乐
商丘生活
旅游
旅游,美食
文艺,体育,娱乐
时尚,旅游,美女,美食,娱乐,军事,游戏动漫,投资理财,IT 数码
投资理财,星座命理,IT 数码
新闻资讯
微博奇葩
娱乐,星座命理
重口味,车世界,搞笑幽默,盐城生活,娱乐
美食,娱乐
旅游,健康,娱乐,美食
旅游,健康,文艺,哄孩子高手,重口味,怀旧,育儿,80 后的尾巴,电影爱好者,旅游美食爱好者
搞笑幽默,旅游,微博奇葩,新闻趣事,广东生活
汽车
旅游

、、、、、,
娱乐
新闻资讯
健康,投资理财,美食,娱乐,旅游
美食,喜欢音乐,懒人,开心,吃货
教育就业,时尚,健康,旅游,星座命理
文艺,娱乐
文艺,旅游,美食,IT 数码,娱乐
猫咪控,游戏动漫
娱乐,体育,IT 数码,旅游美食,健康,旅游,汽车
视频音乐,旅游,新闻趣事,搞笑幽默,时尚
炫酷到没朋友,神烦,东方厨,后期制作,coser,渣画手,酷爱让我毕业,手办,游戏动漫
健康,旅游
娱乐
新闻趣事
汽车,教育就业,娱乐
美食,校园生活,美图摄影,读书分享
视频音乐,汽车
新闻资讯
山东生活
美容,时尚,健康,娱乐,星座命理,美食
文艺,健康,教育就业,新闻资讯
IT 数码
IT 数码
IT 数码
美食
教育就业,旅游,星座命理,美食
新闻资讯,投资理财
苦 B 学生,文艺女青年,90,小清新,名人明星,娱乐
青春正在绽放,投资理财,美食,新闻资讯,教育就业,IT 数码
美女
星座命理,美食,健康,旅游,时尚
旅游,娱乐,文艺
教育就业,旅游,健康,体育,投资理财,汽车
IT 数码
美食,新闻资讯,娱乐
旅游,星座命理,美食,军事,美女
文艺
美食,投资理财,旅游,星座命理
健康,迷糊,学生一枚,旅游美食,星星,胡思乱想,星座控,文艺
新闻资讯,星座命理,体育,投资理财,健康,文艺,军事
健康,娱乐
排球,吃货一枚,射手座,纠结,爱生活,神经大条,咖啡控,旅游
娱乐,美女,游戏动漫,IT 数码,体育

军事,汽车,美女
美女,健康,游戏动漫,IT 数码
健康,旅游,美食
体育,美食,时尚,娱乐,星座命理
美食,汽车,美女,IT 数码
美食,新闻资讯,文艺,娱乐,星座命理,旅游美食
新闻资讯,投资理财
新闻资讯,投资理财,娱乐,体育,美女
听音乐,星座,吃喝玩乐,唱 K,B 型血,动画,巧克力,购物,美食,赛车
娱乐
投资理财,汽车,旅游,美食,军事
汽车,娱乐
IT 数码,旅游,美女,教育就业
文艺
体育,打篮球
文艺,教育就业,美食,旅游,星座命理
文艺
新闻趣事,搞笑幽默,小吖语录
投资理财,健康
旅游,星座命理,美食,体育,娱乐
IT 数码,教育就业,旅游,美食,娱乐
胡思乱想,独立,服装设计,学生一族,人生,水瓶女,笑,自然,坚强,原创
不会晕血的女人,执着学医,热爱医学
名人明星
投资理财,健康,美食,旅游
旅游
文艺,游戏动漫
幸福,美食,美女,旅游,健康,投资理财
娱乐,文艺
爱狗狗爱生活,狗民,萨摩耶
娱乐
文艺,游戏动漫
IT 数码,文艺
文艺,投资理财,旅游,汽车,体育
时尚,文艺,娱乐
娱乐,旅游,时尚,健康,美食
投资理财,体育,健康,新闻资讯,汽车
文艺,投资理财,汽车,游戏动漫,体育,娱乐
投资理财,新闻资讯,军事,美食,美女
娱乐,美食,旅游,健康
音乐达人,篮球,冷笑话,宠物,90 後,神经质,爱旅行,dota,游戏动漫,体育
爱笑,90 后非脑残
游戏动漫,汽车,旅游,美食,文艺
纳兰容若,外表老实内贪玩,古典艺术,旅游

娱乐,汽车,时尚,文艺
时尚,文艺,娱乐
时尚,旅游,星座命理,美食,娱乐
IT 数码
时尚,旅游,星座命理,美食,娱乐,逛街,买衣服,吃,玩
彩妆造型师,明星,化妆师,美女,学化妆,新娘跟妆,时尚
旅游
名人明星
健康,文艺,旅游
新闻资讯,军事,美食,旅游,汽车
时尚,投资理财,旅游,星座命理,美食
美食,游戏动漫,娱乐
旅游,军事,IT 数码,文艺,娱乐
汽车,投资理财,旅游,美食,IT 数码
健康
汽车,体育
shopping,模特,偶稀饭睡觉觉,读书,投资理财,自然,旅游美食,游泳,烹饪,服饰
娱乐
房产,财经资讯,搞笑幽默,旅游,体育资讯
游戏动漫,旅游,文艺
军事,IT 数码,文艺,体育,娱乐
汽车
星座命理,投资理财,财经
时尚,汽车,体育,杭州,会计,腐,网球
培训,中医,新闻资讯,教育就业,投资理财,军事,娱乐
文艺
投资理财,汽车
汽车
遊戲動漫,游戏动漫
文艺
游戏动漫,旅游,娱乐,文艺,时尚
汽车
美食,IT 数码,时尚,汽车,爱情,白羊座,健身
旅游,体育,汽车,爱唱歌爱电影
电脑,电影迷,爱玩,聊天
娱乐
爱周杰伦,美食爱好者,懒
健康,美食,娱乐,游戏动漫
游戏动漫,娱乐
旅游
旅游,健康,体育
教育就业,旅游,美食
军事,娱乐,旅游,IT 数码
学生,上网,美食

健康,教育就业,娱乐,游戏动漫,IT 数码,投资理财,旅游,星座命理,军事
名人明星,文艺,娱乐
教育就业,健康
娱乐
美食,旅游,星座命理,美食,文艺,娱乐
娱乐,旅游,美食
美容服饰
娱乐,美食
文艺,旅游,星座命理,投资理财,娱乐
汽车,新闻资讯
娱乐,旅游,星座命理,美食,文艺
体育,文艺,健康,时尚,投资理财
美食,娱乐,健康,原创,爱唱歌
娱乐,投资理财
时尚,星座命理
新闻资讯
娱乐,时尚,健康
生活大爆炸,射手座,随性,纠结的矛盾体,爱玩也爱宅,闷骚科研男,美食
美食,文艺,旅游,健康,新闻资讯,投资理财
名人明星
文艺
校园生活,娱乐
新闻资讯,投资理财,时尚,健康,娱乐
旅游,IT 数码
文艺,新闻资讯
时尚,星座命理,美食,娱乐
天蝎,安静,健康,旅游,美食,投资理财
汽车,美女,体育
新闻资讯,投资理财,军事
健康,旅游,美女,军事,娱乐
旅游,IT 数码,体育
娱乐,美女
旅游,教育就业,新闻资讯,汽车,军事
时尚
旅游,娱乐,投资理财,健康,美食
活泼,色影爱好者,无厘头,好男人就是他,大学生,善良,双子座,旅游
潮流,旅游爱好者,爱吃,喜欢音乐,美食,文艺,娱乐
娱乐
体育
教育就业
投资理财,教育就业,旅游,奋斗,青春
新闻资讯,投资理财,旅游
娱乐,健康,教育就业,时尚,文艺
新闻资讯

娱乐
游戏动漫
旅游
美食,娱乐
内向
旅游
汽车,文艺
体育
美图摄影,美食,视频音乐,搞笑幽默,济南生活
军事
游戏动漫,IT 数码
新闻资讯,旅游,健康,文艺
汽车,旅游,健康,美食
林峯,o 型血,TVB,唱 k,Music
美食
新闻资讯,汽车,军事,IT 数码,文艺
校园生活,搞笑幽默,IT 数码
宠物图集
美食
美食
美食,新闻资讯
娱乐,文艺,时尚
时尚
新闻资讯
投资理财,时尚,教育就业,文艺,旅游,胡思乱想,睡觉睡到自然醒
IT 数码
健康,旅游,美食,汽车,时尚
投资理财
旅游,美食,美女,IT 数码,娱乐,体育
旅游,文艺,IT 数码,新闻资讯,美女,投资理财
广播,桌游,音乐迷,书籍,自然,健康,旅游,投资理财,游戏动漫
健康,美食
IT 数码
美女,美图摄影,新闻趣事,校园生活,盐城生活
美食,文艺,娱乐
健康,美食,星座命理,时尚,IT 数码
美食,体育
八卦杂谈
健康,美女,汽车
新闻资讯,健康
美女,游戏动漫,旅游,美食,文艺,娱乐
IT 数码
游戏动漫,文艺,美食,健康,美女
视频音乐,穿衣美容,搞笑幽默,时尚,投资理财,旅游,美食,娱乐

旅遊,美食,攝影
文艺,娱乐,体育
新闻趣事,搞笑幽默,微博奇葩,美食,游戏动漫
美女,时尚,娱乐,星座命理,美食
视频音乐,娱乐
时尚,美食,体育,旅游,投资理财
八卦杂谈,IT 数码,新闻趣事,视频音乐,搞笑幽默
健康,旅游
教育就业,美女,投资理财,时尚,星座命理,文艺,娱乐
美女
时尚,美女,汽车,投资理财,星座命理,IT 数码,体育
体育,动漫迷
时尚
双子座,80 后,唱歌,动漫,自由,时尚,旅行,旅游,摄影师,摄影
健康,时尚,美食,旅游
IT 数码,汽车
美女,娱乐,旅游,美食
星座命理,时尚
傻傻分不清楚
名人明星
游戏动漫
旅游
微博奇葩
娱乐
投资理财,时尚,星座命理
娱乐
新闻资讯,游戏动漫,健康,旅游,美食
揭阳生活,美食,娱乐,时尚
健康
健康,军事,文艺,旅游,新闻资讯,汽车,投资理财
娱乐
时尚
旅游,时尚,娱乐
美女,投资理财,星座命理,新闻资讯,汽车,旅游,IT 数码,体育
新闻资讯,时尚,IT 数码,旅游,体育
健康,旅游,娱乐
校园生活,新闻趣事,美女,美图摄影,体育资讯
新闻资讯
汽车
星座命理,IT 数码,体育
美食
设计,宠物,IT 数码
娱乐
新闻资讯,教育就业,军事

文艺,时尚
投资理财,旅游,文艺
汽车,美食,IT 数码
健康,汽车,美食
教育就业,军事,体育
军事,IT 数码,体育
教育就业,军事,体育
学生一枚,健康养生,喜欢旅游,校园,好书,历史,翻译,文字,新闻,情感生活
美女
美女,美食,娱乐
90,购物,看小说,潮流,IT 数码,时尚,健康,星座命理,娱乐
投资理财,军事,IT 数码,文艺
书籍,旅游,健康,文艺,投资理财
北京生活,游戏动漫
星座命理,新闻资讯,旅游,教育就业,文艺,娱乐
汽车,星座命理,IT 数码
星座命理,文艺,美食,时尚
美食
文艺
美食,文艺,健康,旅游,时尚
文艺,游戏动漫,星座命理,体育,美女
汽车,军事,游戏动漫
美食,美女,时尚,旅游,美食,文艺,娱乐
军事
教育就业,新闻资讯,游戏动漫,健康,时尚
旅游,新闻资讯,游戏动漫,文艺
体育
旅游,星座命理,美女,游戏动漫,娱乐
LOL,运动,美女,游戏
新闻资讯,健康,旅游,星座命理,文艺
军事,美食,游戏动漫,IT 数码
IT 数码
旅游,体育
体育
星座命理,名人明星,搞笑幽默,新闻趣事
健康,投资理财,旅游,娱乐,美食
美食爱好者,摇滚,养生,画画,五月天,麦霸,书法,周杰伦,篮球爱好者
教育就业
教育就业,星座命理,IT 数码
我就是我,乐天派,娱乐,星座命理,美食,时尚
健康,投资理财,美女
旅游,美食,电视,小女人,健康
旅游
美女,星座命理,文艺,游戏动漫,投资理财,体育

美食,文艺,娱乐
时尚,旅游,文艺
八卦杂谈,教育就业,投资理财,文艺
教育就业,娱乐,时尚
游戏动漫,美食,星座命理
时尚,健康,旅游,星座命理,文艺
时尚,渴望着…
新闻资讯,时尚,娱乐,旅游,文艺
文艺
文艺,时尚
教育就业,投资理财,娱乐
IT 数码
美女,军事,文艺
时尚,美食,文艺,健康,IT 数码,体育,教育就业,美女,汽车,娱乐
旅游,美女,娱乐,IT 数码
IT 数码
投资创业
看书,跑步,网球,乐观
新闻资讯
军事,健康
投资理财,娱乐
喜欢旅游,爱音乐,蓝色,美食爱好者,摄影爱好者
旅游,娱乐
娱乐
我啊,爱你哟,Ashin,金牛女
旅游,军事
JJ,Project,林宰范,JB
娱乐
搞笑幽默
文艺,体育,娱乐
文艺
萌宠
健康,美食,文艺,新闻资讯,旅游
我就是我,电影爱好者,哈哈,交朋友
汽车,教育就业
娱乐,IT 数码,星座命理,美食,文艺
星座命理,美女
宅女一枚,浪漫,新闻,单纯,自信,拍照,music,购物狂,爱美食,电影迷
旅游,旅游 fashion,美食,波尔多高商,北京,法國,葡萄酒,波尔多,wine
游戏动漫
旅游,文艺,娱乐,健康,星座命理
名人明星,旅游,搞笑幽默,河南生活,游戏动漫,投资理财,星座命理,军事,体育
校园生活,星座运势,名人明星
美食,新闻趣事,旅游,文艺,时尚,新闻资讯

旅游,星座命理,文艺,体育,娱乐
文艺,旅游,投资理财,时尚,健康,美食
健康,新闻资讯,旅游,文艺,IT 数码,娱乐
旅游,星座命理,健康,娱乐
新闻资讯,旅游,美食
星座命理,美食,军事,IT 数码,娱乐
旅游,时尚,IT 数码,文艺,娱乐
健康,IT 数码,体育
娱乐,旅游,美女,游戏动漫
b 型血,美食,教育就业,军事
NBA,乐观,宅女,交友,吃货,时尚,旅游,健康,美食,IT 数码
新闻资讯,投资理财
文艺,娱乐
教育就业,娱乐,美食,文艺,旅游,小任性,白日梦,夜猫子
娱乐
健康,时尚
睡觉,看书,自由,时尚,情感,郑允浩,表演,矛盾体,交朋友,星座命理
星座命理,美食,旅游,时尚,娱乐
自由职业,喜欢唱歌,寂寞
搞笑幽默
大大梦想家,独家限量,big——dreamer,便宜好品质,女装,外贸童装
时尚,游戏动漫,IT 数码,文艺,娱乐
新闻资讯,军事,体育,美女
时尚,健康,星座命理,美食,娱乐
娱乐,星座命理,投资理财,时尚,教育就业
星座命理,90 后
文艺,星座命理,新闻资讯,健康,娱乐
投资理财
体育,美女,娱乐,汽车,军事
自由,摄影,上网,唱歌,旅行,时尚,旅游,IT 数码,投资理财,娱乐
健康
新闻资讯,健康,时尚,旅游,美食
新闻资讯
游戏动漫,新闻资讯,IT 数码,教育就业
新闻资讯,旅游
旅游,美食,健康,新闻资讯,娱乐
新闻资讯,星座命理,投资理财,时尚,健康,美食,娱乐
娱乐
名人明星,微博奇葩,篮球爱好者,喜欢听歌
文艺
雀斑类型,雀斑治疗,雀斑,青春痘,黑头,健康
健康
新闻资讯,时尚
健康,投资理财,新闻资讯,旅游,IT 数码

娱乐
美食, 文艺, 娱乐, 新闻资讯, 旅游, 健康, 时尚
美食, 娱乐, 教育就业, 投资理财, IT 数码
旅游, 娱乐, 教育就业, 游戏动漫
体育
IT 数码
理财, 销售, IT 数码
美食, 时尚, 旅游, 文艺, 娱乐
娱乐, 美食, 旅游, 教育就业, 投资理财
娛樂, 旅遊, 文藝
搞笑幽默
教育就业, 旅游, 娱乐
美女
旅游, 军事
游戏动漫, 娱乐
健康, 美女, 军事, 旅游, 文艺
自虐
名人明星, 新闻资讯, 体育
美女, 星座命理, IT 数码
投资理财, 新闻资讯, 文艺, IT 数码, 军事
娱乐, 体育, 汽车
搞笑幽默, IT 数码, 美图摄影, 旅游, 校园生活
体育, 美食
投资理财, 健康
汽车, 时尚, 游戏动漫
健康
娱乐
旅游
星座命理, 时尚, 健康, 美食, 娱乐
旅游, 美食, 游戏动漫, 文艺, 娱乐
星座命理
健康, 新闻资讯, 时尚, 美食, 娱乐
游戏动漫, 时尚, 美女
健康, 投资理财, 游戏动漫, 旅游, 文艺
投资理财, 新闻资讯, 军事
文艺, 教育就业, 军事
娱乐, 时尚, 文艺
微博奇葩, 文艺, 体育
汽车, 军事, IT 数码, 投资理财, 美女
开心, 喜欢音乐, 美食
星座命理, 新闻趣事, 搞笑幽默, 微博奇葩, 文艺, 美食
汽车
美女
时尚, 文艺, 军事, 新闻资讯, 健康, 投资理财, 教育就业, 娱乐

旅游
八卦杂谈,旅游,其他生活,新闻趣事
汽车
手机控,80后,奋斗 in,善良,平常心
旅游,游戏动漫
喜欢玩,吃货一枚
旅游
教育就业,旅游,文艺
海贼王,青春,双子,爱睡觉
新闻资讯,体育,旅游
汽车,文艺,美食
时尚,旅游,星座命理,美食,娱乐
美食,军事,文艺,体育,娱乐
美女,投资理财,旅游
健康,文艺,时尚,娱乐,旅游
健康,星座命理,新闻资讯,体育,汽车,娱乐
美食,文艺
游戏动漫
游戏动漫,星座命理,时尚,汽车,娱乐
星座命理,健康
汽车,体育,文艺,IT数码,军事
投资理财,星座命理,教育就业,体育
星座命理,美食,旅游
旅游,美女,美食,体育,游戏动漫
文艺,汽车,新闻资讯,IT数码,军事,旅游,投资理财
星座命理,教育就业,时尚,游戏动漫,旅游,美食
旅游,游戏动漫,IT数码,军事,娱乐
投资理财,军事,IT数码
美食,军事,IT数码
时尚,旅游,星座命理
健康,美女,投资理财
微博奇葩,搞笑幽默,新闻资讯,娱乐
健康,新闻资讯,军事
美食,游戏动漫
旅游,IT数码,美食,文艺,娱乐
投资理财
教育就业,文艺,娱乐,星座命理,健康
投资理财,汽车,IT数码
娱乐,教育就业,健康,星座命理,美食
娱乐,美食
娱乐
旅游
广东生活,体育
美食,时尚

旅游,体育,走自己的路,娱乐
新闻资讯,游戏动漫,投资理财,文艺,娱乐
旅游,时尚,投资理财,美食,文艺
娱乐,游戏动漫,时尚,IT 数码,美食,星座命理
健康,娱乐,星座命理
美食
新闻资讯,星座命理,时尚,汽车,娱乐
微博奇葩,搞笑幽默,新闻趣事
IT 数码
简单,运动,双鱼座,旅游美食爱好者,动漫,学生一族,真诚,沉默,健康
IT 数码,新闻资讯
时尚,旅游,新闻资讯
时尚,健康,旅游,美食,娱乐
新闻资讯
旅游
娱乐
健康,新闻资讯,IT 数码,娱乐,体育
娱乐,旅游,汽车
旅游,游戏动漫,健康,美食
教育就业,娱乐,投资理财,文艺,美食
汽车,时尚
视频音乐
文艺
美食,娱乐,文艺,新闻资讯,时尚,旅游
旅游
汽车,游戏动漫,星座命理,体育,IT 数码
时尚,健康,美食,旅游
美食
文艺,les,看小说,漫画
健康,汽车,投资理财,美女,星座命理
seo,爱玩,足球,篮球,AllenIverson,nba,HipHop,Eminem
时尚,美食,文艺,娱乐
汽车,健康,美食
文艺,体育,娱乐,军事,游戏动漫
萌宠
娱乐
看电影,格子控,护肤,明星,英国,游戏动漫,时尚,美女,美食,娱乐
新闻资讯,健康,娱乐,星座命理
时尚
重庆生活,美容服饰,健康
教育就业,投资理财,美食,健康
旅游
萌宠,微博奇葩,视频音乐,新闻趣事,搞笑幽默
美食,美女,视频音乐,娱乐,体育

体育,娱乐
娱乐,体育
汽车,教育就业,娱乐,IT 数码,体育
文艺,美食,星座命理,投资理财,娱乐
娱乐
投资理财,时尚,旅游,美食,娱乐
美食,旅游,星座命理,上海生活,穿衣美容,健康
IT 数码,美女
旅游,娱乐
穿衣美容
游戏动漫,时尚
娱乐,旅游
其他生活
新闻资讯,健康,汽车,文艺
健康
文艺,IT 数码
新闻趣事
投资理财
旅游
娱乐,体育
美食
时尚,旅游,星座命理,美食,投资理财,汽车
星座命理
娱乐,体育,IT 数码,旅游,军事
体育,IT 数码,娱乐
视频音乐,美女,校园生活,搞笑幽默,体育资讯
美食
招工,市人才服务中心,便民方舟,招聘会,宿迁人才市场,人才网,找工作,求职,招聘,宿迁人才网
投资理财,游戏动漫,汽车,星座命理,美食,懒懒的,天秤
汽车
美女,美食,旅游,时尚,健康,新闻资讯,娱乐
文艺,美食
健康,投资理财,时尚,旅游
美食,健康,时尚,旅游
娱乐,幸福辣妈
各种宅,起床困难户,学生,刘诗诗,张根硕,刘亦菲,夏七夕,独木舟,花火,爱睡觉
健康,文艺,旅游,娱乐
澳洲进口奶粉,跑山,GSXR,重机车,进口奶粉,摩托车,汽车,美食爱好者,淡定
IT 数码,娱乐
新闻趣事
旅游,新闻资讯,美食,教育就业,健康
汽车,星座命理,军事,新闻资讯
美食

新闻趣事,IT 数码
IT 数码,体育,生活,安卓控
健康,随性,乐天派,情绪化,90 后但我不脑残,游戏动漫,旅游,星座命理,美食,娱乐
文艺,娱乐,时尚,健康,星座命理
时尚,旅游,星座命理
搞笑,品牌,时装,宅女加腐女,商业,爱生活,帅哥,舞蹈,爱睡觉,生活大爆炸
娱乐
娱乐,游戏动漫,爱猫
美食,创业,设计,健康,军事,娱乐
搞笑幽默
娱乐,搞怪,体育,游戏动漫
动漫
星座命理
美女,旅游,美食,体育,娱乐
健康,娱乐,美食,游戏动漫
旅游,健康
旅游,新闻资讯,投资理财,汽车
苏州生活,娱乐
旅游,美女,教育就业,文艺,娱乐
文艺,旅游,美食,体育,娱乐
汽车,美食,文艺,娱乐,IT 数码
萌宠,名人明星,微博奇葩,搞笑幽默,视频音乐
汽车,时尚,健康,旅游,文艺
游戏动漫,军事,娱乐,星座命理
事业,。情感,责任
新闻资讯,投资理财,旅游,美食,星座命理
美图摄影,娱乐,新闻资讯,健康,时尚,文艺
其他生活
旅游,健康,教育就业
文艺,布丁,暮光之城,大学
旅游,健康,时尚,星座命理,美食,娱乐
时尚,文艺,旅游,健康,美食,娱乐
投资理财,新闻资讯,健康,时尚,旅游,娱乐
娱乐,新闻资讯
文艺,IT 数码,汽车,美女,体育
搞笑幽默,北京生活
时尚
时尚
星座命理,美食
时尚,美食,星座命理,旅游,健康
紫藤,娱乐,公益,情感
娱乐
手机控,小说,90,随性,淡定,单身,聊天,青春
娱乐

文艺,教育就业,新闻资讯,投资理财,IT 数码
游戏动漫,IT 数码
美食
旅游
娱乐
美食,文艺,娱乐,旅游,体育
美食,时尚,娱乐
娱乐
投资理财,时尚,娱乐,美食,IT 数码,文艺,教育就业
娱乐,IT 数码,时尚,美食
搞笑幽默
旅游
名人明星,视频音乐,九零后
旅游
娱乐
体育,游戏动漫
游戏动漫,军事,IT 数码,文艺,体育
名人明星,新闻趣事,旅游
梦想环游世界,跳舞,疯子,视艺术如命
娱乐,music,喜欢旅游,梦想,公益,爱自由,交朋友,苹果,美食,IT 数码
美食,时尚,娱乐
时尚,汽车,军事
新闻趣事,视频音乐,名人明星,美女,穿衣美容
汽车
充气娃娃,避孕套,成人用品
星座命理,新闻资讯,美食,教育就业,投资理财
穿衣美容,娱乐
健康,新闻资讯
时尚,旅游,健康
时尚,淡定,时尚达人,服饰,情绪化
娱乐,健康,文艺,美食,旅游,游戏动漫
美女,娱乐,文艺,美食
动画,文艺,娱乐,时尚,美食
旅游,娱乐,美食
搞笑幽默
时尚
娱乐,美食,美女,游戏动漫,汽车
IT 数码,娱乐
美食
健康,美女,投资理财
美女,美食,IT 数码
娱乐,健康,旅游,体育
美食
游戏动漫,汽车,美女

投资理财, 美食
游戏动漫, 旅游, 星座命理, 军事, IT 数码
健康, 军事
汽车
旅游
旅游
美女, 旅游, IT 数码
娱乐, 文艺, 健康, 旅游, 美食
萌宠, 新闻资讯, 汽车
投资理财, 新闻资讯, 健康, 旅游, 美食, 娱乐
新闻资讯, 健康, 时尚, 美食, 星座命理
娱乐, 文艺, 星座命理, 美女, 时尚
视频音乐, 萌宠, 旅游, 美女, 微博奇葩, 体育
美食
旅游, 美食, 军事, 新闻资讯
娱乐
游戏动漫, 文艺
名人明星
时尚, 娱乐, 健康, 旅游, 文艺
健康, 旅游, 文艺, 体育, 娱乐
娱乐, 文艺
设计, 旅行, 服饰, 努力, 自拍, 健康, 新闻资讯, 教育就业, 旅游, 美食
旅游
屌丝一枚……
教育就业, 新闻资讯, 健康, 旅游, 文艺
湖北生活, 娱乐
健康, 美食, 旅游, 美食爱好者, 80 後, 爱幻想, O 型血, 淘宝, 狮子女, 喜欢旅游
健康, 体育, 娱乐
沈阳生活
搞笑幽默
娱乐, 健康, 时尚, 投资理财, 新闻资讯, 美食, 旅游
文艺
美食
时尚
军事, 视频, 爱自由, 玩
小说迷, 无厘头, 胡思乱想, 吃货, 爱幻想, 孩子气, 偶稀饭睡觉觉, 看小说, 爱美食, 偶像剧
娱乐, 汽车
懒人。。。懒人、、、
文艺, 游戏动漫
文艺
投资理财, 美食, 文艺
教育就业
新闻资讯, 投资理财, 娱乐, 健康, 时尚
美女

微博奇葩,萌宠,名人明星,新闻趣事,搞笑幽默
名人明星
上海生活
体育
名人明星,穿衣美容,新闻趣事,微博奇葩,萌宠
美食,军事,文艺,健康,时尚,星座命理,娱乐
搞笑幽默
美女,游戏动漫
军事
游戏动漫,汽车,健康
外貌协会,完美主义,摩羯女
美食
时尚,文艺,旅游
体育
新闻趣事,旅游,美女,视频音乐
南通生活,校园生活
美女,健康,旅游
体育
投资理财,IT 数码
微博奇葩
高三,怀旧,追星,可爱,爱睡觉,帅哥,美容,星座命理,IT 数码,文艺
文艺
高三,怀旧,追星,可爱,爱睡觉,帅哥,美容,教育就业,旅游,体育
美食,文艺,汽车,体育,IT 数码
军事,美女,汽车
星座命理,搞笑幽默
名人明星,视频音乐,海外生活,教育就业,时尚,娱乐
福建生活,时尚
IT 数码,投资理财,文艺,新闻资讯
新闻资讯
娱乐,文艺
视频音乐
视频音乐
安静
美女
星座命理,体育,时尚
美食,搞笑幽默,新闻趣事,萌宠,微博奇葩
高三,怀旧,追星,可爱,爱睡觉,帅哥,美容,游戏动漫,军事,文艺
旅游,新闻资讯,体育,文艺,娱乐
美食
健康,美女,汽车,旅游
时尚,美食,军事,IT 数码,文艺
活泼开朗,乐天派,爱笑,玩,大学生,射手座,网购,宠物,90 後,营销
深圳生活,时尚,投资理财

时尚,娱乐
美女
新闻趣事,微博奇葩,萌宠,时尚,文艺,星座命理
娱乐,IT 数码,美食,美女,游戏动漫
柯南,街舞,IT 数码,军事,体育,娱乐,游戏动漫
视频音乐,新闻趣事,微博奇葩
多愁善感,跑步,感性,爬山,爱旅行,跆拳道,热爱运动，听歌
美食,新闻资讯
旅游,美食
军事,新闻资讯,IT 数码,体育
美食,美女,娱乐
搞笑幽默,游戏动漫
体育
游戏动漫,美女,体育
四川生活,泸州出租车,泸州的士,酒城的士,出租车预约,IT 数码,娱乐
时尚,星座命理,旅游,汽车,娱乐
军事,广播,矛盾,爱睡觉,天蝎座,听音乐,睡懒觉,游戏,小说,音乐
教育就业
旅游
健康,娱乐
IT 数码,旅游,汽车,美女,军事,文艺,娱乐
星座命理,美食,军事
旅游,美女,视频音乐,搞笑幽默,新闻趣事
美女,IT 数码
文艺
美食,军事,IT 数码
美女,军事,IT 数码,美食,旅游
湖北生活
文艺,体育,娱乐
文艺,教育就业,健康,投资理财,美食
旅游
投资理财,新闻资讯,健康,时尚,旅游
Super,Junior,李赫宰,赫妖,蓝家女人,ELF
江门生活
名人明星,美食,搞笑幽默,狮子女,开朗,bl,学生,任性
美食,健康,旅游,娱乐
吃货,独立,我就是我,90 後
娱乐,时尚,文艺
新闻资讯,美女,游戏动漫
教育就业,时尚,完美主义,宅
时尚,旅游
美食爱好者
娱乐
美女

美女
时尚
微博奇葩,视频音乐,名人明星,美女,搞笑幽默
IT 数码,军事,新闻资讯
星座命理,娱乐
旅游,美食
体育,汽车,星座命理,旅游,IT 数码
江苏生活,爱自由,听歌,90 后
美食,育儿百科
单纯,刘亦菲,漫画,宅女一枚,看小说
健康,时尚
体育
娱乐,投资理财,军事,时尚,旅游
淘宝控,拍照,狗狗,护肤,O 型血,女人,大学生,天秤座,重口味,浪漫
娱乐,美食,游戏动漫,时尚,文艺
美女,汽车,体育
旅游,星座命理,美食,文艺,体育
美食
美女
文艺
体育
健康,时尚,汽车,旅游,美食
文艺,爱猫
美女,IT 数码,汽车
微博奇葩
旅游,娱乐,美女,文艺
美食,新闻资讯,健康,旅游,娱乐,汽车
汽车,体育
旅游,美食,娱乐
美女
时尚,游戏动漫,旅游,美食,娱乐
执着,聊天,简单,天蝎,美食,时尚,文艺,娱乐
动漫
旅游,美食,娱乐
投资理财
搞笑幽默
IT 数码,旅游
星座命理,听歌,旅游,阳光,开朗,90 后。。。。。
投资理财,薄瓷板,瓷艺画,背景墙,新闻资讯,星座命理,汽车
写作
90 后但我不脑残
旅游,北京生活,娱乐,投资理财,健康,google,教育就业,DIY,tvb,i
体育
美女,汽车,旅游,美食,体育

美食,教育就业
美食
娱乐,新闻资讯,文艺
Simon
体育
NEET,抖 S 脸抖 M 心,强迫症晚期,变态
穿衣美容,时尚,娱乐,投资理财
健康,旅游,美食,时尚
IT 数码,新闻资讯,军事,游戏动漫
美食
体育
美食,星座命理,健康,旅游
铁岭生活,爱音乐,看电影,Bazinga,安妮海瑟薇,银魂党,LinkinPark,Coldplay,拜仁死忠,伪文艺
小清新
诗源,云宰,俊熙,郑恩地,徐仁国,HOYA
游戏动漫,星座命理,IT 数码
娱乐
读书分享,鄂州生活,新闻资讯,时尚,投资理财,文艺,娱乐
旅游,天枰座,宅女一枚,IT 数码,娱乐,投资理财,游戏动漫,美食
汽车,美女,体育
校园生活,健康
时尚,星座命理,IT 数码
IT 数码,游戏动漫,体育
娱乐,吃喝玩乐
教育就业,旅游,军事
娱乐
睡懒觉,吃喝玩乐,金牛座
新闻资讯,教育就业,IT 数码
时尚,美女
健康,新闻资讯,时尚,教育就业,投资理财
娱乐,美食,IT 数码,投资理财,汽车
时尚,汽车,旅游,IT 数码,娱乐
新闻资讯,美女,投资理财,IT 数码,体育
视频音乐,IT 数码,游戏动漫,文艺,娱乐
文艺,教育就业
教育就业,娱乐,健康,游戏动漫,投资理财,星座命理
新闻资讯,教育就业
旅游,时尚,汽车,体育
汽车,旅游,IT 数码,体育,娱乐
教育就业,投资理财,IT 数码,旅游,军事
旅游,娱乐
娱乐,体育
金牛女,暮光之城,k 歌,生活,旅游,娱乐

美女,旅游
游戏动漫
这是一个班微博
旅游,新闻资讯
美女,体育
体育
时尚,投资理财,星座命理
美食,星座命理,娱乐,游戏动漫,旅游,健康,美食
文艺,时尚,旅游,新闻资讯
搞笑幽默
娱乐
汽车,胖丫头,爱地瓜,世界杯阿根廷队,大叔控,爱写作,爱电影,爱旅游
穿衣美容,萌宠
旅游,美食,穿衣美容,娱乐
美女
旅游,星座命理,美食,教育就业,IT 数码,娱乐,时尚
旅游,美食,新闻资讯,星座命理,文艺,娱乐
体育
摄影爱好者,格子控,怀旧,娱乐,孩子气,忧郁,懒人,日语,夜猫子,睡觉
旅游
健康
汽车,文艺,军事,娱乐,体育
手机看小说,娱乐,美食,健康,旅游,星座命理
游戏动漫,娱乐,体育
健康,美食
穿衣美容
美女,投资理财,时尚,军事,文艺
时尚
美食,健康
游戏动漫,教育就业,美食
新闻资讯,时尚,投资理财,教育就业,健康
美女,游戏动漫,汽车,IT 数码,娱乐
美食
健康,新闻资讯
美食,新闻资讯,时尚,旅游,娱乐
健康,娱乐,汽车,旅游,美食
搞笑幽默,美女
军事,娱乐,文艺,爱佳老大
旅游,体育
穿衣美容,快乐,酷爱旅游
视频音乐,名人明星,新闻趣事,军事,体育
星座命理
旅游,时尚
美食,时尚,娱乐

喜欢枪类游戏,CF 火线迷,娱乐,IT 数码,游戏动漫
文艺
新闻资讯,旅游,军事,文艺,娱乐
新闻资讯,游戏动漫,军事,文艺,娱乐
娱乐,健康,旅游,美女
IT 数码
视频音乐,时尚,教育就业
新闻资讯,游戏动漫,旅游
健康
旅游,文艺,投资理财,时尚,健康
娱乐
视频音乐,读书分享,动漫,美容服饰,旅游,娱乐,教育就业,游戏动漫,健康,星座命理
幸福,美剧,巨蟹女,新闻资讯
汽车,教育就业,IT 数码,文艺,娱乐
时尚,文艺,旅游,星座命理,美食,娱乐
旅游
旅游,美食,汽车,文艺,娱乐
娱乐,教育就业,时尚,文艺,体育,IT 数码,新闻资讯
文艺,军事,星座命理,健康,教育就业
娱乐
新闻趣事
教育就业,文艺
星座命理,美食
时尚,汽车,体育
娱乐
旅游
时尚,旅游,星座命理,美食,娱乐
武汉生活,军事,新闻资讯,汽车,体育
IT 数码
投资理财,娱乐,星座命理,健康,旅游
旅游,时尚,IT 数码,文艺,娱乐
娱乐
健康,旅游,投资理财,汽车,星座命理,娱乐
自由职业,安全驾驶,汽车
书,music,喜欢旅游,双重性格,网购,天秤座,唱 K,阳光,科技,化妆
娱乐
搞笑幽默,健康
房地产,宠物,体育
搞基,宅男,体育,gay,猛男,肌肉,帅哥,时尚,教育就业,文艺
搞基,宅男,gay,猛男,肌肉,帅哥,同志,汽车,投资理财,体育
娱乐,教育就业,游戏动漫,时尚,IT 数码,文艺
搞基,宅男,体育,gay,猛男,肌肉,帅哥,汽车,旅游,美食
体育,健康,旅游,星座命理,美食,文艺
投资理财,教育就业,IT 数码

汽车
美食,星座命理
娱乐,美女,时尚,军事
美食
旅游,新闻资讯,投资理财,汽车,健康,体育
IT 数码
老江湖,手机,90 后非脑残,射手,学生,旅游美食,文字控,90 后,有木有,听音乐
美女,旅游,娱乐,牵手幸福,真爱,外向,直爽,阳光,开朗,90
咖啡,热爱生活,旅游,美食
旅游,游戏动漫,汽车,体育,IT 数码
旅游,IT 数码,70152B
搞笑幽默,新闻趣事,美食,文艺
文艺,体育,娱乐
娱乐,健康,旅游
娱乐
军事,时尚,美女,体育,星座命理
娱乐,文艺,时尚,游戏动漫
新闻资讯,IT 数码,投资理财
娱乐,美食
穿衣美容,星座控,高中生,浪漫,韩国,时装,美食,时尚,星座命理,娱乐
教育就业,新闻资讯
微博奇葩,穿衣美容,美女,名人明星,健康,娱乐,时尚,旅游
娱乐,旅游
教育就业,投资理财
旅游,时尚,新闻资讯,健康,美女
汽车,时尚,美食,体育,娱乐
美剧,阅读,双子座,上网,睡觉,学生,旅游,美食,文艺,时尚
体育
游戏动漫,旅游,美食,文艺,娱乐
娱乐
微博奇葩,时尚
健康,体育,娱乐
文艺,体育,娱乐
娱乐,美食,旅游,健康,游戏动漫
教育就业,新闻资讯,投资理财,美食,娱乐
汽车,美女
娱乐,美女,军事
星座命理,体育,健康,新闻资讯,教育就业,投资理财,娱乐
新闻资讯,美女,娱乐
教育就业,时尚,新闻资讯,旅游,文艺
旅游
福建生活,体育
体育
文艺,娱乐

娱乐
投资理财,游戏动漫,教育就业,IT 数码
娱乐,美女,汽车,军事,旅游
娱乐,星座命理
体育
新闻资讯
微博奇葩
时尚,健康,旅游,美食,娱乐
穿衣美容
健康,IT 数码,美食,游戏动漫
健康
健康,体育,娱乐
美食
美食
IT 数码,军事
唱歌,睡觉,自由,宅,旅行,美食
文艺,体育,娱乐
微博奇葩,四川生活,文艺,娱乐,健康,旅游,美食
魔羯座,吃货,矛盾体,爱电影,美食
微博奇葩
军事,新闻资讯,时尚,汽车,教育就业,娱乐,无语的小孩
娱乐
军事
任性,日本,90 後
美食,旅游,健康,时尚,文艺
旅游,美食,健康,娱乐
IT 数码,IT 數碼,汽車,旅遊,體育,新闻资讯,美食,旅游,汽车,体育
娱乐,旅游,军事,投资理财,汽车,美食,新闻资讯,美女
教育就业,美食,体育
美食,文艺,健康,时尚,娱乐
文艺,美食,旅游,贤妻良母型,音樂,双子女,面食控
娱乐
搞笑幽默,体育
军事,旅游,健康,投资理财,美食,体育
文艺,汽车,美食
美食
美食,军事
美食,健康,新闻资讯,游戏动漫,星座命理,娱乐,文艺
文艺,星座命理
汽车,时尚,娱乐
IT 数码
旅游,懒惰,美容护肤,爱漂亮衣服,双鱼,自由,80 后,矛盾体,幸福,完美主义
视频音乐,触控,音乐,ACG,伪文艺,宅腐无节操,爱玩,幻想
IT 数码

健康
时尚
妈咪,狗狗,动漫,植物,画画,星座命理,教育就业,健康,旅游,美食
旅游,教育就业,星座命理,娱乐,美食
投资理财,时尚,美食,健康,IT 数码
科学探索
星座命理,健康,军事,新闻资讯,文艺,娱乐
娱乐
美女,娱乐,文艺,时尚
娱乐
娱乐,星座命理
美特斯邦威,折扣店,周杰伦,杰迷
星座命理,体育
教育就业,投资理财,汽车
汽车,教育就业,投资理财,新闻资讯,军事,IT 数码
美图摄影,美食,视频音乐,校园生活,西安生活
时尚,星座命理,文艺,教育就业,娱乐
体育资讯,旅游,星座命理,娱乐,体育
健康,投资理财,军事,旅游,美女
投资理财
穿衣美容
旅游,投资理财,教育就业,汽车,新闻资讯
IT 数码
投资理财,星座命理,健康,时尚,娱乐,旅游,美食
旅游,美食,IT 数码
健康
搞笑幽默,星座命理,娱乐,军事
新闻资讯
杂志,八卦,矛盾体,健康,淘宝,购物,美食,网球,爱电影,爱旅游
IT 数码,时尚,旅游
星座命理
旅游
学生一枚,大学,王力宏,音樂,爱旅游,房地产,美食
游戏动漫,军事,时尚
文艺,时尚
娱乐,游戏动漫,IT 数码,时尚,体育
文艺,游戏动漫
投资理财,健康,汽车,旅游,IT 数码
军事
IT 数码,时尚
星座命理,娱乐,游戏动漫
育儿百科,新闻趣事,名人明星,文艺,娱乐,美食,旅游
健康,军事,娱乐,体育,投资理财,教育就业
江西生活,军事,体育

投资理财
沉默是金
临静策瑜,铃木达央,小野大辅真本命,高绿一生推
旅游,娱乐,汽车
娱乐,文艺
新闻资讯,体育,军事
教育就业
新闻资讯,美食,娱乐,体育,文艺
美食
健康
旅游
健康,美女,娱乐,新闻资讯,旅游,汽车
文艺,健康,美食,娱乐,时尚,旅游
时尚,娱乐,美食,健康,旅游
时尚
IT 数码,美食,旅游
游戏动漫,体育
旅游
娱乐,体育
星座命理,美食,IT 数码,文艺,娱乐
星座命理,文艺,时尚,美食
娱乐
语录,美女,游戏动漫,时尚,健康,新闻资讯,娱乐,军事,IT 数码,文艺
旅游,美食,上海生活
娱乐,文艺,时尚
文艺
美食,美女,时尚,娱乐
时尚,健康,美食
不主动的水瓶座,周期性动物,PMS,有关生活,游走于梦的边缘,收藏癖
贝金汉宫,贝克汉姆,TVB,射手座,盗墓笔记,快乐大本营,爱情,设计
名人明星,搞笑幽默,美女,微博奇葩,军事,汽车,体育,IT 数码
游戏动漫,IT 数码,新闻资讯,教育就业,星座命理
其他生活,旅游,新闻趣事,美食,美女
文艺,IT 数码
娱乐,汽车,军事,IT 数码,体育
健康,教育就业
搞怪
喜欢打游戏。。。。
美食
旅游,新闻资讯
穿衣美容,名人明星,星座命理,湖北生活,文艺,时尚,美食
汽车,体育
新闻资讯,美食,投资理财,教育就业,健康
IT 数码,健康

星座命理,健康,游戏动漫,时尚,体育
娱乐
游戏动漫
旅游
体育
美食,旅游,文艺
美女,美食,娱乐,文艺,IT 数码,汽车,时尚
旅游,时尚,投资理财,娱乐
游戏动漫,旅游,美食,IT 数码,文艺
美食,名人明星,体育
旅游,娱乐,星座命理,美食
游戏动漫,时尚
旅游
星座命理,美食
教育就业,投资理财,健康,旅游,文艺,体育,娱乐
教育就业,文艺,旅游,健康,游戏动漫,时尚
教育就业,汽车,健康
网购,休闲,时尚潮流,时装,矛盾,新闻资讯,时尚,旅游,星座命理,娱乐
强迫症,简单,健康,星座命理,娱乐
文艺
美女
名人明星,微博奇葩,新闻趣事,穿衣美容,发呆,爱音乐,Music,林峯
汽车,美食,新闻资讯,健康
娱乐,美女
美女
旅游
体育,汽车,娱乐,美食
新闻资讯,体育,娱乐
汽车,游戏动漫,美食,体育
投资理财,IT 数码,娱乐
音乐发烧友,星座命理
学生,美食,上网,动漫,时尚,IT 数码,汽车
投资理财,美食,健康,娱乐,时尚
美女
名人明星,微博奇葩,新闻趣事,搞笑幽默
娱乐,时尚
懒人,自由,宅,旅行,互联网,爱笑,发呆,爱电影,爱生活,沉默
广东生活,微博奇葩,搞笑幽默,新闻趣事,新闻资讯,汽车,时尚,美食,IT 数码
日语,狗狗,动漫,写字,天蝎女
文字控
文艺,娱乐,旅游,时尚,健康,星座命理,美食
时尚,投资理财,文艺,健康,娱乐
乐观,宅女,服装,情绪化
旅游,文艺,时尚,美食

歌手
健康,时尚,美女,娱乐,IT 数码
活泼开朗,旅游
美食,折纸控,好奇心旺盛
新闻资讯,汽车,教育就业,娱乐,体育
健康,美食,文艺
美女,旅游
旅游,娱乐,星座命理
上海生活,健康,旅游,时尚,文艺,娱乐
娱乐
时尚
旅游,新闻资讯,体育,娱乐
娱乐,美食,体育,新闻资讯,汽车
宠物图集
时尚,投资理财,旅游,美食,汽车
化妆,美食
美食,IT 数码,时尚,汽车,旅游,娱乐
健康
开心,笑,西安,哈利波特,吃喝玩乐,美食
旅游,娱乐,时尚
美食,旅游,文艺,体育
美食
时尚,星座命理,游戏动漫,IT 数码,娱乐
欧美控,麦霸,篮球爱好者,射手,热情,视频音乐
教育就业,健康,旅游,美食
游戏动漫
旅游,星座命理,美食,文艺,娱乐
投资理财,新闻资讯,体育
体育资讯
搞笑幽默,视频音乐
军事,新闻资讯
健康,星座命理,美食,旅游,文艺,娱乐
美图摄影
娱乐
IT 数码
偶稀饭睡觉觉,宅女加腐女,红酒,睡觉睡到自然醒,学习,爱玩
微博奇葩,娱乐
健康,汽车,投资理财,美食
音乐迷,体育
时尚
EXO,卞白贤,吴世勋,鹿晗,朴灿烈,吴亦凡
搞笑幽默,体育,IT 数码
娱乐
健康,旅游,娱乐,汽车

娱乐
微博奇葩,星座命理,搞笑幽默
美食,时尚,旅游,文艺,娱乐
美图摄影,旅游,视频音乐,美女,搞笑幽默
美食
时尚
星座命理,文艺,游戏动漫,军事,体育
教育就业,新闻资讯,IT 数码,旅游,娱乐
旅游,星座命理,美食,娱乐
新闻资讯
体育
娱乐
贵州生活,娱乐,体育,游戏动漫
星座命理,IT 数码,娱乐,美女
文艺
宠物图集,美容服饰,美食,搞笑幽默,准高三
时尚,投资理财,健康,旅游,娱乐
娱乐
微博奇葩
天蝎座,紫色,懒,巨蟹座,幽默,开朗,O 型血,宅,跑步
时尚,投资理财,旅游,美食,娱乐
时尚,新闻资讯,文艺,美食,体育
娛樂
旅游
娱乐
娱乐,美食,旅游
官方微博,财经,中原,河南,杂志,媒体,中原经济区,新闻资讯
旅游
天津画家,山水画,国画,书画艺术,文艺
淘宝,宅女一枚,街拍,健康,娱乐,时尚,美食
美女,体育
美女,美食,娱乐,时尚,星座命理,健康
校园生活,娱乐
娱乐,时尚,文艺,美食,旅游
汽车,体育
游戏动漫,文艺,时尚,美食
体育
微博奇葩
星座命理
军事
搞笑幽默,健康,军事,新闻资讯
娱乐,美食,时尚,星座命理
穿衣美容,新闻资讯
星座命理,投资理财,旅游,文艺,时尚,健康

体育
娱乐
美女,汽车,教育就业
文艺,体育,娱乐
打篮球,睡懒觉,Music,电视剧,游戏,吃,吉他,英语,网络,校园
三明生活,画画,乐观,吃货,海绵宝宝,游戏动漫
文字,旅游,体育
游戏动漫
新闻资讯,娱乐,投资理财,美食,教育就业
旅游,军事,IT 数码,投资理财,汽车
投资理财,新闻资讯,时尚,旅游,健康
健康
视频音乐
娱乐
旅游,名人明星,视频音乐,新闻趣事,搞笑幽默,IT 数码
名人明星,新闻趣事,微博奇葩,搞笑幽默,娱乐
教育就业,投资理财,美食,时尚,文艺
旅游,健康,时尚,美食,娱乐
旅游,健康
视频音乐,学生一枚
萌宠,旅游,文艺,教育就业,美食,星座命理
教育就业
网络写手
健康,美食,文艺
娱乐
旅游
星座命理,美食
贵州生活,搞笑幽默,荔波,投资理财,时尚,旅游,美食,娱乐
旅游
体育
微博奇葩,娱乐
健康,旅游,美食
教育就业,投资理财,美女,体育
体育
IT 数码,星座命理,体育
教育就业
篮球,唱歌,跑步,电影
听音乐,吸血鬼日记,胡思乱想,学习,爱玩,开朗,简单,90 後,健康,娱乐
汽车,旅游,IT 数码
交友,青春,90 後,爱旅游,旅游,健康
美食
汽车
星座命理
陈绮贞,巨蟹,心理,强迫症,冷笑话,爱音乐,热爱生活,文艺,旅游

旅游摄影,手工,旅游
旅游,星座命理,时尚,娱乐
爱笑,玩,美食控
星座命理
星座命理,健康,时尚,美食,娱乐,文艺
游戏动漫,星座命理,美食,文艺,娱乐
名人明星,文艺,娱乐,武艺,午饭
旅游
IT 数码
80 后,新郎新娘,吃货,婚纱礼服,摄影,婚礼,结婚,娱乐,婚庆,时尚
深圳生活,娱乐
文艺,体育,IT 数码
教育就业
时尚,娱乐
星座命理,体育
福建生活,健康,文艺,时尚
星座命理,旅游,健康
新闻趣事,美食,名人明星,视频音乐,搞笑幽默
穿衣美容,文艺
汽车
旅游,汽车,星座命理,IT 数码,时尚
汽车,美食,体育
历史,好书,教育就业,娱乐,旅游,文艺,游戏动漫
美食,旅游,时尚,文艺
娱乐,教育就业
超级轻土,多肉植物,水彩,软陶,插画
娱乐,时尚
军事
搞笑幽默,旅游
视频音乐,微博奇葩,名人明星,娯樂
美女,游戏动漫,汽车,新闻资讯,体育
娱乐
教育就业
美女
星座命理
汽车,娱乐
美食,萌宠,搞笑幽默,新闻趣事,微博奇葩
闲人
文字,上网,宅,旅行,文艺
旅游,文艺
汽车,美女,时尚,文艺,娱乐,体育
娱乐
健康,文艺,美食,时尚,娱乐
重庆生活,娱乐

旅游,星座命理,时尚,游戏动漫,美食
美剧,红色高跟鞋,日天蝎升摩羯,茨威格,情绪化矛盾体,尼采,叔本华,达利
娱乐,星座命理,美食,汽车,IT 数码
美食,投资理财
美食,教育就业,健康,个性,90
娱乐,文艺
星座命理,健康,娱乐
美女
坤迷,大叔控,音樂控,睡觉睡到自然醒,小哇控,爱幻想
IT 数码,游戏动漫
美女,娱乐,IT 数码,军事
健康,新闻资讯,投资理财,美食,旅游
体育,旅游,美食,IT 数码,娱乐
美女
IT 数码,军事,汽车,投资理财
美食,军事,星座命理,健康,娱乐
军事,娱乐,美女,体育
美女,自由职业,时装
假想徒
娱乐
文艺,健康,新闻资讯,美食,旅游
教育就业,健康,旅游,星座命理
文艺
星座命理
文艺,体育,娱乐
汽车,美女
美女,汽车,娱乐,游戏动漫,军事
美食,健康,旅游
星座命理
青岛生活,文艺,娱乐,时尚,美食
爱睡觉,汽车,健康,微博奇葩,旅游,穿衣美容,视频音乐,湖南生活
美食
星座命理,美食,时尚,娱乐,文艺
美女,文艺,体育
娱乐,星座命理,美食,文艺
星座命理
浙江生活,娱乐
娱乐
星座命理,健康,游戏动漫,体育,娱乐,美食
听歌,胡思乱想,唱歌,学生,雙魚座,Music
游戏动漫,健康,IT 数码,文艺,娱乐
教育就业,文艺,娱乐,游戏动漫,大学生，外向
游戏动漫,娱乐,美女,DNF,lol,动画片，乒乓球
新西兰生活,体育

旅游,穿衣美容,萌宠,娱乐,许嵩控
恐怖片,偶像剧,女人,服饰,活泼,互粉,单身,90,独立,蓝色
时尚,投资理财,旅游,美食,健康
动漫控,猫咪,死神,自拍,喜欢唱歌,时尚,旅游,美食,娱乐
新闻资讯,游戏动漫,IT 数码,娱乐
娱乐,时尚,文艺
IT 数码,星座命理,美女,时尚,娱乐
视频音乐
娱乐,新闻资讯,文艺,美食,旅游
新闻资讯,时尚,教育就业,投资理财,娱乐
汽车,旅游,星座命理,美食,娱乐
健康,时尚
军事
湖北生活
美食
娱乐
娱乐,健康
时尚
健康,娱乐,旅游,星座命理
美女
美食
娱乐,汽车,旅游,军事,新闻资讯
文艺
游戏动漫,美食,旅游
美食,旅游,新闻趣事,搞笑幽默
娱乐,讨厌黑,90 後,朴有天唰韩正宇
音乐、电影、运动
体育,爱睡觉,学生,爱音乐,健康,篮球爱好者
健康,美食
IT 数码,美女,新闻资讯,军事,时尚,美食,旅游,娱乐
旅游,新闻资讯,健康,时尚
旅游,湖南生活,微博奇葩,搞笑幽默,IT 数码,游戏动漫,美食,文艺
体育
游戏动漫,时尚,汽车
随手拍,音樂,攻受,布袋戲,腐宅
购物狂
汽车,美食
90 后,军事,旅游,一辈子,最熟悉的陌生人
美食,娱乐
美食
时尚,IT 数码,美食,健康,美女,娱乐
娱乐,汽车,旅游,IT 数码,体育
美女,游戏动漫,时尚,体育,娱乐
娱乐,旅游,美女,汽车,新闻资讯

微博奇葩
健康,投资理财,旅游,美食
旅游,娱乐,游戏动漫,军事,汽车,IT 数码
美食,娱乐,体育,新闻资讯,健康,旅游,星座命理,美食,时尚,文艺
体育
穿衣美容,新闻趣事,教育就业,投资理财,旅游
旅游
文艺,新闻资讯,游戏动漫
旅游,时尚,娱乐,文艺
旅游,文艺
娱乐
美食,低调,爱电影,新闻资讯,健康,军事
新闻趣事,汽车,投资理财
星座命理
时尚,美食,娱乐
IT 数码,美食
旅游,一个人旅行,路游,到,游,路,一
美食
音乐控,K 歌
体育,娱乐
新闻资讯,汽车,教育就业,投资理财
娱乐,美食,投资理财,时尚
80 后的末班车,睡觉睡到自然醒,腐,语录,文艺,娱乐,待解救青年,技术宅,小清新,华仔
视频音乐
旅游,IT 数码
IT 数码,教育就业,时尚,健康,旅游,文艺
旅游
穿衣美容,搞笑幽默
新闻资讯,健康,投资理财,旅游,星座命理
美女
新闻资讯,时尚,教育就业,文艺,健康
投资理财,新闻资讯,汽车,健康,旅游
体育
文艺,恐怖片女王,乐天派,唱 K,技术宅,B 型血水瓶座,80 后辣妈
名人明星,情感生活,美食,新闻趣事,美容服饰,教育就业,时尚,娱乐
微博奇葩,旅游,画画,吃喝玩乐,动漫
名人明星,EXO,孩子气,大叔控,爱玩,阳光,双子座,睡觉睡到自然醒
文艺
旅游,健康,时尚,美食
美食,新闻趣事,微博奇葩,搞笑幽默,游戏动漫,旅游
体育
汽车,体育,文艺,IT 数码
美食,健康
旅游,文艺,笑话

美食
文艺
汽车
星座命理
IT 数码,平常心
汽车,体育,IT 数码,文艺
新闻资讯,军事,IT 数码,娱乐,星座命理
文艺,体育,汽车,美女,IT 数码,娱乐,新闻资讯
IT 数码,投资理财,健康
美女
时尚,健康
美食,健康
文艺,体育
二货、小丑
搞笑幽默
时尚
娱乐,旅游,教育就业,文艺,美食
时尚,娱乐,旅游,健康
玩，旅游
投资理财
新闻资讯,教育就业,时尚,体育,健康,旅游,汽车
美食,体育资讯,新闻趣事,搞笑幽默,汽车,健康,旅游,文艺,体育
文艺,娱乐,旅游
投资理财,教育就业,美女
新闻资讯,美女,体育,健康,汽车,娱乐
投资理财,健康,旅游,星座命理,时尚
韩国代购淘宝店,韩国代购,代购,时尚,美食,旅游,时尚
美食,娱乐,IT 数码,旅游,文艺
视频音乐
健康,投资理财,时尚
教育就业,星座命理,美食,文艺,娱乐
美女,时尚,旅游,星座命理,美食,娱乐,文艺
时尚,文艺,IT 数码,星座命理,美女
love,睡觉,自由,玩,美食,旅游,星座命理,健康,游戏动漫,娱乐
美食,旅游,IT 数码,时尚,娱乐
汽車,遊戲動漫,IT 數碼
视频音乐,旅游,星座命理,新闻趣事,微博奇葩,文艺
时尚,星座命理,军事,文艺,体育,健康,旅游,美食,投资理财
为学生择业,为企业择人,教育就业
旅游,健康,时尚,游戏动漫,文艺
花样美男,吉林生活
体育,时尚,教育就业,新闻资讯,汽车
美食,健康,文艺,娱乐,星座命理
娱乐,游戏动漫,星座命理,旅游,美食,文艺

健康,教育就业,美女,旅游,美食
旅游,体育
时尚,IT 数码,娱乐
健康,旅游,美食,投资理财,教育就业
文艺,星座命理,IT 数码,娱乐
游戏动漫,美女,体育
健康
军事
星座控,唱歌,学生,自由,动漫,囧,搞笑,吃货一枚,白羊,神经质
美食,新闻资讯,IT 数码,娱乐,汽车,体育
军事,文艺,IT 数码
穿衣美容,新闻趣事,星座命理,美食,旅游,教育就业
星座命理,游戏动漫
健康,旅游,美食
吃货,娱乐,自由,白羊座,市场营销,90 後,爱旅游,快乐,影视,喜欢听歌
汽车,今夕何夕
汽车
视频音乐,健康,投资理财,IT 数码
美食,娱乐
娱乐,体育,时尚,星座命理,美食
IT 数码,投资理财
体育,文艺
娱乐,投资理财,健康,旅游,汽车,军事,13681421626
汽车,时尚,旅游,健康,文艺
旅游,IT 数码,汽车
美食,篮球,吃喝玩乐,日语,火影忍者,无厘头,旅游
美食
搞笑幽默
投资理财,美女
星座命理
体育
星座命理,搞笑幽默,娱乐,美女
时尚,旅游,星座命理,美食,娱乐
IT 数码
时尚,美食
新闻资讯,教育就业,军事
军事,旅游,汽车,娱乐,体育
投资理财,美食,时尚,文艺,娱乐,健康,旅游
文艺,善良,射手座,走走看看,游戏动漫,旅游,汽车,时尚,娱乐
旅游,美食,新闻资讯,文艺,健康
娱乐
时尚,IT 数码
美女,娱乐,时尚,旅游,美食
健康,娱乐

体育
IT 数码
新闻资讯
名人明星,视频音乐
赚钱
潮,名人明星,穿衣美容,旅游
名人明星,八卦杂谈,新闻趣事,校园生活,视频音乐,投资理财
IT 数码,娱乐
时尚,娱乐,文艺,旅游,健康
星座运势,游戏动漫
美食,新闻趣事,搞笑幽默
文艺,体育,娱乐
K 歌,林俊杰,体育
军事
游戏动漫
IT 数码,美食,旅游,时尚,健康
美食
时尚,娱乐
旅游,文艺,时尚,美女
搞笑幽默,连接服务器失败,狮子座
吃货一枚,music,神经质,热爱生活,恐怖片,武艺,dance
军事,美食,游戏动漫,健康,投资理财
投资理财,娱乐
时尚,旅游,星座命理,美食,娱乐
小资,发呆,美食,懒人,书,教师,欧美控,美剧,偶喜欢玩,个性
旅游
美食,娱乐
健康,体育,娱乐
旅游
旅游
星座命理
时尚,旅游,美食,星座命理,文艺
懒,旅游,美食,娱乐
旅游,星座命理,游戏动漫,美女
IT 数码
美食
美食,新闻资讯
时尚
穿衣美容,游戏动漫,时尚,旅游,星座命理,娱乐
新闻资讯,游戏动漫
娱乐
健康,体育,娱乐
上网,仁义,爱吃零食
旅游,娱乐,汽车

投资理财,美食,星座命理,旅游,健康
投资理财,美食,军事,IT 数码
74 先森,音乐信仰,王乃恩,便是晴天,你若安好
旅游
健康,旅游
时尚,教育就业,娱乐,体育,文艺
星座命理,IT 数码,体育
创意,爱生活,85 后
旅游,娱乐
美女,娱乐
军事,汽车
星座命理,文艺,旅游,美食
时尚,旅游,文艺,娱乐,美食
旅游,文艺,时尚,星座命理,娱乐
健康
健身,学习
星座命理,健康
开朗
新闻资讯,投资理财,旅游,IT 数码
美女,教育就业,投资理财,娱乐,体育
新闻资讯
美食,健康
美食
文艺,健康,美食
军事
美女
校园生活,新闻趣事,美食,茂名生活,搞笑幽默,教育就业
健康,冷漠無情便是王
时尚
文艺,娱乐,体育,教育就业,汽车
体育
时尚
美女,娱乐
美食,旅游,健康
游戏动漫,技术宅,音乐控,体育,文艺,新闻资讯,IT 数码
汽车,投资理财
游戏动漫,美食,汽车,娱乐
萌宠,广东生活,视频音乐,搞笑幽默
IT 数码,体育
双子座,偶稀饭睡觉觉,教育就业,时尚,新闻资讯,健康,旅游
美女
文艺
军事
旅游,娱乐

娱乐
美食,时尚
文艺
教育就业,美食,健康,IT 数码,文艺,体育,娱乐
游戏动漫,旅游,IT 数码
美容服饰,六安生活,星座运势
体育
文艺,体育,娱乐
时尚
健康,新闻资讯,美食,体育,娱乐
双重性格,懒,白羊座,浪漫,柯南
体育,白袜,游戏动漫,IT 数码,文艺,美食,娱乐
旅游,IT 数码,时尚,美食,教育就业
健康
军事
旅游,英语,翻译爱好者,字幕组,WongFu,WongFuPro,微电影,短片
游戏动漫
旅游,时尚,娱乐
美食
星座命理,娱乐
美食
军事,汽车,旅游
90 后,学生,路痴
看电影,英语,爱生活,热情,少女时代,爱唱歌,化妆品,投资理财,自拍,心情
汽车,旅游,体育
健康,新闻资讯,旅游
陈奕迅,音乐爱好者,随性,茉莉,星座控,运动,慢热,温柔,简单,坚定
体育
游戏动漫
美食,旅游,娱乐
时尚,旅游,美食,文艺,娱乐
穿衣美容,美食,时尚,文艺,娱乐,星座命理
美图摄影
文艺,汽车,美食,旅游,娱乐
健康,新闻资讯,娱乐
健康,旅游,美食,军事,体育
娱乐,健康
星座命理
游戏动漫,投资理财,文艺
情感生活,时尚
山东生活
名人明星
读书分享,美容服饰,新闻趣事,搞笑幽默,武汉生活,投资理财
体育,娱乐

文艺
美食,美女
情感生活
星座命理
投资理财,新闻资讯
IT 数码,旅游,美食,体育,娱乐
IT 数码,娱乐,旅游,美食,军事
美食,新闻趣事,搞笑幽默,江苏生活,娱乐,投资理财,旅游,IT 数码,体育
星座命理,美食,文艺,美女,娱乐
文艺,解决方案,整体经营,管理咨询,经营管理,中小企业
旅游,美食
军事
体育
旅游,体育
萌宠,名人明星,游戏动漫,体育
自信,胡思乱想,爱生活,创意,阳光,单身,小清新,涂鸦,小资
娱乐
天津生活
健康
健康,旅游
娱乐
美女,健康,军事,旅游,IT 数码
星座命理
90 後,周杰伦,欧美音乐,打球,性感,旅游,美女,汽车,体育,IT 数码
游戏动漫,娱乐,星座命理
美女
娱乐,旅游,美食,美女,汽车
星座命理,文艺,时尚
旅游,数据中心,虚拟化,云计算
美食,娱乐,星座命理
美食,时尚,旅游,娱乐
游戏动漫,汽车,旅游,军事,IT 数码
星座命理,投资理财,健康,时尚,汽车
娱乐
旅游
时尚,文艺,星座命理,美食,娱乐
美食,萌宠
汽车,健康,时尚,星座命理,美食,娱乐
视频音乐
体育,美女,新闻资讯
旅游,投资理财,IT 数码,健康,新闻资讯,教育就业
fashion,时装,金融,购物狂,时尚
旅游
教育就业,时尚,健康,新闻资讯,旅游,文艺,娱乐

教育就业,时尚,汽车
文艺,时尚,游戏动漫,娱乐,美食
美女,旅游,文艺
娱乐,美食,文艺,时尚
时尚,旅游,健康
搞笑幽默,微博奇葩,穿衣美容,视频音乐,健康
投资理财,星座命理
美女,旅游,星座命理
娱乐,投资理财,文艺,健康,旅游
体育
美食
美食
美食
最爱孙燕姿,林峰
IT 数码,海鲜,精品粤菜,美食
投资理财,体育
名人明星
美食,教育就业,健康,新闻资讯
旅游
美食
新闻资讯,游戏动漫,美女,星座命理,军事
娱乐
时尚,健康
娱乐,美女,健康,星座命理,时尚,美食
听音乐,学生一族
时尚,健康,旅游,文艺,娱乐
旅游
旅游,星座命理,IT 数码,娱乐
文艺,动画短片,游戏动漫
我就是我,永远的晴天,教育就业,美食,投资理财,体育,娱乐,时尚,健康
中山生活,体育
感性,自然,爱猫,爬山,羽毛球,电影控,火影忍者,画画,音乐,健康
山西生活
健康,汽车,旅游,军事,IT 数码
美食,时尚,投资理财,星座命理,健康
新闻资讯,健康,体育,军事
文艺,娱乐,旅游,健康,IT 数码
军事
旅游,体育,娱乐,美食
健康
美食,星座命理,穿衣美容,搞笑幽默,四川生活
旅游,投资理财,健康,军事,IT 数码
运动,自信,心理,美食,健康
搞笑幽默,旅游,星座命理,美食,文艺,娱乐

新闻资讯,旅游,美食,文艺,娱乐
时尚,IT 数码,新闻资讯,体育,汽车
美食,文艺,IT 数码,娱乐,体育
美食,喜欢旅游,爱蓝波
旅游,美女,新闻资讯,健康,军事
浙江生活
健康,旅游,美食
美女
插画师,首饰,Gothic,美式复古,唱片收藏
健康
脾气差差,手机控,懒人爱睡觉觉,射手座,小说控,动漫控,音樂,旅游,美食,娱乐
投资理财,汽车,旅游,美食,体育
时尚
爱旅行,网游,游戏动漫,汽车,美女,IT 数码,娱乐
新闻资讯,旅游,健康,美食,娱乐
娱乐
新闻趣事
穿衣美容,时尚
游戏动漫,IT 数码
美食,军事,IT 数码,文艺,旅游,汽车,游戏动漫,美女,体育
旅游,汽车,游戏动漫,美食
美女
旅游,时尚,娱乐,美食
星座命理,旅游,娱乐,文艺,时尚,军事
体育资讯,篮球迷,室内设计
车世界,水瓶座,房地产,德国,创业,摄影控,金融,红酒,摄影爱好者,新闻
星座命理,美食
上海,地产,外贸,艺术
美食爱好者,电影迷,旅游
时尚,星座命理
旅游,健康
旅游,健康,美女,时尚,娱乐,美食
美女,教育就业,旅游,投资理财,体育
教育就业,时尚,健康,星座命理,美食
搞笑幽默,美食
时尚,娱乐
旅游
时尚,完美主义,低调,开心,懒人,宠物,90 後
时尚
学习
体育,美食,文艺,旅游,娱乐
星座命理
健康,美食,汽车,时尚,美女
90 后的宅女,骑车,乒乓球,休闲,K 歌,星座命理,视频音乐,美食,其他生活,穿衣美容

健康
新闻趣事,财经资讯,健康养生,安徽生活
情绪化,怀旧,偶稀饭睡觉觉,安静
投资理财
娱乐
生态甲鱼,野生甲鱼,甲鱼宠物,甲鱼零售连锁,美食
娱乐
娱乐,时尚
美食
汽车,旅游
军事,娱乐,旅游,健康,体育
新闻资讯,旅游,美食,汽车
美图摄影,北京生活,校园生活,完美主义,电影控,星星,美食,文艺,体育,娱乐
八卦,音乐达人,电影迷,爱幻想,k 歌,动漫,90,美食控,爱旅游,古灵精怪
娱乐
健康
成都生活,娱乐
体育
汽车
体育
星座命理,游戏动漫,IT 数码,教育就业
投资理财,新闻资讯,旅游,军事,体育
微博奇葩
健康,时尚,旅游,美食,娱乐
视频音乐,体育
汽车,旅游,体育
新闻资讯
穿衣美容,名人明星,视频音乐,刘昌贤 RICKY,90 后非脑残,权志龙 BigBang
新闻资讯,旅游,文艺,时尚
名人明星,善良,喜欢旅游,开心,金牛座,小清新,学生一枚,广州人,exo,娱乐
健康,文艺,旅游,美食,游戏动漫
美女
健康,星座命理
星座命理,文艺,娱乐,时尚,旅游
IT 数码,文艺,体育,娱乐
美女,新闻趣事,微博奇葩,军事,汽车,游戏动漫,旅游
美图摄影,双子,喜欢听歌,重庆,时尚,汽车
美食
教育就业,投资理财,军事,新闻资讯,美女,游戏动漫,旅游
美女
文艺,投资理财,时尚,健康,旅游
旅游,健康
新闻资讯,投资理财,IT 数码,娱乐,旅游
娱乐,健康,旅游,美食,文艺

萌宠,美食,旅游,名人明星,新闻趣事
新闻资讯,星座命理,娱乐,教育就业,投资理财,美女,游戏动漫,文艺
美女
美女
星座命理,微博奇葩,搞笑幽默,纠结,时尚,健康,娱乐
星座命理,美食,IT 数码,文艺,娱乐
游戏动漫,旅游,IT 数码
蓝色,巨蟹,爱美食,90 后但我不脑残,单纯
搞笑幽默
动漫,狗狗,美术,梦想,游戏动漫,星座命理
娱乐
时尚,新闻资讯,娱乐
玩儿,健康,星座命理
汽车
温州生活,体育,娱乐,美食爱好者,睡懒觉
IT 数码,美食,娱乐,新闻资讯
旅游,星座命理,体育
文艺,时尚
游戏动漫,体育
文艺
娱乐
美食
美食,健康,娱乐,时尚,投资理财
旅游
IT 数码,文艺
时尚
投资理财
游戏动漫,汽车,美食,体育,娱乐
旅游,美食,文艺
健康,教育就业,星座命理
星座命理
时尚,投资理财,健康,旅游,汽车
重庆生活
新闻资讯,旅游,美食,文艺
旅游,体育
美女
毛豆米
运动,乐观,交友,新闻资讯,美食,健康,旅游,星座命理
投资理财,教育就业,游戏动漫,娱乐,体育
汽车,美女,体育,娱乐
爱猫,爱旅行,爱夜生活,爱短发,爱生气,爱港星,爱电影,爱冲动,BEYOND,天蝎
福建生活
军事
旅游,美食,健康,学生,90 後,射手座,坚强,吃货一枚,篮球,我就是我

健康,旅游,美食,娱乐
军事,IT 数码
新闻资讯,投资理财,IT 数码,游戏动漫,时尚,教育就业,文艺,娱乐
新闻资讯
健康,娱乐
美食
汽车,旅游,IT 数码,体育
旅游,汽车
星座运势
美食
移动互联网,PHP,IT,媒体,微博控,技术宅,科技
IT 数码,美女
文艺,旅游,游戏动漫,美女,美食
娱乐,新闻资讯,IT 数码,体育,游戏动漫
体育
美食,旅游,时尚,教育就业,健康
汽车,时尚,文艺
IT 数码,时尚,汽车,健康,旅游
娱乐,健康,美食
新闻资讯
体育,汽车
娱乐
健康
教育就业,娱乐,汽车,文艺
游戏动漫,娱乐
旅游
投资理财,娱乐
教育就业,时尚,健康,星座命理,IT 数码,文艺,汽车,旅游,美食
不解释、不掩饰!
游戏动漫,投资理财,娱乐
投资理财,新闻资讯
旅游,体育,娱乐
投资理财,体育,新闻资讯,教育就业
文艺,教育就业
旅游
美食,投资理财
体育,游戏动漫
文艺,娱乐
美食,时尚,IT 数码,娱乐
时尚
新闻资讯
体育
黄家驹
投资理财,军事,体育

新闻资讯,星座命理,IT 数码
军事
星座命理,投资理财,娱乐,IT 数码,美食,时尚,文艺,旅游
健康,娱乐,投资理财,新闻资讯,文艺
时尚
旅游
时尚,教育就业,健康,星座命理
时尚,新闻资讯,旅游,军事,文艺
时尚,旅游
时尚,健康,旅游,文艺,娱乐
美食,娱乐,文艺,时尚,汽车
文艺,投资理财,健康,美食,娱乐
搞笑幽默
游戏动漫
健康
美食
投资理财,美食,旅游,健康
娱乐,军事,投资理财,教育就业,健康,新闻资讯,体育
新闻资讯,美女,旅游,娱乐,IT 数码
汽车,娱乐,投资理财,时尚
投资理财
汽车,健康,旅游
投资理财
新闻资讯
游戏动漫,健康,IT 数码,军事,娱乐
娱乐
IT 数码
时尚,体育,文艺
游戏动漫,星座命理,IT 数码
旅游,星座命理,IT 数码
美女,IT 数码,创业,软件,感性,赚钱,水瓶,教育,幽默,淘宝控
时尚,教育就业
新闻趣事,搞笑幽默,IT 数码
投资理财,美食,文艺
汽车,投资理财,IT 数码
汽车
投资理财,体育
新闻资讯,娱乐,美食,IT 数码
美食,美女,汽车
航模,摄影,旅游,美食,时尚,汽车,IT 数码
名人明星
新闻趣事,投资理财
旅游,美食
搞笑幽默,新闻趣事,美食,旅游,星座命理,健康

汽车
游戏动漫,娱乐,旅游,美食,文艺
旅游
文艺,健康,教育就业,投资理财,美食,娱乐
美女,游戏动漫
美食
吃吃更健康!!!!
搞笑幽默,娱乐,投资理财,IT 数码
旅游,美食
美女,旅游,体育
新闻资讯,旅游,娱乐,美女,时尚
体育,汽车
萌宠,微博奇葩,搞笑幽默,名人明星,游戏动漫,汽车,美食,文艺,体育
健康,新闻资讯,娱乐
名人明星,文艺,时尚,娱乐
美食
新闻资讯,文艺,娱乐
视频音乐,台湾生活,旅遊,美食,娛樂,文藝
投资理财,军事,娱乐,汽车
旅游,星座命理,美食,文艺,娱乐
汽车,旅游,文艺
星座命理,美食,健康,美食,时尚
旅游
娱乐
灿灿的乖乖,M,SM,爱口红的小清新,90 后但我不脑残,高跟鞋控
娱乐
文艺
IT 数码,旅游,美食,体育
云南生活,汽车,文艺
游戏动漫,体育
新闻资讯,投资理财
汽车,教育就业,军事,娱乐,吃,乐观,幽默,交友,游戏
微博奇葩
旅游,星座命理,穿衣美容,美食,搞笑幽默
时尚,美女
星座运势
IT 数码
搞笑幽默,新闻资讯,音乐迷,80 后,无厘头,韩国,穿衣美容
军事,体育,文艺,IT 数码,娱乐
旅游,美食,时尚,娱乐
健康,投资理财
游戏动漫,汽车,星座命理
投资理财,星座命理
美食,文艺,时尚,健康

美女,美食,旅游,游戏动漫,体育
时尚,旅游
美食,体育
文艺,时尚
娱乐
跳舞
娱乐
投资理财,娱乐,IT 数码,体育
文艺
美食
娱乐
旅游
娱乐
IT 数码
电影控,懒人,爱旅行,天秤
美食,旅游,星座命理,时尚
星座命理
汽车
美女,游戏动漫,时尚,文艺,娱乐
健康,新闻资讯,教育就业,投资理财,旅游
画画,爱睡觉,舞蹈,逛街
旅游
健康,新闻资讯,美食,娱乐
新闻趣事,健康
80 後,军事,娱乐,美女,体育,投资理财
游戏动漫,投资理财,健康
IT 数码,时尚,美女,汽车,设计
美食,娱乐,体育,汽车,IT 数码
阳光,好动男
美食,宅,爱旅游,双子女,爱吃
时尚,娱乐,美食
搞笑幽默,汽车
美食
教育就业
视频音乐,汽车
好性格,单纯,睡懒觉,音乐控,简单,休闲,看电视,偶像剧,宅女,射手
文艺,汽车,时尚,旅游,娱乐
美食,旅游,健康,投资理财,美女
教育就业,新闻资讯,时尚,旅游
旅游
健康,美食,娱乐
军事,文艺,体育,娱乐
时尚,健康,旅游,星座命理,文艺
娱乐

星座命理,时尚,旅游,美食,文艺
美食,投资理财,时尚,汽车,文艺,健康
美食
健康,教育就业,美食
文艺,投资理财,教育就业,汽车,美女,时尚,IT 数码,美食,旅游,健康
美食
游戏动漫,体育,旅游,IT 数码
美食,娱乐,体育
星座命理,游戏动漫,时尚,美女,娱乐
美女,星座命理,美食,娱乐
IT 数码
澳大利亚悉尼,澳洲生活,留学澳洲,澳洲移民,教育就业
开心,公益,音乐迷,视频音乐,移动互联网,朋友,多愁善感,滑雪,开朗
星座命理,美食,文艺,娱乐
IT 数码
健康,旅游,美食,时尚,教育就业,娱乐,IT 数码
旅游,文艺,娱乐
首饰达人,首饰,穿衣美容,美食,CHANEL,美国代购,包包,时尚
美女
美食
美食,旅游,星座命理,文艺
时尚,娱乐,美女
美食,文艺
星座命理,文艺,娱乐,美食,游戏动漫
娱乐
IT 数码
文艺,时尚,美女
新闻资讯
体育,健康,新闻资讯,汽车,军事,IT 数码
文艺,教育就业,娱乐,健康,旅游
美女,游戏动漫,旅游,军事
美女,新闻趣事,微博奇葩,搞笑幽默,新闻资讯,投资理财,教育就业,体育,IT 数码
健康,美食
旅游,游戏动漫,文艺,美食
星座命理,美女,新闻趣事,旅游,娱乐,健康,美食,IT 数码
时尚,汽车,文艺,娱乐,新闻资讯
游戏动漫,投资理财,汽车,IT 数码,军事
校园生活,游戏动漫,梦想,网游,善良,游戏,吃,爱微博,爱生活,我的青春我做主
真爱方卓莹,男神张艺兴,余凯伦 YUKAILUN
旅游
交友,打球,南京,80 后的尾巴,学生,运动,新闻资讯,教育就业,美食,体育
健康,军事
旅游
娱乐

新闻资讯
汽车,IT 数码
名人明星,双鱼座,矛盾体
苏打绿,喜欢旅游,music,星座命理,文艺,小清新,购物狂,手机控,柔情
IT 数码
体育
广东生活,娱乐
旅游,体育,IT 数码,军事
娱乐,游戏动漫,IT 数码,美食
教育就业,体育,美女,游戏动漫
学生,跆拳道,听歌,无厘头,夜猫子,90 後,手机看小说,减肥,爱美食,吃货
时尚,美食,健康,娱乐,文艺
美食,新闻资讯,健康,投资理财,娱乐,IT 数码
娱乐,军事,健康
娱乐,时尚,星座命理,健康
健康,星座命理,美食,文艺,娱乐
时尚,游戏动漫,旅游,美食,文艺
情感生活,爱美食,咖啡,电影迷,滑雪,小提琴
健康,时尚,新闻资讯
体育,美食,旅游,IT 数码
教育就业,娱乐,星座命理,汽车,游戏动漫
娱乐
娱乐,军事
穿衣美容,时尚,旅游,美食,星座命理,文艺
教育就业,健康,旅游,美食,文艺
动漫
旅游
星座命理,娱乐
教育就业,星座命理
文艺,宅女一枚,sj,大学生,艺术,阳光,微笑,爱睡觉,青春,娱乐
健康,美食
体育
军事
聊天
IT 数码,美食,健康,娱乐,文艺,新闻资讯,投资理财
疯一样的驴子
军事
教育就业,投资理财,新闻资讯,健康,旅游,星座命理,IT 数码
健康,教育就业,星座命理
健康,旅游,汽车,教育就业,投资理财
娱乐,星座命理,教育就业,时尚,旅游,文艺,美食,懒,80 后
星座命理,军事,娱乐,健康,体育
文艺,体育
时尚,投资理财,IT 数码,美食,娱乐

时尚,健康,旅游,娱乐,美食
美食
游戏动漫,电影,电影　小说　音乐,美食
娱乐
美食,双子,大学生,爱自由,猫,宅
旅游,体育
游戏动漫
美食,旅游,健康
旅游
星座命理,旅游,美食,文艺
时尚,教育就业,娱乐,文艺
时尚,星座命理,旅游,美食,IT 数码
快乐,科技
学生一枚,舞蹈,睡懒觉,吃货,听音乐
IT 数码,美食,旅游,汽车,体育
美女,健康
考前认真党,忠实的内拉,爱睡觉,发呆 ing
文艺,游戏动漫
教育就业
美食,文艺
新闻资讯,时尚,旅游
健康,新聞資訊,投資理財,旅遊,美食
美食,军事,星座命理
文艺,星座命理,IT 数码,军事,汽车
娱乐,文艺,旅游,美食
投资理财,汽车,IT 数码,新闻资讯,旅游,美食
爱情,美食,美女,体育
新闻资讯,时尚,教育就业,旅游
星座命理,娱乐,体育,文艺
旅游,美食,文艺
健康,美食,旅游,体育,娱乐
宝宝,亲情,旅游美食,爱旅游,家居,美食
美食,星座命理,美女
健康,新闻资讯,文艺,娱乐,美食
任性,感性,超级感性,情感,思考,爱自由,留学,书,听音乐,旅游
星座命理,旅游,娱乐,体育,时尚,游戏动漫,文艺
健康,体育
投资理财,IT 数码
超短线,股市分析,理财,股市,股票
音乐爱好者,爱玩,执着,天蝎女,投资理财,旅游,美食
IT 数码,健康,新闻资讯
新闻资讯,星座命理,娱乐
美女,旅游,文艺,娱乐,IT 数码
娱乐,时尚,健康,文艺,美食,星座命理

体育,投资理财,教育就业,文艺,音乐
健康,文艺,时尚,旅游
新闻资讯,时尚,娱乐,星座命理,汽车
时尚,旅游,IT 数码,美食,娱乐,动画,90 後,美食爱好者,喜欢听歌,星座命理
美女,军事,美食
投资理财,健康,时尚,新闻资讯,星座命理
体育
新闻资讯,美女,美食,旅游,游戏动漫
搞笑幽默,娱乐
娱乐
搞笑幽默
教育就业,星座命理,旅游,时尚,美女
慢熱,冬天,獨立,基督徒,藍色,不倒翁,藝術
娱乐
体育,星座命理,美女,汽车
游戏动漫
洛阳生活,投资理财,美食,旅游,星座命理
鄞州音乐培训,小提琴培训,电子琴培训,艺术培训,少儿音乐,高考音乐培训,吉他培训,古筝培训,钢琴培训,教育就业
新闻趣事,娱乐
娱乐,IT 数码,新闻资讯,投资理财
江西生活,搞笑幽默,名人明星,体育,军事
娱乐
旅游,健康,时尚,美食,娱乐
娱乐
健康,娱乐,汽车,旅游,美食
美女,时尚,IT 数码,汽车,游戏动漫,体育
喜欢听歌,淡定,医生,投资理财
健康,时尚,旅游,娱乐
美食爱好者,热爱生活,敏感,文学,微博,旅游
健康
旅游,教育就业,IT 数码,军事,娱乐,体育,汽车
旅游,游戏动漫,文艺
旅游,IT 数码
it 互联网,旅游爱好者,打篮球,80 後,自然,坚强,美女,IT 数码,汽车,体育
时尚,新闻资讯,教育就业,体育
时尚,美食
健康,IT 数码,娱乐,美食,双重性格,星座命理
搞笑幽默,旅游,星座命理
体育
时尚,旅游,教育就业,健康,新闻资讯,投资理财
小清新,时尚,旅游,星座命理,文艺
欧美音乐,书,电影爱好者,绘画,休闲,咖啡,小清新,英格兰,DIY,摄影控
美女,投资理财,游戏动漫,教育就业,IT 数码

娱乐,屌丝
健康,教育就业,美食
娱乐,坚强,笑,喜欢听歌,热爱生活,阳光,乐观,宅女
体育,美食
新闻趣事,搞笑幽默,名人明星,美食,时尚,健康,旅游,文艺
美图摄影,读书分享,星座运势,视频音乐,美食
娱乐,时尚,美女
新闻趣事,教育就业
娱乐,体育,时尚,文艺
旅游摄影,偶喜欢玩,摄影爱好者,旅游
美食,it 互联网,影视,宅女一枚,爱吃,星座命理,娱乐,旅游
游戏动漫,娱乐,体育
体育,娱乐
星座命理
美食,旅游,文艺,健康,新闻资讯
教育就业,文艺,娱乐,旅游,美食
旅游
平常心
天枰座,美食,星座命理,IT 数码
文艺,体育,汽车
穿衣美容,视频音乐,搞笑幽默,旅游,汽车,美食,IT 数码,健康
健康,投资理财,星座命理,IT 数码,娱乐
ELF,李赫宰,90 后,superjunior
游戏酷爱者,外貌协会 VIP,爱音乐爱生活
陕西生活,星座命理,时尚
金属党,海贼王,电影,摇滚乐,金牛座,闷骚,旅游
科密湖密,体育
美食,健康,新闻资讯,教育就业,体育
体育
游戏动漫
投资理财,星座命理,美食,文艺,健康,旅游,时尚
军事
微博奇葩,搞笑幽默,游戏动漫,娱乐
文艺,体育
娱乐,美食
IT 数码,美女,游戏动漫,文艺
萌宠,娱乐,时尚
旅游,星座命理,会计,神经质,爱好，太爱他了
IT 数码,教育就业,新闻资讯,投资理财,娱乐,旅游,汽车
军事,新闻资讯,娱乐,时尚,美女
军事,体育
熊熊熊熊熊
美女,时尚,星座命理,旅游
娱乐

健康
健康,时尚,IT 数码,文艺,美食
时尚,健康,美食,新闻资讯,娱乐
搞笑幽默,娱乐
旅游,娱乐,美食,投资理财,健康
健康
淘宝,购物,交朋友,可爱控,盟,时尚,手机党,手机控,手机壳,订做手机壳
星座命理
娱乐
投资理财,IT 数码,时尚,健康,星座命理,娱乐
战略忽悠局,皇马,爱伦坡
健康,娱乐
名人明星,美食,星座命理,穿衣美容,娱乐
游戏动漫,时尚,健康,旅游,美食,IT 数码
新闻资讯,旅游,美食,星座命理,文艺,时尚,娱乐
美食,名人明星,旅游,美女,视频音乐
文艺,娱乐
体育,美食,新闻资讯,美女,游戏动漫,时尚
聊天,咖啡,敏感,自信,时装,独立,完美主义者,00 后,广东生活,文艺
娱乐,旅游,健康,星座命理,美食
美食,娱乐
教育就业,星座命理,文艺,美食
文艺,时尚
时尚,文艺
时尚,宽松女装,时尚潮流女装,胖 MM 女装,大码女装,韩版女装
美食,旅游,星座命理,时尚,娱乐
文艺,美食
文艺,娱乐
娱乐
偶稀饭睡觉觉,天秤,胡歌,夜猫子,旅游,投资理财,星座命理,美食,娱乐
财经,爱旅游,体育资讯,车世界,军事天地,吉他,猫控,赛车,游戏动漫
汽车,IT 数码,游戏动漫
美食
美食,文艺,健康,时尚,旅游,星座命理,娱乐
旅游,新闻资讯,星座命理,文艺,娱乐
娱乐
旅游,美食,穿衣美容,视频音乐,新闻趣事
汽车,教育就业,健康,军事,体育
健康
娱乐,美女,投资理财,健康,时尚
健康,新闻资讯,教育就业,投资理财,星座命理
美女,健康,星座命理,时尚,IT 数码
90 后,吃货,双鱼座,手机控,喜欢音乐,懒人
健康,新闻资讯,美食,IT 数码,娱乐

双子座,漫画
文艺,健康,投资理财,娱乐,教育就业
IT 数码,文艺,体育,娱乐,腐,新闻资讯,美女,汽车,教育就业,投资理财
健康,娱乐,美女,时尚,旅游
天秤座,单身,茶,旅游摄影,艺术,浪漫,紫色,英语,娱乐,星座命理
体育,时尚,汽车,IT 数码
教育就业
健康,美食
共青团,微博控,志愿者
体育
文艺,娱乐
娱乐
旅游,体育,新闻资讯,军事,娱乐
文艺
美食,美女,天津生活,视频音乐,星座命理
时尚,星座命理,美食,文艺,娱乐
时尚,文艺,投资理财
美食
美食,投资理财
旅游,健康,美食,星座命理,娱乐
旅游,文艺
微笑,电视剧,巨蟹女,美食控
电影,80 后,哼歌,美食,玩儿,双子座,美剧《lost》,俄语,旅游,雷霆 35 号
时尚,文艺,娱乐,旅游,星座命理,美食,IT 数码
星座命理
文艺,新闻资讯
时尚,军事
娱乐
鞋子包包控,时尚,淘宝掌柜,隐形眼镜专卖,美瞳卖家
旅游,文艺
时尚,健康,娱乐
美食,娱乐,体育
旅游,星座命理,新闻资讯,文艺,娱乐,健康,投资理财
诗歌,双重性格,喜欢音乐,外表主动党,内心被动党,外表柔弱,内心坚强,卡哇伊,爷们儿,开朗
穿衣美容,北京生活,健康,90,猫咪,麦霸,古灵精怪
外贸,猫咪,政治,睡懒觉,书籍,音乐达人,人生,健康,旅游,美食
投资理财
新闻资讯,旅游,游戏动漫,投资理财,美食,体育
文艺
汽车
花样美男,文艺
爱自由,看小说,喜欢音乐,安静,阳光,创意,爱睡觉
减肥,旅游,星座命理,美食,文艺,开朗,90 后但我不脑残

投资理财,娱乐,健康,新闻资讯,美女
星座命理,汽车,旅游,军事
旅游,体育,游戏动漫
旅游
军事,旅游,文艺
美食
健康,旅游,星座命理,美食,汽车
时尚
美女
文艺,IT 数码,娱乐
星座命理
教育就业,IT 数码
处女座的女生,宅,喜欢听歌,热爱生活,完美主义,电影控,音乐爱好者,教育就业
文艺
穿衣美容
旅游,文艺,教育就业,娱乐
勋耶嘿,鹿哈尼,克里斯缇娜,吃货,微笑,咖啡,动漫,猫咪
军事,旅游,体育,游戏动漫
汽车,教育就业,投资理财,新闻资讯,健康
时尚,健康,星座命理,IT 数码,娱乐
军事,文艺,体育,娱乐,旅游
游戏动漫
憎恨欺骗,选择性失忆症,没心没肺傻女人,乐天派,美剧,巧克力,星座命理,时尚,娱乐,文艺
旅游
KAI,金钟仁
娱乐
旅游,娱乐,文艺,汽车,IT 数码
投资理财,汽车,文艺,娱乐,军事
瓶子座,黑皮肤,乐观,篮球,唱歌是我的爱好
美女,娱乐,游戏动漫
游戏动漫,文艺
上海生活,军事,单身
IT 数码
娱乐,健康
上海生活,新闻资讯,文艺
八卦杂谈,旅游
娱乐,军事,体育
新闻资讯,健康,军事,投资理财
娱乐
游戏动漫,新闻资讯,娱乐,体育,文艺
爱生活,爱睡觉,科比,微笑,小女人,时尚,旅游,文艺,娱乐
文艺
星座命理,旅游,时尚,汽车,IT 数码
小清新,旅游,美食,IT 数码

音樂,娱乐,唱歌,拍照,比较广泛
情感生活,美食
美女
画画,b 型血,音乐爱好者,文字控,唱 K,旅游,美女,时尚,汽车,娱乐
旅游,军事,IT 数码,体育,美女
superjunior,单身,90 后,吸血鬼,重庆,胡思乱想,跳舞,90 後,腐,星座命理
健康,星座命理,美食,时尚,投资理财,娱乐
美食,旅游
星座命理
体育,新闻资讯,汽车
旅游,萌宠,文艺,时尚
汽车,健康,美女,体育
美女,健康,美食,时尚,军事,体育
军事
美女
美食,旅游,新闻资讯,投资理财,汽车,IT 数码
IT 数码
名人明星,超人不会飞,潮人,90 后,单个眉毛会动,阿狸控,柯南迷,文字控,水瓶座,唐不苦先森
美女,游戏动漫,星座命理,军事,IT 数码,文艺
健康,时尚,投资理财,娱乐
军事
美食
文艺,旅游,投资理财,美食
投资理财,美食,时尚,健康,旅游
IT 数码,文艺
游戏动漫,娱乐,星座命理
萌宠,星座命理
汽车,健康,军事,美食,休闲生活
美食,教育就业
时尚,娱乐
时尚,新闻资讯
wow,体育,nba
投资理财,新闻资讯
美食
娱乐
文艺,IT 数码,汽车,娱乐
健康,美食,时尚
北京生活,投资理财
投资理财,体育,汽车,星座命理,军事
旅游,体育
萌宠
时尚,健康,美食,文艺,旅游
旅游,星座命理,美食,投资理财,健康,娱乐

投资理财
健康
投资理财,文艺,新闻资讯,汽车,军事
旅游,美女,游戏动漫,美食,体育
游戏动漫,军事,文艺,娱乐
美食
娱乐,星座命理,时尚
美食
新闻资讯,时尚,旅游,军事,IT 数码
美女
体育
投资理财
旅游,投资理财,汽车
新闻趣事,互联网,站长工具,站长网,站长,天羽,IT 数码
汽车,美女,军事,新闻资讯,娱乐,体育,文艺
圣诞公公,藏心爷爷,多重性格的双子,男道主义,唯美主义,个人主义者,慢热,汽车
美食,萌宠,星座命理,微博奇葩,新闻趣事
娱乐,时尚,旅游,星座命理,美食,文艺
美女
汽车
旅游
汽车
时尚
旅游,IT 数码,游戏动漫,娱乐
汽车
美食,时尚,旅游,星座命理
体育
旅游
娱乐,时尚,美食
美食,娱乐
体育
文艺,游戏动漫,美食,旅游
时尚
旅游
社会,登山,读书,心情,医生,教育就业,星座命理,旅游,健康,文艺
狮子狗,屌丝,lol,wow,混日子
旅游,投资理财
健康,旅游,星座命理,美食,娱乐
学霸,机器人,学生,幽默,单机,网络,健康,游戏动漫,娱乐,IT 数码
聚尚网,电影,宅男,NBA,阅读,微博,读书,IT 数码,旅游,体育
游戏动漫,新闻资讯,星座命理,美女,文艺
IT 数码,时尚,汽车,美食,美女,文艺
军事,IT 数码,文艺,体育
娱乐

文艺
军事
齐齐哈尔生活,娱乐,IT 数码
情绪化孩子气,吸血鬼控,微博控,双鱼座,90 后
时尚,娱乐,星座命理
教育就业,投资理财,美食,汽车,新闻资讯
搞笑幽默,校园生活,视频音乐,学生一族,体育
山东生活
美食,美女,游戏动漫,娱乐,IT 数码
体育
娱乐
汽车
旅游,娱乐,投资理财,教育就业,时尚,健康,新闻资讯
爱玩,营销,服装,爱吃,温柔,健康,游戏动漫,时尚,星座命理,娱乐
健康,时尚,美食,娱乐
时尚,军事,文艺,体育
体育
星座命理,美女,时尚,IT 数码,投资理财,汽车,文艺,体育,娱乐
汽车,美食
旅游,美食,娱乐,文艺
嗜睡症,公主病,强迫症,控制欲
娱乐,星座命理
时尚,星座命理,美女,电影,90 后,双子座,情绪化,吃货一枚,快乐,K 歌
美食,健康,娱乐,时尚,文艺
美好的世界,有你才精彩,亲情,美女
旅游,文艺,军事,娱乐,体育
新闻资讯,游戏动漫,旅游
射击游戏,电影,音乐,时事,机械,NBA,篮球,旅游,IT 数码,体育
美女,美食,教育就业,投资理财,军事
四川生活
旅游,星座命理,美食
投资理财,体育
汽车,IT 数码,投资理财,健康,星座命理
时尚,娱乐
娱乐,游戏动漫
爱音乐,双重性格,爱,开心,90,化妆品,青春,真诚,爱情,少女时代
时尚,娱乐
教育就业,新闻资讯
体育,健康,新闻资讯,时尚,投资理财,娱乐
星座命理
体育
娱乐,投资理财
游戏动漫
文艺

娱乐,美食,旅游
微博奇葩
军事,文艺,体育,投资理财,健康,新闻资讯
军事
天蝎女
美女,游戏动漫,时尚,投资理财,娱乐
游戏动漫,星座命理,汽车,IT 数码,娱乐
投资理财,美食,IT 数码
新闻资讯,美食,娱乐,投资理财,文艺
旅游,文艺,美食,时尚,星座命理,娱乐
健康
IT 数码,军事,教育就业,投资理财,汽车
旅游,文艺
文艺
视频音乐,街拍,BigBang,真诚,杂志,hiphop
投资理财,时尚,新闻资讯,健康,旅游
时尚,娱乐,教育就业
文艺,娱乐,美食,旅游,投资理财
时尚,娱乐,旅游,美食,军事
旅游,时尚,美食,娱乐
娱乐
文艺
旅游
健康,时尚,星座命理,美食,娱乐
广西生活,海綿寶寶
新闻资讯,美女
投资理财,汽车,美女,娱乐,军事,美食,新闻资讯
唱歌,看书,科比,K 歌,购物,看电影,爱音乐,文艺,努力,体育
旅游
星座命理,微博奇葩,搞笑幽默,娱乐
天秤座,睡懒觉,手工,音乐爱好者,喜欢音乐,开心,蓝色,潮流,我就是我,娱乐
时尚,美食,新闻资讯,健康,旅游
教育就业
王振,新闻资讯,军事,体育,投资理财,IT 数码,时尚
搞笑幽默,初音,腐女宅女耽美狼,耽美 BL,耽美广播剧,腐女,宅舞,cosplay 爱好者,动漫迷,帅哥控
娱乐
任性,赚钱,健康,旅游,星座命理,美食,文艺
时尚
美女,视频音乐,娱乐,旅游,体育
LANLAN,认定姓黄的我吧
体育,娱乐,健康
新闻资讯,娱乐,健康
美食,娱乐

娱乐,星座命理,时尚
美食,新闻趣事,微博奇葩,萌宠,穿衣美容
时尚
IT 数码,教育就业,旅游,星座命理
旅游,美食,文艺,体育,娱乐
体育,文艺,娱乐,美女,新闻资讯
IT 数码
投资理财,健康,旅游,军事,娱乐,文艺
星座命理,健康,时尚,教育就业,娱乐
汽车,旅游,IT 数码,娱乐,体育
旅游,文艺,时尚,美食
IT 数码
娱乐,体育
游戏动漫,娱乐
天平座,爱旅行,文字,情感,笑话,音乐控,杂志,美食,旅游,文艺
善良,大学生,旅游,新闻资讯,文艺,时尚
健康,投资理财,星座命理,娱乐
娱乐
亲情,韩国,90 后,宅,动漫控,韩剧,无厘头,小说迷,喜欢音乐,懒人
體育,娱乐,游戏动漫
旅游,IT 数码,美女
爱音乐,我就是我
新疆生活
娱乐,IT 数码,文艺
文艺
美食,时尚,IT 数码,游戏动漫,美食,娱乐
时尚,旅游,美食
游戏动漫,汽车,文艺,IT 数码,旅游
教育就业,旅游,健康
美女,娱乐
美女
搞笑幽默,文艺,体育,新闻资讯,娱乐
健康,教育就业,体育
娱乐
旅游
宁波,提问大师,业主打分,段子手,新闻人,断案,爆料,广告人
娱乐
娱乐,教育就业,游戏动漫,美女,旅游
旅游,IT 数码,星座命理,军事,汽车
健康,文艺,娱乐
胡思乱想,美食,旅行,上网,睡觉,金牛女,O 型血,Music
美食
宅,俞灏明的芋头,维尼饭,抹茶控
旅游

新闻趣事,搞笑幽默,偏执狂,旅游摄影,完美主义,书,无聊
萌宠,美食,美女,视频音乐,搞笑幽默,IT 数码,娱乐,游戏动漫
娱乐
体育
美食
新闻资讯,健康,旅游,投资理财,美食
军事,美食
娱乐
娱乐,游戏动漫,旅游,时尚,美食
IT 互联网,随性,爱笑,沉默,皇家马德里,典型天蝎男,足球,篮球,欧美电影,欧美音乐
多愁善感,旅游美食爱好者,安静,咖啡控,文字控,宅女一枚
美食,旅游,美女,汽车,体育
新闻资讯,时尚,旅游,文艺,娱乐
旅游
IT 数码,游戏动漫,投资理财,娱乐
美食,投资理财,旅游,美食,军事,IT 数码
娱乐,时尚,文艺
健康
真诚,新闻资讯
健康,教育就业,娱乐,美女,体育
科学探索,美图摄影,IT 数码,视频音乐,搞笑幽默
至天使
动漫,娱乐,体育,投资理财,游戏动漫
星座命理,文艺,娱乐
军事,IT 数码,星座命理,新闻资讯,教育就业
新闻资讯,体育,军事,IT 数码,美食
体育
娱乐,美食
投资理财,教育就业,美食,文艺,体育
新闻资讯,旅游
军事,IT 数码,汽车,旅游,游戏动漫
娱乐,爱音乐,巨蟹座,电影控
汽车
健康,星座命理,娱乐
时尚,星座命理,美食,娱乐,文艺
旅游,美食,娱乐
新闻趣事,跑步
时尚,星座命理,汽车,娱乐
健康,新闻资讯,投资理财,旅游
母婴团购,进口食品,配方奶粉,Baby,科学教育,母婴护理,潮爸辣妈,健康养生,搞笑宝宝,育儿知识
时尚,旅游,健康,新闻资讯,投资理财,教育就业,文艺,美食
IT 数码
星座命理,爱旅游,发呆,music,双子女

旅游
美女,时尚,游戏动漫,美食,IT 数码,娱乐
投资理财
娱乐,旅游
体育,文艺,旅游,投资理财
时尚,娱乐,文艺,美食,健康
星座命理
旅游
美食
校园生活,视频音乐,娱乐,体育
旅游
美食
任性,做梦,娱乐,旅游,文艺
旅游,美食,时尚,投资理财,娱乐
新闻趣事,新闻资讯,时尚,体育,汽车,军事
文艺,时尚,健康
时尚,美食,健康,旅游,娱乐
吃货,宅女,幽默,文艺,娱乐
旅游,IT 数码,体育,娱乐
唱 K,懒人
教育就业,投资理财,旅游,健康,美食
体育
时尚,旅游,星座命理,美食,文艺
投资理财,健康,旅游,娱乐
遵义生活,游戏动漫
美食
美女,汽车,文艺
音樂,娱乐
金牛座,古典音乐,美食,旅游
IT 数码,星座命理,游戏动漫
娱乐
教育就业,美食,旅游,IT 数码,文艺,游戏动漫,娱乐
教育就业
IT 数码,旅游,星座命理
穿衣美容
投资理财,健康
游戏动漫,汽车,文艺
新闻趣事
新闻资讯,旅游
美女,文艺,体育
新闻资讯,IT 数码,投资理财,教育就业
星座命理,娱乐
投资理财,旅游,文艺,美女,军事
时尚,娱乐

汽车
旅游,美女,时尚,娱乐
广东生活
教育就业,新闻资讯,旅游,体育
美食
娱乐,游戏动漫
汽车,美女,游戏动漫,体育
美食,旅游,娱乐,星座命理
投资理财,时尚,汽车,健康,新闻资讯
美食
投资理财
健康,旅游,美食,体育,文艺
教育就业,美食,健康,新闻资讯,文艺
IT 数码,美食
o 型血
失去自由了我
娱乐,时尚
新闻资讯,时尚,旅游,星座命理,美食
旅游,新闻资讯,汽车,投资理财,文艺,娱乐
名人明星
新闻趣事
汽车,军事
星座命理,美食,美女,新闻资讯,健康
美女,IT 数码,星座命理,旅游,汽车,军事
时尚,旅游,健康
时尚,星座命理,美女,体育,娱乐
文艺,IT 数码,汽车,美食,健康,旅游
搞笑幽默,教育就业,健康,旅游,文艺,娱乐
投资理财,旅游,健康,娱乐,时尚
军事,游戏动漫,旅游,时尚,星座命理,文艺,娱乐
减肥,爱笑,懒
军事,旅游,体育,羽毛球,自行车郊游,钓鱼
美女
时尚,健康,娱乐,星座命理,新闻资讯
美女,美食,星座命理,健康,时尚
时尚,体育,投资理财
爱笑,爱唱歌,BIGBANG,娱乐
时尚,旅游,美食
文艺
新闻资讯,游戏动漫
IT 数码,健康
投资理财,健康,星座命理,旅游
娱乐
时尚

星座命理
娱乐,健康,时尚,星座命理,旅游
美食
时尚,体育
到处走走逛逛,手作爱好者
射手座
市场营销,媒体人,飞轮海,星座命理,娱乐
游戏动漫,星星,海贼王,漫画,动漫迷
美女,娱乐
军事,健康,美女,时尚,汽车,教育就业,娱乐,体育,文艺
美食,时尚
汽车,IT 数码,娱乐,旅游
娱乐,投资理财
教育就业,投资理财,娱乐,旅游
投资理财,时尚,文艺,IT 数码,健康
体育
睡觉,123,旅游,美食,自由,情绪化,汽车,时尚,文艺,娱乐
穿衣美容,旅游,美食,新闻趣事,厦门生活
体育
新闻资讯
旅游
娱乐
美女,美食
屌丝女
娱乐,美女,旅游,美食,星座命理
旅游,文艺
美女,娱乐,汽车
美女
文艺,投资理财,新闻资讯,健康,美食
教育就业,旅游,健康,美食,娱乐
睡觉,意大利,温州生活,金牛座,文艺
旅游
健康,新闻资讯
私摄影,小冬,拍客,私宅,老上海,码字,知道分子,草根堂,寒冬夜行人,安公子
体育
旅游
游戏动漫
美食,情感生活,视频音乐,美图摄影,时尚
时尚,健康
旅游,投资理财
美食
爱睡觉,减肥,射手,新闻资讯
娱乐
微博奇葩,IT 数码

娱乐
美食,旅游,IT 数码,文艺,娱乐
健康
美食
文艺
投资理财
校园生活,美女,体育资讯
文艺,投资理财,教育就业
旅游
旅游
军事,娱乐,体育,文艺,旅游,IT 数码,教育就业,健康,美食
爱自由,爱音乐,妖精,文艺
游戏动漫,汽车,美食,旅游,娱乐
军事,旅游
北京生活,IT 数码
旅游,美食
IT 数码
健康,新闻资讯,娱乐
教育就业,时尚,健康,星座命理,娱乐
旅游
汽车,娱乐,体育,美食
萌宠,名人明星,汽车
IT 数码
IT 数码,体育,娱乐
看电影,技术宅,摩羯座
情感生活,旅游,健康,文艺,体育
安静,爱笑,吃货一枚,文字控,唱 K,喜欢听歌,爱自由,k 歌,吃喝玩乐,唱歌
娱乐
文艺,体育,娱乐
星座命理,美食,军事
汽车,时尚,IT 数码,新闻资讯
文艺,美食,投资理财,健康,旅游,时尚
医学,旅游爱好者,中医,文学,吃,美食,健康
星座命理,游戏动漫,美女,娱乐,体育
娱乐,时尚,旅游,健康,美食
时尚,星座命理
自然,听歌,美食,自由,80 后,宅,苹果控,淘宝,简单,旅游
情感生活,时尚,健康,星座命理,文艺,娱乐
体育,文艺,娱乐
服装,爱吃,爱旅游,90 後,乐天派,拍照,自恋
美食
娱乐
白羊座,古典艺术,欧美,大学生,艺术,视频音乐,时尚,星座命理,文艺
旅游

美女,汽车,军事,新闻资讯
星座命理,时尚,美食,娱乐
健康,时尚
娱乐
教育就业,文艺,军事,美食,健康
IT 数码,美食
文艺,美食,娱乐
美女,投资理财,时尚,旅游,娱乐
美食,健康,文艺,旅游,星座命理,美食,娱乐
穿衣美容,娱乐
娱乐
时尚,健康,旅游,美食
旅游
健康,美女,教育就业
旅游
视频音乐,体育,时尚,健康,新闻资讯,教育就业
赚钱,海贼王,平常心,爱电影,双重性格,幽默,朴有天,读书,旅游
时尚,汽车,教育就业
美食,投资理财,教育就业,游戏动漫,娱乐,唱歌,上网,动漫,学生
美食,旅游,娱乐
体育
时尚,旅游,文艺,娱乐
旅游,体育,军事
时尚,投资理财,文艺,健康,旅游,美食
狗狗,外貌协会,大叔控
旅游,时尚,娱乐
美食,娱乐
新闻资讯,投资理财,IT 数码,健康,娱乐
汽车,军事,IT 数码
美食,健康
汽车,IT 数码
星座命理,花痴,海绵宝宝,射手女,陈奕迅,情绪化,娱乐
时尚
时尚
娱乐,游戏动漫
旅游
娱乐,健康,游戏动漫,美女,体育
时尚
美食
新闻资讯,军事
体育
美食
旅游
体育资讯,体育

新闻资讯
投资理财,军事,健康,美女,娱乐
健康,时尚,文艺,美食,娱乐
游戏动漫
投资理财,健康
IT 数码,时尚,娱乐,投资理财,美食,健康,美女,游戏动漫
旅游,星座命理,美食,娱乐,文艺
搞笑幽默,美食,汽车
文艺,美食,星座命理
军事,娱乐,体育,文艺,新闻资讯,健康
娱乐,汽车,美女
新闻资讯,美食,文艺,教育就业,健康,旅游
旅游,健康,时尚
时尚,汽车,健康,美女,文艺,娱乐
文艺,投资理财,新闻资讯,体育,娱乐
健康,娱乐,文艺
时尚,健康,美食,娱乐,旅游
时尚,健康,旅游,美食,娱乐
低调,文艺
体育
旅游,娱乐
游戏动漫,美女,娱乐,文艺
萌宠,搞笑幽默,穿衣美容,视频音乐,音乐迷,健康,游戏动漫,文艺
星座命理
健康,游戏动漫,星座命理,文艺,IT 数码,体育,娱乐
美食,投资理财,汽车,体育,娱乐,奋斗
教育就业,娱乐,体育,军事
体育
健康
娱乐,投资理财,美食,旅游,健康,时尚,教育就业
搞笑幽默
健康,时尚,星座命理,娱乐
娱乐
新闻资讯,美食,投资理财,星座命理,健康,时尚
文艺,健康,旅游,美女,投资理财
时尚,教育就业,体育,娱乐,旅游,健康,美食
旅游,时尚,健康,文艺,娱乐
新闻资讯,汽车,娱乐,旅游
IT 数码
星座命理,健康,时尚,投资理财,IT 数码,文艺
星座命理,美食,教育就业,IT 数码,投资理财
汽车,体育,IT 数码
教育就业,文艺,美食,健康,旅游
视频音乐,微博奇葩,美女,重庆生活,文艺,IT 数码,教育就业,旅游,体育

文艺,旅游
娱乐,时尚,健康,投资理财
美食
旅游,游戏动漫,娱乐,美食
军事,汽车,教育就业,文艺,新闻资讯
美食,健康
旅游,游戏动漫,体育,投资理财
体育
娱乐
遊戲動漫,美女,時尚,游戏动漫,旅游
文艺,投资理财,时尚,旅游,健康,美食
美女,星座命理,新闻趣事,名人明星,广东生活
搞笑幽默,内向,淡定,计算机,娱乐,体育
文艺,IT 数码,时尚,汽车,美女
军事
投资理财
旅游,军事,娱乐
娱乐
旅游
文艺
旅游
处女座,手机看小说,自然,文艺
文艺
娱乐
内向、篮球、销售
时尚,娱乐,旅游
娱乐,同志,青春,写作,文字控
娱乐
体育
美食
健康,新闻资讯,娱乐,军事,体育
文艺,健康,教育就业,星星,胡思乱想,90 後,低调,蓝色,矛盾体
新闻资讯,健康,娱乐,汽车,IT 数码
游戏动漫
新闻资讯,美女,IT 数码,体育
娱乐
户外徒步,詹姆斯的球迷,重庆妹儿,学生,自由,美食,文艺
IT 数码
新闻资讯
喜欢旅游
健康,时尚,星座命理,美食,旅游,娱乐
投资理财,娱乐
美食
星座命理

新闻资讯,健康,美女,投资理财
美食,美女
广东生活,新闻资讯
80 后的尾巴,音樂,疯子,汽车,健康,旅游
宅女加腐女,喜欢音乐,懒人,健康,文艺
游戏动漫,教育就业,星座命理,娱乐
文艺,游戏动漫,美食,星座命理,旅游
娱乐
时尚,新闻资讯,健康,星座命理,文艺
旅游
娱乐,健康,时尚,文艺
时尚,娱乐,专爱 LAY
文艺,美女,汽车,军事
林峯,美食
西安生活,美食,搞笑幽默,旅游,美女,游戏动漫
投资理财,娱乐
教育就业,时尚,美食
创意,科技,85 后,金融,单身,新闻资讯,美女,投资理财,军事,IT 数码
旅游
娱乐,投资理财
投资理财
汽车,旅游,健康,美食,星座命理
新闻资讯,游戏动漫,娱乐
健康
湖南生活,读书分享,新闻趣事,美食
娱乐
健康,星座命理,娱乐
美食,IT 数码,体育
美食
游戏动漫,旅游,文艺,娱乐,体育
娱乐,新闻资讯,教育就业,星座命理,军事
星座命理,汽车,时尚,IT 数码,娱乐
娱乐,健康
星座命理
星座命理
健康,时尚,旅游,美食,娱乐
BIGBANG,王力宏,严爵,杨幂
新闻资讯,投资理财,军事
娱乐
IT 数码,文艺,旅游,游戏动漫,投资理财
宅女加腐女,大学生,music,看电视,射手女,爱笑,娱乐
体育
美女,军事,IT 数码,体育,娱乐
新闻资讯,美食,健康

军事
健康,美食,文艺
旅行,音乐,电影,自由,读书,公益,健康
看电影,喜欢旅游,逛街,喜欢音乐,中医,看书,运动,水果,舞蹈,娱乐
健康,旅游,星座命理,美食,娱乐
美女,美食,IT 数码,体育,娱乐
娱乐
投资理财,新闻资讯,美女,IT 数码,娱乐
文艺
四川生活,军事
新闻资讯,星座命理,美食,娱乐,美女,IT 数码
美女,军事,汽车,体育
文艺,健康,旅游,教育就业
新闻趣事
娱乐,游戏动漫,文艺
美食,IT 数码,健康,时尚,娱乐,星座命理
IT 数码,美女,游戏动漫,时尚
军事,游戏动漫,时尚,IT 数码
健康,投资理财,美女,旅游,美食
文艺,时尚
娱乐
军事,美女,时尚,娱乐
淘宝控,时尚
美女,文艺,体育
体育
旅游美食
娱乐
旅游,美食,投资理财
健康,旅游,美食
投资理财,教育就业,时尚,旅游,星座命理
星座命理,时尚,娱乐,文艺,新闻资讯
旅游,美食,娱乐,时尚
时尚
游戏动漫,旅游,教育就业,美食,文艺
微博奇葩
汽车,旅游
美女,投资理财
体育
星座命理,美食,教育就业,投资理财,新闻资讯
幸福,韩剧,学生一族,交友,吃货,旅游,星座命理,美食,文艺,娱乐
新闻资讯,投资理财,教育就业,时尚,汽车
体育资讯,娱乐,吉他,健身,足球
健康,新闻资讯,娱乐
新闻资讯,娱乐,旅游

游戏动漫,IT 数码,旅游,时尚,汽车,娱乐
时尚,汽车,游戏动漫,美女,健康
旅游,美食,娱乐,文艺,时尚
娱乐
看小说,耽美
旅游,游戏动漫,文艺
美食,IT 数码
微博奇葩,教育就业
美食,娱乐
星座命理
动漫
体育
新闻资讯,旅游,星座命理,文艺,体育
美女,旅遊,軍事,體育,新聞資訊,文藝,時尚,教育就業,娛樂
旅游,新闻资讯,娱乐,投资理财,时尚,健康,星座命理
文艺,投资理财,旅游,健康,美食
健康,星座命理,娱乐
美食
星座命理
旅游,教育就业
体育
穿衣美容,旅游,美女
美食,星座命理,旅游,健康,时尚
时尚,娱乐
天枰座,电影迷,林峯,青春,动漫迷,爱幻想,游戏,懒人,娱乐,体育
旅游,星座命理,美食,文艺,娱乐
娱乐
IT 数码
美食,军事,新闻资讯,体育,汽车,游戏动漫,星座命理
军事,体育,旅游
星座命理,游戏动漫
美女
时尚,美食
安静,旅游,文艺
娱乐
新闻资讯,文艺,投资理财,旅游,健康,军事
紫色,敏感,睡懒觉,时尚潮流,高中生,射手,喜欢听歌,吃货,囧,时尚
美食
新闻资讯,健康,旅游,时尚,娱乐
星座命理
教育就业,时尚,健康,旅游
理财,新闻趣事,信托,第三方支付
天枰座,笑,90 後,学生一枚
云南生活

新闻资讯,游戏动漫,IT 数码
旅游,新闻资讯
汽车,时尚,美食,星座命理,旅游,娱乐
旅游,健康,娱乐,军事,新闻资讯
IT 数码
时尚,健康,旅游,文艺,教育就业
喜欢唱歌,旅游美食,街拍,爱吃,运动,时尚,娱乐
美女,美食,体育,娱乐,星座命理
新闻资讯
游戏动漫,教育就业,健康,IT 数码
时尚,游戏动漫,美女
投资理财,时尚,星座命理,娱乐,美食
文艺,军事,旅游,美女
投资理财,健康,旅游
健康,时尚,星座命理
星座命理,时尚
星座命理
美食,娱乐,投资理财,游戏动漫,文艺
旅游,星座命理,美食,文艺
星座命理
星座命理,美食
娱乐
星座命理,90 后,糖果控,天秤座,纠结女,BigBang
时尚,旅游,美女,健康,娱乐
体育
军事
星座命理,军事,美食,旅游,娱乐
投资理财,体育,军事
旅游,美女,汽车,文艺,体育
娱乐
投资理财,新闻资讯
旅游
文艺,游戏动漫,汽车,旅游,美女
娱乐,健康
娱乐,时尚
美食
美食
80 後,奋斗,唱歌,钓鱼,重庆,狮子座,房地产,哈雷党,军事
游戏动漫,娱乐,星座命理,美食,文艺
健康,星座命理,时尚,新闻资讯,美食
娱乐
健康,时尚,旅游,美食,军事
美食,星座命理,健康,时尚,娱乐
阳光,美食控,喜欢听歌,爱笑,健康,时尚,娱乐,美食,旅游

IT 数码,美女
新闻资讯
美食,爱旅游,双子女
健康,文艺
体育,美食
教育就业,时尚,健康,投资理财,旅游
娱乐
八卦杂谈,无聊,傻
随性,开始了路程,维他命,娱乐,旅游,星座命理
游戏动漫,军事,IT 数码
汽车,美女
娱乐
体育,汽车,旅游,健康,军事
90 后,搞笑幽默,科技,单身,IT 数码
时尚,健康,美食
美食,星座命理,娱乐
90 后非脑残,吃货一枚,美食控,玩,音樂,狮子女,动漫迷,动漫控,大学,亲情
投资理财,文艺,娱乐,时尚,星座命理
旅游
时尚
投资理财,文艺,体育,娱乐,美女,旅游
游戏动漫,文艺
旅游,IT 数码
IT 数码,美食,旅游,美女,汽车
美女,娱乐
时尚,投资理财,健康,旅游
宅男 80 后,水瓶
IT 数码,健康,新闻资讯,投资理财,旅游,美食
美女
投资理财
旅游,娱乐,教育就业,游戏动漫,美女,美食,军事,文艺,体育
娱乐,美女,时尚
美食
娱乐,游戏动漫
IT 数码,文艺,时尚,健康,美食
体育,文艺
美食,星座命理
健康,旅游,星座命理,娱乐
美女,微博奇葩,游戏动漫
投资理财,娱乐
星座命理,健康
星座命理,美食,文艺,时尚,游戏动漫,娱乐
时尚,教育就业,星座命理,美女,旅游
新闻资讯,健康,旅游,IT 数码

汽车,美食,新闻资讯,文艺,娱乐
萌宠
娱乐
美食,投资理财,时尚,健康,旅游
健康,新闻资讯,时尚,投资理财,星座命理
时尚,旅游,娱乐,美食
IT 数码,时尚,汽车,娱乐,游戏动漫
军事,新闻资讯,体育,投资理财,教育就业,美食
娱乐,时尚
投资理财,美食,时尚,健康
旅游,健康,文艺,时尚
旅游
星座命理,军事,游戏动漫,娱乐,汽车
奶茶,美食
娱乐,文艺,旅游
投资理财,教育就业,汽车,时尚,美食
游戏动漫
教育就业,健康,星座命理,美食,娱乐
文艺,新闻资讯,旅游,教育就业,健康
军事,IT 数码,文艺,体育,娱乐
娱乐,美食,体育,新闻资讯,教育就业,投资理财,IT 数码
投资理财,教育就业,IT 数码
旅游,美女,体育
星座命理,健康,时尚,文艺,娱乐
美女,娱乐
娱乐,IT 数码
娱乐
军事,IT 数码,汽车
星座命理,时尚,旅游,新闻资讯
美女,时尚,汽车,美食,旅游,文艺
健康,游戏动漫
时尚
汽车
新闻资讯,汽车,IT 数码,美女
体育,娱乐,美食,健康
体育,汽车,旅游,美食
美食,文艺
娱乐
新闻资讯
爱音乐,美食,娱乐
娱乐
投资理财,教育就业,时尚,汽车,新闻资讯
宅,听音乐,笑点低,偶稀饭睡觉觉,80 後,狮子座,懒,玩儿,爱吃,唱 k
娱乐,教育就业,时尚,旅游,美食

美女
旅游
军事
IT 数码
美食,文艺,健康,娱乐,投资理财
听音乐,咖啡控,散步,古典音乐,工作,创意,朋友,巨蟹,咖啡,健康
健康,汽车,时尚,旅游,美食
新闻资讯,健康,旅游,教育就业,文艺
娱乐,汽车,新闻资讯,旅游,军事
星座命理,美女,时尚,汽车,IT 数码,文艺,体育,娱乐
清新淡雅狂热分,唧唧歪歪的好人,恐怖片狂热份子,各种强迫症
湖南泥巴公社装,新闻资讯,旅游,文艺,时尚
新闻资讯,美食,军事,体育
健康
文艺,新闻资讯,教育就业,旅游,健康,体育
游戏动漫,美食,体育
星座命理,娱乐,汽车,美女,新闻资讯,旅游,军事
娱乐,星座命理,旅游,健康,时尚,美食
美食
军事
旅游,游戏动漫,投资理财,星座命理,美食
穿衣美容,投资理财
旅游,麦彭寺
文艺,星座命理
新闻趣事
IT 数码
美食
新闻资讯,教育就业,时尚,投资理财,健康,星座命理,娱乐
搞笑幽默,星座运势,宠物图集,旅游,爱自由,重口味,金牛
美食,火腿肠,拖鞋,排骨,耗子,猫
星座命理
汽车
美食,娱乐
时尚,旅游,健康
搞笑幽默,广东生活,热爱生活,天蝎座,80 后宅女
体育
文艺,星座命理,时尚
星座命理
美食
投资理财,文艺,IT 数码,时尚,汽车
游戏动漫,美食,IT 数码,文艺
娱乐
游戏动漫,IT 数码,体育
视频音乐,娱乐,汽车,时尚,新闻资讯,美食

娱乐,IT 数码,新闻资讯,教育就业,健康
花样美男,淘宝,90,韩剧,唱 K
美女,时尚,汽车,旅游,体育
娱乐
健康,星座命理,投资理财,娱乐,旅游
视频音乐,星座命理,微博奇葩,旅游,美食
旅游,健康
时尚,游戏动漫,美女,新闻资讯,健康
体育
旅游,投资理财,教育就业
美女
旅游,美食,娱乐
萌宠,视频音乐,军事,游戏动漫,体育
IT 数码,自由,星座命理
文艺,教育就业,健康,娱乐,星座命理,美食
健康,旅游,投资理财,星座命理,汽车
时尚,文艺,娱乐,星座命理,旅游,健康,游戏动漫
IT 数码,星座命理
投资理财,汽车,美食,旅游,随性,感性,人生,美食爱好者,重庆,新闻
魏晨的乐橙,星座命理,娱乐
时尚
美食
星座命理
时尚
IT 数码,体育
游戏动漫,汽车,娱乐,IT 数码
爱音乐,90 後,爱旅游
健康
名人明星,独立,郭敬明,护士,爱电影
健康,时尚,美食,旅游
游戏动漫,旅游,星座命理,美食,文艺
投资理财,健康,教育就业,新闻资讯
体育资讯
汽车,新闻资讯,旅游,军事,娱乐,美女,体育
睡觉,
美女,美食,军事,娱乐,体育
旅游,时尚,星座命理,教育就业
新闻资讯,美女,体育
星座命理,娱乐,游戏动漫
新闻资讯,视奸狂魔
宅女,星座命理,美食,文艺,游戏动漫
游戏动漫,汽车,美食,娱乐,体育
旅游
旅游,投资理财

娱乐,汽车,游戏动漫,美女,IT 数码
IT 数码,美食,旅游
强迫症,简单,天蝎,美剧控,拍照,影视,欧美,爱幻想,动漫迷,看小说
旅游,健康,美食,游戏动漫
时尚,旅游,健康,娱乐
文艺,新闻资讯
投资理财,教育就业
娱乐
军事,80 後
汽车,新闻资讯,健康,旅游,文艺
贵阳生活,美图摄影,星座运势,情感生活,投资理财,旅游
美术,室内设计,天枰座,时尚,旅游,美食
体育
旅游,文艺
旅游,美食
神经大条,喜欢听歌,重庆,吸血鬼日记,咖啡,爱美食,旅游,时尚,美食,文艺
娱乐,美食,健康,时尚,IT 数码
感性,k 歌,杨幂,游戏动漫,健康,时尚,美食,星座命理
视频音乐,宠物图集,八卦杂谈,健康,旅游,美食
健康,新闻资讯,时尚,娱乐
时尚,星座命理,军事
投资理财,汽车,时尚,文艺,体育
有机肥,新能源,健康,循环经济,生态农业,环保
成都生活,投资理财,娱乐,体育,文艺,新闻资讯
臭美,B 型血,开心,时尚,健康,旅游,美食,娱乐
旅游,美食,美女,汽车,军事,新闻资讯
搞笑幽默,汽车
旅游,美食,体育
旅游
健康,新闻资讯,时尚,教育就业,娱乐
IT 数码,体育
星座命理
美女,时尚,娱乐
电视,英语,购物,健康,爱笑,安静,玩,快乐,美食控,美食
新闻趣事,时尚,娱乐,健康
旅游
星座命理
时尚
美食,美女,娱乐
时尚,星座命理,旅游,美食
娱乐
游戏动漫,旅游,娱乐,美女,IT 数码,文艺
新闻趣事,胡思乱想,旅游,文艺
文艺

美图摄影,许嵩,矛盾体,淡定,睡懒觉
美食
微博奇葩,美食,IT 数码
爱睡觉,双子座,狗痴,宠物,爬山,吃货,旅游
娱乐,旅游,投资理财,健康,星座命理
美食,旅游,娱乐
体育
游戏动漫,时尚,新闻资讯
旅游,文艺,美食,健康
投资理财
上网,学生,小说,愤青,开心,喜欢音乐,懒人,喜欢唱歌,美食,文艺
旅游,游戏动漫,汽车,投资理财
健康
时尚,美女,健康,教育就业,星座命理,美食,军事,娱乐
美食
教育就业,美女,健康,游戏动漫,美食,娱乐,星座命理
新闻资讯,汽车,IT 数码,时尚,健康,旅游,教育就业,体育
IT 数码,汽车,体育
军事
IT 数码,美食,旅游,星座命理,娱乐
娱乐,文艺,教育就业,新闻资讯,旅游,健康
旅游,时尚,文艺,星座命理,美食,IT 数码
旅游,投资理财,美食,健康
体育,美女,文艺,时尚,投资理财,健康,美食
娱乐,健康,时尚,旅游,美食
微博奇葩,时尚
文艺,时尚
微博奇葩,视频音乐,IT 数码,娱乐
投资理财,游戏动漫,体育,娱乐,军事
新闻资讯,时尚,旅游,文艺
fx,f,蒋梦婕,多情媚眼,诗迷嫣迷,美食爱好者,健康,星座命理,美食
IT 数码
娱乐
感性,台球,爱旅行,音乐达人,烹饪,汽车,新闻资讯,健康,旅游,IT 数码
娱乐,汽车,军事
文艺
情感生活,娱乐
娱乐
美食,时尚,健康,旅游,星座命理
健康,游戏动漫,IT 数码,体育,娱乐,旅游
时尚,新闻资讯,健康,旅游,星座命理
美女,汽车
喜欢听歌,汽车
成都生活,新闻资讯,游戏动漫

自拍,乐天派,动漫,小说,学生,爱吃,文艺,娱乐,时尚,美食
军事,旅游,健康
新闻资讯
美食
新闻资讯
美食,娱乐
游戏动漫,IT 数码
星座命理,美食,健康
新闻资讯,健康,美食,旅游,时尚
星座命理,汽车,美女,投资理财,新闻资讯,旅游,军事
军事
旅游,娱乐
美食,文艺
健康,旅游,美食
美食
文艺
狮子座,糖分,银魂
汽车
文艺,汽车
健康,教育就业,星座命理,新闻资讯,旅游,美食,IT 数码,文艺
炫彬,他们生活的世界,天蝎座,80 后,玄饭,金泰坪,玄彬
美食,体育,娱乐,教育就业,健康
名人明星,微博奇葩,美女,新闻趣事,搞笑幽默
娱乐,游戏动漫
游戏动漫,新闻资讯
美食
旅游
旅游,娱乐
新闻资讯,文艺,旅游,健康,美食
文艺,体育
时尚,旅游,美食,IT 数码,娱乐
文艺,游戏动漫,美食
汽车,游戏动漫
新闻资讯,时尚,健康,文艺,教育就业
健康,旅游,文艺,体育
美女,娱乐
IT 数码,文艺,体育,娱乐,美女
时尚,健康,旅游,美食,娱乐
旅游,美食,娱乐,时尚,星座命理
摩羯女,吃货一枚,画画,我就是我,美食
名人明星,军事,IT 数码,文艺,体育
文艺,游戏动漫,旅游,体育,娱乐
体育
美女,汽车,旅游,美食,娱乐

搞笑幽默,文艺,游戏动漫,军事,体育
萌宠,旅游
游戏动漫,健康,旅游,美食,文艺
娛樂,體育
美食,星座命理,美女,游戏动漫
体育
其他生活
杰迷,游戏动漫,星座命理,娱乐
娱乐,新闻资讯,时尚,文艺,健康,教育就业,旅游
美女,名人明星,视频音乐,文艺,旅游,体育,娱乐
游戏动漫,文艺,军事,美食,旅游,教育就业,时尚,娱乐
健康,时尚,旅游,IT 数码,美食
爱过的都姓李,爱旅游爱购物,双子座的矛盾体
旅游,新闻资讯,军事
美女
时尚,美食,投资理财,新闻资讯,健康,旅游,IT 数码
汽车,美食,旅游,IT 数码,健康
投资理财,汽车,军事,IT 数码
时尚,娱乐
投资理财,IT 数码
美食,旅游
健康,IT 数码,星座命理,美食,旅游,娱乐
新闻资讯,文艺,汽车,体育,娱乐
汽车,IT 数码,文艺,体育,娱乐
游戏动漫,美食,健康,文艺,美女,体育
游戏动漫,教育就业,投资理财
时尚
新闻趣事,营口生活,名人明星,文艺
旅游,娱乐,汽车,IT 数码,体育
旅游,天枰座,唱 K,喜欢音乐
体育
旅游,教育就业,IT 数码,体育,娱乐
娱乐,汽车,美女
时尚
星座命理,游戏动漫
健康
游戏动漫,教育就业,IT 数码,文艺,体育
萌宠
星座命理,美食,IT 数码,娱乐
IT 数码,军事,美食,美食,游戏动漫,娱乐,汽车,体育
苏州生活
美女,军事,体育
娱乐,美女,游戏动漫
娱乐,旅游,游戏动漫,时尚

爱交朋友,英国留学
书,闷骚,大叔控,喜欢听歌,驴友
娱乐
萌宠,美食,旅游,视频音乐,搞笑幽默,IT 数码,娱乐,体育,游戏动漫
旅游
美食
穿衣美容,星座命理,美食,旅游,健康,时尚
旅游,新闻资讯,投资理财,教育就业,娱乐
娱乐,文艺,旅游,文学,欧美,旅游美食爱好者,爱音乐,时尚
时尚,体育,健康,旅游,美食
文艺,美女,旅游,时尚
体育,星座命理,旅游
IT 数码,时尚,美食
美食
新闻资讯,美食,文艺,教育就业,健康,旅游,美食
旅游
穿衣美容,宅女一枚,90,购物狂,偶稀饭睡觉觉,摩羯座,自恋,时装
星座命理,旅游,健康,游戏动漫,文艺,娱乐
名人明星
听歌,电视剧,高中生,教育就业,旅游,军事
文藝,時尚,遊戲動漫,娛樂
体育
狗狗,王力宏,Music,90 后但我不脑残,科幻,外貌协会,爱电影,游戏动漫,数码,美食
白羊座
旅游,游戏动漫,IT 数码,娱乐,文艺
英国生活,新闻资讯,投资理财
文艺,体育
娱乐
绘画,军事,美食,新闻资讯,旅游,游戏动漫
名人明星,搞笑幽默,萌宠,新闻趣事
IT 数码,美女,文艺,娱乐
投资理财,健康,旅游,美食,文艺
游戏动漫,美女,美食,体育
教育就业,游戏动漫,新闻资讯,IT 数码,美女,投资理财,体育
军事,文艺,娱乐,体育
萌宠,旅游,美食,星座命理,搞笑幽默,娱乐
双子座,95 后,名人明星,舞蹈,音乐,播音主持,娱乐
健康
穿衣美容,美食,旅游
旅游
美图摄影,体育资讯,校园生活,思考,诗歌,学习,新闻资讯,体育
娱乐,时尚,旅游,美食
汽车,文艺
IT 数码,娱乐,健康,时尚,美食

IT 数码
旅游
美女
音乐控,强迫症,韩寒,哈利波特,摄影控
娱乐
娱乐,体育
时尚,旅游,星座命理,娱乐
娱乐,美食,健康,文艺,星座命理
时尚,游戏动漫,旅游,星座命理,娱乐
车世界
旅游,美食,娱乐
旅游,搞笑幽默,名人明星,打篮球,NBA,体育
为梦想而努力,电影电视剧迷,舞蹈,美食,娱乐
健康,旅游
文艺,体育
时尚,旅游,文艺,娱乐,体育
游戏动漫
娱乐,时尚
军事,文艺
体育
星座命理,美食,旅游,娱乐,美食
娱乐
美女,体育
萌宠,微博奇葩,新闻资讯,娱乐
星座命理
娱乐
新闻资讯,美食,汽车,游戏动漫,旅游
培训
白羊座,娱乐,投资理财,美食,健康,体育
娱乐
投资理财,时尚,文艺,健康,美食
美女,新闻资讯,军事,投资理财,美食,健康
矛盾体,狮子座,偶稀饭睡觉觉,学生一枚
旅游,娱乐,时尚
游戏动漫,IT 数码,娱乐
娱乐,文艺,汽车,健康,旅游
IT 数码,美食,旅游,健康,汽车,体育
文艺,美食,军事,IT 数码,娱乐
废话专用,贤敏,零顺位老少年,王源王俊凯,正太控,广播剧渣,耽美腐宅女,李晟敏,李灿多
文艺,星座命理,美食
投资理财
健康,时尚,美女,娱乐
外貌协会,拍照,睡觉睡到自然醒,双重性格,爱生活,活泼开朗,孩子气,交朋友,星座命理
娱乐,汽车,游戏动漫,时尚,体育

文艺,娱乐
时尚,旅游,爱旅行,天蝎女,欧美控,美食,健康
军事
体育
邓紫棋,陈冠希
吃货无罪,小女子,此女很坏,文艺
军事,体育,旅游
健康,旅游
军事
旅游,体育
星座命理,投资理财,美食,旅游
娱乐
美食,美女,新闻趣事
MunMun 脑残粉,野原新之助,坂田銀時,银魂,蠟筆小新
旅游
简单,娱乐
坚强,自然,心理学,爱电影,房地产,文艺,美食,星座命理,健康,投资理财
新闻资讯,美女
体育
娱乐
体育
健康,时尚,汽车,文艺,IT 数码,娱乐
美食
教育就业,娱乐,时尚,旅游,文艺
健康,投资理财,教育就业,娱乐,军事,体育
文艺,旅游,健康,投资理财,美食
娱乐
美食,文艺,星座命理
体育
医学,摄影控,爱旅游,爱电影,大叔控,爱吃,IT 数码,美食
旅游
美女,IT 数码,军事,时尚,游戏动漫
投资理财,汽车,美女,旅游,军事
汽车
时尚,投资理财
教育就业,军事,IT 数码
投资理财
果粉,美剧控
娱乐,游戏动漫
娱乐,体育,旅游
旅游,军事
时尚,美女
IT 数码
文艺,星座命理,旅游,健康,美女

时尚,汽车,教育就业,美食,IT 数码,笑话,篮球,睡觉,娱乐
新闻资讯,IT 数码,汽车,体育
旅游
新闻趣事,视频音乐,美女
娱乐,健康
体育
旅游
IT 数码,美女
娱乐,时尚,旅游,健康,星座命理
美女,星座命理,美食,萌宠,微博奇葩
娱乐,游戏动漫,旅游,星座命理,美食
星座命理,穿衣美容,微博奇葩,新闻趣事,搞笑幽默,投资理财,时尚,娱乐,文艺
娱乐,时尚,文艺
美食,旅游
投资理财,时尚,健康,文艺,美食
健康,汽车,投资理财,时尚,旅游,星座命理,美食
90,2konline,篮球,爱幻想
游戏动漫,旅游,星座命理,体育,娱乐
旅游,游戏动漫,美食
时尚
娱乐,文艺,旅游,新闻资讯,时尚,投资理财
旅游,美食,游戏动漫,文艺,娱乐
时尚,健康,星座命理,旅游,娱乐
美食,美女,投资理财,体育
游戏动漫,美食,娱乐
旅游,娱乐
旅游,时尚
安阳生活,投资理财,健康,文艺,军事
娱乐
IT 数码
英雄联盟,体育
游戏动漫,新闻资讯,星座命理,旅游,健康
星座命理,娱乐,美女
美女,美食,军事,IT 数码,旅游
娱乐,美食,旅游,健康
动漫,爱旅行,美食爱好者,喜欢听歌,爱电影,旅游,文艺,娱乐
新闻资讯,美食,旅游,美女,军事
旅游
娱乐,时尚,美女,美食,教育就业,文艺,健康,新闻资讯
美食
需要音乐陪伴,International,时尚,BestFriend,全能达人,完美主义
游戏动漫,时尚,汽车,娱乐,体育
汽车,时尚,新闻资讯,星座命理,健康
游戏动漫

美女,娱乐,新闻资讯,游戏动漫,军事
汽车,娱乐,IT 数码,军事,体育
健康,新闻资讯,投资理财,军事
娱乐,美女,星座命理,健康,旅游
游戏动漫,投资理财,体育,娱乐
文艺,投资理财,娱乐
穿衣美容,时尚
旅游
健康,旅游
咖啡控,美食,健康,旅游,娱乐,文艺
游戏,财经资讯,IT 数码,娱乐,美食,美女,投资理财
名人明星,视频音乐
教育就业,旅游,文艺,IT 数码,时尚
健康,星座命理,美食,娱乐
美食,旅游,健康,美女,娱乐
體育
投资理财,新闻资讯,旅游,美食,IT 数码
旅游
旅游
火星人在地球,花花华晨宇,音樂,懒人,睡懒觉,爱笑,听歌,胡夏的虾米,胡夏
健康,美食,旅游,娱乐
娱乐
娱乐,新闻资讯,游戏动漫,时尚,美食
健康,美食,时尚
游戏动漫
玩,快乐,爱笑,喜欢音乐,爱唱歌,学生一枚,书,美食,娱乐
美女,时尚
星座命理,娱乐
娱乐,旅游,投资理财,体育,汽车,美女
个性,无厘头,影视,执着,电脑爱好者,移动互联网,80 后的尾巴
体育,旅游,游戏动漫,娱乐,美食,IT 数码
美女,美食,娱乐
旅游,美食,体育
旅游,美食
教育就业
新闻资讯,文艺,时尚
新闻资讯,美食,文艺,娱乐
游戏动漫
文艺,健康,娱乐,美食
游戏动漫,时尚,IT 数码,文艺,娱乐
星座命理,旅游,时尚,美食,娱乐
时尚,美食,旅游,健康
游戏动漫
旅游,星座命理,美食,娱乐

娱乐
军事,体育
体育
健康,教育就业,娱乐,旅游,文艺,新闻资讯
美女,游戏动漫,汽车,体育,健康
投资理财,时尚,健康,星座命理,美食
娱乐
投资理财
旅游,军事
美食,穿衣美容,美女,不解释
旅游
美食,娱乐,文艺,旅游,健康
娱乐
美女,娱乐
数码,电影迷,娱乐
美女,汽车,时尚,体育,娱乐
星座命理,游戏动漫
娱乐
投资理财,汽车,旅游,健康,体育
汽车
娱乐,死神,忧郁,自拍
名人明星
体育,军事
投资理财
新闻资讯,投资理财
名人明星
文艺,娱乐
时尚,IT 数码
健康
娱乐
美食
创业,财务,文化交流,教育,TORONTO,MBA,新闻资讯
时尚,新闻资讯,旅游,娱乐,星座命理
90 后宅女,星座命理,文艺
时尚,IT 数码,投资理财,教育就业,新闻资讯
美食,文艺
娱乐
游戏动漫,星座命理
文艺
星座命理,新闻资讯,游戏动漫,文艺,娱乐
娱乐
文艺,娱乐,健康,新闻资讯
健康
美女

IT 数码,军事,时尚,体育,美女
时尚,健康,新闻资讯,美食,旅游
星座命理,军事
搞笑幽默
旅游,文艺,娱乐
投资理财,汽车,文艺,星座命理,时尚,美女,娱乐
教育就业,新闻资讯,美食,娱乐,文艺
Vintage,古着,BJD,布艺,DIY,小清新伪文艺,文艺,手作,复古,森林
时尚,健康
美食,健康,投资理财,娱乐,时尚
投资理财,旅游,IT 数码,体育,娱乐
娱乐,时尚,健康,体育,文艺,IT 数码
星座控　爱旅行,孩子气,B 型血,高中生,看书,睡觉,爱笑,懒,乐观,狮子座
文艺,星座命理,旅游,美食,健康
动漫,Bigbang
娱乐
新闻资讯,投资理财,健康,旅游,文艺
睡懒觉,美女,健康,体育,旅游
浙江生活,军事
上网,自拍
星座命理,健康,旅游,美食,文艺
娱乐,体育
热情,单身,阳光,NBA 球迷,摩羯座,自由人,爱旅行,音乐控,享自由
我一定会找到她,美女,时尚
微博奇葩,娱乐
汽车,教育就业,美女,文艺,投资理财,军事,娱乐
时尚
文艺,游戏动漫
星座运势,健康养生,情感生活,宠物图集,新闻趣事,投资理财,教育就业,新闻资讯
军事,体育
新闻资讯,健康
体育,军事,汽车
时尚,健康,旅游,IT 数码,文艺
新闻趣事,ipod,mac,iphone,apple,coodoo,时尚,IT 数码
新闻资讯,时尚,健康,教育就业,娱乐
新闻资讯,时尚,游戏动漫,投资理财,文艺
爱音乐,宅男,文学,哲学,梦想,教育就业
旅游
布丁,旅游美食,手工,朋友,文艺,时尚,健康
娱乐,星座命理
星座命理
汽车,健康,星座命理,旅游,美食
星座命理,娱乐
娱乐

娱乐,美食,文艺,中国风控,武侠控,乐天派,有点懒,爱睡觉爱做梦,爱鼠猫,爱老大
美食,旅游
娱乐
投资理财
天蝎座,教育就业,旅游
旅游,时尚,教育就业,美食,娱乐
娱乐,汽车,星座命理
健康
文艺
美国生活,IT 数码,游戏动漫
冷笑话,爱睡觉,娱乐八卦,破产姐妹,金贤重
学习 ING,完美主义强迫症,杂志癖,又宅又运动,搭配达人,美食爱好者,时尚狂人,吸血鬼,护肤,购物狂
娱乐,旅游,文艺
美女,娱乐,游戏动漫,星座命理
美女,投资理财,健康,旅游,美食
娱乐
娱乐
新闻资讯
时尚,星座命理,美食
体育,IT 数码,文艺
星座命理
投资理财
娱乐
时尚,美食,娱乐
星座命理,娱乐
健康,投资理财,新闻资讯,游戏动漫,IT 数码
旅游
旅游
文艺,美女,时尚,健康
美女,娱乐,军事,IT 数码,游戏动漫
新闻趣事,视频音乐,校园生活,搞笑幽默,八卦杂谈
文艺
留学,美国直邮代购,时尚品牌代购,美国,美国代购,名牌单品,名牌鞋,名牌,名牌包,时尚潮流
娱乐,教育就业,时尚,文艺,汽车,健康,旅游,IT 数码,美食
旅游
娱乐
时尚,体育,IT 数码,游戏动漫,星座命理
美女,汽车,娱乐
车世界,时尚,教育就业,健康,文艺
文艺,娱乐,时尚,美女
娱乐,星座命理,新闻资讯,时尚,文艺
80 后的尾巴,巴萨,足球迷,汽车

时尚,汽车,旅游,娱乐,美食
搞笑幽默,健康
文艺,旅游,投资理财,新闻资讯,军事
娱乐,体育,IT 数码,新闻资讯
旅游,健康,星座命理,美食
体育
美女
体育
娱乐,文艺,体育
K 歌
煞笔青年欢乐多,高贵冷艳,猫控,游戏动漫
娱乐,美食,旅游,美女,时尚
娱乐
星座命理,健康,文艺,军事,IT 数码
投资理财,旅游,美食,健康,时尚
时尚
星座命理,健康,时尚,美女,新闻资讯
游戏动漫
体育,新闻资讯,美食,军事,IT 数码
游戏动漫,美女,新闻资讯,星座命理
美图摄影,健康,游戏动漫,时尚,教育就业,星座命理
穿衣美容,美女
美女
美食,星座命理,旅游,娱乐
文艺
教育就业,文艺,投资理财,美女,美食
IT 数码
旅游
搞笑幽默,90 後,爱美食,音乐控,看电影,天蝎座,动漫
美国生活,宅男 80 后,猫咪控
JPM,邱胜翊王子,邱胜翊
文艺,美女,新闻资讯,IT 数码
时尚,销售,紫色,双鱼座,music,心理,星座,情感随笔,上海大众,汽车
美食,投资理财,旅游,娱乐,健康
游戏动漫
旅游,文艺
汽车,娱乐,IT 数码
新闻资讯,健康,教育就业,娱乐,体育
旅游,星座命理,美食,IT 数码,军事
娱乐
美女,美食,游戏动漫,文艺,娱乐
投资理财,娱乐,体育,时尚,IT 数码,军事,游戏动漫
时尚,美食,汽车,旅游,文艺
汽车,娱乐,时尚,游戏动漫,旅游,美食,IT 数码,射手,服饰,情绪化

IT 数码,美食,游戏动漫,汽车,体育
火速,创业,网站运营,移动网络营销,口碑营销,微博营销,社会化媒体营销,搜索引擎营销,网站建设,网络营销
美食,文艺,旅游
军事,健康,投资理财,文艺,美食
娱乐
车世界,性感,美女
健康,IT 数码,美女,娱乐,游戏动漫
旅游,美食,娱乐
健康,旅游,美食,文艺,娱乐
星座命理
文藝,星座命理,娛樂
教育就业
投资理财
教育就业,文艺,体育
健康,旅游,新闻资讯,教育就业,投资理财
时尚,美国代购,潮牌,fashion 控,留学生,睡觉睡到自然醒
军事,IT 数码,文艺,体育,娱乐
美食
健康
动漫,摩羯,听音乐,羽毛球
旅游,投资理财,游戏动漫,IT 数码,汽车
美剧,双鱼座,减肥,逛街,医学,旅游
微博奇葩,娱乐,体育
文艺,娱乐
美女,美食,体育
搞笑幽默
旅游
音乐控,养生,笑话,心理学,校园,投资理财,新闻资讯,星座命理,旅游,娱乐
时尚
新闻资讯,美食,文艺,健康,旅游
考研
旅游,美食,健康,游戏动漫
体育
文艺
美女,娱乐
视频音乐,文艺
微博奇葩
美食
微博奇葩,乐观,吃货,平常心,网购,傻,天蝎女,学生,宅
名人明星,杰瑞米雷纳,托马斯伯蒂奇,老友记,网球
星座命理,娱乐,时尚,游戏动漫,IT 数码,体育
健康
旅游,时尚,文艺,娱乐

游戏动漫,旅游,军事
文艺,旅游,时尚,星座命理,美食,娱乐
健康,投资理财,旅游,美食
健康,时尚,美食,新闻资讯,教育就业,文艺,娱乐
微博奇葩,健康
IT 数码
时尚,新闻资讯,美食,投资理财,旅游
时尚,教育就业,投资理财,旅游,星座命理
家居,时尚
汽车
汽车,投资理财,旅游,健康,新闻资讯
新闻趣事,中定协精准委,LBS,GIS,CORS,导航定位应用,中定协,行业应用,卫星,北斗
文艺,时尚,旅游,娱乐
游戏动漫,娱乐,健康,IT 数码
时尚,健康,新闻资讯,娱乐,旅游,美食
IT 数码,教育就业,健康,旅游,军事,体育
娱乐,体育
美女,视频音乐,新闻趣事,搞笑幽默,安徽生活,新闻资讯,IT 数码,汽车
娱乐
时尚,旅游,文艺
游戏动漫
娱乐
美食,时尚,文艺,娱乐
新闻资讯,游戏动漫
美食,旅游
90,喜欢唱歌,傻,志愿者,喜欢旅游,双重性格,喜欢听歌,星座命理
时尚,美食
星座命理,旅游,文艺,娱乐,美食
娱乐,美食,旅游
汽车,星座命理,娱乐,健康,美食,旅游,时尚
文艺
健康
视频音乐,搞笑幽默,校园生活,时尚,星座命理,美食,娱乐,IT 数码
时尚,文艺
美女,IT 数码
美食,旅游
新闻资讯,旅游,美食,IT 数码
IT 数码,美食,健康
美食,教育就业,健康,新闻资讯
美食,文艺,IT 数码,娱乐,时尚,美女,健康,旅游
旅游,教育就业,健康,时尚,美食,娱乐
健康
体育
娱乐

旅游,IT 数码,汽车,娱乐,体育
星座命理
新闻资讯
微博奇葩
文艺
新闻资讯,星座命理,文艺,美食,游戏动漫,健康
天蝎座,睡懒觉,旅游,星座命理,美食,文艺,娱乐
健康,美女,游戏动漫,投资理财
美食
投资理财,教育就业,汽车,IT 数码
文艺
娱乐,时尚,星座命理
旅游
投资理财
星座命理
时尚,上网
芯片,体育
时尚,旅游,健康
娱乐,游戏动漫,文艺,体育
健康,时尚,星座命理,美食,旅游
旅游,星座命理,美女,健康,美食
文艺
北京生活,新闻资讯,投资理财,旅游,军事,IT 数码
美食,健康
教育就业
90 后非脑残,驴友,淡定,健康,旅游,娱乐
真实力,安静,旅游,爱旅游
教育就业
旅游,美食,文艺,娱乐
校园生活,美食
文艺,娱乐,体育
娱乐
IT 数码,健康,军事
娛樂,文藝
教育就业,投资理财,旅游,美食,娱乐
宠物图集,娱乐
工作,Android,Music,手机,NBA,美食,IT 数码,体育,美女
自然
lz 宅男一枚
美女
娛樂
体育,时尚,星座命理,娱乐,文艺
体育,游戏动漫
健康

星座命理,旅游,健康,娱乐
美图摄影
时尚,汽车,投资理财,健康,旅游
美女
美女,萌宠,星座命理,微博奇葩,搞笑幽默
星座命理,旅游,健康
星座命理,文艺,娱乐,投资理财,新闻资讯
江苏生活,体育
时尚,投资理财,旅游,美食,娱乐
新闻资讯
时尚,新闻资讯,星座命理,教育就业,旅游,健康
文艺,时尚
体育
美女,健康
美食,旅游
旅游,选择恐惧症,密级恐惧症,三分钟热度,吃货,星座控
新闻资讯,美食,投资理财,IT 数码,娱乐
汽车,美女,IT 数码,投资理财,健康,新闻资讯
方大同,懒人,宅女,做梦,K 歌,网络,移动互联网,正太控,爱唱歌,吃喝玩乐
浙江生活,娱乐
游戏动漫,娱乐
新闻资讯,娱乐
星座命理,体育
美女,美食,文艺,娱乐,体育
美女
新闻资讯,健康,时尚,教育就业,旅游,文艺
健康,新闻资讯,教育就业,时尚,美食
军事,投资理财
体育
旅游,美食,体育,娱乐,时尚,健康
时尚,星座命理
健康,旅游,文艺,星座命理,娱乐
开心豆少儿英语,文艺
旅游,视频音乐,新闻趣事,搞笑幽默,北京生活,美女,新闻资讯,文艺,体育,教育就业
汽车
汽车,游戏动漫,IT 数码,文艺,体育
健康,投资理财,美食,销售,单身,交友,人生,工作,新闻资讯
IT 数码
游戏动漫,体育,IT 数码
美女,文艺
娱乐,文艺,美女
文艺,娱乐,星座命理,旅游,美食
上海生活,美食,军事,体育,新闻资讯,旅游
旅游,IT 数码

娱乐
美食,军事,健康,汽车,IT 数码
娱乐
教育就业,美食,娱乐,汽车,旅游,健康,星座命理,IT 数码
旅游,双重性格,爬山,重口味,自由职业
军事
健康
星座命理,军事,旅游,新闻资讯,汽车
搞笑幽默,微博奇葩
旅游,娱乐,时尚,美食,文艺
健康
新闻资讯
90 后,吃货一枚,娱乐,爱睡觉,画画,b 型血,喜欢听歌,聊天,校园
IT 数码,娱乐,汽车,健康,体育
新闻资讯,汽车,军事,旅游,健康,投资理财,体育
汽车,美女
IT 数码,体育,娱乐
汽车,时尚,美食,文艺,娱乐
时尚,投资理财,教育就业,文艺,IT 数码
文艺,体育,娱乐,健康,美食,旅游,时尚
娱乐
健康,星座命理,文艺,投资理财,时尚
投资理财,美女,健康,IT 数码,娱乐
文艺,娱乐
时尚,美食,健康,投资理财
旗袍,龙凤褂,伴娘服,礼服,婚纱
娱乐,体育,星座命理,美食,旅游,游戏动漫
旅游,娱乐,时尚,文艺,健康
儿童礼物,儿童 APP,儿歌,故事,绘本,漫画,教育,动漫,儿童,61UP
投资理财,健康,旅游,时尚
时尚,新闻资讯,星座命理,文艺
美女,娱乐
娱乐
游戏动漫,军事,体育,美女
健康,旅游,娱乐
娱乐
新闻趣事,视频音乐,星座命理,懒人,开心,看电视,双重性格
旅游
文艺,旅游,美女,星座命理,娱乐
新闻趣事,搞笑幽默,视频音乐,旅游,美食
新闻资讯
IT 数码,教育就业
穿衣美容,微博奇葩,萌宠,娱乐,星座命理
投资理财,新闻资讯,教育就业,健康

旅游,时尚,美食
新闻资讯,文艺,星座命理,美食,教育就业
娱乐
体育
军事,IT 数码,文艺,体育,娱乐
文艺,娱乐,美女,游戏动漫
小白领,POI,云迪,潮缺,神探夏洛克
旅游,文艺,美女,娱乐,时尚
娱乐
健康,娱乐,旅游,美食,文艺
旅游,投资理财,文艺
美食
体育,娱乐,美女
文艺,健康,旅游,时尚,美食
游戏动漫,旅游
美女,广东生活,新闻趣事,视频音乐
美食,狮子女,双重性格
时尚,旅游
旅游,时尚,美女,体育,娱乐
美食,游戏动漫
汽车,娱乐,文艺,教育就业,时尚,IT 数码
健康,新闻资讯,星座命理,美食,美女
美食,星座命理,旅游,健康,娱乐
文艺,投资理财,健康,美食
游戏动漫,美女,旅游,体育,IT 数码
旅游,美食,微博奇葩,穿衣美容,搞笑幽默,时尚,健康,文艺,娱乐
体育
旅游,军事,文艺
文艺,星座命理,旅游,时尚,娱乐
健康,星座命理
好好享受生活,旅游,体育
星座命理
娱乐,美食,o 型血,自由,文艺,吃货一枚
汽车,时尚,健康,IT 数码,美食,教育就业
军事
美食
爱幻想,想游遍世界,古装剧迷,有理想的 90 后,高中生,爱笑,有酒窝,爱唱歌,害怕孤独,宅
旅游,IT 数码,娱乐
健康
新闻资讯,健康,投资理财,游戏动漫,体育
旅游,投资理财,时尚,娱乐,美食
健康,投资理财,星座命理
军事
健康,文艺,娱乐

娱乐
美食,旅游
美女,新闻资讯,健康,汽车,教育就业,投资理财
教育就业
教育就业,投资理财,汽车,军事,IT 数码
娱乐,游戏动漫,星座命理,时尚,健康
投资理财,汽车,旅游,军事,IT 数码,体育
买书狂热者,吴亦凡崔胜铉,爱阅读爱摄影,电影狂热者,不看动漫会死,吃货改变世界,火影脑残粉,森系小清新,英国英剧,古风
夜猫子,旅游美食,吃
美女,娱乐,时尚,健康,军事
萌宠,微博奇葩,看书,体育,军事,IT 数码,文艺,娱乐
美食
行星家
时尚,星座命理,文艺,旅游,美食
娱乐
美食
美食,体育
旅游
美食,咖啡,文艺
体育,健康
旅游,游戏动漫,美食
娱乐
巨蟹座 AB 型,画画,创意,幻想,个性,日语,强迫症,感性,动漫,星座命理
新闻资讯,美食,教育就业,旅游,文艺,美食,军事
健康,时尚,汽车,IT 数码
体育
宠物图集,动漫,花样美男,重口味
IT 数码,美女,时尚
旅游,健康,时尚,汽车,美食
美食,体育
汽车,美食
名人明星
美女,汽车,星座命理
健康
美食,文艺,娱乐
旅游,美图摄影,娱乐
名人明星,娱乐
心情,娱乐
名人明星,健康,旅游
文艺,健康,时尚,旅游,娱乐
文艺
美食,文艺,娱乐,体育,游戏动漫
星座命理,旅游,娱乐

电脑,看小说,教育就业,体育
教育业,健康,教育就业,新闻资讯
新闻资讯,投资理财,美食,文艺,娱乐
文艺,时尚
健康,旅游,美食,军事,文艺
健康
美食,娱乐
动漫,体育资讯,美女,游戏动漫
娱乐,美食
茶,瘦身,美容护肤,摄影控,新闻资讯,旅游,时尚,健康,投资理财
IT 数码,体育
文艺,星座命理,游戏动漫,体育,娱乐
街拍,影视,自由,旅游
汽车
美女,娱乐,时尚,体育,美食,游戏动漫
投资理财,美女,美食,体育
名人明星,美食,穿衣美容,搞笑幽默,江苏生活,时尚
想御姐,粗男,学生一族
娱乐,美女,游戏动漫
美食
健康,投资理财,教育就业
北京生活,教育就业,投资理财,体育,军事,汽车
爱玩,聊天,娱乐,时尚,星座命理
文艺,健康,旅游,美食,美女,时尚
星座命理
IT 数码
美女,时尚,游戏动漫
旅游
娱乐
教育就业,汽车,旅游,体育
娱乐,体育,美食,军事,健康,旅游
美女
旅游
娱乐
旅游,投资理财,美食,IT 数码
文藝
幽默搞笑,体育,娱乐
美食,美女,旅游,星座命理,体育
娱乐
健康,文艺,旅游,美食,时尚
广告,微博,阅读,软件,社会,艺术,生活,快乐,互粉,书籍
时尚,旅游,IT 数码,文艺,娱乐
健康
旅游

娱乐
IT 数码
娱乐,新闻资讯,美女,美食,旅游,星座命理,文艺
名人明星,投资理财
言论自由,愤青,爱自由,政治,时评,爱睡,爱国,爱吃,苏打绿,努力
名人明星,视频音乐,IT 数码,娱乐,美食,旅游
体育
名人明星,视频音乐,上网,穿衣美容,宅,时尚
体育,军事,游戏动漫,美食,星座命理
时尚,旅游,美食
美女
音乐发烧友,美剧,好性格,爱情,微博控,电影迷,自由,睡觉,美图摄影
健康,娱乐
美女,汽车,游戏动漫
新闻资讯,IT 数码,美食,娱乐,美食,体育,旅游,健康
新闻资讯,时尚,旅游,美食,文艺
健康,文艺,美食,IT 数码,投资理财
健康,体育,娱乐
大学生,双鱼座,开朗,重口味,k 歌
新闻资讯
时尚,服装商场,步行街,南屏
体育
健康
军事,教育就业
美食
新疆生活,娱乐
娱乐
90 后但我不脑残,文艺,美食,旅游
时尚,健康,旅游,星座命理
星座命理,健康,旅游
游戏动漫
投资理财,文艺,娱乐
美食,穿衣美容,投资理财,星座命理,旅游,汽车
星座命理
新闻资讯,美食,时尚,娱乐
体育,娱乐,游戏动漫
旅游,教育就业,汽车,体育,娱乐
文艺,游戏动漫,旅游,美食,娱乐
汽车,美女,IT 数码,旅游
健康,旅游,美食,教育就业,文艺
文艺,一根筋,英格兰
文艺,投资理财,旅游,健康,美食
星座命理,新闻资讯,时尚,美食,健康,娱乐
汽车,健康,IT 数码,游戏动漫,美女

时尚, IT 数码, 体育, 汽车
文艺
唱歌, 自由, 文艺, 时尚, 娱乐
新闻资讯, 旅游, 美食, 文艺, 娱乐
银魂, 法语, 宠物图集, 星座命理, 军事
娱乐
娱乐, 摩羯座甜品控, 乐观主义, 歌词创作
健康, 时尚, 娱乐, 投资理财, 旅游
娱乐, 投资理财, 文艺
健康, 旅游
汽车, 游戏动漫, 美食, 旅游, 体育, IT 数码
旅游, IT 数码
时尚, 健康, 教育就业, 星座命理, 文艺
游戏动漫, 投资理财, 教育就业, 汽车, 美食
时尚, 投资理财, 文艺
新闻资讯, 美食, 文艺
IT 数码, 军事, 旅游, 游戏动漫, 时尚, 美食
美食, 健康, 时尚, 星座命理
健康, 时尚, 星座命理, 旅游, 娱乐
双鱼, 欧美, 小女人, 旅游美食, 学生一枚, 时尚, 投资理财, 美食, 文艺, 娱乐
美女
娱乐
影视, 爱吃, 旅游爱好者, 娱乐, 游戏动漫, 时尚, 星座命理, 美食
旅游, 美女, 时尚, 娱乐, 美食
健康, 时尚
文艺
汽车, 时尚, 旅游, 美食, 体育
健康
教育就业, 旅游, 文艺
名人明星, 视频音乐, 旅游, 体育, 星座命理, 游戏动漫, 娱乐
搞笑幽默
娱乐, 新闻资讯, 教育就业, 美女, 体育
体育
娱乐
健康, 娱乐, 美食, 星座命理, 文艺
娱乐
美食, 美食, IT 数码, 文艺, 娱乐
美女, 时尚, 旅游, 文艺
传媒, 爱电影, 摄影, 旅游, 投资理财, 健康, 文艺
娱乐
星座命理, IT 数码, 时尚, 旅游, 美食, 文艺, 娱乐
星座运势, 娱乐
新闻资讯, IT 数码, 文艺, 体育, 娱乐
美食, 时尚, 娱乐

美食
美食,美女,游戏动漫,时尚,娱乐,星座命理
美食
投资理财,美女,明星,热爱生活,真诚,爱玩
工程师达人,工程师技术,工程师,威客
投资理财,军事,娱乐,健康,旅游
旅游,美食,娱乐,星座命理
任性,文艺,时尚,投资理财,教育就业,旅游,汽车,星座命理
文艺
体育,nba,学生一枚,恐怖片,游戏动漫
胡思乱想,睡觉睡到自然醒,双子,music,吃货,运动,搞笑,重口味,唱K,校园
健康,新闻资讯,投资理财,传媒,媒体,玩儿,互联网,育儿,奋斗,文艺
新闻趣事,旅游,美食,美术
旅游,美女,IT数码,投资理财,军事
旅游,美食
投资理财,健康,时尚,旅游,美食
星座命理,学生,唱歌,美女
穿衣美容
美食,星座运势,美女,投资理财,文艺
健康,汽车,军事
健康,星座命理,时尚,汽车,投资理财
日本女星,电影演员,女演员,AV女优,女优,AV,坂口美穗乃,美女
军事,投资理财,美女,汽车
文艺
时尚
游戏动漫,健康,文艺
体育,美食,旅游,健康,时尚
时尚
时尚,健康,美女,文艺,娱乐
萌宠,教育就业,新闻资讯,文艺
投资理财,美食,新闻资讯,旅游,健康
新闻资讯
文艺,娱乐
美女,汽车
星座命理,健康,美食,文艺,娱乐
游戏动漫
健康,IT数码
时尚,美食,旅游,健康,新闻资讯,幸福小女人,快乐,白羊,姑娘身子爷们心,宅妈
娱乐,星座命理,帅哥,逛街,舞蹈,听音乐,健康,奋斗
体育,教育就业,健康,星座命理
吉他初学者,着迷于文学,汉服鸽派,小有执念,诗词歌赋,历史爱好者
文艺,军事,健康,旅游,汽车
美食,娱乐,文艺,旅游,体育
新闻资讯,美女,游戏动漫,娱乐

美食
军事
娱乐,旅游,投资理财,时尚,星座命理
游戏动漫,屌丝,孤僻,不合群
旅游,健康
游戏动漫
旅游,军事
美食,新闻资讯
汽车,体育
汽车,星座命理,美食,投资理财,游戏动漫,体育,娱乐
娱乐
星座命理,健康,旅游
娱乐
美女
游戏动漫,时尚,美食,文艺,娱乐
文艺
星座命理,美女,游戏动漫,军事,体育
健康,美女,时尚,汽车,娱乐
新闻趣事,视频音乐,微博奇葩,搞笑幽默,娱乐
教育就业,军事,汽车,旅游,美女,体育
美女
美食,IT 数码,文艺
时尚,美食,文艺,旅游,星座命理
军事,IT 数码
旅游,美食,投资理财,文艺,时尚
游戏动漫
教育就业,新闻资讯
美食,新闻资讯,军事,教育就业,体育
健康,新闻资讯,旅游,星座命理,娱乐
新闻资讯,旅游,星座命理,IT 数码,汽车
搞笑幽默,新闻资讯
穿衣美容
健康,时尚,旅游
教育就业,时尚,美女
健康,新闻资讯,汽车,旅游,IT 数码
旅游,健康,娱乐,汽车,体育
汽车,IT 数码
游戏动漫,美食,美女,旅游,汽车,投资理财
浪漫,茶,时尚,旅游,投资理财,文艺,娱乐
美食
星座命理
插画,投资理财,教育就业,IT 数码,文艺,娱乐
体育
投资理财,健康,美食

广西生活,新闻资讯
娱乐,时尚,不够成熟,不是很帅,教育就业
体育
娱乐,游戏动漫,美女,旅游,美食,IT 数码
搞笑幽默,美食,旅游,美女,新闻资讯,时尚,汽车,军事,IT 数码
健康,美食,时尚,教育就业,文艺
美食,健康,旅游,军事,娱乐
文艺,美女
体育
IT 数码
美食,旅游
旅游
游戏动漫,娱乐,美食
我爷们,最帅的,我娘们,最美的,说与不说,斑竹,马甲,菜鸟,白骨精,腊鸭
旅游
游戏动漫,军事,娱乐,文艺,旅游,健康,投资理财
娱乐,旅游,时尚,听音乐
宅,少女时代,神经大条,海绵宝宝,娱乐,文艺
时尚,娱乐
娱乐
汽车,游戏动漫,时尚,星座命理,文艺,娱乐
游戏动漫,星座命理,体育,IT 数码
健康
投资理财,新闻资讯,文艺
吃货一枚,爱睡觉,美食
游戏动漫
军事,投资理财,汽车,新闻资讯,IT 数码
IT 数码,旅游,娱乐,体育,美女
旅游,体育
时尚,教育就业,美食,IT 数码,娱乐
投资理财,美食,文艺,教育就业,健康
苹果,我就是我,爱幻想,奋斗,宅,90 后
投资理财
过敏体,CWOWER,间歇性整理控,随性
军事,游戏动漫,汽车,文艺
旅游,健康,教育就业
瑜伽,心静,心灵,正能量,智慧,健康
时尚
星座命理
娱乐,星座命理
时尚,娱乐,旅游
糾結體,衬衫控,阴谋论
健康
投资理财,体育,新闻资讯,军事,旅游

美女,娱乐,游戏动漫,体育
健康,旅游,军事,IT 数码,文艺,体育,娱乐
星座命理,美食,汽车,娱乐
名人明星,搞笑幽默,星座运势,时尚,娱乐,星座命理,旅游
教育就业,时尚,健康,新闻资讯,IT 数码,文艺,娱乐
美食,体育
时尚
娱乐
体育
美食,娱乐
教育就业
星座命理,军事,游戏动漫
新闻资讯,军事
体育
健康,宅女,化妆,美甲,单身
搞笑幽默,军事
旅游,美食,穿衣美容,星座命理,新闻趣事,娱乐,教育就业
时尚,汽车,旅游,美食,娱乐
星座命理,军事
时装,双子,自由
美食,星座命理
星座命理,时尚,旅游,文艺
旅游
旅游
新闻资讯,教育就业,美食,军事,汽车
美食,时尚
微博奇葩,视频音乐
文艺,投资理财,时尚,健康,旅游
美容服饰,感性,创意,shopping,看电视,娱乐,美女,健康,星座命理,时尚
新闻资讯,健康
体育,游戏动漫,军事,健康,星座命理,娱乐,美女
健康,娱乐,汽车,游戏动漫,美女
美女,时尚,娱乐
美食,健康,投资理财,新闻资讯,IT 数码
吃喝玩乐,爱自由,情感,化妆,美食,健康,时尚,旅游,娱乐
搞笑幽默,汽车,美女,文艺
美容服饰,美女
游戏动漫,美食
创意,爱生活,爱音乐,K 歌,旅游摄影,时尚,文艺,新闻资讯,健康,IT 数码
文艺,健康,旅游,美食,IT 数码
文艺
宠物图集,新闻趣事,视频音乐,cosplay,B 型血,乐天派,游戏动漫,娱乐,星座命理
八卦杂谈
教育就业,旅游,IT 数码,娱乐

新闻资讯,娱乐
军事,汽车,娱乐,体育
文艺,娱乐,体育
旅游,IT 数码,娱乐,时尚,星座命理
美女,汽车,IT 数码,游戏动漫
北京,聊天,执着,乒乓球,手机看小说,主持人,新闻资讯,文艺,军事,体育
插画,建筑,装置艺术,设计,摄影,时装,文艺,时尚
健康,新闻资讯,汽车,美食,投资理财
杰伦迷,爱哭鬼,温柔控
美女,时尚,投资理财,军事,体育
旅游,健康,美食,星座命理,投资理财
新闻资讯,时尚,美食
美食
美食
时尚,教育就业,健康,美食,娱乐
体育,新闻资讯,健康,教育就业,文艺
娱乐
投资理财,时尚,健康,美食,旅游
名人明星,娱乐
时尚,新闻资讯,文艺
台湾生活,童可可
新闻资讯
教育就业,投资理财,文艺,体育,时尚,美食,娱乐
美食
新闻资讯,教育就业,旅游,体育,娱乐
90 後,吃货,睡觉
教育就业,旅游,时尚,文艺,美食
美女,新闻资讯,游戏动漫,旅游,娱乐
IT 数码
健康,时尚,教育就业,星座命理,娱乐
微博奇葩,SuperJunior,爱睡觉,90 后非脑残,娱乐
旅游,美食
健康,投资理财,自由,旅行,时尚,宅,80 后,萝莉控,建筑,旅游
萌宠,时尚
游戏动漫,美食
文艺
体育,健康
淡定
旅游
旅游,穿衣美容,微博奇葩,视频音乐,新闻趣事
新闻资讯,娱乐,旅游,时尚,健康,投资理财
军事
教育就业,旅游,美食
健康,IT 数码,娱乐

IT 数码,星座命理
美女,游戏动漫,娱乐
時尚,健康,旅遊,美食
星座命理,健康,娱乐,时尚,旅游
游戏动漫,时尚,汽车,军事,体育
健康
贵州生活,新闻资讯,体育,汽车,军事,娱乐
投资理财
文艺
新闻资讯,旅游,文艺,美食,健康
军事
旅游,星座命理,美食,军事
搞笑幽默
体育
教育就业
健康,教育就业
娱乐,服装设计,文艺,投资理财,美女,美食,时尚
IT 数码,搞笑幽默,海外生活
娱乐
时尚,美食
汽车
旅游,文艺,美食,娱乐
文艺,娱乐
星座命理,旅游
新闻趣事,视频音乐,穿衣美容,搞笑幽默,游戏动漫,美食,IT 数码,娱乐
健康
游戏动漫,投资理财
汽车,文艺,美女,旅游,星座命理,娱乐
IT 数码
旅游,游戏动漫
教育就业
健康
射手女,睡懒觉,小清新,庚澈,股市,小资,利物浦,小提琴,通信,娱乐
美女,新闻资讯,军事,体育,娱乐
健康,美女,娱乐,军事,游戏动漫
文艺,娱乐,旅游,美食,军事
时尚,旅游,汽车,娱乐,美食
旅游
旅游,美食
时尚
穿衣美容,时尚
娱乐
汽车
Akbackup,后宫佳丽三千,摄影爱好者,美剧重症患者,渴望进入二次元

健康,新闻资讯,时尚,投资理财,旅游
星座命理,娱乐,军事,新闻资讯,体育
健康
健康
高中生,学生一族,O型血,90,IT数码
汽车,投资理财,健康,IT数码,体育
健康,旅游,娱乐
高中生,海绵宝宝,钟汉良,吸血鬼,电影迷,冷笑话,时尚,旅游,美食,文艺
美女
军事
时尚
休闲,IT数码,娱乐
星座命理
投资理财,新闻资讯,健康,旅游,体育
文艺,IT数码,旅游
教育就业,旅游,健康,投资理财
IT数码
文艺,旅游,美食,星座命理,健康
旅游,新闻资讯,美食,时尚,娱乐
汽车,IT数码
旅游,文艺
时尚,汽车,星座命理,IT数码,娱乐
美食,迷爱豆,韩剧控,学习,吃货一枚,白羊座,唱k,娱乐
旅游
旅游,美食
美食
娱乐,时尚
旅游,娱乐
吃货一个,沉默,起床困难户,爱猫,月光族
体育,军事,娱乐,文艺
视频音乐,美食
娱乐
IT数码,文艺
美食,时尚,汽车,旅游,星座命理,IT数码,娱乐,体育
娱乐
爱自由,网购,偶稀饭睡觉觉,大学生,善良,艺术,绘画,摄影爱好者,游戏动漫,美食
汽车,美女,教育就业
新闻资讯
杭州生活,游戏动漫,健康,娱乐
天蝎女,追星,90后,EXO,吴世勋,鹿晗
时尚,美食
娱乐
文艺,旅游,教育就业,汽车,新闻资讯,健康,时尚
投资理财,时尚,美食,健康

体育
时尚,星座命理,健康,旅游,娱乐
娱乐,IT 数码
新闻资讯
娱乐
游戏动漫,IT 数码,体育
投资理财,娱乐,新闻资讯,健康,旅游,美食
新闻资讯,军事,娱乐
新闻资讯,时尚,文艺
娛樂
新闻资讯,教育就业,时尚,健康,旅游
广告,阳光,幽默,摄影,时尚,旅游
军事
汽车,旅游,新闻资讯,时尚,投资理财
新闻资讯
娱乐,体育,旅游,游戏动漫,新闻资讯
美食,新闻资讯,教育就业,娱乐,时尚
时尚,旅游,美食,娱乐
房产
新闻资讯,IT 数码,军事,美食
新闻资讯,旅游,健康,美食,娱乐
教育就业,星座命理
体育,健康
旅游,健康,新闻资讯,教育就业,文艺,星座命理
健康,新闻资讯,星座命理
教育就业,星座命理,军事
美食,旅游,娱乐,新闻资讯,时尚
娱乐,星座命理
IT 数码,旅游,时尚,娱乐,文艺
旅游,体育,美食,时尚,健康,投资理财,娱乐
游戏动漫,体育,IT 数码,美女,健康,星座命理,美食,娱乐
心理学,美女,健康,教育就业,旅游,美食
新闻资讯
美女,新闻资讯,文艺,娱乐,体育
健康
专业拖稿 30 年,古风,耽美,游戏动漫
新闻资讯,时尚,旅游,文艺,娱乐
投资理财,美食,文艺
游戏动漫,文艺,体育
自由,文艺,时尚,美食
IT 数码,旅游,美女,汽车
动漫,美图摄影,星座运势,视频音乐,娱乐
教育就业,美女,新闻资讯,旅游,文艺
汽车,星座命理,旅游,娱乐,体育

健康,投资理财,旅游
时尚,汽车,教育就业
游戏动漫
游戏动漫,时尚
IT 数码,军事,健康,新闻资讯,美女
新闻资讯,时尚,健康,美食
时尚,健康,旅游,星座命理,美食
文艺,健康,美食,星座命理,娱乐
时尚,旅游
投资理财,汽车,旅游
时尚,投资理财
娱乐
新闻资讯,时尚,健康,旅游,星座命理
军事,新闻资讯
娱乐,狮子女,杨幂,旅游爱好者,90 後,星座控,微博,听歌
时尚,星座命理,美食,IT 数码
时尚,美食,旅游,健康,新闻资讯
宁波生活,微博奇葩,搞笑幽默,旅游,IT 数码,体育,娱乐
旅游,IT 数码,星座命理,娱乐
时尚,文艺,娱乐
娱乐,投资理财,美食
旅游,健康,教育就业,美食,时尚,娱乐
tvb,90 后非脑残,新闻资讯,教育就业,娱乐,游戏动漫
游戏动漫,体育
旅游,文艺
美食,娱乐
旅游,娱乐
IT 数码
美食,旅游,健康,新闻资讯,IT 数码,体育,娱乐
美食,娛樂
旅游,娱乐
新闻资讯
旅游,文艺
健康
旅游
娱乐,music
时尚,汽车
美食,游戏动漫,娱乐
娱乐
美食
时尚,娱乐
星座命理,健康,汽车,时尚,文艺,旅游
电视剧,五月天,创意,阳光,教育,舞蹈,爱生活
星座命理,IT 数码,体育

电子商务,爬山,爱生活,IT 互联网,瑜伽,小清新,烹饪,音乐爱好者,娱乐,旅游
娱乐,体育,健康
星座命理,美食,美女,游戏动漫,健康,文艺,娱乐
新闻资讯,文艺,教育就业
体育
美食,体育
健康,美食
美女,娱乐
体育
投资理财,美食
美女
文艺
娱乐
新闻资讯,教育就业,投资理财,游戏动漫,IT 数码
新闻资讯,旅游,健康,娱乐
旅游,美食,娱乐,体育
新闻趣事,搞笑幽默,安徽生活,微博奇葩,新闻资讯
汽车,旅游,美食,IT 数码,文艺
白羊座
体育,教育就业
健康,时尚,汽车,教育就业,投资理财
投资理财,健康,教育就业,新闻资讯
江苏生活,新闻趣事,游戏动漫
体育
新闻资讯,茶,健身,美食,体育,健康
军事,健康,汽车,教育就业,体育
娱乐,文艺,喜欢音乐,文字,开朗,热爱生活,写作
体育,美食,旅游,健康,教育就业
美女,军事
IT 数码
时尚,美食,娱乐,文艺,旅游
星座命理
天蝎座,听音乐,登山,星座命理
旅游,星座命理,美食,娱乐
投资理财,教育就业,游戏动漫
腹黑,疯狂,娱乐
娱乐
时尚,美食,旅游
时尚,汽车,娱乐,IT 数码,文艺
IT 数码
娱乐,美食,IT 数码,时尚,文艺
名人明星,游戏动漫
体育,娱乐
视频音乐,游戏动漫,时尚

美食
美女,美食,军事,IT 数码,娱乐
美食,星座命理,娱乐
星座命理
旅游,娱乐,文艺,美食,星座命理
新闻资讯
美食,搞笑幽默
健康,汽车,星座命理,美食,旅游
陕西生活,文艺
游戏动漫,新闻资讯,美女,军事,IT 数码
美食,旅游,新闻资讯,IT 数码,汽车,文艺
美食,美女,健康,时尚
IT 数码,星座命理,游戏动漫
旅游,视频音乐,美女,新闻趣事,搞笑幽默
萌宠,娱乐,文艺,体育
汽车
娱乐,新闻资讯,健康,美食
绘画,旅行,80 后,动漫,时尚,上网,健康,旅游,美食,文艺
IT 数码
旅游
旅游,体育
汽车,美女,游戏动漫,旅游,娱乐
美食,美女,文艺,汽车,IT 数码
文艺
时尚,新闻资讯
汽车,美女,教育就业,健康,美食
新闻资讯,文艺,游戏动漫
旅游
娱乐,美食
画画,成长中
旅游
萌宠,搞笑幽默,穿衣美容,时尚
时尚,旅游,美食,娱乐
模特,拍照,Music,时尚,旅游,星座命理,美食
漫画,羽毛球,淡定,宠物,重口味,美食,游戏动漫
娱乐,星座命理,文艺
自拍,美女,淘宝控,淘宝,网络营销,女装网店,电子商务,网购,互联网,时尚
文艺,旅游,时尚
投资理财,时尚,健康,旅游,美食
美食,搞笑幽默,江苏生活,视频音乐,穿衣美容,美食爱好者,小说,时尚,文艺,娱乐
时尚,文艺,健康,美食,旅游
新闻资讯,投资理财,旅游,娱乐,游戏动漫
健康,文艺,娱乐
健康,时尚,旅游,IT 数码,娱乐

校园生活,搞笑幽默,文艺,游戏动漫
营销,音樂,哲学,快乐大本营,搞怪,健康,新闻资讯,美女,汽车
IT 数码,娱乐
健康,旅游,美食,文艺,娱乐
时尚,娱乐
2B,电影,汽车,旅游,文艺
文艺,旅游,星座命理
校园生活,娱乐,投资理财,旅游,军事,体育
投资理财,美食,旅游,健康,体育
矛盾综合体,成都控,强迫症,五月天
时尚,美食,体育
文艺
IT 数码,教育就业,美女,军事,文艺
时尚
新闻资讯
胡思乱想,睡觉睡到自然醒,格子控,护肤,美容,随性,粤语,怀旧,娱乐,健康
新闻趣事,投资理财,新闻资讯
教育就业,新闻资讯,星座命理,时尚
健康,时尚,美食
旅游,健康,游戏动漫,文艺,IT 数码,星座命理,巨蟹男
投资理财,军事,娱乐
旅游,汽车,IT 数码,体育
游戏动漫
娱乐,健康,文艺
新闻资讯,健康,娱乐,美食,旅游
投资理财
时尚,美女
IT 数码
美食,娱乐,星座命理
星座命理,娱乐,文艺,汽车,美食
军事,新闻资讯,星座命理,文艺,教育就业,健康,美食,娱乐
娱乐,军事,IT 数码,文艺,体育
搞笑幽默
旅游
军事,体育
美女,投资理财,新闻资讯,旅游,军事
淘宝控,张杰,看小说,购物狂,谢娜粉丝,家居,服装,女性,美食,娱乐
娱乐
IT 数码,教育就业,新闻资讯
美食,星座命理
娱乐
娱乐
时尚
星座命理,穿衣美容,美食,旅游

美食
旅游,健康,美食,军事
美食,星座命理
汽车
新闻趣事,校园生活,绍兴生活,体育,教育就业,新闻资讯,旅游,星座命理
体育,旅游,汽车,游戏动漫,军事,娱乐
娱乐
动漫,体育资讯,夏侯惇,三国迷,海贼王,巨蟹座,陈奕迅,动漫迷
星座命理
旅游,健康,时尚
旅游,星座命理,美食,美女,IT 数码
视频音乐,新闻趣事,星座命理,微博奇葩,搞笑幽默,投资理财,游戏动漫,健康
旅游,山东生活,搞笑幽默,穿衣美容,星座命理,娱乐,投资理财,时尚
美食,游戏动漫
时尚,新闻资讯,娱乐,美女,健康,旅游
健康,新闻资讯,美食
娱乐
美食,时尚
美女,时尚,军事
教育就业,游戏动漫,时尚,健康,旅游
名人明星,文艺
旅游,看书,健康
娱乐
美女
体育,军事
健康,美食,IT 数码,投资理财,时尚,旅游,美食,娱乐
旅游
旅游,健康,新闻资讯,教育就业,文艺,娱乐
伊犁生活,投资理财,新闻资讯,文艺,军事
美食,文艺,娱乐,星座命理
时尚,娱乐
旅游
互粉,军事,旅游
投资理财,健康,美女,游戏动漫,IT 数码
体育,汽车,游戏动漫
投资理财,美食
热爱自由的双子,特大号精神病
体育
美女
新闻资讯,汽车,投资理财
游戏动漫
文艺,猫控
校园生活,化妆品,文艺
穿衣美容,90 后,大学生,电影,音乐,时尚,美食,宅女,旅行,睡觉

文艺,时尚,星座命理,旅游
新闻资讯,健康,旅游,投资理财,汽车
时尚,旅游,文艺,游戏动漫,星座命理,美食,娱乐
健康
投资理财,汽车,旅游,IT 数码
军事,游戏动漫,星座命理,美食,娱乐
美女,娱乐,时尚,健康,旅游
投资理财
IT 数码,新闻资讯,旅游,军事,娱乐
汽车
美食,美女,美食,旅游,健康,体育,游戏动漫
体育,投资理财,时尚,旅游,文艺
旅游,美食,时尚,IT 数码,娱乐
旅游,美女,星座命理,体育,娱乐
同志,科技,微博,法律
娱乐,体育
美食,汽车,投资理财
美食,旅游,娱乐
旅游,军事
美食,新闻资讯,健康,星座命理,文艺
美食
冷笑话,团购
南京生活
旅游
IT 数码
军事,投资理财,新闻资讯
体育
娱乐
时尚
健康
交友,90 後,懒人,娱乐,美女,汽车
投资理财,健康
爱吃,旅游爱好者
浙江生活,体育
美女,娱乐,时尚
娱乐
新闻资讯,旅游,美食
芜湖生活,IT 数码,单身,宅,数码,唱歌,音乐,安静
文艺,汽车,游戏动漫
教育就业,新闻资讯,健康,旅游,摄影师,真诚,青春,汽车,军事
旅游
美食,新闻资讯,教育就业,体育,美女
体育
娱乐

旅游
旅游,教育就业
健康,旅游,美食,文艺,娱乐
健康,美食,星座命理,旅游,娱乐
时尚
视频音乐,体育
新闻趣事,视频音乐,微博奇葩,湖北生活,武汉,喜欢听歌,旅游,健康,文艺
娱乐,时尚
爱睡觉,宅,刘同
汽车,IT 数码,军事,美食,文艺
美女,军事
IT 数码
汽车
娱乐
娱乐
娱乐,美食,文艺,旅游
经济,投资理财,军事
美女
健康,投资理财,体育
旅游,星座命理,文艺
军事,健康
汽车
教育就业
美女,旅游,文艺,星座命理,游戏动漫,娱乐
微博奇葩,健康
体育
游戏动漫,汽车,星座命理,IT 数码,娱乐
娱乐
旅游,星座命理,时尚,娱乐,美食
新闻资讯,教育就业,美食,旅游,美女,汽车
汽车,军事,IT 数码,美食,旅游
娱乐
美女,自拍,健康
游戏动漫,体育
游戏动漫
美女
娱乐,美食,时尚,美女,旅游
名人明星,情感生活,美容服饰,迪庆生活,旅游,时尚,游戏动漫
投资理财
美食,文艺
八卦杂谈
IT 数码
天津生活,读书分享,旅游,名人明星,美食,新闻资讯,时尚,体育
体育

旅游
多肉控,金牛座,美剧,健康
星座命理,旅游,游戏动漫,美食,文艺
搞笑幽默,娱乐
健康
文艺,时尚,汽车,旅游,美食,娱乐
旅游
IT 数码
健康,旅游,文艺,体育,娱乐
美食,游戏动漫
旅游爱好者,热情,阳光,爱生活,娱乐,教育就业
美食
云南生活,健康,军事,IT 数码,体育
汽车,旅游
IT 数码
IT 数码,娱乐
星座命理,旅游,健康,美食,娱乐
美食,旅游,汽车,娱乐,军事
健康,投资理财,旅游,时尚,IT 数码,文艺,体育,娱乐
娱乐,美食
投资理财,军事,文艺,新闻资讯,健康
军事
时尚,美女,美食,娱乐
旅游,美食,军事,娱乐,体育
游戏动漫,旅游,体育
健康,旅游
娱乐
旅游
美食
安徽生活,旅游,汽车,IT 数码,娱乐
健康,时尚,美食,文艺
健康,旅游,娱乐
游戏动漫,樱狼,入野自由,音乐,动漫,日剧
星座命理
旅游,美食,长沙生活,美容服饰,穿衣美容
IT 数码,体育
摩天轮,巴黎,普罗旺斯,韩国,旅游,星座命理,美食,娱乐
体育
教育就业,健康,旅游,新闻资讯
投资理财,美女,娱乐
自主创业
吉林生活,旅游,穿衣美容
旅游
游戏动漫

星座命理,爱睡觉,獅子座,爱笑爱哭爱闹,电玩控
星座运势,健康,时尚,娱乐,旅游
健康,旅游,美食,娱乐,新闻资讯,时尚
体育
美食,娱乐,新闻资讯,时尚,美女
时尚
新闻资讯,投资理财
健康
军事
美女
健康,教育就业,投资理财,新闻资讯,文艺
健康,IT 数码,美女,文艺,新闻资讯,汽车,星座命理
萌宠,时尚,文艺
美食,星座命理,IT 数码,体育
新闻资讯,投资理财
娱乐
美食,文艺
时尚,美食
搞笑幽默,星座命理,美食,萌宠,微博奇葩,健康,新闻资讯
美女,游戏动漫,汽车
时尚,健康
军事,体育,IT 数码,投资理财
文艺,健康
投资理财,时尚,健康,旅游,美食
旅游
体育,旅游,健康,美食,军事
娱乐,体育,美女
穿衣美容
健康,汽车,旅游,星座命理,美食
旅游,健康,时尚,美食,文艺
体育
權志龍,耽美,白羊女,小女人,奋斗,喜欢听歌
sj
汽车,星座命理,IT 数码,体育,娱乐
美食,新闻趣事,搞笑幽默
时尚,旅游,投资理财,娱乐
投资理财
体育,军事,投资理财,美女,娱乐
文艺
文艺
文艺
娱乐,旅游,健康,美女,时尚
健康,旅游,美食,投资理财
美食,体育,娱乐,旅游,星座命理

娱乐,美食
视频音乐,搞笑幽默,美容服饰,伊犁生活,健康,星座命理,美食,时尚,娱乐
投资理财,娱乐,新闻资讯,健康,军事
新闻资讯,时尚,文艺,旅游,游戏动漫,星座命理,美食
IT 数码,教育就业,旅游
娱乐
时尚,旅游,美食
美女
美食,时尚,新闻资讯,旅游,文艺
健康,娱乐,汽车,美女
名人明星
其他生活,新闻趣事,旅游,美食,穿衣美容
时尚,旅游,娱乐
文艺,星座命理,时尚,健康,美食
投资理财,军事
VIP
时尚,星座命理,健康,旅游,娱乐
娱乐
IT 数码,旅游,美女,投资理财,娱乐
游戏动漫
新闻资讯
汽车,健康,体育,旅游
美食,星座命理,文艺
美食,旅游,萌宠,穿衣美容
星座命理,健康
完美主义,读书,强迫症,动漫
健康,美食
娱乐,新闻资讯,健康,美食
美食,健康
游戏动漫,娱乐,文艺,旅游,美食
美食
娱乐,时尚,健康,旅游,美食
读书分享,IT 数码,文艺,体育
不了,不明
美食,旅游,文艺,娱乐,军事,体育
舞蹈,魏晨,陈翔,明星,狗狗,武艺,音乐爱好者,旅游,星座命理,美食
健康,美食,文艺
娱乐
旅游,投资理财,新闻资讯,娱乐,时尚
时尚,IT 数码,美食,娱乐
双子女,cosplay,乐天派,美食,游戏动漫
游戏动漫,健康,旅游
新闻资讯
娱乐

时尚,旅游,文艺,娱乐,星座命理
美食,星座命理
时尚,美食,旅游,文艺,娱乐
森林,人道,书虫,懒,在旅途
旅游,新闻资讯,体育,娱乐,汽车
旅游,汽车,美食
旅游摄影,热情,巨蟹
娱乐,温柔,美女
娱乐
美食,娱乐
IT 数码,娱乐
旅游,新闻资讯,投资理财,IT 数码,汽车
穿衣美容,娱乐
时尚,娱乐,美食
新闻资讯,健康,旅游,美食
旅游,美食,文艺,娱乐
军事,美食,旅游,星座命理,汽车,游戏动漫,文艺
娱乐,美食,健康,旅游,星座命理
旅游
汽车,娱乐
听音乐,发呆,各种宅
日本生活,美食
美女
娱乐
新闻资讯,娱乐,时尚,教育就业,旅游
TaeYeon 金泰妍,Nichkhun,2pm,紫色,少女时代,晶茜,宋茜,FX,维尼夫妇
娱乐
旅游,星座命理,娱乐
新闻资讯,美食,教育就业,旅游,文艺,娱乐
汽车,旅游,新闻资讯
投资理财
娱乐
时尚,美食,星座命理,游戏动漫,文艺
军事
星座命理,微博奇葩,美食,娱乐
美女
美食,体育,军事,娱乐
娱乐,旅游
健康,星座命理,文艺
健康,文艺,旅游
健康,娱乐
微博奇葩,孩子氣,天秤座,起床困难户,潇洒,电脑
旅游,教育就业
时尚,IT 数码,娱乐

娱乐
汽车,文艺,旅游,美食,健康
投资理财,新闻资讯,健康,旅游,文艺,教育就业
星座命理
投资理财,教育就业,时尚,汽车
时尚,旅游,美食,娱乐
坚强
投资理财,美食,星座命理,旅游,娱乐,IT 数码
旅游,美女,想去旅行,健康
美女
体育
美女,体育
萌宠,福建生活,搞笑幽默,投资理财,新闻资讯,旅游,美食
健康,时尚,文艺,美食,星座命理
双鱼,摄影爱好者,赛车
诗词,艺术,钢琴,旅游摄影,shopping,时尚,文艺,娱乐,星座命理,旅游
旅游
IT 数码,娱乐,汽车,旅游
文艺
娱乐,美女
旅游,教育就业,投资理财
搞笑幽默,时尚,健康,娱乐
美食
汽车,星座命理,美女,游戏动漫,IT 数码
美女,娱乐
游戏动漫,旅游,文艺,娱乐,星座命理
健康,体育,旅游,时尚,文艺
时尚,娱乐
香蕉
投资理财,文艺,美食,星座命理,教育就业
美食
星座命理
美食,八卦杂谈,名人明星,校园生活,搞笑幽默
80 后,爱生活,心理学,购物狂,幽默,交朋友,音樂,时尚,游戏动漫,娱乐
教育就业,美女,文艺,IT 数码,军事,娱乐
游戏动漫
旅游,文艺,IT 数码,美食,星座命理,娱乐
旅游
体育
投资理财,健康,美食,旅游,文艺
游戏动漫,娱乐
美食,星座命理,娱乐
新闻资讯,旅游,美食,时尚,汽车
尼古拉斯霍尔特,上网,排球,科幻,强迫症,王力宏,射手女,旅游

旅游,美食,文艺,时尚
文艺,星座命理,游戏动漫,娱乐
视频音乐,教育就业
视频音乐
美食,健康,旅游,文艺
旅游,美女,时尚,娱乐,星座命理
军事,星座命理,投资理财,娱乐,旅游,汽车,游戏动漫
新聞資訊,旅遊,健康,美食,娛樂
健康,旅游,IT 数码,军事,宅,上网,学生
星座命理,穿衣美容,视频音乐,美食
IT 数码
汽车
视频音乐,体育资讯,美图摄影,旅游,健康,时尚
投资理财,健康,旅游,军事,美食
健康,投资理财,美食,军事,新闻资讯
旅游,IT 数码
旅游,美女,时尚,文艺,娱乐
旅游,汽车,文艺,星座命理,健康,娱乐
文艺
时尚,星座命理,游戏动漫,健康,旅游,美食
星座命理,吃喝玩乐,星座控,音乐达人,摩羯女,美食,唱 k,娱乐,学生,美食爱好者
时尚,健康,娱乐
娱乐
新闻资讯,文艺,星座命理,健康,投资理财,时尚
文艺,游戏动漫,星座命理,时尚,娱乐
新闻资讯
娱乐
文艺,投资理财,美女,健康,旅游,美食
旅游,汽车,娱乐,IT 数码
旅游,时尚,汽车,投资理财
体育,集贤中学 808
娱乐
文艺,时尚,健康,旅游,美食
穿衣美容,娱乐
教育就业,汽车,投资理财,新闻资讯
健康,美女,游戏动漫,娱乐,体育
新闻资讯,健康,旅游
体育,娱乐,美女,美食
文艺
善良,寂寞,教育就业,游戏动漫,军事,汽车
娱乐,美食,旅游
娱乐
美食
逛街,旅游

健身运动,功能性训练,篮球,英式橄榄球,健康,美食,体育
旅游
IT 数码
时尚,旅游,美食,娱乐,美女
军事,IT 数码,文艺,体育,娱乐
美女,时尚,健康,旅游,文艺
美食
汽车,教育就业,投资理财,军事,娱乐
山东生活,穿衣美容,旅游,时尚,美食,娱乐
健康,美食,文艺,旅游
新闻资讯,教育就业,军事
懒,吃货一枚,爱笑,学生一枚
旅游,文艺
美女,新闻资讯
美食
时尚,娱乐,文艺,美食,美女
新闻资讯,健康,时尚,美食,旅游
新闻资讯,旅游,教育就业,文艺
娱乐
旅游,星座命理,美食,娱乐,文艺
新闻趣事,教育就业,聚会、聊天、旅游
美食,美女,美食
青春,美食
娱乐,IT 数码,美食,游戏动漫
搞笑幽默,时尚
萌宠
体育,娱乐,军事,IT 数码,旅游
简单
健康,新闻资讯,美女,游戏动漫,时尚
文艺,时尚,旅游,美食
娱乐,美食,旅游,投资理财,新闻资讯
游戏动漫
双子座,建筑,吃货,动漫
少女时代,音樂,视频,娱乐,美女,美食,投资理财,健康
汽车
旅游,游戏动漫,美女,体育,娱乐,军事
美食,健康,教育就业,新闻资讯,星座命理,游戏动漫
武汉生活,新闻趣事,微博奇葩,美食,穿衣美容
文艺
星座命理
娱乐,文艺
健康,旅游,美食,娱乐
旅游,健康,美食
旅游,星座命理,重庆生活,搞笑幽默,新闻趣事,新闻资讯

体育
体育
旅游,美食,星座命理
地产,篮球爱好者,旅游,美食,体育,娱乐
旅游,星座命理,美食,文艺,娱乐
星座命理
IT 数码
汽车
旅游,健康,娱乐,星座命理
娱乐,时尚,健康,美食,星座命理
名人明星,教育就业,文艺,体育,新闻资讯,军事
时尚,星座命理,美食,文艺,娱乐
军事,投资理财,新闻资讯,娱乐
文艺
教育就业,健康,旅游,美食,娱乐
星座命理,美女,娱乐,教育就业,美食
旅游,星座命理,娱乐
视频音乐
娱乐,汽车,健康,军事,IT 数码
IT 数码,投资理财,时尚,美女,娱乐
时尚,美食,文艺
投资理财,健康,旅游,时尚,夜猫子,星座命理,文艺,娱乐
文艺,时尚,旅游,健康,美食
美食
新闻资讯
美食,娱乐,旅游,星座命理,体育
旅游,新闻资讯,投资理财,汽车,星座命理
汽车,新闻资讯,投资理财,旅游,娱乐
美食,健康,旅游,星座命理
投资理财,娱乐,文艺,健康,美食
健康
微博奇葩,美食,名人明星,新闻趣事,穿衣美容,投资理财,时尚,健康
游戏动漫,教育就业,文艺,健康,美食
旅游,文艺,星座命理,娱乐,时尚
汽车,IT 数码,体育,新闻资讯
娱乐,文艺,体育
宅女
军事,IT 数码,文艺
军事,新闻资讯
旅游
投资理财,旅游,健康,美食,文艺
旅游,健康,教育就业
美食,体育
文艺,时尚,娱乐,旅游

时尚
体育,星座命理,美女,时尚,汽车
文艺,新闻资讯,体育
名人明星,萌宠,穿衣美容
新闻趣事,新闻资讯
游戏动漫,文艺,时尚,娱乐
健康,教育就业,娱乐,旅游,星座命理
时尚,体育,旅游,新闻资讯,汽车
兄弟控,忠犬控,幼驯染控,吴邪,狱纲,黄黑
不是善人,人体战士,小仙女
军事
娱乐
时尚,文艺,旅游,星座命理,美食,娱乐
投资理财,旅游,美食,文艺,娱乐
军事,娱乐,体育,文艺
名人明星,搞笑幽默,汽车,IT 数码,文艺,体育,娱乐
娱乐,文艺,旅游,文字,双子,睡觉,时尚,学生
健康,投资理财
美食,教育就业,文艺,时尚,健康
旅游,时尚,美女,文艺,娱乐
健康,时尚,投资理财,教育就业
娱乐
O 型血,学生一枚,胡歌,吃货一枚,怀旧,追星,咖啡,电脑,校园,美食控
娱乐,健康,文艺,美女,星座命理
学生一枚,好书,玩儿,娱乐
微博奇葩,江苏生活,星座命理,视频音乐,新闻趣事,新闻资讯
娱乐
糗事,幽默,开心果,娱乐
健康,文艺,军事,旅游,星座命理
星座命理,文艺,美食
发泄,户外,美食,文艺,娱乐,教育就业,时尚,旅游,新闻资讯,健康
娱乐
搞笑幽默,娱乐
汽车,IT 数码,恐怖片,厦门
动漫迷,球迷,游泳爱好者,军事,muslimbrother,真诚做人
微博奇葩,新闻趣事
文艺,投资理财,娱乐,新闻资讯,健康
投资理财,汽车,IT 数码,时尚
文艺,游戏动漫,娱乐,星座命理
忧郁,旅游,美女,汽车,体育,娱乐
新闻资讯,体育,军事
娱乐,美女,美食,游戏动漫,文艺
旅游美食爱好者,平常心,水瓶座加 O 型血,快乐,乐观,教育,娱乐,旅游,健康
星座命理,娱乐

萌宠,娱乐
星座命理
旅游,美食
游戏动漫,健康,旅游,美食,娱乐
娱乐,时尚
军事,游戏动漫,汽车,教育就业,美食,娱乐
健康,星座命理,90 后,宅,游戏动漫,时尚,娱乐
投资理财,星座命理,娱乐,健康,美食
娱乐,游戏动漫
体育
美食,时尚,美食,娱乐
娱乐,教育就业
娱乐,时尚,美食,星座命理,文艺
搞笑幽默,梦想
娱乐
体育
健康,文艺,投资理财
星座命理,体育,娱乐
新闻资讯,文艺
萌宠,视频音乐,搞笑幽默,旅游,美食,游戏动漫,汽车,文艺
文艺,体育,娱乐,汽车,旅游
旅游,时尚,美食,星座命理,娱乐
星座命理
微博奇葩,穿衣美容,美女,视频音乐,星座命理,文艺,娱乐,时尚,健康
教育就业,时尚,旅游,健康,美食
游戏动漫,时尚,娱乐,星座命理,旅游
投资理财
旅游,星座命理,健康,美女,游戏动漫,投资理财,美食
游戏动漫
军事,旅游
游戏动漫
投资理财,文艺,体育,娱乐
新闻资讯,汽车,军事,IT 数码,游戏动漫
笑,天枰座
游戏动漫
时尚
新闻资讯,旅游,美食,健康,娱乐
美女
美剧,宅,爱生活,爱睡觉,明星,狗狗,格子控,爱旅行,美食
美女
娱乐,旅游,美食
娱乐,美食
文艺,娱乐
美食

游戏动漫
时尚
星座命理,美食,旅游
星座命理,美食,游戏动漫,教育就业,娱乐
美女,汽车,旅游,娱乐,体育,游戏动漫
美食,游戏动漫,娱乐
时尚,健康,旅游,投资理财,娱乐
电影,自由,旅行,美食,80后,90后,听歌,时尚,旅游,星座命理
美食,旅游,星座命理,时尚
交友,水瓶座,娱乐
游戏动漫
射手,睡觉睡到自然醒,80後,汽车,娱乐
旅游
新闻资讯,旅游,军事,美食,汽车
娱乐,旅游
体育,美女,旅游
美女,投资理财
吃货,外貌协会,奋斗,懒,安静,快乐,互粉,IT数码,军事,星座命理
文艺
汽车,IT数码,时尚,旅游,美食
体育
文艺
文艺
教育就业
旅游,星座命理,美食,娱乐
健康,旅游,美食,新闻资讯,娱乐
教育就业,美食,健康
汽车,mba联考,赚钱,美女,房地产,教育就业,新闻资讯
娱乐
美女,时尚,星座命理,娱乐
文艺,娱乐
娱乐,美食,游戏动漫
美女,体育,娱乐,美食,游戏动漫,巨蟹
健康,新闻资讯
星座命理
健康,投资理财,旅游,星座命理,美食
娱乐,体育
爱
娱乐,体育,新闻资讯
宁波生活,体育
视频音乐
健康,游戏动漫,娱乐,旅游,文艺,美食,星座命理
新闻资讯,旅游
娱乐,时尚,文艺,投资理财,旅游

新闻资讯
美食,娱乐
旅游
旅游,广东生活,新闻趣事,搞笑幽默,穿衣美容,娱乐
美食,新闻资讯,旅游,投资理财
游戏动漫,教育就业,文艺,时尚,娱乐
健康,新闻资讯,教育就业,游戏动漫
旅游,健康,美食,娱乐,星座命理
娱乐
体育,投资理财,军事,新闻资讯,文艺
美食,新闻资讯
旅游,娱乐,文艺,生活,矛盾体,多愁善感,学生,宅,听歌,90 后
IT 数码,汽车,旅游,美食,娱乐
星座运势,星座命理,娱乐
纠结,旅游摄影,独立,投资理财,无厘头,创业
健康,新闻资讯,时尚,投资理财,星座命理
军事,IT 数码
文艺
美食,文艺,游戏动漫
健康,体育
娱乐
汽车,美女,游戏动漫,旅游,娱乐
旅游,美食,娱乐
健康,美食
游戏动漫,娱乐,美食
星座命理,游戏动漫,美女,IT 数码,体育
旅游,IT 数码,美食,游戏动漫,娱乐
游戏动漫,娱乐
旅游,星座命理,美食
军事,汽车
旅游
科学探索,动漫,搞笑幽默,旅游
美食
时尚,教育就业,娱乐,文艺,旅游,健康
金牛,偶像剧,90 后但我不脑残,天蝎,武汉,追星,炎亚纶,喜欢唱歌,布丁
娱乐
新闻趣事,学生一族,新闻资讯,时尚,文艺
自拍,fashion,80 后,自由,美食,星座命理,娱乐,健康
娱乐,汽车,IT 数码,美女
新闻资讯,教育就业
健康,新闻资讯,时尚,旅游,文艺
沈阳生活
汽车
体育

投资理财,教育就业,时尚,美食,体育
新闻资讯,IT 数码,教育就业
星座命理,旅游,时尚,美食
美食
IT 数码,旅游
学生一枚,疯子,看小说,爱幻想,学习,懒人,古典艺术,文艺
IT 数码
时尚
新闻资讯
文艺,旅游,娱乐
心理,养生,iphone,猫咪控,医生,书籍,电影迷,电脑,新浪读书,格子控
旅游,火星人在地球
健康,新闻资讯,汽车,体育,娱乐
健康,星座命理,IT 数码,投资理财,时尚
体育,游戏动漫,娱乐
美女,时尚,文艺,教育就业,健康,娱乐,新闻资讯
星座命理,IT 数码,我就是我,90 后非脑残,听音乐,旅游,汽车
娱乐
时尚
名人明星
体育
美食
健康,新闻资讯,旅游,文艺
美女,体育
娱乐
动漫控,漫画,画画,三国杀,萌宠,美食
体育,星座命理,美食,军事,IT 数码
新闻趣事,文艺,娱乐
旅游
名人明星,重口味,花样美男,旅游,八卦杂谈,星座命理
电影,自由,旅行,时尚,旅游,美食,80 后,投资理财,军事,体育
娱乐,星座命理
时尚
日本生活,星座命理
海外生活,文艺
IT 数码
星座命理,娱乐
萌男控,奇葩,傻屌
游戏动漫,军事
星座命理,文艺,时尚,娱乐
娱乐,文艺,时尚,美食
娱乐
名人明星,美食,旅游
时尚,文艺,美食,旅游,娱乐

音乐爱好者,热爱生活,爱吃,快乐,视频音乐,微博奇葩,火影,文艺
电影,自由,旅行,旅游,美食,80后,90后,健康,时尚,IT数码
电影,自由,旅行,旅游,美食,80后,90后,时尚,军事,娱乐
美女
电影,自由,旅行,时尚,旅游,美食,80后,新闻资讯,汽车,IT数码
电影,自由,旅行,美食,80后,90后,听歌,时尚,旅游,IT数码
电影,自由,旅行,美食,80后,90后,听歌,时尚,旅游,IT数码
美食
美食,美食
新闻资讯,健康,旅游,星座命理,美食,娱乐
娱乐,教育就业
游戏动漫,美食,时尚,IT数码,体育
旅游,娱乐
文艺,美食,旅游,健康,教育就业,新闻资讯
美食
游戏动漫
练白龙,秋濑或,金越,利艾,伏八,伊东歌词太郎,まふまふ,あまつき
时尚,美食,星座命理,IT数码,文艺,娱乐
电影,自由,旅行,时尚,旅游,美食,80后,健康,投资理财,IT数码
文艺,美食,汽车
星座命理
汽车,娱乐,军事,体育
汽车,IT数码,军事,美食,体育
美女
新闻资讯,健康
新闻资讯,旅游,美食,投资理财,健康
游戏动漫,娱乐,体育,星座命理
星座命理,IT数码,新闻资讯,汽车,军事
娱乐
校园生活,体育资讯,搞笑幽默,健康,新闻资讯,体育
美食
诚信,正品,闲置,义卖,转让,慈善,心理学,爱心传递
美食
星座命理,IT数码,娱乐
健康,IT数码,旅游,军事,汽车
浙江生活,健康,时尚,旅游,教育就业,体育
旅游,娱乐
旅游,星座命理
时尚
厦门生活,时尚
教育就业,旅游
旅游,娱乐,星座命理,美食,文艺
时尚,娱乐,文艺,美食,星座命理
娱乐,汽车,新闻资讯,旅游,IT数码,军事,体育

健康,星座命理,娱乐,时尚
健康,汽车,美食,军事
旅游,健康,时尚,娱乐
双鱼座,爱睡觉,电影爱好者,自拍,旅游美食,向日葵,简单,文艺,时尚,美食
文艺,新闻资讯,汽车,健康,星座命理
星座命理
美食
视频音乐,娱乐
旅游,美女,新闻资讯,星座命理,娱乐
娱乐,love
旅游,名人明星
时尚,旅游,IT 数码,娱乐
体育资讯,粤语,体育
体育,健康
美女,娱乐,游戏动漫
体育
娱乐
娱乐,美食,旅游,投资理财,健康
新闻资讯,投资理财,IT 数码,军事
教育就业,投资理财,娱乐,体育,星座命理
体育
娱乐,美女
旅游,体育
娱乐
文艺,美食,新闻资讯,旅游,健康
健康,游戏动漫,美食,娱乐,星座命理
nba,音乐控,体育,娱乐,游戏动漫,健康,旅游
宠物图集,星座命理
美食
时尚,新闻资讯
时尚,美食,体育,健康
体育
游戏动漫,娱乐
文艺
音乐,电影迷,瘦身,养生,宠物,心理学,数码,娱乐
游戏动漫
军事,美女,旅游,美食,IT 数码,体育
旅游,投资理财,娱乐,美食,健康,时尚
双子座,爱萌物,爱动漫,爱闺蜜,爱紫绿
时尚,娱乐,星座命理,旅游
体育
时尚,旅游,娱乐,健康
汽车,军事
旅游,时尚,文艺,星座命理

时尚,美食
搞笑幽默,美女
健康,旅游,娱乐
美食,娱乐
娱乐
美女
文艺,体育,娱乐
教育就业,时尚,美食,文艺,娱乐
娱乐,文艺,旅游,星座命理,时尚
IT 数码,美食,汽车,时尚,旅游
美食,IT 数码
投资理财,宅,睡觉,我就是我,哈哈,独立,时尚,旅游
新闻趣事,体育,汽车
文艺,美食,娱乐
军事,星座命理,健康,文艺,娱乐
时尚,娱乐
唱 K,美剧迷,90 后,电影,爱笑,创意,爱生活,设计,努力,文艺
军事,美食,星座命理,美女,游戏动漫,时尚,娱乐
健康
新闻趣事,旅游,美食,深圳生活,星座命理
教育就业,时尚,健康,旅游,美食,文艺,娱乐
新闻资讯,投资理财,军事,健康,文艺
健康,旅游
摩羯座,我就是我,矛盾,奋斗,吃货一枚,随性,淡定,休闲,旅游
微博奇葩,星座命理,新闻趣事,搞笑幽默,娱乐
星座命理
文艺
美食
新闻资讯,游戏动漫,汽车,军事,IT 数码
新闻资讯,娱乐,美女,时尚
星座运势,星座命理,聊天,心情,跆拳道,爱幻想
微博奇葩,视频音乐,搞笑幽默,穿衣美容,时尚,娱乐,文艺,健康,旅游
新闻趣事,星座命理,微博奇葩,时尚,游戏动漫,健康,美女,IT 数码
淘宝控,潮流搭配,潮流服饰,女装,服装搭配,购物,时尚
游戏动漫
搞笑幽默,应该是吧,有点色,见光死综合症,电影控,没有可以失去的,20 身体 40 的心,不刮胡须变匪徒,刮掉胡须就文艺
IT 数码,文艺
健康,旅游,星座命理,文艺,娱乐
投资理财,新闻资讯
旅游,美食
投资理财,美食,星座命理,旅游,娱乐
美食,旅游,娱乐,星座命理
文艺

IT 数码,随性,爬山,户外,旅游
旅游,娱乐,美食
文艺,健康
美食,四门语言,厨男,Music,o 型血
星座命理,美食
健康
萌宠,美食,体育
教育就业,健康,美食,文艺,旅游
旅游,健康,时尚
美食,健康,旅游,星座命理,时尚,娱乐
微博奇葩,星座命理,搞笑幽默,投资理财,教育就业,新闻资讯,IT 数码,游戏动漫,健康
游戏动漫,旅游,美食,文艺,娱乐,健康,星座命理
文艺,新闻资讯,健康,旅游,美食,娱乐
星座命理,旅游,时尚,文艺,娱乐
美女,汽车,体育,娱乐
情感生活,美女,汽车,时尚,体育,娱乐
美食,旅游,时尚,娱乐
时尚,娱乐,汽车,旅游,军事
时尚,星座命理,军事,娱乐,旅游
健康,娱乐,汽车,美女
IT 数码,汽车
文艺
体育,美女
时尚,IT 数码
美国生活,投资理财
时尚
涂鸦,动漫,学生,宅,巨蟹,强迫症,爱自由,双重性格,我就是我,狗狗
美食,健康
娱乐,时尚
星座命理,美食,娱乐
美食,新闻趣事,娱乐,汽车,旅游,健康
教育就业
美食
娱乐
旅游,美食,IT 数码,文艺,娱乐
IT 数码,教育就业,投资理财,旅游,娱乐,健康,文艺
旅游,健康,时尚
军事,IT 数码,游戏动漫
军事,旅游,美食,文艺,美食爱好者,搞怪,游戏动漫
体育,美女,健康,旅游,文艺
娱乐,新闻资讯,投资理财,IT 数码,美女
萌宠,动漫,绘画,狮子女,Vampire,人来疯,乐器
美食
囧,搞笑幽默,文艺,梦想,美食爱好者,小清新,善良,真实力,广州,销售

旅游
教育就业
汽车
教育就业
军事
许嵩,音乐发烧友,搞笑，趣图,90 后，幽默，娱乐,QQ,单机游戏,笑话,健康
汽车,投资理财,星座命理
旅游
IT 数码,时尚
娱乐,健康,旅游,新闻资讯,投资理财
时尚,美女,旅游,文艺,美食,汽车,娱乐
文艺
美食,游戏动漫,体育,娱乐
娱乐,投资理财,旅游,健康,时尚
性感
星座命理,肖旭,时尚,文艺,娱乐
美食,新闻资讯,旅游,健康,时尚,投资理财,娱乐
健康,美女,时尚,娱乐,文艺
爱音乐,小清新,k 歌,萝莉控,星座命理,美女
娱乐,游戏动漫
军事,美食,健康
娱乐
文艺
搞基,宅男,体育,猛男,肌肉,帅哥,同志,健康,投资理财,娱乐
游戏动漫
旅游,微博奇葩,视频音乐,新闻趣事,搞笑幽默
美食控,时尚潮流,睡懒觉,音乐达人,投资理财,美食,IT 数码,娱乐,体育
娱乐,旅游
健康,新闻资讯,汽车,时尚,旅游
投资理财
游戏动漫,健康,旅游,时尚
摇滚,爱电影,钢琴,街舞,咖啡,猫咪,音乐达人,自行车,个性,蓝色
IT 数码,时尚,旅游,文艺,健康
娱乐
汽车,美女,军事,旅游,娱乐
时尚
旅游
娱乐,健康,旅游,汽车,军事
IT 数码,时尚,投资理财,旅游,美食
爆发站,投资理财
旅游
体育资讯,视频音乐,新闻趣事,搞笑幽默,美女,体育,娱乐
娱乐,文艺,时尚,游戏动漫,星座命理
体育,美食

搞基, 宅男, 体育, 猛男, 肌肉, 帅哥, 同志, 教育就业, 文艺, 娱乐
时尚, 旅游
娱乐, 时尚, 美食
美女
时尚, 游戏动漫, 旅游, 娱乐
时尚, 美食, 娱乐, 旅游, 健康
文艺
美食, 游戏动漫
投资理财, 旅游, 美食
创业, 街拍, 学生一枚, 天秤, 偶喜欢玩, 美国, 时尚, 旅游, 美食, IT 数码
旅游, 健康, 美食
新闻资讯, 教育就业, 美食, 健康, 旅游
投资理财, 美女, 时尚, 娱乐
新闻资讯
视频音乐, 搞笑幽默, 武汉生活, 星座运势, 娱乐, 健康, 游戏动漫, 星座命理, 美食
娱乐
时尚
星座命理, 娱乐
微博奇葩, 搞笑幽默, 娱乐
军事, 娱乐
娱乐
娱乐
文艺, 时尚
健康, 时尚, 娱乐, 星座命理, 美食
萌宠, 娱乐, 文艺, 游戏动漫, 美食, 星座命理
娱乐
时尚, 军事, 美女, 娱乐
游戏动漫, 美女, IT 数码, 文艺, 娱乐
旅游, 汽车, 体育
汽车
体育, 军事, 旅游, 美食, 汽车
体育, 新闻资讯, 汽车, 投资理财, 健康
教育就业, 时尚, 美食, 文艺, 感性, 喜欢唱歌, 射手女, 学生一枚
游戏动漫, 时尚, 美女, 旅游, 美食
娱乐, 时尚
娱乐
健康
巨蟹, 睡觉, 学生
娱乐, 旅游, 投资理财, 游戏动漫, 汽车
视频音乐, 旅游, 名人明星, 美女, 新闻趣事, 文艺
时尚, 娱乐, 游戏动漫, 旅游, 美食
体育
交朋友, 网游, 娱乐, 旅游, 美女
娱乐

娱乐,游戏动漫
旅游
游戏动漫
IT 数码,娱乐,文艺,旅游,美食
文艺,新闻资讯
游戏动漫
微博奇葩
美女,游戏动漫,娱乐
健康,旅游,美食,娱乐
体育
时尚,美食,星座命理,文艺,娱乐
时尚,娱乐,星座命理,健康
旅游,文艺
娱乐
旅游,美食,娱乐
美食
星座命理,游戏动漫,体育
美女,娱乐
游戏动漫,星座命理
健康,新闻资讯,IT 数码,文艺,体育
娱乐
娱乐
健康,时尚,IT 数码,文艺,娱乐
名人明星,娱乐
安静,吃货一枚,爱旅行,天蝎女,娱乐,美食
游戏动漫,体育,美女,美食,IT 数码
美食,时尚,游戏动漫,娱乐,IT 数码
美食,军事,体育,娱乐,时尚,IT 数码
健康,时尚,旅游,星座命理,美食,文艺
教育就业,投资理财,娱乐,文艺,IT 数码
娱乐
IT 数码,军事,新闻资讯,游戏动漫,娱乐
美女,体育,美食,汽车,时尚
美食
健康,时尚,教育就业,美食,娱乐
美女,旅游,文艺
星座命理,旅游,时尚,美食,文艺,娱乐
IT 数码,美食,旅游,健康,文艺
时尚,文艺,健康,新闻资讯,星座命理
IT 数码,健康,投资理财,美女,时尚,旅游,文艺,美食
星座命理,文艺
新闻资讯,旅游,教育就业,娱乐
培训,自由,销售,健康,旅游
90,闷骚,摄影

美女,旅游,文艺,美食,娱乐
健康,娱乐
美图摄影,娱乐,体育,时尚,健康,美女
看电影,腐女,听音乐,网络,爱生活,阳光,美食,购物,语录,文艺
温州生活
体育
新闻资讯
搞笑幽默
汽车,娱乐,教育就业,美女,军事
健康,娱乐,星座命理
旅游,美食,星座命理,娱乐
美食,健康,旅游
旅游,时尚,健康
时尚,旅游
时尚
娱乐
娱乐
时尚,体育,健康,新闻资讯,美食
游戏动漫,体育
教育就业,健康,美女,旅游,军事
教育就业,投资理财,美食,健康,星座命理
DotA,游戏
热爱生活,Music,开朗,美女
健康,教育就业,投资理财
河北生活,投资理财
视频音乐,健康,文艺,体育
旅游,美食
上海生活,艺术,喜欢音乐,电影爱好者,健康,时尚
旅游,文艺,娱乐
新闻资讯
娱乐,美食,汽车,时尚
文艺,娱乐
美食,新闻资讯,游戏动漫,娱乐,体育
时尚
游戏动漫,投资理财,星座命理
旅游,游戏动漫,娱乐
美食,娱乐
汽车
旅游,娱乐,美食,汽车
教育就业,旅游,IT 数码
时尚,教育就业,旅游,星座命理,娱乐
文艺
美女,游戏动漫,旅游,文艺,美食
军事

搞笑幽默,娱乐
旅游,时尚,娱乐,文艺
健康,娱乐,时尚,旅游,美食
街舞,EXO,旅游,紫色,时尚
娱乐,文艺,美食,星座命理,时尚
汽车,投资理财,健康,新闻资讯,军事
旅游,星座命理,美食,娱乐
酒,吸血鬼,英雄联盟,惊情四百年,天蝎,军事,汽车,游戏动漫,体育
时尚
美女
军事,IT 数码,汽车
时尚,游戏动漫,健康,星座命理,娱乐
00 后,天秤座
旅游,文艺,IT 数码,体育,娱乐
文艺,医生,90 後,shopping,医学,天秤,旅游,娱乐
古典,旅游,文艺
星座命理
IT 数码
娱乐,文艺,旅游,星座命理,美食
婚纱摄影,新闻资讯,投资理财,旅游,时尚,美女
旅游,娱乐,文艺
星座命理,军事,IT 数码,文艺,投资理财
西安生活,汽车
美食
美食
微博奇葩,视频音乐,穿衣美容,美食,星座命理,时尚,娱乐
美食,汽车,文艺,娱乐
新闻资讯,旅游,文艺,娱乐
穿衣美容,健康,新闻资讯,时尚,教育就业,投资理财
娱乐,汽车,星座命理,文艺,体育
旅游,时尚,娱乐
微博奇葩,喜欢睡觉,爱听音乐
美食,投资理财,体育
eminem,maniac,rap,体育,文艺
时尚
喜欢唱歌,90 后但我不脑残,双鱼女,音樂,工作,文艺,开朗,爱美食,咖啡,时尚
新闻资讯,体育,健康,美女,教育就业
娱乐
旅游,教育就业,军事,文艺,游戏动漫
健康,娱乐,美女,汽车,投资理财,旅游
旅游
旅游,汽车,IT 数码
旅游,时尚,星座命理
健康,旅游,文艺,时尚,娱乐

新闻资讯,教育就业,美女,投资理财,娱乐,军事,旅游,健康
时尚达人,标志设计,设计师,IT 数码
投资理财,新闻资讯
旅游
美食,IT 数码,体育
旅游,健康,时尚,美食,文艺
新闻资讯
旅游,文艺,美食,美女,时尚,娱乐,体育
旅游,星座命理,音乐纯粹,爱 V 绝对
情感生活,投资理财,娱乐,星座命理,时尚
健康,体育,旅游
健康,时尚,娱乐
娱乐
跳舞,旅游
星座命理,健康
白羊座,游戏动漫
健康,汽车,旅游,美食
听歌,电影,搞笑幽默,音乐,星座命理
游戏动漫,时尚,军事,体育,娱乐
旅游,新闻资讯
时尚,美食,旅游
教育就业,投资理财,旅游,文艺
娱乐,时尚,投资理财,美食,旅游,文艺
IT 数码,军事,文艺,汽车
汽车,旅游,美食,IT 数码,娱乐
体育,IT 数码,游戏动漫
天蝎座,哈哈,大学生,吃货,休闲,神经大条,服装,情绪化,搞笑幽默
星座命理,博客,娱乐
新闻资讯,投资理财,IT 数码,体育
投资理财,时尚,我就是我
萌宠,搞笑幽默,星座命理,美食,娱乐
健康
上海生活
美食,搞笑幽默,美容服饰,娱乐
新闻资讯
娱乐
时尚
旅游,美食,娱乐,时尚
星座命理,微博奇葩,新闻趣事
投资理财
吃货,心理,散步,双鱼座,少女时代
美食,汽车
美食,娱乐,时尚
新闻趣事,穿衣美容,教育就业

娱乐,新闻资讯,时尚
健康,体育,娱乐
旅游,湖北生活,新闻趣事,投资理财,军事
游戏动漫
娱乐,IT 数码,健康,时尚,星座命理,教育就业
上海生活,新闻资讯
健康,体育,教育就业
时尚,旅游
游戏动漫,IT 数码,美食,汽车,文艺,体育
夜猫子,我就是我,可爱,双重性格,爱自由,游戏动漫,旅游,美食,娱乐
美食
健康,新闻资讯,体育
时尚,娱乐
游戏动漫,娱乐,文艺,星座命理,美食
校园生活
军事,汽车,娱乐,教育就业,星座命理,美食,新闻资讯
射手女,小清新,微博控,听音乐,射手座,吃货一枚,狗狗,古典艺术,手工
旅游
星座命理
娱乐,投资理财
美食,娱乐
星座命理,美食,时尚,娱乐
IT 数码
军事,体育,IT 数码
IT 数码
美食,军事,IT 数码,文艺,体育
文艺,星座命理
娱乐
游戏动漫,星座命理
美女
星座命理,娱乐,美食,游戏动漫
美食,美图摄影,美国生活,菲迷一枚,老炫迷
旅游
旅游,IT 数码,军事,健康,体育
娱乐,游戏动漫,汽车
美食,健康,美女,时尚,文艺
学建筑的小苦逼,林先森的 JM 一枚,白羊女
美食
娱乐
美食,投资理财,教育就业,新闻资讯,健康
健康,星座命理,旅游,文艺
新闻趣事,美女,IT 数码,搞笑幽默,八卦杂谈,dota,健康,教育就业
时尚,美女,娱乐,IT 数码
汽车,旅游,娱乐

来吧，一起疯!!
星座命理,美食,IT 数码,文艺,娱乐
IT 数码
游戏动漫,娱乐
美女,美食,体育,汽车,旅游
美食,文艺,旅游
爱小肥皂,爱旅行,爱睡觉,护肤,吃货一枚
微博奇葩,音樂,处女座
时尚,娱乐,文艺
新闻资讯,汽车
时尚
心理学爱好者,股票投资,股票分析,炒股票,投资理财,娱乐,美女,游戏动漫
文艺,教育就业,游戏动漫,旅游,美食
新闻资讯,健康,旅游,星座命理,美食
娱乐,星座命理
美食爱好者,电影爱好者,B 型血,美食,瘦身,减肥,美体,美容,护肤,健康
时尚,IT 数码,美食,体育,娱乐
街拍,爱吃,旅游爱好者,开心,理财,美食
名人明星
旅游
看书,幽默,医学,心情,手工
名人明星,懒,爱笑,宅,韩国,音乐控,娱乐
美食,星座命理,旅遊,時尚,娛樂
旅游,美食,娱乐
时尚,投资理财,汽车,美女,娱乐
娱乐
美女,穿衣美容
娱乐
时尚,旅游,星座命理,美食,娱乐
懒,玩,快乐,爱笑,星座命理,娱乐
投资理财,娱乐,汽车,游戏动漫,IT 数码
微博奇葩
旅游
旅游,文艺
星座命理,健康,美女,体育
美女,投资理财,体育,娱乐,教育就业,时尚
文艺,星座命理,健康,时尚
星座命理,遊戲動漫,教育就業,IT 數碼,娛樂
旅游
穿衣美容,微博奇葩,萌宠,旅游,军事
体育
文艺,IT 数码,星座命理,游戏动漫
投资理财
旅游,健康,美食,星座命理,投资理财

小资,执着,电影控,爱旅游
旅游
文艺,星座命理,美食,美食,健康,娱乐
旅游,新闻资讯,时尚,文艺
视频音乐,汽车,投资理财,健康,IT 数码,娱乐
星座命理,旅游,美食,文艺,娱乐
穿衣美容,时尚,文艺,美食,娱乐,投资理财
文艺
文艺,娱乐,旅游
汽车,时尚,体育,娱乐
娱乐
美食,娱乐
星座命理,娱乐,时尚
游戏动漫,旅游,美食,文艺,娱乐
美女,军事
美食,健康,文艺,新闻资讯,时尚,投资理财,美食
80 后宅女,天蝎女,美食
名人明星,新闻资讯,投资理财,IT 数码
健康,矛盾体,随性,星座命理,睡觉睡到自然醒,懒人
音樂,淡定,英格兰,伪文艺,小愤青
吸血鬼,爱电影,爱旅游,k 歌,学生一枚
体育
游戏动漫,文艺,娱乐
投资理财,娱乐,文艺,IT 数码,新闻资讯,健康
小女人,天秤,星座命理,美食控,拍照,服饰,娱乐,睡懒觉,文字控,唱 K
旅游,投资理财,文艺,军事,美女
健康,IT 数码,新闻资讯,旅游,美食
旅游,新闻资讯,娱乐,投资理财,健康,教育就业
军事,体育,投资理财,汽车,旅游,文艺,IT 数码
美食,健康,时尚,体育,IT 数码
时尚,游戏动漫,美食,娱乐,文艺
美食
星座命理
时尚
请熟人不要关注,我依然是,你们心中那个,温柔典雅气质佳,贤淑端庄书香气,的小家碧玉
星座命理,游戏动漫
微博奇葩,萌宠,穿衣美容,星座命理,美女
旅游,美食,娱乐
游戏动漫,时尚,美女,旅游,美食,IT 数码
投资理财,旅游,美食,军事
娱乐,游戏动漫
时尚,健康,旅游,美食,娱乐
时尚,汽车,美食,文艺,娱乐,活泼,快乐,美食控
IT 数码

处女座,旅游美食,护士,活泼开朗,唱 k
美食
旅游,投资理财,娱乐,游戏动漫,星座命理
美食,娱乐
星座命理,健康,时尚
星座运势,健康
新闻资讯,教育就业,军事,体育
电视台,驴友,旅游,摄影家协会,采风,摄影,中国摄影网
旅游,健康,新闻资讯,娱乐,时尚
投资理财
微博奇葩,星座命理,穿衣美容,美食,旅游,健康,时尚,娱乐
美女
美食,健康,旅游,军事,美女,汽车,IT 数码,体育,娱乐
时尚
文艺
IT 数码,旅游,汽车,投资理财
体育
健康,新闻资讯,娱乐,美女
娱乐,旅游,时尚,星座命理
健康,新闻资讯,投资理财,美食,体育
口硬心软,爱植物,爱唱歌,欧美歌曲控,吃货,我爱艾薇儿
旅游,时尚
星座命理
银魂,文艺,游戏动漫,动漫,2B 青年,腐,天蝎女
投资理财
外貌协会,运动,节食,丰胸,塑形,减肥狂,好身材
娱乐
名人明星,视频音乐,穿衣美容,时尚,IT 数码,文艺
旅游,星座命理,美食,时尚,娱乐
新闻资讯
时尚,美食,旅游
体育,美食,娱乐,美女,游戏动漫
旅游,文艺
搞基,宅男,体育,猛男,肌肉,帅哥,同志,新闻资讯,时尚,投资理财
汽车
时尚,旅游,健康,新闻资讯,IT 数码,文艺
投资理财
娱乐,教育就业,投资理财,新闻资讯,健康
美食
健康,娱乐,教育就业,文艺,美食
美食,新闻资讯,健康,美食,IT 数码,文艺
IT 数码,军事,汽车,体育,娱乐
星座命理
娱乐

时尚,旅游,IT 数码
汽车,娱乐,美女,健康,IT 数码
娱乐,投资理财,健康,旅游,美食,时尚
投资理财
时尚,美女,汽车,旅游,娱乐,投资理财
IT 数码,美食,营销
汽车
自由职业,好书,疯狂,沉默,发呆,游泳,看电影,爱音乐,旅游
高尔夫球爱好者
美食
美食,体育
游戏动漫,星座命理,IT 数码,体育,娱乐
文艺,IT 数码
上海生活,娱乐
娱乐,汽车,体育
旅游,体育,游戏动漫,文艺
美食,娱乐,时尚,健康
投资理财,新闻资讯
娱乐
美食
旅游,娱乐,星座命理,文艺,呃呃呃
体育
美食
开朗,IT 数码,星座命理,旅游,娱乐
娱乐
体育
旅游,微博奇葩,穿衣美容,美食,视频音乐
张爱马笛,翟傲邦,魔兽世界,起床困难户,内敛,低调,各种宅,日剧,山田孝之,双重性格
教育就业,美女,新闻资讯,军事,体育,游戏动漫
旅游
時尚,體育,軍事,遊戲動漫,文藝,旅遊,新聞資訊
其他生活
文艺
美食,投资理财,旅游,健康,星座命理
旅游,IT 数码,时尚,星座命理,娱乐
美女
投资理财,健康,旅游,星座命理,美食
娱乐,吃货一枚,减肥,爱唱歌,懒,快乐,胡思乱想,感性,强迫症
新闻资讯,娱乐,文艺,体育
健康
时尚
IT 数码,军事,美食,美女,汽车
NBA,幽默,篮球,汽车,体育
汽车,时尚,游戏动漫,军事,IT 数码

时尚,星座命理,文艺
搞笑幽默
健康,旅游
美食,文艺
星座运势,时尚,健康
投资理财,时尚,旅游,美食,娱乐
军事
娱乐
美女,时尚
美食,时尚,健康,娱乐,旅游
教育就业
健康
旅游,健康,投资理财,新闻资讯,美食
教育就业,文艺,游戏动漫,时尚,投资理财
军事,汽车
大爱肚皮舞,电影控,美食,文艺,旅游
自然
美女,旅游,名人明星,萌宠,新闻趣事,游戏动漫,体育
新闻资讯,星座命理,美食,文艺,健康
文艺
新闻资讯,时尚,健康,美食,旅游
美女,投资理财,文艺,新闻资讯,旅游,军事,汽车
新闻资讯,投资理财,美女,星座命理
时尚,汽车,美女,旅游,娱乐
信用卡,保障,理财,自然
星座命理,IT 数码,美食,娱乐,时尚
旅游,美食
健康,体育,军事,游戏动漫,文艺
美女,娱乐
星座命理,时尚,旅游,美食,娱乐
健康
新闻资讯,健康,文艺,娱乐,星座命理
健康,星座命理,文艺,教育就业,娱乐
娱乐,游戏动漫,IT 数码
美女,时尚,美食,文艺,娱乐
健康,文艺,旅游,娱乐
新闻资讯,美食,星座命理,娱乐
娱乐,美食,星座命理,旅游
睡懒觉,人生,旅游
娱乐,美食
90 后非脑残,爱自由,韩剧,偏执狂,射手,时尚,娱乐,旅游,美食
微博奇葩,美食
健康,军事,时尚,汽车,娱乐
旅游

投资理财,IT 数码,美食,游戏动漫,教育就业
教育就业,旅游
教育就业,美女
动漫控
星座命理,投资理财,汽车,美女,时尚
旅游,健康,体育,汽车
美女,汽车,娱乐,军事,旅游
美食
游戏动漫,IT 数码,体育
旅游
军事,文艺,娱乐
时尚,旅游,美食,文艺,娱乐
时尚,汽车,体育,文艺,娱乐
校园生活,处女座,音乐控,我就是我,o 型血,听歌,90 后,文艺,娱乐
教育就业
时尚,汽车,旅游,IT 数码,娱乐
育儿百科,搞笑幽默,旅游,美食,西安生活
星座运势,八卦杂谈,新闻趣事,美食,军事,旅游,健康,娱乐
时尚,娱乐
汽车
旅游
斯诺克爱好者,90 后
时尚,旅游,星座命理,美食,文艺
时尚,美女,旅游,军事,IT 数码
美女
娱乐,新闻资讯,军事,汽车,时尚,体育
冤家宜結不宜解,咬人,打架,美食,旅游
时尚,娱乐,美食,旅游
文艺
新闻资讯,美食,文艺,娱乐,时尚
体育
健康,旅游,星座命理,美食,IT 数码
新闻资讯
投资理财,新闻资讯,旅游,美食,文艺
军事
汽车,旅游,投资理财
美女,新闻趣事,搞笑幽默
投资理财,健康
星座命理,健康,教育就业,美食,娱乐
水瓶座,生化危机,体育,游戏动漫
美食,体育,汽车
娱乐
娱乐,星座命理,时尚,美食,游戏动漫
美女,视频音乐

汽车,星座命理,娱乐,时尚,美食,健康
美食,游戏动漫,旅游
体育,军事
微博奇葩,体育
广西生活,健康
敏感略带神经质,腐宅双修,吉他,钢琴,郭德纲,卖萌,日本文学,独立音乐控,唱K,外貌协会
天秤,90后非脑残,柯南,学生一族,小说迷
健康,新闻资讯,投资理财,军事,IT数码
健康
美食,旅游,美女,投资理财,娱乐
汽车,健康,体育,军事
健康,旅游,星座命理,娱乐
健康,军事,汽车,新闻资讯,投资理财
星座命理,时尚
娱乐
文艺,娱乐
IT数码
穿衣美容
美容服饰,深圳生活,健康,新闻资讯,美食,旅游
旅游,时尚
星座命理,旅游,娱乐,时尚,游戏动漫
投资理财,娱乐
健康,美食,旅游,体育
白羊座,幸福,快乐,活泼,喜欢听歌,娱乐
健康,娱乐,游戏动漫,美食,军事
旅遊,美女
军事
IT数码,时尚,旅游,美食,娱乐
时尚,听音乐,双鱼女,时装
旅游,健康,投资理财,美食
投资理财,健康,新闻资讯,教育就业,体育
美食,旅游
其他生活,邪恶光环
旅游,游戏动漫
娱乐,教育就业,旅游,美食,军事,体育
文艺
美女,游戏动漫,文艺
搞笑幽默,星座命理
娱乐,文艺
名人明星,萌宠,搞笑幽默,视频音乐
时尚,文艺,娱乐,星座命理,美食,IT数码
美食
娱乐
时尚,健康

娱乐,IT 数码
汽车,时尚,旅游,游戏动漫
萌宠,搞笑幽默,微博奇葩,视频音乐,美女,游戏动漫,体育,IT 数码
文艺
美食,星座命理,时尚,文艺,娱乐
旅游,美食
新闻资讯,健康,星座命理,时尚,娱乐,旅游,美食
投资理财,新闻资讯,娱乐,汽车,体育
文艺,娱乐,美食,时尚
旅游,体育,娱乐
时尚,教育就业,投资理财,新闻资讯,健康
文艺,新闻资讯
美食,萌宠,微博奇葩,搞笑幽默,军事,星座命理,体育
视频音乐
郑州生活
教育就业,旅游,娱乐,星座命理,健康
体育,健康,投资理财,旅游,娱乐,IT 数码,阳光,爱自由,羽毛球
校园生活,搞笑幽默,体育资讯,教育就业,游戏动漫,IT 数码,体育,娱乐
旅游
美食,投资理财,文艺,旅游,美食,游戏动漫
娱乐,时尚,ELF,DOTA,WOW,篮球爱好者,喜欢音乐,愤青,汽车,体育
星座命理,娱乐
萌宠,宅,apple,摩羯座,ACG,85 后
跑步,时尚,投资理财,新闻资讯,娱乐,健康,旅游,军事,IT 数码,文艺
宠物图集,健康
游戏动漫,军事,娱乐
教育就业
文艺
投资理财
IT 数码,新闻资讯,旅游
喜欢旅游,处女座,安静,完美主义,感性,喜欢听歌,健康
时尚
娱乐,美食
时尚,美食,娱乐,投资理财,旅游
游戏动漫
旅游,美食,文艺,娱乐
视频音乐,新闻趣事,旅游,美食,星座命理
健康
潮人,服饰
旅游,有机蔬菜,IT 数码,投资理财,娱乐,健康
投资理财,美食,健康,文艺,娱乐
旅游
星座命理,文艺
教育就业,星座命理,美食,旅游,IT 数码,文艺,体育

投资理财,汽车,旅游
体育
IT 数码,时尚,汽车,体育,娱乐
IT 数码,军事,投资理财,美女,娱乐
娱乐
教育就业
或许是玻璃心,半吊子 COSer,或许有高能,二次元生物,WOWer,渣基三
IT 数码,汽车,旅游,军事,娱乐,体育
娱乐
时尚,汽车,美食,文艺,IT 数码
美女,搞笑幽默,遂宁生活,天平座,IT 数码,汽车,娱乐
校园生活,游戏动漫,dota,林俊杰,重口味,文字控,宅女加腐女
美女,时尚,旅游
时尚,汽车,教育就业,投资理财,娱乐
时尚,美女,美食,新闻资讯,旅游
吃喝玩乐
体育,旅游
星座命理
IT 数码
广东生活,听歌,90 后,娱乐,爱电影,潮,炫舞
游戏动漫,美女,旅游,美食,体育
新闻资讯,健康
文艺
美食,旅游,美女,体育,汽车
名人明星,娱乐
IT 数码
娱乐
娱乐
旅游,游戏动漫
美食,时尚,娱乐,游戏动漫,旅游
旅游,星座命理,军事,汽车,健康
美食
萌宠,新闻资讯,教育就业,游戏动漫
星座命理,SM 共同体,情感性精神病,暴躁狂,双重人格,tattoo,Bisexual
星座命理,旅游,健康,时尚,美食
名人明星,美图摄影,读书分享,情感生活,搞笑幽默,娱乐,文艺
教育就业,娱乐,汽车,旅游,健康,时尚
海贼王,90,执着,冷笑话,爱玩,聊天,电脑,感性,军事,IT 数码
O 型血,汽车,旅游,美食,IT 数码,娱乐
教育就业,游戏动漫
文艺
美食,旅游,文艺,美女
旅游
旅游

文艺,娱乐,时尚,旅游,美食
美食
视频音乐,穿衣美容
游戏动漫,军事,娱乐
教育就业,星座命理,文艺
星座命理,娱乐
文艺,投资理财,健康,IT 数码,汽车,美女
IT 数码,娱乐,体育
搞笑幽默,旅游,娱乐
美女,汽车,军事
健康,汽车,旅游,美食,体育
军事,IT 数码,汽车,美女,时尚
旅游,美食,游戏动漫,文艺
美食
旅游,美食,IT 数码
投资理财,星座命理,军事
星座命理,游戏动漫
新闻趣事,新闻资讯,汽车,IT 数码,体育
宠物图集,投资理财
旅游
旅游,健康,美食,文艺,教育就业
搞笑幽默,时尚
旅游
学生一族,星座命理
美食,娱乐,汽车,时尚,美女
时尚,汽车,教育就业,美女,体育
娱乐
教育就业,投资理财,健康,军事,IT 数码,体育
旅游,汽车
搞基,宅男,体育,猛男,肌肉,帅哥,同志,健康,美食,IT 数码
时尚,IT 数码,娱乐
健康
健康,旅游,星座命理,游戏动漫,体育
汽车,娱乐,健康,旅游,IT 数码
军事,旅游,IT 数码,新闻资讯,美女,教育就业,投资理财,体育
健康,教育就业,星座命理
连接服务器失败,咖啡,90 後,爱电影,好性格,多愁善感,文艺,娱乐
娱乐,星座命理
星座命理,旅游,美食
新闻资讯,投资理财
大连,居丽,恩妍,恩敏,智妍,孝敏,T,咸恩静,Tiara,恩静
旅游,新闻资讯,健康,娱乐,美食
IT 数码,游戏动漫,娱乐
美食

军事,健康,美食,文艺,娱乐,投资理财
名人明星,淄博生活,搞笑幽默,校园生活,美图摄影
八卦杂谈
美食,IT 数码,旅游,星座命理,娱乐,文艺,时尚
美女,投资理财,星座命理,旅游,娱乐
旅游,校园生活,八卦杂谈,美食,搞笑幽默,健康,星座命理
旅游,汽车,时尚,投资理财
视频音乐
旅游,哈尔滨生活,八卦杂谈
汽车,旅游,军事,IT 数码,娱乐
胆小鬼,周德东,恐怖,文艺
旅游
娱乐
星座命理
健康,旅游,新闻资讯,时尚,娱乐
时尚
美图摄影,情感生活,星座运势,美食
宠物图集,文艺
文艺,美女,娱乐
健康,投资理财,汽车
旅游,文艺,美食,娱乐,教育就业
IT 数码
美女,体育
新闻资讯,游戏动漫,教育就业,健康,旅游,美食
时尚,美食,美女,游戏动漫
旅游,星座命理,美食
猫控,萌宠控,追星,游戏动漫,美食,娱乐
80 後,男人
小夜。,包子。,IT 数码
健康,旅游,美食,时尚,娱乐
星座命理,旅游
旅游,娱乐,IT 数码,汽车
萌宠,穿衣美容,美食,游戏动漫
军事
旅游,美食,娱乐,投资理财
美图摄影,新闻趣事
旅游,美女,新闻资讯
17 歲
打篮球,学生,旅游,ps3,游戏,漫画
体育
星座命理,汽车,美女,IT 数码,投资理财
投资理财,健康,汽车,军事
文艺
游戏动漫

吼吼
星座命理,文艺,美女,娱乐,体育,美食
K 歌,汽车,娱乐,美食,游戏动漫
文艺
星座命理
新闻资讯,健康,美女,时尚,娱乐
健康,汽车,娱乐,体育,IT 数码
健康,汽车,美食,娱乐,文艺
汽车
娱乐
美食
旅游,体育
星座命理
娱乐,旅游,IT 数码,时尚,教育就业
娱乐,交友等等,网络技术,赚钱
美女,星座命理,娱乐
游戏动漫,IT 数码,娱乐
DNF
娱乐,旅游,时尚
星座命理,娱乐,旅游,IT 数码,文艺
美女,娱乐
90 后,乐动达人,美食,游戏动漫,体育,娱乐
旅游,娱乐,美食
娱乐
视频音乐,校园生活,美图摄影,文艺,时尚,游戏动漫,旅游,娱乐
视频音乐,旅游,娱乐
名人明星,傻,天蝎女,双重性格,矛盾体,懒,闷骚,摄影,睡觉,自由
旅游,健康,时尚
阳光,可爱,爱生活,健康,美食,文艺,娱乐
健康,游戏动漫,美食
美食,汽车,时尚,投资理财,娱乐
旅游,IT 数码,体育
星座命理,教育就业,美食,健康,文艺,娱乐
汽车,军事,投资理财,星座命理,体育
星座命理,汽车,娱乐,军事,新闻资讯
吃货
穿衣美容,旅游,新闻趣事,搞笑幽默
星座命理,美食,娱乐,时尚
美女
美女,四川生活
新闻趣事,健康
穿衣美容,健康
IT 数码
我想要的自由。,娱乐

中国好声音,演唱会,庾澄庆,吉克隽逸,关喆,歌浴森
星座命理,旅游
游戏动漫
娱乐
健康,旅游,军事,文艺,娱乐
教育就业,旅游,文艺
娱乐
游戏动漫
旅游
美女
美食,娱乐,美女,旅游,体育
安静,宅女加腐女,散步
IT 数码
星座命理,旅游,游戏动漫,汽车,投资理财
新闻资讯,时尚,汽车,娱乐,体育
游戏动漫,汽车,IT 数码,军事
投资理财
IT 数码
健康,美女,时尚,美食,娱乐
体育
美女,文艺
时尚,娱乐
新闻资讯,星座命理,美食
IT 数码,投资理财,新闻资讯,旅游
美食,娱乐
汽车,美女,IT 数码,娱乐
爱,篮球,睡觉,学生,唱歌,IT 数码,汽车,体育
美食
客运专线,大机,鹰潭工务机械段,宿营车,捣固车,铁路养护维修,南昌铁路局,大型养路机械,铁路人
星座命理,手机看小说,旅游
游戏动漫,IT 数码,学生族
时尚
汽车,旅游,军事,时尚,星座命理,娱乐
教育就业,投资理财,IT 数码,文艺
美食
美食,汽车,文艺,健康,旅游
旅游
健康,新闻资讯,美食
星座命理,旅游,健康,美食,娱乐
健康,体育,娱乐
新闻资讯,美女,游戏动漫
健康,体育,娱乐
时尚,投资理财,文艺,星座命理,娱乐

多愁善感,喜欢唱歌,90后,水瓶座,视频音乐,健康,娱乐,文艺
军事,IT数码,文艺
健康,体育,娱乐
美女,汽车,旅游,娱乐
娱乐,IT数码
体育
旅游,体育,时尚,娱乐,星座命理
厦门生活,校园生活,美图摄影,八卦杂谈,美女,投资理财,教育就业,文艺,学生
穿衣美容
时尚,娱乐
美食,健康,旅游,新闻资讯,教育就业,美食
大学生,汽车,军事,IT数码,新闻资讯,旅游,投资理财
娱乐
时尚,游戏动漫,健康,星座命理,美食,新闻资讯
文艺,时尚,IT数码,投资理财,教育就业
投资理财,教育就业,美女,娱乐,军事,IT数码
教育就业
搞笑幽默,新闻趣事,萌宠,名人明星,星座命理
美女
时尚,美食,星座命理,旅游,娱乐
娱乐,IT数码,军事,新闻资讯,星座命理
文艺,游戏动漫
娱乐,时尚,美食
宅,学生,美食,旅游
美食,备孕,好吃,韩剧
美食,教育就业,体育,健康,旅游
文艺
娱乐,健康,美食
娱乐,教育就业,投资理财,游戏动漫,美女,IT数码
体育,汽车,时尚,IT数码
美女,妖精的尾巴,英雄联盟
穿衣美容,娱乐
旅游,穿衣美容,萌宠,时尚,美食
旅游
投资理财,汽车,时尚,军事,美女
旅游
旅游,体育,娱乐
学生,吃货
旅游,美食,穿衣美容,新闻趣事,上海生活
健康
健康,旅游,美食,军事,IT数码
美食
健康,时尚,娱乐,美食,文艺
旅游,微博奇葩,视频音乐,搞笑幽默,中山生活,IT数码,体育

旅游,娱乐,美食,美女,时尚
足球,射手座,旅游,萌宠,美食
女夭,洛爱,赛米,刀剑,魔卡
教育就业,健康,旅游,时尚,美女
IT 数码,新闻资讯
微,微信,微信营,微信营销,微电商,微系统,微电影
时尚,美食,文艺
文艺,娱乐
军事,娱乐,美女,投资理财,时尚,旅游,IT 数码,汽车,美食,文艺
健康,游戏动漫
星座命理
美女,游戏动漫,时尚
美女,美食,旅游,汽车,IT 数码,游戏动漫
新闻资讯,美食,文艺,体育,娱乐
宅男,愤青,爱唱歌,文艺,时尚,娱乐
朴灿烈,吴亦凡,exo
娱乐
美女
新闻资讯,教育就业,军事
时尚,美食,文艺
汽车,时尚,旅游,美食,娱乐
游戏动漫,美食,文艺
旅游
美食
娱乐
游戏动漫,娱乐
娱乐,体育,军事,美食,旅游
时尚,旅游,美食,健康,IT 数码
健康
娱乐,IT 数码,星座命理
新闻资讯,游戏动漫,IT 数码,旅游,美食,文艺
美女
投资理财
健康,游戏动漫,时尚,旅游,美食
微博奇葩,穿衣美容,视频音乐,搞笑幽默
Music,NBA,市场营销
娱乐,文艺,军事,投资理财
教育就业,汽车,游戏动漫,健康,军事
神哲神学,天主教,爱主爱人,灵修,神修,内修,基督徒
80 后,设计任务雇佣,第一设计网,设计网,设计生活,创意设计,创意点子,广告,艺术,文艺
音乐控,美食控,蓝色,小说迷,妖精,美食,星座命理,美食,文艺,娱乐
新闻资讯,娱乐,游戏动漫
喜欢音乐,腹黑女,大提琴控,爱音乐,fx,时尚技术宅,自由,听歌,自然,justinbieber
娱乐,星座命理

游戏动漫
健康
美食,旅遊,名人明星
健康,游戏动漫,文艺,IT 数码,娱乐
时尚,星座命理,娱乐
美食
娱乐,汽车,游戏动漫,IT 数码,体育
文艺
星座命理,时尚,美食,文艺,娱乐
IT 数码,文艺,体育
包头生活,喜欢车,朋友,IT 数码,军事,投资理财,汽车,新闻资讯
旅游
旅游,投资理财,美女
游戏动漫,军事,娱乐
茶叶,军事
教育就业
娱乐,美食,旅游
名人明星,新闻趣事,微博奇葩,江苏生活,搞笑幽默,新闻资讯,时尚,IT 数码,体育,娱乐
娱乐,安静的下午,健康,教育就业,投资理财
旅游,美女
星座命理,游戏动漫,文艺,美食
时尚,游戏动漫
理财,新闻资讯,时尚,旅游
美女,娱乐
汽车,时尚,旅游,娱乐,美食
旅游
健康,旅游,体育,时尚,娱乐,美食
军事
娱乐,军事,健康,新闻资讯,美女,时尚
射手座,旅游,健康,时尚,教育就业,文艺
军事,美食,新闻资讯,健康,美女,娱乐,IT 数码
体育
教育就业,时尚,健康,星座命理,娱乐
星座命理,旅游,美食,娱乐
美女,体育
美食
时尚,汽车,美食
汽车,教育就业,军事
星座命理,文艺,娱乐
新闻资讯,旅游,娱乐,文艺,健康
体育,娱乐
游戏动漫
茶
旅游,军事,文艺

时尚,投资理财,旅游
星座命理
星座命理,时尚,新闻资讯,娱乐
投资理财,汽车,新闻资讯,健康,IT 数码
美食,文艺,娱乐,时尚,游戏动漫
健康,教育就业,文艺,体育,美女
投资理财,旅游,新闻资讯,健康,文艺
IT 数码,文艺,娱乐
IT 数码
投资理财,旅游,美食,文艺,娱乐
汽车,游戏动漫,美女,体育,娱乐
投资理财,旅游,文艺
小说,美食爱好者,柯南
健康,时尚,美女,文艺,娱乐
IT 数码,军事,新闻资讯,美女,汽车
时尚,教育就业,投资理财,娱乐,新闻资讯
时尚
教育就业
体育
健康
旅游
穿衣美容,投资理财
美食,旅游
射手座 B 型血,时尚,美食
旅游
新闻趣事,八卦杂谈,美女,视频音乐,搞笑幽默,娱乐
健康,美食,体育
投资理财,星座命理,军事
投资理财,美女,汽车,军事,娱乐,体育,游戏动漫,IT 数码
娱乐,美食
上网,学生,宅
旅游,美食,文艺,星座命理,时尚,娱乐
IT 数码
教育就业,旅游,美食
游戏动漫
美食,健康,时尚,投资理财,文艺
时尚,文艺,星座命理,娱乐
时尚,汽车,娱乐,美食,星座命理
汽车
时尚,娱乐
星座命理,娱乐,美女,投资理财,体育
汽车,美食
文艺,美食,星座命理,旅游,娱乐,健康,美食
时尚,汽车,投资理财,IT 数码,娱乐

搞笑幽默
爱电影,周杰伦,欧美,音乐爱好者,傻,音乐发烧友,旅游,美食,文艺,娱乐
教育就业,文艺,旅游
星座命理,娱乐,时尚,旅游
视频音乐
旅游,娱乐,时尚,健康,美食
美女,娱乐
新闻趣事,朝阳生活,军事
健康
健康,旅游,美食,娱乐,投资理财
微博奇葩,星座命理,穿衣美容,搞笑幽默,名人明星
美食,名人明星,其他生活,新闻趣事,微博奇葩
游戏动漫,星座命理,美食,文艺
狮子座,85后
美女,美食,IT数码,娱乐,体育
IT数码,美女,时尚,娱乐,体育
文艺
旅游
搞笑幽默,健康
健康,体育
星座命理,新闻资讯,美食,文艺,娱乐
汽车,新闻资讯,娱乐,军事
健康,教育就业,旅游,文艺,娱乐
文艺,游戏动漫
宠物,音乐控,笑话,教育,爱睡觉,帅哥,爱生活,可爱,任性,巧克力
体育
美容服饰,美图摄影,星座运势,新闻趣事,美食,美女,娱乐
微博奇葩,IT数码
微博奇葩,视频音乐,美食,旅游,美女
新闻资讯,旅游,文艺,娱乐
体育,娱乐,旅游
穿衣美容,名人明星,美食,视频音乐,星座命理,天平座,韩国,张根硕的鳗鱼,
文艺,体育,娱乐
搞笑幽默,游戏动漫
健康
星座命理,军事
娱乐
IT数码,军事,体育,娱乐,星座命理,游戏动漫
娱乐,美食,美女,新闻资讯
搞笑幽默,IT数码,爱摄影
游戏动漫,军事,娱乐,美食,IT数码
旅游,游戏动漫,军事,IT数码,娱乐,k歌，美食，旅游
时尚,汽车,健康,美食,投资理财
娱乐

IT 数码,美女,体育
健康,时尚,旅游,娱乐,吃货一枚
娱乐,旅游,美食,时尚
健康,新闻资讯,教育就业,美食,娱乐
做梦,服装设计,爱自由,爱电影,奋斗,乐观
游戏动漫
搞笑幽默,时尚,娱乐
渝都互联,西南信息港,商务网
时尚,美女,美食,健康,旅游
星座命理,文艺,游戏动漫,健康,美食
搞笑幽默,军事,IT 数码,教育就业,体育,娱乐
娱乐,汽车,健康,美女
格子控
广东生活,新闻趣事,微博奇葩,穿衣美容
体育
美食
旅游,美食
文艺,动漫控,吉他,小清新,苏打绿
新闻资讯,娱乐,军事,美女,文艺
投资理财,dj,旅游
旅游,IT 数码
旅游,文艺,教育就业,健康,美食
朴素妍,李雅琳,居丽,宝蓝,朴孝敏,含恩静,朴智妍
文艺
旅游,娱乐,体育
汽车,星座命理,O 型血,林峯,Music
旅游,星座命理,美食,新闻资讯,文艺,娱乐
美术,双鱼女,星座命理,文艺
星座命理,娱乐
娱乐
看看新闻
旅遊,美女,體育
文艺,星座命理,美食,旅游,游戏动漫,娱乐
搞笑幽默
婚纱,影视,娱乐,旅游
wow,文字控,煮妇,热爱唱歌我,嘎嘎能乐,懒
旅游,IT 数码,文艺,娱乐,时尚
IT 数码,美女,娱乐,体育
视频音乐,娱乐,汽车,星座命理
体育
游戏动漫,投资理财,教育就业,汽车,IT 数码,军事
美食
游戏动漫
时尚,文艺,美食

美食,星座命理,军事,IT 数码,娱乐
遊戲動漫,文藝,投資理財,旅遊,美食
音乐迷,青春,个性,历史,囧,快乐,music,梦想,情感,旅游
美女
投资理财,IT 数码,文艺,娱乐,汽车
旅游
旅游
娱乐
美女,江苏生活,军事,文艺,IT 数码,体育,娱乐
时尚,体育,文艺,娱乐
旅游,娱乐,时尚,星座命理,美食
时尚
体育,娱乐
游戏动漫,教育就业,汽车
八卦杂谈,车世界,体育资讯
美食,旅游
娱乐,汽车,游戏动漫,军事,时尚
美食
文艺,旅游,新闻资讯,星座命理,美食
新闻趣事
社会,微博,吃喝玩乐,文艺,体育,娱乐,星座命理,美食,军事
体育
旅游,IT 数码,汽车
微博,电视剧,喜欢唱歌,自信,互联网,文艺,娱乐,新闻资讯,美女,体育
社会,美女,语录,屌丝,文艺,体育,娱乐,投资理财,旅游,星座命理
美女,游戏动漫,军事,IT 数码,时尚,文艺
美女
游戏动漫
星座命理
新闻资讯,文艺,旅游,美食,时尚
旅游
微博奇葩,IT 数码,教育就业
教育就业
美食
教育就业,新闻资讯,投资理财,文艺,娱乐
新闻资讯
体育
娱乐
上海生活,星座命理
旅游
文艺,新闻资讯,旅游,投资理财,美食
旅游
龍俊亨,B2ST,自由,搖滾,日韩音乐控,英国,天蝎
娱乐,文艺,投资理财,健康,旅游

娱乐
娱乐,时尚,IT 数码
名人明星,美食
美食,文艺
旅游,体育,美女,健康,美食,IT 数码,汽车
美食,娱乐
文艺,时尚,娱乐,美食,旅游
娱乐,美女,时尚
文艺
娱乐
吃货,腐,盗墓笔记,猫控,游戏动漫
旅游,时尚,星座命理,美食,娱乐
梦想,吃喝玩乐,旅行,上网
健康,美女,时尚
时尚,游戏动漫,新闻资讯,健康,IT 数码,汽车
美女,汽车,娱乐
健康,美食,新闻资讯,汽车,娱乐,体育
美女
美食
体育
旅游,军事,体育
旅游,美食,文艺
投资理财,美女,星座命理,军事,娱乐
文艺
508,3008,408,308,307,207,4S 店,天津,东风标致,汽车
军事
美女,汽车,娱乐,时尚
健康
美食,文艺,汽车,IT 数码,军事,游戏动漫,旅游
体育,笑,随性,傻
美女,旅游
美女,投资理财,游戏动漫,健康,美食,星座命理
游戏动漫,时尚,美食,文艺,娱乐
双鱼,柯南,双重性格,网球,宅,90 后,快乐,互粉,旅游,游戏动漫
名人明星
健康,美食,旅游,娱乐,文艺
健康,新闻资讯,旅游,美食,IT 数码
美食,文艺,旅游
教育就业,投资理财,健康,汽车,美女
新闻资讯
购物,个性,游戏动漫,文艺
星座命理,文艺,美食,娱乐,时尚
美食,娱乐,美食
美食

时尚
健康,美食,娱乐
IT 数码
健康,星座命理,时尚,娱乐
健康,时尚,旅游,美食
教育就业,IT 数码,军事,美女,娱乐
教育就业,时尚
娱乐,文艺
新闻资讯,游戏动漫,娱乐,IT 数码
美女,体育
新闻资讯,美女,教育就业,投资理财,美食
3 毛粉丝,耽美,郑允浩,动漫迷
四川生活,娱乐
微博奇葩,星座命理,搞笑幽默
娱乐
感性,简单,独立,做梦
时尚,娱乐,旅游
旅游,游戏动漫
星座命理
美女,外贸
健康,美食,旅游,文艺,娱乐
健康,旅游,IT 数码
娱乐,健康,星座命理,读书,电影,旅游,文艺
汽车,娱乐,体育,IT 数码,军事,文艺
摄影控,音乐,美食,旅游
娱乐
美食,汽车,娱乐,军事
美食,时尚,旅游
懒,爱旅游,健康
美食
娱乐
投资理财,旅游,美食
新闻资讯,投资理财,旅游,军事,娱乐
星座命理,教育就业,体育
游戏,狮子座,90 后但我不脑残,爱唱歌,交朋友,火影,吃货一枚,盗墓笔记,游泳,旅游
IT 数码
星座命理,时尚,文艺,旅游,娱乐
美女,美食,文艺
中国地理,影视后期,摄影摄像,IT 数码,教育就业,美食,健康,旅游
游戏动漫,投资理财,军事
投资理财,军事,体育
汽车,投资理财,文艺
文艺,星座命理,娱乐,时尚,IT 数码
文艺,娱乐,美食,游戏动漫,IT 数码

星座命理,时尚,娱乐,健康,旅游,美食
微博,单身,电视剧,喜欢唱歌,自信,美食,IT 数码,美女,游戏动漫,军事
汽车,旅游
星座运势,新闻趣事,美食,无锡生活,星座命理,旅游,娱乐,健康,时尚
名人明星,萌宠
游戏动漫,体育,星座命理
新闻资讯
搞笑幽默,微博奇葩,游戏动漫,娱乐,教育就业
时尚
健康,旅游,美食,文艺,娱乐
时尚,健康,美食,文艺,娱乐
娱乐,美食
文艺,娱乐,旅游,星座命理,美食
美食,汽车,健康,星座命理,娱乐
新闻资讯,军事,娱乐,投资理财
美食,旅游,IT 数码,健康,时尚,投资理财
新闻趣事,美女,投资理财,美食,军事
旅游
惊悚悬疑,建築師,腹肌,爱睡觉,设计师,篮球,美剧
美食,视频音乐,搞笑幽默,新闻趣事,新加坡生活,教育就业,新闻资讯,时尚,文艺,娱乐
游戏动漫
学生
旅游
星座命理,时尚
健康
iphone,服饰,健康,娱乐,美食,时尚
娱乐,文艺
旅游,美食,IT 数码,文艺,体育
旅游
旅游,美食
文艺
时尚,美女,美食
体育
投资理财
新闻资讯,体育
旅游,美食,娱乐,文艺,健康,星座命理,IT 数码
娱乐
搞笑幽默,新闻趣事,美图摄影,江门生活,旅游,健康,美女,时尚
健康,星座命理,教育就业,娱乐,时尚,旅游
体育,美女,健康,旅游
旅游
旅游,美食,萌宠,穿衣美容,视频音乐
证券,天通银,天通金,黄金投资,投资理财
美女,娱乐

穿衣美容,星座命理,新闻趣事
健康,新闻资讯,娱乐
健康,汽车,体育
旅游,星座命理,美食,娱乐,游戏动漫
时尚,旅游,军事
时尚
旅游
旅游
星座命理,体育,游戏动漫
健康,教育就业,军事
美食,汽车,健康,旅游,时尚
兵长,腐女,爱自由,神经,TaylorSwift,旅游
旅游,娱乐,投资理财,健康,文艺
时尚,星座命理,IT 数码
新闻资讯,健康,汽车,时尚,IT 数码
游戏动漫,旅游,军事
文艺,娱乐,时尚,健康,旅游
健康,旅游,美食
游戏动漫,教育就业,投资理财
旅游,健康,时尚,汽车,星座命理
健康,美女,文艺
旅游,教育就业,星座命理
爱自由,娱乐
游戏动漫,旅游,美食,体育,娱乐
星座命理,时尚
美女,星座命理,投资理财,体育
旅游,美食,文艺
时尚,文艺,体育,旅游,天秤座
健康,美食,旅游,新闻资讯,文艺
星座命理,汽车,旅游,美食
文艺,多愁善感,粉红控,星座,音樂,传媒,电视剧,跳舞,爱美食,爱玩
运动鞋,白袜子,SM,GAY,体育
娱乐
健康
投资理财,美食,时尚,娱乐,旅游
游戏动漫,IT 数码,美女
教育就业,时尚,美食,旅游,星座命理
健康,旅遊,教育就業,時尚,娯樂
美女
旅游,娱乐,时尚,文艺
游戏动漫
投资理财,汽车,游戏动漫,旅游,娱乐
星座命理,健康,旅游,时尚
德怀特霍华德,俄克拉荷马雷霆,单身,空空,FairyTail,90 后,军事,游戏动漫,体育

投资理财,旅游,军事
游戏动漫,文艺,娱乐
美女,军事,娱乐
汽车,投资理财,体育,IT 数码,文艺,教育就业
IT 数码,美食,星座命理,旅游,游戏动漫,汽车
生活,吃,搞笑,天蝎座,网购,游戏
美食,教育就业,游戏动漫,军事,IT 数码,文艺
旅游
新闻资讯,星座命理,体育
美女,时尚,军事
健康,美女,军事
游戏动漫,文艺,娱乐
星座命理
娱乐
旅游
搞笑幽默,文艺,新闻资讯,游戏动漫,美女,健康,军事,娱乐
电影,摩羯,睡觉睡到自然醒,新闻资讯,体育,娱乐
投资理财,娱乐,时尚,健康
游戏动漫,汽车,娱乐
体育
娱乐,旅游,星座命理,时尚,文艺
文艺
美食,健康,旅游,娱乐
文艺
文艺,星座命理
美食,江苏生活
投资理财,星座命理,文艺
健康,游戏动漫,IT 数码
游戏动漫,健康,旅游,美食
美女,汽车
教育就业,投资理财,文艺,娱乐
学习
旅游
美女,投资理财,军事,汽车
娱乐
游戏动漫,IT 数码,体育
星座命理,文艺,旅游,军事,娱乐
旅游
名人明星
娱乐,时尚,文艺,美食,星座命理
新闻资讯
旅游
汽车,旅游,IT 数码
新闻资讯,汽车,IT 数码

新闻资讯,时尚,IT 数码
汽车,投资理财,IT 数码
旅游,唯美,轻音乐,健康,娱乐,文艺
IT 数码,娱乐,体育,游戏动漫
健康,美食,时尚,娱乐,体育
健康,美食,旅游,体育,军事
美食
双节棍,散打,跆拳道,武术,文艺,美女,美食,娱乐,体育
盐城生活,文艺
钻石珠宝,华东师范大学,结婚钻戒,钻戒,上海,上海人,钻石婚戒,钻石,珠宝,戴欧妮
游戏动漫
游戏动漫,星座命理
好性格,巨蟹座,O 型血,K 歌,时事,软件,哈哈,旅游美食爱好者,运动,篮球
汽车,娱乐
搞笑幽默,游戏动漫,军事,IT 数码,文艺,娱乐
IT 数码,新闻资讯,健康,体育,军事
美女,军事,旅游
军事,投资理财,汽车,星座命理,娱乐
美女,旅游,文艺
投资理财,美食,体育
美女,游戏动漫,星座命理,文艺
旅游
美女,时尚,美食,文艺,娱乐
旅游
星座命理
篮球
游戏动漫,旅游,汽车
时尚,美食
健康,美食
汽车,旅游,新闻资讯,IT 数码,军事
健康,文艺,旅游,星座命理,美食
星座命理,娱乐
时尚,汽车,IT 数码,娱乐
健康,时尚,美食,娱乐,旅游
旅游,文艺,电视剧,狮子座,学生一族,文字控
娱乐,游戏动漫,星座命理,美食,文艺
游戏动漫
时尚,旅游,IT 数码,文艺,娱乐
美食,游戏动漫,时尚,美女,娱乐
健康
聊天,时尚
体育
萝莉控,90 後,偶喜欢玩,时尚潮流,nba,帅哥,狮子座,阳光,单身,上网
文艺,美食,旅游,健康,瘦身,体育,时尚,教育就业,星座命理,娱乐

投资理财,美女,旅游,军事
健康,新闻资讯,旅游,娱乐
游戏动漫,娱乐
美食,军事,IT 数码
教育就业
IT 数码,时尚,旅游,游戏动漫,美女,新闻资讯,美食
美剧控,DOTA,体育,游戏动漫
体育
时尚,娱乐,文艺,旅游,美食
旅游,自由,吸血鬼日记
娱乐,旅游,星座命理,美食
星座命理,旅游,美食,时尚,健康
创业,爱情,单纯,商业,可爱,女人,朋友,爱,交朋友,美女
美食,文艺,体育,旅游
时尚,汽车,旅游,美食,娱乐
娱乐,投资理财,游戏动漫,美食,旅游
搞笑幽默,健康
娱乐,星座命理,美女,汽车,文艺
文艺
品牌,时尚
游戏动漫,教育就业,IT 数码,新闻资讯
星座命理
军事,游戏动漫,娱乐,IT 数码,星座命理
文艺,娱乐,美食,旅游,星座命理
娱乐,美女,教育就业,文艺,体育
健康,旅游,新闻资讯,美食
新闻资讯,投资理财,健康,旅游,美食
幻想,电脑,聊天,美食,新闻资讯,IT 数码,星座命理,时尚,娱乐
军事,IT 数码,文艺,汽车,时尚
好性格,摩羯座,新闻,安静,爱笑,散步,蓝色,热心,和睦
时尚,健康,旅游,教育就业,投资理财
健康,时尚,文艺,娱乐
星座命理,娱乐,投资理财,旅游,健康,美食
情感,哲学,青春,诗歌,双鱼女,读书,音樂,文化,安静,快乐
红酒,幻想,电脑,处女座,微笑,健康,时尚,娱乐,汽车,美食
旅游
读书分享,美女,搞笑幽默,北京生活
新闻资讯,旅游,美食,娱乐,投资理财
美食,教育就业,投资理财,娱乐,健康
新闻资讯,文艺,旅游,美食,IT 数码
新闻资讯,投资理财,时尚,汽车,体育
星座命理
美女
美食,游戏动漫,体育

旅游,军事,IT 数码,文艺,娱乐
投资理财
时尚,我就是我,旅游美食爱好者,交朋友
游戏动漫,娱乐,星座命理
星座命理,娱乐,IT 数码,汽车
星座命理,IT 数码
时尚
星座命理,汽车,IT 数码,游戏动漫,娱乐
旅游,视频音乐,美女
小清新,化妆品,美容护肤,服装,音乐控,90 後,美食控,吃喝玩乐,看电影,星座控
健康,时尚,文艺,星座命理,投资理财
健康
IT 数码,旅游,健康,娱乐,美食
新闻趣事,宝宝,阅读,电视,自然,人生,工作,健康,文艺,美食
八卦杂谈,搞笑幽默,投资理财,健康,美女,文艺,娱乐
新闻资讯,军事,投资理财,健康,教育就业,美食,体育
美女
军事,投资理财
尤文图斯,阿森纳,意大利,威密
旅游,健康,娱乐,时尚
健康
情感生活,文艺
新闻资讯,时尚,军事,娱乐,IT 数码
体育
旅游,游戏动漫,体育,投资理财,汽车
教育就业,健康,IT 数码
教育就业,健康,新闻资讯,星座命理
文艺
吸血鬼日记,音乐发烧友,笑,坚强,自行车,星座命理,旅游,健康,美女,游戏动漫
健康,游戏动漫,星座命理,旅游
游戏动漫
美食,美女,IT 数码
投资理财,时尚,美食,文艺,娱乐
美食
健康,娱乐,美食,IT 数码
健康,时尚,旅游,美食,文艺,娱乐
文艺,时尚,旅游,美食,IT 数码,娱乐
投资理财,执子之手,与子偕老,不靠谱呀
娱乐,文艺,游戏动漫
微博奇葩,搞笑幽默,美女,游戏动漫,IT 数码,军事,体育
文艺,美食,旅游,娱乐
美食,星座命理,新闻趣事,搞笑幽默,穿衣美容,时尚
视频音乐,旅游,美食,微博奇葩,穿衣美容
旅游,新闻趣事,微博奇葩,搞笑幽默,军事,新闻资讯

教育就业
美食
游戏动漫,星座命理
新闻资讯
时线,JJ,林俊杰,天蝎女,90 后,薰衣草,幻想家,美空,紫色控
新闻资讯,时尚,文艺,娱乐,体育
文艺
投资理财,旅游,美食,健康
游戏动漫
旅游
时尚
学生一枚,游泳,羽毛球,摄影爱好者,宅男,手机,NBA,好书,汽车
美食
星座命理,旅游,美食,IT 数码,娱乐
美食
足球,烹饪
美食,健康,美女,游戏动漫,IT 数码
军事,健康,汽车,时尚,体育
处女座女生
星座命理
文艺
游戏动漫,投资理财,美女,IT 数码,娱乐
美食,投资理财,教育就业,新闻资讯,星座命理
旅游
美食
美食
文艺
旅游,微博奇葩,视频音乐,广东生活,搞笑幽默,健康
健康,投资理财,美食
健康,投资理财,新闻资讯,体育,娱乐
IT 数码
美食,娱乐,投资理财,健康,美女,新闻资讯,军事
名人明星,时尚,旅游,星座命理,美食,娱乐
星座命理,旅游
时尚,娱乐,美食
教育就业,星座命理,新闻资讯,美食,时尚,娱乐
新闻资讯,投资理财,教育就业,美食
娱乐,旅游,游戏动漫,体育
旅游,时尚,教育就业,投资理财,星座命理
健康,星座命理,时尚,教育就业,投资理财
文艺,娱乐
投资理财,旅游,健康,星座命理,娱乐
娱乐,旅游,美女
汽车,健康,工作狂

星座命理,军事,IT 数码,汽车,体育
初三党,乐橙,魏晨,休闲,巨蟹座,我就是我,爱唱歌
时尚,旅游
新闻资讯,健康,旅游
旅游,星座命理,健康,新闻资讯
旅游,时尚,美食,美食,投资理财
健康,时尚,星座命理,旅游,娱乐
文艺
文艺
健康,时尚,旅游,美食,文艺
投资理财
自由,自贱自恋,王墨镜不磨叽
游戏动漫
娱乐
文艺
星座命理,新闻资讯,旅游,健康,投资理财
娱乐,健康,IT 数码
美食,娱乐
娱乐
穿衣美容,时尚
文艺
旅游
体育,IT 数码
体育,娱乐
体育,IT 数码,新闻资讯
美食
娱乐
文艺,旅游,美食
视频音乐,萌宠,名人明星,娱乐,星座命理
健康
投资理财
教育就业
娱乐,游戏动漫,军事,IT 数码,体育
星座命理,新闻资讯
汽车,美女
旅游,穿衣美容,新闻趣事,搞笑幽默,惠州生活,时尚
旅游,美食,娱乐
汽车,旅游,美食,文艺,娱乐
旅游,时尚
穷,cl,体育,IT 数码,旅游,汽车
健康,绘画,小动物控,狮子座,旅游,努力 Ing,美文控,音乐迷,幻想
新闻资讯,军事
体育,IT 数码
新闻趣事,校园生活,科学探索,搞笑幽默,星座命理,娱乐

新闻资讯,IT 数码,时尚,投资理财,娱乐
美食,汽车,文艺,旅游,投资理财
旅游,新闻资讯
娱乐
星座命理,新闻资讯,投资理财,娱乐,时尚
游戏动漫
投资理财
健康
美食
美食,军事,游戏动漫
文艺,IT 数码,投资理财,新闻资讯,健康,娱乐
汽车
体育
时尚,文艺,IT 数码,旅游,娱乐
穿衣美容,旅游,名人明星,搞笑幽默,辽宁生活,军事,体育
汽车,美食,星座命理,旅游,娱乐
新闻资讯,旅游,时尚,文艺,娱乐
美食,时尚,旅游,健康,投资理财
旅游,文艺,健康,教育就业
时尚,健康,美食,娱乐,新闻资讯
星座命理,美食
游戏动漫
随性,睡觉睡到自然醒,会计,摩羯座,执着
八卦杂谈
星座命理,娱乐,美食
旅游,山西生活,校园生活
穿衣美容,新闻趣事,搞笑幽默,旅游
文艺
健康,娱乐,旅游,美食,文艺
微博奇葩,文艺苦逼青年,kirs
娱乐,旅游,教育就业,新闻资讯,IT 数码
旅游,健康,文艺,美食,时尚
汽车
娱乐,新闻资讯,美女,美食,幽默,视频,旅游,体育
文艺,旅游,美食,投资理财,健康
娱乐
旅游,美食,娱乐,胡思乱想,天蝎,喜欢听歌,唱歌
睡觉睡到自然醒,汽车,教育就业,娱乐,健康,投资理财
时尚,体育,IT 数码
新闻资讯,游戏动漫,旅游,星座命理,美食
爱幻想
美女,星座命理,军事,文艺,娱乐
旅游
文艺

教育就业,美食,健康,新闻资讯,娱乐
美食,旅游,娱乐
时尚
旅游
汽车
旅游,健康,美食,娱乐
旅游
旅游,星座命理,美食,文艺,娱乐
星座命理,江苏生活,美食,投资理财,文艺
美食,双鱼座,听音乐,爱玩,电影迷,文艺,旅游,健康,时尚
投资理财
游戏动漫,美女
新闻资讯,文艺
时尚,美女,娱乐,汽车,旅游,美食,IT 数码
旅游,名人明星,搞笑幽默,洛阳生活,旅游摄影,通信,体育,美食
美食
旅游,健康
旅游
爱猫,游戏,时尚,旅游,美食
时尚,旅游,娱乐
新闻资讯,健康,汽车,投资理财
向日葵,爱睡觉,帅哥,汽车,时尚,美食,旅游,星座命理
旅游,星座命理,美食,IT 数码,文艺
时尚,娱乐,文艺
青春,个性,恐怖片,商业,朋友,动画,游戏动漫,美女,文艺,娱乐
健康,投资,执着,爱玩,上海,电脑,聊天,吃货一枚,时尚
教育就业,健康,时尚,美食,星座命理
美食,娱乐
教育就业,健康,娱乐
萌宠,名人明星,搞笑幽默
投资理财,健康
星座命理
美食,旅游,投资理财,健康,时尚
教育就业,投资理财,旅游,美食,健康
时尚
娱乐
美食,游戏动漫
旅游,娱乐,健康,新闻资讯,教育就业
IT 数码,汽车,时尚,教育就业,体育
汽车,健康,新闻资讯,军事,体育
体育,文艺,娱乐,时尚,旅游
娱乐,时尚,教育就业,健康,旅游,美食,文艺
旅游
美食,时尚,星座命理,薰衣草,感性,紫色,旅游

时尚
时尚,娱乐
文艺,时尚,健康,旅游,美食
娱乐,旅游,美食
旅游,美食
摩羯女,快乐,喜欢旅游,睡觉睡到自然醒,猫,星座控
健康,军事,旅游,美女,IT 数码
旅游,美女,体育,文艺,IT 数码,新闻资讯
美女
时尚,娱乐,旅游
爱笑,学生一枚,欧美控,喜欢旅游,水瓶,cosplay,交朋友,法国,美食,游戏动漫
娱乐
汽车,旅游,美女,美食,军事,IT 数码,体育
一个人特么无聊,的
新闻趣事,打字，看书
健康
星座命理,文艺
旅游,美食,文艺,娱乐
娱乐
娱乐
娱乐
时尚
汽车
IT 数码,教育就业,新闻资讯,星座命理,时尚,娱乐
健康,娱乐
星座命理,旅遊,美食,娛樂
美食,时尚
健康,时尚,IT 数码,旅游,星座命理
星座命理,旅游,健康,教育就业,文艺
文艺,旅游,美食
时尚,娱乐
游戏动漫,新闻资讯
文艺,星座命理,游戏动漫,健康,旅游,美食,娱乐
名人明星,电脑,潮流,爱美食,K 歌,品牌,音乐发烧友
娱乐
名人明星,旅游,视频音乐,微博奇葩,新闻趣事,搞笑幽默,体育,IT 数码,军事
新闻资讯
美食,穿衣美容
喜欢唱歌,看电影,音乐爱好者,80 後,书法,陈奕迅,娱乐
美食
投资理财,新闻资讯
时尚,游戏动漫,娱乐
偶稀饭睡觉觉
美女,军事,教育就业,时尚,投资理财,新闻资讯,娱乐

爱自由,美女,汽车,旅游,IT 数码,文艺
搞笑幽默
美食,娱乐,时尚,旅游,文艺
文艺
美食,旅游,时尚,健康,娱乐
游戏动漫,娱乐
美食,娱乐,新闻资讯,教育就业
投资理财
军事
体育,娱乐,新闻资讯,投资理财,旅游
摩羯,夜猫子,美食,旅游
文艺
社会,爱旅行,格子控,明星,美食
星座命理,健康,游戏动漫,美女,新闻资讯
游戏动漫,美食,美女,汽车,文艺,IT 数码,军事
体育,娱乐
旅游
基督教信仰,投资理财,旅游,摄影
视频音乐,娱乐
军事,星座命理,美食,娱乐,新闻资讯
旅游,娱乐,星座命理,美食,时尚
美食
娱乐,军事,健康,新闻资讯,文艺
体育,娱乐
投资理财,时尚,健康,旅游,美食
茶叶,木瓜,番石榴,农业,市场营销,财经资讯,旅游,东莞生活,娱乐,体育
IT 数码,新闻资讯,娱乐,旅游,体育
新闻资讯,游戏动漫,军事,IT 数码,娱乐
美食
爱旅行,旅游
名人明星,时尚,娱乐
文艺
体育
通信,科幻,男人,wow,生活大爆炸,旅游,星座命理,游戏动漫,美食
文艺,时尚,美食,娱乐,健康
健康,时尚,IT 数码,娱乐,美食
帅哥美女,日韩,人胎素,原单,美白针,穿衣打扮,价格公道,潮牌,加拿大代购,韩国进口美衣
体育
IT 数码,文艺,星座命理,新闻资讯,汽车
旅游,美食
旅游,美食,星座命理
旅游
美食
新闻资讯,时尚,健康,旅游,星座命理

新闻资讯,爱自由,平常心,怀旧,美剧,汽车,好性格,看电影,听音乐,淘宝
美食
娱乐
娱乐,美女,游戏动漫,星座命理,军事
旅游
旅游
美食,旅游,文艺,娱乐
海南生活,旅游,情感生活,美女,时尚,星座命理,娱乐
教育就业,投资理财,美食,新闻资讯,健康
东软,台球,麻将,投资理财,娱乐,美女,健康,旅游
旅游,文艺,娱乐,时尚,美食,美食
体育,新闻资讯,健康,美食,美女
投资理财,旅游,美食,文艺,娱乐
娱乐,健康,旅游,美食
汽车,健康,旅游,IT 数码,美食
时尚
娱乐
美食
汽车,美女,旅游,体育,娱乐
IT 数码
旅游,美女,时尚,文艺
IT 数码
幽默,交友,休闲,学生,宅,娱乐,体育
教育就业,文艺
旅游,stayreal,相信音樂,阿信,五月天
投资理财,汽车,娱乐,体育
旅游,毽子,羽毛
健康,电影爱好者,音乐爱好者,美食
文艺,美食,时尚,健康
视频音乐,游戏动漫
美女,旅游,健康,文艺,娱乐
美女,游戏动漫,旅游,IT 数码,投资理财,汽车,体育
搞笑幽默,萌宠,矛盾,爱猫,疯狂,沉默,无厘头,投资理财,美食
美女
新闻资讯,文艺,娱乐
美女,文艺,军事,汽车,时尚,体育,娱乐
投资理财,健康,旅游,美食,军事
视频音乐,星座命理,汽车,旅游,娱乐
文艺
旅游,新闻资讯,健康,星座命理,时尚,娱乐
星座命理,健康
旅游,IT 数码,体育,娱乐
新闻趣事,萌宠
双子女,微博控,网购,营销,电子商务,音乐控,星座控,唱 k,B 型血,80 后宅女

旅游,汽车,文艺,IT 数码
时尚,教育就业,文艺,旅游,健康,星座命理
求帅哥肌肉男,空少,武警等,平常心
娱乐,文艺,美食,旅游,游戏动漫
文艺,IT 数码,星座命理,时尚,娱乐
IT 数码,时尚
游戏动漫,IT 数码
文艺
健康,旅游,美食,娱乐
娱乐,美食
活泼开朗,性感,快乐大本营,时装,海绵宝宝,娱乐
娱乐,文艺
美食,军事,文艺,娱乐,体育
时尚
投资理财,健康,美食,娱乐
美女
文艺
旅游,美食
娱乐,健康,旅游,星座命理
健康,时尚,旅游,IT 数码,娱乐
新闻资讯,星座命理,汽车,娱乐
名人明星,美女
汽车
旅游,投资理财,娱乐,IT 数码,军事
新闻资讯,娱乐,汽车
美食,健康,旅游
娱乐,时尚
汽车,旅游,星座命理,健康,投资理财
健康,教育就业,投资理财
美食
健康,汽车
健康,新闻资讯
投资理财
投资理财,新闻资讯,汽车,IT 数码,体育
新闻趣事,星座命理,开朗,教育,画画,文艺,艺术,善良,处女座
娱乐
宅男,健康,新闻资讯,娱乐,军事
星座命理,娱乐
游戏动漫,汽车,旅游,美食
汽车,时尚,星座命理,IT 数码,娱乐
90 后的我,金在中,吃货,爱自由,仙后,爱生活,帅哥,逛街,美食
汽车,游戏动漫,星座命理,军事,IT 数码
游戏动漫,时尚,美食
时尚,美食

旅游
游戏动漫,时尚,美女,娱乐,体育
时尚
投资理财,美食,娱乐
穿衣美容,搞笑幽默,深圳生活,文艺,健康,旅游,美食,游戏动漫
IT 数码,投资理财
健康,投资理财,新闻资讯,军事,IT 数码
美女,旅游,IT 数码
土象星座
娱乐
体育
健康
美女,健康
投资理财,汽车,时尚,健康,IT 数码
旅游,文艺
广东生活,教育就业,文艺,娱乐
健康,汽车,军事,娱乐,体育
时尚,汽车,旅游
IT 数码,星座命理
萌宠,旅游,上海生活,健康
新闻趣事,视频音乐,文艺,教育就业,新闻资讯,游戏动漫,娱乐
美食
旅游,汽车,星座命理,娱乐
微博奇葩,娱乐
健康,美女,投资理财,军事,文艺
旅游,搞笑幽默,视频音乐,新闻趣事
健康,美女,汽车,IT 数码,体育
搞笑幽默,重口味,花样美男,美图摄影,自拍,购物,海绵宝宝,gay,时尚,娱乐
星座命理,新闻资讯,健康,投资理财,军事
美女,游戏动漫
娱乐,健康,时尚,星座命理,文艺,教育就业
美食,旅游,健康,娱乐,动画,拍照,偶喜欢玩,创业
时尚,美食,星座命理,娱乐,文艺
穿衣美容
汽车,军事,IT 数码
星座命理,美食,IT 数码,娱乐,体育
星座命理,旅游,健康,新闻资讯,汽车
时尚,娱乐
娱乐
汽车,游戏动漫
体育
北京生活,健康
日本生活,娱乐,游戏动漫
时尚,美食

文艺,旅游,美食
静静静静静,美食,旅游,电影,看书,看美景,发呆呆,听歌,宅哥哥
旅游,美食,娱乐,健康,新闻资讯
旅游,娱乐,时尚,汽车,新闻资讯,美食,体育
美食,旅游
旅游,美食
旅游,美食,美女,IT 数码
游戏动漫,美食,星座命理,体育,娱乐
八卦杂谈,视频音乐,旅游,校园生活,搞笑幽默
南京生活,教育就业
新闻资讯
旅游美食
娱乐
投资理财,健康,汽车,教育就业,旅游,游戏动漫,IT 数码
教育就业,旅游,美食,IT 数码,娱乐
新闻资讯,星座命理,美女,教育就业,娱乐
教育就业,旅游,旅游美食爱好者,爱睡觉,音乐爱好者,90,阳光,孩子气,时尚
娱乐,投资理财,健康,时尚,文艺
美女,军事,体育
旅游,健康,娱乐
美女,娱乐
文艺
爱自由,时尚,东方神起,书籍,旅游,文艺
美食,天蝎座 80 后,宠物,音乐爱好者,游戏动漫,旅游,美食,娱乐
娱乐,时尚,旅游,文艺
汽车,时尚,IT 数码
军事
旅游
体育,旅游,星座命理,美食,教育就业,新闻资讯,娱乐
投资理财,旅游,IT 数码,娱乐
文艺,体育,娱乐
星座命理
健康
校园生活,时尚
真诚,青春,个性,瘦身,文艺,时尚,旅游,美食,新闻资讯,娱乐
时尚,汽车,旅游,美食,娱乐
投资理财,教育就业,美女,新闻资讯,健康,军事
星座命理
旅游,搞笑幽默,福建生活
健康,时尚,美食
美女,时尚,娱乐,投资理财,IT 数码
旅游
微博奇葩,穿衣美容,星座命理,新闻趣事,时尚,新闻资讯,文艺,娱乐
健康

美食,健康,教育就业,文艺
广东生活,新闻趣事,穿衣美容,星座命理,搞笑幽默,新闻资讯,军事,娱乐,时尚,健康
旅游
旅游
娱乐,软件,电影控,音乐爱好者,双重性格
娱乐
美食,游戏动漫,美女,投资理财
喜欢恋爱的妖精
教育就业,文艺,体育
娱乐,旅游,美女,美食,文艺,体育
文艺
旅游,星座运势,tvb,大学生,90後,旅游美食,执着,狮子座
游戏动漫
时尚,娱乐,星座命理
娱乐,体育
视频音乐
汽车,新闻资讯,IT数码
军事
星座命理
新闻资讯,汽车,文艺,旅游,健康,投资理财,时尚,美食
体育,旅游,星座命理,军事,文艺
旅游
随性,学生一族,散步,快乐,偶稀饭睡觉觉,淘宝控,懒人,美食,文艺
旅遊,旅游
新闻资讯,教育就业,旅游
IT数码,投资理财,汽车,美女,军事
旅游,星座命理,美食,投资理财
美女,美食,时尚,新闻资讯,IT数码
旅游,娱乐,投资理财,汽车,体育
名人明星
投资理财,新闻资讯,星座命理,娱乐
美食,体育,娱乐,游戏动漫
IT数码
发呆,简单,会计,网络营销,旅游美食,美食爱好者,吃喝玩乐,咖啡,我就是我,淘宝
天生很温柔,投资理财,旅游,军事
娛樂
美食,穿衣美容,福建生活,新闻资讯,IT数码,旅游
视频音乐
新闻资讯,美食,旅游,文艺,娱乐
IT数码,健康,星座命理
旅游,时尚
exom是一个集体,唯饭退散,exom团饭
时尚,旅游,IT数码
IT数码,时尚,文艺

汽车,旅游,IT 数码
旅游爱好者,摄影,唱歌,宅,苹果控,维尼夫妇,文艺,IT 数码
美女,娱乐,文艺,体育
娱乐
星座命理,视频音乐,旅游,穿衣美容,娱乐
微博奇葩,娱乐
时尚,教育就业,健康,旅游,美食
娱乐,美食,时尚
美食
搞笑幽默,娱乐
时尚,新闻资讯,投资理财,旅游,IT 数码
娱乐,军事
美食,游戏动漫,星座命理,文艺,时尚,娱乐
个性,瘦身,傻，潮流，瘦身,情绪化,淘宝控,乐天派,音乐控,美食
娱乐,星座命理
旅游
文艺,娱乐,汽车,美女
服装,商场,商场管理,服饰,服装行业,服装批发,花车,龙门架,租赁,道具
体育,健康
投资理财,教育就业
文艺,体育,时尚,旅游,健康
新闻趣事,视频音乐,名人明星,星座命理,搞笑幽默,微博奇葩,文艺,汽车,时尚
投资理财,美食,时尚
韩剧
星座命理,游戏动漫
皇马一恒大,球迷,民族主义分子,抗日分子,新闻资讯,绝对外向,乐观,爱国主义者,超级军事迷
健康,新闻资讯,文艺,体育,旅游
IT 数码
美食
娱乐,游戏动漫,新闻资讯,星座命理,美食
星座命理,时尚,健康,旅游,美食
摇滚,文艺,美女,旅游,美食,体育
旅游
旅游,星座命理,美食,军事,娱乐
娱乐
娱乐,游戏动漫
时尚,投资理财,旅游,教育就业,文艺,健康
萌宠,旅游,新闻趣事,视频音乐,大庆生活
旅游,美食,时尚,投资理财
娱乐,旅游,美食,时尚,体育
体育,娱乐
美女
重庆生活,时尚,美女,旅游,美食,娱乐

耽美,闷骚,腐女,盗墓笔记,美食
体育
名人明星,文艺
美食,健康,新闻资讯,旅游,汽车
健康,旅游,游戏动漫,时尚,娱乐
娱乐,文艺,美食,星座命理,旅游
搜索,导航,搜索框输入法,IT 数码
二,爱笑,宅,吃货,善良,爱猫
时尚,美食,娱乐,文艺,旅游
美食,文艺,IT 数码,新闻资讯,健康,汽车,旅游
星座命理,IT 数码
美食
旅游,汽车,美食,娱乐,时尚
星座命理,美食
教育就业,新闻资讯,美食,星座命理,IT 数码
视频音乐,健康,时尚,娱乐
军事,游戏动漫
娱乐
教育就业
娱乐,星座命理,游戏动漫,体育
娱乐,美食
文艺,娱乐,游戏动漫,IT 数码,军事,旅游,美女
美食,军事
安防,教育就业,旅游,新闻资讯,投资理财,体育
体育,文艺,旅游,美食,娱乐
娱乐
文艺,IT 数码,汽车,时尚,体育
文艺,时尚,美女,健康,娱乐
娱乐
美食
新闻趣事,美女
教育就业,健康,美食,军事,新闻资讯,旅游,体育,文艺
名人明星
游戏动漫,星座命理
旅游
深圳,广州,粤语,广东新闻,广东吃喝玩乐,广东人,广东大学,广东旅游,广东美食,广东
美女,穿衣美容,搞笑幽默,时尚,旅游,美食,娱乐
星座命理,投资理财,旅游,时尚,健康,美食,娱乐
健康,汽车
娱乐
唱 K,美食,旅游,文艺
摄影控
旅游
文艺,旅游,营销,男人,随性,周杰伦

成都生活,娱乐,游戏动漫,汽车
上海生活,网球,双鱼座,深圳,90 后但我不脑残
健康,教育就业,美食,文艺,娱乐
军事,汽车,游戏动漫,IT 数码,娱乐
时尚
游戏动漫,旅游,健康,时尚,娱乐
新闻趣事,南京生活,文艺,体育,娱乐,新闻资讯,军事
新闻趣事,读书分享,动漫
健康
军事,旅游,美女,时尚,娱乐
新闻趣事,搞笑幽默,视频音乐,微博奇葩,时尚,旅游,文艺,娱乐
健康,星座命理,美食,文艺,娱乐
文艺,娱乐
穿衣美容,新闻趣事,搞笑幽默,微博奇葩,体育,打篮球
游戏动漫,IT 数码
时尚,汽车,美女,健康,娱乐
娱乐
新闻资讯,投资理财,美女,军事,IT 数码
名人明星,体育
校园生活,视频音乐,IT 数码,投资理财
新闻趣事,柳州生活,体育资讯,搞笑幽默,汽车,时尚,美食,游戏动漫,旅游
娱乐,欧美乐,体育
旅游,美食,健康,星座命理,文艺,汽车,时尚
美女,游戏动漫,美食,娱乐,体育
投资理财
美图摄影,星座运势,星座命理
双子女,美术,美女
投资理财,教育就业,游戏动漫,汽车,体育
旅游
美食,文艺,美女,游戏动漫,美食,旅游
文艺,健康,娱乐
旅游
旅游
娱乐,健康,投资理财,文艺,美食
娱乐,美女,美食
美女,美食,星座命理,旅游,娱乐,游戏动漫
娱乐,IT 数码,军事,体育
美国,医学,文学,学习,天蝎座,欧美音乐,多愁善感,时事,热情,旅游
时尚,娱乐
睡觉睡到自然醒,娱乐
星座命理,名人明星,美食,萌宠,视频音乐
娱乐
IT 数码,娱乐
小说,爱吃,朋友,玩,快乐,体育,美女,游戏动漫,旅游,美食

娱乐,投资理财,星座命理,旅游,健康
体育,美食,IT 数码,美女,军事
内向,看电影,幼师
EXO,都暻秀,微博奇葩,娱乐,游戏动漫
文艺,时尚,美女,军事,娱乐
新闻资讯,旅游,美食,投资理财,美女,IT 数码,时尚,健康
汽车,时尚,旅游,美食,娱乐
娱乐
时尚
新闻资讯,体育,汽车,投资理财,军事,娱乐
旅游,体育,汽车,军事,新闻资讯
文艺,体育
书法,公关,文艺,江苏生活
汽车,文艺
娱乐,美食
武汉生活,时尚
新闻资讯
交流,读书,展览,摄影,艺术瓷,旅游,收藏,書法,山水画
美食,穿衣美容,旅游
b 型血,金牛女,学生一族,Music,乐观,90 后但我不脑残,微笑,敏感,无怪癖,时尚
名人明星,娱乐,时尚
娱乐,美女,军事,IT 数码,体育
文艺,IT 数码,娱乐
萌宠,旅游
IT 数码
时尚,新闻资讯
时尚
星座命理
旅游,娱乐,文艺,看电影,听音乐,星座,爱音乐,music,喜欢旅游,美食
文艺,健康,旅游,投资理财,娱乐
旅游
健康,时尚,旅游,美食,娱乐
汽车,IT 数码,美食,娱乐,文艺
投资理财
军事,IT 数码
旅游,视频音乐,微博奇葩,搞笑幽默,美食,军事,文艺,娱乐,体育
投资理财
谢容儿贴吧
诗词,段子,伤感,90 后,80 后,美图,美文,文艺
游戏动漫,娱乐
BestFriend,互听党,双生控,沈贤星,金东炫,李政敏,卢珉宇,赵光敏,赵英敏,Boyfriend
文艺,汽车,投资理财,游戏动漫
新闻资讯,投资理财,时尚,健康,旅游
浪漫,美女

时尚
IT 数码,时评,新闻资讯,投资理财,健康,游戏动漫
健康,投资理财,汽车,旅游,娱乐
美食,旅游,时尚,娱乐,投资理财
新闻资讯,投资理财
美食
音乐,90 后,韩国,爱睡觉,星座控,维尼夫妇,爱自由,90 后非脑残,暮光之城,格子控
湛江生活,读书分享,美食,新闻资讯,时尚,教育就业,星座命理
搞笑幽默,微博奇葩,萌宠,淡定,猫控,旅游,美食,娱乐,发呆,多愁善感
美食,旅游,游戏动漫,娱乐,星座命理
美女
时尚,健康,旅游,美食,星座命理
旅游,娱乐,美食
文艺
投资理财,游戏动漫,美女
美食,教育就业
格子控,喜欢唱歌,90 后但我不脑残,水瓶,潮,星座命理,美女,汽车,娱乐,IT 数码
旅游,健康,汽车,娱乐
星座命理
体育,娱乐
娱乐
视频音乐,紫色,90 后但我不脑残,魔羯座,外貌协会,蓝色,喜欢听歌,交朋友,娱乐
娱乐
健康,旅游,教育就业,投资理财,IT 数码,文艺
新闻资讯
教育就业
旅游,文艺,教育就业,新闻资讯
投资理财,军事,新闻资讯,汽车,星座命理
汽车,体育
新闻趣事,时尚,健康,娱乐,文艺,星座命理
健康
时尚,健康,美女
娱乐
娱乐,健康,旅游,美食,文艺
搞笑幽默,美女,娱乐,体育,星座命理
旅游,美食,军事,文艺,娱乐
微博奇葩,IT 数码,游戏动漫
娱乐
教育就業,投資理財,娛樂,時尚,星座命理
娱乐,游戏动漫
红黑黄黑党,朽木白哉,日番谷,二次元生物,银他妈综合症,動漫控,活泼自来熟,黑崎一护,黑子会发光,kuroko
新闻资讯,美女,投资理财,军事,体育
健康,时尚

吸血鬼,孩子气,旅行,上网,看书,音樂,爱笑,乐观,吃货,矛盾体
美食,旅游,健康,娱乐,文艺
IT 数码,美女
健康,美食
娱乐,时尚,旅游,美食,星座命理
疯子,教育就业,游戏动漫,美女,美食,汽车
旅游
萌宠,旅游,美食,穿衣美容,相机控,韩国代购,小黑裙,时装
游戏动漫,美食,新闻资讯,健康
南宁生活,巨蟹座
文艺
美食,文艺,美女,新闻资讯,IT 数码
星座命理
美食,旅游
汽车,健康,时尚,美食,旅游,星座命理
旅游,美食,健康,时尚,体育,娱乐
美女
视频音乐,古典艺术,日语,写作,皇家马德里,NBA,Jpop,akb48,伪文艺青年,MrChildren
健康
IT 数码
旅游
游戏动漫
体育,新闻资讯,健康,文艺
美食,时尚
美女
搞笑幽默,视频音乐,美食,名人明星
健康,IT 数码,星座命理,美食,娱乐
搞笑幽默,星座命理,旅游,美食,穿衣美容,健康,娱乐
教育就业,时尚,投资理财,美食,旅游
游戏动漫,美食
游戏动漫
增肌,长白岛,塑形,减脂,交友,健身,乒乓球,台球,肚皮舞,瑜伽
时尚
投资理财,娱乐,体育,文艺
健康,时尚,旅游,IT 数码,体育
娱乐,文艺
新闻资讯,旅游,投资理财,美食,体育
美女,体育,娱乐
健康
IT 数码,游戏动漫,时尚,美食,星座命理
玩儿,宠物,美女,旅游
时尚,吃货一枚,暮光之城,游戏动漫
娱乐
娱乐

新闻资讯, 文艺, 体育
投资理财, 旅游, 星座命理, 美食, 军事
美食, 美女, 健康
旅游, 汽车, 美女
汽车
书籍, 教育, 文艺, 新闻资讯, 美食, 健康, 教育就业
旅游
娱乐, 旅游, 美食, 新闻资讯, 时尚
美女, 投资理财, IT 数码, 军事, 美食
投资理财
茶, 摩羯座
娱乐, 星座命理
教育就业, 星座命理, 文艺, 旅游, 军事, 体育
美女, 游戏动漫
美食, 新闻趣事, 微博奇葩, 武汉生活, 搞笑幽默, 时尚, 文艺
娱乐, 星座命理
旅游, 健康
新闻资讯, 文艺
时尚, IT 数码, 美女, 教育就业, 文艺
星座命理, 美食, 军事, 文艺, 健康, 娱乐, 新闻资讯, 美女, 体育
名人明星, 旅游, 微博奇葩, 搞笑幽默, 星座命理, 时尚, 文艺, 娱乐
娱乐
时尚, 汽车, 教育就业
健康, 旅游, 美食, 娱乐
投资理财, 时尚, 健康, 旅游, 教育就业
健康, 旅游, 教育就业, 汽车, 新闻资讯
健康, 美食
汽车, IT 数码, 体育
旅游, 文艺, 新闻资讯, 星座命理, 教育就业, 健康
健康, 新闻资讯, 星座命理, 文艺, 娱乐, 美食, 旅游
旅游
游戏动漫
汽车, 教育就业, 旅游, 文艺, 体育
游戏动漫, 美女, IT 数码, 文艺
时尚, 美女, 女性, 生活, 美容顾问, 化妆品, 护肤达人, 美白祛斑, 美容护肤, 健康
健康, 高尔夫, 投资, 钓鱼, 咖啡, 偶喜欢玩, 投资理财, 新闻资讯, 旅游
时尚, 汽车, 旅游, 娱乐
汽车, 旅游, 美食, IT 数码, 娱乐
星座命理
旅游, 体育, 健康, 娱乐
美女, 教育就业, 军事
汽车, 娱乐, 美女, 美食, 旅游
时尚, 体育, 教育就业, 星座命理, 军事
母婴, 健康, 育儿, 准妈妈, 亲子, 黄石

文艺,娱乐,游戏动漫
枣庄生活,殷秀梅,关牧村,谭晶,山东人,佟大为,陈思思,彭丽媛,学生一族,00 后
汽车
飞轮海,善良,音乐爱好者,运动，游戏，乐观
健康,娱乐,旅游,美食
时尚,文艺,星座命理,美食,娱乐
娱乐,健康,军事,IT 数码
星座命理,旅游
体育
娱乐,时尚,旅游,健康,教育就业,文艺
IT 数码,文艺,娱乐,美女,旅游
美女,健康,美食,旅游,游戏动漫
文艺,健康,体育,娱乐,旅游,星座命理,美食
韶关生活
新闻资讯,汽车,IT 数码,体育,娱乐
娱乐
美食,娱乐,白羊座,浪漫,乐观,欧美控,星座控,爱自由,执着,感性
随性,矛盾体,动漫控,90 后,喜欢音乐
游戏动漫,汽车,投资理财,旅游,体育
健康,IT 数码
教育就业,健康
美食,娱乐
娱乐,投资理财,健康,旅游,文艺
文艺,娱乐,新闻资讯,体育
猫咪控,摩羯座,喜欢音乐,80 后的尾巴,乐观,孩子气,单身,娱乐,旅游,美食
旅游,IT 数码,文艺
IT 数码,时尚
星座,看电影,听音乐,汽车,星座命理
健康,星座命理,游戏动漫,体育,娱乐
星座命理,娱乐
IT 数码,娱乐
星座命理,娱乐,文艺,新闻资讯,投资理财,体育,美食
教育就业
娱乐
军事
健康,新闻资讯,美女,IT 数码,文艺,体育,娱乐
时尚,美食
娱乐,汽车,体育,IT 数码,健康,星座命理
旅游,美食
娱乐,美食
旅游
投资理财,旅游,IT 数码,体育
娱乐
美食

穿衣美容
旅游
旅游摄影,天平座,文艺,时尚,投资理财,旅游
时尚
美食
时尚,教育就业,旅游,美食
时尚,健康,旅游,美食,娱乐
旅游,娱乐
穿衣美容,娱乐
星座命理,娱乐
投资理财,娱乐,美女,游戏动漫
美食
体育,游戏动漫,娱乐
美女,IT 数码,娱乐,投资理财,旅游,美食,汽车
健康
美食,星座命理,旅游,健康,文艺
旅游
健康,星座命理
新闻资讯,娱乐,教育就业,健康,旅游
娱乐
娱乐
游戏动漫
广东生活,星座命理
美食,健康,教育就业,文艺,旅游
体育,旅游
时尚
美女,偶喜欢玩,汽车,时尚,IT 数码
新闻资讯,投资理财,美女,旅游,文艺
军事,美食,汽车,游戏动漫,IT 数码
旅游,微博奇葩,搞笑幽默
星座命理,美食,新闻资讯,时尚
旅游,星座命理,时尚,美食,娱乐
投资理财,教育就业,军事,体育
星座命理,旅游,美食
穿衣美容,时尚
IT 数码
美女,美食,娱乐,游戏动漫
旅游
IT 数码
名人明星,视频音乐
旅游,美食,娱乐,体育
军事,旅游,时尚,美食,美女,体育
旅游
美女,星座命理,美食,旅游,美食,娱乐,体育

星座命理,福州生活,时尚
时尚
星座命理
新闻趣事,美食,茂名生活,读书分享,美图摄影,文艺
星座命理
军事,健康
娱乐,旅游,军事,文艺,体育
文艺
娱乐
汽车
体育
娱乐,美食
时尚,文艺,娱乐,美女,美食
美女
SHINee,爱猫,内向,旅遊,美食
汽车,体育,90後,爱旅游,苹果控,chock,白羊,暗恋 ing,好仔,足球
旅游,美女
低调,德国,天蝎座,林俊杰,美食控
时尚,星座命理,娱乐
健康,旅游,美食,娱乐,文艺
新闻资讯,教育就业
娱乐,文艺,新闻资讯,星座命理
娱乐,美食,时尚
美图摄影,娱乐,文艺
时尚,健康,美女,旅游,IT 数码,体育,娱乐
体育
美女,娱乐,汽车,教育就业,旅游,美食
旅游
星座命理,健康,文艺,时尚
美女,汽车,投资理财,IT 数码,娱乐
美女,星座命理
文艺,IT 数码,体育
美食,时尚,娱乐,星座命理,旅游
星座命理
体育,星座命理,娱乐
美女,美食
游戏动漫,美食,文艺,娱乐
健康,旅游,美食
健康,投资理财,新闻资讯,时尚,美食
文艺,时尚,美食,健康,娱乐
美食,娱乐
星座命理
新闻趣事,搞笑幽默,微博奇葩,星座命理,名人明星,时尚
星座命理,游戏动漫

穿衣美容
美食,娱乐
健康
美食,体育
娱乐
新闻资讯,投资理财,健康
90 後,美术,乐观,幻想,发呆,海贼王,听音乐,淘宝,我就是我,时事
旅游
娱乐
美女,娱乐
时尚,健康,旅游,文艺,娱乐
娱乐
美食
游戏动漫,IT 数码
健康,旅游
星座命理,美食
投资理财
搞笑幽默,旅游
健康,游戏动漫
娱乐
游戏动漫
文艺,时尚,旅游,健康
投资理财,时尚,健康,旅游,美食
美女
娱乐
旅游,英文,英语,励志,文艺
文艺,IT 数码,美食,旅游,娱乐
新闻趣事,视频音乐,星座命理,旅游,吉林生活,投资理财,汽车,时尚,IT 数码
娱乐,星座命理
娱乐,投资理财,时尚,健康,星座命理
时尚,美女
文艺,星座命理,美食
时尚,美食,文艺,健康
星座命理
美女,新闻资讯,娱乐,文艺,汽车,投资理财
天秤,娱乐
时尚,旅游,美食,投资理财,健康
旅游,文艺
爱睡觉,神经质
美女,健康,汽车
娱乐
军事,旅游,文艺,体育,娱乐
星座命理,旅游,健康,美女,娱乐
时尚,新闻资讯,IT 数码,旅游,教育就业

视频音乐,娱乐
美食,IT 数码
育儿亲子,育儿经验
健康,美食
美女,星座命理,时尚,体育,篮球爱好者
体育,IT 数码,游戏动漫
星座命理,文艺,旅游,娱乐
新闻趣事,新闻资讯
美食,游戏动漫,娱乐
文艺,娱乐
军事,体育
新闻资讯
美食,投资理财,旅游,IT 数码,文艺
娱乐,汽车,游戏动漫,军事,体育
IT 数码,健康,美女,教育就业,娱乐
旅游,新闻趣事,美食,情感生活,育儿百科
健康
旅游,健康
时尚,娱乐,80 後
新闻资讯,美女,IT 数码,时尚,汽车,教育就业,健康,体育,娱乐
游戏动漫
健康,旅游,军事,文艺,体育
健康,娱乐,星座命理,文艺
江苏生活,美食,星座命理,穿衣美容,搞笑幽默
娱乐,汽车
游戏动漫
搞笑幽默,汽车
美食,星座命理,娱乐
游戏动漫
娱乐
名人明星,娱乐
美食,IT 数码,军事
崇尚真善美,相信直觉,白羊座
美女,时尚,娱乐
投资理财,新闻资讯
文艺,健康,美食,旅游
投资理财,汽车,游戏动漫,IT 数码,体育
投资理财,文艺,娱乐,时尚,美女
体育
旅游,美女,体育
美女,时尚,娱乐
时尚,文艺,健康,旅游,美食
名人明星,娱乐
游戏动漫

文艺,星座命理,健康,旅游,美食
穿衣美容,娱乐,搞笑
达酥控,漫画达人,内向,DOTA 爱好者,涂鸦绘画,漫画,少时控,射手座 O 型血,90 后学生
星座命理,游戏动漫
星座命理,美食,体育,投资理财,汽车
美女,IT 数码,旅游,星座命理,体育,文艺
IT 数码,健康,旅游,娱乐,教育就业
娱乐,投资理财,时尚,旅游,文艺
新闻趣事,教育就业,汽车
美女,娱乐,时尚,游戏动漫,星座命理
时尚,美女,美食,娱乐
旅游,美女,军事,时尚
美食
粉红控,志愿者,赚钱,做梦,SuperJunior,听音乐,发呆,爱音乐,美食,娱乐
星座命理,游戏动漫,投资理财,IT 数码
健康,旅游,星座命理,美食,娱乐
时尚,新闻资讯,旅游,美食,娱乐
文艺
music,萌宠
人生,时事,软件,唱歌,幽默,投资理财,娱乐,教育就业,新闻资讯,军事
IT 数码,文艺,娱乐
时尚,投资理财,美食,旅游
时尚
美女,健康,时尚,教育就业,旅游
旅游,娱乐,美食,文艺,投资理财
时尚,星座命理,美食,娱乐,游戏动漫
美食
体育
o 型血,美剧控,韩剧,狮子女,宅女一枚,IT 数码,文艺,新闻资讯,旅游,美食
星座命理,美食,美女,游戏动漫,投资理财
时尚
健康,教育就业,旅游,美食,娱乐
热爱生活,美食爱好者,投资理财,健康,旅游
文艺,美食
美食,IT 数码
教育就业,新闻资讯,军事,旅游,体育
星座命理,美食,游戏动漫
娱乐,星座命理
健康,娱乐,时尚
健康,投资理财,星座命理,美食,娱乐
军事,健康,娱乐
新闻资讯,时尚,投资理财,健康,娱乐
登山,喜欢旅游,IT 数码,健康
投资理财,教育就业,新闻资讯,旅游,体育

文艺
新闻资讯
美食,看电影,IT 数码,军事,新闻资讯,娱乐,旅游
矛盾体,处女座,90 后但我不脑残,搞笑,乐观,吃货,星座命理,旅游,时尚,美食
美女,旅游,健康,时尚,娱乐
健康,电影,胡思乱想,文艺,娱乐,旅游,美食
投資理財
汽车,体育,娱乐,时尚,美女
新闻资讯,健康,旅游,美食,文艺
娱乐,旅游
星座命理,美食,旅游,娱乐,文艺
汽车,军事,娱乐
德语,翻译,留学生,新闻资讯
90 後,爱笑,孩子气,学生一族,文艺
IT 数码,汽车,游戏动漫,时尚
体育
旅游,教育就业
美食
听音乐,美食,学生,动漫,娱乐,睡觉,IT 数码
体育
旅游
90 后,交友,旅游
热爱运动,包包,眼睛,服装,运动鞋,广告,投资理财,化妆品,体育
潮流,网购,时装,服饰,购物,完美主义,女人,奋斗,欧美控,时尚
体育
美女,喜欢唱歌,互联网,吃,健康,体育,娱乐,教育就业,投资理财,旅游
美食,文艺,体育,娱乐
美女,娱乐,时尚
文艺,体育,美食,游戏动漫
游戏动漫,体育,军事
体育
美食
学生一族,柯南,开心,喜欢音乐,游戏动漫
体育
爱音乐
体育资讯,名人明星
文艺,旅游
体育,汽车
时尚
美食,热爱生活,快乐,自由,双子
娱乐
时尚,旅游,美食,IT 数码,娱乐
投资理财,娱乐,IT 数码
旅游

新闻资讯,投资理财,教育就业,健康,旅游,文艺
时尚,旅游,文艺,美食
美食
文艺,娱乐
教育就业
旅游,军事
星座命理
旅游
星座命理,娱乐
美食
健康,娱乐
美女
美女
美食,投资理财,时尚,健康,文艺
软件,交朋友,o 型血,建筑,新闻资讯,星座命理,汽车,旅游,娱乐,IT 数码
娱乐,旅游,新闻资讯,美女,游戏动漫,体育
娱乐,健康,旅游,时尚,美女,文艺
文藝
时尚,娱乐,星座命理
坚强,笑,傻,胡思乱想,爱自由,感性多于理性,獨立,80 後,水瓶座 B 型血,时尚
服装商标,服装鞋帽,25 类商标转让,25 类商标,品牌转让,品牌,商标设计,商标注册,商标转让,商标
新闻资讯,旅游,星座命理,美食,娱乐
娱乐,文艺
美食
文艺,体育,旅游,汽车,军事
旅游
星座命理,运动狂人,十六中二班,执信人,摩羯座 O 型,自然,钢琴,摄影,旅游
美图摄影,宠物图集,新闻趣事
美食,旅游
IT 数码,娱乐
旅游,时尚,文艺,cosplay,舞蹈,绘画,美食,游戏动漫
美图摄影,二货神经质怪胎
美女,旅游,IT 数码,娱乐
投资理财,美女,新闻资讯,IT 数码
旅游,萌宠,新闻趣事,美女,广东生活,o 型血,亲情,羽毛球
新闻资讯,美女,投资理财
军事,旅游,健康,美女,汽车,体育,IT 数码
宅,旅游,听歌,90 后,星座命理,美食,IT 数码,文艺,娱乐
美食,娛樂
旅游
上海生活,美食
旅游
游戏动漫

新闻资讯,旅游,体育
美食,健康,美女,娱乐,星座命理
健康
美食,体育
健康,美食,娱乐,新闻资讯
游戏动漫,教育就业,健康
旅游,新闻资讯,健康,美女,美食
时尚,旅游,星座命理,美食,娱乐
美食,旅游,美食,IT 数码
美食,文艺,投资理财,时尚,健康
旅游,时尚
睡觉,狗狗,美食
印章,玉石古玩,陶瓷艺术,字画收藏,古董收藏
文艺,新闻资讯
娱乐,投资理财,旅游,时尚,教育就业
娱乐
睡懒觉,学生一枚,管理,猫,健康,游戏动漫,娱乐,星座命理,文艺
摩羯,80 後,休闲,杭州,旅游,星座命理
爱,书店,书
美食,旅游,体育
投资理财,教育就业,旅游,健康,文艺
文艺
健康,美女
星座命理,恐怖片,吸血鬼,美食爱好者,时尚达人,热爱生活,淡定,娱乐
旅游,教育就业
娱乐
星座运势,校园生活,视频音乐,青春,旅游美食,偶像剧,媒体人,看电影,90 後,听音乐
游戏动漫,美食,体育,娱乐
旅游,投资理财,健康,娱乐
技术宅,无限犯困,贱贱水瓶座,长不胖的吃货,坚挺是我得本性,伪文艺青年,怪蜀黍,美食,时尚
宠物图集,新闻资讯,时尚,美食,健康
文艺,IT 数码,旅游,健康,美食
游戏动漫,娱乐
军事,旅游,体育
美食,hiphop,乐观
美食
教育就业,军事,体育
雅思,留学,英国,新加坡,澳洲,澳大利亚,马来西亚,新西兰,新闻资讯,IT 数码
视频音乐,旅游,名人明星,穿衣美容,时尚,美食
吃货
教育就业,IT 数码
旅游
视频音乐

健康,旅游,时尚,娱乐,体育
游戏动漫
投资理财,娱乐
新闻趣事,体育
游戏动漫,美女,美食
投资理财,军事,美女,文艺,美食
星座命理,美食,体育,美女,游戏动漫
美女,美食,军事,IT 数码,文艺
旅游,美食
新闻资讯,汽车,游戏动漫,军事,IT 数码,文艺,体育
体育
文艺
搞笑幽默,郑州生活,教育就业,文艺,体育
星座命理,体育,时尚,文艺,娱乐
投资理财,健康,旅游,时尚,娱乐
健康,美食,娱乐
星座命理,健康,美女,美食,娱乐
美女,军事,体育,娱乐
穿衣美容,健康,时尚
汽车,星座命理,军事,IT 数码,娱乐
健康,新闻资讯,军事
美食,旅游
新闻资讯
汽车,投资理财,旅游,IT 数码
旅游,星座命理,时尚,娱乐,体育
美食,娱乐,体育,文艺
交朋友,旅游美食爱好者,旅游摄影,IT 互联网,爱电影,爱旅游,美食,新闻资讯,美女,时尚
视频音乐,体育
微博奇葩,时尚,文艺,娱乐
爱情,好性格,投资理财,旅游,美食,体育,娱乐
旅游,教育就业,健康,军事,娱乐
健康,时尚,旅游,美食,娱乐
新闻资讯,文艺,教育就业,时尚,星座命理,旅游,美食,娱乐
美食,体育,娱乐
IT 数码,娱乐,时尚
教育就业,美食,星座命理,健康,时尚,IT 数码
新闻资讯,军事,教育就业,IT 数码
时尚
美女,汽车,体育
新闻资讯,旅游,教育就业
旅游,健康,投资理财,时尚,娱乐
汽车,文艺,娱乐,投资理财
旅游,美食
游戏动漫

美食
美食,美女,汽车,军事
娱乐
旅游,星座,文艺,美容,逛街,娱乐
娱乐,体育,美女,新闻资讯,健康
新闻资讯
旅游,星座命理,美食,文艺,娱乐
时尚,军事,旅游
dj
时尚,旅游,星座命理,美食,娱乐
汽车,美食,美女
新闻资讯,汽车,军事,IT 数码
游戏动漫,时尚,旅游
SHU,彈琴不談情,終極,異能行者,聯盟,功夫,首席,爵士,音樂,ROCKER
娱乐,汽车,投资理财,时尚,健康,旅游
旅游,健康,投资理财,星座命理,文艺
美女,军事
矛盾体，强迫症,独一无二，吉他,真诚,交朋友
美食
娱乐
美食,星座命理
IT 数码
旅游,热爱自由,爱听爱唱爱跳,流浪、
时尚,旅游,新闻资讯,健康,星座命理,美食,娱乐
汽车,美食
汽车,旅游,美食,IT 数码,娱乐
美食
视频音乐,八卦杂谈,搞笑幽默,校园生活,美食
美女,时尚,星座命理
投资理财,娱乐,健康,军事,IT 数码
校园生活,情感生活,常州生活,美食,江苏生活
旅游
投资理财,旅游,星座命理,汽车,美食
科比,周杰伦
娱乐
娱乐,美女,文艺,旅游,美食
旅游,美食,娱乐,玩儿,爱旅游,爱电影,音樂,90 后,双子座,吃
娱乐
时尚,旅游,新闻资讯,文艺,娱乐
健康,体育,游戏动漫
投资理财
汽车
二次元汉纸嫁我,动漫宅久矣,强迫症很严重,学医的倒霉孩子,声控没得救了,女王傲娇控
文艺,体育

美女,健康,新闻资讯,旅游,娱乐
新闻资讯,健康,时尚,投资理财,文艺
时尚,旅游,美食,健康,投资理财
文艺,美食,星座命理,健康,旅游
健康,时尚,星座命理,美食,文艺
吉他
娱乐,投资理财,军事,IT 数码,美女
文艺,健康,新闻资讯,游戏动漫,星座命理
美食,旅游,娱乐
星座命理,军事,汽车,时尚,娱乐
美女,时尚,健康,旅游,美食
健康,美食
时尚
新闻资讯,美食,星座命理,游戏动漫,体育
视频音乐,新闻趣事,惠州生活,搞笑幽默,新闻资讯,健康,美女,IT 数码,游戏动漫
文艺,娱乐,健康,时尚,美食,星座命理
美食,娱乐
完美主义,爱吃,经济,服装,哈哈,我就是我,旅游美食爱好者,武术,韩国,温柔
投资理财,旅游,美食,文艺,娱乐
时尚,投资理财,文艺,美食,娱乐,天秤座,文艺性宅女一只,理财
美女
汽车,美食,IT 数码,文艺,体育,k 歌
新闻资讯,旅游,军事,IT 数码,体育
时尚,美食
文艺,美食,时尚
时尚,游戏动漫,美女,娱乐
娱乐,体育,文艺,旅游
旅游,新闻资讯,军事,体育
健康
文艺,健康,旅游
新闻资讯,文艺,IT 数码,军事,美食,旅游,健康,美女,体育
旅游,美食,投资理财,娱乐
旅游
教育就业
美女,娱乐
健康,游戏动漫,星座命理,美食,汽车
汽车
美食
投资理财,文艺,游戏动漫,体育
游戏动漫,房产,快乐大本营,火影,看书,Android,80 后,wow,军事,IT 数码
美女,旅游,文艺,新闻资讯,星座命理,健康,投资理财
IT 数码,新闻趣事,八卦杂谈,美女,台州生活,游戏动漫
星座命理,美女
健康

星座命理
文艺,星座命理,美女,娱乐,军事,旅游
旅游,新闻资讯,汽车,军事,体育
健康
星座命理,旅游,健康
nba,金庸迷
旅游
娱乐
双鱼
文艺,IT 数码,美食
时尚,旅游,星座命理,美食,娱乐
时尚,旅游,美女,健康,娱乐
新闻资讯,健康,投资理财,IT 数码
美女
游戏动漫,IT 数码,体育,文艺,旅游
其他生活,搞笑幽默,美食,萌宠,穿衣美容
美食,时尚,旅游,健康,新闻资讯
汽车,星座命理,IT 数码,军事,娱乐
星座命理,旅游,时尚
体育
汽车,体育
85 后,时尚,娱乐
新闻资讯,旅游,健康,投资理财,军事,体育
娱乐
海归,珠宝,多米尼加,琥珀,钻石,宝石,海纹石
体育
游戏动漫,汽车,美女,体育,娱乐
娱乐,游戏动漫
陈翔,美食控,偶喜欢玩,武艺,自拍,听音乐,双子女,爱自由,爬山,爱旅游
文艺,旅游
黑龙江生活
教育就业,文艺,美食,健康
搞笑幽默,时尚,文艺
教育就业,美食,娱乐,文艺,体育
文艺,美食
力挺晨晨新专,魏晨控,乐橙一枚
文艺,娱乐,游戏动漫
旅游
美女,新闻资讯,娱乐,IT 数码
珠海生活,文艺,懒,学生一枚,玩儿
时尚,教育就业,体育
新闻资讯,星座命理,美食
美食,时尚
健康,新闻资讯,时尚,旅游,娱乐

旅游,美食,文艺
娱乐,体育,IT 数码,游戏动漫,美女
娱乐,健康
IT 数码
韩剧,微博控,90 后,喜欢听歌,时尚,旅游,文艺,美食,娱乐
新闻趣事,沈阳生活,新闻资讯,汽车,投资理财,时尚,健康,旅游
娱乐,旅游,美食
新闻资讯,IT 数码,投资理财,体育,保险,理赔,服务,健康,旅游,汽车
星座命理,时尚,娱乐
IT 数码,娱乐
汽车,游戏动漫,旅游,娱乐,体育
搞笑幽默,美女,军事,时尚,IT 数码,文艺
旅游
娱乐
文艺,美食,美女,时尚,娱乐
游戏动漫,投资理财,旅游,军事,体育
美女,游戏动漫,汽车,新闻资讯,健康
汽车,健康,旅游
时尚,文艺,旅游,健康,美食
情感生活,新闻趣事,美食,南昌生活,旅游
文艺,妖精
美女,军事,投资理财,星座命理,旅游,汽车,IT 数码
美女
体育
文艺,娱乐
视频音乐,新闻趣事,微博奇葩,陕西生活,文艺,投资理财,时尚,新闻资讯,旅游
校园生活,教育就业
体育
美食,汽车,时尚,游戏动漫,体育
情感生活,星座命理,IT 数码,时尚
汽车
美食,娱乐,星座命理
教育就业
星座运势,健康,新闻资讯,时尚,星座命理,娱乐
游戏动漫,投资理财,星座命理,军事,IT 数码
娱乐,时尚,汽车,投资理财,旅游
汽车,IT 数码
健康,投资理财,教育就业,汽车,军事
美食,星座命理,文艺,娱乐,体育
健康
乐队,Underground,Alternative,IndieMusic,lofi,摇滚,RocknRoll
军事
体育,教育就业,时尚,汽车
旅游

投资理财,健康,旅游,美食,星座命理,娱乐
文艺,新闻资讯,教育就业
汽车
娱乐,健康,美食,时尚,文艺
旅游,微博奇葩,星座命理,搞笑幽默,四川生活,健康,新闻资讯,时尚,文艺
健康,文艺,美食,旅游,星座命理
时尚,旅游,健康,星座命理,美食
健康
IT 数码
旅游
搞笑幽默,微博奇葩,萌宠,旅游,重庆生活
名人明星,甘孜生活,时尚
旅游,体育
时尚,旅游,IT 数码,美食,投资理财,汽车,军事
文艺
旅游,美食,美女,时尚,娱乐,音乐爱好者,单身,寂寞,90 後,林俊杰
美女,时尚
搞笑幽默,新闻资讯,文艺,投资理财,娱乐
旅游,文艺,美食,美女,时尚
教育就业,汽车
汽车,IT 数码,军事,时尚,新闻资讯
投资理财,旅游
商业,男人,互粉,活泼,电影控,旅游美食,music,体育
广州医药,广州药业,广药集团
文艺,美食,新闻资讯,健康,旅游,军事
懒人,美食,海贼王
美女,旅游,美食
星座命理,时尚,新闻资讯,文艺,娱乐
健康,美女,投资理财
美女,汽车,体育,ELF,旅游
新闻趣事,星座命理,名人明星,旅游,IT 数码,新闻资讯,健康,投资理财,美食
时尚,旅游,星座命理
搞笑幽默,微博奇葩
时尚,美食
健康,时尚,旅游,星座命理,美食
美食
美女,娱乐
体育
文艺,游戏动漫,旅游,娱乐,天龙八部,各种宅,80 后
健康,汽车,旅游,美食,娱乐,投资理财
美食,旅游,穿衣美容,名人明星,搞笑幽默
时尚,文艺,新闻资讯
萌宠,健康,旅游,星座命理,美食,军事
旅游,美食,教育就业,娱乐,文艺,IT 数码,游戏动漫

lol,游戏动漫
美食
体育,星座命理,游戏动漫
搞笑幽默,娱乐
搞笑幽默,娱乐
旅游,美女,美食
时尚
新闻资讯,IT 数码,美女,游戏动漫
新闻资讯,旅游,时尚,美女,娱乐,汽车
游戏动漫,新闻资讯,旅游,美食,IT 数码
汽车
旅游,校园生活,IT 数码,美图摄影
IT 数码,健康,时尚,军事,旅游,汽车
健康
旅游
美食
石家庄生活,星座命理,新闻趣事,旅游,健康,投资理财,军事
江苏生活,体育
投资理财,时尚,健康,旅游,逛街,哈哈,交朋友,我就是我,开朗,热情
美食,汽车
美女,娱乐
健康,投资理财,军事,美食,新闻资讯,旅游
美食,健康,新闻资讯,旅游,IT 数码
时尚,健康,星座命理,美食,文艺,娱乐
娱乐,新闻资讯,美食,时尚
巨蟹,双重性格,80 后的尾巴,手机,时尚
新闻趣事,懒,玩
IT 数码,旅游,健康,时尚,美食
穿衣美容,贵州生活,发呆,听音乐,旅游,美食,文艺,游戏动漫,时尚
浙江生活
星座命理
健康,旅游,文艺,体育,新闻资讯,投资理财
旅游,游戏动漫
星座命理,微博奇葩,萌宠,美女,搞笑幽默
IT 数码,娱乐
星座命理,健康,教育就业
娱乐,新闻资讯,文艺,IT 数码
其他生活
投资理财,教育就业,健康,旅游,星座命理
教育就业,新闻资讯,投资理财
旅游爱好者,外贸,篮球,摄影,高尔夫,汽车,时尚,投资理财,体育
体育
教育就业,投资理财,旅游,美食,IT 数码
娱乐

美女,星座命理,美食,军事,娱乐,体育
美食,健康,时尚
IT 数码,文艺,新闻资讯,旅游,美食
游戏动漫,旅游,军事,IT 数码,娱乐
时尚,健康,旅游,娱乐
星座命理
文艺
美食
旅游,美食,星座命理,时尚,娱乐
娱乐,汽车,游戏动漫,体育
广州生活,新闻趣事,搞笑幽默
体育,投资理财,汽车,旅游,军事
IT 数码,游戏动漫
鄂尔多斯生活
游戏动漫,文艺,美食
体育,游戏动漫
汽车
投资理财,娱乐,军事
交朋友,喜欢听歌,bigbang,吃货一枚,笑,hiphop,美食
美食,旅游,健康,时尚,购物狂,耽美,爱吃,化妆品,朋友,神经大条
游戏动漫
星座命理
视频音乐,美女,幻想,生活,爱音乐,摄影师,金融,旅游,时尚,文艺
旅游,体育,美食
美女,军事,健康
新闻资讯
美食,文艺
游戏动漫
文艺,汽车,旅游,军事,体育
游戏动漫,娱乐,文艺,IT 数码,美食
好性格,喜欢听歌,娱乐
健康
旅游
旅游,时尚,星座命理,美食,娱乐
星座命理
文艺
娱乐
旅游
美食
健康,旅游,美女,时尚
健康,新闻资讯,教育就业,军事,娱乐
时尚,旅游,美食,娱乐
游戏动漫,IT 数码,军事
旅游,爱唱歌,喜欢音乐,休闲,会计,歌手,烹饪,体育

学生,美国,留学,求职,工作
娱乐,旅游,健康,美食,时尚,星座命理
旅游,娱乐,时尚
游戏动漫,美食,军事,体育,娱乐,汽车
教育就业,新闻资讯,娱乐,美食
投资理财
健康,新闻资讯,投资理财,旅游,文艺
电影爱好者,聊天,音樂,朋友,k 歌,Music
汽车,投资理财,体育,娱乐,时尚
教育就业,新闻资讯
娱乐
旅游,时尚
美女,教育就业,娱乐,时尚,美食
足球迷,电视剧,健身,美食控,商业
新闻资讯,旅游,星座命理,美食,娱乐
新闻资讯,体育,健康,美食,旅游,娱乐
美女,汽车,时尚,游戏动漫,娱乐
健康,新闻资讯,教育就业,投资理财,军事
IT 数码
爱着自己的傻逼,执着的
教育就业,时尚,IT 数码,文艺,娱乐
旅游,娱乐,IT 数码,文艺,体育
时尚,旅游,美食,娱乐
美食,时尚,娱乐
美食
文艺,汽车
美女,美食,娱乐
旅游
星座命理,时尚
健康,时尚,美食
zymk,龙族,学习,奋斗,爱幻想,无聊,游戏动漫
视频音乐,时尚,美食,体育,娱乐
旅游
IT 数码
游戏动漫
时尚
旅游
旅游,军事,新闻资讯,健康,IT 数码,娱乐
健康,星座命理,投资理财,旅游
美食,旅游,健康,娱乐,体育
美食,体育
娱乐,新闻资讯,健康,旅游,美食控,快乐,懒,安静
穿衣美容,教育就业,时尚,投资理财
视频音乐

美食,健康,时尚,星座命理,投资理财,旅游,美食,娱乐
喜欢唱歌,小说,唱歌,听歌,90 后,娱乐
健康,新闻资讯,教育就业,IT 数码,时尚
80 后,旅行,美食,军事,时尚,旅游,娱乐
旅游
汽车,旅游,IT 数码,娱乐,文艺,体育
美女,体育,娱乐,文艺,汽车
美食,娱乐
美女,美食,IT 数码,文艺,娱乐
娱乐,健康,美食,新闻资讯,时尚
体育,IT 数码,旅游,汽车,健康,文艺
爱旅游,懒,微笑,90 后但我不脑残,发呆,旅游,美食
美食
名人明星,穿衣美容,娱乐
美女,新闻资讯,健康,军事,体育
美食,文艺,健康,旅游,星座命理,天蝎女
文艺,教育就业,健康,时尚,娱乐
IT 数码,文艺,汽车,教育就业,投资理财
时尚,美食,文艺,电影,娱乐
娱乐
旅游,文艺
汽车,军事,IT 数码,健康,投资理财,新闻资讯
大一，自恋。爱玩
文艺,时尚,健康,新闻资讯,军事
美食
搞笑幽默,游戏动漫,军事,体育
汽车,IT 数码
娱乐,体育
美食,娱乐,时尚,星座命理,旅游,美食
娱乐
教育就业,IT 数码,健康,投资理财,体育,游戏动漫,星座命理,娱乐
V 迷,吃货,嵩控,文艺,娱乐,游戏动漫,体育
美食
汽车,健康,时尚,旅游,美食
旅游,汽车,教育就业,投资理财,文艺
游戏动漫
时尚,健康,旅游,星座命理,娱乐
旅游,美食
双鱼座,摄影,旅行,强迫症
投资理财,体育
爱生活,IT 数码,汽车,娱乐,美食,投资理财
电影控,绘画,宅,摄影
汽车,美女,健康,IT 数码,体育
军事,IT 数码,体育

健康,时尚,旅游,星座命理,娱乐
新闻资讯,健康,体育,投资理财
桌球,生活,艺术,搞笑,美女
淡定,天秤座,90 後,音乐达人,大学生,音乐爱好者,文艺,体育
娱乐,IT 数码,投资理财,旅游,时尚
汽车
视频音乐,星座命理,娱乐
美食,时尚,娱乐,星座命理
旅游
娱乐
旅游
文藝
教育就业,文艺
军事
旅游,文艺
名人明星,娱乐
摄影控,学生一族,管理,古典艺术,漫画,军事,诗词,美术,互粉,文艺
教育就业,美食,娱乐
教育就业,星座命理,娱乐,健康,旅游
IT 数码,美女,娱乐
八卦杂谈,搞笑幽默,美食,游戏动漫,娱乐
管理
美女,体育
健身,快乐,爱笑,潮,爱生活,偶像剧,潮流,音乐控,笑话,时尚
美女
时尚,IT 数码,文艺,娱乐,旅游,体育
真诚,青春
灯座,宠物鸟,洞桥八戒西瓜,节能灯,led,速卖通,拍拍,淘宝,魔域
旅游,汽车
美食,健康,投资理财,娱乐,文艺
星座命理
美食
健康
喜欢听歌,旅行,摄影,志愿者,爱唱歌,善良,军事,娱乐,健康,文艺
旅游
体育
娱乐,游戏动漫,军事,IT 数码,体育
文艺,美食,健康,完美主义,奋斗,学习,水瓶座,神经大条,懒人,A 型血
体育
名人明星
总经理,董事长,经济管理,房地产,定制家私品牌,家装趋势,家具设计与制造,办公家具,定制家具,企业管理
健康,投资理财
IT 数码,游戏动漫,军事

旅游
投资理财
军事,文艺,娱乐,美食,旅游,汽车,IT 数码,体育
旅游,投资理财,汽车,IT 数码,新闻资讯
汽车,旅游
搞笑幽默,娱乐
体育,美食,星座命理,游戏动漫,汽车
娱乐
游戏动漫
娱乐,美食,投资理财
投资理财,体育,新闻资讯,星座命理,军事
娱乐
旅游,文艺
军事,旅游,汽车,体育
军事,美女,上网,唱歌,睡觉,自由,时尚,旅行,旅游,娱乐
星座命理
健康,时尚,旅游,美食,娱乐
美食,健康,新闻资讯,时尚,投资理财,文艺
娱乐,旅游,美食
星座命理,健康,文艺
新闻趣事,娱乐,新闻资讯,教育就业
娱乐,星座命理,旅游
娱乐
星座命理
汽车,教育就业,健康,旅游,美食
美女,投资理财
时尚
军事,健康,星座命理,新闻资讯
时尚,新闻资讯,美食,健康,投资理财
娱乐
健康,美女,汽车
博客,海贼王,耽美,白羊座,80 后,les,写作即人生,双性恋,旅游,游戏动漫
美女,娱乐,体育
教育就业
娱乐
健康,汽车,旅游,IT 数码,体育
汽车,时尚
美食
健康,教育就业,汽车,体育,军事
美食,穿衣美容
游戏动漫
教育就业,时尚,美食,文艺,娱乐
美食,体育,IT 数码,旅游,星座命理,军事
搞笑幽默,微博奇葩,旅游,萌宠,星座命理,游戏动漫,文艺

搞笑幽默,情感生活,八卦杂谈,校园生活,IT 数码
旅游,汽车,体育
娱乐,k 歌
体育,旅游,IT 数码,汽车
旅游,娱乐,时尚,IT 数码,美女
娱乐
汽车
体育,美食,娱乐,教育就业,星座命理
新闻资讯,汽车
汽车,美女
娱乐
健康,时尚,旅游,星座命理,娱乐
旅游,名人明星,投资理财
娱乐
穿衣美容,星座命理,微博奇葩,搞笑幽默,上海生活
新闻资讯,旅游,时尚,美食,娱乐
文艺,美食,旅游,星座命理,时尚,娱乐
娱乐
时尚,旅游,美食,文艺
搞笑幽默,投资理财
美食
健康,新闻资讯,娱乐,时尚,美女
星座命理,旅游,文艺,时尚,娱乐
时尚,娱乐,旅游
健康
新闻资讯,旅游,美食,IT 数码,汽车
新闻资讯
娱乐,时尚,健康,旅游,美食,投资理财
时尚,汽车,IT 数码,旅游,娱乐
美女,汽车,旅游
文艺,看电影,淘宝,投资理财,健康,新闻资讯,教育就业,美食
IT 数码,游戏动漫
新闻资讯,健康,投资理财,旅游,美食
健康,文艺
读书分享,新闻趣事,视频音乐,校园生活,新闻资讯,娱乐,IT 数码
旅游,大叔控
校园生活,名人明星,美图摄影,情感生活,星座运势,教育就业
聊天,旅游,星座命理,文艺,娱乐
健康,IT 数码,时尚,美食,娱乐
文艺,娱乐,军事
娱乐
IT 数码
健康,旅游,美女,时尚,娱乐
看电影,爱音乐,爱唱歌,狗狗,瘦身,蓝色,学生一族,电影迷,健康

美食
旅游,军事,娱乐,汽车
健康,游戏动漫,娱乐,文艺,新闻资讯
美食,文艺,音樂,睡覺,阅读,电影,旅行
时尚,健康,美食,文艺
文艺,美食,健康,旅游
教育就业
投资理财,时尚,健康,旅游,娱乐,新闻,紫色,美女
星座命理,游戏动漫,旅游,文艺,美食
浙江生活
旅游,新闻资讯,军事,投资理财,汽车
体育
教育就业,新闻资讯,健康,美食,IT 数码,体育,娱乐
时尚,新闻资讯,健康,美食,旅游,文艺
美食,旅游,健康,时尚,文艺,娱乐
时尚,投资理财,旅游,美女,汽车
文字,设计,巴黎,摄影,钢琴,宅女加腐女,爱吃,完美主义,电影控,美国
娱乐,旅游
娱乐
名人明星,视频音乐,时尚,文艺,娱乐,旅游
新闻资讯,汽车,体育,投资理财,军事
军事,美女
健康,汽车,新闻资讯,时尚,IT 数码
美女
美食,美食
IT 数码,体育,汽车,教育就业,美女
旅游,体育,新闻资讯,投资理财,汽车
文艺,时尚,美食,旅游,星座命理
汽车,教育就业,体育,娱乐,时尚,文艺
旅游,投资理财
旅游,教育就业,娱乐
爱猫,淘宝,爱生活,文艺
娱乐,教育就业,旅游,健康,时尚
美女,文艺,健康,旅游,教育就业
旅游
娱乐,时尚,教育就业,投资理财,美食
娱乐,投资理财,时尚,健康,美食
美食,军事,健康
搞笑幽默,遵义生活,名人明星,美女,军事,健康,文艺
星座命理,汽车,时尚,IT 数码
时尚,美食
IT 数码
健康
体育

美食,高三,学生,玩
旅游,娱乐,教育就业
投资理财
旅游,美食,娱乐
旅游,娱乐
健康,文艺,娱乐
美女,健康
旅游,文艺,星座命理,美食,娱乐
校园生活,搞笑幽默,八卦杂谈,美女,文艺,美食,游戏动漫
星座命理,旅游,时尚,娱乐
旅游,游戏动漫,美女,娱乐,新闻资讯,健康,体育
旅游,体育
投资理财,旅游,汽车,IT 数码
穿衣美容,新闻趣事
游戏动漫,星座命理,美食
旅游,新闻资讯,教育就业
旅游
娱乐,体育
CL
奋斗,养生,90 後,自由,上网,学生,宅,旅游,狗狗,平常心
美食,时尚,旅游
健康,时尚,投资理财,美食,娱乐
美食
名人明星,美食,新闻资讯,教育就业,旅游,投资理财,体育
军事,新闻资讯
美食
娱乐,文艺,时尚,旅游
旅游,娱乐,体育,军事,IT 数码
爱唱歌丶,向日葵丶,小花痴丶,高中生丶,爱香橙丶,爱猫咪丶,微博控丶,音乐控丶,文字控丶,Jay 迷丶
旅游,美食,娱乐,时尚,投资理财
汽车,游戏动漫,旅游,美食,IT 数码
安徽生活,投资理财,新闻资讯
时尚,旅游,娱乐
旅游
军事,新闻资讯,IT 数码
旅游,投资理财
美食
美食,健康
美食
文艺
汽车,体育
校园生活,星座命理
名人明星,萌宠,微博奇葩,视频音乐,搞笑幽默,娱乐

教育就业,时尚,娱乐,文艺,美食
汽车,旅游
K 歌,90,美食,旅游,体育,IT 数码,娱乐
军事,汽车,旅游
投资理财,健康,时尚,IT 数码,星座命理
教育就业,新闻资讯,汽车,健康,旅游
娱乐,旅游
游戏动漫,汽车,娱乐
文艺,娱乐
成都生活,时尚,体育,IT 数码,旅游,美食
健康,新闻资讯,美食,娱乐,时尚,教育就业
游戏动漫,旅游,美食
体育资讯,大连生活,搞笑幽默,IT 数码,游戏动漫,旅游,军事,体育
美女
旅游,美食
美食,时尚,娱乐,文艺,投资理财
美食,感性
娱乐,IT 数码
网球,投资理财,军事
旅游
汽车,健康,游戏动漫,美食
旅游,新闻趣事,搞笑幽默,山东生活,游戏动漫,时尚,美女,娱乐,体育
秦砖汉瓦
教育就业,时尚,旅游,健康,文艺,娱乐
时尚,投资理财,旅游
时尚,体育,文艺,健康,旅游
文艺,健康,旅游
美容服饰,新闻趣事,文艺
旅游,健康,新闻资讯,文艺,教育就业
穿衣美容,孤独主义,森系,冰淇淋,软妹子,原宿风
音樂,狮子女,娱乐,美食,旅游,游戏动漫,IT 数码
穿衣美容
新闻资讯,汽车,IT 数码,美女,教育就业,文艺
娱乐,旅游,健康,游戏动漫
时尚,新闻资讯,健康,美食,旅游
时尚,宅女一枚
名人明星,旅游,美食,视频音乐,搞笑幽默
健康,旅游,星座命理,投资理财
IT 数码
淘宝,爱自由,个性,青春,新闻,文字,时尚,旅游,美食,娱乐
娱乐,美食,新闻资讯,文艺,体育
旅游,教育就业,游戏动漫,美女,娱乐
教育就业,投资理财,军事,时尚,文艺
自由,喜欢唱歌,歌手,喜欢旅游,淡定,热爱生活,k 歌,music,音乐迷,文艺

美食,星座命理,旅游,健康,娱乐
投资理财,文艺,娱乐
军事,美食,游戏动漫,体育
文艺,新闻资讯
教育就业,美食,健康,旅游,新闻资讯
游戏动漫
美女
娱乐,军事
美食
新闻趣事,视频音乐,搞笑幽默,星座命理,旅游,时尚,体育,汽车
美女,时尚,汽车,体育,娱乐
教育就业
旅游,时尚,美食,IT 数码,娱乐
娱乐
娱乐,文艺,美食,教育就业,投资理财
旅游,时尚,星座命理,美食,娱乐
时尚,汽车,旅游,IT 数码,文艺
汽车,美女,美食,健康,旅游
新闻资讯
时尚,健康,旅游,美食,文艺
娱乐,旅游
旅游,星座命理
美食
穿衣美容,文艺,游戏动漫
健康,汽车,军事,旅游,娱乐
娱乐
新闻资讯,游戏动漫,军事,教育就业
娱乐
视频音乐,美女
教育就业,旅游,健康,文艺,体育
电影迷,白羊女
旅游
名人明星,张翰
K 歌,k 歌
文艺,旅游
珠海生活,巨蟹,90 后
健康,体育
健康
娱乐,IT 数码,旅游,美食,时尚
美食,军事,汽车,IT 数码,娱乐
时尚,新闻资讯,旅游,投资理财,美食
阿根廷
白羊座 A 型血
文艺,体育,娱乐

文艺, 娱乐
美食
文艺, 体育, 娱乐
张翰, 90 后但我不脑残, 听音乐, 爱音乐, 喜欢唱歌, 娱乐
星座运势, 杰娜控, 微博控, 幸福, 巨蟹女, 学生星
心内科, 文艺
时尚, 投资理财, 新闻资讯, 健康, 旅游
文艺, 体育, 娱乐
爱旅行, 企业家
教育就业, IT 数码, 旅游, 星座命理, 娱乐
美女
投资理财, 新闻资讯, 汽车, 娱乐, 体育
文艺, 星座命理, 美食, 旅游, IT 数码
旅游, 健康, 体育
体育, 美女
绵阳生活, 新闻资讯
汽车, 星座命理
美食, 汽车, 游戏动漫
时尚, 投资理财, 美食, 娱乐, 体育
视频音乐, 旅游, 新闻趣事, 星座命理, 微博奇葩
新闻资讯, 投资理财, 娱乐, 文艺, 健康, 旅游, 美食
投资理财, 健康
健康, 美女, 军事
教育就业, 健康, 美食, 文艺, 娱乐
数字 119 快乐, 96 小天使嫁我, 阳炎深度中毒
健康, 时尚, 教育就业, 美食, 旅游
时尚, 汽车, 军事
微博奇葩, 美女, 搞笑幽默, 新闻资讯, 汽车, 投资理财, 教育就业, 军事
健康, 文艺, 旅游, 投资理财, 美食
健康, 旅游, 游戏动漫, 美食, 娱乐
娱乐, 健康, 美食, 时尚
星座命理, 美食, 体育, 娱乐
IT 数码
娱乐
时尚, 新闻资讯, 美食, 教育就业, 汽车, 体育, 文艺
IT 数码, 投资理财, 时尚, 旅游, 汽车, 爱白痴, 爱睡觉, 喜欢安静
搞笑幽默, 美图摄影, 娱乐, 美食, 体育, 文艺, 旅游
旅游, 时尚, 娱乐, 美食, 文艺, 美女
娱乐
旅游, 军事, 娱乐
旅游, 美食, 文艺
八卦杂谈, 美女, 搞笑幽默
游戏动漫, 旅游, 美食
旅游, 文艺, 汽车, 时尚, 体育

美食,教育就业,投资理财,文艺,时尚,健康,娱乐
娱乐,时尚
美食
游戏动漫,投资理财,美女,汽车,娱乐
体育,娱乐,文艺,旅游,美食
文艺,旅游,娱乐,游戏动漫,美食
教育就业,美食,娱乐
美图摄影,视频音乐,美食
视频音乐,新闻趣事,搞笑幽默,宁夏生活,时尚,美女
星座命理,设计,怀旧,文艺
娱乐,文艺
动漫,海绵宝宝,90,看小说,巨蟹座
娱乐,体育,旅游,星座命理,军事
健康,旅游,娱乐,军事
体育
军事,娱乐
时尚
旅游,美食,健康
水彩,萌宠,画画,游戏动漫
体育,娱乐
汽车,美女
娱乐,投资理财,时尚,教育就业,健康,旅游,IT 数码,文艺
汽车,时尚,星座命理,美食,娱乐
新闻资讯,汽车,IT 数码
懒
美女,娱乐,星座命理,时尚,教育就业,健康
美食,金庸武侠小说,仙剑奇侠传,魔兽真三国无双,电影迷,魔兽世界,80 後
体育,军事,旅游,汽车
游戏动漫,教育就业,IT 数码
自行车,睡觉,旅行,电影唱 k 听歌,魔方,游泳爱好者,水瓶座加 O 型血,羽毛球,85 后,台球
美食,星座命理,旅游,健康,投资理财
文艺,旅游,军事,星座命理,IT 数码
微笑转身
新闻趣事,旅游,搞笑幽默,广州生活,投资理财,美食,娱乐
娱乐,文艺,体育
美食,文艺,娱乐
新闻资讯,教育就业,文艺,娱乐,美食
搞笑幽默,娱乐
投资理财,旅游,美食,汽车,体育
旅游
美食,教育就业,娱乐,旅游,健康,文艺
新闻趣事,新闻资讯,教育就业,娱乐,健康
IT 数码,旅游,星座命理,娱乐,体育
健康,投资理财,教育就业

娱乐,旅游,IT 数码,美女,体育
读书分享,美图摄影,美食,游戏动漫,娱乐
美女,娱乐,旅游,时尚
时尚
新闻资讯
旅游,IT 数码,美女,健康,娱乐
时尚
娱乐
健康,时尚,文艺,IT 数码,娱乐
音樂,汽车,IT 数码,旅游,体育
健康,游戏动漫
IT 数码,旅游,美食,星座命理,健康
娱乐
军事,IT 数码,娱乐
文艺,旅游,美食,IT 数码,娱乐
健康,军事,旅游,IT 数码,文艺
娱乐
体育
文艺
教育就业,新闻资讯,健康,旅游
校园生活
旅游,游戏动漫
旅游,健康,时尚,教育就业,娱乐,文艺
美女,健康,新闻资讯,投资理财,美食
摩羯,搞笑
游戏动漫,军事
体育,文艺,IT 数码,军事,时尚
娱乐
时尚,星座命理,美食
健康
健康,游戏动漫,文艺,美食,星座命理
星座命理,新闻资讯,美食,健康,旅游
健康,新闻资讯,娱乐
旅游,美食,新闻资讯,文艺,体育
教育就业
美女,汽车,娱乐,美食,体育
旅游,时尚,汽车,投资理财,健康
美食,旅游
美食,娱乐
健康
美食,体育,娱乐,旅游,汽车,军事
IT 数码,旅游,教育就业,投资理财,文艺
娱乐
新闻资讯,汽车,军事

娱乐,体育,IT 数码,旅游,汽车,新闻资讯,投资理财,星座命理
美女,游戏动漫
时尚
环保,童装,公主裙,潮流,时尚
美食,旅游,时尚,美食,星座命理,文艺,娱乐
时尚,新闻资讯,娱乐,文艺
健康
旅游,美食,星座命理,文艺
健康
游戏动漫,娱乐
娱乐
旅游
游戏动漫
旅游,星座命理,娱乐,美食,健康,时尚,投资理财,IT 数码
美女,美食,娱乐
娱乐,游戏动漫
时尚,健康,旅游,星座命理
文艺
软件,听歌,打篮球,新闻,健康,新闻资讯,投资理财,IT 数码,体育
旅游,IT 数码,星座命理,游戏动漫,军事
搞笑幽默
旅游
汽车,美食,旅游,健康,美女,娱乐,体育
健康,新闻资讯,时尚,星座命理,文艺
宅，还是宅
美食,时尚
体育
时尚
体育,旅游,文艺
健康
新闻资讯,时尚,教育就业,投资理财,美食,文艺,旅游
教育就业,娱乐,文艺,时尚
新闻资讯,旅游,汽车,IT 数码,军事
名人明星
星座命理,美食,IT 数码,文艺,娱乐
搞笑幽默,娱乐
教育就业,汽车,时尚,娱乐,美女,旅游,健康,投资理财
游戏动漫,体育
汽车,美女,健康,美食,时尚
美食,星座命理,健康,旅游,新闻资讯
娱乐,美食,美女,时尚
娱乐,教育就业,美食,游戏动漫,美女,美食,IT 数码
游戏动漫,旅游,娱乐
健康,旅游,军事

面膜控,护肤品,化妆品,梦想,娱乐,旅游,时尚
汽车,游戏动漫,体育
汽车,美女,游戏动漫,陌生人,娱乐,新闻资讯,美食,文艺
旅游,美女,体育
时尚,娱乐,文艺,美食,游戏动漫
校园生活,搞笑幽默,马鞍山生活
双子座
搞笑幽默
搞笑幽默,游戏动漫
运动,健康,新闻资讯,投资理财,旅游
文艺
娱乐,时尚,星座命理,旅游,发呆,听音乐,文艺,美食
育儿,购物狂,宝宝,金融
娱乐,星座命理,旅游,游戏动漫,美食
时尚
学生一族,摄影爱好者
娱乐,文艺
IT 数码
时尚,旅游,星座命理
旅游,时尚
新闻资讯,时尚,娱乐,美女,体育
星座命理,投资理财,健康,新闻资讯,IT 数码
IT 数码
美食
游戏动漫,时尚,IT 数码
北京生活
旅游,游戏动漫
游戏动漫,娱乐
教育就业,娱乐,时尚,投资理财
游戏动漫,旅游,星座命理,娱乐,美女
军事,旅游,星座命理,美食,IT 数码
IT 数码
星座命理
娱乐
军事,小说迷,宅男,美食
IT 数码
文艺,教育就业,旅游,美食,娱乐
旅游,娱乐
健康,教育就业
健康,IT 数码
旅游,汽车
健康,新闻资讯,娱乐
化疗,淋巴瘤,肺癌,肿瘤治疗,胸腹水
体育

汽车,投资理财,旅游,美食,体育
彩妆造型,彩妆师,80後,BIGBANG,潮流,旅游,时尚,娱乐,美食
军事,体育,汽车,美食,IT数码
美食,旅游,游戏动漫,汽车,娱乐
星座命理
时尚,投资理财,健康,旅游,美食
体育
名人明星,视频音乐,微博奇葩,时尚,娱乐,文艺
游戏动漫,美女,美食,军事,娱乐
汽车
体育
IT数码
视频音乐
体育
体育
娱乐,投资理财,新闻资讯,美食,文艺
娱乐,投资理财,汽车,旅游,美食
健康,新闻资讯,投资理财,教育就业,时尚
吃货,小说,娱乐
双子座双重性格,宅,听歌,90后,乒乓球,快乐大本营,时尚,游戏动漫,文艺,娱乐
囧,巧克力,美食,穿衣美容,时尚,偶稀饭睡觉觉,大学生
搞笑幽默,星座命理,穿衣美容,美女,美食,时尚,娱乐
执着,爱旅行
星座命理,美食,时尚,文艺,游戏动漫
时尚
军事,教育就业,美女,娱乐
游戏动漫
旅游
IT数码,汽车,旅游,游戏动漫,娱乐
IT数码
教育就业
旅游
娱乐,游戏动漫,军事,IT数码,体育
视频音乐
旅游,文艺
旅游,娱乐
投资理财
名人明星,湖北生活,汽车
美食,健康,旅游,娱乐,星座命理
汽车,旅游,军事,IT数码
时尚,旅游,美食,文艺
其他生活
旅游,陈乔恩,宅女一枚,闷骚90后,说走就走的旅行,拖延症,看书,纽约尼克斯
体育

汽车,美女
娱乐,旅游
IT 数码
体育,IT 数码,娱乐
美食,健康,投资理财,时尚,新闻资讯
旅游,娱乐,健康,文艺,体育,游戏动漫,时尚,美食
娱乐,星座命理
旅游,IT 数码,娱乐
IT 数码,时尚,旅游,体育,娱乐
美食,搞笑幽默,微博奇葩,穿衣美容
旅游,星座命理,美食,娱乐,文艺
视频音乐
美女,教育就业,时尚,新闻资讯,IT 数码
娱乐,投资理财,时尚,美食,美女
新闻资讯
娱乐
贵州生活,搞笑幽默
美食,健康
美食
旅游,健康,投资理财,IT 数码,娱乐
健康,新闻资讯,时尚
美食
文艺
文艺,美女
IT 数码,体育
微博奇葩,旅游,军事,汽车,体育,新闻资讯
文艺
游戏动漫,美食,新闻资讯,娱乐,文艺
旅游,游戏动漫,美食,IT 数码,娱乐
旅游,健康
旅游,美食
新闻资讯,美女,投资理财
体育,汽车
健康,美女,IT 数码
體育,時尚,健康,新聞資訊,旅遊
投资理财
佛山生活
新闻趣事,视频音乐,名人明星,微博奇葩,搞笑幽默,游戏动漫,新闻资讯,文艺,娱乐
美食,旅游,娱乐
旅游
投资理财,新闻资讯,健康,汽车,体育,娱乐
爱,喜欢音乐,健康,时尚,娱乐,教育就业,星座命理
搞笑幽默,电影爱好者,军事
健康

新闻资讯,星座命理,军事,娱乐,健康
热爱生活
娱乐
汽车
星座命理,微博奇葩,旅游,视频音乐
搞笑幽默,健康,时尚,娱乐
星座命理
销售,投资理财,美女,娱乐
美食
娱乐,美食
娱乐,美食,时尚
美食,健康,文艺
旅游
文艺,体育,娱乐,时尚
美食
教育就业,娱乐,美食
体育
IT 数码
时尚
投资理财,旅游,娱乐,IT 数码,美食
视频音乐,搞笑幽默,模拟人生,the,sims,搞笑,宅女,吃货,幽默
游戏动漫
IT 数码,时尚,健康,娱乐,投资理财
唱 k,任性,古灵精怪,健康,美食,星座命理
娱乐
体育
游戏动漫,旅游,美食,IT 数码,体育
时尚,教育就业,娱乐,投资理财
美食,文艺,娱乐
萌宠,微博奇葩,广东生活,搞笑幽默,美食,IT 数码
旅游,美食,体育
旅游,美食
IT 数码
美食
娱乐
文艺
新闻资讯,IT 数码
旅游,新闻趣事,视频音乐,江苏生活,教育就业,投资理财,军事,IT 数码
娱乐
射手座,音乐爱好者,动漫控
新闻资讯,汽车,美食
美女
娱乐
军事,IT 数码,体育

微博奇葩
美食,IT 数码,文艺
游戏动漫,投资理财,IT 数码
时尚
新闻资讯,旅游,军事,美食,IT 数码,投资理财
休闲小游戏,斗地主,扎金花,军旗,飞行棋,象棋,围棋,休闲游戏,棋牌游戏,游戏动漫
美食,旅游,时尚,游戏动漫
旅游
重庆生活,新闻趣事,穿衣美容,新闻资讯,健康,旅游,时尚,美食
旅游
健康,旅游,IT 数码
娱乐
旅游,美食,汽车,健康
游戏动漫
美食,健康,文艺,旅游
爱疯爱玩爱闹
投资理财
星座命理
体育
摄影工作室,台钓,茶艺茶道,旅游,美食,汽车,IT 数码
军事,IT 数码,时尚,美食,文艺,娱乐,体育
健康
萌宠,美女,搞笑幽默,星座命理,投资理财,军事,美食,体育,娱乐
美女,旅游,军事,时尚,娱乐
教育就业,星座命理,IT 数码,投资理财,健康,新闻资讯
娱乐
时尚,军事,美食,文艺,体育
旅游,美食,健康,时尚,文艺
名人明星,旅游,星座命理,美女,搞笑幽默,体育
游戏动漫,IT 数码
娱乐
旅游,游戏动漫,星座命理,美食,娱乐
搞笑幽默,IT 数码
美女,娱乐,教育就业,投资理财,汽车
体育
时尚写真,摄影师,武艺,上网,听歌,旅游,娱乐,文艺,美女,时尚
时尚,汽车,健康,投资理财,美女,娱乐,体育
美女,健康,旅游,文艺,投资理财,时尚,星座命理,IT 数码
健康,投资理财,军事,旅游,体育
网购,搞笑,乐观,新闻资讯,旅游,投资理财,文艺,IT 数码
美食,时尚,旅游
我就是我,美食
汽车
旅游,教育就业,美食,文艺

军事
星座命理,时尚,娱乐
美食,旅游,星座命理,文艺
萌宠,游戏动漫
投资理财
喜欢的人埋心底,Music,很垃圾,吃货,淡定,矛盾,狗狗,魔羯座,爱自由
投资理财,文艺,旅游
视觉系画室,淮北画室,画画艺术生,美术绘画,美术生,淮北生活
娛樂,體育,旅遊
投资理财,教育就业,汽车,游戏动漫,IT 数码
教育就业,新闻资讯,星座命理,文艺,娱乐,细节决定成败
旅游,军事
娱乐,美女,新闻资讯,军事,投资理财
体育,汽车
时尚,美食
美食,教育就业,新闻资讯,游戏动漫
旅游,汽车,IT 数码
美食,汽车,健康,体育,旅游
文艺
旅遊
健康,旅游,星座命理,美食,时尚
旅游,体育,IT 数码,美女
游戏动漫,旅游,美食,文艺
游戏动漫,星座命理,娱乐
娱乐,美女,游戏动漫,美食,新闻资讯,文艺
证券,美食,美女,游戏动漫,汽车,文艺
美女,汽车
旅游,汽车,投资理财,体育,娱乐
体育
汽车,投资理财,体育,旅游
游戏动漫,美女,美食,娱乐,星座命理,健康,汽车,时尚
美女,时尚,旅游,游戏动漫,汽车,娱乐,体育
美食,娱乐,文艺,体育,健康
游戏动漫,汽车,体育
体育,美食,美女
搞笑幽默
健康,时尚,娱乐,文艺
体育
旅游,新闻趣事,体育
时尚,健康,新闻资讯,星座命理,教育就业
新闻趣事
读书分享
美食,娱乐,文艺,时尚,旅游
动漫,娱乐

旅游,娱乐
汽车
娱乐
美女,IT 数码
游戏动漫,IT 数码
体育
搞笑幽默
时尚
美食,时尚,旅游,健康,娱乐
旅游,美食
新闻,星座命理,教育就业,健康,新闻资讯,投资理财
美食,健康,娱乐
娱乐
美女,旅游,军事
吃货，睡货，玩货
旅游,美食
文艺,体育,星座命理
美女
时尚,美女,低调,旅游,汽车,娱乐,投资理财,体育
游戏动漫
文艺
汽车,旅游,美食,军事
吃,网球,汽车,设计,喜欢音乐,火影忍者,时装,跑步,平面设计,时尚
IT 数码,汽车,体育
娱乐,美食
星座命理
化妆品,女人,中医
健康,时尚,体育,美食,旅游
新闻资讯,旅游,美食,投资理财,军事,时尚
星座命理
旅游,健康,星座命理,时尚,汽车
投资理财,星座命理,美食
美女,体育
旅游
IT 數碼,娛樂,時尚,旅遊,文藝
体育,美女
体育,娱乐
美女,设计师,销售,自信,汽车
杭州生活,时尚,汽车,投资理财,娱乐,文艺
旅游,文艺,投资理财
影视,打篮球,旅游,健康,IT 数码,文艺,时尚,美女,娱乐
文艺,娱乐
时尚
星座命理,教育就业

汽车, 教育就业, 军事, 体育, 美女
旅游, 美食, IT 数码, 体育, 军事
健康, 教育就业, 美食, 文艺, 娱乐
时尚, 体育, 美食, 健康, 汽车, 文艺, 娱乐
娱乐
IT 数码
杭州生活, 军事
美食, 宠物图集, 旅游, 兰州生活, 搞笑幽默, 游戏动漫, 星座命理
时尚, 娱乐, 星座命理, 美食, 旅游, 健康, 文艺
重庆生活, 体育
文艺
健康
娱乐
昂帕帕斯, 古川雄辉, 搞笑幽默, 时尚, 健康, 旅游, 星座命理, 文艺
新闻资讯, 旅游, 美女, 体育, 美食, 娱乐, 游戏动漫, IT 数码
美食, 旅游, 文艺, 体育, 娱乐
新闻资讯, 健康, 旅游, 时尚, 投资理财
新闻趣事, 视频音乐, 美食, 旅游, 江苏生活, 投资理财, 娱乐
IT 数码, 美食, 新闻资讯, 时尚, 娱乐
美女, 新闻资讯, 时尚, 文艺, 娱乐
美食
娱乐
搞笑幽默, 健康养生, 美食, 旅游, 新闻趣事
游戏动漫, 文艺, 娱乐
旅游
旅游, 娱乐, 时尚, 健康, 美食
美图摄影, IT 数码, 新闻趣事, 校园生活
IT 数码, 美女, 娱乐
娱乐
IT 数码
IT 数码, 游戏动漫
文艺
娱乐, 时尚, 健康, 星座命理
新闻趣事
美女
体育, 喜欢听歌, 篮球, 睡懒觉, 唱歌, 学生, 自由
美食, IT 数码, 美女, 文艺, 旅游, 投资理财, 健康
IT 数码
IT 数码, 紫色, 时尚, 汽车, 健康, 娱乐, 文艺
IT 数码, 美食, 娱乐, 文艺
淡定, 吃喝玩乐, 美食爱好者, 旅游摄影, 外貌协会, 我就是我, 财经, 睡觉睡到自然醒, 猫咪控, 臭美
星座命理, 旅游, 健康, 汽车, 美食
军事, 新闻资讯

美食
文艺,IT 数码,健康,娱乐
健康,体育,时尚,新闻资讯,投资理财
笑话,神经质,时尚
IT 数码,天津生活,搞笑幽默,房产,教育就业,旅游,新闻资讯,汽车,文艺
娱乐
健康,投资理财,美食,爬山,新闻资讯,时尚,娱乐,旅游
名人明星,娱乐
美食
健康,新闻资讯,时尚,星座命理,投资理财
萌宠,视频音乐,搞笑幽默,IT 数码,美食,健康,时尚,娱乐
美女
娱乐
军事,投资理财,健康,美食,体育
美女,IT 数码
旅游,单身,85 后,热爱生活,善良
新闻资讯,汽车,旅游,美食,军事
IT 数码
星座命理,健康,文艺,旅游,新闻资讯
喜欢听歌,处女座,咖啡,爱玩,文艺,娱乐
健康,旅游,军事
文艺,美食,健康,旅游
旅游
旅游,体育
投资理财,新闻资讯,教育就业,时尚,星座命理,文艺,娱乐
美食,汽车,IT 数码,时尚,娱乐
美容服饰,东莞生活,育儿百科,单纯,资讯,双鱼女,投资理财,旅游,星座命理,娱乐
美食,娱乐
格子控,明星,唱 k,金俊秀,随性,星座命理
时尚,星座命理,娱乐
美女,星座命理,体育,美食,时尚,IT 数码,文艺,娱乐
新闻资讯,教育就业,旅游,美食,娱乐
IT 数码
杨幂,明星,谢娜,天蝎女,服饰,小清新,90,网购,上网,时尚
时尚
水瓶,唱歌,围棋爱好者,魔兽争霸爱好者,科比球迷,Eminem 粉丝
娱乐,投资理财,美女,美食
新闻资讯,美女,时尚,娱乐,体育
美女,教育就业,军事
时尚,文艺,汽车
新闻资讯,时尚,美女,投资理财,美食,娱乐
时尚,投资理财,健康,旅游,美食
健康,美食
新闻资讯

汽车,星座命理,娱乐
旅游,投资理财,时尚,美食,娱乐
娱乐
旅游
旅游,军事,IT 数码,娱乐,体育
教育就业,新闻资讯,旅游,娱乐,文艺
娱乐
营销,电子商务,85 后,商业,新闻资讯
健康
娱乐,美食
美女
娱乐,体育,文艺
体育,娱乐
文艺,美食,旅游,星座命理,娱乐
汽车,旅游,美食,IT 数码,娱乐
娱乐,体育,旅游,美女,游戏动漫,时尚,汽车
白羊座,养生,美食控,好书
文艺,美食,娱乐,时尚
娱乐
文艺,体育
旅游
大连生活,视频音乐,汽车
IT 数码
体育
美女,游戏动漫,体育
美食,旅游,时尚
健康,教育就业
旅游,文艺
新闻资讯,投资理财,教育就业,健康,旅游
球料,足彩,篮彩,竞彩,体育
新闻资讯,健康
娱乐,旅游,星座命理
旅游
时尚,投资理财,新闻资讯,健康,旅游
旅游,星座命理,IT 数码,美女,投资理财
健康,旅游,汽车,美食,IT 数码,娱乐
搞笑幽默
文艺
旅游
执着,善良,低调,林峯,聊天,天蝎女,活泼,喜欢听歌,热爱生活,感性
星座命理,游戏动漫,体育,美女
文艺,教育就业,IT 数码,娱乐,旅游
微博奇葩,搞笑幽默,游戏动漫,娱乐
游戏动漫,娱乐

新闻资讯,汽车,星座命理,体育,娱乐
健康,娱乐,时尚,弹琴,画画,幽默, 乐观
娱乐,美女,IT 数码,时尚,游戏动漫
娱乐
体育,游戏动漫
星座命理
星座命理,穿衣美容,中山生活,搞笑幽默,新闻趣事,IT 数码,娱乐
时尚,投资理财,美食,娱乐
新闻趣事
教育就业,健康,投资理财,星座命理,美食
娱乐,星座命理
文艺,新闻资讯,军事
新闻资讯,健康,教育就业,娱乐,美食
娱乐,游戏动漫,文艺
自由插画师,平面设计师,迷恋海贼王,雙子男,b 型血,动漫,80 後,美剧,传媒,时尚
星座命理,汽车,90,美食,旅游,时尚,娱乐
星座命理,新闻资讯,美女,游戏动漫,IT 数码
经历使我成长,人生难求知己,美食,旅游
美食,游戏动漫,旅游,星座命理,军事,娱乐
陕西生活
汽车,美食,体育
网购达人,军事,时尚,美女,教育就业,IT 数码,旅游
佛教,爱生活,文化,红酒,读书,汽车,电影爱好者,新闻资讯,投资理财,旅游
星座运势,美食,游戏,车世界,娱乐
投资理财
视频音乐,娱乐
名人明星,美女,体育,娱乐
美食
名人明星,文艺
汽车,体育,旅游,星座命理,美食,文艺,娱乐
旅游,体育,IT 数码,游戏动漫
时尚,欧美音乐
娱乐
美食,游戏动漫
爱电影,简单,好书,喜欢旅游,手工,文艺
时尚,汽车,美食,IT 数码,体育
搞笑幽默
坚强,时尚,唱歌,学生一族,开朗
美食,旅游,美女,文艺
美女,旅游,星座命理,新闻资讯,健康
娱乐
钓鱼,汽车,美食,旅游,健康,新闻资讯
美食,IT 数码
健康,旅游,文艺

旅游,健康,时尚,体育,IT 数码,新闻资讯,汽车
美女,时尚
游戏动漫,星座命理
旅游,美食,文艺
旅游
旅游
美食,旅游,军事,体育,吃货一枚,盗墓笔记
娱乐,星座命理,投资理财
教育就业,文艺
旅游,投资理财,健康,星座命理,美食,娱乐
游戏动漫
旅游,星座命理,美食,娱乐,时尚
汽车,美食,IT 数码,文艺,娱乐
新闻资讯,旅游,教育就业,星座命理
娱乐,游戏动漫
娱乐,旅游,美食
健康
文艺,时尚,旅游,美食,娱乐
投资理财,健康,旅游,娱乐
旅游,体育
时尚
汽车,IT 数码,时尚,美食,娱乐
健康,旅游,美食,投资理财,文艺
烹饪,看电影,爱音乐,购物,摄影爱好者,睡懒觉,音乐达人,美食,旅游
帅哥,减肥,淘友,星座,服装搭配,美食,运动健身,艺术,时尚,胖子
成都生活,时尚,文艺
安徽生活,热爱生活,独立,幸福,乐天派,善良,活泼开朗
体育,军事,星座命理,旅游,时尚,娱乐
游戏动漫,星座命理,美食,IT 数码,娱乐
IT 数码
旅游
星座命理,军事,文艺
教育就业,新闻资讯,健康,IT 数码,汽车
娱乐
军事
睡觉,宅,美女,美食,娱乐
名人明星,美食,视频音乐,旅游,成都生活,汽车,军事,IT 数码,体育,文艺
游戏动漫
娱乐
时尚,健康,新闻资讯,星座命理,文艺
投资理财
健康,投资理财,游戏动漫
新闻资讯,军事,娱乐,IT 数码
新闻资讯,旅游,美食,文艺

IT 数码
美食
健康
云南生活,青春,时尚,90 后,奋斗,咖啡,学习,爱幻想,寂寞
汽车,美食,旅游,娱乐,美女
娱乐
汽车,健康,军事
健康,旅游,美食,时尚,娱乐
旅游
搞笑幽默,娱乐,新闻资讯,体育
体育
旅游
健康,时尚,美食,文艺,娱乐
美食
云南生活,新闻资讯,体育
IT 数码,军事
军事
文艺,娱乐,新闻资讯,旅游,美食
IT 数码
军事,美食,旅游
娱乐,教育就业,健康,旅游,美食
美食
高三,怀旧,追星,可爱,爱睡觉,帅哥,美容,五月天,动漫,上网
科学探索,动漫,美图摄影
娱乐,美食,旅游
美女,军事,IT 数码,体育,娱乐
高三,怀旧,追星,可爱,爱睡觉,帅哥,美容,五月天,动漫,上网
旅游,我爱周柏豪,我爱狗狗,我爱拉拉跟小哈
健康,时尚,投资理财,美食,旅游
高三,怀旧,追星,可爱,爱睡觉,帅哥,美容,五月天,动漫,上网
旅游,星座命理,健康
星座命理,健康,游戏动漫,文艺,军事
娱乐,健康,新闻资讯,时尚,旅游,美食
教育就业,体育
投资理财,新闻资讯,美女,军事,娱乐
体育,娱乐
娱乐,星座命理,新闻资讯,游戏动漫,投资理财
星座命理,教育就业
军事,美食,汽车,体育,娱乐
搞笑幽默,星座命理,视频音乐,福建生活,旅游,健康,投资理财,教育就业,娱乐
时尚,健康,旅游,美食,娱乐
体育,新闻资讯,军事,投资理财,汽车,IT 数码
星座命理
时尚,健康,IT 数码,体育

美食
健康
投资理财,新闻资讯,健康,娱乐,时尚
IT 数码
旅游
时尚,IT 数码
星座命理
星座运势
健康,旅游,游戏动漫,时尚,IT 数码
星座命理,文艺,娱乐
娱乐
娱乐
健康,投资理财,旅游,美食,体育
体育,星座命理,军事,汽车
新闻趣事
健康,时尚,旅游
星座命理,时尚,健康
新闻资讯,健康,星座命理,文艺,娱乐
微博奇葩,美女
身心灵健康,催眠,舞动治疗,艺术治疗,精神分析,心理医生,心理咨询
花样美男,潜水,双重性格射手座,时尚
美食,健康,时尚,投资理财
文艺,娱乐,旅游,美女,体育
星座命理,美食,娱乐,文艺,IT 数码
旅游,星座命理
美食
superjunior,elf,宅女加腐女,psp,SuperJunior
圭贤的服务员儿,圭贤
旅游,美食,IT 数码,投资理财,汽车
IT 数码,汽车,新闻资讯
星座命理
投资理财
IT 数码,教育就业,新闻资讯,军事,文艺
工作,书籍,天秤,旅游美食,旅游,教育就业,军事,文艺,体育
时尚,宅女加腐女,古灵精怪,乐观,吃货
新闻资讯,娱乐
游戏动漫
星座命理
健康,新闻资讯,星座命理,文艺,娱乐
旅游,massimodutti,潮人,时尚
娱乐,时尚,IT 数码,美食,健康,美女
吃货一枚,新闻资讯,娱乐,游戏动漫,美食,星座命理
健康,旅游
四川生活,娱乐

体育
娱乐
时尚,投资理财,IT 数码,文艺,娱乐
投资理财,教育就业,军事,体育,娱乐
汽车,健康,美食,星座命理,游戏动漫,IT 数码
娱乐,美食,旅游
星座命理,时尚,美女
星座命理
旅游,美食,娱乐,汽车,美女
美食,军事,游戏动漫,时尚,IT 数码
四川生活,新闻趣事,投资理财
美食
旅游,新闻资讯,星座命理
教育就业,新闻资讯,健康,旅游,美食
娱乐,IT 数码,旅游,游戏动漫
IT 数码,军事
减肥,美女,育儿,日本,吃,love,90 后非脑残,武艺,忧郁,文艺
健康,新闻资讯,汽车,投资理财,IT 数码
军事,IT 数码,游戏动漫,汽车,美女
健康
婚纱摄影,摄影爱好者,设计师 ing
旅游,星座命理,美食
巨蟹座,旅游
时尚,军事
文艺
游戏动漫,健康,军事,IT 数码
情感生活,星座命理
美女,娱乐
健康,旅游,美食,娱乐,文艺
fashion,街拍,秀场,超模,时尚
体育,IT 数码
美食,旅游,投资理财
娱乐
娱乐
旅游
时尚
金花茶,投资理财,新闻资讯,健康
广东生活
军事
健康,星座命理,投资理财,美女,汽车,军事,IT 数码
微博奇葩
美女,旅游,美食,文艺,体育
健康,教育就业,美食,娱乐,旅游
时尚,旅游,美食,娱乐

美食,旅游
游戏动漫
旅游,美女
90 后,神经大条,睡懒觉,无厘头,爱音乐,爱睡觉,水瓶座女生
教育就业,旅游,汽车,美食,健康,美女,IT 数码
健康,旅游,美食,文艺
新闻资讯
，文艺青年,90 你懂
高三,怀旧,追星,可爱,爱睡觉,帅哥,美容,五月天,动漫,上网
旅游
连接服务器失败,涂鸦
高三,怀旧,追星,可爱,爱睡觉,帅哥,美容,五月天,动漫,上网
新闻资讯,健康,旅游,星座命理,美食
健康,时尚,美食,旅游
娱乐
游戏动漫,美女,健康,IT 数码
投资理财,新闻资讯,汽车,军事,体育
新闻资讯,时尚,健康
美女,旅游
美女,文艺,娱乐,时尚
旅游,星座命理
旅游,星座命理
名人明星,林峰,上网,睡觉,学生,时尚,美食,旅游,娱乐
文艺,汽车
正太控,时尚潮流,帅哥,温柔,怀旧,旅游美食,爱睡觉,逛街,音乐发烧友,拍照
健康,娱乐,美食,学生一枚
健康
时尚,健康,旅游,文艺,教育就业
体育,文艺,IT 数码,健康
时尚,文艺
健康
名人明星,济南生活,视频音乐,美女,搞笑幽默,娱乐
健康
星座命理,美食,健康,新闻资讯,旅游,文艺,娱乐
健康,新闻资讯,旅游,星座命理,美食
投资理财
视频音乐,文艺
美食,体育,旅游
穿衣美容,互粉,幻想,時尚,梦想,旅游摄影,美食爱好者
美食控,文艺,旅游
体育,美女
数码,闷骚,流浪,吸血鬼日记,音乐发烧友,重口味,旅游爱好者,电影迷,90,天秤
旅游,美食,IT 数码
旅游,娱乐

健康
时尚
美女,娱乐
体育
旅游,搞笑幽默
时尚,投资理财,星座命理,美食,娱乐
汽车,教育就业,旅游,军事,体育
娱乐,新闻资讯,健康,星座命理,美食
投资理财,星座命理,游戏动漫,健康,旅游
新闻资讯
星座命理
新闻资讯,旅游,IT 数码,文艺,汽车
连环画,邮票,钱币,收藏,书籍,影视,美剧迷,投资理财
星座命理
遊戲動漫,游戏动漫
文艺
水瓶座,娱乐
搞笑幽默
旅游
时尚,旅游,文艺,娱乐,星座命理
美食,娱乐,投资理财,教育就业,体育,游戏动漫
穿衣美容,娱乐
懒,睡觉,麦霸,好书,美剧,玩儿,文字控,文艺,美食,旅游
新闻资讯
文艺,健康,美女,时尚
游戏动漫,星座命理,汽车,文艺,娱乐,射手座,睡觉睡到自然醒
美女
健康,旅游,美食,军事,体育
汽车,美女,旅游,娱乐,美食,军事
热爱生活,懒人,音乐爱好者,睡觉睡到自然醒
教育就业,美食,旅游,星座命理,健康
旅游,体育,文艺,娱乐,美食
星座命理,美食
健康,美食,娱乐,体育,星座命理
经济,财经,重庆,新闻资讯,投资理财
新闻资讯,健康,旅游
投资理财
时尚,文艺,娱乐,primadonna,AOA,FTI,李弘基
美食,时尚,娱乐
投资理财
美食
娱乐
视频音乐,娱乐
投资理财,新闻资讯,美女,IT 数码,美食

美女
有时提刀,有时提笔,政治很性感,思维出走,假甜美,画画的不出名,熊猫,恐怖片,传统文化,GucciRUSH2
娱乐
特价商品,网购推荐,网购包邮,秒杀团购促俏,秒杀活动,秒杀网,99 包邮,包邮,购物
汽车,体育,军事,美食,投资理财
娱乐,军事,旅游,美食,游戏动漫
美女
娱乐,维他命,爱吃,夜猫子,死神,文艺
视频音乐,美食,摄影控,独立,学生一族,文艺
游戏动漫
美女,娱乐
旅游,美食,时尚,健康
健康
美女,视频音乐,体育资讯,IT 数码,旅游
文艺,星座命理,健康,娱乐,时尚
新闻资讯,娱乐,体育,IT 数码,星座命理
娱乐
教育就业,IT 数码,投资理财,星座命理,新闻资讯,文艺
FTISLAND
美女,汽车,体育,旅游
旅游,文艺,娱乐
体育
健康,投资理财,美食,文艺,娱乐
文艺,娱乐
军事,新闻资讯,旅游,美食,IT 数码
思考,性感,影视,双鱼,星座控,新闻资讯,游戏动漫,时尚,文艺,娱乐
狮子座,游戏,交友,小说,教育就业,IT 数码,娱乐,游戏动漫
美食,时尚
八卦杂谈,星座运势,情感生活,新闻趣事,时尚,旅游,文艺,娱乐,星座命理
体育
汽车,体育
娱乐
健康,新闻资讯,投资理财,美食,旅游
娱乐
旅游,纠结,宅女一枚
星座命理,游戏动漫
汽车,文艺,IT 数码,军事
搞笑幽默,休闲
IT 数码,时尚,汽车,投资理财,娱乐
搞笑幽默,上海生活,微博奇葩,旅游,新闻资讯,娱乐,文艺,美食
健康,投资理财,旅游,美食,娱乐
教育就业,汽车,游戏动漫
健康,时尚,娱乐,美食,星座命理,旅游

时尚,美女,旅游,娱乐
教育就业,军事,健康,星座命理,体育,新闻资讯
娱乐
旅游,投资理财
旅游
美女,娱乐
文艺,投资理财,娱乐,时尚,旅游
巨蟹,逛街,室内设计,白羊座,旅游爱好者,完美主义,服装,美食,娱乐,时尚
旅游,狗狗,羽毛球,射手女,自然,爱美食,咖啡,宅女,娱乐
体育
健康,美食,旅游,时尚,文艺
音乐爱好者,动漫控,追星族,电影控,广东生活,娱乐
校园生活,美图摄影,游戏动漫
美容服饰,加拿大生活,旅游,娱乐,时尚,美女,美食
游戏动漫,军事,IT 数码,文艺,体育,娱乐
旅游,健康,星座命理,娱乐
新闻趣事
投资理财,娱乐
宜昌生活,娱乐
美女,时尚,旅游,美食,娱乐
文艺,新闻资讯,时尚,娱乐
投资理财,教育就业,美女,旅游,军事,文艺,娱乐
投资理财,新闻资讯,IT 数码,健康,军事
健康,旅游,新闻资讯,时尚
偶稀饭睡觉觉,安静,小女人,听音乐
娱乐
体育,军事,IT 数码,汽车
旅游,美食,健康,体育,娱乐
旅游
教育就业,时尚,健康,新闻资讯,旅游,美食,IT 数码,文艺,娱乐
新闻资讯
娱乐
新闻资讯,教育就业,旅游,美食
美食,IT 数码,健康,新闻资讯,教育就业,文艺,娱乐
健康,美食,旅游,星座命理
娱乐,文艺
娱乐,旅游,美食,时尚,文艺
IT 数码,新闻趣事,八卦杂谈,搞笑幽默,杭州生活
书籍,水瓶女
旅游,美食
微博奇葩,文艺
文艺
旅游,军事,文艺,娱乐,美女
微笑,健康,旅游,体育,美食,游戏动漫

军事
体育,游戏动漫
蚌埠生活,新闻趣事,新闻资讯
旅游,美食,健康
视频音乐,娱乐
健康,美食,创意,营销,美食爱好者,文艺,IT 数码,时尚,汽车
新闻资讯,汽车
娱乐,美食,旅游,时尚,游戏动漫
娱乐
星座命理,游戏动漫,娱乐
微博奇葩,旅游,穿衣美容,时尚
投资理财,新闻资讯
美食
汽车,IT 数码,体育
视频音乐,文艺
fertigacid,土壤改良剂,滴灌肥,叶面肥
文艺,旅游,健康,时尚,美食
星座命理
萌宠
八卦杂谈,军事,投资理财
历史,互联网,建筑,法律,摄影,旅游,健康,新闻资讯,汽车
健康,教育就业,IT 数码,娱乐,旅游
时尚,文艺
健康
美女
投资理财,健康,旅游,美食,娱乐
动漫控,BL,喜欢旅游,钓鱼,搞怪,香橙,读书,美剧,猫咪控,财经
美女,美食
美食
文艺,美女,时尚,娱乐,旅游
江苏生活,宏迪专用
文艺,娱乐,健康,旅游,美食
投资理财
娱乐
穿衣美容
美女,星座命理
健康,旅游,文艺,时尚
文艺,旅游
无聊,体育,旅游
时尚
美食,文艺,IT 数码,时尚,健康,旅游
旅游,美女,娱乐
旅游,双鱼女,世界杯,文艺
娱乐

天雷桃！,嗯,额,天雷桃
美女
IT 数码,时尚,美食,星座命理,娱乐
冷笑话,互联网,幽默,电子商务,网络营销,健康
IT 互联网,時尚,真诚,旅遊,爱音乐
美女,娱乐
新闻资讯,军事,娱乐,旅游
摩羯,阳光,K 歌,电影,音乐,旅行,90 后,体育,汽车,星座命理
娱乐,汽车,文艺,旅游
旅游
体育,旅游,健康,美食,投资理财,娱乐
汽车,娱乐
游戏动漫,旅游,星座命理,美食
健康
娱乐,游戏动漫,体育
陈奕迅 Eason
幻想。睡觉。
美女
冷门控,真性情,拖延症,歌词控,国学迷,强迫症,有节操,讲原则,单细胞,中二病
星座命理,健康,投资理财,汽车,美女
美食,娱乐,汽车,旅游,新闻资讯,游戏动漫,体育
投资理财,汽车,体育,娱乐
美女,游戏动漫,娱乐
汽车
自由职业,迪拜
时尚
健康,旅游,美食,时尚
旅游,星座命理,健康,美食,教育就业,文艺,娱乐
健康,时尚,美食,文艺,娱乐
时尚,美食,旅游
汽车,美食,旅游,IT 数码,娱乐
健康,美食
投资理财,时尚,健康,旅游,美食
教育就业,旅游,美食,健康,娱乐
投资理财,新闻资讯,军事,文艺,娱乐
文艺,健康,星座命理,新闻资讯,时尚,体育
汽车,娱乐,体育,美女
搞笑幽默,减肥,爱旅游,90 後,娱乐
教育就业,投资理财,新闻资讯,健康,时尚
70 后,投资理财,新闻资讯,时尚,军事,体育
胡思乱想,睡觉睡到自然醒,龟梨和也,爱旅游,吃货一枚,神经大条,涂鸦,无聊,闷骚,天枰座
美女,新闻资讯,奋斗,80 后的尾巴,水瓶座,思考,自由,随性,爱生活
教育就业,健康,旅游,美食,文艺,娱乐
美食,健康,时尚,娱乐,星座命理

娱乐,健康,旅游,美食,星座命理
情感生活,美食,旅游
健康,娱乐
星座命理
美女,娱乐,新闻资讯
美女,娱乐,美食,汽车,时尚
投资理财,美食,健康,体育,娱乐,汽车,教育就业,军事
投资理财,新闻资讯,军事
新闻资讯,投资理财,旅游,美食,健康,体育,娱乐
体育
美食
时尚,旅游,文艺,美食,娱乐
星座命理,旅游,美食,娱乐,游戏动漫
魔兽,天蝎女,娱乐,游戏动漫,美食,星座命理,文艺
名人明星
美食
睡觉,自由,80後,汽车,新闻资讯,IT 数码
汽车
美女,时尚,娱乐
旅游
漫画,爱玩,体育,美食,旅游,军事
美食
美女,IT 数码
视频音乐,星座命理,美食,旅游
微博奇葩
美女,投资理财,文艺,体育,教育就业
旅游
文艺
娱乐,IT 数码,旅游,健康,时尚
美女,体育
健康,教育就业,新闻资讯,娱乐,时尚,旅游
投资理财,旅游,新闻资讯,时尚,健康
IT 数码
视频音乐,浙江生活,搞笑幽默,美女,汽车,投资理财,新闻资讯,IT 数码,娱乐
时尚
健康,时尚,旅游,IT 数码
旅游
旅游
体育
旅游
娱乐,美食
可爱,感性,爱幻想,爱玩,爱笑,自恋,偏执狂,时尚,懒训猪,吃货
旅游,健康,娱乐,文艺,美食
美食

体育
地质工程,英雄联盟,电影爱好者,Eason 陈奕迅,教育就业,军事,O 型血,唱 K,喜欢听歌,学生
视频音乐,音乐迷,美女,娱乐
汽车,时尚,娱乐,体育
美女
美食
爱旅行,热爱生活,驴友,阳光,旅游,文艺
游戏动漫
教育就业,新闻资讯,投资理财
时尚潮流,时尚,游戏动漫,美女,体育,娱乐
许昌生活,美容服饰,时尚,教育就业,新闻资讯,健康
江苏生活
娱乐
军事,健康,旅游,汽车
游戏动漫,美食,旅游,星座命理,健康,投资理财
旅游,美食,文艺,IT 数码,娱乐
新闻资讯,游戏动漫,旅游,星座命理
HelloKitty 控,甜品控,代购,時尚,音樂,行街街,电影,烹飪,天枰座 O 型女,80 後
旅游
娱乐
健康,时尚,星座命理,IT 数码,娱乐
旅游美食爱好者,旅游,法国,怀旧,好书,运动,吃货,天蝎座
游戏动漫,美女,IT 数码,体育,娱乐
美女,旅遊
投资理财,娱乐,新闻资讯,汽车
旅游,教育就业,体育,娱乐
旅游
军事,IT 数码,文艺,体育,娱乐
健康,娱乐,文艺,时尚,旅游,美食
学生一枚,生活大爆炸,电影控,音乐发烧友,乐观,运动,吃货,幽默
星座命理,美食
新闻资讯,文艺,娱乐,星座命理
旅游,广西生活,搞笑幽默,军事,星座命理,娱乐
新闻资讯,美食,时尚,文艺,健康
射手座,游戏动漫
IT 数码,旅游,美食
游戏动漫,90 后但我不脑残,手机看小说,看电影,腐女,发呆,网购,吃,搞笑,双子女
文艺,时尚,教育就业,投资理财,娱乐
福尔摩斯迷,电影迷,书,美剧控,猫
文字,八卦,旅游,美食,文艺,娱乐
新闻趣事,穿衣美容,吕梁生活,视频音乐,新闻资讯,旅游
游戏动漫,美食,体育,娱乐
摩羯,情感,疯子,宅女加腐女,爱吃,服装,玩,懒,星座命理
微博奇葩,星座命理,搞笑幽默

旅游,汽车
穿衣美容,新闻趣事,娱乐,汽车,美女,旅游
娱乐,旅游,健康,教育就业,文艺
户外,狗狗,打球,我就是我,爬山,孙燕姿,听歌,淡定,dota,低调
美食,投资理财
娱乐
文艺
美食,美女,星座命理,投资理财,时尚
娱乐,美食,体育
时尚,教育就业,健康,美食,娱乐
旅游
投资理财,时尚,美女,娱乐,文艺,旅游
IT 数码
美食
健康,美食
军事,IT 数码,80 后,强迫症,宅男,爱淘宝,梁静茹,市场营销,热爱生活,周杰伦
美食,美女,武术,法国,爱情,微博,金牛座,爱自由,k 歌,少女时代
美女,旅游,军事,IT 数码,汽车
重口味,看电影,爱音乐,购物,财经,玩儿,吸血鬼,学生一枚,宅女加腐女,时尚
投资理财,星座命理,旅游,美食,娱乐
百鬼夜行抄,英语,日语,美食,白羊座,时评,运动,搞笑,科学探索,听音乐
健康,旅游,星座命理,美食
IT 数码,体育,乒乓球,篮球
军事
娱乐,体育,军事
娱乐,IT 数码
健康,旅游,文艺,美食
旅游,美食,星座命理,娱乐,时尚
美食,旅游,娱乐
美女
包包控,淘宝掌柜,娱乐,时尚
旅游
美食,搞笑幽默,视频音乐,新闻趣事,IT 数码,新闻资讯,旅游,教育就业,体育
汽车,IT 数码,旅游,美食,娱乐
游戏动漫,美食,IT 数码
美食,时尚,汽车,旅游,游戏动漫
教育就业,时尚,娱乐
美女,娱乐
体育
时尚,星座命理
汽车,投资理财,旅游,星座命理,IT 数码,文艺
旅游,星座命理,美食
美食,教育就业,旅游
娱乐

游戏动漫,汽车,旅游,娱乐,体育
旅游,星座命理,美食,娱乐,文艺
IT 数码
汽车,美食,娱乐,投资理财,旅游
时尚,文艺,旅游
投资理财
时尚,旅游,健康,美食,文艺
投资理财,美食,文艺,美食
军事
汽车,美女,旅游
名人明星
新闻资讯,旅游,娱乐,汽车,军事
福建生活,美女,名人明星,健康,娱乐,旅游,美食
娱乐,新闻资讯,健康,旅游,美食,军事,美女
教育就业,文艺,美食,健康
新闻资讯,时尚,健康,美女,体育,娱乐
新闻资讯,游戏动漫,娱乐,体育,文艺
娱乐
教育就业,新闻资讯
健康,时尚,美食
投资理财,旅游,健康,美食,体育
娱乐
旅游,IT 数码,文艺,体育,娱乐
军服,腐宅基,八卦
投资理财,汽车,脐橙蜜桔中介,脐橙,寻乌蜜桔,赣南脐橙
旅游
双鱼,80 后
娱乐,旅游,游戏动漫,汽车,体育
美食,星座命理,教育就业,娱乐
汽车,投资理财,军事,文艺,新闻资讯,健康
教育就业,时尚,新闻资讯,娱乐,健康,美食
娱乐
时尚,星座命理,健康,旅游,美食
美图摄影,新闻资讯,旅游,游戏动漫,娱乐
投资理财
KimSungHwan,金成焕,TimeZ
旅游,健康,IT 数码,美食,文艺,游戏动漫,星座命理
新闻资讯,娱乐,美女,游戏动漫,IT 数码
双子,90 后非脑残,爱玩,火影忍者,白贤,exo,星座命理,娱乐
旅游,美女,星座命理,军事,娱乐,体育
旅游,文艺
娱乐
汽车,军事,IT 数码,娱乐,体育
时尚,新闻资讯,旅游,健康,星座命理

美食
娱乐
旅游,娱乐
时尚,旅游,星座命理,美食,文艺,娱乐
旅游
星座命理
汽车,美女,娱乐,时尚,IT 数码
朝阳生活,搞笑幽默,视频音乐,文艺,美食,旅游,体育
旅游,美食,文艺,娱乐
投资理财,星座命理,文艺
巨蟹女,电影迷,红酒,音乐爱好者,旅游美食爱好者
军事
游戏动漫,美女,新闻资讯
健康,投资理财,教育就业,新闻资讯,文艺
时尚,健康,旅游,星座命理,娱乐
军事,美食,娱乐,体育
体育
娱乐
娱乐,体育,旅游
时尚,文艺
娱乐,IT 数码,军事,美食,星座命理,时尚
文艺
IT 数码,新闻资讯,汽车,投资理财,旅游,体育,娱乐
吉他,唱歌,徐娇,篮球,跑步,时尚,文艺,体育,娱乐
美食,美女
云浮生活,体育
健康
旅游,IT 数码
娱乐,旅游,美食
时尚,娱乐,美女,投资理财
IT 数码,投资理财,旅游,娱乐,星座命理
萌宠,IT 数码
时尚,旅游,美食,IT 数码
娱乐
健康,军事
IT 数码,电影爱好者,动漫
新闻趣事,重庆生活,投资理财,汽车,新闻资讯,娱乐
健康,美食,时尚,体育,旅游
旅游,美女,时尚,星座命理,娱乐
娱乐,星座命理,旅游
旅游,健康
娱乐
旅游,时尚,美食
教育就业,军事,IT 数码

美食,美女,投资理财,游戏动漫,娱乐
星座命理,汽车,美食,IT 数码,体育
美女
IT 数码,旅游,投资理财,娱乐,小说,宅女,美食,海绵宝宝,网络,微笑
搞笑幽默,吃货一枚,懒,媒体,传媒,唱 k,O 型血,唱 K,笑,麦霸
文艺,时尚,健康,旅游,美食,娱乐
投资理财,新闻资讯
健康,文艺,游戏动漫,旅游,美食,体育
美食,娱乐
旅游
新闻趣事,健康,时尚,文艺
吃货
搞笑幽默
江苏生活,游戏动漫,娱乐,IT 数码
健康,新闻资讯,文艺,娱乐,美食
投资理财,教育就业,健康,汽车,旅游,文艺,娱乐
投资理财
文艺,旅游,美食,星座命理,IT 数码
投资理财,教育就业,文艺
视频音乐,唐山生活,新闻趣事,美女,搞笑幽默,娱乐,体育
娱乐,GDrayon,VIP,BIGBANG
娱乐,旅游,文艺
健康
时尚
搞笑幽默,美女,游戏动漫
视频音乐,新闻趣事,娱乐
健康,旅游
文艺,旅游,健康,游戏动漫,美食,汽车,娱乐
旅游,美食,健康,星座命理,娱乐
娱乐,星座命理,旅游,美女,时尚
IT 数码,时尚,健康,美食,汽车,旅游
娱乐,时尚,健康,旅游,美食
美女,娱乐
文艺,美食,时尚,健康,旅游
时尚
军事,美食,游戏动漫,教育就业,旅游
汽车,健康,旅游,美食,娱乐
美食,娱乐,汽车,游戏动漫,美女
网王同人女一枚,网王迷,日语,电脑,帅哥,猫,猫咪控,处女座,正太控,游戏
新闻资讯,时尚,星座命理,旅游,健康
娱乐
视频音乐
军事,新闻资讯,文艺,投资理财
IT 数码,文艺,美食,游戏动漫,旅游

美女,汽车,投资理财,美食,娱乐
健康
旅游,体育,IT 数码,投资理财
IT 数码,文艺,游戏动漫,娱乐,体育
广告创意,育儿交流,胶片,摄影,摄影工作室,儿童,儿童心理,儿童摄影师,儿童摄影,时尚
星座命理
投资理财
美女,游戏动漫,美食,星座命理,娱乐
新闻资讯
新闻趣事
IT 数码,娱乐
美食爱好者,双子座、
美女,旅游,星座命理
体育,娱乐,星座命理
投资理财,开朗,美容,化妆,双鱼女,潮人
娱乐
名人明星
军事,星座命理,旅游,游戏动漫,娱乐
美食,美容服饰,旅游,军事,汽车,文艺,时尚,IT 数码
游戏动漫,军事,美食,体育
教育就业,汽车,游戏动漫,美女
IT 数码
游戏动漫,旅游,IT 数码
美食
微笑,执着,巨蟹座,胡思乱想,平面设计,爱睡觉,好性格,烹饪,夜猫子,发呆
健康
文艺,军事
娱乐
名人明星,娱乐
娱乐,游戏动漫,星座命理
美女,游戏动漫
娱乐,体育,文艺,美食,美女
文艺
新闻资讯,IT 数码,文艺,体育,娱乐
文艺
娱乐
旅游
体育,军事,IT 数码
视频音乐,健康
新闻资讯,娱乐,健康,星座命理,陈奕迅,音乐,心情,旅游
旅游,美食
娱乐,时尚,体育
美食,星座命理
娱乐

投资理财,娱乐,美食,星座命理,新闻资讯,健康
美食
美食,新闻资讯,旅游,军事,文艺
三国杀,粤语,美食
旅游,美食
美女,体育,IT 数码,游戏动漫,娱乐
文艺
时尚,懒人一枚,爱睡觉,星座命理,游戏动漫
美食
星座命理,体育,旅游
时尚,旅游,美食,文艺,娱乐
健康,美女
新闻资讯,健康,投资理财,军事
游戏动漫
游戏动漫,星座命理,旅游,时尚,投资理财,娱乐
娱乐,美食
视频音乐
美女,汽车,健康,娱乐,体育
娱乐,健康
美食,微博奇葩,搞笑幽默,游戏动漫
星座命理,三分钟热度,动漫,猫猫控
时尚
李弘基,我爱抱抱熊,水果,娱乐,旅游,美食,文艺
娱乐,美食,旅游,投资理财
健康,旅游
体育
美女,游戏动漫,健康
娱乐
旅游,健康,教育就业,投资理财,星座命理,体育
体育
体育
健康,IT 数码
新闻资讯
时尚,教育就业,美食,文艺,IT 数码
美女
美食
投资理财,娱乐,旅游,体育,军事
游戏动漫
sara,韩国童星
美女
娱乐
美食,旅游,80 后,自由
教育就业,美女,健康,美食,娱乐
八卦杂谈,旅游,文艺

文艺,娱乐,健康
汽车,军事
北京生活,美食,旅游,视频音乐,体育
星座命理,旅游,IT 数码,美食,娱乐
娱乐,投资理财,时尚,旅游,文艺
教育就业,美食,军事,IT 数码,体育
旅游
美食,健康,美女,投资理财,娱乐
娱乐,时尚,星座命理,旅游,美食
美食
文艺,美女,娱乐,时尚,新闻资讯
健康,娱乐,星座命理
游戏动漫,娱乐,体育
美食,无聊,巨蟹,玩儿,90,连接服务器失败,娱乐
志愿者,旅游,军事,时尚,汽车,健康,IT 数码
健康
体育,星座命理
投资理财
搞笑幽默,视频音乐,旅游
美食,美女,游戏动漫,体育
娱乐,旅游,汽车
健康,娱乐,美食,星座命理,旅游,文艺,时尚
军事,IT 数码,文艺,体育,娱乐
视频音乐,萌宠,旅游,美女,星座命理,娱乐
美食,体育,文艺,IT 数码,娱乐
时尚,娱乐,教育就业,游戏动漫,星座命理
文艺,汽车,体育,时尚,健康,旅游
美女,IT 数码
电影爱好者,学生一族,美食
美食,新闻资讯,游戏动漫,教育就业,IT 数码
投资理财
新闻资讯,星座命理,军事,娱乐
时尚,汽车,IT 数码,教育就业,投资理财
新闻资讯,游戏动漫,投资理财,汽车,旅游
新闻趣事,美食
投资,执着,简单,天蝎,美女,文艺,娱乐,体育,投资理财
游戏动漫
文艺,娱乐,体育,时尚
赵薇,戚薇
娱乐
汽车
美女
教育就业
娱乐

星座命理,健康
娱乐,健康,美食,时尚,汽车,体育
文艺
娱乐,体育,时尚,汽车,旅游
体育,娱乐,旅游,健康,时尚
搞笑幽默
投资理财,军事,体育,娱乐,新闻资讯
搞笑幽默,相声,IT,传媒,通信,足球
健康,美食,娱乐
广东生活,娱乐
时尚,星座命理,美食,娱乐
体育
游戏动漫,教育就业,美食,汽车,旅游,美女
旅游,萌宠,搞笑幽默,美食,IT 数码,时尚,文艺,健康
投资理财,娱乐,体育
时尚,汽车
新闻趣事,新闻资讯
娱乐,时尚,教育就业,旅游,健康,美食
美食
新闻资讯,时尚,教育就业,投资理财,文艺
IT 数码,军事
意法希范,花神大爱比宝,超级木头傻小孩
星座命理,健康,军事,投资理财,体育
美食,健康,时尚,教育就业,投资理财,星座命理
娱乐,旅游,美食,文艺
搞笑幽默
军事
娱乐,游戏动漫,IT 数码
微博奇葩,娱乐,体育,文艺,IT 数码
体育,娱乐,游戏动漫,美女,时尚,旅游
美食,旅游,视频音乐,微博奇葩,新闻趣事
体育,旅游,娱乐,军事,游戏动漫
美食
娱乐,游戏动漫
搞笑幽默
视频音乐,校园生活,动漫,汽车
音乐达人,唱 K,健身,健康,投资理财,时尚,汽车,IT 数码
视频音乐,广西生活,体育
健康,时尚,教育就业,美女,娱乐
旅游,美食
健康,军事,体育,美女,美食
美食,体育,娱乐
游戏动漫,时尚
美女,健康,星座命理,娱乐,旅游

睡觉睡到自然醒
新闻资讯,教育就业,文艺,健康,旅游,军事
新闻资讯,投资理财,时尚,健康,娱乐
星座命理,电视剧,美食
美女,時尚,汽車,體育,娛樂
随性,旅游
教育就业
新闻资讯,健康,美食,旅游,投资理财
时尚,健康,投资理财,教育就业,文艺
体育
旅游
美食,星座命理
娱乐
旅游
游戏动漫
投资理财,娱乐,汽车,星座命理,军事,游戏动漫,美女,IT 数码
仙后,dota,天蝎座的 90 后,exo,完美主义,时尚潮流,星座命理,时尚,投资理财,娱乐
互粉,北京,爱睡觉
军事,文艺,娱乐
旅游
美食,IT 数码,时尚,游戏动漫,文艺
德国,新闻资讯,投资理财
军事,娱乐,体育
美食,投资理财,娱乐,星座命理
健康
动漫,娱乐,时尚,美食,文艺
吃喝玩乐,吃货,学生一族,服装设计,爱旅行,小资,80 后宅女,美女,时尚
汽车,旅游,IT 数码,军事,游戏动漫
游戏动漫
新闻资讯,健康,旅游,娱乐
游戏动漫,汽车,旅游,军事,体育
汽车,体育
视频音乐,旅游,星座命理,搞笑幽默,辽宁生活,双重性格,喜欢听歌,游戏,文艺,娱乐
新闻资讯,体育
心理学,旅游,IT 数码,新闻资讯,军事
新闻资讯
娱乐
视频音乐,随性
福州生活,健康,教育就业
旅游,搞笑幽默,文艺
娱乐,美食,旅游
美食,娱乐,文艺
新闻资讯,旅游,星座命理,美食,文艺
娱乐,IT 数码,体育

旅游,健康,时尚,娱乐
汽车,时尚,美女
教育就业,健康,旅游,时尚,投资理财
体育,IT 数码
体育
健康,体育
IT 数码,新闻资讯
汽车,游戏动漫,旅游,娱乐,体育
旅游
新闻资讯,美女,健康
投资理财,旅游,星座命理,美食,娱乐
健康,路边的风景在变
娱乐,教育就业,美食,旅游,时尚,文艺
美女,汽车,娱乐,IT 数码
TG 党,权志龙,BIGBANG
游戏动漫,美女,娱乐
美女
美食,体育,健康,旅游
潍坊生活,新闻资讯,健康
潮人,陈奕迅,吉他,互粉,钢琴,微博,旅游,时尚,汽车
新闻资讯,星座命理,美食,时尚,健康
体育,娱乐
新闻趣事,深圳生活,美容服饰,搞笑幽默,旅游摄影,美食爱好者,爬山,唱 k,康熙来了,苹果控
游戏动漫,旅游,星座命理,美食,娱乐
搞笑幽默,星座命理,美食,旅游
星座命理,美食
时尚,美食,星座命理,娱乐
浙江生活,微博奇葩,萌宠,旅游,健康,美食,娱乐,文艺
名人明星,娱乐,文艺
旅游,美食,IT 数码,汽车,娱乐
美食
潭潭
时尚,健康,文艺,IT 数码,娱乐
星座命理,美食,健康,游戏动漫,娱乐
文艺
新闻资讯,旅游,美食,投资理财,教育就业
美女,游戏动漫
星座命理,娱乐
文艺,娱乐,时尚,旅游,投资理财
体育,健康,旅游,军事,IT 数码
淘宝购物,淘宝,投资理财,IT 数码,新闻资讯,美女,娱乐
在看冰与火之歌
上海生活,游戏动漫,时尚,体育

校园生活
美食,汽車
新闻趣事,音樂,新闻资讯,汽车,军事,体育
健康,美食,娱乐
文艺,新闻资讯,美女,星座命理
广东生活,体育,运动,看电影,交朋友
健康,时尚,旅游,美食,娱乐
文艺,娱乐
星座命理,新闻资讯
美食,星座命理,穿衣美容,新闻趣事,旅游
时尚,游戏动漫
休闲,宅女,娱乐,环保,跑步,IT 互联网,策划,女裤,猫咪控,时尚
美食,健康,旅游,时尚,星座命理
体育
新闻资讯,汽车,旅游,军事,IT 数码
摄影
星座命理
军事
赛车,摄影爱好者,心理,旅游,美食
旅游,娱乐,军事
旅游,军事,IT 数码,体育,娱乐
名人明星,四川生活,时尚,体育,娱乐
娱乐,体育,IT 数码
名人明星,时尚
教育就业,投资理财,健康,游戏动漫,美食
娱乐,时尚,美食,旅游
时尚,新闻资讯
游戏动漫,美食,旅游,星座命理
IT 数码,旅游,体育,汽车
娱乐,游戏动漫,星座命理
新闻资讯,旅游
娱乐,美食,IT 数码
汽车,美女,美食,投资理财,IT 数码
星座命理,旅游,文艺
游戏动漫,时尚,汽车,体育,IT 数码
娱乐
文艺,旅游,时尚,美食,娱乐,星座命理
新闻资讯,美食,健康,教育就业,投资理财
娱乐
搞笑幽默,美图摄影,美女,校园生活,亳州生活,新闻资讯,时尚,文艺,娱乐
江苏生活,时尚
麻豆們,李秀赫,安宰賢,金宇彬
娱乐
IT 数码,新闻资讯,时尚,投资理财

汽车,娱乐,唱 K,苏打绿,时尚潮流,85 后
水瓶座,游戏,电影，听音乐,O 型血
新闻资讯
时尚,新闻资讯,娱乐,军事,文艺
娱乐,星座命理,时尚,游戏动漫,美女
星座命理
旅游,新闻资讯
军事,游戏动漫,旅游,娱乐,文艺,体育
游戏动漫,文艺,新闻资讯,汽车,投资理财,旅游,娱乐
旅游
军事,美女,体育,文艺,IT 数码
美食,游戏动漫
美图摄影,法国生活,旅游,旅游用车,NUK,巴黎代购,IT 数码,汽车
旅游
娱乐
娱乐,健康,文艺,美食
文字控,旅游美食爱好者,电影控,小清新,宅女加腐女,星座命理,健康,游戏动漫,文艺,美食
美女,美食,旅游,星座命理,IT 数码
汽车,新闻资讯,健康,旅游,IT 数码
美食,旅游,体育,游戏动漫,娱乐
汽车,IT 数码
娱乐,体育
文艺,新闻资讯,游戏动漫,娱乐
萌宠,天秤座
旅游
微博奇葩,搞笑幽默,视频音乐,新闻资讯,游戏动漫
美食,体育,娱乐,韩国,热情,快乐大本营,好性格,志愿者,表演,星座命理
新闻资讯,教育就业,IT 数码
旅游,文艺,娱乐
美食,美女,微博奇葩,浙江生活,搞笑幽默
娱乐,美女,IT 数码
体育,星座命理,美女,时尚,娱乐
汽车,游戏动漫,旅游,IT 数码,文艺
美食,娱乐
美食,旅游,时尚,健康,娱乐
工作狂
文艺,旅游,时尚,星座命理,娱乐
旅游,文艺,时尚
游戏动漫,旅游,IT 数码
娱乐,我就是我,旅游美食爱好者,爱猫,欧美,疯子,插画
星座命理,自恋,真诚,任性,教师
穿衣美容,时装,韩国,街拍
星座命理,时尚,美食,旅游,文艺
娱乐

美女,旅游
新闻资讯
旅游,时尚,文艺,娱乐
美女,軍事,IT 數碼
美女,军事,游戏动漫,体育,娱乐
时尚
视频音乐
广告,台球,简单,狮子座,生活,游戏动漫,新闻资讯,健康,文艺
教育就业,健康,时尚,旅游,文艺
娱乐,IT 数码,投资理财,美食
投资理财,IT 数码,文艺,娱乐
教育就业,健康,时尚,文艺,星座命理
校园生活,娱乐
美女
聊天
游戏动漫
北京生活
星座命理,健康,时尚,投资理财,IT 数码
新闻资讯,汽车,军事,IT 数码,娱乐
娱乐
星座命理,穿衣美容,萌宠,美食,旅游,健康
健康,文艺,美食,旅游,娱乐
文艺,旅游,娱乐
游戏动漫,汽车,军事,体育
新闻资讯,文艺,美食,旅游
旅游
健康,星座命理,旅游
意大利,旅游,时尚
旅游,美食,IT 数码,娱乐,文艺
投资理财,文艺,教育就业,健康,旅游,星座命理
狂想调,腐,游戏动漫,教育就业,新闻资讯,旅游,文艺
健康
教育就业
投资理财,美女
美食
美女,娱乐
体育,90 后,爱笑,散打,运动
星座命理
旅游,健康
时尚,文艺,娱乐
军事,IT 数码,汽车,体育
体育
旅行,建筑,天枰座,汽车
旅游

星座命理,军事
娱樂
教育就业
时尚
美食,游戏动漫,体育
微博奇葩,健康,新闻资讯,旅游,星座命理,军事
文艺,娱乐
旅游,文艺,娱乐,军事
军事,IT 数码
娱乐,健康,旅游,星座命理,汽车
fashion,时尚
宅女一枚,强迫症,减肥,天秤
恐怖片,90 后但我不脑残,思考,梦想,喜欢唱歌,吸血鬼,美剧迷,活泼开朗,游戏动漫,美女
发呆,jomoo,重庆生活,四川生活,图书馆,音乐,电影,汽车,牛牛熊集团
文艺,星座命理,美食,IT 数码,娱乐
旅游,IT 数码,感性,美女,汽车,文艺
文艺
游戏动漫,新闻资讯,时尚,IT 数码
娱乐,教育就业,健康,时尚,旅游
校园生活,文艺小青年
医疗卫生,健康
汽车,军事
创业,文艺,汽车,时尚,新闻资讯
、、
健康,旅游,美食
足球迷,世界杯,足球,巴萨,销售,梅西,管理,运动,看书,时事
文艺,IT 数码,星座命理,娱乐,美女
美食
娱乐
时尚,游戏动漫
军事
旅游,文艺,娱乐,美女
娱乐
IT 数码,减肥,文艺,健康,星座命理,娱乐
健康,旅游,美女,美食,新闻资讯,游戏动漫,娱乐,体育
美食,旅游
星座命理
投资理财,时尚,星座命理
汽车,文艺,娱乐,美女,健康
美女
军事
旅游,穿衣美容,新闻趣事,搞笑幽默
时尚,IT 数码
爱幻想,单纯,90,海绵宝宝,无厘头

星座命理,文艺,娱乐,投资理财,旅游,时尚
旅游,健康,美食,文艺,娱乐
古诗辞,时政
IT 数码
文艺
游戏动漫,时尚,投资理财,娱乐,美女
文艺,旅游,美女,投资理财,体育,汽车
文艺,美食,旅游,健康,教育就业
美食,时尚,娱乐
汽车,娱乐
美女,新闻趣事,穿衣美容,搞笑幽默,健康,时尚,投资理财,娱乐
体育
美女,旅遊,汽車,遊戲動漫,時尚
新闻资讯,投资理财,培训,桌球,健康,美食
健康,新闻资讯,军事,旅游
旅游,新闻资讯,星座命理
旅游,时尚,文艺,体育
游戏动漫,文艺,娱乐,美女,体育
时尚,IT 数码,美女,投资理财,汽车,体育
水瓶座,记者,爱曾轶可,90 後
旅游,投资理财,文艺
IT 数码,体育,娱乐
体育
游戏动漫,星座命理,文艺
高压焊接
萌宠,投资理财,健康,军事,教育就业,文艺
体育,游戏动漫
娱乐,健康
文艺,新闻资讯,娱乐,体育,军事
旅游
美食,娱乐,时尚,健康
健康,投资理财,美食
FEngineering,爱宠族,英语,金融,感性,文艺
交友,娱乐,休闲,创意,玩儿
美女,美食,IT 数码,文艺,体育,时尚
新闻资讯,时尚,教育就业,娱乐,军事
投资理财,旅游,时尚
投资理财
旅游,文艺,时尚,健康,星座命理,教育就业
体育,天蝎座
娱乐
星座命理,旅游,娱乐,美食
娱乐,美食
教育就業,新聞資訊,旅遊,美食,健康,文藝

汽车,IT 数码,美女,体育,娱乐
八卦杂谈
旅游,星座命理,投资理财,时尚,新闻资讯
爱笑,爱自由,睡懒觉,香橙,烹饪,视频,娱乐,星座命理,健康,美食
文艺
佛教,新闻资讯,电子商务,分析师,互联网,媒体,广告,网络营销,汽车
投资理财,健康,星座命理,娱乐
时尚
娱乐,新闻资讯,游戏动漫,军事,体育
校园生活
娱乐
网购,微博控,美容护肤,逛街
时尚,旅游,教育就业,健康,IT 数码,文艺
时尚,旅游,美食,娱乐
IT 数码,汽车,游戏动漫,文艺,体育,娱乐
体育,IT 数码
时尚,文艺,娱乐,旅游,美食
穿衣美容,旅游
美食
无厘头,神经质,看电影,魔羯座,媒体人,旅游,文艺
IT 数码,时尚,旅游,美食
旅游爱好者,星座命理
娱乐,旅游
文艺,娱乐
军事,文艺,教育就业,游戏动漫
穿衣美容,星座命理,新闻趣事,健康,旅游,美食,时尚,文艺
陕西生活,微博奇葩
教育就业,美食
美女,汽车,健康,旅游,美食,教育就业,娱乐
时尚,文艺,投资理财,教育就业,健康,美食
娱乐
娱乐,旅游,时尚,文艺
文艺
娱乐,体育
星座命理
投资理财,教育就业,汽车,IT 数码,时尚
教育就业,美女,旅游
娱乐,旅游,美食,新闻资讯,健康
时尚,健康,美食,旅游,文艺
星座命理
美女,汽车
名人明星
美女,新闻资讯
文艺,游戏动漫,娱乐

旅游,健康,体育,IT 数码,时尚
健康,旅游,星座命理,文艺,体育
娱乐
美食,汽车,旅游,IT 数码,娱乐
娱乐
IT 数码,美食
搞笑幽默,娱乐,IT 数码
美女
旅游,美食,文艺
其他生活
美食,新闻资讯
体育
本溪生活,微博奇葩,美女,美食,汽车,旅游,IT 数码,文艺,娱乐
旅游
时尚,投资理财,星座命理,美食,文艺,娱乐
美食
旅游
云南旅行社,云南导游,云南旅游
健康,旅游,娱乐
汽车
文艺,娱乐,游戏动漫,美食
旅游,健康,文艺,美食,体育
搞笑幽默,旅游,穿衣美容,萌宠,美女
视频音乐,90 後,0 型血,唱 K,互粉,文艺
山西生活,健康
美食,健康,美食
吃货
星座命理,美女,健康,美食,时尚
娱乐,美食,旅游
美食,穿衣美容,微博奇葩,宁波生活,娱乐,新闻资讯,时尚,健康,旅游
星座命理,篮球,喝茶
新闻资讯,教育就业
文艺,旅游,娱乐,美食
健康
健康,星座命理,旅游,美食,体育
文艺,星座命理,美食,健康,风采展示,成功之路,人物访谈,艺术设计,教育就业,旅游
美食,军事,时尚,健康,旅游
时尚,美女,汽车,宅
广播
娱乐,时尚,文艺,健康,IT 数码
教育就业
生活,魔兽世界,历史,军事
IT 数码,娱乐,教育就业
健康,文艺

美女,IT 数码
微博奇葩,视频音乐,体育,IT 数码,娱乐
旅游,文艺,娱乐,体育,教育就业,游戏动漫
娛樂
投资理财,娱乐
健康,娱乐
旅游,游戏动漫,IT 数码,娱乐,星座命理
汽车,时尚,美食,IT 数码,娱乐
娱乐,时尚,美食,文艺,星座命理
教育就业
时尚,游戏动漫,旅游
星座命理
投资理财,时尚,健康,旅游,美食
体育,游戏动漫,星座命理
美女,美食,军事
汽车,文艺,IT 数码,娱乐,体育
搞笑幽默
星座命理
文艺,时尚,娱乐
美女,投资理财,新闻资讯,军事,IT 数码
旅游,美食,军事,文艺,体育
文艺,体育,娱乐,美女
美食,汽车,投资理财,时尚,健康,旅游
旅游,穿衣美容,搞笑幽默,陕西生活,名人明星
文艺
搞笑幽默,视频音乐,读书分享,新闻趣事,健康,教育就业,新闻资讯,文艺,美食
时尚,旅游,文艺,投资理财,游戏动漫,军事
文艺
娱乐,游戏动漫,军事,体育
新闻资讯,美女,旅游,星座命理,美食
星座命理,汽车,美女
美食
娱乐,时尚,健康,美食,旅游
游戏动漫,娱乐,美食
吃,吃东西,喜欢热闹,交朋友
文字,吃货,幽默,交友,学生一枚,迷糊,夜猫子,美食,星座命理,美女
新闻趣事,投资理财,汽车,健康,军事
健康,旅游,美食,文艺
旅游
时尚,旅游,美食,星座命理
Exo 组合,EXOM,exo,青春,美女,时尚
文艺
旅游
文艺

新闻资讯,投资理财,军事,美女,游戏动漫,娱乐,体育,文艺
旅游
教育就业,新闻资讯,健康,旅游,美食
健康,旅游,时尚,体育
美女,萌宠
美女,游戏动漫,体育
体育
汽车,教育就业,投资理财
时尚,教育就业,体育
美女
时尚,健康
美食,教育就业,投资理财,新闻资讯,旅游
体育,IT 数码
韩国,矛盾,街舞,咖啡,幻想,听音乐,旅游,美食,独立，,阳光
K 歌，写作，做梦
文艺,小太阳,良民一枚,小哇,钟汉良,旅游
游戏动漫
健康,美食,文艺
美女
旅游,美食,穿衣美容
微博奇葩,星座命理,视频音乐,名人明星,搞笑幽默,bigbang,漫画,紫色,星星,娱乐
健康,时尚,旅游,美食,文艺
健康,旅游,美食
太极,健身,茶,投资理财,运动,健康
娱乐
投资理财,教育就业,军事,体育
星座命理,IT 数码,新闻资讯,健康,旅游,美食
健康,时尚,美食
教育就业,旅游,星座命理,美食,文艺
投资理财,汽车
投资理财,教育就业,健康,新闻资讯,文艺
时尚,星座命理,旅游,美食,健康
韩国,美女,时尚,健康
投資理財,搞笑幽默,投资理财,游戏动漫,星座命理,文艺,娱乐
臭美,小清新,学生一枚,爱笑,萌宠
名人明星,健康,旅游,文艺,娱乐,美食,游戏动漫,教育就业,IT 数码
美女,汽车
投资理财
星座命理,文艺,IT 数码
蓄电池,铅酸蓄电池,胶体蓄电池,健康,时尚,娱乐
文艺,教育就业,新闻资讯,旅游
体育
汽车,美女
健康,游戏动漫,时尚,汽车,投资理财,旅游,娱乐

微博奇葩,视频音乐,搞笑幽默,新闻趣事,云南生活
新闻趣事,新闻资讯
旅游
时尚,健康,星座命理,娱乐,美女,美食
我就是我,感性,强迫症,五月天,胡思乱想,睡觉睡到自然醒,music,囧
旅游,漳州生活,教育就业,军事,文艺
旅游
新闻资讯
琥珀原石,琥珀,蜜蜡,星座命理,美食,旅游,体育,娱乐
休闲,时尚潮流,冷笑话,玩儿,喜欢音乐,搞笑幽默
健康
搞笑幽默,旅游,穿衣美容,文艺
旅游
叫声凡哥哥俩好
爱生活,执着,简单,旅行,自由
八卦杂谈,名人明星,搞笑幽默,洛阳生活,宅女一枚,旅游,健康
香港,培训,活泼开朗,努力
新闻资讯,汽车,体育,IT 数码,冷暖自知。
美女
新闻资讯,军事,投资理财,游戏动漫,IT 数码,娱乐
美女,志愿者,水瓶座,乒乓球,爱音乐,爱旅游,o 型血,打篮球,旅游
娱乐
健康,时尚,投资理财,新闻资讯,文艺,美食
体育资讯,游戏动漫
健康
文艺,健康,时尚,新闻资讯,旅游
新闻资讯,汽车,美食,IT 数码,娱乐
文艺,旅游,游戏动漫
新闻资讯,旅游,体育
娱乐
羽绒服精品推荐,正品名牌羽绒服,羽绒服女款,羽绒服品牌大全,羽绒服背心,羽绒服
美女
美食,健康,时尚,投资理财,文艺
旅游,投资理财,体育,健康,游戏动漫
游戏动漫,旅游,汽车,美女,时尚
旅游,星座命理,美食,新闻资讯,教育就业
时尚
文艺
汽车,投资理财,旅游,文艺,美女,星座命理
爱好是打羽毛球,爱运动,喜欢跳舞,文学,喜欢唱歌,双子座
旅游,星座命理,微博奇葩,新闻趣事,教育就业,投资理财,美女,健康,军事
美食
军事,汽车,投资理财,健康,体育
教育就业,新闻资讯,健康,旅游,娱乐

健康,星座命理,旅游,美食,娱乐
時尚
健康,投资理财,旅游,文艺,娱乐
汽车
教育就业
美食
美女
美食
军事,美食,IT 数码
文艺,美食,旅游,新闻资讯,教育就业,娱乐
IT 数码
娱乐,旅游
微博奇葩,文艺,投资理财,游戏动漫
山东生活,新闻资讯,青岛六十六中,内高班,教育就业
健康,星座命理,游戏动漫,文艺
旅游,健康,新闻资讯,文艺,娱乐
时装,双子女,多愁善感,音乐达人,八卦,投资理财,时尚,星座命理,美食,娱乐
健康,旅游,美食
努力,爱自由,投资理财,新闻资讯,汽车,文艺
冷笑话,旅游美食爱好者,娱乐,美食
时尚,健康,美女
汽车
投资理财,思考,教育就业,旅游,军事,体育
新闻趣事,阳光,思考,纠结
旅游,美女,汽车,美食,娱乐
名人明星,游戏动漫
美女
唱歌,美食,星座命理,时尚,娱乐
健康,星座命理,旅游,娱乐,时尚
时尚,健康,娱乐,星座命理,美食,教育就业,文艺,旅游
视频音乐,娱乐,时尚
美图摄影,星座命理
时尚
旅游,星座命理,美食,军事,文艺
游戏动漫,IT 数码
星座命理,文艺,军事,旅游,游戏动漫
游戏动漫,新闻资讯,时尚,体育,IT 数码
健康,IT 数码,新闻资讯,星座命理,旅游
美女
体育,美食,游戏动漫
体育,文艺,健康,旅游
北京生活,简单,北京,矛盾体,懒,爱旅游
教育就业,旅游,美食,IT 数码,文艺
娱乐,新闻资讯,美食,教育就业,游戏动漫

星座命理,时尚,娱乐,文艺
吃喝玩乐,时装,音乐迷,设计,fashion,外贸,玩,美食控,生活,网购
搞笑幽默,美女,IT 数码,旅游,视频音乐
新闻资讯,美食
汽车,美食,时尚
搞笑幽默,汽车,时尚,新闻资讯
旅游,美食,文艺
健康,旅游,星座命理,美食
旅游,文艺,星座命理,美女,教育就业,投资理财,健康,新闻资讯
文艺
美女,投资理财,文艺,健康,星座命理,娱乐,军事,汽车,旅游,美食
健康,旅游,星座命理,娱乐,文艺
时尚
美女,健康,旅游,美食,娱乐
美女,时尚,娱乐,旅游,美食
星座命理,投资理财,健康,娱乐,汽车
星座命理,时尚
曼联,移动互联网,台球,手机,天下足球,军事,美食,游戏动漫,体育
新闻资讯,美女,投资理财,IT 数码,体育
搞笑幽默
健康
名人明星,体育
教育就业
星座命理,美食,娱乐,旅游
广告,减肥,爱美食,健康,旅游,美食,IT 数码,时尚,美女
时尚,教育就业,健康,旅游,美食
教育就业,时尚,文艺,娱乐,美食,旅游
美食,投资理财,健康,新闻资讯
体育
体育,健康,汽车,旅游,美食
读书分享,美图摄影,新闻趣事,智立方,think3,淘宝,杨石头,正能量,营销,创意
投资理财,娱乐,军事,文艺,健康
汽車,時尚,遊戲動漫,旅遊,文艺,体育,娱乐
教育就业,时尚,健康,星座命理,文艺,娱乐
沈阳生活,美食,军事,游戏动漫,星座命理,旅游
天平座,娱乐,时尚,旅游,星座命理,美食
美食,新闻资讯
美女
旅游,美食
玩,睡觉,幽默,语录,媒体,情感,吃喝玩乐,爱音乐,孩子气,开心
娱乐,军事,健康,文艺,新闻资讯,体育,教育就业
旅游,健康
搞笑幽默
投资理财

美食,微博奇葩,常州生活,新闻趣事,穿衣美容,文艺
时尚,星座命理,娱乐,文艺
文艺,美食,星座命理,旅游
IT 数码,汽车,体育,娱乐,音乐,HIPHOP,北京地铁,巨蟹座,90 后,游戏动漫
娱乐
历史控,游戏迷,白羊座 B 型血,银魂党,IT 互联网
体育
时尚
星座命理,文艺,旅游,美食,健康
美食,旅游
健康,旅游,新闻资讯,投资理财,教育就业
重口味
文艺,体育,健康,美食,旅游
娱乐
教育就业,文艺,健康
星座命理,旅游,健康,美食,娱乐
IT 数码
汽车,时尚,健康,旅游,美食
新闻资讯,户外,天蝎座,时尚潮流,品牌策划,传媒人,旅游,星座命理,美食,文艺
游戏动漫,美食,旅游,娱乐,懒人一枚,吃货一枚,情绪化,神经大条
新闻资讯
随性,新闻资讯,健康,旅游,文艺,投资理财
IT 数码
旅游
IT 数码,吃喝玩乐,羽毛球爱好者,摄影爱好者
文艺,旅游,美食,美女,时尚,娱乐
哈密生活,视频音乐
旅游,教育就业,IT 数码,文艺,娱乐
美食
新闻资讯,IT 数码
投资理财,汽车
文艺,美食
时尚
美女
休闲,文艺,新闻资讯,健康,汽车,旅游,美女
星座命理,美男控
娱乐,投资理财
星座命理,IT 数码,新闻资讯,健康,军事,娱乐
IT 数码,健康
健康,IT 数码,美食,游戏动漫,娱乐
美女
旅游,美食
美食,娱乐
IT 数码

美女
漫画控,绘画党,爱自由,萌宠
发呆,看电影,军事,文艺,投资理财,新闻资讯,健康
大学,喜欢唱歌,心情,真诚,青春,美女,投资理财,娱乐,IT 数码,汽车
游戏动漫,时尚,汽车,IT 数码,娱乐
健康,娱乐,美食,旅游
成都生活,新闻资讯
游戏动漫
旅游,美食
时尚,娱乐,美食,IT 数码
旅游,军事,IT 数码,体育
时尚
游戏动漫
投资理财,娱乐,美食,旅游
视频音乐,娱乐
汽车,健康,体育,美女,旅游
汽车,健康,新闻资讯,文艺,体育,娱乐
旅游,新闻资讯,美食,文艺,淘宝控,愤青,大叔控,诗词,主持,喜欢音乐
美食,旅游,娱乐,文艺,IT 数码
美甲,美睫,医学美容,整容,微整形,美女,时尚
投资理财,健康,星座命理,军事,美食
旅游,游戏动漫
汽车,旅游,IT 数码,星座命理
星座命理,娱乐
美女
文艺
时尚
旅游
美食
旅游,体育
旅游,新闻资讯,教育就业,军事,文艺,体育
南京生活,游戏动漫,IT 数码,文艺,娱乐
汽车,时尚,新闻资讯,健康,旅游
教育就业,新闻资讯
游戏动漫
游戏动漫,娱乐
汽车,旅游,美女,美食,体育
汽车,旅游,健康,美女,美食
美食,旅游,游戏动漫,健康,IT 数码
教育就业,健康,美食,投资理财,时尚
娱乐
新闻资讯,健康,体育,娱乐,汽车
新闻资讯,汽车,体育,教育就业,旅游,军事,IT 数码
军事

健康,教育就业,旅游,星座命理,美食
教育就业,新闻资讯,美食
美女
星座命理
健康,汽车
萌宠,穿衣美容
严重欧美帅哥控,小任性,欧美男神,超级完美主义,爱纠结,幸福 ing
娱乐
汽车,新闻资讯,投资理财,IT 数码,教育就业
书,吉他,咖啡,爱唱歌,自行车,文字,旅行,好书,驴友
新闻趣事,新闻资讯,投资理财
IT 数码
美食爱好者,cosplay
时尚,旅游,健康
教育就业
时尚,教育就业,娱乐,文艺,体育
美女,美食,健康
文艺
时尚,旅游,美食,健康
旅游,健康,娱乐,美女,美食,游戏动漫
汽车,美女,IT 数码
微博奇葩
投资理财,游戏动漫,美女,军事,星座命理,新闻资讯,健康
文艺,美食,时尚,体育,娱乐
健康,新闻资讯,时尚,文艺,旅游
新闻趣事,新闻资讯,IT 数码,娱乐,文艺
旅游,娱乐
美食
星座命理,IT 数码,旅游,娱乐,文艺,时尚
旅游,娱乐
游戏动漫,娱乐,军事
时尚,文艺,美女,娱乐
教育就业,时尚,健康,旅游
大楷榜书书法,用汉语记忆单词,英语毛笔书法,观念作家,文艺,教育就业
LOL,游戏动漫,体育
军事,健康,美食,IT 数码,娱乐,体育
旅游
娱乐
北京生活,微博奇葩,新闻趣事,搞笑幽默
娱乐,文艺
创业,展会,品牌加盟,早教加盟,餐饮加盟,项目推介会,深圳创业展,深圳加盟展,新闻资讯,
教育就业
勒个笨蛋!
体育资讯,汕头生活

美食,娱乐
投资理财
文艺
体育,游戏动漫,美女,IT 数码,军事,汽车
娱乐
旅游,爱旅行,狗狗,爱唱歌,护肤,文艺
美食,星座命理,新闻资讯,投资理财,美女
美食,军事,体育
军事,美食,娱乐
湖北生活,微博奇葩,美女,新闻趣事,娱乐,新闻资讯
搞笑幽默,旅游,北京生活,体育,军事,IT 数码,汽车,投资理财
美食,旅游,美女,星座命理,新闻趣事
旅游,新闻资讯,军事,文艺,健康
萌宠,穿衣美容,星座命理,旅游,美食,娱乐
军事
时尚,娱乐
游戏动漫,旅游,文艺,体育,娱乐
健康,时尚,新闻资讯,美食,体育,娱乐,旅游
萌宠,美食,新闻趣事,星座命理,搞笑幽默
体育,旅游,美女,游戏动漫,娱乐
汽车,星座命理,IT 数码,投资理财,军事,教育就业
美食,旅游,健康,美女,投资理财
健康,看书,娱乐,音乐迷,冷笑话,文艺
娱乐,时尚,旅游,文艺,美女,教育就业,星座命理,健康,汽车,军事
科幻,旅游,大学,校园,减肥
文艺,美女,娱乐,汽车,体育
名人明星,微博奇葩,美女,新闻趣事
搞笑幽默,娱乐
时尚,娱乐
情感生活
星座命理,体育
教育就业,星座命理,旅游,美食,娱乐
星座命理
书法,爱吃
美食
文艺,时尚,健康,旅游,美食
爱情,看书,汽车,音乐爱好者,90 後,打篮球,狮子座,旅游
旅游
旅游,美食,娱乐,体育
分享,文艺,教育就业,旅游,健康,投资理财
法律,历史,思考,文字,看书,新闻资讯,文艺
体育,游戏动漫
体育
美食,投资理财,健康,旅游

美食,文艺,娱乐,投资理财,教育就业
美食
美食,旅游
涂鸦
可爱,喜欢音乐,开朗,减肥,美容,八卦,语录,美食,时尚,健康
娱乐,美女
汽车,旅游
美女,游戏动漫,体育,娱乐
搞笑幽默,娱乐,文艺
汽车,体育,娱乐,时尚,美食,IT 数码
星座命理,娱乐,教育就业,健康,美女
时尚,旅游,IT 数码
游戏动漫,星座命理
健康,教育就业,汽车,体育,文艺
星座命理,军事,新闻资讯,娱乐,健康
健康,投资理财,美食,旅游
汽車
美女,游戏动漫,军事,星座命理,IT 数码
文艺,娱乐,星座命理
汽车,旅游
教育就业,星座命理
美图摄影,娱乐
美女,军事,IT 数码,汽车
时尚,IT 数码,文艺,娱乐,汽车,旅游
海绵宝宝,美女,汽车
美食,新闻资讯,教育就业
新闻资讯,投资理财,教育就业
健康,教育就业
娱乐,星座命理
美图摄影,新闻资讯
体育
美食,文艺,IT 数码,健康,旅游
搞笑幽默,微博奇葩,美食,旅游
强迫症,睡觉睡到自然醒,萌宠,游戏动漫,旅游
健康,美食
简单
美女
军事,游戏动漫,文艺
投资理财,星座命理,文艺,美食,旅游
体育,IT 数码,美女
搞笑幽默,汽车,时尚
娱乐,时尚,美女,旅游,美食
娱乐,旅游,IT 数码
娱乐

时尚,投资理财,星座命理,健康,美食
IT 数码
文艺,汽车,时尚,美女,健康
文艺
娱乐,新闻资讯,IT 数码,军事
自由
娱乐,美女,旅游,军事
星座命理,游戏动漫
美女,游戏动漫,旅游,体育,明基模力女孩,星座命理,汽车,IT 数码,娱乐
星座命理,旅游,健康,美食
游戏动漫,娱乐
新闻资讯,娱乐
健康,娱乐,文艺
IT 数码,设计是一种态度,天枰座,实习,90 后,姿迷,小清新,美食
读书分享,健康,教育就业,文艺
视频音乐,校园生活,新闻趣事,美图摄影,星座运势
健康,星座命理,时尚,美女,美食,投资理财
文艺
书,咖啡,佛教,爱情,阅读,美剧,好性格,创业,旅游,新闻资讯
健康
吃,名人明星,文艺,动漫宅,卖萌给你看,颜文字,导演,演员
美女
娱乐,旅游,文艺,美食
旅游,新闻趣事,汽车,IT 数码
努力,大学生,旅游
时尚,美女,旅游,娱乐
旅游
搞笑幽默,视频音乐,星座命理,旅游,文艺
新闻趣事,微博奇葩,时尚,旅游,健康,游戏动漫,美食
时尚,旅游,美食,文艺,娱乐
汽车,游戏动漫,旅游,体育
新闻趣事,八卦杂谈,搞笑幽默,IT 数码,游戏动漫,健康,美女,时尚,各种不会职业者
奋斗,学生一枚,90 後,狮子座,校园,体育
旅游,汽车,军事
娱乐,美食
新闻资讯,教育就业,军事,娱乐,文艺
有点宅有点懒,白羊座 B 型血
美女,旅游,美食,文艺,体育
体育,娱乐,美女
旅游,投资理财,娱乐,IT 数码,时尚,教育就业
投资理财
娱乐
穿衣美容,娱乐
旅游,健康,文艺,IT 数码

娱乐,健康,旅游
时尚,旅游,IT 数码
娱乐,时尚
其他生活
汽车
新闻资讯,军事,IT 数码,娱乐,美食,教育就业,美女,体育
新闻资讯
美女,LED 显示屏,创业,爱情,好性格,阅读,读书
娱乐,旅游
旅游,新闻资讯,美女,美食,军事
美食
教育就业,旅游,军事
美食,文艺,游戏动漫
健康,旅游,美食,娱乐,体育
健康,美食,文艺,时尚,旅游
微博奇葩,新闻资讯
旅游
娱乐,游戏动漫,投资理财
文艺,娱乐,星座命理
旅游,美食
阅读,服饰,漫画,美食,教育就业,时尚,健康,娱乐
新闻资讯
娱乐,文艺
旅游,娱乐,美食
天水生活,文艺,健康
台州生活
体育,IT 数码,星座命理
娱乐,游戏动漫,健康,IT 数码
旅游,投资理财,健康,娱乐
娱乐
健康
汽车,体育
美女,IT 数码
体育,娱乐
旅游,健康,新闻资讯,时尚,汽车
美食
体育,投资理财,旅游
教育就业,健康,旅游,美食,娱乐
旅游,美食
营销,音乐,篮球,NBA,登山,美女,美食,旅游,娱乐,体育
旅游,教育就业,时尚,健康,美食
健康,旅游,美食
文艺
美食,投资理财,旅游,健康,新闻资讯

旅游
文字控,交朋友,星座命理,美食
体育
星座命理,美食
90 后非脑残,美食,游戏,音乐
手链控,项链控,脚链控,耳环控,饰品控,时尚
微博奇葩,旅游,搞笑幽默,新闻趣事,国际足球,文艺,健康,新闻资讯,娱乐,体育
教育就业,星座命理,体育,娱乐,健康,汽车,美食
萌宠,90,麦霸,爱吃,影视,打篮球,游戏动漫
时尚,健康,旅游,美食,娱乐
旅游,健康,娱乐,星座命理,汽车
文艺,新闻资讯,健康,汽车,投资理财
时尚,健康,文艺,娱乐
投资理财
体育,娱乐
新闻资讯,娱乐,健康,美食,时尚
旅游
游戏动漫,美女,旅游,体育
时尚,旅游,美食,文艺,娱乐
健康,旅游,军事,IT 数码,文艺
教育就业,投资理财,旅游,IT 数码,体育,汽车
旅游,星座命理,文艺,投资理财,娱乐
美女
游戏动漫,体育,教育就业,时尚,星座命理,军事,健康,汽车,美食
健康,旅游,时尚,娱乐,文艺
游戏动漫,美食,旅游,星座命理
美图摄影,体育资讯,新闻趣事,旅游,IT 数码,时尚,新闻资讯,健康,娱乐
星座命理,娱乐
新闻资讯,体育,旅游,军事
IT 数码
投资理财
汽车,美食,军事,IT 数码,文艺
新闻资讯,汽车
投资理财,时尚,娱乐,美食,星座命理
文艺,时尚,美食,娱乐,星座命理
美食
培训,留学,日语,幸福,茶,教育就业,美食,娱乐,体育
教育就业,时尚,旅游,美食,娱乐
星座命理
美女,娱乐,汽车
星座命理
美食,教育就业,投资理财,健康,时尚
体育
娱乐,文艺

搞笑幽默,美女,投资理财,汽车,健康,旅游,IT 数码
旅游
时尚,美食
娱乐,电视,博客,听音乐,韩剧,管理,爱美食,社会,上网
星座命理
dido,写作,IT,古典音乐,烹饪,孙燕姿,游戏动漫,美食,旅游,健康
美女,历史,时评,教师,教育就业,愤青,新闻资讯
美食,旅游,时尚
体育,旅游,美食
健康
星座命理,文艺
游戏动漫
文艺
旅游
新闻资讯,投资理财,时尚,健康,旅游
文艺
投资理财
砭石,泗滨,保健,饰品,养生,旅游,健康,美女,时尚,文艺
娱乐,IT 数码,汽车,健康,美食,游戏动漫,教育就业,军事
旅游,文艺
游戏动漫,美食,文艺,娱乐,IT 数码
新闻资讯
娱乐,汽车
IT 数码,新闻资讯,旅游,健康
旅游,教育就业,健康
动漫
新闻资讯,教育就业,时尚,健康,文艺
美食
娱乐,旅游,投资理财,时尚,文艺
文艺,新闻资讯,旅游,健康,美食
爱旅游,爱电影,会计专业,偶稀饭睡觉觉,白羊座 B 型血,90 后不张扬,教育就业,健康,旅游,娱乐
读书分享,美容服饰,新闻趣事,名人明星,搞笑幽默
CHARLES,天秤,90
投资理财,健康,旅游,文艺,美食
搞笑幽默,陕西生活,文艺
军事,投资理财,美女
时尚,旅游,美食
汽车,美食
体育
爆料,奇葩,骗子
时尚,星座命理,IT 数码,娱乐
体育,IT 数码,旅游,时尚,文艺,娱乐,投资理财,健康,美食
旅游

投资理财,教育就业,旅游,新闻资讯,星座命理
体育
时尚,娱乐,体育,文艺,IT 数码,游戏动漫
汽车,时尚,IT 数码
吃货一枚,大学生,偶稀饭睡觉觉,听音乐,90 後
微博奇葩
热爱生活,教育就业
东营生活,文艺,投资理财,教育就业,新闻资讯
写作,自然,自由职业,好书,文艺,旅游
娱乐
健康
名人明星,旅游,美食,苏州生活,视频音乐,IT 数码
名人明星,娱乐
美女
临汾生活
娱乐,星座命理
穿衣美容,福建生活,时尚
星座命理,健康,教育就业,娱乐,时尚
健康,旅游,美食
美食,娱乐
投资理财,娱乐,新闻资讯,时尚,汽车
旅游,美食,游戏动漫
IT 数码,娱乐,体育
时尚,美食,娱乐
巧克力,执着,爱时尚,爱漂亮的天枰女,内心脆弱,需要一个他,无敌便利帖,IT 数码,娱乐,星座命理
健康,时尚,旅游,星座命理,文艺
美食,新闻资讯
健康,星座命理,美食,文艺,旅游
健康,美食,旅游,星座命理,娱乐
体育,星座命理,娱乐,文艺
美食
舞蹈,销售,80 后的尾巴,喜欢音乐,淘宝控,柯南,美女,汽车,时尚,旅游
微博奇葩,视频音乐,搞笑幽默,投资理财,娱乐,体育
美食,娱乐
娱乐
旅游,美女,军事
新闻资讯
美女,时尚
娱乐,汽车,星座命理,旅游,健康
汽车,文艺,健康,投资理财,教育就业,旅游,体育
旅游,美食,娱乐
旅游,陈奕迅,新闻资讯,美食,文艺,娱乐
时尚

新闻资讯,汽车,旅游,文艺,体育
体育,汽车,时尚,IT 数码,娱乐
文艺,旅游
教育就业
星座命理
旅游,健康
新闻资讯,军事,投资理财,健康
健康,旅游,美食,IT 数码,娱乐
美女
旅游
浙江生活,微博奇葩,名人明星,搞笑幽默,新闻趣事
文艺,健康,教育就业,娱乐
新闻资讯,旅游,投资理财,健康,IT 数码
旅游
投资理财,旅游,文艺,体育,教育就业
传媒,财经,语录,购物,新闻趣事,旅游,美食
健康
教育就业,投资理财,新闻资讯,健康,时尚,高级心理咨询师
美食
游戏动漫
天蝎座,看小说,爱美食,美容,文艺,美食,旅游,游戏动漫
文藝
IT 数码,军事
汽车
美女,汽車,文藝,健康
摄影,旅游,体育
时尚,娱乐,星座命理,美食,健康,汽车,美女,投资理财,军事
视频音乐
体育,旅游
健康,星座命理,IT 数码,娱乐,时尚
军事
健康,投资理财
美女
美女,时尚
投资理财
美食
文艺,美食,娱乐,旅游
新闻资讯,星座命理
健康,旅游,新闻资讯,时尚,文艺
美食,90 後
旅游,文艺
美食,军事,IT 数码
健康,旅游,教育就业,文艺,体育
宣城生活,美食,健康

美女,搞笑幽默
穿衣美容,上海生活,健康,美食,时尚,娱乐
健康
娱乐
雙子座,爱自由,90後,学生一枚,笑,文艺,旅游,美食
娱乐,美食,时尚
汽车
文艺,美食,健康,时尚
健康,教育就业,旅游,文艺
时尚,旅游
娱乐,游戏动漫,旅游,星座命理,美食
美食
旅游,健康,时尚,投资理财,美食
美容,浪漫,交朋友,家居,结婚用品,电子喜帖,婚纱摄影,婚礼筹备,网购,时尚
美食
新闻资讯,时尚,文艺,IT数码,娱乐
新闻资讯,时尚,投资理财,旅游,健康
文艺
体育,娱乐
娱乐,星座命理,新闻资讯,美食,健康,文艺
IT数码,体育,新闻资讯,游戏动漫,美女
文艺
娱乐
幸福,中医,青春,爱吃,旅游爱好者,古典音乐,小说迷,无厘头,努力,活泼开朗
旅游,美食
幸福,经济,服装
视频音乐,微博奇葩,搞笑幽默,美食,体育
星座命理
投资理财
投资理财
看书,上网,娱乐
汽车,教育就业,投资理财,娱乐
星座命理,旅游
美食
美食,旅游,美食,娱乐,教育就业,健康
娱乐,游戏动漫
粉红控,孩子气,美剧迷,大学生,矛盾,音乐,水瓶座加O型血,读书,动漫
IT数码
时尚,文艺,娱乐
穿衣美容,旅游,美食
美食,旅游,体育,时尚
IT数码,旅游,娱乐,新闻资讯,健康,时尚
新闻资讯
投资理财,汽车,健康,旅游,娱乐,教育就业,美食

低碳交通,低碳城市,生态文明,城市文化,创新型城市,科技创新与政策,科学技术与社会
动漫控,iphone,睡觉睡到自然醒,星座命理,健康,美食
时尚,IT 数码,文艺,娱乐
汽车,文艺,娱乐,健康,旅游
美女,娱乐
教育就业
旅游
新闻资讯
游戏动漫
北京生活,健康
娱乐,唱歌,购物狂,网购,生活,吃,大学生,时尚潮流,逛街,护肤
健康,美女,文艺,体育
军事,新闻资讯,IT 数码,文艺,投资理财
游戏动漫,旅游,美食,IT 数码,体育
江苏生活,旅游,穿衣美容,美女,美食,交友,吃货,宅女,娱乐
美女,游戏动漫,IT 数码,文艺,体育
旅游,新闻资讯
军事,美女,时尚,文艺,美食,旅游,投资理财
投资理财,旅游,新闻资讯,美女,娱乐
IT 数码,美食,旅游,娱乐,游戏动漫
旅游,汽车
时尚
旅游,健康
旅游
心理学,爱旅游,军事
旅游,美食,文艺
娱乐,体育
游戏动漫
文艺
汕头生活
旅游,娱乐,游戏动漫,美食,体育
时尚,旅游,星座命理,美食,娱乐
体育,美食
微博奇葩
吃,水瓶座,乐天派,80 后的尾巴,育儿,喜欢唱歌,乐观
游戏动漫,IT 数码,文艺,星座命理,时尚,娱乐,新闻资讯
旅游,美食,文艺
IT 数码
时尚
旅游,美食,娱乐,体育,时尚
美食,投资理财,时尚,教育就业,健康,星座命理
娱乐,文艺,美女
视频音乐,旅游,星座命理,搞笑幽默,神经大条,吃,水瓶女,阳光
IT 数码,文艺,美食,游戏动漫,娱乐

星座命理,美食,娱乐,游戏动漫
文艺
娱乐
美女,搞笑幽默,旅游,新闻资讯,军事,IT 数码
旅游,健康,娱乐,时尚,文艺
搞笑幽默,体育,美食,美女
时尚,汽车
娱乐
娱乐
星座命理
体育
IT 数码,军事
投资理财,活泼,快乐
体育,娱乐
旅游,美食,娱乐
健康
视频音乐,上海生活,美图摄影,美女,文艺,汽车,旅游,体育
体育,IT 数码,娱乐
健康,美女,IT 数码,军事,教育就业,新闻资讯,汽车,娱乐
时尚
旅游,娱乐
美食,文艺
游戏动漫,学生一族,90 后非脑残,幻想,游戏,狮子座,喜欢音乐
美女,新闻资讯,军事,文艺,健康,旅游,美食,汽车
吃货一枚,宠物,粉红控,玩
时尚,游戏动漫,旅游,IT 数码,娱乐
上海生活,玩中学,浦江镇,棒棒贝贝,儿童科技教育,搭建,积木,乐高
健康,星座命理,旅游,美食,娱乐
美食
投资理财,时尚,旅游,美食,体育
旅游,文艺
旅游
旅游,文艺
美食,旅游,微博奇葩,萌宠,搞笑幽默
IT 数码,文艺,娱乐
美女,美食,IT 数码,娱乐,军事
文艺,娱乐,美食
美食,旅游,健康,新闻资讯,军事
文艺,健康,旅游,美食
旅游
微博奇葩,旅游,美食,时尚,教育就业,投资理财
娱乐,星座命理,美食,旅游,IT 数码
游戏动漫
穿衣美容,旅游,视频音乐,搞笑幽默,汽车,投资理财,美食,时尚,健康

体育资讯
时尚,美食,健康,新闻资讯,汽车
星座命理
新闻资讯,IT 数码
名人明星,视频音乐,搞笑幽默,美女,汽车,IT 数码,娱乐,体育
新闻资讯,美食,娱乐,旅游,体育
搞笑幽默,军事,游戏动漫,体育
IT 数码
文艺,健康,星座命理,爱玩,乒乓球,无聊,音乐爱好者,电影控,完美主义,读书
军事
美食,文艺,娱乐
时尚,美食,娱乐,健康,旅游,文艺
摄影,翡翠,珠宝首饰,双子座,时尚,健康,旅游,美食,娱乐
美食,时尚,文艺,旅游,健康,娱乐
美食,游戏动漫,听歌唱歌,篮球,动漫,cos,腐宅,睡
娱乐
娱乐
娱乐,健康
汽车,旅游,美食,娱乐,体育
美女
体育,娱乐,旅游,美食,游戏动漫
星座命理
搞笑幽默,漫威,电影,金牛座,游戏动漫
IT 数码,汽车
美女
游戏动漫
旅游,星座命理,美食,文艺,娱乐
搞笑幽默,体育
汽车
文艺,汽车
名人明星,美女,视频音乐,搞笑幽默,娱乐,新闻资讯
体育,汽车,健康,旅游,美食,军事,时尚
校园生活,汽车,体育
美女,游戏动漫
旅游,军事,游戏动漫,汽车
时尚,游戏动漫,美食
美女
旅游,美食,文艺,体育,娱乐
娱乐,IT 数码
娱乐,美食,时尚,汽车,IT 数码
旅游,搞笑幽默,k 歌
教育就业,健康,时尚,文艺,娱乐
搞笑幽默,美食
水瓶座,电影,B

美女,旅游,星座命理,文艺
IT 数码
娱乐,健康
娱乐,新闻资讯,游戏动漫,文艺,星座命理
汽车
游戏动漫,文艺,旅游,美食,新闻资讯,娱乐
House,Music,DJane,Female,Sanja,DJ,音乐,健康,时尚,旅游
游戏动漫
旅游,美食,时尚
新闻资讯,军事,投资理财,教育就业,健康
美食,穿衣美容,新闻趣事,江苏生活,游戏动漫
时尚,旅游,文艺,新闻资讯,健康
健康
投资理财,旅游,新闻资讯,时尚,文艺
微博奇葩,星座命理,文艺,娱乐,IT 数码
娱乐
新闻资讯,投资理财,旅游,IT 数码,健康
星座命理,美食,投资理财,体育
美食
新闻资讯,军事,汽车
文字,胡思乱想,南京,文化,美食,文艺
娱乐
美女,美食
时尚,健康,旅游,文艺
旅游,美食,娱乐,星座命理
娱乐,星座命理
汽车
投资理财,旅游,IT 数码,体育,娱乐
拜仁
文艺
星座命理,IT 数码
奇怪女生
新闻资讯,教育就业,军事,体育,娱乐
青春,个性,神经大条,交友,笑,娱乐
三分之二观不正
体育,时尚
时尚,娱乐,旅游,文艺,健康
穿衣美容,体育
美食,娱乐,游戏动漫
星座命理,娱乐,旅游,美食
旅游
westlife,火影忍者,篮球爱好者,跑步,时事,电影爱好者,电影控,哈利波特,军事,体育
教育就业,星座命理,游戏动漫,军事,IT 数码
时尚,旅游,美食,健康,娱乐

娱乐
娱乐,投资理财,汽车
星座命理,旅游
美食,文艺
宅女一枚,90 後,娛樂,爱自由,广告,网络营销,宠物,互粉,喜欢听歌
名人明星
穿衣美容,健康,时尚
美食
美女,汽车,娱乐
体育,美女,美食,旅游,文艺,汽车
汽车,投资理财
汽车,娱乐,时尚,体育,星座命理
旅游,游戏动漫,健康,美食
游戏动漫,IT 数码,体育,娱乐
体育,新闻资讯,健康,文艺,娱乐
湖南生活
房产
健康,旅游,美食,星座命理,娱乐
美女,汽车,文艺,旅游,健康,新闻资讯,娱乐,体育
旅游,美食,游戏动漫,汽车,娱乐
学生,上虞,百度贴吧,上虞中学吧,校园
投资理财,IT 数码,汽车,美女
IT 数码
时尚,健康,旅游,美食,娱乐
新闻资讯,美食,IT 数码,娱乐,军事
名人明星,娱乐
文艺,投资理财,健康,教育就业,美食,旅游
五月天,宅女,唱 K,b 型血,书法,坚强,巨蟹座,文艺
美女,娱乐
时尚,旅游,星座命理
体育
投资理财,健康,体育,军事,IT 数码
穿衣美容
游戏动漫,体育
文艺,天蝎女,星座控,幻想,星座命理
旅游,时尚,IT 数码,娱乐
游戏动漫,时尚,投资理财,旅游,星座命理
微博奇葩,丝袜,胡思乱想,恋物癖,文爱,美女,星座命理,文艺,新闻资讯,娱乐
娱乐,游戏动漫,军事
文艺
IT 数码
健康
星座命理
娱乐

星座命理,新闻资讯,游戏动漫,体育,军事
旅游,文艺,教育就业,时尚,新闻资讯,健康
新闻趣事,广东生活,旅游,穿衣美容,时尚,娱乐,美食
IT 数码,汽车,教育就业,美食,投资理财
Geek,游戏机实用技术
汽车
健康,新闻资讯
新闻资讯,健康,美食,时尚,娱乐
汽车,教育就业,文艺,娱乐,时尚,健康,旅游,军事
IT 数码,娱乐
乒乓球,双子,古典艺术,文艺
游戏动漫,美食
美食,文艺,健康,投资理财,时尚
娱乐,军事,投资理财,新闻资讯,健康
网购,最高比率
美女
搞笑幽默,旅游
星座命理,体育,娱乐
学生一族,魏晨控,90 后,摩羯座
旅游,娱乐
娱乐
娱乐
文艺,IT 数码,时尚
教育就业
教育就业,投资理财
美女,体育
健康,新闻资讯,教育就业,投资理财,旅游
旅游,美食,健康
文艺,旅游
娱乐
美食,旅游
新闻资讯
巨蟹女,快乐大本营,南京,睡觉睡到自然醒,爱美食,我就是我,90 後,Music
旅行,80 后的尾巴,书籍,猫咪,无厘头,摩羯
美食
健康,教育就业,投资理财
汽车,旅游,体育
名人明星,吃喝玩乐,90,博爱者,2B,宅女加腐女
名人明星,时尚,星座命理,娱乐
健康,新闻资讯,娱乐
旅游,IT 数码
体育
新闻资讯,娱乐
旅游,军事,美女,汽车

投资理财
教育就业,投资理财,娱乐,美食,旅游,新闻资讯
我爱台球,爱睡觉
美食,健康,新闻资讯,教育就业
娱乐
文艺
汽车,游戏动漫,时尚,美食,文艺,娱乐
娱乐
旅游
好书,搞笑,运动,政治,o 型血,钓鱼,房地产,新闻资讯,个性,水瓶
娱乐,美食,游戏动漫
旅游,游戏动漫
健康,时尚,教育就业,投资理财
旅游,星座命理,健康
文艺
体育
星座命理,军事,健康,娱乐,美女
美食,文艺,美女,健康,旅游
娱乐,体育,文艺,IT 数码,军事
时尚,健康,美食,星座命理,军事,汽车
健康,美食
健康,旅游,体育
娱乐
旅游,娱乐,文艺,时尚,美食,健康
美女
美女,美食
娱乐,星座命理,游戏动漫
苦逼工科女,多愁善感,文艺,Music,娱乐
文艺,星座命理,健康,旅游,美食
投资理财,IT 数码,旅游,健康,军事
旅游,美食,星座命理,新闻趣事
星座命理
美食,旅游,IT 数码,时尚,娱乐
蓝色,摩羯女,简单,臭美,70 后,懒人,情绪化,文字控,发呆,淘宝
时尚,教育就业,美食,军事,体育
娱乐,IT 数码,体育
健康,文艺,体育,娱乐
健康,新闻资讯,投资理财,旅游,美食
军事,娱乐,体育,汽车,IT 数码
时尚
神经大条,美食
美食
娱乐,爱美食,歌唱,奋斗
星座命理,美食,军事,娱乐

旅游
健康,时尚,投资理财,旅游,美食
投资理财
美女,新闻资讯,美食,体育,时尚
星座运势,八卦杂谈,星座命理
时尚
汽车,健康,旅游,星座命理,美食
娱乐,星座命理
美食,健康,新闻资讯,投资理财,娱乐
汽车,美女,体育
新闻趣事,搞笑幽默,美食
美食,时尚,娱乐,文艺,旅游
IT 数码,文艺,军事,娱乐,新闻资讯
娱乐,新闻资讯,健康,美食,旅游,教育就业
御姐,文艺怀旧,英语日语粤语,购物狂,弟弟控,爱音乐,电影爱好者,欧美,旅游美食,教育就业
健康,娱乐
投资理财,时尚,健康,旅游,美食
健康,投资理财,星座命理,美食
投资理财
IT 数码,文艺,星座命理,美食,时尚,旅游
汽车
娱乐
健康,美女,娱乐
体育
天蝎女
美食
美食,新闻资讯,星座命理,游戏动漫,学生一枚,爱睡觉
健康,娱乐,星座命理,IT 数码,体育,美女
新闻资讯,体育,投资理财,美女,美食
旅游,健康,新闻资讯,文艺,投资理财
美女,广东生活
时尚,文艺
正太控,电视剧,爱睡觉,独立,大叔控,懒人,淘宝控,娱乐
画画,旅行,奋斗 ing,散步,爱唱歌,爱自由,孩子气,简单,吃货,金牛座
无厘头
旅游,美食,IT 数码,体育,投资理财
汽车,健康
美女,健康,IT 数码,文艺,时尚
旅游
湖南生活,活泼开朗,文艺
汽车
投资理财,新闻资讯,健康,娱乐
美女,时尚,星座命理,娱乐

闷骚,影视,好性格
教育就业,汽车,美女,旅游,健康
旅游,娱乐
格子控,高考,猫控,bigbang,巧克力,做梦,郭敬明,文艺,星座命理,美食
旅游,美食,IT 数码,文艺,体育
五月天,苏打粉
美食,星座命理
搞笑幽默
兰州生活,投资
娱乐,体育
美食
时尚,健康,美食
美食
娱乐,新闻资讯,投资理财,旅游,美食
娱乐,吃货一枚,music,自拍,花痴
时尚
旅游,健康,娱乐
健康
新闻趣事,微博奇葩,视频音乐,名人明星,教育就业,新闻资讯,娱乐
娱乐,IT 数码,旅游
游戏动漫,IT 数码
旅游,美食,娱乐
美食,北京生活,美女,视频音乐,旅游
健康
游戏动漫
旅游
体育,美女,娱乐
健康,娱乐,旅游,汽车
IT 数码
宅,唱歌,上网,睡觉,娱乐
美女
军事
新闻资讯
咸阳生活
旅游,美食,美女,投资理财,时尚
娱乐,文艺,游戏动漫,美食,星座命理
搞笑幽默,微博奇葩,时尚,美食
神经大条,朋友,自恋,IT 数码
旅游,游戏动漫,美食,汽车,IT 数码
娱乐,健康
美食,军事,IT 数码
美食
娱乐,时尚,教育就业,新闻资讯,美食
娱乐

游戏动漫,时尚,文艺,娱乐,体育
娱乐,体育
新闻资讯,健康
体育
健康,体育,美食,新闻资讯,旅游
汽车,美食,旅游,美女,娱乐
旅游,时尚,汽车,文艺
新闻资讯,娱乐,汽车,健康,美女,投资理财,美食
美食,健康,旅游,娱乐,时尚
美食
娱乐
体育
文艺,投资理财
健康,游戏动漫,美食,军事,新闻资讯,星座命理
美食
IT 数码
美食,军事
健康,军事,娱乐,体育,文艺
美食,健康
汽车,军事,IT 数码,体育,娱乐
汽车,新闻资讯,时尚,文艺,娱乐
娱乐,新闻资讯,美食,时尚,汽车,IT 数码,军事
文艺
暮光之城,吃货一枚,哈利波特,时尚,旅游,IT 数码,娱乐
军事,娱乐,健康,新闻资讯,美女,文艺
汽车,时尚,美女,游戏动漫,IT 数码
美食,娱乐
教育就业,汽车,星座命理,健康,文艺,娱乐
旅游
汽车
游戏动漫,美食,娱乐
娱乐,游戏动漫,星座命理
旅游
投资理财,新闻资讯,美食,健康,教育就业
星座命理
时尚
小说,娱乐
旅游,投资理财,汽车,美女,军事
搞笑幽默,体育
文艺,纹身刺青
时尚,美食,娱乐
美食,健康,旅游,新闻资讯,文艺
德国,拜仁慕尼黑,足球迷,美食,游戏动漫,旅游,军事,体育
新闻资讯,娱乐,文艺

新闻资讯,文艺,健康,教育就业
游戏动漫
BullCrap,音乐好,Crablover,新闻资讯,军事,IT 数码
时尚,星座命理,健康,教育就业,美食
娱乐,汽车,体育
时尚
美食,时尚,美女,体育,娱乐
文艺,美食,IT 数码,时尚,旅游
美食,娱乐
旅游
健康
时尚,健康,投资理财,旅游,文艺
美女,体育,旅游,IT 数码
投资理财,股票,网购,处女座,旅游美食爱好者,八卦,美食
时尚,美食,健康,文艺,旅游,美食
搞笑幽默,星座命理
体育,娱乐,教育就业,时尚,文艺
IT 数码,新闻资讯,健康,教育就业,军事
健康
投资理财
宅,IT 数码
穿衣美容,娱乐
旅游,健康,美女,时尚,娱乐
新闻资讯
美食,旅游
穿衣美容,爱旅游,爱电影,旅游爱好者
投资理财,游戏动漫,旅游,文艺
美食
美食,文艺,娱乐,健康,教育就业
旅游,星座命理,时尚,文艺,美食
旅游,时尚,健康,美食,娱乐
娱乐,旅游,健康
健康
美女,游戏动漫,IT 数码
美女,美食,体育
旅游,上海生活
穿衣美容,微博奇葩,北京生活,投资理财,教育就业,旅游,美食,文艺
体育,娱乐
军事
贵阳生活
旅游,新闻趣事,搞笑幽默,军事天地,车世界,游戏动漫,汽车,体育
平面设计,搞笑幽默
运动,乐观,活泼,单身,微笑,时尚
体育,健康,IT 数码,新闻资讯,文艺

娱乐,体育
时尚,健康,旅游,美食,文艺
美女,游戏动漫,美食,军事,IT 数码
娱乐,旅游
搞笑幽默
美女,娱乐,新闻资讯,投资理财,军事
汽车,军事,旅游,新闻资讯
星座命理,文艺
名人明星,新闻趣事,文艺,军事
游戏动漫,体育
微博奇葩
新闻资讯,军事,体育,IT 数码
美食,娱乐
名人明星,微博控,HelloKitty 控,唐嫣的糖蜜,学生一族,爱吃棒棒糖
旅游
文艺,时尚,旅游,娱乐
完美主义,喜欢旅游
广东生活
篮球,音乐,上网,旅游,体育,时尚,美食,娱乐
体育
体育,汽车,IT 数码
美食,旅游,娱乐
搞笑幽默,汽车
投资理财,星座命理,美食,IT 数码,文艺
游戏动漫,文艺,体育
教育就业,新闻资讯,投资理财,娱乐,健康
投资理财,文艺,时尚,健康,汽车,星座命理,美食
体育
汽车,美食,旅游,军事,娱乐,体育,健康,美女
娱乐
旅游,游戏动漫,新闻资讯,军事,体育
军事,旅游,健康,美食,体育
星座命理
健康,IT 数码,美女,娱乐,游戏动漫
娱乐
文艺
天枰座,自由,猫
娱乐
gay,音乐达人,紫色,宅,90 後,爱旅游,腐,活泼开朗,耽美，BL，宅，腐
娱乐,IT 数码,汽车
广州生活,健康
汽车,文艺,娱乐,旅游,美食
体育,文艺,娱乐,汽车,新闻资讯,星座命理,IT 数码
旅游,游戏动漫,IT 数码,娱乐,文艺

舞蹈教育,舞蹈家协会,舞蹈,教育就业,文艺,体育,娱乐
幸福,音乐爱好者
娱乐
坚韧,淡定,低调,猫控,extrover,银白色,爱音乐,90后,双子女,EXO
美食
投资理财,星座命理,军事,美女,健康
政治,书籍,人生,喜欢唱歌,教育就业,美食,健康,体育,娱乐
椰子,王野,文艺,IT数码,时尚,体育,娱乐
健康,美食,时尚,娱乐
双子女
高三党,新乡市三中,金牛座,90後,跳舞
教育就业
IT数码
强迫症,BIGBANG,处女座
美女,美食,文艺
娱乐
简单
新闻资讯,旅游,时尚
护肤,偶稀饭睡觉觉,天秤座,乐观,快乐,写字,听音乐,旅游,美食,健康
健康,时尚,旅游,美食,娱乐
旅游,健康,时尚
游戏动漫
娱乐,美食
新闻资讯,体育,文艺
军事,体育,娱乐
时尚,星座命理,文艺
军事,娱乐
IT数码,新闻资讯
美食,旅游,健康,游戏动漫,IT数码,美女
时尚,汽车,美食,娱乐
投资理财,时尚,文艺,星座命理,娱乐,美食
时尚,旅游
旅游
旅游,美食,娱乐,IT数码,汽车
健康,文艺,旅游
学生,ALL绵,ALL白,EXO,腐女
娱乐
旅游,星座命理,时尚,IT数码
军事,健康,IT数码,新闻资讯,娱乐,星座命理,美食
健康,新闻资讯,娱乐
时尚,IT数码,星座命理,娱乐,教育就业
文艺,新闻资讯,美食,旅游,健康
星座命理
校园生活,文艺,娱乐

旅游,汽车,军事,娱乐
文艺,娱乐
文艺,旅游,投资理财,时尚
别致優雅的瘋子,我亦漂零久,滿腦子漿糊,強迫症半治癒
旅游,穿衣美容,重庆生活,娱乐
高温刹车片润滑,刹车分泵润滑剂,电缆附件硅脂膏,管状电机润滑脂,小包装润滑脂,食品级润滑油脂,阻力油脂,硅脂,电机齿轮箱,润滑脂
游戏动漫
缘之空,Vintage,心声,文艺
星座命理,军事,游戏动漫,教育就业
体育,娱乐
美食,星座命理,视频音乐,旅游,穿衣美容,营销,亲情,好书,校园
娱乐
新闻资讯,美食,文艺,娱乐
体育
美女,投资理财,体育
文艺
美食
游戏动漫
汽车,IT 数码,体育,旅游,新闻资讯,军事
娱乐,美食
教育就业,文艺,健康,时尚,汽车
时尚
投资理财
教育就业,时尚,健康,新闻资讯,娱乐
体育
星座命理,疯疯癫癫,囧,赖床户
文艺,游戏动漫,旅游
会计,宅女一枚,水瓶女,睡懒觉,小清新,时尚,星座命理,旅游,投资理财,健康
旅游,美食,娱乐
美食,军事,娱乐
旅游
惠州生活,汽车,军事,娱乐,体育
新闻资讯,健康,旅游,娱乐,美食,星座命理
旅游,时尚,娱乐
娱乐,美女,汽车
时尚达人,热爱生活,电影发行,电影控,营销策划推广,IT 数码
娱乐,健康,时尚
IT 数码
水晶,宝石,琥珀,蜜蜡,玉石,健康,时尚,旅游,星座命理,体育
打篮球,90,火影,海贼王,音乐爱好者,新闻资讯,美女,旅游,体育,娱乐
宁德生活,娱乐,健康
IT 数码,星座命理,游戏动漫,文艺,娱乐
美女,娱乐,教育就业,新闻资讯,军事

游戏动漫,娱乐,星座命理
世勋,勋鹿,sehun,exo
健康,游戏动漫,旅游,星座命理,娱乐
文艺
旅游,美食,教育就业,健康,星座命理,娱乐
星座命理,文艺,娱乐
新闻趣事,旅游,投资理财,健康,文艺,时尚
简单,看电影,文艺控,
游戏动漫,体育,汽车
新闻资讯,旅游,星座命理,美食,教育就业
新闻趣事,微博奇葩,天津生活,视频音乐,旅游,时尚,IT 数码,文艺,娱乐
娱乐,游戏动漫,健康,教育就业,文艺,时尚
美食
时尚,汽车,美女,体育,娱乐
象棋,三体,美女
文艺,时尚,旅游,美食,娱乐
游戏动漫
教育就业,汽车,军事,投资理财,新闻资讯,健康,IT 数码
美食,旅游
美女,健康,旅游,娱乐
投资理财
火锅,高考,医学,心理学,科幻,卖萌,美食
新闻资讯
我爱米月月
文艺,投资理财,旅游,美食
美食,体育
军事
娱乐
吃喝玩乐,巧克力,90 後,活泼,生活,搞笑,吃货,旅游,汽车,美食
旅游,汽车
B 型血,环保,搞怪,文化,坚强,东营生活
健康,投资理财,娱乐
新闻资讯,教育就业,时尚,美女,旅游,军事,IT 数码
体育
星座命理,旅游,IT 数码,美女
星座命理,文艺,美食,IT 数码,娱乐
科学探索,VINTAGE,时尚,娱乐
时尚,旅游,星座命理,文艺,娱乐
旅游,健康,美食,娱乐
体育
旅遊,文艺
美食,旅游,文艺,娱乐
娱乐,游戏动漫
娱乐,时尚,健康

VIP,BigBang,学生一枚,旅游美食,旅游,健康,时尚,娱乐
旅游,游戏动漫,投资理财,体育,娱乐
投资理财,旅游,体育,IT 数码,汽车
旅游,IT 数码,教育就业,新闻资讯,健康,军事,文艺
搞笑幽默,时尚美容,投资理财
娱乐
文艺,美食,健康,体育
旅游,军事,体育,文艺,娱乐
IT 数码,美食,新闻资讯,健康,星座命理,旅游
新闻资讯,娱乐,旅游,健康,教育就业
游戏动漫
新闻资讯,星座命理,文艺
香港人在大陆,艺术青年,韩国音乐,单反摄影,SMTOWN
旅游,投资理财
搞笑幽默,游戏动漫,体育
，最爱少时，,汉哥,，无聊的人，,高中生,帅哥
高级按摩师,武术教练,气功大师,健康
穿衣美容,美食,旅游,星座命理,健康,美女,娱乐
新闻资讯,健康,美食
IT 数码,美食,健康
体育,IT 数码
美食,新闻资讯,时尚,文艺,健康
教育就业,旅游,星座命理,美食,IT 数码
时尚
汽车
投资理财
文艺
工作,足球,移动互联网,电脑,新闻资讯,美食,IT 数码,汽车,旅游,体育
旅游
旅游,新闻趣事,视频音乐,搞笑幽默,校园生活,旅行,交朋友,桌游,新闻资讯,体育
娱乐,粤语,搞笑,乐观,吃货,幽默
天枰座,懒人,大叔控,柯南,cosplay,游戏动漫,星座命理
爱准基,游戏动漫,旅游,娱乐,汽车,星座命理
星座命理,新闻资讯,娱乐,美食
电视,手工,娱乐,新闻资讯
星座运势,八卦杂谈,娱乐
视频音乐,热爱生活,旅游摄影
投资理财
娱乐
健康,IT 数码,游戏动漫,美女
健康,汽车,旅游,美食,体育
学生
娱乐
文艺,娱乐,健康,时尚,美食

旅游,健康,美食
娱乐,旅游
军事
时尚
教育就业
体育,旅游,教育就业
星座命理,娱乐
IT 数码,娱乐
美食,时尚,投资理财,新闻资讯,娱乐
文艺,投资理财,时尚,健康,娱乐
新闻资讯
教育就业,投资理财,旅游,星座命理,美食
网购,创意,电脑,努力,营销,低调,欧美,市场营销,广州人,美食
投资理财,汽车,美女,IT 数码,娱乐
新闻资讯,健康,星座命理,娱乐,旅游
美食,时尚,美女,星座命理,美食,娱乐
IT 数码,娱乐,投资理财
文艺,美女
美女
视频音乐,萌宠,新闻趣事,微博奇葩,深圳生活,丑妹妹,小矮人
时尚,投资理财,健康,教育就业,美女
文艺,时尚
美食,汽车,投资理财,旅游,美食,娱乐
搞笑幽默,穿衣美容,美食,星座命理
旅游,游戏动漫,娱乐
新闻资讯,旅游,时尚,文艺,娱乐
游戏动漫
星座命理,汽车,娱乐
北京生活,美食
新闻资讯,美食,IT 数码,娱乐
旅游,军事,星座命理,新闻资讯,投资理财
文艺,时尚,新闻资讯,教育就业,星座命理,美食
教育就业,星座命理,IT 数码
les,名人明星,美食
穿衣美容,旅游,名人明星,微博奇葩,新闻趣事,时尚,文艺,新闻资讯,娱乐
军事,游戏动漫
教育就业
娱乐,文艺,星座命理
游戏动漫,IT 数码
教育就业
体育,文艺,娱乐,健康
健康,文艺
旅游,健康,美食,文艺
军事,游戏动漫,美女,体育

美女,游戏动漫,娱乐,汽车
美食
美食,娱乐,旅游
视频音乐
淡水秋长,惠阳淡水,秋长,淡水,惠阳
时尚
时尚,健康,星座命理,IT 数码,娱乐
新闻资讯
设计,设计师,文艺
旅游,时尚,文艺,娱乐,美食
娱乐
游戏动漫
时尚,旅游,美食,IT 数码,娱乐
娱乐
旅游
IT 数码,汽车,军事
娱乐
文艺,健康,时尚,旅游,美食
娱乐,新闻资讯,文艺,旅游,星座命理
穿衣美容,视频音乐,新闻趣事,美食,旅游
时尚
新闻资讯,军事,旅游,体育,投资理财
旅游,NBA,体育
娱乐,新闻资讯
投资理财,健康,美食,时尚,娱乐
美食,IT 数码,时尚,文艺,娱乐
娱乐
文艺
旅游
健康,投资理财,旅游,文艺,体育
星座命理
IT 数码,文艺
汽车,军事
文艺
新闻资讯,健康,旅游,美食,汽车
教育就业,文艺
旅游,台湾生活,旅遊
新闻资讯,投资理财,旅游,IT 数码,体育
旅游,新闻趣事,美容服饰,育儿百科,美食
美女,时尚
时尚,新闻资讯
娱乐
娱乐,游戏动漫
吃,宅女,睡觉睡到自然醒,自恋,懒,外貌协会,天蝎,美术,腐女,游戏动漫

星座命理,美食,搞笑幽默,北京生活,起床困难户,摩羯座 O 型,技术宅,IT
旅游
美食
美女,旅游,新闻趣事,搞笑幽默,其他生活
娱乐
美食,体育
游戏动漫,美女,体育,娱乐
体育
旅游
古灵精怪,音乐迷
旅游,时尚,投资理财
健康,星座命理,旅游
美食
宅,SHE,90 後,PS
IT 数码,军事,美食,新闻资讯,健康
娱乐,文艺,新闻资讯,美食,星座命理
萌宠
星座命理,旅游,文艺,IT 数码,娱乐
美食,文藝,教育就業,健康,娛樂
美女,健康,军事
时尚,教育就业,文艺,娱乐,星座命理
体育,时尚,文艺,娱乐
娱乐,美食,星座命理,游戏动漫
美食
晋江原创网,动漫迷,大学党,同人文,手冢本命,名人明星
汽车,营销,双鱼座,80 后的尾巴,吃,社会
时尚,IT 数码,文艺,体育,娱乐
时尚,娱乐
五月天,双子,闷骚,单身,欧美音乐,我就是我,Music,唱 K,唱歌,娱乐
新闻资讯
军事
投资理财,听歌,移动互联网,化妆,文艺,时尚
美食,游戏动漫
游戏动漫
娱乐
营销,自信,健康,写作,传媒,媒体,亲情,数码,旅游,星座命理
健康,汽车
旅游
旅游,娱乐,健康
摄影,电脑,体育
新闻资讯
军事
娱乐,汽车,时尚,IT 数码,文艺
体育

双子,爱自由,看电影,90後,爱音乐,星座命理
体育
旅游,IT数码,军事,娱乐,投资理财,体育
军事,娱乐,文艺,新闻资讯,旅游
游戏动漫
投资理财,健康,旅游
文艺
美女,IT数码,汽车,体育,娱乐
搞笑幽默
日本,天秤座
摄影控,白羊,美食
文艺,体育,娱乐
游戏动漫
旅游,时尚,文艺
时尚
听歌,80后,唱歌,上网,美食,金贤重控,爱睡觉
IT数码,健康,美女,新闻资讯,时尚
微博奇葩,萌宠,广告,SEM优化,WOW,好静,阅读
珠海生活,时尚,娱乐
娱乐
美女,汽车,时尚,文艺,娱乐
ChrisBrown,JasonStatham,科蜜湖蜜,旅游,体育
时评,真诚,学习,书籍,文艺
文艺,教育就业
娱乐
IT数码,军事,美食,旅游,汽车,体育
娱乐
美女,时尚
娱乐,体育,旅游
旅游,新闻资讯,教育就业
美食,健康,娱乐
投资理财,美女,娱乐,体育
美女,投资理财
体育,旅游,美食,时尚,娱乐
电视剧,爱电影,90後,吃喝玩乐,爱自由,高中生,爱,开心,喜欢音乐
美图摄影,视频音乐,校园生活,搞笑幽默,美女
美食
旅游
文艺
汽车,美食,旅游,体育,娱乐
娱乐
旅游,娱乐
娱乐,美女,体育
健康,文艺,娱乐

旅游
旅游,健康,新闻资讯,时尚,娱乐
美食
娱乐,星座命理,时尚,美食,文艺
文艺,娱乐,时尚,军事,体育
健康,IT 数码,娱乐,星座命理,旅游
星座命理,旅游,健康,娱乐
穿衣美容,视频音乐,新闻趣事,搞笑幽默,遵义生活
新闻资讯,汽车,IT 数码,文艺,美食,旅游,健康,投资理财,教育就业
搞笑幽默,游戏动漫
旅游,健康,投资理财,新闻资讯,教育就业
汽车
微博奇葩,汽车
尹施允,张根硕,JustinBieber,赤脚的朋友们
美女,健康
信阳生活,美女
健康,娱乐,旅游,星座命理
IT 数码,教育就业,旅游,时尚,美食,娱乐
搞笑幽默,新闻趣事,美食,文艺
健康
旅游,肌肉,白袜子,健身,时尚,娱乐
旅游,新闻资讯,时尚,文艺,娱乐
新闻资讯,体育
IT 数码,汽车,投资理财,美食,文艺,娱乐
旅游,新闻资讯,IT 数码,投资理财,文艺,健康,时尚
美食
健康,旅游,美食,文艺,娱乐
娱乐
fx,彩妆,美容,时尚潮流,韩国,笑话,喜欢听歌,娱乐,美食,新闻资讯
投资理财,旅游,军事,IT 数码,体育
新闻资讯,美食
IT 数码,汽车,旅游,体育
体育,旅游
教育就业,星座命理,健康,新闻资讯,娱乐
体育
旅游
娱乐,美女,体育,军事
文艺,军事,汽车
投资理财,美食,旅游,IT 数码,体育
旅游
美女,新闻资讯
汽车,IT 数码
旅游
时尚,投资理财,美食,教育就业,文艺

萌宠,美女,music,漫画,苏打绿,美剧迷,游戏动漫,军事
体育,娱乐
文艺,旅游
时尚,娱乐
福建生活,美女,旅游,娱乐,文艺
娱乐
教育就业,美女,旅游,军事,文艺
时尚
新闻趣事,健康,军事,文艺,IT 数码,新闻资讯
执着,强迫症,懒,双子,宠物,90 後,爱幻想,迷糊,学生一枚,爱吃
美女,视频音乐,军事
文艺
美食,旅游,时尚,教育就业,文艺
美女,旅游,投资理财,体育,军事,健康
动漫,名人明星,星座命理,游戏动漫
娱乐
娱乐,旅游,IT 数码
军事,新闻资讯
舞蹈,爱自由,摄影控,爱生活,射手座,读书分享,娱乐,时尚,美食,旅游
美女,四川生活,IT 数码
文艺,新闻资讯,星座命理,军事,娱乐
美女,时尚,汽车,娱乐,美食
文艺,健康
娱乐,美女,美食,文艺,游戏动漫
时尚,旅游,美食,娱乐,文艺
汽车
娱乐,体育
时尚,健康,旅游,美食,投资理财,教育就业,星座命理,娱乐
旅游,星座命理,文艺,汽车
新闻资讯,投资理财,美食,旅游,文艺
美食,文艺,娱乐,体育,旅游
美食
星座命理,投资理财,教育就业,体育,娱乐
名人明星
文艺
投资理财,汽车,新闻资讯,美食,健康,旅游,军事
汽车,时尚,游戏动漫,教育就业
旅游,娱乐,时尚
体育
时尚
旅游,IT 数码,文艺,体育,娱乐
美容,养生,A 型血,设计师,化妆,时尚
爱睡觉,爱生活,爱自由,思考,减肥,奋斗,美剧迷
娱乐

旅游,美食
健康,旅游
游戏动漫,时尚,IT 数码
旅遊,体育,自信,单身,心理
文艺,娱乐
汽车,新闻资讯,文艺,IT 数码
IT 数码,体育
新闻资讯,健康,旅游,文艺,娱乐
情感生活,读书分享,新闻趣事,搞笑幽默,教育就业,时尚,美食,投资理财
娱乐,教育就业,时尚,旅游,文艺
汽车,旅游,美食,军事,娱乐
教育就业,健康
游戏动漫,娱乐
美食,旅游,健康,新闻资讯
冷笑话,DIY,设计师,欧美控,投资,美术,IT 数码,美食,美女,汽车
娱乐
教育就业
穿衣美容,时尚
洛阳传媒公司,洛阳广告公司,三维动画,广告公司,洛阳,军事,时事要闻,户外广告,旅游
娱乐,旅游,时尚,美食
教育就业,体育,IT 数码,美食,旅游,娱乐
娱乐,旅游
旅游,健康,时尚
美女,军事,IT 数码,体育,旅游
娱乐
视频音乐,教育就业,健康,文艺
健康,旅游,星座命理,美食,娱乐
时尚,娱乐
视频音乐,学生一枚,Music,文艺
健康
娱乐
其他生活
健康,IT 数码,娱乐,旅游
美食,旅游,娱乐
穿衣美容
美食,爱美食
文艺
dj
军事,投资理财
新闻资讯,美女,游戏动漫
新闻资讯,汽车,文艺,健康,娱乐,体育
投资理财,健康
新闻资讯
笔亲,潮牌控,BEGINS,畅畅亲麻,Music

名人明星
旅游,文艺,时尚,美食,娱乐
美食,旅游,文艺
星座命理,新闻资讯,旅游,美食,娱乐
搞笑幽默,cs
游戏动漫,娱乐
美食
文艺
时尚,IT 数码,文艺,健康,射手天蝎合体,旅游,美食
时尚,旅游,娱乐,体育
星座命理,娱乐
游戏动漫,美女,IT 数码,娱乐
新闻资讯,教育就业,IT 数码
IT 数码,娱乐,体育,游戏动漫,旅游
娱乐
时尚,美食,娱乐,健康,爱音乐,完美主义,喜欢猫猫狗狗,死五迷
体育,IT 数码,美食,旅游,星座命理,游戏动漫
文艺,娱乐
穷,摄影爱好者,技术宅,MAD,网络,游戏动漫
文艺,星座命理,健康,美食,娱乐
90 后
美女
射手,淘宝,星座控,懒
穿衣美容,微博奇葩,星座命理,搞笑幽默,健康,旅游,教育就业,体育,娱乐
健康
美食,旅游,文艺,健康,游戏动漫
娱乐
美食
娱乐,健康,星座命理,美食,旅游
健康,旅游,星座命理,文艺,美食
星座命理,美食
體育,娛樂,健康,美食,文藝
娱乐
文艺
广州生活,娱乐
游戏动漫,健康
星座命理,时尚,旅游,IT 数码,娱乐
健康,文艺,星座命理,体育,游戏动漫
美食,生活,育儿,教育,旅游
乐观,交友,自信,文化,睡觉睡到自然醒,胡思乱想,市场营销,男人,真诚,善良
旅游,星座命理,汽车,投资理财,娱乐
投资理财,星座命理,时尚,健康,美食
健康,文艺,娱乐,投资理财,时尚
时尚,汽车,体育,旅游,娱乐

美食, 美女, 淘宝控, 销售, 欧美控, 护肤, 彩妆, 健康
星座命理, 娱乐
IT 数码, 文艺, 健康, 时尚, 娱乐
游戏动漫
投资理财
新闻趣事, 微博奇葩, 旅游, 搞笑幽默, 健康, 星座命理, 文艺
IT 数码
文艺, 美食, 时尚
星座命理, 健康
旅游, 投资理财, 美女, 美食, 时尚
教育就业
健康, 投资理财, 旅游, 美食
星座命理, 美食, 听音乐, 睡觉, 学生, 小说
投资理财, 汽车, 军事, 文艺, 娱乐
美食, 娱乐
健康, 旅游, 新闻资讯, 投资理财, 体育
运气好点吧
名人明星, 健康, 爱生活, 怎能有传奇, 不经历风雨, 人在做天在看, 要有善心多积德, 生活是五彩的
娱乐, 游戏动漫
汽车, 体育
旅游美食爱好者, 宠物, 股票, 小资, 喜欢音乐, 开朗, 爱美食
美食, 微博奇葩, 星座命理, 运城生活, 游戏动漫, 体育
星座命理, 教育就业, 投资理财, 美女, 健康
美女, 娱乐
吃, 笑, 东阳哝, 爱睡觉, 水瓶座, 看书, 90 後, 旅游, 美食, 星座命理
游戏动漫, 娱乐, 旅游, 美食
搞笑幽默
文艺, 旅游, 时尚
时尚
汽车
投资理财, 时尚
新闻资讯, 时尚, 旅游
美食, 投资理财, 健康
旅游
游戏动漫, 美食
投资理财
体育
健康, 时尚
时尚
广西生活, 教育就业, 汽车, 新闻资讯, 健康
投资理财, 美食, 军事, 体育, 文艺
娱乐, 旅游, 教育就业, 体育
美食, 新闻资讯, 娱乐, 文艺, 教育就业, 旅游, 时尚, 健康

娱乐
娱乐
文艺,美食,旅游
娱乐,新闻资讯
游戏动漫,星座命理,旅游,IT 数码,娱乐
诗词,朋友,玩儿,吃货,乐观,自由职业,亲情,美食
美女,萌宠,穿衣美容,视频音乐
娱乐
文艺,IT 数码,游戏动漫,旅游
健康,美食
娱乐,投资理财,健康,旅游,文艺
健康,美食,旅游
美女
美女
美食,教育就业,文艺,健康,旅游,星座命理
美食,文艺,新闻资讯,时尚,游戏动漫,娱乐
娱乐,时尚,美食,旅游,健康
时尚
IT 数码,美女,旅游,时尚,汽车,娱乐
健康,时尚,旅游,星座命理
名人明星,星座命理,旅游,穿衣美容,美食,健康,文艺,娱乐,IT 数码
旅游,美容服饰,投资理财,健康,星座命理,娱乐
旅游,星座命理,健康,娱乐,美食
文艺,星座命理,娱乐
投资理财,教育就业,时尚,健康,旅游
旅游
考研,英语,MTI,大学生
时尚,旅游,星座命理,美食,文艺
文艺
时尚,健康,旅游,美食
美食,星座命理,穿衣美容,新闻趣事,搞笑幽默,新闻资讯,教育就业,娱乐,文艺,时尚
时尚
娱乐,旅游
娱乐
游戏动漫
摇滚,猫,视频音乐,美食,萌宠,旅游,穿衣美容,文艺
娱乐
星座命理,旅游,健康,文艺,体育
新闻资讯
汽车,IT 数码,星座命理,美食,旅游
娱乐
美女,旅游,游戏动漫,汽车,体育
娱乐,美食,旅游,军事,汽车
美食,时尚,汽车

美食
IT 数码,汽车
IT 数码,娱乐
旅游,美食,娱乐
旅游,美食,微博奇葩,新闻趣事,搞笑幽默
时尚
新闻资讯,汽车,文艺
投资理财
旅游,游戏动漫,时尚,娱乐,军事
娱乐
健康,教育就业,娱乐,旅游,星座命理
北京生活,文艺
健康,旅游,星座命理,IT 数码,文艺
文艺,美食,汽车,娱乐,健康,旅游,游戏动漫
美食,文艺,投资理财,时尚,健康
旅游,新闻资讯,投资理财,教育就业,军事,IT 数码,文艺
娱乐
投资理财,新闻资讯,健康,星座命理,娱乐
教育就业,投资理财,健康,旅游,美食
IT 数码,旅游,美食
体育,健康
星座运势,搞笑幽默,新闻趣事,美食,校园生活,娱乐
投资理财,新闻资讯,汽车,IT 数码,美女
军事,IT 数码,美食,旅游,汽车
名人明星,搞笑幽默,穿衣美容,健康,时尚,旅游,美食,娱乐
娱乐
滑板 skating,看电影,MJ,游戏动漫,体育
快乐,学生一族,小清新,书籍,旅游
新闻资讯,健康,军事,体育
游戏动漫
视频音乐,军事
健康,美食,教育就业,体育
军事,美食,旅游,娱乐
新闻资讯,健康,汽车,旅游,IT 数码
星座命理,新闻资讯,健康,娱乐,时尚,美食
旅游
美容服饰,时尚
IT 数码
广东生活,旅游,星座命理,搞笑幽默,新闻趣事,军事
美食,文艺
汽车
星座命理,新闻资讯,投资理财,IT 数码,文艺,娱乐
时尚,娱乐,新闻资讯,健康,旅游,星座命理
教育就业

娱乐,文艺,教育就业,时尚,星座命理
军事,体育,IT 数码,游戏动漫,娱乐
文艺,时尚
娱乐,八卦,宅,购物,游戏动漫,旅游,文艺
体育,旅游,星座命理,文艺,娱乐
美女
汽车,健康,美女,IT 数码,体育
汽车,军事,娱乐,体育,文艺
IT 数码
军事,文艺,汽车,星座命理,体育
教育就业,健康,投资理财,娱乐,新闻资讯,文艺
星座命理,时尚,旅游,美食,汽车,娱乐
美食控,时尚,文艺,旅游,美食,娱乐
娱乐,体育,电脑,旅游,IT 数码,游戏动漫,汽车,文艺
旅游
视频音乐,新闻趣事,广东生活,新闻资讯,健康,旅游,美女,时尚
浙江生活,健康,新闻资讯,军事,教育就业,投资理财
娱乐,汽车,健康,时尚,旅游,星座命理,美食
微博奇葩,旅行,听歌,90 后,感性,喜欢唱歌
吃喝拉撒睡,短发小青年,微博控
娱乐,健康
IT 数码,文艺,体育
游戏动漫
星座命理,美食,军事,游戏动漫,美女
搞笑幽默,汽车,旅游,文艺,体育,娱乐
IT 数码,新闻资讯
户外,足球,打篮球,汽车,投资理财,教育就业,旅游,80 后的尾巴
文艺,娱乐,星座命理
投资理财,教育就业,IT 数码
宅,80 后,科技,阅读,读书,语录,大学,美女,游戏动漫,IT 数码
时尚,旅游,娱乐
旅游,游戏动漫,美女,星座命理
星座命理,时尚,文艺,游戏动漫,汽车,旅游,IT 数码
星座命理,微博奇葩,旅游,美食,娱乐
骑行爱好者,户外运动与旅行,移动互联网创业,互联网创业,安卓 android
美女,娱乐
IT 数码,旅游,投资理财,娱乐,汽车
新闻资讯,健康,旅游,时尚,娱乐
游戏动漫,体育
体育
美食,旅游,娱乐,IT 数码,时尚
美食
汽车,文艺,美女,军事,IT 数码
旅游,体育

新闻资讯,教育就业,时尚
教育就业,投资理财,美食,游戏动漫,星座命理,军事
健康,游戏动漫,星座命理,美食,娱乐
娱乐,健康,新闻资讯,旅游,教育就业,投资理财,文艺
新闻资讯,美食,文艺,汽车,体育,美女,星座命理,娱乐
时尚
金融,爱音乐,看电影,狮子女,爱旅游,90後,灏明,教育就业,娱乐,美食
健康,旅游,美食
星座命理,教育就业,投资理财,健康,旅游,美食
美食
美食,文艺,娱乐
新闻资讯,时尚,汽车
宅男,旅游,健康,美女,体育,文艺
IT数码,娱乐
QQ,互联网,手机看小说,WOW,娱乐,IT数码
美女,投资理财,美食
游戏动漫,汽车,美食
游戏动漫,体育,IT数码,美女,汽车
旅游
旅游,美食,文艺
旅游,娱乐,文艺,汽车,投资理财,IT数码,体育
教育就业
星座命理,旅游,游戏动漫,汽车,娱乐
时尚,星座命理,美食,健康
游戏动漫,文艺,娱乐
学习ing,幽默,手机控,摩羯座,90后,孩子气
萌宠,星座命理,搞笑幽默,健康,旅游,文艺,时尚,美食
IT数码,美食
新闻资讯,文艺,健康,旅游
IT数码,旅游,体育资讯,美女,动漫
旅游
旅游,美食,游戏动漫
娱乐,旅游,文艺,美食
体育,星座命理,健康,军事
旅游,时尚
娱乐
野蛮女人
娱乐
时尚,旅游,美食,娱乐,文艺
闷骚,热爱生活,IT数码,汽车,体育
南海金马,桂城金马,佛山金马,金马旅行社,桂城旅行社,南海旅行社,金马国旅,金马
视频音乐,星座命理,广东生活,搞笑幽默,新闻趣事,音乐控
星座命理
体育,汽车,美女,时尚,投资理财,文艺,娱乐

健康,时尚,旅游,美食,IT 数码,投资理财,娱乐
教育就业,投资理财
时尚,美女,娱乐
汽车,新闻资讯
健康,时尚,星座命理,投资理财,娱乐
李钟硕,火影忍者,动漫
旅游,教育就业,新闻资讯,美食,娱乐,文艺
汽车
搞笑幽默
汽车,IT 数码,投资理财
娱乐
旅游,健康,时尚,娱乐,美食
星座命理,军事,游戏动漫,时尚
美女,星座命理,搞笑幽默
视频音乐,娱乐,新闻资讯,健康,旅游,教育就业,投资理财
投资理财,汽车,星座命理,美食,体育
娱乐
视频音乐,名人明星
体育
体育
娱乐
旅游,体育,娱乐
娱乐,IT 数码,军事,旅游,汽车
美食,文艺,健康,时尚,新闻资讯,汽车,娱乐,投资理财,星座命理,IT 数码
美食,军事,旅游,时尚,娱乐,文艺
名人明星,旅游
新闻资讯,健康,时尚,教育就业,美食
娱乐
美女,星座命理,IT 数码
时尚,娱乐,旅游,美食,健康
哲学,情感,管理,化妆,文艺,旅游,体育,时尚,健康
神经质,宅男,神瞎
视频音乐
淘宝
美女
游戏动漫,汽车,教育就业,军事,娱乐
旅游,健康,时尚
游戏动漫,汽车,旅游,IT 数码,文艺,美食
美女,娱乐
微博奇葩,名人明星,美食,星座命理,视频音乐,健康,文艺,娱乐
新闻资讯,汽车,娱乐,军事
旅游,新闻资讯,军事,体育
搞笑幽默,疯子,沉默
文艺

健康,旅游,美食,时尚,娱乐
星座命理,时尚,美食,IT 数码,文艺,娱乐
投资理财,时尚,美女,旅游,星座命理
IT 数码
旅游
娱乐,美食,旅游,游戏动漫,文艺
天然呆
娱乐,健康,汽车,美食
星座命理
健康,投资理财,汽车,娱乐,IT 数码
游戏动漫,美食
美食
搞笑幽默
汽车
搞笑幽默,美女
IT 数码,汽车,健康,旅游,娱乐
健康
健康
投资理财,美食,汽车
文艺,IT 数码,投资理财,娱乐,游戏动漫,美女
台湾生活,时装,打球,摩羯,美食,新闻趣事,穿衣美容,旅游,绵阳生活,体育
名人明星,娱乐
搞笑幽默,娱乐,军事
美食,时尚,文艺,体育,娱乐
美食,汽车,游戏动漫
新闻趣事,郑州生活,天蝎座,篮球爱好者,听音乐,旅游美食,绘画,爱睡觉,娱乐
时尚,新闻资讯,星座命理
健康,旅游,美食,时尚,娱乐
美食,时尚,娱乐
旅游,美食
新闻资讯,汽车,投资理财
投资理财,教育就业
娱乐
文艺,健康
时尚,娱乐
视频音乐
美食,星座命理
星座命理,美食,军事
美女,投资理财,旅游,汽车,文艺
时尚,星座命理,新闻资讯,美女,娱乐
旅游,陕西生活,搞笑幽默,星座命理,美女,美食,网络,羽毛球
微博奇葩,名人明星,游戏动漫
娱乐
时尚,文艺

LOL,巨蟹,健康
时尚,娱乐,星座命理
旅游
星座命理,娱乐
新闻资讯,投资理财,IT 数码
投资理财,美食
体育,汽车
投资理财,旅游,美食,IT 数码,美女,汽车
娱乐,水瓶女,吃货一枚,爱玩
新闻资讯,教育就业,投资理财,娱乐,体育
时尚
旅游
新闻资讯,健康,美食
教育就业,时尚,旅游,美食,娱乐
旅游
搞笑幽默,视频音乐,星座命理,微博奇葩,汽车,投资理财,美食,时尚,娱乐
美女,旅游,汽车,游戏动漫,体育
IT 数码
军事,游戏动漫,美女,汽车,旅游,IT 数码,美食,娱乐
美食,健康,时尚,星座命理,旅游
星座命理
健康
汽车
教育就业,时尚,星座命理,文艺,娱乐
时尚,美食,星座命理,旅游,娱乐
健康,美女,时尚,文艺,娱乐
娱乐
星座命理,娱乐,健康,旅游,美食
星座命理,新闻趣事,视频音乐,游戏动漫,IT 数码,娱乐,新闻资讯,文艺
IT 数码,美食
旅游,新闻资讯
旅游
旅游
娱乐
搞笑幽默,游戏动漫
汽车,游戏动漫,军事,体育
90 后非脑残,摩羯女,暮光之城
旅游,美食,IT 数码,体育,娱乐
时尚
体育,娱乐
美食
IT 数码,投资理财,健康,旅游,时尚
娱乐
文艺,美女,旅游,美食

绵阳装饰装修,绵阳装修,拍照,外贸,汽车,旅游
体育
健康,投资理财
穿衣美容,上网,睡觉,自由,投资理财,娱乐,文艺,教育就业
方齐禾,EVA,陈永通,EXO
美食,文艺,时尚,汽车,娱乐,IT 数码,星座命理
游戏动漫,美食,娱乐,时尚,旅游,星座命理
教育就业
健康
健康,新闻资讯,时尚,旅游,娱乐
自信,睡觉睡到自然醒,红酒,文化,原创,时尚,美女,新闻资讯,旅游,体育
星座命理,游戏动漫,娱乐,青春,爱情,阅读,新闻资讯,文艺
爱旅行,旅游
搞笑幽默,教育就业,大学生,养生,健康,化妆,美容,护肤,彩妆
旅游,美食
懒,喜欢音乐,淘宝控,爱睡觉,画画,潮流,90 后但我不脑残,时尚,娱乐,游戏动漫
穿衣美容,美食,旅游,美女
体育
娱乐,汽车,健康,旅游,美食
吃货,怀旧,音乐,友情
教育就业,汽车,健康,旅游,体育
旅游,教育就业,健康,文艺,体育,娱乐
娱乐
教育就业,时尚,军事,健康
视频音乐,美食,娱乐,新闻资讯,旅游,健康
IT 数码,星座命理
投资理财,娱乐,旅游
星座命理,娱乐
星座命理,文艺,汽车,时尚,娱乐,旅游,美食
体育,新闻资讯,文艺,IT 数码,军事
IT 数码,新闻资讯,旅游,美食,文艺,娱乐
娱乐,游戏动漫,军事,体育
双子女,旅游
健康,娱乐,星座命理
搞笑幽默,淘宝网店店主,主营日单,韩单男装
体育,美女,美食,娱乐
游戏动漫,旅游,文艺,娱乐,美食
广州生活,星座命理
健康,新闻资讯,娱乐
文艺,汽车,旅游,IT 数码,美食
汽车
星座命理,文艺
娱乐
个性,美容护肤,shopping,网购,双鱼座,iphone,情绪化,低调,爱电影,娱乐

娱乐
美食
教育就业,星座命理,旅游
教育就业,时尚,游戏动漫,健康,文艺
汽车
时尚,旅游,文艺,娱乐
旅游,美食,文艺
旅游
健康
娱乐,时尚,美食
视频音乐
军事,武术,现场报道,新闻资讯,喜欢音乐,80 后的尾巴
体育
美食
俞承豪,朴施厚,尹恩惠,我爱韩国,美食,IT 数码,时尚
科神老大黑曼巴,科比布莱恩特,娱乐,新闻资讯,IT 数码,体育
星座命理,健康,时尚,文艺,娱乐
视频音乐,新闻趣事,时尚,健康
健康
星座命理
广东生活
美食,星座命理,旅游,体育,娱乐
美食,IT 数码,文艺,健康,美女
小说迷,90 后但我不脑残,拍照,计算机,旅游摄影,美食控,交朋友,美食,旅游,IT 数码
90,设计师,高中生,时尚,娱乐
微博奇葩
美食,文艺,星座命理
新闻资讯,IT 数码,娱乐,汽车,体育
旅游,美食,星座命理
文艺,游戏动漫,汽车,健康
周杰伦,微笑,健康,旅游,娱乐
体育,美食,美女,汽车,投资理财
经济,文艺,开朗,创意,吃喝玩乐,真实力,完美主义,午饭,烹饪,美食
星座命理,时尚,游戏动漫,娱乐,旅游,IT 数码
旅游,娱乐,投资理财,新闻资讯
时尚,旅游
文艺,娱乐
健康,美食
微博奇葩,外貌协会,神经大条,耽美
健康
游戏动漫,军事,IT 数码
时尚
新闻资讯,娱乐,汽车,美食,军事
美食

娱乐,美食
旅游,美女,娱乐,美食,时尚
娱乐,IT 数码,健康,旅游,新闻资讯,教育就业,美食
美食,娱乐,投资理财,时尚,健康
视频音乐,名人明星,美图摄影,星座运势,校园生活,娱乐
美女,汽车,投资理财,健康,IT 数码
游戏动漫
星座命理,文艺,健康,美食,娱乐,教育就业
健康,时尚,投资理财,旅游,美食
娱乐,新闻资讯,美女
娱乐
美食,文艺,娱乐,时尚,汽车
美食
汽车,投资理财,旅游,体育,健康,刚正
搞笑幽默,娱乐
娱乐,健康
贵州生活
健康,游戏动漫,IT 数码,体育,娱乐
IT 数码,旅游,美食,娱乐,健康
美食,游戏动漫,IT 数码
文艺,健康,投资理财,旅游,美食
旅游摄影,喜欢户外活动,文艺
微博奇葩,萌宠,搞笑幽默,视频音乐,曲靖生活,时尚,投资理财
旅游
星座运势,文艺,星座命理,娱乐
健康,养生,新闻
旅游,娱乐,IT 数码,健康
游戏动漫,美食,文艺,娱乐
娱乐
旅游,体育,文艺,美女,IT 数码
汽车,投资理财,健康,旅游,美食
搞笑幽默,阳光帅气,乐观开朗,体育,新闻资讯,汽车,军事,文艺
娱乐
文艺
IT 数码,美女,汽车,时尚,游戏动漫
宅。,打酱油。
时尚,新闻资讯,旅游,美食,文艺
时尚,美食,IT 数码,投资理财,娱乐
游戏动漫
新闻资讯,旅游,军事
星座命理
新闻资讯,旅游,星座命理,美食,娱乐
美食,娱乐,新闻资讯
天秤,爱玩,旅遊,看电视,吸血鬼日记,电影发烧友

娱乐
健康,汽车,时尚,美女,娱乐
娱乐,投资理财,文艺
岳阳生活,文艺,青春,真诚
旅游
游戏动漫,投资理财,教育就业,美女,时尚,旅游,星座命理,娱乐
旅游,美食,投资理财,星座命理,健康
IT 数码
文艺,健康,美食
IT 数码
健康,体育,娱乐
美女,投资理财,教育就业,美食,娱乐
娱乐
美食,军事,旅游,健康,体育,新闻资讯,投资理财,汽车
娱乐
潮州生活,穿衣美容
娱乐,时尚,星座命理
时尚,健康,旅游,美食,娱乐
写作,亲情,执着,文学,70 后,体育,文艺,旅游,美食,健康
吃货,90 後,音樂,购物狂,爱美食,户外,动漫控,韩剧,水瓶女
美食,时尚,健康,游戏动漫
旅游,娱乐
日剧,健康,星座命理,旅游,美食
旅游,娱乐,美食,时尚,投资理财
健康,体育
投资理财,教育就业
健康,教育就业,旅游,美食
新闻资讯
美食,旅游
济南生活,政治,人生,工作,足球,阅读,创业,军事
天津生活,新闻资讯,旅游,时尚,文艺,娱乐
读书分享
新闻资讯,美食,健康,时尚,文艺
体育,娱乐
娱乐
喜欢听歌,时尚达人,书,家居,喜欢旅游,旅游美食爱好者,电影爱好者,重口味,IT 数码,军事
旅游
军事
名人明星,动漫,搞笑幽默,视频音乐,八卦杂谈,耽美狼,力宏控
游戏动漫,汽车,美女,星座命理,美食,娱乐
新闻资讯,美食,时尚,IT 数码,投资理财
IT 数码,体育
Google,游戏动漫,泡利不相容的三
投资理财,旅游

旅游
数码,电子商务,财经,校园,好书,营销,努力,地产,资讯,军事
时尚,文艺,娱乐
美女,教育就业,投资理财,健康
文艺,娱乐
IT 数码
美食,名人明星
美国生活,娱乐
搞笑幽默,名人明星,娱乐,好性格,吃喝玩乐,爱自由,唱 K
时尚,旅游,娱乐
星座命理
古玩鉴赏,字画,玉石翡翠,古瓷,鉴宝,收藏爱好者,古玩字画,收藏,文艺,投资理财
新闻资讯,星座命理,文艺
美图摄影,体育资讯,视频音乐,体育,文艺,美食,旅游,汽车
时尚,美食,星座命理,投资理财,文艺
文艺,健康,汽车,星座命理,娱乐
教育就业
投资理财,时尚,健康,旅游,文艺
新闻资讯,旅游,投资理财,娱乐,体育
体育,旅游,健康
历史,爱自由,听音乐,IT 互联网,射手座
微博奇葩,萌宠,文艺,教育就业,时尚,游戏动漫,魔方
旅游,娱乐,IT 数码,美女,美食
星座命理,时尚,美食,旅游,娱乐
军事
旅游,体育,军事
文艺,星座命理,旅游
时尚,健康,旅游,星座命理,美食
美食,瑜伽,韩语,音乐,文艺
美食,旅游,时尚,娱乐
健康,时尚,旅游,美食,文艺
旅游
娱乐
健康,星座命理,时尚,娱乐,IT 数码,新闻资讯
文艺,汽车,旅游,军事,体育
健康,新闻资讯,时尚,旅游,美食,娱乐
星座命理,美食,旅游,文艺,投资理财,健康
汽车,旅游,军事,体育,娱乐
健康,美女
旅游
时尚,健康,美食,IT 数码
汽车,旅游,军事,娱乐,体育
时尚,健康,新闻资讯,旅游,美食,文艺,娱乐
IT 数码

美食,星座命理
搞笑幽默
舟山生活,搞笑幽默,游戏动漫
宠物图集,李宇春
新闻资讯
吸血鬼,看书,星座命理
健康,新闻资讯,游戏动漫,体育,学生,毛笔字迷
IT 数码,美女,汽车
文艺,娱乐
美食,IT 数码,游戏动漫,体育,胡思乱想,校园,吃货,网络,手机,羽毛球
IT 数码,体育,教育就业,旅游,投资理财,娱乐
新闻趣事,体育
电影,海绵宝宝,娱乐,星座命理,旅游,美女,美食
微博奇葩,搞笑幽默,美食,娱乐,时尚,健康,旅游
投资理财,娱乐,美食,时尚
其他生活,搞笑幽默,无聊
旅游,视频音乐,新闻趣事,重庆生活,搞笑幽默,娱乐
文艺,时尚,教育就业,旅游,美食,星座命理,娱乐
娱乐,文艺,星座命理
汽车,游戏动漫
新闻资讯,健康,美食,文艺,时尚,投资理财
美食
音樂
美食,健康,军事,美食,文艺,体育,旅游
视频音乐
星座命理,IT 数码,娱乐
新闻资讯
旅游
娱乐
时尚
游戏动漫
美女
轻度强迫症,轻微洁癖,朴有天,吃货一枚,IT 数码,旅游
播音主持,播音员主持人,健康,文艺,旅游,星座命理,娱乐
娱乐,时尚
星座命理,健康,新闻资讯,美食,投资理财,娱乐
我是新手
健康,投资理财,时尚,文艺,娱乐
教育就业,旅游,健康,美食,娱乐
美女,体育
微博奇葩,搞笑幽默,穿衣美容,名人明星,萌宠,娱乐,游戏动漫
文艺,时尚,健康,星座命理,娱乐
星座命理,美食,娱乐,睡懒觉,吃货一枚,魔羯座,淘宝,时尚,健康,旅游
投资理财,汽车

美女,娱乐,体育,文艺,IT 数码
汽车,体育,星座命理,旅游,游戏动漫,军事
健康,文艺,教育就业,时尚,娱乐
娱乐,时尚,美女,美食
潮,bigbang,随性,星座命理
旅游,投资理财,美食,健康
娱乐
广告,重口味,夜猫子,外貌协会,流浪
新闻资讯,时尚,音乐,艺术,舞蹈,低碳环保,爱动物,爱生活
旅游,投资理财,汽车,健康
足球,体育
美食,美女,新闻资讯,教育就业,文艺
星座命理
微博奇葩,游戏动漫
体育,文艺
娱乐,游戏动漫,体育,美女,星座命理
新闻资讯,健康,旅游,汽车,美食,娱乐
旅游,美食,娱乐
健康,时尚,美食
新闻资讯
旅游,星座命理
娱乐,IT 数码
Digital
大理生活,新闻资讯
文艺,体育,旅游
美女,游戏动漫,娱乐
泉州生活,时尚
星座命理
娱乐,美食,健康,教育就业,新闻资讯,旅游,IT 数码
娱乐
军事,投资理财,文艺,体育,娱乐
娱乐
星座命理,娱乐
新闻资讯,娱乐,投资理财
游戏动漫,北京,玩,安静,房地产
星座命理,旅游,新闻资讯,体育,文艺
美女,游戏动漫,汽车,旅游,娱乐
投资理财,新闻资讯
星座命理,美食,时尚,旅游,IT 数码
时尚,健康,美食,文艺,娱乐
旅游
美女
美食
美食,娱乐

四川生活
旅游
时尚,旅游,美食,文艺,娱乐
文艺,美食
旅游,美食,星座命理,文艺
游戏动漫,娱乐,美食
成都生活,婚礼背景音乐,婚礼仪式音乐,婚礼司仪,婚礼主持人,旅游,时尚,美女,娱乐
宠物,白羊座,听音乐,吃喝玩乐,旅游,美食
手机,内向,简单
时尚,IT 数码,新闻资讯,游戏动漫,军事
时尚,汽车,教育就业,体育,文艺
文艺,娱乐,健康,星座命理
军事,娱乐,美女
O 型血,唱歌,宅,时尚,星座命理,文艺,体育
微博奇葩
美食,美女,时尚,IT 数码
IT 数码,军事
教育就业
爱生活,神经大条,志愿者,军事,娱乐,矛盾体,钢琴,减肥,猫咪控,旅游美食
健康,文艺,旅游,时尚,怀旧,猫咪控,玩,睡懒觉,购物,听音乐
娱乐,健康,旅游,星座命理,文艺
美食,新闻资讯,文艺,体育,旅游,娱乐
新闻趣事,娱乐
新闻趣事,穿衣美容,微博奇葩,福建生活
旅游
游戏动漫,体育,汽车,星座命理,军事
娱乐,星座命理
旅游
美食,健康,时尚
烹饪,自拍,无聊,巨蟹,星座控,时尚,健康,IT 数码,美食
体育
旅游
美食,IT 数码
名人明星,娱乐
美女,美食,军事,文艺,娱乐
汽车,娱乐,美食,星座命理,健康
时尚,投资理财,娱乐,美食,教育就业
体育,游戏动漫
文艺,健康,美食,星座命理
搞笑幽默
文艺,游戏动漫,时尚,健康,旅游,星座命理
爱硕
体育,汽车,娱乐
星座命理,設計師,雙重性格,愛自然

旅游
美女
萌孩控,追星族
新闻资讯,汽车,游戏动漫,美女,军事
体育,健康,汽车,IT 数码,娱乐
山东生活,游戏动漫,娱乐,军事,体育,汽车
视频音乐,娱乐,IT 数码
花草植物,韩寒,羽毛球爱好者,学生
微博奇葩,游戏动漫
健康,文艺
会计,瑜伽,新闻资讯,健康,投资理财
文艺,娱乐
创意设计
新闻资讯,体育,健康,投资理财,旅游,星座命理,文艺
新闻资讯
娱乐
时尚,健康,星座命理
汽车
投资理财,娱乐,时尚,星座命理,健康
美食,游戏动漫,美女,健康,体育
时尚,健康,星座命理,新闻资讯,娱乐
娱乐,Android,tvb,bl,SuperJunior,i,狮子女
军事
文艺,星座命理
娱乐
娱乐
星座命理
搞笑幽默
投资理财,健康,新闻资讯
看书,学生,自由,唱歌,娱乐,旅游,美食
旅游,娱乐
动画,美食
游戏动漫,体育,娱乐
新闻资讯
时尚,文艺,美食,健康,旅游
健康
美食,旅游
军事,教育就业,美食,文艺,娱乐,游戏动漫
看书,dota,运动,吃货,双子女,K 歌,吉他,瘦身,银魂,游戏动漫
美女
购物狂,猫控,韩剧,美食
娱乐
游戏动漫,时尚,文艺,军事,旅游
美女,投资理财

新闻趣事
旅游,美食,娱乐
美女,健康,文艺,教育就业,时尚,娱乐
美食
娱乐
B 型血,摩羯座,美食，头等大事,资深女屌丝,喜暴力,爱悬疑,深度欧美控,中等自虐狂,轻微强迫症
微博奇葩,健康
时尚,健康,旅游,美食,娱乐
美容服饰,新闻趣事,时尚
美女,游戏动漫,娱乐
美女
汽车,时尚,投资理财,文艺,娱乐
音乐达人,山西生活,IT 数码,健康,军事
娱乐,文艺,美食,健康,时尚
娱乐
娱乐,美食,军事,旅游,美女
香港,时尚,美女,娱乐,健康
美食,菜鸟
校园生活,健康,美女,星座命理,汽车,投资理财,军事
新闻资讯,健康,汽车,体育,娱乐
黄金价格走势图,炒黄金炒白银,黄金白银投资,工商银行纸黄金,工商银行纸白银,现货黄金投资,黄金白银 td 交易,黄金投资
美食,娱乐
星座命理
名人明星,娱乐
微博奇葩,IT 数码,体育,游戏动漫
旅游
星座,星座命理
美食
健康,娱乐,投资理财
旅游,健康,新闻资讯,投资理财,体育
新闻资讯,汽车,时尚,旅游,军事,体育
健康,旅游,美女,体育,娱乐
文艺,IT 数码,汽车,旅游
文字,母婴用品,母婴行业,辣妈,语录心情,互粉平台,8090 夹缝的一代,娱乐,交友,搞笑
江苏生活
健康,旅游,星座命理,新闻资讯,文艺
健康,教育就业,旅游,美食,文艺
汽车,时尚,健康,美食,投资理财
旅游,80 后的尾巴,法律,神经大条,紫色
时尚,健康,旅游,美食
舞蹈,体育
旅游,投资理财,汽车,IT 数码

汽车,美女,文艺,军事,娱乐
游戏动漫
美食
旅游,善良,艺术,体育,汽车,军事,娱乐,IT 数码
KY,腹黑,死宅腐,伪面瘫,中二病,动漫
FFF！,程序猿都是团员,贫乳控,武林外传,DOTA
体育,娱乐
投资理财,娱乐,美食,旅游,时尚
美食,旅游,文艺
时尚,娱乐,汽车,文艺,旅游,健康,投资理财
娱乐
时尚,健康,美食,旅游,娱乐
搞笑幽默,时尚
新闻资讯,美女,军事,体育,娱乐
爱情,化妆,shopping,韩国,理财,懒人,外貌协会,时尚,星座命理,健康
时尚
新闻趣事,搞笑幽默,河南生活
文艺,健康,投资理财,新闻资讯,旅游,体育
美女
游戏动漫
健康,旅游,美食,文艺,娱乐
美食
健康,新闻资讯,娱乐
娱乐
汽车,教育就业,投资理财,IT 数码,游戏动漫
娱乐,文艺,旅游,时尚
美食
娱乐,健康
体育
体育,娱乐
教育就业
美食,新闻资讯,投资理财,汽车,娱乐
娱乐,文艺
粤语,爱旅行,游泳,听音乐,爱音乐,新闻资讯,羽毛球,爬山,乒乓球,体育
健康,文艺,娱乐,美食,时尚
哈哈,电影爱好者,superjunior,欧美,IT 数码,美食,健康
健康,美女,投资理财,文艺,体育
90 后,互粉,喜欢听歌,爱自由,巨蟹女,时尚达人,热爱生活,时尚
娱乐
投资理财,旅游,军事,健康,娱乐,体育
IT 数码,娱乐,文艺
文艺,体育,娱乐
星座命理,旅游,美食,文艺,娱乐
美食

时尚,汽车,旅游
时尚,旅游,娱乐
时尚
娱乐,文艺,游戏动漫,时尚,星座命理
军事,新闻资讯,教育就业
美食,星座命理,旅游,健康,体育
新闻资讯,娱乐,时尚,教育就业,健康,文艺
投资理财,美女
军事,体育
投资理财
巨蟹,狗狗,音樂,猫咪,宅男,动漫控,学生一枚,神经质
时尚,健康,美食
汽车,军事,IT 数码,娱乐
星座命理
美女,文艺,娱乐,旅游,体育
星座命理
美食
美食,手机,爱美食,微博控
娱乐,美女,体育
旅游,出境游,辉煌国际旅行社,南通,签证
健康,娱乐
娱乐,健康,投资理财,星座命理,文艺
游戏动漫,娱乐
文艺,旅游,美食,体育,娱乐
投资理财,旅游,IT 数码,美食,娱乐
美女,新闻趣事,IT 数码
美女,时尚,新闻资讯,健康,旅游,军事,娱乐
娱乐
汕尾生活,爱幻想,宅,双重性格,单身,学生一枚,水瓶女
旅游,美食,星座命理,微博奇葩,视频音乐
游戏动漫
学习,电影爱好者,投资,股市,投资理财
投资理财,体育,IT 数码
旅游
旅游,名人明星,美女,云南生活,视频音乐
时尚,旅游,星座命理,强迫症,神经质,笑话,娱乐,美食
汽车
新闻趣事,北京生活,美食,搞笑幽默,微博奇葩,娱乐,汽车,体育,IT 数码,游戏动漫
美食,健康
游戏动漫,QQ 飞车,CG 逆战,CF 穿越火线
军事,新闻资讯,投资理财,汽车,旅游
时尚,汽车,美女,旅游,体育
娱乐,美食,80 后的尾巴,心理学,养生,巨蟹座
星座命理

旅游,美食
唱歌,宅,自由,动漫,时尚,上网,睡觉
搞笑幽默,体育,游戏动漫,美女
美女,旅游,时尚,IT 数码,文艺,体育
娱乐
美食,文艺
一个人的安乐
体育
美女
娱乐,汽车
微博奇葩,游戏动漫
旅游,体育,美食,军事,IT 数码
娱乐
投资理财,教育就业,健康,新闻资讯
时尚
文艺,新闻资讯,娱乐,美女,投资理财
娱乐
新闻资讯
美女
投资理财,IT 数码,美食,体育,娱乐
星座命理,美食,军事,游戏动漫,美女
新闻资讯,旅游,美食,军事,文艺
星座命理,IT 數碼,新聞資訊,健康,美女
美图摄影,搞笑,运动,双重性格,喜欢旅游
投资理财,汽车
娱乐,旅游,美食
宅,小说控,音乐爱好者,韩剧
娱乐,新闻资讯,时尚,健康,旅游
视频音乐,时尚,娱乐,新闻资讯
视频音乐,美女,荆州生活,进的了厨房,上得了厅堂,温柔,娱乐
娱乐,旅游
娱乐,时尚,健康,文艺
娱乐
美食,文艺,健康,娱乐
文艺,投资理财,旅游,娱乐
体育
娱乐,文艺
新闻资讯,投资理财,体育,教育就业,旅游,军事
教育就业,汽车,文艺
美女
军事
美食,投资理财,娱乐,健康
星座命理
游戏动漫

美女,名人明星,萌宠,微博奇葩,搞笑幽默,游戏动漫
摩羯,音乐控,90 后非脑残,青春,星座命理
美食
时尚,旅游,健康,文艺,娱乐
健康,星座命理,双重性格,喜欢音乐,水瓶座,善良,自由职业,幽默,娱乐,唱歌
YY,体育
军事,娱乐,旅游,文艺,美食,星座命理
美女
健康,文艺
教育就业
旅游
旅游,美食
美食,旅游
WWE,吉他,电影爱好者,美食,体育,娱乐
新闻资讯,教育就业,汽车
车品牌,社会化营销,品牌推广,品牌策划,品牌营销,汽车
投资理财
美女,美食,健康,投资理财,时尚
搞笑幽默,娱乐
旅游,美食,文艺,娱乐
游戏动漫
星座命理,娱乐,美食
美食
银川生活,新闻趣事,校园生活,娱乐,旅游,星座命理
星座命理,美食
星座命理,旅游,汽车,时尚,美食
时尚,教育就业,投资理财,美食,旅游
美女,娱乐,IT 数码,体育,旅游
芳疗,茶艺,yoga,园艺,旅游,美食
校园生活,美食,文艺,娱乐,时尚,星座命理
美女,投资理财
健康,投资理财,旅游,体育
文艺
自由,健身,体育
军事
娱乐,美女,体育
娱乐
名人明星,美食,浙江生活
IT 数码
美食
时尚,IT 数码,教育就业,文艺,新闻资讯,健康,旅游
投资理财,健康,旅游,美食,文艺
名人明星,我爱美伦子
投资理财,美食,旅游,健康,新闻资讯

健康, 娱乐, 文艺, 旅游, 新闻资讯
娱乐
娱乐
时尚, 美食, 文艺, 健康, 娱乐
游戏动漫
美食
美女
90, 爱旅游, 爱电影, 跳舞, 美食控, 活泼, 星座命理, 旅游, 美食, 娱乐
健康, IT 数码
美食, 文艺
娱乐
美食
体育资讯, 文艺, 军事
娱乐, 时尚, 体育
韩国生活, 萌宠, 名人明星, 穿衣美容, 美食, 时尚, 娱乐
娱乐, IT 数码, 汽车, 游戏动漫, 体育
美食
美食, 体育, 汽车, IT 数码, 旅游
旅遊, 美食, 娛樂
娱乐, 游戏动漫
学生一族, 小清新, 白羊座, 孩子气, 90 後
体育
穿衣美容, 娱乐
IT 数码, 健康, 投资理财, 教育就业, 旅游, 美食
娱乐, 游戏动漫, 旅游, 文艺
搞笑幽默, 娱乐, 旅游
游戏动漫
文艺, 健康, 汽车, 时尚, 星座命理
娱乐, 旅游, 美女
微博奇葩, 旅游, 搞笑幽默, 美食, 文艺, 投资理财, 星座命理
爱生活, 巨蟹女, 矛盾体, 猫咪控, 动漫控, 旅游, 体育
投资理财, 旅游, 军事, 文艺, 体育
体育
教育就业, 新闻资讯, 健康
英国, 时装, 快乐大本营, 旅游, 汽车
娱乐, 美食
名人明星, 搞笑幽默
汽车, 旅游, 美女, 投资理财, 体育
游戏动漫, 时尚, 娱乐
娱乐, 时尚, 美女, 体育
丽江生活, 娱乐
美女, 文艺, 健康, 旅游, 星座命理
IT 数码, 美女, 旅游, 体育, 汽车
旅游

美女,娱乐
娱乐,体育
星座命理,旅游
澳大利亚生活,文艺
投资理财
微博奇葩,娱乐
金牛座,星座命理,美食,文艺,娱乐
旅游,搞笑幽默
游戏动漫
IT 数码,教育就业
微博奇葩,萌宠,名人明星,旅游,美食
Marketing,SEO,策划推广,网店运营,电子商务,运动,公益,摄影,管理,互联网
文艺,星座命理,旅游,时尚,娱乐
美女
90 后但我不脑残,爱睡觉
星座命理,时尚
留学生,广播,黑莓,散步,品牌,陈翔,孩子气,玉米,笔亲,偶像剧
新闻资讯,美食,军事,IT 数码
旅游,健康,美食,体育,娱乐
娱乐
财经,好书,新闻资讯
时尚,美女,健康,旅游,星座命理,文艺
爱情,美女,旅游,时尚,星座命理,美食,文艺
美女,文艺,娱乐,时尚
游戏动漫
教育就业,健康,旅游
健康,新闻资讯,旅游,美食,IT 数码
美食,美女,健康,汽车,旅游
娱乐,旅游,文艺,美女,时尚
娱乐
新闻资讯,文艺,时尚,汽车
健康,投资理财,新闻资讯,旅游,军事,文艺
搞笑幽默,娱乐
娱乐
时尚,旅游
旅游,星座命理,健康,时尚
体育
古典文学,nba,体育
电影迷,看小说,奋斗,爱幻想,学习,笑,吸血鬼日记,自行车,汽车,军事
IT 数码
郑州生活,搞笑幽默
美女,IT 数码,军事,美食
旅游,教育就业,体育
名人明星,健康

新闻资讯,投资理财
游戏动漫,汽车,IT 数码
游戏动漫
汽车,旅游,美食,IT 数码,体育
搞笑幽默,旅游,健康,投资理财,教育就业,美女,娱乐
娱乐,旅游,美食
单身,玩儿,大学生,学生一枚,健康,美女,汽车,IT 数码,娱乐
投资理财
娱乐,IT 数码
文艺
新闻资讯,旅游,星座命理
体育,娱乐,美女,投资理财,新闻资讯
健康,文艺,娱乐,时尚,旅游,美食
粤语,重口味,美剧迷,切尔西球迷,TVB 迷,旅游
男护士
时尚,文艺
教育就业,时尚,新闻资讯,投资理财,旅游
健康,体育,文艺,娱乐,美食
美女,新闻趣事,搞笑幽默,视频音乐,文艺,旅游
旅游
微博奇葩,萌宠,旅游,健康,星座命理,美食,文艺,娱乐
搞笑幽默
娱乐
游戏动漫,军事,文艺,娱乐
新闻资讯,教育就业,娱乐,投资理财,美食,旅游,健康
美女,星座命理,健康,文艺,时尚
美女
萌宠,旅游,美食,穿衣美容,教育就业,新闻资讯
热爱生活,听音乐,爱音乐,广告,媒体,新闻资讯,美食,娱乐,体育,文艺
微博奇葩,IT 数码,美女
旅游,汽车
教育就业,时尚,健康,娱乐,美食
时尚,教育就业,投资理财,旅游,美食
汽车
时尚,新闻资讯,健康,星座命理,娱乐
汽车
新闻资讯
新闻资讯,汽车,投资理财,体育
娱乐,美女,时尚,汽车,军事
汽车,新闻资讯,旅游,时尚,军事,文艺
健康,文艺,娱乐
星座命理,旅游
美女,健康,旅游,星座命理,文艺
旅游,娱乐

新闻资讯, 娱乐
美女, 美食, 旅游, 河北生活, 时尚, 娱乐, 体育, 文艺
旅游, 汽车, 军事
旅游, 健康, 时尚, 美食, 娱乐
美食, 美女, IT 数码, 汽车, 旅游, 军事, 体育, 娱乐
好性格, 电影爱好者, 自由, 摄影, 学生, 健康, 旅游, 文艺, 美食, 娱乐
投资理财, 健康, 美食, 文艺
健康, 娱乐
娱乐
流浪
时尚, 文艺
美食, IT 数码
插画
旅游, 时尚, 娱乐, 美食, 文艺
美女, 游戏动漫, 娱乐
娱乐
健康, 文艺, 军事, IT 数码
微博奇葩, 搞笑幽默, 军事, 健康, 教育就业, 文艺, 体育
健康, 教育就业, 星座命理, 新闻资讯
爱旅游, 宠物, 摄影, 唱歌, 上网, 文艺
星座命理, 娱乐
新闻趣事, 旅游, 游戏动漫, 时尚, 汽车, 娱乐
美女, 投资理财, 美食, 军事, 文艺
娱乐, 飞轮海, 视频, 妖精, 音樂, 美食, 星座命理, 汽车, 游戏动漫
娱乐, IT 数码
文艺
福建生活, 时尚
旅游
娱乐, 游戏动漫
北京生活
穿衣美容, 健康, 新闻资讯, 美食, 教育就业
星座命理
体育
怀旧, 猫控, 美食, 健康, 旅游
娱乐
星座命理, 新闻资讯, 健康, 娱乐, 文艺
时尚
时尚, 汽车
投资理财, 旅游, 美女, 美食, 娱乐, IT 数码
金牛, 狗狗控, 宠物控, 学习, 爱玩, 旅游, 投资理财, IT 数码
游戏动漫
新闻资讯, 健康, 文艺
新闻资讯
IT 数码, 文艺, 旅游, 教育就业, 时尚

天蝎女,摄影爱好者,浪漫,时尚,娱乐,美食
美食,旅游,文艺
美食,旅游,健康,新闻资讯,军事
旅游,娱乐,体育,文艺,IT 数码
体育,教育就业,娱乐,星座命理,军事
娱乐,投资理财
星座命理
星座命理,健康,投资理财,美食
时尚
新闻资讯,娱乐,汽车,旅游
旅游,星座命理,娱乐,文艺
健康,美食,军事,旅游,娱乐
美图摄影,新闻趣事,校园生活,南京生活,视频音乐,等待幸福,天蝎座 90 后宅女,安静
旅游,美食,健康,娱乐
文艺,IT 数码,新闻资讯,旅游,体育
美食,游戏动漫,原创设计,原创漫画
美食,文艺,旅游,投资理财,游戏动漫
胡思乱想,人生,美食,娱乐,健康
美女
游戏动漫,旅游,美食,文艺,娱乐
时尚,汽车,美女,军事,娱乐
美食
新闻资讯,汽车,投资理财
玉米,吃货一枚,狗狗,绿色环保低碳
美食,旅游,教育就业,汽车,体育
美女,汽车,IT 数码,时尚,教育就业,美食,娱乐,投资理财
旅游,汽车,健康,军事,体育
美女,健康,星座命理,美食,娱乐
娱乐,教育就业,军事,文艺,体育
时尚,体育,新闻资讯,健康,投资理财
教育就业
IT 数码,文艺,体育,娱乐
新闻资讯,教育就业,娱乐
健康,教育就业,汽车,体育,娱乐
体育
娱乐
健康,投资理财,教育就业,星座命理,文艺
投资理财,时尚,美食,旅游,娱乐
旅游
文艺
娱乐,美食,投资理财,健康,旅游,新闻资讯
微博奇葩,新闻资讯
健康,娱乐,旅游,星座命理,美食
新闻资讯

学习,时尚,搞笑幽默,视频音乐,旅游,美食
体育
萌宠,美食,体育,吃货妥妥的,阿德勒,蝙蝠侠,生于武汉,波尔,展昭,灌篮高手
教育就业,投资理财,旅游,美食,体育,健康
旅游
星座命理
教育就业
时尚,健康,星座命理,美食,娱乐
河南生活
教育就业
新闻资讯,美食,体育,健康,旅游
美食,新闻资讯,文艺,娱乐,旅游
健康,新闻资讯,旅游,美食,体育
新闻资讯,旅游,美食
教育就业,时尚,健康,美食,旅游
投资理财,教育就业,健康
健康
美食,健康,投资理财,教育就业
美食,时尚,旅游,健康,文艺
投资理财,健康,旅游,时尚
美食,新闻资讯,教育就业,体育,军事
文艺,旅游,健康,教育就业,美食
文艺,新闻资讯,旅游,教育就业
文艺,健康,投资理财,教育就业
游戏动漫,美食,白酒内幕,白酒市场,营销策划,红酒,白酒销售,白酒,新闻资讯,投资理财
美女,体育
美女
新闻资讯,文艺,娱乐,星座命理,投资理财
时尚
只网购不出门,经常 10 天不出门,不织布爱好者,爱拼布爱手工,资深二手烟烟民,减肥上瘾,又腐又宅,网购狂人,80 后小主妇,减肥中的胖纸
文艺,娱乐
美女,交友,聊天,娱乐
IT 数码
美食,新闻资讯,投资理财,军事,IT 数码
健康,军事,新闻资讯,美女,娱乐
汽车
汽车
文艺,时尚,旅游,IT 数码,体育,娱乐
唱 K,时尚,旅游,美食,娱乐
文艺,游戏动漫,星座命理
健康,投资理财,时尚,旅游,美食,文艺
健康
文艺,娱乐

娯樂,星座命理
旅游
偶稀饭睡觉觉,巨蟹座,外貌协会,小女人,美食控,好性格,美剧,旅游,美食,时尚
旅游,真诚,温柔,教育,shopping,蓝色,新闻,心情,购物,健康
投资理财
美食,健康,教育就业,投资理财,文艺
旅遊
健康,美食,IT 数码,汽车,美女
浙江生活,视频音乐,搞笑幽默,汽车
星座命理,健康,时尚,美食,娱乐
军事,美女,投资理财,体育,美食,旅游
娱乐
娱乐
星座命理,健康,美女,娱乐,体育
汽车,投资理财,体育
健康
搞笑幽默,体育,娱乐
萌宠,还有好多好多,爱生活,爱运动
旅游,健康,IT 数码,文艺,娱乐
时尚
微博奇葩,搞笑幽默,娱乐,星座命理,游戏动漫
娱乐,旅游
玩,恐怖片,狮子座,吃,影视,动漫
游戏动漫,新闻资讯,文艺,时尚,星座命理,娱乐
美女,汽车,旅游,美食,IT 数码
体育,星座命理,旅游,游戏动漫,汽车
时尚
文艺,时尚,娱乐
IT 数码,星座命理,美女
旅游,健康,新闻资讯,美女,游戏动漫
投资理财
算是小清新吧,霸气十足,小清新,音乐爱好者,语言控,德国,登山,文艺
汽车,军事,IT 数码,体育
美食,娱乐
娱乐
健康,星座命理,唱 k
南京,娱乐,旅游,新闻资讯,美食
体育
美食
星座命理,搞笑幽默,大连生活,视频音乐,旅游,时尚
汽车,时尚,旅游,体育,娱乐
旅游,游戏动漫
时尚,星座命理
旅游,旅游爱好者,体育爱好者,音乐

萌宠,视频音乐,新闻趣事,游戏动漫,美食,旅游,健康
文艺
美食,健康,时尚,新闻资讯,娱乐
美食,军事
文艺
美食,新闻资讯,健康,文艺
健康,旅遊,旅游
时尚
旅游,汽车,星座命理,美食,军事,IT 数码
旅游,教育就业,美食
健康,文艺,旅游,美食
了解的是心,不了解的是自己,旅游,健康,游戏动漫,教育就业,娱乐
执着,矛盾体,柯南,双鱼,90 後,腹黑
旅游,健康,投资理财,时尚
读书分享,新闻趣事,八卦杂谈,搞笑幽默,合肥生活
美女
汽车
护肤,迷糊,矛盾,幻想,爱美食,神经质,心理学,爱电影,爱旅游,跳舞
美食
旅游,健康,时尚,美食
动漫,爱睡觉做梦,伤感文学,文艺
文艺,旅游,健康,新闻资讯,汽车
跳舞,潮,文艺,腐女,淘宝,感性,街拍,时尚潮流,旅游
汽车,健康,旅游,IT 数码,娱乐
文艺,健康,投资理财,星座命理,美食
旅游
欧美风,婚纱定制,高级定制,婚纱礼服,时尚
各種宅,无厘头,银魂,汽车,影视,高三,新闻趣事,视频音乐
旅游
AZ,17AU,劲舞团,自编,领舞,音乐,激舞,娱乐
美食,文艺,教育就业,健康,旅游
时尚
教育就业,投资理财,美女,游戏动漫,体育
安徽生活,文艺
随性,偶稀饭睡觉觉,蓝色,原创,好性格,设计,足球,旅游美食,IT 数码,音乐迷
娱乐,健康,新闻资讯,旅游,军事,投资理财
游戏动漫,美食,娱乐
游戏动漫
健康,美食,娱乐
美女,健康,旅游
星座命理,娱乐,游戏动漫,时尚
文艺
时尚,娱乐
投资理财,军事,教育就业,新闻资讯,娱乐,文艺

美女,旅游
星座命理,睡觉,吃货
电影迷,搞笑,巨蟹,蔡依林,韩国,暮光之城,听音乐,传媒,热情,娱乐
时尚,娱乐,文艺,旅游,健康
IT 数码,文艺,汽车,美食,旅游
IT 数码,校园生活,广州生活,时尚潮流,热爱生活,时装,EXO
名人明星,搞笑幽默,微博奇葩,穿衣美容,张根硕,爱帅哥爱美女,卑微,健康,星座命理,娱乐
搞笑幽默,美女,游戏动漫,时尚,汽车,娱乐
娱乐
旅游,时尚,游戏动漫,汽车,美食
美女
文艺,星座命理,美女,游戏动漫,娱乐,IT 数码
游戏动漫,军事,体育,汽车,美女
旅游,教育就业,健康,时尚,文艺
新闻资讯,旅游,军事,投资理财,汽车
爱人心理学
汽车,旅游,美食
搞笑幽默,旅游,投资理财,汽车
文艺,美女
星座命理
时尚,娱乐
娱乐
坚强,自然,杂志
军事,摄影师,执着,娱乐,旅游
IT 数码
投资理财,旅游,游戏动漫,娱乐
体育
旅游
星座命理,美食,时尚,娱乐,教育就业,健康,旅游
新闻资讯,投资理财,美食,健康,旅游
搞笑幽默,游戏动漫,IT 数码,娱乐
投资理财,新闻资讯,教育就业,美食
美食爱好者,欢迎爆料,感人事件,传奇故事,策划约稿,情感倾诉,文字控
广东生活
时尚,新闻资讯,健康,星座命理,娱乐
网络集成,网络华为,网络路由,网络工程,网络
娱乐,文艺,军事,体育
旅游
美女,健康,旅游,时尚,美食
美女,微博奇葩,名人明星,穿衣美容,视频音乐
新闻资讯,健康,投资理财,教育就业,旅游
游戏动漫
娱乐,时尚
吃喝玩乐,服装设计,微博控,巧克力,B 型血,紫色,休闲,开朗,游泳,星座

健康,美女,汽车,娱乐,军事
电影控,邋遢完美主义,猫,小说迷,旅游,游戏动漫
新闻资讯,星座命理
文艺,健康,教育就业,旅游,美食
美食
娱乐
单曲循环,乒乓,旅行,轻微洁癖,经济男
旅游,娱乐
IT 数码,时尚,娱乐,投资理财
美食,文艺
娱乐,星座命理,美食
搞笑幽默,游戏动漫
汽车,娱乐,体育,时尚
游戏动漫,美食,汽车,体育,娱乐
教育就业,体育,健康
郑州生活,旅游,美容服饰,新闻趣事,搞笑幽默
旅游,美食,健康
文艺,旅游,美食,娱乐
yoga 瑜伽,军事,新闻资讯,旅游,教育就业,健康
旅游,IT 数码,游戏动漫,文艺,娱乐
学生一族,看电影,O 型血,睡懒觉,写字,旅游
教育就业,时尚,健康,旅游,娱乐
文艺
教育就业
娱乐,体育
娱乐,健康,美食,IT 数码
旅游,健康,文艺
美女,投资理财,游戏动漫
投资理财,娱乐
情绪化,神经大条,怀旧,文字,幻想,睡觉,陈奕迅
军事
游戏动漫
林俊杰,感悟,心言
美女,新闻趣事,视频音乐,洛阳生活,教育就业,新闻资讯,军事,娱乐
懒,单纯,幽默,宅女,看书,小说,唱歌,吃货,搞笑,娱乐
娱乐
汽车,军事,美食
体育篮球,汽车,健康,娱乐,体育
名人明星,宠物图集,新闻趣事,美食,搞笑幽默,娱乐,新闻资讯
文艺,星座命理,时尚,娱乐
体育资讯,星座命理
视频音乐,旅游
娱乐
娱乐,旅游,美食,星座命理,文艺

游戏动漫,教育就业,投资理财,旅游,健康,新闻资讯,文艺
新闻资讯,健康,IT 数码,时尚,汽车
西安装修公司,装修公司,居泰隆公司,陕西居泰隆,西安居泰隆,实体家居,居泰隆装饰,居泰隆家居,北京居泰隆,居泰隆
美食,娱乐
旅游,美食,健康
漫画,看书
90,单身,互粉,喜欢听歌,活泼开朗,健康
娱乐
汽车,旅游,美食
美女
新闻趣事
游戏动漫,军事
娱乐
娱乐
教育就业,娱乐,旅游,美食,星座命理
娱乐,美食
投资理财,旅游,汽车,美食,体育
IT 数码,军事
新闻资讯,星座命理
汽车,游戏动漫,军事
投资理财
星座命理,双子座,睡觉,吃货,悲观主义者,乐天派,音乐,文字控
文字,文艺
娱乐
健康,星座命理,时尚,汽车,娱乐
文艺,娱乐,美食,时尚,游戏动漫,体育
娛樂,旅遊
游戏动漫
游戏动漫,教育就业,体育,IT 数码
文艺
时尚,汽车,健康,军事,旅游
IT 数码,游戏动漫,旅游,美食
娱乐
美食,健康,旅游
欧美控,Justin,娱乐
体育,健康
游戏动漫,时尚
旅游
新闻趣事,旅游,广东生活
娱乐,汽车,旅游,美女
90 後,音樂,爱唱歌,球迷,健康,体育
文艺,美女,时尚,旅游,美食,娱乐
美女,IT 数码,游戏动漫,体育

娱乐
B 型处女座,偏执狂,摄影,KPOP,懒人,淘宝控,PhotoShop,时尚
教育就业,游戏动漫,体育,娱乐
文艺,时尚,旅游
娱乐,健康,军事,时尚,美女
美女
美食,插画,腐女,淘宝,成都,大叔控,懒人,帅哥,广播剧
体育
星座命理,旅游,IT 数码,投资理财,娱乐
美女
文艺,健康,时尚,旅游,娱乐
新闻资讯,文艺,游戏动漫,娱乐
美食爱好者,旅游,美食,时尚,健康,娱乐
时尚,文艺,体育,娱乐,美女
游戏动漫,军事,体育,IT 数码
时尚
娱乐,星座命理
投资理财,旅游,健康,汽车,军事
游戏动漫,星座命理,美食
时尚,旅游,星座命理,美食,文艺,军事,娱乐
美女,游戏动漫,IT 数码,娱乐
娱乐
旅游,娱乐
體育,美食,娛樂,健康,旅游,时尚,娱乐,IT 数码
新闻资讯
星座命理,军事,娱乐
娱乐
星座命理,健康,新闻资讯,时尚,汽车
星座命理,美女
美食
IT 数码,美食,健康,时尚,娱乐
小清新
时尚,投资理财,文艺,娱乐,旅游
旅游,美女,健康,时尚,娱乐
体育
娱乐,旅游
旅游,健康,新闻资讯,时尚,文艺
新闻资讯,军事
军事
时尚
美女,汽车,娱乐,体育
体育
美女
军事,时尚,体育

体育
体育,IT 数码,汽车
爱音乐,桌球,傻,善良,艺术,文学,宅男,NBA,体育,娱乐
文艺,时尚,美食,IT 数码,旅游,娱乐
美女,旅游,体育,娱乐
新闻资讯
IT 数码
健康,投资理财,美食
IT 數碼,美女,遊戲動漫,娛樂
读书分享,新闻趣事,重口味,情感生活,旅游
娱乐,健康,美食,旅游
教育就业,体育
爱世勋爱生活
娱乐
IT 数码,健康,文艺
娱乐,汽车,IT 数码,美女,时尚,文艺
星座命理,美食,旅游,健康
搞笑幽默
英语,电子商务,投资理财,星座命理,军事,文艺,体育
文艺,体育,娱乐,汽车,投资理财
文艺,美食,新闻资讯,健康,时尚,娱乐
聊天
游戏动漫
军事,文艺,IT 数码,旅游,美女,娱乐
沉默,完美主义,游戏动漫,迷糊,随性,矛盾体,喜欢唱歌,无厘头,宅女加腐女,睡觉
美女,健康,星座命理,时尚,旅游
健康,时尚,游戏动漫,旅游,美食,娱乐
美食
微博奇葩,时尚,文艺
游戏动漫
新闻资讯,健康,教育就业,游戏动漫,娱乐
星座命理,新闻资讯
健康,旅游,星座命理,美食,娱乐
疯子,奋斗,读书,创业,旅游,时尚,投资理财,汽车
读书分享,星座运势,情感生活,新闻趣事,安阳生活,星座命理
健康,投资理财,美食,时尚,娱乐
新闻资讯
时尚,旅游,IT 数码
星座命理,新闻资讯,时尚,投资理财,美食,健康
娱乐
名人明星,体育
体育
小奋青,创业者,旅游
文艺,美食,旅游,军事,娱乐

军事
娱乐
星座命理,IT 数码,美女,时尚,娱乐
发呆,双重性
旅游,体育
旅游
时尚,汽车,娱乐
旅游
文艺,旅游,体育,娱乐
旅游,视频音乐,东莞生活
体育,娱乐
时尚,游戏动漫,美食,IT 数码,娱乐
IT 数码
游戏动漫,汽车,体育
文艺,汽车,投资理财,旅游,时尚,娱乐
时尚,娱乐,美食,健康
汽车,体育,健康,新闻资讯,投资理财,星座命理,旅游,军事
教育就业,投资理财,文艺,体育,娱乐
娱乐,教育就业
星座命理,旅游,穿衣美容,搞笑幽默,沈阳生活,娱乐
投资理财,健康,旅游,星座命理,美女,时尚,游戏动漫,娱乐
旅游,新闻资讯,美食,娱乐
上网,天平座,疯子,动画,服饰,90,music,K 歌,无聊,娱乐
IT 数码
娱乐,时尚
IT 数码,娱乐
游戏动漫，美食,星座命理,文艺,游戏动漫,时尚,旅游,美食,娱乐
娱乐,时尚
美食
旅游,娱乐
文字控,宅女,旅游美食,减肥,亲情,健康,旅游
旅游,新闻资讯,星座命理,美食,教育就业,投资理财
时尚,美食,旅游,娱乐,教育就业,新闻资讯,美女,健康
新闻趣事
星座命理,投资理财,旅游,文艺,娱乐
文艺,投资理财,美食,时尚,健康,旅游
新闻资讯,娱乐,汽车,IT 数码
吃货,旅游
微博奇葩,搞笑幽默,北京生活,旅游,汽车,体育
美食
汽车
教育就业,娱乐,汽车,新闻资讯,健康,星座命理
情绪化,射手,旅游,星座命理,美食
时尚

美女,微博奇葩,搞笑幽默,深圳生活,时尚,IT 数码,旅游
健康,美食
游戏动漫,体育
旅游,美女,新闻资讯,游戏动漫,体育
运动,音乐
旅游,文艺,美食,娱乐,健康
星座命理,北京生活,媒体人,白羊女,韩剧,娱乐,旅游,美食,时尚
时尚,文艺,汽车,娱乐,体育
美食,健康,旅游,投资理财,文艺,体育
新闻资讯,星座命理,美食,健康
美食
美食,健康,体育
教育就业,健康,旅游,星座命理,美食
时尚,美食,大学,蔡依林,爱音乐
娱乐
美食
汽车,娱乐
投资理财,文艺,星座命理,健康,旅游,美食
时尚,星座命理,娱乐
投资理财,教育就业,新闻资讯,健康,旅游
汽车
时尚,娱乐,IT 数码,美食,星座命理
娱乐
美食
健康,时尚,星座命理
美女,音乐控,爱旅行,执着,交朋友,自然,爱笑,摄影爱好者,帅哥,时尚
听音乐,看电影,媒体,美食
旅游,星座命理,美食,美女
娱乐
健康,娱乐,旅游
旅游
喜欢音乐,看 mv 第一眼灿烈,赵光敏,boyfriend,美食,星座命理
投资理财,时尚,美食,健康,娱乐
游戏动漫,美女,健康,星座命理,娱乐
视频音乐,体育资讯,校园生活,美图摄影,搞笑幽默,体育
有梦想的吃货
云南生活,健康,美食,教育就业,汽车,旅游
健康
文艺,旅游,星座命理,美食,娱乐
毕业在即,張根碩,90,校园,喜欢音乐
爱睡觉,看电影,游戏动漫
美女,时尚,IT 数码
IT 数码,体育,娱乐
娱乐,美食,旅游,军事,IT 数码

时尚,美食,旅游,星座命理,投资理财
时尚,旅游,星座命理,美食,娱乐
星座命理,时尚,娱乐,美食,旅游,文艺
美女
IT 数码,教育就业,新闻资讯,访客机,访客系统,身份阅读器,大头,强迫症,爱猫猫,德生
教育就业,文艺,娱乐
美食,投资理财,IT 数码,游戏动漫,体育
美食,穿衣美容
美食,投资理财,美女,汽车,娱乐
体育
投资理财,新闻资讯,健康
90 后,胡思乱想,狗狗,青春,高中生,孩子气,行星饭,阿狸,吃货一枚,处女座女生
军事,IT 数码
娱乐
旅游
星座命理,娱乐
投资理财,新闻资讯
美女
游戏动漫,旅游,文艺
搞笑幽默,美女
健康
游戏动漫
名人明星,文艺
文艺,怀旧,狗狗,坚强,敏感
时尚,旅游,美食,文艺,娱乐
视频音乐,微博奇葩,旅遊,娛樂,IT 數碼
体育,军事
游戏动漫,娱乐,体育,IT 数码
名人明星
体育
健康,美食
教育就业,旅游,投资理财,时尚,汽车
文艺,健康,美女,IT 数码,娱乐
旅游
健康
新闻资讯,流浪,梦想,打球,时尚,娱乐,文艺,体育
江苏生活,文艺,新闻资讯,IT 数码
视频音乐,微博奇葩,名人明星,新闻趣事,搞笑幽默,时尚
文艺,娱乐
教育就业,时尚,健康,旅游,美食
英格兰,游戏动漫,IT 数码,娱乐,体育
军事,汽车,美女,文艺,体育
体育
宠物图集,动漫,游戏动漫

军事,新闻资讯,娱乐
投资理财,美食,娱乐
时尚,美食,健康,旅游
汽车,IT 数码,游戏动漫
教育就业,星座命理,娱乐,IT 数码,新闻资讯,投资理财,军事
新闻资讯,旅游,美食,娱乐,IT 数码
健康,旅游,新闻资讯,美食,教育就业
很傻很天真,古风控,非常穿越,宅女一枚,美术,穿越小说控,设计是一种态度
娱乐
搞笑幽默
美食,娱乐
文艺
健康,星座命理,免费体验治疗,肤美先,皮肤病,牛皮癣,皮炎,湿疹
娱乐
时尚,新闻资讯,投资理财,旅游,健康,军事
汽车,文艺,娱乐,体育,旅游
郑州生活,旅游,投资理财
汽车,美食,旅游,健康,投资理财
游戏动漫,体育
游戏动漫,娱乐,IT 数码,汽车
萌宠,视频音乐,天津生活,游戏动漫,IT 数码
时尚,娱乐,文艺,健康,旅游,学生一枚
爱看星星的猫!!
旅游,投资理财,体育,军事
美食,视频音乐,旅游
文艺
旅游,星座命理,新闻趣事,美食,洛阳生活
穿衣美容,星座命理,微博奇葩,视频音乐,萌宠,健康,游戏动漫,文艺,娱乐
投资理财,健康,旅游,美食,娱乐
网络,手机,双子座,美食控,新闻资讯,美女,时尚,旅游,娱乐,美食
美食,星座命理
娱乐
穿衣美容,星座命理,时尚,娱乐
娱乐,汽车,美食,文艺
美食,旅游,时尚
文艺
健康,新闻资讯,旅游,美食,文艺
美食,文艺,娱乐
星座命理,新闻资讯
军事,新闻资讯,健康,文艺,投资理财
娱乐,教育就业,时尚,旅游,军事
旅游,游戏动漫
星座运势,南京生活,美容服饰,时尚,健康,旅游,星座命理,IT 数码
文艺,健康,教育就业,时尚,娱乐

微博奇葩,星座命理,时尚,旅游,健康
娱乐,时尚,美食
游戏动漫,IT 数码,军事
旅游,健康
体育
投资理财
文艺
校园生活,汽车
健康
新闻资讯,娱乐,投资理财,汽车
体育,投资理财,文艺,新闻资讯,IT 数码
穿衣美容,文艺,娱乐,健康
体育
时尚,游戏动漫,文艺,体育
美容护肤,旅游,养生,宅,看小说,逛街,时尚
健康,好性格,90,跑步,睡懒觉,美食
磨人,懒人,强迫症,感性,爱旅行,多愁善感,Music,唱 K,高三,旅游
乐天派,爱睡觉,娱乐,旅游,美食
娱乐
新闻资讯,娱乐
时尚
购物狂,电子商务,养生,投资理财,旅游
美食,旅游,星座命理,娱乐
旅游爱好者,双鱼女,大学,投资理财,教育就业,时尚,新闻资讯,美食
教育就业,新闻资讯,投资理财,旅游,健康
时尚
IT 数码
新闻资讯,体育,文艺,汽车
健康
文艺,娱乐,新闻资讯
旅游
美食,军事,文艺,体育
杭州生活,时尚,星座命理,健康
休闲,旅游
新闻资讯,旅游,时尚,汽车,军事,美女,美食
娱乐,星座命理
体育,美女,投资理财,IT 数码
IT 数码
旅游,美食,娱乐
星座命理,教育就业,文艺,美女,健康
星座命理,娱乐
美食
美女,体育
娱乐,游戏动漫,美女,旅游,体育

健康,旅游,美食,时尚,娱乐
星座命理
旅游,娱乐,健康,教育就业,美食
居家,励志,语录,写作,文艺,杭州,流浪,书迷
时尚
新闻趣事,健康,文艺,时尚,新闻资讯
娱乐
动漫迷,爱幻想,看小说,购物狂,文艺,娱乐
旅行爱好者,写作,美食
时尚,新闻资讯,汽车,美女,军事
军事
旅游,健康,文艺,星座命理,时尚
时尚,汽车,文艺,星座命理,旅游,娱乐
美女
萌宠
娱乐
时尚,健康,美食,娱乐,旅游
娱乐
健康,旅游,文艺
体育,IT 数码,投资理财,汽车
汽车,娱乐,体育,游戏动漫
旅游,游戏动漫,文艺
体育,游戏动漫
娱乐,健康,投资理财,旅游
外貌协会,网游,神经大条,自恋,看电视,喜欢旅游,双重性格,爱,星座命理,娱乐
星座命理,美女,新闻趣事,搞笑幽默,厦门生活,文艺,体育,教育就业,投资理财,娱乐
新闻资讯,教育就业
游戏动漫
新闻趣事,搞笑幽默,微博奇葩,视频音乐,美女,教育就业,体育,投资理财
睡觉睡到自然醒,读书人
娱乐
视频音乐,星座运势,读书分享,美食
体育
宅
交友,新闻资讯,投资理财,旅游,汽车
投资理财,教育就业
美食
投资理财,旅游,军事,文艺,娱乐
文艺,体育,娱乐
美食,体育
旅游,汽车,投资理财,新闻资讯
旅游,IT 数码,时尚,汽车,娱乐
淡定,文藝,奋斗,阳光,安静
旅游

星座命理,娱乐
星座命理
名人明星,新闻趣事,芜湖生活,旅游,美女,文艺
游戏动漫,时尚,星座命理,文艺,娱乐
旅游,新闻资讯,军事,教育就业,汽车,IT 数码
微博奇葩,萌宠,穿衣美容,新闻趣事,交友,健康,旅游,汽车,时尚
时尚,娱乐
微博奇葩,吃货,任性,爱睡觉,宅,电影迷,90 后但我不脑残,双鱼女
游戏动漫
美女,旅游,体育
娱乐,体育,文艺,美食,美女,旅游,游戏动漫,军事
游戏动漫
微博奇葩,IT 数码,娱乐,体育
女装网店,潮流,搭配,时尚,健康,美女
美食,健康
美食,旅游,体育
娱乐,体育
文艺,娱乐,星座命理,校园,90 后,内向,执着,粉红控
美食
旅游,健康
娱乐
美食,健康
汽车,时尚,旅游,军事,体育
文艺
娱乐,教育就业,健康,新闻资讯,投资理财
新闻趣事,其他生活
文艺,汽车,投资理财,时尚,健康,旅游
时尚
健康,体育
美食,旅游,健康,娱乐
旅游,文艺,娱乐
星座命理,时尚,娱乐
游戏动漫
美食,时尚,喜欢听歌,服饰
简单
新闻资讯,娱乐,美食
星星,热爱生活,创意,旅行,美景,感性,巨蟹座
体育,篮球
美食,新闻资讯
宅,懒人,巨蟹女,新闻资讯,文艺,旅游,星座命理,美食
美食
旅游,游戏动漫,美食,IT 数码
健康,时尚,星座命理,美食,文艺
时尚,旅游

IT 数码
微博奇葩, 教育就业
娱乐
旅游, IT 数码, 汽车
健康
投资理财, 健康, 美食, 旅游, 体育
教育就业, 投资理财, 新闻资讯, 健康, 旅游
校园生活, 视频音乐, 美图摄影, 体育资讯, 旅游, 娱乐, 汽车, 文艺, 体育
美女, 游戏动漫, 星座命理
星座命理, 健康, 时尚, 教育就业, 投资理财
IT 数码, 视频音乐, 投资理财
美食
游戏动漫, 时尚, 娱乐, 文艺
娱乐, 时尚, 旅游, 美食
新闻资讯, 投资理财, 文艺, 旅游, 军事, 娱乐
投资理财
旅游, 美食
娱乐
时尚, 健康, 美食, 投资理财, 新闻资讯, 文艺
旅游, 美食, 文艺
健康, 美食, 游戏动漫, 娱乐, 星座命理
美食, 时尚, 星座命理, 游戏动漫, 娱乐, 旅游
动漫控, 写作, 阳光，开朗, 听歌　看小说
IT 数码, 游戏动漫, 新闻资讯, 汽车, 美食, 时尚, 娱乐, 投资理财
旅游, 投资理财, 军事, 文艺, 体育
学生一枚, 好书, 视频音乐, 文艺
收藏, 乐天派, 法国, 爱自由, 哲学, 公益, 思考, 梦想, 时尚
健康, IT 数码, 娱乐
名人明星, 美女, 穿衣美容, 新闻趣事, 其他生活
美女, 时尚
运动, 看书, 唱歌, 上网, 品牌, 美食, 游戏动漫
时尚, 文艺
体育
美食
旅游, 星座命理, 美食
囧, 郭敬明, 射手, 文艺男青年, 星座命理, 美食, 娱乐, 旅游, 文艺
文艺
健康
军事
军事, 文艺, 娱乐, 教育就业, 健康, 美食, 旅游
汽车
名人明星, 娱乐
教育就业, 汽车, 娱乐, 军事
健康, 旅游, 美食

美女,吃货一枚
汽车
汽车,娱乐,星座命理,军事
时尚,健康,旅游
娱乐,时尚,旅游,美食,IT 数码
文艺,体育,娱乐
胡思乱想,瑜伽,猫控,热爱生活,唱歌,动漫,听音乐,IT 数码
新闻资讯,美女,游戏动漫
体育
新闻资讯
娱乐,文艺,美食,时尚,旅游
文艺,健康,旅游,时尚,娱乐
美图摄影,星座运势,视频音乐,校园生活,娱乐,旅游,文艺
美女,文艺
娱乐,天蝎女,文艺
美食
旅游爱好者,经济,完美主义,打篮球
汽车
文艺,体育,娱乐
娱乐
情感生活,读书分享,校园生活
西安,奋斗,独立,o 型血,学生,书籍,人生,军事
旅游
游戏动漫
文艺,旅游,健康,教育就业,新闻资讯
娱乐,新闻资讯,教育就业,汽车,体育
健康,新闻资讯,星座命理,娱乐,投资理财
投资理财
游戏动漫,文艺,娱乐,时尚
娱乐,IT 数码,美女,游戏动漫,体育
旅游
北京生活
时尚,游戏动漫
健康,旅游,时尚,娱乐,文艺
旅游,新闻资讯,IT 数码,健康,星座命理
美食
汽车,新闻资讯,军事
it 互联网,软件,IT,朋友,懒人,喜欢听歌,计算机,IT 互联网,喜欢音乐,IT 数码
美食
美女,娱乐,文艺
健康
90 后,腐女一枚,仙后,夜猫子,外貌协会,睡觉睡到自然醒,强迫症,爬山,水瓶女,美食
旅游,美食,娱乐,时尚
美食,娱乐,游戏动漫

时尚,汽车,投资理财,娱乐
美食
周杰伦,游戏动漫
娱乐
游戏动漫
娱乐,IT 数码,美食,旅游,游戏动漫,体育
体育
体育资讯,夜猫子,善良,电脑,三国杀,我就是我,体育
旅游
小说,旅游,美食,美容,时尚
娱乐
美女
投资理财
笑,发呆,游戏动漫,猫控,迷糊,大叔控,热爱生活,懒人,爱美食,军事
教育就业,文艺
旅游,美食,IT 数码,体育,娱乐
搞笑幽默,旅游,美食,萌宠,微博奇葩,体育
music,爱旅行,多愁善感,星座控,90,单纯,健康,娱乐,文艺,时尚
时尚,投资理财,健康,旅游,美食
时尚
文艺,新闻资讯,娱乐,90 后但我不脑残,美食,游戏动漫,投资理财,旅游,时尚
娱乐
时尚,汽车,教育就业,投资理财,娱乐
新闻趣事,新闻资讯
80 后,低调,旅游美食爱好者,管理,投资,市场营销
娱乐,游戏动漫,旅游,体育,汽车
星座命理
旅游,体育,文艺,星座命理,美食
娱乐,旅游,星座命理,健康,美食,时尚
河南微信营销,河南微信公众平,河南微营销,微营销
美食
爱幻想,快乐,睡懒觉,爱吃,旅游
娱乐,IT 数码,美食,军事,游戏动漫,体育
健康,娱乐
懒人,健康
旅游,美食
穿衣美容,名人明星,旅游,视频音乐,时尚,娱乐
健康,爱美,化妆品代购,时尚
美女,健康,教育就业,时尚,军事,美食,娱乐
美图摄影,动漫
微博奇葩,时尚,文艺
交朋友,努力,活泼开朗,新闻,网络,浪漫,体育,投资理财,军事,美女
时尚,娱乐,美食
教育就业,游戏动漫,旅游,星座命理,美食,文艺,健康

军事,汽车,时尚,体育
搞笑幽默,自然,学生一族,吃,搞笑,乐观,健康
健康,旅游,IT 数码,时尚,投资理财
美图摄影,IT 数码,美女,校园生活,北京生活
投资理财,时尚,旅游,娱乐
汽车,教育就业,文艺
体育
健康,新闻资讯,美食,军事,体育
名人明星,旅游,视频音乐,游戏动漫,时尚,美食,星座命理,娱乐
健康,体育,教育就业,文艺,新闻资讯,星座命理,游戏动漫,娱乐
时尚,美食,星座命理
风行菱智,风行景逸,柳汽,汽车销售,汽车服务,汽车,东风风行,4S 店,淮南润景,风行汽车
美食,星座命理,IT 数码,新闻资讯,健康
加拿大生活,文艺
健康,新闻资讯,游戏动漫,美食,娱乐
汽车
体育
娱乐
海淘代购,爱美食,宅,80 后妈妈,金牛
吉林生活,宅女,娱乐
文艺,半只女屌,布拉德皮特,单身待解救,偽文藝女青年,美食,娱乐
学生一枚
汽车,时尚,游戏动漫,IT 数码
IT 数码,星座命理,旅游,美食,军事
纸艺,艺术,设计,剪纸,文艺
健康,汽车,时尚,旅游,IT 数码
投资理财,军事,新闻资讯,星座命理,文艺
星座命理,文艺
IT 数码,体育资讯,美女,汽车
旅游,娱乐,汽车,时尚,明基模力女孩,美食
健康,新闻资讯,汽车,投资理财,旅游
新闻趣事,健康,新闻资讯,星座命理,美食,文艺
吃货,射手女,90 後,爱旅游,神经质
健康,美女,体育
故事,文学,微博营销,IT 数码,时尚,网上购物,推广,生活,健康
投资理财,旅游
军事,IT 数码,时尚,旅游,汽车
美食,军事,IT 数码
文艺,旅游,新闻资讯,汽车,军事
TNC,BNC,SMA,连接器
体育,游戏动漫
微博奇葩,动漫控,手机控,微博控
视频音乐,娱乐,汽车
旅游,美女,职场招聘,自贡生活,投资理财,新闻资讯

健康,旅游,美食,文艺,娱乐
搞笑幽默,娱乐
美食,文艺,体育
时尚
时尚,文艺,星座命理
健康,投资理财,军事,文艺,旅游
娛樂,美女,星座命理
娱乐,文艺,游戏动漫
游戏动漫
娱乐,军事,教育就业,新闻资讯,体育
小可爱,吃货,爱生活,爱舞蹈,小宝王一鸣
新闻趣事,视频音乐,江苏生活,健康,时尚,美女,旅游,娱乐
听歌,90 后,萌宠,美食,星座命理,时尚
美女,大家好,广告人,旅游
文艺,时尚,美食,旅游,星座命理,娱乐
娱乐,旅游
湖南生活
汽车,新闻资讯,美女,体育,游戏动漫
文艺
文艺
旅游,新闻资讯,游戏动漫,文艺,体育,娱乐
旅游,火影,85 后,运动,英语,看小说,吃,影视
美食,旅游
游戏动漫,汽车,娱乐,IT 数码
娱乐,美食
星座运势,动漫,体育资讯,视频音乐,搞笑幽默
自由,创意设计,乐观,旅游美食爱好者,福建生活,时装,热情,时尚,文艺,娱乐
健康,美食,时尚
新闻资讯,健康,汽车,旅游,美食,娱乐
旅游,娱乐,汽车,时尚,美食
体育,美女,娱乐,汽车,IT 数码
美女
搞笑幽默,体育
时尚
文艺,iphone,外貌协会,热情,计算机,投资理财,军事,IT 数码,娱乐,旅游
汽车,新闻资讯,游戏动漫,美食,军事
娱乐,时尚,健康,新闻资讯,美食
文艺,美食,美女
旅游
文艺,体育,教育就业
美食,健康,旅游
美国生活,肉村,室长 prpr,佐狘宜
美食
美食

温州生活
情感,个性,美食
新闻趣事,美容服饰,情感生活,视频音乐,东莞生活,教育就业,星座命理,军事,文艺,娱乐
体育,美食,娱乐
游戏动漫
健康,旅游,美食,IT 数码,体育
娱乐,新闻资讯,旅游,美食,美女
搞笑幽默,旅游,名人明星,美女,军事,汽车,时尚,体育,IT 数码
旅游,星座命理,新闻资讯,教育就业,投资理财
文艺,时尚,旅游,美食
新闻资讯,文艺,旅游,健康,美食
美食,健康,教育就业
时尚,投资理财,教育就业,游戏动漫,娱乐
健康,旅游,星座命理,美食,娱乐
健康
美容服饰,时尚
摩羯
汽车,IT 数码
时尚,星座命理,旅游,娱乐
动画,美食
旅游,文艺,IT 数码,健康,教育就业
时尚,汽车,文艺
名人明星
教育就业,新闻资讯,投资理财,时尚,旅游,文艺
投资理财,IT 数码
爱音乐,开朗,投资理财,新闻资讯,军事,旅游
新闻资讯
美型大叔控,心理學,鑽石控,吃貨逛吃逛吃,小騷粉 or 紫粉,各種萌各種 2,愛哭愛笑愛生活,巨蟹阿喂,感情潔癖,軟妹紙一枚
投资理财,健康,旅游,美食,娱乐
游戏动漫,旅游,文艺
视频音乐,娱乐,动漫,学生,幸福
娱乐,投资理财,IT 数码,文艺,旅游,汽车
新闻资讯,美食,时尚,美女,体育
新闻资讯,汽车,游戏动漫,军事,IT 数码
美女,IT 数码
旅游,游戏动漫,美女,文艺,娱乐
美食,文艺,健康,星座命理,旅游
文艺,时尚,美女
军事
时尚
文艺,时尚
文艺,投资理财,旅游,健康,美食
汽车,旅游,军事

旅游,美女,娱乐,汽车,时尚
星座命理
大学,随性,动漫迷,金融,90 后,爱幻想,旅游
视频音乐,校园生活,新闻趣事,旅游,健康,星座命理,美食,文艺
时尚,美食
娱乐,美食
体育
汽车
叶池霸气汉子,娛樂,音樂
旅游
体育
汽车
游戏动漫,娱乐,文艺
旅游
美食,星座命理,健康,游戏动漫
汽车,游戏动漫,美女,体育
体育
教育就业
体育,美食
手机,爱自由,IT
美女,娱乐,旅游
教育就业,旅游,美食
文艺
新闻资讯,星座命理,文艺
娱乐
美食,微博奇葩,视频音乐,新闻趣事,搞笑幽默,娱乐,文艺,IT 数码,体育
教育就业,投资理财,健康,IT 数码,娱乐
星座命理
娛樂,文藝,美食,健康
IT 数码,教育就业,投资理财,美食,文艺,旅游
旅游,爱生活,外貌协会,文艺
魔羯座,小说,宅男,旅游美食爱好者,我就是我,电影爱好者,矛盾体,玩儿,游戏动漫,体育
娱乐,文艺,体育
时尚,文艺,娱乐
旅游,IT 数码,体育
健康,新闻资讯,文艺
新闻资讯,美女,时尚
深圳生活,健康
新闻资讯
文艺,新闻资讯,投资理财
健康,新闻资讯,旅游,娱乐
娱乐
文艺,时尚,美食
自由,射手女,吃货,乐观,健康,旅游,文艺

体育,健康,美食,文艺,时尚,娱乐
爱自由,军事,娱乐,旅游,健康
爱笑,互粉,睡觉,看书,摄影,自由,时尚,北京,做梦,坚强
凉山生活
时尚,星座命理,美食
搞笑幽默
美女,萌宠,美食,四川生活,IT 数码,体育
名人明星,微博奇葩,视频音乐
健康,星座命理,娱乐
3D 打印,无线电,单片机,Arduino,开发板,开源硬件,物联网,智能控制,电子电路,电子 DIY
游戏动漫,旅游,文艺,体育,娱乐
时尚,音乐控,学生一族,美食
健康,时尚,美食,娱乐,投资理财
娱乐,新闻资讯,文艺,旅游,美食,游戏动漫
美图摄影,爱音乐,旅行,听歌,娱乐,时尚
IT 数码,文艺,时尚,汽车,美女,星座命理,娱乐
旅游
新闻资讯,旅游,美女,汽车,娱乐
体育,宅,听歌,旅行,电影
呈贡街舞,大学生街舞培训,街舞比赛,昆明街舞,大学生街舞,呈贡大学城,呈贡大学城街舞,
云南街舞培训
美食,投资理财,健康,新闻资讯,教育就业
新闻资讯,汽车
相信自己
时尚,韩国,网络营销,摄影爱好者,美女,时装,购物狂,女人,文艺
吃货一枚,懒,80 后的尾巴,孩子气
星座命理,娱乐
健康
文艺
美食,星座命理,娱乐,文艺,时尚
逛街,吃货一枚,臭美,爱生活,看电影,爱自由,登山,旅游
美食
娱乐,星座命理,旅游,时尚
文艺,游戏动漫,娱乐
美食
自由,聊天,感性,学生一族,平常心,网购,张杰,活泼,偶喜欢玩,IT 数码
美女,娱乐,新闻资讯,汽车,体育
IT 数码,星座命理,文艺,娱乐
体育
旅游
小说,学生,自由,闷骚,影视,文艺,娱乐,旅游,IT 数码,游戏动漫
新闻资讯
娱乐
双鱼女,偏执狂,时尚,文艺

旅游,健康,新闻资讯,美女
时尚,娱乐
健康,美食,娱乐
游戏动漫,旅游,美食,文艺,娱乐
IT 数码,新闻趣事,美女,美图摄影
美食
健康
美女
喜欢唱歌,心情,时尚,旅游,娱乐,服饰美容,购物,健康,美食,音乐爱好者
星座命理,时尚
美容服饰
强辩乐团,强辩黄牛,黄少谷,强辩
美食,IT 数码,文艺,时尚,投资理财
健康,投资理财,新闻资讯
军事,体育
美食
头像控,欧美范,BIGBANG,娱乐,文艺
文艺,美食,娱乐
时尚,娱乐,美食,星座命理,旅游
旅游,汽车
美食
娱乐
吸血鬼
新闻资讯,军事
娱乐,汽车,美女,军事,体育
美食
求男饭勾搭,团饭一枚,CP 饭,行星饭
旅游
文艺,体育,娱乐,投资理财
萌宠,时尚
宠物图集,八卦杂谈,名人明星,娱乐,星座命理,文艺
新闻资讯
汽车,IT 数码,文艺
新闻趣事,文艺
健康,投资理财,旅游,娱乐,汽车,美女
教育就业,健康,旅游,文艺
搞笑幽默,视频音乐,微博奇葩,美食,穿衣美容,娱乐
旅游,星座命理
娱乐
读书分享,校园生活,美图摄影,情感生活,我是 90 后,花痴,狗狗,健康,时尚,体育
dota 爱好者
教育就业,投资理财
美女
投资理财

时尚,旅游,美食,星座命理,娱乐
时尚,文艺
时尚
时尚,健康,美食,文艺,娱乐
旅游,健康,星座命理,美食,娱乐
宅,自由,睡觉,学生,电视剧,游戏,90 后,dota,射手座,体育
旅游
名人明星,穿衣美容,视频音乐,搞笑幽默,时尚,文艺,娱乐
旅游,初级代购一枚
IT 数码
娱乐,美食
名人明星,娱乐
新闻资讯,娱乐,时尚,旅游,文艺
娱乐,文艺,体育
穿衣美容,投资理财,健康,IT 数码,文艺,乐橙,IT 互联网,旅游摄影,计算机,热情
美食,文艺,旅游,时尚
搞笑幽默,军事
星座命理,微博奇葩,90
其他生活
健康,新闻资讯
白羊座 O 型血,奋斗,善良,服饰,高中生,神经大条,吃货一枚,任性,shopping,蓝色
美女,游戏动漫,体育
打游戏，看电影
美女,时尚,旅游,汽车,娱乐
军事,健康,娱乐,IT 数码,美食
娱乐,IT 数码,旅游,美食
旅游,美食
健康,新闻资讯,新浪读书,新闻,考研,旅游,文艺,汽车,体育,娱乐
体育,美女
美食
阅读,文艺,体育
萌宠,微博奇葩,旅游,搞笑幽默,穿衣美容
仿古卫浴,高档家具,生活,高品质,奢侈,新闻资讯,旅游
汽车,军事,体育
娱乐
媒体,传媒,广告,IT 数码,文艺
投资理财,旅游,健康,娱乐
白羊座,简单,摄影,吃货一枚,文艺
星座命理,萌宠,微博奇葩,搞笑幽默,新闻趣事,教育就业,文艺,娱乐,新闻资讯
文艺,美食,旅游,时尚,新闻资讯
美图摄影,体育资讯,沈阳生活,视频音乐,旅游,美食,娱乐,体育,文艺
IT 数码
名人明星,视频音乐,新闻趣事,微博奇葩,搞笑幽默,游戏动漫,汽车,体育
教育就业,投资理财,健康,旅游,美食

搞笑幽默
美女,体育,旅游,星座命理,娱乐,投资理财,汽车
教育就业
江苏生活,淡定,纠结,内向,旅游,星座命理,IT 数码,文艺,娱乐
旅游,娱乐,IT 数码,体育
新闻资讯,健康,旅游
美食,娱乐,文艺,时尚,美食,旅游
旅游
娱乐,新闻资讯,美女,游戏动漫,美食,IT 数码
星座命理,旅游
美食,IT 数码,体育,娱乐,时尚
游戏动漫,时尚,星座命理
美图摄影,新闻趣事,沈阳生活,搞笑幽默,文艺,娱乐,健康
时尚,娱乐
美女,文艺
健康,汽车,军事
汽车,旅游,体育
教育就业,文艺,新闻资讯
名人明星
搞笑幽默,娱乐
娱乐,文艺
体育资讯,90 后但我不脑残,娱乐,篮球,电影控,射手座,矛盾体,科比,赛车
时尚,健康,文艺
时尚,旅游,文艺
品牌,星座命理,投资理财,体育
美食,教育就业,旅游,健康,美女,美食
时尚,娱乐,星座命理,美食,健康
游戏动漫
文艺,娱乐
旅游,健康,时尚,文艺,星座命理,美食
教育就业,健康,星座命理
新闻资讯,汽车,投资理财,旅游,军事,IT 数码
读书分享,文艺,教育就业,新闻资讯
美女,康熙来了,娱乐
视频音乐,美食,萌宠,搞笑幽默
美食
文艺,IT 数码,军事,美食,星座命理
文艺,娱乐,美女
健康,IT 数码
IT 数码,军事
娱乐
投资理财,旅游
搞笑幽默,娱乐
美食,教育就业,文艺

旅游,新闻资讯,健康,美食
IT 数码,美女,新闻资讯
娱乐,汽车,军事,旅游,美女
美食
整理控,双鱼座,80 后
教育就业,时尚,军事,健康,旅游
旅游
钓鱼,双重性格,看电视,篮球爱好者
体育,旅游,美女,时尚,文艺,娱乐
体育
体育
旅游
健康,旅游,美食
广州生活,健康
投资理财,美女,汽车
娱乐,美女,军事,IT 数码,游戏动漫
90 后非脑残,化妆,旅游摄影,猫控,处女座,腐女,武汉,coser,动漫控
IT 数码
汽车,投资理财,军事,IT 数码,体育
搞笑幽默,教育就业,娱乐,文艺,军事
单身,空调扇,环保空调,工业冷风机,家用冷风机
旅游,汽车,星座命理
旅游
汽车,布丁,美食,自由,旅行,听歌,宅,90 后
投资理财
投资理财,文艺,美食,时尚,IT 数码,旅游,星座命理,娱乐
文艺,投资理财,新闻资讯,旅游
新闻资讯,投资理财
军事,美女
文艺,游戏动漫,美女,娱乐,体育
文艺,天枰,卡通动漫,文字,欧美,傻逼,小清新
星座命理
美食,新闻资讯,健康,文艺
搞笑幽默,视频音乐,旅游
娱乐
旅游
时尚,星座命理,美食,文艺,娱乐
健康,美女,星座命理,娱乐,时尚
美食,时尚,娱乐,健康
新闻资讯,汽车,教育就业,娱乐,旅游
Music,文字控,时尚,交朋友,宅女一枚,彩妆,减肥,时装,快乐大本营
娱乐
文艺,娱乐,时尚
新闻资讯,旅游,健康,美食

读书分享,吃货,体育
无油水润滑,空压机
健康,旅游,美食
新闻资讯,游戏动漫,美食,娱乐
美食
星座命理,投资理财,新闻资讯,健康,旅游
军事,旅游,教育就业,汽车,IT 数码,文艺,体育,娱乐
文艺,健康,旅游,星座命理,时尚
时尚
游戏动漫,旅游,军事,体育,娱乐
新闻趣事,穿衣美容,昆明生活,汽车,时尚,文艺,旅游
新闻资讯,时尚,娱乐,星座命理
新闻趣事,穿衣美容,星座命理,美食,新闻资讯,健康,旅游
星座命理,汽车,军事,IT 数码,投资理财
投资理财,星座命理,体育
娱乐,文艺
娱乐
时尚
娱乐,汽车,美女,新闻资讯,IT 数码
娱乐,时尚
投资理财,旅游,新闻资讯,汽车,军事
健康,娱乐,文艺
文艺,健康,旅游,时尚,教育就业
健康,旅游,文艺,娱乐,美食
美食
娱乐,星座命理
狮子女,活泼开朗,真实力,玉米,多愁善感,公益,亲情,自由
军事,IT 数码,文艺,投资理财,美食
IT 数码,军事,游戏动漫
娱乐,星座命理
游戏动漫,时尚,体育,美女,娱乐
投资理财,娱乐,美食,旅游
教育就业,新闻资讯,健康,旅游,文艺
新闻资讯,美食,汽车,时尚,健康,军事
娱乐
娱乐,新闻资讯,汽车,教育就业,游戏动漫,美食
健康,时尚,旅游,星座命理,美食,文艺,娱乐
教育就业,美食
体育
星座命理,时尚,健康,汽车
美食
健康,星座命理,投资理财,美食,娱乐
上海生活,新闻资讯
桌球,时尚

美女,娱乐,体育
新闻资讯,文艺,美食,星座命理
IT 数码,娱乐,汽车,时尚,游戏动漫
汽车,旅游,健康,美女,体育
美食,星座命理,游戏动漫,娱乐,投资理财
文艺,娱乐,旅游,美食,健康,时尚
美女
吃货,穿衣美容,视频音乐,美食,山东生活,时尚,教育就业,星座命理
军事,新闻资讯,教育就业
哈里森,网球,Ryanharrison,体育
旅游,美食,美女,新闻趣事,广东生活,体育
汽车,投资理财,时尚,IT 数码,文艺,娱乐
张大大,小乖,霸气侧漏,爱睡觉
美食,星座命理,文艺,娱乐,健康,体育
IT 数码
娱乐,健康,旅游,文艺,时尚
文艺,健康,美食
新闻资讯,娱乐,旅游
音乐达人,睡懒觉,摩羯女,工作,疯狂,潮,随性,乐天派,粤语,娱乐
时尚
美女
双鱼女,新闻资讯,时尚,旅游,美食,IT 数码
美食,娱乐
军事
娱乐,军事
投资理财
时尚,编织,健康
美食,娱乐,旅游
福建生活,搞笑幽默,新闻趣事,微博奇葩,体育,游戏动漫,IT 数码,汽车
医学,创意,美食,户外,摄影,唱歌
广东生活,汽车
娱乐,时尚,文艺
新闻资讯,健康,星座命理,美食
浙江生活,80 后,美食
旅遊,体育
美食,星座命理
娱乐,体育
健康,美食,旅游,投资理财
旅游
体育
时尚,健康,旅游
娱乐
IT 数码
文艺,看电影

汽车,旅游,投资理财,军事,文艺
星座命理,时尚,教育就业,投资理财,美食,娱乐
体育,游戏动漫
旅游
旅游,时尚
娱乐
美食,星座命理,旅游,文艺
军事
新闻资讯,汽车,健康,旅游,美食
健康,时尚,娱乐
Beauty,Melody,Beast,Btob
好性格,服饰,自由,家居,可爱,减肥,美容,健康,时尚,文艺
投资理财,娱乐,美食,健康,旅游
旅游,美食,星座命理,体育,娱乐
动漫控,吃货一枚,我就是我
健康
娱乐,文艺,健康,旅游,投资理财
娱乐
美女,军事,文艺,美食
娱乐,教育就业,健康,旅游,星座命理
体育资讯,投资理财,汽车,体育
时尚,健康,星座命理,旅游,美食
时尚,旅游,娱乐,游戏动漫,星座命理
旅游,投资理财,美女
美食
时尚,汽车,健康,美女
游戏动漫
90 後,音乐爱好者,篮球,努力,时尚,投资理财,旅游,文艺,体育
娱乐
新闻资讯
星座命理,旅游,时尚,美食,娱乐
美食,美女
旅游,视频音乐,星座命理,新闻资讯,时尚
新闻趣事,视频音乐,微博奇葩,健康,新闻资讯,旅游,美食,文艺,娱乐
娱乐,美食,旅游,星座命理,时尚
健康,时尚,美食,娱乐
星座命理
IT 数码
搞笑幽默
体育,投资理财,时尚,美女,汽车
美食爱好者,开心,情绪化,学生一族,独立,娱乐,时尚,健康,旅游,美食
健康,美食
新闻资讯
美食

IT 数码,时尚,健康,教育就业,美食
游戏动漫,旅游,星座命理,美食,体育
教育就业,文艺
新闻资讯,投资理财,美女,汽车,体育
星座命理
投资理财
星座命理,美食,娱乐
旅游,健康,新闻资讯
旅游,情绪化,投资理财,教育就业,美食,娱乐
娱乐,新闻资讯,时尚,文艺,旅游
新闻资讯,汽车,美女,旅游,美食
旅游,读书分享,星座命理
搞笑幽默,新闻趣事,上海生活,军事,教育就业,投资理财
奋斗,工作,生活,低调,网购,大学生,旅游
旅游,美食,IT 数码
动漫,健康,旅游,教育就业,时尚,文艺
新闻资讯,文艺,IT 数码,健康,教育就业,投资理财
四川生活,旅游,萌宠,穿衣美容,迷糊,动画,爱睡觉,美食
旅游,汽车,IT 数码,体育
娱乐,文艺,星座命理,美食,美女,时尚,淡定,热爱生活,时尚达人,淘宝购物达人
汽车,IT 数码,游戏动漫,娱乐
文艺,新闻资讯
处女座,韩国,绘画,music,古典艺术,梦想,浪漫,时尚,健康,旅游
美食
旅游,美食,星座命理,四川生活,美女,新闻资讯
美食
双重性格,狮子男,哈尔滨,80 后 90 前,休闲,健康,投资理财,美食,军事,汽车
体育
旅游,投资理财,新闻资讯,美女,时尚
健康,新闻资讯,投资理财,时尚,旅游,美食
游戏动漫
投资理财,时尚,健康,旅游,美食
教育就业,投资理财,时尚,文艺,娱乐
视频音乐,校园生活,搞笑幽默,动漫,名人明星,学生党
游戏动漫
美女,遊戲動漫
穿衣美容,美食,旅游,游戏动漫
搞笑幽默,美食,体育,游戏动漫
搞笑幽默,萌宠,美食
文艺,美食控,喜欢听歌,情绪化,偏执狂,服饰,潮人,青春,睡懒觉,旅游
美女,旅游,美食,军事,娱乐
娱乐
时尚,音乐控,心理学,火影,文字,旅游,娱乐,IT 数码,星座命理,文艺
旅游,IT 数码,新闻资讯,汽车

汽车,新闻资讯,健康,旅游,娱乐
健康,时尚,娱乐,旅游,美食
汽车,体育,IT 数码,游戏动漫
旅游
池州生活
搞笑幽默
文艺,旅游,美食,娱乐,时尚
汽车
军事,娱乐,IT 数码,体育
旅游
健康
健康,时尚
健康,文艺,美女,时尚,娱乐
旅游,美食,体育,娱乐
健康,教育就业,投资理财
娱乐
健康,星座命理,旅游,美食
文艺
娱乐,游戏动漫
健康,新闻资讯,旅游,美食,文艺
搞笑幽默,美术设计,场景原画,CG 插画,游戏原画设计师,设计师,动漫迷,动漫控,绘画,游戏动漫
健康,旅游
健康,娱乐
游戏动漫,体育
文艺,星座命理
美女
微博奇葩,广州生活,新闻趣事
旅游
健康,体育,美食
微博奇葩
时尚
游戏动漫,文艺,娱乐
时尚,文艺,健康,体育
投资理财
萌宠,穿衣美容,星座命理,文艺
文艺,体育,娱乐
教育就业
美食
游戏动漫,美女,时尚
娱乐
健康
娱乐
新闻资讯

新闻趣事,视频音乐,微博奇葩,北京生活,穿衣美容
美食
旅游,军事,IT 数码,文艺,娱乐,体育
娱乐
体育
时尚,体育
旅游
军事,IT 数码,体育,娱乐
美女,穿衣美容
体育
时尚
旅游,娱乐
星座命理,健康,游戏动漫,汽车,时尚,投资理财,IT 数码,文艺,体育,娱乐
名人明星,旅游,穿衣美容,娱乐
游戏动漫,体育
江苏生活,投资理财
美食
星座命理,时尚,投资理财,娱乐
新闻资讯,游戏动漫,军事,文艺,娱乐
美女,汽车,时尚,旅游,美食,体育
时尚,健康
娱乐,军事
军事,IT 数码,体育,汽车,投资理财
旅游
游戏动漫
香港生活,IT 數碼
旅游
娱乐
体育,文艺
美女
美食
新闻资讯
游戏动漫
新闻资讯,健康,美女,汽车,文艺
旅游
美食,教育就业
旅游
美女,娱乐,文艺,投资理财,旅游
购物,音樂,减肥,爱睡觉,玩儿,八卦,懒,爱电影,爱旅行
美女,娱乐,体育,游戏动漫
时尚
美女,汽车,星座命理,体育,娱乐
北京生活,体育
游戏动漫,新闻资讯,投资理财

旅游,美食,时尚,文艺,娱乐
军事,IT 数码
时尚,教育就业,美女,新闻资讯,军事
星座命理,文艺
美食
美食,时尚,娱乐,体育,投资理财
旅游,新闻资讯,投资理财,教育就业,健康
教育就业,游戏动漫,旅游,娱乐
新闻资讯,健康,投资理财
新闻资讯
搞笑幽默,穿衣美容,汽车
时尚,星座命理,美食,游戏动漫,娱乐
美食,旅游,健康,时尚,星座命理
娱乐,文艺,时尚
美食,旅游,健康,美女,教育就业
视频音乐,娱乐
健康,军事,IT 数码,美女,投资理财
80 后宅男,汽车,美食,旅游,军事,体育
美食,文字,摄影,旅行,朋友
时尚
新闻趣事,美女,搞笑幽默,星座命理,视频音乐
汽车
汽车
学生一族,动漫,时尚
汽车,时尚,星座命理,美食,军事,文艺,新闻资讯,美女
单身,爱睡觉,红酒
新闻趣事
美食,投资理财,旅游,文艺,娱乐
IT 数码,旅游,游戏动漫,时尚,娱乐
新闻趣事,美食,旅游,微博奇葩,搞笑幽默,娱乐
健康,时尚,旅游,星座命理,美食
旅游
旅游,美食,娱乐
文艺,新闻资讯,星座命理,娱乐,教育就业
投资理财,文艺,娱乐
赫海,ELF,SuperJunior
投资理财,汽车
吉他爱好者,冷笑话,校园,双重性格,学生一枚,摄影师,搞笑幽默,喜欢听歌
游戏动漫
湖人,吃货一枚,美女
星座命理,军事,体育
搞笑幽默,健康,教育就业,文艺,娱乐,游戏动漫
时尚,旅游,美食,文艺,娱乐
旅游,健康,新闻资讯,娱乐,美食

美女,健康,旅游,美食,娱乐
体育
旅游,美食,萌宠,星座命理,搞笑幽默,娱乐
体育
新闻资讯,时尚
娱乐,新闻资讯,体育
美食,旅游,美女,时尚,健康
孩子气,多愁善感,日语,双子座,矛盾,看小说,动漫迷,爱玩
体育,健康,旅游,时尚,娱乐
时尚,娱乐
健康,游戏动漫,旅游,美女
萌宠,微博奇葩,娱乐,时尚,旅游,星座命理,美食
美食,旅游,文艺
搞笑幽默,己所不欲,勿施于人
新闻资讯
美食,旅游,娱乐,爱旅行,白羊女,生活大爆炸,90,懒人
投资理财
舞蹈爱好者,瑜伽,旅游美食爱好者,爱美食,疯子,独立,文艺
新闻资讯,军事
旅游,体育
IT 数码
星座命理
体育
美食,旅游,汽车,新闻资讯,娱乐
旅游,健康,文艺,汽车
健康
时尚,娱乐
美食
健康,投资理财,旅游,星座命理,美食
名人明星,体育,娱乐,新闻资讯,文艺
北京生活,军事,汽车,体育
投资理财,教育就业,旅游
美女
策划,市场营销,习酒,茅台酒,白酒销售
新闻资讯,美女,体育
上海生活,旅游,健康,教育就业,文艺,IT 数码
健康,新闻资讯,体育,娱乐,军事
新闻资讯,教育就业,星座命理,美食,文艺
90 后
美女,娱乐,Music,学生一族,宅女加腐女,爱生活,音乐达人,懒,爱玩
美食,宅,星座,吃货,体育,80 后
体育
绿色无公害,有机蔬菜,生态养殖,农业,美食
文艺,美女

军事,美食,文艺,IT 数码
时尚,新闻资讯,星座命理,文艺,体育
情感生活
教育就业,体育,游戏动漫
美食
旅游,娱乐
星座命理,娱乐,美食,文艺
星座命理
文艺,娱乐,美食
李東海,李赫宰,SJM,赫海,DongHae,EunHyuk,EunHae,superjunior,旅游
搞笑幽默,穿衣美容,时尚,文艺
阳光,喜欢听歌,娱乐
文艺
娱乐
文艺,娱乐,电影爱好者,80 后的尾巴,爱音乐
IT 数码,时尚,旅游,星座命理,美食
旅游美食爱好者,育儿,时尚
星座命理,旅游
娱乐,汽车,新闻资讯,旅游
文艺
旅游
IT 数码,文艺,体育,娱乐
健康
文艺,娱乐,美食,活波开朗
游戏动漫,美女
新闻资讯,时尚,美食,旅游,健康
体育,教育就业
阅读,拍照,音乐,天蝎,星座命理,旅游,健康
IT 数码,娱乐,游戏动漫
苏州生活
健康,星座命理
萌宠,时尚
时尚,汽车,游戏动漫
室内设计,90 後,IT 数码,娱乐
星座命理,健康,娱乐,动画,时尚,旅游爱好者
体育
法国生活
IT 数码
健康,平常心
体育,娱乐
娱乐
旅游,美食,文艺,IT 数码
IT 数码,旅游,时尚,新闻资讯,汽车,游戏动漫,投资理财,健康
时尚,健康,投资理财,汽车,星座命理

体育,投资理财,汽车,美食,旅游,星座命理
旅游
投资理财
健康,时尚,美食,娱乐
时尚,娱乐,文艺
汽车
手工 diy,日系,森林系,时尚,旅游,美食,文艺
时尚
B 型血,吃货一枚,愤青,完美主义,设计师,爱,看电视,喜欢旅游,旅游,时尚
旅游,美食,娱乐
文艺,美食,健康
汽车,IT 数码
美食,娱乐
化妆,欧美音乐,养生,单身,神经大条,街舞,外贸,吃货一枚,活泼开朗,美食
穿衣美容
健康
时尚
IT 数码
体育,军事
时尚,军事,文艺,美食,投资理财,汽车,体育
新闻资讯
美食,星座命理,健康,娱乐,时尚,旅游
娱乐
娱乐,美食,IT 数码,体育,投资理财
名人明星
汽车
美食,时尚
体育,游戏动漫
时尚,健康,星座命理,美食,旅游
新闻资讯,旅游,星座命理,美食,健康
文艺,旅游,体育,星座命理
逛街
体育
美女,美食,IT 数码,汽车,体育
花样美男,美容服饰,八卦杂谈,新闻趣事,宠物图集,娱乐
文艺
文艺
美食,时尚
游戏动漫,汽车,时尚,美女
穿衣美容
IT 数码,体育,娱乐
旅游,娱乐,文艺,体育
旅游,体育
新闻资讯,美食,健康,旅游,体育

军事
IT 数码, 旅游, 美食, 体育, 娱乐
体育
游戏动漫
爱旅游, 美剧, 天蝎座, 美女, 购物, 美食, 淘宝, 自信, 时尚
美食, 投资理财
票房, 新番旧番
新闻趣事, 投资理财, 新闻资讯, 健康
娱乐, 星座命理, 军事, 汽车, 游戏动漫
美女
旅游, 体育
旅游
游戏动漫, 投资理财, 新闻资讯, 健康, IT 数码, 体育
美女, 爱睡觉, 爱生活
穿衣美容, 快乐, 爱笑, IT 数码, 文艺, 娱乐
文艺
旅游, 星座命理
时尚, 文艺
新闻趣事
星座命理, 美食, 健康, 娱乐, 投资理财, 旅游
文艺, 健康
时尚
娱乐, 美食, 体育, 游戏动漫, 文艺
娛樂
文艺
搞笑幽默, 军事, 游戏动漫, 文艺, 时尚
IT 数码
搞笑幽默, 娱乐
投资理财
体育
明星汇, 洪辰
体育, 投资理财
旅游, 新闻趣事, 搞笑幽默, 美食, 衡阳生活
旅游
健康
游戏动漫, IT 数码, 体育
教育就业
新闻资讯
星座命理, 文艺, 健康, 时尚, 娱乐
军事
视频音乐, 搞笑幽默, 新闻趣事
旅游, 美食, 文艺, 娱乐, 游戏动漫
军事, 旅游, 文艺, 体育, 娱乐
体育, 旅游, IT 数码, 美食, 文艺

心事,树洞,表白,暗恋
多愁善感,睡觉睡到自然醒,强迫症,大学,校园,听歌,90 后,IT 数码,时尚,文艺
美女,体育,娱乐
体育
旅游,汽车,游戏动漫
游戏动漫,汽车,美食,旅游,IT 数码
健康,旅游,教育就业,娱乐,文艺
旅游,文艺,健康,星座命理,美食,时尚
旅游,朋友,天秤,jugging,单曲循环,电影,日料,英伦控,自由
体育
洛阳生活,新闻趣事,穿衣美容,美食,时尚,星座命理,娱乐
旅游,新闻资讯,健康,IT 数码,体育
篮球爱好者,开心,单身,活泼,漫画,阳光,淡定,跑步,内向,文艺
旅游
教育就业,健康
佛学感悟,狼亲,健康,旅游
美女,游戏动漫,星座命理,体育,娱乐
旅游,健康,时尚,教育就业,美食,文艺,娱乐
时尚,美女,游戏动漫,娱乐,体育
搞笑幽默,美女,旅游,湖北生活
旅游,教育就业,投资理财,军事,文艺,处女座,单身,宅
健康,新闻资讯,美食
萌宠,旅游,美女,搞笑幽默,美食
河北生活
重口味,减肥,开朗,大学生,偶稀饭睡觉觉,双子座
旅游,星座命理,军事,文艺,体育
读书分享,新闻资讯,IT 数码,教育就业,投资理财,星座命理
游戏动漫
游戏动漫,时尚,健康,新闻资讯,汽车
娱乐
IT 数码,游戏动漫,美女,教育就业,娱乐
新闻资讯,旅游,星座命理,娱乐
健康,旅游,美食,娱乐
内蒙古生活,新闻趣事,视频音乐,旅游,时尚,健康,美食,体育
军事,旅游,健康,投资理财,时尚
健康,游戏动漫,IT 数码,美食,军事
军事,星座命理,时尚,文艺
白羊座,音乐,自然,电影,游戏,阿信,海贼王
时尚,健康,美食,旅游
星座命理,IT 数码,军事,旅游,汽车,游戏动漫
名人明星,文艺,旅游,时尚,娱乐
教育就业,文艺,健康,旅游,美食
汽车,文艺,时尚
腐,爱笑,钢琴,欧美,音乐生,东莞,虎门,金贤重,旅游,电影

时尚,新闻资讯,健康,旅游
娱乐,游戏动漫
学生
娱乐,时尚,星座命理,美食,健康
健康,体育,旅游
旅游
健康,时尚
旅游
教育就业,健康,美食,文艺,新闻资讯
新闻资讯,旅游,投资理财,文艺,娱乐
娱乐
安徽生活,体育,新闻资讯,教育就业,投资理财
旅游,美食,汽车,美女,IT 数码,时尚
时尚
游戏动漫
投资理财,健康,IT 数码
文艺,时尚,健康,教育就业,娱乐
健康,文艺,娱乐,星座命理
美女
娱乐
星座命理
美食,投资理财,娱乐
忧郁,85 后,单身,爱生活,工作,沉默,睡懒觉
新闻资讯,军事,美食
旅游,新闻趣事,搞笑幽默,美女,娱乐
娱乐
游戏动漫
娱乐,投资理财
双子座,格子控,文字控,O 型血,宠物,美食
汽车,美食,军事,好学僧,宅男,90 后,IT 数码,体育,娱乐
音乐爱好者,投资理财,IT 数码
文艺,旅游
艺术,平常心,网购,双鱼座,健康,新闻资讯,美女,时尚,IT 数码
文艺,吃货一枚,空姐,娱乐
O 型血,摄影爱好者,手机控
名人明星,娱乐
娱乐
娱乐
星座命理,星座控,爱自由,校园,a 型血
健康
搞笑幽默,时尚,投资理财,教育就业,游戏动漫,娱乐
文艺
健康
美食

真心吃货,ACCA 学员,总要出去历练吧,河南郑州人,奋斗中的 90 后,设计师梦想破灭,晚睡强迫症
新闻资讯
搞笑幽默,美食,猫咪控,爱睡觉,爱美食
健康,时尚,美女,投资理财
娱乐,旅游,新闻资讯,教育就业,美食
时尚
舞蹈,喜欢唱歌,双子女,音乐爱好者,臭美,瑜伽,执着,90 后,快乐,星座控
视频音乐,穿衣美容,搞笑幽默,美女,微博奇葩
美食,娱乐
星座命理,文艺
视频音乐,幽默,篮球,摄影,唱歌,娱乐
自由,美女,教育就业
美女,汽车,娱乐
军事,健康
贵州生活,娱乐
娱乐
健康,旅游,军事,娱乐,文艺
星座命理
妖媚,复古控,Dancer,时尚,文艺,娱乐
旅游,文艺,健康,新闻资讯,美食
时尚,新闻资讯,健康,教育就业,旅游
猫咪控,网购,吃,shopping,旅游
时尚,娱乐,星座命理
时尚,旅游,文艺,娱乐
新闻资讯,汽车
娱乐
文学,金牛座,羽毛球,娱乐,时尚,美食,IT 数码
睡觉睡到自然醒,萌宠,旅游,新闻趣事,北京生活,美女
旅游,教育就业,游戏动漫,美女,IT 数码
美食,旅游,时尚,文艺,娱乐
新闻趣事,文艺
投资理财
动漫,搞笑幽默,体育
时尚
时尚
体育
感性,多愁善感,夜猫子,文艺,健康,旅游,新闻资讯
美食,旅游
文艺,娱乐
投资理财,汽车,IT 数码,文艺,游戏动漫
旅游
投资理财,教育就业,健康,星座命理,文艺
时尚

新闻资讯,美女,健康
重口味
旅游
IT 数码,娱乐
吃,游戏动漫,娱乐
美女,时尚
教育就业,旅游,美食
美女,美食,军事,IT 数码,娱乐
时尚,IT 数码,星座命理,文艺,娱乐
时尚
游戏动漫
游戏动漫,军事,文艺,体育,娱乐
投资理财,旅游,新闻资讯
游戏动漫
娱乐
教育就业,新闻资讯,健康,游戏动漫,旅游
文艺
投资理财,美女,体育,汽车,教育就业,健康,娱乐
新闻资讯
健康,文艺,新闻资讯,旅游
美女
萌宠,美短米,边境米,龙猫米
新闻资讯,健康,游戏动漫,投资理财,军事,娱乐,IT 数码
投资理财,娱乐,文艺,体育
娱乐,体育
娱乐
萌宠,游戏动漫,文艺,体育
投资理财
体育
时尚
星座命理,美食
体育,旅游,美女,游戏动漫,军事
健康,教育就业,投资理财,娱乐,时尚
视频音乐,旅游,美食,搞笑幽默,美女
美女,时尚,旅游,文艺,体育
校园生活,花样美男,读书分享,动漫,IT 数码,游戏动漫,星座命理,娱乐
娱乐,文艺,时尚,旅游,游戏动漫
情感生活,九江生活,爱美食,爱音乐,懒兔一只
旅游,文艺,时尚,低调,音乐爱好者,美女,健康,娱乐
娱乐
新闻资讯,汽车,旅游,美食,IT 数码
新闻资讯,美食,星座命理,文艺,IT 数码
IT 数码,健康,游戏动漫,时尚,娱乐
星座命理,时尚

文艺,时尚,健康,旅游,美食,娱乐
新闻资讯
旅游,潮流玩具,Frank,Toy2r,Mindstyle,Kidrobot,3A,dunny,潮玩店
许嵩,文藝,杨泌,圆三三,穷二代
健康,IT 数码,汽车,时尚
娱乐,汽车,旅游
星座命理
健康,投资理财
投资理财,健康,旅游,IT 数码,军事
旅游
健康,时尚,汽车,文艺,娱乐
文艺,美食,星座命理,健康,娱乐
游戏动漫
娱乐,90 後,宅女加腐女,k 歌
教育就业,军事,娱乐
旅游,时尚,娱乐,美食
健康,汽车,文艺,体育
文艺,健康,投资理财,星座命理,娱乐
美女
IT 数码,星座命理,美女,健康
星座命理,美食,娱乐
花样美男,旅游,读书分享,美容服饰,IT 数码
健康
IT 数码,文艺,体育,娱乐,时尚
时尚,娱乐,美食,旅游,健康
美食,时尚,娱乐,旅游,星座命理
时尚,美女,军事,娱乐,文艺
时尚,美女,IT 数码
健康,教育就业,星座命理,娱乐
娱乐,体育,文艺,IT 数码
游戏动漫,美食,军事,IT 数码,汽车
新闻资讯,健康,旅游,美食,娱乐
矛盾体,听音乐,90,搞笑,幽默,温柔,旅游,美食,时尚,健康
娱乐
星座命理,美食
汽车
搞笑幽默,娱乐,健康,时尚
辽宁生活,文艺
娱乐,美食,文艺,时尚,星座命理
美食,旅游
娱乐,时尚,旅游
体育,教育就业
崔珉豪,SHINee
体育,旅游

健康,美食,IT 数码,体育
星座命理,旅游,美食,穿衣美容,搞笑幽默
健康,星座命理
健康,旅游,星座命理,美食,娱乐
健康,教育就业,旅游,美食
美食,星座命理,军事,文艺,娱乐
文艺,体育,娱乐
游戏动漫
IT 数码,军事,凌志众成,领导力培训,教练技术,教育就业
IT 数码,文艺,娱乐,潮,粤语,体育
新闻资讯,旅游,美食,投资理财,健康,IT 数码,汽车
健康
娱乐,旅游,健康,新闻资讯,文艺,时尚,星座命理
星座命理,微博奇葩,美食,旅游,搞笑幽默
时尚
旅游,美女,美食,IT 数码,汽车
搞笑幽默
听音乐,旅游,音乐,娱乐
旅游,健康
Golf,摄影旅游
历史,互联网,dota,游戏动漫
多愁善感,日语,恐怖片,90 后非脑残,表演,学生一枚,热情,文艺,时尚,娱乐
美女,游戏动漫,军事,IT 数码,娱乐
搞笑幽默,宠物图集,星座运势,美图摄影,旅游
娱乐
娱樂
萌宠,微博奇葩,北京生活,IT 数码,文艺,新闻资讯
游戏动漫,投资理财,军事
文艺
游戏动漫,军事,汽车,IT 数码
教育就业,健康,新闻资讯,军事,体育
旅游,体育,娱乐,星座命理,文艺
投资理财,军事,娱乐
星座命理,IT 数码,军事,美女,旅游,健康
娱乐
微博,爱情,体育,旅游,美食,娱乐
娱乐
娱乐,星座命理,文艺,军事
娱乐
娱乐,星座命理,美食
健康,投资理财,IT 数码
校园生活,搞笑幽默,体育,军事,IT 数码,教育就业
娱乐,军事,旅游,汽车,体育
娱乐

新闻资讯,旅游,美食,文艺,娱乐
校园生活,汽车
美女,体育
健康,汽车,美食
文艺,时尚,娱乐
旅游,健康,美女
新闻资讯,时尚,星座命理,美食,文艺
苹果控,热爱生活,阅读,随性,怀旧,旅游,美食,教育就业,文艺,健康
找个老婆,奋斗 ing,樂天派,体育,汽车
汽车,星座命理,美食,娱乐,文艺
娱乐,美食
现货黄金,期货,股票,伦敦金,投资理财
微博,囧,玩儿,文字,单纯,赚钱,美女,IT 数码,文艺,娱乐
美女,教育就业,军事,体育
幽默风趣,幽默,Music,文字控,重口味,汽车,时尚,娱乐
投资理财,美食,健康
教育就业,美食,军事
时尚,星座命理,美食,文艺,娱乐
娱乐
汽车,美女,游戏动漫,娱乐,体育
美食,汽车,IT 数码,娱乐,游戏动漫
健身,玩,微博控,金融,旅游
新闻资讯,健康,美食,旅游
游戏动漫
体育,投资理财,新闻资讯
主持,电影,美食,完美主义,奋斗 ing,健康,旅游,星座命理,文艺,娱乐
文艺,娱乐
美食
娱乐,新闻资讯,教育就业,旅游
美食,时尚,旅游,文艺
美食,美女,体育
名人明星,娱乐
时尚,投资理财,汽车,娱乐,健康,美食
旅游,健康,美女,文艺,时尚
娱乐,美女
美食
美食,旅游,文艺,健康
美食,健康
游戏动漫,娱乐
美食,新闻资讯,文艺,体育,汽车
天蝎女,时尚
美女,投资理财
新闻资讯,娱乐,旅游,美食,投资理财
时尚,军事,IT 数码

文艺,教育就业,健康,时尚,美食
美食,健康
娱乐
广州猎头,手机,金牛座,教育就业
时尚
美女,美食
旅游,健康,教育就业
名人明星,体育,娱乐,219
时尚,文艺,健康,新闻资讯,娱乐
健康
旅游,娱乐
健康,文艺,旅游,娱乐
美女,时尚
新闻资讯,IT 数码,军事,体育,旅游
旅游,娱乐,IT 数码
投资理财
投资理财,新闻资讯
微博奇葩,名人明星,视频音乐,美食,旅游,投资理财,娱乐,体育,IT 数码,文艺
健康
旅游,美食
听音乐,低调,体育,健康
美食
文艺,美食,星座命理,旅游
美食
时尚,星座命理,娱乐
健康,游戏动漫,娱乐,美女
美食,旅游,文艺,娱乐
北京生活
视频音乐,慢递,永恒,梦想,公益,高尔夫,浪漫,文艺
万达集团,万达,万达广场,王健林,管理,语录,慈善,企业家,文化产业,商业地产
军事,健康
猫,游戏动漫,美食,旅游
娱乐
IT 数码
健康,投资理财
穿衣美容,旅游,健康,文艺,投资理财
体育,汽车,军事,IT 数码
喜欢音乐,旅游,健康,美食
时尚,星座命理
美食,娱乐
娱乐,时尚,汽车
教育就业
IT 数码,文艺,娱乐,旅游,新闻资讯,投资理财,汽车,军事
娱乐,游戏动漫

体育
新闻资讯,汽车,投资理财,IT 数码,美女
游戏动漫,健康,旅游,星座命理,文艺
游戏动漫,投资理财,IT 数码
体育,美女,游戏动漫
汽车
汽车
健康,汽车,娱乐,星座命理
设计师,绘画,设计,电影爱好者,房产,喜欢旅游,热爱生活,策划,环保
b 型血,美食爱好者,服饰,潮人,娱乐,健康,旅游,美食,时尚
美食,旅游
时尚,旅游,军事,文艺,娱乐
我希罕动漫
艺术,爱生活,自由,时尚,旅行,安静,文艺
娱乐,体育
阿熙,爱吃,绘画,陈奕迅,文艺
晋中生活,视频音乐,美食,旅游,投资理财,时尚,健康
星座命理,旅游,健康,新闻资讯,美女
情感生活,美图摄影,读书分享,美食,搞笑幽默,super,superjuniorELF
投资理财,旅游,美食,IT 数码,汽车
娱乐
体育,汽车
健康,娱乐,文艺,时尚,美食
营销,电子商务,家居服,睡衣,旅游
军事
娱乐,文艺
旅游,时尚,星座命理,美食,娱乐
新闻资讯,美食,美女,体育,娱乐
游戏动漫,投资理财,文艺
教育就业,星座命理,健康,娱乐,文艺,美食
娱乐,IT 数码
健康,旅游
搞笑幽默,游戏动漫
体育,军事,旅游,投资理财,新闻资讯
文艺,体育,娱乐
美女,健康,美食
TVB 剧,90 后宅女,美容护肤,香港代购,靠谱,时尚
文艺,游戏动漫,旅游,星座命理,美食,娱乐
旅游,娱乐
重庆生活,娱乐
娱乐
新闻资讯,时尚,军事
美女,新闻资讯,投资理财,体育,汽车
旅游,娱乐,游戏动漫

健康,体育
文艺
IT 数码
娱乐,美食,投资理财
健康,投资理财,IT 数码
旅游,娱乐,新闻资讯,教育就业,时尚,文艺
穿衣美容,时尚,文艺,健康
名人明星
教育就业,投资理财,文艺,美食,娱乐
娱乐
娱乐,美食,旅游
美女,旅游,美食,时尚,文艺
搞笑幽默,游戏动漫,射手女,90 后但我不脑残,学生一族,爱美食,大学,耽美,吃货,宅女
淘宝掌柜,零食控,海洋大学,青岛大学,吃货,青岛嫚儿,青岛人,零食,美食
文艺,健康,旅游,时尚
健康,投资理财
新闻趣事
旅游,健康,美女,美食,娱乐
时尚
娱乐,IT 数码,体育
美女,体育
美食
护肤,美容,化妆,开心,淘宝控,喜欢音乐,懒人,美女,时尚
旅游,时尚,美食
美食
星座命理,军事,文艺
旅游,健康,时尚,汽车,美食
新闻趣事
互联网创业,互联网营销,互联网公司,互联网资讯,互联网评论,电子商务,公益,创业,热爱生活,网络营销
美女,旅游
美食,新闻资讯,健康,投资理财
新闻资讯,美食,军事
投资理财,健康,旅游,娱乐
搞笑幽默,美食,新闻趣事,广州生活,新闻资讯
美食,美食
新闻资讯,美食,IT 数码,娱乐
健康,旅游,美食,时尚,文艺
体育
搞笑幽默,微博奇葩,游戏动漫
名人明星
娱乐
健康,时尚,旅游,娱乐
军事,汽车

美女
旅游
游戏动漫,美食,美女,旅游,时尚,汽车
旅游
美食,旅游,健康
萌宠,微博奇葩,健康,旅游,星座命理,美食
孤枕,唱片,嗨,爱人
翻译,笑话,情感,旅游
新闻资讯
健康,投资理财
时尚,娱乐
唱K,爱音乐,狮子座,唱歌
体育,汽车,军事,IT数码,娱乐
胡思乱想,做梦,高中生,偏执狂,疯子,健康
健康,旅游,娱乐,星座命理
视频音乐,娱乐
文字控,热爱生活,健康,购物,美食,服饰,浪漫,文艺,淘宝控,时尚
星座命理
文艺
教育就业,文艺,IT数码,娱乐
娱乐
投资理财
军事,旅游,美食,文艺,娱乐
IT数码,时尚,健康,文艺,美食,美女,旅游
美食
星座命理,新闻趣事,名人明星,旅游,搞笑幽默
爱睡觉,书,music
新闻资讯,时尚,汽车,投资理财,军事
星座命理,健康,美女,投资理财,教育就业
文艺,IT数码,美食,时尚,娱乐
公益,梦想,体育,新闻资讯,健康,军事,汽车
旅游
星座命理,娱乐,汽车,美女
新闻资讯
美食
教育就业,IT数码,军事,健康,美女
美食
懒,开朗,海贼王,旅游,娱乐,游戏动漫,军事,IT数码,文艺,体育
视频音乐
90后,名人明星,疯子
文艺,时尚,旅游,星座命理,娱乐
搞笑幽默,游戏动漫,美女,娱乐,军事,体育
时尚,美食,星座命理,娱乐
文艺

学生一族,团购,学习,旅游美食,投资理财
军事
广州生活,时尚,娱乐
体育
健康,旅游,美食
文艺
时尚,IT 数码
教育
文艺
美女
文艺,健康,时尚,美食
体育
相信爱情,偏执的天秤,强迫症,完美主义,诗词,文艺
名人明星,健康
投资理财,星座命理,新闻资讯,旅游,IT 数码,军事,汽车
美食,美女,游戏动漫,体育,旅游
时尚,旅游,星座命理,美食,娱乐
汽车,旅游
幽默,无厘头,留学,天秤座,潮,热爱生活,90 後,阳光,撸啊撸,NBA
美食,星座命理,健康,旅游,文艺
文艺,美食,星座命理,美女,时尚
重庆生活,体育资讯,搞笑幽默,美女
娱乐,星座命理,美食,旅游
健康,美食,旅游,教育就业,投资理财
fighting,射手座,鹿晗,吴亦凡,exom
河南生活,教育就业
奋斗 ing
健康,旅游,文艺,体育,新闻资讯
旅遊,天平座,自由,青春,文艺,听音乐
新闻资讯,时尚,娱乐,旅游
健康,文艺
茂名生活
投资理财
娱乐,美食,美女,旅游,游戏动漫
文艺,健康,时尚,美食,娱乐
文艺,时尚,美食,星座命理,游戏动漫,娱乐
文艺,汽车,拍照,可爱,CC 绵小兮
军事
星座命理,美食,健康,旅游,汽车
JAZZ,Skateboard,poppin,breaking,Rap,hiphop,娱乐
体育,教育就业,汽车,时尚,美食
微博奇葩,新闻资讯,投资理财,文艺
汽车,娱乐,健康
旅游

文艺,时尚,娱乐,旅游,星座命理
时尚,健康
新闻资讯,投资理财,教育就业,旅游,文艺
娱乐,文艺,美食,星座命理,新闻资讯
文艺,健康,IT 数码
体育
时尚,军事,美食,旅游,美女
文艺
旅游,健康,时尚,娱乐
旅游,IT 数码,军事,体育
旅游
微博奇葩,娱乐
娱乐,星座命理
文艺,旅游,美食
看电视,美剧控,喜欢旅游,宅女,娱乐,旅游,美食,时尚,IT 数码
美食
美女
投资理财,星座命理,军事
美食,文艺
美女,旅游,IT 数码,体育,娱乐
新闻资讯,IT 数码,教育就业,健康
汽车,文艺,旅游,军事,IT 数码,娱乐
旅游,娱乐
小清新,偶喜欢玩,吃货,搞笑,吃,爬山,真诚,文艺,娱乐,旅游
投资理财,健康,旅游,星座命理,娱乐
灶具,烟机,热水器,厨卫论坛
健康,美食,旅游,投资理财,时尚,汽车,教育就业
旅游
美食,星座命理
娱乐,IT 数码,时尚,游戏动漫,美女
娱乐,旅游
时尚,健康,旅游,文艺,娱乐
汽车
新闻趣事,淡定，睡觉，学生
美女
娱乐,汽车,美女,美食,IT 数码
新闻资讯,健康,文艺,娱乐,时尚
美食,旅游,星座命理,健康,时尚,美女
旅游,体育
游戏动漫,娱乐,文艺,美食,美女
游戏动漫,娱乐,IT 数码
美食,韩剧,电影控,互粉,单身,美食控,购物狂,90 后非脑残,TVB,唱 k
穿衣美容,视频音乐,娱乐,文艺
汽车,游戏动漫,时尚

美食
体育,汽车,军事,游戏动漫,文艺
新闻资讯,健康
文艺,娱乐,游戏动漫,美食,星座命理
新闻资讯,健康,时尚
健康,美食
时尚
美食,旅游,健康,时尚,娱乐
健康,星座命理,时尚,娱乐,美食
IT 数码,军事,汽车,旅游
军事,娱乐,游戏动漫,投资理财,旅游
投资理财,娱乐,旅游,健康
搞笑幽默,汽车
IT 数码
旅游,健康
游戏动漫,汽车,IT 数码
美女,娱乐,游戏动漫,美食,星座命理
游戏动漫,投资理财,IT 数码
萌宠
投资理财,健康,旅游,文艺
健康,旅游,娱乐,美食
健康,军事,体育
搞怪,星座控,爱自由,可爱,爱睡觉,开朗,广州,爱美食,爱玩,文艺
新闻资讯,汽车,IT 数码,文艺,娱乐,时尚,旅游,教育就业
游戏动漫,旅游,美食,IT 数码,娱乐
新闻资讯,军事
名人明星,军事
汽车,新闻资讯,文艺,健康,旅游,星座命理,体育
游戏动漫
文艺,健康,时尚,旅游,美食,娱乐
影视拍摄,网页设计,脚本撰写,平面设计,电影特效,影视后期
穿衣美容
视频音乐,快乐大本营,明星,旅游,星座命理,美食,娱乐
视频音乐,微博奇葩,海南生活,美女,搞笑幽默
逛街,淘宝,化妆,个性,美容护肤,瘦身,品牌,活泼开朗,美女,时尚
体育
教育就业,健康,旅游,美食,体育,娱乐
旅游
游戏动漫
美女,IT 数码,健康,汽车,体育
新闻资讯,美女,游戏动漫
搞笑幽默,娱乐,美食,文艺
文艺
美食,IT 数码

星座命理,健康,时尚
新闻资讯,美女,游戏动漫
体育,教育就业
萌宠
美食
穿衣美容,萌宠,健康,教育就业,旅游,星座命理,美食
历史,品牌,市场营销,低调,爱旅游,时尚,新闻资讯,汽车,军事,IT 数码
娱乐
娱乐
美食,健康
娱乐
体育,美女,健康,军事,娱乐,汽车
天蝎座,邓超
名人明星
IT 互联网,电子,旅游
新闻资讯,美女,游戏动漫
音乐爱好者,上网,唱歌,逛街,看电影,发呆,化妆品,多愁善感,双鱼,K 歌
文艺,娱乐
美女,游戏动漫
文艺,教育就业,投资理财,新闻资讯,娱乐
星座命理
文艺,军事,旅游,游戏动漫,娱乐
美容服饰
玩儿,执着,漫画,TVB,美食,善良,网购,游戏
星座命理,游戏动漫
IT 数码
听音乐,爱音乐,海贼王
投资理财,旅游,星座命理
视频音乐,八卦杂谈
游戏动漫,体育,娱乐
星座命理,IT 数码,美女,健康,教育就业
娱乐,文艺,旅游,健康,体育
做生活中的智者
旅游
美食,军事,文艺,体育,娱乐
文艺,大学生,善良,睡觉,看书,喜欢听歌,美食爱好者,淡定,旅游摄影,成都
时尚,旅游
文艺,时尚
旅游
娱乐,游戏动漫
媒体人,新闻资讯
娱乐,美食
互粉,星座命理,旅游,娱乐,时尚,美食
娱乐

美食
文学,爱情,美女,时尚,健康,星座命理,娱乐
健身,团购,医学,执着,冷笑话,新闻资讯
游戏动漫,旅游
体育
美食
巨蟹座,喜欢听歌,健康,新闻资讯,投资理财,旅游
娱乐
学生一族,名人明星,浙江生活,新闻趣事,搞笑幽默,健康,新闻资讯,星座命理,娱乐
旅游,军事,投资理财,健康,星座命理,教育就业
教育就业,汽车,娱乐
星座命理,美食,文艺,茶文化爱好者,旅游,健康
游戏动漫,教育就业,投资理财
旅游,娱乐
双鱼座,爱生活,创意,五月天,爱美食,电影迷,时尚
新闻资讯,投资理财,旅游,星座命理
游戏动漫,IT 数码
新闻趣事
娱乐,张国荣,90 后,荣迷,哥哥
体育,旅游,文艺,美女
美食,星座命理,健康,新闻资讯,教育就业
八卦杂谈
名人明星,IT 数码
美女,投资理财,体育,教育就业
游戏动漫,星座命理,娱乐
体育,军事
文艺,体育
娱乐,体育
爬山,潮流,瑜伽,美容,化妆,娱乐,时尚,美女,健康
星座命理
文艺,美女,IT 数码
萌宠
时尚
游戏动漫,美食,教育就业,汽车,投资理财
LOL,大学生,足球迷,美食爱好者,AC 米兰,健康
新闻资讯,教育就业,汽车,健康,体育
投资理财
韩剧
旅游,游戏动漫,娱乐
汽车,时尚,健康,IT 数码,娱乐
旅游
娱乐,文艺,星座命理,体育
娱乐,美食
星座命理,游戏动漫

阜阳生活,文艺,佛门书画,千年古刹,实证实修,皇家寺院
天蝎座的90后,电影控,泰勒斯威夫特,哈利波特,生活大爆炸,学生一枚,欧美
melochin一生推,不吐槽會死星人,精神分裂症重患,树哥厨,动漫
美食,娱乐
星座命理,体育
时尚,新闻资讯,美食,文艺,娱乐
车世界,汽车
搞笑幽默,美食,幽默,爱睡觉,阳光,创意,爱生活,浪漫,吃货一枚,星座命理
美食,时尚,文艺,娱乐,星座命理
微博奇葩,搞笑幽默,文艺,娱乐,旅游,美食
哈尔滨生活,设计,孩子气,宅女一枚,快乐大本营
美食
时尚,旅游
时尚
新闻资讯,IT数码,时尚,文艺,娱乐
时尚
时尚
娱乐
文艺,游戏动漫
IT数码,文艺,体育
美女,IT数码,体育,娱乐,美食
游戏动漫,美食,星座命理,娱乐
IT数码
汽车,IT数码,投资理财,时尚,文艺,旅游
新闻资讯,健康
IT数码,游戏动漫
文艺,时尚
美女,美食,健康,时尚,文艺
娱乐
娱乐
体育
咖啡控,汽车,苹果控,乐天派,傻,强迫症,喜欢音乐,淘宝控,爱旅行,美食
新闻资讯
其他生活
旅遊,美食,時尚
旅游,入门级背包客,背过包,去过雪山,搭过车,徒过步,骑行环过湖
游戏动漫,投资理财,IT数码
娱乐
教育就业
娱乐
美女,游戏动漫
读书分享,美食,新闻资讯,文艺,游戏动漫
新闻资讯,汽车,军事
美食

美食
美女
时尚,旅游
体育
石家庄生活,搞笑幽默,新闻趣事,健康,娱乐,星座命理
深圳生活,旅游,穿衣美容,微博奇葩,搞笑幽默,娱乐
汽车,时尚,美食,IT 数码,文艺
看小说,爱美食,旅游摄影,热情,火影忍者,开朗,旅游,健康,游戏动漫,IT 数码
星座命理
体育,娱乐,教育就业,投资理财
旅游,健康,IT 数码,军事,美食
穿衣美容,娱乐
娱乐
宅女,音乐
旅游,时尚,星座命理,健康,娱乐
旅游,健康,美食,美女,娱乐
时尚,体育,文艺,美食
90 后但我不脑残,军事天地,90 後,IT 數碼,电影控,足球迷,音乐控,奋斗,汽车,房产
新闻资讯,健康,旅游,星座命理,娱乐
美食,星座命理,穿衣美容,旅游,文艺
娱乐,游戏动漫,新闻资讯,健康,汽车
投资理财,旅游,体育,美食,汽车
睡觉，听歌
搞笑幽默,新闻资讯,时尚,娱乐,投资理财
汽车,娱乐,文艺,体育,游戏动漫
旅游,美食,投资理财,军事,体育
名人明星,娱乐
文艺,娱乐,时尚,健康,美食
文艺
新闻资讯
体育
健康,旅游,美食,文艺
美食,娱乐,文艺
上海生活,搞笑幽默,视频音乐,新闻趣事,军事
梅里雪山,香格里拉,丽江旅游,互粉,旅游
IT 数码
时尚,旅游,星座命理,美食,文艺
美食,广东生活,体育
娱乐,教育就业,旅游,时尚,健康
美食,时尚,娱乐,健康,星座命理
健康
投资理财,游戏动漫,健康,IT 数码,娱乐
志愿者,公务员,90,浪漫,翻译,自然,投资理财,美食,旅游,教育就业
娱乐,投资理财,教育就业

游戏动漫,健康,旅游,星座命理,美食
时尚
文艺,旅游,游戏动漫,时尚,美女
教育就业,汽车,旅游,美食,IT 数码
文艺,爬山,读书,跑步,发呆
时尚,旅游,星座命理,美食,娱乐
体育
双鱼,电视剧控,小说迷,看书,文艺,美食,旅游
时尚,健康,旅游,文艺
游戏动漫,星座命理,美女,文艺,娱乐
投资理财
视频音乐
美容服饰
娱乐
美女,投资理财,娱乐
美女
星座命理,娱乐,美女,游戏动漫,时尚,汽车
搞笑幽默,教育就业,新闻资讯
微博奇葩
旅游,穿衣美容,星座命理,微博奇葩,搞笑幽默
健康,游戏动漫,美食,文艺,娱乐
苗圃,王洋,刘婧,90 后,A 型血,90 后非脑残,巨蟹,学生,思考
汽车,游戏动漫
新闻资讯,音乐迷
文艺,娱乐
时尚,投资理财,旅游
新闻资讯,时尚,投资理财,旅游,体育
懒人,吃货一枚,旅游,美食,双子座,爱自由,动漫
汽车,投资理财,旅游,星座命理,娱乐
健康,新闻资讯,时尚,投资理财
吉他爱好者,摄影,微博控,美女,汽车
美女
正能量,插画师,游戏开发,插画,设计师,设计,动画,文藝,娛樂,旅遊
美女,娱乐,星座命理,文艺,健康,时尚
美食,穿衣美容,星座命理,微博奇葩,健康,旅游
娱乐,体育
唱歌,星座控,爱玩,漫画,爱美食,创意,爱生活,爱睡觉,吃货,看书
投资理财
娱乐
健康,美女,汽车,娱乐,时尚,美食
IT 数码,投资理财,教育就业,新闻资讯,星座命理
军事,文艺,娱乐,投资理财,体育
娱乐
旅游,健康,新闻资讯,教育就业,汽车,投资理财

搞笑幽默
美女,体育
体育,文艺
游戏动漫,文艺
健康
旅游,时尚,美食
健康,时尚,娱乐,旅游,美食
娱乐
旅游,微博奇葩,视频音乐,新闻趣事,搞笑幽默,IT 数码
文艺,健康,美食,时尚,娱乐
娱乐
旅游,体育
你能成大事,幽默,运动,周杰伦,80 后,搞笑,娱乐
美食
体育
健康,星座命理,娱乐
文艺,时尚,美食
美女,时尚
健康,投资理财
娱乐
美女
旅游,星座命理,文艺
猫咪
美女,投资理财
文艺,娱乐,体育,教育就业,时尚,健康
体育,投资理财,教育就业,军事
游戏动漫,娱乐
旅游,IT 数码
花盆,雕塑,茶玩,紫砂花盆,茶宠,紫砂,茶壶,摆件,宜兴紫砂壶,紫砂壶
美食,星座命理,娱乐
汽车
汽车,投资理财,美女,IT 数码,娱乐
美食
名人明星,微博奇葩,时尚,文艺,娱乐
名人明星,情感生活,美食,校园生活,长沙生活,娱乐,时尚
旅游
汽车
健康,时尚,星座命理,美食,娱乐
娱乐
娱乐
汽车,教育就业,IT 数码
新闻资讯
教育就业,美食,旅游,健康,时尚
文艺,教育就业,健康,美食,娱乐

娱乐
文艺
体育,文艺
汽车,IT 数码,体育,娱乐,美女
游戏动漫
处女座,狮子座,大学生
游戏动漫
美食
穿衣美容,视频音乐,新闻趣事,搞笑幽默,健康,娱乐
健康,美食,旅游,体育
体育
娱乐
健康,旅游
视频音乐,IT 数码
美食,娱乐
日照婚礼策划,日照婚庆,戴娜高端婚礼,日照婚礼,婚礼策划师,婚礼定制,DIY 婚礼
旅行,摄影,八卦,教育就业,美食,娱乐
旅游
向日葵,媒体,欧美控,王力宏,服装,喜欢听歌,热爱生活,健康,文艺,娱乐
校园生活,搞笑幽默,娱乐
投资理财
美女,时尚,IT 数码,文艺,娱乐
郁可唯,书,健康,娱乐,旅游,美食,文艺
文艺
健康,教育就业
美女,QQ 群 321104837,店内买家 QQ 群,代购,吃货,淘宝店主,爱购物,自然,坚强,时尚
时尚
动漫控,钢琴,话剧,恐怖片,海绵宝宝,写字,苏打绿,旅游,IT 数码,文艺
教育就业,美女,美食,娱乐,体育
阅读,动物,旅行,电影,摩羯座
时尚,娱乐
游戏动漫,旅游,美食,文艺,娱乐
许嵩,旅游爱好者
新闻资讯,教育就业,投资理财,IT 数码,娱乐
汽车
教育就业
旅游
健康,时尚,教育就业,投资理财,娱乐
文艺,娱乐,新闻资讯,投资理财,教育就业
美食,文艺,投资理财,旅游,游戏动漫
健康,新闻资讯,娱乐
投资理财,新闻资讯
投资理财,娱乐,汽车
穿衣美容,娱乐

新闻资讯,IT 数码,军事
美女,时尚,美食,娱乐
文艺,时尚,游戏动漫,旅游,美食
健康,体育,时尚
美食,娱乐
娱乐
名人明星,千年老妖,生化危机,系统控,卡饭,巨蟹
美食,微博奇葩,河南生活,娱乐
美食
微博奇葩,文艺,游戏动漫
搞笑幽默,旅游,美食,穿衣美容,星座命理,娱乐
美食,旅游,星座命理,娱乐,健康
娱乐,文艺
旅游,小资,拍照,巨蟹,写字,文艺
名人明星,娱乐
旅游
游戏动漫,星座命理,美食,娱乐,体育
时尚,娱乐,IT 数码
军事,IT 数码,体育,投资理财,游戏动漫
IT 数码,旅游,体育
体育
名人明星,时尚,文艺,娱乐
新闻趣事
随性,桌球控,喜欢听歌,淡定,热爱生活,爱猫,旅游,文艺
新闻资讯,美食,旅游,健康,美女,体育,娱乐
游戏动漫,教育就业,汽车,时尚,美女,IT 数码
90,恐怖片,宅女一枚,云南生活,学生一枚,军事,熊猫,双鱼女,星座命理
微博奇葩,浙江生活,搞笑幽默,美食,IT 数码
美女,娱乐,教育就业,体育
旅游,健康,美食
健康,旅游,新闻资讯,时尚,投资理财
体育
旅游
文艺,健康,时尚,旅游,美食
时尚,健康,美食,教育就业,娱乐,旅游
娱乐
娱乐,旅游,美食,游戏动漫
汽车
穿衣美容,名人明星,重庆生活,搞笑幽默,娱乐,时尚,新闻资讯,健康,旅游
忧郁,互粉,懒,安静,爱玩,幻想,冷笑话,胡思乱想,星座命理,文艺
美食
时尚
穿衣美容,时尚,健康,文艺
IT 数码,文艺,娱乐

投资理财,旅游,IT 数码
健康,新闻资讯,娱乐
美女,美食,旅游,汽车,时尚
旅游,美食
西安生活,文艺,教育就业
时尚,健康,旅游,美女
游戏动漫,文艺,健康,星座命理,娱乐
健康,体育,娱乐
军事,文艺
娱乐,美食
娱乐,体育,旅游,投资理财,汽车
旅游,汽车,军事
星座命理,体育
时尚,美女,美食,星座命理,娱乐
美食
教育就业,健康,娱乐,星座命理,美食
星座命理,娱乐,美食
杭州生活,新闻趣事,搞笑幽默,健康,IT 数码,投资理财,汽车
美食
星座命理
教育就业,投资理财,新闻资讯
美食,娱乐
美食,娱乐,游戏动漫,美女,IT 数码
游戏动漫,文艺,IT 数码,新闻资讯,娱乐,时尚
文艺
星座命理
文艺,旅游,美食
旅行,摄影,八卦,宅,购物,时尚,投资理财,旅游
娱乐,新闻资讯,体育
美食,健康,娱乐,时尚,星座命理
文学,情感,音乐达人,书籍,旅游爱好者,瑜伽,温柔,法语,健康,文艺
汽车,美女,军事
旅游
体育,娱乐,星座命理,教育就业,IT 数码
济南生活,时尚,新闻资讯,IT 数码
健康,美食
旅游,汽车,体育,美女,投资理财,IT 数码
汽车,IT 数码,美女
娱乐,旅游,美食,投资理财
时尚
教育就业,新闻资讯
体育,娱乐,教育就业
娱乐,IT 数码
IT 数码,游戏动漫,时尚,投资理财,娱乐,文艺

niconico 動画,使命召唤,bilibili,技术宅,百合控,软妹控,显卡吧,芙兰朵露,动漫控,东方Project
搞笑幽默,IT 数码
时尚,文艺
健康
勤工俭学,交友谈心,大学生活,人生励志,教育培训
健康
北京生活,游戏动漫
淘宝控,美食,投资理财
文艺
搞笑幽默,娱乐
搞笑幽默,投资理财,汽车,IT 数码
文艺
温州生活,新闻资讯
美食,星座命理,娱乐,时尚
美女,IT 数码
旅游,文艺,美食
娱乐
旅游,体育
体育资讯,搞笑幽默,美女,体育
穿衣美容,睡觉,摄影,动漫
投资理财,时尚,星座命理
美食
星座命理,体育
娱乐
体育,旅游,投资理财,美食,文艺
投资理财
美女
美女,时尚,文艺,美食,游戏动漫,星座命理,娱乐
时尚
旅游,军事,美女,美食,新闻资讯
IT 数码,教育就业,投资理财,娱乐,文艺,美食,军事
体育,美女,美食,旅游,娱乐,睡觉,游戏
文艺,娱乐
IT 数码,娱乐
美食
教育就业,投资理财,娱乐,旅游
美食
美食,萌宠
体育
时尚,玩,音乐爱好者
时尚,娱乐,军事
感性,学习,喜欢音乐,爱,健康,旅游,教育就业,新闻资讯
旅游,新闻资讯,文艺,娱乐,时尚

读书分享,情感生活,新闻趣事,搞笑幽默,新闻资讯,文艺,星座命理,旅游,娱乐
大学生,爱幻想,看小说,动漫迷,美食爱好者,宅女加腐女,水瓶女,bl,我就是我,懒人
穿衣美容,视频音乐,美女,美食,星座命理,室内设计,双鱼座,汽车
上海生活,水瓶座,学生一族,懒,安静
投资理财
教育就业,时尚,健康,旅游,美食
旅游,美食
娱乐
星座命理
军事
新闻资讯
时尚,体育
视频音乐,搞笑幽默,美食,文艺,体育,娱乐
教育就业,投资理财,新闻资讯,健康,娱乐
美食,IT 数码,教育就业,旅游,美女,投资理财,文艺,新闻资讯,军事,体育
新闻资讯,星座命理,体育
美食,文艺
美容服饰,护肤,美妆护肤,日本药妆,潮流
向日葵,看电影,爱音乐,音乐达人
美食
打游戏
旅游
时尚,文艺,教育就业,投资理财,娱乐
娱乐
时尚,IT 数码
新闻资讯
内蒙古生活,管理,自由,建筑,阅读,健康,旅游
教育就业,新闻资讯,健康,星座命理
体育资讯,体育
新闻资讯,IT 数码,美食
旅游,IT 数码,娱乐,汽车,健康
娛樂,文藝,美食
美女,游戏动漫,星座命理,娱乐,旅游,美食,体育
体育
IT 数码,时尚
吉林生活,名人明星,星座运势,美图摄影,美容服饰
旅游,美食,娱乐
汽车
娱乐,旅游,美食,时尚,星座命理
搞笑幽默,汽车
汽车
新闻资讯
健康,新闻资讯,体育,IT 数码,星座命理
旅游,汽车,体育

旅游
搞笑幽默,娱乐
美女,美食
校园生活,成都生活
体育,文艺,新闻资讯,娱乐
星座命理,旅游,时尚,文艺,娱乐
美女,汽车,星座命理
新闻趣事,健康,时尚,教育就业,文艺,娱乐
新闻资讯,旅游
旅游,军事,新闻资讯,美女,投资理财
新闻趣事,真诚
游戏动漫,美食,文艺,星座命理
文艺,美食
军事,IT 数码,旅游,汽车,娱乐
名人明星
投资理财
教育就业,时尚,文艺,新闻资讯,娱乐
新闻趣事,游戏动漫,汽车,军事,体育
时尚,娱乐,旅游
婚纱摄影,婚礼,婚礼策划师,婚庆,婚纱
旅游,文艺,娱乐
校园生活,连接服务器失败,吃货,90 後,学生一族,交朋友,宅
汽车,美食,旅游,投资理财,美食,星座命理,体育
旅游,美食,娱乐
星座命理,美食,时尚,娱乐
新闻趣事,美食,旅游,南宁生活
美食,时尚,娱乐,好性格,睡懒觉,范二
微博奇葩
文艺,旅游,新闻资讯,健康,军事
星座命理,IT 数码,娱乐,时尚
新闻资讯,文艺,IT 数码,体育,娱乐
游戏动漫,教育就业,文艺,体育
旅游
军事
旅游,星座命理,美食,文艺,娱乐
游戏动漫
其他生活,旅游,活泼,快乐
教育就业,汽车,游戏动漫,IT 数码,军事
娱乐,美食,旅游,健康,时尚
美女
新闻资讯
IT 数码
旅游
娱乐

游戏动漫
爱音乐,90後,旅遊,朋友,开心,上海生活,体育
美女,美食,健康,旅游
教育就业,旅游,军事,美食,娱乐
星座命理,IT 数码,娱乐
IT 数码,美女
美女,时尚
新闻趣事,公益志愿者,教育就业,文艺,新闻资讯,体育
娱乐
其他生活,搞笑幽默
名人明星,星座命理,视频音乐,微博奇葩,穿衣美容
健康,新闻资讯,旅游,星座命理,美食
娱乐,健康,时尚,投资理财
至上励合,星座,政治,90後,军事
文艺
互粉,新闻资讯,工作狂,中医,养生,社会,文艺,时尚,美食,健康
旅游,军事
美食,旅游,星座命理,美女
新闻资讯
体育,时尚,新闻资讯,娱乐,文艺
健康,新闻资讯,体育
旅游
我就是我,旅游,时尚,娱乐,新闻资讯,健康
时尚,娱乐,游戏动漫,星座命理
娱乐,游戏动漫,文艺
游戏动漫,星座命理
旅游,射手,无厘头,双重性格,臭美,无聊,喜欢音乐,愤青,80 后的尾巴
搞笑幽默
时尚,文艺,旅游,星座命理,娱乐
美女,娱乐,旅游
逛街,美食
美女
娱乐,时尚
上海生活,娱乐
文艺,星座命理,美食
娛樂
星座命理
健康,时尚,美女,美食,旅游
旅游,美食,萌宠,微博奇葩,四川生活,文艺,体育
娱乐,文艺,美女,美食,旅游
花样美男
名人明星,美女,微博奇葩,星座命理,搞笑幽默
美食
视频音乐,微博奇葩,时尚,旅游

旅游
视频音乐,新闻趣事,美图摄影,健康,投资理财,时尚,旅游,美食
自由,阿信,五月天,宅女,小说,看书,美食,旅游
美女
搞笑幽默,体育
投资理财
游戏动漫,美女
北京生活,美食
星座命理,体育,美食
文艺,IT 数码,汽车,体育
IT 数码
教育就业,时尚,新闻资讯,旅游,星座命理
体育,健康,新闻资讯,IT 数码,汽车
娱乐,美食
娱乐
新闻资讯,健康,旅游,星座命理,文艺
游戏动漫
美女,军事
娱乐,美食,健康,时尚,文艺
文艺,时尚,美食
成都生活
投资理财,文艺,星座命理,旅游,健康
新闻趣事,旅游,美食,星座命理,名人明星,文艺,娱乐
美食,品牌,90,时尚
投资理财,娱乐,体育,军事,星座命理
猫咪控,宅女,美男控
娱乐,时尚
乡村爱情
体育
美食,旅游,娱乐,时尚,体育
投资理财,体育
美食,娱乐,教育就业,投资理财,健康
新闻资讯,文艺,时尚
搞笑幽默
星座命理,美女,新闻资讯
美女,军事
美女,教育就业,时尚,文艺
健康,新闻资讯,美食,IT 数码,文艺
健康,旅游,星座命理,美食,投资理财
新闻资讯
时尚,汽车
时尚,娱乐
旅游,美女,美食,体育
美女,文艺

文艺,健康,美食
教育就业
爱笑,文艺,旅行
美女,山东生活,微博奇葩
时尚,健康,星座命理,美食,娱乐
教育就业,游戏动漫,文艺,健康,IT 数码,美食,军事,娱乐,体育
娱乐,旅游
新闻资讯,旅游,美食,娱乐,唱 k,吃货一枚,学生一族,乐天派,纠结
教育就业,健康,微电影,志愿者,助人自助,公益活动,大学生,社会工作者
时尚,旅游,美食,文艺
健康,文艺
美食,美女,时尚,汽车,娱乐
旅游,听歌,听雨,听海,单车旅行
美女,游戏动漫,星座命理,旅游,IT 数码
读书分享,视频音乐,新闻资讯,美食
军事
新闻趣事,穿衣美容,美食
旅游,美食,穿衣美容,游戏动漫
游戏动漫
搞笑幽默
四川生活,穿衣美容,时尚,星座命理,旅游,美食,健康
新闻资讯
IT 数码
苏州生活,健康,新闻资讯
维权,求助
懒人,90 後,电影迷,看电视,喜欢旅游,动漫控,旅游美食,胡思乱想,自信,学生一族
旅游,80 後,无肉不欢,全科医师,宣武医院,Madonna,魔力红,中国女排,网球,小威廉姆斯
新闻趣事,体育
旅游
美食,投资理财,娱乐,美女,IT 数码
艺术,网购,喜欢旅游,爱电影,爱旅游,创意,电视剧,美食爱好者,大学生,文艺
自信,文化,原创,睡觉睡到自然醒,星星
旅游,教育就业
娱乐
美容服饰,娱乐
时尚
健康,旅游,星座命理,娱乐,美食
教育就业,文艺,美食,旅游,娱乐,健康,时尚
教育就业
旅游,体育
美女,摩羯,大学生,努力,古典音乐,书法,90 後,低调,诗歌,看书
娱乐,军事,健康
新闻资讯,时尚,文艺
微博奇葩,山东生活,美食,视频音乐,旅游

娱乐,美食,投资理财,时尚,健康,快乐,喜欢唱歌,90
旅游,时尚,娱乐,健康,美食
游戏动漫
时尚,文艺,健康,旅游
文艺
旅游,文艺
旅游,视频音乐,bass,jazz,网购,育儿,健康
美女,文艺
娱乐
无锡生活,军事,美食,体育
旅游,游戏动漫,娱乐,时尚,星座命理
美女,游戏动漫,投资理财,IT 数码,娱乐
汽车,时尚,美食
美食
旅游
娱乐,美食,健康,时尚,投资理财
游戏动漫,时尚,健康,星座命理,文艺
健康,汽车,旅游,文艺,娱乐
文艺,健康,新闻资讯,投资理财,旅游
微博奇葩,新闻趣事,穿衣美容,旅游,健康,新闻资讯,时尚,投资理财,文艺
军事
娱乐
星座命理,旅游,时尚,美食,IT 数码,娱乐
娱乐,IT 数码,星座命理,时尚,文艺,旅游
旅游,美食,健康
古风控,盗墓笔记,言情小说,刘诗诗,双鱼座,十八只,superjunior,东方神起
IT 数码
蛋糕达人,北京,生日蛋糕,蛋糕店,芝士蛋糕,甜食,甜品,美食
新闻趣事,旅游,美食,穿衣美容,搞笑幽默
娱乐,星座命理,游戏动漫,军事,体育
美食,新闻资讯
美食
健康,新闻资讯,体育
怀旧,钟汉良,美食,旅游
旅游,健康,文艺,军事,汽车,时尚,游戏动漫,美女
时尚
娱乐
搞笑幽默,新闻趣事,体育,汽车
健康,旅游,时尚
娱乐,体育
历史向二次元,文艺学术宅,中高考培训
美食,美女,娱乐,文艺,时尚
美食,投资理财,健康,旅游
军事,美女,汽车,旅游,娱乐

80 后宅女,星座命理,投资理财,时尚,美女,健康
娱乐
旅游,健康,美女,时尚,美食
高三,随性,乐天派,蓝色,好性格,动漫,游戏动漫
星座命理
喜欢听歌,囧,健康,汽车,美女,教育就业,IT 数码
娱乐,教育就业,新闻资讯,健康,星座命理,体育
旅游,游戏动漫,娱乐,时尚
星座命理
汽车,星座命理,旅游,美食,娱乐
旅游,美食,娱乐,汽车
星座命理,搞笑幽默,旅游,时尚,娱乐,美食
美食,旅游,健康,文艺
美女,汽车,旅游,美食,娱乐
娱乐
旅游,三维扫描,DIY,快速成型,IT,个性定制,创意,互联网,3D 打印,创客
旅游,体育
北京生活,福玉祥珠宝北京,福玉祥国际珠宝,福玉祥珠宝学园,一站式珠宝选购,福玉祥珠宝品牌,福玉祥珠宝佛公,福玉祥珠宝观音,福玉祥珠宝翡翠,福玉祥珠宝城
健康
旅游,教育就业,新闻资讯,健康,投资理财
美女,娱乐,教育就业,健康
新闻趣事,搞笑幽默,旅游,美图摄影,八卦杂谈
文艺
旅游,学生一枚,花痴,健康,教育就业
新闻资讯,旅游,文艺,星座命理,美食
吉林生活,体育
IT 数码,文艺,娱乐,教育就业,旅游
文艺,IT 数码,娱乐,健康,旅游
汽车,美女,体育
平常心,单身,发呆,玩,射手座,music,体育,健康
美食,旅游,娱乐,IT 数码,美女
美女
旅游,美食,时尚,文艺
穿衣美容,旅游,美食,搞笑幽默,广东生活
健康,娱乐,军事,美女,旅游
时尚
美女,美食,IT 数码,文艺,娱乐
星座命理,时尚
文艺,新闻资讯
娱乐,旅游,体育,健康
摄影,数码,八卦,宅,购物,教育就业,投资理财,军事
美食,时尚,汽车,文艺,娱乐
旅游,美食

微博奇葩,文艺
时尚,投资理财,文艺,IT 数码,教育就业,新闻资讯,健康
时尚
投资理财,美食,军事,IT 数码,体育
旅游,健康
IT 数码,娱乐
时尚,投资理财,娱乐,美食,健康
名人明星,文艺,体育
星座命理,健康,时尚,投资理财,娱乐
星座命理,美食,军事,娱乐
新闻资讯,时尚,娱乐,健康,投资理财,旅游,美食
IT 数码,健康,投资理财
体育
娱乐,教育就业
美女,星座命理,投资理财,娱乐,IT 数码
旅游
美女,投资理财,汽车
美女,美食,旅游,投资理财,时尚
萌宠,时尚
旅游,时尚
旅游,视频音乐,搞笑幽默,新闻趣事,云南生活
军事,新闻资讯
健康
美女
体育,汽车,新闻资讯,星座命理,IT 数码
旅游,文艺,娱乐
时尚,旅游,美食,IT 数码,文艺
投资理财,汽车,美女,旅游,IT 数码
旅游,情感生活,名人明星,美容服饰,视频音乐
娱乐,体育
游戏动漫,旅游,美食,文艺,娱乐
旅游,健康,娱乐,投资理财,军事,文艺,新闻资讯
体育
美食
新闻资讯,星座命理,军事,游戏动漫,文艺,娱乐
时尚
娱乐,旅游,健康,星座命理
娱乐
新闻资讯,摩羯,美剧,投资理财,军事,文艺
体育,文艺,新闻资讯,教育就业
娱乐
旅游
美女,a 型血,深圳,咖啡,NBA
美食,健康,旅游

娱乐,星座命理,美女,体育
穿衣美容,时尚,投资理财,娱乐
美女,IT 数码,体育
星座命理,袜控,皮带,领带,衬衫,脚控,微 SM,皮鞋控,正装控
揭阳生活,星座命理,游戏动漫,娱乐
文艺
IT 数码
娱乐
Kpop 死忠饭,天秤最高,少女心的女汉纸,极端矛盾爱纠结,爱草莓爱鸡腿,理性大于感性,表演系,影视配音专业,粉色控,HelloKitty 控
新闻资讯,汽车,旅游,健康
美食,游戏动漫
穿衣美容,时尚
IT 数码,体育
80 後,宠物,唱歌,宅,动漫,睡觉,时装,星座命理,娱乐,游戏动漫
健康,教育就业,美食,星座命理,娱乐
美图摄影,搞笑幽默,北京生活,新闻资讯,健康,旅游,投资理财,美食
美女
时尚,健康,旅游,美食,娱乐
汽车,IT 数码
黄冈百事通,草根,黄冈,新闻,草根播报平台,新闻资讯
美女,军事,IT 数码
美食,美女
美食,娱乐
时尚,IT 数码,汽车,文艺,娱乐,投资理财
投资理财,IT 数码
名人明星,娱乐,投资理财,教育就业,军事,IT 数码
旅游
汽车,娱乐,旅游,美食
搞笑幽默,汽车
旅游
旅游
多动症的肉丸,搞笑王
美食,美女,新闻资讯
美食,旅游
娱乐
美女
美食,旅游,新闻资讯,体育
搞笑幽默,新闻资讯
健康,美女,旅游,体育
体育
体育
大型建筑企业,团委,人才培养,志愿者,青年,五四,共青团
游戏动漫

微博奇葩, 文艺, 游戏动漫
军事, 时尚
音樂, 视频, 美女, 旅游, 健康, 文艺, 时尚
IT 数码
旅游, 美食, 教育就业, 投资理财, 文艺
健康, 旅游, 军事, 体育
新闻资讯
水瓶, 心理学, 矛盾, 烹饪, 医学, 爱唱歌, 狗狗, 美食
IT 数码, 游戏动漫, 娱乐
广东生活, 美食
旅游
IT 数码
时尚
时尚, 美女
视频音乐, 校园生活, 新闻趣事, 体育, 教育就业, 健康, 新闻资讯
美女
体育
投资理财, 时尚, 汽车, 教育就业, 健康, 星座命理
旅游
美女, 旅游, 军事
旅游, 投资理财, 教育就业, 美食, 星座命理, 文艺
娱乐
健康
美食
时尚, 汽车, 体育
投资理财, 健康, 旅游, 星座命理, 美食
美女, 文艺, IT 数码, 美食, 游戏动漫
北京生活, 萌宠, 微博奇葩, 新闻趣事, 搞笑幽默
美女
文艺, 军事
文艺
美食, 健康, 文艺, 投资理财, 新闻资讯
微博奇葩, 星座命理, 美女, 新闻趣事, 搞笑幽默
旅游, 汽车
起床困难户, 笔记本电脑, 龙之谷, 汽车, 体育
娱乐
淘宝, 购物狂, 奋斗, 欧美, 80 后宅女, 开朗, 逛街, 游泳, 快乐, 时尚
旅游, 新闻趣事, 穿衣美容
美食, 旅游
搞笑幽默, 视频音乐
IT 数码, 文艺, 体育, 娱乐
文艺
搞笑幽默, 视频音乐, 宠物图集
名人明星, 旅游, 美食, 美女, 穿衣美容, 朴有天, 美剧控, 看电视, 狮子座, 时尚

时尚,投资理财,健康,新闻资讯,美食
新闻资讯,体育,健康
娱乐,体育,游戏动漫,星座命理,IT 数码
健康,时尚,旅游,星座命理,美食
名人明星,信托,基金,艺术品投资,股市,艺术,证券,财经,股票,投资理财
新闻资讯,美食
时尚
文艺
游戏动漫
旅游
健康,美食,星座命理,时尚,娱乐,文艺,IT 数码
旅游
穿衣美容,旅游,健康,新闻资讯,娱乐
旅游,IT 数码,军事,教育就业,新闻资讯
美女,IT 数码,体育
汽车
美女,体育
济南生活,文艺,新闻资讯
教育就业,投资理财,健康,美食,旅游
美女
旅游,文艺,教育就业,游戏动漫,娱乐
时尚
娱乐
美食
理财,大叔控,人生,休闲,投资理财
2012 出生的宝宝,娱乐
穿衣美容,视频音乐,名人明星,萌宠,美女,健康,文艺,娱乐
时尚,星座命理,娱乐,旅游,健康,美食
娱乐,健康,游戏动漫,文艺,体育
交友,时尚
美女,时尚
文艺,旅游
文艺
学习
体育
娱乐
切尔西球迷,夜猫子,情感,火影忍者,汽车,游戏动漫,体育
军事
美图摄影,中考复习,学冠教育,中考高分班,初三数学,中考,初三语文,中考培训
IT 数码
体育,旅游,美女,美食,文艺
投资理财,旅游,健康,教育就业,娱乐
健康
北京生活

旅游,健康,美食,娱乐,时尚
娱乐,旅游,时尚,美食,星座命理
娱乐
时尚,娱乐,文艺,美食
投资理财
时尚,教育就业,新闻资讯,美食,文艺
文艺
名人明星,星座命理,穿衣美容,新闻趣事,搞笑幽默
旅游
音乐爱好者,美剧,文艺,体育,娱乐
娱乐
旅游,军事,文艺,娱乐
文艺,体育,教育就业
汽车,美女,美食,军事,体育
睡觉
欧美,吸血鬼日记,穿衣美容,娱乐
穿衣美容
美女
视频音乐,旅游,新闻资讯,体育,文艺
新闻趣事,搞笑幽默,时尚,新闻资讯,健康,星座命理
美食
体育,娱乐
游戏动漫,美食
时尚,游戏动漫,星座命理,娱乐
萌宠,小蜗控,成就党,WOW,天蝎,游戏动漫,旅游
汽车
影魔,狮子王,乒乓球,心理,写作,咖啡
游戏动漫,美食,IT 数码,健康,教育就业,新闻资讯
IT 数码,旅游,游戏动漫
投资理财,娱乐
汽车
娱乐
时尚,美食
时尚,汽车,IT 数码
体育,游戏动漫
美食,旅游,时尚,文艺
美女,美食,投资理财
名人明星,搞笑幽默,穿衣美容,美食,时尚,文艺,旅游,娱乐
娱乐,文艺
旅游,美食,时尚
美食
军事,新闻资讯,旅游,健康,娱乐
新闻资讯,汽车
爱音乐,星座命理,旅游,教育就业,文艺,军事

新闻趣事,新闻资讯
IT 数码,Gamification,交互设计,积极心理学
美食,IT 数码,文艺,投资理财,娱乐
美女
文艺,旅游
投资理财,新闻资讯,汽车,IT 数码
汽车,游戏动漫,IT 数码,文艺,体育
美食,巨蟹座,执着,感性,听歌,敏感
机械制造业,自动化技术,自动拉丝机,自动抛光机,抛光机,砂带机,新闻资讯
健康,文艺,教育就业,投资理财,旅游
旅游,花样美男,美食,美图摄影,游戏动漫,看电影,听音乐,淘宝
新闻资讯,美女,游戏动漫
北京生活,时尚
新闻资讯,汽车,体育
穿衣美容,微博奇葩,美女,搞笑幽默,游戏动漫,投资理财,体育,IT 数码,娱乐,文艺
美食,星座命理,旅游,健康,时尚
美食
旅游,娱乐,美食
游戏动漫,美女,汽车,时尚
投资理财,IT 数码,旅游,美食
健康,时尚,美食,文艺,娱乐
微博奇葩,旅游
美图摄影,时尚,美食,星座命理,文艺,娱乐
旅游,军事,IT 数码,娱乐,体育
游戏动漫,IT 数码
投资理财
旅游,文艺,IT 数码,新闻资讯,游戏动漫,汽车
汽车,游戏动漫,时尚,文艺,娱乐
游戏动漫,新闻资讯,健康,投资理财,美食
体育
时尚,健康,新闻资讯,教育就业,星座命理
爱玩爱闹,小 P 孩,疯癫
科学探索,车世界,新闻趣事,IT 数码,搞笑幽默,汽车
美食,旅游,健康,娱乐
时尚,健康,旅游,娱乐,文艺
美女,52cpb,台球,Music,睡觉,运动,动漫,电影,鹦鹉
军事
娱乐,文艺
新闻趣事,旅游,美食,努力,无厘头,青春,吉他,阳光,阅读,文艺
快速卷帘门,工业提升门,快速门,门类,电子商务,营销
旅游,美食,游戏动漫,时尚,IT 数码
娱乐
新闻趣事,教育就业
军事,体育,汽车,投资理财,美食,旅游,健康

其他生活,汽车
文艺,娱乐,时尚,体育,游戏动漫
美女,美食,体育
时尚,IT 数码,体育
旅游,美食,军事
投资理财,旅游,娱乐
健康,星座命理,军事,IT 数码,娱乐
军事,IT 数码,文艺,体育,娱乐
美女,时尚
游戏动漫,军事,体育
健康,新闻资讯,IT 数码
时尚,汽车,旅游,文艺,娱乐
爱好：睡觉。
美食
时尚
旅游
教育就业,旅游,美食,IT 数码,娱乐,美食
娱乐
旅游,游戏动漫,文艺
美女
汽车
娱乐
健康,时尚
文艺,新闻资讯,健康,旅游,军事,体育
美食
新闻资讯,美食,IT 数码,汽车,美女
搞笑幽默,LOL 控,娱乐
文艺,体育,娱乐
健康
游戏动漫,美女,IT 数码,娱乐
美食,军事,IT 数码
美图摄影
投資理财,星座命理
军事
新闻资讯
IT 数码,汽车
新闻资讯,时尚,投资理财,旅游,健康
时尚,娱乐
健康,旅游,星座命理
旅游
体育,文艺
教育就业,健康,旅游,美食,娱乐
军事,IT 数码,文艺
旅游,体育,娱乐

印刷包装,不干胶,不干胶标签
健康,游戏动漫,体育
名人明星,视频音乐,微博奇葩,搞笑幽默,90后但我不脑残,死神,看小说,动漫迷,奋斗
健康,教育就业,时尚,旅游,文艺
爱时尚,爱音乐,爱电影
任性,娱乐,时尚,汽车,美食
健康,体育,娱乐
星座命理,旅游,文艺,体育,娱乐,喜欢胡夏,90后,ab,天蝎座
游戏动漫,时尚,文艺
视频音乐,搞笑幽默,美食,文艺,娱乐,健康,旅游
美女,游戏动漫,IT数码
时尚,游戏动漫,新闻资讯,体育,娱乐
健康,美女,时尚
IT数码
星座命理
新闻趣事
体育,健康,教育就业,投资理财
健康,新闻资讯,体育
新闻资讯,美食,军事,汽车,投资理财
美食,教育就业,新闻资讯
新闻资讯,教育就业,文艺
时尚,汽车,投资理财
美食,旅游,文艺
美食,投资理财,健康,时尚
教育就业
健康
娱乐,投资理财,时尚,美女,星座命理
健康,新闻资讯,美食
军事,IT数码,文艺
时尚,体育
重庆生活,IT数码
旅游,新闻资讯,文艺,教育就业
美女
游戏动漫,娱乐
健康,新闻资讯,旅游
汽车
体育
星座运势
美女,旅游,文艺
魔羯座,开朗,减肥,健康
粉红控,维他命,爱自由,康熙来了,留学,努力,杂志,美食控,睡懒觉,时尚
健康,投资理财,星座命理,旅游,美食
旅游
文艺,教育就业,体育,投资理财,娱乐

汽车
健康,新闻资讯
健康,旅游,美食,星座命理,游戏动漫
教育就业,文艺,时尚,美女
时尚
搞笑幽默
旅游,美食,投资理财,教育就业,体育
美女,教育就业,体育
计算机,K 歌,人生,沉默,睡懒觉,工作,音乐达人,娱乐
游戏动漫,汽车,娱乐
健康,时尚,美食
健康,汽车,星座命理
投资理财,军事,体育
美女,美食,体育
军事,教育就业,文艺,娱乐
汽车,星座命理,文艺
游戏动漫,娱乐,健康,旅游,星座命理
文艺,娱乐,旅游,军事,体育
美女,游戏动漫,时尚
娱乐,文艺,旅游,健康,美食
美女,文艺,娱乐
教育就业,汽车,时尚,美女,旅游,娱乐,美食,体育
宠物图集,怀孕妈咪,孕妈咪,怀孕,母婴行业,母婴
名人明星,微博奇葩,穿衣美容,新闻趣事,美食
军事
健康,美食,娱乐
娱乐
健康,体育,娱乐
娱乐
文艺,体育,娱乐
健康,新闻资讯,娱乐
电视剧,心理学,爱旅游,钢琴,房地产,娱乐,投资理财,军事,教育就业,新闻资讯
时尚,健康
教育就业,旅游,美食
娱乐,投资理财
教育就业
名人明星,旅游,搞笑幽默,新闻趣事,星座命理
娱乐
音樂,旅游摄影,生活,购物狂,旅游
美食,旅游,IT 数码,娱乐
新闻资讯,美女,汽车
魔羯座,幻想,宅女加腐女,爱自由,心情,旅游
健康,美女,IT 数码
时尚,军事,健康,星座命理,娱乐

美食
投资理财
新闻资讯,美食,体育
游戏动漫
游戏动漫,时尚,美食
汽车,旅游,军事
视频音乐,新闻趣事,萌宠,旅游,福建生活,军事
旅游,星座命理,军事,IT 数码,美女
游戏动漫,星座命理,文艺
IT 数码,旅游,游戏动漫,教育就业,新闻资讯
新闻资讯,美女,游戏动漫
星座命理,美女,游戏动漫,体育
妩媚小女人,服饰,喜欢听歌,星座命理,娱乐,白羊女
教育就业,星座命理,文艺
房产,投资理财
IT 数码,校园生活,搞笑幽默
游戏动漫,体育,美食,美女,娱乐
健康,军事,娱乐
旅游,IT 数码,文艺,教育就业,体育
时尚,教育就业,娱乐
美食,美女,文艺,体育
新闻趣事,穿衣美容,美食,萌宠,搞笑幽默,教育就业,时尚,文艺,健康
健康,体育,娱乐
娱乐
美食
星座命理,投资理财
时尚
游戏动漫,时尚,汽车
游戏动漫,汽车,投资理财
时尚,星座命理,美食
汽车
美食
汽车,教育就业,文艺
游戏动漫,时尚,娱乐
美女,健康,旅游,美食,文艺,娱乐
美食,娱乐,文艺,旅游,时尚,教育就业,新闻资讯
教育就业,IT 数码,娱乐
投资理财,旅游,星座命理
教育就业,投资理财,旅游
不二咲千寻,日向创,狛枝凪斗,七海千秋,弹丸论破,暴走漫画,腐女一枚
娱乐
教育就业,健康,新闻资讯,旅游,军事,IT 数码,体育
旅游,文艺
健康

游戏动漫
时尚,星座命理,体育
简单,旅行,散步,唱 k,广州,书
时尚,IT 数码,体育
体育
情感生活,搞笑幽默,美食,星座命理,旅游,健康,娱乐
旅游,娱乐,美食,教育就业,新闻资讯
娱乐,美女
原则性强,网站站长,测试经理,测试工程师,图书作者
宠物图集,性感,臭美,美女,开放,运动
新闻资讯,游戏动漫,军事
娱乐,IT 数码
健康,新闻资讯,娱乐
新闻资讯,星座命理,军事
单纯,销售,朋友,真实力,服饰,时尚达人,自然,音樂,小清新,汽车
汽车,美食
美女,军事
萌宠,美女,旅游,美食,文艺,娱乐
体育,星座命理
时尚,体育
美女
大连同城,微博控,美女,衣食住行,娱乐休闲,旅游,吃货一枚,购物狂,美食攻略,娱乐
汽车,时尚,IT 数码,文艺,体育
学生,宅,狮子座,吃货,双重性格,腐女,动漫,睡觉,看书,学生一族
微博奇葩,搞笑幽默,北京生活,体育,投资理财,健康,旅游,美食
新闻趣事,新闻资讯
星座命理,时尚,美食,文艺,娱乐
健康,时尚,新闻资讯,投资理财,教育就业
搞笑幽默,文艺,体育,IT 数码,娱乐
新闻资讯,健康,教育就业
时尚,健康,旅游,文艺,娱乐
视频音乐,搞笑幽默,游戏动漫
体育
娱乐,汽车,新闻资讯
星座命理
流年带不走厦伤,乐观,幽默,星座命理
安徽生活,汽车
耽美
新闻资讯,教育就业,投资理财,军事
时尚,美食,旅游
美食,教育就业,旅游,军事,时尚,美女,娱乐
龙岩生活,旅游,娱乐
旅游,健康
IT 数码,投资理财,文艺,游戏动漫,教育就业,美食,星座命理,体育

游戏动漫,星座命理,军事
娱乐
新闻资讯,美食,军事
体育
美女,我爱我自己
时尚,旅游,体育,文艺,娱乐
娱乐,星座命理,美食
游戏动漫
名人明星,文艺
美食
IT 数码,健康,旅游,星座命理,美食
文艺
微博奇葩,星座命理,时尚
旅游,娱乐,商业,喜欢音乐,音乐爱好者,异想天开。
美食,娱乐,新闻资讯,健康,旅游,美食,文艺
时尚,健康,美食,文艺,娱乐
名人明星,萌宠,新闻趣事,微博奇葩,搞笑幽默,文艺,娱乐
美女,星座命理
旅游,文艺,娱乐
娱乐
时尚,娱乐,旅游
游戏动漫
星座命理,宅,时尚,旅游美食
游戏动漫
娱乐
美食,旅游,健康,美女,时尚
星座命理,游戏动漫
双鱼座,睡觉,动漫,宅女加腐女,音乐控,心理学,B 型血,猫咪控,美食,星座命理
视频音乐,IT 数码
时尚,健康,星座命理
美食,熟人别关注
名人明星
简单,囧,娱乐
娱乐
星座命理,旅游
星座命理,健康,汽车,娱乐,美女
星座命理,娱乐
旅游
军事,汽车,美女,娱乐,体育
时尚,娱乐,游戏动漫,星座命理
娱乐,游戏动漫,美食
美女
视频音乐,文艺
美食,旅游,星座命理,广东生活,文艺,教育就业

娱乐
搞笑幽默
时尚
投资理财,旅游
吃货,电影爱好者,恐怖片,时尚
游戏动漫
美丽的杜鹃
时尚,娱乐
美女,娱乐
汽车,投资理财,军事
新闻资讯,旅游,娱乐
美图摄影,小提琴爱好者,跆拳道爱好者
投资理财
时尚,IT 数码,文艺,体育,娱乐
文艺,美食
军事,IT 数码,汽车
时尚,IT 数码,文艺
美食
听音乐,发呆,创意,艺术,处女座,胡歌,杨幂,潮,旅游
新闻趣事
旅游,星座命理,时尚,娱乐,文艺
娱乐,文艺
娱乐,快乐
南充生活,军事
IT 数码,旅游,游戏动漫,投资理财,娱乐
婚纱,健康,旅游,美食
广东生活,娱乐,文艺,健康,星座命理
萌宠,微博奇葩,搞笑幽默,美食
音乐控,发呆,搞笑幽默,校园生活,视频音乐,神经质,水瓶座,自然,学生一族,矛盾
喜欢音乐,爱
安静,奋斗,美食爱好者,90 後,钢琴,爱旅游,爱电影,学生一族,时装,娱乐
星座命理
教育就业,军事,文艺
健康,新闻资讯
生活大爆炸,旅游,时尚
健康,娱乐,旅游,时尚
美食
旅游,健康,美食,娱乐,星座命理
星座命理,娱乐
名人明星,搞笑幽默,宠物图集,八卦杂谈,娱乐
时尚,旅游,美食,体育,娱乐
视频音乐,湖南生活,微博奇葩,萌宠,旅游,健康,文艺,游戏动漫
美食
娱乐,文艺

游戏动漫,时尚,文艺
游戏动漫,星座命理,娱乐
娱乐
旅游,文艺,娱乐
娱乐
安徽生活,新闻资讯
文艺,投资理财,教育就业,健康
星座命理
时尚
旅游
星座命理,旅游
文艺,娱乐,旅游,美食,时尚
新闻趣事,穿衣美容,视频音乐,美女,微博奇葩,娱乐,文艺
90 后非脑残,懒人
娱乐
娱乐,星座命理,体育
名人明星,美女
其他生活
娱乐,旅游,文艺,美食,星座命理,新闻资讯
健康,文艺
教育就业,汽车,游戏动漫,健康
美女
文艺,时尚
健康养生,车世界,搞笑幽默,南昌生活,教师考试,教师招聘,事业单位,公务员考试
詹姆斯,文艺控,体育,星座命理
旅游
文艺
美女
娱乐,文艺
军事,体育,游戏动漫
萌宠
旅游,军事,嗜睡,旅行,Lol,Dota
体育资讯,体育
游戏动漫,时尚,IT 数码
娱乐
旅游
搞笑幽默,黑龙江生活,爱吃,巨蟹女,宅女一枚,瑜伽,健康,文艺,汽车,教育就业
美食,娱乐
星座命理
游戏动漫
文艺
新闻趣事,IT 数码,新闻资讯,教育就业
娱乐,体育,旅游,国标舞
时尚,新闻资讯,投资理财,星座命理,娱乐

穿衣美容,杰出领袖,超人,女装,戴安琪,DAIANQI,Fantasy
投资理财,IT 数码,美女,新闻资讯,汽车
旅游,时尚,军事,娱乐,文艺,体育
穿衣美容
美食,美女,文艺
皮划艇,骑行,跑步,户外,体育
视频音乐,萌宠,穿衣美容,时尚,投资理财,旅游,美食,娱乐
IT 数码,文艺,体育
埃菲尔铁塔,林夕,强迫症,摩羯
星星,睡觉睡到自然醒,新闻资讯
新闻资讯,美女,星座命理,娱乐,文艺,教育就业,投资理财,游戏动漫
体育
旅游,时尚,汽车,文艺,娱乐
体育
穿衣美容,新闻趣事,搞笑幽默,新闻资讯,教育就业,星座命理,美食,娱乐
穿衣美容,娱乐
美图摄影,爱美食,爱电影
体育
美食
美女
旅游,美食,健康
投資理财,K 歌,插画,自然,爱笑
教育就业,旅游
旅游,新闻趣事,视频音乐,吃喝玩乐,爱自由,汽车,投资理财,时尚,娱乐
游戏动漫
美食,新闻资讯,健康,时尚,教育就业
旅游
健康,时尚,美食
文艺
名人明星,视频音乐,旅游,星座命理,美食
大学,拍照,情绪化
搞笑幽默,Android 开发,iOS 开发,移动互联网,手机应用,移动开发,手机应用开发,软件外包,app
电影爱好者,微博控,天蝎女
时尚,美食,文艺
北京生活,新闻趣事,投资理财
游戏动漫
星座命理,旅游,健康,投资理财,IT 数码
美白,飘宣 99 墨泥白官,韩国 99 墨泥白,飘宣 99 墨泥白,99 墨泥白,墨泥白
游戏动漫
新闻资讯,时尚,旅游,美食,娱乐
投资理财
IT 数码
娱乐

娱乐
杭州生活,新闻资讯
娱乐
文艺
健康,体育,娱乐
文艺
旅游,美食
游戏动漫,娱乐,健康,星座命理,美食
美食
旅游
旅游
汽车,时尚,美女
石嘴山生活
健康,新闻资讯,旅游,军事,娱乐
IT 数码,旅游,健康,美食,娱乐
星座命理
IT 数码
旅游,美女,时尚,文艺,IT 数码
投资理财,旅游,星座命理
旅游,时尚,娱乐
IT 数码
广东生活,穿衣美容,娱乐
咖啡控,90 後
时尚,健康,旅游,美食
美食
旅游,美食
新闻资讯,美女,文艺,美食,IT 数码,健康,体育
时尚,汽车,军事
听音乐,少女时代
新闻趣事,旅游,江苏生活
美图摄影
旅游,美女,投资理财,娱乐,体育
校园生活
美食,旅游
汽车,时尚,文艺,体育,娱乐
星座命理
吃货一枚,摩羯,宅女加腐女,音乐迷,萌宠
健康,体育,娱乐
投资理财,汽车,时尚,健康,娱乐
体育
全能会计
校园生活,新闻资讯
汽车,教育就业,军事
时尚,军事,体育

旅游
旅游,新闻资讯,IT 数码,娱乐,时尚
美食,时尚,旅游,文艺,娱乐
美图摄影,时尚
星座命理
简单,美食,听音乐,新闻,小清新,爱情,摄影爱好者
旅游,文艺
旅游,汽车,教育就业,IT 数码
文艺,体育,娱乐

金秀贤,文彩元,花样姐姐,张悦轩,李沇熹,이승기,李昇基,两天一夜,IU 李智恩

河北生活
我不有趣,不喜欢看我干嘛
健康,娱乐
美食,时尚,健康
体育
时尚
教育就业,游戏动漫,体育,娱乐
健康
汽车
新闻资讯,娱乐,旅游
新闻资讯,旅游
IT 数码
文艺,体育,娱乐
90 後,听音乐,搞笑幽默,娱乐
健康,文艺,爱睡觉,逛街,90,双重性格,爱笑,爱电影,玩,安静
摄影师,k 歌,旅游美食爱好者,打球,旅游美食,旅游,美食,时尚,汽车,娱乐
投资理财,教育就业,新闻资讯,旅游
健康,体育,娱乐
IT 数码
时尚,星座命理,健康,娱乐
搞笑幽默,微博奇葩,上海生活,美食,穿衣美容
搞笑幽默
美食,视频音乐,体育,娱乐,美女,汽车,IT 数码
时尚
教育,校园生活,亲情,校园,电子商务,写作,大学,好书,文艺,教育就业
努力,音乐迷,nba,王力宏,球迷,美女,军事,文艺,体育
科学探索,旅游,读书分享,创意,白羊座,90 後,林宥嘉,拍照,影视,爬山
健康,体育,娱乐
浙江生活
娱乐
穿衣美容,娱乐
萌宠,投资理财,新闻资讯,军事
新闻资讯,军事,美食,旅游

新闻资讯,美女,时尚,汽车,军事,IT 数码,文艺
搞笑幽默
教育就业,汽车,时尚,健康,娱乐
IT 数码,时尚
投资理财,军事
吃货一枚,单身,睡觉,双重性格,90
搞笑幽默,IT 数码,美女,时尚
美食
游戏动漫
娱乐,自由，空闲
IT 数码,文艺,体育
锡林郭勒盟生活,内蒙古生活
文艺
旅游,健康,娱乐,教育就业,美食
旅游,美食
美食,黔南生活
体育
搞笑幽默,新闻资讯,健康,娱乐
睡觉睡到自然醒,插画,古灵精怪,萝莉控,长发控,爱睡爱吃,别扭,减肥,游戏动漫,美女
美食
旅游美食,投资,感性,健康
娱乐
美食
美食,娱乐
搞笑幽默,微博奇葩
旅游,娱乐
美食
体育
穿衣美容,微博奇葩,浙江生活,美女
娱乐,健康,美食,星座命理,旅游
旅游
体育,星座命理,美女,娱乐,时尚
美食
八卦杂谈,旅游,搞笑幽默,陕西生活,美女
美女,搞笑幽默,新闻趣事,体育资讯,名人明星
旅游
视频音乐
旅游,美食
教育就业,时尚,健康,投资理财,旅游
名人明星,娱乐
投资理财,教育就业,新闻资讯,IT 数码,体育
健康
健康
健康

汽车
娱乐
新闻资讯,军事,汽车,娱乐,时尚
唐山生活,IT 数码,娱乐
新闻趣事
文艺
时尚,娱乐,美食
健身,美剧,健康,美女,体育
新闻资讯,汽车,IT 数码,军事
时尚
娱乐,美食
星座命理,IT 数码,汽车,美女
美食爱好者,罗志祥,服饰,自行车,林宥嘉,逛街,追星,傻,汽车,军事
星座命理,教育就业,投资理财,新闻资讯,娱乐
A 型血
微博奇葩,美食,萌宠,穿衣美容,湖南生活
教育就业,旅游,星座命理,美食,娱乐
90,天枰座,爬山,上网,看书,玩,懒,安静,爱笑,爱美食
娱乐,IT 数码,体育
旅游,娱乐,文艺
娱乐
古风
文艺,娱乐,旅游,星座命理,美食
简单,爱生活
时尚,教育就业,美食,文艺,星座命理
新闻资讯,游戏动漫,娱乐,文艺,IT 数码
视频音乐,神经大条,简单
美女
旅游,健康,文艺,美食
健康,娱乐,旅游,美食
娱乐
娱乐
名人明星,广东生活,美女
游戏动漫
新疆生活,健康,教育就业
A 型血,跆拳道,吸血鬼
情感生活
穿衣美容,视频音乐
音乐控,资讯,hiphop,搞笑幽默,名人明星,90 后,双子座,杰迷
旅游,娱乐,时尚,游戏动漫,星座命理
时尚,娱乐
教育就业,旅游,美食
新闻资讯,投资理财
搞笑幽默

时尚,新闻资讯,教育就业,投资理财,星座命理
新闻资讯,军事,投资理财,健康,体育
视频音乐,旅游,美食
甘肃生活
娱乐
时尚,美女,健康,旅游,军事,教育就业,娱乐,美食
健康,教育就业,汽车,新闻资讯,投资理财
教育就业,新闻资讯,文艺
时尚
文艺
新闻资讯,投资理财,体育
学生一枚,温柔,坚强,阅读
其他生活
健康,旅游,文艺,体育
旅游,健康,美食,星座命理,娱乐
美食
投资理财
星座命理,新闻资讯,游戏动漫,娱乐
北京生活,旅游,美女,健康,美食,娱乐
星座命理
星座命理
时尚,旅游,美食,文艺,娱乐
旅游
搞笑幽默,美女
教育就业,投资理财,旅游
星座命理,广东生活,微博奇葩,视频音乐,搞笑幽默
健康
投资理财,美食
美女,娱乐
娱乐,文艺,IT 数码,美女,游戏动漫
星座命理
沉默,疯狂,潮流,娱乐,吃货,屌丝,贱人,萌神,开朗,文艺
美女,星座命理,发呆,跑步,咖啡控,Android,文化,羽毛球,看小说
旅游,投资理财,IT 数码,体育,汽车
娱乐
新闻资讯,军事,IT 数码,体育,游戏动漫
时尚,旅游,星座命理,美食,娱乐
娱乐,游戏动漫,汽车,体育,旅游
美女,体育,军事,汽车
美食,名人明星,穿衣美容,旅游
美食
北京生活
新闻趣事
搞笑幽默,名人明星,体育

香港,摩羯女,美食,Beyond,摇滚,吉他,唱 K,Music,法语,外语
星座命理,游戏动漫,时尚,健康,旅游
运动的阳光男孩
投资理财
体育资讯,时尚,IT 数码,汽车,体育
教育就业
健康,娱乐
美女,投资理财,娱乐,文艺,IT 数码
天蝎,吃喝玩乐,爱自由,星星
汽车,IT 数码,旅游,健康
教育就业,投资理财,旅游
投资理财,旅游,星座命理
星座命理,美食,军事
文艺,体育,娱乐
健康,旅游,娱乐,时尚,投资理财
游戏动漫,IT 数码,汽车,时尚
IT 数码
汽车,IT 数码,90 后,旅游,喜欢听歌,个性，开朗，阳光
限量控,奢侈品,专业买手,巴黎直送,欧洲代购,时尚
萌宠
星座命理,体育
北京生活,文艺
美食,游戏动漫,旅游
农产品,农业,禽蛋,蔬菜,新鲜,水果,生态,绿色,健康,美食
IT 数码
视频音乐,星座命理,搞笑幽默,穿衣美容,美女
搞笑幽默,萌宠,美食,穿衣美容,微博奇葩,时尚
美食,健康,美女,汽车,军事
娱乐
健康,美食,娱乐,时尚,汽车
健康,星座命理,文艺,教育就业,新闻资讯,旅游,投资理财
穿衣美容,美食,文艺,健康,时尚,时装,音乐发烧友,许嵩,微笑,青春
汽车,IT 数码,体育
名人明星
Gay,90 後,互粉,漫画,东京,吃,二次元症候群,娱乐,星座命理,游戏动漫
时尚,旅游,美食,教育就业,娱乐
新闻资讯
游戏动漫,时尚,美食,旅游,娱乐
体育,娱乐
投资理财,美食
汽车,旅游,IT 数码
美食
体育,投资理财,汽车,旅游,军事
健康

会计,管理,学习,奋斗,爱美食,杂志,爱旅游,笑,多愁善感,星座控
美食,投资理财,时尚,IT 数码,新闻资讯,教育就业
旅游
投资理财
旅游,文艺
时尚,健康,旅游,教育就业,文艺,娱乐
美图摄影,视频音乐,搞笑幽默,星座运势,大连生活
娱乐
美女,90 后,爱笑
汽车
文艺,邢台地产圈,邢台,牛城,地产圈,广告人,策划人,广告,策划,房地产
娱乐
美食,娱乐,旅游,游戏动漫
健康,美女,时尚,汽车,娱乐
投资理财,新闻资讯,体育,娱乐,IT 数码
时尚,游戏动漫
娱乐
文艺
美女,星座命理,文艺
教育就业,娱乐
美食,教育就业
娱乐,星座命理,时尚,健康,旅游
旅游,健康
射手女,欧美,美食
名人明星,星座命理
娱乐
LOL
游戏动漫,军事,博客,青春,仙剑,广播,喜欢音乐,90 后
疯一样的女纸,血型 B,水瓶女,整疯日记,文案,房地产广告
天枰座,自由,睡觉,90 后,郭敬明,许嵩 Vae,喜欢音乐,柯南,游戏动漫,娱乐
美食,投资理财,教育就业,健康,旅游
微博奇葩,健康
90 後,多愁善感,新浪读书,孩子气,文艺,健康,旅游,美食,IT 数码
星座命理,视频音乐,穿衣美容,美食,名人明星
健康,文艺,美食,旅游
旅游
健康,美食,旅游,时尚
美食,文艺,旅游,健康,投资理财
时尚,新闻资讯,汽车,军事,健康,旅游,娱乐
文艺
美食,旅游
投资理财
旅游
美女,体育,星座命理,游戏动漫

广东生活,IT 数码,教育就业,健康,星座命理,旅游
星座命理
娱乐
娱乐,文艺,旅游,美食
新闻资讯,健康,旅游,星座命理,娱乐
新闻资讯,游戏动漫,星座命理,IT 数码,娱乐
时尚,娱乐,旅游
游戏动漫
教育就业,时尚,文艺,娱乐
体育
懒人,喜欢旅游,爱旅游,咖啡控,苹果控,时尚,旅游,美食,IT 数码,汽车
投资理财,新闻资讯,旅游
美食,名人明星,穿衣美容,时尚
桌球,成都,IT 数码
微博奇葩,军事
美女,文艺
旅游,星座命理,美食,IT 数码,娱乐
体育,娱乐
文艺,投资理财,旅游,时尚,健康
旅游,微博奇葩,微信,互联网,营销,教育,资讯,培训,教育就业,IT 数码
天蝎座,自由,无厘头,搞笑,炎亚纶,随性,乐天派,活泼,我就是我,幸福
娱乐,时尚
旅游
星座命理,视频音乐,新闻趣事,穿衣美容,名人明星,游戏动漫
旅游
时尚,健康,星座命理,美食,投资理财,娱乐
文艺,时尚,星座命理,娱乐
体育
投资理财,教育就业,娱乐,旅游,美食
星座命理
文艺
媒体,电视剧,教育就业,汽车,健康,旅游
新闻资讯
旅游
名人明星
时尚,游戏动漫,旅游,星座命理,美食
四川生活,微博奇葩,美食,游戏动漫,旅游,美女,IT 数码
美女,汽车,IT 数码
旅游,娱乐,美食
娱乐
电视剧,学生一族,懒,平常心,健康,随性,高中生,天秤座,倔脾气
星座命理,时尚,汽车,娱乐,投资理财
文艺,摄影,美术,旅游
健康

我喜欢穿丝袜,喜欢丝袜,喜欢丝袜爱丝袜
美食,游戏动漫,娱乐,美女
IT 数码,教育就业,旅游,投资理财,美食
南京生活
旅游,健康,星座命理,美食,娱乐
教育就业,健康,旅游,星座命理,美食
美女,游戏动漫,娱乐,体育
美食
广西,学习,交友,90 后,桂林校园,新鲜事,航院,桂林航天工业学
销售行业,快乐,运动,摄影控,工作,交友,乐观,幽默,时事
文艺
旅游,文艺
美女
旅游,文艺
IT 数码,文艺,健康,美女,教育就业
星座命理,美食
美食,汽车
新闻资讯,军事,娱乐,星座命理,美食
旅游,时尚,新闻资讯
旅游
投资理财,美食,文艺,新闻资讯,时尚,IT 数码
娱乐,星座命理,文艺,美女
健康,美食,美女,汽车,体育
娱乐
娱乐,时尚,健康,旅游,新闻资讯
投资理财,体育,健康,旅游,美女
搞笑幽默
90 后但我不脑残
娱乐
投资理财,军事,新闻资讯,教育就业
投资理财,娱乐,旅游,时尚,美食
新闻资讯,旅游,体育
吃货一枚,杂志,自行车,林宥嘉,hiphop,康熙来了,坚强,自然,意大利
旅游,新闻资讯,教育就业,游戏动漫,健康,美食
军事
新闻资讯,旅游,汽车,教育就业,IT 数码
体育
萌宠
星座命理
新闻资讯,教育就业,健康,旅游,文艺,娱乐
健康,时尚,美食
强迫症,发呆,听音乐,美食,淘宝,纠结
旅游,新闻资讯,游戏动漫,IT 数码,文艺
旅游,美食,时尚,汽车,娱乐

美食,时尚,健康,新闻资讯,旅游
名人明星,文艺,健康,旅游,美食
新闻资讯,旅游
搞笑幽默,时尚,游戏动漫
新闻资讯,投资理财,旅游
汽车,IT 数码,娱乐
美容服饰,名人明星,美食,旅游
体育
美女,广东生活
旅游
美食,星座命理,穿衣美容,娱乐
微博奇葩,文艺,美食,旅游,健康
汽车,美食,娱乐
时尚
育儿百科,星座命理,红楼梦,随性,军事,健康
投资理财,旅游,美食
美食,娱乐
名人明星,喜欢旅游,看电视
房产
军事
特搞,留学,娱乐
上海,意大利,娱乐
学生一枚,健康,美食,天蝎,爬山,美食控
汽车,美食,旅游
健康,娱乐,文艺,旅游,美食
娱乐
金牛座,股票,文学,NBA,摄影爱好者,宅男,美女,网络
教育就业
旅游
健康,投资理财,新闻资讯,IT 数码,旅游,时尚,教育就业,美食
新闻趣事
军事,时尚,娱乐
美食
视频音乐
新闻资讯
萌宠,视频音乐,新闻资讯,时尚,文艺,娱乐
汽车,投资理财,军事
名人明星,腐女,海贼王,SUJU 团饭,娱乐
健康,旅游,文艺
旅游,重庆生活,娱乐
IT 数码,时尚,美食,旅游,娱乐
美食,旅游,健康,新闻资讯,游戏动漫
旅游
娱乐

时尚,文艺,军事,美女,汽车,娱乐
80 后的尾巴,视频音乐
健康
游戏动漫,IT 数码
时尚,美食,娱乐
娱乐
美食,星座命理,搞笑幽默,穿衣美容,河南生活
教育就业
摄影,淘宝,玩,执着,其他生活
美女
热情,大叔控,快乐,活泼,美食控,睡觉,爱吃,陈奕迅,90,双子座
游戏动漫,投资理财,军事
视频音乐
新闻资讯,教育就业,军事
新闻资讯,投资理财,IT 数码
汽车,时尚,娱乐
美图摄影,时尚
旅游,其他生活
时尚,摄影,购物,美食,体育,娱乐
搞笑幽默
美女,搞笑幽默
星座运势,名人明星,视频音乐,搞笑幽默,沈阳生活
微博奇葩
汽车,美食,体育
星座运势,美容服饰
旅游,美食
美食,旅游,时尚,文艺,娱乐
娱乐
旅游,娱乐
体育,游戏动漫
游戏动漫,旅游,IT 数码
体育
微博奇葩
穿衣美容,时尚,娱乐
时尚
旅游,星座命理,美食,娱乐
萌宠,美女
校园生活
名人明星,旅游,视频音乐,搞笑幽默
健康,旅游,美食,游戏动漫
旅游,美食
游戏动漫,时尚,健康,美食
赚钱,无聊,聊天,90 後,巨蟹座
名人明星

旅游,努力努力努力
游戏动漫,美女,星座命理,娱乐,体育,文艺
教育就业,健康,文艺,美食,旅游
双重性格
健康,时尚,美食,旅游,娱乐
体育
留学,新闻资讯,旅游,星座命理
搞笑幽默,时尚,娱乐
新闻趣事,体育资讯,视频音乐,搞笑幽默,看书，听音乐
文艺,新闻资讯
旅游,美食
教育就业,军事,文艺
新闻趣事
微博奇葩
军事
IT 数码,文艺
教育就业,IT 数码
汽车,旅游
旅游
美食,美图摄影,时尚
吃货一枚,教育就业,美食
美食,新闻资讯
体育
新闻趣事,体育
健康,美食,文艺,娱乐
体育,时尚,新闻资讯,健康
游戏动漫
娱乐
体育
时尚,投资理财,旅游,美食,文艺
旅游
娱乐,文艺
微博奇葩,美女,新闻趣事,穿衣美容,星座命理
旅游,娱乐,时尚
资源共享,同学交流,财经,社团,大学生,留学,学生一枚,投资理财,新闻资讯,教育就业
游戏动漫
宠物图集,沈阳生活,视频音乐,文艺,旅游,新闻资讯,美食,娱乐
美图摄影,工商注册,公司注册,深圳工商注册,深圳注册公司,投资理财
健康,旅游,汽车,IT 数码
巧克力,搞笑幽默
投资理财,娱乐,旅游,星座命理,美食,时尚,文艺
娱乐,美食
时尚,旅游,娱乐
美食

新闻资讯
IT 数码,搞笑幽默,军事
IT 数码,娱乐,文艺,旅游,星座命理
投资理财,汽车,娱乐
快乐,热爱生活,自由
娱乐
文艺
旅游,娱乐
美食
萌宠
娱乐
星座命理
美女,旅游,IT 数码,娱乐,时尚
星座命理,萌宠,视频音乐,旅游,爱上阳光的味道,娱乐,时尚
话剧爱好者,相声爱好者,相声,“花痴”,宫崎駿,打篮球,爱吃,旅游,文艺
游戏动漫,旅游
微博奇葩,搞笑幽默,体育
美食,旅游,健康
英格兰,滑雪,涂鸦,庚澈,龟梨和也,体育,文艺,IT 数码,星座命理,健康
时尚
健康,旅游,美食,文艺,娱乐
文艺青年,版画创作,油画,美术高考,少儿美育,绘画
娱乐
旅游,美食,汽车,体育
萌宠,新闻趣事,北京生活,教育就业
时尚,旅游,健康,美食,娱乐
爬山,海贼王,情感,猫,傻,星座命理,萌宠
星座命理,文艺,娱乐,白羊座
IT 数码
投资理财,美女,星座命理,IT 数码,娱乐
娱乐,星座命理,时尚,健康,旅游
星座运势,搞笑幽默,宠物图集,美食,旅游,游戏动漫,星座命理
海贼王,爱旅行,天蝎女,浪漫,热爱生活,健康,教育就业,文艺
美食,时尚,娱乐,旅游
健康,新闻资讯,投资理财,美女,汽车
美女,体育
射手,低调,平面设计,音樂,摄影爱好者,90,文字,旅游美食爱好者,娱乐
时尚,健康,新闻资讯,美食,旅游
旅游,娱乐,时尚,星座命理,文艺,健康
汽车,体育
淡然,看淡,无争,坦然,善良,教育就业,时尚,汽车,健康,娱乐
教育就业,健康,旅游,美食,文艺
健康
科幻,爱情,美女,教育就业

健康
星座命理,游戏动漫
健康,体育,我就是我,找个人陪伴
星座命理,游戏动漫,娱乐,美女,美食
搞笑幽默
鄂州生活,娱乐
文艺
IT 数码,军事,娱乐,汽车,健康
旅游
娱乐
视频音乐
旅游,新闻趣事,搞笑幽默
搞笑,学生一枚,自信,单身,睡觉睡到自然醒
长春生活,新闻资讯,娱乐
体育资讯,美女,搞笑幽默,南京生活,视频音乐
腹黑,玩,互粉,A 型血,火影,学生一族,文艺,游戏动漫,IT 数码
时尚,文艺,娱乐,美食,星座命理
旅游,娱乐,体育
微博奇葩,时尚,娱乐,大学生,偶稀饭睡觉觉,腐女
时尚,娱乐,美食,新闻资讯,投资理财
娱乐
懒,爱笑,90 後,旅遊,广东生活,浪漫,星座命理
IT 数码,娱乐,旅游,军事,健康,美食,投资理财,文艺,新闻资讯
娱乐
旅游,搞笑幽默,南宁生活,教育就业,文艺,美食,健康,时尚
旅游
微博奇葩
睡觉,电视剧,狮子女,爱生活,股票,学生一枚,动画,爱音乐,暮光之城,k 歌
互粉,社团,生物协会,阜阳大学,阜师院,阜阳师范学院,管理,大学,校园
搞笑幽默,微博奇葩
旅游,体育
娱乐
情感生活,其他生活,星座运势,美食
旅游,星座命理,美女,游戏动漫,体育
美食
健康,游戏动漫,军事
星座命理
汽车,军事,文艺
星座命理,军事,健康,娱乐,投资理财
名人明星
爱
旅游
蜜逗兔,桃子,旺仔,美食
旅游,星座命理,军事

新闻资讯,美女,汽车
健康,投资理财,体育
教育就业,美食
时尚,旅游,IT 数码
汽车,投资理财,体育
健康,美食,军事
沈阳生活,娱乐
健康,汽车,星座命理
汽车,投资理财,旅游
健康,教育就业,体育
时尚,汽车,星座命理
时尚,美食,军事
星座命理,游戏动漫
时尚,投资理财,娱乐
时尚,娱乐,教育就业,体育,IT 数码
游戏动漫,汽车
时尚,军事,文艺
新闻资讯,美女,军事
名人明星,视频音乐,微博奇葩,萌宠,时尚,文艺,美食,娱乐
美女,星座命理,体育
视频分享,幽默视频,搞笑,今夜无眠,谁来陪伴,寂寞,孤独,搞笑视频,娱乐
汽车,军事,IT 数码
美女
情感生活,星座运势,美食,校园生活,搞笑幽默
教育就业,旅游,美食
健康,教育就业,星座命理
旅游
新闻资讯,时尚,IT 数码
游戏动漫,美食,文艺
名人明星,文艺
健康,投资理财,体育
汽车,投资理财,美食
微博奇葩,时尚
娱乐,体育
娱乐,文艺,时尚,旅游,健康,美食,IT 数码
新闻趣事,美女,其他生活,微博奇葩,搞笑幽默,教育就业
健康,旅游
美食,游戏动漫,美女
美食
体育
美食,旅游
旅游
90 後,周杰伦,爱电影,媒体人,飞轮海,星座命理,随性,美女,体育,IT 数码
健康,新闻资讯,美女,体育

旅游,美女,汽车,体育
投资理财,时尚,健康,旅游,美食
美食,旅游,教育就业
健康,文艺,IT 数码,体育
双鱼座
美女
美食,萌宠
文艺
新闻资讯,美食,时尚,体育
美女,文艺,IT 数码,军事,汽车,和你到老,和你逛街,和你吃饭,和你在一起
娱乐
娱乐
搞笑幽默
文艺,汽车,DOTA,乐天派,90 后,通信,打球
搞笑幽默
时尚,IT 数码,美女,旅游,星座命理,美食,文艺
娱乐,教育就业,时尚,新闻资讯
微博奇葩,体育,娱乐
旅游
教育就业,IT 数码,美食,游戏动漫,体育
新闻资讯
体育,教育就业,游戏动漫,军事,IT 数码
时尚,游戏动漫,娱乐
时尚,美女,星座命理,文艺,娱乐
旅游
美女
视频音乐
星座命理,军事,文艺,时尚,汽车,教育就业,体育,健康,新闻资讯,娱乐
娱乐,美食
星座命理
读书,军事,文艺,体育,旅游
旅游,体育资讯
真诚,笑,旅游,90 后,电影,巨蟹女,青春,文艺
娱乐,旅游,美女,美食,体育
军事
时尚,教育就业,文艺,IT 数码,娱乐
娱乐
90 后,化妆品,韩剧控,高跟控
游戏动漫,大家好,我是小若,运动,小说,旅游,美食,娱乐,文艺
IT 数码,军事
爱笑,娱乐
美食,看电影
时尚
穿衣美容

美女
投资理财,军事,娱乐
教育就业,美食,军事
健康,旅游,美食
美食,体育,健康,新闻资讯,娱乐,教育就业,IT 数码,时尚,汽车,文艺
草莓晶,玉髓,绿幽灵
文艺,体育,娱乐
教育就业,旅游,新闻资讯,健康,美食
汽车,时尚,旅游,美食
萌宠
新闻趣事,搞笑幽默,健康,教育就业,旅游,时尚
微博奇葩
旅游,娱乐,美女,新闻资讯,时尚
搞笑幽默
美食,健康,文艺
微博奇葩,娱乐,体育
文艺,时尚,健康,娱乐,星座命理
新闻资讯,美女,娱乐
美食,星座命理
减肥,心情,微电影,妖妖
视频音乐,娱乐
健康,旅游,文艺,投资理财,IT 数码
美女,搞笑幽默,投资理财
旅游
美食,健康,教育就业,娱乐,体育
时尚,星座命理,美食,旅游
烹饪,自由,手工蛋糕
旅游
NBA,动漫,80 后,赤木晴子,樱木花道,流川枫,篮球,美女,灌篮高手,游戏
美女,娱乐
美食,文艺
娱乐,旅游,IT 数码
视频音乐,娱乐
教育就业,汽车,美食,娱乐,美女
文艺
娱乐
健康,新闻资讯,体育
旅游,体育
游戏动漫,文艺,汽车,星座命理,美食,军事,IT 数码
IT 数码,文艺,健康,旅游,美食
美食
娱乐
娱乐
校园生活,健康

美食,星座命理,时尚,娱乐
美食,视频音乐,搞笑幽默
军事
襄阳生活,新闻趣事,美食,汽车,投资理财,教育就业,军事,健康
游戏动漫
文艺,星座命理,旅游,美食,娱乐
美食
旅游
汽车,教育就业,投资理财
军事,汽车,新闻资讯
IT 数码,投资理财,教育就业,美食
旅游,健康,娱乐,文艺
旅游,IT 数码,娱乐,体育
笑,巧克力,双鱼女
新闻资讯
名人明星,文艺,IT 数码,体育
游戏动漫,时尚,投资理财,汽车,军事,IT 数码,文艺
真诚,闷骚,读书,蓝色,奋斗 ing,金牛座,K 歌,胡思乱想,影视
汽车
星座命理
小说,娱乐
时尚
投资理财,汽车,时尚,旅游,IT 数码
动漫
时尚,娱乐
体育,娱乐
大连生活,自然,85 后,巨蟹
美女,视频音乐,新闻趣事,锦州生活,文艺,健康,旅游
文艺,旅游,健康,时尚,投资理财,福尔摩斯
投资理财,文艺,娱乐
职场招聘,旅游,北京生活,美女,视频音乐,旅遊,星座命理,娛樂,時尚
美女,时尚,体育
旅游
时尚,潮牌,购物,美食,旅行,摄影,天蝎座,愛狗
准博士,PhD,老男人,缺乏安全感,小豆的学生,研究僧
新闻资讯,游戏动漫,汽车,美食,IT 数码
美容服饰
感性,旅行,自由,80 后,交朋友,电影爱好者,欧美音乐,fashion,时尚,体育
旅游
校园生活,教育就业
IT 数码
投资理财
孩子气,疯狂,美食,旅游
美女,军事,新闻资讯,美食,汽车,投资理财,旅游

军事,教育就业,投资理财,IT 数码,体育,娱乐
新闻资讯,旅游,星座命理,美食,军事
穿衣美容
四川生活
星座命理
新闻资讯,健康,旅游,文艺,教育就业
旅游,时尚,健康,美食,文艺
体育
旅游,投资理财
黄石生活,娱乐
星座命理
名人明星,萌宠
吃，喝，玩，乐
宅女无敌
微博奇葩,萌宠,美食,名人明星,视频音乐
美女,娱乐
旅游,娱乐
文艺,美女
时尚,游戏动漫,旅游
其他生活,健康
文艺,体育,娱乐
新闻资讯,体育,健康,美女,文艺
武术,拍照,体育
花样美男
温州生活
娱乐
星座命理
甘肃生活
教育就业
爱笑,搞笑图片,笑話,搞笑派,搞笑幽默,体育
美女,娱乐
旅游
K 歌,计算机,无聊,热情
英国生活,时尚
闷骚…
健康,新闻资讯,游戏动漫
美女,汽车,投资理财,娱乐
时尚,新闻资讯,娱乐,星座命理,美食
名人明星
旅游
旅游,星座命理,美食
爱幻想,宠物,强迫症,吸血鬼,快乐大本营,电影,传媒,广播电视编导,大学
军事,文艺,娱乐
娱乐,军事,IT 数码,文艺,体育

微博奇葩,视频音乐
吸铁石,磁扣,塑料,五金工具,磁性材料,Ndfeb,铁钕硼,磁钢,磁铁,健康
星座命理
微博奇葩,搞笑幽默,广东生活,投资理财,旅游,游戏动漫,时尚,IT 数码
投资理财,IT 数码,旅游,汽车,体育
健康,新闻资讯,汽车,旅游,美食
体育
武術,武术培训,武术指导,武术爱好者,武术强身,武术搏击,武术散打,武术,教育就业,体育
星座命理,美食,体育
旅游,美食,娱乐
娛樂
陈学冬,刘诗诗的小狮子,张翰,JPM,小时代
健康,星座命理,投资理财,文艺,体育
美食
吃货,爱旅行,大叔控
北京生活,财经资讯,投融资,律师,内部控制,法律风险管理,武术,双子
视频音乐,军事,体育
时尚,教育就业,体育
新闻资讯,IT 数码,体育
临安中心,临安单身公寓,繁华配套,国际名师设计,苕溪边,人民广场边,美式精装公寓,创富办公,中心商业
游戏动漫
新闻资讯,时尚,汽车,教育就业,IT 数码
投资理财,美食,IT 数码
美女
文艺
视频音乐,美女,时尚
游戏动漫
游戏动漫,健康,旅游,星座命理,美食
娱乐,健康
文艺,教育就业
music,乐观,写字,坚强
体育
汽车
星座命理,时尚
健康
旅游
搞笑幽默,新闻趣事,新疆生活,微博奇葩
其他生活,新闻趣事,视频音乐,穿衣美容
健康
娱乐,时尚,美食
健康,旅游,新闻资讯,教育就业,文艺
珠海生活,穿衣美容,名人明星
胡思乱想,新闻趣事,搞笑幽默,视频音乐,微博奇葩,安徽生活,新闻资讯,IT 数码,体育

视频音乐,汽车
健康,教育就业,旅游,美食,娱乐
健康,教育就业,投资理财,时尚,娱乐
美食,旅游,新闻资讯,健康,时尚
名人明星,旅游,微博奇葩,新闻趣事,搞笑幽默
美女,娱乐
健康,娱乐
育儿百科,情感生活,美容服饰,新闻趣事,山东生活,新闻资讯
娱乐
名人明星
体育
美食,娱乐,健康,旅游,文艺
星座命理
旅游,军事,娱乐
蓝色,音樂,怀旧
微博奇葩,教育就业,体育,文艺,IT 数码,娱乐
穿衣美容,娱乐,健康,时尚
美食,星座命理,四川生活,名人明星,娱乐
文艺,体育,娱乐
健康,体育,娱乐
娱乐
健康,美女,体育
美女,教育就业,星座命理
健康,新闻资讯,娱乐
新闻资讯,美女,游戏动漫
汽车,星座命理,美食
投资理财,旅游,美食
游戏动漫,旅游,美食
汽车,旅游,美食
名人明星,新闻趣事,搞笑幽默,穿衣美容,星座命理
新闻资讯,健康,旅游,军事,娱乐
娱乐,星座命理,美食
文艺,体育,娱乐
教育就业,旅游,体育
文艺,体育,娱乐
汽车,军事,IT 数码
汽车,教育就业,投资理财
美女,美食,军事
时尚,军事,IT 数码
时尚,美食,IT 数码
投资理财,旅游,星座命理
美食,IT 数码,文艺
军事,IT 数码,文艺
投资理财,美食,军事

美女,时尚
宅女一枚,影视,听音乐,蓝色,笑,自然,90 后非脑残,时尚,娱乐
游戏动漫,教育就业,投资理财
文艺,体育,娱乐
星座命理,美食,娱乐
汽车,投资理财,IT 数码
游戏动漫,时尚,军事
教育就业,文艺,时尚,新闻资讯,健康
文艺,体育,娱乐
投资理财,军事,文艺
健康,星座命理,娱乐
时尚,旅游,娱乐
星座命理,美食,军事
文艺,体育,娱乐
健康,旅游,美食,IT 数码,娱乐
美女,游戏动漫,星座命理,娱乐
美女,投资理财,星座命理
游戏动漫,美食,文艺
投资理财,旅游,星座命理
文艺,娱乐,时尚,旅游,美食
美女,旅游,星座命理,IT 数码,汽车
娱乐,体育
教育就业,军事,文艺
游戏动漫,时尚,军事
美女,健康
健康,时尚,军事
投资理财,旅游,星座命理
汽车,教育就业,体育
搞笑幽默,美食,天津生活,新闻资讯,汽车,体育
教育就业,新闻资讯,文艺
健康,新闻资讯,体育
时尚,IT 数码
健康,体育,娱乐
其他生活
健康,新闻资讯,体育
汽车,体育
健康,美食,体育
健康,体育,娱乐
文艺
教育就业,旅游,体育
新闻资讯,游戏动漫,星座命理
投资理财
军事,IT 数码,文艺
文艺

新闻趣事,美食,穿衣美容,星座命理,其他生活
萌宠,名人明星,文艺
军事,体育,娱乐
时尚,教育就业,娱乐
朋友,创业,足球迷,自由,NBA,80 後,市场营销,广告,90 後,移动互联网
星座命理,娱乐,时尚,IT 数码
健康,新闻资讯,娱乐
新闻资讯,美女,游戏动漫
搞笑幽默,体育
旅游
美食,旅游
健康,新闻资讯,军事
游戏动漫,时尚,军事
美女,时尚,汽车
时尚,汽车,旅游
创意设计,科学探索,车世界,军事天地,工作,政治,军事,IT 数码,汽车
健康,体育,娱乐
游戏动漫,投资理财,文艺
美女,投资理财,旅游
IT 数码,体育,娱乐
新闻资讯,投资理财,军事
新闻资讯,汽车,美食
文艺,体育,娱乐
视频音乐,职场招聘,读书分享,健康,汽车,旅游,美食,文艺
搞笑幽默
视频音乐,娱乐
旅游
游戏动漫,军事,娱乐
美食,军事,IT 数码
汽车,星座命理,文艺
美女,旅游,文艺
投资理财,美女,健康
健康,汽车,投资理财
教育就业,星座命理,文艺
游戏动漫,美食,军事
健康,体育,娱乐
教育就业,军事,体育
游戏动漫,美食,娱乐
上网,幻想,古典音乐,设计师,独立,文化,可爱,艺术,诗词,爱自由
健康,新闻资讯,娱乐
新闻趣事,投资,社会,投资理财,新闻资讯,娱乐
美女,投资理财,军事
新闻资讯,IT 数码,娱乐
美食,工作,人生,互联网,教育就业,旅游,健康,新闻资讯

健康,教育就业,文艺
美女,汽车,美食
美食,健康,旅游,星座命理,文艺
新闻资讯,汽车,体育
美食,军事,IT 数码
美女,时尚,娱乐
成都生活,大学生,职业能力,万学职业精英社,万学教育,教育就业
文艺,体育,娱乐
IT 数码,体育
汽车,教育就业,投资理财
美女,美食,文艺
新闻资讯,美女,文艺
教育就业,旅游,体育
健康,新闻资讯,娱乐
时尚,星座命理,旅游,娱乐
美食,旅游
微博奇葩,文艺
车世界,视频音乐,重口味,动漫,美图摄影
都市菜园,自主管理,CSA 模式,养,养生,养生农业,绿色农业,有机农业
健康,新闻资讯,时尚,美食,娱乐
厦门生活,单车旅行,旅游爱好者,人力资源管理,HR,教育就业
文艺,美食,旅游,时尚,娱乐
时尚
旅游,美食,时尚,文艺,娱乐
投资理财
娱乐
投资理财,军事,体育,汽车
时尚,旅游,美食,文艺,娱乐
游戏动漫,星座命理,文艺
旅游,教育就业,汽车,健康,美女,娱乐
IT 数码
教育就业,文艺,时尚,新闻资讯,旅游
健康,新闻资讯,汽车,教育就业,投资理财
美食
性感美女,性感,睡觉睡到自然醒,吃货一枚,交朋友,苹果,健康,美女,时尚,美食
搞笑幽默
体育资讯,体育
微博奇葩,游戏动漫
旅游,微博奇葩,视频音乐,娱乐,游戏动漫,美食,文艺,动漫、小说、吃
娱乐,体育,IT 数码
萌宠,健康快乐,七步庄园,Evangelion,美女
农产品,电子商务,美食,旅游,健康
IT 数码,新闻资讯,健康,旅游,美食
时尚

娱乐
新闻趣事,旅游,微博奇葩,视频音乐
旅游,新闻趣事,广东生活,穿衣美容,健康
视频音乐,穿衣美容,美食,旅游,名人明星,汽车
美食爱好者,健康,旅游,时尚
科学探索
微笑
时尚
视频音乐,旅游,美女,新闻趣事,西安生活
游戏动漫,IT 数码
投资理财,美女,时尚,新闻资讯,军事,健康
美容服饰,名人明星,吉安生活,娱乐,教育就业,文艺,时尚
摄影,娱乐,文艺,旅游,美女,时尚
旅游,健康,时尚,文艺,娱乐,美食
军事,时尚,文艺,教育就业,旅游,体育
文艺,健康,旅游,星座命理,美食
旅游,星座命理
爱自由,学习,自信,跆拳道,吃货,时尚潮流,射手女,90 後
投资理财
旅游,星座命理
贵州生活,星座命理,美食,旅游,穿衣美容
美食,旅游,娱乐,体育
萨德,旅游,电影,水瓶座,女王主義,自由,SM,黑貓
名人明星
星座命理,时尚,娱乐
旅游,IT 数码,文艺
汽车,旅游,文艺
教育就业,美食,文艺
新闻趣事,美食,萌宠,娱乐,健康,新闻资讯
美食
旅游,娱乐,美女
健康,教育就业,文艺,旅游,投资理财
美食,健康
美图摄影
新闻趣事
体育
打篮球,自由,网络,教育就业
娱乐,军事,美女,旅游
大学,投资,聊天,法律,唱歌,旅行,心情,朋友,奋斗,学习
美食,旅游,军事,投资理财,教育就业
美女
成都生活,Linux,移动互联,WEBAPP,OA,JAVA,汽车,新闻资讯,美女,IT 数码
时尚
穿衣美容,新闻趣事,搞笑幽默,美食,旅游

微博奇葩,新闻趣事
新闻趣事,新闻资讯,教育就业,体育,文艺
活泼,外向,喜欢交朋友
情绪化,90 後,睡觉睡到自然醒,影视,旅游爱好者
游戏动漫
星座命理,健康,投资理财,旅游,IT 数码
北京生活
健康,投资理财,时尚,娱乐,体育
投资理财,健康,体育
星座命理,IT 数码,体育
汽车,旅游,IT 数码
教育就业
军事,IT 数码,文艺,体育
多面性，羽毛球
星座命理,美食,军事
文艺,美食,新闻资讯
时尚,新闻资讯,汽车,文艺,体育
文艺,健康
佛系女子,BG 小战士,让让子亲妈,利佩利伊滚蛋,利韩本命
四川生活,新闻趣事,旅游,微博奇葩,搞笑幽默
娱乐,体育,旅游
美女,旅游,视频音乐,名人明星,新闻资讯,星座命理,军事,汽车,娱乐
微博奇葩,教育就业,新闻资讯,时尚,文艺
游戏动漫,旅游,美食
新闻趣事,视频音乐,美女,名人明星
时尚,星座命理,文艺
健康,教育就业,IT 数码
时尚,美食,娱乐
投资理财
军事
文艺控,小说推荐,晋江文学城,红袖,潇湘,红薯小说网,小说技巧,萌 H,小说迷
美女
美女,穿衣美容,鲜花速递,手机充值,对外汉语,淘宝店主,游戏动漫,美食,IT 数码
新型农村社区,教育就业,投资理财,汽车,文艺
旅游
美食,军事,IT 数码,旅游,游戏动漫
搞笑幽默,军事
穿衣美容,名人明星,视频音乐
健康,投资理财,旅游,星座命理,IT 数码
文藝,遊戲動漫,美食
健康,旅游,星座命理,美食,娱乐
美女,美食,穿衣美容
时尚,美食,娱乐
旅游,IT 数码,体育,娱乐

娱乐
时尚,旅游,美食,健康,娱乐
郑州生活,新闻资讯
穿衣美容
健康,娱乐,新闻资讯,旅游,美食,体育
美女
美食,搞笑幽默,新闻趣事,文艺,汽车,美女
微博奇葩,搞笑幽默
强迫症,神经质,非典理工男,很靠谱,爱生活
美女
湖北生活,教育就业,游戏动漫
健康,时尚,旅游,星座命理,美食
体育,臭美,90 后但我不脑残,花痴,喜欢唱歌,自拍
咖啡控,电影控,狗狗
八卦杂谈
投资理财
脑残
教育就业,IT 数码
新闻趣事,新闻资讯
新闻资讯,教育就业,美食
时尚,旅游,游戏动漫,娱乐,美食
旅游,美食,游戏动漫
美女,健康,汽车,旅游
旅游,搞笑幽默,动漫,星座命理,微博奇葩,娱乐,军事,游戏动漫
时尚,娱乐
对联,古典文学，诗词,音乐，旅游
勇士
美食,文艺,IT 数码,时尚,娱乐
IT 数码
体育资讯,美食
旅游
穿衣美容
健康,旅游
名人明星,视频音乐,搞笑幽默,旅游,新疆生活
文艺,游戏动漫,美女
听音乐
时尚,新闻资讯,旅游,美食,文艺
娱乐,体育
旅游,汽车,新闻资讯,健康,IT 数码
旅游
名人明星,IT 数码,投资理财,教育就业
新闻趣事,时尚,游戏动漫
娱乐,游戏动漫
游戏动漫,文艺,美食

旅游, 新闻资讯
美食, 健康, 娱乐, 体育
美女
旅游, 健康, 教育就业, 军事, 新闻资讯
游戏动漫, 汽车, 美食, 文艺, 旅游
穿衣美容, 喜欢尝试新事物, 爱玩爱交友, 狮子座
汽车, 旅游, 健康, IT 数码
娱乐, 军事, 体育
投资理财, 健康, 娱乐
健康, 旅游, 美食, 娱乐
情绪化, 双鱼女, 文字, 紫色, 沉默, 幻想, 双重性格, 文艺
其他生活
按摩师, 中医养生, 电脑, 狗狗, 投资理财, 游戏动漫, 体育
旅游, 金牛, 乐天派, 绘画
IT 数码, 时尚, 汽车
军事, 投资理财
巴音郭楞生活
军事
策划, 政治, 公益, 管理, 新闻, 市场营销, 健康
时尚
旅游, 时尚, 教育就业, 文艺, 娱乐
健康, 娱乐, 文艺, 时尚
海贼王, 摄影控, 火影, 潮流, 好书, 搞笑幽默, 四川生活, 微博奇葩, 美食, 旅游
时尚, 旅游, 星座命理, 美女, 美食
网络营销, 微博奇葩, 萌宠, 视频音乐, 新闻趣事, 郑州生活
新闻资讯, 旅游, 文艺, 体育, 娱乐
时尚, 星座命理, 娱乐
搞笑幽默, 微博奇葩
美食
健康, 旅游, 美食, 文艺, 娱乐
笑, 音乐发烧友
丝袜奶茶, 张家港, 美食
微博奇葩, 搞笑幽默, 视频音乐, 美女, 穿衣美容
新闻趣事, 旅游
搞笑幽默, 娱乐
搞笑幽默, 旅游
旅游, 时尚, 投资理财, 娱乐
游戏动漫, 娱乐
佛山生活, 旅游, 萌宠
星座命理
游戏动漫
体育, 娱乐, 时尚, 旅游
时尚, 健康, 旅游, 美食, 娱乐
军事, IT 数码, 文艺, 体育, 娱乐

安徽生活,新闻资讯,时尚,投资理财
国际空运,国际快递,邮政小包,国际货运代理
音乐,文艺,健康,旅游
音乐迷,欧美,视频音乐,穿衣美容,时尚
时尚
时尚,新闻资讯,体育,军事
健康,时尚,星座命理,文艺,娱乐
文艺,旅游
时尚,文艺,娱乐
健康
美女
双鱼座,视频音乐,旅游,其他生活,美食
新闻趣事,湖北生活,美女,旅游,名人明星
游戏动漫
旅游,星座命理,美食,时尚,IT 数码
新闻趣事,微博奇葩
旅游,穿衣美容
星座命理,汽车,体育,新闻资讯
微博奇葩,搞笑幽默,汽车,投资理财,教育就业,体育,娱乐
健康,美女,娱乐,IT 数码,投资理财
美容服饰,校园生活,视频音乐,青岛生活,汽车,时尚,美食,文艺,娱乐
美食,健康,星座命理,旅游,时尚
旅游
微博奇葩,穿衣美容,爱笑,萝莉
星座命理
新闻资讯
旅游,美食,娱乐
新闻资讯,时尚,星座命理,美食,娱乐
汽车,新闻资讯
搞笑幽默,新闻趣事,读书分享,教育,星座运势
美食,美食,教育就业,新闻资讯,娱乐,文艺
名人明星,旅游,微博奇葩,视频音乐,穿衣美容
健康,时尚,旅游,文艺,娱乐
IT 数码,体育,娱乐,汽车,时尚,美食
健康
美食
健康,体育,IT 数码,新闻资讯,旅游
美女,游戏动漫
北京生活,文艺
游戏动漫,星座命理
名人明星
宠物图集
美女,IT 数码
美食,娱乐

微博奇葩,美女,旅游,新闻趣事,重庆生活
旅游,视频音乐
游戏动漫
时尚
西安生活,娱乐
美发，美容，旅游
娱乐,健康,旅游,美食
新闻趣事
视频音乐,搞笑幽默
时尚,星座命理,娱乐
星座命理,美食,娱乐,文艺
广东生活
美女
上海生活,新闻资讯,投资理财
时尚,旅游,美食,文艺,娱乐
美女,文艺,时尚,娱乐,体育
娱乐,教育就业,旅游,美食,文艺
花样美男
新闻趣事,汽车
美食,星座命理,旅遊,教育就業,文藝
微博奇葩,新闻趣事,星座命理,美女,浙江生活,健康
时尚
健康,体育,娱乐
美食,文艺
旅游
娱乐
健康,投资理财,旅游,军事,文艺
体育
星座命理,健康,汽车,投资理财
娱乐,体育,文艺,IT 数码,投资理财,健康,旅游,星座命理,美食
乐天派,天蝎女,单身,美食控,我就是我,小说迷,发呆,淡定,宅女加腐女,90 後
文艺,新闻资讯,时尚,健康,旅游
时尚
娱乐,教育就业,健康,星座命理
时尚,汽车,军事
搞笑幽默,星座运势
矛盾体,哈哈,娱乐
美女,旅游,IT 数码
穿衣美容,广西生活,美女
名人明星,新闻趣事,教育就业,文艺,旅游,娱乐
教育就业,游戏动漫,新闻资讯,娱乐,文艺,IT 数码,投资理财
美食,娱乐
手串,美食,摄影,旅行,美好,温度,原创设计,时尚,文艺
美食,旅游,健康,文艺,星座命理

美食,甜品
视频音乐,星座命理,美食
美女,时尚,IT 数码
旅游,策划,建筑,财经,房地产
星座命理
爱懒懒、微博控
活泼,跆拳道,巨蟹座
娱乐,文艺
娱乐
星座命理,游戏动漫,美食
美食,娱乐,文艺
视频音乐,快乐,投资理财
新闻趣事,星座命理,时尚,健康,投资理财
军事,体育,文艺,新闻资讯,教育就业
萌宠,搞笑幽默,穿衣美容,健康,旅游,时尚,美食,娱乐
星座命理,娱乐
时尚,IT 数码,娱乐
星座命理
美食
时尚
美食,旅游
健康
健康,游戏动漫,娱乐
美图摄影,新闻趣事,校园生活,旅游,健康,时尚,文艺,美食
IT 数码
文艺,体育,娱乐
星座命理,娱乐
旅游,美食,体育
星座命理,娱乐
娱乐
健康
文字控,美食,穿衣美容,星座命理,旅游
名人明星,视频音乐,校园生活,广东生活,搞笑幽默
人生,简单,音乐控,爬山,文艺,娱乐,军事,投资理财,旅游
汽车,文艺,旅游,游戏动漫,娱乐,时尚
健康,新闻资讯,时尚,娱乐,美食
教育就业,旅游,时尚,健康
音乐发烧友
娱乐
名人明星,娱乐,游戏动漫
时尚,娱乐,旅游
娱乐,文艺
娱乐
搞笑幽默,视频音乐

其他生活,文艺
游戏动漫,旅游,美食
上海生活,健康,IT 数码
新闻趣事,微博奇葩,搞笑幽默,乐观,幽默,篮球,NBA,动漫控,电影控,体育
汪東城忠實 FANS,美食,星座命理,娱乐
微博奇葩,搞笑幽默,游戏动漫
旅游,爱自由,热情,电影控,文字控,唱 K,胡思乱想,睡觉睡到自然醒,敏感,娱乐
穿衣美容,美食
时尚,健康,旅游,美食,文艺
美容服饰,名人明星,美食
游戏动漫,美女
吃喝玩乐
星座运势,视频音乐,政治,外贸,书籍,水瓶女,人生,音乐达人,娱乐,星座命理
美食,健康,新闻资讯,时尚,投资理财
萌宠,微博奇葩,搞笑幽默,游戏动漫
健康,星座命理,IT 数码,美女,娱乐
健康,星座命理,时尚
游戏动漫,美食,健康,旅游,娱乐
健康,教育就业,体育
搞笑幽默
美食
新闻资讯,美女,美食,游戏动漫,体育
娱乐,文艺
搞笑幽默,旅游,美食,穿衣美容,星座命理
音乐,小号,长笛,单簧管,大提琴,小提琴,吉他,萨克斯,钢琴,乐器
旅游,教育就业
新闻资讯,娱乐
旅游,教育就业,时尚,军事,文艺
萌宠,微博奇葩,美食
如果没有如果
巨蟹女,开朗,任性,爱生活,可爱,爱睡觉,新闻资讯,健康,时尚,美食
music,美剧控,影视
美食
旅遊,時尚,娛樂
自由,商业,销售,看电影,开朗,景逸,汽车,时事,旅游
星座命理,投资理财
辽宁生活
90 后
文艺,旅游
搞笑幽默,山东生活,美女
旅游
爱笑,娱乐,时尚
健康,新闻资讯,体育,教育就业,文艺
微博奇葩,IT 数码

游戏动漫,健康,旅游,美食
旅游
美食
韩饭一枚,视频音乐,名人明星,娱乐
动漫,游戏动漫
旅游
投资理财,健康,星座命理,文艺,IT 数码
娱乐
健康,旅游
美容服饰,时尚,健康,旅游,美食,娱乐
星座命理
健康,军事,体育
文学,浪漫,向日葵
旅游,娱乐,时尚
旅游,美食,名人明星,搞笑幽默,微博奇葩
穿衣美容,新闻趣事,搞笑幽默,旅游,新闻资讯,美食,娱乐,文艺,乐天派
投资理财,健康,旅游,文艺,娱乐
时尚,娱乐,星座命理,文艺,投资理财
健康,新闻资讯,娱乐
新闻趣事,搞笑幽默,新疆生活,美食,教育就业,娱乐,健康,旅游
星座命理
星座命理,美女,游戏动漫
小说,阅读，90 后,音乐
旅游,健康,美食,文艺,娱乐
校园生活,搞笑幽默,其他生活
美食,新闻资讯,时尚,文艺,娱乐
旅游
体育
健康,体育,娱乐
健康,新闻资讯,娱乐
健康
美容服饰,搞笑幽默
投资理财
星座命理,时尚
旅游,美食,美女,时尚,娱乐
名人明星,健康
gay,昆明滴孩纸,吸血鬼日記,吃货一枚,旅游
时尚,健康,星座命理
视频音乐
犬夜叉,爱动漫
新闻趣事,旅游,投资理财,娱乐,时尚,新闻资讯
名人明星,娱乐
视频音乐,投资理财
游戏动漫,美食,星座命理,旅游,IT 数码

微博奇葩,美食,穿衣美容,旅游,时尚
情绪化,学生一枚,搞笑幽默,篮球运动员
健康,体育,娱乐
新闻趣事
体育资讯,NBA,科比,孩子气,体育
美食,旅游,IT 数码
游戏动漫
文艺,零零后,摩羯女,李炜,上海,麦霸,维他命,写字,爱唱歌
JustinBieber,Jay,音乐,旅游
旅游
穿衣美容
新闻趣事,IT 数码,军事,旅游,星座命理,娱乐
娱乐,文艺,旅游,星座命理,美食
美食,旅游,星座命理,汽车,听歌
娱乐,体育
游戏动漫,健康,星座命理,美食,军事
化妆品,动画,星座命理,文艺,时尚
新闻资讯,时尚,旅游,美食,文艺
时尚,旅游,IT 数码
美食,搞笑幽默,投资理财,娱乐
搞笑幽默
美食
美食,时尚,游戏动漫
旅游,星座命理,美食,文艺,娱乐
美女,美食
投资理财
穿衣美容,时尚
娱乐,投资理财,时尚,新闻资讯,军事
刘亦菲,刘诗诗,娱乐,舞蹈,旅游
搞笑幽默
90 後,爱电影,微博奇葩,星座命理,搞笑幽默
军事,体育,旅游,文艺
恋爱,单身,美女,棉湖中学,棉湖,旅游,游戏动漫,娱乐
娱乐
娱乐
娱乐,美女,健康,美食,文艺
新闻趣事
体育,游戏动漫,文艺,娱乐,时尚
美女,娱乐,健康,旅游,体育
旅游,IT 数码,教育就业
娱乐
名人明星,文艺
游戏动漫
搞笑幽默,新闻资讯,军事,IT 数码

手机,动漫迷,南非,看电视,IT,IT 数码
体育
娱乐,体育
汽车
搞笑幽默,投资理财
搞笑幽默
视频音乐,娱乐
游戏动漫,汽车,旅游,美食,军事,IT 数码
旅游,星座命理,美女,新闻趣事,搞笑幽默,汽车,IT 数码
90 后非脑残,K 歌,情绪化,怀旧
游戏动漫
娱乐
健康,时尚,美食,文艺,娱乐
健康,教育就业,新闻资讯,旅游,IT 数码
军事,旅游,美女
八卦杂谈
美女
新闻资讯,时尚,旅游,文艺,娱乐
美食,旅游,星座命理,汽车,听歌
时尚,美食,娱乐,旅游
IT 数码,美食,时尚,娱乐
美食
教育就业
穿衣美容,视频音乐,搞笑幽默,星座命理,汽车,美女,娱乐,体育
美食,旅游
搞笑幽默,游戏动漫
新闻资讯,IT 数码,旅游,美食,投资理财
视频音乐,萌宠,美女,微博奇葩,旅游
健康
汽车,音乐,看书
旅游,美食
美女,游戏动漫,时尚,美食,娱乐
美女
星座命理
美食
北京生活
视频音乐,搞笑幽默,画画,摄影,自行车,健康,汽车,旅游,文艺,IT 数码
玩,爱吃,护肤,快乐大本营,美容护肤,狗狗,明星,广西生活,娱乐
美食,新闻趣事,搞笑幽默,浙江生活,上网,购物,电脑,股票,游戏
娱乐
健康,美食,新闻资讯,文艺,旅游
教育就业,时尚,健康,旅游,美食
瘦身,美剧,双重性格,美食,旅游,猫咪,看电影,Music,瑜伽,文学
名人明星,体育,游戏动漫

早安,我的朋友
旅游,美食,体育,军事
美女
宠物图集
美食
娱乐,游戏动漫,星座命理,IT 数码,文艺
新闻资讯,旅游,军事,投资理财,汽车
校园生活,文艺
体育
新闻资讯
旅游,星座命理,美食
新闻趣事,穿衣美容,美女,旅游
体育,旅游,汽车,电影爱好者,青春,文艺
视频音乐,旅游
美食,娱乐,星座命理,文艺,健康
文艺,汽车,旅游,军事,体育
美食
美女
双鱼座,发呆,高调,嚣张,善变,不完美主义,爱睡觉,三分钟热度
时尚
星座命理,军事,文艺,游戏动漫,投资理财,IT 数码
美食
健康,新闻资讯,旅游,美食,娱乐
萌宠,文艺,旅游
搞笑幽默,文艺,娱乐
文艺,美食,健康,新闻资讯,美女
旅游
体育
重口味,未满 18,腐妹纸,ML,女性,内涵,神秘,文艺
IT 数码
旅游,投资理财,新闻资讯,体育,娱乐,汽车
美食
天津生活
投资理财
旅游,名人明星,新闻趣事,视频音乐,微博奇葩
投资理财,新闻资讯,健康,游戏动漫,旅游
电影,棋牌,看电影
可爱,帅哥,美容,青春,潮流,养生,其他生活,娱乐,文艺,健康
时尚
宠物图集,胡思乱想
健康,新闻资讯,娱乐,星座命理
搞笑幽默,美女,军事
美女,军事,汽车
名人明星

文艺,体育,娱乐
健康,文艺,娱乐
星座运势,美图摄影,美食,深圳生活,投资理财,文艺
视频音乐,校园生活,美图摄影,搞笑幽默,工作狂 ing,事业王,美女
体育,娱乐
体育,娱乐
微博,韩剧
IT 数码
新闻资讯,旅游,美食,体育
旅游,美食,时尚,汽车,体育
搞笑幽默,文艺
不知道说啥
时尚,旅游
旅游,美食,娱乐
IT 数码
健康
旅游
篮球爱好者
投资理财
健康
健康
娱乐,投资理财,体育
IT 数码
投资理财,文艺,军事,新闻资讯,健康
政治,外贸,美剧,大学生,摄影师,穿衣美容,旅游,视频音乐,新闻趣事,搞笑幽默
星座命理,娱乐,教育就业,美食,健康,旅游
萌宠,安徽生活
校园生活,文艺
投资理财,军事
微博奇葩,新闻资讯
自由职业
校园生活
时事,足球迷,阿森纳
名人明星
搞笑幽默,名人明星,旅游,美食,健康养生
旅游
汽车
济南生活,山东生活
旅游
美图摄影,健康,新闻资讯,美食,时尚
重庆,羽毛球,淘宝,游戏动漫
健康
名人明星
时尚,汽车,美女

军事,体育,投资理财,时尚
河南生活
时尚,星座命理
cos,二次元,ACG,不基不腐不基三
美食
宠物图集,视频音乐,校园生活,星座运势,名人明星,体育,娱乐
美剧迷,健康,美食,娱乐,文艺
星座命理,健康,美食,美女,体育
萌宠,投资理财
时尚
新闻资讯,投资理财,美食
手机,喜欢音乐,懒人,外貌协会,耽美,唱 K,手工,旅游美食,学生一枚,健康
湖南生活
水瓶,腹黑,沉默,摄影爱好者,跆拳道,高中生,魔兽,心理学爱好者,小提琴,喜欢音乐
美食
情感生活,八卦杂谈,花样美男,动漫,搞笑幽默
娱乐
星座命理
旅游
美食,文艺,汽车,健康,新闻资讯
时尚,美食,星座命理,健康,文艺
教育就业,星座命理,投资理财,汽车,文艺
美女,教育就业,星座命理
新闻趣事,微博奇葩,娱乐
时尚,文艺,游戏动漫,美食,娱乐
娱乐,游戏动漫,军事
娱乐,时尚
游戏动漫,娱乐,美食,星座命理,时尚
吉林生活
名人明星
时尚,健康,旅游,美食
新闻趣事
揭阳生活,美食,情感生活,美图摄影,旅游
娱乐
星座命理
搞笑幽默,健康,时尚,娱乐
美食
时尚,旅游
军事,IT 数码,文艺,娱乐,体育
汽车,军事,IT 数码,文艺,娱乐
美食
娱乐,旅游,汽车
L 饭,DN 粉,吃货,睡货,咖啡控,空间控,铁塔控,动漫控
健康

坚强,历史,我就是我,简单,电脑,巨蟹女,快乐,活泼,电视
gay,网购,吃货,爱音乐,腐女,吸血鬼日记,旅游,微博奇葩,重庆生活,美食
投资理财
文艺,体育,娱乐
教育就业,IT 数码,健康,新闻资讯,娱乐
文艺,体育,娱乐
健康,体育,军事,文艺,娱乐
新闻资讯
旅游美食爱好者,艺术,烹饪,DIY,b 型血,流浪,旅游摄影,音乐达人,汽车,美食
星座命理,娱乐,文艺,爱音乐,腐女,爱美剧,多重性格,双鱼座,粗神经,喜欢音乐
游戏动漫
娱乐吉他手
时尚,旅游,IT 数码
比较内向,打篮球,学生
文艺
活泼,漫画,科技,互粉,单身,穿衣美容,旅游,新闻趣事,美食,天津生活
时尚,文艺
美食
星座命理,文艺
美女
星座命理,美食,军事,文艺,体育
娱乐,美食
遊戲動漫
美女,IT 数码,美食,旅游,健康,懒到不行的人,美术,天枰座,疯子
星座命理,美女,健康,时尚,旅游,娱乐,文艺,美食
搞笑幽默,名人明星,新闻趣事,河北生活
校园生活
娱乐
搞笑幽默,视频音乐,美图摄影,星座运势,情感生活
美食
文艺,体育
美食,游戏动漫
文艺,星座命理,旅游
交朋友,我就是我,爱自由,水瓶座,搞笑,篮球爱好者,喜欢旅游,主持,户外,团购
旅游
美食,美女,微博奇葩,旅游,视频音乐,IT 数码,游戏动漫,教育就业
视频音乐
娱乐,汽车,IT 数码
社区交友,通讯聊天,翻转课堂,情商教育,亲子教育
单身,美食控,漫画,互粉,活泼,娱乐,星座命理,时尚,健康,游戏动漫
星座命理,健康,娱乐,游戏动漫
微博奇葩,体育
旅游,娱乐,美食
游戏动漫

娱乐,文艺,旅游
IT 数码
旅游
微博奇葩,新闻趣事,名人明星,慢半拍的冷,狗鱼一家亲,冷笑话,经典台词,运动,伍佰,德国三大品牌
旅游
深圳生活,旅游,星座命理,游戏动漫,美女
体育
体育,旅游,打篮球
游戏动漫,IT 数码
美食
美食
投资理财
星座命理,旅游,名人明星,典型双子女
旅游,新闻趣事,读书分享,情感生活,校园生活
游戏动漫,文艺,体育
美女,汽车
教育就业,IT 数码
名人明星,时尚
美女,新闻资讯
健康,翻译服务,专业翻译,英语专业
北京生活
军事,文艺,美女,健康
旅游
美女,游戏动漫,时尚
健康,体育,娱乐
郑州生活
旅游,汽车,美食,新闻资讯,健康,旅游美食爱好者
购物,代购,营养素,USANA,深圳,健康
体育,时尚,旅游,美食,文艺
健康,教育就业,美食
时尚,服装设计,电影爱好者,旅游,美食
android
美食
名人明星
搞笑幽默
宠物图集,军事,新闻资讯,投资理财,教育就业,文艺
柯南,动漫,90 后
健康,汽车,旅游,军事,体育
军事,美食,投资理财,教育就业,新闻资讯,旅游,健康
搞笑幽默,星座命理,萌宠,手机,动漫控
文艺,时尚,汽车,旅游
旅游
微博奇葩,汽车,IT 数码

文艺
汽车,时尚,旅游,美食,娱乐
武汉生活,萌宠
时尚,美食,健康,旅游,文艺
旅游,健康,星座命理,文艺,娱乐
遂宁生活,娱乐
文艺
有点小宅,
电影,书籍,旅游,游戏动漫,星座命理,美食,娱乐
夜猫子
商业,房产,新闻资讯,投资理财
美食
旅游,视频音乐
搞笑幽默
美女,工作狂,写字,星座命理
时尚,汽车,旅游,娱乐,体育
时尚,投资理财,军事
其他生活
健康
爱生活,善良,坚强,微博,爱旅行,梦想,电子商务,交朋友
星座命理
娱乐,时尚
体育,教育就业
星座命理
摄影,电影控,爱自由,听音乐,喜欢旅游,书,新闻资讯,旅游
汽车,体育,美女,游戏动漫,时尚
湖北生活,文艺
星座命理
美食
汽车,时尚,健康,旅游,美食
哲学,心理学,白羊双鱼交界,单纯,星星,学习,偶稀饭睡觉觉
旅游,美食
浪漫,护肤,美容护肤,化妆,品牌,fashion,情感生活,上网,微博,旅游
穿衣美容
视频音乐,美食,旅游,上海生活,搞笑幽默
温州生活,娱乐
时尚
旅游,穿衣美容,新闻趣事,名人明星,美食,时尚,健康
视频音乐,美女
旅游
旅游
健康,旅游,时尚,游戏动漫,星座命理
工作狂
旅游,娱乐

阳光,爱幻想,画画
游戏动漫,美女,新闻资讯,军事
微笑
矛盾体,IT 互联网,视频音乐,旅游,教育就业,健康,IT 数码,新闻资讯,文艺
旅游,贵州生活,名人明星,星座命理,娱乐
音乐控,爬山,美食
新闻趣事,穿衣美容,旅游
美女,美食
美食,旅游,健康,投资理财,IT 数码
韩国生活,文艺,美女
游戏动漫
星座命理
名人明星,新闻趣事,搞笑幽默
时尚
文艺,时尚,健康,新闻资讯,IT 数码,投资理财
名人明星,旅游,美食,穿衣美容,星座命理
旅游
娱乐
学生一枚,吃货一枚,95 后,爱笑,双鱼女,懒人,文字,萌宠,美食
北京生活
动漫,美图摄影,星座运势,视频音乐,星座命理,文藝,遊戲動漫,旅遊,美食
新闻资讯,美食,投资理财
宠物图集,名人明星,美食,校园生活,南京生活
旅游,浙江生活
穿衣美容
视频音乐,时尚
新闻资讯,汽车,IT 数码
健康,旅游,文艺
搞笑幽默,广东生活,微博奇葩,美女,视频音乐
八卦杂谈,新闻趣事,视频音乐,校园生活,搞笑幽默
美食,娱乐
散步,格子控,宅男,淡定,听音乐,cosplay,萝莉控,O 型血,矛盾,音乐控
教育就业,投资理财,新闻资讯,旅游,美食
时尚
穿衣美容,娱乐
搞笑幽默
宠物图集
旅游
新闻趣事
新闻资讯,健康,文艺
美食
美食
福建生活,游戏,学生,高中生,快乐大本营,学习,育儿,校园,高考,教育
汽车,星座命理,美食,健康,旅游

旅游,美食,文艺,娱乐,学生,自由,宅,上网,睡觉,傻
娱乐,旅游,美食,星座命理
穿衣美容
星座命理
游戏动漫,美食,星座命理,健康,旅游
新闻资讯,健康,教育就业,汽车,美食
名人明星
搞笑幽默,新闻趣事,校园生活
穿衣美容,旅游,湖北生活
搞笑幽默
北京生活
重庆生活,萌宠,视频音乐,搞笑幽默,音乐,电影,旅游
视频音乐,搞笑幽默
旅游
跆拳道,武术,科幻,游戏动漫,汽车,旅游,军事,体育
新闻趣事,读书分享,美图摄影,视频音乐,星座运势
其他生活
教育就业,健康,新闻资讯
美食
健康,体育,娱乐
穿衣美容
游戏动漫,体育
微博奇葩
名人明星,体育
旅游
搞笑幽默,新闻趣事,旅游,萌宠,美女
健康,旅游,军事,文艺,体育
旅游,星座命理,美食,时尚,健康
文艺,旅游
星座命理,旅游,健康,新闻资讯
动漫,搞笑幽默
美食
时尚,教育就业,IT 数码
穿衣美容,吃货,运动,宅女,交友,幽默
名人明星,微博奇葩,搞笑幽默,穿衣美容,旅游
旅游
健康,游戏动漫,娱乐,军事,体育
旅行,时尚,数码,八卦,宅,美女,教育就业,IT 数码
数码,宅,购物,健康,教育就业,美食
萌宠
搞笑幽默
微博奇葩,视频音乐,名人明星,旅游,搞笑幽默
游戏动漫
汽车,IT 数码

广州生活,搞笑幽默,广东生活,美食
娱乐
军事
旅游,娱乐
大学,校园,白羊女
视频音乐
游戏动漫,教育就业,旅游,IT 数码
相声,地产,笑,爱旅游,美食爱好者,热爱生活,汽车,读书,山东生活
美女
萌宠
旅游
视频音乐,读书分享,美图摄影,搞笑幽默,泉州生活
名人明星,旅游,搞笑幽默,新闻趣事,微博奇葩
旅游,摄影师,文艺,游戏动漫,IT 数码
美食
星座命理,旅游,美食,穿衣美容,新闻趣事
微博奇葩,星座命理
名人明星
时尚,教育就业,星座命理,娱乐
投资理财,健康,新闻资讯,教育就业
健康,旅游,美食,魏晨,偶像剧
娱乐
爱玩,美食,娱乐
文艺,体育,娱乐
热爱生活,有机农耕,健康养生,思考,爱自由,其他生活
新闻趣事,河南生活
美食
八卦,好书,互联网,紫色
新闻趣事
读书分享,搞笑幽默,名人明星,情感生活,校园生活
名人明星,美食,星座命理,穿衣美容,视频音乐
语录,幸福,投资理财,旅游,星座命理,文艺,娱乐,新闻资讯,美女,体育
社会,喜欢唱歌,互联网,睡懒觉,健康,娱乐,IT 数码,文艺,体育
社会,微博,生活,腐女,投资理财,旅游,星座命理,时尚,汽车,IT 数码
美容服饰,时尚,文艺
穿衣美容,湖北生活,旅游,美食,视频音乐,阳光型的 90 后
社会,吃喝玩乐,语录,健康,新闻资讯,文艺,体育,娱乐
时尚
体育资讯,体育
美图摄影
旅游,美食,萌宠,星座命理,视频音乐
衰人来噶
美食,游戏动漫,IT 数码
文艺

旅游,美容服饰,星座运势,花样美男,名人明星
新闻趣事
SuperJunior,飞轮海,电脑,我就是我,音乐达人,NBA,音乐爱好者,体育,旅游,娱乐
军事
星座命理,娱乐,文艺,IT 数码,美食,时尚
美女,体育
视频音乐,新闻趣事,穿衣美容,微博奇葩,搞笑幽默
美食,时尚,健康,娱乐
美女
美图摄影,美食,美容服饰,情感生活,宠物图集
微博,单身,电视剧,旅游,星座命理,美食,健康,文艺,体育,娱乐
旅游,星座命理
新闻趣事,旅游,广东生活
美食
体育
搞笑幽默
时尚,美食,旅游,新闻资讯
旅游
美女
文艺
美食
教育就业
新闻资讯,投资理财,IT 数码
彩妆,爱美食,减肥,化妆品,瑜伽,美容护肤,射手女,时装,服装,养生
美容服饰
新闻资讯,时尚,星座命理
安静,快乐,休闲,视频音乐
体育,美女,教育就业,投资理财,汽车
美女
数学竞赛,心理学,真三 DotA,棋牌通,三国物语,什么都是略懂,生物钟混乱
IT 数码
时尚,汽车,娱乐
叶祖新,韩栋
其他生活
娱乐,健康,美女,时尚,文艺
健康,时尚,军事
时尚,旅游,健康,教育就业
星座命理
美食
旅游,多面,电影,摄影,音乐,文字,旅行,读书,安静
90 后,游戏动漫
文艺,体育,娱乐
教育就业
星座命理

美图摄影,视频音乐,校园生活,搞笑幽默,名人明星
健康
美食,旅游,娱乐
游戏动漫
汕头生活,GCT 辅导,公务员辅导,汕大 MPA 培训,汕大 MBA 培训
做梦,郭敬明,巧克力,时尚,文艺,旅游,星座命理
萌宠
北京生活
旅游
时尚,美女,体育,旅游,娱乐
音乐控,喜欢音乐,微博控,青春,游戏,吃货,幽默,偶喜欢玩,微博,校园生活
新闻趣事,视频音乐,体育,美女,游戏动漫,娱乐
搞笑幽默
星座命理,美女,游戏动漫
工作狂,医学,健康,IT 数码
汽车,体育,IT 数码
新闻资讯
IT 数码,新闻资讯,旅游,体育
娱乐
美食,美食,旅游,娱乐
视频音乐,星座命理,达州生活
美食
天蝎女,麦霸,喜欢音乐,懒人,教育就业,旅游,娱乐,笑话,音乐控
汽车,美女,旅游,IT 数码
视频音乐
新闻资讯,健康,旅游,文艺
旅游
名人明星
旅游,文艺,体育,娱乐,IT 数码
搞笑幽默
娱乐
音乐控,军事,唱歌,跑步,赚钱,看小说,奋斗,汽车,旅游美食爱好者,IT 数码
旅游
微博奇葩
美女,旅游,健康,投资理财,汽车
美食,旅游,汽车,投资理财,体育
健康,新闻资讯,游戏动漫,教育就业,军事
巨蟹,旅游爱好者,吃货
美食,时尚,投资理财,旅游,健康
IT 数码,旅游,搞笑幽默,校园生活,美女
时尚,旅游,美食,文艺,娱乐
教育就业
体育,汽车,旅游,娱乐,游戏动漫
娱乐,健康,教育就业

美女,天津生活,美食
旅游,美容服饰,宠物图集,美食,搞笑幽默
微博奇葩,视频音乐,名人明星,搞笑幽默,美女,网上兼职,网上购物,网上创业
美食,娱乐
旅游
小说迷,自恋,旅游,文艺
天蝎座,工作狂,唱歌,摄影,喜欢旅游,时尚潮流,连接服务器失败,睡觉睡到自然醒,摩羯,星座命理
美女,教育就业,汽车,体育,IT 数码
教育就业,娱乐,文艺
90 後,单身,宅女,看电视,涂鸦
时尚潮流,时尚达人,服装,服饰,潮人,欧美控,完美主义,时装,购物
健康
美食,体育,娱乐,游戏动漫
教育就业,投资理财,美女,星座命理,IT 数码
旅游,穿衣美容,微博奇葩,星座命理,搞笑幽默
游戏动漫,时尚,IT 数码
新闻趣事,星座命理,搞笑幽默,名人明星,河北生活,梦想,狮子座,听音乐,小脾气
教育就业,投资理财,旅游
健康,文艺,旅游,星座命理,美食
娱乐
手机控,吐槽帝,人不中二枉少年,真相帝,动漫控,文艺
吃货,宅女,交友,乐观
军事
时尚,健康,旅游,星座命理,美食
旅游,星座命理,美食
美女
IT 数码,军事,汽车,旅游,健康,体育
健康,文艺,娱乐
健康,娱乐
娱乐
搞笑幽默,囧,冷笑话,连接服务器失败,体育
旅游
美食
娱乐,旅游
美女,时尚,汽车,文艺,娱乐
微博奇葩,旅游,搞笑幽默,新闻趣事,视频音乐
时尚,les,音乐,篮球,90 后,美术,双子座
逛街,销售,萝莉控,爱旅行,房产,减肥,职场招聘,喜欢音乐,吸血鬼日记,时尚
美食,文艺,时尚
旅游,时尚,星座命理,文艺,娱乐,起床困难户,上班族,乐天派
军事,体育,文艺,时尚,娱乐
星座命理,娱乐,投资理财,时尚,健康
视频音乐

火影,美食
投资理财,旅游,美食,文艺
健康,教育就业,时尚,旅游,星座命理,投资理财,新闻资讯,娱乐
时尚
其他生活
其他生活
天秤座,音乐,阅读
星座命理,娱乐,时尚,旅游
美食,视频音乐,新闻趣事,广东生活,搞笑幽默
军事
幽默,影视,打篮球,旅游爱好者,寂寞,驴友
旅游,时尚,文艺,体育,娱乐
闷骚男,摩羯座,互粉,健康
搞笑幽默,旅游,微博奇葩
时尚,星座命理,健康,娱乐,文艺
流浪
时尚
旅游,星座命理,健康,教育就业,投资理财
搞笑幽默,新闻趣事,微博奇葩,体育
文艺
微博奇葩,搞笑幽默,汽车,军事,娱乐
穿衣美容
美女,旅游,文艺
微博奇葩
新闻资讯,时尚,旅游,美食,娱乐
美容,时尚,投资理财,旅游,美食,娱乐
微博奇葩,美食,旅游,娱乐,时尚
投资理财
穿衣美容
美女,军事,汽车,体育
IT 数码,美食,游戏动漫,新闻资讯,投资理财,动漫,睡觉,学生
军事,体育,投资理财,新闻资讯,时尚,朝阳新玛特
IT 数码
搞笑幽默
微博奇葩
娱乐,文艺
教育就业,健康,星座命理,时尚,新闻资讯
电子厂,电子
校园生活,娱乐
旅游
合肥生活,体育
摄影,喜欢听歌,爱电影,记者,健康养生,90 后但我不脑残,搞笑幽默,校园生活,视频音乐,美图摄影
健康,投资理财,IT 数码

名人明星,新闻趣事,美图摄影,视频音乐,娱乐,时尚,文艺
文艺
美女
美女
天秤座,游戏动漫
旅游
搞笑幽默,IT 数码
海口生活
旅游
健康,汽车,美食
搞笑幽默
娱乐
娱乐
旅游,健康,时尚
美食,时尚
搞笑幽默,美女
汽车,文艺
旅游,星座命理,美食
福建生活
文艺,时尚,投资理财,IT 数码,旅游
笑,巨蟹女,宅,自由,旅行
娱乐
穿衣美容,时尚
娱乐
健康
游戏动漫,投资理财,IT 数码
美女
星座命理,美食,穿衣美容,旅游
视频音乐
旅游
心理,情感,美食,时尚
宠物图集,读书分享,美食
搞笑幽默,名人明星
美食,广东生活
美女
萌宠,時尚
星座命理
穿衣美容,美食,搞笑幽默,视频音乐,名人明星
健康,美食,文艺
时尚,文艺,娱乐
听音乐
娱乐
游戏动漫,投资理财,军事
IT 数码,名人明星

IT 数码
星座命理
新闻资讯,时尚,投资理财,教育就业,旅游
搞笑幽默,娱乐
健康
腐,cosplay,格子控,宅,学生,动漫
娱乐
体育
游戏动漫,投资理财,IT 数码
军事,娱乐
健康,旅游,美食,投资理财,文艺,娱乐
宠物图集
旅游,美食,军事,文艺,体育
文艺,美食,健康,旅游,娱乐
穿衣美容
新闻趣事
美女,娱乐
打球,看电影
穿衣美容,视频音乐,美女,名人明星,旅游
星座命理,美食,体育,娱乐
懒,爱笑,爱电影,发呆,股市,文艺,美食,游戏动漫,旅游,投资理财
死爱面子,悲观主义者,帆布鞋控,KRISing,外冷内热,看小说,EXO,摩羯座,90 后非脑残
视频音乐,旅游
旅游
美女
美图摄影,情感生活,星座运势,视频音乐,宠物图集,美容服饰,IT 数码,八卦杂谈,名人明星,新闻趣事
文艺
健康,美女,汽车,旅游,体育
美食,旅游
娱乐
爱睡觉,娱乐,美食,旅游,时尚
揭阳生活,美女,旅游,星座命理,军事,投资理财
拉萨,西藏旅游,音乐 mv,微电影,写真,婚纱,西藏摄影
懒人,爱唱歌,球迷,篮球,个性
体育
星座命理,穿衣美容
搞笑幽默
旅游
星座命理
八卦杂谈,星座命理
美女,健康
美食
视频音乐,体育

驴友,个性,自由,学生
汽车,教育就业,新闻资讯
娱乐,健康,教育就业,旅游,美食
星座命理
美容服饰
郭敬明,睡觉,旅游,学生
新闻资讯,游戏动漫,教育就业,娱乐,体育
文艺,美食,星座命理,时尚
健康,美食,汽车,旅游,体育
湖南生活
汽车,美女,健康
日本生活
江西生活
时尚,健康,旅游,星座命理,娱乐
汽车
游戏动漫
开朗又内向
新闻趣事
旅游
视频音乐,旅游,健康,时尚,娱乐,星座命理
军事,美食,旅游,体育,娱乐
美女,视频音乐
瑜伽,美食,爱音乐,微笑,旅游爱好者,宅女一枚
视频音乐,美食,旅游
穿衣美容,名人明星,微博奇葩,新闻趣事,搞笑幽默
投资理财,汽车,IT 数码,体育
吃喝拉撒
时尚,美女,文艺,娱乐,星座命理
娱乐
美食
星座命理,微博奇葩,搞笑幽默,穿衣美容,新闻趣事,健康
狮子座,微笑,喜欢音乐,开心,互粉,自信,胡思乱想,健康,文艺,星座命理
名人明星,旅游,美食,穿衣美容,视频音乐
名人明星
游戏动漫,投资理财,IT 数码
漢語學習中,电视剧,台灣,可爱,电影,刘诗诗,美女,步步惊心,美食,日本
名人明星
名人明星,搞笑幽默,时尚,娱乐
星座命理,美食,娱乐,游戏动漫,IT 数码
萌宠,江苏生活
名人明星,美食,搞笑幽默
旅游,IT 数码,体育
娱乐,美女,时尚,美食
旅游,视频音乐,美食

新闻趣事,广州生活,搞笑幽默,美女,星座命理,投资理财
投资理财,旅游
校园生活
娱乐
IT 数码
蚁巢制作,宠蚁,娱乐,美食
健康,星座命理,文艺
微博奇葩,穿衣美容,投资理财,星座命理,旅游,时尚,汽车
浪漫,IT 数码,影视,旅行,幸福,流行,科技,快乐,时尚,互联网
旅游,新闻资讯
微博奇葩,搞笑幽默,新闻趣事,星座命理,美食
广东生活,学生一枚,娱乐
广州生活
搞笑幽默
微博奇葩,星座命理,美食,名人明星,视频音乐,汽车,游戏动漫
体育
文艺
搞笑幽默
视频音乐,健康
微博奇葩,星座命理,穿衣美容,美女
旅游,新闻资讯,娱乐,美食,体育
旅游,视频音乐,旅游美食爱好者,服装设计,时尚,文艺,健康
美食,健康
娱乐
名人明星,视频音乐,娱乐,旅游,美食,体育
美女
星座命理,游戏动漫,娱乐
布丁,疯狂,时尚
白羊座,真诚,k 歌,逛街,看电影,活泼,娱乐
设计,成都,会计,时尚达人,淡定,电影爱好者,房地产,健身,汽车
时尚
时尚,军事,体育
美食,娱乐
时尚,军事
教育就业,娱乐,投资理财,健康,美食
健康,新闻资讯,教育就业,旅游,文艺
游戏动漫,旅游,娱乐
美女,搞笑幽默
教育就业,体育,文艺,IT 数码,军事
娱乐,时尚,旅游,文艺
IT 数码,游戏动漫,娱乐
旅游
文艺
文艺,汽车,娱乐

主持人,天秤座,小清新,大学生,爱幻想,旅游摄影,阅读,乐观,努力,音樂
旅游
旅游,时尚,星座命理,娱乐
旅游
其他生活
军事,体育
美食
汽车
旅游
美食,游戏动漫
新闻趣事,视频音乐,美食,旅游,安徽生活
娱乐,星座命理
游戏动漫
美食,微博奇葩,视频音乐
游戏动漫,教育就业,星座命理
搞笑幽默,汽车
旅游
旅游,美食,天枰座,80 后
时尚,健康,IT 数码,星座命理
美女
游戏动漫,投资理财,星座命理,美女,IT 数码,文艺
汽车,IT 数码
汽车,教育就业,体育,旅游,新闻资讯
交朋友,爱旅行,庚饭
游戏动漫
体育,星座命理,美食,文艺,娱乐
游戏动漫,娱乐,健康,IT 数码,体育
旅游,星座命理,健康
拍照
萌宠,穿衣美容,星座命理,美女,视频音乐,搞笑幽默,娱乐
美食
娱乐,新闻资讯,星座命理
时尚
星座命理,美食,旅游,萌宠
娱乐
美食
微博奇葩,美女,视频音乐,新闻趣事,搞笑幽默
美女,视频音乐
文艺
投资理财,军事,IT 数码
游戏动漫
北京生活,工会,地理信息,测绘
旅游,美食,投资理财,时尚,健康
旅游

旅游
美女,教育就业,旅游
娱乐,文艺,游戏动漫,星座命理
娱乐
邯郸生活,美女,搞笑幽默,爱逗喵星人
教育就业,体育,美女,星座命理,文艺
湖北生活
文艺,美食,时尚,旅游
文艺,健康,星座命理,时尚,娱乐
旅游,美食
美容服饰,旅游,星座运势,美食,芜湖生活
商丘生活,美图摄影,视频音乐,IT 数码,旅游,文艺,娱乐
健康
文艺,旅游,美食
旅游
教育就业
IT 数码
新闻资讯,投资理财,时尚,汽车,旅游
视频音乐,美食,旅游,穿衣美容,书籍,音乐达人,育儿,快乐,美食控,健康
健康,旅游,美食
投资理财,文艺,娱乐
音乐,唱歌,影视,交朋友,热爱生活,爱幻想,学生一枚
搞笑幽默,动漫,宠物图集
美女
新闻资讯
cosplay,动漫控,魔羯座,it 互联网,迷糊,美女
新闻趣事
教育就业
汽车
投资理财,教育就业,旅游,美食,体育
搞笑幽默
视频音乐,娱乐
体育资讯,美图摄影,视频音乐,杭州生活
旅游,南京,睡觉睡到自然醒,90 后,爱自由,猫,摄影爱好者,喜欢旅游,爱,怀旧
懒,摩羯,自由,健康
旅游,美食,娱乐
搞笑幽默,名人明星,美女,体育资讯,视频音乐,这是神马东东？？,美食,时尚,娱乐,体育
健康
干物女,不受控制的體重,黑夜守护者,日本国,个高腿长易绊倒,有毛哺乳类生物
旅游,名人明星,视频音乐,微博奇葩,星座命理
名人明星
一对一,外教,英语培训,托福英语,雅思英语,商务英语,英语口语,eLearning,厦门,在线英语
美食,旅游,星座命理,娱乐
体育,汽车,游戏动漫

新闻资讯,旅游,教育就业,健康,娱乐
徐州生活
金牛座,瘦身,迷糊,海绵宝宝,小女人,双重性格,睡懒觉,微博,青春
美女
新闻趣事,搞笑幽默
新闻资讯,教育就业,星座命理,旅游,娱乐
投资理财,体育,军事,IT 数码,游戏动漫
游戏动漫,投资理财,军事
穿衣美容
新闻资讯
娱乐
美食,星座命理,旅游
工作,时装
新闻资讯,游戏动漫,娱乐,文艺,体育
旅游,美食,白羊座,贫乳控,文艺,星座命理
新闻趣事,投资理财,新闻资讯
健康,IT 数码
投资理财,文艺,军事,新闻资讯,教育就业
搞笑幽默,名人明星
旅游,美图摄影,梅州生活,IT 数码,穿衣美容,篮球爱好者,艺术
拍照,旅游控，Music
微博奇葩,萌宠,美食,旅游
搞笑幽默,体育,偷吃米
体育,娱乐
朱丹控,美食
投资理财
文艺
旅游
旅游,星座命理
教育就业,星座命理,旅游,文艺,时尚
汽车,体育,双鱼座,看电影,海贼王,火影,篮球,动漫,热爱生活,美女
微博奇葩
B 型血,滑雪,小提琴
体育
旅游
微博奇葩,萌宠
自由,努力,美女
时尚
旅游,萌宠,新闻趣事,视频音乐,微博奇葩
时尚
乐观,吃,天秤座,娱乐
汽车
旅游,微博奇葩,搞笑幽默,萌宠,美女,体育
体育

时尚,娱乐,旅游
旅游
星座命理,教育就业
美女,健康,体育,娱乐,旅游
情感生活,健康,文艺
旅游,美食,时尚,汽车
美食
旅游,文艺
体育
其他生活
视频音乐
搞笑幽默
美女,美食
投资理财,健康
新闻资讯,文艺,娱乐,教育就业,健康,美食,军事
旅游
新闻资讯,汽车,旅游,体育
名人明星,美食,其他生活
时尚,美食,IT 数码,文艺,娱乐
旅游,军事,IT 数码
文艺,体育,娱乐
广东生活,摄影,IT 數碼
搞笑幽默,微博奇葩,减肥,外貌协会 VIP,腐女,爱运动,爱动漫,爱宅,学生
美食
萌宠
娱乐
微博奇葩,文艺
健康,新闻资讯,娱乐
澳大利亚生活,星座运势,旅游,美图摄影,海外生活
汽车,旅游,IT 数码,美食,文艺
新闻趣事,视频音乐,汽车,新闻资讯,娱乐
汽车
穿衣美容
娱乐
旅游,娱乐
文艺,体育,娱乐
搞笑幽默
星座命理,美食
视频音乐,校园生活,搞笑幽默,其他生活,美女
娱乐
名人明星
旅游,投资理财,新闻资讯,体育
微博奇葩,新闻趣事,广东生活,穿衣美容
文艺,体育,娱乐

穿衣美容,娱乐
美食
微博奇葩
军事,娱乐,汽车,教育就业,健康,旅游,星座命理
投资理财
萌宠
美食,军事,IT 数码
美食,穿衣美容,星座命理,搞笑幽默
教育就业,娱乐,游戏动漫
旅游
动漫迷,媒体,夜猫子,看小说,工作,吉他,旅游美食
动漫迷,健康,小资,新闻资讯,旅游,IT 数码,购物狂,爱生活,微博控,旅游美食
体育,IT 数码,文艺,游戏动漫
凉山生活,美食,旅游,娱乐
职场招聘,汽车,新闻资讯,旅游,文艺,体育
搞笑幽默,军事,旅游,体育
教育就业,旅游,军事,健康,IT 数码,汽车,美食,文艺
时尚,军事,美女,投资理财,IT 数码
IT 数码,时尚,娱乐
教育就业,死因鉴定,亲子鉴定,遗体捐献,学霸,法医学,醫學生
文艺
旅游,美食,游戏动漫
军事
美食,视频音乐
IT 数码,旅游,游戏动漫,娱乐,体育
旅游,视频音乐,校园生活
汽车,旅游,美食,健康,美女
星座命理
搞笑幽默,游戏动漫
名人明星,搞笑幽默
穿衣美容
视频音乐,美女,投资理财,时尚,娱乐,IT 数码
名人明星
旅游,健康,体育
旅游,搞笑幽默
微博奇葩,旅游,名人明星,视频音乐,北京生活
美食,娱乐
娱乐
校园,搞怪,曼联
美食,投资理财
旅游,微博奇葩,新闻趣事,穿衣美容,星座命理,教育就业,新闻资讯,IT 数码,文艺
新闻资讯
旅游,汽车,体育
旅游

这是小号,高冷,禁勾搭
星座命理
星座命理,时尚,美女
旅游
新闻趣事
美图摄影,宠物图集,星座命理,文艺,时尚,旅游,娱乐,美食
旅游,娱乐,投资理财,游戏动漫,体育
穿衣美容,星座命理,教育就业,旅游
时尚,美食
旅游,新闻资讯,教育就业,文艺,健康
名人明星
桂林生活,名人明星,健康,我不曾爱过
新闻资讯,IT 数码,体育,汽车,旅游
微博奇葩
旅游,美食
穿衣美容,时尚
投资理财,旅游,星座命理
新闻趣事,旅游,美食,北京生活
教育就业
星座命理,美女
星座命理
穿衣美容,名人明星,新闻趣事,搞笑幽默
娱乐,文艺
摄影,爱猫,爱狗,动漫,爱旅游,小说
投资理财,旅游
健康,文艺,新闻资讯,旅游,美食,IT 数码,娱乐
泉州生活
IT 数码,投资理财,新闻资讯,娱乐
旅游
美食
汽车,教育就业,美女,文艺,军事,旅游,健康,爬山,简单,养生
文艺
爱笑,90 後,跳舞,爱电影,爱旅游,女人,猫咪控,潮人,小清新,美食
车世界
星座命理,美食,旅游,穿衣美容
文艺
时尚,旅游,文艺,娱乐,美食
美女,旅游,娱乐,军事,汽车
新闻趣事,旅游
旅游,体育,投资理财,健康,教育就业
名人明星,娱乐
陈奕迅
健康
美食,名人明星,其他生活

文艺,视频音乐
文字控与自由控,选择恐惧症患者,萌物控,低调饭,腹黑 F 女,90 後,水瓶女
视频音乐
旅游
穿衣美容
搞笑幽默,军事,文艺
投资理财,时尚,健康,旅游,文艺
wow,桌游爱好者,游戏,美食,娱乐
旅游
投资理财,健康,时尚,娱乐
旅游
体育资讯,其他生活
开心,80 后的尾巴,喜欢音乐,健康,时尚,美食,旅游,娱乐
星座命理
搞笑幽默,美女
军事
体育,汽车
穿衣美容
旅游,吃货一枚,音乐控,摄影控,电影,旅行,潮流,时尚
巨蟹座,音乐,军事
游戏动漫
体育资讯
时尚,娱乐
美食
文艺,体育,娱乐
娱乐,安静的做自己,缺父爱,最喜欢泡泡堂,宅女,吃货一枚,偶像剧控
内蒙古生活,健康
IT 数码
旅游,微博奇葩,穿衣美容,星座命理
教育就业
名人明星,旅游,新闻趣事,视频音乐,军事,娱乐
美食,新闻趣事,娱乐,星座命理
名人明星,美食,旅游
穿衣美容,星座命理,美食
美食,体育
新闻趣事,搞笑幽默,娱乐,美食,健康,游戏动漫,美女
新闻趣事,搞笑幽默,微博奇葩,穿衣美容,星座命理
旅游
教育就业,健康
校园生活,视频音乐,新闻趣事,美女,搞笑幽默
美食,娱乐
新闻资讯,健康,旅游,星座命理,美食
美食
投资理财,爱吃,睡觉睡到自然醒,影视,新闻资讯

美图摄影,校园生活,新闻趣事,旅游,佛山生活
香港生活
美女,名人明星,广东生活,娱乐
美食,娱乐,文艺,美女
娱乐,星座命理,文艺,体育,军事,看电影,游泳
书,汽车,时尚,旅游,美食,IT 数码
旅游,河北生活,搞笑幽默,名人明星,视频音乐
美女,爱好篮球
IT 数码,娱乐
美容服饰,星座运势,名人明星,情感生活,校园生活
美女,汽车,旅游,美食,军事
教育就业,投资理财,星座命理,旅游,美食
娱乐
旅游
视频音乐
搞笑幽默,星座运势,美容服饰,旅游,动漫,星座命理,新闻资讯,教育就业
销售与市场,微博控,传媒,媒体,写作,时尚,汽车,投资理财,旅游,星座命理
汽车,美女
名人明星
江西生活
名人明星,微博奇葩,穿衣美容,新闻趣事,搞笑幽默
投资理财,娱乐,新闻资讯,健康,时尚
甘肃生活,新闻资讯
旅游,时尚,汽车,美女,娱乐
视频音乐,穿衣美容,美食,萌宠,旅游,新闻资讯,健康,游戏动漫,IT 数码
游戏动漫,健康,娱乐,体育,汽车
新闻趣事
电视剧,学生,爱美食,美食控,吃货一枚,支持唐峥艳,00 后,开心,可爱
时尚,美食,娱乐
娱乐,新闻资讯,美女,军事,IT 数码,文艺
IT 数码,汽车
发财梦
时尚
游戏动漫
娱乐,美女
旅游
名人明星
旅游
旅游,星座命理,美食
时尚
旅游,美食,IT 数码,投资理财,娱乐
时尚
星座命理,文艺,体育,刘德华,巴萨,摩羯座
美食,美女,文艺,娱乐,健康

星座命理
游戏动漫, 娱乐, 美食, 旅游, IT 数码
旅游, 军事
汽车
体育, IT 数码, 美食, 娱乐, 旅游
旅游
旅游, 微博奇葩, 美女, 搞笑幽默, 视频音乐
穿衣美容
视频音乐
四川生活, 健康
八卦杂谈, 校园生活, 视频音乐, 体育资讯, 美女, 体育
视频音乐, 旅游, 美食
智能手机, 手机, 娱乐
时尚, 鞋子控, 萝莉, 听音乐, 娱乐
手机壳, 娱乐
文艺, 投资理财
美食
时尚
新闻资讯
旅游, 萌宠, 视频音乐, 新闻资讯, 时尚, 文艺, 娱乐
旅游, 美食, 文艺, 娱乐, 时尚
搞笑幽默, 广东生活, 名人明星, 美女
体育, 娱乐, 投资理财, 教育就业, 新闻资讯
穿衣美容
体育
娱乐, 健康, 美食, 美女, 星座命理
娱乐, 游戏动漫, 美女, 文艺, 时尚
旅游
美食, 名人明星, 视频音乐, 江苏生活, 体育
大叔控, 狮子座, 文字
IT 数码, 美女, 健康, 旅游, 美食, 时尚
上海生活, 文字, 安徽生活, 情感, 幸福, 自然, 微笑, 美食, 健康
旅游, 教育就业
名人明星, 视频音乐
新闻趣事, 美食, 星座命理, 搞笑幽默, 广东生活
新闻资讯, 时尚, 投资理财, 星座命理, 娱乐, 90 后, 要结婚, 要出柜, 懒答应
健康, 时尚, 娱乐
健康, 时尚, 美食, 娱乐
投资理财
军事, IT 数码, 时尚, 汽车, 文艺
美食, 宠物图集, 搞笑幽默, IT 数码, 旅游, 健康, 娱乐
美食, 新闻资讯
文艺, 娱乐
投资理财, 旅游, 美食

读书分享,美食,新闻趣事,搞笑幽默,文艺,旅游,新闻资讯,教育就业
美女
IT 数码,娱乐,iphone,快乐大本营,健康,时尚,旅游,美食,星座命理
萌宠,娱乐
美食,投资理财,文艺,体育
活泼,阳光,电影爱好者
旅游,星座命理,时尚
教育就业,旅游,时尚,文艺,娱乐
娱乐
萌宠
浙江生活,新闻趣事,微博奇葩,娱乐,IT 数码,文艺
游戏动漫,文艺
微博奇葩,时尚
旅游
旅游
宗教,LES,80 后,佛,佛具,佛珠,北京生活
宠物图集,美食
旅游摄影,拍照,旅游,搞笑幽默
江西生活
新闻资讯,投资理财,教育就业,时尚,美食,健康
汽车
视频音乐,新闻趣事,旅游
军事,娱乐,文艺
投资理财,IT 数码,军事,旅游,星座命理
旅游,娱乐
旅游,时尚,美食,娱乐
旅游,IT 数码,娱乐
新闻资讯,时尚
英国生活,教育就业
旅游
手机控,喵星人,美食,海贼王,睡觉睡到自然醒,吃货,淘宝控
衣橱,娱乐
穿衣美容
体育
文艺,旅游,娱乐,美食
视频音乐,搞笑幽默
文艺,新闻资讯,教育就业,投资理财,军事
名人明星
新闻资讯,IT 数码
穿衣美容
穿衣美容,名人明星
视频音乐,新闻趣事,汽车,健康,美食,时尚,投资理财,娱乐,文艺
健康,娱乐
美食

美食,旅游,穿衣美容,搞笑幽默,厦门生活
低调
科学探索
旅游,IT 数码,军事,游戏动漫,体育
新闻趣事,穿衣美容,星座命理,美食,旅游
上海生活,视频音乐,名人明星,旅游,美食
美图摄影,美食,旅游
校园生活,名人明星
娱乐,自恋,小清新,生活,整理,收藏,瓶子控,杯子控,本子控,杂货铺
教育就业,文艺
文艺
健康,旅游,美食,星座命理,娱乐
娱乐
美食
旅游,文艺,教育就业,新闻资讯,健康,军事
体育,汽车,健康,军事,旅游,娱乐,文艺
电影爱好者
美女,爱自由
时尚
搞笑幽默,星座命理
搞笑幽默,娱乐,新闻资讯
军事,IT 数码,文艺,体育,娱乐
健康,旅游,美食,IT 数码,娱乐
旅游,微博奇葩,星座命理,穿衣美容,搞笑幽默
湖南生活
娱乐,健康,美食,时尚,文艺
旅游,娱乐,教育就业,时尚,文艺,体育
逛街,购物,眼镜,丝巾,娱乐
娱乐,汽车,旅游,体育
成都生活,投资理财,健康,美食
文艺
汽车,投资理财,健康,新闻资讯,旅游
上海,意大利,美国,娱乐
英伦风,娱乐
旅游
新闻资讯,星座命理,娱乐,睡懒觉,90 后但我不脑残
投资理财,汽车,新闻资讯,旅游,娱乐
自主创业,民主自由,创造财富,教育就业
鬼故事,逆战
游戏动漫,美食
搞笑幽默
微博奇葩,高中生,音乐爱好者,任性,巧克力,做梦
教育就业,娱乐,投资理财,健康,旅游,星座命理,美食
时尚,星座命理,健康,美食,旅游

搞笑幽默
爱自由,欧美控,金牛座,敏感,看小说,陈奕迅,吃货,萌宠,视频音乐,新闻趣事
星座命理,名人明星,旅游,新闻趣事,萌宠
北京万国医院,北京万国白癜风,白癜风治疗,白癜风治疗费用,白癜风治疗多久,白癜风治疗药物,北京白癜风专科,北京白癜风专家,北京白癜风,穿衣美容
文艺,新闻资讯,军事,娱乐
lol,鸭梨
思考,乐天派,偶稀饭睡觉觉
音乐控,文学,好书
视频音乐
萌宠,美食,旅游,南京生活,时尚,紫晶环球,大厂,女装
娱乐
文艺,投资理财,汽车,健康,旅游
视频音乐,游戏动漫,文艺,体育
星座命理,娱乐
娱乐
名人明星,旅游,美食,萌宠,星座命理
教育就业,新闻资讯
教育就业,健康,旅游,新闻资讯,投资理财
健康,美食,文艺,旅游,时尚,教育就业,投资理财,娱乐
福建生活
军事,美女,游戏动漫,汽车,文艺,体育
旅游,美食,广西生活
生活大爆炸,心理学,怀旧,90,单纯,新闻趣事,美女,名人明星,旅游
美食,搞笑幽默,爱自由,湖北生活,旅游,微博奇葩
教育就业
旅游,汉语桥,中华情,今日亚洲,主持人,CCTV4
吉林生活,健康
爱旅游,爱电影,新闻趣事,美食,搞笑幽默,穿衣美容
新闻趣事,健康
杨宗纬,点儿,鴿子,海泉,羽凡,白百合,羽泉迷,陈奕迅
咖啡控
美食,旅游
健康
美食,旅游,游戏动漫
上海生活
旅游
投资理财,汽车,旅游,军事,体育
体育
美食
美国生活,巧克力,90后,纯音乐钢琴曲,插画,双鱼座,文字,教育就业,文艺
北京生活,游戏动漫,时尚,投资理财,娱乐,美食
时尚,游戏动漫,汽车,教育就业,投资理财,美女
佛教,军事,投资理财,星座命理,汽车,游戏动漫

军事
旅游,星座命理,文艺,娱乐,美食
娱乐
IT 数码,时尚,健康,美女
IT 数码
旅游
体育资讯,计算机,90 后但我不脑残,英语,军事,DIY,汽车
穿衣美容,萌宠,美食
美女,汽车
文艺,美女,游戏动漫,健康,星座命理,美食,娱乐
文艺
IT 数码,美食
体育
健康,新闻资讯,旅游,军事,娱乐
新闻资讯,文艺,军事,游戏动漫,旅游,体育,教育就业
娱乐
娱乐
时尚,开心,80 后的尾巴,销售,淘宝控,喜欢音乐,美女,投资理财,育儿,广州
星座运势
新闻资讯,旅游,文艺
新闻资讯,娱乐
时尚,汽车,体育,娱乐
新闻资讯,文艺,投资理财,教育就业
IT 数码,军事,美食,健康,新闻资讯
旅游,汽车
娱乐,时尚
美食
游戏动漫
时尚,健康,娱乐,星座命理
视频音乐
旅游
健康
科学探索,读书分享,美容服饰,旅游,美食
美食,IT 数码,体育
体育,美食
美女,浙江生活
旅游,美食
搞笑幽默,微博奇葩
视频音乐,教育就业
美食,文艺,娱乐
教育就业,体育,娱乐
娱乐
美食,旅游,茶,吃货一枚
穿衣美容

云南生活,好书,活泼开朗,旅游美食爱好者
旅游,新闻资讯,IT 数码,军事,娱乐
小清新,K 歌
名人明星,微博奇葩,视频音乐,搞笑幽默,黑龙江生活,多愁善感
新闻趣事
北京生活
名人明星,娱乐
体育
美女,投资理财
美女,搞笑幽默,星座命理,视频音乐,穿衣美容
教育就业,健康,美食,旅游
美女,新闻趣事,搞笑幽默
教育就业
娱乐,时尚,美食,教育就业,健康
80 后,网购,狮子座,宅男,医学,臭美,购物狂,爱幻想,时尚潮流,闷骚
fashion,Taobao
星座命理
健康
美食
搞怪,宅女,幽默,学生一枚,90 后但我不脑残,K 歌,旅游,娱乐,美食
sai 党,小说推荐,耽美,动漫迷,听歌向,广播剧,古风,大学生
娱乐
美食,军事,娱乐,旅游,健康
微博奇葩,搞笑幽默
健康,文艺,旅游,美食
宅男,肌肉,帅哥
搞基,体育,帅哥
美女,美食,文艺,搞基,宅男,gay,帅哥
美食,视频音乐,旅游,微博奇葩
美容服饰,美女,穿衣美容
旅游
动漫,读书分享,视频音乐,美容服饰,军事,汽车,时尚,旅游,体育
名人明星,视频音乐,新闻趣事,搞笑幽默,美女,汽车,投资理财,娱乐,体育
旅游
新闻趣事,萌宠,图论,小动物
时尚,健康,旅游,美食
文艺
体育
健康,旅游
穿衣美容,旅游,美食,新闻趣事,辽宁生活
旅游,美食,视频音乐,北京生活
搞笑幽默,内蒙古生活
微博奇葩,搞笑幽默
新闻趣事

视频音乐
购物,郭敬明,建筑,苹果,留学,冷笑话,法律,执着,投资
搞笑幽默
微博奇葩
萌宠
美女,文艺
外向
娱乐
美女
视频音乐,旅游
体育资讯,体育
金牛座,爱睡觉
星座命理
宅女,交友,唱歌,电视剧,K歌,散步,漫画,活泼,海绵宝宝,美食
旅游,娱乐
新闻趣事,旅游,视频音乐,星座命理,穿衣美容
穿衣美容
旅游,微博奇葩
体育,教育就业,星座命理,美食
体育,娱乐,健康,新闻资讯,美女
健康,高三,微笑,地产,护肤,医生,咖啡控,教育就业
搞笑幽默
娱乐,旅游
科技,APP,应用,搞笑幽默
IT数码,旅游,投资理财,游戏动漫
宅,时尚,美食,听歌,90后,投资理财,游戏动漫,美女,IT数码,娱乐
搞笑幽默,军事
IT数码,新闻资讯,体育
旅游,视频音乐
搞笑幽默,视频音乐,穿衣美容,新闻趣事,星座命理
视频音乐,时尚
旅游,军事,游戏动漫
穿衣美容
视频音乐,名人明星,美食,萌宠,星座命理
微博奇葩
其他生活
娱乐
星座命理,其他生活,美食,搞笑幽默,新闻趣事
星座命理
江苏生活
品牌,网络营销,管理,五月天,Android,金牛
健康,旅游,文艺,体育,gay,肌肉
小清新,感性,天蝎,教育,可爱,微博,阅读,娱乐
旅游,美食,投资理财,健康,时尚

文艺,娱乐,体育,名人明星,星座命理,旅游,视频音乐,搞笑幽默
美食
投资理财,新闻资讯,IT 数码,教育就业,旅游,文艺
茶痴,旅游,健康
军事
新闻资讯,星座命理,时尚,教育就业
新闻趣事
名人明星
逛街,购物,眼镜,娱乐
北京,上海,意大利,娱乐
上海,意大利,娱乐
名人明星
逛街,购物,眼镜,娱乐
邱胜翊粉丝
90,时尚
投资理财,教育就业,体育,文艺,健康,新闻资讯
名人明星
莆田生活
新闻趣事
音乐爱好者,文艺,外贸,天津生活
时尚
旅行,时尚,数码,八卦,购物,游戏动漫,旅游,IT 数码
摄影,八卦,宅,购物,时尚,旅游,体育
汽车,美食,IT 数码
IT 数码,文艺,体育
旅游,体育,娱乐
健康
青海生活,白羊座,个性,动画,真诚,青春
拍照,搞笑幽默,山东生活,汽车,IT 数码,娱乐
奋斗,神经质,音乐控,90 後,爱自由,梦想,爱玩,冷笑话,大学
娱乐,健康,美食,星座命理,旅游
我就是我,旅游,时尚,健康,美食,摩羯座
新闻趣事,旅游,视频音乐
萌宠
新闻趣事
新闻资讯,旅游,IT 数码
小清新,手机,校园,爱自由,散步,美食,体育
篮球 NBA,体育篮球,名人明星,视频音乐,旅游,美食
美女,正在奋斗的青年
平面设计,韩国,宠物,时尚潮流,天蝎女,美术,文艺
文艺
旅游,视频音乐,微博奇葩,星座命理,搞笑幽默
搞笑幽默
健康,星座命理,文艺,游戏动漫,旅游,军事

美女
旅行,时尚,八卦,宅,教育就业,投资理财,IT 数码
旅行,摄影,宅,购物,游戏动漫,投资理财,文艺
旅行,八卦,宅,购物,健康,汽车,文艺
游戏动漫,时尚,星座命理,文艺,双鱼就是我了,爱音乐，爱悲伤。
随性,吃喝玩乐,学生一枚,猫,梦想,佛教,白羊女,感性,简单,爬山
八卦,宅,美食,IT 数码,娱乐
娱乐
美食,旅游,浙江生活
搞笑幽默,名人明星
娱乐
旅游,其他生活
美女,星座命理,文艺,美食,IT 数码,体育
健康,星座命理,时尚,娱乐,美食
多愁善感,视频音乐
新闻资讯,教育就业,军事
旅游,美食,文艺
穿衣美容,健康,旅游,美食,投资理财
IT 数码,健康,时尚,旅游,美食
视频音乐,萌宠,搞笑幽默,微博奇葩,游戏动漫,军事
汽车,美食,文艺,新闻资讯,教育就业,军事
娱乐
萌宠
视频音乐
搞笑幽默,石家庄生活,体育,娱乐
汽车,旅游,军事
蓝色,微笑,向日葵,理财,迷糊
旅游,视频音乐,穿衣美容,美食
文艺
汽车,旅游,军事
娱乐
辽宁生活
名人明星,旅游,美食,视频音乐
旅游
星座命理
美女,军事,体育
视频音乐
游戏动漫
美女,萌宠,韩国生活,穿衣美容
北京生活,动漫控,睡觉睡到自然醒,IT 互联网
美食,星座命理
微博奇葩,搞笑幽默
新闻资讯,美食,军事
投资理财,娱乐,新闻资讯,教育就业,军事

美女,旅游,IT 数码
投资理财,IT 数码,游戏动漫,新闻资讯,教育就业,军事
健康,美食,文艺,金镶玉,珠宝,翡翠,钻石,和田玉,生活,娱乐
汽车,游戏动漫,旅游,军事,教育就业,美食,体育
时尚,美女,投资理财,汽车,美食,星座命理,军事,教育就业,旅游,IT 数码
特搞,留学,法国,娱乐
新闻资讯,IT 数码,汽车,星座命理,军事,游戏动漫,美食,娱乐
健康,娱乐,美食
娱乐
时尚,健康,旅游,星座命理,美食
美食爱好者,无厘头,旅游美食,阳光,k 歌,敏感,爱自由,咖啡控
广东生活,体育
健康,时尚
汽车,体育
美食,文艺,娱乐,时尚,健康
微博奇葩,视频音乐,搞笑幽默
微博奇葩
八卦,宅,购物,新闻资讯,美女,娱乐
时尚,数码,宅,投资理财,旅游,星座命理
旅行,时尚,娱乐,八卦,购物,新闻资讯,美女,文艺
旅行,时尚,摄影,八卦,宅,美女,教育就业,文艺
游戏动漫
美食
健康,旅游,星座命理,美食,美女
健康,星座命理,美女,时尚,娱乐
游戏动漫,IT 数码,汽车
美女,汽车,游戏动漫,娱乐,体育
美图摄影,星座运势,新闻趣事,旅游,名人明星,美女,穿衣美容
日韩,娱乐
街拍,娱乐
TVB,爱睡觉,懒人,胡思乱想,睡觉睡到自然醒,盗墓笔记,美食控,互粉
搞笑,特搞,留学,娱乐
衣橱,娱乐
穿衣美容,其他生活,星座命理
娱乐
名人明星,CUBE,徐恩光,BTOB,韩国明星,韩国组合,娱乐
娱乐,IT 数码
健康
游戏动漫,时尚,美女,军事,体育
双鱼座,书,玩,出街,买东西
健康,旅游
教育就业,美食,新闻资讯,健康,旅游
美女,游戏动漫
视频音乐,旅游,美食,搞笑幽默,穿衣美容

萌宠
名人明星, 萌宠, 搞笑幽默
搞笑幽默, 星座命理
文艺
美女
投资理财
美女
体育
星座运势, 读书分享, 校园生活, 梦想创业, 旅游, 美食, 星座命理, 穿衣美容
旅游, 视频音乐
星座命理
美食, 军事, 文艺, 体育, 娱乐
体育, IT 数码, 健康, 投资理财
宠物图集
健康, 体育, 娱乐
搞笑幽默, 体育
90 后
游戏动漫, 星座命理, 娱乐, 健康, 旅游
萌宠, 美食, 视频音乐
幽默
时尚, 娱乐
美食, 时尚, 旅游, 汽车, 体育
宅
投资理财, 时尚, IT 数码, 娱乐
文艺, 旅游, 娱乐
美食
山东生活, 搞笑幽默, 新闻趣事, 美食, 健康, 新闻资讯, 汽车, 教育就业, 旅游
旅游, 时尚, 星座命理, 美食, 娱乐
读书分享
IT 数码
星座命理, 教育就业, 健康, 投资理财, IT 数码
投资理财, 健康, 新闻资讯, 教育就业, 文艺
文艺
时尚
旅游, 健康, 投资理财, 娱乐
军事
汽车, IT 数码, 旅游, 文艺, 娱乐
旅游, 名人明星, 视频音乐, 搞笑幽默, 新闻趣事
摩托, 美妞, 工作犬, 马犬, 汽车
穿衣美容
健康, 新闻资讯, 娱乐, 旅游
时尚
视频音乐
旅游, 时尚, 投资理财, 健康, 文艺

教育就业
军事
新闻资讯
投资理财
美食
时尚
旅游
体育
时尚,旅游
星座命理,美女,娱乐
穿衣美容,娱乐
体育,军事,美女
美女
时尚,旅游,IT 数码
美食,军事,IT 数码
美女,运动,交友
旅游,健康
旅游
新闻趣事,穿衣美容,搞笑幽默,时尚
汽车
旅游,娱乐
美食,旅游
视频音乐,星座命理
宴席,地方特色,少数民族,茅台,特产,白酒,贵州,饮料,食品,酒
游戏动漫
萌宠
新闻资讯
美女,穿衣美容,星座命理
美食
新闻资讯,时尚,投资理财,旅游,娱乐
淡淡的聆听
微博奇葩
旅游,美女,健康,文艺,娱乐
美女
投资理财,星座命理,旅游,时尚,娱乐
各种体育运动
军事
娱乐,美食,游戏动漫,IT 数码
星座命理
微博奇葩,投资理财
娱乐,体育
游戏动漫,教育就业
读书分享,搞笑幽默,校园生活,情感生活,体育
投资理财

视频音乐,美女
教育就业,星座命理,投资理财,体育
旅游
美食,旅游
美女,读书，写诗,电影，篮球，动漫
星座命理
体育资讯
其他生活
萌宠,微博奇葩,搞笑幽默,新闻资讯,旅游,美食,游戏动漫,文艺,娱乐
中医,瑜伽,时尚达人,执着,旅游美食,跳舞,爱旅游,养生,爱生活,健康
美女,美食,文艺
游戏动漫,投资理财,美食
娱乐
电影控,韩剧,服装,经济,周笔畅,爱吃
90 後,跳舞,周杰伦,神经质,心理学,东方神起,台球,养生
旅游,汽车,教育就业,投资理财,娱乐,体育
美食
旅游
写字,星座命理,汽车
广东生活
娱乐
时尚
医生
视频音乐,美食,微博奇葩
军事
四川生活
名人明星
旅游
微博奇葩,北京生活,搞笑幽默
美女,时尚,汽车,旅游,美食
星座命理
文艺
娱乐
IT 数码,文艺,体育,娱乐
穿衣美容,新闻趣事,河南生活,微博奇葩,名人明星,健康,时尚,娱乐
喜欢听音乐!
新闻资讯,汽车,IT 数码
河北生活
健康
星座命理,娱乐,时尚,健康,美食
名人明星,美食,微博奇葩,新闻趣事,穿衣美容
军事
新闻趣事,北京生活,搞笑幽默,健康,美女,教育就业,投资理财,旅游
北京生活

视频音乐,校园生活,八卦杂谈,娱乐
新闻趣事,搞笑幽默,广东生活,旅游
美食
星座命理,萌宠,美食
美女,教育就业,IT 数码
健康,旅游,IT 数码
投资理财,教育就业,旅游,健康
电影,美食,旅游,教育就业,星座命理,文艺
游戏动漫,教育就业,军事
时尚,旅游,星座命理
文艺
美女,文艺
体育,汽车,美女,IT 数码
游戏动漫,美食,文艺
星座命理,美食
健康,旅游,美食
体育
星座命理
名人明星
时尚,星座命理
美食
新闻趣事,广东生活,搞笑幽默,美女
江苏生活
视频音乐,江苏生活,穿衣美容,名人明星,新闻趣事
投资理财,教育就业,健康,娱乐,文艺,新闻资讯
旅游,美食,穿衣美容,视频音乐,上海生活,睡懒觉,迷糊，听歌，
娱乐
新闻趣事,陕西生活,美女,搞笑幽默,微博奇葩
Android,香橙,读书,忧郁,设计师,爱唱歌,星星
文字,玩儿,八卦,互联网,囧
排球,记者,朴有天,乐橙,时事,温柔,阿森纳,魔兽
时尚
游戏动漫,美食,IT 数码,体育
教育就业,文艺,娱乐
星座命理,名人明星,穿衣美容,视频音乐,旅游
数码
王力宏,TF,军事,购物,耽美,粤语,粉红控,HR,旅游,星座命理
娱乐,时尚,旅游,文艺,星座命理,美食,军事,健康
程序员,科研工作者,新闻趣事
星座命理
萌宠,游戏动漫,星座命理,美食,文艺
新闻趣事,健康,投资理财,美食,军事,体育
搞笑幽默
健康,娱乐,旅游,IT 数码

名人明星,星座命理,搞笑幽默
旅游,文艺,美食,投资理财,娱乐
美食,微博奇葩,搞笑幽默,星座命理
美女
搞笑幽默,新闻趣事,视频音乐,美食,旅游
穿衣美容
旅游,娱乐
军事,IT 数码,体育
健康,新闻资讯,文艺
娱乐
体育
星座命理,时尚,投资理财,IT 数码
礼品定做,个性礼品,自助拍照,儿童摄影,个人写真,创意自拍,快乐精灵,自拍狂,自拍照相馆
汽车,新闻资讯,娱乐,文艺,投资理财
视频音乐,名人明星,北京生活,美食
文艺,娱乐
军事,新闻资讯,时尚,投资理财
旅游,名人明星
旅游,懒人
穿衣美容,星座命理,时尚,文艺
旅游
听歌,电影,自由,旅行,体育,汽车,美食,文艺,军事
美女,美食
视频音乐
投资理财,星座命理,美女,军事,IT 数码
旅游
旅游,娱乐
名人明星,旅游,上海生活,搞笑幽默,视频音乐
视频音乐
美食,穿衣美容,美女,其他生活
军事,IT 数码,文艺,体育,娱乐
美食,体育,IT 数码,新闻资讯,旅游,星座命理,娱乐
穿衣美容
体育,汽车,游戏动漫
微博奇葩,BreconBeacons,特卡波
美女,名人明星,视频音乐,搞笑幽默
文艺,环保标志,代办,黄转绿资讯
搞笑幽默,IT 数码
娱乐,文艺,星座命理,体育,时尚
视频音乐,新闻趣事,搞笑幽默,微博奇葩,广东生活
搞笑幽默
微博奇葩
搞笑幽默
IT 数码,投资理财

旅游,广州生活
搞笑幽默
美女
旅游
萌宠,微博奇葩
视频音乐
美女,旅游,IT 数码
文艺
公平能定天下
美女,视频音乐,校园生活,体育资讯,其他生活
钟辰乐,美食
健康,旅游,时尚,美食
新闻资讯
美食
情感生活,校园生活,临沧生活,旅游,星座命理,美食,文艺,娱乐
视频音乐,名人明星
新闻趣事
名人明星
娱乐,文艺,旅游,美食
旅游
IT 数码,美女,视频音乐,八卦杂谈,名人明星
音乐控,K 歌,厦门,爱旅行
美女,健康
娱乐,游戏动漫
时尚
时尚
美食
旅游,新闻资讯,健康,娱乐
视频音乐
新闻资讯,星座命理,军事,IT 数码
街拍,姐妹淘,纽约北京连线,奢侈品狂热者,北京妞,北京,哥伦比亚大学,纽约,美国生活,穿衣美容
视频音乐,穿衣美容,美女
美食
星座运势,处女座,星座
美女,时尚,IT 数码,文艺,体育
娱乐,美食
美女
读书,吸引力法则,网络营销,猫控,创业投资
视频音乐,新闻趣事,搞笑幽默,济南生活,美女
无节操,伪二次元,银魂,盗墓笔记,瓶邪
穿衣美容
视频音乐
旅游

旅游,美食
时尚
视频音乐
穿衣美容
音乐，运动
搞笑幽默,娱乐
时尚,旅游,健康,娱乐
体育
山西生活,体育
汽车,旅游,IT 数码,娱乐
穿衣美容,时尚
视频音乐
美食
美食
军事,汽车,体育
体育
娱乐
美食
穿衣美容
文艺,娱乐,投资理财,汽车,时尚
梅州生活,搞笑幽默,娱乐
游戏动漫,旅游
体育,娱乐,旅游,游戏动漫
安徽生活
萌宠,美食,旅游,辽宁生活,视频音乐,快乐,幸福的活着,旅游摄影
投资理财,健康,军事,星座命理,体育
校园生活,读书分享
美食,穿衣美容,美女,微博,爱旅行,双鱼,o 型血,冷笑话,时事,music
美女,新闻资讯,文艺,投资理财
旅游,游戏动漫,时尚,文艺
车世界,财经资讯,北京生活,新闻趣事,IT 数码,汽车,健康
搞笑幽默,视频音乐,穿衣美容,时尚,旅游,美食,文艺,娱乐
美女,游戏动漫,美食,体育,娱乐
喜欢唱歌,简单,感性,穿衣美容,萌宠,美食
星座命理,旅游,娱乐,文艺
80 後,新闻,杂志,自拍,寂寞,西安,小女人,陕西生活
旅游,穿衣美容,新闻趣事,搞笑幽默,星座命理
美女
旅游,娱乐
星座运势
美女
旅游,美食,新闻趣事,美女,安徽生活,秦时明月粉丝,秦时明月
美女,体育
投资理财,娱乐,星座命理,IT 数码,汽车

游戏动漫,时尚,汽车,体育,娱乐
美女,汽车,文艺,娱乐,投资理财
广东生活
名人明星,微博奇葩,萌宠,文艺
O 型血,懒,爱旅游,iphone,读书,健身,搞笑幽默
搞笑幽默,微博奇葩
视频音乐,美食,穿衣美容,旅游
美食
美食
文艺
八卦杂谈,名人明星,搞笑幽默,新闻趣事
旅游
新闻趣事,美食,北京生活,军事
教育就业,投资理财,新闻资讯,IT 数码,时尚,汽车,健康,文艺,体育,娱乐
游戏动漫,旅游,体育,时尚,星座命理,美食
美女,四川生活
娱乐
投资理财,新闻资讯,教育就业,游戏动漫,旅游,军事,娱乐
娱乐
微博奇葩
美食
美食,旅游,健康,教育就业,娱乐
读书分享
时尚
美食,旅游,新闻趣事,浙江生活,娱乐
我喜欢吃和贱彬
旅游,复古风,星座命理,文艺,时尚,新闻资讯
搞笑幽默,娱乐
美食
穿衣美容,旅游,美食,星座命理,萌宠
美食,文艺
时尚,旅游,美食,娱乐,美女
重庆生活
美食,美女
旅游,视频音乐,搞笑幽默,新闻趣事
新闻资讯,IT 数码,体育
旅游,健康,时尚,文艺,娱乐
旅游
淘宝差评,淘宝,维权达人,维权投诉,网购维权,消费者维权,淘宝新手,淘宝申诉,淘宝售后,
淘宝维权
教育就业
新闻资讯,美食,文艺
旅游
IT 数码,娱乐

美女,新闻趣事,萌宠,搞笑幽默,名人明星
营销,培训,平面设计,摄影
上海生活,娱乐
新闻资讯
穿衣美容,时尚
车世界,美图摄影,IT 数码
军事,体育
旅游,文艺,健康
文艺
美食,娱乐
文艺,娱乐,旅游,IT 数码
视频音乐
教育就业,美女,文艺
教育就业,健康,新闻资讯,娱乐
名人明星,新闻趣事,穿衣美容,视频音乐,微博奇葩,搞笑幽默,美食,健康,新闻资讯,娱乐
娱乐,时尚,旅游
搞笑幽默,穿衣美容,旅游,新闻趣事
新闻资讯,投资理财
旅游,美食,时尚,美女,娱乐
健康
新闻资讯,旅游,星座命理,娱乐,体育
好书,爱美食,简单,志愿者,吃货,80 後,服装设计,情绪化,独立,时尚
娱乐,美女,汽车,体育
游戏动漫
投资理财,旅游,娱乐,文艺,军事
美食,微博奇葩
健康,时尚,旅游,美食,文艺
娱乐,IT 数码,健康,美食,时尚,文艺,体育
旅游,美女,军事,娱乐
娱乐
视频音乐,广西生活,新闻趣事,文艺,教育就业,军事,IT 数码
健康
时尚,美食,娱乐,教育就业,健康
新闻资讯,娱乐,时尚,健康,星座命理
星座命理,美食,美女,娱乐,游戏动漫
旅游,美食
健康,时尚,旅游,美食,娱乐
视频音乐
搞笑幽默,军事
旅游,美图摄影,八卦杂谈,校园生活,搞笑幽默
视频音乐,旅游,文艺
重口味,新闻趣事,搞笑幽默,车世界,体育
时尚,文艺,娱乐
新闻趣事,北京生活

旅游,娱乐,时尚
教育就业,健康,星座命理,美食,旅游
美女,校园生活
搞笑幽默,体育,篮球运动方面
旅游,星座命理,视频音乐
名人明星
美女,美食,旅游,健康,体育
投资理财,娱乐,文艺,美食,体育,健康
搞笑幽默
时尚,投资理财,旅游,星座命理,美食
娱乐
娱乐
美食
新闻资讯,健康,教育就业
美食,旅游,微博奇葩,美女,搞笑幽默
IT 数码,娱乐,投资理财,汽车,游戏动漫,体育,美女
美女,穿衣美容,泉州生活,时尚
小白
小清新,爱睡觉,爱柯南,双鱼座,爱旅行,动漫控
娱乐
游戏动漫,时尚
新闻资讯,旅游
美食
名人明星,美女,旅游
美女,军事,体育
新闻资讯
旅游
美食,体育,自行车,茶,睡懒觉,投资理财,健康,旅游
新闻资讯,时尚,旅游
北京生活,IT 数码
萌宠,美食,香港生活,搞笑幽默,旅游
时尚,健康,旅游,美食
教育就业
丹华北路 71 号,母婴用品,国产奶粉,进口奶粉,奶粉
校园生活,娱乐,健康
IT 数码,星座命理,教育就业,时尚,美食,健康
旅游
微博奇葩,美女,视频音乐,新闻趣事,搞笑幽默
穿衣美容,新闻资讯
新闻资讯,美女
旅游,美食,文艺
文艺,教育就业,IT 数码,星座命理,时尚
健康,时尚,娱乐,文艺,美女,美食
动漫,篮球,漫画,吃货,旅游美食爱好者,海贼王,青春,喜欢听歌,热爱生活,投资理财

汽车,体育
教育就业,娱乐,星座命理,新闻资讯
小提琴,听音乐,爱音乐,健康,活泼开朗,游戏,体育,美食,游戏动漫,娱乐
美容服饰,情感生活,校园生活,穿衣美容
旅游
新闻趣事,名人明星,情感生活,搞笑幽默
时尚,教育就业,旅游
旅遊,旅游
星座命理,汽车,IT 数码
校园生活,旅游,唱歌，拍照
娱乐
心理学,双鱼座,善良,工作狂,男人,科幻,90,喜欢音乐
健康,金在中是我男人,銀魂,旅游,美食
新闻资讯
投资理财,健康
旅游,健康,美食,娱乐
文艺,娱乐,旅游,时尚,游戏动漫
IT 数码
时尚
健康
时尚,健康,旅游,IT 数码,美食
搞笑幽默,台湾生活,萌宠,健康
旅游,时尚,美食,IT 数码,娱乐
教育就业,新闻资讯,投资理财,娱乐,健康,美食
美女,美食
文艺,星座命理,军事,旅游,IT 数码,新闻资讯,投资理财,美食
瓶邪,黑花,风景控,小说控,音乐控,吃货一枚,盗墓笔记,星座命理,游戏动漫,文艺
汽车,时尚,体育,美食,旅游
健康,美食,游戏动漫,文艺,体育
微博奇葩,妞,宝宝摄影,摄影,妈妈,育儿,教育,亲子,淘宝,教育就业
星座命理,投资理财
美食,搞笑幽默,新闻趣事,视频音乐
美食,时尚,文艺,星座命理,娱乐
喜欢音乐,旅游
投资理财,教育就业,游戏动漫,IT 数码,体育
健康,美食
美女
健康,新闻资讯,投资理财,军事
IT 数码,娱乐
搞笑幽默,科大神,篮球篮球,金牛座,军事,游戏动漫,汽车,体育
搞笑幽默,音乐达人,感性,文艺
文艺
汽车,旅游,游戏动漫,娱乐,IT 数码
双子,艺术,巧克力,单身,美容护肤,苏打绿,随性,小女人,孩子气

美食
文艺,娱乐,美食
新闻资讯,旅游,美食,健康,星座命理,文艺
娱乐
名人明星
文艺
校园生活,八卦杂谈,娱乐
美食,健康,旅游,文艺,娱乐
体育资讯
微博奇葩
名人明星,新闻趣事,视频音乐
美食
投资理财,教育就业,新闻资讯,旅游,体育
健康,星座命理,旅游,娱乐,时尚
美食
新闻趣事,微博奇葩,IT 数码,新闻资讯,健康,旅游,美食
新闻趣事,萌宠,微博奇葩,视频音乐,穿衣美容
名人明星,视频音乐
军事,娱乐,汽车
游戏动漫,文艺,体育,娱乐,美女
文艺
萌宠,视频音乐,娱乐
娱乐,时尚
投资理财,新闻资讯,健康,时尚,娱乐
穿衣美容,美食,微博奇葩,搞笑幽默,love,90 后,开朗,天蝎
感性,聊天,微笑,单身,阳光,爱生活,爱吃,旅游
新闻趣事,建筑设计,房地产,建筑,摄影,设计师,文艺
名人明星
微博奇葩,旅游,美国生活,搞笑幽默,游戏动漫,时尚,美食,娱乐
军事,旅游,汽车
投资理财
情感随笔,成长,心灵感悟,生活,巨蟹,写作随心,语录,文艺青年,文字控,90 后
美女
视频音乐,健康,文艺,娱乐,时尚
时尚
穿衣美容
蛋疼加蛋定,巨蟹座,80 后宅女,外貌协会,游戏动漫,教育就业,娱乐,美食,星座命理
旅游
文艺,美食,娱乐
美女,视频音乐,新闻趣事,搞笑幽默
旅游,创意
排球、跑步、商人,篮球,羽毛球,乒乓球
投资理财,教育就业,爱唱歌,证券,摄影控
游戏动漫,美女,体育

娱乐,美食,时尚,旅游,文艺
娱乐
游戏动漫
美图摄影
视频音乐
广东生活,新闻趣事,视频音乐,名人明星,星座命理
娱乐,文艺,IT 数码,健康,美食
体育
科学探索,读书分享,体育资讯,旅游,视频音乐,健康,投资理财,军事,美食,文艺
投资理财,新闻资讯
素描,色彩,艺考培训,美术绘画,美术生
旅游,IT 数码,星座命理,美食,游戏动漫,军事
健康
娱乐,文艺,旅游,健康
旅游,新闻趣事
娱乐
吃喝玩乐,旅游爱好者,健身,玩,旅游美食,朋友,女人,中医,shopping,娱乐
自由人
江西生活,视频音乐,旅游,穿衣美容
小女人,爱旅行,矛盾体,陈奕迅,美食
重庆生活
旅游
新闻趣事,北京生活,搞笑幽默,微博奇葩,美食,汽车,体育,游戏动漫,军事
健康,时尚,旅游,美食,娱乐
双子,健身,爱美食,广西生活,新闻趣事,搞笑幽默,穿衣美容,美食
旅游
吃喝玩乐,爱自由,个性,90 后但我不脑残,赚钱,美女,星座命理,时尚
旅游,校园生活,视频音乐,名人明星,搞笑幽默
花事,青春,伪文艺女青年,公子嫣
打篮球
搞笑,精分宅
旅游
微博奇葩
投资理财
搞笑幽默,视频音乐,投资理财,星座命理,军事
美食,文艺,娱乐,游戏动漫,健康,体育
健康,美食,时尚,娱乐
美食,文艺,娱乐,体育
文艺,旅游,娱乐,游戏动漫
旅游,娱乐,星座命理,美食,健康,时尚,美女
美食
手机控,偶喜欢玩,摄影爱好者,星座,游戏,动漫
视频音乐,美食,健康,投资理财,教育就业,时尚
美食,文艺,投资理财,旅游,娱乐

游戏动漫,美女
搞笑幽默
穿衣美容,成都生活,健康,星座命理,美食,旅游,时尚
旅游
动画,天蝎女,美食
教育就业
时尚,娱乐
旅游
新闻资讯,时尚,旅游,美食,文艺
微博奇葩
文艺
军事,汽车
竞技游戏爱好者,吃货,小说,音乐爱好者,户外,旅游
篮球,足球迷,搞笑幽默,体育,娱乐
美女,八卦杂谈,视频音乐,泉州生活,轻度摇滚小情结,重度资讯海绵,九把刀下魂,杰迷,暴走一族,纸片党员
旅游
军事
美女,汽车,旅游,投资理财,健康
彩妆,吃喝玩乐,可爱,旅游摄影,听歌,80后,美食
高三党
穿衣美容,时尚
八卦杂谈,新闻趣事,视频音乐,搞笑幽默,汕尾生活
教育就业,时尚,美食,星座命理
情感生活,重口味,美图摄影,美女,视频音乐,游戏动漫
旅游
投资理财,体育,旅游,IT数码,文艺
星座命理,美食,娱乐
旅游,美食,新闻趣事,搞笑幽默,江苏生活
健康,星座命理,旅游,时尚,文艺
陈绮贞,旅游摄影,淘宝,文艺,音乐迷,旅游
娱乐
汽车,军事
娱乐,星座命理
星座运势,视频音乐,旅游,健康,新闻资讯,教育就业,投资理财
旅游
美食,旅游,美女,健康,文艺,娱乐,汽车,时尚
旅游,宠物图集,财经资讯
看电影,帅哥,潮,怀旧,文化,旅游,健康,星座命理,美食,文艺
美女
电影,音乐,文艺,娱乐
美女
教育就业,旅游
游戏动漫

娱乐,美女,时尚,汽车
旅游
美食,旅游,游戏动漫,星座命理,美食,娱乐,Nickhun,f（x）,00后,天蝎座
搞笑幽默,淋雨迷,旅游,文艺,笑中带愁
旅游
汽车,旅游,体育
旅游,新闻资讯,投资理财
时尚,健康,星座命理,美食,娱乐
新闻资讯,游戏动漫,美女,娱乐,体育
宅女,动漫,旅游
体育
新闻资讯
健康,新闻资讯,时尚,美食,旅游
新闻趣事,美女
名人明星
旅游,视频音乐,星座命理,穿衣美容
上海生活,时尚
旅游
美食
美食,娱乐,健康
汽车,星座命理,IT 数码
美女,旅游,投资理财,IT 数码
达州生活,健康
萌宠,新闻趣事,搞笑幽默,微博奇葩,新闻资讯,文艺,娱乐
英国生活
时尚,开朗,公益,看电影,星座,浪漫,拍照,K 歌,跑步
穿衣美容,视频音乐,搞笑幽默,星座命理,美食
游戏动漫,军事,美女,娱乐,体育,睡觉晒太阳
微博奇葩,萌宠,星座命理,美食,时尚,健康,娱乐
游戏动漫
怀旧,张国荣,一些事一些情
开朗，K 歌，学生
游戏动漫
时尚,健康,投资理财,旅游
名人明星,娱乐
体育
美食
新闻趣事,健康,娱乐
旅游,文艺,健康
游戏动漫
科技,电脑,软件,苹果,人力资源,服装设计,花痴,工作,驴友,美女
游戏动漫
旅游,娱乐
游戏动漫

游戏动漫
游戏动漫
娱乐
游戏动漫
时尚,星座命理,IT 数码
美图摄影,电子商务,夜猫子,原创,微博控,白羊
爱朋友,爱家人,爱零食,爱运动,军事,游戏动漫,汽车,IT 数码
贵州生活,娱乐
体育,汽车,旅游,娱乐,IT 数码
星座命理,视频音乐,新闻趣事,盗墓笔记,阅读
电影,运动,旅游,爱睡觉,爱生活,美女,汽车,健康,体育,娱乐
旅游,军事
搞笑幽默
天蝎座,90 後
旅游
时尚,星座命理,IT 数码
游戏动漫
视频音乐,美女,微博奇葩,星座命理,搞笑幽默
山东生活,nba,体育
搞笑幽默
穿衣美容,名人明星,美食,江西生活,手串,棉麻控
汽车,旅游,IT 数码,娱乐,体育
浪漫,热情
爱篮球,发呆,白羊座,吃货一枚,爱音乐
搞笑幽默
体育资讯
美食,新闻趣事,穿衣美容
安徽生活
新闻资讯,体育,军事,游戏动漫
游戏动漫
看电影,美食
低调
游戏动漫,军事,新闻资讯
搞笑幽默
IT 数码,体育,娱乐
旅游,星座命理,文艺,汽车,健康
星座命理,微博奇葩,萌宠,旅游,美食
新闻趣事,视频音乐,广东生活,搞笑幽默,投资理财,教育就业,娱乐,文艺,星座命理
投资理财
名人明星,视频音乐,萌宠
旅游,星座命理
新闻趣事,搞笑幽默,微博奇葩,娱乐
军事
搞笑幽默,星座命理,旅游,美女,穿衣美容,服饰搭配

健康,时尚,教育就业,新闻资讯,旅游
穿衣美容
美食
时尚,美食,投资理财,旅游,美女
IT 数码,文艺,美女,游戏动漫,旅游
浙江生活
美女,旅游,体育
搞笑幽默,星座命理,美食
娱乐,星座命理
微博奇葩
时尚,星座命理,美食
美食
惠院政法系法学
微博奇葩,搞笑幽默
星座命理,旅游
星座命理,体育
汽车,时尚,旅游,健康,新闻资讯
足球迷,汽车,闷骚,天津生活,视频音乐,新闻资讯,美女
美女
美女,娱乐,文艺,军事,新闻资讯
旅游,IT 数码
新闻趣事,娱乐
喜欢唱歌,美食,星座命理,旅游,文艺
快乐,奋斗,学习,乐观,宅女,吃货,运动,旅游,美食,文艺
美食,时尚,娱乐,文艺,美女
体育,摄影,起床困难户
汽车
星座命理,文艺,军事,时尚,美女
新闻资讯,健康,旅游
视频音乐,星座命理,体育
旅游
95 后,水瓶,游戏动漫,时尚,星座命理,娱乐,文艺
投资理财,新闻资讯,健康,军事
搞笑幽默,体育
军事
重口味,科学探索,读书分享,鹤岗生活,情感生活,投资理财,星座命理,军事
美食
将军,奴隶,脑力,体力
旅游
星座命理,美食
IT 数码
新闻趣事
新闻资讯,时尚,美女,游戏动漫
娱乐

旅游
名人明星,旅游,搞笑幽默,穿衣美容,湖北生活
健康,时尚,美食,娱乐,创意,活泼,阳光
时尚,新闻资讯,旅游,文艺,娱乐
男人,搞笑幽默
穿衣美容
美女,IT 数码,娱乐,汽车
体育,新闻资讯,时尚
交朋友,视频音乐
美图摄影,美食,其他生活,名人明星,搞笑幽默
有时候很神经质,巨蟹女,正太控,学生一枚,维尼夫妇
视频音乐,吃货
文艺,健康
旅游,视频音乐,军事,IT 数码,文艺,体育,娱乐
健康,教育就业,美食,新闻资讯,投资理财,文艺
微博奇葩,萌宠,美食,四川生活,搞笑,巨蟹,创意,篮球,汽车,电子产品
偶像剧,朋友,自恋
新闻趣事,视频音乐,搞笑幽默,微博奇葩
文艺,教育就业,美女,美食
视频音乐,微博奇葩,游戏动漫
美女,旅游,汽车,投资理财
健康,娱乐
呼和浩特生活
名人明星,四川生活,搞笑幽默
健康,旅游,美食,娱乐
爱 lol 爱生活
旅游,健康,美女,军事,体育
名人明星,美食,旅游,视频音乐,新闻趣事
旅游,娱乐,星座命理
游戏动漫,美女,文艺,体育,娱乐
娱乐
拍照,文艺,娱乐
娱乐,新闻资讯,IT 数码,军事,美女
娱乐
时尚,美女,美食,文艺,娱乐
减肥,美食,80 后,听歌
搞笑幽默,娱乐
体育
美女
时尚
时尚
穿衣美容,天津生活,时尚
健康,汽车,美食,旅游,体育
文艺,星座命理,时尚,美食,娱乐

投资理财
美食,健康,时尚,娱乐,星座命理
新闻趣事,搞笑幽默,湖北生活,微博奇葩,旅游
美女,投资理财,新闻资讯,旅游,娱乐
旅游,IT 数码,体育
游戏动漫
水瓶　音乐,健康,旅游,美食,娱乐
教育就业
90 後,IT 数码
美女,美食,文艺,投资理财,健康
教育就业,星座命理,旅游,美食,IT 数码
星座命理,娱乐
笑点低,吃货,游戏,天蝎座,上网,幽默
IT 数码
旅游,娱乐,时尚,健康,星座命理
体育
投资理财,新闻资讯,军事,文艺,IT 数码
文艺,娱乐,教育就业,旅游,IT 数码
娱乐
美女,健康,星座命理,旅游,美食
麦霸,主持,文字控,咖啡,幻想,旅游,文艺,娱乐
娱乐,IT 数码,星座命理,游戏动漫
名人明星,文艺,健康
文艺,旅游,美食,星座命理,娱乐
健康,新闻资讯,旅游,美食,体育
星座命理
军事,IT 数码,文艺,体育,娱乐
旅游,娱乐
写真,结婚,婚纱照,宜兴
投资理财,美女,娱乐
美女,IT 数码
爱旅游,温柔,大学,散步,狮子座
文艺
名人明星,敏感,纠结,文艺,体育,娱乐
娱乐,旅游,健康,时尚
娱乐
IT 数码
文艺,新闻资讯,旅游,IT 数码,娱乐
时尚,教育就业,投资理财,健康,美食
新闻趣事,文字,娱乐,教育就业,时尚
投资理财,健康,新闻资讯,时尚,文艺,旅游
旅游,睡觉睡到自然醒,美术,设计师,动漫控,王力宏,欧美控,爱旅行,多愁善感,娱乐
新闻趣事,搞笑幽默,美食,时尚,游戏动漫,美女,娱乐
体育资讯,体育

教育就业,美食,健康,时尚,娱乐
时尚,游戏动漫,娱乐,体育
广东生活,体育
旅游
时尚,娱乐
旅游
搞笑幽默,教育就业
娱乐
健康,汽车,美女,投资理财,教育就业
娱乐
娱乐
美食
穿衣美容,游戏动漫,文艺
旅游
文艺
神经大条,淡定
娱乐,美食,旅游,美女,汽车
美食,健康,星座命理,娱乐,投资理财,时尚
星座命理,时尚,新闻资讯,旅游,文艺,娱乐
军事
文艺
时事,大学生,新闻资讯,文艺
体育资讯,搞笑幽默,巨蟹座,90後
时尚,旅游,美食,娱乐
美食,旅游,文艺,娱乐,时尚,美食
美食,旅游,星座命理
名人明星,文艺,体育
名人明星,美食,文艺,星座命理,旅游,美女,体育
幼稚,阳光,路痴,健忘不记仇,淡定,活泼,学生,成长ing,00后,摩羯女
旅游,游戏动漫
滳,213小青年,自闭症
时尚,美食,游戏动漫
体育,军事,投资理财,汽车,娱乐
旅行者
娱乐,旅游
新闻趣事,新闻资讯,游戏动漫,娱乐,军事,体育
仙后,动漫宅,海贼王,迷糊,学生一枚,美食,旅游,健康
名人明星,娱乐
健康,新闻资讯,美食
美女
生活,网购,午饭,烹饪,爱旅行,养生,手工,旅游美食,新闻资讯,美食
娱乐,旅游,时尚,游戏动漫,文艺,美食
搞笑幽默,IT数码
美女,游戏动漫,汽车,旅游,体育

星座命理,游戏动漫,紫色,随性,笑,阳光,相声,喜欢音乐,漫画,动画
情感生活,高尔夫,篮球,暗恋中,睡懒觉,抓娃娃,文艺
娱乐
新闻趣事,搞笑幽默,投资理财,美女,军事,汽车,体育
投资理财
旅游,IT 数码,娱乐,教育就业,文艺
厦门生活,教育就业
娱乐,美女,汽车,体育
宅萌控,夜猫控,爱笑控,逛街控,追星控,美食控,音樂控
体育
江苏生活,旅游,美食,教育就业
星座命理,娱乐
新闻资讯
旅游
时尚,娱乐
美食,娱乐
新闻趣事,美女,搞笑幽默
视频音乐,天津生活,旅游,美女,搞笑幽默
旅游
名人明星
美食
读书,日记,旅行,管理,领导哲学
自由,听歌,美食
美女,汽车,旅游,美食,体育
游戏动漫,新闻资讯,投资理财
游戏动漫,体育
穿衣美容,视频音乐,美女,旅游,健康,IT 数码,美食,游戏动漫
投资,日剧,护士,读书,武术,人力资源,心理学,教育就业,IT 数码,娱乐
旅游
旅游,娱乐,美食
投资理财,健康,体育
搞笑幽默,体育
高考,交朋友,欧美控,随性,时尚潮流,学生一枚,学生一族,时尚,娱乐
潮人,美食,旅游,健康
娱乐
美图摄影,旅游,新闻趣事,校园生活,搞笑幽默,军事
澳大利亚生活
美女
新闻趣事,搞笑幽默,O 型血,旅游,IT 数码
财经,品牌,好书,投资理财,健康,IT 数码,旅游
搞笑幽默
美女,军事,体育
美女
投资理财,汽车,时尚,旅游,娱乐

旅游,时尚,美食,星座命理,健康
汽车,IT 数码,体育,娱乐
育儿,爱美食,小说,网络营销
娱乐,军事,美女,健康,投资理财
美女
星座命理
旅游,军事,IT 数码,文艺,体育,娱乐
IT 数码,投资理财,汽车
搞笑幽默,娱乐
苏州生活,汽车,时尚,军事,文艺,娱乐
娱乐,游戏动漫
旅游
爱电影,爱笑,喜欢听歌,淡定,娱乐,健康,文艺
美食,时尚,娱乐,IT 数码
汽车,体育
美图摄影,音樂,完美主义,旅游美食爱好者,时尚
美食,娱乐
新闻趣事,旅游,新闻资讯,文艺,教育就业
B 型血,喜欢音乐,看电影,金牛,林宥嘉
IT 数码,文艺,投资理财,美食,旅游
时尚
美食,美女,时尚,投资理财,健康
旅游,美食,体育,娱乐
游戏动漫,娱乐,美食
文艺,旅游,健康
美食,美女,时尚,娱乐,旅游
美食,娱乐
游戏动漫
汽车,美女,美食,IT 数码,体育
天平座
美女,时尚
美女,娱乐
星座命理
校园生活
娱乐
宅,自由,睡觉,淘宝,社会,情绪化,休闲,执着,娱乐,时尚
搞笑幽默,名人明星
星座命理,健康
投资理财,教育就业,健康,旅游,美食
娱乐
时尚
甘肃生活,视频音乐,美食,穿衣美容,时尚
体育
美食,星座命理,游戏动漫,时尚,文艺

娱乐, 时尚, 游戏动漫
美食, IT 数码, 时尚, 健康, 旅游
游戏动漫
时尚, 健康, 旅游, 星座命理, 美食
文艺, 美食, 健康, 时尚, 娱乐
旅游, 新闻资讯, 健康, 时尚
健康, 时尚, 芜湖万达广场, 军事, 新闻资讯, 娱乐, 美食, 旅游
健康, 投资理财, 新闻资讯, 美食
新闻资讯, 体育
美食
IT 数码, 体育, 游戏动漫
旅游, 时尚, 娱乐
爱音乐, 听音乐
时尚, 旅游, 星座命理, 美食, 娱乐
萌宠, 娱乐
搞笑幽默, 文艺
新闻资讯, 时尚, 娱乐, 文艺
英语口语, 成人英语, 外语培训, 新概念英语, 高考英语, 中考英语, 学英语, 英语, 旅游, 新闻资讯
网赚, 登山旅游, 化石收藏, 奇石收藏, 艺术, 画画, 收藏, 投资, 创业, 投资理财
美女, 投资理财
旅游, 健康, 时尚, 美女, 娱乐
旅游, 美食
搞笑幽默, 校园生活, 视频音乐, 体育资讯, 美图摄影
旅游, 健康, 文艺, 娱乐
美食
温柔, 爱猫, 天平座, 爱睡觉, 美容, 逛街, 旅游, 健康, 美食
IT 数码, 美女, 游戏动漫, 体育, 娱乐
星座命理, 旅游
时尚
军事
健康, 教育就业, 娱乐, 旅游, 美食
穿衣美容, 娱乐
汽车, 美食, IT 数码, 体育, 娱乐
文艺, 星座命理, 旅游, 健康, 娱乐
文艺, 军事, 新闻资讯
游戏动漫, 美女, 军事, IT 数码
设计师, 文艺, 时尚, 旅游
美食
文艺
新闻资讯, 旅游, 美食, 文艺, 娱乐
健康, 军事, 投资理财
汽车, 美女, 美食, 健康, 旅游
投资理财, 时尚, 旅游, 新闻资讯, 健康

娱乐
IT 数码
时尚,健康,美女,旅游,美食
IT 数码
名人明星,娱乐
K 歌,拍照,表演,时装,护肤,狗狗,健康,星座命理,游戏动漫,时尚
美女,时尚,IT 数码,娱乐,体育
微博奇葩
健康,娱乐,星座命理
名人明星,娱乐
校园生活
北京生活,汽车
娱乐,时尚
美女,星座命理,投资理财,文艺,汽车
美容服饰,健康,美食,时尚
洛阳生活,校园生活,时尚,文艺,教育就业,美食,娱乐
穿衣美容,教育就业
美女
星座命理,旅游,新闻资讯
新闻趣事,搞笑幽默,美图摄影,时尚,美女,IT 数码,汽车,娱乐,体育
旅游,体育,教育就业,健康,投资理财
日本剑道,南京剑道,剑道,体育
笑,IT 数码
穿衣美容
旅游
美食,新闻资讯,军事,旅游
搞笑幽默,新闻趣事,投资理财,旅游,游戏动漫,健康,文艺
投资理财
投资理财,文艺,美食,新闻资讯,娱乐,教育就业,健康,旅游
视频音乐,IT 数码,新闻资讯,文艺,时尚
善良,艺术,阅读,时尚潮流,简单,快乐大本营,旅游摄影,陈奕迅,奋斗,music
教育就业
文艺,美食,娱乐,旅游,IT 数码
旅游,名人明星,穿衣美容,新闻趣事,重庆生活,美食
军事
健康,美食
军事
IT 数码,娱乐,美女,军事,体育
搞笑幽默,游戏动漫
新闻资讯,旅游,星座命理,美食,文艺
新闻资讯,游戏动漫
美女,体育,娱乐,时尚,教育就业
旅游
健康,旅游

微博奇葩
星座命理
英国斗牛犬,10 后,雌雄同体,北京,旅游,娱乐
新闻趣事,旅游,时尚,娱乐,投资理财
视频音乐,佛教,日语,大学生,金牛座,纠结,钟汉良,水瓶女,猫控,听音乐
创意,搞笑幽默,数码,手机,自行车,游戏,囧,黑莓,游戏动漫
投资理财,体育
美食,投资理财,旅游,健康,新闻资讯,星座命理
名人明星,旅游,微博奇葩,新闻趣事,新加坡生活,新闻资讯,汽车,健康,体育
健康,时尚,娱乐,旅游,星座命理
时尚,文艺,娱乐,旅游,游戏动漫
新闻趣事,笑
旅游美食,金融,创意,阳光,爱生活,军事,策划,快乐,善良,IT 数码
徐州生活,music,喜欢旅游,时尚,咖啡,IT 数码
文艺,时尚
美食,旅游
旅游
IT 数码,文艺,时尚
美食,旅游,健康,投资理财
美食
搞笑幽默,文艺,教育就业,新闻资讯,IT 数码
美食
旅游,文艺,美食,健康,时尚
时尚,文艺,新闻资讯,健康,美女
一窗山海,体育,教育就业,文艺,娱乐,健康
军事,游戏动漫
新闻资讯,文艺,美食,旅游
星座命理,美食
健康,时尚,娱乐,旅游
健康
美食爱好者,格子控
旅游,投资理财,汽车
教育就业,新闻资讯,文艺,投资理财,美食
视频音乐,空间素材吧,吴亦凡,exo,小清新,文艺青年
EXO,TAO,黄子韬
游戏动漫,美女,娱乐
汽车
煎饼,酒吧,三里屯,汉堡,北京,美食
不信邪,AMei,针尖恐惧,册那
健康养生,健康
淮安生活,娱乐,健康,汽车,IT 数码,体育
时尚
时尚
星座命理

自信,阳光,摄影爱好者,微博,爱美食,购物狂,内向,环保,音乐迷,恐怖片
教育就业,星座命理,IT 数码,军事,投资理财,汽车,娱乐
健康,汽车,旅游
新闻资讯,健康,旅游,时尚,汽车
美食,星座命理,投资理财,时尚,娱乐
Free!,中孝介,SPYAIR,盗墓笔记,鬼灯的冷彻,银魂,真遥,吃货,粉五,岚少
旅游
投资理财
搞笑幽默,旅游,视频音乐,新闻趣事,星座命理,游戏动漫
IT 数码
新闻资讯,IT 数码
时尚,旅游,星座命理,美食,娱乐
时尚,娱乐,投资理财,美女
美食,旅游,体育,娱乐
求粉丝,时尚,原宿,宅女,爱狗狗,喵星人,文字控,射手女,网购达人,美女控
星座命理
搞笑幽默,微博奇葩
旅游,新闻资讯,美食,时尚,娱乐,投资理财
爱美食,手机
宅女一枚,热爱生活,善良,执着
星座命理
时尚,美女,健康,旅游,体育
旅游,穿衣美容,时尚
90 後,做梦,吃,胡思乱想,自然,双子座
旅游,美女,新闻资讯,健康,美食
校园生活,娱乐
文艺
星座命理,游戏动漫
时尚
体育资讯,军事,体育
新闻资讯,娱乐,教育就业,美女,军事
健康,IT 数码,旅游,星座命理,娱乐
新闻资讯,教育就业,旅游,美食,体育
旅游
名人明星,微博奇葩,北京生活,新闻趣事,搞笑幽默,军事,IT 数码,文艺,体育,娱乐
唱 K,视频音乐,名人明星
健康,文艺,教育就业
搞笑幽默
美食,旅游
旅游,新闻资讯,投资理财
新闻趣事,微博奇葩,搞笑幽默,河南生活,健康,文艺,旅游,时尚,美食
文艺,健康,时尚
时尚,汽车,旅游,体育,文艺
名人明星,新闻趣事,美食,时尚,娱乐,文艺

游戏动漫
IT 数码,汽车,健康,旅游,军事
小说迷,书籍,旅游
安顺生活,旅游,美女,汽车,游戏动漫,美食
文艺,美食,投资理财,时尚,健康
music,拍照,IT 数码,娱乐,汽车
时尚,娱乐,旅游,文艺,体育
萌宠,海绵宝宝,90 后但我不脑残,学生一枚,喜欢听歌,大学,单身,古灵精怪,游戏动漫
新闻资讯,健康,美食,娱乐
旅游,美食,教育就业
汽车,教育就业,新闻资讯,IT 数码
麻豆,淘宝,文艺,健康,时尚,美食,娱乐
搞笑幽默,萌宠,视频音乐,美女,游戏动漫,体育,美食,文艺,娱乐
娱乐,体育,旅游,时尚,汽车
美食,不吃咸,偏执狂,张悬
时尚,游戏动漫,健康,旅游,星座命理
奢侈品控,美食
搞笑幽默,文艺
文艺,健康,时尚,汽车,旅游,体育
娱乐
美食,搞笑幽默,视频音乐,穿衣美容,健康
美女,娱乐,投资理财,新闻资讯,体育
旅游,微博奇葩,搞笑幽默,名人明星,美女
名人明星,广东生活
星座命理,新闻资讯
汽车,旅游
旅游,汽车,健康,美食
美女,投资理财,娱乐,文艺,体育
游戏动漫,时尚,新闻资讯,投资理财,星座命理
娱乐,文艺,合肥路 636 号,思南公馆,新天地,树洞咖啡
偶稀饭睡觉觉,懒,90 后,美食,新闻资讯,文艺,娱乐
美食
成都生活,读书分享,视频音乐,文艺,教育就业,旅游,IT 数码,娱乐
文艺
旅游
美体,潮流,彩妆,网购达人,化妆,fashion,美女,时尚
体育,感性,打球,学生,麦霸,听音乐,健身,魔羯座
新闻趣事,看电视,荷兰,幸福
时尚,投资理财,文艺,娱乐,新闻资讯
萌宠,旅游,疑难杂症活标本,硬汉哎呦我擦,甜食重度成瘾患,反撸斗士,优质人渣,暴力犯罪嫌疑人
美女,体育
上海生活,汽车,体育
投资理财

美食,旅游
旅游,美食,文艺,时尚,新闻资讯
美食,健康,旅游,文艺,娱乐
美女
健康,游戏动漫,美食,旅游
健康,星座命理,文艺,感性,文字,跳舞,90 后非脑残,性感,演员,绘画
新闻趣事,湖北生活,新闻资讯
萌宠,文艺
娱乐
体育
健康,旅游,美食,时尚
美食
美女,美食,健康,时尚
资讯,新闻资讯
投资理财
教育就业
八卦杂谈,旅游
汽车
新闻资讯
美女,健康,投资理财,汽车
美食
萌宠,新闻趣事,搞笑幽默,微博奇葩,浙江生活,健康,文艺,新闻资讯,时尚
音乐爱好者,旅游
投资理财,星座命理,健康,时尚,娱乐
美食,娱乐
美女
文艺
旅游
旅游,美食,美女,IT 数码,娱乐
名人明星,美女,旅游
美食
健康,娱乐
娱乐
时尚,美食,游戏动漫,IT 数码,娱乐
旅游,汽车
旅游,美女,文艺,娱乐,时尚
体育,美女
旅游,健康,文艺,美食,星座命理
时尚,娱乐,美食,旅游
搞笑幽默,微博奇葩
投资理财
fashion,手机,摄影爱好者,摄影师,爱旅行,娱乐,旅游,文艺,IT 数码,新闻资讯
体育
健康,美女,时尚,汽车,投资理财

健康,时尚,美食
旅遊,汽車,投資理財
新闻趣事,娱乐,健康,教育就业
旅游
娱乐,文艺,教育就业,美女,星座命理
时尚
旅游
体育
星座命理,教育就业,健康,美女,美食,娱乐
旅游,游戏动漫,美食
环保源于生活,而高于生活
健康,美食,娱乐
文艺,体育,军事
怀旧,宠物,基督徒,睡觉睡到自然醒,猫咪,喜欢旅游
学生一族,爱玩,旅游爱好者,热爱生活,感性,可爱,学生一枚,执着,完美主义,健康
娱乐,文艺,敏感,星座命理,生活
健康,美食
教育就业,时尚
旅游,星座命理,美食,IT 数码,娱乐
潮人,营销,微博控,创意,品牌,时尚
游戏动漫
搞笑幽默,美女,新闻趣事,美食,微博奇葩
情感生活,水瓶女,衣服控,时尚,文艺
交朋友,努力,活泼开朗,创意,阳光,睡觉睡到自然醒,忧郁,平常心,射手座,善良
美女
美食,文艺
视频音乐,微电影,武汉理工大学,原创,文艺
投资理财
美食,穿衣美容,山西生活,搞笑幽默
视频音乐,美食
美食,旅游
时尚
星座命理
美食,美女,旅游,名人明星,穿衣美容
欧美音乐,台球,淘宝,新闻资讯,汽车
搞笑幽默,美食
世勋心头好,EXO
新闻资讯,健康,旅游
新闻资讯,投资理财,健康,旅游,美食
时尚
娱乐
文艺
自然,娱乐
美食

文艺
新闻资讯
新闻趣事,健康
星座命理,旅游,美食,娱乐
美女,IT 数码,时尚
乐观,吃货,喜欢旅游,摩羯女,游戏动漫,星座命理,旅游,美食,文艺
文艺,健康,旅游,新闻资讯,投资理财
美女,娱乐
娛樂,遊戲動漫,星座命理
时尚
时尚,健康,美食,娱乐
旅游,娱乐,教育就业
新闻趣事,搞笑幽默,微博奇葩,视频音乐,新闻资讯,娱乐,美食
时尚
古典音乐,美图摄影,萌宠,唱歌,向日葵,星座命理
文艺,教育就业,时尚,健康,美食,娱乐
新闻资讯,旅游,美食,文艺,体育
娱乐,军事,星座命理,健康,体育
美食,军事,文艺,体育,娱乐
视频音乐,新闻资讯,IT 数码,娱乐
汽车,体育
美食
美食
旅游,娱乐,健康
军事,娱乐,投资理财,健康,旅游
美食
美食
星座命理,投资理财,文艺,新闻资讯,军事
搞笑,音乐,GEM 鄧紫棋,体育,文艺,时尚,娱乐
新闻资讯,汽车,旅游,军事,体育
淘宝,网购,服饰,美容护肤,瘦身,登山,美女,时尚
星座命理,文艺,体育
娛樂,星座命理
投资理财,文艺,汽车,新闻资讯,旅游
新闻资讯
旅游,美食,时尚
美食
微博奇葩
湖南生活,美女,时尚,娱乐,汽车,投资理财
新闻趣事,汽车
汽车,美女,娱乐,体育
美女
娱乐
跑步,西安,旅游,IT 数码,文艺,投资理财,娱乐

IT 数码,军事,美食,娱乐
娱乐,星座命理
低调,购物狂,新闻资讯,健康,旅游,美食,娱乐
美女,军事,美食,旅游,星座命理
新闻资讯,投资理财
美女
体育,游戏动漫
娱乐,健康,新闻资讯,时尚
新闻资讯,文艺,娱乐
娱乐
健康,旅游,新闻资讯,教育就业,娱乐
汽车
开心果无忧无虑,吃得好睡得香,流浪,幸福,搞笑,爱睡觉,快乐大本营,新闻资讯,旅游,体育
美食,文艺
微博奇葩,投资理财
娱乐
健身,极限飞盘,Ultimate,体育
穿衣美容,微博奇葩,新闻趣事,搞笑幽默,福建生活
文艺,星座命理,美女,游戏动漫,时尚
健康,旅游,美食,文艺,投资理财
教育就业,健康,时尚,娱乐,美食
新闻资讯,游戏动漫,教育就业,娱乐,体育
体育
健康,旅游,时尚
体育,投资理财,美食,娱乐,新闻资讯
美女,时尚,旅游,美食
视频音乐,时尚,shopping,90 后但我不脑残,喜欢听歌,热爱生活,街舞,爱玩
穿衣美容,健康,娱乐
视频音乐,时尚,娱乐
股票,财经,A 股,顶,底,谈股论金,大盘预测,龙头板块
美女
体育
新闻资讯,健康,文艺,美食,娱乐
新闻趣事,搞笑幽默,旅游,美食,体育
微博奇葩,爱美食,搞怪,梦想睡到自然醒,金牛
投资理财,旅游,星座命理,美食,娱乐
军事
新闻资讯,教育就业,健康,美食,投资理财
时尚,美女
音乐,旅游,时尚,摄影
健康,旅游,星座命理,美食,娱乐
星座命理
航空航天,机械制造,职业教育,软件培训,星座命理,体育,汽车,新闻资讯,军事
IT 数码,新闻资讯,健康,旅游,体育

情感生活,名人明星,校园生活,视频音乐,娱乐,旅游
体育
娱乐
健康,旅游,文艺,娱乐
时尚
美食
美食,投资理财,汽车,美女,体育
汽车,文艺,旅游,健康,时尚
音乐,张瑶,黄征,安又琪,阿杜,胡彦斌,阿兰,韩庚,周笔畅,乐华娱乐
上海生活,微博奇葩,文艺,健康,旅游,星座命理,美食
投资理财,娱乐,美食,星座命理,时尚
新闻资讯,汽车
时尚,旅游,美食,军事,美女
文艺
旅游,星座命理,军事,游戏动漫,娱乐,体育
sj,gossipgirl,music,cosplay,love,android,fashion
投资理财,星座命理,军事
搞笑幽默
新闻资讯
搞笑幽默,星座运势,名人明星,视频音乐,美食,星座命理,娱乐
旅游
穿衣美容,双子座,新闻资讯,健康,时尚,娱乐,美食
体育,健康
北京生活,新闻趣事,健康,新闻资讯,投资理财,IT 数码
时尚,娱乐,星座命理,健康
美食,文艺,星座命理,健康,娱乐
娱乐
星座命理,文艺,军事,新闻资讯,游戏动漫,娱乐
美女
萌宠,美食,时尚,旅游
时尚,投资理财,健康
旅游
星座命理
搞笑幽默
美女,K 歌,拍照,火影忍者,游戏动漫
星座命理
星星,漫画,蓝图,文字控,星座,摩羯女,喜欢旅游,喜欢听歌,宅女,90 後
教育教学,娱乐
八卦杂谈,长沙生活,校园生活,旅游
游戏,名人明星,搞笑幽默,游戏动漫
游戏动漫,军事
美术,处女座,微笑,青春,银魂,文艺
星座命理,旅游,健康,娱乐,文艺
微博奇葩

美食,漫画迷
星座命理,娱乐
娱乐,IT 数码,体育
安静,努力,孩子气,旅游,星座命理,美食,文艺,娱乐
美食,视频音乐,游戏动漫,投资理财
旅游,娱乐
星座运势,美食,搞笑幽默,新闻资讯
职场招聘
微博奇葩,星座命理,穿衣美容,视频音乐,名人明星,体育,文艺,娱乐
IT 数码
美食
双重性格,时尚,美食,星座命理
娱乐
时尚,旅游,星座命理,美食,娱乐
时尚,教育就业,美女,星座命理,美食,旅游,娱乐
搞笑幽默,情感生活,美容服饰,美图摄影,新闻趣事
投资理财,健康
游戏动漫
体育,文艺
看书,睡觉,上网,巧克力,猫咪,音乐达人,纠结,跑步,美食,文艺
幸福,星座命理,西瓜红控,狮子座 O 型男,双鱼座 O 型女,神经质,无厘头,情绪化,宅女一枚,微博控
投资理财
美食,旅游,健康,教育就业,美食
旅游,美食,宠物图集
成都生活,视频音乐,执着,简单,娱乐,旅游,美食,星座命理
娱乐
深圳生活,美图摄影,宠物图集,美食
名人明星,八卦杂谈,视频音乐,校园生活,星座运势,体育
投资理财
美食,娱乐,体育,文艺,旅游
美食
甘肃生活
娱乐,军事
投资理财,美女,教育就业,健康,旅游
微博奇葩,海南生活,搞笑幽默,旅游,健康,美食,娱乐
视频音乐,体育
文艺
新闻资讯,美女,游戏动漫,美食,视频音乐,新闻趣事,搞笑幽默,上海生活
汽车,投资理财,军事,娱乐,美女
旅游
天枰座,天秤,动漫控,文艺,星座命理,时尚,娱乐
旅游,时尚,文艺,星座命理
健康,文艺,投资理财,IT 数码

时尚,娱乐,交友,单身,电音,努力
新闻资讯,教育就业,投资理财,旅游,文艺
日本综艺,游戏动漫,文艺,娱乐
游戏动漫
星座命理,汽车,健康,旅游,美食,娱乐
美食,穿衣美容,娱乐,时尚
教育就业,健康,旅游,美食,体育
顶级定制,原版皮,奢侈品狂热者,奢侈品复刻,奢侈品牌控,高级定制,奢侈品,包包,鞋控
八卦杂谈
旅游,时尚
旅游
IT 数码,军事,美食,时尚,文艺,旅游,星座命理,娱乐
健康,新闻资讯,美食,军事,旅游
汽车,游戏动漫,美食,军事,IT 数码
水瓶座,懒人,音乐爱好者,纠结,水瓶女,旅游,时尚,星座命理,娱乐
穿衣美容,文艺,志愿者,巧克力,吃货一枚
娱乐
穿衣美容,旅游,广东生活,萌宠,教育就业,文艺,游戏动漫,健康,美食
美食,旅游
军事,美食,新闻资讯,旅游,健康
新闻趣事,微博奇葩,名人明星,搞笑幽默,文艺,美女,娱乐
汽车
自由,奋斗,狮子座,大学生,90 後,臭美,活泼开朗,热爱生活,军事
健康,投资理财,时尚,旅游,美食
文艺,娱乐,体育
美国代购,Birkin,奢侈品代购,Dior,Hermes
娱乐
娱乐
娱乐,时尚,投资理财,旅游,美食
娱乐
新闻资讯,健康,旅游,星座命理,美食
体育
搞笑幽默,娱乐
卡卡,资讯,任性,双鱼女,赚钱,健康,旅游,IT 数码,娱乐,投资理财
星座命理,游戏动漫,军事,体育,娱乐
文艺,健康,娱乐,军事,美女,旅游,体育,汽车
娱乐
生物仪器,流体,流体设计,传感器,医疗传感器,比例阀,分析仪器,生命科学仪器,环境监测仪器,医疗器械
健康,星座命理,体育,娱乐,美女
时尚,教育就业,文艺
文艺
美食
天蝎座,教育就业,健康,旅游,星座命理,美食

登山,睡觉睡到自然醒,偶喜欢玩,旅游,文艺
摄影控
星座命理,IT 数码,娱乐
健康,娱乐,时尚
美食
旅游
娱乐,时尚
投资理财,IT 数码,汽车,新闻资讯,军事
文艺
文艺,美女,健康,美食,旅游
娱乐
旅游
体育,娱乐
旅游
娛樂,美食
搞笑幽默,shopping,感性,收藏,水瓶,摄影师,潮流,投资理财,星座命理,军事
美女,时尚,旅游,文艺,娱乐
游戏动漫
美食
旅游
考研党,考研奋斗,李俊秀,infinite
编导,电影,汽车,娱乐
新闻资讯,时尚,旅游,健康,投资理财
健康,教育就业
时尚,游戏动漫,汽车,旅游,美食
文艺,健康,IT 数码,时尚,体育,娱乐
美女
游戏动漫,投资理财,美女,娱乐
军事,汽车,体育,美女,时尚
星座命理
美女,旅游
iphone,爱美食
娱乐
汽车
美食,穿衣美容,微博奇葩,新闻趣事
萌宠,搞笑幽默,游戏动漫,星座命理
娱乐
美剧迷,单身,花痴,吃货,乐观,闷骚,B 型血,聊天,简单,感性
美食,军事,体育
文艺,美女
微博奇葩,娱乐
IT 数码
文艺,星座命理,美食,游戏动漫,新闻资讯
美食

骑行,钓鱼,旅游,美食,化妆造型,摄影,时尚
时尚,新闻资讯,旅游,星座命理,娱乐
教育就业,星座命理,美食,娱乐,赚钱,我喜欢设计,天蝎座 80 后,随性,发呆,听音乐
微博奇葩,浙江生活,搞笑幽默,文艺,新闻资讯,娱乐
美食
时尚,美食
游戏动漫,体育,娱乐
旅游,教育就业,时尚,健康,美食
娱乐,美食,新闻资讯,旅游,健康
军事
猫咪控,天蝎座
美女
投资理财,简单的快乐
娱乐,文艺,军事,新闻资讯,游戏动漫
娱乐
星座命理,旅游,新闻资讯,投资理财,游戏动漫,美女,文艺,娱乐
新闻趣事,搞笑幽默,云南生活,萌宠,视频音乐,健康,体育,教育就业,投资理财,时尚
动漫,游戏动漫
新闻趣事,搞笑幽默,微博奇葩
时尚
美食,美女,游戏动漫
旅游,时尚
荆州生活,健康,新闻资讯,教育就业,投资理财,娱乐
美食
传媒
时尚,教育就业,健康,美食,旅游
旅游
文艺
金融,热爱生活,旅游
金融,互粉,游泳,旅游
娱乐,旅游
张艺兴,紫色,巨蟹女,猫控
美食,旅游,游戏动漫,健康
新闻趣事,军事
旅游,新闻资讯,星座命理,娱乐,军事,美食
WOW,shinee,psp,DIY,娱乐
视频音乐,其他生活,穿衣美容,旅游,美食
时尚
娱乐,教育就业,新闻资讯,健康,军事
美食,健康,游戏动漫
心有多大,梦想就有多大
美食,旅游,新闻资讯,健康
星座命理
体育资讯,娱乐,旅游

旅游
新闻资讯,投资理财,健康,旅游,美食
微博奇葩,教育就业
微博奇葩,游戏动漫
八卦杂谈,搞笑幽默
动漫,中小企业信息化,美剧,双鱼座
文艺,投资理财,时尚,星座命理,娱乐
购物,淘宝,时尚,健康,文艺,娱乐
教育就业,美女,军事,IT 数码,娱乐
教育就业,美女,军事,健康,体育
爱自由,公益,听音乐,发呆,腐女,新闻资讯,旅游,军事,文艺,娱乐
星座命理
娱乐,投资理财,军事,文艺
教育就业,健康,旅游,文艺,娱乐
投资理财
视频音乐,IT 数码,旅游
军事,健康,投资理财,旅游,美食
娱乐,投资理财,时尚,文艺,旅游,美食
健康,旅游,星座命理,美食
新闻资讯,教育就业,健康,旅游,投资理财
吃货一枚,80 后,时尚,投资理财,旅游,健康,美食
娱乐
健康,文艺
85 后射手女,旅游摄影,看电影,听音乐,爱音乐,星座,文艺,投资理财,IT 数码,时尚
星座命理,美食,文艺
游戏动漫,体育,星座命理,文艺,娱乐
游戏,新闻资讯,健康,军事,体育,娱乐
新闻资讯
微博奇葩,旅游美食,电视,游戏动漫
美食
文艺
旅游
美食
投资理财
旅游,星座命理
旅游是我的最爱,体育,汽车,IT 数码,旅游
健康
健康,娱乐
美食
吃货,傻
名人明星,娱乐
美女,IT 数码,体育,娱乐
旅游
汽车,时尚,体育,娱乐

娱乐
80 后大宅女,合拍的处女座,混合性格,呆芒果,旅游,健康,游戏动漫,时尚,美食
体育
美食
IT 数码,文艺,娱乐
汽车
投资理财
新闻趣事,IT 数码
体育
房地产从业人员,美食
文艺,旅游,健康,娱乐
K 歌,拍照,旅游,健康,时尚,美食,星座命理
娱乐,教育就业,时尚
星座命理,旅游
娱乐
美女,时尚,娱乐
旅游,美食,投资理财,健康
情感生活,美图摄影,新闻趣事,美食,金牛,文艺,新闻资讯,娱乐,旅游
健康
美食
旅游
投资理财,旅游,文艺,体育,娱乐
娱乐
爱玩,疯子,文艺,时尚,旅游,汽车
美女,美食,健康,时尚,娱乐,教育就业
深圳生活,时尚,健康,星座命理,文艺,娱乐
时尚,汽车,体育,娱乐
美女,风行,80 後,摄影控,娱乐,时尚
体育
美女
穿衣美容,投资理财,时尚
时尚,投资理财,美食,星座命理,娱乐
投资理财
IT 数码,游戏动漫,娱乐
娱乐
娱乐,美食,健康,时尚,文艺
湖南生活,娱乐
穿衣美容
文艺,旅游,美食,IT 数码,时尚,娱乐
新闻资讯,教育就业,汽车,IT 数码,体育
文艺,健康,美食
美食
娱乐,投资理财,时尚,军事,美女
星座命理

名人明星,娱乐,健康
娱乐
美女
手工,宠物,美食,旅游
美食
上海生活,旅游,穿衣美容,萌宠,天蝎座 80 后,爱猫族,美食
美女,文艺,IT 数码,星座命理,美食
新闻资讯,美食,旅游
美食,娛樂,汽車
美女,军事
游戏动漫
旅游,星座命理,美食,文艺,娱乐
健康,旅游,美食
旅游,健康,体育
IT 数码,教育就业,军事,美女,游戏动漫
旅游
IT 数码,时尚
温柔,音乐达人,房产,服饰,爱幻想,购物狂,网络美女,模特,美女,大学生
旅游,IT 数码,健康,星座命理,美食,时尚,文艺
星座命理,旅游,健康,美女,时尚,文艺,娱乐
新闻资讯,美食,投资理财,旅游,健康
军事,游戏动漫
汽车,健康,娱乐,美食
旅游,美食
健康,旅游,教育就业,娱乐,IT 数码
健康,旅游,星座命理,美食,娱乐
IT 数码,教育就业,美女,娱乐,体育
健康
爱心， 慈善,， 学生,媒体人,广播,记者,公益,医学,健康
军事
旅游,健康,时尚,娱乐
旅游,文艺,时尚,星座命理,美食
新闻资讯
旅游
健康,时尚,投资理财,旅游,美食
娱乐,文艺,军事,美食,旅游
汽车,娱乐,美食,旅游,时尚
旅游,新闻资讯,教育就业,投资理财,IT 数码
交友,上网,休闲,音樂,奋斗,微笑,新闻资讯,旅游,军事,娱乐
搞笑幽默
健康,体育,娱乐
健康,旅游,时尚,星座命理,娱乐
健康,舒服,感冒,胖,难受,瘦身,腰,酸痛,无力,头晕
蒙特利尔,多伦多,温哥华,加拿大移民,加拿大,加拿大留学生,加拿大留学

Unicorn,FinnMertens
美女,游戏动漫,体育
上海生活,体育
微博奇葩
旅游,娱乐
美食,时尚
旅游,健康,军事,娱乐
湖南生活,文艺
美女,娱乐
体育
家装设计,美食,文艺
体育
时尚,体育,旅游,美食
文艺,娱乐
娱乐,文艺,新闻资讯,美女,军事
新闻资讯,投资理财,军事,旅游,美食
文艺
名人明星,打篮球,汽车,体育
广西生活,新闻资讯
宅女
时尚
文艺,旅游
广东生活,茂名,信宜,高州,化州
投资理财,汽车,健康,新闻资讯
体育,娱乐,时尚,新闻资讯,美女
美食
体育
美食,名人明星,校园生活,搞笑幽默
微博奇葩,美女,辽宁生活,娱乐,健康
星座命理,旅游,娱乐,体育,IT 数码,时尚
星座命理,旅游,美食,IT 数码,娱乐
微博奇葩,萌宠,视频音乐,时尚,汽车,旅游,军事,IT 数码
游戏动漫,美食,娱乐,时尚,旅游,美食,美女
投资理财,体育
旅游,美食,娱乐
娱乐,健康,美食,星座命理,IT 数码,文艺
网络营销
旅游
时尚,健康
萌宠,表演,厦门,奋斗,娱乐
积极向上的 90 后,娱乐,IT 数码,文艺
搞笑幽默,体育,娱乐,时尚,文艺
旅游,穿衣美容,美食,视频音乐,时尚,健康,娱乐,文艺,星座命理
湿。

汽车
喜欢音乐,狗狗,猫咪控,时尚
八卦杂谈
娱乐,教育就业,军事
搞笑幽默,广东生活,星座命理,旅游,穿衣美容,新闻趣事
低调,音樂
旅游,IT 数码,文艺,新闻资讯
旅游,健康,娱乐,美食,IT 数码
听歌,90 后,搞笑,乐观,吃货,幽默,爱情,好性格,爱美食,幻想
旅游,星座命理,美食
美食,搞笑幽默,健康
汽车,美女,娱乐
时尚,投资理财,文艺,娱乐,星座命理
体育,汽车
时尚,美女,娱乐
星座命理,IT 数码
新闻趣事,娱乐
新闻趣事,微博奇葩,穿衣美容,星座命理,文艺,IT 数码,娱乐
星座命理,健康
新闻趣事,军事
视频音乐,广东生活
厦门生活
健康,新闻资讯,投资理财,汽车
新闻资讯,时尚,IT 数码
新闻资讯,体育,文艺,军事,IT 数码
美食,文艺
旅游,IT 数码
旅游,健康,时尚,文艺
娱乐
多愁善感,85 后,安静,我就是我,随性,美女,单纯,投资理财,美食,健康
文艺,健康,美女,汽车,时尚
娱乐,教育就业,时尚,旅游,健康
香港代购,名牌包包
格子控,吃,宅女,小说,睡懒觉,懒人,赚钱,完美主义,动漫控,天蝎
穿衣美容,美女,娱乐
娱乐
美女,IT 数码
投资理财,汽车,旅游,体育
文艺
体育,美女,娱乐,汽车,文艺,IT 数码,时尚
美食,文艺
搞笑幽默,文艺,体育
军事
搞笑幽默

健康,新闻资讯
旅游
读书分享,武汉生活,新闻趣事,校园生活,教育就业,文艺,美食
娱乐,体育,文艺,军事,美食
美食
旅游,新闻趣事,视频音乐,美女,搞笑幽默
微博奇葩,名人明星,美女,视频音乐
IT 数码
健康,新闻资讯,旅游,娱乐,教育就业
健康
娱乐
旅游,星座命理,美食,文艺
视频音乐,美女,搞笑幽默,美食,游戏动漫,时尚,IT 数码,娱乐
投资理财,IT 数码,美女
名人明星,林峰,快乐大本营,大学生,娱乐
娱乐,时尚,文艺
微博奇葩,其他生活,美食,ACG,腐,BL,广播剧,宅,文艺
军事,美食,旅游,游戏动漫,体育
游戏动漫
微博奇葩,视频音乐,新闻趣事,搞笑幽默,名人明星
汽车,美女,旅游,IT 数码,体育
新闻资讯,娱乐,美食
IT 数码
美女,文艺,体育,娱乐
星座命理
视频音乐,汽车,娱乐,体育
娱乐,旅游
忧郁与阳光并存,完美主义,楠瓜
美食
时尚,星座命理,健康,娱乐
文艺,艺术,音乐,摄影,建筑结构,建筑,新闻资讯,旅游
美食
山东生活,搞笑幽默,新闻趣事
汽车,投资理财,娱乐
萌宠,游戏动漫
名人明星,微博奇葩,视频音乐,旅游,美女,文艺,娱乐,时尚
文艺,娱乐
军事,娱乐
美食,旅游,时尚,IT 数码
乐观,吃,保健养生,腰突,新闻资讯,健康
视频音乐,新闻趣事,名人明星,萌宠
寻找爱情
广安生活,娱乐
美女,时尚,娱乐,体育

巨蟹座,时尚潮流,新闻资讯,美女,时尚,旅游,美食
娱乐
汽车,健康,时尚,教育就业,投资理财,美食
美食
美食,新闻资讯,IT 数码,健康,游戏动漫
星座命理,时尚,娱乐
旅游,美食,文艺,娱乐
新闻资讯,美女,体育,汽车,时尚
双鱼,营销
名人明星,星座运势,视频音乐,星座命理,娱乐
旅游,视频音乐,美女,搞笑幽默,微博奇葩
美食,健康,时尚
汽车,时尚,新闻资讯,美女,游戏动漫
汽车,娱乐,搞笑幽默
体育
旅游,美食
穿衣美容,视频音乐,微博奇葩,名人明星
名人明星,校园生活,体育资讯,旅游,IT 数码
新闻资讯
旅游,教育就业,健康,新闻资讯,美食,文艺
文艺,军事,旅游,美女,美食
文艺
新闻资讯,健康
旅游
美食
创意设计,旅游,美容服饰,新闻趣事,读书分享,处女座,吃货
星座命理
娱乐,美食
娱乐
教育就业,健康,时尚,文艺,美食
时尚,星座命理,旅游,文艺,健康
娱乐,军事
穿衣美容,汽车
情感生活,佛山生活,美容服饰,视频音乐,新闻趣事
萌宠
名人明星,新闻趣事,穿衣美容,搞笑幽默,星座命理,健康,娱乐,时尚
时尚
名人明星,娱乐
娱乐,健康,新闻资讯,旅游,星座命理
体育,娱乐
投资理财,教育就业,汽车,美女,IT 数码
遊戲動漫,美食
健康,时尚,投资理财,旅游,文艺
时尚,投资理财,IT 数码,美食,健康

新闻资讯,健康,旅游,娱乐
美食,微博奇葩,搞笑幽默,新闻趣事,浙江生活
旅游,新闻资讯
旅游,美食
体育
军事
名人明星,娱乐
音乐达人,爱美食,吃,小女人,it 互联网,双重性格,吃喝玩乐,敏感,古灵精怪,天蝎
教育就业
娱乐,星座命理
仙后,银魂,渣作者,静临青黄,宅腐双修
美女,文艺,IT 数码,体育,娱乐
美食
旅游
新闻资讯,健康,星座命理,美食,娱乐
汽车,游戏动漫,娱乐
星座命理,文艺,娱乐
体育
名人明星,文艺,体育,娱乐
时尚
健康
天津生活,搞笑幽默,军事,IT 数码
星座命理,水瓶座,重情,恋旧,唱歌,听歌,DOTA,IT 数码,娱乐
搞笑幽默
汽车,投资理财,娱乐,旅游,IT 数码
新闻资讯,健康,时尚,教育就业,旅游
名人明星,娱乐
文艺
游戏动漫
时尚
星座命理,娱乐
文艺,体育,娱乐
美食,娱乐
上海生活,文艺
文艺
健康,美食,旅游,文艺
IT 数码
体育
军事,IT 数码,文艺,体育,娱乐
健康,汽车,投资理财,美食,军事
美女,新闻资讯,投资理财,娱乐,时尚
星座命理,娱乐,时尚
娱乐,汽车,游戏动漫,旅游,体育
成都生活,星座运势

美食,游戏动漫,娱乐,体育,美女
娱乐,文艺,美食
视频音乐,爵士乐日,jazzday,爵士论坛,爵士乐讲座,爵色影展,上海爵士,爵士乐,娱乐
旅游,新闻趣事,美女,穿衣美容,汽车,IT 数码,体育,娱乐
时尚,投资理财,健康,旅游,美食
时尚,汽车,旅游,美食,文艺
美食,旅游,新闻资讯,时尚,娱乐
IT 数码,美女,视频音乐,旅游
美食,文艺
时尚,旅游,美食,体育,娱乐
美食,娱乐,健康
名人明星
汽车爱好者,汽车用品,旅游
娱乐
旅游,美食,文艺
美食,北京生活,宝宝妈妈,羽毛球,小龙虾,美食控
IT 数码
汽车
文艺,旅游,健康,美食,星座命理
美食,旅游,健康,时尚,娱乐
汽车
IT 数码,旅游,星座命理
动漫,爱吃,爱自由,恐怖片,爱猫,爱幻想,粤语,广告人,星座命理
IT 数码,娱乐,体育
风一样的女子
双子座,旅游,美女,美食,娱乐
萌货,女神经,睡货,吃货
名人明星,体育
体育
汽车,星座命理,IT 数码
爱生活
投资理财,贷款融资,公积金贷款,低调,房地产,二手房按揭贷款,善良,旅游,新闻趣事,广东生活
美食
投资理财,游戏动漫,时尚,健康,星座命理
摇滚,游戏动漫,美女,IT 数码
星座命理,游戏动漫
汽车,体育
茶马古道,文化遗产,骑行,徒步
新闻趣事,健康
娱乐,健康,美食,星座命理
学生一枚,旅游,穿衣美容
娱乐,美食
视频音乐,微博奇葩,名人明星,美女,火影,教育就业,IT 数码,文艺,体育,娱乐

健康,旅游,美食
双鱼座,美食,时尚,健康,旅游,星座命理
文艺,北京生活
萌宠,投资理财
旅游,搞笑幽默,游戏动漫,军事
游戏动漫,体育
名人明星,投资理财
健康
搞笑幽默
名人明星
强迫症,手机看小说,宅女一枚
旅游,娱乐,汽车,文艺,时尚
文艺
搞笑幽默
宠物图集
美女
旅游,教育就业,健康,美食,时尚,星座命理,娱乐
教育就业,娱乐,旅游,美食,星座命理
新闻资讯,美食,军事
星座命理,视频音乐
穿衣美容,时尚
文艺,娱乐,星座命理,教育就业
四川生活,新闻趣事,美女,星座命理,视频音乐
搞笑幽默
娱乐,美食,投资理财,旅游,IT 数码,文艺
美食,重庆生活,微博奇葩,旅游
文艺
娱乐
文艺
美女,投资理财,娱乐,游戏动漫,星座命理
军事
娱乐
健康
搞笑幽默,善良,游戏,搞笑
唱歌,寂寞,Music,EXO,星座命理,娱乐
穿衣美容,娱乐
游戏动漫,美食,健康
巴西,广播剧,时尚,文艺,美食,娱乐,美女
新闻资讯,娱乐,旅游,军事,文艺
美食,时尚
搞笑幽默,白羊女,狮子女,山下智久,沈昌珉,娱乐
IT 数码,星座命理,旅游,时尚,娱乐,文艺
视频音乐,娱乐
名人明星,娱乐,健康,新闻资讯,军事

美食,文艺,军事,娱乐,体育
娱乐
音乐爱好者,绘画,设计师,吃喝玩乐,爱自由,A 型血,梦想,室内设计,化妆,唱 k
名人明星,视频音乐,校园生活,搞笑幽默,教育就业,新闻资讯,星座命理,IT 数码,体育
旅游
名人明星
美食,旅游
旅游,新闻资讯,娱乐
娱乐,时尚,健康,旅游
学习,网络,旅游
军事
梦想
视频音乐,美女,搞笑幽默
娱乐
新闻趣事,名人明星,广州生活,美食,穿衣美容,娱乐
娱乐
美食
星座命理
旅游,贵阳生活
汽车,军事,文艺,IT 数码,娱乐
汽车,美女
校园生活,IT 数码
体育,娱乐,教育就业,新闻资讯,健康
时尚
搞笑幽默,校园生活,旅游,新闻趣事,新闻资讯,军事,IT 数码,汽车,投资理财
时尚,文艺,健康,教育就业,旅游
健康,旅游,娱乐,文艺
杭州生活,电脑,淡定,汽车,旅游
美食
投资理财,教育就业,星座命理,旅游,文艺,娱乐
游戏动漫,旅游,军事,文艺,体育
星座命理,娱乐,游戏动漫
视频音乐,微博奇葩,新闻趣事,搞笑幽默,云浮生活,健康,娱乐,体育
名人明星
美食,健康,时尚,娱乐,文艺
旅游
新闻资讯
山东生活,普通本科,大学,教育就业
微博奇葩
美容服饰
听歌,视频音乐,宅女加腐女,电影控,动漫控,bigbang,文字,美术
唱 K,懒,发呆,吃货一枚,喜欢听歌,萝莉控,宅女加腐女,美食
宅女一枚,樂意交友,愛虹藍不解釋,吃貨,BINGBANG,學生,90 後
名人明星,娱乐

视频音乐,萌宠,名人明星,星座命理,微博奇葩,娱乐
八卦杂谈,新闻趣事,邯郸生活,星座命理,健康,教育就业,新闻资讯
搞笑幽默,娱乐,星座命理,美食
文艺,美女,星座命理,健康,时尚
美容护肤,摄影爱好者,设计是一种态度,天蝎女,驴友,喜欢听歌,睡懒觉,90後,户外
汽车,新闻资讯,时尚,旅游,健康,美女
旅游,美食
多愁善感,TVB,唱k,o型血,听音乐,爱音乐,浪漫,紫色,互联网,玩儿
娱乐
视频音乐,摇滚,足球,罗本,文艺,IT数码,军事,娱乐,体育
惠州生活
穿衣美容,名人明星,搞笑幽默,时尚,健康,旅游,美食,娱乐
旅游,美食,娱乐
搞笑幽默
时尚,娱乐
文艺,旅游,IT数码,美食,星座命理
健康,旅游
游戏动漫
美食
旅游,名人明星,星座命理,穿衣美容,美女
阳光,学生一枚,小说控,文字,蓝色,狮子座,宅女,吃货,music,90后
美食
文艺,旅游,星座命理,美食,娱乐
苏州生活,旅游,八卦杂谈,视频音乐,搞笑幽默,新闻资讯,时尚,投资理财,军事
旅游,时尚,健康,美食,娱乐
文艺,旅游,美食
旅游,文艺
情感生活
美女
时尚
购物狂,睡觉睡到自然醒,美女,时尚,美食
教育就业,军事,美女,新闻资讯,旅游,体育
健康,时尚
青春,瑜伽,爱自由,睡觉睡到自然醒,玩,爱笑,爱电影,旅游摄影,张杰,Music
美食,旅游
美食,文艺
穿衣美容,爱生活,开心,shopping,拍照,动漫控,美容护肤,摄影师
双鱼女
新闻资讯,美女,军事
苹果控,间接性整理控,美剧,完美主义
娱乐
游戏动漫
星座命理,娱乐,新闻资讯,健康,旅游
健康,旅游,体育,新闻资讯,娱乐,教育就业,美食,文艺

星座命理,体育
游戏动漫,美食,旅游
微博奇葩,文艺
健康,美女,时尚,投资理财,美食
心理学,90后,重口味,热爱生活,懒人,爱吃,微博奇葩
文艺,时尚,旅游,娱乐
汽车,美女
星座命理,时尚
投资理财,IT数码,新闻资讯,健康
美女
瘦身
视频音乐,搞笑幽默,穿衣美容,文艺,教育就业,健康,星座命理,美食
IT数码,健康,美食
旅游,微博奇葩,搞笑幽默,穿衣美容
动漫,娱乐
时尚,IT数码
时尚
教育就业,健康,新闻资讯,旅游,文艺
美容护肤养生,搞笑幽默,微博奇葩,养心,养生,健康,文艺,旅游,美食
时尚,美女,星座命理,IT数码,娱乐
时尚
就业与创业,大学生就业培训,毕业生就业,就业培训,实习就业,就业信息,就业指导,教育
美女
军事,体育
美食
BIGBANG,文艺,娱乐
名人明星
新闻趣事
旅游,游戏动漫
八卦杂谈,体育
体育
时尚,旅游,美食,文艺,娱乐
名人明星,教育就业
美女,游戏动漫,体育,娱乐
健康
军事
微博奇葩,新闻趣事
投资理财,时尚,IT数码
教育就业,投资理财,IT数码
星座运势,台州生活,射手,星座控,文艺,教育就业,汽车,健康,星座命理
旅游,美容服饰,视频音乐,微博奇葩,美食,美女,穿衣美容,搞笑幽默
健康,旅游,星座命理,美食,新闻资讯
简单,迷糊,夜猫子,学生一枚,发呆,听音乐,魔兽,B型血,喜欢旅游,睡懒觉
时尚

时尚,新闻资讯,旅游,星座命理,娱乐
美食,美女,游戏动漫,汽车,IT 数码
健康,文艺,时尚,星座命理,旅游
台湾生活
微博奇葩,搞笑幽默
大连生活,罗姓思想,罗姓名人,罗姓故事,罗姓家族,罗姓文化,罗姓,365 家族网
美食,健康,时尚,文艺,娱乐
穿衣美容,微博奇葩,萌宠,旅游,名人明星
美女
科幻,烹饪,恐怖片,海绵宝宝,游戏动漫,旅游
军事,娱乐,美女,IT 数码
美女
投资理财
军事,文艺
时尚
星座命理,娱乐
萌宠,美食,搞笑幽默,星座命理,文艺,旅游
文艺,旅游,健康
游戏动漫
健康,时尚
新闻趣事,美女,搞笑幽默,美食,微博奇葩,体育
军事
美女,汽车,教育就业,IT 数码,军事,旅游
娱乐
文艺,旅游,娱乐
矛盾体,O 型血,星座控,好性格,紫色,仙剑,萌宠,穿衣美容,星座命理,文艺
美食
穿衣美容
娱乐,美食,健康,时尚
时尚
娱乐
娱乐,旅游,健康,教育就业,萝莉控,杂志,购物狂,懒,北京,上网
搞笑幽默,IT 数码,娱乐
美女,军事,旅游,体育,文艺
IT 数码,教育就业,汽车,新闻资讯,体育
旅游,娱乐
美图摄影
汽车
娱乐
汽车,星座命理,文艺,IT 数码,娱乐
健康
视频音乐
单身一族,休闲,跑步,90 後,粤语,我就是我,白羊座,唱歌,狗狗,工作狂
重庆生活,八卦杂谈,名人明星,新闻趣事,投资理财,娱乐,汽车,美女

旅游,新闻资讯,游戏动漫,时尚,文艺
游戏动漫,体育,旅游
文艺
宠物图集,娱乐
美食,日本语,淘宝店主,日本直邮,日本文化,日本购物,日本代拍,日本代购,时尚,文艺
美食
军事,新闻资讯,投资理财,时尚,旅游,美食,娱乐
投资理财,健康,旅游,美食
汽车
苹果,吉他手,台球,巨蟹座,爱音乐,90後,K歌,科技,冷笑话,财经资讯
教育就业,汽车
新闻资讯,旅游,娱乐
文艺,投资理财,旅游
名人明星,美女,娱乐
旅游,娱乐,体育,汽车,美食
旅游,星座命理,娱乐
体育
旅游,新闻趣事,搞笑幽默,微博奇葩,游戏动漫,教育就业,娱乐,文艺
汽车
时尚,旅游,美食,娱乐
健康,旅游,美食
天秤座,文艺
穿衣美容
上海生活,体育
文艺,旅游,健康,星座命理,投资理财
韩流控,SONE,重庆生活,美食
教育就业,IT数码
旅游,穿衣美容,美女,新闻趣事,山东生活
IT数码,时尚,健康,投资理财,旅游,美食
旅行,摄影,数码,八卦
投资理财,星座命理,军事
旅游,自由,时尚,安静,爱电影,爱旅游
思考,自然,诗词,热爱生活,音乐爱好者,喜欢旅游,魔羯座,探讨人生
投资理财
穿衣美容
娱乐
文艺
美食,娱乐,文艺,星座命理,时尚
旅游
美女,娱乐,IT数码,时尚,旅游,军事,文艺,体育
体育,教育就业,旅游
微博,交朋友,潮人,服饰,music,美食,旅游,健康,时尚,娱乐
穿衣美容,美食,IT数码,健康,旅游,娱乐
穿衣美容,时尚

美女
娱乐
旅游,新闻资讯,投资理财,军事,健康
萌宠,投资理财
教育就业,投资理财,军事,体育,娱乐
萌宠,美食,搞笑幽默,视频音乐,穿衣美容
新闻资讯
视频音乐,校园生活,搞笑幽默,教育就业,游戏动漫,娱乐,体育
汽车
时尚
美女,投资理财
投资理财
交朋友
新闻资讯,娱乐,文艺
校园生活
美女,健康
投资理财,体育,文艺
娱乐,旅游
健康
星座命理,娱乐
体育
星座命理,美食
星座命理,旅游,体育
旅游
游戏动漫
美女,新闻资讯,美食,军事,体育
旅游
体育
IT 数码
教育就业,时尚,健康,旅游,美食
军事,IT 数码,投资理财,汽车,新闻资讯
名人明星,娱乐
大学在校生,英雄联盟,LoL,广告人,活泼开朗,热情,跑步,旅游摄影,游戏动漫
美女
发型设计,美发师,美发造型,形象设计,美容护肤,美发行业,新疆生活
星座命理,微博奇葩,江苏生活,投资理财,时尚,新闻资讯,健康
军事,健康,娱乐,文艺,时尚
台州生活,新闻资讯,文艺,健康,旅游,美食
星座命理
美女,游戏动漫
教育就业,旅游,娱乐,游戏动漫,军事
投资理财,旅游
台湾梦,旅行,睡觉,吃货,苏打绿演唱会,吴青峰,娱乐八卦,康熙,电影,苏打绿
时尚,文艺,旅游,星座命理,美食

健康
搞笑幽默,安徽生活,美食,名人明星
星座命理,时尚,旅游
旅游,美食
健康,教育就业,新闻资讯,美女,旅游,美食
旅游,美食
时尚,汽车,文艺,体育,娱乐
美食
IT 数码
美食
钓鱼,家居,双子,书,教师,拍照,天蝎座,睡觉,123,摩羯
吃货,自恋,朋友,nba,神经大条,证券,旅游,时尚,人生哲学,星座天蝎座
牡丹江,三星专卖店,三星电子,GalaxyS4,Galaxy,投资理财,娱乐,IT 数码
电影演员,歌手,双重性格,感动,听歌,微博控
视频音乐
旅游,萌宠,视频音乐,搞笑幽默,游戏动漫,健康,新闻资讯,教育就业,投资理财,IT 数码
美食,文艺,IT 数码,时尚,健康,旅游
新闻趣事,美食,旅游,视频音乐
视频音乐,搞笑幽默,美女,萌宠,微博奇葩
美食,时尚,娱乐
旅游
体育
IT 数码
搞笑幽默
美女,汽车,体育,旅游,IT 数码
新闻资讯,IT 数码,旅游,体育,90 后,音樂,喜欢听歌,健身,博客,科技
娱乐,旅游,新闻资讯,教育就业,时尚,投资理财
鞍山生活,财经资讯,车世界,美女,文艺,体育,投资理财,娱乐,IT 数码,健康
娱乐,旅游,美食
游戏动漫
视频音乐,懒人,漫画,玩,美食控,吃喝玩乐,新闻资讯,游戏动漫,军事,美食
美食
游戏动漫
安静
射手女,90 后非脑残,瘦子一枚,衣服控,爱萌物,韩剧,美剧,吃货
体育,军事,健康,新闻资讯,旅游
娱乐,视频音乐,名人明星
美食,穿衣美容
音乐发烧友,唱歌,咖啡,情绪化,偏执狂,高中生,猫咪控,娱乐,旅游,文艺
美食,游戏动漫,文艺,星座命理,IT 数码
名人明星
军事,IT 数码,新闻资讯,星座命理,视频音乐,新闻趣事,微博奇葩,搞笑幽默
健康,旅游,星座命理,美食,娱乐
古灵精怪,宅女加腐女,设计师,动漫,新闻趣事,视频音乐,旅游,安徽生活

穿衣美容,游戏动漫
体育
美女,教育就业,美食,IT 数码,音樂,神经质,90 後,星座命理,大学,吃
IT 数码,娱乐,汽车,美女,军事
旅游,北京生活
视频音乐,新闻趣事,新闻资讯,星座命理,娱乐,文艺,体育
名人明星,娱乐
新闻趣事,名人明星,旅游,搞笑幽默,穿衣美容
校园生活,星座命理
健康,美食,美女,娱乐,时尚,新闻,人物
交朋友,努力,巨蟹女,真诚,网购,生活,时装,笑,自然,旅游
投资理财,美女,美食,旅游,娱乐
独立,视频音乐
星座命理
新闻趣事,搞笑幽默,山东生活
动漫,游戏动漫
教育就业,新闻资讯
听歌,电影,美食
娱乐,汽车,旅游,IT 数码,体育
八卦杂谈,女神经病,快乐大本营
娱乐
名人明星
娱乐,星座命理
游戏动漫,娱乐
美女
异国恋,混血宝宝,美剧控,旅游,时尚,美食
视频音乐,萌宠,美食,美女,搞笑幽默
美食,时尚,旅游,文艺,娱乐
星座运势,名人明星
文艺,教育就业,新闻资讯,投资理财,IT 数码
视频音乐,微博奇葩,旅游,搞笑幽默,江苏生活
搞笑幽默,新闻趣事,视频音乐
新闻趣事,视频音乐,微博奇葩,穿衣美容,旅游
新闻资讯
健康,星座命理,美食,娱乐
时尚,IT 数码,娱乐
视频音乐,名人明星
庆典蛋糕,开业花篮,生日鲜花,洛阳鲜花,鲜花速递,生日蛋糕,郑州鲜花,新乡鲜花,洛阳蛋糕,许昌蛋糕
时尚,投资理财,健康
穿衣美容,时尚
穿衣美容
体育
游戏动漫,文艺

室内设计,爱睡觉,爱音乐,90 后,爱笑
校园生活
新闻趣事
视频音乐,旅游,美食,山东生活,星座命理
新闻资讯,体育,娱乐,旅游,IT 数码
新闻资讯,时尚,星座命理,IT 数码,美女,教育就业,健康,旅游,美食
聊天,动漫控,90 后但我不脑残,爱睡觉,写字,美食控,喜欢唱歌,电视,帅哥
娱乐,IT 数码,星座命理
旅游,汽车,教育就业,投资理财,健康
游戏动漫,美女
旅游,投资理财
美女,文艺
名人明星,投资理财
视频音乐,新闻趣事,搞笑幽默,南京生活
游戏动漫,投资理财
萌宠,微博奇葩,美女,搞笑幽默,IT 数码
健康,美食,旅游,教育就业,新闻趣事
娱乐,萌宠,星座命理,新闻趣事,搞笑幽默,云南生活
努力,李宇春,喜欢音乐,淘宝控,萌宠,视频音乐,旅游
汽车
游戏动漫,美女,健康,美食,旅游
IT 数码
美食
名人明星,新闻趣事,穿衣美容,旅游
视频音乐,美女,萌宠,微博奇葩,美食
时尚,美食
美食,游戏动漫,IT 数码
百天照,专业儿童摄影,重庆儿童摄影
游戏动漫
美食,旅游
娱乐,旅游
打篮球,I,can,do,all,things
时尚,娱乐
星座命理,汽车,新闻趣事,微博奇葩,名人明星
旅游
旅游
学生,偶尔胡思乱想,90 後,美食,娱乐
美女,美食
视频音乐
搞笑幽默,旅游,广东生活,新闻趣事,视频音乐
文艺,爱音乐,热爱生活,快乐,爱幻想,阅读,教师,人生,双子座,养生
旅游,财经,宅,旅行,电影,健康
萌宠
美女

视频音乐,娱乐
旅游,文艺
搞笑幽默,江苏生活
娱乐,美食,旅游,新闻资讯,健康
美食,健康,旅游,娱乐
新闻趣事
荷兰式域名拍卖,荷兰式拍卖,域名荷兰式拍卖,域名捡漏,域名淘宝,域名交易,域名投资,域名店,卖域名,域名拍卖
美食,旅游
IT 数码
福建生活,穿衣美容,美食,健康,旅游
旅游,江苏生活
旅游
星座命理,游戏动漫
旅游,汽车
名人明星,星座运势
美女,文艺
美女
张杰,梦想,文艺,娱乐
美食
萌宠,旅游,星座命理,微博奇葩
时尚
科技中专,老师,广东生活,汕头
美食,文艺,娱乐
IT 数码
美食
美食,萌宠,名人明星,旅游
投资理财,旅游,军事,汽车,游戏动漫
穿衣美容
健康,星座命理,娱乐
淘宝,新闻趣事,电子商务,爱旅行,音乐达人,旅游摄影,时尚潮流,网络营销,娱乐,体育
旅游
美女,时尚,IT 数码
旅游,美食,IT 数码,体育,娱乐
体育
腐,懒,金牛,正太控,shopping,死神,听音乐,瓶邪,小提琴,星座命理
健康
健康,旅游
GDRAGON 权志龙,bigbang,taobaoModel,余蕾蕾,天秤座,93 年 9 月 26
健康,时尚,美女,文艺,娱乐
旅游,美食,星座命理,微博奇葩,搞笑幽默,游戏动漫
旅游,淡定,射手女,星星
新闻趣事,山东生活
广东生活

搞笑幽默
新闻趣事,美食,名人明星,校园生活,搞笑幽默,娱乐
时尚
搞笑幽默,新闻资讯,时尚,美食,旅游,娱乐
旅游,美食,时尚,IT 数码,娱乐
美食,旅遊
爱情,陈翔,魏晨,粤语,暮光之城,睡觉睡到自然醒,游戏动漫,文艺,娱乐,星座命理
娱乐,美食
游戏动漫
娱乐,美女
西藏生活,新闻资讯
星座命理,美食,穿衣美容,旅游,搞笑幽默
旅游,新闻资讯,汽车,IT 数码
浙江生活
美女,90 后,手机,旅游
美女
新闻资讯,军事,IT 数码,汽车
广东生活,娱乐
佛山生活,环保,妈妈团,k 歌
厨房,互粉互注,互粉,旅游,美食,健康,教育就业
美食
视频音乐
武汉生活,视频音乐,八卦杂谈,新闻资讯,旅游,汽车,时尚,IT 数码
快乐男声,文艺,歌手,许多年以后,再见吧校园,李佳霖,歌手李佳霖
健康,旅游,教育就业,美食,娱乐
旅游,美食,美女,穿衣美容
健康,文艺,教育就业,旅游,跳舞,考研,自由,看书,唱歌
狗狗,猫,自然,吃货一枚,懒,粉红控,喜欢音乐,开心,爱,旅游
浙江生活
IT 数码
IT 数码,美图摄影,旅游,新闻趣事,搞笑幽默
新闻资讯,军事,教育就业,IT 数码,文艺
天蝎座,听音乐,潮,美食,娱乐
名人明星,视频音乐,新闻趣事,搞笑幽默,娱乐,文艺,星座命理
微博奇葩,美食,视频音乐,萌宠
军事,IT 数码
美食,旅游
策划,舞蹈,画画,音乐,时尚,美容,文艺,人生,历史,穿衣美容
穿衣美容,美女,视频音乐,新闻趣事,美国生活
名人明星,旅游
视频音乐,篮球,音樂,爱玩,美女,旅游,游戏动漫,体育
旅游,汽车,娱乐,文艺
黑龙江生活,美食,穿衣美容,星座命理,搞笑幽默
爱幻想,看小说,动漫迷

微博奇葩,穿衣美容,新闻趣事,搞笑幽默
星座命理,健康
旅游
美食,文艺,IT 数码,旅游,健康,教育就业,投资理财,娱乐
旅游,文艺,娱乐,时尚,健康
星座命理,新闻趣事,美食,旅游,穿衣美容
体育
心理学,码字,多愁善感
星座命理,时尚,旅游,健康,美食
IT 数码
旅游
营口生活,现货交易,金融投资
游戏动漫,旅游,星座命理,体育,娱乐
星座命理,体育
教育就业,健康,旅游,文艺,娱乐
摄影,美容护肤,美发造型,时尚
旅游
吃货一枚,我爱榆林,榆林美食,榆林小吃,爱打折,美食达人,精明消费,美食攻略,时尚潮流,娱乐
体育,美食,文艺,星座命理,旅游
旅游,名人明星,江苏生活,美女,穿衣美容
搞笑幽默
新闻趣事
星座命理
娱乐,新闻资讯,军事,体育
娱乐,IT 数码
美食
微博奇葩,星座命理,新闻趣事,搞笑幽默,美女,喜欢音乐,疯疯癫癫
其他生活
美女
美食,游戏动漫
穿衣美容,时尚
名人明星,游戏动漫
美女,体育,娱乐,游戏动漫
搞笑幽默,学生一枚,教育就业,时尚,健康,旅游,美食
视频音乐,音乐爱好者,娱乐
旅游,微博奇葩,穿衣美容,视频音乐,香港生活
广东生活
摄影,电脑,运动,偶喜欢玩,爱旅行,美女
美食
美女,视频音乐,新闻趣事,搞笑幽默,河南生活
彩妆学习星人,囤货狂,底妆控,护肤控
娱乐
美女

娱乐,汽车,旅游,美食,文艺
旅游,美女,文艺,IT 数码,娱乐
时尚,娱乐,健康,美食,星座命理
美女,汽车,体育
游戏动漫,旅游
美食,IT 数码
健康,美女,汽车,体育
旅游,星座命理,美食,娱乐
美食
旅游,时尚,教育就业,IT 数码,文艺,健康,美食
旅遊,名人明星
萌宠
视频音乐,微博奇葩
搞笑幽默
微博奇葩,搞笑幽默,新闻资讯,时尚,美食,文艺,娱乐
穿衣美容
视频音乐
IT 数码
星座命理
天津生活,旅游,名人明星,美食,星座命理
穿衣美容,娱乐
创业,就业,创业指导,大学生就业,就业信息,就业指导
美图摄影,美食,上海生活,美容服饰,旅游,穿衣美容
体育资讯,视频音乐,搞笑幽默,名人明星,新闻趣事
美食
时尚,美女,汽车,旅游,娱乐
旅游美食爱好者,欧美音乐,健康,时尚,美食,文艺,娱乐,旅游
穿衣美容,旅游,微博奇葩,新闻趣事,梅州生活,健康,教育就业,娱乐
吃货一枚,80 后的尾巴,自恋,美容,军装控,喜欢音乐,幻想
电影控,文艺,IT 数码,军事,美食,健康
名人明星,娱乐
美女,汽车,投资理财,军事,体育
Dior,MHS 奢侈品,奢侈品牌,奢侈品代购,高仿奢侈品,chanel,Celine,复刻奢侈品,奢侈品狂热者,爱马仕
健康
名人明星,高雄丁小雨,蔡宛臻 Angela
美女,游戏动漫,时尚,文艺,体育
美食,旅游
手机控,巨蟹座,偶稀饭睡觉觉,真诚,旅游
视频音乐,微博奇葩,新闻趣事,搞笑幽默,广东生活
游戏动漫,军事
小杨幂
名人明星,泉州生活,八卦杂谈,娱乐
健康,旅游,美食

旅游
旅游
时尚,娱乐
娱乐
时尚,汽车,旅游,星座命理,娱乐
教育就业,投资理财,文艺
文艺,体育,娱乐
美食,时尚,健康,旅游,娱乐
文艺,时尚,健康,美女,美食
美食
旅游
旅游,新闻资讯,美食,美女,汽车,投资理财
星座命理,教育就业,健康,旅游,体育
文艺,美女,游戏动漫,时尚,旅游,美食,娱乐
文艺,时尚,旅游,新闻资讯,体育
文艺,韩庚,强迫症
搞笑幽默,美女
健康
健康,汽车,新闻资讯,娱乐,文艺
A 型血,diy,喜欢旅游
文艺,无厘头,学生一族,Music,唱 K,文字控,情绪化
绵阳生活,吐槽,校园食堂,表白墙,校园百态,新鲜传媒,兼职,学生,绵阳校园,新闻资讯
美女
穿衣美容,化妆品,美容,美容护肤,健康,时尚
文艺
美女
微博奇葩,美食
福建生活,搞笑幽默,投资理财,新闻资讯,健康,娱乐,文艺
娱乐
时尚,汽车,IT 数码
文艺
搞笑幽默
视频音乐
投资理财
新闻趣事
新闻资讯,健康,旅游,美食
微博奇葩
旅游
美女,视频音乐,美食,收藏,茶,杂志,旅游
时尚,健康,美女,娱乐
新闻资讯
娛樂
教育就业,时尚,健康,旅游,体育
穿衣美容,IT 数码,时尚,汽车,健康,投资理财

网络,手机,美食,时尚,投资理财,健康
微博奇葩,星座命理
新闻资讯,旅游,军事
娱乐
时尚,美食,健康
旅游,搞笑幽默
海外生活
新闻趣事,美食
美食,旅游,美女
新闻资讯,旅游,健康,美食,娱乐
新闻趣事
旅游,娱乐,体育
旅游,新闻趣事,美食
时尚达人,生活,短信营销,会员短信,短信群发,健康,投资理财,IT 数码,美女,汽车
教育就业,投资理财,美食
美女
文艺,美女,新闻趣事,江苏生活,搞笑幽默,美食
穷游,养花,音乐,阅读,晒晒太阳,美食,旅游,新闻资讯,时尚,文艺
闷骚,重口味女青年,丧尸控,月光光,猫奴
视频音乐,搞笑幽默,北京生活,穿衣美容
健康
视频音乐,贵州生活
时尚
新闻资讯,美女,旅游,IT 数码,体育
美女
新闻趣事,旅游
新闻资讯,投资理财,旅游,娱乐,文艺
投资理财,旅游,健康
旅游,健康,体育,游戏动漫,汽车
美食
好玩,漂流,旅游
萌宠
美女
娱乐
情绪化,新疆生活
搞笑幽默,八卦杂谈,来宾生活
星座命理
星座命理,美食,时尚,新闻资讯,美女
新闻资讯,健康,教育就业,美食,旅游
旅游
校园生活,会计,娱乐
胡思乱想,休闲,杭州,天枰座,平面设计,90 后但我不脑残,咖啡控,玩儿,健康,时尚
美食
拍照,篮球爱好者

美食,浙江生活
投资理财,新闻资讯,旅游,美食,娱乐
旅游,穿衣美容
搞笑幽默,微博奇葩,穿衣美容
娱乐
遊戲動漫,瀕死狀態,MAGI
穿衣美容
時尚
美食
娱乐,美食,旅游,汽车,体育
美食爱好者,爱音乐,爱笑,安静,猫,IT 数码,汽车,旅游,娱乐
旅游,体育
时尚,娱乐,旅游,美食,文艺
微博奇葩,新闻趣事,旅游,搞笑幽默,云南生活
文艺
动画片,动画电影,3D,原创,动画,搞笑幽默
美食
游戏动漫,时尚,美女,文艺,体育
娱乐,四川生活
星座命理
美食,新闻趣事,西安生活,视频音乐,搞笑幽默,时尚,旅游，读书。国画
互粉,摄影,旅游摄影,时尚
体育
体育,娱乐,健康,心理,爱自由,设计师
新闻资讯
旅游,健康,体育
遊戲動漫,美食,文藝
视频音乐
新闻趣事
搞笑幽默
IT 数码,新闻资讯,投资理财,娱乐
微博奇葩,搞笑幽默
名人明星
美女,穿衣美容
汽车,美食,旅游,健康
80 后,魔兽世界,萝莉控
搞笑幽默
美女,美食,旅游,健康,新闻资讯
麦霸,矛盾体,胡思乱想,大学生,睡懒觉
瑞典生活,海外生活
星座运势,美图摄影,新闻趣事,美容服饰,漯河生活,微博奇葩,穿衣美容,河南生活
双鱼座的我,很快乐,时尚,娱乐,旅游,美食
星座命理,新闻资讯,美食,时尚,娱乐
淘宝,acg,动漫迷,电影迷,电脑,冷笑话,喜欢听歌,IT 数码,美女,游戏动漫

新闻资讯
教育就业
旅游,名人明星,山东生活,新闻趣事,搞笑幽默
美女,美食,娱乐,文艺,IT 数码,军事
穿衣美容,文艺
穿衣美容
视频音乐,搞笑幽默
读书分享,搞笑幽默,娱乐,文艺,健康,旅游,美食
搞笑幽默
旅游,广东生活
周杰伦,包包控,McCartney,Stella,旅游,娱乐,星座命理,美女,时尚,名人明星
新闻趣事,广东生活,星座命理
美食,娱乐
视频音乐,娱乐,旅游,美食
旅游
旅游,娱乐
穿衣美容,时尚
旅游,视频音乐,搞笑幽默,美食,星座命理
旅游,视频音乐,新闻趣事,搞笑幽默,南昌生活,IT 数码
教育就业,旅游,文艺,单身,喜欢旅游,微笑,茶,宅女一枚
旅游,时尚,美食,IT 数码,娱乐
名人明星
美食,娱乐
旅游
美食,搞笑幽默,上海生活,旅游,视频音乐
旅游
视频音乐
投资理财,体育,娱乐,美女,新闻资讯
名人明星
旅游,新闻资讯,美食,娱乐,游戏动漫,星座命理
星座命理,旅游,美食,时尚,娱乐
旅游
健康
湖北生活
体育
天真
美食,旅游
无纺布袋,钛金标牌,铜制标牌,铝制标牌,丝印标牌,蚀刻标牌,金属标牌,标牌
视频音乐
名人明星
微笑,执着,感性,奋斗,好性格,处女座,散步,主持,视频音乐
气质高贵
文艺
星座命理

微博奇葩,芜湖生活,旅游,星座命理,娱乐,体育
视频音乐
体育,时尚,旅游,IT 数码,文艺
美图摄影,新闻资讯
微博奇葩,视频音乐,新闻趣事,穿衣美容
搞笑幽默,河南生活,旅游,萌宠,微博奇葩
湖北宜昌,吃喝玩乐,美食达人,旅游
小时代
新闻资讯
时尚
时尚,旅游,美食,IT 数码,娱乐
名人明星,美女
嘉兴生活
90 後,天蝎座
时尚,健康,星座命理,美食,游戏动漫
打篮球,吸血鬼日记,IT,好书,互联网,旅游,美食,文艺,教育就业,体育
旅游,美食,四川生活,微博奇葩,星座命理
萌宠,游戏动漫
穿衣美容
美食,文艺,健康,军事,体育
安静,舞蹈,交友,运动,旅游,美食,穿衣美容
旅游,文艺,娱乐
篮球,室内设计,设计师,狮子座,新闻资讯,娱乐,健康,文艺,体育
旅游
宠物图集,新闻趣事,萌宠
星座命理
发呆,看电影,爱音乐,新闻趣事,美食
80 后,学生一族,懒人,爱吃,爱幻想,可爱,朋友,k 歌,android,小资
旅游,天津生活
IT 数码,健康,文艺,时尚,星座命理,娱乐
微博奇葩,萌宠,搞笑幽默,新闻趣事,北京生活
健康,旅游,美食
美女,汽车,娱乐
军事
搞笑幽默,旅游,星座命理,微博奇葩,视频音乐
美瞳贩子
新闻资讯,健康,旅游,美食
萌宠,星座命理,微博奇葩,视频音乐,名人明星
时尚,星座命理,健康
要活的漂亮
穿衣美容
星座运势,懒人
音乐控,吃货一枚,唱歌,旅游,美食
投资理财,星座命理,时尚,旅游,美食

名人明星
体育
新闻趣事,搞笑幽默,微博奇葩
旅游摄影,环保,教育就业,育儿,生活,健康,新闻资讯,文艺
美容服饰
时尚
湖北生活,萌宠,新闻资讯,健康,星座命理,旅游,美食
健康,IT 数码,军事,美女,娱乐
搞笑幽默
新闻资讯,健康
萌宠,爱旅行,摩羯座,学生,美食控,小清新,白羊座,时尚,娱乐
IT 数码
美食,美女
旅游
视频音乐
星座命理
体育,新闻资讯,旅游,娱乐,美食
杭州生活
穿衣美容
名人明星
文艺,军事
文艺,投资理财,星座命理,娱乐
体育
珠海生活,娱乐
新闻趣事,旅游,微博奇葩,北京生活
清教徒,新教徒,普通外科,医学生,神学,基督徒,旅游
天津生活,美女,娱乐,文艺
美食
名人明星,视频音乐
军事,体育,文艺,IT 数码
星座命理,美食,健康,旅游
名人明星
萌宠,安徽生活,抓周宴,满月酒,宝贝庆生,生日派对,合肥,亲子关系
视频音乐
微博奇葩,文艺
时尚,美食,旅游,彩妆造型,旅游摄影美食,美容护肤
时尚,投资理财,文艺,穿衣美容,新闻趣事,星座命理,旅游
美女
美食,旅游,穿衣美容,星座命理,健康,教育就业,投资理财
健康,旅游,美食,文艺,娱乐
爱情公寓,电影迷
教育就业,健康,美食,文艺,娱乐
新闻资讯,健康,旅游,时尚
游戏动漫

投资理财
教育就业,健康,旅游
星座命理,美女
菲律宾生活
广州生活,文艺
内蒙古生活
搞笑幽默
娱乐,舞蹈,贵阳,hiphop,breaking,jazz,CD 舞蹈工作室
新闻趣事,穿衣美容,微博奇葩
恐怖片,新疆生活
美女,文艺,教育就业
文艺,美女,娱乐
爱笑,逛街,爱旅游,喜欢听歌,穿衣美容
视频音乐
搞笑幽默,健康,投资理财,时尚,娱乐
名人明星
向日葵,穿衣美容,LOL 英雄联盟,多啦 A 梦,互粉,荧光控,自拍,可爱,吃货
娱乐
时尚,健康,美女,美食,文艺
体育
娱乐,旅游,新闻资讯,时尚
视频音乐,搞笑幽默
新闻趣事,微博奇葩,美女
星座命理,美食,文艺,娱乐
广东生活,搞笑幽默,IT 数码,投资理财,星座命理,美食,美女
美食,穿衣美容
旅游,视频音乐,陕西生活
娱乐
搞笑幽默,旅游,视频音乐,美食,江苏生活
游戏动漫,IT 数码,新闻趣事
时尚,健康,星座命理,旅游,美食
星座命理,微博奇葩,新闻趣事
学生一枚
投资理财,时尚,新闻资讯,美食,娱乐
美食
健康,美食,IT 数码,娱乐,体育
美食,90 后但我不脑残,阅读,读书,留学生,自行车
搞笑幽默
宠物图集
新闻资讯,健康
搞笑幽默
唱 K,爱笑,创业,搞怪,喜欢音乐,爱吃,影视,服装,视频音乐,穿衣美容
纠结,执着,摩羯座,懒,手机看小说,吃喝玩乐,耽美,旅游美食爱好者,美食
安徽生活

星座命理,旅游,美食
情感生活,四川生活
文艺,美食
新闻资讯
星座命理,微博奇葩,搞笑幽默,新闻趣事,北京生活
时尚,美女,星座命理,文艺
情感,笑话,原创,博客,寂寞,互联网,八卦,美女,搞笑幽默,新闻趣事
微博奇葩,美食,旅游,穿衣美容,新闻趣事
视频音乐,微博奇葩,搞笑幽默,健康,旅游,星座命理,娱乐
美食,娱乐,健康,时尚,旅游
健康,美食
视频音乐,浙江生活,美食,旅游,穿衣美容
旅游,院感无小事
搞笑幽默,旅游,娱乐
时尚,教育就业,美食,娱乐
实习,校园招聘,实习生,暑期实习,暑期实习生,银行招聘,企业招聘,教育就业
新闻趣事,西安生活,娱乐
北京生活
微笑,帅哥,快乐,爱旅行,喜欢旅游,睡觉睡到自然醒,旅游,IT 数码
文艺
美图摄影,小说,喜欢音乐,旅游
福建生活
新闻资讯,健康,文艺,美食,旅游
穿衣美容,美食,广东生活
旅游,时尚,健康,星座命理,美食,文艺
视频音乐,搞笑幽默,美女,旅游
时尚,健康,美食,文艺,体育
星座命理,穿衣美容,教育就业,投资理财,旅游,健康,美食
浙江生活,文艺,汽车,新闻资讯,IT 数码,娱乐
微博奇葩,视频音乐,新闻趣事,搞笑幽默,美女
山东小伙,西电研究生,计算机视觉,深度学习
北京生活
美女,江苏生活,搞笑幽默,投资理财,IT 数码
文艺,星座命理
名人明星,新闻趣事,搞笑幽默,微博奇葩,穿衣美容
美食,文藝
搞笑幽默,汽车,文艺
搞笑幽默,穿衣美容,星座命理,萌宠,视频音乐
其他生活
金融,科技,爱旅游,笑,创意,胡思乱想,文艺,新闻资讯,美食
新闻资讯,军事,IT 数码,娱乐,体育
名人明星
美女,娱乐
文艺

移民,文化,旅游,香港
连接服务器失败,les,囧,宅女一枚,腐女
娱乐
穿衣美容,娱乐
星座命理,健康,IT数码,娱乐,时尚
星座命理,美食,名人明星,视频音乐
视频音乐,旅游
新闻趣事
美图,美景,摄影,設計師,music,爱生活,时尚
宠物图集,你觉得呢
微博奇葩
美食,投资理财
美食
黑花,瓶邪,勋鹿,灿白
动漫,旅游,情感生活,宠物图集,微博奇葩,萌宠,视频音乐
旅游,爱电影,阳光,咖啡,爱美食,购物,博客
视频音乐,娱乐
美食,时尚,新闻资讯,健康,旅游
搞笑幽默,健康
美食
微博奇葩
搞笑幽默,娱乐
淘宝控,淘宝店主,搞笑幽默,萌宠,微博奇葩
美食
时尚
微博奇葩,新闻趣事,搞笑幽默,鞍山生活,娱乐
星座命理,耽美
白羊,学习,广州,娱乐,IT数码,新闻资讯,时尚,旅游
投资理财,旅游,新闻资讯,教育就业,体育
搞笑幽默,旅游,视频音乐,穿衣美容,香港生活
星座命理
山东生活
新闻资讯,美食,军事
陕西生活
美女
育儿,电视剧,舞蹈,减肥,80後,金牛,娱乐
游戏动漫
美女
90後
美容服饰,新闻趣事,美食,旅游,搞笑幽默,瘦身
洛阳生活,旅游
美食,微博奇葩,新闻趣事,搞笑幽默,其他生活
体育
视频音乐,微博奇葩,搞笑幽默,名人明星

健康
高中生,视频音乐,穿衣美容,娱乐
爱生活,自信
游戏动漫,亦都钟意啦啦队,钟意打机
搞笑幽默,向日葵
美食,健康,旅游,文艺
萌宠,星座命理,美女
搞笑幽默
IT 数码,健康,教育就业,军事,新闻资讯
吃货一枚,上网,动漫,睡觉,宅,文学,金牛座
名人明星,新闻资讯,文艺,健康,旅游,美食
美食
旅游,微博奇葩,视频音乐,搞笑幽默
萌宠
新闻资讯,时尚,IT 数码,旅游,美食
游戏动漫
星座命理
娱乐,时尚
时尚,星座命理,军事,娱乐
武汉生活,健康,时尚
广东生活
星座命理,军事
娱乐,美食,健康
健康,旅游,新闻资讯,教育就业,文艺,美食,娱乐
活泼开朗,时尚,美女,旅游
山东生活,微博奇葩,新闻趣事,搞笑幽默,吃货,乐观,体育,游戏动漫,美食
90,校园生活,健康
时尚,投资理财,旅游,体育,娱乐
体育,IT 数码,美食,新闻资讯
美女,汽车
文艺
性别是韩吉,变态突破天际,高级绅士,hentai,大丧尸,脑残生化饭,怪蜀黍,宅基腐
旅游,星座命理,投资理财,美食
视频音乐
星座命理,视频音乐,美女,新闻趣事,江西生活,汽车,投资理财,新闻资讯,文艺,体育
时尚达人
狮子座,玩儿,90 后,学生一族,寂寞
美食,文艺,投资理财,时尚,健康,旅游
体育
健康,旅游,文艺,娱乐
游戏动漫
微博奇葩,新闻趣事,搞笑幽默
萌宠,美女
遵义生活,游戏动漫

旅游,美食,教育就业,娱乐
时尚,教育就业,投资理财,星座命理,美食,军事
美食,萌宠,微博奇葩,新闻趣事,搞笑幽默
娱乐
新闻趣事,美食,旅游,江苏生活
汽车,旅游,体育,娱乐,军事
时尚,旅游,星座命理,美食,娱乐
美食,美女,时尚,娱乐
娱乐,星座命理,时尚,美食,文艺
星座命理,时尚
微博奇葩,搞笑幽默,广告,媒体,文艺
穿衣美容
萌宠,美食,星座命理
IT 数码,游戏动漫,微博奇葩
军事,旅游,教育就业,体育,文艺
搞笑幽默
爱笑,爱电影,90 後,旅游,搞笑幽默
美食,旅游,星座命理
美食,视频音乐,搞笑幽默,汽车,美女,健康,旅游,军事
教育就业
思考,幸福,打篮球,浪漫,学生一族,大学生,人生,文艺,军事,教育就业
公益热线,医学,江苏生活
健康,旅游,IT 数码,时尚
旅游,美食
名人明星
互粉,吃喝玩乐,美女,微博奇葩,搞笑幽默
湛江生活
香港生活
星座命理,美女
健康,美食,文艺,时尚,娱乐
完美主义,户外,文艺,旅游,星座命理,IT 数码,娱乐
美食,健康
娱乐,旅游,时尚
文藝,健康,旅遊,星座命理
汽车,时尚,旅游,IT 数码,娱乐
新闻资讯,娱乐
旅游
军事
新闻资讯
视频音乐,搞笑幽默
旅行,宅,购物
娱乐,健康
时尚,娱乐,美食,IT 数码,健康
美食

旅游
文艺, 娱乐
名人明星
娱乐
强迫症, 各种懒
星座运势
微博奇葩, 视频音乐, 萌宠, 美女, 搞笑幽默
游戏动漫, 投资理财, 新闻资讯, IT 数码, 美食
美食
星座命理, 旅游, 美女, 穿衣美容, 美食
健康
美食, 体育
文艺, 健康, 旅游, 美食
减肥, 吃货一枚, 喜欢听歌
美食
美食, 旅游, 新闻趣事, 视频音乐, 搞笑幽默
公务员, 奋斗, 学习, 文艺
微博奇葩, 搞笑幽默, 新闻趣事, 视频音乐, 星座命理, 真诚, 网球
阳光, 爱旅行, 魔羯座, 吃喝玩乐, 执着, 漫画, 街拍, 疯子, 户外, 美食
星座命理
娱乐
美女, 微博奇葩, 搞笑幽默
旅游, 新闻趣事, 穿衣美容, 微博奇葩, 广东生活, 娱乐, 时尚, 新闻资讯, 健康, 投资理财
萌宠, 新闻趣事, 微博奇葩, 湖南生活, 美女
矛盾体, 篮球, 电影爱好者, 神经大条, 美食
二代东方神起, 允浩在中昌珉, 邪教中心魔教, 日剧, 日语, 摄影控, 美剧
新闻趣事, 旅游
新闻资讯, 汽车, IT 数码, 体育, 旅游
搞笑幽默
旅游, 星座命理, 美食, 文艺
名人明星, 娱乐
向日葵, 旅游, 美食, 搞笑幽默
新闻趣事
时尚
名人明星, 文艺
旅行, 时尚, 数码, 宅
淘宝, 网购, 青春, 搭配, 女装, 潮流服饰, 美女, 时尚
历史, 八卦, 好书, 电子商务, 健康, 文艺, 娱乐, 星座命理
健康, 时尚, 旅游, 文艺, 娱乐
视频音乐, 娱乐, 文艺, 体育
娱乐
教育就业, 星座命理, 美食
读书分享, 美容服饰, 名人明星, 八卦杂谈, 新闻趣事
旅游, 教育就业, 文艺

穿衣美容,新闻趣事,娱乐,美食,星座命理,旅游,健康
旅游,名人明星,视频音乐,美食
微博奇葩
自行车,唱K,旅游
旅游,大学生兼职,创业
娱乐,教育就业,投资理财,旅游,新闻资讯
穿衣美容,娱乐,文艺,时尚,游戏动漫
新闻资讯,教育就业,时尚,IT数码,旅游
健康,星座命理,新闻资讯,教育就业,投资理财
美食
情绪化,购物狂,拍照
旅游,时尚,健康
庚澈是伤口,青峯小男神,观自在,lukris❤krislu,鹿爷是攻,凡凡小公主,晗晗大宝贝
穿衣美容,文艺
新闻趣事,旅游
娱乐
游戏动漫,旅游,美食,IT数码,娱乐
广东生活,IT数码,旅游,教育就业
新闻趣事,旅游
旅行,时尚,摄影,八卦,购物
萌宠,健康,体育
搞笑幽默,投资理财,汽车,新闻资讯
新闻趣事
美女,微博奇葩,旅游
搞笑幽默
游戏动漫
名人明星,旅游,微博奇葩,穿衣美容
搞笑幽默,旅游,新闻资讯,文艺,游戏动漫
美食
旅游,视频音乐,美食,微博奇葩
教育就业,汽车,健康,旅游,美食
游戏动漫,美食
视频音乐,新闻趣事,微博奇葩,宁夏生活
美女
美食,北京生活
娱乐
视频音乐,旅游,美女,微博奇葩
投资理财,时尚,健康,美食,娱乐
美食
名人明星,游戏动漫
穿衣美容,健康
军事
星座命理
旅游,教育就业,汽车,军事,娱乐,IT数码

汽车,美女
旅游
旅行,时尚,摄影,娱乐,八卦
音乐,学习,爱情,表白,星座命理
美食
美容护肤,shopping,淘宝,网购,搭配,配饰,潮流服饰,女装,穿衣美容
北京生活,IT 数码
健康,旅游,投资理财
新闻趣事,上海生活
名人明星,时尚
汽车,旅游,文艺,体育,IT 数码,时尚,星座命理,美食
视频音乐,星座运势,美食,名人明星,军事,IT 数码,文艺,体育,娱乐
IT 数码
星座命理
美食
旅游,汽车,体育
时尚
美食,旅游,星座命理
穿衣美容,时尚
体育
吃货
微博奇葩,IT 数码
星座命理
文艺,投资理财,军事,新闻资讯,美女
搞基,健身达人,有氧舞蹈,瑜伽导师,健身教练,舞蹈,同志,旅游
美女
美女
新闻资讯,健康,教育就业,娱乐,IT 数码
电子商务,男人,旅游
美女
视频音乐
美女
旅游,美食,投资理财,健康,军事
投资理财,文艺,体育,娱乐
旅游,教育就业
健康,旅游,娱乐
视频音乐,搞笑幽默,四川生活,新闻趣事
微博奇葩,北京生活,萌宠
旅游,美女,搞笑幽默,新闻趣事,视频音乐
微博奇葩,搞笑幽默,新闻趣事,旅游,新疆生活
微博奇葩,新闻趣事,甘肃生活,搞笑幽默
娱乐
浙江生活,教育就业
旅游,汽车,教育就业,投资理财

健康
文艺
广州生活,旅游
星座命理,旅游,美食,美食
教育就业
教育就业,军事,IT 数码
文艺,投资理财,时尚,旅游,美食
江苏生活,微博奇葩,视频音乐,搞笑幽默,新闻趣事
赚钱,旅游,名人明星,美女,视频音乐,新闻趣事,搞笑幽默
旅游
名人明星,搞笑幽默,美食,穿衣美容
健康,星座命理,旅游,美食,美女
IT 数码,旅游,美食,健康
美食,校园,娱乐,星座命理,时尚,文艺,旅游
时尚,体育,新闻资讯,健康,文艺,IT 数码
搞笑幽默,新闻趣事,星座命理,微博奇葩,江西生活
北京生活,升学辅导,提分顾问,1 对 1 课外辅导,教育培训,亲子关系,家长学堂,寒暑假乐园,
教育就业
新闻资讯
体育,美女,教育就业,汽车,娱乐
新闻趣事,文艺
旅游,美食
游戏动漫,娱乐,旅游
蛋纸商务,我是香飘飘,我不是优乐美,都说中分是女神,待业中,舟山普陀
微博奇葩
穿衣美容,星座命理,美食,旅游,泉州生活,教育就业,文艺
娱乐
大学副学士,副学士学位,香港副学士,香港留学,教育就业
旅游,星座命理,微博奇葩,新闻趣事,搞笑幽默,游戏动漫
美女
旅游,健康
好性格,搞笑幽默,新闻趣事,星座命理
美女,时尚,旅游,IT 数码
上网,旅游,外汇,证券,投资理财
旅游,健康,投资理财,美食,娱乐
穿衣美容
游戏动漫
穿衣美容
美女,穿衣美容,视频音乐,新闻趣事,搞笑幽默,娱乐,文艺
搞笑幽默
星座命理
娱乐,体育
萌宠,美女
搞笑幽默

穿衣美容,搞笑幽默,星座命理,新闻趣事,广东生活
陈翔橙,陈翔控,格子控,偶稀饭睡觉觉,学生一族,活泼,阳光,游戏动漫,美食,娱乐
IT 数码,健康,时尚,军事,文艺,体育
萌宠,微博奇葩,星座命理,穿衣美容,搞笑幽默
教育就业,投资理财
娱乐
校园生活,搞笑幽默
时尚潮流,爱生活,微博控,淘宝,购物,网络营销,愤青,淘宝控,购物狂,奋斗
GEEK,动漫,自由,宅
美女,IT 数码,娱乐,体育,星座命理
美女,娱乐,新闻资讯,美食,投资理财
教育就业,文艺,旅游,健康,时尚
新闻趣事,文艺
文艺,时尚
体育
军事,文艺
乐橙,浪漫,温柔,魔兽,武术,健康,星座命理,旅游,军事,娱乐
美食,旅游
新闻趣事,微博奇葩
美食,健康,时尚,文艺,娱乐
美食,时尚
90 後,侦探推理,海贼王,创意,户外,看书
旅游
旅游,名人明星,视频音乐,新闻趣事,微博奇葩
名人明星
健康,时尚,美食,娱乐
视频音乐,搞笑幽默,娱乐,美女,美食,旅游,文艺
IT 数码,投资理财,教育就业
娱乐,文艺,旅游,健康,美食
新闻资讯,汽车,投资理财,文艺,IT 数码
健康,美食,娱乐,时尚
美食
旅游,美食,萌宠,浙江生活,穿衣美容
搞笑幽默,军事天地,新闻趣事,财经资讯,成都生活
星座运势,名人明星,体育资讯,八卦杂谈,搞笑幽默
美女
旅游
健康
旅游
视频音乐,新闻趣事,搞笑幽默
天枰座,台州生活,美女,吃货一枚,大学,活泼开朗
萌宠,福建生活,游戏动漫,旅游,美食,娱乐,体育
穿衣美容,美食
星座命理,美食,投资理财,娱乐

绵阳生活,文艺
美女
美食,旅游,娱乐
美容服饰,育儿百科,读书分享,新闻趣事,搞笑幽默,健康,时尚
旅游,美食,娱乐
动漫,古川雄辉,日剧控,五月天,文字控,康熙来了
旅游,文艺,时尚,美食,娱乐
教育就业,IT 数码
健康,星座命理,时尚,投资理财,美食
美女,教育就业,投资理财,娱乐,IT 数码
视频音乐,星座命理,名人明星
音樂,爱自由,梦想,看电影,旅游
情感生活,美容服饰,读书分享,视频音乐,军事,教育就业,新闻资讯,时尚,美食
视频音乐,校园生活,新闻趣事,搞笑幽默,美女
旅游,穿衣美容,时尚,新闻资讯
游戏动漫
健康
新闻资讯,汽车,旅游,军事,娱乐
美女,搞笑幽默,萌宠
旅游
美女,体育
投资理财,教育就业,新闻资讯,文艺,军事
美食,微博奇葩,四川生活,旅游
旅游
穿衣美容,娱乐
娱乐,健康,旅游,新闻资讯,时尚,美食
微博奇葩,搞笑幽默,天津生活,萌宠
娱乐,时尚,体育,汽车,旅游
教育就业
美女
自由,唱歌,摄影,上网,娱乐
安静,快乐,神经质,爱旅游,淘宝控,懒人,娱乐
葡萄酒品鉴,进口红酒,葡萄酒文化,进口葡萄酒,美食美酒,意大利,红酒,苏州,美食
旅游,教育就业,美食,健康
新闻资讯,健康,时尚,旅游,美食
旅游
星座命理,穿衣美容,美食,旅游,游戏动漫,美女,娱乐
美女,IT 数码,体育,娱乐
旅游,娱乐,星座命理
上网,天蝎座,看小说,音乐迷,文艺,体育,星座命理,美女,游戏动漫
健康,旅游,美食,投资理财
健康
安静,奋斗,乐观,运动,简单
美食,时尚,娱乐

美食,微博奇葩,穿衣美容,新闻趣事,搞笑幽默
微博奇葩
文艺,时尚,娱乐
美国生活,时尚
汽车
时尚,旅游,美食,体育,文艺
美女,时尚,体育,娱乐
新闻资讯,旅游,文艺,时尚,娱乐
搞笑幽默,旅游,穿衣美容,星座命理
视频音乐,微博奇葩,游戏动漫,娱乐
互粉,阳光,娱乐,旅游,美女,美食,新闻资讯
简单,感性,执着,摇滚,投资,美女,微博奇葩,搞笑幽默,视频音乐,新闻趣事
旅游
名人明星,健康,星座命理,娱乐
微博奇葩,新闻趣事,搞笑幽默
校园生活,爱美食,爱听歌,爱微笑,爱时尚,爱自由,爱生活
历史,交友,书法,营销,武术,看书,互联网,新闻,美女,旅游
教育就业,健康
時尚,汽車,健康,體育,娛樂
美女,旅游,军事,文艺,体育
娱乐
旅游,游戏动漫,美女,IT 数码,文艺
文艺
美女,旅游,美食,军事,IT 数码,活泼开朗,自由
看小说,爱幻想,90 后,听歌,旅游,时尚,美食,名人明星,穿衣美容,搞笑幽默
美女,娱乐,文艺
心情,90,爱生活,娱乐
美食,教育就业
广东生活,微博奇葩,新闻趣事,搞笑幽默,新闻资讯
美女,美食
上海生活,微博奇葩
广东生活
搞笑幽默
美食,娱乐
微博奇葩,穿衣美容
新闻趣事,体育资讯,名人明星
新闻趣事
美鞋控,好性格,自由职业,时尚达人,潮流,自由,时尚,宅,美女
体育
美女,娱乐
旅游
美女,完美主义,强迫症,宅,文艺
游戏动漫
美女,微博奇葩,旅游,新闻趣事,河南生活

时尚,健康,投资理财,美食,娱乐
萌宠
宅,90后,狮子座,简单,强迫症,学生一枚,娱乐,美食
江苏生活,视频音乐
星座命理,娱乐
美女,广东生活
投资理财,新闻资讯,美女,旅游,星座命理,IT数码
死飛,女汉子,街舞hiphop,音乐
娱乐
穿衣美容,娱乐
娱乐
美女
娱乐,时尚,旅游,投资理财,汽车
搞笑幽默,穿衣美容
投资理财
随性,腹黑,矛盾体,天秤座
娱乐,健康,旅游
老子信了你的邪,掉节操,棒球
旅游
健康
游戏动漫
时尚,娱乐,健康,投资理财,旅游,美食
合肥生活
江苏生活,健康
美图摄影,投资理财
游戏动漫,星座命理,娱乐,文艺
美食
美食,旅游,健康,时尚
娱乐,美女,时尚,文艺
旅游
穿衣美容,星座命理
體育,汽車,軍事,IT數碼,娛樂
萌宠
健康,美食,娱乐,旅游
K歌,科比,劉德華,投资理财,新闻资讯
时尚,游戏动漫,新闻资讯,健康,旅游
娱乐,体育
新闻趣事
星座命理
萌宠,投资理财
名人明星,旅游,搞笑幽默,投资理财,教育就业,IT数码,文艺,体育
新闻资讯,美食,旅游,文艺,娱乐
旅游
名人明星,娱乐

游戏动漫,娱乐
志愿者,交朋友,运动,天秤座,活泼开朗,开心,销售,看小说,奋斗,健康
娱乐
美食,搞笑幽默
朱瑜曼,奋斗,文艺,纠结矛盾体,没有伞的孩子,双子女,爱做梦,陈奕迅 Eason
新闻趣事
娱乐
体育
FixedGear
吃货,电影迷,美食,健康,星座命理,娱乐,时尚
搞笑幽默,军事,健康
体育
搞笑幽默,旅游,穿衣美容,视频音乐,IT 数码,体育,美食,娱乐
健康,体育
美食,文艺,旅游,游戏动漫,时尚
美食,文艺,健康
美食
微博奇葩,投资理财
新闻趣事,娱乐,体育
投资理财,旅游,美食
娱乐
旅游,美食,美女,娱乐,文艺
fashion,shopping,梦想
新闻资讯,时尚,教育就业,文艺,娱乐
美食
旅游,美女,时尚,文艺,娱乐
美食
教育就业,汽车,美女,文艺,娱乐
听音乐,90 後,美术,起床困难户,旅游
巨蟹座女生,90 后宅女,旅游摄影,爱吃美食,文艺女青年,爱电影,爱音乐
汽车,时尚
星座命理,文艺
穿衣美容
文艺
陕西生活,健康,娱乐
旅游,旅游爱好者,爱吃,服装,影视,瑜伽,文艺,体育,娱乐
娱乐
美女,旅游,军事,文艺,娱乐
星座命理,娱乐,美食,健康,旅游
潮人,驴友,美食爱好者,耽美,心理,理财,处女座,活泼开朗,漫画,星座命理
穿衣美容,娱乐
微博奇葩,搞笑幽默,健康,游戏动漫,娱乐
美女,旅游,美食,星座命理,穿衣美容
美食,军事,IT 数码

旅游,IT 数码,文艺,教育就业,健康
娱乐
穿衣美容,游戏动漫,时尚,文艺,美食,娱乐
搞笑幽默
旅游,美食,军事,文艺,娱乐
新闻资讯,娱乐,教育就业,健康,美食
新闻资讯,健康,旅游,IT 数码,体育
旅游
美食,文艺
微博奇葩,穿衣美容
游戏动漫,美女,IT 数码,投资理财,娱乐
新闻趣事
旅游
名人明星
穿衣美容,美食
新闻趣事,旅游,视频音乐,投资理财,教育就业,新闻资讯,健康,军事
教育就业,新闻资讯,美食,娱乐,文艺
视频音乐
名人明星
旅游,美食,娱乐
自由职业,电子商务,TI,阿里 express,淘宝,天猫,美丽说,蘑菇街,时尚
美食
娱乐,美食,游戏动漫
游戏动漫,新闻资讯,旅游,投资理财,IT 数码
名人明星,穿衣美容,星座命理,新闻趣事,微博奇葩
健康,游戏动漫,旅游,美食,军事
旅游,美女,时尚,汽车,娱乐
娱乐
汽车,星座命理
旅游,健康,时尚,美食,娱乐
名人明星,娱乐
搞笑幽默
韩剧,活泼开朗,音樂,微博控,love,乐橙
旅游,穿衣美容,视频音乐,名人明星,IT 数码,时尚,美女,娱乐
娱乐
旅游,健康,投资理财,美食,娱乐
游戏动漫
美伦,肯爱一生,煜宠一世,沈氏夫夫,奇葩,肯小兔,沈煜伦,肯尼
萌宠,新闻趣事,旅游,美食,视频音乐
中国风,古典,唐诗宋词,诗词,唯美古诗词,古诗词,静美,阅读,最美中国风,古风诗词
网购,购物狂,美容护肤,品牌,新闻资讯,美食,时尚
时尚,文艺
旅游
美女,文艺,健康,美食,投资理财

旅游,视频音乐,新闻趣事,搞笑幽默,广东生活
其他生活
健康,教育就业,美女,美食,娱乐
穿衣美容,美食,星座命理,视频音乐,新闻趣事
新闻资讯,时尚,军事,美女,体育
娱乐,汽车,时尚,文艺,旅游
星座命理
体育
IVD,转染,体检,FISH,分子诊断,HPV
美图摄影,文艺
娱乐
美食,旅游,健康
视频音乐,新闻趣事,搞笑幽默,文化,射手,娱乐,IT 数码,文艺,新闻资讯,游戏动漫
微博奇葩
汽车,时尚,文艺
名人明星,娱乐
搞笑幽默,视频音乐,投资理财,时尚,文艺,体育,娱乐
旅游
美女,体育
新闻资讯,娱乐,旅游,美食,星座命理
搞笑幽默
健康
视频音乐,浪漫主义诗人,矛盾双子综合体,脑袋大脖子粗,伪球迷
美女,汽车
文艺
军事,教育就业,投资理财,汽车
汽车,时尚,健康,体育,美食
健康,旅游
健康,投资理财,旅游,星座命理,军事
美女
教育就业,投资理财,美食,文艺,健康
星座命理
旅游,IT 数码
健康,美女,汽车,教育就业,体育
汽车
美女,教育就业,娱乐
星座运势,天枰座,双重性格,吃喝玩乐,懒,爱笑,美食
美食,穿衣美容,天平座 A 型女,狗狗,我就是我,旅游美食爱好者,敏感,爱玩,执着
搞笑幽默,娱乐
娱乐,旅游
美食,星座命理,穿衣美容,微博奇葩,新闻趣事,健康,新闻资讯,教育就业,投资理财,娱乐
旅游,娱乐,投资理财,星座命理,新闻资讯,健康
新闻资讯
军事

视频音乐,健康,文艺
星座命理,美女,时尚,健康,文艺,娱乐
喜欢旅游,金牛座,简单,自信,努力,90 後,房地产,热爱生活
双鱼女,吃喝玩乐,电影控,80 後,陈奕迅,时尚,美食,文艺,娱乐
文艺,时尚
唯爱视觉,闺蜜照,儿童照,婚纱照,摄影,时尚
美食,时尚,娱乐
体育
旅游,美食
美食
时尚潮流,好性格,DOTA,单身,正太控,交朋友,心理,幸福,人生,娱乐
娱乐,星座命理,旅游,体育,美食,打篮球,好性格
下得了厨房,喜欢看侦查片,英语学习者,渴望成长,偶尔小可爱,喜欢无拘无束笑,懂那么点坚强,旅游
90,手机,女人,看电影,花痴,臭美,文字控,喜欢音乐,旅游,娱乐
旅游,文艺
艺术,收藏,拍卖
时尚,旅游,美女,美食
汽车
上海生活,教育就业
教育就业,星座命理,新闻资讯
娱乐,教育就业,时尚,旅游,投资理财
搞笑幽默,四川生活,天蝎
美容服饰,吃喝玩乐,时尚,娱乐
美食,搞笑幽默,新闻趣事,美女,视频音乐
名人明星
文艺,旅游,娱乐,美女
萌宠,昆明生活,名人明星,旅游,美食,文艺,时尚,汽车
穿衣美容
腹黑,幽默,原创,IT 互联网,爱幻想,奋斗,绘画,设计师,疯狂,爱生活
教育就业,投资理财,旅游,时尚,健康
美食,娱乐,新闻资讯,文艺,星座命理
美食,游戏动漫,新闻资讯,旅游,时尚
星座命理,健康,文艺,时尚,体育,美食
美食,新闻资讯,汽车,体育,IT 数码
萌宠,星座命理,穿衣美容,美女,完美主义,健康,旅游,时尚,教育就业,投资理财
时尚
文艺,娱乐,健康,美食
山东生活,美女,新闻趣事,搞笑幽默,微博奇葩
旅游
美女,汽车
迷茫是主线,带脑袋是显的高,纠结别人,纠结自己,呆滞,毒舌,吃醋精,傲娇
美食,健康,教育就业,IT 数码,娱乐
文艺

娱乐,汽车,旅游,星座命理,美食
星座命理,文艺,健康,娱乐
美食
房产,美女,汽车,旅游,军事,体育
网站,CRM,网上直报,统计分析
美食,旅游,江苏生活,星座命理,穿衣美容
游戏动漫
SM,美食,旅行
湖北生活
微博奇葩
旅游,健康,时尚,美食,娱乐
旅游,美女,美食,新闻趣事,搞笑幽默
旅游,美食,萌宠
娱乐
美食
星星,宅人加懒人一枚,夜猫子,冷笑话,爱美食,萌宠
名人明星
浙江生活,新闻趣事,视频音乐,旅游,美食
90後,shopping,矛盾体,爱旅行,逛街,减肥,爱玩,DIY,旅游,娱乐
新闻资讯,美女,时尚,旅游,健康,投资理财
旅游
北京生活,文艺
新闻资讯
八卦杂谈,娱乐
穿衣美容
内涵,搞笑,段子
职场招聘,天津生活,新闻趣事,文艺,教育就业,健康,新闻资讯,投资理财
排球,艾尔撒独存,摩羯座
微博奇葩,视频音乐,旅游,穿衣美容,星座命理
萌宠,视频音乐,星座命理,游戏动漫
游戏动漫,教育就业,娱乐
名人明星,游戏动漫,娱乐
穿衣美容,时尚
平面设计师,热爱旅游和美食,吃貨,A型血水瓶座,90后,教育就业,健康,旅游,美食,娱乐
体育
新闻趣事,广东生活
文艺,游戏动漫
娱乐
搞笑幽默,旅游,美食,甘肃生活,新闻趣事
体育,新闻资讯,健康,美食,汽车
美女,新闻资讯,旅游,体育,娱乐
上海生活
旅游,星座命理,文艺,娱乐
文艺

视频音乐
新闻趣事,美女
穿衣美容,宅,听歌,90 后
名人明星,微博奇葩,旅游,美食,星座命理
旅游,健康,新闻资讯,军事
武汉生活,情感生活,视频音乐,健康
萌宠
星座命理,娱乐,文艺
美食,旅游,星座命理,游戏动漫,娱乐
旅游
淘宝,听音乐,购物,美食,新闻趣事,搞笑幽默,美女,穿衣美容
穿衣美容,美食,旅游
美女,美食,娱乐,体育,文艺
外貌协会,自恋,钓鱼,喜欢旅游,电视剧,减肥,自由,上网,睡觉睡到自然醒,旅游
视频音乐,广东生活,新闻趣事,搞笑幽默
文艺
星座命理,视频音乐,旅游,微博奇葩,辽宁生活
浙江生活,新闻趣事,美食
新闻趣事,名人明星,文艺,新闻资讯,教育就业,军事,娱乐
教育就业,新闻资讯,文艺,美食,健康
微博奇葩,旅游,美食
旅游
星座命理
视频音乐
投资理财,健康,旅游
搞笑幽默,平常心,水瓶座,生活
旅游,教育就业,投资理财
读书分享,美食,美图摄影,视频音乐,健康,旅游,时尚,娱乐
澳大利亚生活
重庆生活
体育,娱乐,美女
健康
娱乐
视频音乐
汽车,投资理财
教育,美食
名人明星
穿衣美容
创意,动画,电影迷
甜蜜转身,甜品加盟,满记甜品,知念甜品,老港记甜品,酒窝甜品,茗记甜品,杏记甜品,香港盈福记甜品,盈记甜品
新闻资讯
时尚,IT 数码
搞笑幽默,美食,时尚,娱乐

新闻资讯,军事,娱乐
穿衣美容
创意,微笑,设计,读书
文字控,看小说,听音乐,爱自由,胡思乱想,懒人,旅游,时尚
微笑,天蝎女,美食
美图摄影,美女,名人明星
山西生活,艺术,娱乐
美食,娱乐
做一位好妈妈,好妻子,穿衣美容
吃货一枚,蔡依林,英语,方大同,90 后但我不脑残,睡懒觉,任性,郭敬明,美女,穿衣美容
微博奇葩,旅游
教育就业
汽车,IT 数码
微博奇葩,名人明星,视频音乐,时尚,游戏动漫,星座命理,IT 数码,娱乐
心理,时尚,旅游,星座命理,上海生活
旅游
搞笑幽默,美食,广州生活,旅游,广东生活
搞笑幽默,上海生活,旅游,美食,娱乐
投资理财,教育就业,时尚,健康,娱乐
新闻资讯,教育就业
美食
情感生活,名人明星,视频音乐,梦想,巧克力,文艺,健康,娱乐
时尚
旅游,美食,新闻趣事,搞笑幽默,石家庄生活
美女,健康,时尚
旅游,美食,时尚
射手女,爱旅游,听音乐,淘宝,拍照,热情,外貌协会,爱唱歌,狗狗,美食
江苏生活
旅游,美食,IT 数码,文艺,娱乐
无厘头,90 后非脑残,爱生活,爱睡觉,萌宠,美食,旅游,名人明星,加拿大生活
文艺,娱乐,旅游,健康,时尚
美食,星座命理,微博奇葩,搞笑幽默,新闻趣事
星座命理,微博奇葩,穿衣美容
美食,文艺,娱乐,健康
旅游,美食,美女
星座命理,河北生活,美食,名人明星,穿衣美容
新闻趣事
学生一族,感性,旅游,文艺,娱乐
微博奇葩
搞笑幽默,视频音乐,新闻趣事,穿衣美容,旅游
文艺
美女
新闻趣事
汽车,投资理财

美食
新闻资讯, 时尚, 投资理财, 美食, 娱乐
健康
新闻趣事
体育
旅游
星座命理, 美食
萌宠, 星座命理, 视频音乐, 新闻趣事, 搞笑幽默
旅游, 英国生活
广西生活
书法, 友情, 蛋白质, 格斗
攘夷志士
搞笑幽默, 美食, 穿衣美容, 星座命理, 四川生活
北京生活, 视频音乐
新闻趣事, 文艺
星座命理, 旅游, 名人明星, 穿衣美容, 视频音乐
新闻趣事, 旅游, 搞笑幽默, 广东生活, 美食
萌宠, 新闻趣事, 搞笑幽默, 旅游, 健康, 美食, 娱乐
萌宠
萌宠
自拍, 正太控, 名人明星, 美女
体育资讯, 美女, 校园生活, 视频音乐, 搞笑幽默
穿衣美容, 搞笑幽默, 教育就业, 星座命理, 文艺
健康
新闻趣事, 新闻资讯
搞笑幽默
游戏动漫, 星座命理, 文艺
游泳, 发呆, 医生, 乐观, 吃货, 天蝎座, 好性格, 快乐大本营, 美食, 文艺
美食
旅游
娱乐, 星座命理, 新闻资讯, 健康, 汽车
游戏动漫
视频音乐, 名人明星, 微博奇葩
体育
旅游, 星座命理, 视频音乐, 穿衣美容, 搞笑幽默
娱乐, 健康, 旅游, 美食, 体育
新闻资讯
微博奇葩, 娱乐
搞笑幽默
健康, 投资理财, 旅游, 星座命理, 美食
美食
好书, 校园, 视频音乐
广东生活, 时尚
健康, 时尚, 娱乐

汽车,旅游,体育
美食,美女,投资理财,娱乐
体育
美女,云南生活,旅游
旅游
视频音乐,梅州生活,微博奇葩,搞笑幽默,时尚,美食,娱乐,文艺
视频音乐
美女
美女,微博奇葩,搞笑幽默,广东生活
游戏动漫
时尚,旅游,星座命理,美食,娱乐
穿衣美容,时尚
搞笑幽默,新闻趣事,福建生活,视频音乐,旅游
健康,时尚,星座命理
搞笑幽默,微博奇葩
汽车,星座命理,健康,娱乐
名人明星
南通生活,萌宠
美食,星座命理,体育,健康,旅游
搞笑幽默
VIP
视频音乐
新闻趣事
旅游,娱乐,IT 数码,时尚,教育就业
美食,名人明星,旅游,新闻趣事,美女
微博奇葩
美食
文艺
搞笑幽默,新闻趣事,微博奇葩,视频音乐,时尚,教育就业,体育,游戏动漫,娱乐
娱乐
旅游,体育,文艺,教育就业,健康
读书分享,重口味,动漫,美图摄影,搞笑幽默,微博奇葩
微博奇葩
健康,旅游,新闻资讯,美女,美食
视频音乐,教育就业
美食,美女
汽车,健康,旅游,星座命理,美食
看小说,看电影,吃喝玩乐
星座命理
时尚,旅游,游戏动漫
IT 数码
营销,订购,生产,鼠标垫,吧台垫,游戏垫,地垫,卓垫,广告门垫,瑜珈垫等
北京生活
微博奇葩

陈绮贞,移民留学,投资移民,Eason,出国移民,感性,足球迷,陳綺貞,市场营销策划,体育
文艺,军事,新闻资讯,教育就业,投资理财
文艺
旅游
新闻趣事,搞笑幽默,辽宁生活,名人明星
美女,微博奇葩,名人明星,穿衣美容
时尚,旅游,星座命理,美食,娱乐
旅游
IT 数码
名人明星,旅游,美食,星座命理,北京生活
微博奇葩,IT 数码
投资理财,健康,旅游,美食,星座命理
交朋友,金牛座,购物,淘宝,广东生活,旅游,美食,穿衣美容
教育就业,旅游,健康
辽宁生活,旅游,美食,穿衣美容,搞笑幽默
美食
旅游,新闻趣事,体育,娱乐,文艺,IT 数码
旅游,陕西生活
美食,时尚,汽车,娱乐
微博奇葩,穿衣美容,新闻趣事,搞笑幽默,名人明星
GossipGirl,穷游,美食
旅游,军事
新闻趣事,搞笑幽默,情感生活,视频音乐,娱乐,健康,星座命理,美食
美容服饰,保养,淘宝,网购,护肤,化妆品,时尚,美女,穿衣美容
美食,旅游,穿衣美容,上海生活
旅游,微博奇葩,新闻趣事,搞笑幽默,广东生活,体育,汽车
新闻趣事
搞笑幽默
帅哥,喜欢听歌,B 型血,市场营销,房地产,健康
萌宠
萌宠,文艺
星座命理,旅游,穿衣美容
旅游,健康,美食
美食,旅游,娱乐
微博奇葩,搞笑幽默,萌宠,美女
奶粉代购,澳洲代购
黑龙江生活
旅行,唱 k,看小说,平常心,白羊座
旅游爱好者,文学艺术
广东生活,I,love,basketball
视频音乐,旅游,萌宠,微博奇葩,搞笑幽默,聊天,K 歌啊～,男人,乐天派
美女,搞笑幽默,微博奇葩,重庆生活
旅游,萌宠
穿衣美容

廊坊生活
成都生活
美食,杨幂,真实力,白羊
湖北生活,视频音乐,穿衣美容,新闻趣事
美食
读书分享,搞笑幽默,校园生活,美食,萌宠,微博奇葩
校园生活,新闻趣事,读书分享,搞笑幽默,昆明生活
读书分享,动漫,搞笑幽默,新闻趣事,视频音乐,星座命理
微博奇葩
美女,小说,互粉,动漫,打篮球,矛盾,搞笑幽默
美女,名人明星,视频音乐,星座命理,搞笑幽默
名人明星
新闻趣事,视频音乐,名人明星,旅游,美食
美食,穿衣美容,星座命理,茂名生活,爱睡觉,爱粤语歌,爱电影
字体美化,张小杰欧耶,星星
穿衣美容
搞笑幽默
搞笑幽默
视频音乐
视频音乐,搞笑幽默
浙江生活,旅游
名人明星
星座命理,微博奇葩,视频音乐,名人明星
微博奇葩,美女,萌宠,河北生活,搞笑幽默
视频音乐
萌宠,穿衣美容,视频音乐,搞笑幽默
星座命理,萌宠,美食,穿衣美容,视频音乐,微博奇葩
美食
穿衣美容
微博奇葩,美食,名人明星,美女,搞笑幽默
名人明星
视频音乐,搞笑幽默
搞笑幽默
萌宠
广西生活
名人明星,搞笑幽默,旅行,视频音乐,创意,潮流,创业,读书,设计,大学生
广东生活
名人明星,穿衣美容,视频音乐
微博奇葩,白茶,正山小种,金骏眉,红茶,茶,茶叶销售,茶叶批发,福建生活
看电影,音樂,影视,宅女一枚,90,懒人,爱唱歌,追星,爱睡觉,写字
美食,穿衣美容,视频音乐,旅游,搞笑幽默
美女
微博奇葩,视频音乐,星座命理,名人明星
视频音乐,穿衣美容,旅游,微博奇葩

搞笑幽默,微博奇葩
其他生活
販賣,日韓代購款,服装,fashion,日韩,原宿,美图摄影,美容服饰
美食
新闻趣事
名人明星,穿衣美容,美食,旅游
搞笑幽默,视频音乐,穿衣美容,旅游,名人明星
微博奇葩,搞笑幽默,视频音乐
旅游,美食,微博奇葩,星座命理
互粉,90,偏执狂,看书,名人明星,新闻趣事,美食,视频音乐
狮子女
微博奇葩
美女
星座命理
江苏生活
美女,星座命理,影视,90 后但我不脑残,校园,睡觉睡到自然醒,天秤,郭敬明,五月天,电影爱好者
聊天,美食,旅游,视频音乐,搞笑幽默,香港生活
美图摄影
情感生活
美食
美食
名人明星
美食
旅游,微博奇葩,新闻趣事,搞笑幽默
旅游,名人明星,美食,星座命理,穿衣美容
美女
旅游,美食,星座命理,穿衣美容
美食
美食
美食
美食
美食
新闻趣事,四川生活,微博奇葩
旅游
新闻趣事,微博奇葩,搞笑幽默
福州生活,名人明星,搞笑幽默
新闻趣事,名人明星,穿衣美容,旅游,美食
视频音乐,美食,河南生活,微博奇葩,穿衣美容
美食
美食,旅游,视频音乐,其他生活
美女
美术绘画,视频音乐,穿衣美容,萌宠,广东生活
宠物图集

广东生活
星座命理,穿衣美容
音乐迷
河南生活
英国,简单,爱旅行
情感生活,宠物图集,名人明星,新闻趣事
新闻趣事,八卦杂谈
视频音乐
云南生活,搞笑幽默,新闻趣事,视频音乐,旅游
新闻趣事,萌宠
美食
新闻趣事,八卦杂谈
名人明星
名人明星
视频音乐
穿衣美容
美女
美食
视频音乐,韩国生活,名人明星
萌宠
追求时尚,电影，逛街,双子座,阳光开朗、,90 后,看小说、睡觉、,听歌、
搞笑幽默
名人明星
旅游,美食
搞笑幽默,旅游,名人明星
星座命理,美食,萌宠,微博奇葩,上海生活
萌宠,西安生活,微博奇葩,搞笑幽默
旅游
新闻趣事,美食,重庆生活,旅游
微博奇葩
穿衣美容
上海生活
搞笑幽默
视频音乐
视频音乐
淄博生活
北京生活
微博奇葩,视频音乐
美食,旅游,微博奇葩,搞笑幽默
东方神起,沈昌珉
IT 数码,美图摄影
搞笑幽默
美食
新闻趣事

旅游
视频音乐,搞笑幽默
旅游,穿衣美容
视频音乐,搞笑幽默,美女
八卦杂谈
旅游
美图摄影
视频音乐
微博奇葩
时尚
美食
娱乐
美女,IT 数码
美女
搞笑幽默,青海生活
美女
游戏动漫
旅游
美食,IT 数码,文艺
新闻趣事
美女,穿衣美容,北京生活,旅游
旅游
娱乐
美女,名人明星
旅游
重庆生活
湖南生活
射手座 O 型血,射手女,淘宝店主,文艺,星座命理,健康,美女,时尚
湖北生活
美食
美女
美食,旅游,星座命理
新加坡生活,新加坡留学,工业设计,好莱坞,学校,游戏开发,电影,概念设计
汽车,美女,旅游,健康
新闻趣事,桌游,散步
美食
军事
IT 数码,时尚,新闻资讯,星座命理,美食
IT 数码
互粉,电影迷,90 后但我不脑残,校园生活,搞笑幽默,穿衣美容,美女,视频音乐,新闻趣事,湖北生活
搞笑幽默
美食,文艺,健康,美女,时尚,旅游
文艺,星座命理,健康,娱乐

搞笑幽默,旅游,穿衣美容,美食,美女,时尚,文艺,娱乐
新闻趣事,搞笑幽默
新闻资讯,文艺,美食,教育就业,娱乐
萌宠,陕西生活,视频音乐
时尚,健康,星座命理,娱乐,IT 数码
娱乐
搞笑幽默,名人明星
微博奇葩,美女,新闻趣事,搞笑幽默,广东生活
健康
新闻趣事,搞笑幽默,湖南生活,视频音乐,旅游
视频音乐
投资理财,健康,旅游,美食,体育
时尚
时尚,汽车
新闻资讯,教育就业,投资理财,IT 数码
美女,旅游
旅游
IT 数码,星座命理
旅游,星座命理,IT 数码
微博奇葩,穿衣美容
旅游,陈奕迅,三国杀,音乐爱好者,游戏动漫
穿衣美容,新闻趣事,搞笑幽默,美女,名人明星
美女,健康,旅游,娱乐
游戏动漫,美食
搞笑幽默
旅游
娱乐
穿衣美容,名人明星,萌宠,美食
美食
文艺,娱乐,美女,时尚
情感生活,白羊女,音樂,旅游
海贼王,JYJ,東方神起
IT 数码,情感生活,新闻趣事,美食,名人明星
期货投资,外盘期货投资,外汇投资,投资理财,新闻资讯
星座命理,娱乐
福建生活,美女,穿衣美容,健康
星座命理
汽车,文艺,旅游
k 歌,美食,旅游,汽车,文艺,娱乐
穿衣美容
名人明星
美女,内向,跑步
游戏动漫
搞笑幽默,穿衣美容,旅游,美食,广东生活

美食
动漫,星座命理
娱乐
穿衣美容
娱乐
旅游,旅行,美图,摄影,摄影爱好者,文艺
皇马,幽默,搞笑,军事,体育
深圳奇迹,深圳实习,深圳求职,深圳,深圳全职,深圳人,深圳招聘,深圳兼职,深圳广告
搞笑幽默,微博奇葩
名人明星,微博奇葩
萌宠,游戏动漫
旅游,微博奇葩,搞笑幽默,广东生活,新闻趣事
文艺,新闻资讯,旅游,娱乐,IT 数码
健康
体育,汽车,新闻资讯,娱乐,美女
文艺,IT 数码,游戏动漫,旅游,娱乐
广东生活,星座命理
旅游
内向
世博志愿者,爱生活,吉他,巨蟹座,旅游爱好者,打篮球,幸福,视频音乐,旅游,山西生活
宅女加腐女,流浪,登山,志愿者,神经大条,星座命理,旅游,美食,文艺,娱乐
萌宠
美食,新闻趣事,搞笑幽默,视频音乐,旅游
穿衣美容,时尚
教育就业,体育,娱乐,新闻资讯,旅游
旅游,美食,北京生活,穿衣美容,新闻趣事
其实我很好,编辑
美食,湖北生活
穿衣美容,新闻资讯
名人明星,体育,娱乐
美食,萌宠,微博奇葩,穿衣美容,新闻趣事
名人明星,星座命理,旅游
美食,娱乐
江苏生活,摄影控,狮子女,淘宝控,周杰伦,开朗,美术,军事
穿衣美容
旅游
旅游,搞笑幽默,深圳生活,美容服饰,育儿百科
摄影,自由,视频音乐
吃货一枚,游戏动漫,美食爱好者,旅游
旅游,游戏动漫
教育就业,汽车
喜欢一个人,时尚,星座命理,军事,文艺,新闻资讯
教育就业
湖北生活,IT 数码

美食
娱乐
美食
汽车,IT 数码,美女,新闻资讯,军事
微博奇葩,时尚
旅游
体育
星座命理,美食,IT 数码,健康,游戏动漫,时尚,娱乐
萌宠,娱乐
星座命理
美食
旅游,美食,IT 数码,文艺,娱乐
美食
旅游
网球爱好者,各种宅,90 后非脑残,旅游,美食
美女,游戏动漫,娱乐
旅游
新闻趣事,珠海实习,珠海广告,珠海招聘,珠海全职,珠海兼职,珠海人,珠海,广东生活
上海生活,投资理财
江苏生活
美女,文艺
美食,北京生活,微博奇葩,新闻趣事,搞笑幽默
娱乐,文艺,IT 数码,星座命理,新闻资讯
视频音乐
电脑,篮球爱好者,搞笑幽默
旅游
湖北生活
旅游,文艺,游戏动漫,军事,娱乐
美女,搞笑幽默,重庆生活
萌宠,微博奇葩
IT 数码,汽车,投资理财
美女
美食,时尚,健康
安静,B 型血,渴望自由,摩羯座,旅游
美女
新闻趣事,微博奇葩,旅游,美食,湖北生活
教育就业
时尚
名人明星,穿衣美容,文艺
旅游,好书,热爱生活,自信
时尚,旅游,星座命理,文艺,娱乐
北京生活
美女
文艺

零食,中秋节,月饼,营口生活,食品,休闲,面包,蛋糕,美食
广东生活
娱乐
新闻资讯,健康,美食,娱乐
山东生活,汽车,健康,新闻资讯,投资理财
深圳生活,广东生活
爱猫,迈克尔杰克逊,旅游,美食,新闻资讯
星座命理,搞笑幽默,美食,名人明星
穿衣美容
美食,旅游,萌宠,美女,新闻趣事
视频音乐
时尚,汽车,教育就业,投资理财,军事
汽车,娱乐,体育
文艺,娱乐
法国生活
分格体之四,介离,无晓,方生,七杀
健康,旅游,文艺,美食
美女,视频音乐
娱乐
星座命理,健康,美食,时尚,娱乐
美食,时尚,投资理财,娱乐,IT 数码
汽车,IT 数码,教育就业,投资理财,体育
游戏动漫,星座命理,文艺
游戏动漫
美食,健康
美食
侈饰品代购,侈奢品,YSL,DIOR,HERMES,GUCCI,LV,CELINE,Chanel,爱马仕
汽车,星座命理,旅游
旅游,名人明星
唱歌,爱美食,爱笑,感性,简单,健康,时尚,美食,体育,娱乐
星座命理,美食,视频音乐
健康,新闻资讯,星座命理,文艺,娱乐
吉他爱好者,视频音乐
微博奇葩,星座命理
体育
教育就业,新闻资讯
名人明星,八卦杂谈,美食,娱乐
旅游,健康,教育就业,体育
汽车,旅游,娱乐
名人明星
游戏动漫
名人明星
美食
美女,微博奇葩

听音乐,搞笑幽默
新闻资讯,文艺,美食,娱乐
文艺,汽车,星座命理,游戏动漫
游戏动漫
星座命理,新闻趣事,美女,搞笑幽默
美女,汽车,时尚,游戏动漫,娱乐
娱乐,旅游,星座命理
娱乐
旅游
娱乐
视频音乐
汽车,游戏动漫,文艺,体育,娱乐
军事
萌宠,时尚
新闻资讯
美食
美食,宠物图集,教育就业,新闻资讯,健康,旅游
视频音乐,娱乐
搞笑幽默,管理,公益,哲学,猫
旅游
文艺,旅游,美食,军事,美女
视频音乐,健康,美食,星座命理,游戏动漫
美容服饰
视频音乐,名人明星
美女,旅游,名人明星
美食,搞笑幽默,旅游,游戏动漫,星座命理,文艺,娱乐
萌宠
新闻资讯,美女,美食,时尚,IT 数码
游戏动漫
搞笑幽默,名人明星,游戏动漫
汽车
娱乐,文艺,游戏动漫
济南生活,星座命理,穿衣美容,旅游
娱乐,旅游,时尚,美食
萌宠
时尚
江苏生活
淘宝控,服装,可爱,舞蹈,创意,画画,美国,感性,DIY,搞笑幽默
微博奇葩
穿衣美容,时尚,文艺,旅游,健康,美食
游戏动漫
名人明星,美食,旅游,微博奇葩,搞笑幽默
美食,微博奇葩,旅游,新闻趣事,搞笑幽默,新闻资讯,军事
名人明星,视频音乐,穿衣美容,美食,娱乐,IT 数码,文艺,时尚,旅游

美食,娱乐
动漫控,绘画,文字,星座命理
情感生活,英语,文艺,画画
90 後,白羊,古风,学生一枚,王爷党,动漫,COSPLAY,萌宠
旅游
长春生活,旅游
教育就业,IT 数码,时尚,投资理财,汽车
娱乐,体育
微博奇葩
音乐,娱乐
美图摄影,穿衣美容
娱乐,健康,旅游,美食
新闻趣事,校园生活,名人明星,旅游,搞笑幽默
文艺,汽车
时尚,文艺,娱乐
教育,武汉生活,新闻资讯,教育就业,husters,huster,华中大学子网,华中大,华中科技大学,HUST
视频音乐
穿衣美容,美女,名人明星,微博奇葩,湖北生活
旅游
体育
旅游,名人明星,穿衣美容,新闻趣事
体育
小文艺,电影,90 后,青春,生活,大学生,美食
视频音乐,游戏动漫
视频音乐,时尚,文艺,娱乐
星座命理
美食,旅游,搞笑幽默
健康
旅游,新闻资讯,星座命理
星座命理
穿衣美容
名人明星,旅游,萌宠,美食,视频音乐
文艺,美食,健康,美女,时尚
浙江生活,体育
搞笑幽默
广东生活
搞笑幽默,视频音乐,萌宠,微博奇葩
时尚
名人明星
90 后非脑残,矛盾,微笑,自由,校园,学习,学生
喜欢喜欢我的人,强烈精神洁癖,强烈占有欲
赚钱,穿衣美容,美食
微博奇葩,搞笑幽默

旅游,美食,湖北生活,穿衣美容,视频音乐
书法,名人明星
小说,唱歌,看书,动漫,文艺
旅游
星座命理,穿衣美容,小资,哈哈,笑,自然,坚强,文艺,娱乐
军事
云南生活,旅游,美女,视频音乐,搞笑幽默
名人明星,新闻趣事,搞笑幽默,辽宁生活
新闻趣事
微博奇葩,搞笑幽默,美食
江苏生活
美女
新疆生活
名人明星
视频音乐,搞笑幽默
旅游
八卦杂谈
微博奇葩
视频音乐
动画,爱
湖南生活
搞笑幽默,上网,音乐,改变 ing,CrossFire,LOL,Leo
多伦多,美衣美鞋,韩国代购
搞笑幽默,新闻趣事,旅游,湖北生活
穿衣美容,名人明星
旅游,名人明星
穿衣美容,视频音乐,萌宠,美食,旅游
Khuntorian,Am,I,Forever,Khuntoria
美食,穿衣美容,新闻趣事,浙江生活
旅游,穿衣美容,星座命理,微博奇葩,美女
微博奇葩,美食,新闻趣事
星座命理,穿衣美容,新闻趣事
旅游
星座命理,四川生活
河南生活,旅游,微博奇葩,视频音乐,搞笑幽默
其他生活,搞笑幽默,穿衣美容,美食
美容服饰,穿衣美容
旅游
福建生活
新闻趣事
新闻趣事
美图摄影,星座运势,新闻趣事,名人明星,视频音乐
美女,新闻趣事,搞笑幽默,马鞍山生活,微博奇葩
名人明星,穿衣美容,新闻趣事,视频音乐,搞笑幽默

穿衣美容
旅游
美容护肤,狮子女,时尚达人,淘宝控,潮流,淘宝,彩妆,shopping,宅女一枚,美容服饰
萌宠
穿衣美容,新闻趣事,搞笑幽默,微博奇葩,美食
名人明星,视频音乐
星座运势,情感生活,校园生活,名人明星,穿衣美容,新闻趣事,微博奇葩,广东生活
美食
娱乐
搞笑幽默,穿衣美容,旅游,美食,江苏生活
旅游
体育资讯,福建生活,新闻趣事,视频音乐,旅游
投资,潮流,养生,旅游,美食,娱乐
星座命理
时尚,旅游,体育,娱乐,健康
搞笑幽默,名人明星,星座命理,微博奇葩,视频音乐,时尚,娱乐
娱乐,旅游,美食
搞笑幽默,健康
文艺
玉树生活,旅游,新闻资讯
强迫症,体育
星座命理,文艺,娱乐
美女,新闻趣事,湖南生活
时尚,美食
穿衣美容,美食,星座命理,健康,旅游,文艺
新闻趣事,穿衣美容,上海生活
搞笑幽默
名人明星,美女,微博奇葩,新闻趣事,山东生活
文艺
星座命理
名人明星,穿衣美容,微博奇葩,林宥嘉,康熙来了,开朗,娱乐,文艺
美食
美女,汽车,娱乐,军事,体育
文艺,娱乐,旅游,美食,健康
美图摄影
旅游,星座命理
萌宠,旅游,美食,江苏生活,穿衣美容
旅游
广东生活
新闻趣事,美女,旅游,美食,重庆生活
热爱生活,重庆生活,家居,K 歌,90,购物狂,独立,浪漫,微博,爱情
山东生活,旅游,新闻趣事
投资理财,旅游,美食,文艺,娱乐
活泼,梦想,看小说,名人明星,新闻资讯,教育就业,文艺,娱乐

视频音乐
视频音乐
重庆生活
美食,旅游,吉林生活,穿衣美容
纠结,篮球迷,高中生,宅男,Bieber 迷,感性
新闻趣事
搞笑幽默
搞笑幽默
旅游
美食,旅游
萌宠,美食,旅游,视频音乐,新闻趣事
搞笑幽默
孩子气,旅游,穿衣美容,搞笑幽默,视频音乐,名人明星
团购,新闻趣事,美食,旅游,星座命理,浙江生活
游戏,微博奇葩,搞笑幽默
萌宠,名人明星,视频音乐
微博奇葩
炫舞,柠檬,煜伦党
微博奇葩,新闻趣事,星座命理,搞笑幽默
名人明星,旅游,穿衣美容,新闻趣事,视频音乐
萌宠,旅游,微博奇葩,美食,穿衣美容
美女
美食,搞笑幽默,旅游
微博奇葩
美食,旅游,美女,搞笑幽默,山西生活
重口味,动漫,欧美,美剧控,微博奇葩,名人明星,视频音乐,搞笑幽默
视频音乐
搞笑幽默,睡眠不足星人,wp8
搞笑幽默
拍照,90 後,微博,爱笑,漫画
穿衣美容
旅游
ACT
视频音乐,微博奇葩,新闻趣事,搞笑幽默,江苏生活
微博奇葩,搞笑幽默,旅游
旅游,自由,音乐控,书籍,爱旅游
微博奇葩,搞笑幽默,新闻趣事,愤青
旅游,视频音乐,星座命理
微博奇葩
视频音乐,重庆生活
搞笑幽默,微博奇葩,旅游
柯南,新闻趣事,视频音乐,美食,广东生活
青春,偶稀饭睡觉觉,旅游,搞笑幽默,新闻趣事,星座命理,穿衣美容
美女

个性,美食,旅游
星座命理,美食,旅游,河北生活,搞笑幽默
新闻趣事,美女,视频音乐,搞笑幽默
旅游,新闻趣事
视频音乐,搞笑幽默
旅游,浙江生活,搞笑幽默,视频音乐,新闻趣事
旅游
旅游,星座命理,视频音乐
搞笑幽默,上海生活,名人明星,新闻趣事
新闻趣事
微博奇葩
居来提,JURAI,BBOY,2013 快乐男声
新闻趣事
星座命理,名人明星
大学,校园,医学,新闻趣事,四川生活
旅游,广东生活,名人明星
湖人,音楽,笑
星座命理
美女
美食,福建生活
旅游
萌宠
新闻趣事
广东生活
青春,K 歌,原创,乐队,喜欢音乐
美食,星座命理,旅游,穿衣美容
旅游,山东生活,穿衣美容
玩,生活,新闻趣事
饶永不瘠,平永不乱
新闻趣事
新闻趣事,旅游,福建生活,搞笑幽默,名人明星
济南生活,新闻趣事,搞笑幽默,穿衣美容,星座命理,微博奇葩,山东生活,旅游
新闻趣事,微博奇葩,萌宠,美食,旅游
美女
美女
视频音乐,旅游,美食,萌宠,穿衣美容
星座命理,旅游,美食,视频音乐,穿衣美容
微博奇葩,新闻趣事,穿衣美容,美食,星座命理
名人明星,星座命理,微博奇葩,视频音乐,穿衣美容
搞笑幽默
美食
北京生活,穿衣美容
微博奇葩,美女,新闻趣事,福建生活,搞笑幽默
名人明星

搞笑幽默
名人明星,旅游,美食,星座命理,山东生活
武汉生活,湖北生活
星座命理,旅游,美食,穿衣美容,天津生活
美女,星座命理,新闻趣事,广东生活,视频音乐
微博奇葩
旅游
名人明星,旅游,美食
旅游,视频音乐,新闻趣事,微博奇葩,搞笑幽默
美食
重庆生活,搞笑幽默,视频音乐,旅游,穿衣美容
福建生活
微博奇葩,新闻趣事
搞笑幽默,名人明星,美食,福建生活,新闻趣事
穿衣美容
视频音乐,名人明星,美食,旅游,穿衣美容

易勾搭易推倒ㅅ,边伯贤生生推,all 白,性感蠢萌金钟仁,软糯可口吴白久,开白开白,勋白最

有爱,牛白萌死人,WeAreOne,软糯可口吴世勋
视频音乐,穿衣美容,搞笑幽默
校园生活,美女
名人明星,旅游,美食,微博奇葩
搞笑幽默,微博奇葩,新闻趣事,美女
美食
美食,星座命理,穿衣美容
新闻趣事,天津生活,微博奇葩,星座命理,搞笑幽默
视频音乐,美食,旅游,穿衣美容
名人明星
八卦杂谈,微博奇葩
美食,旅游,重庆生活
萌宠,穿衣美容,旅游
娱乐,幽默,看书,摄影,唱歌,生活大爆炸,睡觉睡到自然醒,江苏生活,旅游,美食
说走就走的旅行,星座命理,美女
微博奇葩,美食,旅游,视频音乐,新闻趣事
名人明星
美食,美女,视频音乐
搞笑幽默,微博奇葩,美食
旅游,美食,北京生活
名人明星,穿衣美容,星座命理,微博奇葩,搞笑幽默
时尚潮流,淘宝,互粉,摄影爱好者,白羊座
同人游戏,AVG,GAL,真恋,原创游戏,二次元,ACG
微博奇葩,新闻趣事,四川生活,穿衣美容
微博奇葩,星座命理,名人明星,视频音乐,美食
校园生活,四川生活

穿衣美容,名人明星
微博奇葩
搞笑幽默,重庆生活
玩,美食控,穿衣美容,星座命理
穿衣美容
北京生活
湖北生活
搞笑幽默,新闻趣事,旅游,美食
美食,星座命理,名人明星,穿衣美容,美女,江苏生活
美食
名人明星,安徽生活
微博奇葩,萌宠
贵州生活
名人明星
美女,星座命理,微博奇葩,新闻趣事,搞笑幽默
爱音乐,五月天,篮球,科比,麦迪,语录,美食
美女
穿衣美容,美食,旅游
名人明星,美食,搞笑幽默,旅游
旅游,搞笑幽默,穿衣美容,微博奇葩
宅女一枚,旅游美食,疯狂,人生,古典音乐,吃货一枚,学生一枚,旅游,萌宠,名人明星
读书分享,八卦杂谈,情感生活,新闻趣事,萌宠,美食,星座命理
搞笑幽默,新闻趣事
星座命理,美食,旅游,穿衣美容,名人明星
政治,游戏,电影,动漫,音乐,搞笑幽默
搞笑幽默,新闻趣事,视频音乐,美食,旅游
爱旅游,留学,dota,星座控,教育培训,音乐,电影,小说迷,美剧迷,名人明星
注册电气工程师,高富帅
穿衣美容,双子女,90后非脑残,音乐迷
穿衣美容
美女
搞笑幽默
美食,旅游
星座命理,美食,穿衣美容,视频音乐
美食
睡觉睡到自然醒,胡思乱想,旅游,微博奇葩,视频音乐,新闻趣事,名人明星
自由飞翔,不用理我,饭爱豆不混饭圈,人间不信
上海生活,名人明星
微博奇葩
广东生活
萌宠
视频音乐
萌宠
名人明星

旅游,美食,穿衣美容
吃货一枚,DOTA,NBA,睡觉睡到自然醒,90 后但我不脑残,交朋友,淘宝控,韩剧,学生一族,小说迷
Cute,美食
穿衣美容
电影,牛奶咖啡组合,视频音乐
新闻趣事,湖南生活
读书分享,新闻趣事,校园生活,视频音乐
美食,浙江生活,旅游,搞笑幽默,微博奇葩
体育资讯,萌宠,微博奇葩,新闻趣事,广东生活,搞笑幽默
旅游,美女
美食,名人明星,穿衣美容,搞笑幽默,新闻趣事
新闻趣事,搞笑幽默,美女,微博奇葩,视频音乐
搞笑幽默,河南生活,美食,美女,名人明星
旅游,视频音乐,美食
美女,新闻趣事,穿衣美容,微博奇葩,搞笑幽默
乐观,吃货,猫控,开心,阳光,00 后,可爱,单纯
旅游,美女
看小说,吸血鬼,明星,时尚,娱乐,时尚潮流,冷笑话,生活,美食,穿衣美容
穿衣美容
重口味,动漫,校园生活,石嘴山生活,新闻趣事,穿衣美容,星座命理,微博奇葩
陈奕迅,爱笑,爱吃,微博奇葩,穿衣美容,旅游,美食,名人明星
星座命理,穿衣美容
美图摄影,美女,旅游
宅,NBA,海贼王,beatbox 初学者,微博奇葩,美食,美女,搞笑幽默
新闻趣事,美食,名人明星,美女,穿衣美容,澳大利亚生活
名人明星
微博奇葩,视频音乐
萌宠
美女
名人明星
搞笑幽默,广东生活,星座命理,穿衣美容,旅游
视频音乐
微博奇葩,新闻趣事,北京生活,萌宠
新闻趣事,搞笑幽默,名人明星,旅游,美女
新闻趣事
搞笑幽默,微博奇葩,视频音乐,旅游,萌宠
穿衣美容
新闻趣事,穿衣美容,旅游,美食,名人明星
搞笑幽默,视频音乐,微博奇葩,名人明星
辽宁生活,新闻趣事,视频音乐,旅游,星座命理
体育赛事,视频音乐,旅游,美食,微博奇葩,湖北生活
美食,名人明星
搞笑幽默

美食,名人明星,美女,旅游,穿衣美容
视频音乐
穿衣美容
美容服饰,新闻趣事,搞笑幽默,甘肃生活,旅游,美食
微博奇葩
视频音乐,搞笑幽默
旅游
搞笑幽默,魔兽世界玩家,bjd,金光布袋戏,霹靂布袋戲
名人明星
搞笑幽默,星座命理,萌宠,穿衣美容
动漫周边,动漫,潮流,搞笑幽默,日本生活
美女
新闻趣事,视频音乐,旅游,美食,山西生活
驴友,音乐控,疯狂,搞笑幽默,旅游
星座命理,美食,名人明星,旅游,穿衣美容
视频音乐,旅游美食爱好者,搞笑幽默
星座命理
名人明星,美食,旅游,星座命理,搞笑幽默
视频音乐,美食,旅游
名人明星,微博奇葩,旅游,美食
萌宠,上海生活,新闻趣事,美女
喜欢李易峰,学习,宅女一枚,神经质,随性
旅游,美女,新闻趣事
旅游
学生一族,摄影师,青春,幻想,电影迷,懒人,淘宝控,旅游,美食,四川生活
微博奇葩,新闻趣事
搞笑幽默,新闻趣事
旅游,河南生活,搞笑幽默,名人明星,新闻趣事
旅游
美食,穿衣美容,搞笑幽默,旅游,江苏生活
视频音乐,星座命理,穿衣美容,旅游,美食
美女
美食,穿衣美容,新闻趣事,搞笑幽默,辽宁生活
旅游
重庆生活,美食,旅游,名人明星
视频音乐,名人明星,微博奇葩
美女
旅游,四川生活,名人明星,新闻趣事
微博奇葩
学生一族,星座命理,视频音乐,名人明星,旅游,美食
穿衣美容,新闻趣事,美食,名人明星,旅游
Nichkhun
微博奇葩,名人明星,新闻趣事,搞笑幽默,星座命理
名人明星

搞笑幽默,微博奇葩,新闻趣事
星座运势,美图摄影,星座命理,名人明星
手链,玉髓,松石,海蓝宝,琥珀,水晶,玛瑙,天然石项链,绕韵天然石,绕韵围巾
微博奇葩
90,宅女
旅游
名人明星
旅游
微博奇葩,旅游,美食,视频音乐
搞笑幽默
手机看小说,腐女,江苏生活,搞笑幽默,萌宠
郑州生活,新闻趣事
名人明星,搞笑幽默,旅游,视频音乐
萌宠
上海生活,爱美食,朋友,旅游
搞笑幽默,美女
看电影,射手座,名人明星
旅游
微博奇葩,电影爱好者,90
视频音乐,名人明星,穿衣美容
NBA,萌宠,视频音乐,搞笑幽默,美食
美食,美女,星座命理,浙江生活
名人明星
B 型血,听音乐,聊天,天秤座,美女
云南生活
美食,恩施美术,恩施街舞
新闻趣事
吃货,天蝎座,宅女,幽默,旅游
视频音乐
星座命理,萌宠,美食,名人明星
名人明星
旅游
穿衣美容
旅游
新闻趣事
旅游
搞笑幽默
新闻趣事
穿衣美容,美食
穿衣美容
美女
美食
微博奇葩
码字,旅游,星座命理,视频音乐,搞笑幽默,美女

微博奇葩
名人明星,搞笑幽默,美女,萌宠,穿衣美容
我爱听歌唱歌,一直孩子,感性的娃,爱哭爱笑,执着所爱,简单快乐,任性随心,文艺女青年,我爱三毛,热爱生活
视频音乐,名人明星,微博奇葩,搞笑幽默,穿衣美容
美食,上海生活
美图摄影
美食
星座命理,旅游,美食,萌宠,微博奇葩
旅游,新闻趣事,视频音乐,名人明星,搞笑幽默
搞笑幽默,橡皮章,BGBL 两头爬,冲神一生推
穿衣美容,名人明星,美食,新闻趣事,旅游
微博奇葩,四川生活,美食,旅游
旅游,视频音乐,新闻趣事,河北生活
旅游
宅女,双子座,听歌
穿衣美容
名人明星
情感生活,穿衣美容
新闻趣事,旅游,美食,穿衣美容
山东生活,旅游,美食,微博奇葩,搞笑幽默
美食
旅游,视频音乐,搞笑幽默,穿衣美容,名人明星
河南生活,新闻趣事,名人明星,穿衣美容,美女
名人明星
萌宠,搞笑幽默
缺爷一生堆,次元级乱入,拖延症晚期,女神经病,大本命权志龙
爱好就是没爱好
星座命理
旅游,美女,新闻趣事,搞笑幽默,福建生活
美女
名人明星,福建生活
情感生活,宠物图集
新闻趣事,美女,微博奇葩,搞笑幽默,广东生活
穿衣美容
校园生活,旅游,体育资讯,名人明星,新闻趣事,美食,星座命理
陕西生活
美女
星座命理,穿衣美容,美女
萌宠
视频音乐
旅游,名人明星
星座命理
美女,微博奇葩,视频音乐,北京生活,新闻趣事

星座命理
视频音乐,旅游,美食
美女
旅游,美食,美女,视频音乐,搞笑幽默
新闻趣事
旅游
视频音乐
星座命理,穿衣美容,美食,萌宠,微博奇葩
搞笑幽默,新闻趣事,名人明星,穿衣美容,美女
名人明星,萌宠
碉堡了,表情帝,漫画,动漫,app,图片,搞笑视频,搞笑幽默,美女
阅读,爱自由,宅男,矛盾体,神经质,O 型血,福建生活
旅游,美食
美食,新闻趣事,视频音乐,旅游,穿衣美容
搞笑幽默
巨蟹,O 型血,杭州,浙江生活,绘画,music,旅游美食,摄影,电影迷
微博奇葩,萌宠,穿衣美容
穿衣美容
旅游
星座命理,湖北生活,微博奇葩,搞笑幽默,视频音乐
胡思乱想,自然,古典音乐,文化,散步,搞笑幽默
美容服饰,美图摄影,搞笑幽默,广东生活,穿衣美容,视频音乐,旅游
穿衣美容,名人明星
旅游,萌宠,微博奇葩,搞笑幽默,河北生活
名人明星,搞笑幽默,新闻趣事,视频音乐,微博奇葩
广西生活
微博奇葩,穿衣美容,旅游,搞笑幽默,江苏生活
视频音乐
旅游,视频音乐,美女,搞笑幽默,美食
暮光之城,校园生活,读书分享,情感生活
视频音乐
搞笑幽默
微博奇葩
穿衣美容,视频音乐,微博奇葩,搞笑幽默,美食
名人明星,美食,浙江生活
搞笑幽默
高中生,思考
陕西生活
旅游
新闻趣事,美女,视频音乐,搞笑幽默,微博奇葩
美女
视频音乐
星座命理
教育,拓展,演讲,跆拳道,拳击,情商,户外

广东生活,名人明星,新闻趣事,微博奇葩,搞笑幽默
旅游
星座命理,搞笑幽默,名人明星,萌宠,微博奇葩
金牛,喜欢听歌,漫画,执着,学生一族,大学,90后但我不脑残
天秤座,聂小凤,sasha,美剧,GL小说,御姐控,百合控,赵雅芝
新闻趣事,搞笑幽默,微博奇葩,名人明星,美女
单身,糗百,运动
旅游,搞笑幽默
湖北生活
新闻趣事,视频音乐,美女,旅游,名人明星
穿衣美容,星座命理,视频音乐,旅游,美食
穿衣美容
名人明星,萌宠,新闻趣事,星座命理,搞笑幽默
视频音乐
梧州生活,微博奇葩,新闻趣事,视频音乐,广西生活,搞笑幽默
红酒,基督徒,旅游美食,旅遊,娯樂,听音乐,阳光,爱笑,美食
旅游,微博奇葩,搞笑幽默,山西生活,新闻趣事
旅游
旅游,名人明星,搞笑幽默,美食,穿衣美容
新闻趣事
搞笑幽默,广州生活,广东生活
穿衣美容,萌宠,美食,旅游
体育篮球,广东生活
红衫绿,低调,视鞋如命,白羊座,dream,season,chukka,flyknit,sneaker,reebok
新闻趣事,旅游,广东生活,穿衣美容,美女
微博奇葩
湖北生活
名人明星,美食,江苏生活,视频音乐,穿衣美容
宅,卖萌,天然呆,宠物,app,漫画,动漫,萌宠,美女
中山生活,萌宠,搞笑幽默,穿衣美容
名人明星
新闻趣事,宠物图集,微博奇葩,萌宠,搞笑幽默
新闻趣事,穿衣美容,视频音乐,名人明星,星座命理
视频音乐,搞笑幽默,名人明星
微博奇葩
萌宠,视频音乐
微博奇葩
爱猫,幽默,画画,动漫控,动漫迷,插画
旅游,美食,汕头生活,广东生活,新闻趣事,视频音乐
名人明星,穿衣美容,旅游,美食,美女
新闻趣事
微博奇葩,萌宠,旅游,北京生活
星座命理
星座命理,搞笑幽默,广东生活,视频音乐,美女

旅游,星座命理,搞笑幽默,内蒙古生活
浪漫,射手,潮人,服饰,家居,喜欢旅游,喜欢听歌,美食爱好者,时尚达人,热爱生活
美女,穿衣美容,星座命理,山西生活,新闻趣事
搞笑幽默,穿衣美容,星座命理
星座命理,骨灰级玩家,写手一枚,音乐,动漫爱好者一只
搞笑幽默,美女
美食,萌宠
旅游,微博奇葩,搞笑幽默,名人明星,美食
河北生活,搞笑幽默,微博奇葩,新闻趣事
健康,旅游,美食,北京生活
搞笑幽默
旅游,美食,新闻趣事,穿衣美容,搞笑幽默
搞笑幽默
搞笑幽默,新闻趣事,河南生活
萌宠,美食,微博奇葩,新闻趣事,搞笑幽默
四川生活
校园生活,哈尔滨生活,学生会,HIT,哈尔滨工业大学,社会实践
投资理财,大盘,短线高手,内参,短线黑马,股票分析,股票投资
逛街,帅哥,看电影
名人明星,穿衣美容,新闻趣事,搞笑幽默,辽宁生活
微博奇葩
视频音乐
新闻趣事,视频音乐,星座命理
星座命理,湖南生活
穿衣美容
花开花落,夕阳西下,狐狸君小幂,新时代女孩
体育资讯,澳大利亚生活
美女,美食,旅游
美食,旅游,穿衣美容,搞笑幽默,海南生活
睡觉睡到自然醒,热情,校园,O 型血,唱 k,旅游,名人明星
搞笑幽默
微博奇葩
湖北生活
驻马店生活
萌宠
搞笑幽默,微博奇葩
英语,星座命理
搞笑幽默
名人明星,旅游,四川生活
旅游,新闻趣事,美食,名人明星,广东生活
gif,app,表情帝,图片,漫画,阿狸,动漫,萌宠,美女
微博奇葩
名人明星
视频音乐,星座命理

美食,旅游
微博奇葩
微博奇葩,新闻趣事,旅游,美食,萌宠
新闻趣事,河南生活,搞笑幽默,星座命理
武汉,曳步舞
二宫和也,大野智,樱井翔,松本润,相叶雅纪,arashi 嵐,Arashi 团饭,arashi
网购,段子,时尚,搞笑,音乐,心情语录,80 后
名人明星,美女,美食,穿衣美容
旅游,广东生活,微博奇葩
萌宠
KangSora,姜素拉,韩国女演员,韩国明星
视频音乐,穿衣美容,美食
搞笑幽默
足球,网球,欧美音乐,美剧,皇马,视频音乐,名人明星,旅游,美食,微博奇葩
星座命理
名人明星
微博奇葩,美食,穿衣美容
校园生活,星座运势,美图摄影,美食,名人明星
坚强,娱樂,听音乐,名人明星,新闻趣事,美食,搞笑幽默
美食,旅游,星座命理,四川生活,穿衣美容
广东生活
美食,新闻趣事,微博奇葩,名人明星,搞笑幽默
校园,舞蹈,音乐,钢琴,视频音乐
北京生活,名人明星
美食
新闻趣事
搞笑幽默
名人明星,美女,旅游,星座命理,萌宠
微博奇葩
名人明星,旅游
旅游
视频音乐,新闻趣事,台湾生活,美食,美女
视频音乐
穿衣美容
旅游,澳门生活
微博奇葩,星座命理,新闻趣事,搞笑幽默,北京生活
视频音乐,美食
新闻趣事,美食
视频音乐
微博奇葩,北京生活
搞笑幽默
微博奇葩
穿衣美容
美食

微博奇葩
视频音乐, 微博奇葩, 萌宠, 广东生活, 搞笑幽默
微博奇葩, 旅游, 美女
视频音乐
新闻趣事, 搞笑幽默, 视频音乐, 美食, 旅游
美女, 星座命理
萌宠, 搞笑幽默, 旅游, 美食
美食
搞笑幽默, 微博奇葩
旅游
萌宠, 名人明星, 搞笑幽默
美食, 旅游
美女, 穿衣美容, 萌宠
美女, 江苏生活, 新闻趣事, 搞笑幽默, 名人明星
郑州生活, 穿衣美容, 旅游
搞笑幽默
名人明星
旅游, 四川生活, 美食, 新闻趣事, 穿衣美容
名人明星, 新闻趣事, 旅游, 四川生活, 穿衣美容
旅游
搞笑幽默
茶道, 茶德, 茶艺
穿衣美容
美女, 广东生活
名人明星
美食, 旅游, 天津生活, 新闻趣事, 搞笑幽默
石家庄生活, 美食, 河北生活
视频音乐, 穿衣美容, 新闻趣事, 美食
旅游, 山东生活
萌宠
旅游, 美女, 视频音乐, 搞笑幽默, 山东生活
微博奇葩
穿衣美容
萌宠
福州生活, 福建生活
穿衣美容, 视频音乐, 搞笑幽默, 旅游, 美食
射手座, 新闻趣事, 美女, 旅游, 美食, 陕西生活
其他生活, 穿衣美容
美食, 星座命理, 名人明星
视频音乐
赛车, 冷笑话, 电影迷, 旅游, 搞笑幽默, 广东生活, 微博奇葩, 美女
情感生活, 八卦杂谈, 美食, 新闻趣事, 搞笑幽默, 山东生活
新闻趣事, 名人明星, 视频音乐, 北京生活, 旅游
旅游, 微博奇葩, 新闻趣事, 搞笑幽默

微博奇葩
新西兰生活
信仰科比,篮球缔造者
情感生活,育儿百科,苏州生活,旅游,微博奇葩,搞笑幽默,江苏生活,穿衣美容
美食爱好者,单纯,赚钱,K 歌,狗狗,水瓶,宝宝
搞笑幽默
广东生活
搞笑幽默
旅游,美食,微博奇葩,新闻趣事,广东生活
新闻趣事,搞笑幽默,福建生活,美食,旅游
名人明星,校园生活,美女,云南生活
微博奇葩
微博奇葩,旅游,美食
搞笑幽默
穿衣美容,旅游
美食,萌宠,穿衣美容,视频音乐,新闻趣事
广西生活
名人明星
名人明星,旅游,新闻趣事,美女,微博奇葩
巨蟹座公主,刘诗诗的小狮子,刘诗诗,四川生活
旅游
萌宠
MyEverything,LeeMinho,Minoz,李敏镐,小清新,学生一枚
美食,萌宠,微博奇葩,天津生活,搞笑幽默
江苏生活
搞笑幽默,视频音乐,微博奇葩,名人明星
自由,90 后,电子商务,潮流控,时尚,美女
搞笑幽默,新闻趣事,美食,旅游,福建生活
新闻趣事,海南生活
暗恋中但尚在团,美图摄影
旅游
美容服饰,新闻趣事,校园生活,花样美男,视频音乐,bigbang,完美主义,音乐爱好者
视频音乐
视频音乐
创意产业,创意设计,创投,科技,3d 打印
视频音乐
旅游
美容服饰,青春,欧美,爱猫,星座命理
胡思乱想,双子座,盗墓笔记,朋友,微博控,85 后,爱自由,涂鸦,死神,旅游
美食,旅游,搞笑幽默,新闻趣事,星座命理
新闻趣事
视频音乐,新闻趣事,美食
视频音乐
星座命理

新闻趣事,视频音乐,美女,旅游,名人明星
旅游,吉林生活,视频音乐
旅游
广东生活
巨蟹座,美食
旅游,善良
美女,微博奇葩,美食,旅游,视频音乐
名人明星,美食,穿衣美容,私人定制,烫睫毛,自然纯貂毛睫毛,韩国嫁接睫毛,持久
穿衣美容,Superjunior
上海生活,搞笑幽默,向日葵
名人明星
时尚达人,美食,旅游,美女
名人明星
八卦杂谈
美女,搞笑幽默,星座命理,穿衣美容,湖南生活
情感生活,购物狂,爱美食,电影迷,爱音乐,苹果控,赚钱,美女
新闻趣事,视频音乐,搞笑幽默,上海生活
浙江生活,旅游,萌宠
旅游,美女,名人明星,微博奇葩,北京生活
星座命理,新闻趣事,重庆生活,名人明星,穿衣美容
休闲,穿衣美容,星座命理
美食
视频音乐
微博奇葩,新闻趣事,江苏生活
浙江生活
穿衣美容
穿衣美容,新闻趣事,视频音乐,旅游,浙江生活
美女,美食,旅游,搞笑幽默,广西生活
美食
视频音乐
花样美男,情感生活,名人明星,美食
北京生活,新闻趣事
校园,插画,android,蓝色,巨蟹女,淡漠
微博奇葩,萌宠
新闻趣事,视频音乐,名人明星
视频音乐
新闻趣事,广东生活
星座命理
唱歌听歌,处女座,中医,文艺
美食,旅游,穿衣美容
穿衣美容
搞笑幽默
美食,美女,穿衣美容,搞笑幽默,广东生活
微博奇葩,视频音乐

搞笑幽默
中国基督教,青年人福音事工,敬拜,传福音,圣经,云南基督教
视频音乐
搞笑幽默,广东生活,新闻趣事,视频音乐,名人明星,打球是一种快感
爱自由,销售,投资理财,房地产业,微博奇葩
美女
萌宠
微博奇葩,新闻趣事,搞笑幽默
星座命理,微博奇葩,旅游,穿衣美容,广东生活
名人明星
穿衣美容
星座命理,四川生活,穿衣美容,美食,旅游
搞笑幽默,新闻趣事
萌宠
旅游
视频音乐,学生一枚,music,看电视,90
旅游,爱自由
美食,穿衣美容
名人明星,视频音乐
搞笑幽默
面包车,校车,客车,江苏生活
星座命理
纠结,学生,喜欢听歌,爱旅游,钢琴,爱笑,电影控,美食,旅游,美女
星座命理,视频音乐
名人明星,穿衣美容,北京生活,新闻趣事,搞笑幽默
旅游
爱读书爱小说,好性格时尚,温柔爱逛街,幽默搞笑,金牛女,猫咪控,喜欢音乐,游戏动漫,宅女一枚,设计
星座命理
情感生活,山东生活
穿衣美容,偶稀饭睡觉觉,旅游美食爱好者,小说迷,美食控,耽美,吃货,韩国,看小说,视频音乐
搞笑幽默
星座命理,微博奇葩,视频音乐,旅游,北京生活
八卦杂谈,微博奇葩
湖北生活
微博奇葩,名人明星
北京生活
美食
美食,喜欢听歌,旅游
河南生活,新闻趣事
微博奇葩,星座命理,视频音乐,新闻趣事,搞笑幽默
美食,视频音乐,旅游,重庆生活
旅游

萌宠,旅游,星座命理,美食
搞笑幽默
星座命理
美食,星座命理,名人明星,新闻趣事,旅游
穿衣美容,视频音乐,搞笑幽默,黄冈生活,名人明星
美食,旅游,新闻趣事,搞笑幽默,福建生活
读书分享,南京生活,江苏生活
新闻趣事,视频音乐,旅游,微博奇葩,湖北生活
新闻趣事,穿衣美容,美食,旅游,微博奇葩,安徽生活
微博奇葩
宅男,篮球爱好者,90后,双鱼座,视频音乐
美女
随性,bigbang,法国,视频音乐,福建生活
名人明星,萌宠
旅行,唱歌,视频音乐
美国生活,穿衣美容,旅游,美食
广东生活
宅男,淘宝控,旅游摄影,美食爱好者,热爱生活,时尚达人,策划,天蝎座的男生,天蝎座80后,
广东生活
陕西生活
旅游
穿衣美容,旅游,微博奇葩,搞笑幽默,新闻趣事
新闻趣事
美食
萌宠,旅游,美食,星座命理
旅游
太原生活,旅游,美食,名人明星,宠物图集
新闻趣事
金奎丽,萌
美女
微博奇葩
美食,新闻趣事,搞笑幽默,江西生活,视频音乐
微博奇葩
名人明星
搞笑幽默,新闻趣事,名人明星,穿衣美容,微博奇葩
微博奇葩,搞笑幽默,新闻趣事,江苏生活
美女
萌宠,旅游,美食,搞笑幽默,上海生活
新闻趣事,旅游,名人明星,视频音乐
美食,搞笑幽默,旅游,湖北生活
旅游,美食
郭静,CBA,曼联,美食
视频音乐,美女,搞笑幽默
星座命理

美食,微博奇葩,北京生活
搞笑幽默
美食,海南生活,视频音乐,旅游
视频音乐
旅游
视频音乐
江西生活
广东生活,搞笑幽默,新闻趣事
甘肃生活
美女
搞笑幽默,新闻趣事
名人明星,动画,艺术,绘画,喜欢听歌
穿衣美容
新闻趣事
搞笑幽默
名人明星,美女,香港生活
减肥,美女
萌宠,音乐,学生,小说控,小狗,时尚,王俊凯,王源
美食,旅游
星座运势,90 後,听音乐,广东生活
微博奇葩
旅游,搞笑幽默
新闻趣事,美女,星座命理,北京生活
星座命理,囧
美食
旅游
微博奇葩
搞笑幽默
旅游,美食,搞笑幽默,贵州生活,穿衣美容
旅游
美图摄影,80 後,耶稣爱你,Christian,信仰耶稣
名人明星
微博奇葩,贵州生活
旅游
四川生活
美女
萌宠,亮光,光亮
美女,微博奇葩,新闻趣事,搞笑幽默,美国生活
新闻,汽车,汽车展,展览,展会,山东生活
山 P 命,银魂控
90 后,巨蟹座,吃货
浙江生活,搞笑幽默
名人明星,搞笑幽默,新闻趣事,江苏生活
职场招聘,旅游,新闻趣事,河北生活

名人明星,旅游,视频音乐,新闻趣事
微博奇葩,新闻趣事,搞笑幽默
微博奇葩
穿衣美容
穿衣美容,名人明星,微博奇葩,江苏生活,新闻趣事
视频音乐
星座命理,视频音乐,美食,旅游,云南生活
旅游
江苏生活
名人明星,搞笑幽默
无锡生活,美食,江苏生活,穿衣美容,搞笑幽默
中亚商场,驰球,家用保险箱,保险柜,旅游,搞笑幽默
美食,萌宠,微博奇葩,搞笑幽默
广东生活,萌宠,旅游,搞笑幽默
微博奇葩
萌宠
旅游,新闻趣事,搞笑幽默,上海生活
美食,名人明星,旅游,星座命理,穿衣美容
穿衣美容
庚饭,五月天,欧美音乐,superjunior,摩羯座,青春
美图摄影,名人明星,美食,星座命理,新闻趣事,搞笑幽默
旅游,名人明星,美食,穿衣美容,微博奇葩
名人明星,视频音乐,微博奇葩,搞笑幽默,星座命理
星座命理,美女,萌宠,微博奇葩,美食
体育资讯,名人明星
搞笑幽默
视频音乐,猫,乐橙,善良,艺术
美女,搞笑幽默,微博奇葩,穿衣美容,视频音乐
時尚,八卦杂谈,创业,美女
旅游,内蒙古生活,美食,穿衣美容,视频音乐
北京生活
萌宠,穿衣美容,视频音乐,黑龙江生活,搞笑幽默
星座运势,读书分享,星座命理
视频音乐
旅游
美食
广西生活,微博奇葩,美食,搞笑幽默,名人明星
湖北生活
微博奇葩,新闻趣事,旅游,美食,星座命理
美食
搞笑幽默,旅游,美女
抹茶绿泥,GEO 防晒,闪电瘦,鲜橙瘦批发
北京生活,新闻趣事
婚礼,旅游,四川生活,美食

旅游, 穿衣美容, 浙江生活, 星座命理, 视频音乐
旅游, 搞笑幽默, 星座命理, 穿衣美容, 视频音乐
八卦杂谈, 校园生活, 新闻趣事, 温州生活
搞笑幽默, 新闻趣事, 校园生活, 宠物图集
八卦杂谈, 美图摄影, 其他生活, 旅游, 搞笑幽默
名人明星
视频音乐, 美食, 萌宠, 搞笑幽默
名人明星
美食
视频音乐, 微博奇葩, 旅游, 美女
穿衣美容
搞笑幽默, 新闻趣事, 视频音乐, 旅游, 美食
视频音乐
视频音乐
美女
美食, 美图摄影, 微博奇葩, 视频音乐, 名人明星, 旅游, 穿衣美容
旅游, 乐观
美女
旅游
旅游, 微博奇葩, 新闻趣事
旅游
摩羯姑娘
穿衣美容, 微博奇葩, 美食, 新闻趣事, 搞笑幽默
诗词, 文艺, 新闻, 文字, 搞笑幽默
搞笑幽默
搞笑幽默, 校园生活, 名人明星, 穿衣美容, 美食, 旅游, 视频音乐
美食, 穿衣美容, 名人明星, 微博奇葩, 搞笑幽默
美食
旅游, 穿衣美容
名人明星
搞笑幽默, 新闻趣事
吃货
微博奇葩, 旅游, 名人明星, 美女, 视频音乐
美食, 其他生活
微博奇葩
重庆生活, 紫菜
旅游
美食
美食
旅游
搞笑幽默, 岱超岱, 微博奇葩
微博奇葩, 穿衣美容, 星座命理, 美食, 旅游
音乐爱好者, 吃货, 交友, 散步, 写字, 听歌, 宅, 90 后, 睡懒觉
旅游

美女,美食,旅游,穿衣美容,星座命理
打游戏
萌宠
名人明星
穿衣美容
新闻趣事
旅游,房产,美食,健康养生,美女
星座命理
喜欢旅游,休闲,无聊,工作狂,散步,时装,寂寞,80后的尾巴,淘宝控,理财
视频音乐,北京生活,微博奇葩,美食,名人明星
视频音乐
天津生活
名人明星,美女
新闻趣事
旅游
微博奇葩,名人明星
美食,旅游,上海生活,萌宠,星座命理
名人明星
穿衣美容
萌宠,搞笑幽默
新闻趣事,微博奇葩,旅游,美食,视频音乐
山东生活
美女,穿衣美容,星座命理
旅游,视频音乐
美图摄影,动漫,视频音乐,校园生活,Base,白羊座
贵州生活
美女
搞笑幽默
美食,学生一族,睡懒觉,宅但是不想宅,柯南
上海生活
名人明星
旅游
旅游,自由,旅行,学生,热爱生活
云南生活
微博奇葩
旅游,星座命理,完美主义,音乐爱好者,动漫控
美女
星座命理
旅游,美食,新闻趣事
视频音乐
养生,健康,穿衣美容,山东生活
旅游
美食,温州生活,穿衣美容
旅游,视频音乐,四川生活

搞笑幽默,视频音乐
新闻趣事
旅游
微博奇葩
不入流投资,旅游
美女,名人明星,视频音乐,微博奇葩,搞笑幽默
微博奇葩,美女,新闻趣事,名人明星,搞笑幽默
视频音乐
穿衣美容,微博奇葩,美女,旅游,美食
旅游,新闻趣事,微博奇葩,四川生活
穿衣美容,美食,新闻趣事,江苏生活,名人明星
宅,游戏,动漫,搞笑幽默,视频音乐
美食,穿衣美容
搞笑幽默
旅游
旅游
新闻趣事,视频音乐
吃喝玩乐,爱自由,新闻趣事,江苏生活,搞笑幽默,微博奇葩
穿衣美容
穿衣美容,萌宠
微博奇葩
广西生活
穿衣美容,名人明星,旅游,美女,美食
微博奇葩,美女,旅游,视频音乐
旅游,穿衣美容,微博奇葩,搞笑幽默,北京生活
穿衣美容,美女
搞笑幽默
穿衣美容
搞笑幽默,吃喝玩乐,敏感,好性格,读书,双子座
其他生活
旅游
搞笑幽默
广东生活,微博奇葩,美女
广东生活,名人明星,新闻趣事,旅游
视频音乐,萌宠,搞笑幽默
微博奇葩,新闻趣事,搞笑幽默
微博奇葩
袁崇焕中学,LOL,懒
情感生活,美图摄影
旅游,视频音乐
黑龙江生活
名人明星
视频音乐
名人明星

名人明星
旅游,新闻趣事
包头生活
名人明星
搞笑幽默
夜猫子,资讯,单纯,囧,英语,互联网,营销,玩儿,八卦,穿衣美容
萌宠,美食
新闻趣事,八卦杂谈,名人明星,其他生活,美食
视频音乐
穿衣美容
单身,85 后,k 歌,养生,独立,穿衣美容,美食,微博奇葩,名人明星,搞笑幽默
宠物图集
穿衣美容
搞笑幽默
新闻趣事
搞笑幽默
星座命理,搞笑幽默
搞笑幽默
名人明星
名人明星
星座命理,名人明星,萌宠,旅游,穿衣美容
耽美,时尚潮流,动漫控,日本,时装,动漫,萌宠,美食
搞笑幽默,新闻趣事,萌宠,穿衣美容
穿衣美容
江苏生活
视频音乐
名人明星
新闻趣事,美食,萌宠,旅游,微博奇葩
新闻趣事
搞笑幽默
淘宝控,福州生活,欧美控,外貌协会,时装
美容服饰
茶,人生,音乐爱好者,高考
搞笑幽默
美食
美食
爱时尚，爱购物
视频音乐,云南生活
名人明星
读书分享,情感生活,搞笑幽默,旅游
上海生活
视频音乐
搞笑幽默
视频音乐

郑州生活,美食,旅游,新闻趣事,搞笑幽默,河南生活
星座命理
美女
美食
汉中生活
旅游
旅游,美食
微博奇葩
搞笑幽默,美容服饰,户外,時尚,旅游摄影,电视
名人明星,微博奇葩,星座命理,穿衣美容,视频音乐
星座命理
化妆品,自恋,投资理财,青春,名人明星,穿衣美容,山西生活,视频音乐,新闻趣事
美女
穿衣美容,新闻趣事,搞笑幽默,微博奇葩,视频音乐
新闻趣事,情感生活
爱家爱爸妈,爱自由,真诚坦率,重感情,爱猫狗,奋斗 ING,爱摄影,爱 K 歌,爱旅行
EXO,宅,听歌,90 后,萌宠,名人明星,穿衣美容
视频音乐,萌宠,日本生活
旅游
其他生活
成都生活
旅游,美食
美食,星座命理,视频音乐
台湾生活
美女
江西生活
萌宠
视频音乐
体育资讯,广州
搞笑幽默,新闻趣事
新闻趣事,美图摄影,美食,情感生活,IT 数码,视频音乐,美容服饰,名人明星,搞笑幽默,八卦杂谈
名人明星,美食,星座命理,穿衣美容,搞笑幽默
旅游
名人明星,旅游
搞笑幽默,四川生活
职场招聘,视频音乐,宠物图集,美食,情感生活,搞笑幽默,美容服饰,八卦杂谈,星座运势,IT 数码
美食,新闻趣事,校园生活,名人明星,济南生活
旅游
体育资讯
萌宠,美食,旅游,视频音乐,微博奇葩
名人明星,穿衣美容
视频音乐,旅游

陕西生活,搞笑幽默,新闻趣事,旅游
动漫,宁波生活,视频音乐
名人明星
名人明星
搞笑幽默,微博奇葩,视频音乐
学生一枚,时事,新闻趣事
穿衣美容
微博奇葩,美女,新闻趣事,视频音乐,搞笑幽默
新闻趣事
微博奇葩
职场招聘,名人明星,情感生活,视频音乐,星座运势,宠物图集,IT 数码,八卦杂谈,花样美男,美食
美食,名人明星,微博奇葩,新闻趣事,搞笑幽默
微博奇葩
旅游
微博奇葩,新闻趣事,美食
名人明星
搞笑幽默,河南生活
旅游,纠结
天津生活
美图摄影,喜欢旅游,股票,财经资讯,投资理财,看书
视频音乐,体育资讯
旅游,视频音乐
名人明星
萌宠
萌宠,新闻趣事,微博奇葩,搞笑幽默
沈阳生活,搞笑幽默,微博奇葩
星座命理
旅游,北京生活
仙剑,瓶邪,听音乐,旅游,穿衣美容,微博奇葩,星座命理,搞笑幽默
耽美,服装设计,美術生,开兴,双吴,吴亦凡,旅游
名人明星
搞笑幽默
美女
拍照,购物狂,名人明星,听歌,音乐,欧美控,外貌协会,穿衣美容,美女,香港生活
视频音乐,穿衣美容,旅游,美食
旅游,微博奇葩,名人明星
穿衣美容,萌宠,微博奇葩,新闻趣事
穿衣美容,自然,坚强,k 歌,汽车,双子座,偶稀饭睡觉觉,金融,阳光,爱生活
视频音乐
萌宠,视频音乐,微博奇葩,名人明星
天平座的孩子,工作是我的乐趣,生活点滴,美女爱好者,音乐爱好者,动漫爱好者
微博奇葩
美食

搞笑幽默,萌宠
美食,视频音乐,旅游
美女,穿衣美容
搞笑幽默
游戏,新闻趣事,江苏生活,搞笑幽默,美食,旅游
微博奇葩,美食,旅游,穿衣美容,广州生活
Travel,旅游
穿衣美容,视频音乐,美食,微博奇葩,旅游
旅游,微博奇葩,新闻趣事,搞笑幽默,上海生活
旅游
完美主義者,强迫症,美食,旅游,穿衣美容
河南生活,名人明星
名人明星
微博奇葩,广东生活,旅游,美食,搞笑幽默
旅游,微博奇葩,穿衣美容,新闻趣事,搞笑幽默
深圳生活,广东生活
少女时代,90後,学生一族,摄影控,化妆,情感,爱自由,旅游,视频音乐,广西生活
穿衣美容
旅游
视频音乐,搞笑幽默,新闻趣事,广东生活,美食
游戏,美食,IT数码,其他生活
搞笑幽默,北京生活
旅游,美食,穿衣美容,星座命理,搞笑幽默
旅游
广东生活,新闻趣事
微博奇葩,新闻趣事,旅游,美食,山西生活
旅游
新闻趣事,名人明星,微博奇葩,搞笑幽默,贵州生活
读书分享,美图摄影,读书,水瓶,吃货,神经质,强迫症,搞怪,收藏,旅游
星座命理,视频音乐,美食,浙江生活,美女
旅游,微博奇葩,四川生活
software,Software,软件,Photoshop,ps,旅游
美食,健身,游泳,肌肉,胸肌,翘臀,成熟,温柔,man,1
名人明星
星座命理
自行车,爱音乐,爱自由,新闻资讯,shopping,运动,单身,交朋友,睡觉,微博奇葩
穿衣美容
穿衣美容
旅游,美食,广东生活
大学生,上海生活
美食,美女,穿衣美容
搞笑幽默,名人明星,打球,懒人,玩
名人明星,萌宠
搞笑幽默,B计划,宝妮,后期,翻唱,CV,广播剧,绿冶仙踪

视频音乐
美食,旅游,穿衣美容,日本生活
旅游,微博奇葩
旅游,穿衣美容,萌宠,星座命理,搞笑幽默
名人明星,美食,旅游,视频音乐
美食
赣州,关西围屋,小武当,龙南县,微博奇葩,旅游,江西生活,新闻趣事,搞笑幽默
名人明星
球迷,米兰圈,AC 米兰,米兰,体育资讯
视频音乐,搞笑幽默
美女
旅游
广东生活
龙族,90 后,巨蟹座,家庭教师 Reborn
视频音乐
微博奇葩,搞笑幽默,旅游,美食,穿衣美容
搞笑幽默
美食,四川生活,新闻趣事,穿衣美容,微博奇葩
视频音乐
外貌协会,吃货一枚,新闻趣事,八卦杂谈,微博奇葩,美食,视频音乐,搞笑幽默,旅游
美食,云南生活,新闻趣事
视频音乐,美食,萌宠,星座命理,穿衣美容
微博奇葩,福建生活,搞笑幽默
山东临沂,80 後,原创,文化,志愿者,旅游,搞笑幽默,新闻趣事,山东生活,微博奇葩
美食,新闻趣事,动漫,校园生活,日本生活,日本留学中,私立希望峰学院
爬山
视频音乐,娄底生活,湖南生活
美食,穿衣美容
搞笑幽默
美食,旅游,名人明星,搞笑幽默,微博奇葩
萌宠,名人明星,学生一枚,旅游,视频音乐,美食
视频音乐,穿衣美容
名人明星,旅游,美食,微博奇葩,新闻趣事
视频音乐,读书分享,美图摄影,旅游
星座命理,美食,萌宠,名人明星,旅游
新闻趣事
视频音乐
视频音乐
美食
爱笑,散步,服饰,随性,自然
名人明星
密室逃脱,真人密室逃脱,东山口
小泰克,玩具,动漫,海贼王,圣衣神话,变形金刚,哆啦 A 梦,动画
美食

旅游
新闻趣事
搞笑幽默, 穿衣美容, 星座命理
穿衣美容, 美食, 旅游, 汕头生活, 新闻趣事
天津生活, 美食, 萌宠, 微博奇葩, 旅游
美食
旅游
穿衣美容, 星座控, 无厘头, 外貌协会, 爱幻想
美食, 旅游, 萌宠, 新闻趣事, 福建生活
微博奇葩, 搞笑幽默
美食, 广东生活
团团爱
萌宠
视频音乐, 旅游, 新闻趣事, 搞笑幽默, 北京生活
造型师, 摄影, 礼服, 婚纱
10 号球衣, 国安是冠军, 双子座, 德拉克斯勒, 阿森纳, 德国
美容服饰, 安徽生活, 搞笑幽默, 新闻趣事, 美食, 萌宠
江西生活
视频音乐, 名人明星
名人明星, 视频音乐, 微博奇葩, 搞笑幽默, 新闻趣事
视频音乐, 美容服饰, 花样美男, 上海生活, 服装, 时尚
视频音乐
河南生活
星座命理, 上海生活, 旅游, 美食, 萌宠
搞笑幽默, 美食
旅游, 视频音乐, 新闻趣事, 搞笑幽默, 广东生活
美食, 旅游, 名人明星
视频音乐, 美食, 新闻趣事, 微博奇葩, 北京生活
微博奇葩, 搞笑幽默
北京生活, 星座命理
星座命理
名人明星
江西生活
武汉生活, 新闻趣事, 搞笑幽默
穿衣美容, 名人明星, 旅游, 美食, 视频音乐
广东生活
美食
美女
新闻趣事, 萌宠, 美食, 微博奇葩
旅游, 美食
旅游, 上海生活, 星座命理
穿衣美容, 美食, 名人明星
微博奇葩
美食, 旅游, 名人明星, 穿衣美容, 星座命理

新闻趣事,安徽生活,微博奇葩
RTS,MOBA
名人明星
美女,微博奇葩,搞笑幽默,新闻趣事
旅游
搞笑幽默,旅游
河北生活,搞笑幽默
搞笑幽默,名人明星,穿衣美容,微博奇葩
穿衣美容
八卦杂谈,广州生活,新闻趣事,微博奇葩,名人明星,搞笑幽默,星座命理
巨蟹女,动漫迷,学习,fashion,日本,旅游
绿色与白色,爱睡觉,积极男士,NBA 球迷,投资理财,汽车,胡歌的胡椒粉,90 后,时尚潮流,重庆生活
美食,新闻趣事,名人明星,穿衣美容,美女
美食
搞笑幽默,浙江生活
真实力,美食
微博奇葩,视频音乐,搞笑幽默
河南生活
旅游
旅游
新闻趣事
旅游
北京生活
搞笑幽默,韩日店
名人明星
山东生活,新闻趣事,旅游,微博奇葩,星座命理
旅游,萌宠,穿衣美容,美女,视频音乐
搞笑幽默,微博奇葩
视频音乐,美食
旅游,名人明星,美食,穿衣美容,浙江生活,新闻趣事
视频音乐
读书分享,美图摄影,新闻趣事,校园生活,微博奇葩,旅游
搞笑幽默
新闻趣事
旅游,其他生活
新闻趣事
穿衣美容
广东生活
搞笑幽默,新闻趣事
搞笑幽默,微博奇葩,视频音乐,广东生活
旅游,美女,微博奇葩,新闻趣事,搞笑幽默
爱玩,冷笑话,执着,视频音乐,旅游
名人明星

旅游
微博奇葩, 旅游, 穿衣美容, 河北生活
互粉, 星座命理, 学生一族, 火影, 柯南, 理财, 淘宝控, 赛车, 微博奇葩, 搞笑幽默
视频音乐, 搞笑幽默, 新闻趣事, 旅游
美食, 视频音乐, 美女
旅游
美食, 山东生活
美女
视频音乐, 美食, 穿衣美容
新闻趣事, 名人明星, 穿衣美容, 星座命理
搞笑幽默
幸福的小女人, 成人用品商城, 搞笑幽默, 美食, 旅游, 穿衣美容, 美女
旅游, 新闻趣事
旅游, 美食, 萌宠
微博奇葩
旅游, 美食, 视频音乐, 新闻趣事, 江西生活
穿衣美容, 搞笑幽默, 辽宁生活
广东生活
福建, 美食, 旅行, 阅读, 文学, 新闻趣事
美图摄影, 旅游, 美食, 穿衣美容, 视频音乐, 新闻趣事
萌宠, 旅游, 穿衣美容
爱旅游, 爱电影, 穿衣美容
旅游, 胡海龙
旅游, 新闻趣事, 视频音乐, 穿衣美容, 美食
河北生活
美女
视频音乐
名人明星, 美女, 新闻趣事, 穿衣美容, 美食
江苏生活, 萌宠, 美食, 旅游
名人明星
美食
交友, 淘宝控, 微博奇葩, 视频音乐
名人明星
视频音乐
名人明星, 旅游, 搞笑幽默, 萌宠, 美食
视频音乐, 新闻趣事, 穿衣美容, 旅游, 美食
穿衣美容
美图摄影, 读书分享, 情感生活, 校园生活, 星座命理, 旅游, 穿衣美容
微博奇葩, 美女, 日本生活
搞笑幽默
旅游, 视频音乐
北京生活
萌宠
旅游

名人明星
萌宠
旅游, 美食, 萌宠, 搞笑幽默, 穿衣美容
微博奇葩, 时尚潮流, 唱歌, 蓝色, 火影, 创意, 绘画
萌宠
视频音乐, 安徽生活
新闻趣事, 旅游, 美食, 穿衣美容, 美女
穿衣美容, 美食
搞笑幽默, 美女
搞笑幽默, 微博奇葩
穿衣美容, 新闻趣事, 广东生活
学生一枚, 90, 乐橙一枚, 宅女一枚, 乐橙, 疯狂
学术研究, 脑科学, 视频音乐, 加拿大生活
萌宠, 北京生活, 视频音乐, 美食
新闻趣事, 美食, 美女, 搞笑幽默, 微博奇葩
微博奇葩, 上网, 淡定
萌宠
广东生活
名人明星
新疆生活, 萌宠
视频音乐
重口味, 搞笑幽默, 名人明星, 新闻趣事, 美女
北京生活
名人明星
穿衣美容
星座命理
搞笑幽默
旅游, 美食, 星座命理, 江苏生活
旅游
美食
星座命理
美女
旅游, 美食
新闻趣事, 视频音乐, 搞笑幽默
音乐爱好者, 舞蹈演员, 摄影
美食, 旅游, 穿衣美容
穿衣美容, 美食, 搞笑幽默
穿衣美容
名人明星, 美食, 萌宠, 星座命理
搞笑幽默, 穿衣美容
搞笑幽默
美女
燕山大学, 骑行天下, 摄影爱好者, 旅游
旅游

福建生活,红茶,乌龙茶,铁观音,普洱茶,茶叶,茶艺,茶文化,紫砂壶,茶道
搞笑幽默
微博奇葩
美食,金华生活,浙江生活
欧美控,重口味,幽默,星座控,坚强,阳光,浪漫,网购,喜欢听歌,生活大爆炸
X 昂,战勇,战士,45 号讨魔分队,罗斯
美女
美食,新闻趣事,视频音乐,旅游
穿衣美容
漫画,小说,秦时明月,古风音乐,法律,东野圭吾,游戏动漫,龙族,诛仙,宅
旅行,读书,教育
视频音乐,星座命理
美食
福建生活
视频音乐
名人明星,穿衣美容
穿衣美容,星座命理,微博奇葩,搞笑幽默,视频音乐
新闻趣事,名人明星,旅游,星座命理,穿衣美容
重庆生活,新闻趣事,搞笑幽默,美食,旅游
视频音乐
视频音乐,美食,萌宠,星座命理,美女
星座,美食,个性,天蝎座,吃货,女人,seo,市场营销,生活大爆炸,爱吃
休闲,时尚潮流,穿衣美容
广东生活
旅游,希腊语
搞笑幽默,名人明星
旅游,美食,萌宠,微博奇葩,视频音乐
新闻趣事,美食,搞笑幽默,四川生活
新闻趣事,星座命理,名人明星,微博奇葩
名人明星
浙江生活
少女时代,美食
名人明星
新闻趣事,广东生活,穿衣美容,美女
名人明星,新闻趣事,穿衣美容,美食,旅游
其他生活
美食控,阳光,逛街,开心,双鱼,穿衣美容,创意,小说,喜欢音乐,旅游
广东生活
爱唱歌,喜欢音乐,懒人,旅游,穿衣美容
茂名生活,广东生活
自由职业,旅游
微博奇葩,名人明星,旅游,视频音乐
狮子女,射手男,处女男,白羊男,穿衣美容,美女
萌宠

无厘头,美食控,看电影,穿衣美容
美食,旅游,穿衣美容,新闻趣事,搞笑幽默
名人明星,美食,旅游,视频音乐
视频音乐
旅游
名人明星
星座命理
穿衣美容
星座命理,视频音乐,穿衣美容
视频音乐
穿衣美容
佛山生活,广东生活
萌宠
美食,新闻趣事,名人明星,旅游,陕西生活
上海生活
搞笑幽默,穿衣美容,美食,旅游,视频音乐
微博奇葩
新闻趣事,名人明星,萌宠,美食,旅游
旅游
美女,名人明星,搞笑幽默,新闻趣事,微博奇葩
旅游
新闻与传播学院,2013 届考生,新闻与传播学,传播学,新闻学,南京师范大学院
美食
穿衣美容
美女
搞笑幽默
旅游
微博奇葩,视频音乐,星座命理
萌宠,美食,旅游
名人明星,美食,旅游,视频音乐
美图摄影,宠物图集,搞笑幽默,微博奇葩,萌宠,旅游,美女,视频音乐
视频音乐
常德生活
搞笑幽默,广东生活,旅游,星座命理,视频音乐
广西生活
旅游,四川生活
体育资讯
咸宁生活
新福克斯,厦门,视频音乐,美女,旅游,福建生活
湖南生活
美女
旅游
美女,微博奇葩,搞笑幽默,视频音乐,美食
视频音乐

闺蜜照,摄影工作室,拍照,宝宝照,全家福,时尚摄影,艺术写真,梦工场婚纱,梦工场,常州婚纱摄影
新闻趣事,星座命理,江苏生活
新闻趣事
美女,执着,体育,双子,维尼夫妇,学生,自由,杂志,吃货一枚,爱生活
视频音乐,萌宠,微博奇葩,名人明星,新闻趣事
Music,唱k,胡思乱想,耽美,同性恋支持者,欧美音乐,视频音乐
微博奇葩,旅游
美食
美女,零配件,及,电脑,手机,出售
星座命理,新闻趣事,旅游,美食
赚钱,冷笑话,电影迷,街舞,淘宝掌柜,穿衣美容,美女,名人明星,浙江生活,视频音乐
权志龙,山下智久
美食
搞笑幽默
萌宠
美图摄影,上网,旅行,唱歌
美食
萌宠
美食,微博奇葩,搞笑幽默
搞笑幽默
私人手工定制,优秀婚礼跟拍,成都手工婚礼,创意婚礼定制,成都生活,旅游,摄影师,情感,love
美食
旅游
视频音乐
搞笑幽默
桂林生活
北京生活
微博奇葩,搞笑幽默,广东生活
视频音乐,新闻趣事,美食
搞笑幽默,旅游,视频音乐,名人明星,微博奇葩
美食爱好者,喜欢听歌,旅游
宅男,爱音乐,紫色,旅游,视频音乐
萌宠,搞笑幽默,微博奇葩,广东生活,新闻趣事
微博奇葩
萌宠,微博奇葩,搞笑幽默,穿衣美容,星座命理
福建生活,搞笑幽默,视频音乐,美女
新闻趣事
体育,手机,移动互联网,书,music,篮球爱好者,世界杯,皇马,旅游
名人明星
丧心病狂,EXO,金钟仁,旅游
旅游
名人明星

新闻趣事
90 後,低调,创造力,明星梦,擅长唱歌,爱范冰冰,喜欢唱歌,爱陈赫,演员梦，潮，唯美
新闻趣事,微博奇葩,搞笑幽默,星座命理,江西生活
村民,兴迷,吉他,电影迷,00 后,学生（作业）党,exo 中毒者
微博奇葩
星座命理
名人明星,新闻趣事,星座命理
旅游
长腿偶吧控
视频音乐
穿衣美容,星座命理
读书分享,旅游
名人明星
视频音乐,新闻趣事
微博奇葩
美女
名人明星,旅游,新闻趣事,山东生活,穿衣美容
体育资讯,新闻趣事,美女,搞笑幽默,篮球爱好者,NBA,篮球,皇马,爱音乐,玩儿
星座命理,萌宠
美食
旅游
微博奇葩,搞笑幽默
普通人,在路上,森系,英国控,文艺青年,美食
江苏生活,穿衣美容,美食,新闻趣事,名人明星
美女
90
音樂,小清新,读书分享,美图摄影,校园生活
星座命理
视频音乐
微博奇葩
视频音乐,焦作生活,美食,八卦杂谈,美女
北京生活,搞笑幽默
萌宠,穿衣美容
微博奇葩
微博奇葩
名人明星
美女
穿衣美容
名人明星,山东生活
旅游,星座命理,穿衣美容,美食,名人明星
健康养生,美食,美容服饰,其他生活
搞笑幽默
美食,旅游
欧美控,浪漫,旅游,设计,奢侈,时尚,Burberry,Prada,奢侈品代购,湖北生活

旅游,搞笑幽默,新闻趣事,萌宠,微博奇葩
北京生活,穿衣美容,名人明星
视频音乐,美食,旅游,搞笑幽默
新闻趣事
英国生活
名人明星,视频音乐
名人明星,新闻趣事,视频音乐,微博奇葩,美女
服装,美女,微博奇葩,广东生活
喜欢听歌,爱美食,吃货一枚,真诚,夜猫子,萌宠,美食,星座命理,视频音乐,搞笑幽默
旅游
视频音乐,旅游,穿衣美容,执着,90,狮子座,音乐爱好者,沉默
新闻趣事,莆田生活,吃喝玩樂,小县大爱,老乡会,记录点滴生活
广东生活,新闻趣事,旅游,美女,美食
名人明星
穿衣美容
搞笑幽默
名人明星
萌宠
美食
咖啡,微博奇葩,新闻趣事,旅游,萌宠,搞笑幽默
视频音乐
旅游,书籍,旅行,摇滚
微博奇葩,美女
旅游,广东生活,美食
名人明星,微博奇葩
微博控,新闻趣事,安阳生活,搞笑幽默,河南生活,视频音乐
微博奇葩,美食,美女,视频音乐,新闻趣事
萌宠
搞笑幽默,新闻趣事,视频音乐,旅游,美食
星座命理
微博奇葩,化妆品,穿衣美容,新闻趣事,广东生活,美女,女人,搞笑幽默,旅游,美食
旅游,名人明星
美女,广东生活
海贼王,旅游摄影,音乐,人生,心情随笔,动漫,美图,插画,旅游,星座命理
北京生活,微博奇葩,新闻趣事,星座命理,搞笑幽默
美食控,名人明星,搞笑幽默,微博奇葩,萌宠
旅游
星座命理,萌宠,穿衣美容,微博奇葩,搞笑幽默
名人明星
萌宠,微博奇葩,北京生活,搞笑幽默
名人明星,美女,旅游,视频音乐,贵州生活
美食,视频音乐,IT 数码,搞笑幽默,成都生活
名人明星,视频音乐
美食,旅游

搞笑幽默
广东生活
美女,旅游,萌宠,视频音乐
新闻趣事,穿衣美容,名人明星,旅游,美食
旅游,exo,足球,摩羯座
视频音乐
视频音乐,星座命理,美食,萌宠
旅游,云南生活,名人明星,视频音乐
名人明星,潮流,无聊,逛街,爱睡觉
星座命理
旅游,穿衣美容,搞笑幽默,微博奇葩,四川生活
视频音乐
招牌发光字,发光字营销,发光字制作,发光字,招牌字,广告字,城市亮化,定做招牌字,LED 发光字,东莞做字
足球爱好者,德国,南宁生活,rose,爱睡觉,吃货一枚,学习,海绵宝宝,小王子
旅游
星空控,偶尔文艺犬,神坑小学生,拜师科比,火影苦追
穿衣美容
二次元,宅,ACG,游戏,动漫,视频音乐,微博奇葩,搞笑幽默
旅游
美女,北京生活,旅游,美食
北京生活,穿衣美容,名人明星,旅游,美食
视频音乐,IT 数码,体育资讯,唐山生活,搞笑幽默,星座命理,微博奇葩
疯子,敏感,善良,爱自由,坚强,人生,心情,宅,小清新,新闻趣事
陕西生活
美女
搞笑幽默
旅游
动漫
美食
其他生活
香港生活
乐天派,武艺
爱逛街,爱睡觉,爱设计,爱看小说,爱打羽毛球
体育资讯,校园生活,江西生活
穿衣美容
交友,运动,足球迷,河南生活,美食,旅游,美女,搞笑幽默
美女
搞笑幽默
星座命理,搞笑幽默,新闻趣事,视频音乐,微博奇葩
视频音乐,旅游,美食,星座命理,美女
视频音乐
葫芦岛生活,穿衣美容
美食,穿衣美容,美女,安徽生活

萌宠,视频音乐,美女,星座命理
美女,体育,90,喜欢听歌,猫控,视频音乐
北京生活
名人明星,视频音乐
美女
旅游,美食,星座命理,穿衣美容
名人明星
旅游,穿衣美容
微博奇葩
微博奇葩
百色生活
搞笑幽默
新闻趣事
美女
名人明星
美食,新闻趣事,视频音乐,美女,旅游
名人明星,视频音乐,搞笑幽默,k歌,臭美,善良,喜欢看偶像剧,做最美的自己,浪漫,微博
萌宠,微博奇葩,搞笑幽默,视频音乐
白羊座,爱音乐,海贼王,看电影,怀旧
美食
名人明星
美食,名人明星,视频音乐,星座命理,穿衣美容
搞笑幽默,八卦杂谈,星座运势,情感生活
愤青,喜欢音乐,诗歌,淘宝控,开心,美食
微博奇葩,美食,旅游
穿衣美容
旅游,微博奇葩,星座命理,穿衣美容,新闻趣事
新闻趣事,福建生活
旅行,网购,美食,吉他
赣州生活,美食,旅游,新闻趣事,搞笑幽默
星座命理
旅游摄影,梦想,电脑,时尚潮流,淡定,冷笑话,随性,科技,矛盾体,小清新
旅游
视频音乐
名人明星
美食,微博奇葩,视频音乐,穿衣美容,名人明星,浙江生活
美女
星座命理
视频音乐
美女,萌宠
新闻趣事,搞笑幽默,微博奇葩,穿衣美容,名人明星
穿衣美容,美食
南昌生活,搞笑幽默,星座命理,穿衣美容,名人明星
视频音乐,新闻趣事,搞笑幽默,天津生活

搞笑幽默,视频音乐,微博奇葩,美食,名人明星
搞笑幽默,旅游,穿衣美容,视频音乐,美食
名人明星,天津生活
八卦杂谈
视频音乐
搞笑幽默
美食,微博奇葩
视频音乐,新闻趣事,搞笑幽默,名人明星
时尚,摄影,数码,八卦
数码,八卦,宅,购物
旅行,时尚,摄影,数码,八卦
马上就要毕业了,宅,学生一枚,发呆,90後,电影,军事,游戏动漫
搞笑幽默,新闻趣事,旅游,肇庆生活
美女,名人明星,视频音乐
天津生活
美食,视频音乐,广东生活
视频音乐
其他生活
时尚,香水,逛街
广州90后,穿衣美容,萌宠
视频音乐
狮子女,music,科比,hiphop
美发,健康,看书
新闻趣事
视频音乐
其他生活,美食
美发,瘦身,瑜伽,音乐,星座
美发,瘦身,香水,音乐,星座
旅游
名人明星
美食,搞笑幽默,名人明星,旅游,萌宠
旅游
视频音乐,名人明星,美女,搞笑幽默,微博奇葩
穿衣美容,视频音乐,新闻趣事,搞笑幽默,云南生活
美食,美女,穿衣美容
没什么好的
名人明星,视频音乐,新闻趣事,搞笑幽默,长沙生活
搞笑幽默
穿衣美容,美食
旅游美食,星座命理
主持,谢娜,穿衣美容
八卦杂谈,名人明星,视频音乐,搞笑幽默,校园生活
美女,微博奇葩,穿衣美容,旅游
萌宠,视频音乐,澳大利亚生活

美食,旅游,名人明星,穿衣美容,视频音乐
视频音乐
旅游,美食,微博奇葩,穿衣美容,美女
名人明星,美女,开封生活,新闻趣事,视频音乐,河南生活
美图摄影,体育资讯
穿衣美容
星座命理,搞笑幽默,微博奇葩,新闻趣事
穿衣美容,视频音乐,名人明星,搞笑幽默,微博奇葩
搞笑幽默,美国生活
搞笑幽默
教育,时尚,健康,电影
彩妆,香水,电影
旅游,游泳,电影
新闻趣事
美发,瘦身,星座
八卦杂谈
重口味,Music,名人明星
爱幻想,睡觉,宅,90后,心理,育儿,书法,善良,吃,爱唱歌
旅游,星座命理,多愁善感,胡思乱想
搞笑幽默,宝宝,睡觉,动漫,学生
美女,重口味,情感生活,八卦杂谈,视频音乐
穿衣美容
情感生活,莆田生活
萌宠,搞笑幽默,穿衣美容
旅游,医学,体育
新闻趣事
DIY,文学,旅游,名人明星,微博奇葩,穿衣美容,视频音乐
新闻趣事,校园生活,视频音乐,搞笑幽默,北京生活
穿衣美容
天津生活
上海生活,新闻趣事,微博奇葩,搞笑幽默
旅游,护肤,天蝎女,爱自由,猫,浪漫,看书,上网,80後,旅游美食爱好者
搞笑幽默
微博奇葩,名人明星,视频音乐
视频音乐,美食,新闻趣事
体育资讯
音乐,军事,汽车
学生一族,fashion,女人,南宁生活,美食,穿衣美容,广西生活
化妆造型设计师,穿衣美容
萌货
浙江生活
旅游,美食,星座命理,穿衣美容,美女
视频音乐
搞笑幽默

北京生活
名人明星, 微博奇葩
视频音乐, 新闻趣事, 搞笑幽默, 浙江生活
爱自由, 耽美, 潮人, 时尚达人, 完美主义, 梦想, 名人明星
广东生活
足球装备, 上海生活, 时尚, 运动, 足球, 旅游
搞笑幽默
旅游, 新闻趣事, 美女, 微博奇葩, 搞笑幽默
旅游
搞笑幽默, 新闻趣事, 河南生活
美女
音乐, 时尚, 摄影, 手表, 汽车, 旅游
南阳生活
搞笑幽默
校园生活, 美图摄影, 宠物图集, 读书分享, 名人明星, 美女, 穿衣美容, 微博奇葩
新闻趣事
搞笑幽默
黑龙江生活, 搞笑幽默, 穿衣美容
名人明星
校园生活, 美食, 搞笑幽默
新闻趣事, 视频音乐, 美容服饰, 旅游, 美食, 广东生活, 穿衣美容
美食, 游戏, 动画, 旅游
美女, 旅游, 搞笑幽默, 萌宠, 加拿大生活
美食
体育资讯, 新闻趣事
天蝎, 马英九脑残饭, 神经敏感, 教师, 传媒, 唱歌, 播音主持, 钢琴
名人明星, 微博奇葩, 视频音乐, 新闻趣事, 搞笑幽默
新闻趣事, 视频音乐, 穿衣美容, 名人明星, 重庆生活
搞笑幽默, 新闻趣事, 美食, 旅游, 四川生活
旅游, 新闻趣事, 穿衣美容, 微博奇葩, 搞笑幽默
文艺, 图书馆, 东华, 咖啡, 美食
新闻趣事
穿衣美容
穿衣美容, 视频音乐, 新闻趣事, 福建生活, 旅游
微, 金融, 微金融, 大数据, 综合金融, 中国平安, 平安财富
微博奇葩, 搞笑幽默, 视频音乐, 穿衣美容, 名人明星, 美女
新闻趣事
视频音乐, 新闻趣事, 搞笑幽默
旅游
美容服饰
旅游
古灵精怪, 皮鞋控, 黑袜控, 板鞋控, 白袜子, 金融系在读生, 小傻瓜, 白羊座, 执着, 随性
美女
旅游, 穿衣美容, 星座命理, 搞笑幽默, 北京生活

新闻趣事
搞笑幽默
视频音乐,搞笑幽默,旅游,美食
完美主义,低调,我就是我,双重性格,旅游
名人明星,旅游,微博奇葩,新闻趣事
穿衣美容,星座命理,旅游,美食,萌宠
穿衣美容
旅游
云南生活
霹雳布袋戏道友,平胸,不睡觉会死,双子座,旅游,微博奇葩,星座命理,穿衣美容,美食,萌宠
光疗甲,美甲
皮肤科,皮肤病,医生,医疗,医学,健康,DR,祥云医疗,新闻趣事
新闻趣事,视频音乐,星座命理,旅游,微博奇葩
美食
摩羯,萌宠,旅游,新闻趣事,搞笑幽默,广东生活
萌宠
美图摄影,新闻趣事,浙江生活,美食,微博奇葩,视频音乐,搞笑幽默
旅游
新闻趣事
穿衣美容
名人明星
名人明星
微博奇葩
名人明星
微博奇葩,夜猫子,迷糊
名人明星,星座命理,穿衣美容,旅游
视频音乐
美食
美图摄影
微博奇葩
佛教,美食
搞笑幽默
名人明星,美食,星座运势,美容服饰,旅游,萌宠,穿衣美容,美女
名人明星
火影忍者,海贼王,JAY,动漫迷,视频音乐
淮北生活,美食,美女,新闻趣事,搞笑幽默
视频音乐
美食
美食
体育资讯,美图摄影,IT 数码,金融,运动,科技,篮球
名人明星
美食,新闻趣事,视频音乐,搞笑幽默
视频音乐
旅游

星座命理
微博奇葩
视频音乐
旅游
搞笑幽默
名人明星,新闻趣事,微博奇葩,穿衣美容
穿衣美容,微博奇葩,新闻趣事,搞笑幽默,绵阳生活
校园生活,搞笑幽默
星座命理
美女
教育就业,团购,it 互联网,IT 数码
旅游,军事天地,车世界,财经资讯,美女,蒙古,警察,新疆生活
搞笑幽默
唱歌,摄影,时尚潮流,美食,星座命理,新闻趣事,搞笑幽默
美女,穿衣美容,新闻趣事,视频音乐,名人明星
美食,湖北生活,新闻趣事,美女,搞笑幽默
日本生活,美图摄影,搞笑幽默
美食,旅游,视频音乐,搞笑幽默
旅游,新闻趣事,浙江生活
吃,吃货,美食家,食品安全,膳食营养,美食天下,绿色健康,聚会聚餐,健康养生,美食达人
微博奇葩,名人明星
四川生活,搞笑幽默,微博奇葩
美食,长沙生活,微博奇葩,搞笑幽默,新闻趣事,湖南生活
美食
重口味,IT 数码,八卦杂谈,名人明星,搞笑幽默
吃货一枚,狗狗,欧美控,动漫控
新闻趣事,视频音乐,搞笑幽默,微博奇葩
萌宠
穿衣美容,名人明星,新闻趣事,美食,加拿大生活
搞笑幽默,微博奇葩,新闻趣事,视频音乐,福建生活
星座命理
新闻趣事
旅游,美食
灿烈,一块二,鹿晗,美食,旅游
新闻趣事,微博奇葩,旅游,搞笑幽默,视频音乐
广东生活,快乐有我
萌宠
新闻趣事
科研,看电影,网球,旅游
搞笑幽默
美女
微博奇葩
星座命理,微博奇葩
旅游

电影,宅,安静,天蓝,篮球
美女
旅游,微博奇葩,美食,美女,穿衣美容
视频音乐
视频音乐,美食,萌宠,微博奇葩
视频音乐,微博奇葩,穿衣美容,新闻趣事,搞笑幽默
湖北生活
校园生活,情感生活
新闻趣事,穿衣美容,美食
美女
视频音乐,搞笑幽默,旅游,萌宠
新闻趣事
穿衣美容
搞笑幽默
疯狂,腹黑,台球,爱听歌,爱睡,爱吃,篮球,曳步舞者,白羊,中二
视频音乐
微博奇葩
微博奇葩
名人明星
宠物图集
美容服饰,穿衣美容
搞笑幽默,微博奇葩,视频音乐
美食
爱穆,90后,睡觉睡到自然醒
旅游
穿衣美容,搞笑幽默,新闻趣事,星座命理
唯爱成,爱小草,爱二贝,爱款爷,爱小腻
小说迷,交朋友,学生一族
名人明星,星座命理
搞笑幽默
美图摄影,读书分享
搞笑幽默
美食
视频音乐
校园生活,旅游,新闻趣事,八卦杂谈,美女
美女
美女,辽宁生活,旅游爱好者,天平座,思考
双鱼女,韩国,BIGBANG,双重性格,爱玩,宅女,seo,海绵宝宝,狗狗
新闻趣事,穿衣美容,美女,星座命理,旅游
双鱼座,模特,学生,电影演员,时尚,80后,香港藝人,生活
视频音乐,新闻趣事,美食,旅游,呼和浩特生活
名人明星
星座运势,旅游,美容服饰,育儿百科,名人明星
名人明星

搞笑幽默
美食
星座命理
广东生活
时尚,浪漫,生活,证券市场,移动互联网,美食,小说,双鱼座
双鱼座,天蝎座,电影,短信,许嵩,时尚,香港藝人,炎亚纶,双子
微博奇葩,广东生活,搞笑幽默,新闻趣事,视频音乐
穿衣美容
搞笑幽默
创意,科技,美剧,美剧控,游戏动漫,旅游,微博奇葩,搞笑幽默
视频音乐
偏执狂,IT 互联网,车世界,奋斗,90 後,爱自由
视频音乐,名人明星
微博奇葩
穿衣美容
双鱼座,梁咏琪,旅行,魔域,偶稀饭睡觉觉,80 后,手机微博,旅游,爱自由
穿衣美容,美食,萌宠,名人明星,喜欢音乐,快乐大本营,爱睡觉,活泼,懒人,开心
狮子座,大学,重口味,好性格,哈哈,强迫症,心理学,微博奇葩,旅游,穿衣美容
美食,旅游,名人明星,穿衣美容
名人明星,搞笑幽默,汕尾生活,美女,穿衣美容
美食,萌宠,旅游,微博奇葩,搞笑幽默
视频音乐
旅游,视频音乐,新闻趣事,微博奇葩,搞笑幽默
旅游,穿衣美容,搞笑幽默
视频音乐
微博奇葩
视频音乐
旅游,萌宠,视频音乐,名人明星,搞笑幽默
美食,湖南生活
做梦,穿衣美容,旅游
穿衣美容
名人明星
芜湖生活,微博奇葩,陈奕迅,新闻趣事,穿衣美容,搞笑幽默
美女
美容服饰,星座运势,八卦杂谈,新闻趣事,视频音乐,穿衣美容,星座命理,搞笑幽默
旅游,视频音乐
旅游,美食,IT 数码,新闻趣事,搞笑幽默
美女
视频音乐
会计,爱自由,重庆,白羊,搞笑幽默
美女
微博奇葩,名人明星
韩国生活
新闻趣事,穿衣美容,搞笑幽默,旅游,名人明星

南太铉
怀旧,运动,服装,网络,钓鱼,品牌,平面设计,85 后,穿衣美容,浙江生活
美容服饰,穿衣美容
旅游
星座命理
机智如我
搞笑幽默
福建生活
SKE48,松井珠理奈,松井玲奈,松井 JR,W 松井
北京生活
穿衣美容,旅游,美食,萌宠,视频音乐
视频音乐
旅游
新鲜水果,货到付款,送货上门,株洲购物,株洲水果,水果,美食
美女
新闻趣事,星座命理,搞笑幽默,视频音乐,穿衣美容
沧州生活,穿衣美容,新闻趣事,搞笑幽默,旅游
视频音乐
台湾,台北,民宿,旅游
新闻趣事
美女
萌宠
旅游,穿衣美容
旅游,名人明星
北京生活
美女
美女
文字,执着,爱睡觉
新闻趣事,名人明星,星座命理,陕西生活,搞笑幽默
旅游,美食
新闻趣事,微博奇葩,美食,旅游,搞笑幽默
旅游
旅游,名人明星
疯狂,搞笑幽默
旅游
欧美控
视频音乐,搞笑幽默,微博奇葩
星座命理
视频音乐,美食,旅游
微博奇葩
体育资讯,视频音乐,新闻趣事,搞笑幽默,旅游
穿衣美容,新闻趣事,微博奇葩,星座命理,西藏生活
美食
搞笑幽默

共青团,寿宁县,斜滩镇,共青团斜滩镇委,团委
穿衣美容,浙江生活
搞笑幽默
旅游
BV,Gucci,MCM,普拉达,塞琳,HERMES,CHANEL,LV,奢侈品牌控,奢侈品
新闻趣事,浙江生活
美食,旅游,视频音乐
旅游,搞笑幽默,名人明星,美女,视频音乐
美女,星座,游戏玩家,文学,历史爱好者,军事政治,萌宠,搞笑幽默,旅游,美食
跳舞,温柔,爱玩,爱自由,美女,旅游,视频音乐,新闻趣事,合肥生活,安徽生活
美食
旅游,美食,搞笑幽默,穿衣美容,星座命理
浙江生活
搞笑幽默,视频音乐,体育资讯,名人明星
美食,旅游,萌宠
新闻趣事
美食,广西生活
穿衣美容
美食
名人明星
名人明星,美女,美食,视频音乐,穿衣美容
微博奇葩,名人明星,美女,穿衣美容
旅游,新闻趣事,星座命理,云南生活,搞笑幽默
萌宠,美女,星座命理,微博奇葩,新闻趣事
足球达人,美容养生,化妆品专卖,精油美容,旅游达人,美食达人,美食,浙江生活,旅游,穿衣美容
穿衣美容
音乐发烧友,自然,杂志,萌宠
名人明星
微博奇葩
微博奇葩,名人明星,美食,穿衣美容,星座命理
视频音乐
福建生活,新闻趣事
交朋友,吉他,品牌
美女,穿衣美容,新闻趣事,名人明星,旅游
广州生活,旅游,视频音乐,搞笑幽默,新闻趣事,广东生活
微博奇葩,新闻趣事,旅游,萌宠,美食
新闻趣事
紫东名苑居委会,方松街道
穿衣美容,星座命理,搞笑幽默,微博奇葩,浙江生活
旅游,视频音乐,美食,名人明星,星座命理
美食,吃喝玩乐,吃货,绵阳,四川生活
香港 Lifestyle,穿衣美容
旅游,美食,穿衣美容

山西生活
优质内容,学习,阅读
美食
新闻趣事,视频音乐,美女,美食,湖北生活
穿衣美容
BIGBANG,法语,爱玩,穿衣美容,旅游
美食,旅游
新闻趣事,视频音乐,搞笑幽默,美食,名人明星
名人明星,美食,新闻趣事,旅游,浙江生活
搞笑幽默,坚强,自由,不认输,一缕阳光,乐观,夜的钢琴
微博奇葩
美食
微博奇葩
搞笑幽默
名人明星,旅游,美食,微博奇葩,陕西生活
职场招聘,武汉生活,视频音乐,湖北生活,新闻趣事,微博奇葩,搞笑幽默
名人明星,阳光
新闻趣事,微博奇葩,视频音乐,美女,穿衣美容
江苏生活
穿衣美容,美食,微博奇葩,搞笑幽默,北京生活
新闻趣事,微博奇葩
美食
穿衣美容
穿衣美容
腐女,爱幻想,魔羯座,旅游,美食
旅游,新闻趣事,浙江生活
新闻趣事,宁夏生活
视频音乐,新闻趣事,其他生活,旅游,美食
穿衣美容
视频音乐
犯二好姑娘,奋青,吃货
搞笑幽默,福建生活,穿衣美容,名人明星,美食
名人明星
旅游,美食,微博奇葩,遵义生活,搞笑幽默
视频音乐
海外生活
微博奇葩
美国生活,名人明星,旅游,穿衣美容,美女
美女,穿衣美容,微博奇葩,新闻趣事,搞笑幽默
旅游,水瓶座,巨蟹座
美食
穿衣美容,搞笑幽默,旅游,美食,星座命理
旅游
美食

重庆生活,微博奇葩,视频音乐,美女,搞笑幽默
美食,旅游,穿衣美容,美女,广东生活,新闻趣事
美图摄影
旅游,美女,星座命理,搞笑幽默,穿衣美容
旅游
美女
美容护肤,瘦身
情感生活
名人明星
搞笑幽默
名人明星
搞笑幽默,新闻趣事,名人明星,宠物图集,情感生活
外贸美衣,精致首饰,精品名表,太阳镜,奢侈品,美女,时尚
育儿百科,看电影,护肤美容,玫瑰,向日葵
旅游
校园生活
上海生活
搞笑幽默
名人明星
搞笑幽默
新闻趣事
本溪生活
美食,新闻趣事,旅游
视频音乐,微博奇葩,海南生活
视频音乐
游戏，吃货，活跃
公务员,天秤座,情绪化,Android,星座
搞笑幽默,微博奇葩
其实都是伪文艺,Britpop,土摇爱好者,摄影,摇滚,音乐爱好者
新闻趣事
美食,旅游
旅游,视频音乐,微博奇葩,新闻趣事
强迫症
会计,职业资格,考前培训,教育教学,教育培训
美食
新闻趣事,搞笑幽默,星座命理,美女
搞笑幽默,美女,穿衣美容
陕西生活
旅游,视频音乐,古风，荼靡
福建生活
新闻趣事
穿衣美容,旅游,萌宠,新闻趣事,搞笑幽默
旅游
辽宁生活,美食,萌宠,旅游,穿衣美容

旅游,美食,萌宠,微博奇葩
视频音乐,搞笑幽默,新闻趣事,宠物图集,读书分享
新闻趣事,美食,搞笑幽默,美女,河北生活
热爱生活
时尚潮流,真实力,书法,杂志,新闻,摄影爱好者,经济,文艺,学习,新闻趣事
学生,歌,环球,美食,穿衣美容,彩妆
浙江生活
矛盾体,摄影控,猫咪控
旅游,视频音乐,微博奇葩,萌宠,搞笑幽默
视频音乐
搞笑幽默
视频音乐
旅游,Kris,吴亦凡,粤语
湖北生活
宁波生活,美食
美食
美食
名人明星
旅游
美食,穿衣美容,新闻趣事,微博奇葩
新闻趣事
旅游,美食,微博奇葩,新闻趣事,搞笑幽默
搞笑幽默,旅游,新闻趣事,萌宠,福建生活
旅游,穿衣美容,新闻趣事,搞笑幽默,海南生活
微博奇葩,美女,穿衣美容,新闻趣事,浙江生活
浙江生活,新闻趣事,视频音乐,美食,旅游
微博奇葩,新闻趣事,美食,旅游
穿衣美容
美食
旅游,名人明星,穿衣美容,星座命理
搞笑幽默,美女
新闻趣事
搞笑幽默
广东求职招聘,旅游美食,呼叫中心外包,运动,生活感悟,社会,职业规划,深圳招聘,招聘信息
公益活动,暑期社会实践,三下乡,中南民族大学,环保
名人明星,河北生活,新闻趣事
音乐发烧友,快乐大本营,it 互联网,喜欢听歌,睡觉
代购,化妆品,黄金,数码产品,购物
广东生活,新闻趣事,萌宠
新闻趣事,八卦杂谈,校园生活,搞笑幽默,其他生活
美图摄影,笨傻,小洁癖,90 后,爱阳阳
宠物图集,萌宠
视频音乐,搞笑幽默
旅游

星座运势
八卦杂谈
大学等着我
名人明星,美食
搞笑幽默
美食
旅游,美容服饰,宠物图集
美食,名人明星,旅游,穿衣美容
视频音乐
芜湖生活
美女
宁波生活,萌宠
搞笑幽默
情感生活,星座运势,美图摄影,搞笑幽默
视频音乐,星座命理
搞笑幽默,读书分享,美图摄影,武汉生活
新闻趣事,校园生活,旅游,视频音乐,广州生活
旅游
微博奇葩,旅游,美食,厦门生活,名人明星
旅游
新闻趣事,萌宠,名人明星,美女,搞笑幽默
搞笑幽默
微博奇葩,新闻趣事,搞笑幽默,江苏生活,视频音乐
旅遊,矛盾,新闻趣事
新闻趣事
上网,iphone,减肥,美剧迷,美食,粤语,水瓶座,单身,爱笑,偏执狂
视频音乐
名人明星
校园生活
IT 数码,搞笑幽默,微博奇葩,青岛生活,盗墓笔记,简单,读书,好性格,美剧,天平座
微博奇葩,穿衣美容,旅游,萌宠
名人明星
新闻趣事,美女,穿衣美容,萌宠,美食
美食
学生,搞笑幽默
情感生活
星座命理,山东生活,视频音乐,穿衣美容,搞笑幽默
新闻趣事,搞笑幽默,穿衣美容,星座命理,旅游
视频音乐
名人明星
视频音乐,新闻趣事,旅游
微博奇葩,视频音乐,新闻趣事,搞笑幽默,湖北生活
美食
搞笑幽默

佛山生活,随性,文字控,狗狗,笑,孩子气,吃货,小女人,淘宝
名人明星
名人明星
水瓶女,时尚达人,四川生活,穿衣美容
视频音乐
美女,微博奇葩,名人明星,搞笑幽默,眉山生活
玩具控,早教,重庆生活
星座命理,旅游,美食,萌宠,陕西生活
奢侈品,美膚心得,香港代購,美國代購,母嬰用品,護膚,美妝,旅游
旅游
视频音乐,微博奇葩
LV,PRADA,顶级品质,专柜品质,奢侈品狂热者,旅游,穿衣美容
漫画,育儿经验,设计,毛里求斯,萌宠
旅游,新闻趣事,美女,北京生活,搞笑幽默
旅游,辽宁生活
购物,留学,吃喝玩乐,潮人,穿衣美容
新闻趣事,广东生活,美食,旅游,搞笑幽默
穿衣美容,深圳生活,香港,剁手,护肤,代购
新闻趣事,美食,旅游,美女,山东生活
美女
新闻趣事
网络,英语,时尚达人,热爱生活,潮人,生活,平常心,湖北生活
旅游,重庆生活,美食,搞笑幽默,穿衣美容
旅游,美食
新闻趣事,星座命理,穿衣美容,名人明星,美食
微博奇葩
穿衣美容,萌宠,美食,旅游,星座命理
旅游,穿衣美容
福建生活
穿衣美容
名人明星,新闻趣事,美食,旅游
搞笑幽默,河南生活,美女
名人明星
穿衣美容
美女
微博奇葩,搞笑幽默
广东生活
穿衣美容
新闻趣事,微博奇葩,美食,旅游,穿衣美容
名人明星
旅游
美女
上海生活
搞笑幽默

美食
美食, 穿衣美容, 搞笑幽默
新闻趣事
旅游
旅游
美女, 视频音乐, 搞笑幽默, 其他生活
名人明星
美图摄影
名人明星
穿衣美容
美食, 大连生活, 读书分享
音乐, 舞蹈
美女, 运动
搞笑幽默
旅游
新闻趣事, 搞笑幽默, 美女, 名人明星
广州生活
视频音乐
旅游
名人明星
美食, 美女, 名人明星, 穿衣美容
微博奇葩
微博奇葩, 美食, 穿衣美容
穿衣美容
名人明星
搞笑幽默
搞笑幽默, 穿衣美容, 名人明星, 台湾生活, 旅游
萌宠
星座运势
视频音乐, 微博奇葩, 搞笑幽默
新闻趣事, 名人明星, 美女, 穿衣美容, 广州生活
动漫
微博奇葩
穿衣美容
情感生活, 美容服饰
腐, 动漫, 名人明星, 美食, 旅游, 视频音乐
河源生活
视频音乐, 旅游, 美食, 新闻趣事, 搞笑幽默
美女, 旅游, 搞笑幽默
旅游
微博奇葩
视频音乐, 名人明星, 爱唱歌, 武艺控, 午饭党
搞笑幽默, 萌宠
美容服饰

新闻趣事
感性,努力,创业,平常心,生活,狮子座,旅游,视频音乐,美食,贵州生活
微博奇葩,美女,美食
美女,名人明星,新闻趣事
星座命理,美女,美食,穿衣美容,搞笑幽默
成都生活
名人明星,搞笑幽默,星座命理,新闻趣事,视频音乐
搞笑幽默,新闻趣事
音乐达人
微博奇葩
名人明星,美女,深圳生活,搞笑幽默
美女
微博奇葩
南京生活,美食,穿衣美容
新闻趣事
宠物图集,射手座,笑话,完美主义,电影控,旅游,星座命理,穿衣美容,湖南生活,萌宠
连接服务器失败,学习,唱K,美食,健康,语录,穿衣美容,视频音乐,搞笑幽默,湖北生活
旅游,萌宠
新闻趣事,搞笑幽默,星座命理,微博奇葩,澳大利亚生活
喜欢旅游,music,球迷
穿衣美容
美食,旅游,微博奇葩,视频音乐,新闻趣事
新闻趣事,视频音乐,旅游,美食,萌宠
星座命理,微博奇葩,搞笑幽默,旅游,美食
美容服饰,时尚潮流,吃喝玩乐,服饰,潮人,创意,服装,外贸,穿衣美容
视频音乐
新闻趣事
星座命理,美食,视频音乐,旅游,名人明星
萌宠,美食,旅游
星座命理,视频音乐,穿衣美容,旅游,名人明星
视频音乐,微博奇葩,搞笑幽默
美食,旅游,视频音乐
湖北生活,美食,穿衣美容
极品,搞笑幽默
旅游,名人明星,新闻趣事,微博奇葩,搞笑幽默
美食,视频音乐
旅游,萌宠,视频音乐
美女
搞笑幽默,广东生活,旅游,微博奇葩,新闻趣事
蔡依林,Jolin,略屌嘻嘻,Swimming,Christina,Aguilera,Xtina,美食
摩羯,星座命理,搞笑幽默,视频音乐,名人明星,新闻趣事
名人明星,新闻趣事,美食,微博奇葩,内蒙古生活
名人明星
穿衣美容

美女,美食,视频音乐,穿衣美容,搞笑幽默
萌宠
新闻趣事
美食,福建生活
名人明星
搞笑幽默
美食,郭敬明,睡觉睡到自然醒,看电影,爱音乐,小说,上网,睡觉,看书,旅游
肇庆生活,美图摄影
山西生活
VIP,GD,视频音乐,旅游
穿衣美容
微博奇葩,星座命理
搞笑幽默,星座命理,萌宠,微博奇葩,穿衣美容
旅游
温职专团学,团委学生会,温职专,新闻趣事
星座命理
穿衣美容
名人明星
广州大行,番禺大行,大行折叠车,折叠车爱好者,视频音乐,美女,旅游,微博奇葩,广州生活,
广东生活
美女
搞笑幽默
新加坡生活
旅游,名人明星,穿衣美容
视频音乐,搞笑幽默,新闻趣事
微博奇葩
美食
潮人,微博奇葩,穿衣美容,广东生活
电影爱好者,射手,看小说,学生一族,夜猫子,江苏生活
广东生活
美食,名人明星,福建生活,旅游,穿衣美容,新闻趣事
美食,旅游,名人明星,穿衣美容,美女
重度拖延症,收藏癖,资源控,本尊控,剧粉,歌迷,纯良民
云南生活
养生,美容,逛街,团购,购物狂,欧美控,网络营销
自我矛盾纠结体,我就是不说话,德玛西亚,苦逼高二党,渣画手渣手画
萌宠
河北生活
名人明星,微博奇葩,萌宠,美食,视频音乐
穿衣美容
美食,旅游,上海生活
穿衣美容
广东生活
星座命理

名人明星
上网,星座命理
旅游,星座命理,穿衣美容
萌宠,美食
穿衣美容
名人明星,旅游,美食,萌宠,搞笑幽默
上海生活
旅游
萌宠
美图摄影,发呆,爱音乐,听音乐,摇滚,天秤,喜欢唱歌,小木头,神经大条,视频音乐
灭菌牛奶,哈密瓜牛奶,松鹤牌年糕,香蕉牧场牛奶,蓝莓牧场牛奶,草莓牛奶,香蕉牛奶,延世牛奶
编辑,环球旅行梦,背包客,摄影师,小清新,美食
穿衣美容
美食,旅游,名人明星,美女,穿衣美容
搞笑幽默
微博奇葩,广东生活,视频音乐,萌宠,搞笑幽默
名人明星
河南生活
微博奇葩,新闻趣事,名人明星,旅游
喜欢唱歌,宅,学生,动漫,自由,懒人,喜欢音乐,爱唱歌,写作,视频音乐
星座命理,视频音乐
旅游
穿衣美容,萌宠,美女
旅游
襄阳生活,育儿百科,名人明星,美食,美容服饰
粉色控,茜茜控,天秤座,EXO,名人明星
美食
名人明星
长沙生活,湖南生活,新闻趣事,旅游,美女,搞笑幽默
陕西生活
旅游
微博奇葩
新闻趣事,美食,安徽生活
美食
新闻趣事,星座命理,穿衣美容,旅游
旅遊
音乐爱好者,美食爱好者,杨幂
萌宠,旅游
旅游
视频音乐,穿衣美容,星座命理
旅游,美食,穿衣美容,湖北生活
新闻趣事,美女
搞笑幽默,微博奇葩,名人明星,星座命理,视频音乐

美女
旅游,星座命理,视频音乐,新闻趣事,名人明星
萌宠,搞笑幽默,新闻趣事,视频音乐,微博奇葩
搞笑幽默
视频音乐
微博奇葩
吴世勋,勋鹿，灿白，EXO！,名人明星
萌宠
名人明星
美食,旅游,穿衣美容,美女,新闻趣事
萌宠
美女
星座运势,90後,学习ing,梦想ing,喜欢听歌,吃货,乐观,幽默
名人明星,星座命理,美食,萌宠,穿衣美容
美女
穿衣美容
视频音乐,穿衣美容,旅游,萌宠,美食
旅游
美食,美女,旅游,江苏生活
美女
萌宠
萌宠,新闻趣事,搞笑幽默,旅游,美食
新疆生活,美食
旅游
名人明星,视频音乐,美女,新闻趣事
杂志,美图摄影,视频音乐
视频音乐,微博奇葩,星座命理,搞笑幽默,穿衣美容
不良之年少轻狂,盗墓笔记,动漫
腐女
澳大利亚生活,穿衣美容,名人明星,旅游,美食
新闻趣事
EXO,乐观,美食控,学生,双子女,睡觉睡到自然醒,偶稀饭睡觉觉,韩国,欧美控
旅游,名人明星,微博奇葩,美女,搞笑幽默
文化,环保,偶喜欢玩,任性,旅游
烤瓷牙,补牙,拔牙,牙齿矫正,正畸专家,隐形正畸,牙齿正畸,口腔正畸,重庆生活,穿衣美容
英国生活,美食,旅游,穿衣美容
美食,星座命理,旅游,微博奇葩,新闻趣事
搞笑
潍坊生活
加拿大生活
美女
新闻趣事,旅游,搞笑幽默,星座命理
美食,旅游,穿衣美容,新闻趣事,辽宁生活
校园生活,情感生活,读书分享,美食

广东生活
搞笑幽默
新闻趣事,美食,星座命理,穿衣美容
名人明星
交友平台
活泼开朗,孩子气,名人明星,旅游,美食,视频音乐,浙江生活
河南生活,穿衣美容,搞笑幽默,旅游
美食
美食,视频音乐,美女,萌宠
北京生活
微博奇葩,新闻趣事,安徽生活
星座命理,名人明星,旅游,视频音乐,美女
旅游,新闻趣事,微博奇葩
情感,天枰座,自由,旅行,时尚,动漫,上网,睡觉,午饭,烹饪
房产,军事天地,财经资讯,视频音乐,太原生活,山西生活
微博奇葩,旅游,美食,北京生活
发呆,听音乐,爱音乐,美食,视频音乐,名人明星,搞笑幽默,微博奇葩,新闻趣事
穿衣美容
学生,吃货一枚,喜欢听歌,懒,fashion
新闻趣事,搞笑幽默,视频音乐,名人明星
新闻趣事,美国生活,搞笑幽默,美女
旅游,星座命理
美食,北京生活
腐女,懒人,宠物,动漫,学生,旅游,上网,休闲,穿衣美容,美食
江苏生活
各种宅腐,月光族,哈韩骨灰级,手机控,起床困难户,街舞,exo 行星饭
美食,旅游,星座命理
视频音乐
名人明星
旅游
情感生活,新闻趣事,校园生活,名人明星,星座命理,穿衣美容,搞笑幽默
视频音乐,穿衣美容
新闻趣事,微博奇葩,搞笑幽默,视频音乐
穿衣美容
名人明星,美食,穿衣美容,美女
新闻趣事,搞笑幽默,美食,名人明星
星座命理,美食,旅游
星座命理,微博奇葩,旅游,美食
搞笑幽默,体育资讯
搞笑幽默
遥控飞机,遥控车,视频音乐,萌宠,星座命理,微博奇葩,搞笑幽默
浙江生活
穿衣美容
双子

星座命理,美食,视频音乐,新闻趣事
石家庄生活,穿衣美容
旅游
名人明星,美女,星座命理
广东生活,穿衣美容
名人明星
双重性格,天蝎座,宅女,星座命理
美食
山西生活
新闻趣事,北京生活
穿衣美容,新闻趣事,星座命理,旅游,名人明星
名人明星,读书分享,情感生活,星座运势,星座命理,穿衣美容,视频音乐
名人明星
电影
新闻趣事
教育,睡觉,健康,培训,独立,重庆,热爱生活,哲学,爱情,爱自由
视频音乐
旅游
艺术照,写真,摄影,创意,江西生活
搞笑幽默,福建生活
美食,星座命理
搞笑幽默,小提琴,笑话,跳舞,喜欢音乐,music
美食,穿衣美容
名人明星,美食,星座命理,穿衣美容,广东生活
News,Entertainment,Video,Food,Travel
李琦
新闻趣事,美食,旅游
穿衣美容,美食,旅游,新闻趣事,南宁生活
浙江生活
穿衣美容,星座命理,新闻趣事,搞笑幽默,重庆生活
其他生活
情感生活,搞笑幽默,新闻趣事,旅游,美食,穿衣美容,星座命理
滑冰,跳舞,穿衣美容
美女
名人明星,旅游,搞笑幽默,新闻趣事,视频音乐
搞笑幽默
搞笑幽默
新闻趣事,美食,穿衣美容,微博奇葩
名人明星,穿衣美容,视频音乐,新闻趣事,北京生活
搞笑幽默,新闻趣事,穿衣美容,美食
萌宠,旅游,重庆生活
搞笑幽默
名人明星
穿衣美容

美女
艺术,設計,低调,汽车,运动,旅游
美剧控,美剧,英剧迷,欧美客,动漫,电影,蓝光高清,爱尔兰生活,旅游
名人明星,穿衣美容
穿衣美容
萌宠,微博奇葩,美女
旅游,安徽生活,视频音乐,新闻趣事
名人明星,旅游,美食,穿衣美容
音樂
穿衣美容
香港生活
室内设计,家居设计,创意设计
家居控,生活方式,家居装饰,情感生活
李宇春,个性,听音乐,纠结,淡定,双重性格,喜欢旅游,看电视
强迫症,射手座,音樂,发呆,90 後,爱睡觉,帅哥,文艺,看电影
宅女,小说,娱乐,唱歌,小说迷,白羊,腐女,星座,购物,穿衣美容
澳洲,美食,旅游,穿衣美容,澳大利亚生活
美食
名人明星,旅遊,美食,视频音乐,星座命理
名人明星
美女
爱睡觉,帅哥,逛街,开朗,视频音乐
新闻趣事
名人明星
其他生活,名人明星,微博奇葩,新闻趣事,搞笑幽默
白羊女,视频音乐,名人明星
设计,玩儿,好书,微博奇葩,美食
新闻趣事
旅游
互粉,IT 数码,运动,爱电影,品牌,美剧迷,学生一族,正太控,苹果
星座命理,名人明星
英语,体育,90 后非脑残,开朗,朋友,动画,真诚,中医,自行车,视频音乐
孩子气,美食,美女,旅游
美食,微博奇葩,搞笑幽默,穿衣美容
美女,新闻趣事,福建生活
美女
常州生活,美女,体育资讯,球迷,青春,互粉,单身,交朋友
名人明星
新闻趣事,萌宠,微博奇葩,视频音乐,搞笑幽默
萌宠
搞笑幽默,旅游,新闻趣事,四川生活
名人明星,微博奇葩,新闻趣事,穿衣美容,美女
新闻趣事
视觉设计,创意,微电影,视频音乐

视频音乐
星座命理,爱吃,学生一族,90,羽毛球,爱笑,懒,Music,唱k,微笑
书,双鱼,多愁善感,学生一族,孩子气,o型血,爱笑,学习,爱美食,幻想
微博奇葩
音乐,kazakh,吸血鬼控,街舞,旅游
美食
旅游,美容服饰,星座运势,情感生活,穿衣美容,萌宠,美食
美女,旅游,美食,星座命理,浙江生活
搞笑幽默
视频音乐,体育资讯,爱音乐,Music
视频音乐,美女,新闻趣事
搞笑幽默,安徽生活
旅游
搞笑幽默
星座命理,穿衣美容,搞笑幽默,微博奇葩
佛教
日本生活
美图摄影
搞笑幽默
视频音乐,名人明星
湖南生活
云南生活
萌宠,微博奇葩,搞笑幽默,美食,穿衣美容
视频音乐,新闻趣事,微博奇葩,旅游,美女
新闻趣事,美食,穿衣美容,视频音乐,星座命理
名人明星,时尚,音乐,90后
自由,摄影,艺术,绘画,旅游
科学探索,名人明星
安徽生活
吃货一坨
萌宠,美食,旅游,韩国生活
萌宠,旅游
北京生活
星座命理
美图摄影,佛山生活,视频音乐,广东生活
名人明星,愤青,电影控,魔羯座,喜欢听歌
好书,自由职业,爱玩,聊天,执着,简单,美食,穿衣美容,新闻趣事,旅游
美女
名人明星
新闻趣事,山东生活,旅游,穿衣美容,微博奇葩
吃货,微博控,时尚,80后,fashion,美食
穿衣美容,微博奇葩,搞笑幽默,视频音乐
新闻趣事,美女
微博奇葩,上海生活,美食

视频音乐
旅游,浙江生活
搞笑幽默
云南生活
萌宠,旅游,美食,美女,穿衣美容
视频音乐,江苏生活,美食,名人明星,旅游
穿衣美容,旅游,名人明星
萌宠,美食,旅游,星座命理,福建生活
穿衣美容
搞笑幽默,旅游
美食
穿衣美容,美食
天津生活
搞笑幽默
名人明星,幽默,唱歌,宅女,娱乐,音乐控,笑话,电视剧,视频音乐
广州生活,穿衣美容,美食,萌宠,微博奇葩
旅游
吃喝玩乐,学生一枚
视频音乐,新闻趣事,萌宠,微博奇葩,搞笑幽默
IT 数码,动漫
名人明星
美食
杭州,旅游摄影,时尚潮流,音乐发烧友,星星,80 後,巨蟹座,苹果,潮
搞笑幽默
广播剧,视频音乐
名人明星
微博奇葩,河南生活
视频音乐
财经资讯,涨停板,证卷,金融,财经,私募,股票
江苏,工作,旅游,微博奇葩,美女,新闻趣事,搞笑幽默
旅游
视频音乐
爱自由,睡觉,学生,宅女,上网,小说,90 後,听音乐,交友,天秤座
穿衣美容
美食
设计,向日葵,美剧控,旅游美食,偏执狂,古典艺术,喜欢音乐,奋斗,粉红控,青春
旅游
微博奇葩
旅游
搞笑幽默,旅游
美食,澳大利亚生活
旅游,视频音乐
名人明星
名人明星

穿衣美容
名人明星
美女
名人明星
穿衣美容
交朋友, 愤青, 真诚, 听音乐, 购物, 健康, 旅游, 美食, 新闻趣事, 美女
美食
视频音乐
美图摄影, 萌宠
美食, 微博奇葩, 新闻趣事, 搞笑幽默
旅游
旅游, 美食, 萌宠, 星座命理, 穿衣美容
视频音乐, 十堰生活, 情感生活, 美食, 名人明星
旅游, 美女, 视频音乐, 美食, 搞笑幽默
名人明星, 美食, 星座命理, 穿衣美容, 旅游
美食, 育儿百科, 聊城生活, 山东生活, 新闻趣事
大师, 生肖, 命理, 风水, 周易
星座运势, 搞笑幽默, 视频音乐, 星座命理, 名人明星
广东生活, 旅游, 美食
80 后, 休闲, 新闻资讯, 美女
宁德生活, 福建生活
八卦杂谈
穿衣美容
搞笑幽默, 美女, 视频音乐, 微博奇葩, 新闻趣事, 青春
美女
搞笑幽默, 星座命理, 穿衣美容
萌宠, 旅游, 美食, 穿衣美容, 新疆生活
福建生活, 新闻趣事, 旅游, 穿衣美容, 视频音乐
美女
香港生活
美食, 旅游, 新闻趣事, 穿衣美容, 星座命理
搞笑幽默
美女
冷笑话, 处女座, 瘦身, 搞怪, 日语, 微博奇葩, 视频音乐, 穿衣美容, 旅游, 美食
视频音乐
罗志祥, 王菲, 旅游, 名人明星
旅游, 玉溪生活, 视频音乐, 名人明星
旅游, 小说, 欧阳少恭, 高三党, 古剑奇谭, 手绘, 画画, 古风
搞笑幽默
视频音乐
美食, 美女, 微博奇葩, 新闻趣事, 旅游
名人明星, 萌宠, 穿衣美容, 微博奇葩
浙江生活, 旅游
穿衣美容

胡思乱想,睡觉睡到自然醒,旅游
星座命理,视频音乐,穿衣美容
旅游,美食,新闻趣事,搞笑幽默,宁波生活
口腔医疗
美食
健康保健,保健品,海外代购
广东生活
原版,1 比 1,复刻,高仿奢侈品,普拉达,GUCCI,香奈儿,奢侈品,爱马仕,LV
時尚潮流,美体瘦身,汝阳,美发造型师,美发造型,美容护肤
美食,星座命理,名人明星,萌宠,穿衣美容
山东生活
新闻趣事,名人明星,搞笑幽默,星座命理,穿衣美容
美女
双鱼,爱唱歌,学生,看书,小说,动漫
旅游
美女
微博奇葩,新闻趣事,上海生活,名人明星,旅游
奢侈品,包包,帅哥,教师,旅游,美女,上海生活,星座命理,穿衣美容
名人明星
穿衣美容
萌宠
旅游
广东生活,新闻趣事,搞笑幽默,视频音乐
其他生活
旅游,新闻趣事
StreetStyle,Design,StreetClothing,Fashion, 角头,HipHop,AirJordan,Underground,XTER,0319
微博奇葩,美女,视频音乐,新闻趣事,搞笑幽默
汽车,电影迷,玉米,电影,交朋友,90 后非脑残,美食,美女
新闻趣事
视频音乐
武汉生活,原文,穿衣美容,旅游,美食,微博奇葩,湖北生活
家居,名人明星,旅游,星座命理,穿衣美容,美女
音樂,娛樂,品位生活,穿衣美容
旅游
旅游
微博奇葩,美食
萌宠
微笑,医生,宅女,搞笑,美食,台湾生活,穿衣美容
新闻趣事
读书分享,情感生活,名人明星,星座运势,萌宠,微博奇葩,星座命理,新闻趣事
广东生活,旅游,穿衣美容,美食,萌宠
答疑解惑,交流经验,运行道苦中作乐,江苏生活
萌宠

旅游,美食
美图摄影,旅游摄影,K 歌,美食
穿衣美容
穿衣美容
视频音乐,旅游,美食,新闻趣事,云南生活
美食,旅游,穿衣美容,萌宠
穿衣美容
视频音乐
浙江生活
视频音乐,萌宠
辽宁生活
旅遊
美食,穿衣美容
美食,新闻趣事,旅游,上海生活,上海,生活,房产
单身,吃货一枚,美食爱好者,看小说,旅游
唯美,婚纱控
厦门生活,美女,穿衣美容,新闻趣事,搞笑幽默
旅游爱好者,学生一族,打球,动画,涂鸦
英雄联盟,LOL,淘宝,游戏动漫,网游,IT 數碼,旅游
美食
佛山生活
瘦身,时尚,潮流,服饰,女装,欧美,旅游,美女,穿衣美容
临沂生活
新闻趣事
视频音乐
穿衣美容
搞笑幽默
多愁善感,90 后非脑残,钢琴,网络营销,独立,新闻趣事
美容服饰,美女
厦门生活
旅游
视频音乐,F1
美剧,阅读,偶喜欢玩,爱情,创业,微博奇葩,美食,旅游,新闻趣事,海口生活
青岛 12349,青岛残联,脑瘫,关注弱势群体,助残学习,智力障碍,残疾人托管,助残康复
宠物图集,美食
职场招聘,游戏,名人明星,美女,旅游
萌宠
韩装,官网,东大门,实体店,代购,加拿大,穿衣美容
八卦杂谈,情感生活,媒体,Music,瑜伽,汽车,音乐,穿衣美容,微博奇葩,萌宠
美女,萌宠,成都生活,搞笑幽默,微博奇葩
IT 数码,美女,八卦杂谈,旅游,美食,视频音乐,萌宠
名人明星,美食,微博奇葩,济南生活,视频音乐
搞笑幽默,视频音乐,旅游,台湾生活
泉州生活,小疯子,白羊座,吃货,技术宅,手机控

美女
新闻趣事,搞笑幽默,读书分享,美容护肤,旅游,美食,萌宠,穿衣美容
萌宠,名人明星,美食,旅游,美女
名人明星
读书分享,旅游
射手座,活泼开朗,苏打绿,无厘头,音乐迷,爱唱歌,微博,偶喜欢玩,平常心,搞笑幽默
美图摄影,穿衣美容
情感生活,新闻趣事,穿衣美容,美食,萌宠
旅游,穿衣美容,汕头生活
南昌生活,美食,旅游,穿衣美容
苏州生活
穿衣美容,星座命理,美女
微博奇葩,新闻趣事,搞笑幽默
江门生活,新闻趣事,穿衣美容
旅游,美食,其他生活,微博奇葩
IT 数码,海外生活,微博奇葩,萌宠
篮球,唱歌,游戏
旅行,吃货,宅女,法国,微笑,音乐控,奋斗
旅游
星座命理
名人明星,搞笑幽默,新闻趣事,微博奇葩,丽水生活
八卦杂谈
美食,星座命理,旅游,赣州生活
搞笑幽默,美女
生活用品,地摊,福州生活,穿衣美容,美食
美图摄影,美食,美容服饰,视频音乐,搞笑幽默
微博奇葩
名人明星
美食,星座命理,微博奇葩,汕头生活,搞笑幽默
旅游
名人明星
名人明星,美食,视频音乐,新闻趣事,微博奇葩
摄影,钢铁侠,黄子华,曼联,广州,体育
法语培训,法语学习视频,长沙法语,外教在线,法语学习,魁北克移民,法国留学,法国生活
旅游,新闻趣事,微博奇葩
视频音乐,美食,旅游
美女,萌宠,旅游
夜猫子,瓶邪,火影忍者,90 后但我不脑残,广播剧,游戏动漫,cosplay,盗墓笔记,金在中,动漫
旅游
旅游
名人明星
美女
校园生活,搞笑幽默,微博奇葩,美食,星座命理

旅游
搞笑幽默,视频音乐,新闻趣事,深圳生活,美女
搞笑幽默,美女
天秤座,运动,幽默,听歌,90后,潮流,美食
读书分享,新闻趣事
视频音乐,愤青，90后，闷骚
搞笑幽默,美食,上海生活
美女,萌宠,美食,穿衣美容,微博奇葩
usdm,jdm,audi,gtr,subaru,rare,lowlife,cars,stance,hellaflush
穿衣美容,美食
穿衣美容
搞笑幽默,新闻趣事,穿衣美容
美食,微博奇葩,旅游,穿衣美容,星座命理
美女
学生一族,百度徐良吧,徐良
贵港生活
搞笑幽默,萌宠,美食,旅游,名人明星
搞笑幽默
名人明星
新闻趣事,视频音乐,名人明星,搞笑幽默,微博奇葩
小说,学生,巨蟹座,漫画,搞笑幽默
粉丝,来吧,钢琴,微博奇葩,视频音乐,星座命理,旅游,萌宠
美食,视频音乐
校园生活,微博奇葩,利威尔兵长,海贼王
搞笑幽默,连云港生活,穿衣美容,名人明星,旅游,美食,星座命理
穿衣美容,名人明星,爱自由,化妆,随性,游泳,睡觉睡到自然醒,羽毛球,摄影爱好者
新闻趣事
新闻趣事
吃喝玩乐,猫,布艺DIY,手工DIY,石头,水晶,饰品,美食,旅游
嘉兴生活,浙江生活
新闻趣事
动漫,星座运势,喜欢音乐,书,看电视,喜欢旅游,旅游,美食,微博奇葩,星座命理
微博奇葩
搞笑幽默,视频音乐,美食,萌宠,名人明星
微博奇葩,EXO,柯南,表演,海绵宝宝,搞笑幽默,名人明星,萌宠,视频音乐
搞笑幽默,穿衣美容,旅游,美食,微博奇葩
旅游
视频音乐
美食,旅游,搞笑幽默,视频音乐,穿衣美容
湖北生活
旅游,新闻趣事
军事天地,微博奇葩,音乐
搞笑幽默,新闻趣事,名人明星,穿衣美容
视频音乐,萌宠,星座命理,搞笑幽默,旅游

穿衣美容,视频音乐,美食
偶稀饭睡觉觉,水瓶座
名人明星
八卦杂谈,柠檬,颖火虫,香橙
古川雄辉,旅游
名人明星
美食,旅游
旅遊,穿衣美容,微博奇葩,萌宠,美食
微博奇葩,旅游,美食,穿衣美容,美女
旅游,萌宠
穿衣美容
可爱,爱自由,无厘头,活泼,萌宠,美食,旅游,穿衣美容,新闻趣事
日音,Attitude,GAY,设计,芯片,工艺,微电子,AKB48,Oricon
名人明星
文字,音乐爱好者,摄影,上网,化妆,室内设计,美术,自拍,fashion,穿衣美容
旅游
IT 数码,美图摄影
新闻趣事,萌宠,名人明星,搞笑幽默,微博奇葩
福建生活,美食,旅游,新闻趣事
读书,美剧,制片人,编剧导演,影视制作,新闻趣事,旅游,萌宠,美食
陕西生活,新闻趣事,搞笑幽默
新闻趣事,星座运势,金华生活,搞笑幽默
萌宠
星座命理,新闻趣事,搞笑幽默,名人明星
穿衣美容
新闻趣事,美食,微博奇葩,广东生活
美食,旅游,萌宠,视频音乐,美女
美女
金融 315,消费维权,金融消费者,消费者研究,金融业
翡翠控,翡翠商,翡翠
美女,河南生活
搞笑幽默
搞笑幽默,浙江生活
名人明星,微博奇葩,星座命理,搞笑幽默,新闻趣事
美食
美女
微博奇葩,视频音乐,穿衣美容
新闻趣事,山东生活
搞笑幽默
微博奇葩,视频音乐,搞笑幽默,美食
福建生活,旅游,美食
旅游
金牛座,旅行,思考,强迫症,发呆
旅游

江西生活, 搞笑幽默
视频音乐
名人明星
穿衣美容, 微博奇葩, 美食
双子女, 高三, 旅游
名人明星
名人明星, 旅游, 新闻趣事, 美食, 江门生活, 广东生活
穿衣美容, 视频音乐, 湖北生活, 美食, 搞笑幽默
美图摄影, 视频音乐, 搞笑幽默, 新闻趣事, 美食, 穿衣美容
观其舞, 知其德
微博奇葩, 视频音乐
名人明星
搞笑幽默
旅游, 美食, 新闻趣事, 搞笑幽默, 山东生活
名人明星
韩国明星, 边伯贤
美食, 上海生活
时尚, 新闻资讯, 音樂, 美食, 旅游
萌宠, 微博奇葩, 搞笑幽默, 四川生活
美食, 旅游, 穿衣美容
搞笑幽默
名人明星, 穿衣美容, 星座命理, 西藏生活, 旅游
旅游, 美食, 萌宠, 微博奇葩, 搞笑幽默
北京生活, 新闻趣事, 搞笑幽默
美女
穿衣美容
旅游, 穿衣美容
穿衣美容, 四川生活
校园生活, 旅游
旅游
校园生活, 名人明星
名人明星
八卦杂谈, 星座命理, 微博奇葩, 美女
苏志燮, 大叔控, 强迫症
旅游
名人明星
搞笑幽默, 美食, 旅游, 星座命理
搞笑幽默
上海生活
美女
美食, 新闻趣事, 微博奇葩, 搞笑幽默
旅游
视频音乐, 新闻趣事, 微博奇葩, 搞笑幽默, 广东生活
吃货, 电视剧, 睡懒觉, 散步, 爱美食

新闻趣事,旅游
广东生活
搞笑幽默
美女,穿衣美容
忧郁,无厘头,小说,狮子座,桌游,仙剑,学生一族,90后但我不脑残,游戏动漫,搞笑幽默
绅士般的执着,管杀不管埋,脑内剧场星人,英雄联盟,幻想大陆,FE,天蝎座,萌LX
穿衣美容
视频音乐,名人明星,穿衣美容,微博奇葩,搞笑幽默
名人明星
萌宠,旅游,美食
新闻趣事,视频音乐,名人明星,微博奇葩,搞笑幽默
名人明星,视频音乐
名人明星,美女,新闻趣事,浙江生活,搞笑幽默
重庆生活
视频音乐,新闻趣事,旅游,美食,上海生活
微博奇葩,搞笑幽默
星座命理,视频音乐,穿衣美容,旅游,广东生活
浙江生活,星座命理
健康养生,减肥,美容服饰,潮人,fashion,美食,旅游,美女,星座命理,穿衣美容
广东生活,美食,星座命理,视频音乐
新闻趣事
旅游
名人明星,视频音乐,美女,穿衣美容
搖滚,湖南卫视芒果台,直通春晚,天娱传媒,快乐男声,歌手明星,歌手
视频音乐,美食,微博奇葩
DOTA,篮球爱好者,开朗,视频音乐,微博奇葩,美食,名人明星,搞笑幽默
视频音乐,名人明星,旅游,美食
星座命理,搞笑幽默
新闻趣事
视频音乐,科学探索,花样美男,动漫,小说爱好者,推理迷,名人明星,星座命理,搞笑幽默
浙江生活,美食,穿衣美容,搞笑幽默
名人明星
陈奕迅,周杰伦,帅哥爱好者,时尚潮流,时尚女包,包包控,美食,穿衣美容,视频音乐,搞笑幽默
体育资讯
微博奇葩
江苏生活
活泼开朗,街舞,猫,淘宝,衣衣,美食,美女,旅游,穿衣美容,广东生活
安徽生活,穿衣美容,新闻趣事,搞笑幽默,微博奇葩
美食,名人明星,星座命理,新闻趣事,广东生活
美食,星座命理,福建生活,搞笑幽默,视频音乐
校园生活,搞笑幽默,星座命理
自由,時尚,梦想,旅游美食,热爱生活
名人明星,新闻趣事,微博奇葩,美食,穿衣美容

微博奇葩,穿衣美容,萌宠,美食
星座运势,美图摄影,音樂,双子女,学生一枚,视频音乐,微博奇葩,萌宠
萌宠
视频音乐,美食,搞笑幽默,穿衣美容,萌宠
校园生活,草根站长,家长,80 後
成都生活,微博奇葩
香港生活
穿衣美容
微博奇葩
旅游
美图摄影,美女
穿衣美容
旅游,新闻趣事,萌宠,视频音乐,美女
尼坤,Nichkhun,2PM
广东生活
视频音乐
视频音乐,美食
淘宝控,看书,交友,心理,矛盾体,休闲,忧郁,赚钱,新闻,宅男
校园生活,我就是我,舞蹈,爱音乐
美食
美图摄影,新闻趣事,穿衣美容,旅游,美食,视频音乐
牛绵汪,金俊勉永远是宝,不喜欢求别看,团饭,初心边伯贤灿白
陕西生活,搞笑幽默,新闻趣事,视频音乐
名人明星,旅游,视频音乐,四川生活,搞笑幽默
新闻趣事
视频音乐,美食,星座命理,微博奇葩,旅游
多肉植物控,多肉植物,萌宠,穿衣美容,美女,搞笑幽默
广东生活
摩羯座,看电影,爱睡觉,活泼,互粉,萌宠,微博奇葩
星座命理,穿衣美容
欧洲代购,sneaker,球鞋,鬼冢虎,ASICS,NewBalance,潮人,supreme,美国代购,潮牌
微博奇葩,新闻趣事,穿衣美容,名人明星,搞笑幽默
萌宠,新闻趣事,星座命理,美食,微博奇葩
星座命理,视频音乐,旅游,美食,微博奇葩
情感生活
旅游
穿衣美容
香港生活
旅游,星座命理,搞笑幽默,新闻趣事,江苏生活
创业,美食,摩羯座,音乐迷,新闻趣事,微博奇葩,搞笑幽默,美女,穿衣美容
美图摄影
花样美男,K 歌,天枰座,时尚,听歌
旅游
萌宠,微博奇葩,星座命理,搞笑幽默,美食

新闻趣事
名人明星,视频音乐
搞笑幽默
星座运势,EXO
其他生活
校园生活,星座命理,萌宠
校园生活
房产,深圳,0755,爱,梦想,美食,美女,深圳生活
旅游
动漫,偶尔人妻,抖M,呆萌,傲娇,腹黑
名人明星,视频音乐
穿衣美容,旅游,名人明星,保定生活,视频音乐
懒人,健身,武术,爱玩,爱吃,足球,篮球,运动,投資理財,美女
名人明星,搞笑幽默,南京生活
搞笑幽默,旅游,视频音乐
新闻趣事
校园生活,哈尔滨生活,搞笑幽默,微博奇葩,视频音乐,旅游
苏州生活
星座运势,情感生活,美女,星座命理,穿衣美容,连云港生活
旅游,美食,穿衣美容
搞笑幽默
视频音乐
非典型性男人,旅游,美女過敏
名人明星,美女,新闻趣事,视频音乐,咸宁生活
穿衣美容
心情
旅游,美图摄影,校园生活,新闻趣事,福州生活
名人明星
微博奇葩
微博店,韶大面交,穿衣美容
潮人,瑜伽,摄影控,杂志,画画,爱旅行,音乐
美女
旅游,微博奇葩,穿衣美容,星座命理
惠州生活,搞笑幽默
名人明星
旅游
瓦尔登湖,猪一样的队友,麻将团炮手,爱打望,处女座,Dota菜鸟,懒人,美食,旅游
移动互联网,汽車,乐天派,文字控,疯狂,臭美,旅游,新闻趣事,广州生活
新闻趣事
新闻趣事
名人明星
搞笑幽默,穿衣美容,美女,视频音乐
动漫,外貌协会,钢琴,耽美,宅,银魂,爱自由,瓶邪,双重性格
搞笑幽默,微博奇葩,穿衣美容,旅游,来宾生活

穿衣美容
新闻趣事, 名人明星, 汕头生活
名人明星
珠海生活
星座命理, 旅游, 穿衣美容, 台湾生活
投资, 喜欢听歌, 美食控, 赚钱, 旅游, 新闻趣事
穿衣美容, 微博奇葩, 名人明星
新闻趣事
啦啦欧美, 陈翔控
穿衣美容, 视频音乐, 名人明星, 新闻趣事, 美食
名人明星, 视频音乐
广东生活
美食, 新闻趣事, 美女, 广州生活, 旅游
搞笑幽默
购物, 吃货一枚, 美食
旅游, 新闻趣事, 美容服饰, 美女, 穿衣美容
搞笑幽默
视频音乐
旅游, 星座命理
名人明星, 广西生活
穿衣美容, 美食, 旅游, 山东生活, 搞笑幽默
名人明星
針灸, 傷寒
名人明星
美女
漫画
穿衣美容
穿衣美容
旅游
视频音乐, 搞笑幽默
旅游
互粉 V 粉团, 互粉互注, 互粉, 电缆, 电线, 中国, 福建, 厦门, 电线电缆, 福建生活
美食
萌宠
穿衣美容, 美食, 名人明星, 旅游, 江苏生活
新闻趣事, 结婚那点事儿, 爱情, 婚礼摄影, 婚礼策划师, 婚礼策划, 婚礼
美女
校园生活, 美食, 懒人, 淘宝控, 服饰, 欧美控, 美女
新闻趣事
新闻趣事
八卦杂谈, 名人明星, 微博奇葩, 新闻趣事, 搞笑幽默
星座命理, 喜欢旅游, 哲学, 搞笑幽默, 旅游
美女
平常心, 善良, 坚强, 男人, 搞笑幽默

美容,化妝師,化妆品控,穿衣美容
搞笑幽默
绍兴生活
夜猫子,爱幂脑残粉,吃货,A 型血,射手座,童星
美图摄影,新闻趣事,视频音乐,旅游,美女
美食
搞笑幽默
影视,打篮球,爱吃
搞笑幽默
东莞生活
美女
淮安生活
搞笑幽默
武汉生活
沈昌珉是唯一,最爱的是小珉珉
视频音乐
旅游
视频音乐
无厘头,学生,神经大条,小说迷,懒人
搜索淘宝店,topshow 代购,美容服饰,旅游,穿衣美容,美女
搞笑幽默,广州生活,穿衣美容,新闻趣事
美食,上海生活,搞笑幽默
新闻趣事,搞笑幽默
动漫,新闻趣事,视频音乐,微博奇葩,美食,搞笑幽默
奋斗,学习,开朗,乐观,独立,射手
搞笑幽默,美图摄影,星座运势,宠物图集,穿衣美容,萌宠,星座命理,视频音乐,白羊座,90 后
旅游,穿衣美容
美食
美女
台湾生活
搞笑幽默,美女
穿衣美容,加拿大生活
LOL,NBA,BigBang
新闻趣事,搞笑幽默,星座命理,微博奇葩,莆田生活
太原生活
美女,搞笑幽默
旅游,穿衣美容,美女,美食,温州生活
90 後,爱旅游,自恋,爱幻想
新闻趣事,八卦杂谈,自由,重口味,90,创意,无厘头,微博奇葩
星座命理,旅游,美食,搞笑幽默,视频音乐
旅游
太原生活
深圳生活
美食,视频音乐,星座运势,茂名生活,布丁,炎亚纶,shopping,水瓶座,喜欢音乐,名人明星

动漫,死宅,二次元
微博奇葩
自拍,臭美,服饰,爱旅游,爱电影,80後,B型血,潍坊生活
名人明星
穿衣美容,美食,旅游,新闻趣事,武汉生活
东莞生活
名人明星
美食
美食,微博奇葩,搞笑幽默
旅游
名人明星,视频音乐,新闻趣事,搞笑幽默,微博奇葩
星座命理,宁波生活,搞笑幽默,美女
视频音乐,名人明星,星座命理,美女
微博奇葩,美食,新闻趣事,温州生活
搞笑幽默
萌宠
美图摄影,美食,微博奇葩,新闻趣事,搞笑幽默,懒,女汉子,旅游
ulzzang,饰品,化妆品,美容,服饰,代购,韩国代购
医学,爱生活,养生,美容,爱美食,爱笑,美食,健康,医生
微博奇葩,旅游,美食,名人明星,穿衣美容
旅游,厦门生活,微博奇葩,搞笑幽默
美女,旅游,名人明星,穿衣美容,视频音乐
搞笑幽默
美食,旅游,穿衣美容,天水生活,萌宠
美食
搞笑幽默
重庆生活,萌宠,旅游,美女,新闻趣事
搞笑幽默
新闻趣事
其他生活
搞笑幽默
搞笑幽默
微博奇葩
设计师,小女人,工作,爱吃,画画,睡觉睡到自然醒,穿衣美容
搞笑幽默
搞笑幽默
搞笑幽默
微博奇葩
旅游
深圳生活
美女
名人明星,穿衣美容,搞笑幽默
美女
穿衣美容,星座命理,视频音乐

旅游
深圳生活
搞笑幽默,微博奇葩
搭配,时尚,淘宝,网购,女装,淘宝控,模特,美女,穿衣美容
名人明星
科学探索,美食,萌宠,星座命理,旅游,视频音乐
新闻趣事,搞笑幽默
美食,活泼开朗
淘宝控,懒人,美食,旅游,名人明星,视频音乐,穿衣美容
名人明星
搞笑幽默
名人明星
新闻趣事,美食
折扣,配饰,搭配,淘宝,网购,女装,shopping,美女,穿衣美容
搞笑幽默,新闻趣事,视频音乐,微博奇葩,名人明星
旅游,服装设计,服装品牌,服装皮衣,时尚皮衣
穿衣美容,旅游
美女
情感生活,搞笑幽默,星座运势,读书分享,美食,穿衣美容
西宁生活
美食,视频音乐,旅游
星座命理,搞笑幽默,名人明星,视频音乐,深圳生活
美食
演员梦
名人明星
新闻趣事,桂林生活,美食
职场招聘,石英砂,云母,蛭石,彩砂,矿,工厂,建筑装修,涂料,大理石
西宁生活
旅游,大连生活,美食
旅游,穿衣美容,视频音乐,新闻趣事,广州生活
微博奇葩
喜欢学习,搞笑幽默,微博奇葩
名人明星,广州生活,穿衣美容
视频音乐,美容服饰,美食,穿衣美容,微博奇葩,广州生活,搞笑幽默
旅游,新乡生活
想要学习外语,微笑,动漫控,美食,名人明星,星座命理
名人明星
搞笑幽默,COSPLAY,汕头生活
旅游,搞笑幽默,萌宠,美女,湖州生活
汕头生活
美食
广州生活,美女,美食,新闻趣事,搞笑幽默
随性,乐天派,车生活,来自车健康,萌宠,旅游,微博奇葩,新闻趣事,南充生活
配饰,搭配,网购,时尚,女装,购物,听音乐,淘宝,美女,穿衣美容

校园生活,美女
旅游,美食,搞笑幽默,天津生活
漳州生活
穿衣美容,美女,视频音乐,旅游,美食
萌宠,名人明星,微博奇葩,视频音乐
商业,产品经理,互联网
美食,旅游,萌宠
微博奇葩
搞笑幽默,旅游
萌宠
美食
星座命理,美食,新闻趣事,旅游,穿衣美容
视频音乐
穿衣美容
视频音乐,搞笑幽默
微博奇葩
换物,二手,受物,magi
伪文艺女青年,外星人,肤浅没文化,摄影,臭美,美食,旅游
茂名生活
摄影摄像,品牌策划,影视广告制作,视频音乐
萌宠,穿衣美容,视频音乐,搞笑幽默,微博奇葩
微博奇葩,新闻趣事,穿衣美容,美女,昆明生活
穿衣美容
搞笑幽默
新闻趣事
美食,旅游,杭州生活,新闻趣事
美女,旅游,搞笑幽默
郑州生活,新闻趣事
名人明星
搞笑幽默
视频音乐,旅游
微博奇葩,新闻趣事,美女,视频音乐,搞笑幽默
名人明星
视频音乐,低调,心理学,90 後,穿衣美容,旅游
视频音乐,搞笑幽默,旅游
音乐,购物,台球,动漫
萌宠
名人明星,Alin,徐佳瑩,天蝎座,B 型血,紫色,美剧,读书,音乐控,心理学
美女
旅游,穿衣美容
名人明星
美容服饰,新闻趣事,搞笑幽默,旅游
旅游
萌宠,厦门生活

美食,新闻趣事,穿衣美容,美女,旅游
微博奇葩,搞笑幽默,威海生活,新闻趣事
美女
常州生活,萌宠,美食,旅游,微博奇葩
夜猫子
美食,搞笑幽默,旅游,穿衣美容
泰国生活
体育资讯,新闻趣事,视频音乐,校园生活,微博奇葩,搞笑幽默
美食
广州生活
星座命理,旅游
搞笑幽默,新闻趣事,微博奇葩
懒,吃货一枚,偶稀饭睡觉觉,双子座,睡觉睡到自然醒,音乐爱好者,苏打绿,单身,EXO
微博奇葩,新闻趣事
新闻趣事,名人明星,旅游,美女,视频音乐
新闻趣事,搞笑幽默,旅游,美食,穿衣美容
美女
乒乓球,自由,学生,90 后非脑残,打篮球,体育
澳大利亚生活,美食
新闻趣事,传媒,媒体
温州生活
名人明星,旅游,穿衣美容,微博奇葩,视频音乐
旅游,新闻趣事,八卦杂谈,视频音乐,搞笑幽默
南京生活
临沂生活
美女,体育资讯,校园生活,郴州生活,搞笑幽默,星座命理
奋斗,美容,商业,教师,nba,湖人,个性,喜欢唱歌,水瓶,穿衣美容
新闻趣事
名人明星
科学探索,重口味,情感生活,体育资讯,美图摄影,穿衣美容,微博奇葩,美食
美食,沈阳生活,美女,穿衣美容
广州生活
视频音乐,旅游,美食,穿衣美容,深圳生活
情感生活,星座运势,新闻趣事,穿衣美容,搞笑幽默,名人明星,星座命理
视频音乐,名人明星
旅游
美图摄影,宠物图集,新闻趣事,微博奇葩,北京生活,旅游,萌宠
南宁生活,萌宠,美食
名人明星,穿衣美容,美女,视频音乐,新闻趣事,搞笑幽默
搞笑幽默,萌宠,美食,穿衣美容,美女
旅游,美食,美女,穿衣美容
职场招聘,游戏,IT 数码,星座命理,北京生活,新闻趣事
旅游
旅游爱好者,执着,化妆品,摄影师,乒乓球,爱唱歌,随性,美食,穿衣美容,厦门生活

时尚,汽车,体育,篮球,爱吃,爱玩,玩,美食,星座命理
新闻趣事,海南生活,旅游,美食,美女
搞笑幽默
扬州生活
看书,小资,敏感,喜欢旅游,喜欢音乐
读书分享,宠物图集,穿衣美容,视频音乐,重庆生活,萌宠,旅游
名人明星,榆林生活,星座命理,新闻趣事,穿衣美容
星座命理,新闻趣事,穿衣美容,武汉生活,名人明星
萌宠
北京生活
新闻趣事,笑话,情感,萌宠
星座命理,美食,视频音乐,新闻趣事,搞笑幽默
台州生活
以石会友,园林艺术,奇石收藏
IT 数码,旅游
搞笑幽默,腐女,安静,吃货一枚
电影控,义工志愿者,户外运动与旅行
2b 青年,神经病,爱吃,喜爱宠物,减肥减不下去,脸大
搞笑幽默
美女,美食,旅游,视频音乐
搞笑幽默
美食
视频音乐
美食
新闻趣事,美食,搞笑幽默,武汉生活
神经质,偶像剧,音乐迷,我爱阿狸,阿狸控
美容服饰,旅游
名人明星,旅游,穿衣美容,美食,重庆生活
萌宠,美食,新闻趣事,搞笑幽默,微博奇葩
美食,旅游,微博奇葩,新闻趣事,名人明星
穿衣美容,视频音乐,美食,微博奇葩,新闻趣事
体育,健康,喜欢音乐,美食
香港生活
美女
搞笑幽默
微博奇葩
厦门生活
星座命理,新闻趣事,搞笑幽默
星座命理,新闻趣事,黔南生活
百合控,普奥,攻控,全职冷 CP 党,咪子鱼,下克上,兰雁已弃疗,FATE
美食,微博奇葩,搞笑幽默,视频音乐,黄山生活
廊坊生活
搞笑幽默,美食,宠物图集,动漫,重口味,吃货一枚,北京生活,冷笑话
八卦杂谈,兰州生活,萌宠,美食

娱樂,媒体,广告,旅遊,传媒,新闻趣事,名人明星
摄影爱好者,旅游
微博奇葩
天津生活,穿衣美容,星座命理,萌宠,美食
宠物图集,励志语录,时尚女装,素年锦时の小铺
名人明星
新闻趣事,搞笑幽默,美食,旅游,穿衣美容
旅游
摄影爱好者,NBA,阅读,微笑,搞笑幽默
名人明星,美食,穿衣美容
追踪王,新大洲,上海生活
绘画,文具控
微博奇葩,星座命理,新闻趣事,美女,旅游
投资理财,视频音乐,搞笑幽默,穿衣美容,星座命理
NICHKHUN,GD,TOP,SEHUN,EXO,SNSD,BEAST,2PM,BIGBANG,韩流饭
美容服饰,旅游,美食,穿衣美容,视频音乐,新闻趣事
穿衣美容,Music,自信,时尚,汽车,名人明星
星座命理
美食,名人明星,微博奇葩,穿衣美容,旅游
视频音乐
南平生活,视频音乐
视频音乐
贪吃,爱旅行,萌,爱书
李五岁,李思懿
白举纲,2013 快乐男声,小小白,设计
星座命理
视频音乐
宗教文化,宗教信仰,安息日会,基督教会
搞笑幽默
红粉家族,王爱红吧,民营越剧,粉丝团队,越剧演员,喜欢越剧,越剧迷,越剧爱好者,台州生活
微笑,摇滚,情感,喜欢唱歌,真诚,视频音乐,美食,穿衣美容,旅游,星座命理
美图摄影,创意,绘画群
成都生活
宁波生活
美食
穿衣美容,中山生活,搞笑幽默
广告,网络营销,IT 互联网,移动互联网,微博奇葩
旅游
囧,交朋友,双鱼,IT 数码,85 后,单身,足球,唱歌,上网,美女
萌宠
星座命理,萌宠,日本生活,穿衣美容
美女
汕尾生活
旅游

名人明星,萌宠,新闻趣事,搞笑幽默,微博奇葩,手机看小说,欧美控,猫,笑话,冷笑话
历史,美容服饰,冷笑话,fashion,怀旧,校园,亲情,爱,哲学,猫咪控
视频音乐
吃货一枚,夜猫子,苏打绿,文藝,心理学控,爱幻想,双鱼,文青,美术
乐天派,微笑,孩子气,流浪,编辑,健康,文艺,星座命理,美食,萌宠
视频音乐,搞笑幽默
新闻趣事,狗狗控,90 后非脑残,书,情绪化,吳青峰,蘇打綠,天蠍,胡思乱想,山东生活
石家庄生活
旅游,美食,萌宠,安阳生活
美女
美国生活
荆门生活,美女,新闻趣事,视频音乐,搞笑幽默
懒,阳光,随性,发呆,爱笑,爱睡觉,90 後,爱自由,执着,学生一枚
明星,服装设计,帅哥,喜欢音乐,爱美食,美剧迷,汽車,偶像剧,摄影师,旅游
诗词曲赋,cos,动漫控,二次元生物,萌宠,微博奇葩,美食,搞笑幽默
重庆生活
微博奇葩,搞笑幽默
宠物图集,北京生活
美图摄影
影视,阿根廷,吸血鬼日记,时尚潮流,冷笑话,星座命理,新闻趣事,微博奇葩,视频音乐,搞笑幽默
微博奇葩,搞笑幽默,星座命理,穿衣美容
美食
星座运势,名人明星,美食,新闻趣事,视频音乐
视频音乐,美女
穿衣美容,美食
旅游
旅游,美食,杭州生活
名人明星,美食,新闻趣事,搞笑幽默,萌宠
新闻趣事,情感生活,美容服饰,旅游,微博奇葩,穿衣美容,星座命理,重庆生活
微博奇葩
泰州生活,美食
穿衣美容,名人明星,旅游,萌宠,视频音乐
美食
穿衣美容,视频音乐,名人明星,星座命理
IT 数码,旅游,美图摄影,视频音乐,新闻趣事
电影,关注时事政治,喜欢历史人物,爱好音乐,爱好名著,长不大的小白兔,旅游,萌宠
摩羯座,搞笑,心理,英语,萌宠,星座命理
旅游,美食,穿衣美容,新闻趣事,搞笑幽默
情感生活,旅游,微博奇葩
爱情,法国,随性,幻想,O 型血,打球,白羊座,视频音乐
铜陵生活,名人明星
其他生活
美食,萌宠,穿衣美容,新闻趣事,搞笑幽默

新闻趣事,搞笑幽默
旅游,穿衣美容,广州生活
性感,少女时代,K 歌,喜欢音乐,美女
美女
旅游,美女,新闻趣事
综艺,真人秀,观众,电视,美女,视频音乐,新闻趣事,搞笑幽默
美食,南昌生活
Columbia,哥伦比亚大学
旅游
移动互联网,录音,男神,美女控,广告,娱樂,听音乐,旅游,搞笑幽默,视频音乐
新闻趣事,搞笑幽默,穿衣美容,旅游,绵阳生活
搞笑幽默,视频音乐,美食
销售,IT 数码,电子商务,执着,热爱生活,完美主义,管理,经济,市场营销
南京纳斯达克,南京幼儿英语,南京少儿英语,新闻趣事
萌宠,搞笑幽默,微博奇葩,美食,名人明星
穿衣美容,旅游,名人明星
新闻趣事,微博奇葩,名人明星,视频音乐,北京生活
爱睡觉,发呆,爱自由,90 後,心情,朋友
星座命理
微博奇葩,搞笑幽默,美女
珠海生活
视频音乐,镇江生活
微博奇葩,视频音乐,新闻趣事,搞笑幽默,宁波生活
穿衣美容,视频音乐,新闻趣事,搞笑幽默,西宁生活
江门生活,美食
OA 协同办公,泛微协同办公,办公管理平台,OA 办公管理,办公平台,协同办公,泛微软件,泛微 OA,OA,IT
名人明星
东莞生活
排行榜,观众,电影,电视,美女,名人明星,视频音乐,新闻趣事,搞笑幽默
房产
动漫,猫,小清新,音樂,EXO,名人明星,旅游,美食,萌宠
微博奇葩
穿衣美容
读书分享,感性,街舞,旅游,视频音乐
体育资讯,搞笑幽默,游戏,视频音乐,IT 数码,哈尔滨生活
动漫,美食
美图摄影,旅游
宜宾生活
八卦杂谈,美图摄影,新闻趣事,校园生活,长沙生活
美食,新闻趣事,北海生活
旅游,广告,音乐,微电影,动画,短片,视频
农业,农产品,农村,农民,板栗,美食,生活,投资
星座命理

电影,真人秀,音乐秀,综艺,电视,iphone,视频音乐,名人明星,美女
PayPal,女裝,購物,包郵,服飾,shop,網店,舊金山,三藩市,美國
搞笑幽默,新闻趣事,旅游,宁波生活
读书分享,美图摄影
福州生活,美食,旅游
旅游,美食,新闻趣事,名人明星
旅游
旅游
视频音乐,名人明星
美食
新闻趣事,美容服饰,搞笑幽默,苏州生活,微博奇葩,穿衣美容,美女
视频音乐,旅游,美食,新闻趣事,搞笑幽默
穿衣美容,视频音乐,爱尔兰生活,萌宠
视频音乐
美女,搞笑幽默,新闻趣事,视频音乐
视频音乐,星座命理
名人明星,旅游,美食,萌宠,搞笑幽默
军事天地,旅游,搞笑幽默
美女
视频音乐,新闻趣事,旅游,美食,遵义生活
承德生活
商洛生活,新闻趣事,微博奇葩
萌宠
美食,美女,微博奇葩,新闻趣事,重庆生活
疯子,美女
爱猫,自恋,射手座
美女
天王盖地虎,高级黑,大叔控腹黑控,耽美,周杰伦铁杆粉丝,偶稀饭睡觉觉,萌宠,微博奇葩,搞笑幽默,成都生活
萌宠
新闻趣事
名人明星,视频音乐
职场招聘,微博奇葩
穿衣美容,美女
美食,穿衣美容
美图摄影,读书分享,宠物图集,新闻趣事,旅游
视频音乐,美食,旅游,星座命理,微博奇葩
新闻趣事,视频音乐,IT 数码,旅游,美图摄影
流浪,hiphop,旅游,搞笑幽默,微博奇葩,穿衣美容,新闻趣事
微博奇葩,搞笑幽默
穿衣美容
妖精,SJ,ELF
美容服饰,穿衣美容
搞笑幽默,视频音乐,微博奇葩,新闻趣事,穿衣美容

旅游
名人明星
杭州生活,美食,旅游
美食,微博奇葩
电影控,自由,微博奇葩,生活大爆炸
珠海生活,学习,娱乐
旅游,Celebrities
搞笑幽默
星座命理,视频音乐,新闻趣事,搞笑幽默,济南生活
穿衣美容,天津生活,微博奇葩,旅游,萌宠
名人明星
旅游,美女,穿衣美容,星座命理,微博奇葩
名人明星
可爱控,个性,Music,天蝎,零食小达人
微博奇葩,深圳生活,旅游,星座命理,穿衣美容
美容服饰,育儿百科,名人明星,美食,微博奇葩,穿衣美容,江门生活
名人明星,惠州生活
腐女,重口味,摄影控,神经质,萌宠,旅游
财经资讯,旅游
旅游,美食,穿衣美容,视频音乐,郑州生活
河池生活
体育资讯,IT 数码,美女
萌宠
爱生活,发呆,爱睡觉,爱音乐,搞笑幽默
微博奇葩,美食,旅游,搞笑幽默,深圳生活
新闻趣事
美女,美食,视频音乐,旅游
搞笑幽默
视频音乐
搞笑幽默,喜欢一切美好,喜欢古风,淡定（真的？）,随性,音乐控,喜欢听歌,美食控,阳光
视频音乐,微博奇葩
星座命理
美女,视频音乐
美食,软妹御姐爱好者,日语渣,三次元
萌宠
美食
追星,萌宠,星座命理,视频音乐,名人明星
微博奇葩
新闻趣事,搞笑幽默,名人明星
穿衣美容,新闻趣事,搞笑幽默,星座命理,美食
IT,A 股,美女,美食,旅游
美女
新闻趣事,视频音乐
旅游

搞笑,语录,八卦爆料,宠物搞笑,新闻趣事,服饰,偶喜欢玩,微博互粉,小资,微博奇葩
十堰生活
旅游
美食
美食
书,美食,猫咪,摩羯座,学生
美食,穿衣美容,台湾生活
宠物图集
搞笑幽默
穿衣美容,名人明星,星座命理,微博奇葩,新闻趣事
星座命理
搞笑幽默
视频音乐
爱玩,爱生活,爱幻想,学生族九零后,爱吃
搞笑幽默
美女
非你莫属,爱情保卫战,今夜有戏,真人秀,天津卫视,美女,搞笑幽默,视频音乐,名人明星,星座命理
90 后,音乐,大学生,双鱼座,成都,music,微博控,吃喝玩乐,美女,搞笑幽默
旅游
体育资讯
萌宠,美食
音乐发烧友,幻想家,水瓶座,成都,张继科,旅游
嘉兴生活
美食
旅游,美食,搞笑幽默,穿衣美容,星座命理
少儿创意,艺术,绘画,动手,diy,积木,创意绘画,旅游,新闻趣事
吉安生活
原创,美食爱好者,吃货一枚,美食控,吃喝玩乐,旅游美食爱好者
美女
八卦杂谈,微博奇葩
读书分享,唯美,感动,微小说,文字
粉红控,旅游美食爱好者,发呆,购物,爱睡觉,看电影,爱音乐,微博奇葩,新闻趣事,柳州生活
广告,电视,时尚,花儿朵朵,青海卫视,名人明星,美女,视频音乐,新闻趣事
名人明星
穿衣美容
穿衣美容
只要文爱,旅游
狗狗,天蝎,天蝎座 90 后,瑞士糖,星座运势,学生,天蝎座的 90 后,小摩尔,RK 联盟,摩尔庄园
视频音乐
体育资讯,旅游,美食,游戏,上海生活
新闻趣事,名人明星
美食
星座命理,新闻趣事,旅游,美食

旅游
视频音乐
吴亦凡
游戏,职场招聘,美女,美食
傻
音乐,VOCALOID,镜音连,手绘,新番,COSPLAY,动漫
宜昌生活,旅游,美女
新闻趣事,美食,旅游,深圳生活,搞笑幽默
搞笑幽默
搞笑幽默,星座命理,美女,名人明星,视频音乐
视频音乐
美图摄影,北京生活
福州生活
美女,美食,新闻趣事,搞笑幽默,安阳生活
搞笑幽默,星座命理,穿衣美容,名人明星
北京生活,德国,喜欢听歌,音乐发烧友
名人明星
综艺,主持人,大地飞歌,真人秀,湖北卫视,美女,视频音乐,新闻趣事,名人明星,星座命理
搞笑幽默,西安生活,穿衣美容,名人明星,视频音乐
微博奇葩,搞笑幽默,绍兴生活,新闻趣事
旅游
微博奇葩
微博奇葩,搞笑幽默
名人明星
校园生活
校园生活,视频音乐,新西兰生活
苦逼医学生,94,长沙,李宇春,神话
美食
新闻趣事
惠州建设,惠州规划,惠州房产,惠州衣食住行,惠州海港,惠州旅游胜地,惠州娱乐购物,惠州最热门,惠州天气
穿衣美容
我是个纯娘们,不打架但留长发,不抽烟不喝酒,疯疯癫癫是二货
萌宠,美食,星座命理,穿衣美容
搞笑幽默,美女,美食,微博奇葩,新闻趣事
搞笑幽默
搞笑幽默
穿衣美容
搞笑幽默
美女,名人明星
绍兴生活
美食,旅游
乌鲁木齐生活
萌宠

旅游
生活, 腹黑
美食
香港生活
穿衣美容
旅游, 隔油, 化粪池, 污水处理
新闻趣事, 搞笑幽默, 北京生活
搞笑幽默, 星座运势, 萌宠, 微博奇葩, 视频音乐, 新闻趣事, 星座命理
美容服饰, 穿衣美容
旅游
视频音乐
读书分享, 美图摄影
美女
搞笑幽默, 名人明星, 萌宠, 视频音乐, 微博奇葩
杭州生活
搞笑幽默, 微博奇葩, 穿衣美容, 旅游, 美食
美食, 武汉生活
重口味, 懒人, 新闻趣事
旅游, 美食, 微博奇葩, 穿衣美容, 成都生活
旅游, 新闻趣事, 穿衣美容, 星座命理, 微博奇葩
旅游
穿衣美容
新闻趣事
微博奇葩
视频音乐, 爱自由, 音乐控, 赛车, 爱玩, 爱幻想, 穿衣美容, 名人明星, 萌宠
视频音乐, 游戏, 微博奇葩, 旅游, 萌宠, 新闻趣事, 搞笑幽默
旅游
插画, 书, 电影爱好者, 绘画, 神经质, 平面设计, 宅女加腐女, 美食
搞笑幽默
历史文化, 游学, 旅游攻略, 商务考察, 国外自驾, 海外自驾, 自驾, 海外, 旅游
生物工程, 健康产业, 房地产开发, 科技, 中医, 黄石生活
常州生活
运动, 乐观, 吃, 美食, 狮子座, 搞笑, 游戏, 天蝎座, 宅女, 天秤座
名人明星, 旅游
海外生活
美女
旅游
萌宠
德阳生活, 旅游, 新闻趣事, 搞笑幽默
搞笑幽默, 美图摄影, 名人明星, 穿衣美容
名人明星
星座运势, 名人明星, 星座命理, 微博奇葩, 搞笑幽默
星座命理, 美食, 萌宠, 旅游, 视频音乐
搞笑幽默, 宁德生活, 旅游, 微博奇葩, 新闻趣事

胡思乱想,青春
旅游
广州生活
旅游,美食,鄂州生活,星座命理,名人明星
新闻趣事
微博奇葩,新闻趣事,搞笑幽默
微博奇葩
校园生活
旅游,穿衣美容,搞笑幽默
海外生活
美容服饰,读书分享,美食,新闻趣事,上海生活
视频音乐,星座命理,穿衣美容,名人明星,旅游
新闻趣事
文学,学习,学生一族,热爱生活
新闻趣事
美食,星座命理
旅游
视频音乐
美食,萌宠,星座命理,穿衣美容
十堰生活
星座命理
旅游,美食
新闻趣事,旅游,美食,视频音乐,广州生活
美食,视频音乐,新闻趣事,搞笑幽默
名人明星
李炜,喜欢音乐,爱自由,爱音乐,粉红控,狮子女,听音乐,音乐发烧友,猫咪,学生一族
新闻趣事,石嘴山生活,萌宠
芜湖生活,新闻趣事,旅游,美食,穿衣美容
星座命理,视频音乐,名人明星,旅游,美食
微博奇葩,穿衣美容,新闻趣事,名人明星,美女
星座命理
双鱼,狗狗,淡定,动漫迷,闷骚,80 後,设计师,维他命,新闻趣事
微博奇葩,星座命理,视频音乐
喜欢听歌,爱美食,安静,奋斗,旅游,搞笑幽默,新闻趣事,宜宾生活
教育,科比,摩羯,旅游,搞笑幽默,南京生活
旅游,美食,微博奇葩,穿衣美容
视频音乐,学生一枚,巨蟹座,韩国,b 型血,维尼夫妇
新闻趣事,美女,穿衣美容
新闻趣事
休闲,音樂
视频音乐
微博奇葩,星座命理,新闻趣事,搞笑幽默,宁波生活
搞笑幽默
八卦杂谈

搞笑幽默
广州生活
美女
美女
美食
星座命理
星座命理,微博奇葩,搞笑幽默
名人明星
旅游,美女,穿衣美容,包头生活,星座命理
穿衣美容
八卦杂谈
美女
美女
小吃,特产,湘菜,美食
杂志,教师,美剧,阅读,文学,手机看小说,考研,微博奇葩,新闻趣事
桂林生活
简单,旅游,美食,文字控
美食,视频音乐,名人明星,旅游,新闻趣事
谢娜,哲学,开心,蓝色,旅游,美食,新闻趣事,微博奇葩,北京生活
微博奇葩
美图摄影,星座运势,名人明星,新闻趣事,校园生活
美食
美女,其他生活,视频音乐,名人明星,搞笑幽默
搞笑幽默,美容服饰,西安生活,微博奇葩,名人明星,穿衣美容,美食
穿衣美容,新闻趣事,美食,星座命理,搞笑幽默
旅游,视频音乐,搞笑幽默,微博奇葩
搞笑幽默
穿衣美容
Food,Travel,Video,北京 Lifestyle
美食,穿衣美容,星座命理,搞笑幽默,武汉生活
漳州生活,穿衣美容
名人明星
搞笑幽默,萌宠
搞笑幽默,微博奇葩,梅州生活
穿衣美容
真人秀,高清,电影,电视剧,乐视,美女,名人明星,视频音乐,新闻趣事,搞笑幽默
视频音乐
穿衣美容
名人明星,微博奇葩
美食,旅游,名人明星,星座命理,穿衣美容
新闻趣事,搞笑幽默
搞笑幽默
美食
旅游

绍兴生活
美食,旅游,星座命理
微博奇葩,旅游,视频音乐
穿衣美容
睡觉,自由,学生,宅,微博奇葩
sa,拉稀饭,sk
搞笑幽默,埃及生活
梅州生活
名人明星
视频音乐,搞笑幽默,新闻趣事,穿衣美容,福州生活
睡觉爱好者,恋家的巨蟹座,喜欢温暖的人,独立,美食,穿衣美容,武汉生活
新闻趣事,IT 数码,搞笑幽默,旅游,微博奇葩,视频音乐
搞笑幽默
旅游
美女,科学探索,车世界,视频音乐,搞笑幽默
海口生活,穿衣美容
福州生活,名人明星,美食,旅游,新闻趣事,巧克力
美食,旅游,星座命理,微博奇葩,新闻趣事
旅游,美食,萌宠,微博奇葩,搞笑幽默
微博奇葩,搞笑幽默
校园生活
星座命理,微博奇葩,新闻趣事,搞笑幽默,盐城生活
肇庆生活
绍兴生活
星座命理,搞笑幽默
旅游,动漫,科学探索,美图摄影,宠物图集,萌宠
动漫,体育资讯,美食,视频音乐,搞笑幽默,美女,星座命理
iBreak,iSport,iSong,iTour,iFood,iParty,iFlower,iMusic,iNike,MACE
程序员,阿森纳
视频音乐
美图摄影
嘉兴生活,萌宠,美食,穿衣美容
篮球爱好者,双鱼座,好书,银魂,考研,美女,萌宠
名人明星
天蝎女,武汉,偏执狂,喜欢听歌,萌宠
萌宠,美食,星座命理,微博奇葩,搞笑幽默
北京生活,旅游
时尚,八卦,宅,购物
视频音乐,萌宠,搞笑幽默,美食,微博奇葩
新闻趣事
通行证,签证,虎门,专业团队,门票,机票,酒店,旅行社,旅游,东莞生活
Fun,音乐,啤酒,电影,桌游,私房火锅
南平生活
美食,搞笑幽默,福州生活

搞笑幽默,新闻趣事,美女,旅游,微博奇葩
视频音乐
北京生活,简单,爱电影,爱旅游,大学,财经
星座运势
理财,会计
新闻趣事,视频音乐,名人明星,微博奇葩,搞笑幽默
旅游,萌宠,美食,穿衣美容,搞笑幽默
广州生活
新闻趣事,厦门生活,穿衣美容,旅游,美食
星座命理,微博奇葩,名人明星,穿衣美容
名人明星
石家庄生活,搞笑幽默,视频音乐
微博奇葩,旅游,星座命理,美女,搞笑幽默
美食,旅游,星座命理,穿衣美容,上海生活
美女
沈阳新娘,婚纱出租,韩式造型,沈阳婚庆,美容服饰,沈阳生活,穿衣美容
新闻趣事
视频音乐
旅游
澳大利亚生活
美食
IT 数码
美图摄影,星座运势,美食,搞笑幽默,名人明星,萌宠
名人明星
美食,旅游,星座命理,视频音乐,新闻趣事
股票,证券,财经,A 股,投资,理财,金融,股市
旅游
吃喝玩乐,情感生活,热爱生活,徐州生活
名人明星
旅游,美女
旅游
苏州生活,视频音乐,萌宠,美食,穿衣美容
美女
美女
旅游,美女,穿衣美容
美女
名人明星,旅游,视频音乐,美图摄影
旅游,厦门生活
美图摄影,校园生活
微博奇葩
健康养生,新闻趣事,旅游,视频音乐,微博奇葩,潍坊生活
微博奇葩,穿衣美容,旅游,美食
创业,臭美,90 后但我不脑残
穿衣美容

搞笑幽默
IT 数码,校园生活,电脑,仲恺,新闻趣事,搞笑幽默,广州生活
视频音乐,美女,武汉生活,搞笑幽默,微博奇葩
美食,新闻趣事,佛山生活
美女
美女
美女
校园生活,东莞生活
八卦杂谈,校园生活,搞笑幽默,星座命理,微博奇葩,新闻趣事,潍坊生活
萌宠,搞笑幽默,新闻趣事,穿衣美容,美食
旅游,德宏生活,美食
搞笑幽默
蜡笔小新,灌篮高手,海绵宝宝,大嘴猴,HelloKitty 控,哆啦 a 梦,火影忍者,海贼王,壁纸控,动漫迷
美女
视频音乐,美女,名人明星,旅游,穿衣美容
旅游,美食
娛樂,数码,快乐大本营,移动互联网,美女,美食,新闻趣事,旅游
搞笑幽默,美容服饰,旅游,健康养生,视频音乐,微博奇葩,穿衣美容,美女
穿衣美容
搞笑幽默
名人明星
功夫,篮球,环保,喜欢唱歌,體育,天枰座,艺术
穿衣美容,微博奇葩,搞笑幽默,视频音乐,新闻趣事
旅游
搞笑幽默,美食,IT 数码,美容服饰,搭配
厦门生活
名人明星,穿衣美容,星座命理,搞笑幽默
八卦,时尚,萌,电影
时尚
平常心,善良,双鱼座,EXO,看书,爱音乐,热爱生活,乐天派,学生一枚,潮流
微博奇葩
美食
子女教育,德鲁克实践者,民企成长关注者,连续创业者,长跑者
时尚,萌,电影,音乐
时尚,娱乐,八卦
视频音乐
时尚,娱乐,八卦
浪漫情怀,电商,鲜花,旅行,北京生活
穿衣美容,名人明星
穿衣美容
时尚
时尚
时尚

时尚
时尚
时尚
时尚
旅游,美食,美女
微博奇葩,星座命理,美食,新闻趣事,宁波生活
武汉生活
新闻趣事
微博奇葩,星座命理,旅游
旅游,美食,星座命理,新闻趣事,萌宠
新闻趣事,搞笑幽默,微博奇葩
美食,旅游,萌宠,穿衣美容
名人明星
穿衣美容
服装设计,少女时代,EXO,BIGBANG,维尼夫妇,西安生活,旅游,名人明星,美食,视频音乐
乐橙,观众,广告,时尚,河南卫视,美女,名人明星,视频音乐,新闻趣事
搞笑幽默
美女
名人明星
商业,水瓶座,微平台建设营销,微信平台,微博营销,微博奇葩,新闻趣事,搞笑幽默,大连生活,美女
北京生活
郑州生活
星座命理,穿衣美容,旅游,美食,衢州生活
旅游
穿衣美容
漳州生活
时尚
美食,新闻趣事,微博奇葩,搞笑幽默
穿衣美容,乌鲁木齐生活
美食,网购,新闻趣事,穿衣美容,情感,喜欢唱歌,市场营销,旅行,听歌,90后
美食,旅游,微博奇葩,新闻趣事
旅游,美食,萌宠
名人明星
微博奇葩
新闻趣事,微博奇葩,美女,萌宠,搞笑幽默
穿衣美容
北京生活,情感生活,美女
时尚,摄影,八卦,购物
摄影,娱乐,宅
摄影,数码,宅,购物
旅行,摄影,宅
旅行,数码,娱乐,购物
游戏

美容服饰,动漫,搞笑幽默,八卦杂谈,美图摄影
信阳生活
佛山生活,旅游,穿衣美容
八卦,时尚,萌,电影,音乐
八卦,萌,电影,音乐
微博奇葩
八卦,萌,电影,音乐
八卦,时尚,萌,音乐
旅游
美食,名人明星
美女
美女
穿衣美容
美食
微博奇葩,搞笑幽默,美食,穿衣美容,新闻趣事,奢侈品,时尚潮流,旅行,阅读爱好者,购物达人
旅游
文化,大连生活
旅行,时尚,娱乐,八卦,宅
时尚,数码,娱乐,八卦,购物
旅行,摄影,娱乐,宅
旅游
美图摄影,校园生活,新闻趣事
活动,建筑,嘉兴,公益,街拍,家居,律师,嘉善,摄影,旅游
美女
微博奇葩,搞笑幽默,美食,视频音乐,哈尔滨生活
幽默,趣图,段子,搞笑
美食,视频音乐,名人明星
微博奇葩,视频音乐,合肥生活,星座命理,穿衣美容
微博奇葩,石家庄生活,搞笑幽默,星座命理,视频音乐
西安生活
搞笑幽默
减肥瘦身,溶脂针,瘦脸针,人胎素,美白针,玻尿酸,微整形
精,精丰,精丰粗,精丰粗粮
电视,时尚,云南卫视,品牌,fashion,名人明星,美女,视频音乐,新闻趣事
江门生活,萌宠
旅游
香港生活
校园生活,郑州生活
文字控,宅女加腐女,娛樂,数码,云南卫视,名人明星,美女,视频音乐,新闻趣事
无节操党,BC,神探夏洛克,妇联,抖森,LEEPECE,腐女,微博奇葩
动漫,萌宠,美女
郑州生活
旅游

新闻趣事,美女,校园生活,搞笑幽默,微博奇葩,雅安生活
DIY,创意,大学生,个性,奋斗,珠海生活
搞笑幽默
美食
旅游
美食,搞笑幽默,穿衣美容,广州生活
泰州生活
拍照,情绪化,怀旧,代购,护肤,美食,旅游
安阳生活,名人明星
视频音乐
名人明星
泉州生活,美食,穿衣美容
综艺,电影,电视剧,云南卫视,时尚,名人明星,美女,新闻趣事,视频音乐
美图摄影
昆明生活,穿衣美容,美食,旅游
美女
名人明星
八卦杂谈,美食
搞笑幽默,视频音乐
IT 数码,搞笑幽默,新闻趣事,美女,校园生活
杭州生活,新闻趣事
新闻趣事,名人明星,美女,微博奇葩,搞笑幽默
天蝎,大学生,大学,交朋友,美术,好性格,学生一族,星座控
爱旅行,搞笑幽默
旅游,中山生活,视频音乐
微博奇葩,新闻趣事,揭阳生活,搞笑幽默
其他生活
新闻趣事,成都生活,搞笑幽默,微博奇葩,视频音乐
读书分享,视频音乐,校园生活,红河生活,旅游
美食,穿衣美容,微博奇葩,搞笑幽默,北京生活
吉他,夜猫子,兔子,水瓶座
星座控,美食
美女,名人明星,视频音乐
星座运势,其他生活
中国好声音,快乐男声,流行音乐,周杰伦,摇滚,名人明星,美女,视频音乐,新闻趣事
盗墓笔记,搞怪,文学,创意,心理学
广州生活
郑州生活
微博奇葩,搞笑幽默
美食
美食,旅游,美女,穿衣美容,南阳生活
爱鼓捣,创客,开源硬件,软硬结合
美容服饰,穿衣美容
名人明星,美食,新闻趣事,穿衣美容,星座命理

时尚,中国好声音,苏打绿,小众音乐,摇滚,名人明星,美女,视频音乐,新闻趣事
广州生活
微博奇葩,美女,旅游,搞笑幽默,淄博生活
萌宠,视频音乐,徐州生活,美食,旅游
新闻趣事,旅游
搞笑幽默
南宁生活,搞笑幽默
上海生活
萌宠,微博奇葩,穿衣美容,搞笑幽默,名人明星
穿衣美容,美食,杭州生活
珠海生活
搞笑幽默,旅游,美食,德阳生活
我是歌手,快乐男声,中国好声音,摇滚,影视歌曲,名人明星,美女,视频音乐,新闻趣事
美食
微博奇葩,萌宠
穿衣美容
美女,萌宠
美食
笔记本,书签,明信片,创意,艺术,手绘定制,美食,旅游,北京生活
旅游,名人明星,体育资讯,视频音乐,搞笑幽默
穿衣美容
旅游,美食,穿衣美容,新闻趣事,六安生活
坚强,喜欢音乐,爱美食,双鱼,旅行,电影,听歌,90 后
美食
穿衣美容,微博奇葩,搞笑幽默
穿衣美容,名人明星,微博奇葩,新闻趣事,美女
传媒,爱旅行,浪漫,热情,微博控,時尚,互粉,美食
视频音乐,新闻趣事
旅游
IT,电子商务,帅哥,美食,微博奇葩,新闻趣事,搞笑幽默,潮州生活
情感生活,名人明星,旅游,美食,穿衣美容,合肥生活
旅游,新闻趣事
世界杯,笑话,90,火影,成都,自由职业,好书,亲情,财经,低调
美图摄影,星座运势,情感生活,名人明星,遂宁生活,微博奇葩
美女
视频音乐
新闻趣事
微博奇葩
视频音乐
美食,搞笑幽默,微博奇葩,旅游,泉州生活
美食
微博奇葩,搞笑幽默
旅游
旅游,美食

旅游
名人明星
搞笑幽默
爱力宏,爱生活爱旅游,个人猎头,人才中介,德盛人才
情感生活,玩,睡懒觉
美容服饰,情感生活,星座运势,新闻趣事,名人明星
搞笑幽默,日韩,服装,美女
美食
读书分享,宠物图集,校园生活,搞笑幽默,新闻趣事,微博奇葩
美食,名人明星,萌宠,星座命理,视频音乐
旅游,名人明星,北京生活
美食,旅游,名人明星,上海生活,搞笑幽默
美女,温州生活
双重性格,吃,乐观,宅女,吃货,旅游
微博奇葩
旅游
爱玩,学生一族,睡懒觉,我就是我,90 后但我不脑残,懒人,爱吃,射手,宅,搞怪
其他生活
新闻趣事,名人明星,BIGBANGISVIP,90 后非脑残,VIP 一枚
萌宠
alin,苏见信,俄克拉荷马雷霆,Beyonce,Aguilera,MariahCarey,巴萨
名人明星
美食
挺宅的,喜欢音乐,漫画控,金牛座
美食,视频音乐,微博奇葩
动漫迷,耽美,萌宠,旅游,穿衣美容,搞笑幽默,美食
美食,视频音乐,长沙生活,萌宠,旅游
名人明星,旅游,视频音乐,搞笑幽默,珠海生活
新闻趣事
搞笑幽默,新闻趣事,河源生活,微博奇葩
萌宠
微博奇葩,名人明星,视频音乐,新闻趣事,星座命理
搞笑幽默,微博奇葩
新闻趣事,搞笑幽默,视频音乐,旅游,美食
穿衣美容
IT 互联网
杂志,视频音乐,CNBLUE,爱音乐,韩剧,快乐大本营
旅游
美食
爱某某,敏感,80 後,瑜伽,北京生活
宠物图集,萌宠
旅游,视频音乐,北京生活
美女
DKIT 中国学联,DKITCSU,DKIT,唐道克理工学院

搞笑幽默
哈尔滨生活
淘宝,网购,女装,港台剧,时尚,名人明星,美女,视频音乐,新闻趣事
穿衣美容,星座命理,旅游,美食
视频音乐
萌宠,旅游,青岛生活
校园生活
校园生活
成都生活
全装备,激情,热血男人,健身,腰旗橄榄球,美式橄榄球
旅游
名人明星,美食,旅游,星座命理,穿衣美容
名人明星,微博奇葩,穿衣美容,沧州生活
穿衣美容,搞笑幽默,微博奇葩,名人明星,新闻趣事
读书分享,美容服饰,搞笑幽默,视频音乐,情感生活
新闻趣事,搞笑幽默
穿衣美容
穿衣美容
穿衣美容
名人明星
美食
美容服饰
北京生活
重口味,校园生活,视频音乐,其他生活,搞笑幽默,微博奇葩
电子商务,美食控,爱旅游,时尚,美女,爱猫,摄影控,双子,营销,穿衣美容
郑州平面设计,河南印刷,郑州彩印,郑州印刷
兰陵王,两个爸爸,冲上云霄,港台剧,少女时代,名人明星,美女,视频音乐,新闻趣事
美食,穿衣美容,新闻趣事,三明生活,微博奇葩
视频音乐
吃货,日本,B 型血,交朋友,逛街,看电视,玩游戏,喜欢音乐,90 後,互粉
旅游,美女,名人明星
重庆生活
大学生活,运动赛事,校园文化,RUCer,视频音乐,旅游,名人明星,北京生活
美女,名人明星,微博奇葩,兰州生活,穿衣美容
宠物图集,双子座,90 后但我不脑残,爱猫一族,美瞳控,萌宠
读书分享,穿衣美容
温州生活
穿衣美容
视频音乐
泰国,写小说,80 後,A 型血,IT 數碼,重口味,上网,摄影,水瓶女,旅游
北京生活
美食,搞笑幽默,旅游
旅游,美食,新闻趣事,搞笑幽默
校园生活,环保,志愿者组织,慈善公益

美食
北京生活
旅游, 新闻趣事, 微博奇葩, 温州生活
旅游
健身, 爬山
美食, 旅游
搞笑幽默
穿衣美容, 美食, 汕头生活, 旅游
美图摄影, 微博奇葩
读书分享, 香港生活
乐橙, 90 后, 双鱼座, 广东生活, 旅游美食, 潮人
烹饪, 移动互联网, 广告, 媒体, 传媒, 娛樂, 影视, 名人明星, 美食, 旅遊
朋友, 交友, 沉默, 内向, 心情, 视频音乐, 微博奇葩, 新闻趣事, 搞笑幽默, 十堰生活
音乐, 读书, 电影, 温暖, 治愈, 民谣, 卡尔西法, 电台
杭州生活
名人明星, 美食, 旅游, 视频音乐, 穿衣美容
穿衣美容
搞笑幽默
90 后, 音乐, 名人明星
音乐, 搞笑幽默, 视频音乐, 穿衣美容, 美食, 星座命理
宠物图集, 美食, 视频音乐, 搞笑幽默, 名人明星
旅游, 美女, 厦门生活
天津生活
旅游, 美食, 星座命理, 济宁生活
美食
新闻趣事
旅游
星座命理
美女
新闻趣事
衡水生活
微博奇葩, 宜宾生活, 搞笑幽默
互粉, 旅遊, 90 後, 美食, 搞笑幽默, 丽水生活
星座命理
网络, 外贸, 房产, 科技, 金融, 新闻资讯, 投资理财, 法律, 新闻趣事, 旅游
星座运势, 新闻趣事
奋斗, 简单, 爱电影, 摇滚, 书, 偏执狂, 阳光男孩, 沉默, 自由, 精彩语录
微博奇葩, 新闻趣事, 搞笑幽默, 重庆生活
旅游, 美食, 穿衣美容
穿衣美容
微博奇葩, 旅游, 新闻趣事, 搞笑幽默, 大庆生活
八卦杂谈, 体育资讯, 名人明星, 新乡生活
情感生活, 搞笑幽默, 星座运势
绵阳生活, 视频音乐, 旅游

动漫
视频音乐,名人明星,星座命理,微博奇葩,搞笑幽默
广州生活
旅游
名人明星
北京生活
名人明星,微博奇葩,视频音乐,新闻趣事,搞笑幽默
旅游
育儿百科,旅游,廊坊生活
旅游
星座命理,搞笑幽默,旅游,穿衣美容
乐天派,蓝色,音乐,汽车,梦想,张杰控
新闻趣事
视频音乐
美食
搞笑幽默
微博奇葩,萌宠,星座命理
临沂,鲁能泰山球迷
搞笑幽默,微博奇葩
新闻趣事
搞笑幽默,穿衣美容
校园生活
视频音乐,微博奇葩
MEL,墨尔本,假发,丝袜,日本,日系风格,日系,团购,shopping,澳大利亚生活
美食,南京生活
搞笑幽默
读书分享
经济,管理,旅游,美食,新闻趣事
新闻趣事,视频音乐
西安生活,飞出地球到太空,去旅行,爱音乐,爱电影,爱自由,背包客
动漫
校园生活,新闻趣事
职场招聘,合肥生活
南宁生活,美容服饰
三明生活
搞笑幽默,新闻趣事,苏州生活,美食,旅游,历史文化,读好书,冷笑话大王,健康生活,心理学
育儿百科
旅游,美图摄影,视频音乐,合肥生活
玩,睡懒觉,爱吃,可爱,美图摄影,情感生活,搞笑幽默,名人明星,美食
校园生活,新闻趣事,宠物图集
车世界,视频音乐,搞笑幽默,体育资讯
IT 数码,旅游,搞笑幽默,美图摄影,校园生活,摄影控,学生
八卦杂谈,IT 数码,校园生活,新闻趣事,搞笑幽默
打篮球,爱旅游,爱电影,爱生活,90,爱唱歌,善良,水瓶座,校园生活

美图摄影,美食,新闻趣事,旅游,泰国生活
宠物图集,新闻趣事,情感生活,美食,校园生活
美食
化妆品,美图摄影,校园生活,视频音乐,美食,名人明星
名人明星
游戏,视频音乐
穿衣美容
杭州生活,美女
视频音乐,搞笑幽默,名人明星,旅游,美食,穿衣美容
男饭,exo,baekhyun,白贤,边伯贤
搞笑幽默
自然,善良,音樂,85后,文学,旅游美食爱好者,完美主义,吃货一枚,白羊女,小女人
视频音乐
搞笑幽默
河源生活
穿衣美容
美女,深圳生活,视频音乐
微博奇葩,视频音乐,新闻趣事,旅游
美图摄影,新闻趣事,视频音乐,八卦杂谈,搞笑幽默
健身
黑暗组织,宫野志保,灰原哀,语C,快青,平和,新兰,名侦探柯南
搞笑幽默,旅游,萌宠,美食
化妆,乒乓球,外贸,時尚,电影迷,旅游摄影,电影控,美食爱好者,互粉,健身
名人明星
旅游,微博奇葩,搞笑幽默,新闻趣事,湛江生活
名人明星,旅游,西安生活,微博奇葩,穿衣美容
旅游
宠物图集,萌宠,星座命理,美女,穿衣美容,原创设计,淘宝店长,淘宝掌柜
街拍,微博奇葩,新闻趣事,萌宠,旅游,三门峡生活
科学探索,读书分享,体育资讯,校园生活,星座命理,新闻趣事
美食,旅游,穿衣美容,搞笑幽默,长沙生活
新闻趣事,名人明星,旅游,星座命理,视频音乐
电脑,B型血,汽車,wow,历史,英语,旅游,视频音乐,美女,微博奇葩
公益
新闻趣事
星座命理,名人明星,美食,搞笑幽默,天津生活
新闻趣事,美女,美食,宜宾生活
旅游
视频音乐,新闻趣事,美食,西安生活,名人明星
旅游,美女,视频音乐,搞笑幽默,新闻趣事
视频音乐
星座命理
财经资讯
北海生活

昆明生活,校园生活
星座运势
美容服饰,星座运势,情感生活,读书分享
健康养生,教育,美图摄影,美食,情感生活
搞笑幽默
美食
香港生活
大连生活,情感生活,星座运势,名人明星
星座运势
新闻趣事
车世界,视频音乐,IT 数码,旅游,搞笑幽默
情感生活
八卦杂谈,新闻趣事,厦门生活
新闻趣事,宠物图集
美图摄影
搞笑幽默
新闻趣事
精雅,睡衣,内衣,文胸,☆十色衣☆,淘宝店铺
游戏,美女,体育资讯
美食,搞笑幽默,庆阳生活,旅游,新闻趣事
济南生活
名人明星,美图摄影
名人明星,校园生活,广州生活
星座运势,自行车
搞笑幽默,校园生活,情感生活
读书分享
旅游
名人明星
搞笑幽默
宠物图集,重庆生活,星座运势,美食,名人明星
视频音乐,美图摄影,体育资讯,校园生活
名人明星,搞笑幽默,校园生活,情感生活
美图摄影,美食,新闻趣事,宠物图集,星座运势
校园生活,视频音乐,美容服饰,美图摄影,美食
搞笑幽默,资阳生活,健康养生
濮阳生活,美容服饰
三国杀,朋友,无聊,文字控,苹果,80 后宅女
美图摄影
新闻趣事
读书分享
美图摄影
芭比,光疗,美甲控,IBD,OPI,美甲店,爱美甲
新闻趣事
美容服饰

读书分享,名人明星,宠物图集,新闻趣事,校园生活
美容服饰
IT 数码,美图摄影,旅游,美食,美容服饰
视频音乐,世界在改变
美容服饰
搞笑幽默
想跳槽的,应届生往届生,就业信息,葡萄牙语
校园生活,IT 数码,体育资讯,视频音乐,新闻趣事
吃货,番茄,车,天气,木耳,美容养生,红枣,桂圆肉,养生,美食
视频音乐,新闻趣事,名人明星
视频音乐,新闻趣事,星座运势,校园生活,湖州生活
读书分享,美图摄影,情感生活,成都生活
心情,摄影控,旅游美食爱好者,创意,淡定,简单,爱电影,化妆品,计算机,散步
搞笑幽默,美容服饰,北京生活
新闻趣事,美图摄影,苏州生活,美食
北京生活
东莞生活
旅游,育儿百科,情感生活,名人明星,深圳生活
名人明星,校园生活,宠物图集,星座运势,美食
新闻趣事,宠物图集,美图摄影
美女,搞笑幽默
八卦杂谈,花样美男,名人明星,美食,搞笑幽默
旅游,八卦杂谈,搞笑幽默,其他生活,新闻趣事
美容服饰
搞笑幽默
双语教学,英文电影,mooc,公开课,纪录片,英剧,美剧,英语学习,爱电影,科学探索
美食
宠物图集,新闻趣事,美食,校园生活,搞笑幽默
韩国生活,多肉植物,韩国多肉,肉肉,韩国代购,多肉代购
新闻趣事,旅游,视频音乐,校园生活,上海生活
美食,读书分享,校园生活,视频音乐,搞笑幽默
山东同乡会,深圳大学
校园生活
美图摄影,体育资讯,IT 数码,旅游,校园生活
欧美,天秤,家居,猫控,计算机,遊戲動漫
旅游
美女
名人明星,校园生活,视频音乐,八卦杂谈,搞笑幽默
育儿百科
轩贝,萧亚轩
名人明星
视频音乐
天秤座,善良,美食,网购,上海生活,美容服饰
美食

揭阳生活
神枪手,军事
小糊涂,luhan,EXO,开心的舔屏饭,勋鹿,天秤纠结女,鹿晗是心头肉
校园生活
搞笑幽默
美容服饰
八卦杂谈,新闻趣事,视频音乐,美女,搞笑幽默
天秤座,90 后但我不脑残,美食控,星星 and 娜米,懒人,互粉,影视表演学生,维尼夫妇,尼坤 Nichkhun,张杰
新闻趣事,名人明星,美图摄影,搞笑幽默,海口生活
吃货,神经病,科比,周杰伦
吃货,帅哥控,美容,粉色系
旅行,美剧,紫色
爱哭,星座控,自恋,萝莉,紫色
爱哭,神经病,周杰伦,帅哥控,粉色系
韩剧,美剧,单身达人
时尚,吃货,科比,自由,单身达人
校园生活,读书分享,IT 数码
哈密瓜,2NE1
体育资讯
动漫
西式,浪漫,咖啡控,咖啡馆
美食,花样美男
潮男,型男,时尚,In,潮流,男装
名人明星
星座运势
视频音乐,美食,新闻趣事,IT 数码,北京生活
运动,登山,摄影爱好者,书法
搞笑幽默
连云港生活
职场招聘
美图摄影,新闻趣事,其他生活,视频音乐,美食
搞笑幽默
腐女,古代言情小说迷,f（x）函数,紫色控,honey 峯迷,金牛座,双重性格,腹黑女
育儿百科
双鱼,火影,游戏动漫,体育资讯
宠物图集,星座运势,视频音乐,搞笑幽默
美食,保定生活,视频音乐,读书分享
赚钱,足球迷,新闻趣事,美女,视频音乐,体育资讯,旅游
学习,梦想,广东生活,唱 k,o 型血,市场营销,白羊,90 后,音乐
80 后的尾巴,打篮球,吃货,听音乐,睡觉,唱 k,美食,游戏,视频音乐
情感生活
美食,美图摄影,读书分享,深圳生活,美容服饰
其他生活,美食,美容服饰,视频音乐

旅游,美图摄影,校园生活,大连生活,搞笑幽默
名人明星,俞 DD,俞承浩,海芋星
美女,旅游,游戏,美食,IT 数码
情感生活,育儿百科,美容服饰,名人明星,清远生活
搞笑幽默
旅游
IT 数码,美图摄影,美女,视频音乐,搞笑幽默
美女,泉州生活
读书分享,新闻趣事,体育资讯,旅游,名人明星,校园生活
青黑党,打鸡血的白羊,别扭小孩,蟑螂仇人,霸道爱逞强,動漫,宅腐双修恋声癖,口是心非
八卦杂谈,体育资讯,新闻趣事,搞笑幽默,北京生活
情感生活,花样美男,新闻趣事,名人明星,美容服饰
体育资讯
宠物图集,搞笑幽默,星座运势
美女,光山县,光山二高,光山
车世界,美食,美女,IT 数码,旅游
爱生活,家居,时尚潮流,团购,互粉,時尚,淘宝网购,淘宝控,购物达人,购物狂
新闻趣事,美食,情感生活,花样美男,美容服饰
搞笑幽默
搞笑幽默
情感,科学,艺术,交友,阅读,公益,思考,简单,哲学,文学
美容服饰,校园生活
旅游,搞笑幽默
名人明星
福州生活,美图摄影
投资理财
微博,爱情,阅读,贵阳生活,旅游,游戏,新闻趣事,IT 数码
情感生活
自由,听音乐,街拍,情感生活,交友,摄影,流浪,写字
搞笑幽默
高新一中
算命,八字,面相,占卜,塔罗,周易,易经,星座,命理
黄山房产超市网,家装,租房,二手房,新房,黄山房产
美食,美容服饰,时尚达人,化妆技巧,服装搭配,摄影电视摄像,航空服务,中医养生,人物形象
设计,教育培训
美女,搞笑幽默
情感生活,名人明星,搞笑幽默
动漫,校园生活,美图摄影
洋洋
美图摄影,乐天派,韩庚,爱电影,爱生活,射手女,旅行,90 后,自然,真实力
淘宝,发呆,购物,看电影,歌手,美食,动漫
上海生活,搞笑幽默
旅游,简单,体育资讯
动漫,其他生活,旅游

美食,旅游,视频音乐,美容服饰,搞笑幽默
疯子,文化,电影,古灵精怪,搞笑幽默,好友,旅游,谈天说地,红酒,意大利
八卦杂谈
美图摄影,美容服饰
读书分享,其他生活
搞笑幽默
美食,新闻趣事,旅游,健康养生,广州生活
视频音乐
健康养生,财经资讯,旅游,教育,泉州生活
八卦杂谈
心情,好性格,90 后,新闻资讯,王力宏,新闻趣事,IT 数码,体育资讯,校园生活,搞笑幽默
财经资讯,新闻趣事,美食
体育资讯
名人明星
重口味,搞笑幽默,视频音乐,校园生活,动漫
旅游,IT 数码,美图摄影,新闻趣事,北京生活
动漫,八卦杂谈,搞笑幽默,美女,校园生活
90 后非脑残,打篮球,台球,潮人,nba,遊戲動漫,动画,爱唱歌,笑话,电脑
美图摄影,美食,搞笑幽默,视频音乐
唱歌,自由
电台,名人明星,独立,娱乐
视频音乐
美容服饰,淘宝
吃货,互联网,电子商务,旅行,摄影,上网,美食控,户外活动,烧烤控,广州生活
美图摄影
聖武龍皇
体育资讯,搞笑幽默
高尔夫,GOLF
情感生活
星座运势
读书分享,宠物图集,搞笑幽默,新闻趣事
IT 数码
美图摄影
旅游美食,海产干货,海产,土特产,特产美食,读书分享,美食,温州生活
校园生活,读书分享
美食,美容服饰
读书分享,美食,新闻趣事,校园生活,桂林生活
搞笑幽默
潮流,逛街,新闻趣事,美食
美食,旅游,搞笑幽默,美容服饰,沈阳生活
重口味,星座运势,动漫,搞笑幽默
星座运势
电影,80 后,游泳,听歌,美食,美女,动漫,语录
搞笑幽默

读书分享,宠物图集,株洲生活,搞笑幽默
重庆生活
旅游
校园生活
艺术,HarryVanGestel,欧洲油画,荷兰当代油画
校园生活
八卦杂谈
名人明星,美容服饰
宅女,我就是我
旅游,名人明星,情感生活,美女,视频音乐
美女,八卦杂谈,搞笑幽默
IT 数码,美容服饰,美食,视频音乐,搞笑幽默
长沙生活,校园生活
美女
校园生活
IT 数码,新闻趣事,美图摄影,美女,校园生活
逛街,吃货一枚,喜欢听歌,IT 数码,广州生活
名人明星,体育资讯,美图摄影,校园生活
书,电影爱好者,文字,文学,古典艺术,自然,书籍,哲学,旅游摄影,向左走向右走
星座运势,新闻趣事,校园生活,情感生活
美食
环保
情感生活
宠物图集,科学探索,重口味,新闻趣事,搞笑幽默
八卦杂谈,美容服饰,旅游,宠物图集,搞笑幽默
车世界
视频音乐,美图摄影,读书分享
愤青,工作,文化,理财,向日葵
旅游,职场招聘,IT 数码,美女,徐州生活
读书分享,宠物图集,星座运势,大连生活
体育资讯
学生一族,完美主义
育儿百科,美容服饰,搞笑幽默,石河子生活
贵阳生活
教育,美食,财经资讯,健康养生
视频音乐
淡定,随性,偏执狂
海外生活
星座运势
花样美男,搞笑幽默,宠物图集,星座运势,美图摄影
宝宝,球球
简单,喜欢听歌,爱笑,爱旅游
美食
IT 数码

新闻趣事,搞笑幽默
美图摄影
搞笑幽默,好性格,双子座,小清新,萌宠
旅游
成都生活,乡村骑士,为爱行走,募款,立人乡村图书馆
校园生活,射手,高中生,文字,羽毛球,手机,烹饪,宅女加腐女,迷糊
睡觉
爱笑,天秤,知足,乐观,可乐,华晨宇
校园生活
美图摄影,美食
星座运势,校园生活,美图摄影,美食
名人明星,八卦杂谈
校园生活,视频音乐
读书分享,情感生活,星座运势,美食,校园生活
阅读,创业,创意设计,平面设计,90,电视剧,乐观,天蝎座,美食,吃货
养生,笑话,爱,时装,生活,网购,玉米,小资,情感生活,美容服饰
游泳,跑步,时尚,音乐,旅游,美食,宠物,桌球
美女
快乐,爱吃,爱幻想,原创,神经大条,重口味,90后非脑残,绘画,美术,自由
新闻趣事,育儿百科,旅游,美食,杭州生活
旅游,搞笑幽默,厦门生活
读书分享,新闻趣事,宠物图集,武汉生活,搞笑幽默
法律顾问,创业,合同管理,审合同
电影,听歌,情感生活,名人明星,星座运势
玩,睡懒觉,神经质,杭州,射手,动漫
新闻趣事,美食
爱美食
名人明星,宠物图集,视频音乐
IT数码,搞笑幽默
为了视频洒节操,多语言吐槽,野生JC,人活着，为逼格,节操掉了手捡,手机测评,IT数码
其他生活
美容服饰,苏州生活
成都生活
旅游,美女,游戏,车世界
90后，宅男，极客
名车,养车,车饰,购车,车模,时尚,车迷,汽车保养,汽车用品,汽车控
星座运势,名人明星,武汉生活,美食
列侯,兄控,夏侯廉,夏侯惇之弟
新闻趣事,旅游,视频音乐,金华生活
美食,新闻趣事,读书分享,美图摄影,杭州生活
IT数码
读书分享,旅游
情感生活
新闻趣事,宠物图集

视频音乐,校园生活,动漫,美食,星座运势
可爱,学生一族,梦想,神经质,青春,花样美男,留学生,新闻趣事,美食,名人明星
动漫,美图摄影,星座运势,搞笑幽默,校园生活
读书分享,情感生活,搞笑幽默,校园生活,视频音乐
星座运势
校园生活,宠物图集,名人明星,读书分享,美容服饰,美图摄影
旅游,手机,宅男,大爱梁静茹,音乐发烧友,搞笑幽默,英雄联盟
校园生活
美食,上海生活
名人明星
青春,爱自由,爱生活,购物,女人
旅游爱好者,新闻趣事,读书分享,情感生活
名人明星
其他生活,健康养生,美食,旅游,美容服饰
美容服饰
新闻趣事,重口味,视频音乐,美容服饰,八卦杂谈
动漫
重庆生活
三亚生活,校园生活,美食,名人明星,视频音乐
美女,科学探索,动漫,八卦杂谈,视频音乐
搞笑幽默
体育资讯
时尚,摄影,八卦,购物
旅行,数码,八卦
时尚,摄影,八卦,购物
数码,娱乐,购物
时尚,数码,娱乐,宅,购物
美图摄影,读书分享,旅游,美女,搞笑幽默
八卦杂谈
新闻趣事
美食,搞笑幽默
美女,游戏,苏州生活
其他生活
校园生活,动漫,美食
摄影,娱乐,八卦,购物
时尚,摄影,娱乐,宅,购物
旅行,摄影,数码,娱乐,宅
时尚,摄影,八卦
校园生活
美图摄影,美食,情感生活,新闻趣事
八卦杂谈,LOL,水瓶座,唱歌
爱音乐,音乐爱好者,周杰伦
星座运势,读书分享,情感生活,新闻趣事,柳州生活
教育

旅游,新闻趣事,视频音乐,搞笑幽默,青岛生活
校园生活,温州生活,体育资讯
美容服饰
市场营销
搞笑幽默
星座运势
欧美,80 后,旅行,穿衣美容,健康,文艺
校园生活
时尚,数码,娱乐,八卦,宅
喜欢漂亮衣服,吃货,西安生活
IT 数码
视频音乐,旅游,美女,校园生活,沈阳生活
名人明星
视频音乐,读书分享,新闻趣事,名人明星,搞笑幽默
新闻报道,时事评论,文化艺术,财经金融,南京生活
听音乐,写作,游泳,喜欢音乐,温柔,桌游,爱生活,单身
八卦杂谈,新闻趣事,搞笑幽默
美容服饰
杭州生活,美女
奋斗小青年,苦逼实习 ing
爱购物,爱美,女包,潮流,时尚
10 后,金牛座
校园生活
读书分享,宠物图集,新闻趣事,校园生活,搞笑幽默
旧设备进口清关,红酒进口清关,红酒进口报关,报关行,食品进口报关,进口清关,进口报关
宠物图集
长沙,搞笑幽默,岳阳生活,旅游,职场招聘,美女
搞笑幽默
阳光洒满大地,一路与您相伴,龙岩生活
美女
美女
视频音乐
美食,视频音乐,旅游,温州生活,新闻趣事
书籍,美食爱好者,人生,微博控,浪漫,交朋友,文艺,潮人,音乐迷,新闻趣事
搞笑幽默
美图摄影,搞笑幽默,新闻趣事,视频音乐,校园生活
情感生活
美女
校园生活,广州生活
搞笑幽默,校园生活
宅,旅行,电影,美容服饰,名人明星,新闻趣事,搞笑幽默,美食
美容服饰
新闻趣事,康熙来了
寂寞,生活大爆炸,篮球爱好者,游戏,体育资讯,IT 数码

情感生活,心情
情感生活,美容服饰,搞笑幽默,视频音乐,其他生活
视频音乐
美食,美容服饰,新闻趣事,情感生活,搞笑幽默
A 股,旅游爱好者,IT 数码,新闻趣事,美女,韶关生活
房产,美容服饰,美食,珠海生活,宠物图集
時尚,资讯,夜猫子,互联网,囧,潮人,爱旅行,写字,摇滚,情感
旅游,新闻趣事,名人明星,搞笑幽默,徐州生活
皇马
旅游,花样美男,读书分享,情感生活,宝宝,人力资源,囧,紫色,减肥,吃喝玩乐
活动策划,展览,策划,活动,建筑,艺术设计,平面设计,摄影艺术,创意,创业
校园生活,视频音乐,成都生活
新闻趣事,视频音乐,八卦杂谈,IT 数码,南昌生活
美食,新闻趣事
旅游
八卦杂谈,搞笑幽默,动漫,敏感,爱自由,听音乐,90 後,学生一枚,情感生活
美图摄影
美女
情感生活,新闻趣事
八卦杂谈
宠物图集,视频音乐,搞笑幽默,成都生活,美食
珠海生活
体育资讯,IT 数码,名人明星,校园生活,常州生活
搞笑幽默,长沙生活,旅游,读书分享,新闻趣事
日妆代购
美容服饰
k 歌,摄影控,校园生活
美食
美容服饰
财经资讯,视频音乐,IT 数码,搞笑幽默,丹东生活
诗歌,臭美,偶像剧,疯狂,杭州,重庆生活,宠物图集,视频音乐,旅游,花样美男
奥特朗公司,奥特朗热水器,奥特朗厨宝,即热式电热水器,速热式电热水器,预即双模热水器
美女
搞笑幽默,读书分享,名人明星,校园生活,青岛生活
文艺,娱乐,时尚,音乐,摄影,旅游,美图摄影,石家庄生活
情感生活,美食,名人明星,读书分享,美图摄影
广告礼品,企业形象礼品,会议礼品,外事礼品,文化衍生品,VIP 积分礼品,福利礼品,商务礼品
武汉生活,搞笑幽默
旅游,新闻趣事,IT 数码,搞笑幽默,北京生活
柳州生活
新闻趣事
美容服饰
美女,新闻趣事,美食,兰州生活

搞笑幽默
睡懒觉,湖北生活,音乐爱好者
视频音乐,搞笑幽默,情感生活,美图摄影,读书分享
搞笑幽默
星座运势,美食,视频音乐,搞笑幽默
美食爱好者,看小说,互粉
美食,做温暖的菇娘,写字,看书
美食,健康养生,旅游,搞笑幽默,成都生活
读书分享,新闻趣事,校园生活,搞笑幽默,天津生活
IT 数码,宠物图集,职场招聘,新闻趣事,车世界
校园生活
美图摄影
爱旅行,淘宝,网络,旅游爱好者
美容服饰
旅游,美食,美容服饰,北京生活
巨蟹,新闻趣事,宠物图集,读书分享,美图摄影,名人明星
车世界,汽车,IT,社会,博客
校园生活
军事天地,财经资讯,新闻趣事,搞笑幽默,苏州生活
私语,真情,文艺,时光机,校园
美女,新闻趣事,旅游,东莞生活,美图摄影
英国生活
美图摄影,名人明星,视频音乐
收缩毛孔,美白,祛斑,舒展干纹,祛痘除印,美容,护肤,去黑眼圈,去黑头
体育资讯,搞笑幽默,校园生活
新闻趣事,星座运势,美图摄影,视频音乐,黑河生活
八卦杂谈,美食,水果快递,水果,水果达人,武昌水果配送,精品水果,进口水果,武汉生活
宠物图集
业主论坛,万科物业,长安万科城,业主,万科,西安万科城,万科城
八卦杂谈
星座运势,情感生活,宠物图集,盘锦生活,搞笑幽默
美女
IT 数码,摄影,育儿,照相馆,孕妇摄影,孕妇照,亲子全家福,亲子照,亲子,亲
深圳生活,八卦杂谈,新闻趣事,校园生活,星座运势
动漫
旅游,美食,视频音乐,搞笑幽默,上海生活
上海生活,大功率水冷电机,直驱电主轴,舞台设备,高效节能电机,注塑机电机,变频电机,高速电机,伺服电机,电机销售
乐观,重庆,旅游,美食,新闻趣事,搞笑幽默,重庆生活
视频音乐,新闻趣事,搞笑幽默,石家庄生活
搞笑幽默
八卦杂谈,校园生活,美图摄影,美女
读书分享,情感生活,新闻趣事,宁波生活,校园生活
广州生活,校园生活

IT 数码,美食控,旅游爱好者,摄影爱好者,苹果控,激情人生,单车爱好者,死飛
名人明星,旅游,新闻趣事,美容服饰,南平生活
校园生活
美图摄影,新闻趣事,视频音乐,校园生活,桂林生活
潮州生活
美食
创意,爱吃,开朗
校园生活
校园生活
星座运势,美容服饰
IT 数码,视频音乐,昆明生活,搞笑幽默,音乐发烧友,大学生,重金属,摇滚,痴情,游戏
重口味,科学探索,其他生活
济南生活,宠物图集,车世界,游戏,IT 数码
星座运势,广州生活,搞笑幽默,新闻趣事
美容服饰
校园生活
美图摄影
搞笑幽默
情感生活,名人明星
闷骚,射手座,美食爱好者,火影忍者,唱 K,巴萨,足球,汽车,旅行
旅游,读书分享,美图摄影,美食,视频音乐
星座运势
其他生活
IT 数码,旅游,视频音乐,搞笑幽默
搞笑幽默
名人明星
唱 k,正太控,生活大爆炸,新闻趣事,育儿百科,视频音乐
美图摄影,星座运势,新闻趣事,视频音乐,佳木斯生活
IT 数码,美女,美图摄影,体育资讯
校园生活,新闻趣事
游戏,旅游,新闻趣事,IT 数码
美食,情感生活,上海生活
美容服饰,新闻趣事
卡卡,猫,篮球爱好者,体育资讯
美食
美容服饰,名人明星,星座运势,新闻趣事,情感生活
听音乐,爱自由,看电影,美剧控,搞笑幽默
车世界
体育资讯
搞笑幽默,美食,旅游,海口生活
信息,死亡笔记,进击的巨人,旅游,责任,成长,拼搏,自由,学习,固执
瑜伽,化妆,美剧控,星座运势,情感生活,美图摄影
美食,星座运势,宠物图集,贵阳生活
网球,微笑,情感,喜欢唱歌,视频音乐,美容服饰,动漫

视频音乐,美容服饰,花样美男,情感生活,银川生活
职场招聘,宠物图集,搞笑幽默,旅游
杭州,爱生活,中医,健康,情感生活,旅游,美食,杭州生活
美食,旅游,新闻趣事,IT 数码
旅游,美图摄影,八卦杂谈,搞笑幽默
美国生活
美容服饰
美容服饰
教育,新闻趣事,健康养生,视频音乐,旅游
校园生活,新闻趣事,美图摄影,情感生活,名人明星
职场招聘,新闻趣事,体育资讯
名人明星,视频音乐,体育资讯,旅游,IT 数码
美容服饰,美食,名人明星,搞笑幽默,视频音乐
校园生活,搞笑幽默,八卦杂谈,美女,德国生活
美容服饰
美容服饰
美容服饰
美图摄影
自由,自信,摄影师,单身,其他生活,时尚潮流,电脑,主持人,喜欢音乐,美图摄影
职场招聘,搞笑幽默,美女
视频音乐
旅游,美容服饰,美食
读书分享,美容服饰,情感生活,八卦杂谈,美食
南阳生活
旅游,美食,法国生活
名人明星
星座运势,美食,温州生活,搞笑幽默
视频音乐,读书分享,校园生活,美图摄影,长沙生活
校园生活
上海生活
名人明星,美图摄影
美图摄影,读书分享,科学探索,校园生活,八卦杂谈
美食,搞笑幽默,深圳生活
美图摄影
强迫症,摩羯女,格子控,敏感,欧美控,時尚,Music,iphone,咖啡,奋斗
旅游
车世界,IT 数码,NBA,羽毛球,巨蟹,90 後,打篮球,体育资讯,美图摄影
搞笑幽默,校园生活
南京生活
新闻趣事,读书分享,美图摄影,郑州生活,搞笑幽默
新闻趣事
新闻趣事
旅游,搞笑幽默,马来西亚生活,八卦杂谈
体育资讯

小女人,护肤,新闻趣事,八卦杂谈,名人明星,美女
视频音乐,新闻趣事,美图摄影,名人明星,美食
读书分享,美容服饰,旅游,视频音乐,美图摄影
其他生活,无厘头,人生,欧美音乐,看电视
美图摄影,情感生活,北京生活,视频音乐,名人明星
名人明星
美容服饰,育儿百科,美食,搞笑幽默
学生一族
宠物图集,美食,搞笑幽默,体育资讯,八卦杂谈
情感生活,肇庆生活
育儿百科
美女
财经资讯,新闻趣事,职场招聘,IT 数码,南京生活
职场招聘
CV 控,广播剧爱好者,动漫
苏州生活
名人明星
美容服饰
搞笑幽默
新闻趣事,美食,宠物图集,美容服饰,搞笑幽默
饭兄弟情,key,白羊座,SHINee
美食,新闻趣事
读书分享,美容服饰,校园生活,美食,美图摄影
新闻趣事
美容服饰,情感生活,美食,搞笑幽默,无锡生活
IT 数码
湛江生活
美容服饰,视频音乐,旅游
视频音乐
美食,北京生活
佛山生活
新闻趣事
台州生活
杭州生活,美容服饰,育儿百科
搞笑幽默,情感生活,星座运势
读书分享
读书分享,美图摄影,新闻趣事,校园生活,福州生活
双重性格,干物女,遊戯動漫,猫控
动漫,社会,书,宅,90 后,漫画,学生一枚,旅游,新闻趣事,搞笑幽默
视频音乐,美食,搞笑幽默,美图摄影,名人明星
星座运势,动漫,IT 数码,搞笑幽默
校园生活
美食
八卦杂谈

校园生活
旅游,视频音乐,美女,名人明星
听音乐,视频音乐,新闻趣事,美女,IT 数码,搞笑幽默
名人明星
新闻趣事,美食,旅游,美容服饰,芜湖生活
情感生活,美图摄影,读书分享,视频音乐,杭州生活
美图摄影,宠物图集
浪漫,微博,自信,真实力,美女
美女
新闻趣事
育儿百科,宠物图集,情感生活,新闻趣事,重庆生活
美容服饰
体育资讯
旅游,名人明星,美食,美容服饰,搞笑幽默
财经资讯,新闻趣事,视频音乐,IT 数码,重庆生活
永州生活
搞笑幽默,美食
新闻趣事
涨姿势,科普,爆笑,趣闻,女生,男生,两性
搞笑幽默,新闻趣事,旅游,名人明星,常州生活
福州生活
旅游,美食,海外生活,美图摄影,读书分享
情感生活,美食,美容服饰,新闻趣事,旅游
读书分享,旅游
情感生活,名人明星,校园生活,搞笑幽默,宝鸡生活
武汉生活
宠物图集
视频音乐,美食
读书分享
美食,科学探索,新闻趣事,校园生活,读书分享
学生,校园生活,动漫
广州生活
美图摄影,旅游
云浮生活
美容服饰,读书分享,校园生活
美食,IT 数码,唐山生活,美女,搞笑幽默
铁岭生活
视频音乐
美食
校园生活
校园生活
搞笑幽默
上海生活,美容服饰,搞笑幽默
简单,淘宝,购物,爱生活,团购,休闲,自由职业,登山,化妆品,北京生活

旅游,情感生活
美女,千叶县,日本动漫,数学,铁路
旅游
网购,体育资讯
美图摄影
读书分享,美食,名人明星,上海生活,校园生活
美食
美食,宠物图集
玩儿,时装,美图摄影
读书分享,美图摄影,美食,新闻趣事,校园生活
情感生活,星座运势,名人明星,校园生活,搞笑幽默
星座运势
旅游,宠物图集,情感生活
旅游,欧美范儿,服饰鞋子搭配,女靴,女潮人,鞋子控,时尚百搭,购物狂,淘宝控,流苏鞋
花样美男
读书分享,视频音乐,校园生活,美食,星座运势
八卦杂谈,生活,日常,潮货
松原生活
美图摄影
星座运势
宠物图集,星座运势
搞笑幽默,育儿百科,新闻趣事,名人明星,福州生活
校园生活,广州生活
校园生活
新闻趣事,搞笑幽默,读书分享
IT 数码
车世界,美图摄影,新闻趣事,漳州生活,校园生活
体毛控,健身爱好者,背心,黑袜,正装控,巨蟹
翰爽,张翰,郑爽,华天齐,穆小妍,方亦菲,胜女的代价 2,俞灏明,魏晨,朱梓骁
视频音乐
创意,品牌,电子商务,萌宠,摄影爱好者,原创,猫控,动画,多肉植物,美食
校园生活,加拿大生活
读书分享
贵阳生活
厨卫商学院,厨房电器,卫浴洁具,厨卫家电,整体橱柜,微营销,O2O,家居厨卫,酒店用品
河南生活,喜欢听歌,美食控,阳光,宠物图集
重庆生活,IT 数码,体育资讯,八卦杂谈,搞笑幽默
摄影,宅女,吃货,80 后,奢侈品,时尚
旅游,星座运势,读书分享,名人明星,视频音乐,桂林生活
旅游
美图摄影,车世界
旅游,美容服饰,视频音乐,读书分享,美图摄影
情感生活,育儿百科,美容服饰
理财,赚钱,书,服装,淘宝控,聊天,日本,英国生活,美食,美女

美容服饰,美食,旅游,重庆生活,情感生活,80後,小说迷,粉红控,佛教
李秀景,尹斗俊,用餐吧
旅游,IT 数码,搞笑幽默
读书分享,IT 数码,房产,旅游,乌鲁木齐生活
成都生活,视频音乐,动漫
美图摄影
观鸟,美图摄影,旅游
八卦杂谈,维修,养车,车载 APP,爱车,保养,洗车
职场招聘,贵港生活,搞笑幽默
怀旧,聊天,拍照,情感生活
上海生活
青岛生活
健康养生
IT 数码
郑州生活,搞笑幽默
原创,休闲,购物狂
八卦杂谈,新闻趣事,视频音乐,搞笑幽默
新闻趣事,美食,旅游,军事天地,美女
搞笑幽默,美女,校园生活,阳江生活,八卦杂谈
新闻趣事,八卦杂谈,视频音乐,搞笑幽默
新闻趣事,旅游,美食,美容服饰
搞笑幽默
美食,视频音乐,新闻趣事,搞笑幽默
搞笑幽默,视频音乐,名人明星,新闻趣事,美女
IT 数码,新闻趣事,体育资讯
旅游,名人明星,保山生活
新闻趣事,八卦杂谈,视频音乐,搞笑幽默
视频音乐,美食,兰州生活
深圳生活
搞笑幽默
旅游,美食,健康养生,情感生活,教育
情感生活,美容服饰,读书分享,美图摄影,校园生活
库尔勒,新疆巴州,香梨,梨城,巴州资讯,库尔勒资讯,库尔勒晚报,我在库尔勒,库尔勒生活
成都生活
成都生活
金融,房产
无锡生活
处女座,阳澄湖大闸蟹,美食
旅游
旅游
秦皇岛生活
车世界
旅游
星座运势

旅游爱好者,完美主义,搞笑幽默,美女,八卦杂谈
重庆,大学生,科技,校园生活
宠物图集,美食
爱音乐,美女,电影,完美主义,80后,水瓶座,幽默,陈奕迅,警察
旅游
杭州生活
车世界,海口生活
八卦杂谈
视频音乐,美食,旅游
旅游,美食
旅游,美食,新闻趣事,安庆生活
新闻趣事
房产,名人明星,IT数码
美女,名人明星,旅游,网购
历史讲坛,活泼,文藝,胡思乱想,南昌生活
读书分享,情感生活,美容服饰,星座运势,宠物图集
搞笑幽默,微博,交朋友,影视,翻译,留学生
搞笑幽默,名人明星,房产,财经资讯,体育资讯
美食,房产,IT数码,财经资讯,新闻趣事
读书分享
桂林生活
体育资讯
爱睡觉,90後,搞笑幽默
新闻趣事
车世界,财经资讯,美食,美女,旅游
亲子,小学数学,小学教育,校园生活
校园生活,搞笑幽默,西安生活,美食,美图摄影
社会,经济,青春,八卦杂谈,看电影,爱音乐,90後,媒体,美图摄影,创业
搞笑幽默
育儿百科,情感生活,新闻趣事,青岛生活,搞笑幽默
杂志,平面设计,旅遊,传媒,重庆生活
美容服饰
厦门生活,美食
美容服饰,旅游,美食,新闻趣事,兰州生活
杭州生活
人生,爱幻想,善良,网购,睡懒觉,低调,读书分享,情感生活,宠物图集,新闻趣事
教育
乐山生活,搞笑幽默,名人明星,视频音乐
爱美食,旅行,萌宠,吃货,法语,厦门生活
唱K,90,营销,男人,铜陵生活
旅游,美图摄影,新闻趣事,搞笑幽默,西安生活
贵阳生活
视频音乐
日本生活,美图摄影,旅游

美女
大师英语,北京 tesol,tesol
美图摄影
视频音乐
佛山生活
美图摄影,宠物图集,新闻趣事,美食,校园生活
美食,搞笑幽默,美容服饰
BTC,mtgox,比特币中国,okcoin,中本聪,挖矿,矿机,莱特币,比特币,bitcoin
美食
星座运势,名人明星,新余生活
校园生活
宠物图集,情感生活,育儿百科,美食
旅游
情感生活,宠物图集,旅游,新闻趣事,搞笑幽默
郑州生活
校园生活
星座运势,美食,大连生活,校园生活
星座运势
美图摄影
读书分享
育儿百科,情感生活,旅游,美容服饰,武汉生活
校园生活
IT 数码,新闻趣事,职场招聘,旅游,美食
korea,韩流,海购,休闲娱乐,时尚,韩国,旅游
旅游
美图摄影,星座运势,重口味,美食,美容服饰
重口味,科学探索
财经资讯
校园生活
美食
校园生活
校园生活
美图摄影,美食,广州生活
资讯,潮人,服饰搭配,服装设计,服装网店,服装,原创,美图摄影
影视音乐,阅读,电子商务,互联网,IT,KM,团队建设,项目管理,知识管理
新闻趣事
名人明星
育儿百科,新闻趣事,旅游,美容服饰,搞笑幽默
校园生活
完美主义,偶喜欢玩
搞笑幽默
美容服饰
搞笑幽默
名人明星,美女,视频音乐,搞笑幽默,娄底生活

新发型介绍,发型设计,发型师,红牛,CF 造型工作室,90 后宅男,大吃货
名人明星
旅游,情感生活,职场招聘,视频音乐,美女
体育资讯
美容服饰
深圳生活
植物爱好者,园艺爱好者,景天科,多肉植物
校园生活
旅游
搞笑幽默
情感生活,房产,视频音乐,新闻趣事,唐山生活
北京生活
美食,IT 数码,体育资讯,天津生活,搞笑幽默
情感生活
美食
美容服饰,长沙生活,八卦杂谈,搞笑幽默,新闻趣事
视频音乐
校园生活
八卦杂谈,房产,职场招聘,无锡生活
星座运势
美容服饰
美食,视频音乐,武汉生活,旅游,美女
星座运势
随性,跆拳道,90 后
上海生活
上网,动漫,美图摄影
美容服饰,旅游,动漫,读书分享,宠物图集,校园生活
漫画,赚钱,愤青
情感生活,美食,搞笑幽默,合肥生活,名人明星
美图摄影,美食,动漫,宠物图集,承德生活
育儿百科
美容服饰,美国生活
视频音乐
体育资讯,职场招聘,游戏,搞笑幽默,美女
欧美,美食,视频音乐,校园生活,情感生活,星座运势
美图摄影
搞笑幽默
动漫
职场招聘,IT 数码,八卦杂谈,新闻趣事,徐州生活
宠物图集
新闻趣事
视频音乐,名人明星,新闻趣事,搞笑幽默,楚雄生活
上饶生活
搞笑幽默

搞笑幽默,视频音乐
女式包包,男装精品,女装网店,情感生活,八卦杂谈,美容服饰
体育资讯,IT 数码,搞笑幽默,名人明星,新闻趣事
美食,旅游,视频音乐
美食
美容服饰
珠海生活
新闻趣事
创意设计
体育资讯
搞笑幽默
糗事段子,趣闻糗事,搞笑糗事,热门糗事,搞笑幽默,校园生活,美女,体育资讯,新闻趣事
梧州生活
TanMusic,音乐,于湉
美图摄影,名人明星,美食,视频音乐,搞笑幽默
巧克力控,咖啡控,零食达人,意大利美食,旅游,意大利生活
唠嗑,新闻趣事
校园生活
美女
名人明星
日本生活,动漫
体育资讯,挑战赛,唇枪舌战,口技,Battle,Beatbox
小说,情感,诗词,博客,扬州生活
美食,育儿百科,新闻趣事,搞笑幽默,郑州生活
名人明星
IT 数码,美图摄影,视频音乐
美图摄影,视频音乐
旅游,房产,美食,美女,搞笑幽默
美女,校园生活
美女,搞笑幽默
读书分享,北京生活,旅游
视频音乐,其他生活,美图摄影,旅游
上海生活
上海生活
美食,旅游,搞笑幽默
名人明星
育儿百科,新闻趣事,旅游,美食,八卦杂谈
新闻趣事,美食,搞笑幽默
上海生活
上海生活
上海生活
上海生活
美食,游戏,魔兽军团
体育资讯

名人明星,动漫,美容服饰,美食,情感生活
校园生活,旅游
做梦,唱 k,无厘头,美容护肤,搞笑,网购,吃,美图摄影,时装,猫咪控
搞笑幽默
上海生活
上海生活
上海生活
投资理财,贵金属,黄金,纸白银,白银 TD,现货白银,天通银
上海生活
上海生活
校园生活
郑州生活
美图摄影
上海生活
上海生活
上海生活
搞笑幽默,美图摄影
东莞生活,育儿知识,育儿亲子,育儿交流,吃货,教育,购物狂
美图摄影
财经资讯
美图摄影,美食,天津生活
旅游
上海生活
上海生活
上海生活
校园生活
星座运势
美女
校园生活
校园生活
热爱生活,旅游
美图摄影
Music,欧美控,摄影控,视频音乐
美食,视频音乐,宠物图集,美图摄影,加拿大生活
美食
上海生活
美女
旅游
搞笑幽默
车世界,动漫,体育资讯
新闻趣事
情感生活,搞笑幽默,美图摄影,新闻趣事
美图摄影,视频音乐,IT 数码
IT 数码

房产
superjunior,名人明星,搞笑幽默
美食
视频音乐
旅游,体育资讯,八卦杂谈,搞笑幽默,南昌生活
动漫
北京生活
科学探索
Girlsday,李惠利,朴素珍,方敏雅,金亞榮
体育资讯,读书分享,车世界,视频音乐,美女
车世界,旅游,体育资讯,美图摄影,校园生活
旅游
南通生活
搞笑幽默
南充生活
宠物图集,搞笑幽默,美食,广州生活
校园生活,上海生活
美女
古灵精怪,羽毛球,手机,美食,搞笑,吃货
旅游
新闻趣事,名人明星,IT 数码,视频音乐,搞笑幽默
摄影,潮流,新闻趣事,美食,校园生活,广州生活,搞笑幽默
体育资讯
美图摄影,校园生活
古玩,文化,画廊,收藏,艺术
动漫
美食
动漫,美容服饰
南京生活
IT 数码
搞笑幽默
深圳,深圳高薪,广东人才,广东工作,深圳招聘,深圳人,深圳市,深圳工作,深圳人才,深圳生活
新闻趣事,天津生活
动漫
八卦杂谈,美食,名人明星,搞笑幽默
搞笑幽默
北京生活,美食
天津生活
海外生活
IT 数码,体育资讯,新闻趣事,校园生活,搞笑幽默
新闻趣事
视频音乐
香港生活

IT 数码
八卦杂谈,健康养生,财经资讯,新闻趣事,旅游
美图摄影,旅游,美食
校园生活
情感生活,北京生活
星座运势,新闻趣事,时尚控,爱购物,搞笑幽默
武汉生活
八卦杂谈,搞笑幽默,名人明星,美食,新闻趣事
名人明星,读书分享,情感生活,美图摄影,美女
IT 数码
美食
动漫,八卦杂谈,校园生活
十二星座,白羊,金牛,星座,星座研究,星座物语
搞笑幽默,名人明星,情感生活,美容服饰
校园生活,美食,读书分享,美容服饰,动漫
代购,時裝
成都生活,美图摄影
美容服饰,美食
八卦杂谈,旅游
高跟鞋控,原单
美容服饰,名人明星
浪漫,爱情,NBA,美图摄影
八卦杂谈
星座运势,猫咪控
美食,旅游,搞笑幽默
音乐爱好者,交友,运动,志愿者,工作,男人
美食,名人明星
银票票,霹雳,动漫
视频音乐
搞笑幽默,美容服饰,美食,北京生活
动漫
美食
名人明星
IT 数码
海外生活
台湾生活,麦霸,自信,搞笑幽默,宁波生活
花样美男,读书分享,IT 数码
花样美男,IT 数码
视频音乐
美食
科学探索,校园生活
财经资讯,古典音乐,娱乐,佛教,高尔夫,广东生活,爱旅行,车世界
晋城生活
星座运势

双子,美食
篮球,期货,股票,财经资讯
移动互联,网络游戏,手机游戏
90後
美图摄影,IT 数码,旅游
toryburch,kipling,coch,katespade,JuicyCouture,starbucks
北京生活
动漫
教育,车世界,八卦杂谈,搞笑幽默,上海生活
加拿大生活
校园生活,南京生活
体育资讯
便利店,外卖,餐饮,购物,重庆,本地生活,重庆生活
